# Probing the Metabolic Network in Bloodstream-Form *Trypanosoma brucei* Using Untargeted Metabolomics with Stable Isotope Labelled Glucose

**DOI:** 10.1371/journal.ppat.1004689

**Published:** 2015-03-16

**Authors:** Darren J. Creek, Muriel Mazet, Fiona Achcar, Jana Anderson, Dong-Hyun Kim, Ruwida Kamour, Pauline Morand, Yoann Millerioux, Marc Biran, Eduard J. Kerkhoven, Achuthanunni Chokkathukalam, Stefan K. Weidt, Karl E. V. Burgess, Rainer Breitling, David G. Watson, Frédéric Bringaud, Michael P. Barrett

**Affiliations:** 1 Drug Delivery, Disposition and Dynamics, Monash Institute of Pharmaceutical Sciences, Monash University, Parkville Campus, Parkville, Victoria, Australia; 2 Centre de Résonance Magnétique des Systèmes Biologiques, Université de Bordeaux, CNRS UMR-5536, Bordeaux, France; 3 Wellcome Trust Centre of Molecular Parasitology, Institute of Infection, Immunity and Inflammation, College of Medical, Veterinary and Life Sciences, University of Glasgow, Glasgow, United Kingdom; 4 Department of Public Health, Institute of Health and Wellbeing, College of Medical, Veterinary and Life Sciences, University of Glasgow, Glasgow, United Kingdom; 5 Centre for Analytical Bioscience, School of Pharmacy, University of Nottingham, University Park, Nottingham, United Kingdom; 6 Department of Medicinal and Pharmaceutical Chemistry, Faculty of Pharmacy, University of Tripoli, Tripoli, Libya; 7 Systems and Synthetic Biology, Department of Chemical and Biological Engineering, Chalmers University of Technology, Göteborg, Sweden; 8 Glasgow Polyomics, Wolfson Wohl Cancer Research Centre, Garscube Campus, College of Medical Veterinary & Life Sciences, University of Glasgow, Glasgow, United Kingdom; 9 Manchester Institute of Biotechnology, Faculty of Life Sciences, University of Manchester, Manchester, United Kingdom; 10 Strathclyde Institute of Pharmacy and Biomedical Sciences, University of Strathclyde, Glasgow, United Kingdom; Oregon Health & Science University, UNITED STATES

## Abstract

Metabolomics coupled with heavy-atom isotope-labelled glucose has been used to probe the metabolic pathways active in cultured bloodstream form trypomastigotes of *Trypanosoma brucei*, a parasite responsible for human African trypanosomiasis. Glucose enters many branches of metabolism beyond glycolysis, which has been widely held to be the sole route of glucose metabolism. Whilst pyruvate is the major end-product of glucose catabolism, its transamination product, alanine, is also produced in significant quantities. The oxidative branch of the pentose phosphate pathway is operative, although the non-oxidative branch is not. Ribose 5-phosphate generated through this pathway distributes widely into nucleotide synthesis and other branches of metabolism. Acetate, derived from glucose, is found associated with a range of acetylated amino acids and, to a lesser extent, fatty acids; while labelled glycerol is found in many glycerophospholipids. Glucose also enters inositol and several sugar nucleotides that serve as precursors to macromolecule biosynthesis. Although a Krebs cycle is not operative, malate, fumarate and succinate, primarily labelled in three carbons, were present, indicating an origin from phosphoenolpyruvate via oxaloacetate. Interestingly, the enzyme responsible for conversion of phosphoenolpyruvate to oxaloacetate, phosphoenolpyruvate carboxykinase, was shown to be essential to the bloodstream form trypanosomes, as demonstrated by the lethal phenotype induced by RNAi-mediated downregulation of its expression. In addition, glucose derivatives enter pyrimidine biosynthesis via oxaloacetate as a precursor to aspartate and orotate.

## Author Summary

In this work we have followed the distribution of carbon derived from glucose in bloodstream form trypanosomes, the causative agent of African trypanosomiasis, revealing it to enter a diverse range of metabolites. The work involved using ^13^C-labelled glucose and following the fate of the labelled carbon with an LC-MS based metabolomics platform. Beyond glycolysis and the oxidative branch of the pentose phosphate pathway the label entered lipid biosynthesis both through glycerol 3-phosphate and also acetate. Glucose derived carbon also entered nucleotide synthesis through ribose and pyrimidine synthesis through oxaloacetate-derived aspartate. Appreciable quantities of the carboxylic acids succinate and malate were identified, although labelling patterns indicate they are not TCA cycle derived. Amino sugars and sugar nucleotides were also labelled as was inositol used in protein modification but not in inositol phospholipid headgroup production. We confirm active and essential oxaloacetate production in bloodstream form trypanosomes and show that phosphoenolpyruvate carboxykinase is essential to these parasites using RNA interference. The amount of glucose entering these metabolites is minor compared to the quantity that enters pyruvate excreted from the cell, but the observation that enzymes contributing to the metabolism of glucose beyond glycolysis can be essential offers potential new targets for chemotherapy against trypanosomiasis.

## Introduction

*Trypanosoma brucei* is a protozoan parasite, sub-species of which are responsible for human African trypanosomiasis (HAT) and animal African trypanosomiasis (Nagana) [1]. Current treatments for HAT are inadequate, and new preventative and therapeutic options are urgently required [2]. The parasite is transmitted between mammalian hosts by a tsetse fly vector. The physiological environments of mammalian blood and the tsetse midgut diverge significantly, and bloodstream form and insect form parasites reveal adaptive differences in biochemistry to allow optimised survival in these environments [3,4].

*T*. *brucei* possess glycosomes, peroxisome-derived organelles that contain enzymes required for glycolysis [5]. Within the tsetse fly, glucose is generally scarce with proline a key energy source [6]. Procyclic form (PCF) trypanosomes preferentially use proline as a source of carbon and energy [6,7,8], although in glucose-rich culture medium they preferentially utilize glucose through glycolysis [7,8]. For many years it has been widely accepted that bloodstream form (BSF) trypanosomes exhibit greatly reduced metabolic potential, where glycosomal glucose utilization through glycolysis is the sole energy source [4,5,9,10]. Under aerobic conditions pyruvate was considered the sole end-product, with the redox balance maintained by a mitochondrial alternative oxidase shunt acting to regenerate dihydroxyacetone phosphate (DHAP) from glycerol 3-phosphate [11]. Under anaerobiosis, equal quantities of pyruvate and glycerol are produced, where the reverse reaction of glycerol kinase (GK) generates glycerol from glycerol 3-phosphate [9]. Early studies on BSF trypanosomes isolated from rodents identified other secreted metabolites, including succinate, aspartate and alanine [12–14]. However, the fact that these products were present in small amounts and that stumpy form trypanosomes, a non-replicative form of the parasite pre-adapted for life in the tsetse fly [15,16], could contaminate the slender BSF preparations, led to these observations being considered of limited relevance in formulating models of a simplified pathway for glucose catabolism in the slender replicative form of *T*. *brucei* [9].

Evidence for a more diverse fate of glucose in BSF trypanosome metabolism has, however, emerged. For example, NMR analysis of glucose metabolism [17,18] confirmed glycerol and pyruvate as the major end products of glycolysis, while also detecting significant amounts of alanine. The conversion of pyruvate to alanine may even be essential to BSF trypanosomes, since it was not possible to knockout the alanine aminotransferase (AAT) gene [19], although significant reduction of its transcript by RNAi was possible [19]. An oxidative branch of the pentose phosphate pathway (PPP) is operative [20], and 6-phosphogluconate dehydrogenase is essential to these forms [21,22]. Glucose-derived ribose was also shown to be incorporated into cellular nucleotides in BSF trypanosomes [23,24], and inositol derived from glucose is incorporated into the glycosylphosphatidyl inositol anchors attached to the abundant variant surface glycoprotein coat of these cells [25,26]. Recently it was shown that pyruvate dehydrogenase (PDH) becomes essential if slender bloodstream forms are deprived of threonine as a source of acetate [27]; in this situation PDH is needed to generate acetate from glucose metabolism.

The systematic application of reverse genetics to PCF trypanosomes combined with NMR analyses of end products of metabolism has demonstrated the importance of succinate fermentation pathways in both the glycosome and mitochondrion, through a route independent of the classical TCA cycle [28–30]. A glycosomal succinate shunt converts phosphoenolpyruvate (PEP) produced in the cytosol to oxaloacetate using PEP carboxykinase (PEPCK) following glycosomal reuptake. PEP can also be converted to pyruvate, which enters the mitochondrion to feed the PDH complex for production of acetyl-CoA, which is converted by the mitochondrial acetate:succinate CoA-transferase (ASCT) and acetyl-CoA thioesterase (ACH) to produce acetate [30,31].

The advent of metabolomics technology [32–34] allows an unbiased analysis of metabolism across diverse pathways in *T*. *brucei*. Stable-isotope labelling permits direct detection of active metabolic pathways within a live cell system [35–39] and offers a direct route to follow the distribution of atoms from a precursor substrate through the metabolic network. We have previously applied the approach to PCF trypanosomes [38], and here extend the methodology to make a comprehensive assessment of the distribution of glucose-derived carbon through the BSF trypanosome.

## Results

### Extensive glucose utilization for anabolic processes in bloodstream form *T*. *brucei*

**Fig 1:**
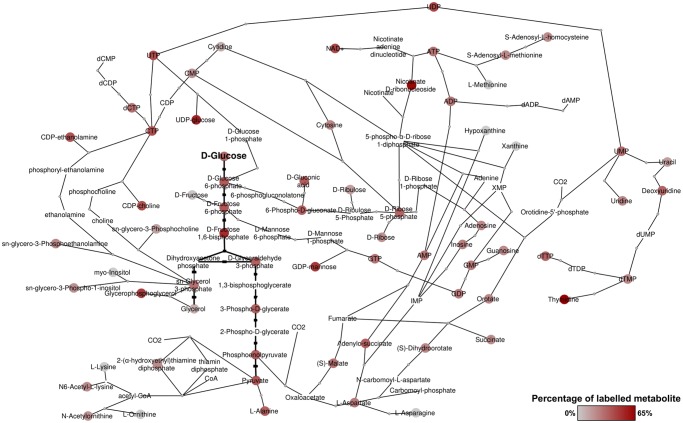
Overview of metabolites labelled from D-glucose in bloodstream form *T*. *brucei*. Metabolic network derived from detected metabolites and annotated enzymes in *T*. *brucei*. Red nodes indicate labelled metabolites from 50% U-^13^C-glucose, with darker red indicating higher percentage labelling. Grey nodes indicate no labelling detected. Nodes without spots indicate that the metabolite was not detected, but the presence is suggested by adjacent metabolites in the network.

BSF *T*. *brucei* were fed a 50:50 mixture of U-^12^C and U-^13^C-glucose as the major carbon source for one hour and 24 hours. Glycolytic intermediates were rapidly labelled, as expected as a result of high flux through the glycolytic pathway [10], with complete (50%) labelling of the major end-product, pyruvate. Significant labelling was observed in metabolites of many other pathways, indicating extensive anabolic utilization of glucose. In total, over 150 metabolites were detected with glucose-derived carbon labelling, including sugar phosphates, sugar nucleotides, lipids and secondary metabolites derived from glycolytic intermediates and end-products (S1 Table). Whilst excreted pyruvate accounts for the majority of catabolised glucose [10], glucose-derived pyruvate also serves as a major source of alanine and acetyl-CoA within the cell. Furthermore, metabolism of glucose through the oxidative branch of the PPP provides ribose 5-phosphate, as evidenced by its being 50% U-^13^C-labelled, which in turn is utilized for nucleotide biosynthesis. Of significant interest was the labelling of numerous carboxylic acids including malate, fumarate and succinate, and extensive labelling in aspartate and various pyrimidines. In all, these results demonstrate extensive utilization of glucose for anabolic purposes in bloodstream form *T*. *brucei* (Fig 1).

### Revisiting glycolysis in bloodstream form *T*. *brucei*

**Fig 2:**
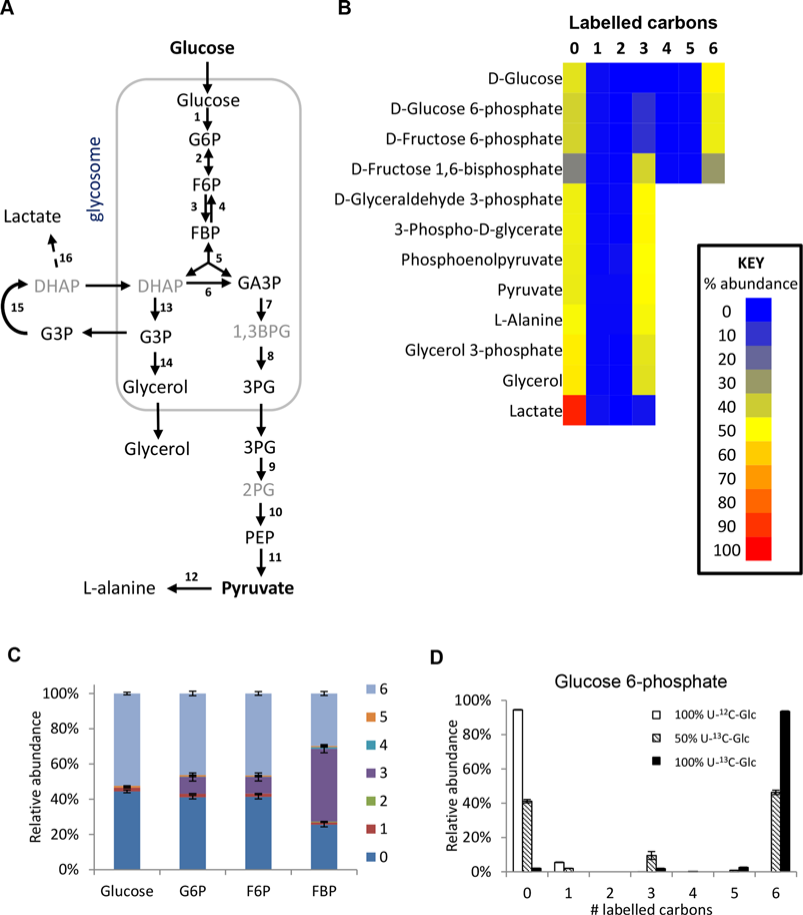
Labelling of glycolytic intermediates from 50% U-^13^C-glucose in BSF *T*. *brucei*. (A) Schematic of the glycolytic pathway in BSF *T*. *brucei*. Black font = detected on LCMS, grey font = inferred intermediates not detected in this assay. (B) Heatmap of relative isotopologue abundances for glycolytic intermediates after labelling for 24 hours with 50% U-^13^C-glucose in HMI11. 0 = all carbons unlabelled (e.g. U-^12^C-glucose), 6 = six carbons labelled (e.g. U-^13^C-glucose). (C) Isotopologue abundances of hexose phosphates after labelling for 24 hours with 50% U-^13^C-glucose (n = 3, mean ± SD). (D) Isotopologue abundances of glucose 6-phosphate after labelling for 24 hours with 100% U-^12^C-glucose (white columns), 50% U-^13^C-glucose (hatched columns) or 100% U-^13^C-glucose (black columns) (n = 3, mean ± SD). Abbreviations: G6P, glucose 6-phosphate; F6P, fructose 6-phosphate; FBP, fructose-1,6-bisphosphate; DHAP, dihydroxyacetone phosphate; GA3P, glyceraldehyde 3-phosphate; 1,3BPG, 1,3-bisphosphoglycerate; 3PG, 3-phosphoglycerate; 2PG, 2-phosphoglycerate; PEP, phosphoenolpyruvate; G3P, glycerol 3-phosphate. Enzymes: 1, hexokinase; 2, glucose-6-phosphate isomerase; 3, phosphofructokinase; 4, fructose-1,6-bisphosphatase; 5, aldolase; 6, triosephosphate isomerase; 7, glyceraldehyde 3-phosphate dehydrogenase; 8, phosphoglycerate kinase; 9, phosphoglycerate mutase; 10, enolase; 11, pyruvate kinase; 12, alanine aminotransferase; 13, glycerol-3-phosphate dehydrogenase; 14, glycerol kinase; 15, mitochondrial FAD-dependent glycerol-3-phosphate dehydrogenase; 16, methylglyoxal detoxification pathway.

The glycolytic pathway is highly active in *T*. *brucei*, which explains the 6-carbon labelling in glucose 6-phosphate (G6P), fructose 6-phosphate (F6P) and fructose-1,6-bisphosphate (FBP), and the 3-carbon labelling in the triose phosphate intermediates of lower glycolysis (Fig 2).

Interestingly, additional 3-carbon labelled isotopologues of hexose phosphates were observed. These isotopologues represent 10% of the intracellular G6P and F6P, and 40% of the FBP. These metabolites were identified by exact mass and retention time using authentic standards, although we cannot absolutely rule out other metabolites of the same mass and retention time (i.e. unanticipated hexose phosphate isomers) co-eluting with these metabolites. These findings are inconsistent with unidirectional glucose flow through the Embden-Meyerhof-Parnas glycolytic pathway. Labelling studies with 100% U-^13^C-glucose produced very little 3-labelled hexose phosphate (~2% of the hexose phosphate is 3-labelled even when only ~97% of the glucose is labelled inside the cells), indicating that all carbons in the hexose phosphates are originally derived from glucose (Fig 2d). Considering this lack of another source and the absence of other reactions that could produce 3-labelled hexose-phosphates, the 3-labelled hexose-phosphates most likely derive from the 3-labelled fructose bisphosphate (possibly via the reverse phosphofructokinase reaction although a contribution from fructose bisphosphatase cannot be ruled out). Gluconeogenesis from amino acids or other carbon sources is not identified, consistent with previous observations [20].

The high percentage of 3-carbon labelled FBP when using the 50:50 mix of U-^13^C-glucose and U-^12^C-glucose seems counter-intuitive, given the very strong net forward flux through glycolysis and the high concentration of FBP. However, our computational analysis shows that it is not impossible. The aldolase reaction is reversible (the net aldolase flux = forward flux—reverse flux). The observed labelling would require that the part of the FBP pool created by the “reverse” aldolase reaction, working in the direction of FBP production, would represent 85% (in HMI medium) or 97% (in CMM medium) of the total FBP pool (see S1 Text for detailed explanations). These large contributions by the reverse reaction are possible under certain conditions, in spite of the net flux strongly favouring the forward direction. Simulations using a previously published collection of models which are described in detail in [40] show that under the classical assumption that glycosomes are impermeable, reverse aldolase fluxes can vary widely with plausible kinetic parameter values, most frequently with between 0 and 60% of the FBP pool coming from the “reverse” aldolase reaction (i.e. between 0 and 29% being potentially 3-labelled). However, if the glycosome is semi-permeable to metabolites smaller than FBP itself (model 3 in ref 40) due to non-selective pore-forming channels in the glycosomal membrane [41], high reverse aldolase flux, accounting for 60–99% of the FBP pool is favoured (S1 Text). This would be in excellent agreement with the observed labelling pattern.

In addition to pyruvate, its transamination product alanine was produced, probably via AAT, an enzyme previously shown to be essential to BSF trypanosomes [19]. Very low levels of labelled lactate were also detected, with all three carbons labelled. No lactate dehydrogenase has been identified in *T*. *brucei*, although L-lactate production has been shown to occur through an unusual methylglyoxal detoxification pathway in trypanosomes [42], which may correspond to the metabolite we find of the corresponding mass.

A number of potential pyruvate-derived conjugated metabolites were observed, although their significance is unknown. For example, an abundant metabolite with either 3C or 6C labelling corresponding to the molecular formula C_6_H_8_O_6_, was putatively identified as parapyruvate [43]. Pyruvate also appeared to form adducts with basic amino acids to produce metabolites with mass, labelling and predicted retention times consistent with carboxyethyl-L-arginine and carboxyethyl-L-ornithine (S1 Table). Another abundant novel 3-carbon labelled metabolite was detected with a molecular formula of C_6_H_9_NO_4_S, a likely condensation product of pyruvate and cysteine, while C_7_H_11_NO_5_ may correspond to the formation of a pyruvate-threonine adduct. These metabolites were more abundant in cells grown in HMI11 compared to CMM, consistent with the greater abundance of L-cysteine and L-threonine in the richer medium. In the absence of authentic standards, however, further analysis will be required to confirm the structure and physiological relevance of these compounds.

### Pentose phosphate pathway

**Fig 3:**
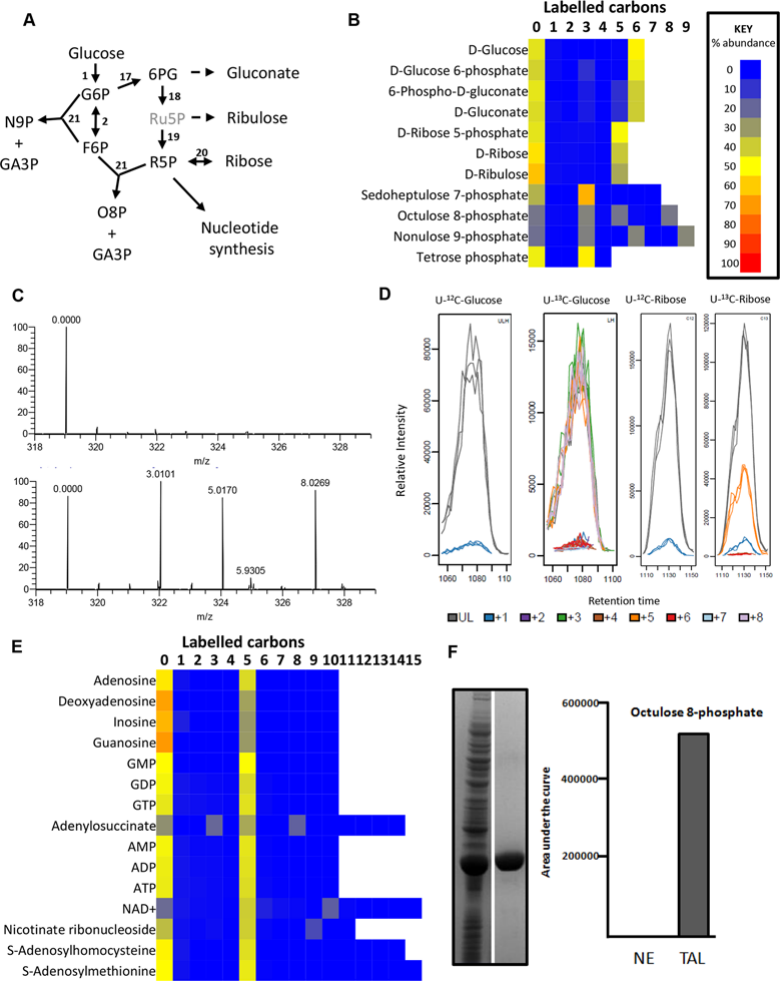
Labelling of pentose phosphate pathway and purine nucleotides from 50% U-13C-glucose in BSF *T*. *brucei*. (A) PPP in BSF *T*. *brucei*. Black font = detected on LCMS, grey font = inferred intermediates not detected in this assay. (B) Heatmap of relative isotopologue abundances for pentose phosphate pathway intermediates after labelling for 24 hours with 50% U-^13^C-glucose in HMI11. (C) Representative mass spectra for octulose 8-phosphate from *T*. *brucei* incubated with unlabelled (U-^12^C) glucose (top) and labelled (U-^13^C) glucose (bottom). The +3, +5 and +8 isotopologues are predominant in the labelled sample. (D) Chromatograms for all octulose 8-phosphate isotopologues from *T*. *brucei* incubated with unlabelled (U-^12^C) glucose, labelled (U-^13^C) glucose, unlabelled (U-^12^C) ribose and labelled (U-^13^C) ribose. (n = 3). (E) Heatmap of relative isotopologue abundances for purine nucleotides and cofactors after labelling for 24 hours with 50% U-^13^C-glucose in HMI11. Abbreviations: G6P, glucose 6-phosphate; F6P, fructose 6-phosphate; 6PG, 6-phosphogluconate; Ru5P, ribulose 5-phosphate; R5P, ribose 5-phosphate; O8P, octulose 8-phosphate; N9P, nonulose 9-phosphate; GA3P, glyceraldehyde 3-phosphate. (F) The *T*. *brucei* transaldolase gene was heterologously expressed in *E*. *coli* and the protein purified (left hand panel, with the crude *E*. *coli* extract on the left and purified protein to the right). Purified transaldolase was shown to produce octulose 8-phosphate when provided with ribose 5-phosphate and fructose 6-phosphate as acceptor and donor substrates respectively (right hand panel). Enzymes: 1, Hexokinase; 2, glucose 6-phosphate isomerase; 17, glucose 6-phosphate dehydrogenase; 18, 6-phosphogluconate dehydrogenase; 19, ribose 5-phosphate isomerase; 20, ribokinase; 21, transaldolase.

The pentose phosphate pathway intermediates (6-phosphogluconate and ribose 5-phosphate) are fully labelled, indicating a linear oxidative PPP derived from glucose 6-phosphate (Fig 3). The complete (5-carbon) labelling of ribose in purine nucleotides confirms that this pathway is the major source of ribose 5-phosphate for nucleotide synthesis (Fig 3e). This contrasts with the situation in PCF *T*. *brucei*, which also possess an active non-oxidative branch of the PPP [20] and show the characteristic 2-, 3- and 5-carbon ribose 5-phosphate labelling as a consequence of the transaldolase and transketolase (TKT) reactions that combine fully labelled and fully unlabelled precursors [38]. The lack of these isotopologues thus confirms the lack of TKT in BSFs [20,44].

Two high molecular weight sugar phosphates, octulose 8-phosphate (O8P) and nonulose 9-phosphate (N9P) were detected and putatively identified by accurate mass and predicted retention time (as authentic standards are not available). The labelling patterns (Fig 3c-d) indicate production by transaldolase activity from a three carbon unit donated from an aldose donor to either a pentose phosphate (to produce O8P) or hexose phosphate (to produce N9P). Interestingly, BSF *T*. *brucei* have previously been shown to possess transaldolase activity [20], in spite of the absence of TKT. The absence of 2C- and 6C- labelled O8P in BSFs, while they are present in PCFs [38], indicates that a PPP intermediate is the precursor for this metabolite. We expressed and purified the gene encoding *T*. *brucei* transaldolase (Tb927.8.5600) in *Escherichia coli*. When the enzyme was mixed with ribose 5-phosphate and F6P as substrates, mass spectrometry revealed that O8P was produced, confirming the ability of this enzyme to carry out this reaction (Fig 3f). Moreover, cells were labelled with U-^13^C-ribose which resulted in extensive labelling of ribose 5-phosphate (produced by ribokinase), and significant 5-carbon labelling in O8P (Fig 3d).

The detection of metabolites with formulas consistent with sedoheptulose 7-phosphate (C_7_H_15_O_10_P) and erythrose 4-phosphate (C_4_H_9_O_7_P) was surprising in a cell without TKT activity. However, the 50% U-^13^C-glucose labelling reveals only the 3-carbon labelled isotopologue of each, confirming that these are not products of TKT. Further investigation found that the abundance of the C_7_H_15_O_10_P peak was very low in all samples, and the retention time of C_4_H_9_O_7_P was inconsistent with the authentic standard for erythrose 4-phosphate. More work is therefore required to elucidate the identity and source of these metabolites, and to confirm that a non-canonical pathway involved in sugar phosphate remodelling exists in trypanosomes.

### Nucleotide salvage

The lack of uniform labelling in purine bases, nucleosides and nucleotides confirms the requirement for exogenous purine bases in trypanosomatids which lack purine biosynthetic pathways (Fig 3e). However, glucose-derived ribose produces 5-carbon labelled purine nucleotides, as purine salvage pathways are highly active [45]. Transporters for the efficient uptake of purine nucleosides have been characterized, and guanosine and inosine appear to be the major purine sources in *in vitro* culture, even in the presence of excess hypoxanthine [46]. These labelling data also confirm that nucleoside hydrolases [47] that form purine bases are the primary route for nucleotide salvage from imported nucleosides and that glucose-derived ribose 5-phosphate is added through purine phosphoribosyltransferase [48]. The almost complete labelling of the ribose moiety of adenosine nucleotides indicates minimal purine salvage from exogenous adenosine by adenosine kinase under our *in vitro* growth conditions, in spite of its high activity in these cells [49].

Intracellular synthesis of various cofactors, including NAD^+^ and S-adenosyl methionine (AdoMet), was also confirmed by incorporation of labelled ribose into the nucleotide subunits. AdoMet is an important precursor in polyamine synthesis, resulting in production of 5’methylthioadenosine (MTA). The 5-carbon labelling in these two metabolites confirms that the labelling is in the ribose moiety. However, the absence of labelling in methionine precludes activity of a methionine salvage pathway as previously proposed [50]. Methionine uptake from serum [51] apparently fulfils all methionine requirements of BSF parasites. The absence of labelling in α-ketomethylthiobutyrate (KMTB), the last intermediate in the methionine salvage pathway, further confirms the lack of function in this proposed pathway. KMTB probably arises instead from transamination of methionine, and it was previously shown to be excreted from parasites along with other amino acid-derived keto acids [46].

### Carboxylic acid derivatives of catabolised glucose

An active TCA cycle is reportedly not operative in BSF *T*. *brucei*, and the observation that <1% of detected 2-oxoglutarate contains label from glucose corroborates this (S1 Table). A small amount of acetyl-coA and oxaloacetate were, nevertheless, directed into citrate, as seen/observed by the 2-, 3- and 5-carbon labelling in excess of the natural abundance of ^13^C by a total of approximately 1% in cells grown in HMI11, and 5% in CMM-grown cells (S2 Table). The relevance of this low level citrate production is not known. PCF *T*. *brucei* do not use the classical citrate—malate shuttle, instead utilizing a unique acetate shuttle to provide acetyl-CoA to the cytosol [31].

**Fig 4:**
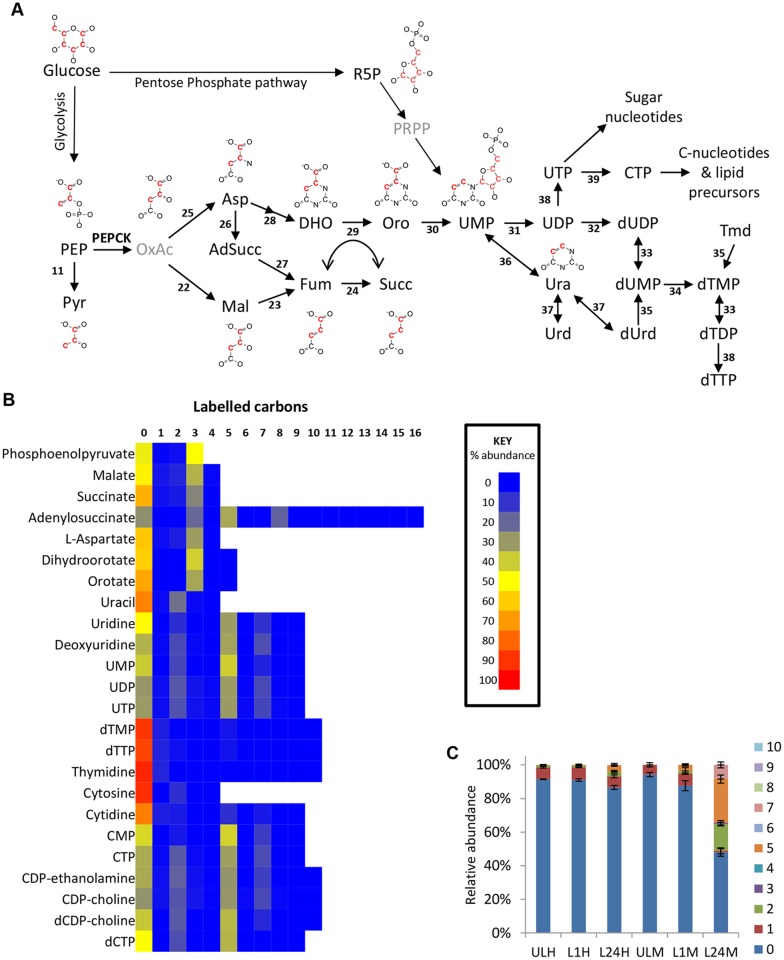
Glucose-derived dicarboxylic acid and pyrimidine metabolic pathways in BSF *T*. *brucei* indicate activity of PEPCK. (A) Metabolic pathways of dicarboxylic acid and pyrimidine synthesis in BSF *T*. *brucei*. Red carbons in structures indicate the predominant ^13^C labelling. (B) Heatmap of relative isotopologue abundances for dicarboxylic acid and pyrimidine intermediates after labelling for 24 hours with 50% U-^13^C-glucose in HMI11. (C) Isotopologue abundances of dTTP after labelling for 0 (U), 1 (L1) and 24 (L24) hours with 50% U-^13^C-glucose in HMI11 (H) or CMM (M) (n = 2–3, mean ± SD). Extensive *de novo* dTTP synthesis was only observed in CMM (i.e. in the absence of exogenous thymidine). Abbreviation: PEP, phosphoenolpyruvate; Pyr, pyruvate; OxAc, oxaloacetate; Mal, malate; Fum, fumarate; Succ, succinate; Asp, Aspartate; AdSucc, adenylosuccinate; DHO, dihydroorotate; Oro, orotate; Ura, uracil; Urd, uridine; dUrd, deoxyuridine; Tmd, thymidine. Enzymes: 11, pyruvate kinase; 22, malate dehydrogenase; 23, fumarase; 24, NADH-dependent fumarate reductase); 25, aspartate aminotransferase; 26, adenylosuccinate synthase; 27, adenylosuccinate lyase; 28, aspartate carbamoyltransferase and dihydroorotase; 29, dihydroorotate dehydrogenase; 30, orotate phosphoribosyltransferase and orotidine 5-phosphate decarboxylase; 31, nucleoside diphosphatase; 32, ribonucleoside-diphosphate reductase; 33, thymidylate kinase; 34, thymidylate synthase; 35, thymidine kinase; 36, uracil phosphoribosyltransferase; 37, uridine phosphorylase; 38, nucleoside diphosphate kinase; 39, cytidine triphosphate synthase.

Isotope analysis of intracellular metabolites from cells incubated with 50% U-^13^C-labelled glucose reveals three-carbon labelled isotopologues of succinate and malate, representing 35% (70% when corrected for 50% labelled glucose precursor) of the intracellular malate (Fig 4) and 26% of succinate (52% corrected). This labelling pattern is consistent with the production of succinate via a succinate fermentation pathway. The first enzyme of this pathway, PEPCK, is found in BSF trypanosomes, albeit at a lower level than PCF [52,53]. Although oxaloacetate is not detectable on this analytical platform, the observation of the 3-carbon labelled isotopologue in aspartate (a transamination product of oxaloacetate) supports the production of oxaloacetate from PEP. The total labelling in aspartate (62% corrected) is comparable to malate (70% corrected), demonstrating that intracellular aspartate is primarily synthesized from glucose-derived oxaloacetate, rather than being taken up from the surrounding medium, which contains aspartate at 100 μM [46]. BSF trypanosomes failed to demonstrate appreciable aspartate transport capability [54]. *T*. *brucei* encodes three separate isoforms of malate dehydrogenase (MDH), localized to the mitochondrion, glycosomes and the cytosol, respectively [55]. The glycosomal isoform is reportedly absent from the BSF, while the cytosolic isoform is present at higher levels in BSF than procyclics [55]. Additionally a small amount of the mitochondrial isoform is reportedly present [55–57]. The succinate fermentation pathway therefore might involve oxaloacetate produced in the glycosome via PEPCK which might re-enter the cytosol and possibly the mitochondrion for further metabolism. Further work, systematically removing or knocking down expression of different isoforms, followed by metabolic profiling, will be required to deconvolute the sources of these partially reduced products of glucose catabolism in the BSF trypanosome.

### Aspartate use in nucleotide biosynthesis

Oxaloacetate-derived aspartate is a key precursor in pyrimidine synthesis, being converted to orotate via dihydroorotate. Dihydroorotate and orotate were detected with isotope enrichment equivalent to aspartate, ~35% 3-labelled carbons (70% corrected), confirming *de novo* synthesis of pyrimidines in this way. Uracil labelling was slightly lower (44% corrected), suggesting some uptake of uracil from the medium. However, higher levels of 2-labelling in UMP and UDP suggest that *de novo* synthesized orotate is the major source of pyrimidines under these conditions (Fig 4). Labelling of uridine and cytidine nucleotides was not significantly different between the two different culture media used in this study, HMI11 and CMM. However, significant differences were observed in thymidine nucleotides. dTTP and dTMP abundance in CMM-grown cells was only half that observed for HMI11, which contains 20 μM added thymidine. The isotope labelling patterns of dTTP and dTMP in CMM matched those of UMP and the other pyrimidine nucleotides, indicating complete synthesis from *de novo* synthesized pyrimidines by thymidylate synthetase in thymidine-poor media [58,59]. dTMP and dTTP in HMI11-grown cells were less than 10% labelled, confirming a preference for thymidine salvage by the action of thymidine kinase when exogenous thymidine is available (Fig 4c).

Aspartate is also an important intermediate in the purine salvage pathway, in which the formation of adenylosuccinate and release of fumarate results in nitrogen transfer from aspartate to IMP, producing AMP. Adenylosuccinate was detected with 3-carbon labelling, which confirms the role of glucose-derived aspartate in the purine salvage pathway (Fig 3e).

### PEPCK is essential to BSF *T*. *brucei*

**Fig 5:**
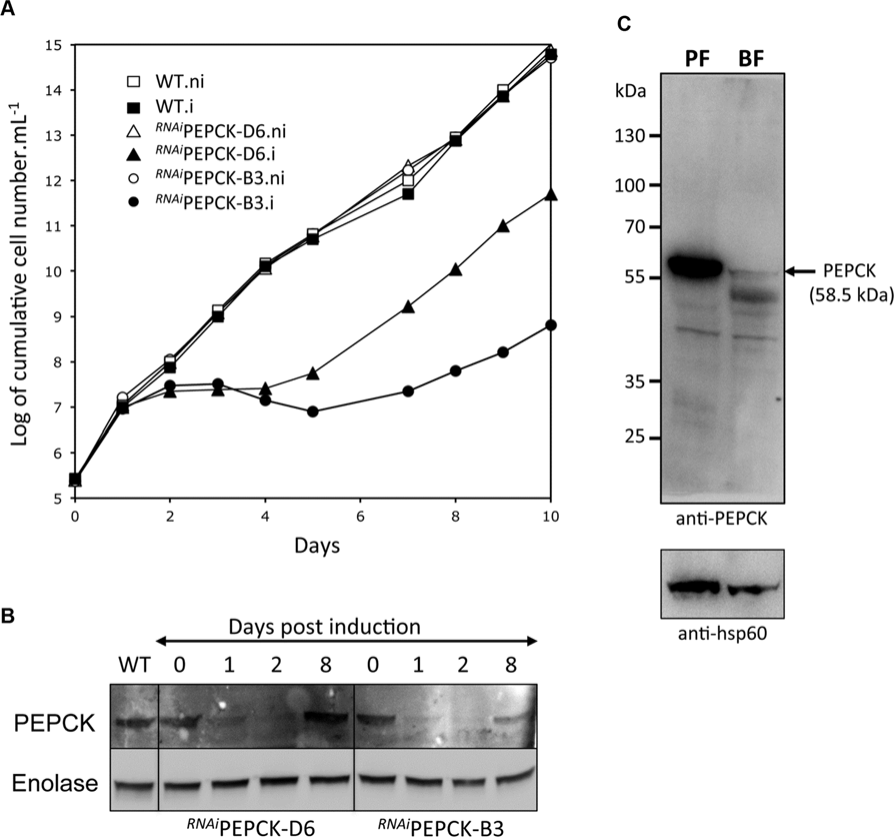
PEPCK is essential to bloodstream form *T*. *brucei*. (A) Growth curve of two different clones showing down-regulation of *pepck* by RNAi (^*RNAi*^PEPCK-D6 and ^*RNAi*^PEPCK-B3) and the parental 427 BSF strain (WT) incubated in the presence (.i) or in the absence (.ni) of 10 μg/ml tetracycline. Cells were maintained in the exponential growth phase (between 10^5^ and 2x10^6^ cells/ml) and cumulative cell numbers have been normalized for dilution during cultivation. (B) Western blot analyses of the parental (WT) and mutant cell lines with the anti-PEPCK and anti-enolase sera containing antibodies as indicated in the left margin. (C) Western blot demonstrates significantly higher levels of PEPCK in procyclic form (PF) compared to bloodstream form (BF) WT *T*. *brucei*. *Analyses were performed on 5 x 10^6^ cells per sample and HSP-60 is shown as the loading control.

The discovery that glucose enters amino acid and nucleotide pathways via oxaloacetate indicates that PEPCK might play an unexpectedly important role in BSF trypanosomes. Indeed, a genome-wide RNAi screen indicated the gene could be essential [60]. To test this further, western blot analysis was performed with anti-PEPCK immune serum, showing that PEPCK is expressed in the BSF, albeit at much lower abundance than in the PCF (Fig 5). Two cell lines (^*RNAi*^PEPCK-B3 and ^*RNAi*^PEPCK-D6) were then generated to investigate the role of PEPCK in BSF *T*. *brucei* by RNAi down-regulation of *pepck* gene expression. Both cell lines showed a growth arrest two days after tetracycline induction. A phenotypic reversion 5 days post-induction correlated with a re-expression of PEPCK (Fig 5).

### ^RNAi^PEPCK labelled metabolomics

**Fig 6:**
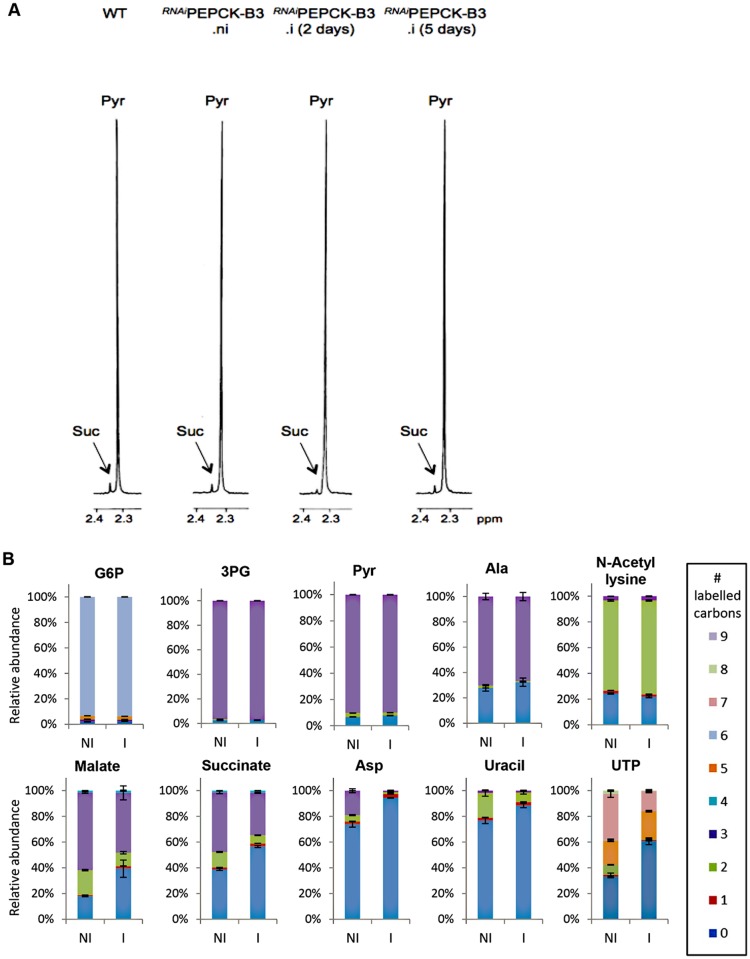
Metabolic profile of ^*RNAi*^PEPCK-B3 BSF *T*. *brucei* confirms depletion of dicarboxylic acids and pyrimidines. (A) Proton (^1^H) NMR analysis of excreted pyruvate and succinate from glucose. Pyruvate (Pyr) and succinate (Suc) excreted by the bloodstream form 427 cell line (WT), and the ^*RNAi*^PEPCK-B3 mutant non-induced (ni), tetracycline-induced 2 days (i 2 days), and tetracycline-induced 5 days (i 5 days) was determined by ^1^H-NMR. The cells were incubated in PBS containing 4 mM glucose for 4 hours. Each spectrum corresponds to one representative experiment from a set of at least 3. A part of each spectrum ranging from 2.2 ppm to 2.4 ppm is shown. (B) LC-MS analysis of intracellular glycolytic intermediates. Relative isotopologue abundances following incubation in PBS containing 4 mM U-^13^C-glucose for 4 hours for metabolic intermediates from ^*RNAi*^PEPCK-B3 mutant non-induced (NI) and tetracycline-induced for 2 days (I). Significant decreases in labelling were observed for PEPCK-derived metabolites including 3-carbon labelling in dicarboxylic acids and aspartate, and 2-carbon labelling in pyrimidine nucleotides (or 7-carbon labelling for ribose-containing metabolites). Labelling was not significantly decreased in glycolytic intermediates or in other glucose-derived metabolites, including alanine, acetate (shown here as acetyllysine) and ribose (shown here as 5-carbon labelling in UTP).

In order to measure the metabolic role of PEPCK, the production of succinate from glucose in BSF trypanosomes was measured using ^1^H-NMR, as previously described for PCF *T*. *brucei* [61]. The parental BSF 427 strain was incubated for 5 hours in PBS/NaHCO_3_ medium containing 4 mM D-glucose as the only carbon source and the incubation medium was analyzed by ^1^H-NMR spectroscopy. In addition to pyruvate, detectable amounts of succinate were quantitatively measured, at levels ~2.7% of the excreted pyruvate (Fig 6a and Table 1). The amount of excreted succinate was decreased 4.3 fold in the ^*RNAi*^PEPCK-B3 mutant after two days of induction compared to the wild-type cells (47 ±17 *versus* 201 ±89 nmol/h/mg of protein). Succinate secretion increased five days after induction (79 ±18 nmol/h/mg of protein) (Fig 6a and Table 1), as a consequence of *pepck* re-expression (Fig 5).

**Table 1:** Production of succinate, pyruvate, alanine and acetate from glucose in PEPCK RNAi *T*. *brucei* measured by ^1^H-NMR.

Cell line	n	Excreted molecules from glucose			
		Pyruvate	Succinate	Alanine	Acetate
* nmol / h / mg of protein*					
WT^a^	3	7534±1535	201±89	821±174	564±42
^*RNAi*^PEPCK-3.ni^b^	6	7281±634	127±26	754±49	328±51
^*RNAi*^PEPCK-B3.i^c^ 2days	7	6913±760	47±17	731±8	361±18
^*RNAi*^PEPCK-B3.i^c^ 5days	3	6880±797	79±18	722±64	281±44

^*a*^ Strain 427 bloodstream-form *T*. *brucei*

^*b*^ Non-induced ^*RNAi*^PEPCK-B3 (without added tetracycline)

^*c*^ Induced ^*RNAi*^PEPCK-B3 (with added tetracycline)

To confirm reduction of succinate production in the ^*RNAi*^PEPCK-B3 tetracycline-induced cell line (at 2 days), we determined incorporation of ^13^C-labelling from U-^13^C-glucose into intracellular metabolites by LC-MS. A reduction of ^13^C_3_ incorporation into malate (24 ± 10%) and succinate (29 ± 2%) was observed in the induced ^*RNAi*^PEPCK-B3 cell line compared to the non-induced cells (Fig 6b). Labelling in other products of PEPCK-derived oxaloacetate was also significantly reduced, including aspartate (15-fold), uracil (3-fold) and UTP (2-fold). In contrast, ^13^C incorporation into metabolites produced upstream of the PEPCK step (G6P, 3PG and pyruvate), and those in other pathways derived from glycolytic intermediates (alanine, acetyl-CoA, and ribose 5-phosphate) were not significantly altered (Fig 6b).

### Acetate production in BSF *T*. *brucei*

**Fig 7:**
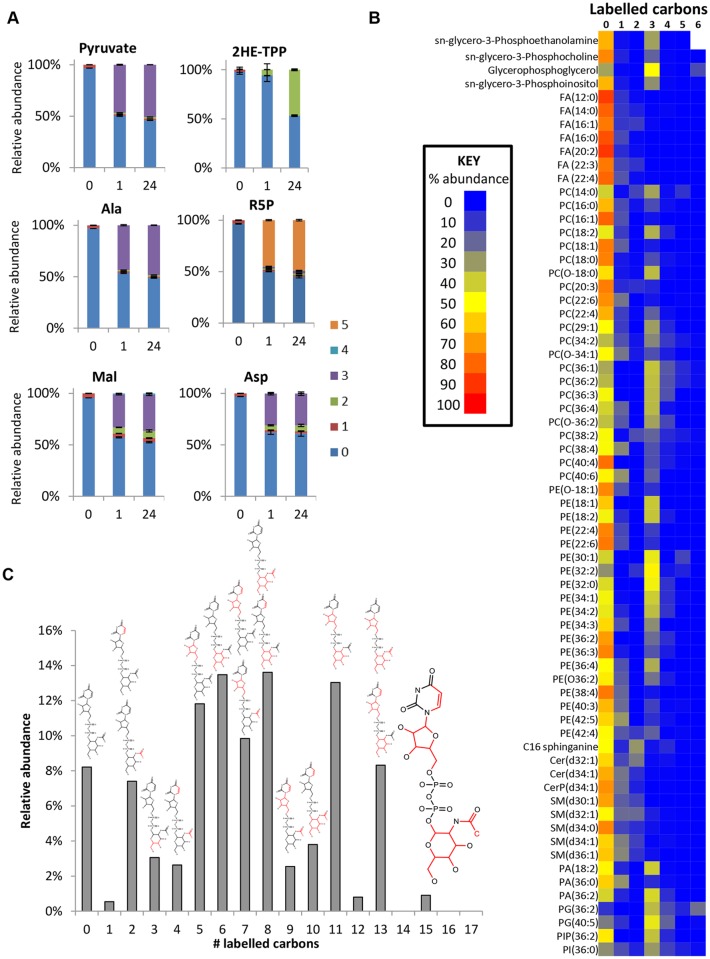
Glucose is a carbon source for acetate, lipids and sugar nucleotides in WT BSF *T*. *brucei*. (A) Time-dependent incorporation of glucose into diverse pathways. Relative isotopologue abundances for unlabelled cells (0) and cells incubated with 50% U-^13^C-glucose in HMI11 for one hour (1) and 24 hours (24). Labelling reaches equilibrium within 1 hour for pyruvate, alanine, malate, aspartate and ribose 5-phosphate. Minimal labelling is observed in 2-hydroxyethyl-TPP after 1 hour, but complete (50%) labelling occurs within 24 hours, indicating a relatively slow flux towards acetate production by this pathway. (n = 3, mean ± SD). (B) Heatmap of relative isotopologue abundances for lipids after labelling for 24 hours with 50% U-^13^C-glucose in HMI11 indicating a combination of salvage mechanisms with de novo synthesized glycerol phosphate and limited incorporation of glucose-derived acetyl-CoA. After manual curation, no isotopologues containing more than 6 labelled carbons could be confirmed for the detected lipids. (C) Isotopologue abundances of UDP-N-acetyl glucosamine (UDP-GlcNAc) after labelling for 24 hours with 50% U-^13^C-glucose in HMI11 reveal incorporation of labelled carbon through glucosamine, acetate, ribose and uracil. Structures represent the likely isotopologue(s) for each mass with ^13^C atoms and bonds shown in red. Additional minor isotopologues have been omitted for clarity, the most abundant of which are isotopologues containing 3-labelled glucosamine 6-phosphate.

In addition to its secretion and conversion to alanine, pyruvate is a major source of acetyl-CoA in BSF trypanosomes. Although the intracellular concentration of acetyl-CoA was below the limit of detection for our analytical method, evidence of its production is provided by 2-hydroxyethyl thiamine pyrophosphate (Fig 7a), the intermediate in pyruvate’s conversion to acetyl-CoA by PDH. The presence of two labelled carbons indicates formation directly from glucose-derived pyruvate. Labelling was minimal after one hour, but complete labelling was observed at 24 hours, suggesting that flux through this pathway is significantly slower than through most other pathways (Fig 7a). It has recently been shown [27] that PDH knockdown is conditionally lethal in BSF *T*. *brucei*. Both threonine, via a threonine dehydrogenase (TDH) pathway, and glucose, via PDH, can provide acetate and the latter route becomes essential when threonine is absent, or redundant due to loss of TDH [27]. Furthermore, acetylated metabolites including acetylcarnitine, acetyllysine, acetylornithine and acetylglutamine were all detected with 2 labelled carbons from glucose (S1 Table). The unlabelled proportion of these metabolites probably relates to threonine’s key role in acetate provision [27].

### Glucose contributions to fatty acid and lipid biosynthesis

*De novo* fatty acid synthesis was detected in BSF *T*. *brucei* by incorporation of two labelled carbon units in free fatty acids and phospholipids (Fig 7b). However, the very low level of labelling in fatty acids suggests that *de novo* fatty acid synthesis is minor compared to salvage mechanisms, and a high level of lysophospholipid uptake may represent the principal source of fatty acids in these cells [62] with elongation accounting for modelling fatty acids of different chain length [63].

The additional 3-carbon labelling observed in glycerophospholipids demonstrates that lipid salvage utilizes glycolysis-derived glycerol 3-phosphate for synthesis of phospholipid head-groups. Despite the requirement for salvage of host (or culture medium) lipids for the provision of choline, and most fatty acids, around half of the glycerol phosphate in lipid head-groups was derived from glycolysis (ranging from 8 to 100% corrected labelling for detected PC (phosphatidylcholine) and PE (phosphatidylethanolamine) lipids; Fig 7 and S1 Table, the variability relating to the fact that the relative contributions of the salvage and *de novo* synthesis pathways are not uniform across lipid species.

### Nucleotide sugars and glycoconjugate precursors

Several other labelled metabolites were detected (S1 Table) indicating that glucose enters amino sugars (N-acetyl-D-glucosamine and N-acetyl-D-glucosamine 6-P), sugar nucleotides (UDP-Glc, or UDP-Gal GDP-man and UDP-GlcNAC; Fig 7c) and low-level labelling in other sugars (tentatively identified as myo-inositol, fructose, glucuronate and arabinonate, although accurate identification of sugars is difficult using accurate mass-based metabolomics, as many isomers are theoretically possible for most carbohydrate metabolites; hence, additional orthogonal approaches are required to confidently identify these structures. Nothwithstanding their abundance is clearly low compared to glucose and fructose the two primary hexoses. Mannose is absent in its free form, its generation occurring only when nucleotide conjugated and in phosphorylated form [24]). Targeted studies of sugar nucleotide biosynthesis have already demonstrated *de novo* biosynthesis of UDP-Glc [64] and GDP-Man [24] from glucose, and analysis of inositol metabolism demonstrated *de novo* synthesis of inositol for protein glycosylation, in addition to inositol salvage for lipid biosynthesis [25,26]. Data from the current study demonstrated less than 10% labelling in inositol, and confirmed that the inositol moiety of the lipid headgroup glycerol-phosphoinositol was unlabelled (i.e. not formed by *de novo* synthesis from glucose, consistent with targeted analyses conducted elsewhere to investigate the origin of inositol in *T*. *brucei* [25,26]).

## Discussion

The introduction of metabolomic technologies, particularly in conjunction with stable isotope tracing has, in recent years, transformed our ability to analyse metabolism in biological systems. For example, isotopologue studies into *Leishmania* [65], *Plasmodium* [36,37] and *Toxoplasma* [35] have identified novel pathways and resolved long-standing questions of metabolism. The African trypanosome, *Trypanosoma brucei*, is the causative agent of human African trypanosomiasis, a neglected tropical disease of sub-Saharan Africa, for which new drugs are urgently needed. The slender bloodstream form of these parasites has generally been considered to have a highly streamlined glucose catabolic pathway [3–5,9,10]. The localization of the first seven enzymes of the glycolytic pathway to membrane-bounded peroxisome-like organelles known as glycosomes [5] has been proposed to be instrumental to the regulation of flux through glycolysis by providing an environment in which ATP/ADP and NAD/NADH can be balanced [9,10]. This arrangement has enabled the production of a well-defined dynamic mathematical model of glycolysis [9,10] and the models have been able to predict several important phenomena, such as the contribution of different enzymes to the overall control of the pathway. Recent iterations have included the addition of parameters that explicitly take into account uncertainty related to the system [66] and also the activity of the pentose phosphate pathway [22] and the presence of permeability pores that allow free diffusion of several glycolytic intermediates between the cytosol and glycosome [40].

Here we have taken the systematic route to analyse the full extent of glucose metabolism in long slender BSF trypanosomes.

Extensive pyruvate production was observed, consistent with existing models of glycolytic flux for energy production. However, the 3-carbon labelled isotopologues observed in glycolytic hexose phosphates reveals previously unreported complexity in this pathway. The lack of transketolase [20,42] indicates that a canonical non-oxidative pentose phosphate cycle is missing from these cells and thus is not the source of 3-labelled hexose phosphates. The role of transaldolase in BSFs is uncertain, and transaldolase-mediated production of 3-labelled fructose 6-phosphate (from F6P and GA3P) could be hypothesized, although this appears to be a redundant reaction. Alternatively, aldolase [67], acting to combine either U-^13^C or U-^12^C DHAP with U-^13^C or U-^12^C GA3P would produce the relative proportions of 0-, 3- and 6-labelled fructose bisphosphate as identified here. The further production of 3-labelled F6P (and G6P) from FBP would require fructose-1,6-bisphosphatase activity, and both transcriptomic [68] and proteomic [69] data indicate that this enzyme is present in BSF *T*. *brucei*. A family of models, simulating flux through glycolysis whilst taking into account our uncertainties about the system and its distribution between glycosome and cytosol [40], reveals that if metabolites smaller than FBP are able to exchange between compartments while FBP is not, then a significant proportion of the total FBP in the cell can indeed arise through the condensation of GA3P and DHAP. The data, therefore, supports the proposed role of pores creating a semi-permeable glycosome [41]. Further work is required to fully investigate the intermediate fluxes in glycolytic metabolism and to determine the temporal and spatial aspects of metabolism that give rise to these unexpected observations.

At this point we cannot rule out definitively the production of a compound with the mass and retention time of FBP as an artefact of the experimental system. The same might be true of other exotic, unexpected species identified in this study. However, a non-enzymatic aldol condensation of triose phosphates is highly unlikely, given that non-enzymatic condensations and additions generally require extreme pH conditions (<1 or > 9). To confirm this, DHAP and GA3P were mixed in CMM medium followed by extraction in the conditions used to extract our cellular lysates and storage for seven days at -80°C, and no FBP or other potential condensation or addition products were observed by LC-MS. We therefore consider it likely that many of the metabolites that we describe here are *bona fide* products of trypanosome metabolism, and the incorporation of carbon-13 label confirms the endogenous production of these metabolites (compared to the unlabelled metabolites primarily acquired from the serum in the growth media), although their physiological function, or indeed relevance remains unknown. Metabolites can be produced either enzymatically or chemically in cells as inevitable by-products of metabolism, methylglyoxal being an example already discussed here [42].

Apart from the identification of numerous unexpected metabolites and pathways operating in BSF trypanosomes, our experiments also rule out the operation of other pathways previously assumed to be functional. For example, a rapid deamination of aromatic amino acids was believed to contribute to a methionine cycle involved in provision of decarboxylated S-adenosylmethionine used in polyamine synthesis [50]. Here, however, we demonstrate that carbons from glucose do not enter methionine, which is provided exclusively through cellular uptake instead [51]. This means that the pathways of methylthioadenosine detoxification remain to be elucidated in the BSF trypanosome.

While the majority of glucose flows to secreted pyruvate under aerobic culture conditions, we show that glucose metabolism is pervasive in the BSF parasites. In addition to pyruvate and glycerol, various other metabolites are secreted, including alanine, the transaminated product of pyruvate. This reaction appears to be essential since the alanine transaminase gene could not be knocked out of the *T*. *brucei* genome [19], although knockdown by RNAi was possible. Succinate was also secreted. The succinate produced in BSF trypanosomes is primarily labelled in three carbons, which indicates its derivation from glycolytic phosphoenolpyruvate, rather than from a canonical TCA cycle, which would produce primarily 2-carbon and 4-carbon labelled isotopologues. Analysis of the phosphoproteome of BSFs identified glycosomal NADH-dependent fumarate dehydrogenase [70]. However, it is not possible to distinguish between glycosomal, cytosolic or potentially mitochondrial derivation of these metabolites, and systematic metabolite profiling following gene knockout or knockdown of various enzymes will be necessary to deconvolute these pathways.

Although it has been suggested that glucose makes negligible contribution to anabolic processes in BSF trypanosomes [10], numerous isolated studies have revealed that glucose serves as a precursor in processes including sugar nucleotide and glycoconjugate synthesis [23], inositol production [25,26] and recently acetate production [27]. Glucose also enters pyrimidine biosynthesis via oxaloacetate and lipid metabolism via glycerol 3-phosphate as well as acetate production. RNAi knockdown of PEPCK confirmed this activity as being essential in BSF trypanosomes. The same enzyme was not essential to PCFs, although its contribution is major in this parasitic insect form [71]. We recently demonstrated that loss of bound phosphate from the glycolytic pathway in the glycosome via the PPP requires a mechanism to retain the bound phosphate balance [22]. PEPCK offers an obvious means to restore glycosomal ATP, and this link to the PPP might explain the unexpectedly essential nature of this enzyme to the BSF trypanosome.

Although the untargeted mass spectrometry platform we use here is intrinsically unable to quantify the total flux of glucose carbon into the various pathways discussed here, it is clearly the case that the overwhelming majority of glucose is catabolised to pyruvate (Table 1). The total amount glucose entering these other pathways would be in the order of just a few percent of the total consumed, in agreement with the measurements of Haanstra *et al* [10]. Although only a small minority of the total glucose consumption, however, glucose is the major carbon source for the production of many of these extra-glycolytic metabolites, and this flux is clearly important given the fact that several enzymes, including PEPCK shown here, turn out to be essential to the metabolism of these parasites.

The identification of novel essential enzymes in the BSF trypanosome may have important implications for chemotherapy. Glycolysis has long been considered an attractive target given the absolute dependency on glucose for energy and carbon provision to these cells [72]; our results show that additional pathways around the periphery of glycolysis might be equally promising.

## Materials and Methods

### Parasite culture

*T*. *brucei brucei* bloodstream form (s427) was cultured *in vitro* in HMI11 [73] and CMM [44] supplemented with 10% fetal calf serum (FCS) Gold (PAA, Piscataway, NJ) at 37°C, 5% CO_2_. Initial 5 ml cultures in 25 ml vented flasks (Corning) were grown to a maximum density of 4 × 10^6^ cells/ml and subcultured by 1-in-100 or 1-in-1,000 dilution every 2 or 3 days, respectively. A hemocytometer (Neubauer) was used for cell counts.

### Labelling experiments and sample preparation

Two days prior to the extraction, 2 × 10^4^ cells/ml were seeded in 30 ml of HMI11 and CMM. Growth media was then replaced with media containing 50% of ^13^C-labelled glucose (12.5 mM of ^12^C-glucose + 12.5 mM of ^13^C-glucose for HMI11, and 5 mM of ^12^C-glucose and 5 mM of ^13^C-glucose for CMM culture) at 24 h and 1 h prior to extraction. All samples were extracted at the same time atequivalent cell densities of approximately 2 ×10^6^ cells/ml. To yield 5 ×10^7^ cells, appropriate volumes of cell culture were harvested and quenched in EtOH/dry ice bath as previously described [74]. Cells were centrifuged at 1250 ×*g* for 10 min and spent media removed; cells were washed with pre-cooled phosphate buffered saline (PBS) at 4°C. After centrifugation at 1250 ×*g* for 5 min, the supernatant was removed and metabolites extracted by adding 100 μl of (ice cold) chloroform/methanol/water (1:3:1) and mixed vigorously for 1 h at 4°C. The extraction mixtures were centrifuged at 13,000 ×*g* for 10 min and the supernatants collected and stored at -80°C prior to analysis. In order to assess instrument performance, one pooled quality control (QC) sample was prepared by mixing an equal volume of all the samples. Independent biological replicates were prepared on different days.

### LC-MS methodologies

Hydrophilic interaction liquid chromatography (HILIC) was carried out on a Dionex UltiMate 3000 RSLC system (Thermo Fisher Scientific, Hemel Hempstead, UK) using a ZIC-pHILIC column (150 mm × 4.6 mm, 5 μm column, Merck Sequant) as previously described [74]. Briefly, the column was maintained at 30°C and samples were eluted with a linear gradient (20 mM ammonium carbonate in water, A and acetonitrile, B) over 46 min at a flow rate of 0.3 ml/min as follows: 80% B to 20% B at 30 min, to 5% B at 32 min and held to 39 min for washing, to 80% B at 40 min and held to 46 min for re-equilibration. The injection volume was 10 μl and samples were maintained at 4°C prior to injection. For the MS analysis, a Thermo Orbitrap Exactive (Thermo Fisher Scientific) was operated in polarity switching mode and the MS settings were as follows: resolution 50,000, AGC 10^6^, m/z range 70–1400, sheath gas 40, auxiliary gas 5, sweep gas 1, probe temperature 150°C, and capillary temperature 275°C. For positive mode ionisation: source voltage +4.5 kV, capillary voltage +50 V, tube voltage +70 kV, skimmer voltage +20 V. For negative mode ionisation: source voltage-3.5 kV, capillary voltage-50 V, tube voltage-70 V, skimmer voltage-20 V. Mass calibration was performed for each polarity immediately prior to each analysis batch. The calibration mass range was extended to cover small metabolites by inclusion of low-mass contaminants with the standard Thermo calmix masses (below *m/z* 1400), C_2_H_6_NO_2_ for positive ion electrospray ionisation (PIESI) mode (*m/z* 76.0393) and C_3_H_5_O_3_ for negative ion electospray ionisation (NIESI) mode (*m/z* 89.0244). To enhance calibration stability, lock-mass correction was also applied to each analytical run using these ubiquitous low-mass contaminants.

### Data processing and analysis

Raw LC-MS data were processed with XCMS for untargeted peak detection [75], and mzMatch.R [76] was employed for peak matching and annotation of related peaks. Tentative metabolite identification was carried out by IDEOM using the default parameters [77]. Metabolite identification was performed by matching accurate masses and retention times of authentic standards (MSI confidence level 1, i.e. an annotated compound matched to a standard with two orthogonal approaches), but when standards were not available, predicted retention times were calculated by a previously validated model [78] (MSI confidence level 2, i.e. a putatively annotated compound based on its exact mass determination). Published literature and pathway/genome databases were considered to improve annotation in cases where isomers could not be differentiated based on accurate mass and retention time. Metadata to support the identification of each metabolite is available in the IDEOM file for each study (S3 Table). Metabolites included in the manuscript were manually annotated using authentic standards where available. However, note that many of the metabolite names given in the Ideom file are generated automatically as the software provides a best match to database entries of the given mass and formula. In the absence of additional information these must be considered as putatively-annotated hits; the confidence score in the column adjacent to that hit serves as a guide to this. Clearly it is beyond the scope of any study to provide authenticated annotations to many hundreds of detected compounds, but the full datasets are included in the spirit of open access data.

The mzmatch-ISO software [39] was used to extract all isotopologue abundances from all identified and putative annotated metabolites. Raw data are available in the associated IDEOM files (S3 Table) and chromatograms are in separate pdf files (S1–S2 Figs). Labelled metabolites are given in S4 Table and S5 Table. Data analysis and interpretation was based on the 24 hour time-point, at approximate steady state, unless otherwise stated.

### NMR experiments

*T*. *brucei* bloodstream form cells (2.5 x10^7^) were collected by centrifugation at 1,400 g for 10 min, washed with PBS containing 4 mM glucose and incubated for 5 h at 37°C in 2.5 ml of incubation buffer (PBS supplemented with 5 g/l NaHCO_3_, pH 7.4) containing 4 mM glucose. The integrity of the cells during the incubation was checked by microscopic observation. The supernatant was collected and 50 μl of malate solution in D_2_O (20 mM) was added as internal reference. ^1^H-NMR spectra were performed at 125.77 MHz on a Bruker DPX500 spectrometer equipped with a 5 mm broadband probe head. Measurements were recorded at 25°C with an ERETIC method. This method provides an electronically synthesized reference signal [79]. Acquisition conditions were as follows: 90° flip angle, 5,000 Hz spectral width, 32 K memory size, and 9.3 sec total recycle time. Measurements were performed with 256 scans for a total time close to 40 min. Before each experiment, phase of ERETIC peak was precisely adjusted. Resonances of obtained spectra were integrated and results were expressed relative to ERETIC peak integration.

### Inhibition of *pepck* gene expression by RNAi

Inhibition of *pepck* gene expression by RNAi in the 427 strain was performed by expression of stem-loop “sense/anti-sense” RNA molecules of the targeted sequences introduced in the pHD1334 expression vector. First, the pLew-PEPCK-SAS plasmid was constructed to target a 481 bp fragment of the *pepck* gene (from position 22 bp to 503 bp). Briefly, a PCR-amplified 569 bp fragment, containing the antisense *pepck* sequence with restriction sites added to the primers was inserted into the *Hind*III and *Bam*HI restriction sites of the pLew100 plasmid. Then a PCR-amplified fragment containing the sense *pepck* sequence (505 bp) was inserted upstream of the anti-sense sequence, using *Hind*III and *Xho*I restriction sites (*Xho*I was introduced at the 3’-extremity of the antisense PCR fragment). The resulting plasmid (pLew-PEPCK-SAS) contains a sense and antisense version of the targeted gene fragment, separated by a 52 bp fragment, under the control of the PARP promoter linked to a prokaryotic tetracycline (Tet) operator. Then the PEPCK-SAS *Hind*III-*Bam*HI cassette extracted from the pLew-PEPCK-SAS plasmid was inserted in *Hind*III-*Bam*HI digested pHD1334 vector. The ^*RNAi*^PEPCK cell lines were produced by transfecting the 427 “double marker” (Hyg-Neo) monomorphic cell line with the pHD-PEPCK-SAS plasmid. Transfected cells were selected in IMDM medium containing, hygromycin (5 μg/ml), neomycin (2.5 μg/ml) and phleomycin (2.5 μg/ml). Induction of double-stranded RNA expression was performed by addition of 10 μg/ml tetracycline.

### Cloning and expression of the *T*. *brucei* transaldolase gene

A gene annotated as transaldolase in the *T*. *brucei* genome database Tb427.08.5600, was amplified with the following primers 5’-GCCATATGAATCAACTGGAGAGCCT-3’ and 5’-GCCTCGAGTTACAAAAGTGTAGCGTGA-3’. The PCR product was first cloned into the vector pGEM-T easy and then transferred to the pET28a(+) expression vector using the introduced *Nde*1 and *Xho*1 restriction sites. The resultant plasmid was sequenced to confirm fidelity and transformed into *E*. *coli* strain BL21 (DE3) for expression at 37°C or 4 hours after induction with IPTG. Expressed protein was observed by SDS protein gel electrophoresis and purified using Ni^2+^ chelate chromatography (Fig 2f). The ability of transaldolase to produce octulose 8-phosphate was determined by mixing ribose 5-phosphate (5 mM) and fructose 6-phosphate (5 mM) in Tris-HCl (pH 7.2) and adding transaldolase (1.2 mg/mL) then incubating at 20°C for 2 hrs. Alternatively the reaction was set up with no enzyme. The presence of octulose 8-phosphate was determined by direct infusion of post-reaction mix into the Exactive mass spectrometer (Fig 2f).

### Western blot analyses

Total protein extracts of wild-type or mutant BSF of *T*. *brucei* (5 x10^6^ cells) were size-fractionated by SDS-PAGE (10%) and immunoblotted on Immobilon-P filters (Millipore) [80]. Immunodetection was performed as described [81,82] using primary antibodies, rat anti-*T*. *brucei* PEPCK (diluted 1:1000; gift from T. Seebeck, Bern, Switzerland), rabbit anti- *T*. *brucei* enolase (diluted 1:1,000, gift from P. Michels, Edinburgh, UK) and mouse anti-HSP60 (diluted 1:10 000) [83], and as secondary antibodies, anti-rat, anti-rabbit or anti-mouse IgG conjugated to horseradish peroxidase (BioRad, 1:5,000 dilution). Revelation was performed using the SuperSignal West Pico Chemiluminescent Substrate as described by the manufacturer (Thermo Fisher Scientific). Alternatively, for quantitative analyses, revelation was performed using the Luminata Crescendo Western HRP Substrate (Millipore). Images were acquired and analyzed with a KODAK Image Station 4000 MM and quantitative analyses were performed with the KODAK MI application.

## Supporting Information

S1 Text PDF outlining use of computational models of glucose metabolism to predict relative contributions to the fructose 1,6 bis-phosphate pool. (PDF)

S1 Table csv file of relative isotopologue abundances from BSF *T*. *brucei* grown with 50%-U-13C-glucose in HMI11. (CSV)

S2 Table csv file of relative isotopologue abundances from BSF *T*. *brucei* grown with 50%-U-13C-glucose in CMM. (CSV)

S3 Table IDEOM macro-enabled Excel file of metabolomics data from BSF *T*. *brucei* grown with 50%-U-13C-glucose in HMI11 and CMM. (ZIP)

S4 Table csv file of relative isotopologue abundances from BSF *T*. *brucei* grown with 50%-U-13C-glucose in both CMM and HMI11. (ZIP)

S5 Table Table showing the composition of HMI11 and CMM medium. (DOCX)

S1 Fig PDF of chromatograms from positive mode ESI for all isotopologues detected for identified and putatively annotated metabolites from BSF *T*. *brucei* grown with 50%-U-13C-glucose. (PDF)

S2 Fig PDF of chromatograms from negative mode ESI for all isotopologues detected for identified and putatively annotated metabolites from BSF *T*. *brucei* grown with 50%-U-13C-glucose. (PDF)
