# Supplementary material for: Probing the Metabolic Network in Bloodstream-Form Trypanosoma brucei Using Untargeted Metabolomics with Stable Isotope Labelled Glucose
Source: PLoS Pathog. 2015 Mar 16;11(3):e1004689. doi: 10.1371/journal.ppat.1004689 (PMC4361558; doi:10.1371/journal.ppat.1004689)
Supplement: S1 Text — (PDF) [file ppat.1004689.s001.pdf]

# Percentage of fructose bisphosphate produced via the reverse aldolase reaction as calculated based on different models of glycolysis

Aldolase is a reversible reaction. The net flux through aldolase is therefore equal to the forward flux (consumption of fructose 1,6-bisphosphate) minus the reverse flux (production of fructose 1,6-bisphosphate). If the fructose 1,6-bisphosphate (Fru16BP) pool was created entirely by forward glycolysis, and no aldolase reaction occurred in the reverse direction (i.e. producing Fru16BP), then starting with 50% U-$^{13}$C glucose, the expected isotopologue distribution of Fru16BP would be:

| Number of $^{13}$C | 0 | 1 | 2 | 3 | 4 | 5 | 6 |
|---|---|---|---|---|---|---|---|
| % of Fru16BP | 48.9 | 2.2 | 0 | 0.1 | 0 | 0.7 | 48 |

If the Fru16BP pool was created entirely by the reverse aldolase reaction, then the Fru16BP isotopologue distribution would be:

| Number of $^{13}$C | 0 | 1 | 2 | 3 | 4 | 5 | 6 |
|---|---|---|---|---|---|---|---|
| % of Fru16BP | 23.6 | 1.1 | 0.2 | 48.6 | 1.1 | 0.2 | 25 |

Assuming that the peak matching the mass and retention time of Fru16BP indeed represents this metabolite, we measured 47% of 3-13C Fru16BP in cells grown in CMM medium and 41% in HMI medium. As explained above, the forward flux would produce 0.1% of 3-$^{13}$C labelled Fru16BP and the re-

verse flux would produce 48.6% of 3-13C labelled Fru16BP. We can therefore predict how much of the forward and reverse fluxes is needed to be consistent with the measured proportion of 3-13C Fru16BP. The measured proportions are consistent with respectively 97% (97%*48.6 + 3%*0.1 = 47%) and 85% (85%*48.6 + 15%*0.1 = 41%) of the Fru16BP pool being created via the reverse aldolase reaction.

In order to assess how plausible this is, we used published computational models of glycolysis based on ordinary differential equations [1]. These models all explicitly considered uncertainty about the exact enzyme kinetic parameters, as well as a small proportion of the activity of the glycosomal glycolytic enzymes present in the cytosol. They differ in their topologies reflecting different scenarios concerning the possible permeability of the glycosomal membrane to small metabolites (see table 1 for definitions).

Previously, these different models and the different parameter sets were compared to experimentally measured metabolite concentrations and fluxes by calculating log-likelihoods. The higher the log-likelihood, the better the match between the experimental data and the simulations. Here, we considered the 1% best parameter sets of each model topology (*i.e.* 1000 parameter sets for each topology) as previously simulated with a concentration of external glucose of 50 mM (concentration used in the experimental measurements of intracellular metabolite concentrations) and available in [1]. For each of these models and for each parameter set, we calculated the percentage of the Fru16BP pool that is created via the reverse aldolase reaction. For that, we calculated the ratio $\frac{V_{reverse}}{V_{forward}}$ in both the glycosomes and the cytosol using the equation defined the models.

We then calculated the global percentage of Fru16BP that came from reverse aldolase considering the relative volumes of the glycosomes and the cytosol as defined in the models.

Figure 1 shows the predicted percentages of Fru16BP created via the reverse aldolase reaction in each model and for all 1% best parameter sets,

| **Model** | **Definition** |
|---|---|
| 1a | Impermeable glycosomes + specific transporters accross the glycosomal membrane for: glucose, 3-phosphoglycerate, glycerol + antiporter for the transport of dihydroxyacetone phosphate and glycerol 3-phosphate |
| 1b | Same as 1a but independent transport of dihydroxyacetone phosphate and glycerol 3-phosphate |
| 2 | 1b + glycosomal membrane permeable to glyceraldehyde 3-phosphate |
| 3 | 2 + glycosomal membrane permeable to: 1,3 bisphosphoglycerate, glucose 6-phosphate, fructose 6-phosphate |
| 4 | 3 + glycosomal membrane permeable to frucose 1,6-bisphosphate |
| 5 | 4 + glycosomal membrane permeable to AMP |
| 6 | 5 + glycosomal membrane permeable to ADP and ATP |

Table 1: **Definitions of the different model topology.** All model includes a small fraction of glycosomal glycolytic enzymes in the cytosol. See [1] for details.

separated according to their log-likelihood. Figure 2 represents the same histograms for the 3 best model topologies according to [1] and the best parameter sets (defined as having a log-likelihood above -20).

These results show that in model 1a (which has impermeable glycosomes and an antiporter to transport glycerol 3-phosphate with dihydroxyacetone phosphate) very few parameter sets allow the fraction of labelled Fru16BP to be consistent with the measured isotopologue distribution. However, a high percentage of 3-$^{13}$C Fru16BP, matching the experimentally observed data, is the most likely scenario when the glycosomes are permeable to all metabolites smaller than (and not including) Fru16BP (model 3). When the glycosomes become permeable to Fru16BP itself, the number of parameter sets allowing the simulations to match the measured percentage of 3-$^{13}$C Fru16BP decreases again, and alternative predictions with lower flux in the reverse direction become more likely.

# References


[1] Fiona Achcar, Michael P. Barrett and Rainer Breitling (2013) *Explicit consideration of topological and parameter uncertainty gives new insights into a well-established model of glycolysis.* FEBS J., **280**(18):4640-4651

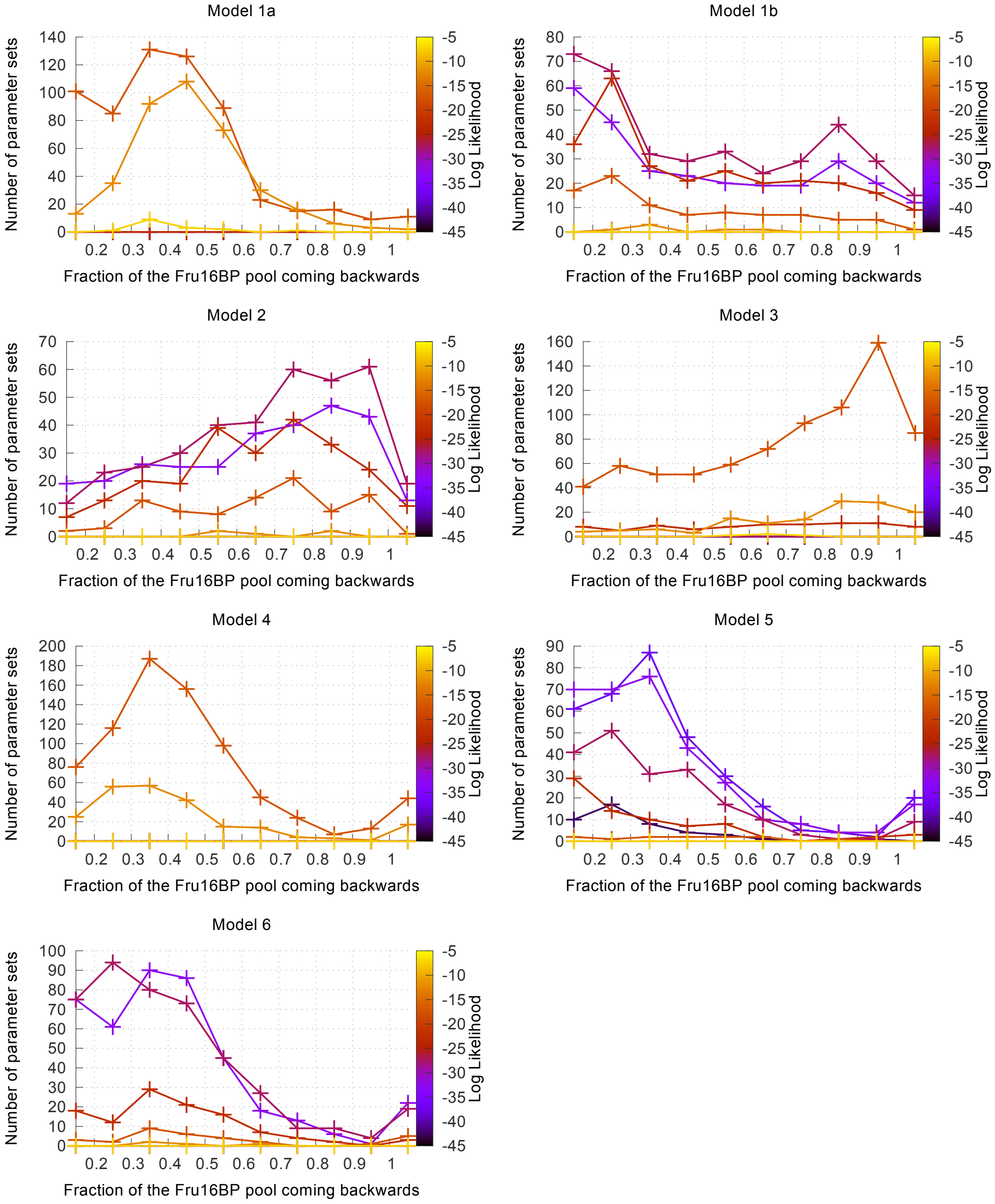


Figure 1: **Histogram of the percentage of Fructose bisphosphate produced via the reverse aldolase reaction for each model and for different log-likelihood ranges.** The parameter sets that best match the data (experimental concentrations and flux measurements) have the highest log-likelihood. Models 1a, 3 and 4 have by far the largest fraction of well-matching parameter sets and are compared in more detail in Figure 2.

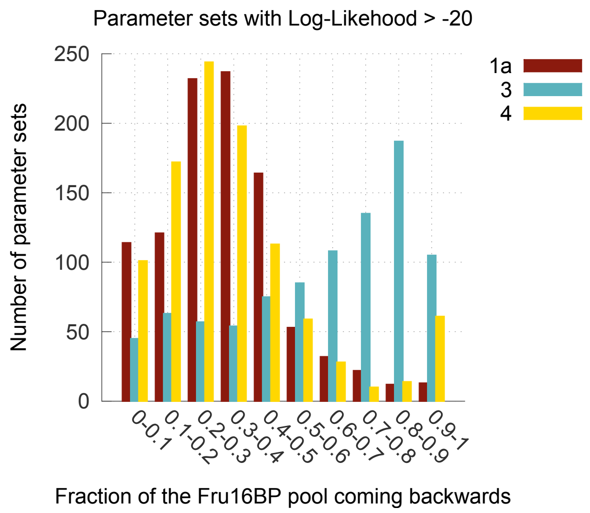


Figure 2: **Histogram of the percentage of Fructose bisphosphate produced via the reverse aldolase reaction for the best models and the best parameter sets.**
