# Supplementary material for: Probing the Metabolic Network in Bloodstream-Form Trypanosoma brucei Using Untargeted Metabolomics with Stable Isotope Labelled Glucose
Source: PLoS Pathog. 2015 Mar 16;11(3):e1004689. doi: 10.1371/journal.ppat.1004689 (PMC4361558; doi:10.1371/journal.ppat.1004689)
Supplement: S5 Table — (DOCX) [file ppat.1004689.s006.docx]

Supporting Table 5: Composition of HMI11 and CMM media

| **Component** | **HMI11 recipe**  **(µM)** | **CMM recipe**  **(µM)** |
| --- | --- | --- |
| Foetal Bovine Serum | 10% | 10% |
| D-Glucose | 25,000 | 10,000 |
| L-Cysteine | 1,504 | 1,000 |
| L-Glutamine | 3,998 | 1,000 |
| L-Phenylalanine | 400 |  |
| L-Tryptophan | 78 |  |
| L-Tyrosine | 574 |  |
| L-Methionine | 201 |  |
| L-Arginine | 482 |  |
| L-Leucine | 801 |  |
| L-Isoleucine | 801 |  |
| L-Valine | 803 |  |
| L-Lysine | 999 |  |
| L-Threonine | 798 |  |
| L-Serine | 400 |  |
| L-Proline | 348 |  |
| L-Histidine | 271 |  |
| Glycine | 400 |  |
| L-Glutamate | 510 |  |
| L-Asparagine | 189 |  |
| L-Aspartate | 226 |  |
| L-Alanine | 281 |  |
| L-Cystine | 379 |  |
| Pyruvate | 1,295 |  |
| Hypoxanthine | 1,000 |  |
| Thymidine | 161 |  |
| myo-Inositol | 40 |  |
| Choline | 38 |  |
| Nicotinamide | 33 |  |
| Pyridoxal | 24 |  |
| Pantothenate | 18 |  |
| Thiamin | 15 |  |
| Folic acid | 9 |  |
| Riboflavin | 1 |  |
| Biotin | 0.053 |  |
| cyanocobalamin | 0.010 |  |
| HEPES | 25000 | 25000 |
| NaCl | 77590 | 77590 |
| NaHCO_3_ | 36000 | 36000 |
| CaCl_2_ | 1500 | 1500 |
| KCl | 4400 | 4400 |
| MgSO_4_ | 814 | 814 |
| KNO_3_ | 0.75 | 0.75 |
| Na_2_SeO_3_ | 0.065 | 0.065 |
| NaH_2_PO_4_ | 900 | 900 |
| Mercaptoethanol | 192 | 192 |
| Bathocuproine disulfonic acid | 52 | 52 |
| Phenol Red | 42 | 42 |
