# Supplementary material for: Probing the Metabolic Network in Bloodstream-Form Trypanosoma brucei Using Untargeted Metabolomics with Stable Isotope Labelled Glucose
Source: PLoS Pathog. 2015 Mar 16;11(3):e1004689. doi: 10.1371/journal.ppat.1004689 (PMC4361558; doi:10.1371/journal.ppat.1004689)

L−cysteine sulfinate

Formula: C3H7NO4S Mass: 153.01 Std.RT: 930.2097108 Ion: NEG

G1

UL +1 +2 +3

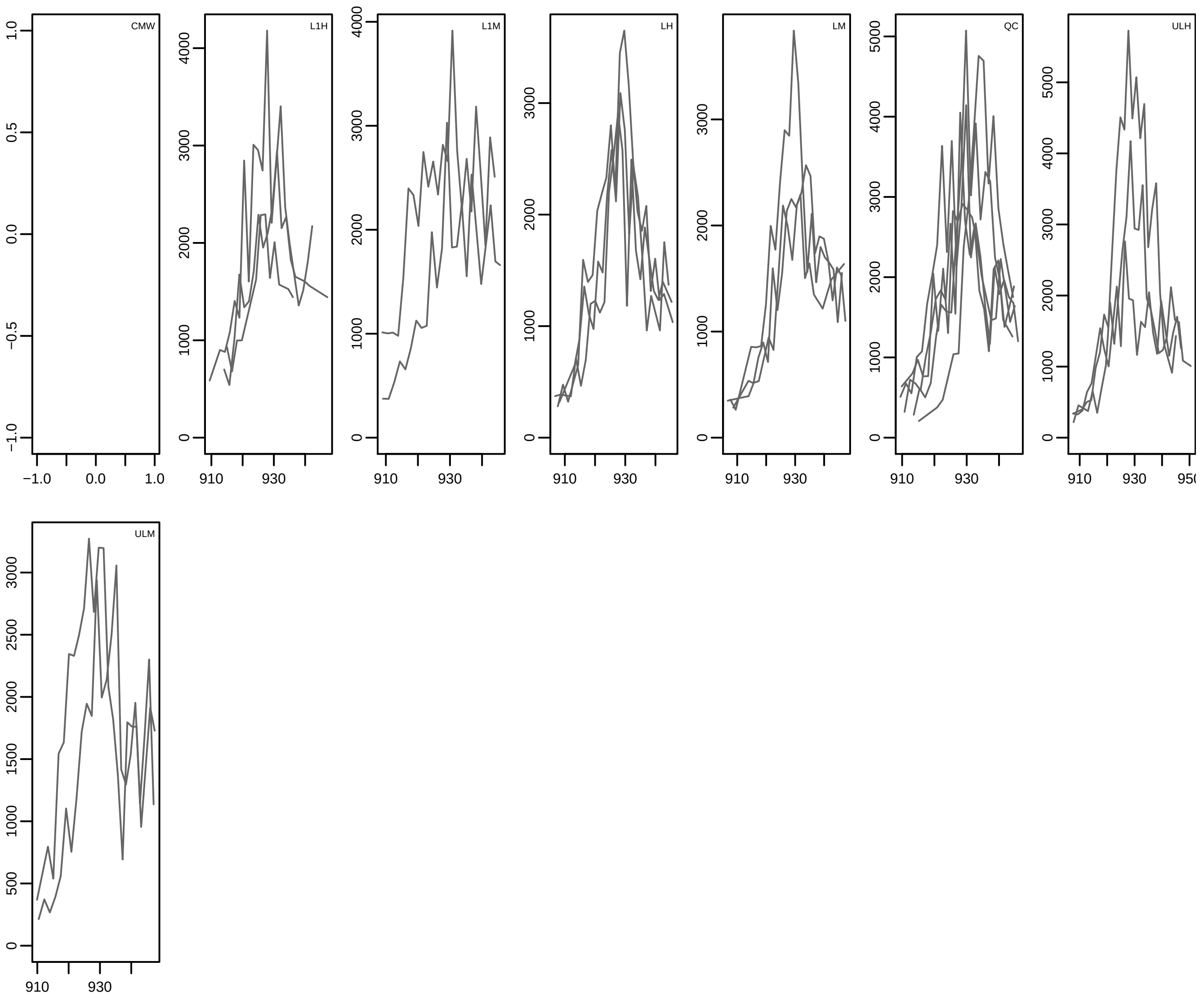

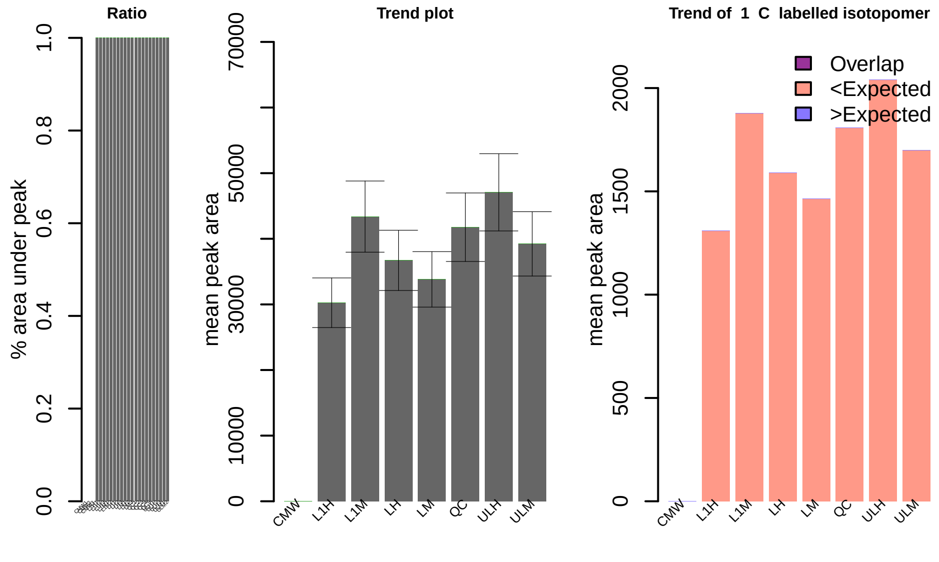

L−Aspartate

Formula: C4H7NO4 Mass: 133.038 Std.RT: 963.9665046 Ion: NEG

G1

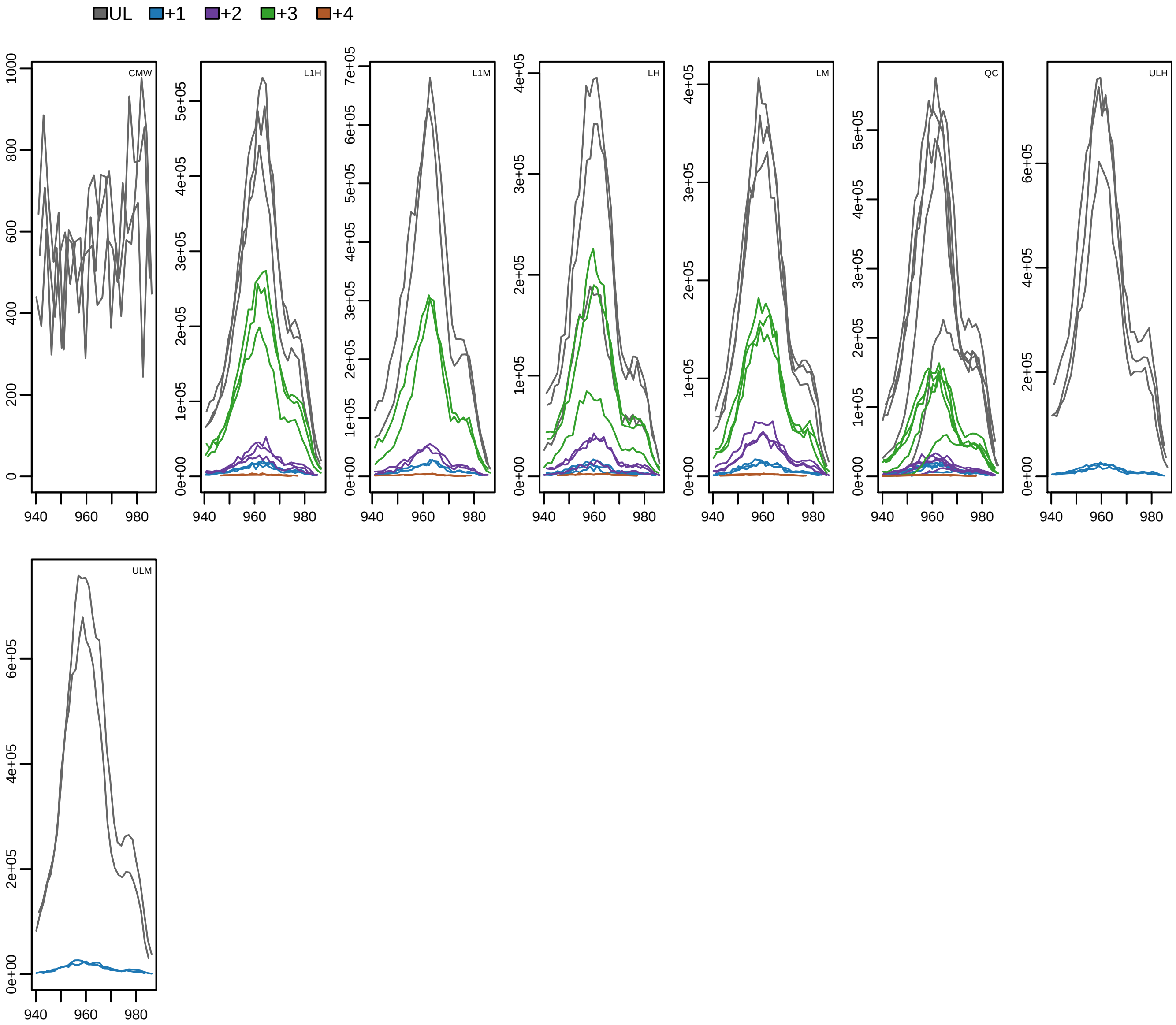

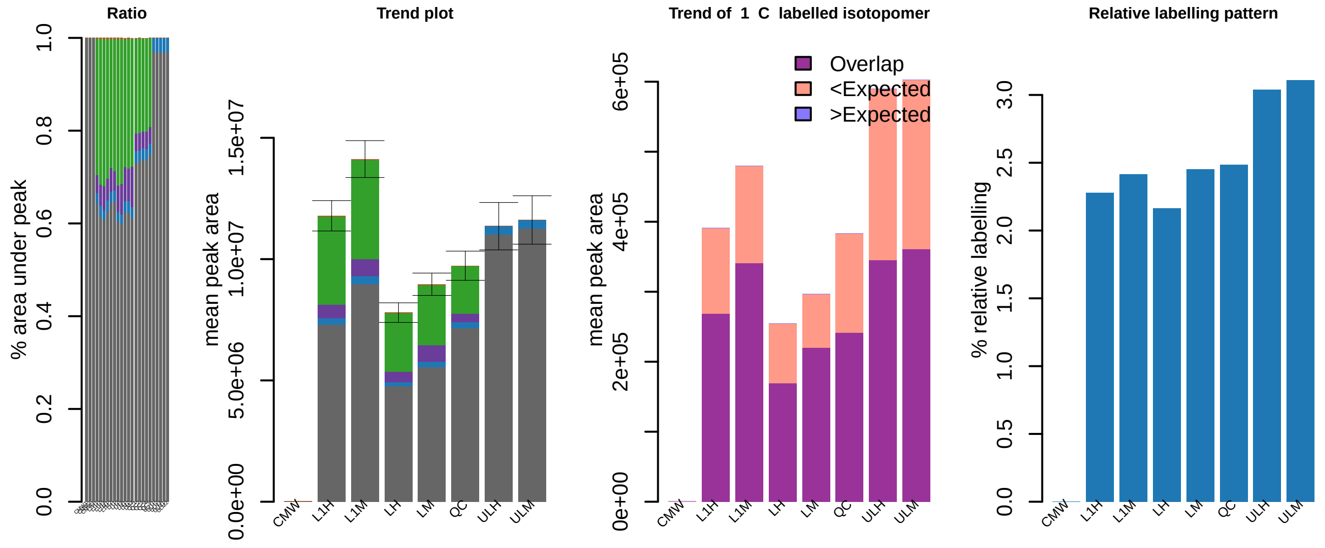

Hydroxymethylphosphonate

Formula: CH_5O_4P Mass: 111.993 Std.RT: 938.1511494 Ion: NEG

G1

UL +1

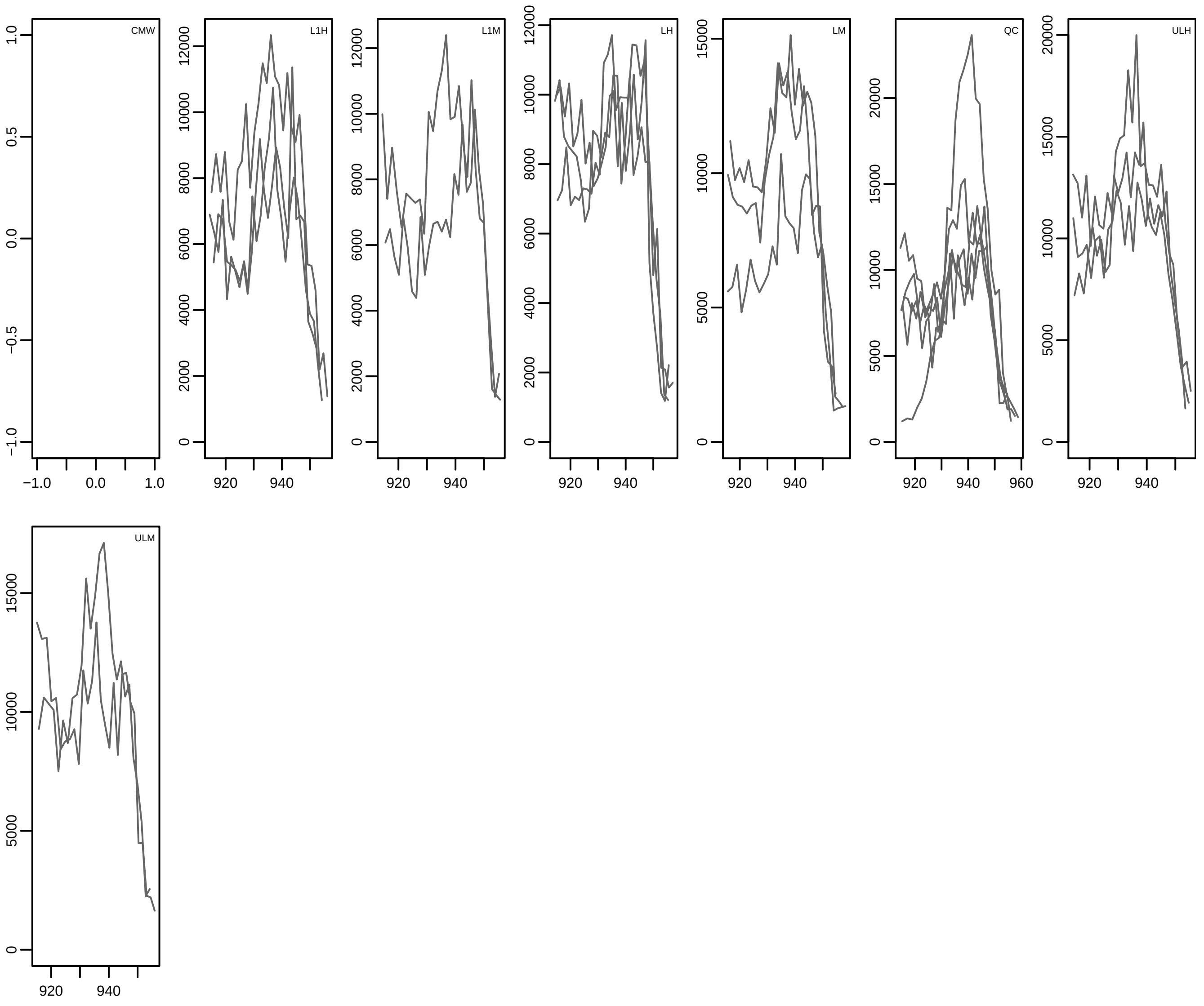

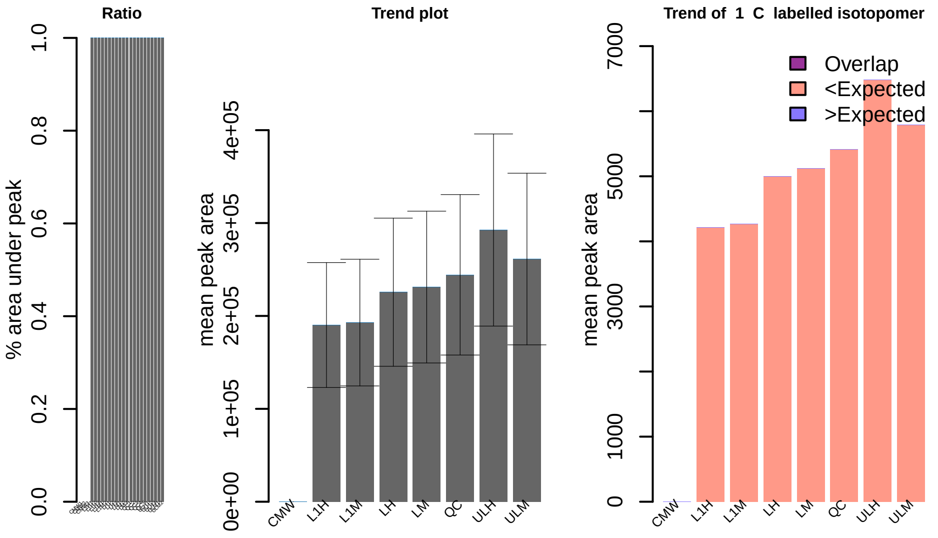

N−Acetyl−L−glutamate

Formula: C7H11NO5 Mass: 189.064 Std.RT: 957.1572468 Ion: NEG

G1

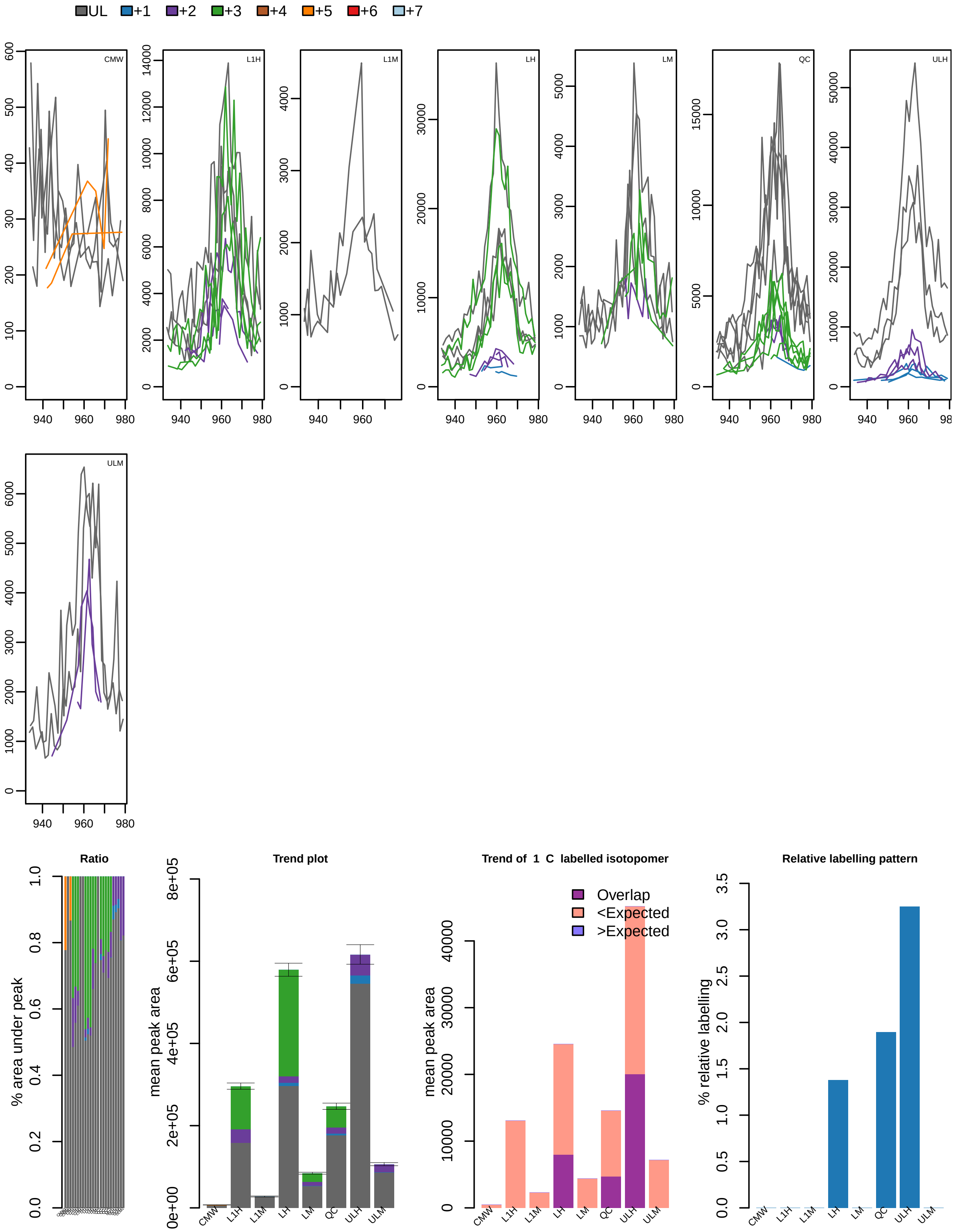

N2−Succinyl−L−ornithine

Formula: C9H16N2O5 Mass: 232.106 Std.RT: 829.7585652 Ion: NEC

G1

UL +1 +2 +3 +4 +5 +6 +7 +8 +9

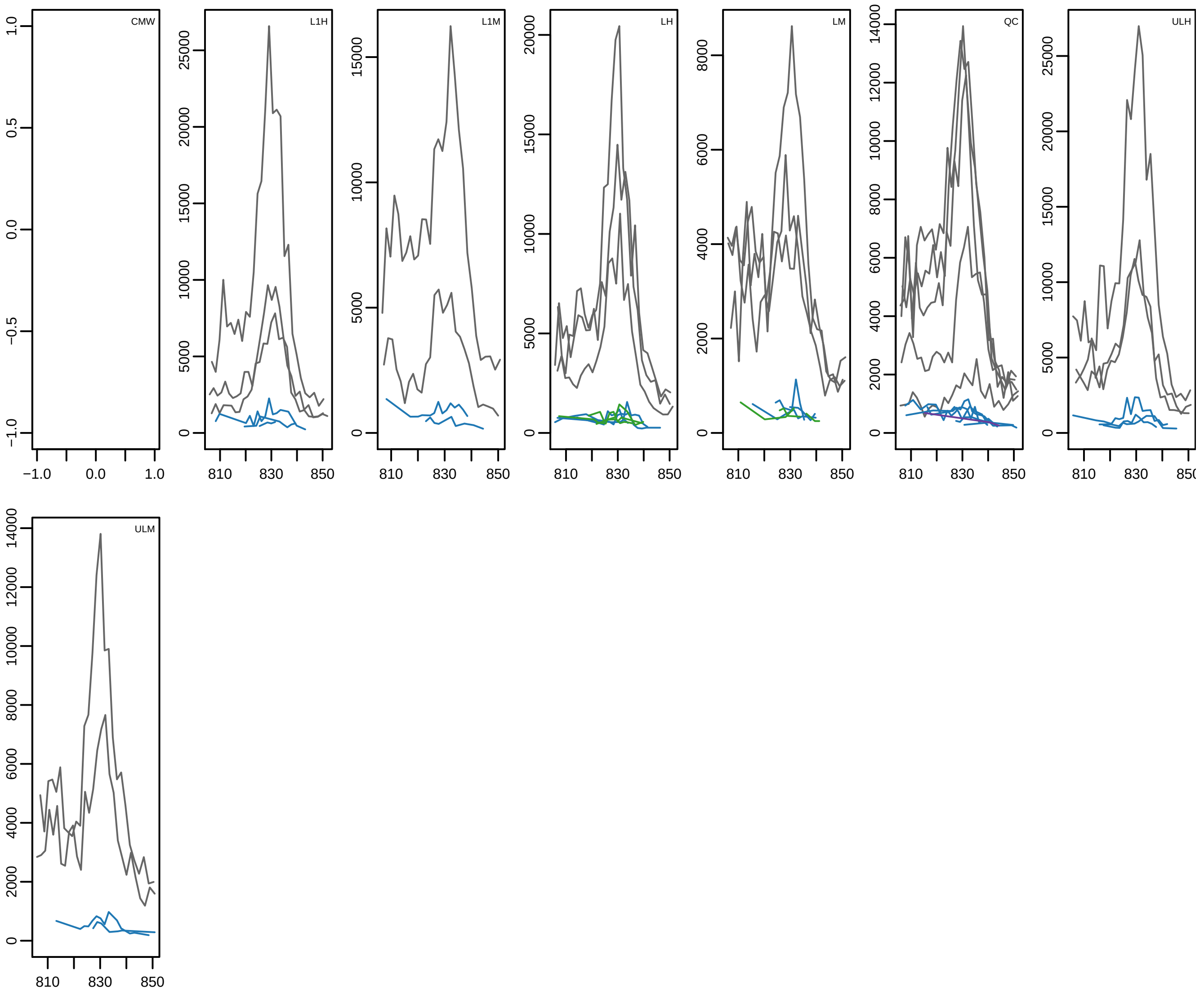

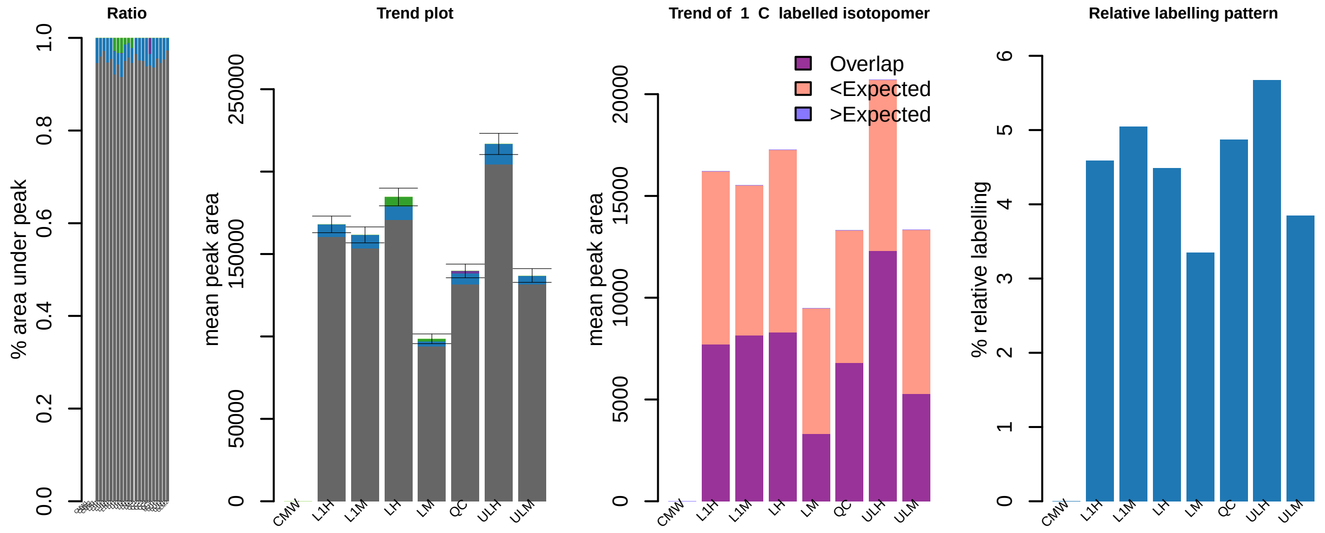

N2−Succinyl−L−ornithine

Formula: C9H16N2O5 Mass: 232.106 Std.RT: 829.7585652 Ion: NEC

G2

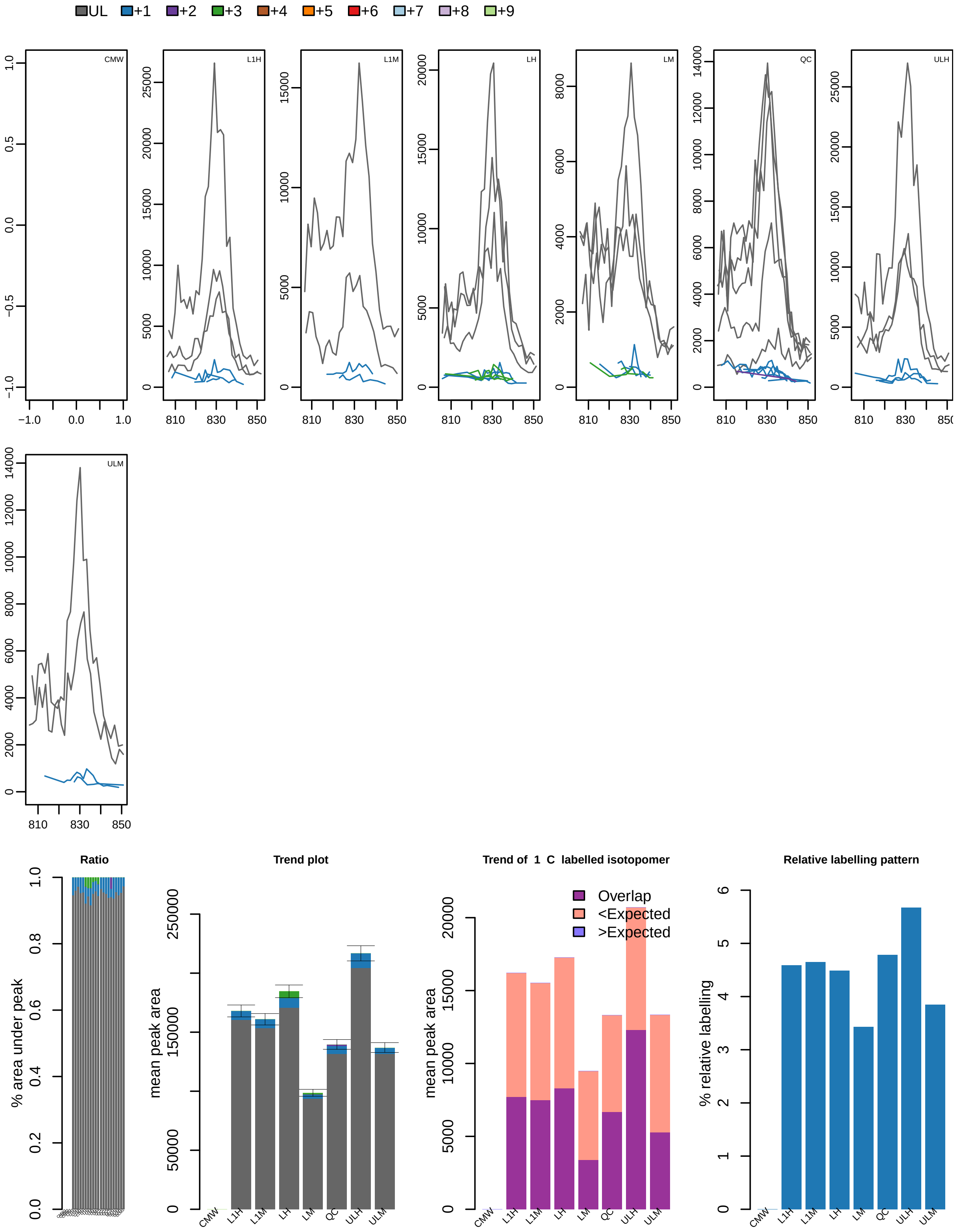

L−1−Pyrroline−3−hydroxy−5−carboxylate

Formula: C5H7NO3 Mass: 129.043 Std.RT: 628.0602492 Ion: NEG

G1

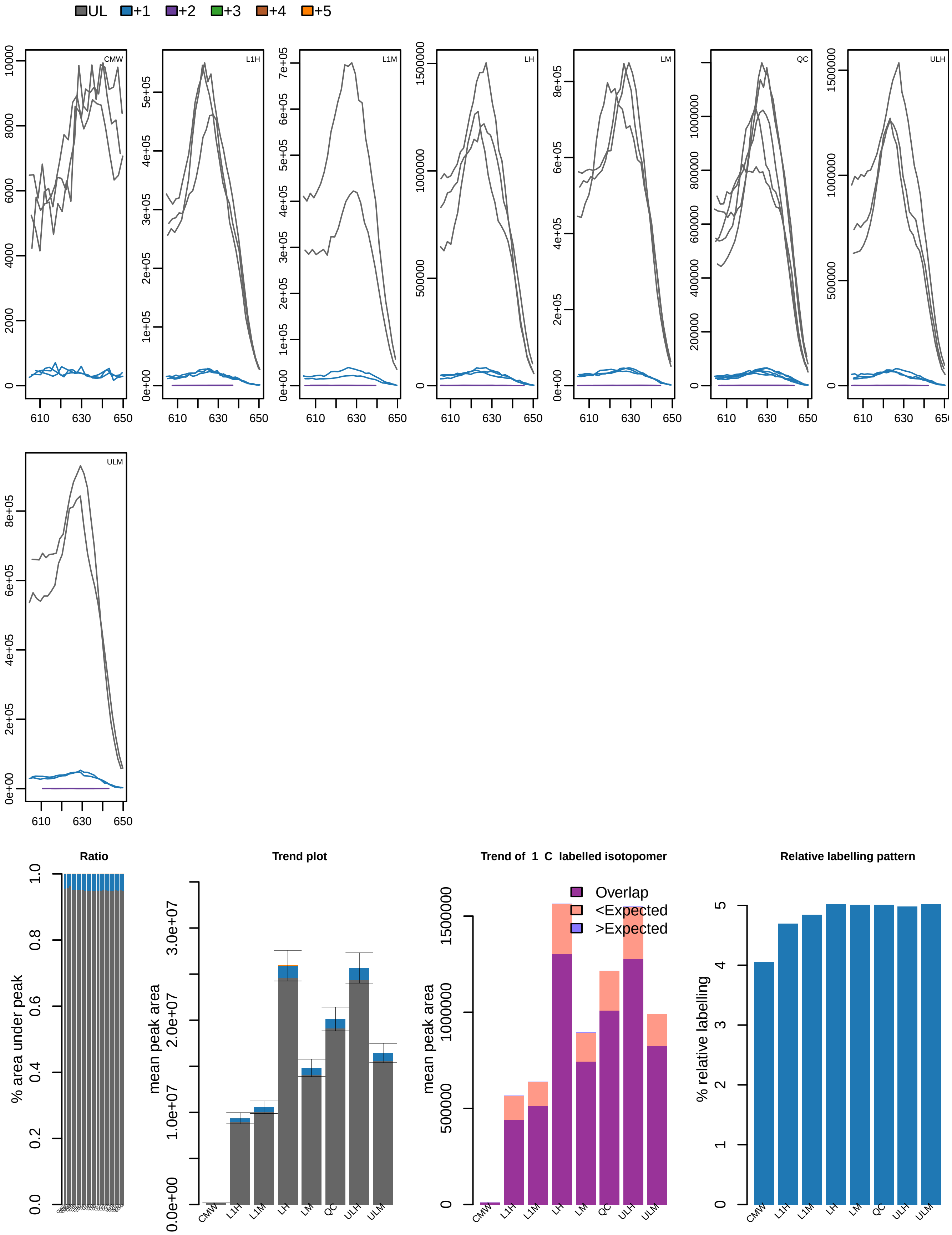

Urea−1−carboxylate

Formula: C2H4N2O3 Mass: 104.022 Std.RT: 667.6412556 Ion: NEG

G1

UL +1 +2

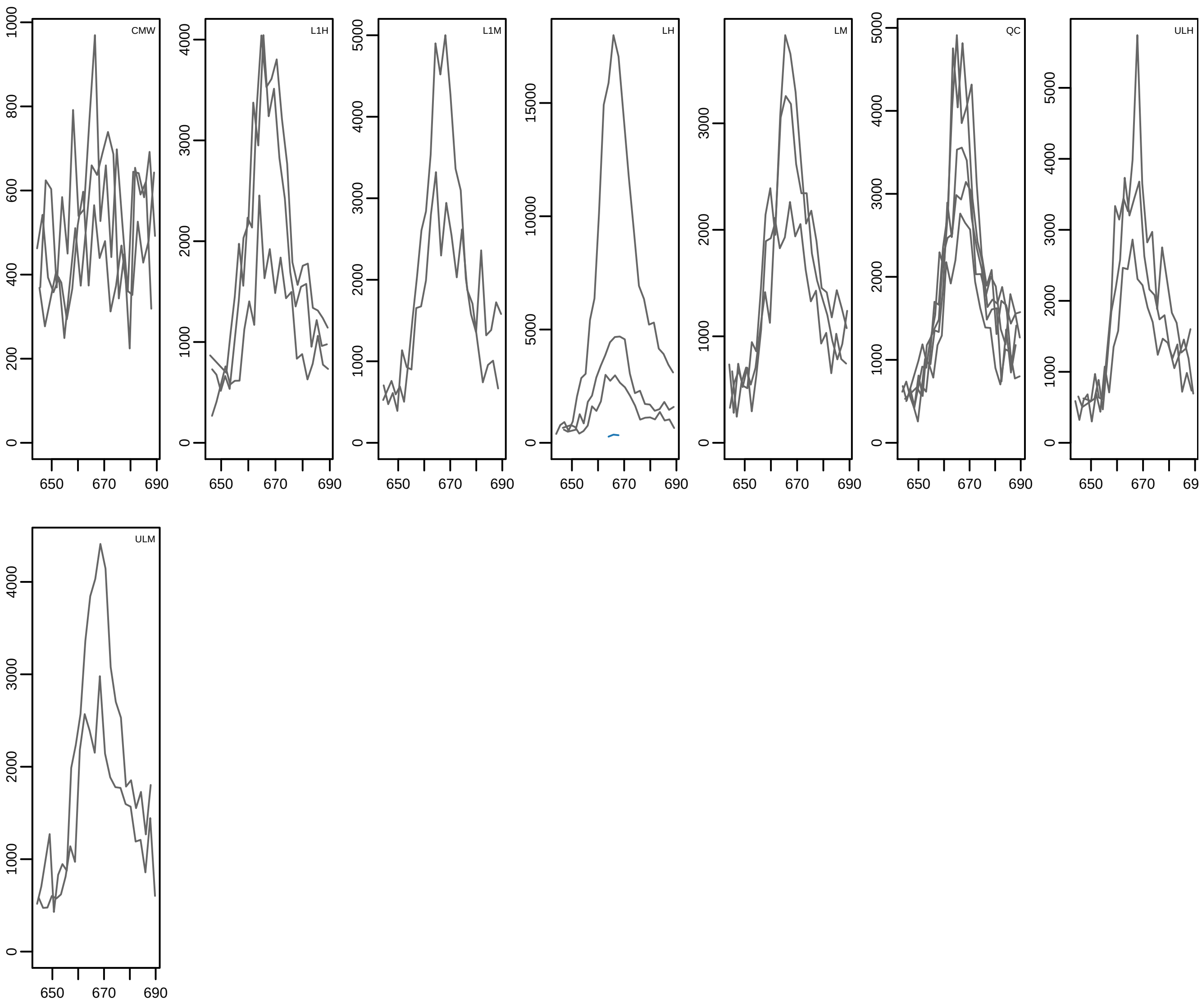

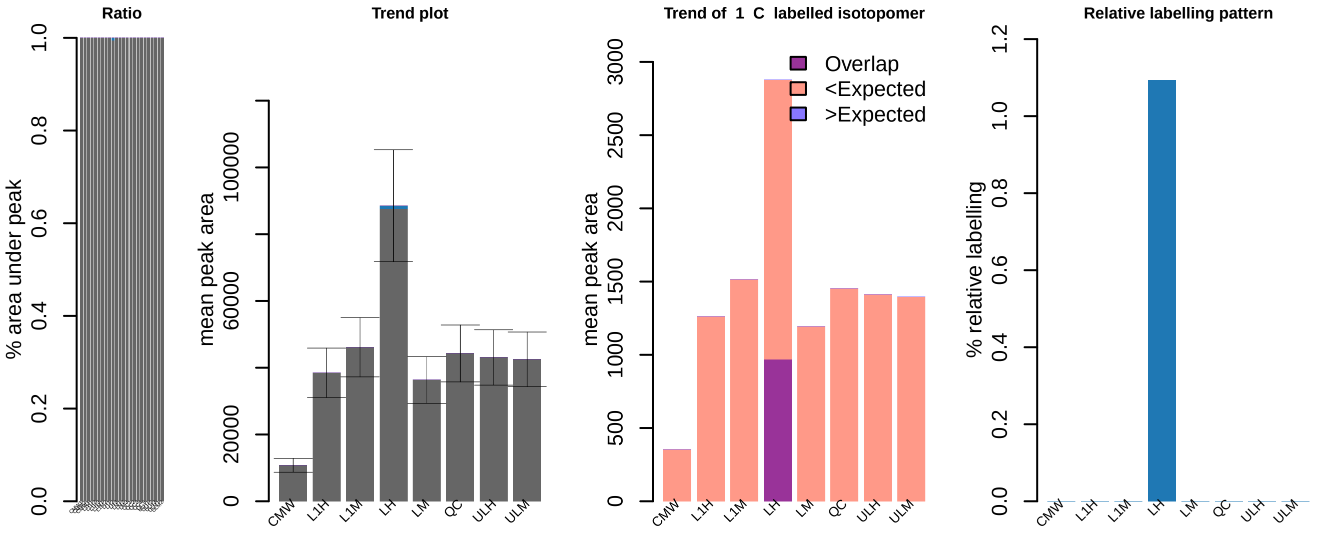

L−Glutamate

G1

Formula: C5H9NO4 Mass: 147.053 Std.RT: 952.8405024 Ion: NEG

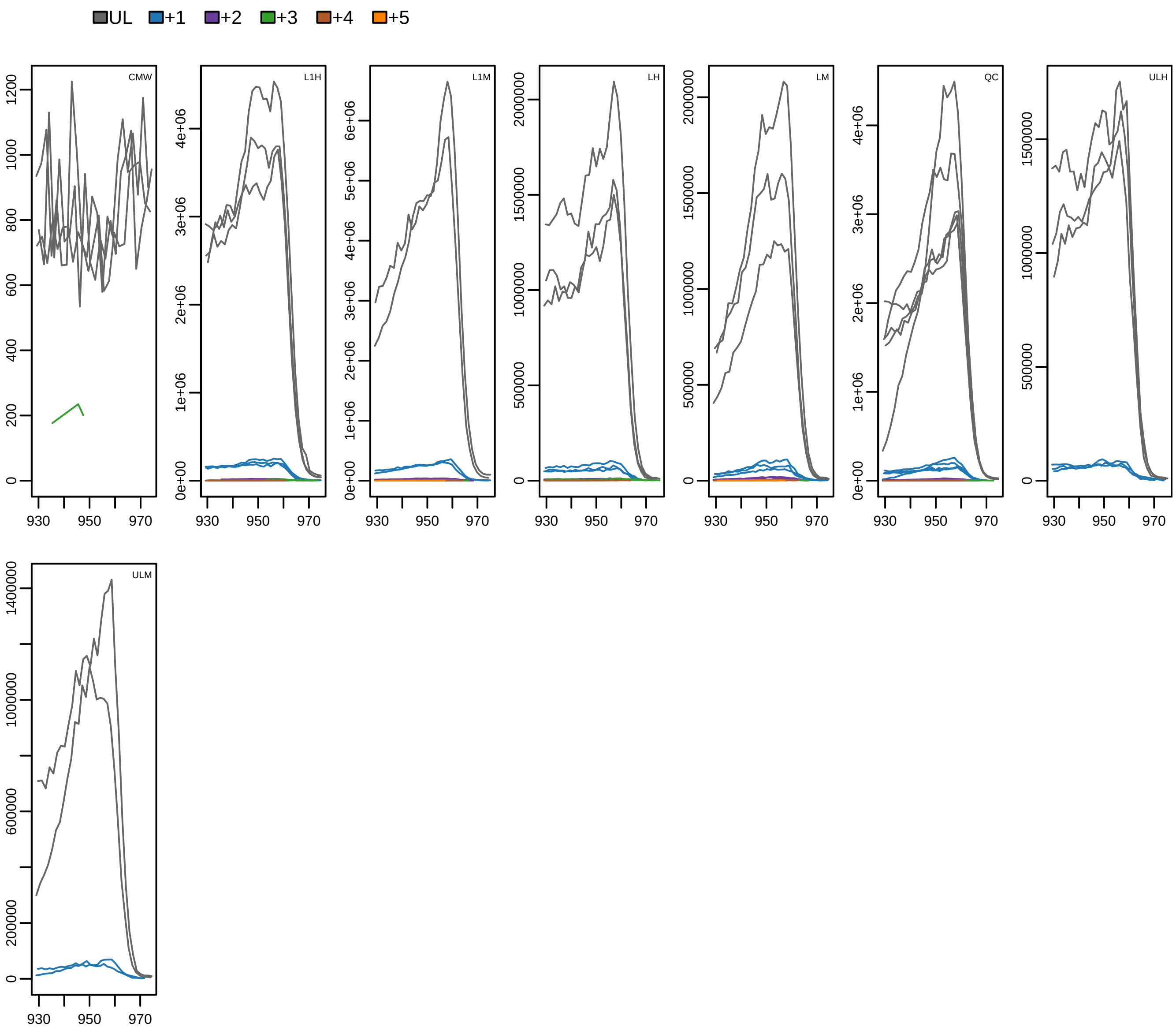

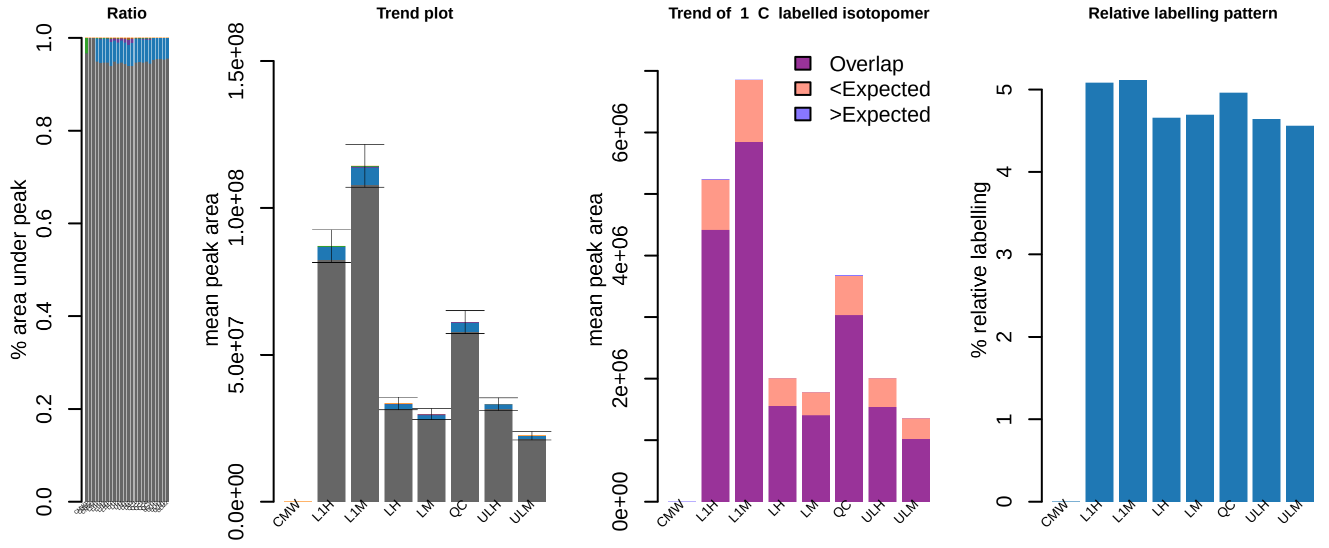

Carbamoyl phosphate

G1

Formula: CH4NO5P Mass: 140.983 Std.RT: 2151.8066508 Ion: NEG

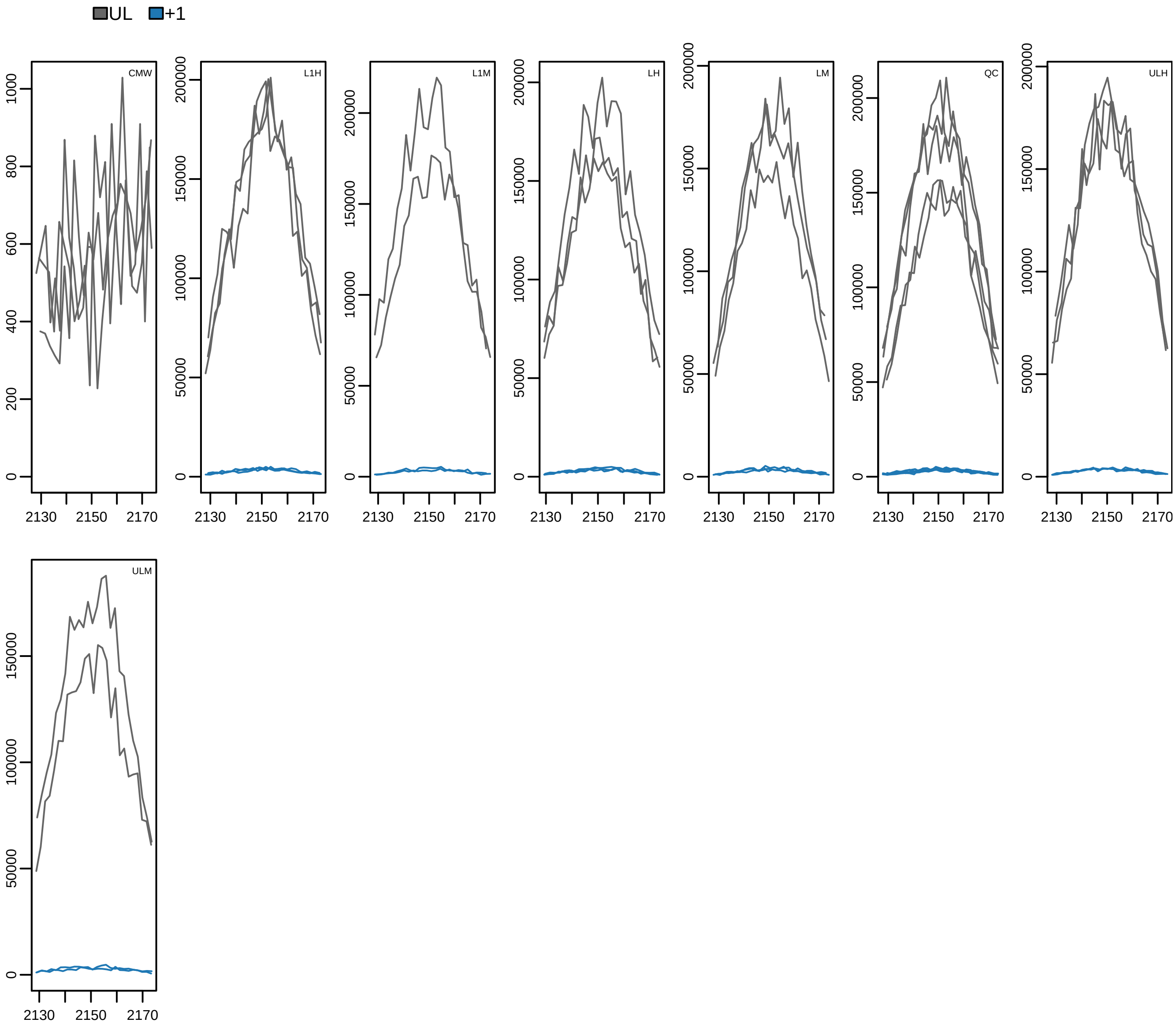

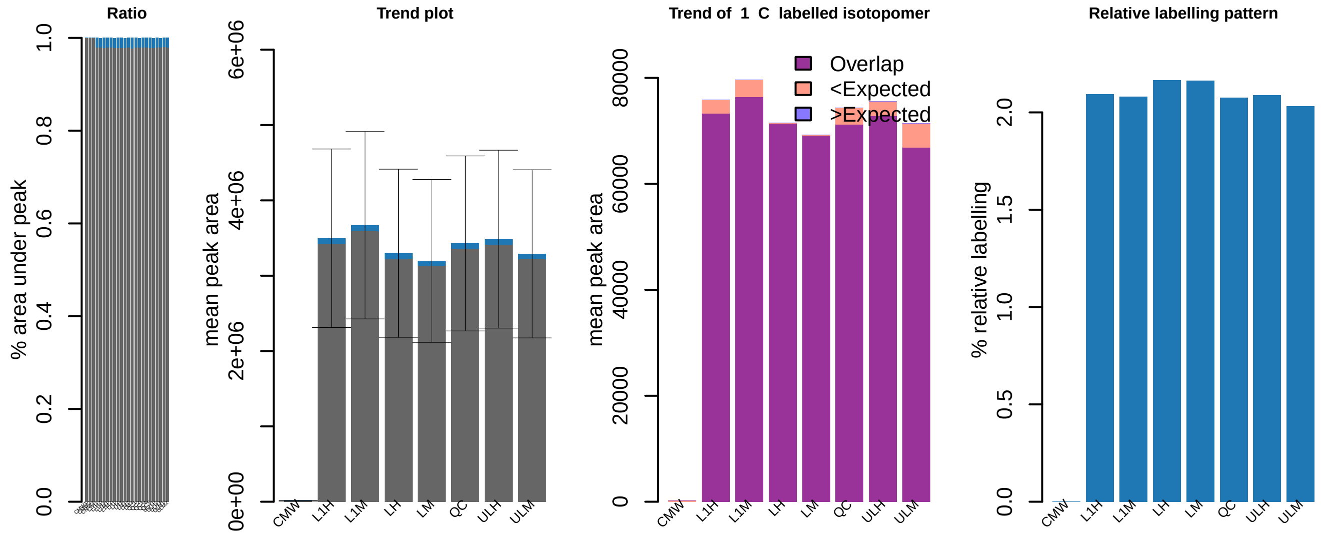

Glycine

Formula: C2H5NO2 Mass: 75.032 Std.RT: 1016.4774222 Ion: NEG

G1

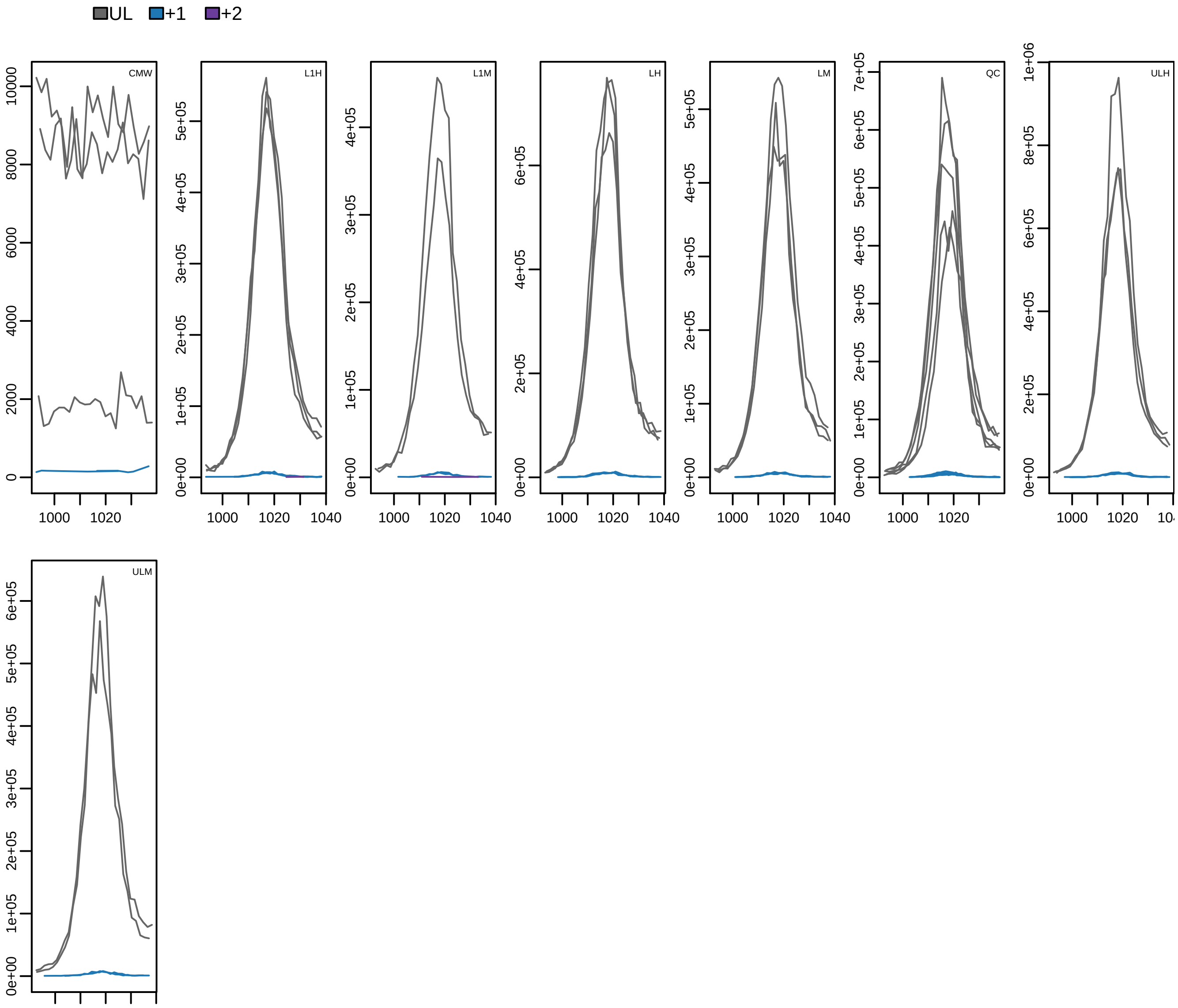

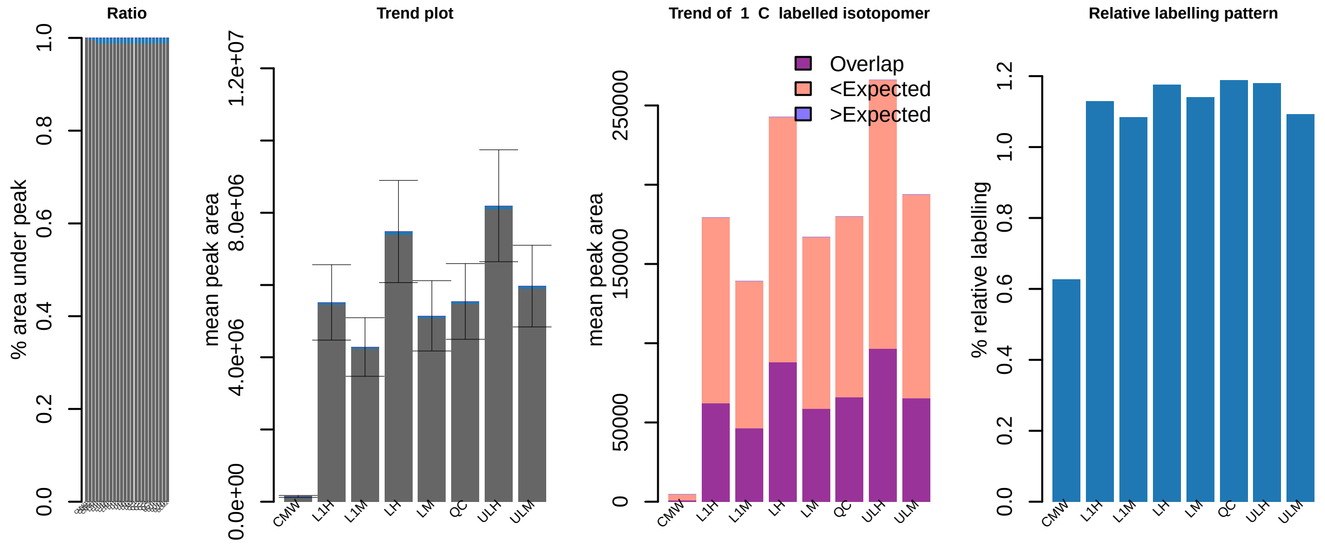

O−Acetyl−L−serine

Formula: C5H9NO4 Mass: 147.053 Std.RT: 667.6364976 Ion: NEG

G1

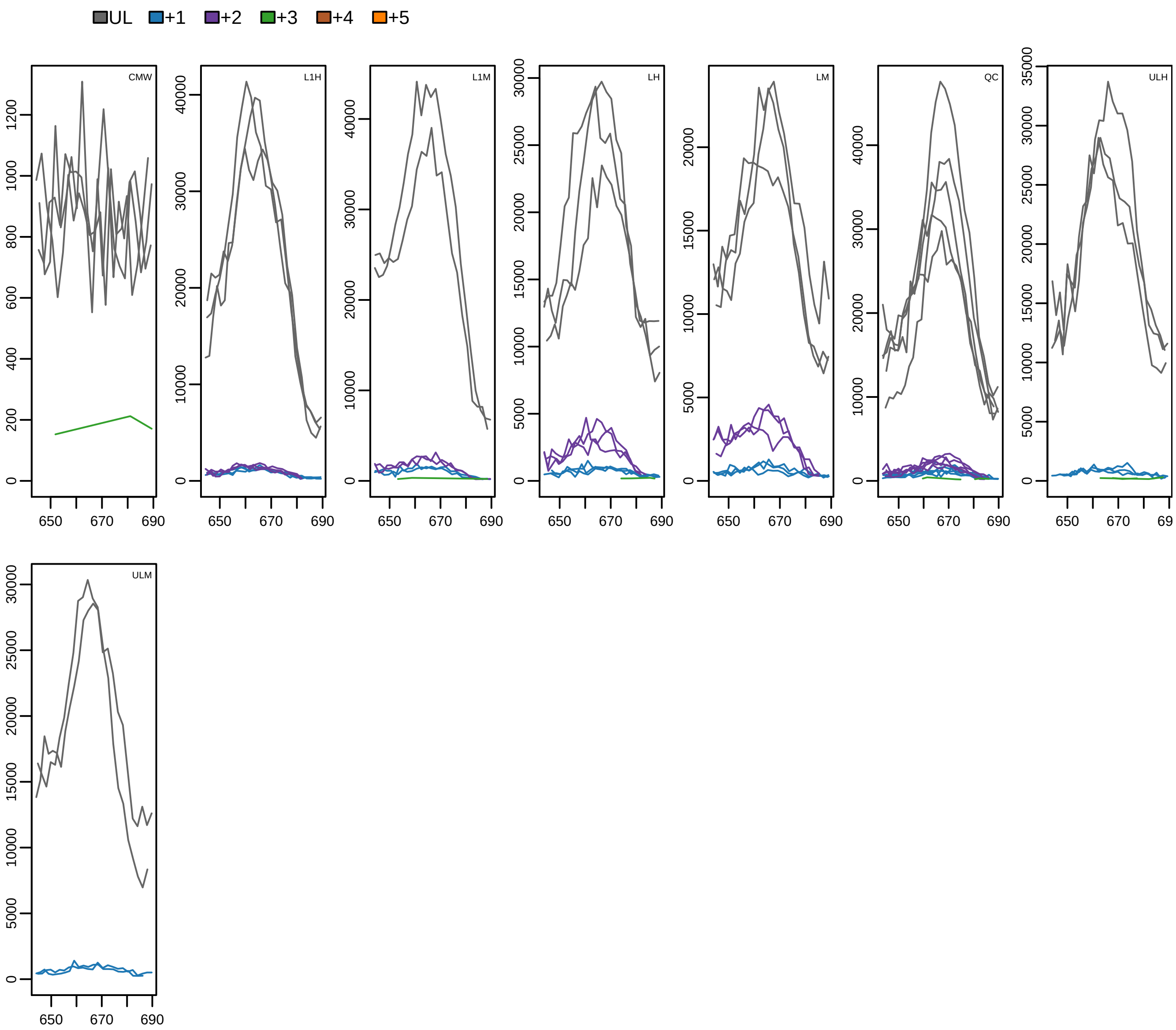

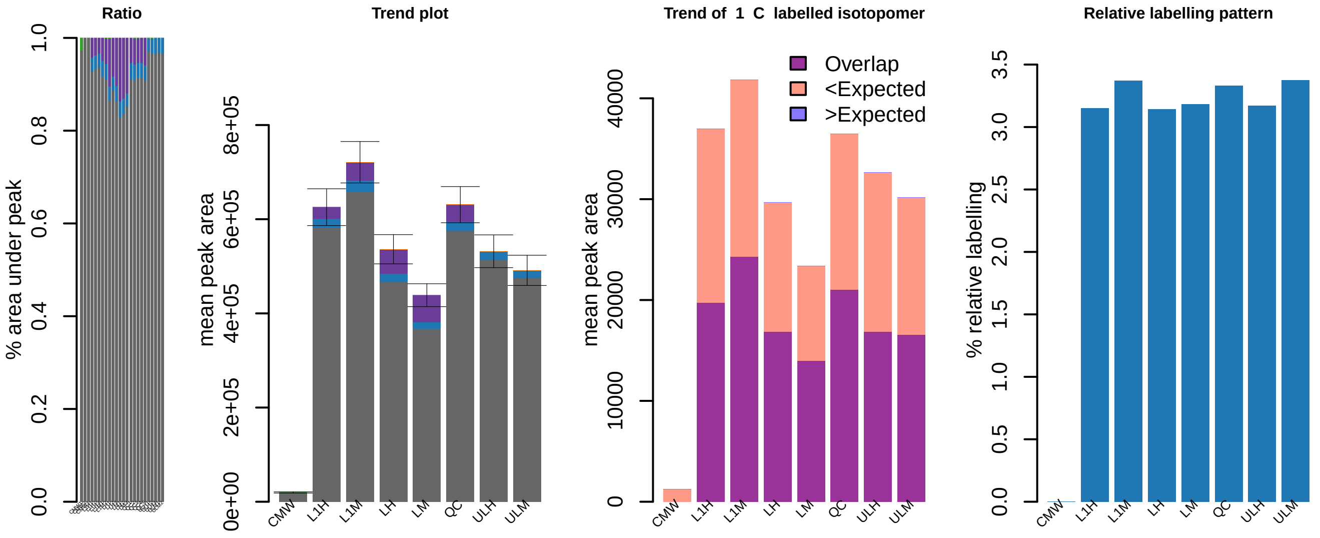

(R)−2−Hydroxyglutarate

Formula: C5H8O5 Mass: 148.037 Std.RT: 964.0135038 Ion: NEG

G1

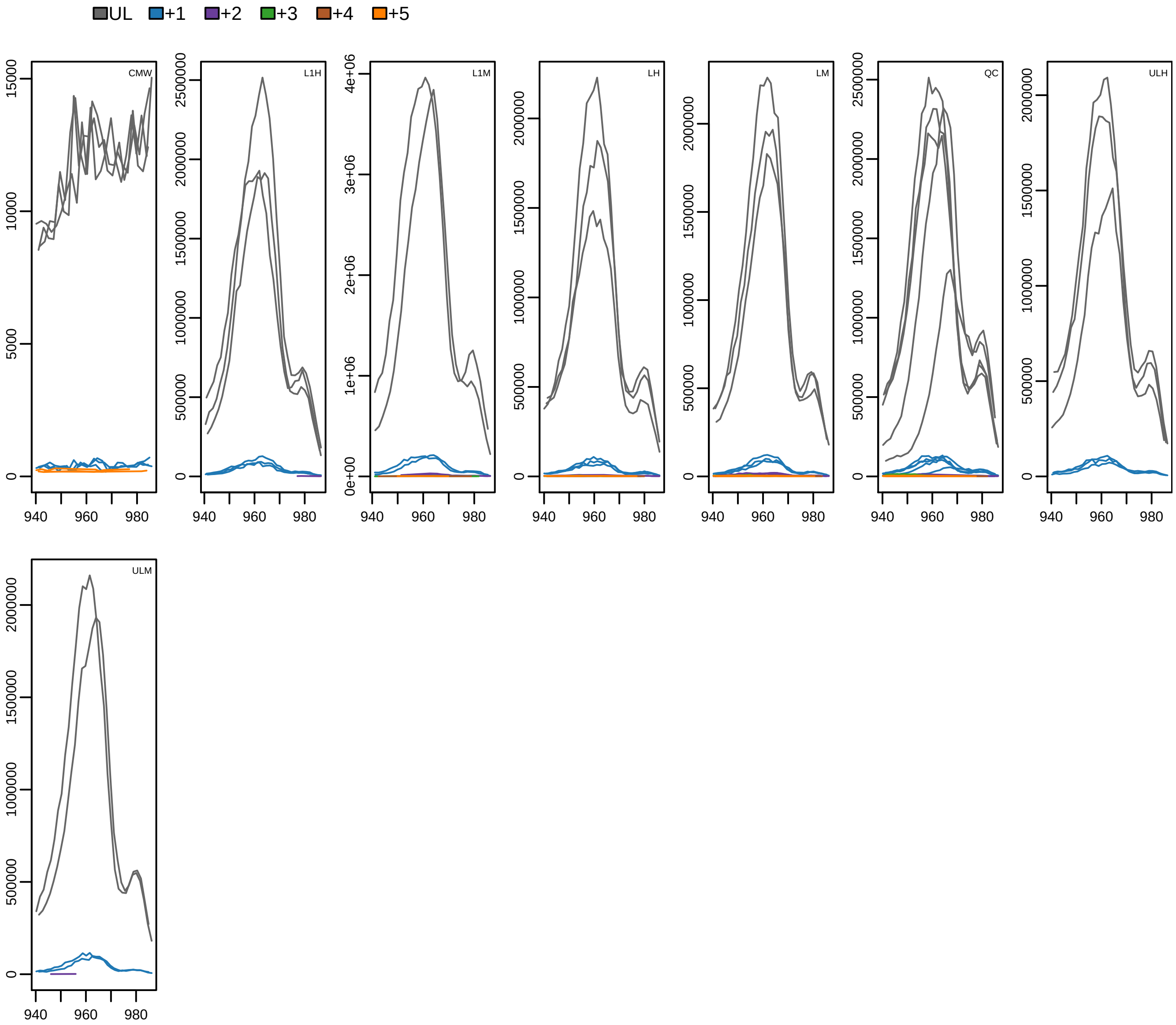

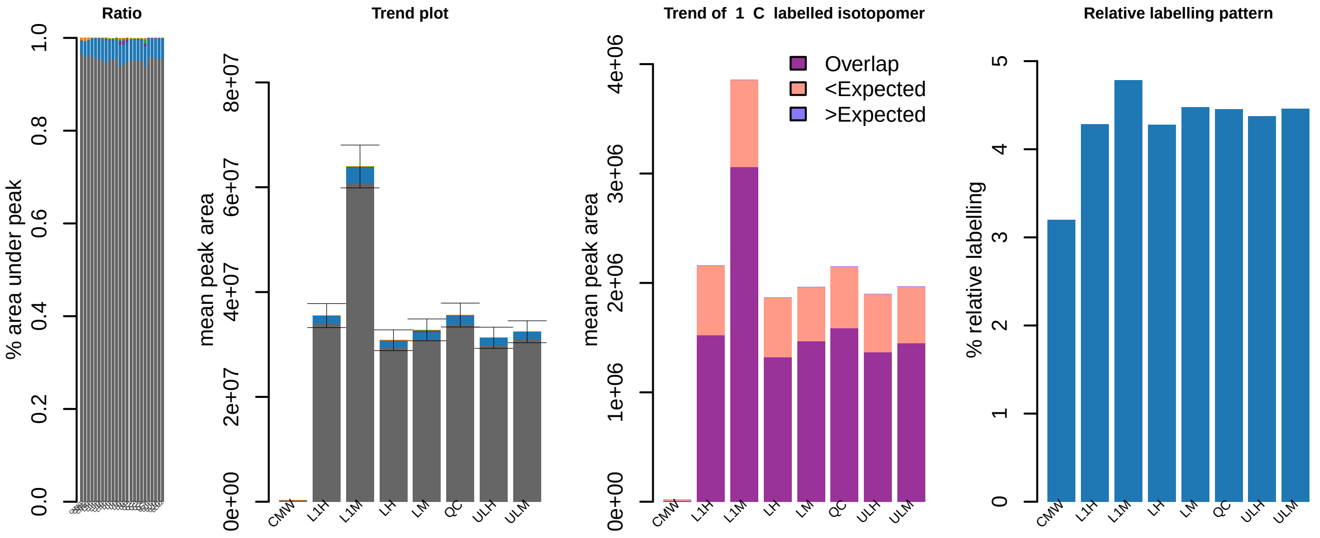

2−Oxoglutaramate

Formula: C5H7NO4 Mass: 145.038 Std.RT: 641.5785042 Ion: NEG

G1

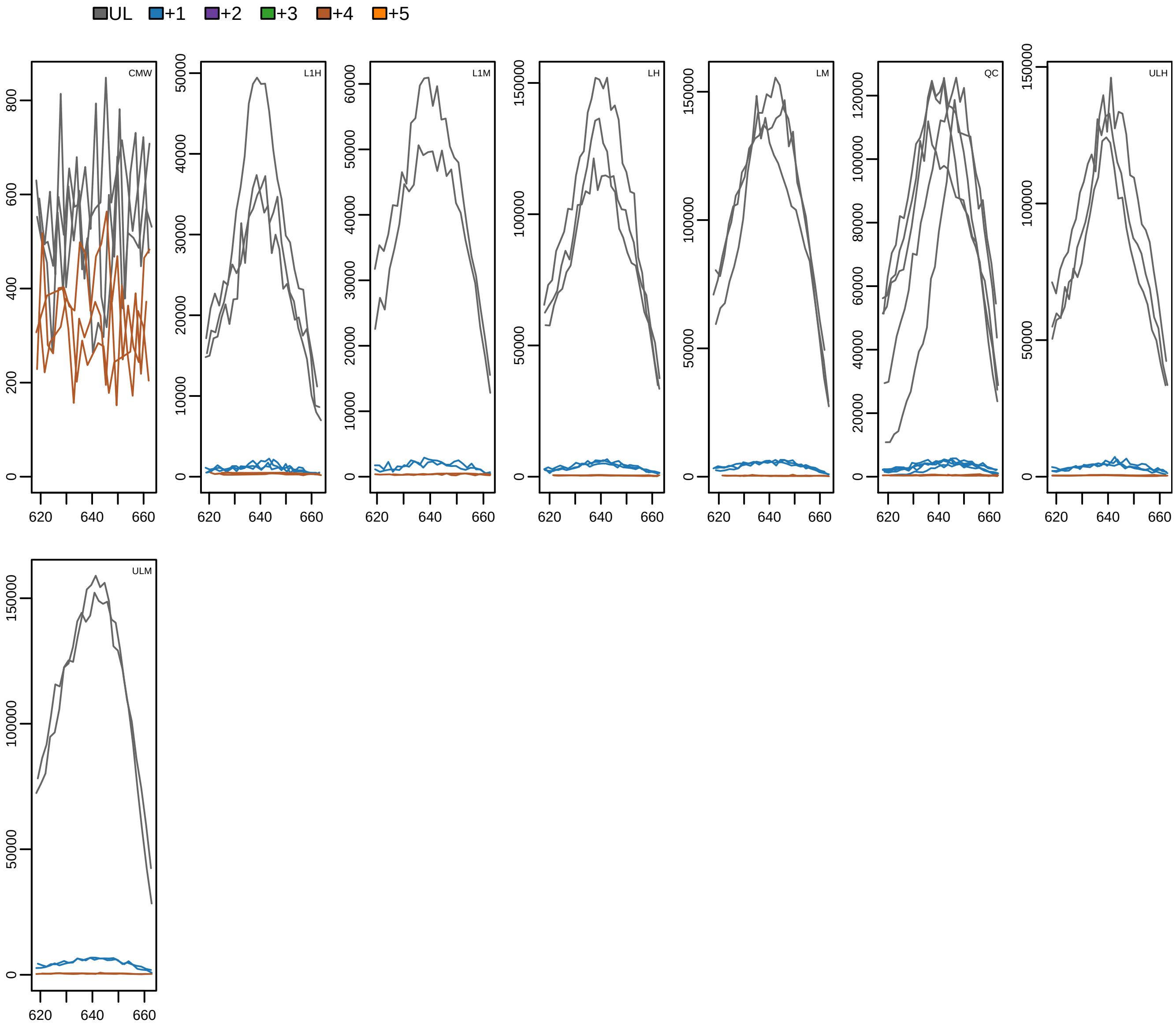

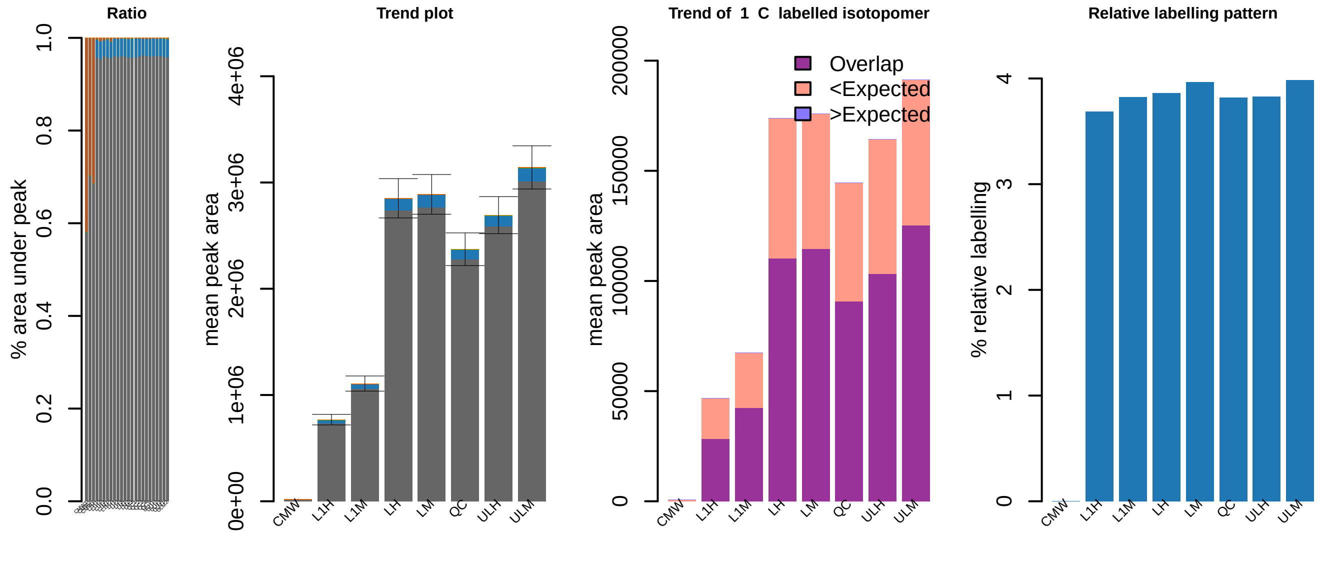

N−Acetyl−D−glucosamine 6−phosphate

Formula: C8H16NO9P Mass: 301.056 Std.RT: 986.2202934 Ion: NE[illegible]

G1

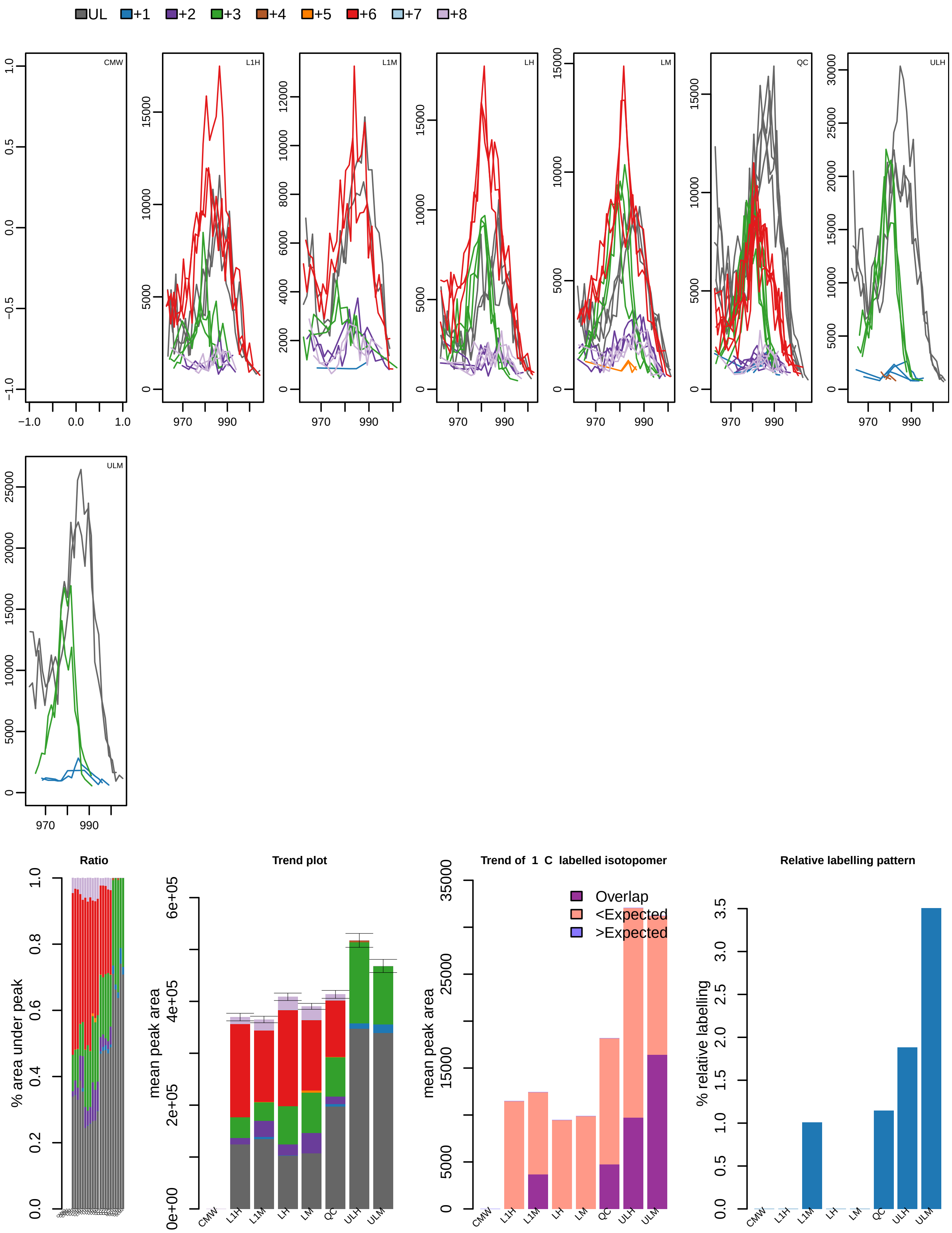

D−Glucosamine 6−phosphate

Formula: C6H14NO8P Mass: 259.046 Std.RT: 1003.4276148 Ion: NE

G1

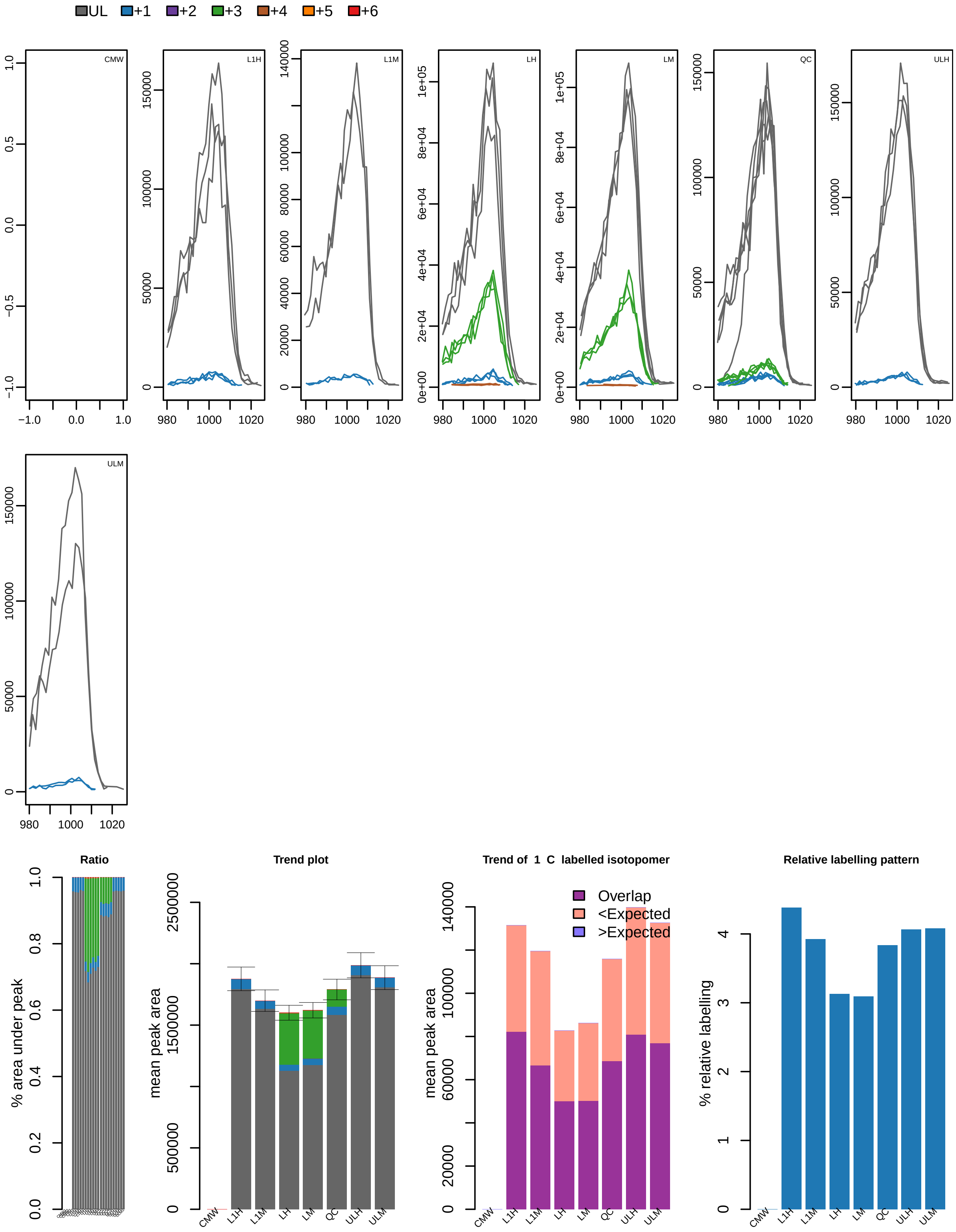

5−Oxoproline

Formula: C5H7NO3 Mass: 129.043 Std.RT: 806.964249 Ion: NEG

G1

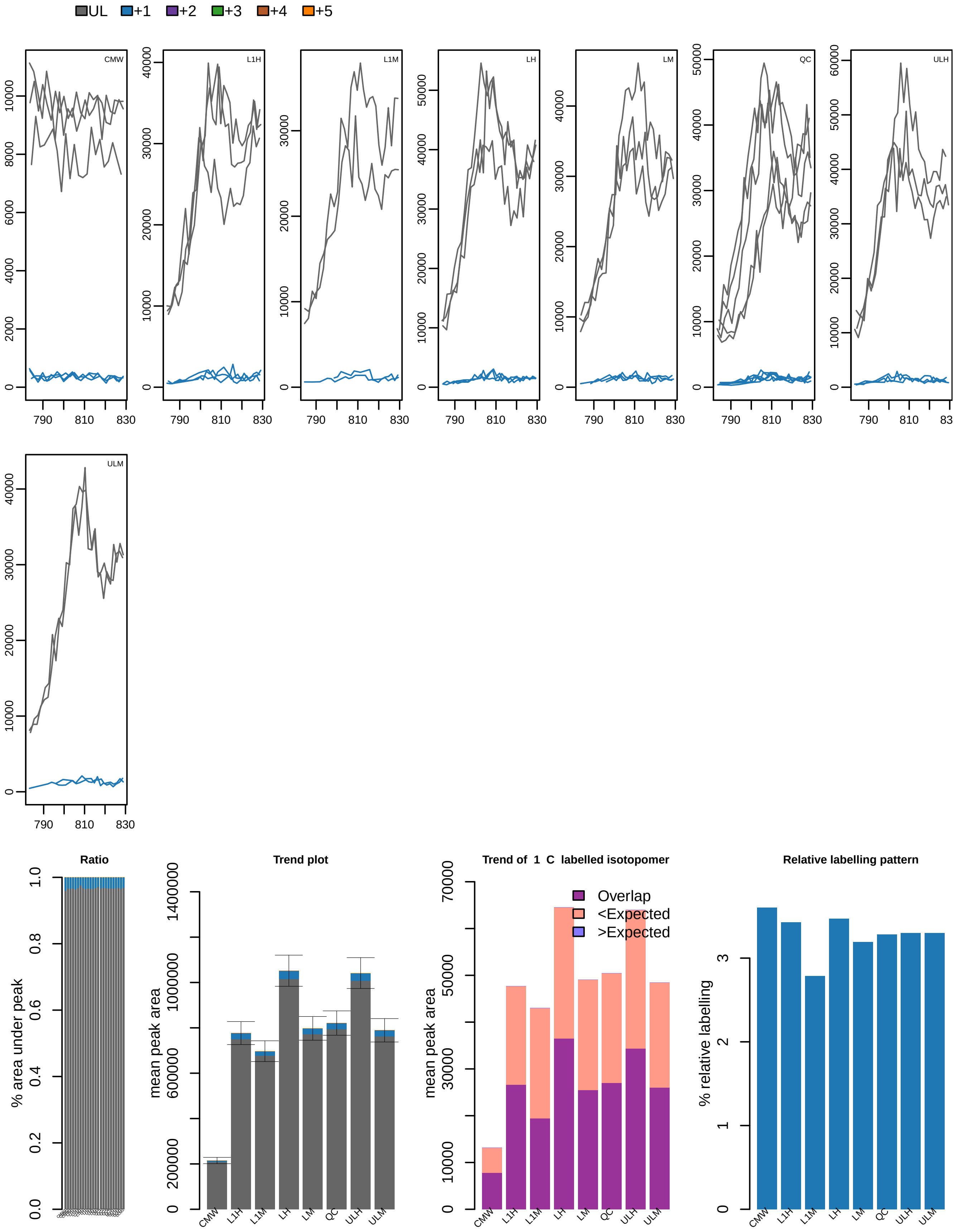

Ethanolamine phosphate

Formula: C2H8NO4P Mass: 141.019 Std.RT: 1049.5780902 Ion: NE(

G1

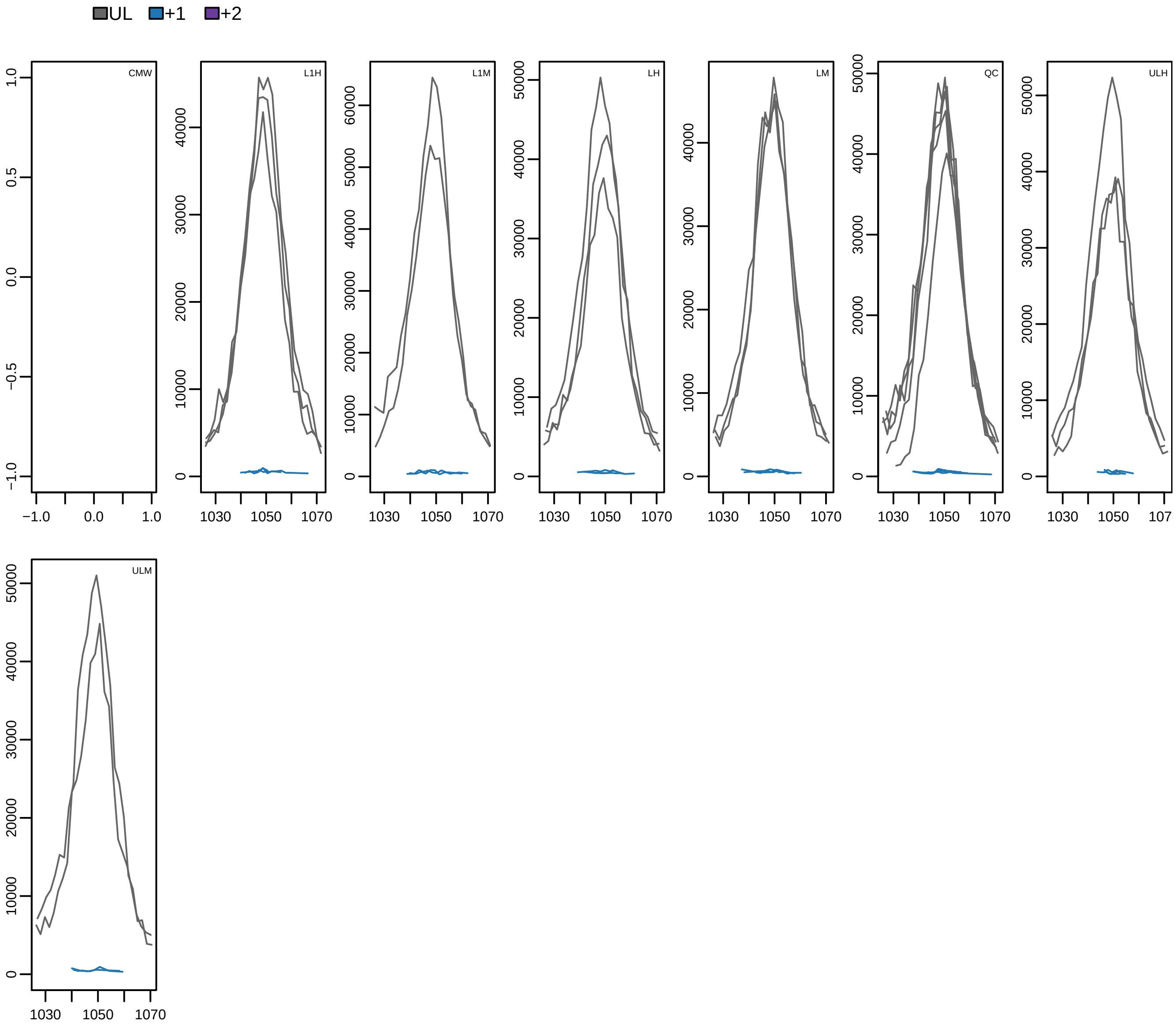

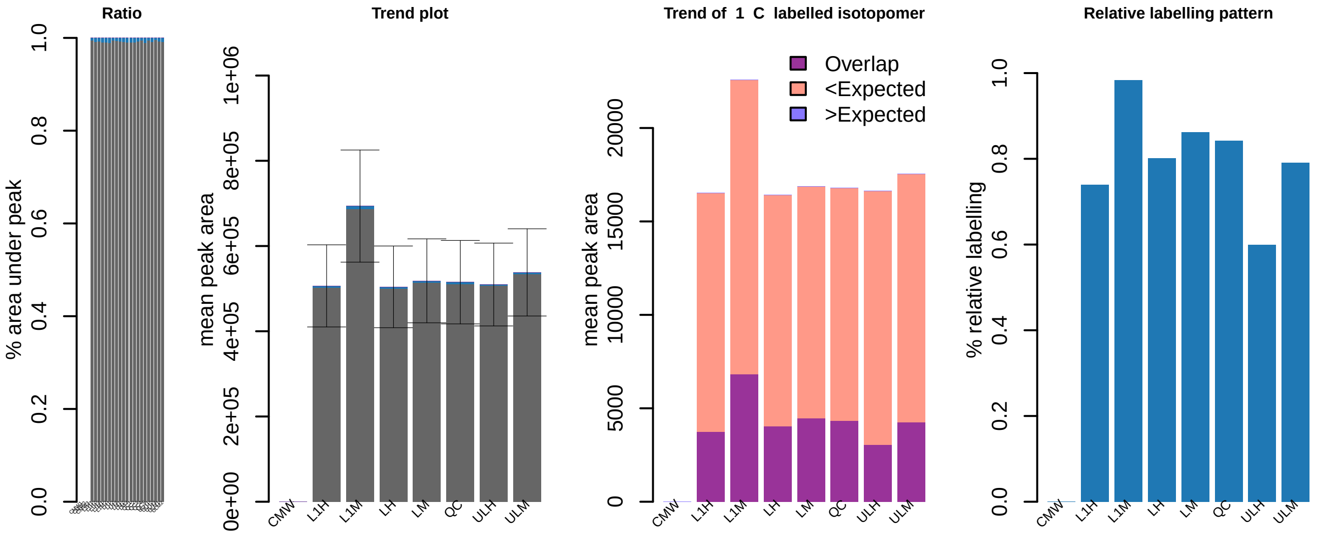

(1−Ribosylimidazole)−4−acetate

Formula: C10H14N2O6 Mass: 258.085 Std.RT: 902.0539986 Ion: NE

G1

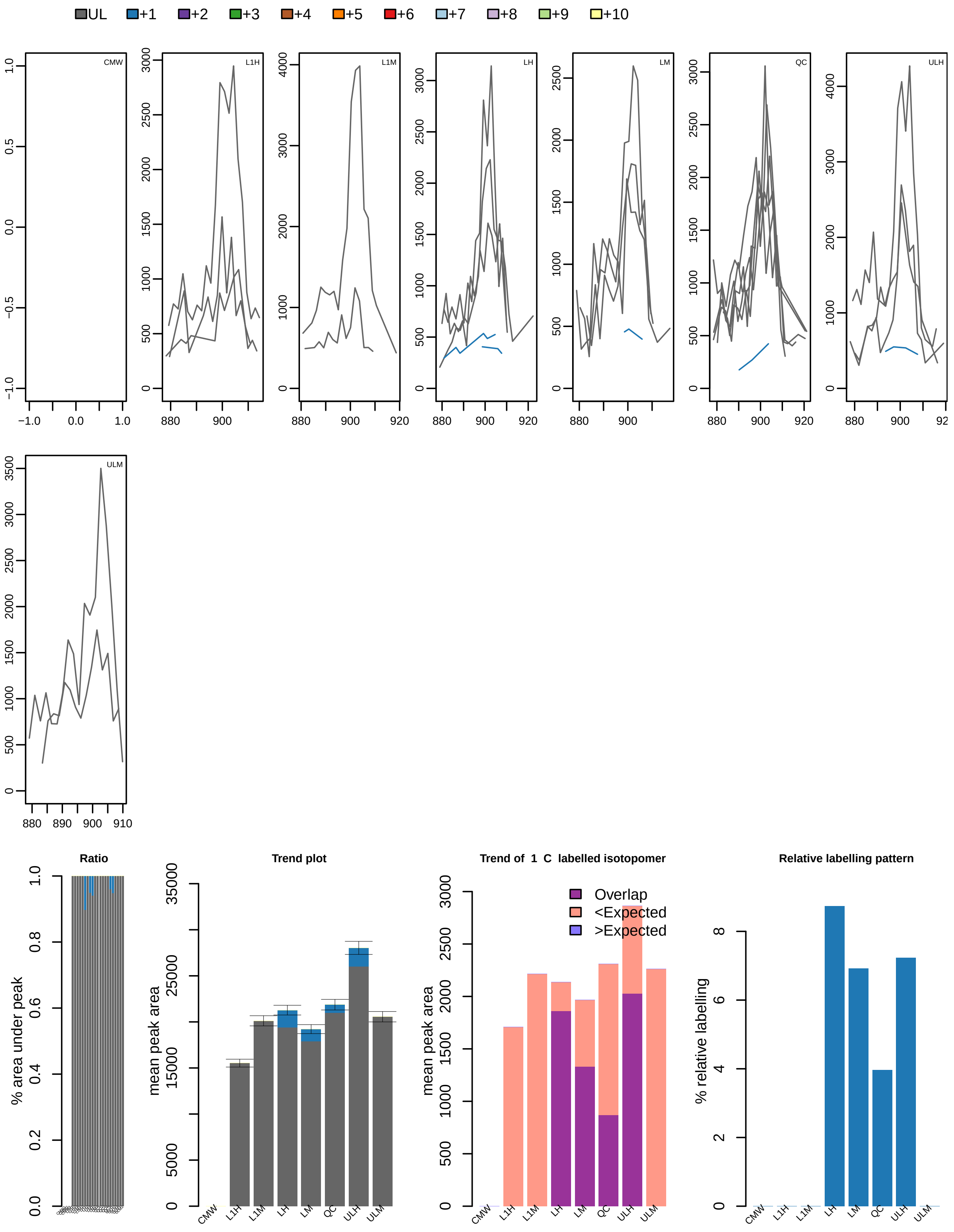

4−Imidazolone−5−acetate

Formula: C5H6N2O3 Mass: 142.038 Std.RT: 804.5667546 Ion: NEG

G1

UL +1 +2 +3 +4 +5

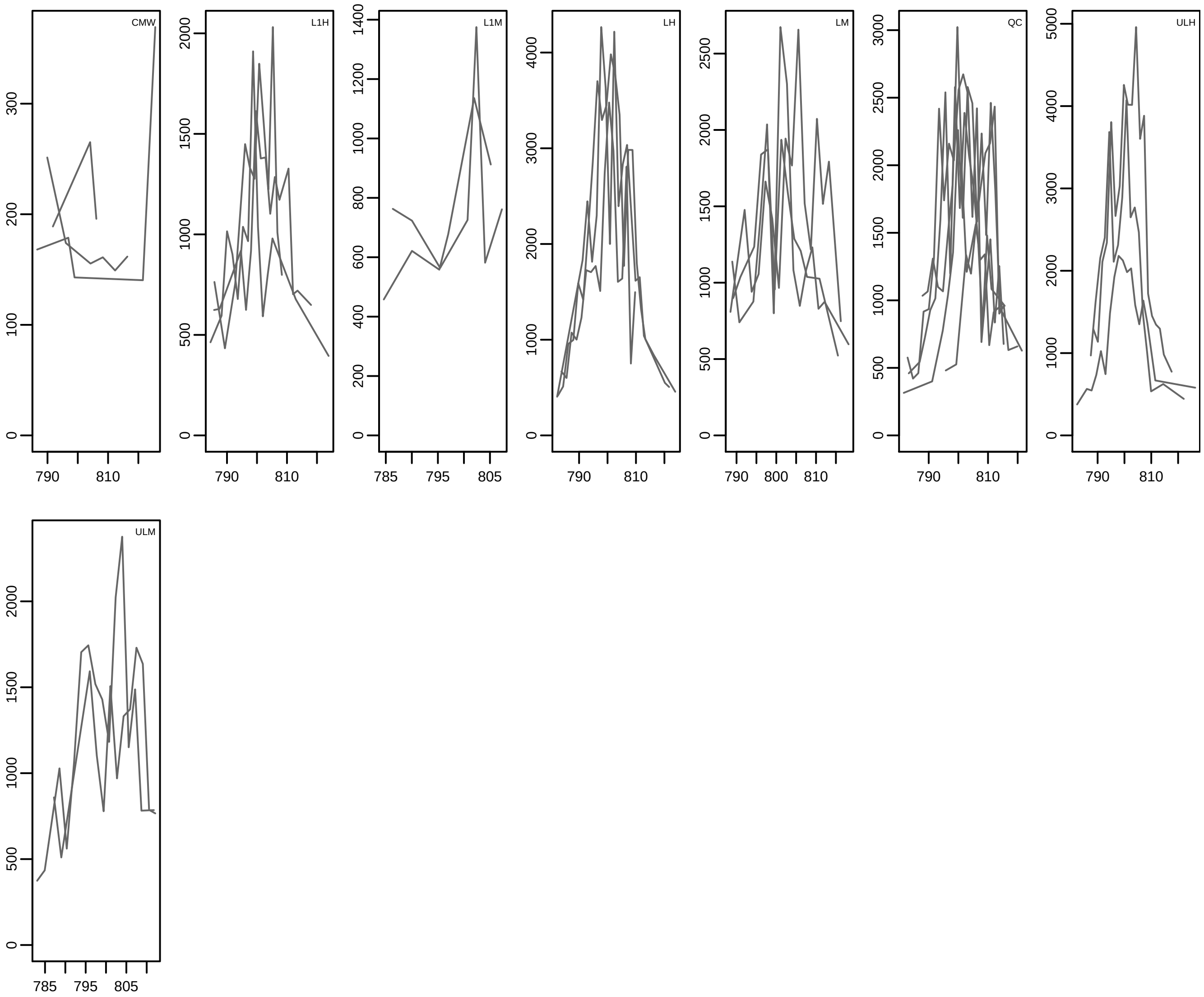

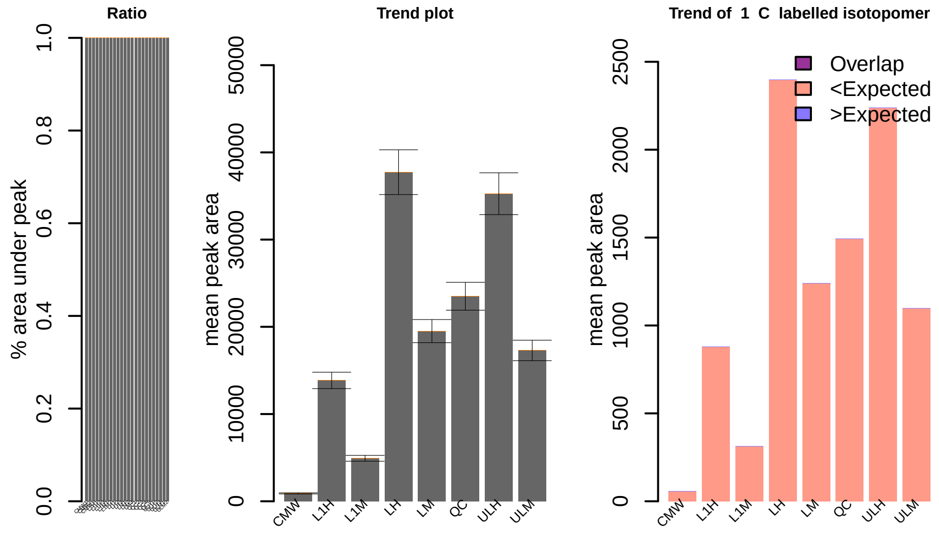

N−Succinyl−LL−2,6−diaminoheptanedioate

Formula: C11H18N2O7 Mass: 290.111 Std.RT: 815.8988592 Ion: NE

G1

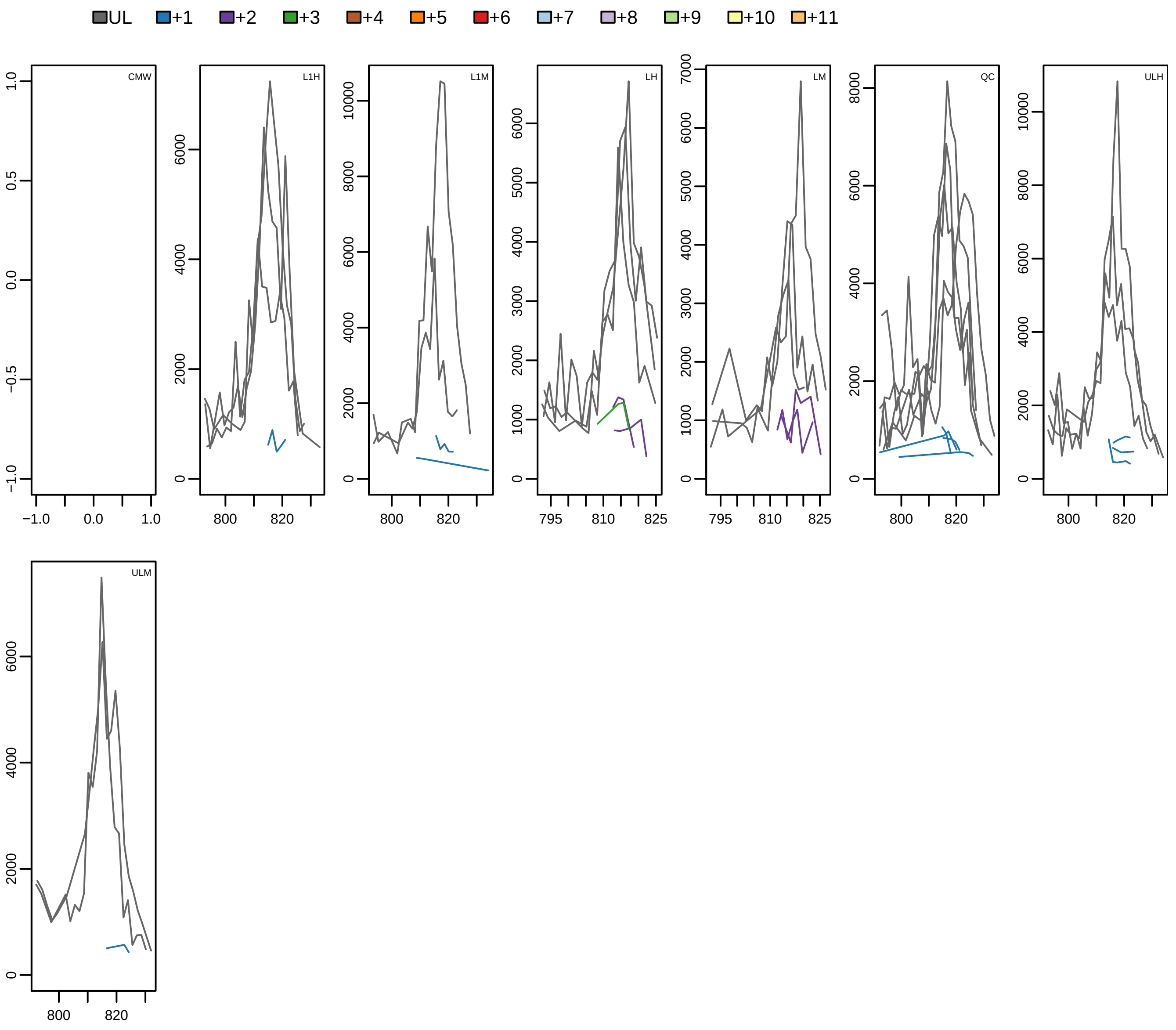

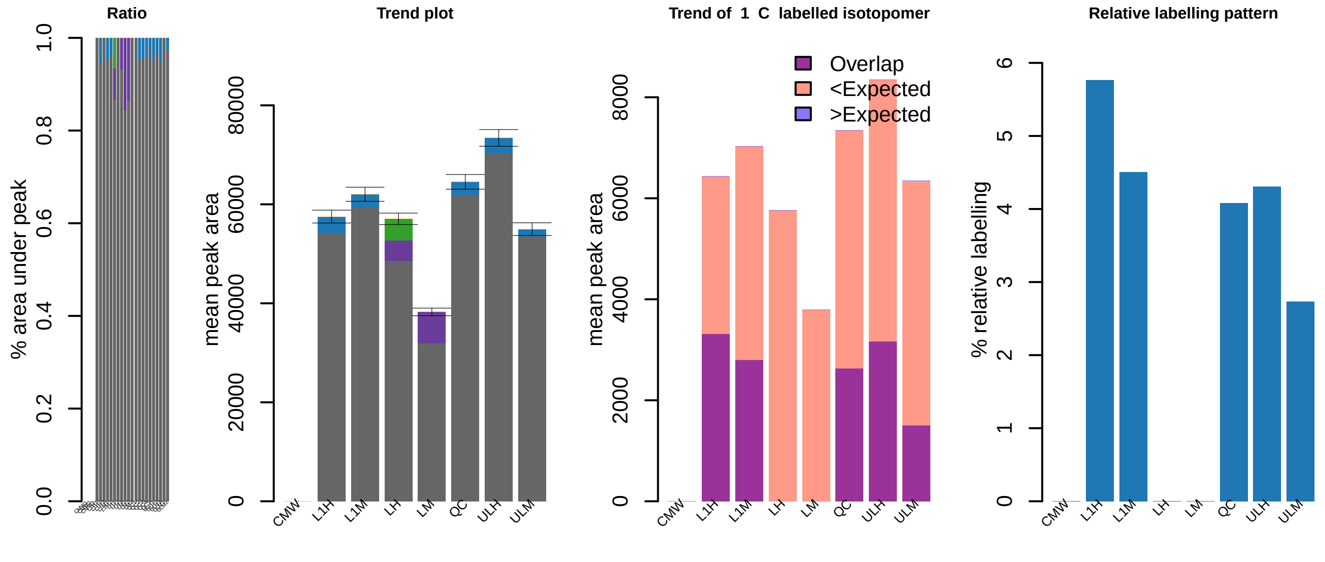

5−Oxopentanoate

G1

Formula: $C_5H_8O_3$ Mass: 116.047 Std.RT: 358.27246092 Ion: NEG

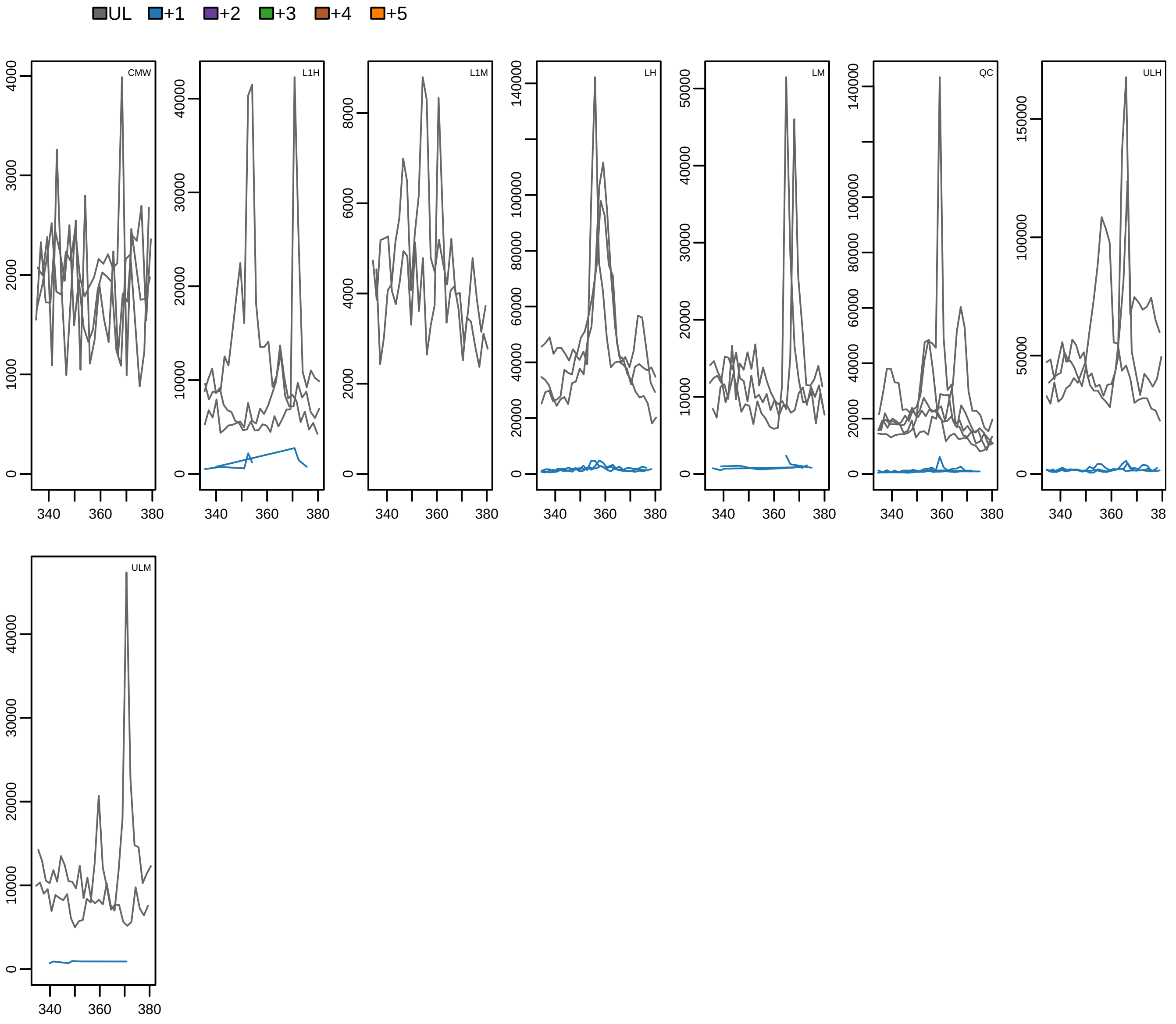

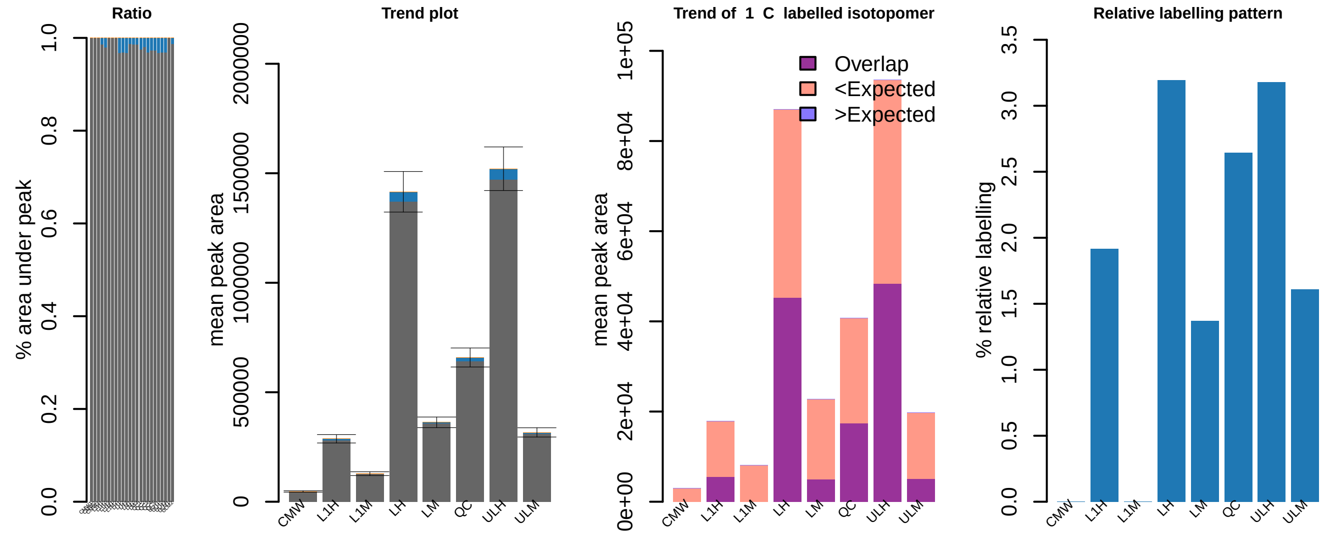

[FA oxo,methyl(4:0)] 2−oxo−4−methylthio−butanoic acic

Formula: C5H8O3S Mass: 148.019 Std.RT: 270.13081002 Ion: NEG

G1

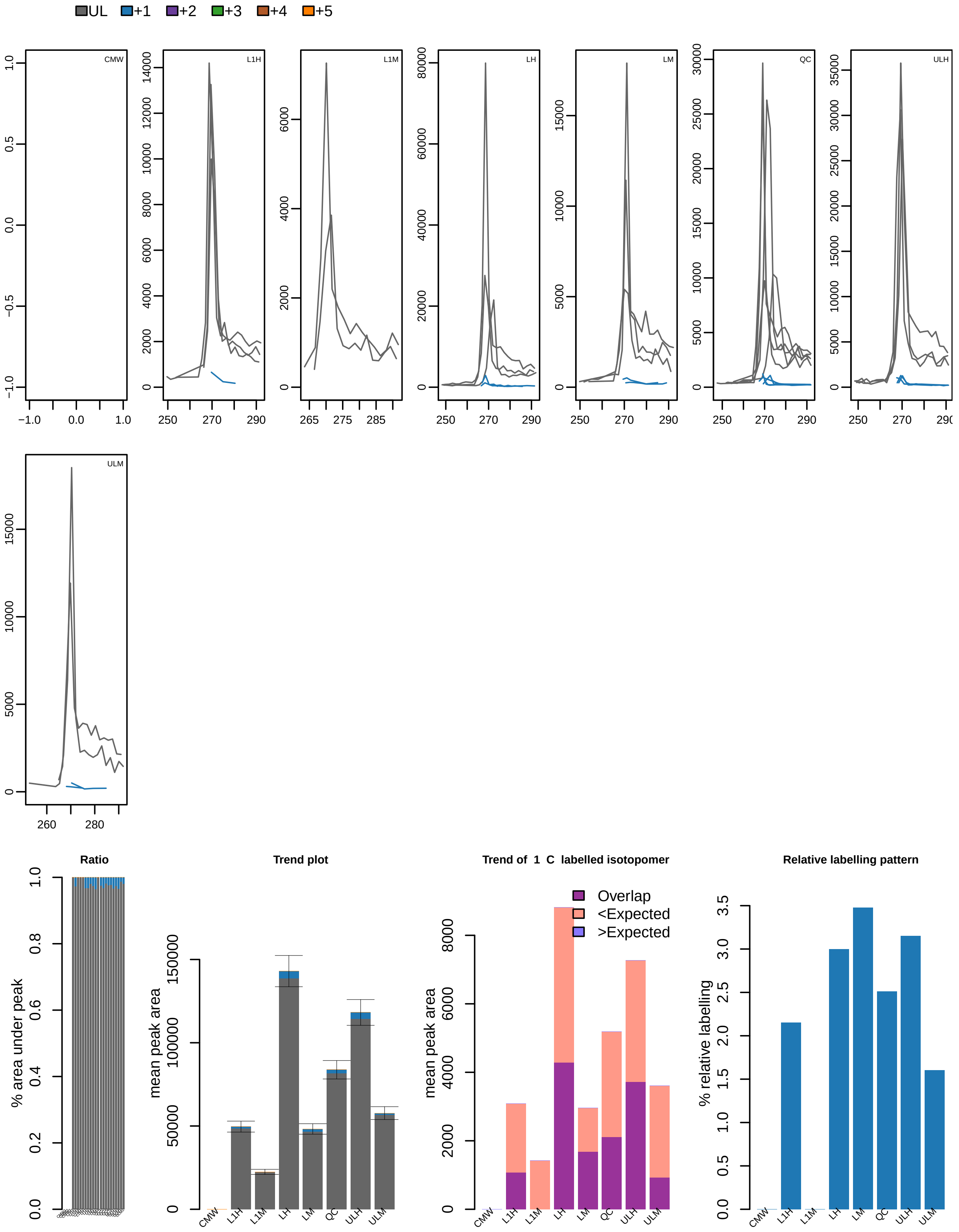

Phenylacetylglycine

Formula: C10H11NO3 Mass: 193.074 Std.RT: 270.2710851 Ion: NEC

G1

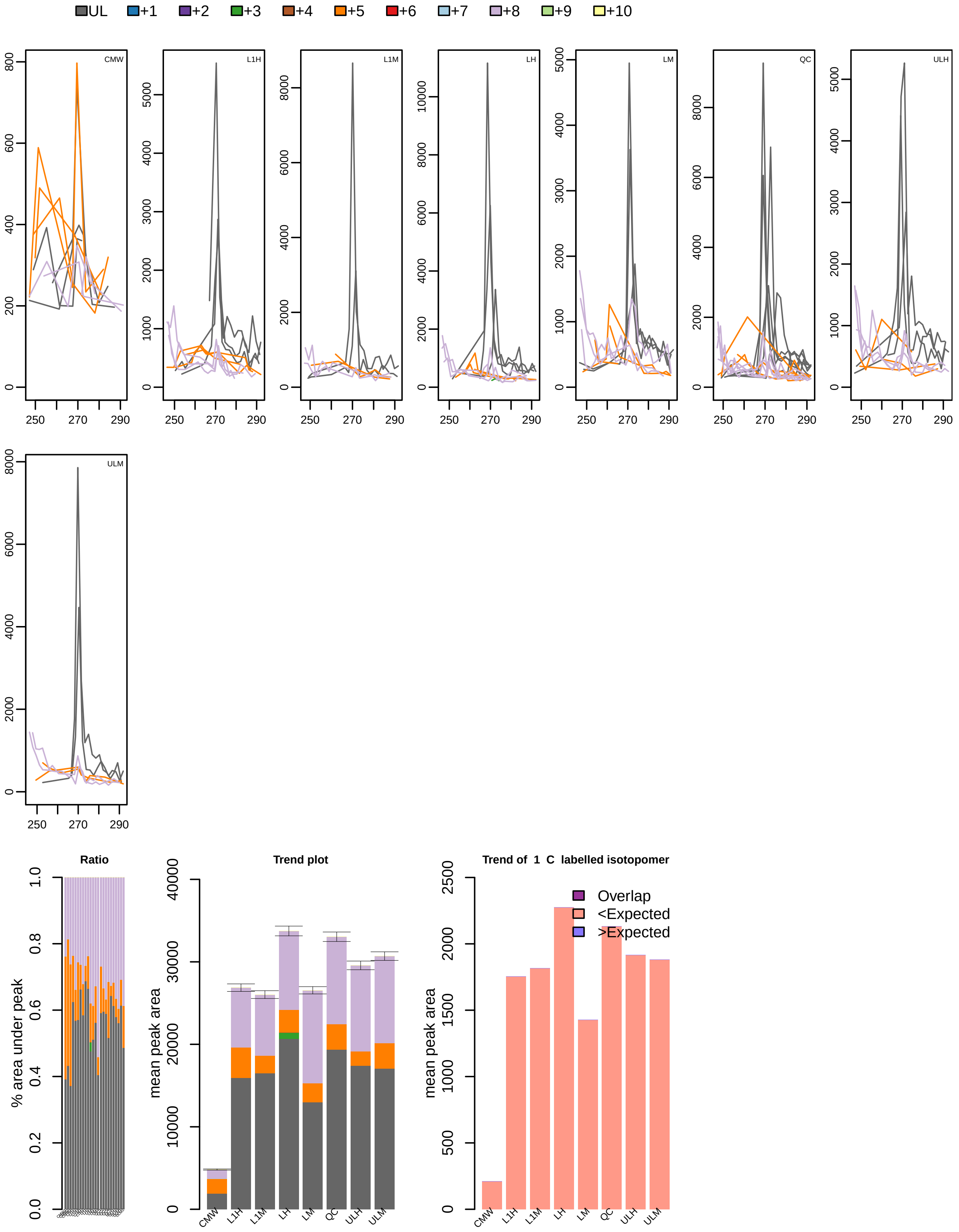

2−Hydroxy−2,4−pentadienoate

Formula: C5H6O3 Mass: 114.032 Std.RT: 958.032504 Ion: NEG

G1

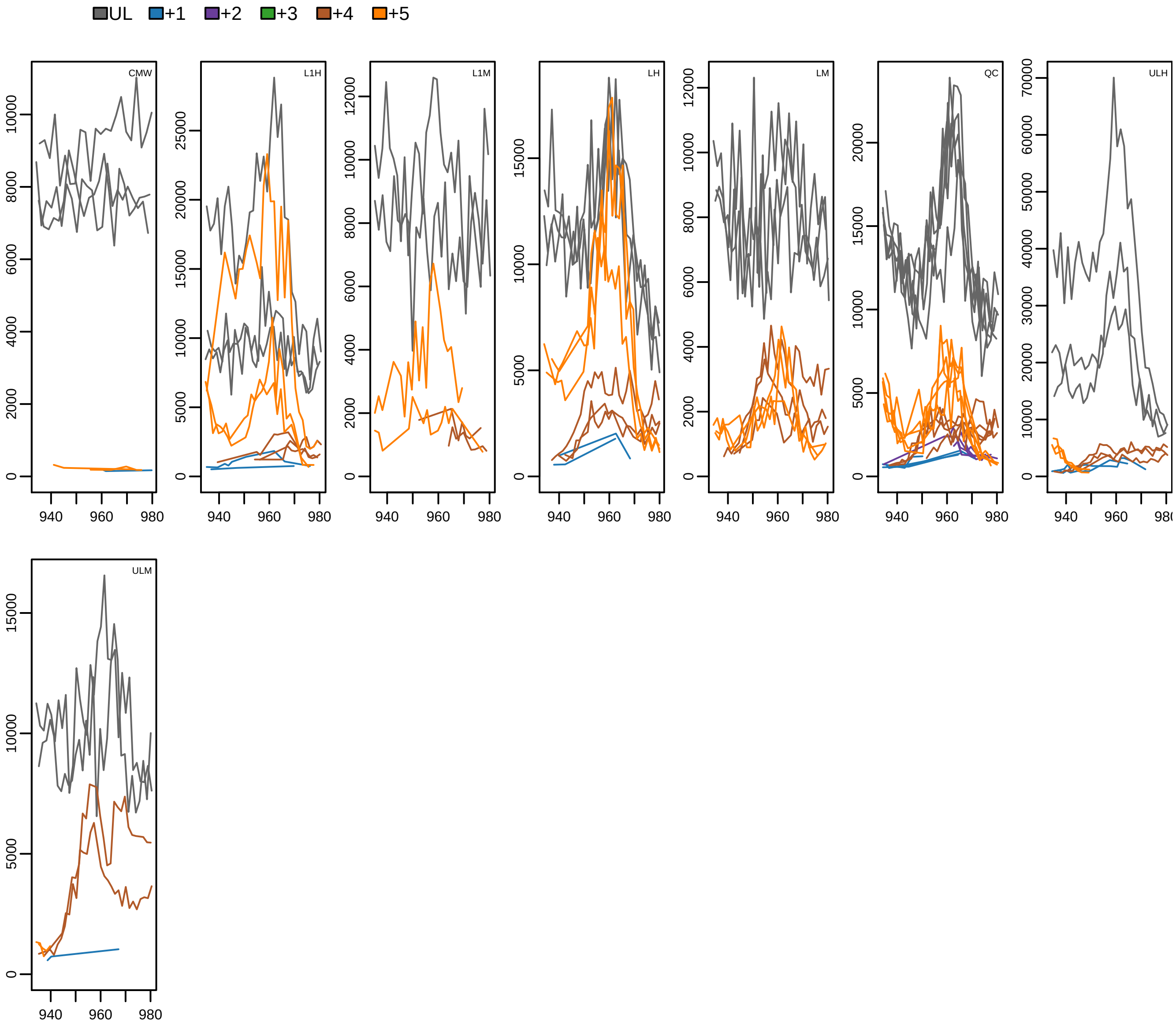

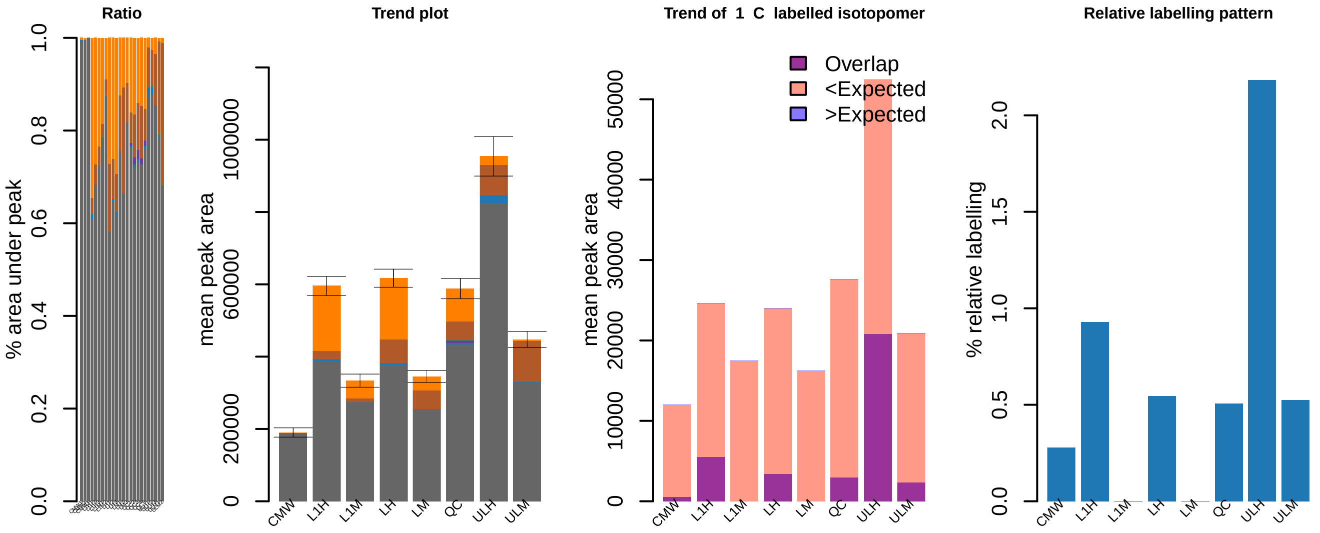

Phenylpyruvate

G1

Formula: C9H8O3 Mass: 164.047 Std.RT: 257.93895912 Ion: NEG

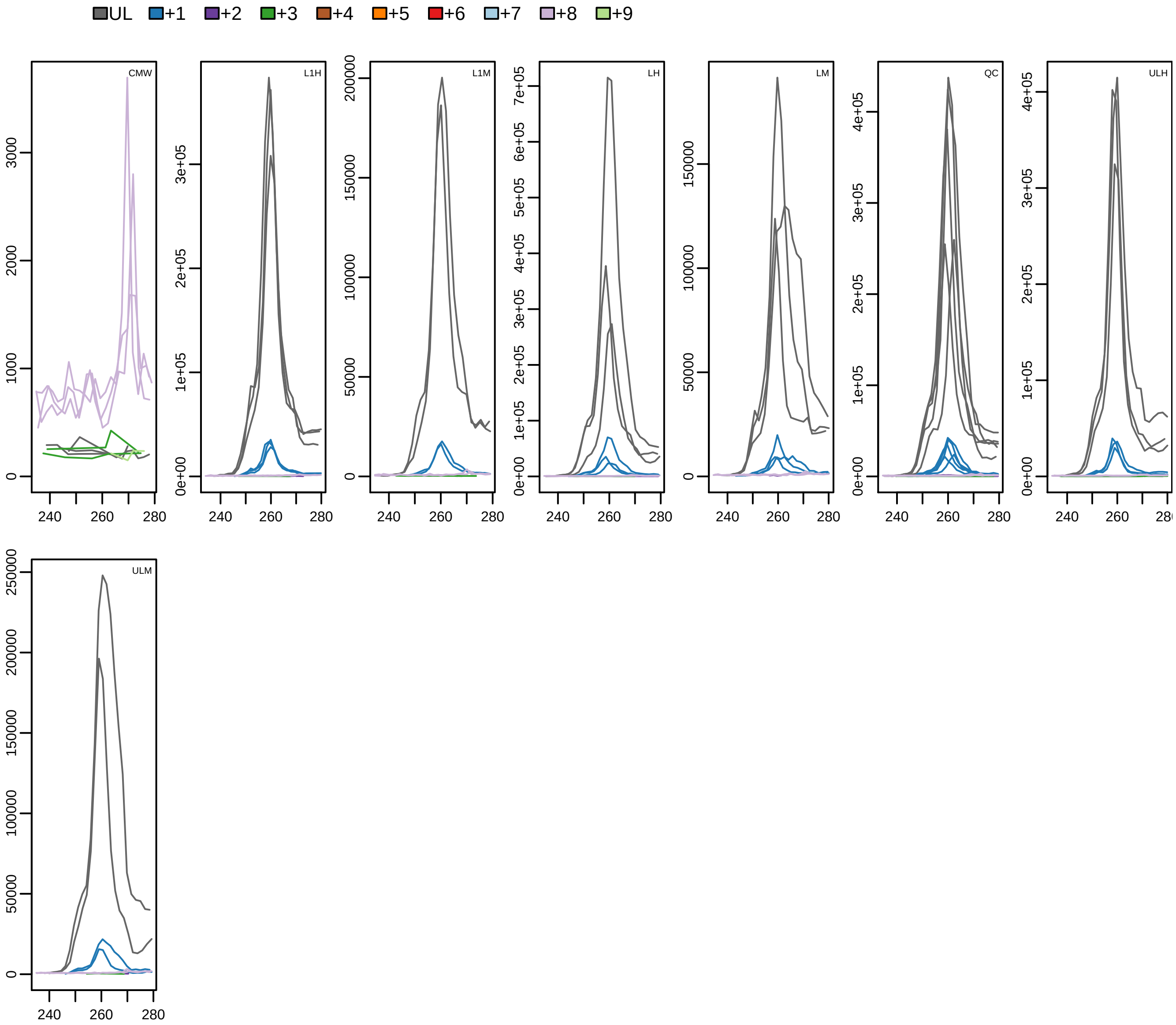

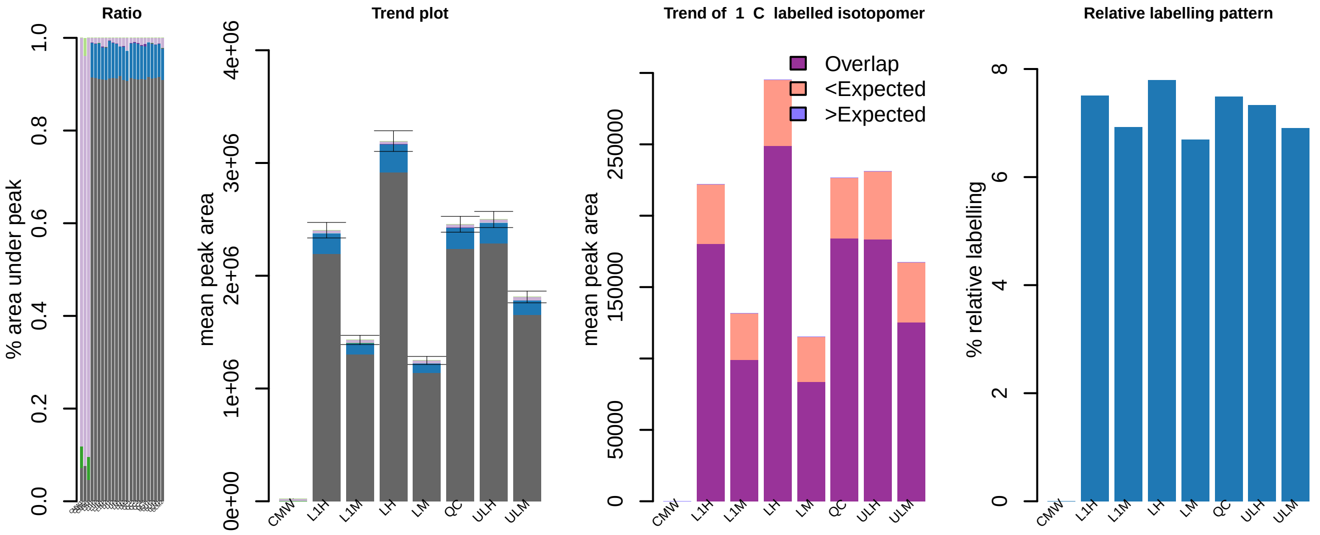

Phenylpyruvate

Formula: C9H8O3 Mass: 164.047 Std.RT: 257.93895912 Ion: NEG

G2

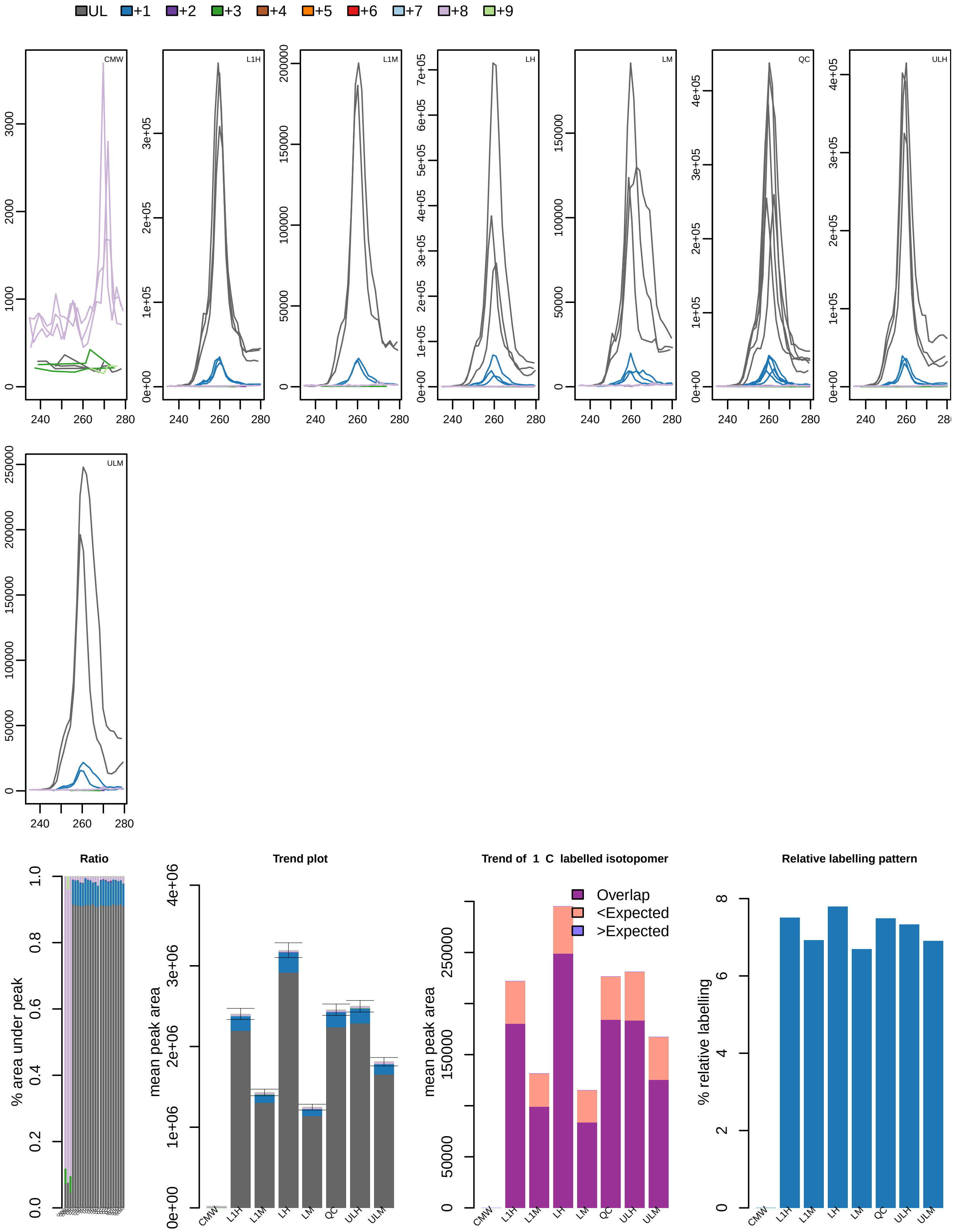

Quinate

Formula: $C_7H_{12}O_6$ Mass: 192.063 Std.RT: 943.0802484 Ion: NEG

G1

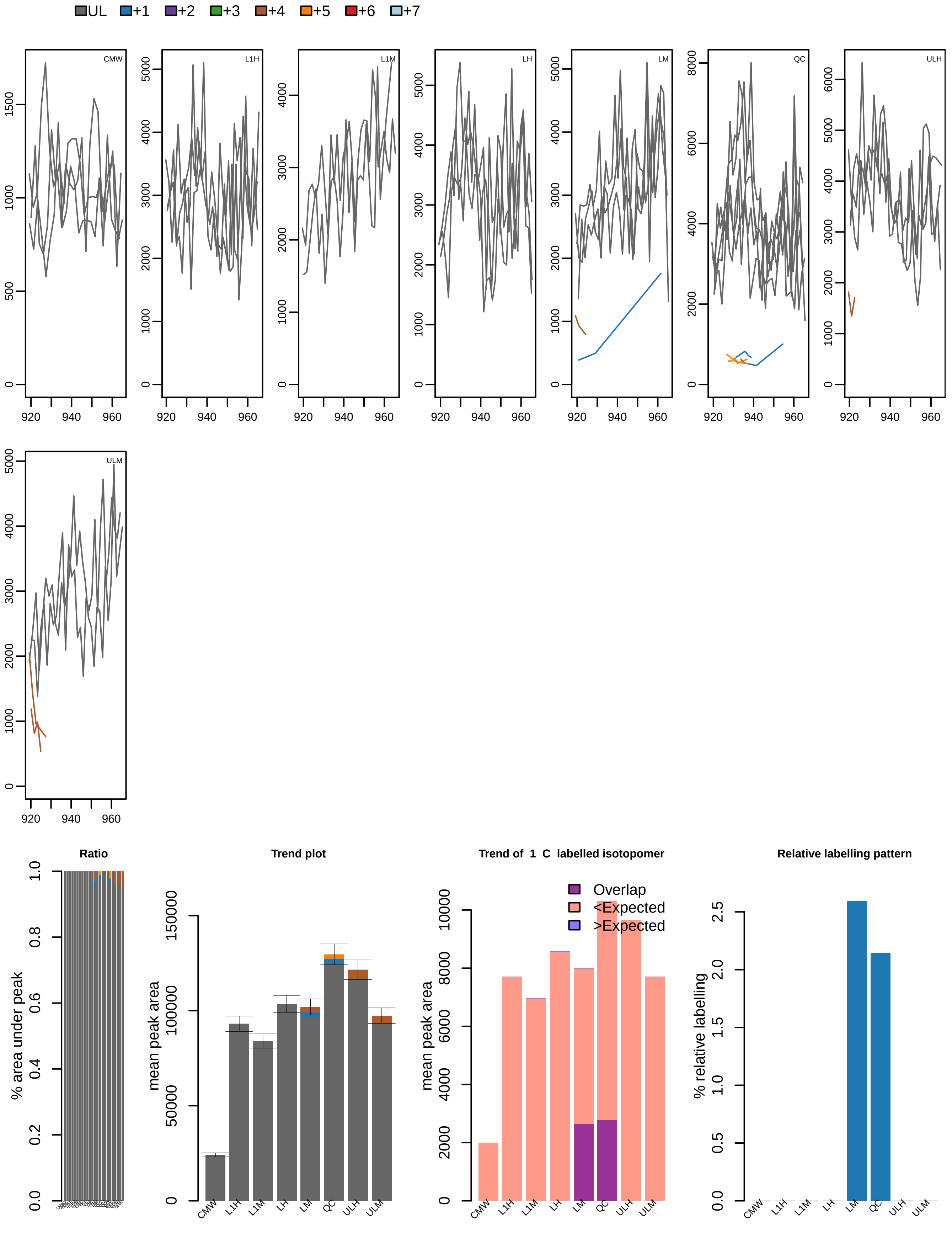

3−(4−Hydroxyphenyl)lactate

Formula: C9H10O4 Mass: 182.058 Std.RT: 502.37014038 Ion: NEG

G1

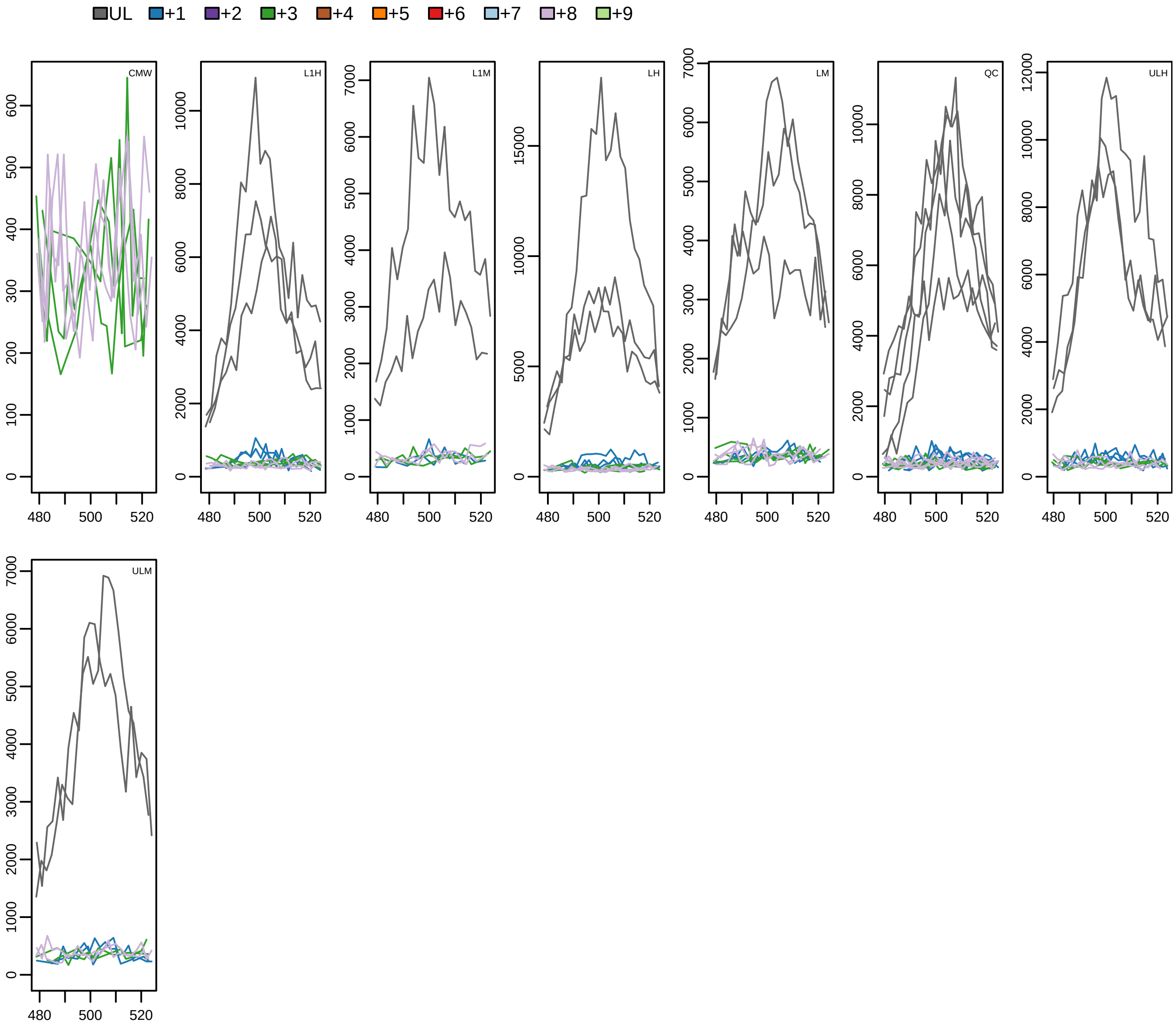

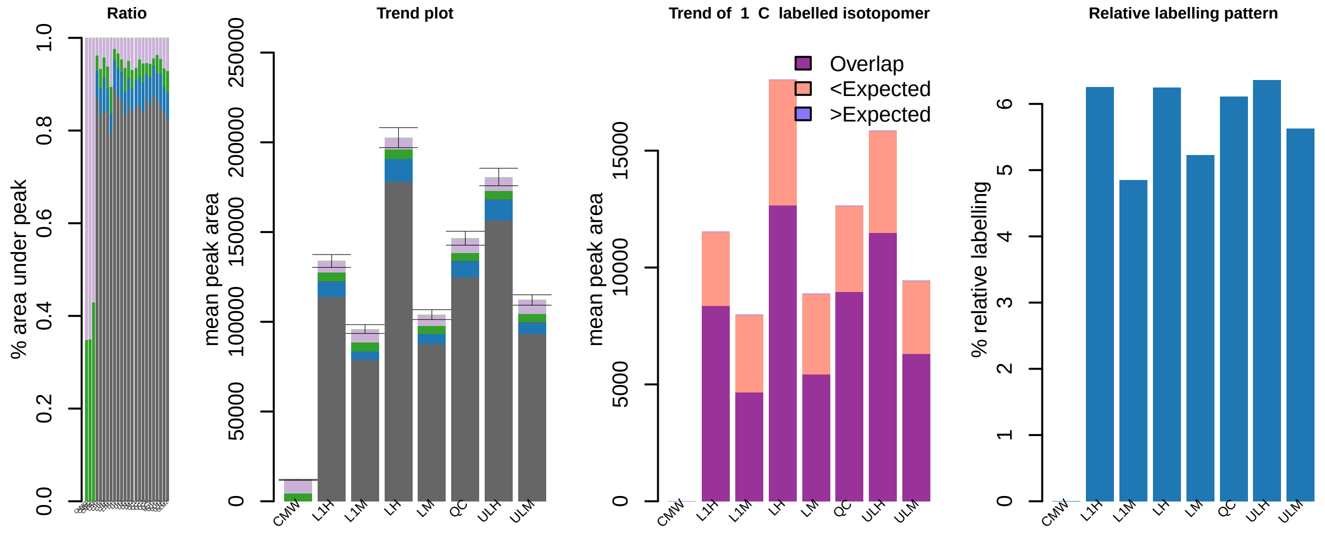

[FA hydroxy(7:1/2:0)] 2,4−dihydroxy−2−heptenedioc aci

Formula: C7H10O6 Mass: 190.048 Std.RT: 666.1242474 Ion: NEG

G1

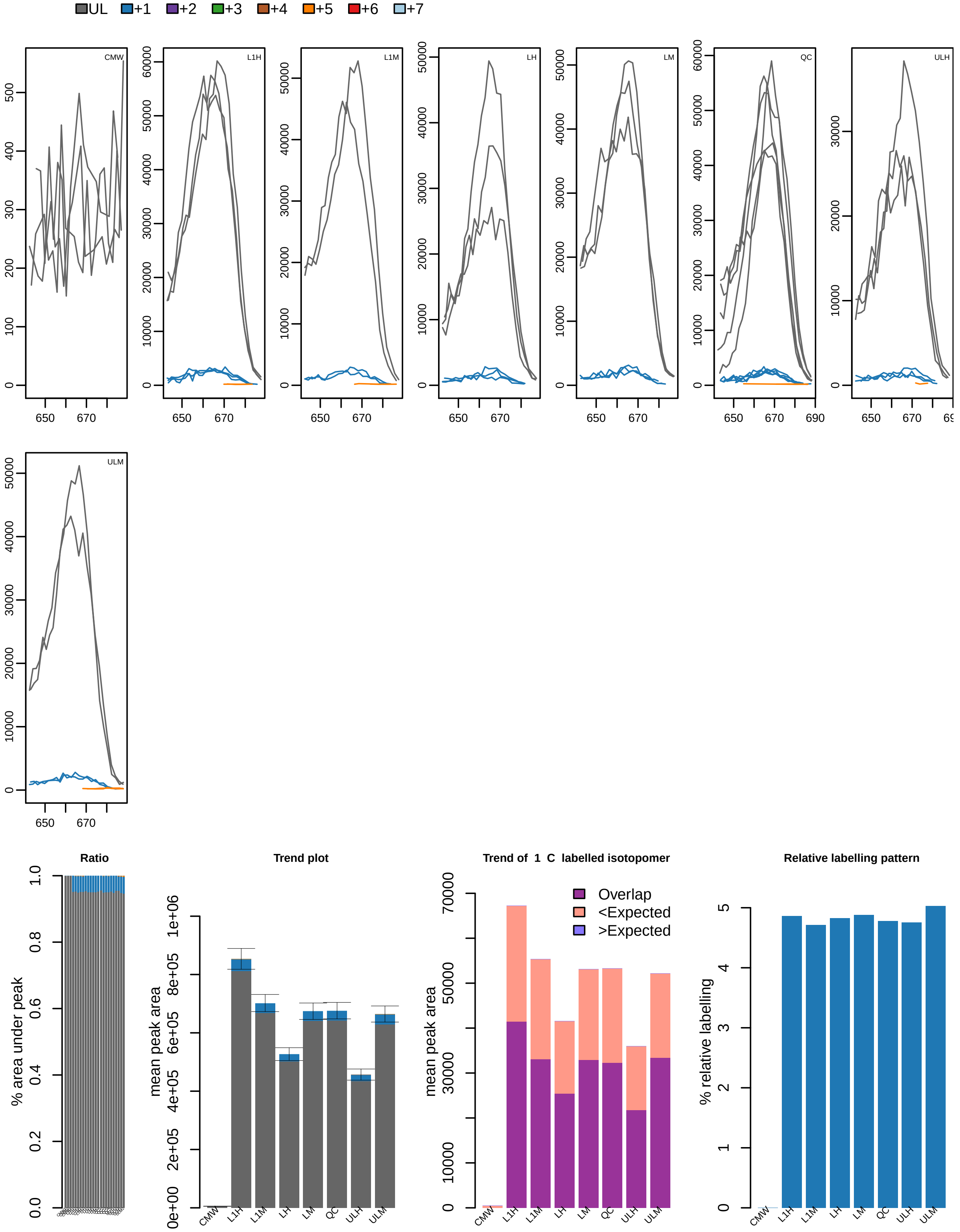

3−Methoxy−4−hydroxyphenylacetaldehyde

Formula: C9H10O3 Mass: 166.063 Std.RT: 268.59149928 Ion: NEG

G1

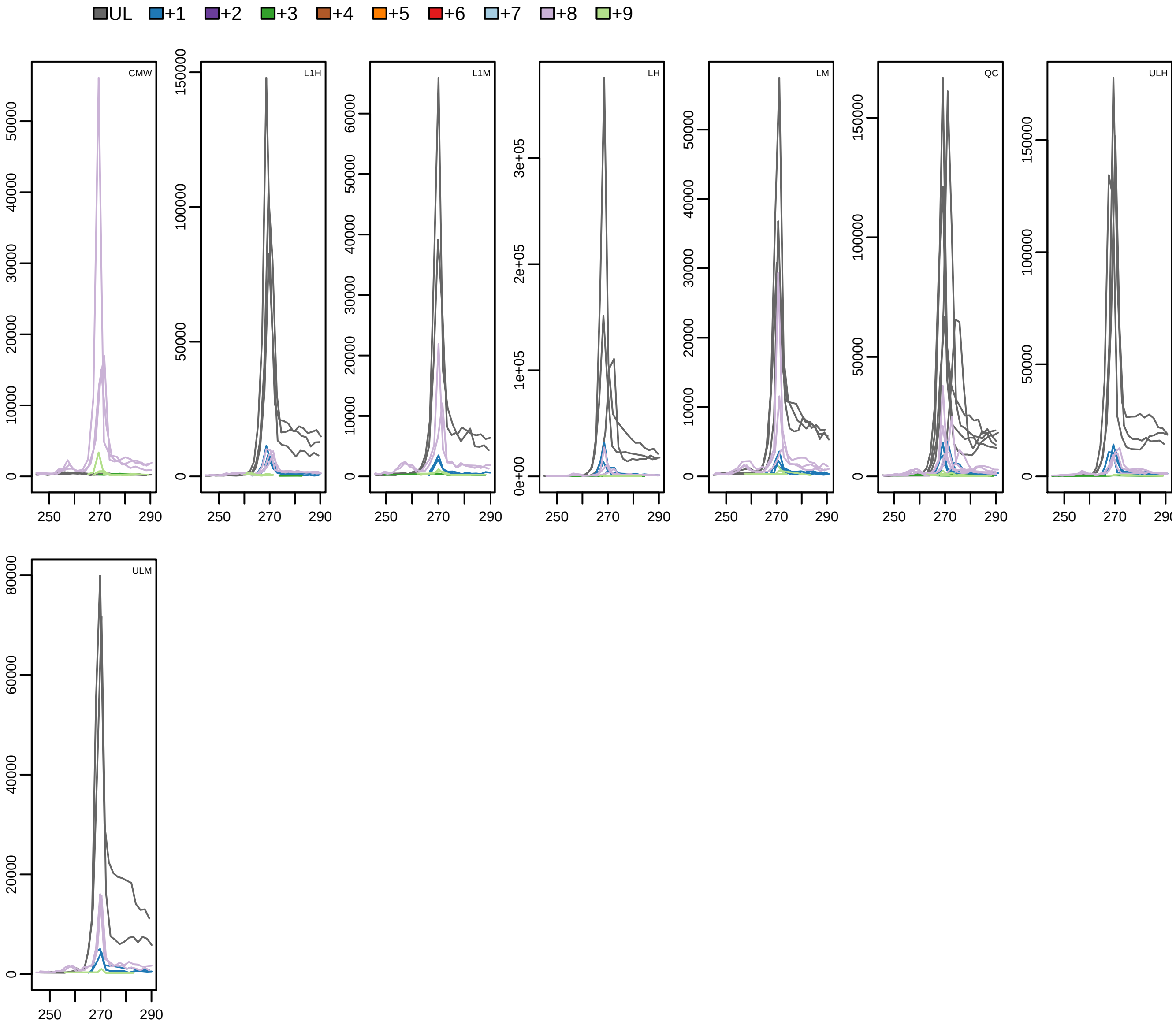

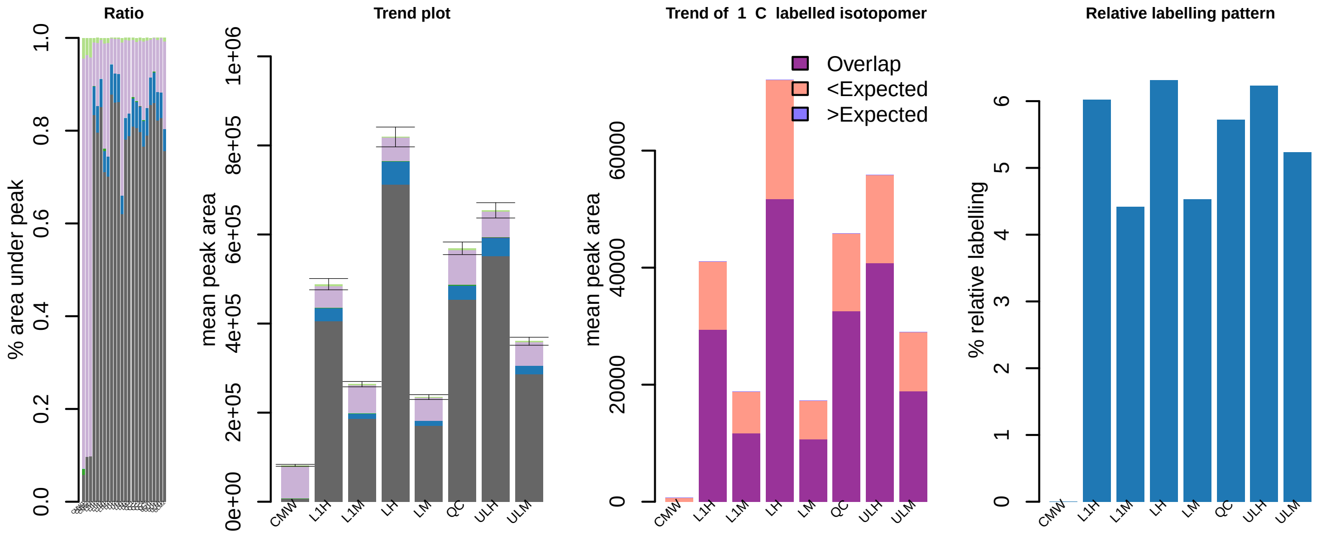

3−(4−Hydroxyphenyl)pyruvate

Formula: C9H8O4 Mass: 180.042 Std.RT: 433.33956762 Ion: NEG

G1

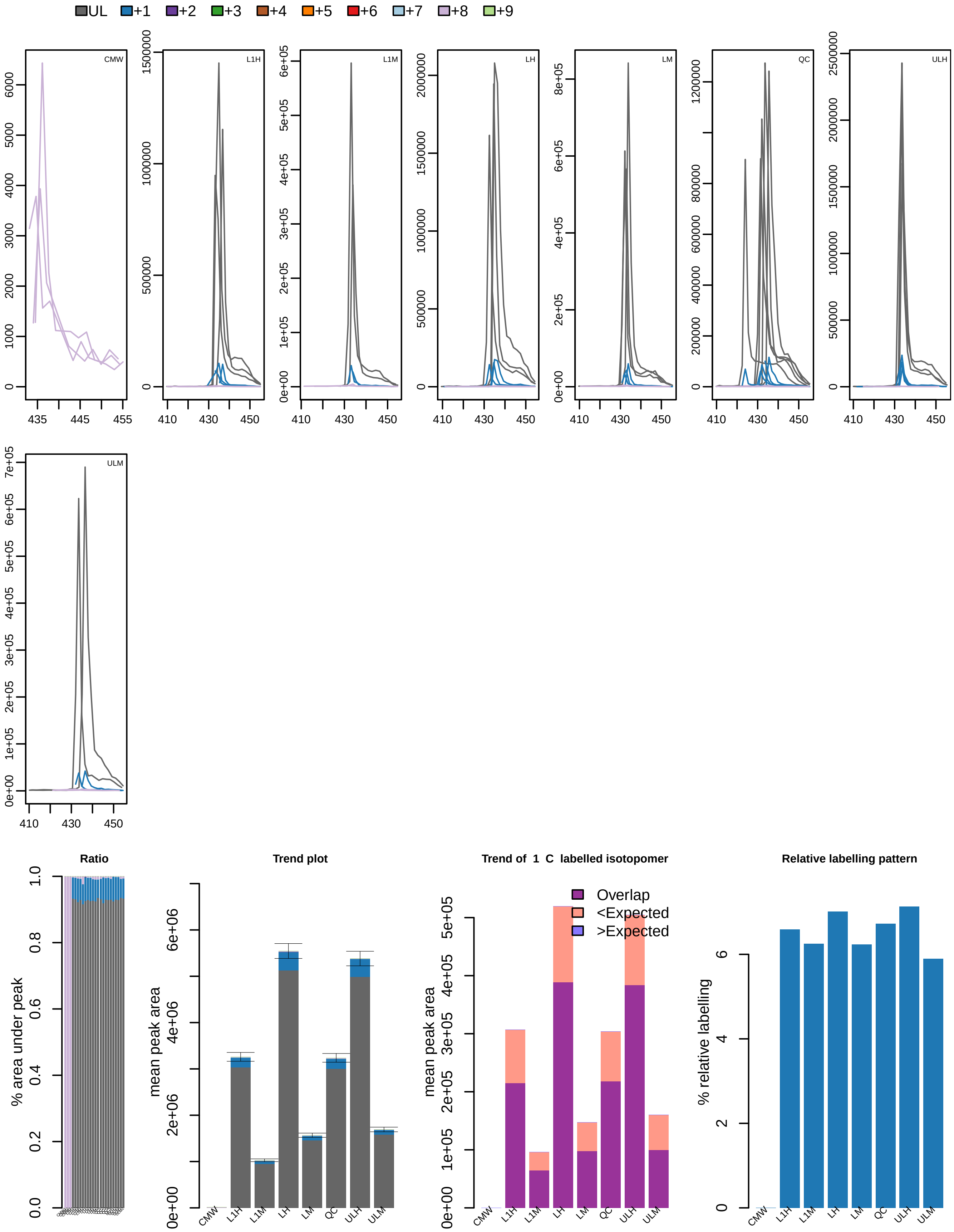

(S)−3−Methyl−2−oxopentanoic acid

Formula: C6H10O3 Mass: 130.063 Std.RT: 260.7654165 Ion: NEG

G1

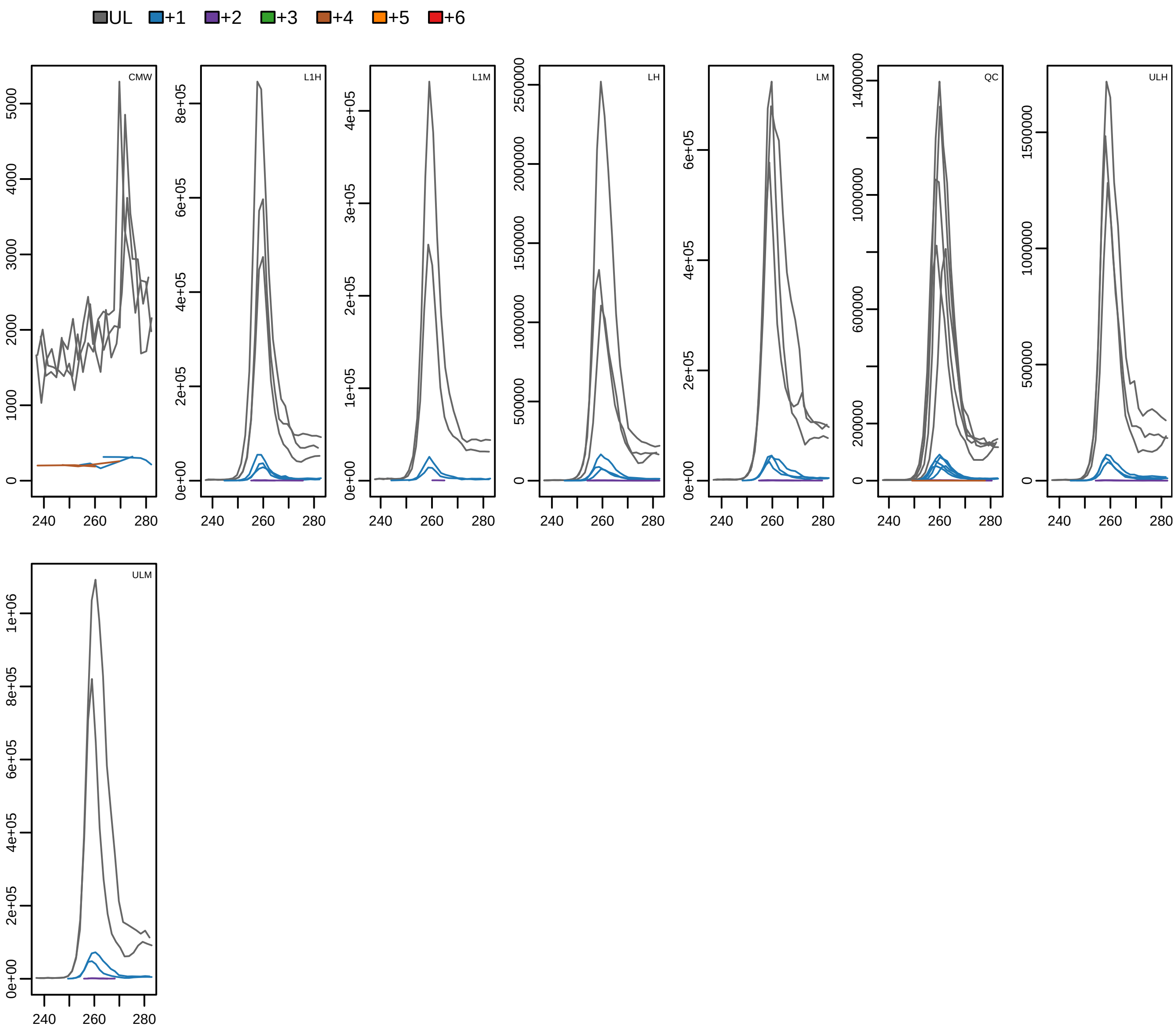

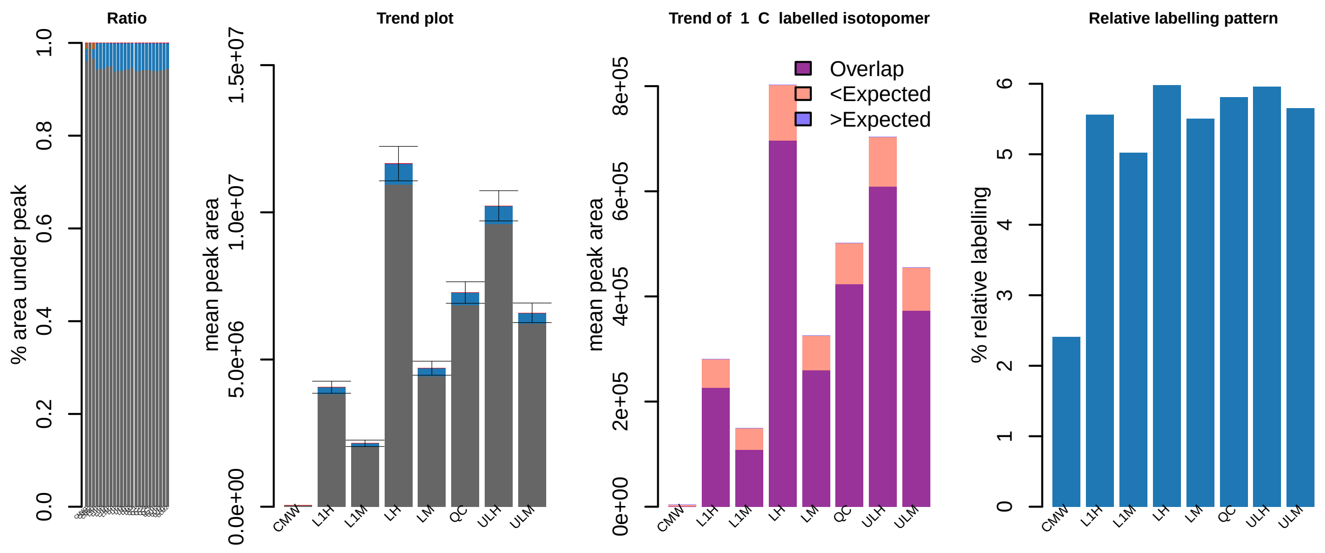

3−Methyl−2−oxobutanoic acid

Formula: $C_5H_8O_3$ Mass: 116.047 Std.RT: 275.44858548 Ion: NEG

G1

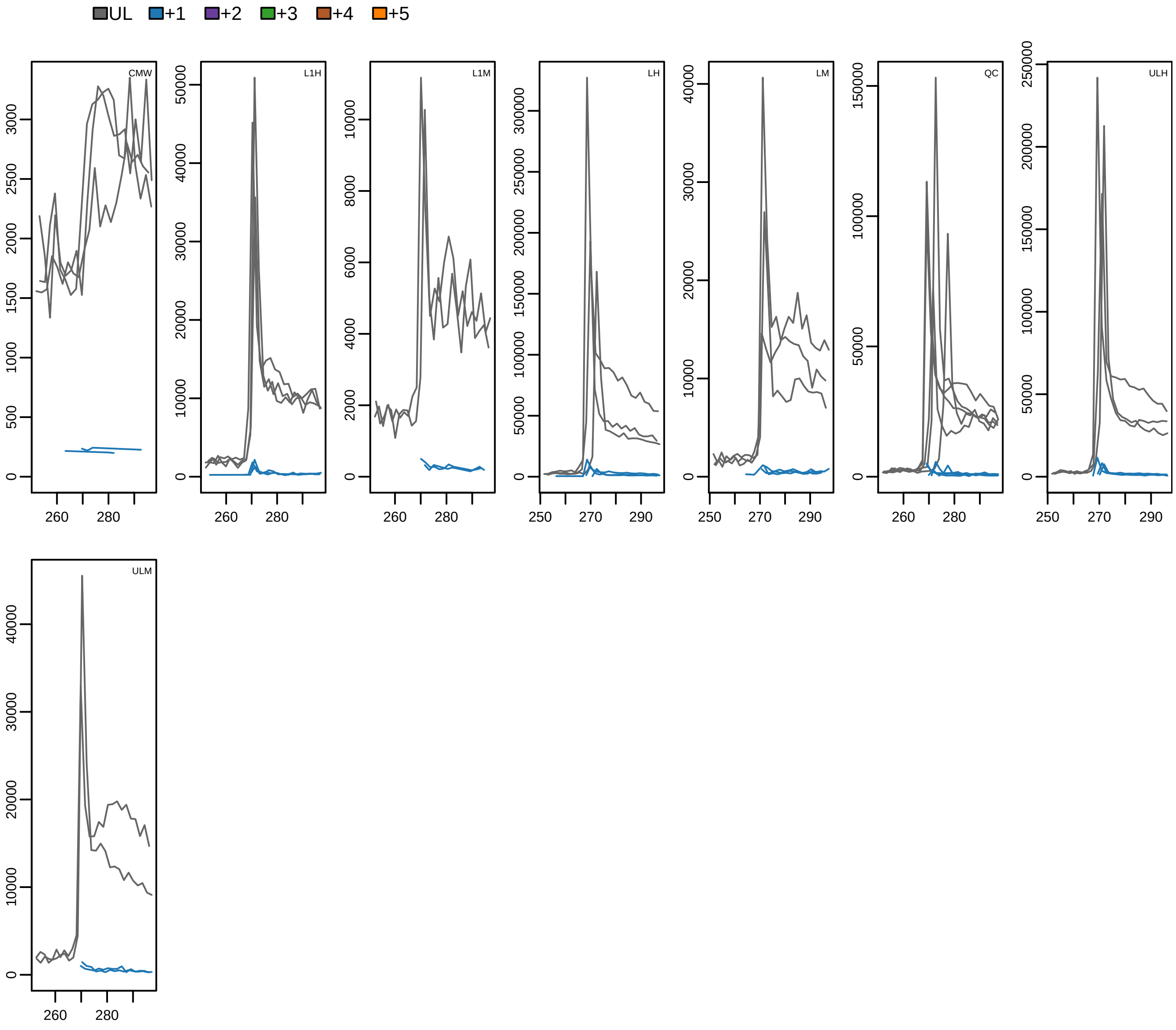

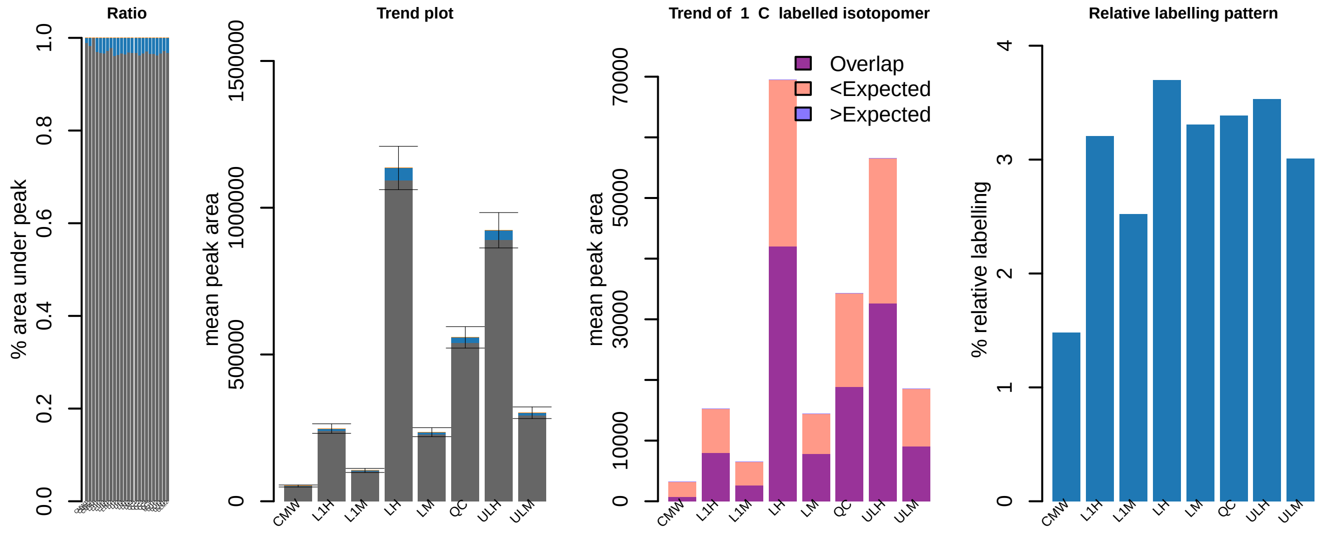

L−Valine

Formula: C5H11NO2 Mass: 117.079 Std.RT: 804.6172458 Ion: NEG

G1

UL +1 +2 +3 +4 +5

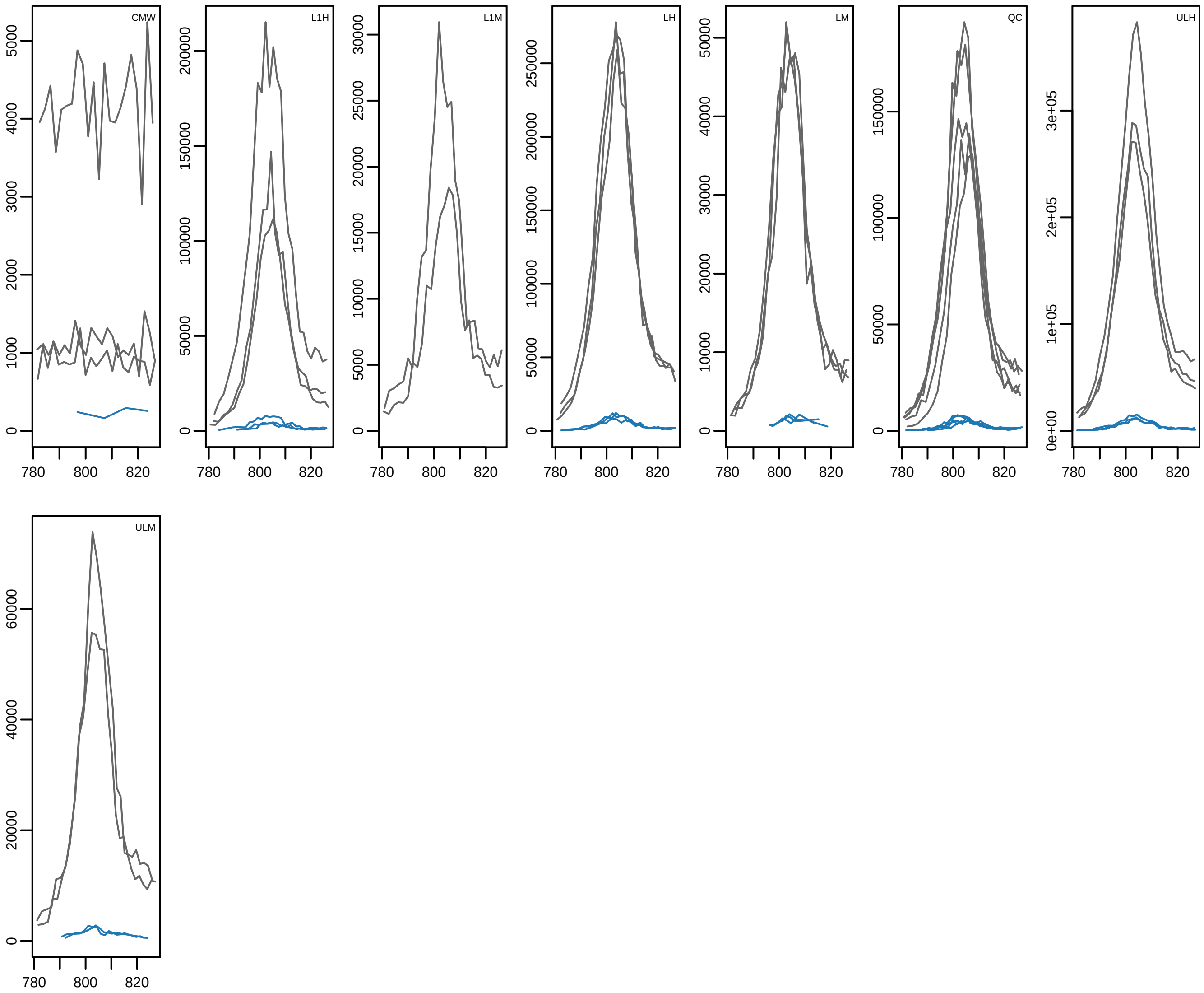

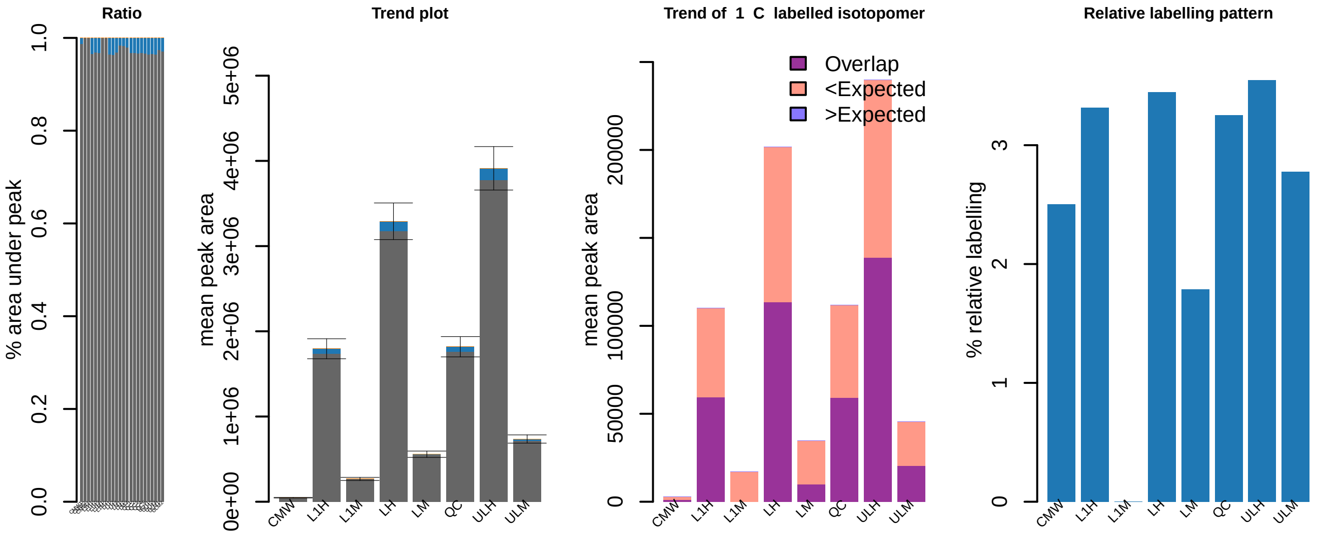

Erythrulose 1−phosphate

Formula: C4H9O7P Mass: 200.009 Std.RT: 798.507429 Ion: NEG

G1

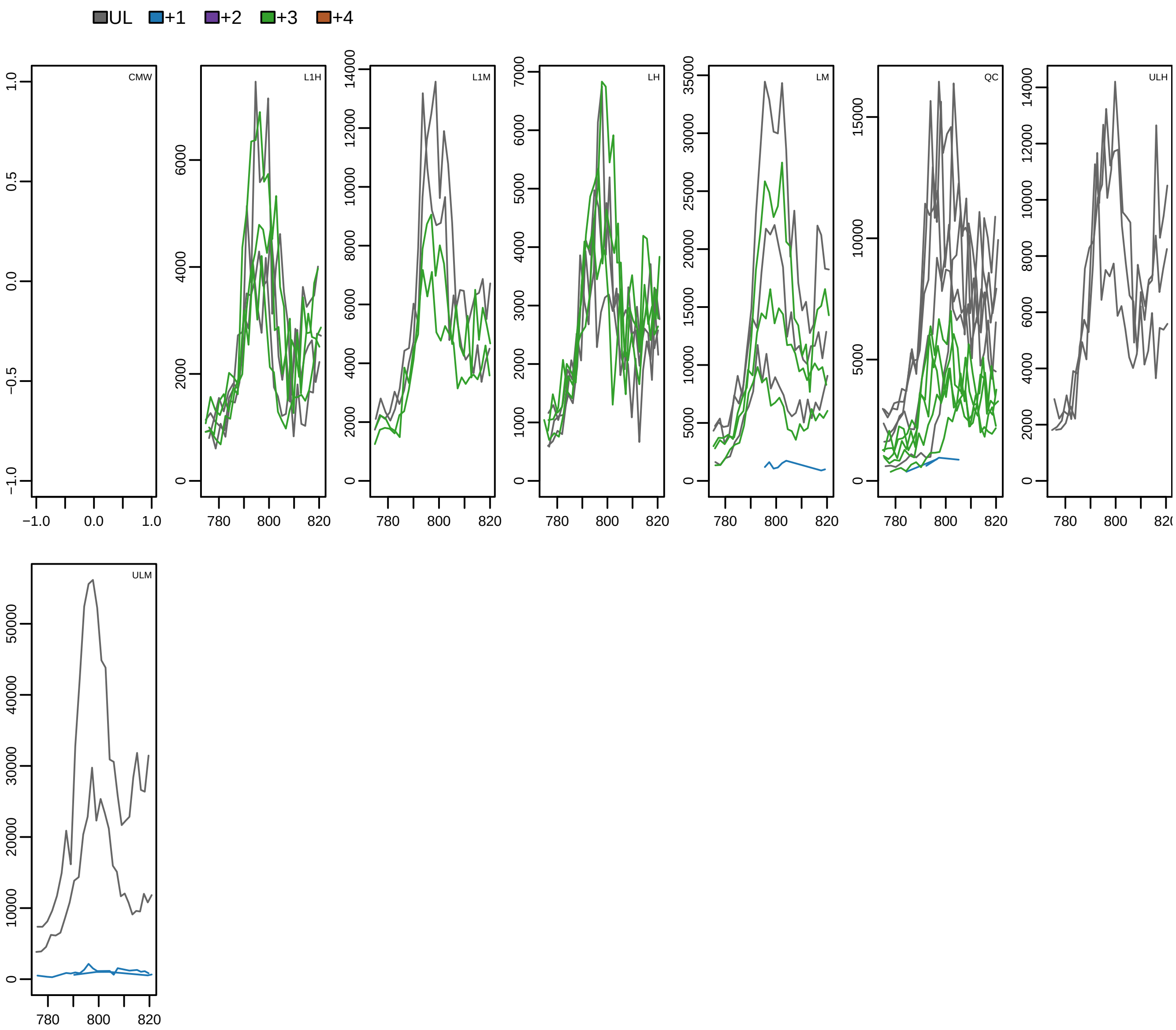

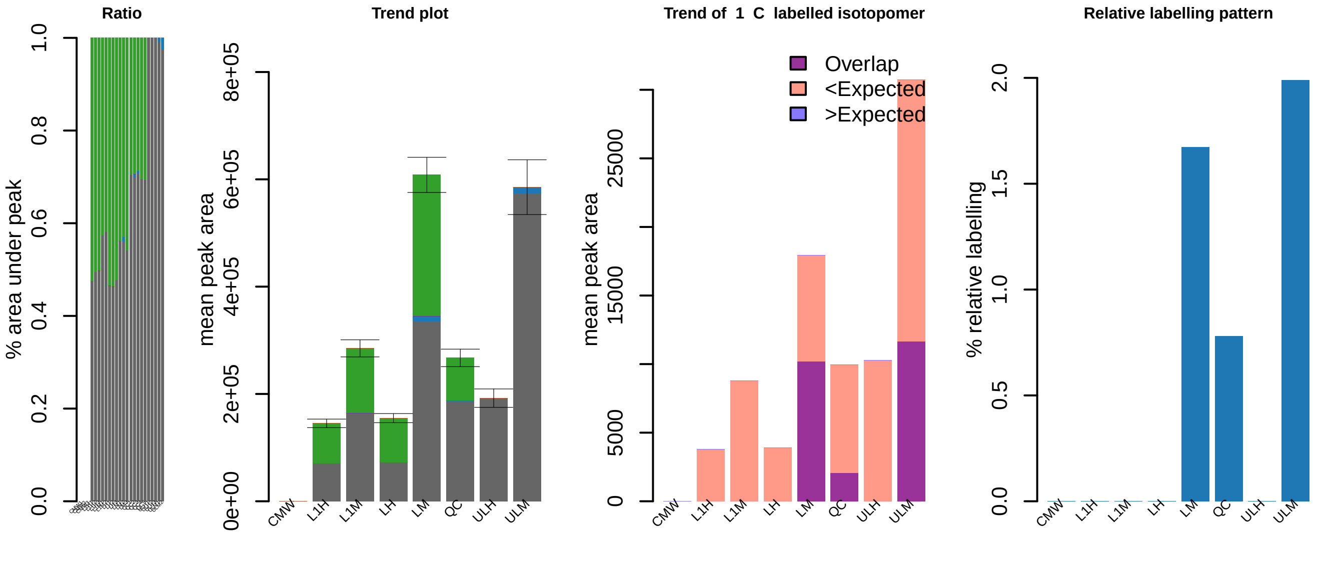

Erythrulose 1−phosphate

G2

Formula: C4H9O7P Mass: 200.009 Std.RT: 798.507429 Ion: NEG

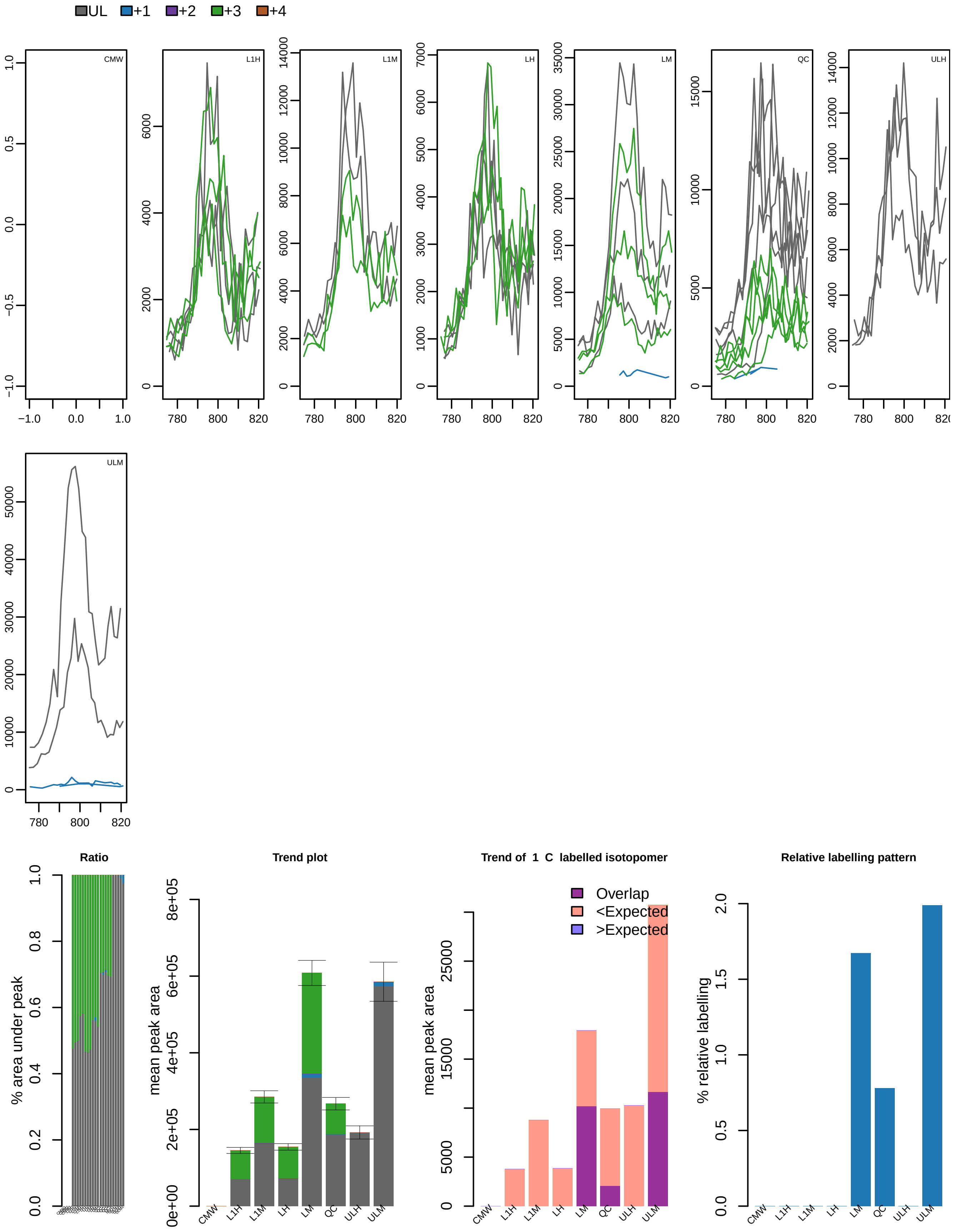

Chitobiose

G1

Formula: C16H28N2O11 Mass: 424.169 Std.RT: 1033.9099992 Ion:

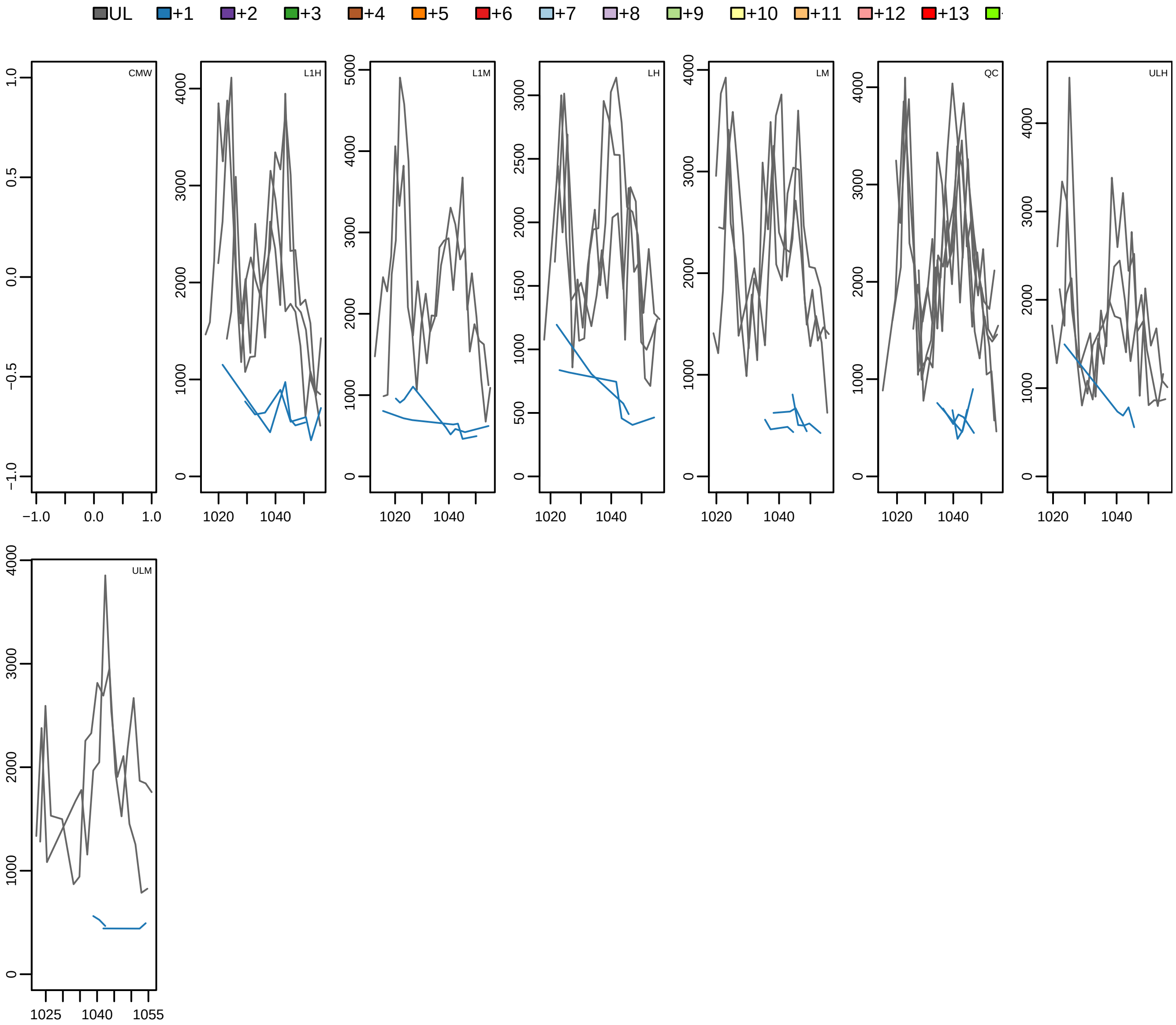

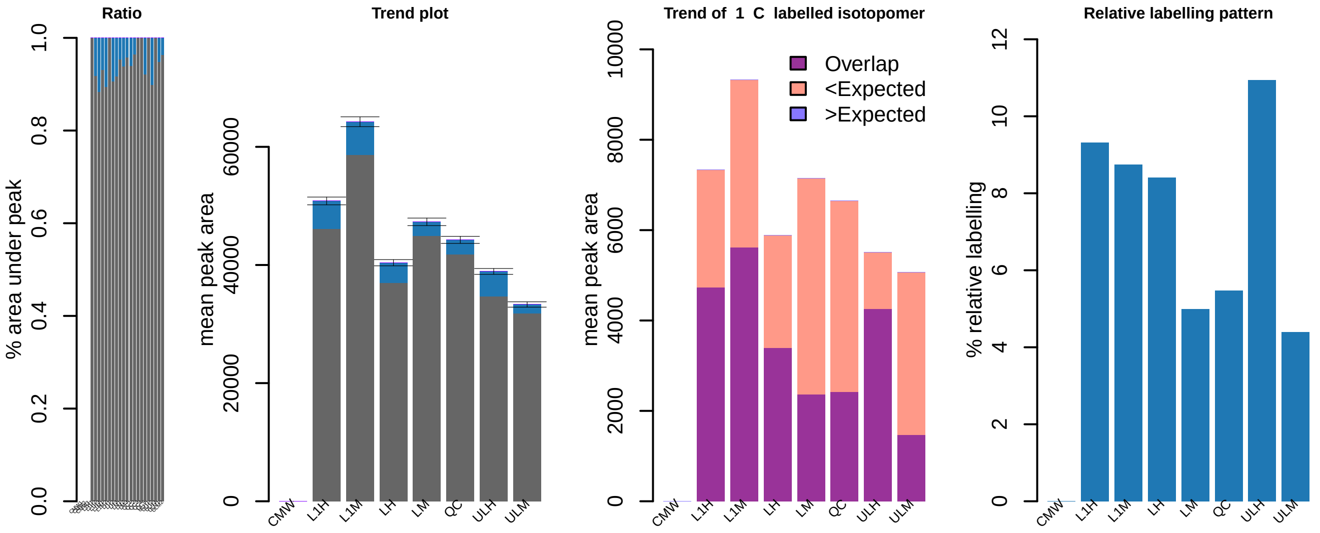

UDP−N−acetyl−D−glucosamine

Formula: C17H27N3O17P2 Mass: 607.082 Std.RT: 980.7077112 Ion:

G1

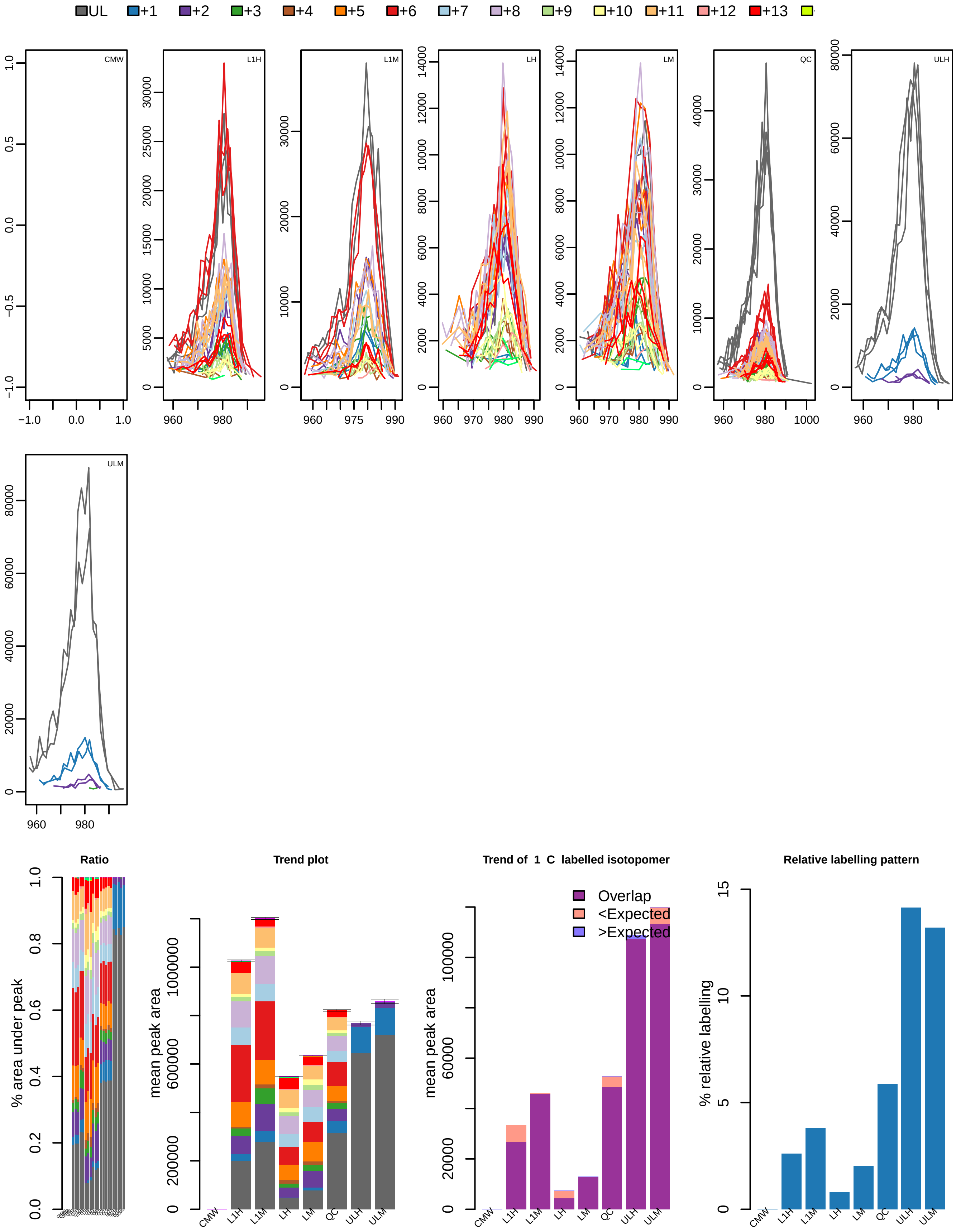

L−Arabinonate

Formula: C5H10O6 Mass: 166.048 Std.RT: 818.359749 Ion: NEG

G1

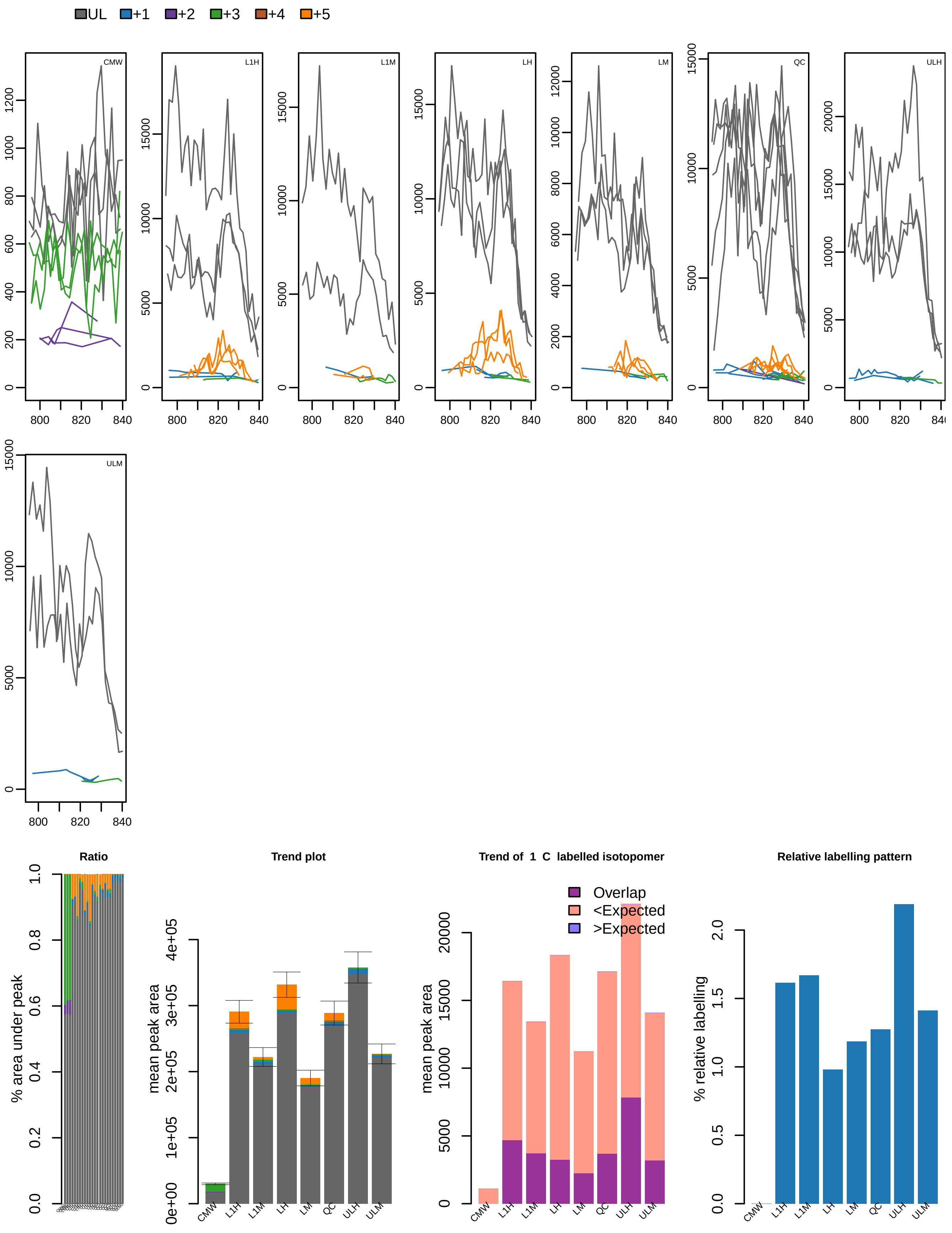

L−Arabinonate

Formula: C5H10O6 Mass: 166.048 Std.RT: 818.359749 Ion: NEG

G2

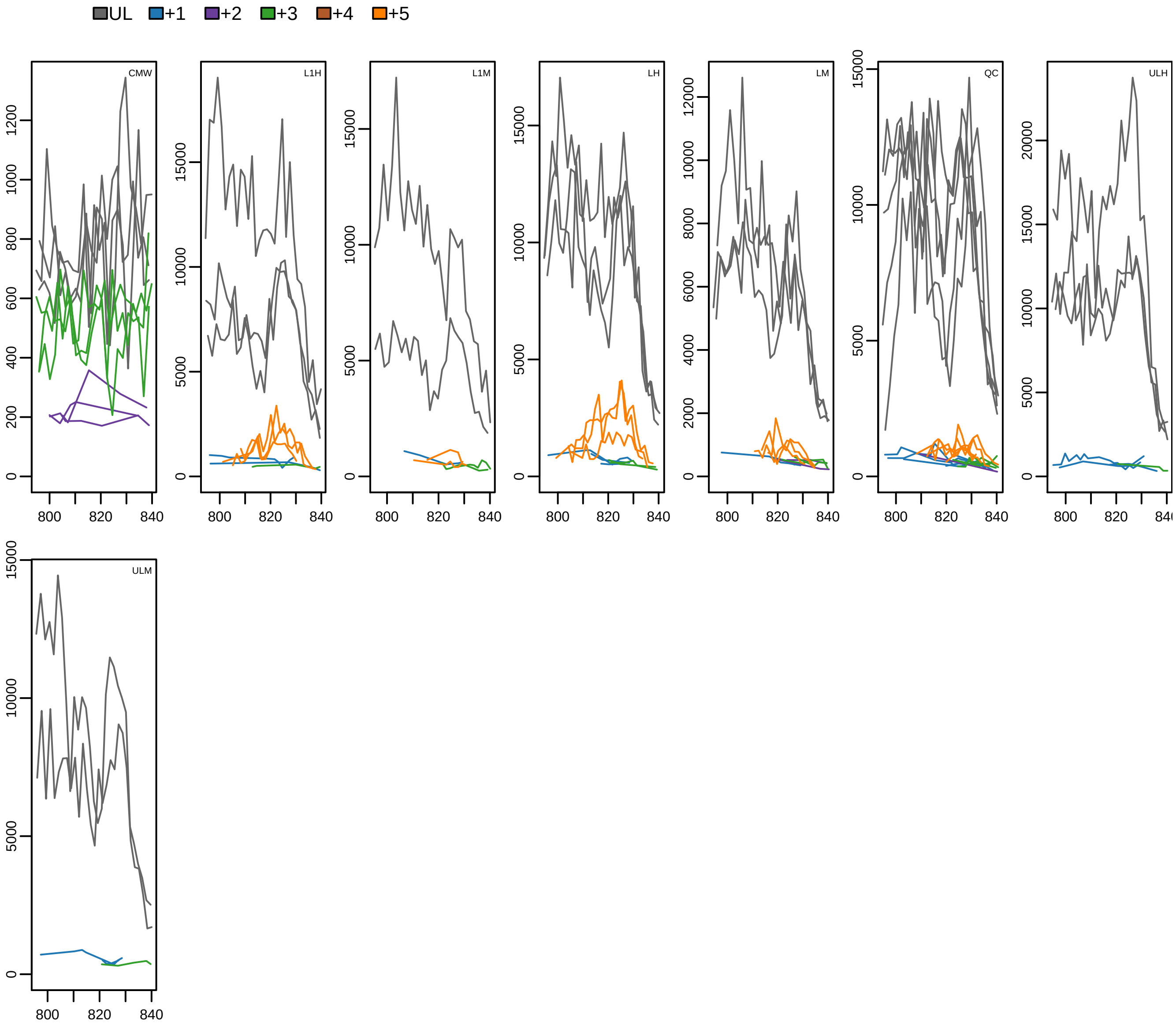

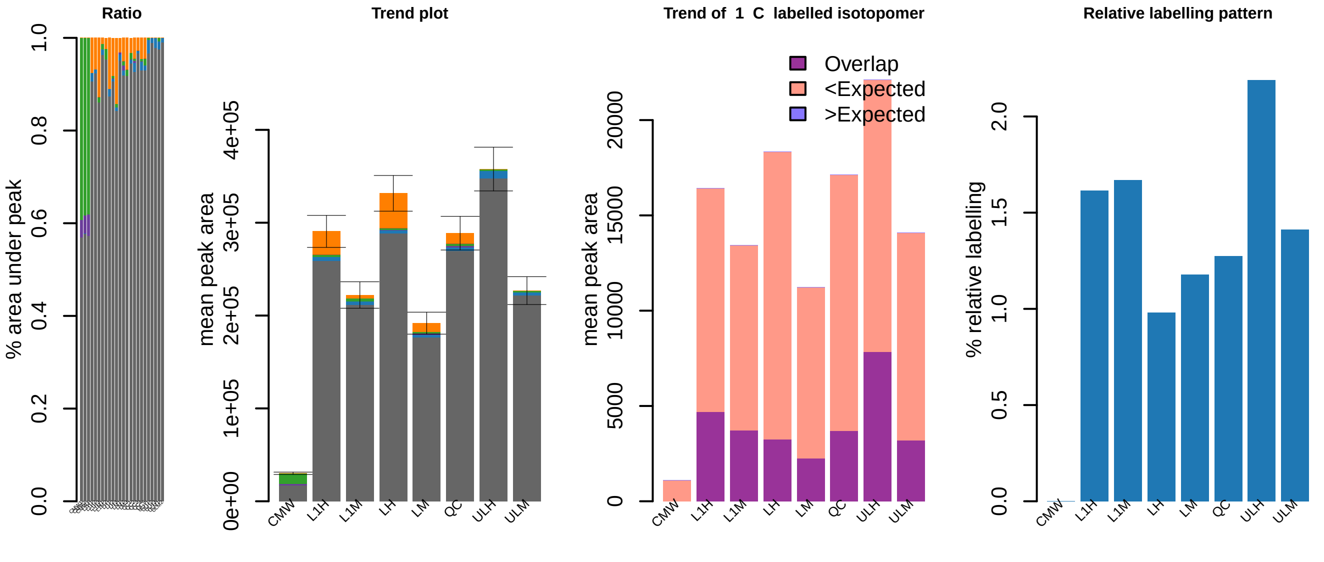

D−Glucarate

Formula: C6H10O8 Mass: 210.038 Std.RT: 1088.947149 Ion: NEG

G1

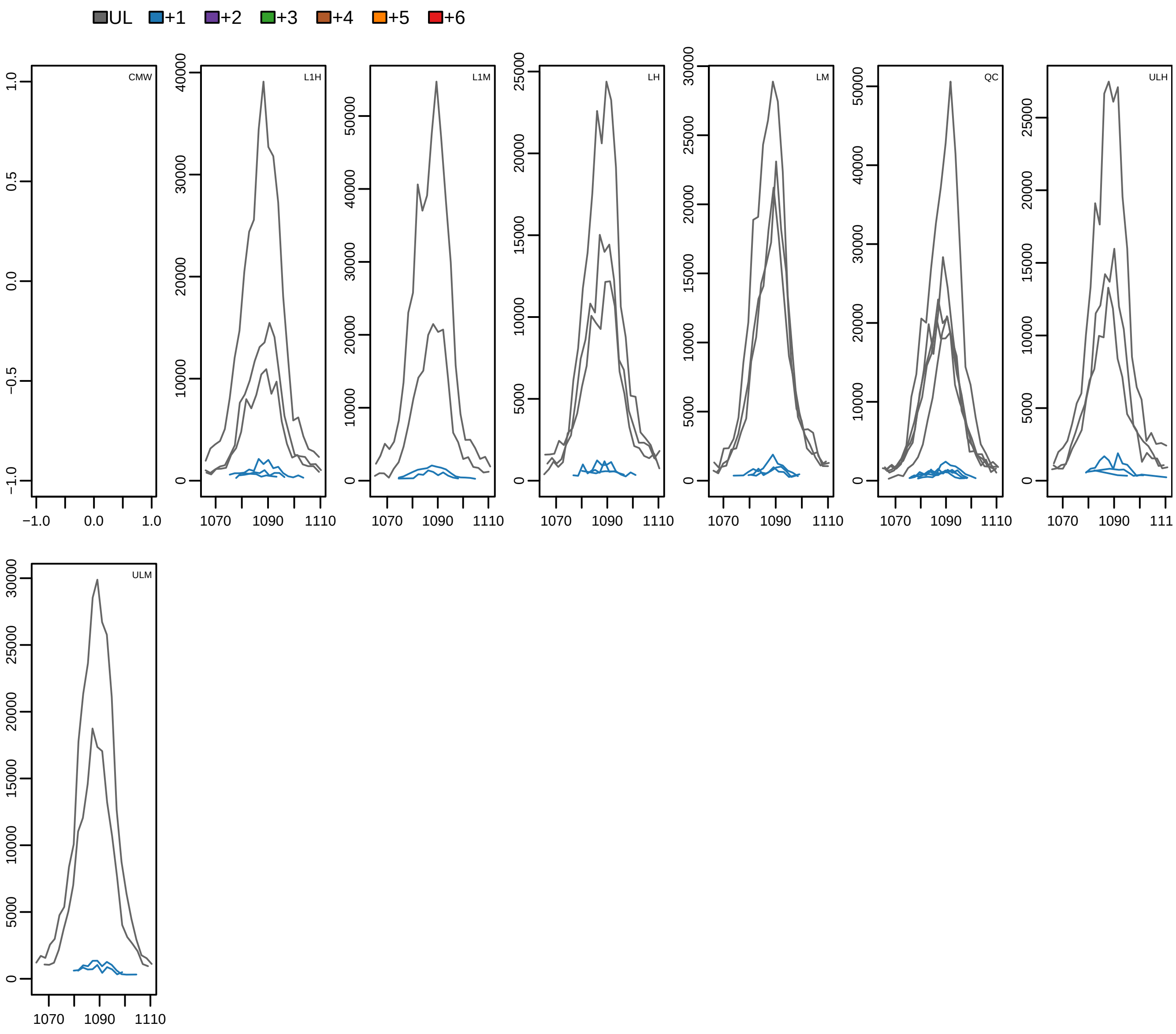

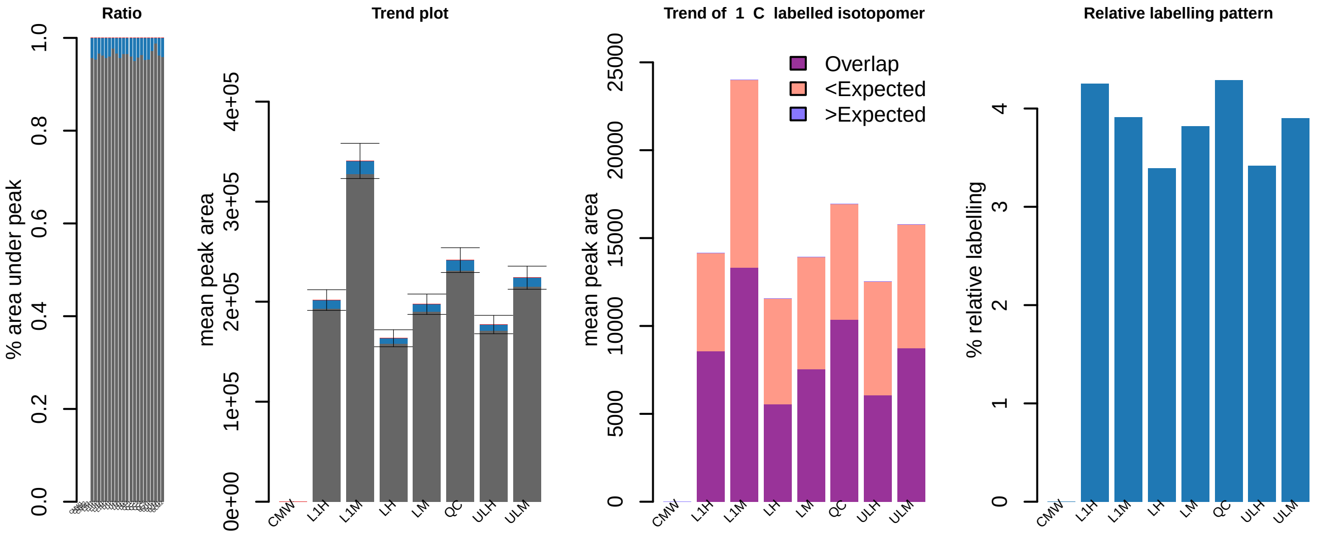

[FA trihydroxy(4:0)] 2,3,4−trihydroxy−butanoic acid

Formula: C4H8O5 Mass: 136.037 Std.RT: 786.4229964 Ion: NEG

G1

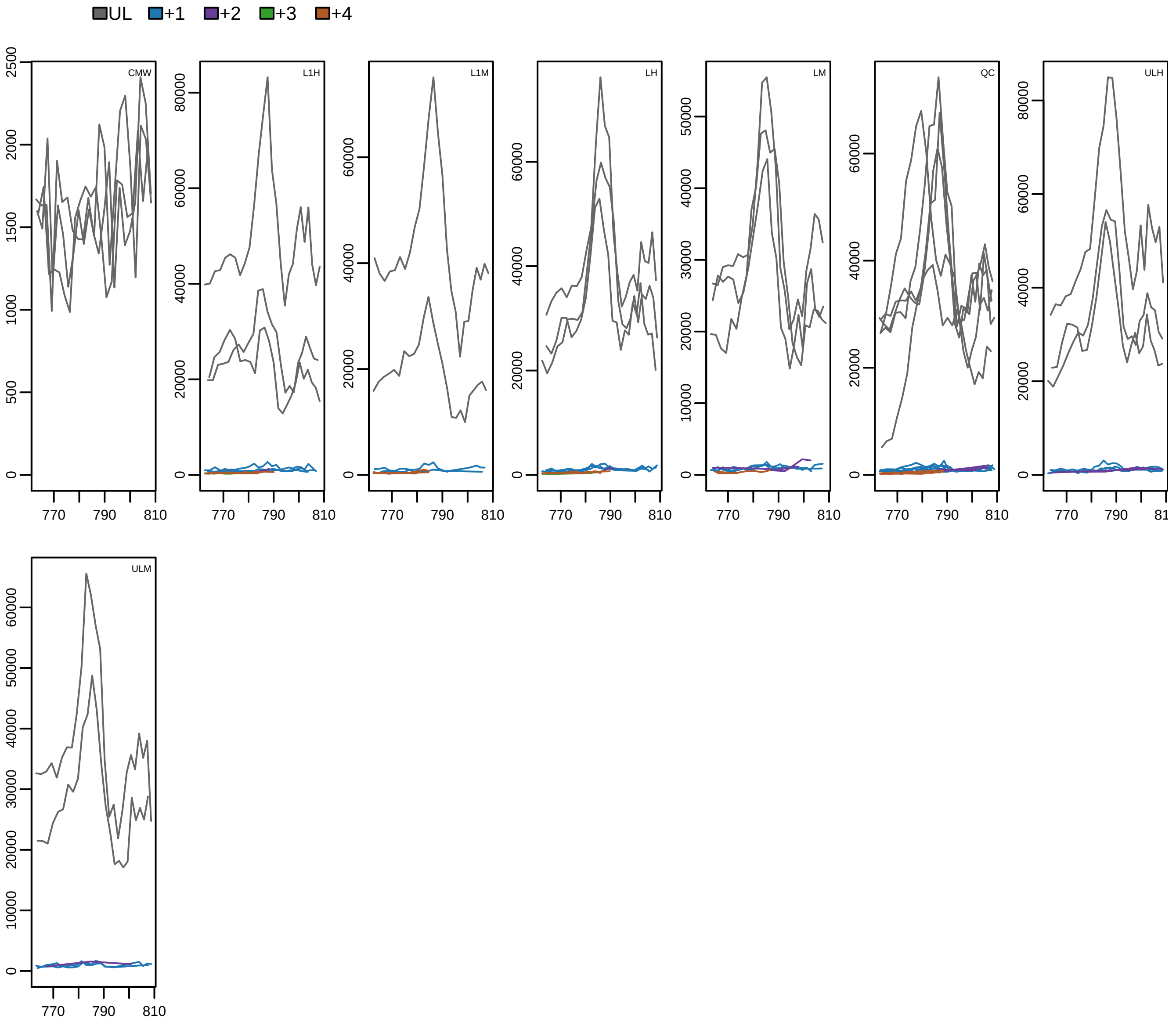

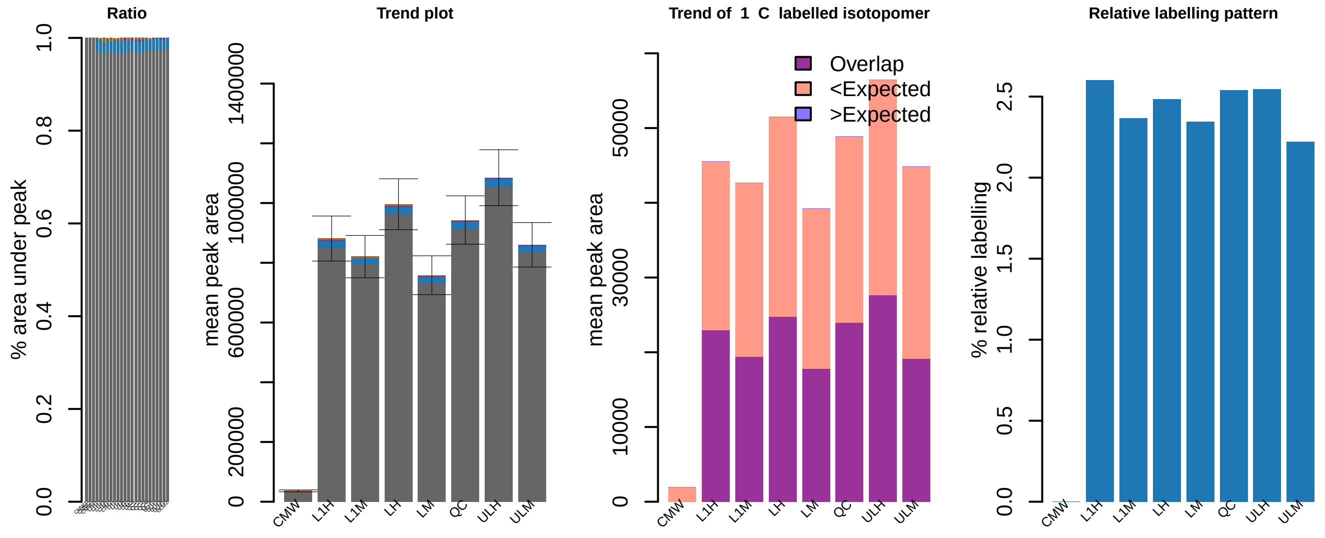

[FA trihydroxy(4:0)] 2,3,4−trihydroxy−butanoic acid

G2

Formula: C4H8O5 Mass: 136.037 Std.RT: 786.4229964 Ion: NEG

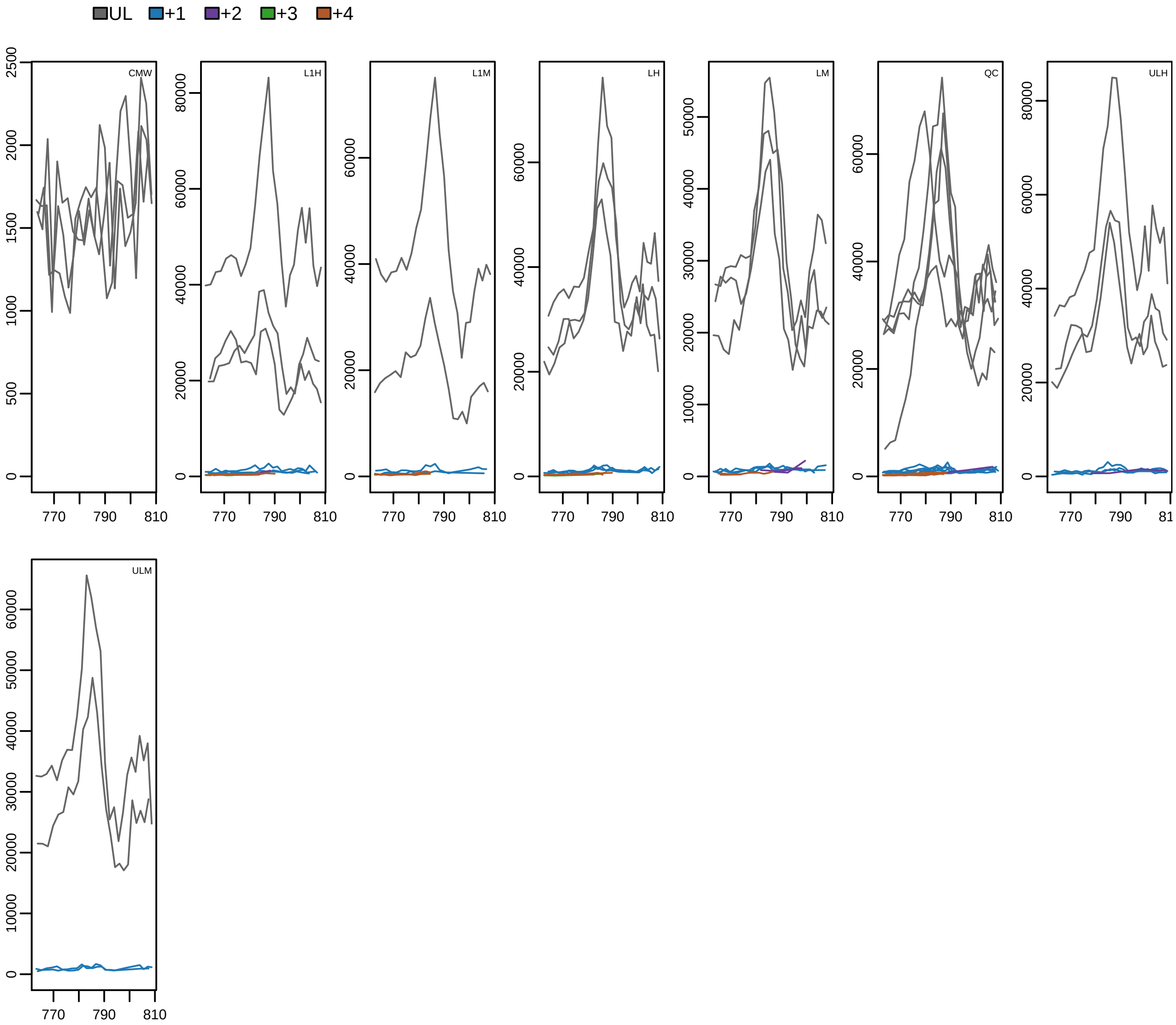

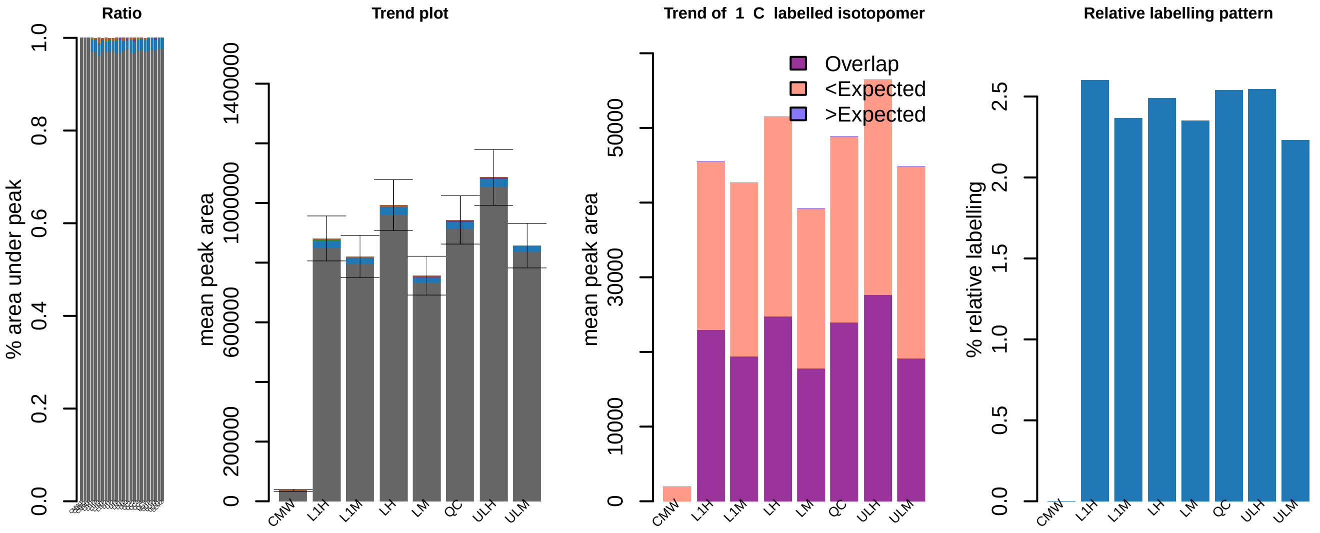

2−Hydroxy−3−oxopropanoate

Formula: C3H4O4 Mass: 104.011 Std.RT: 1008.5145852 Ion: NEG

G1

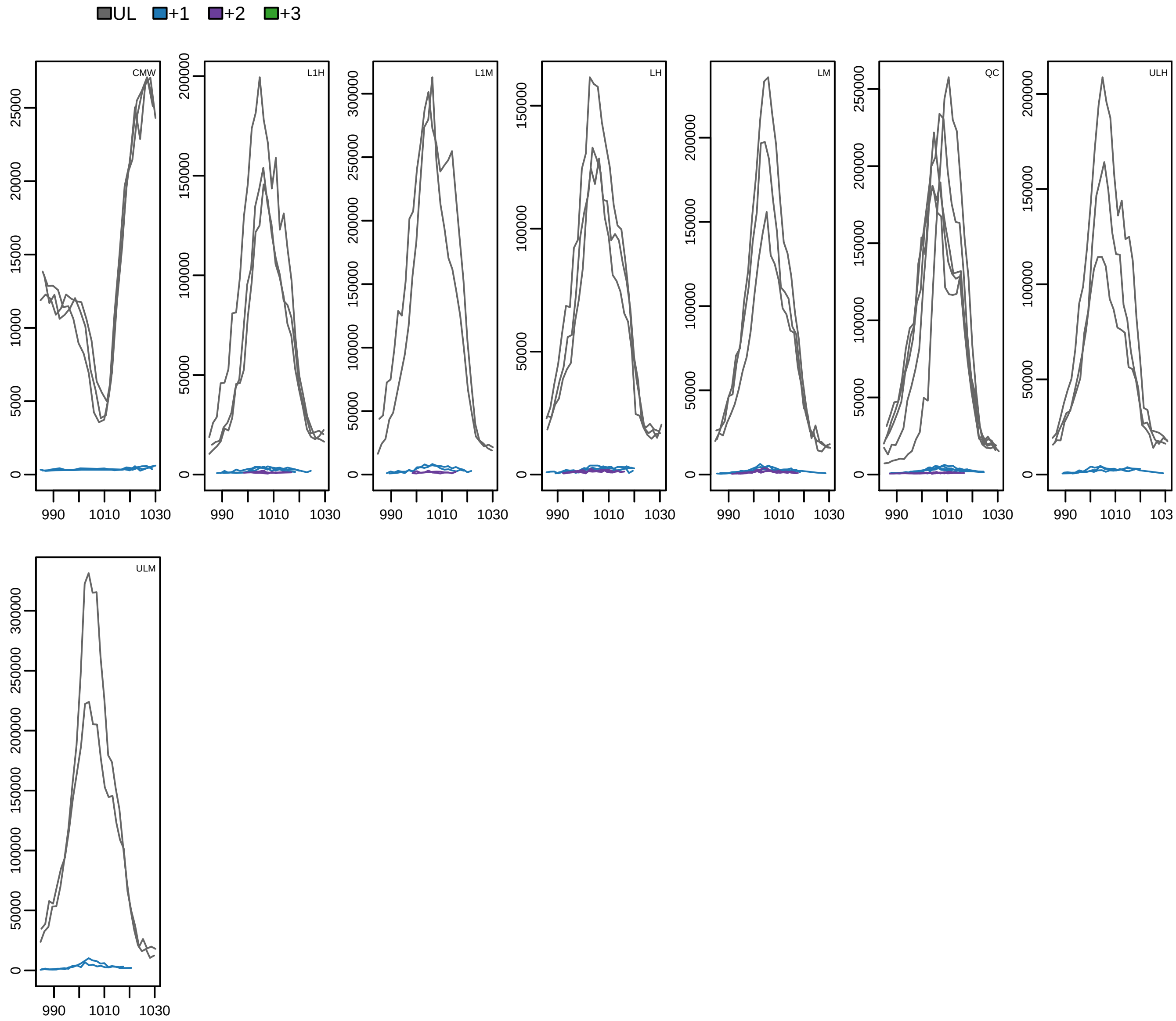

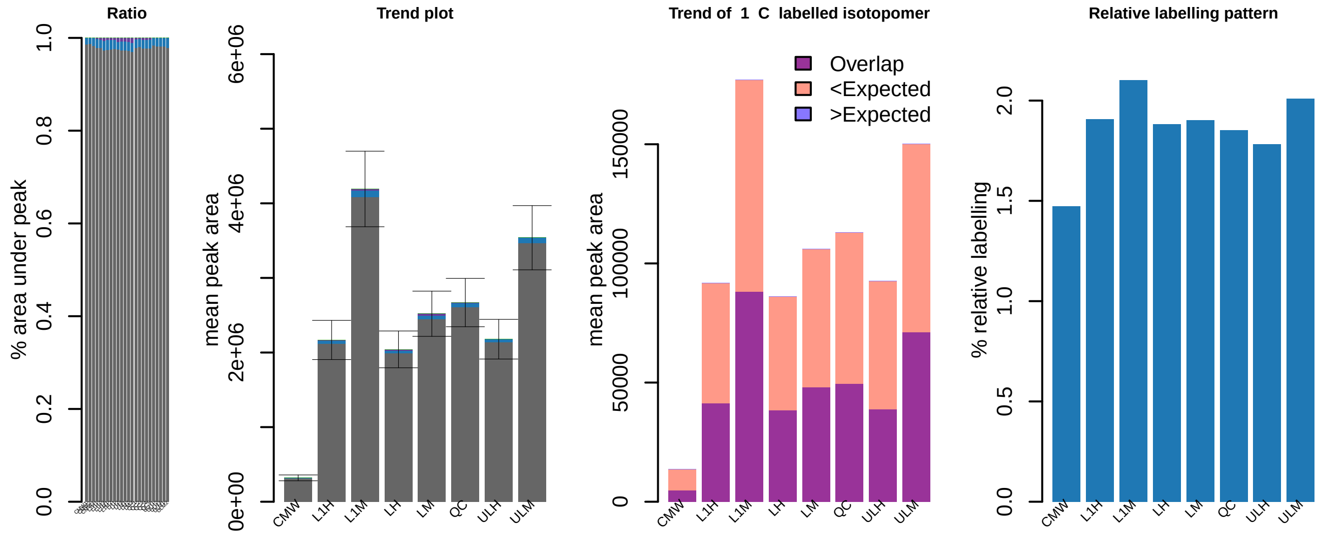

(R)−3−((R)−3−Hydroxybutanoyloxy)butanoate

Formula: C8H14O5 Mass: 190.084 Std.RT: 813.8952486 Ion: NEG

G1

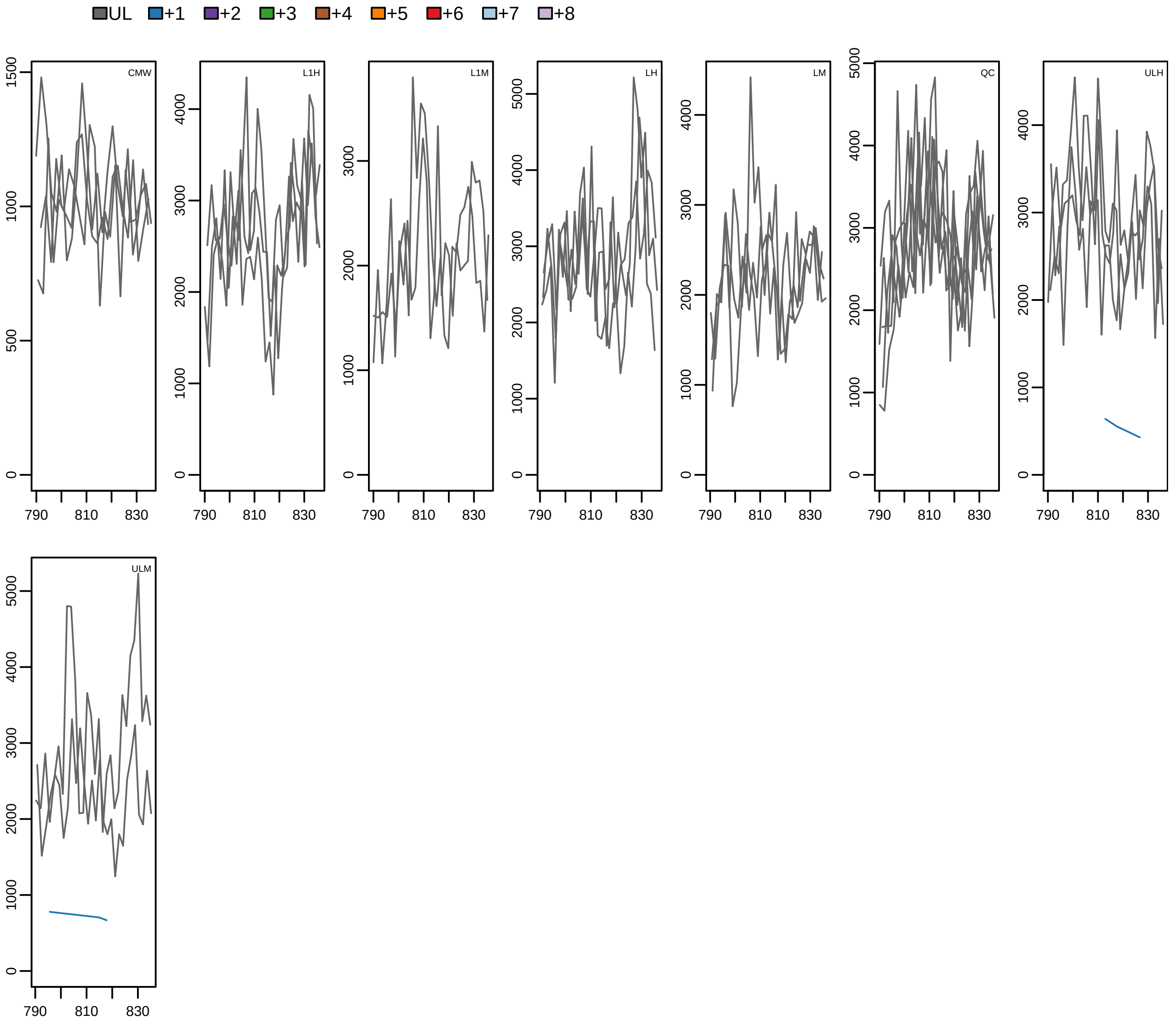

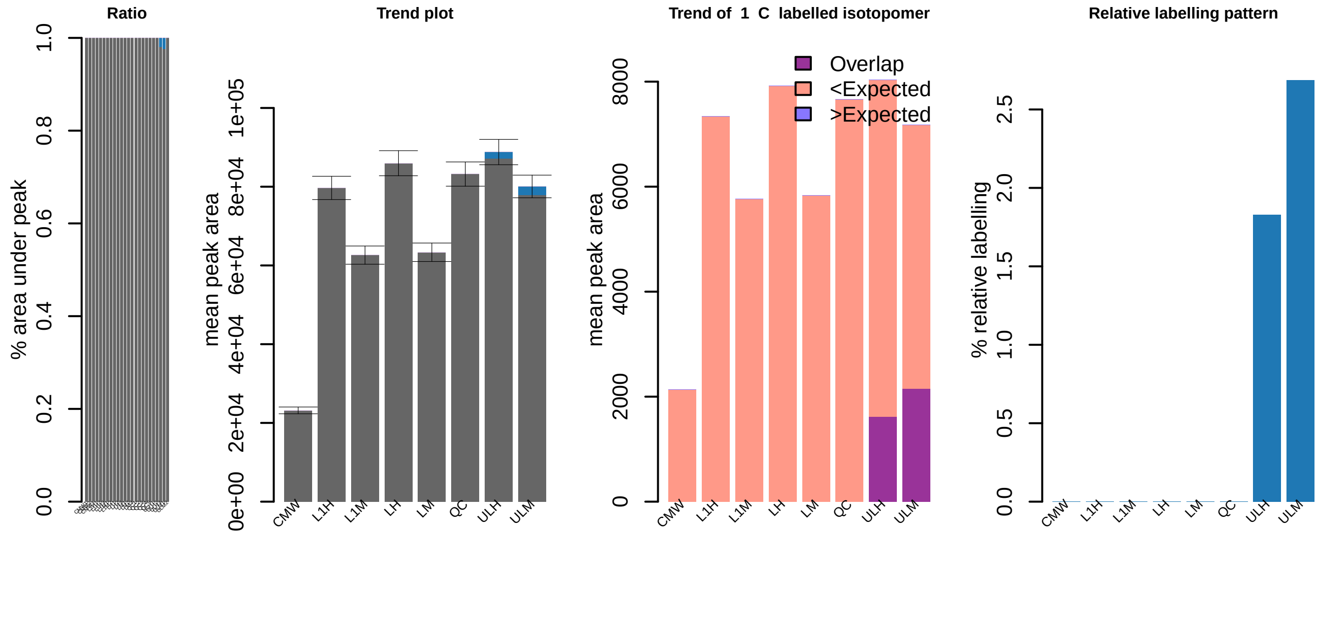

2−Acetolactate

Formula: C5H8O4 Mass: 132.042 Std.RT: 948.5175042 Ion: NEG

G1

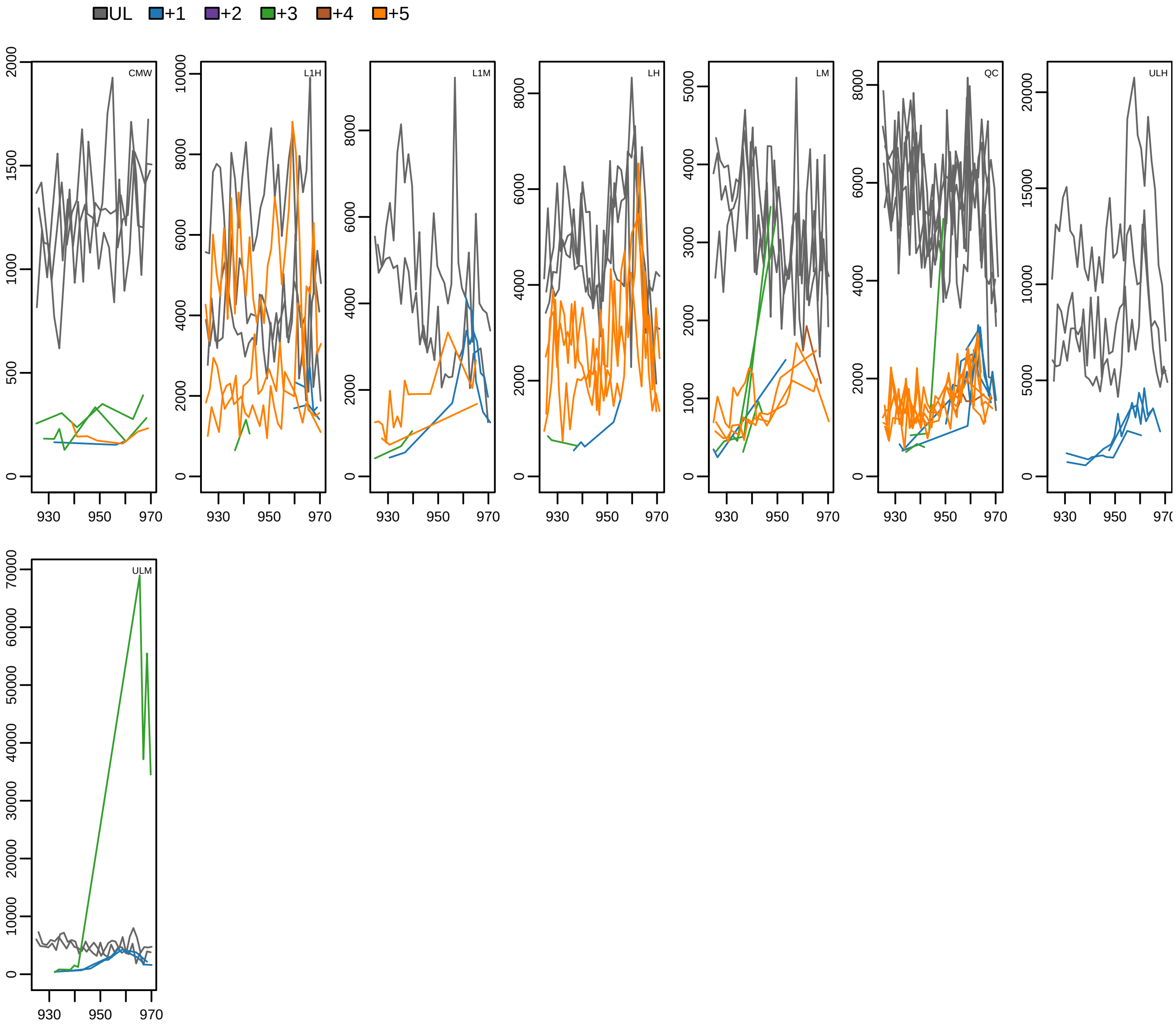

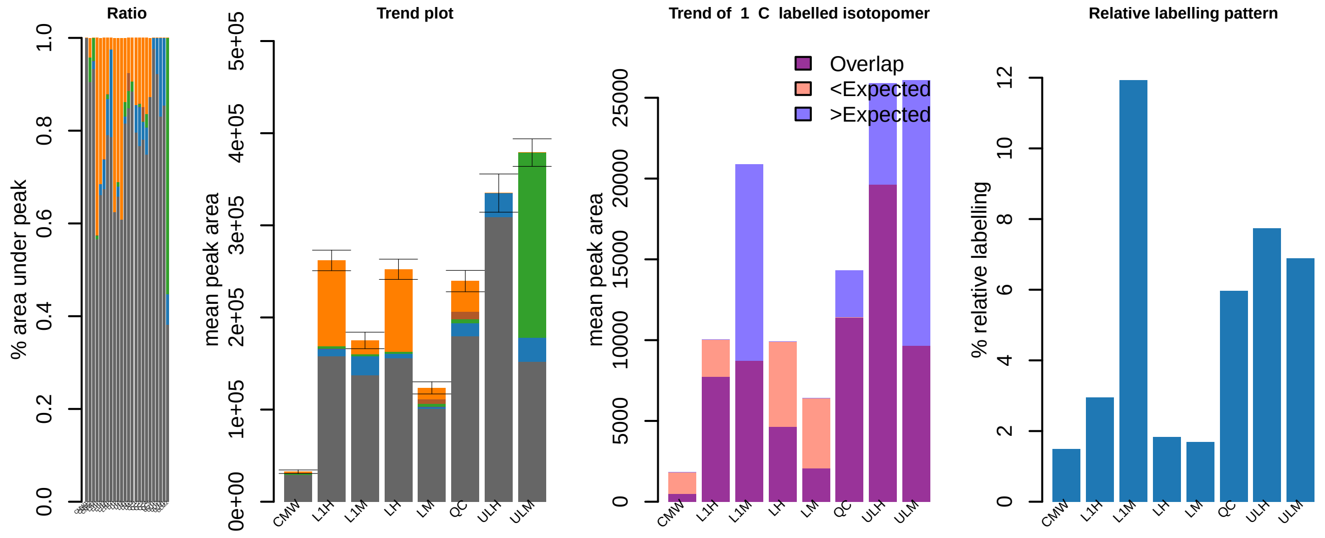

4−Methylene−L−glutamate

Formula: C6H9NO4 Mass: 159.053 Std.RT: 824.7282462 Ion: NEG

G1

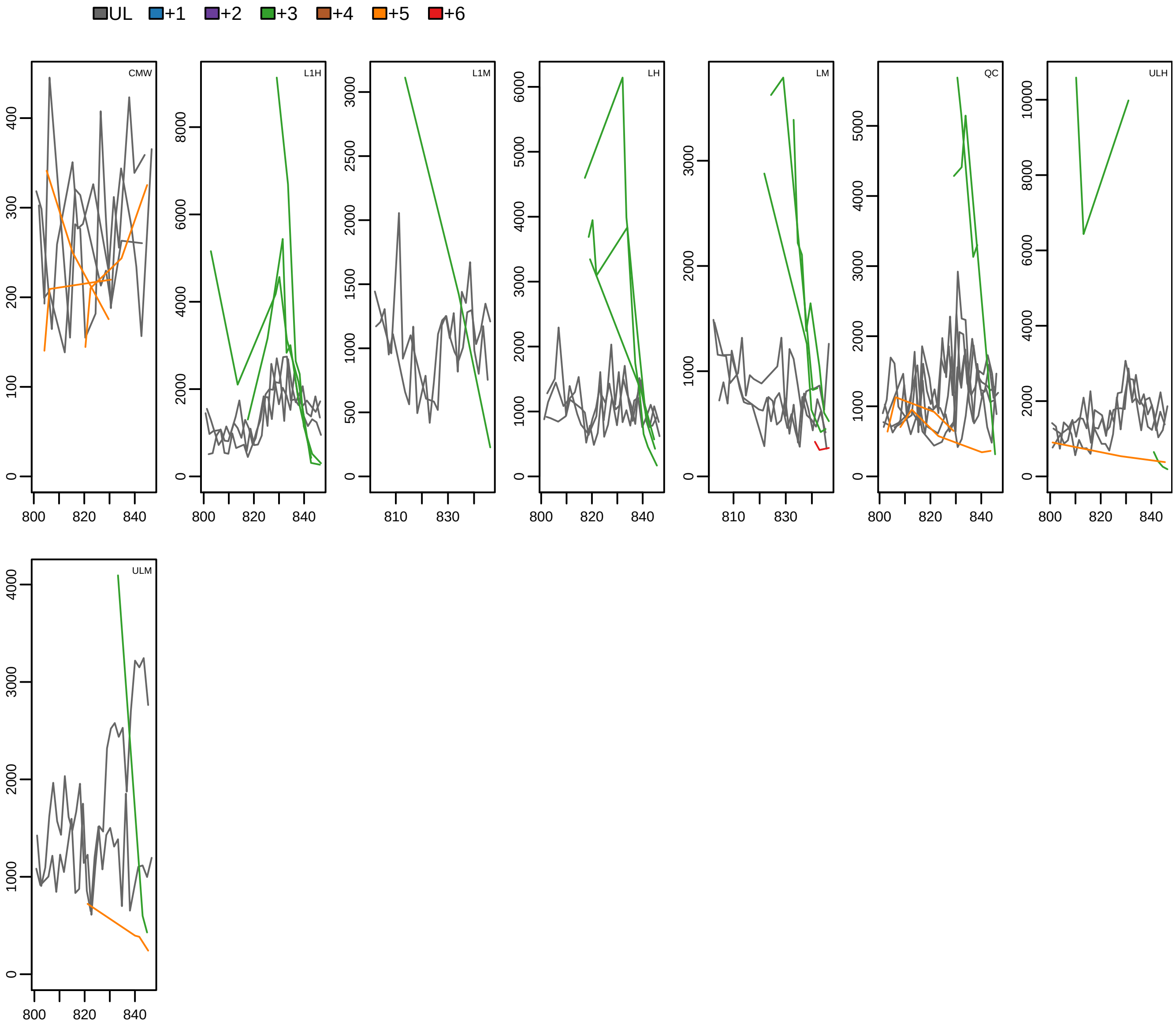

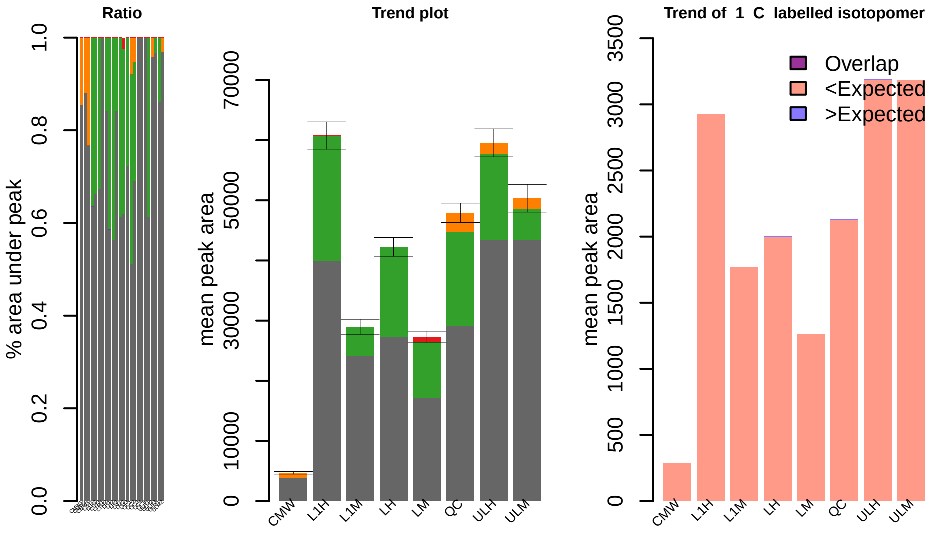

2−Oxoglutarate

Formula: C5H6O5 Mass: 146.022 Std.RT: 991.6538418 Ion: NEG

G1

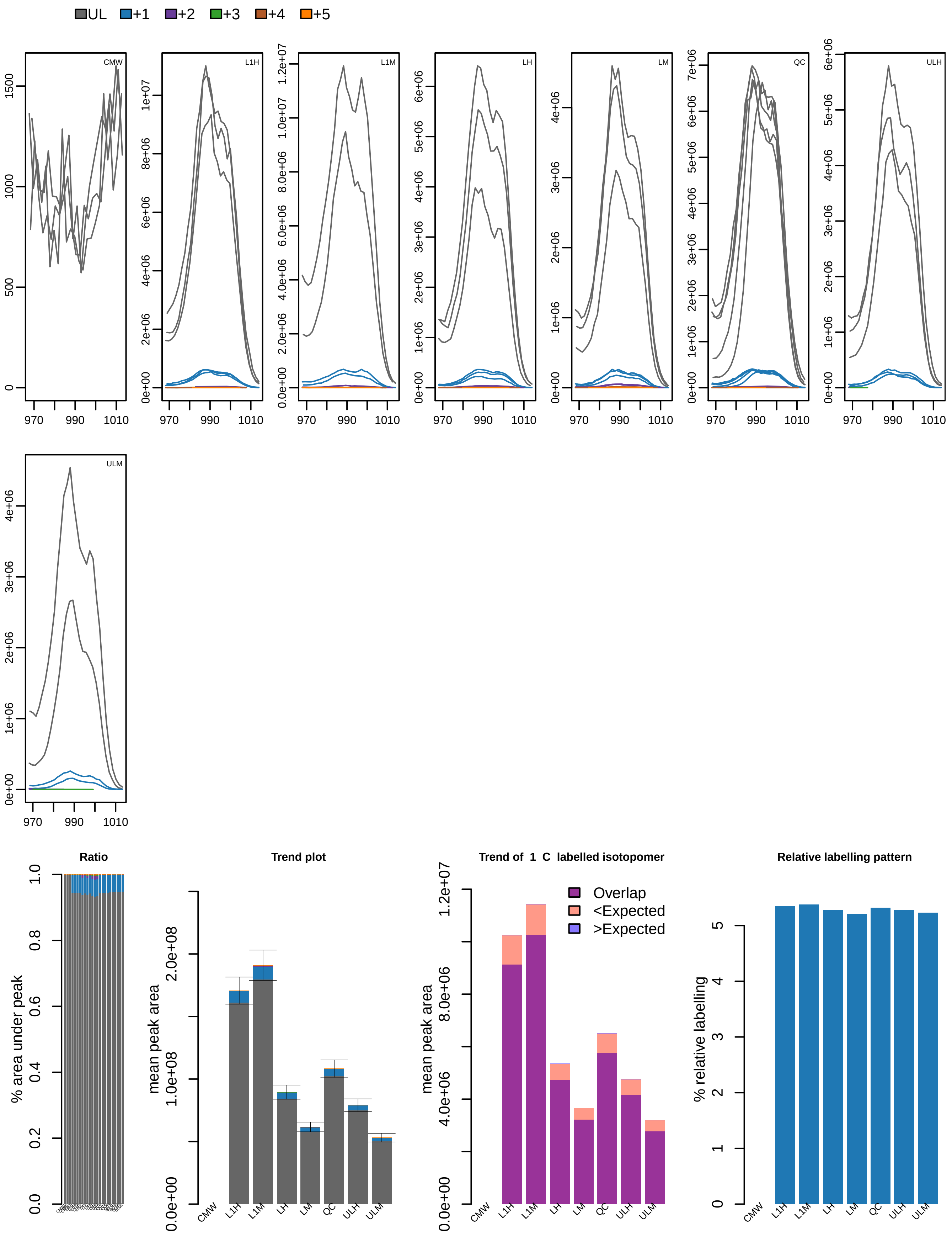

CoA

Formula: C21H36N7O16P3S Mass: 767.115 Std.RT: 880.4337036 Io

G1

UL +1 +2 +3 +4 +5 +6 +7 +8 +9 +10 +11 +12 +13

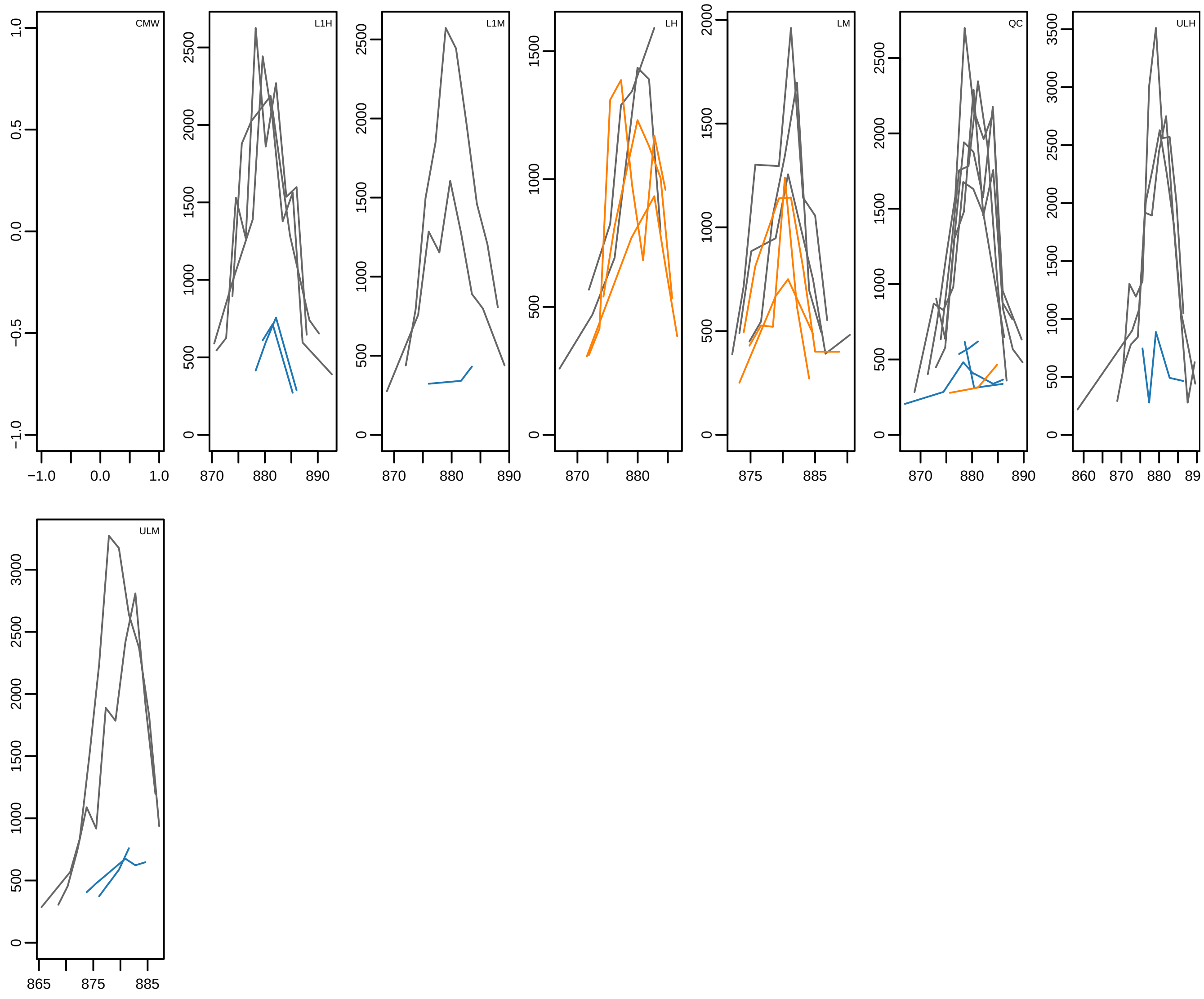

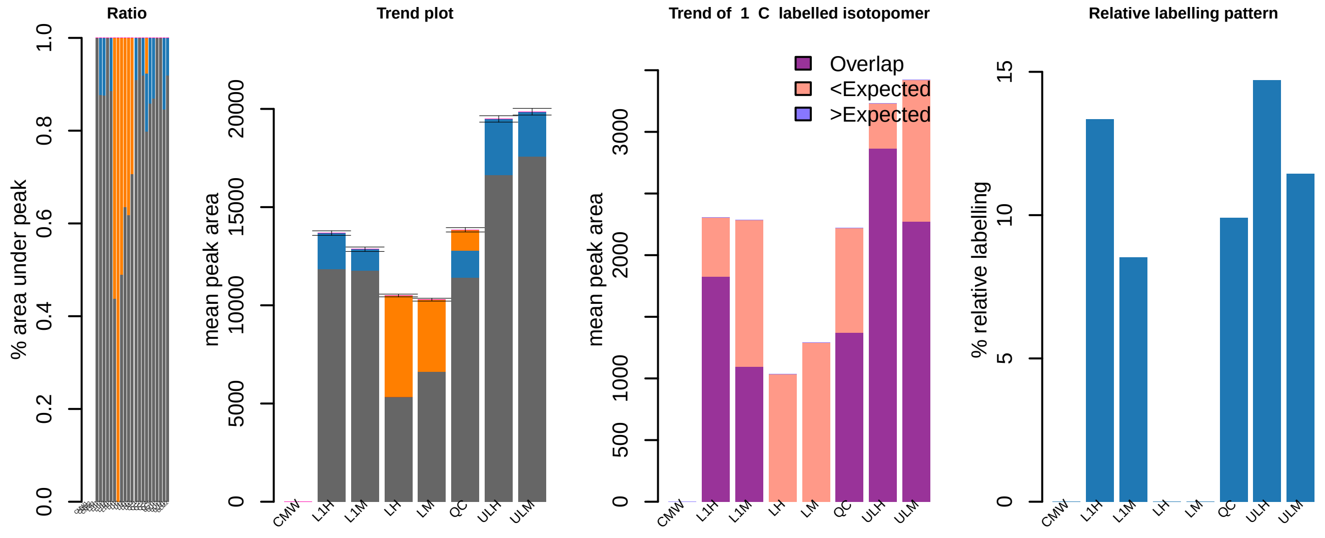

Oxaloacetate

Formula: C4H4O5 Mass: 132.006 Std.RT: 1024.5037512 Ion: NEG

G1

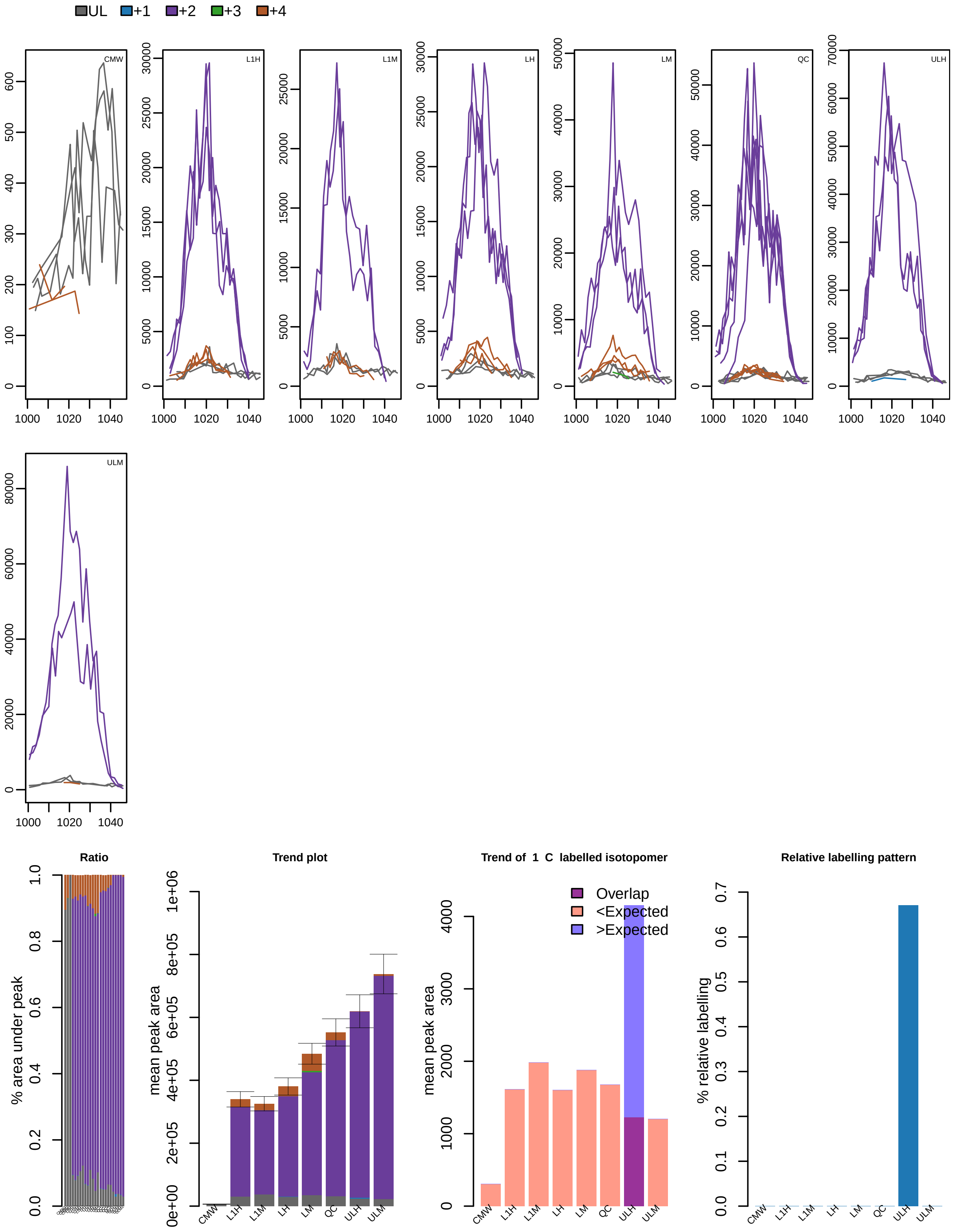

Citrate

Formula: $C6H8O7$ Mass: 192.027 Std.RT: 1150.420008 Ion: NEG

G1

UL +1 +2 +3 +4 +5 +6

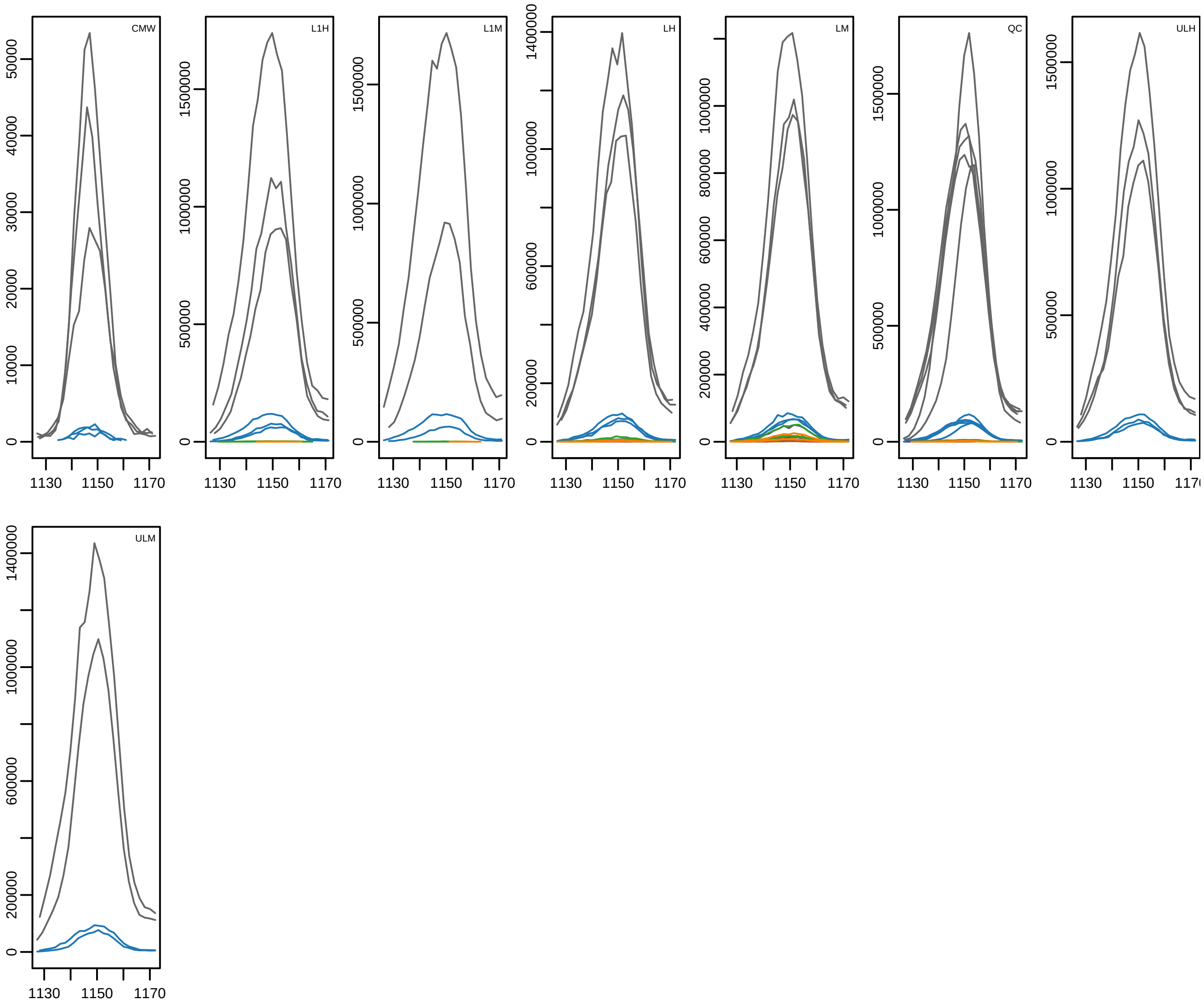

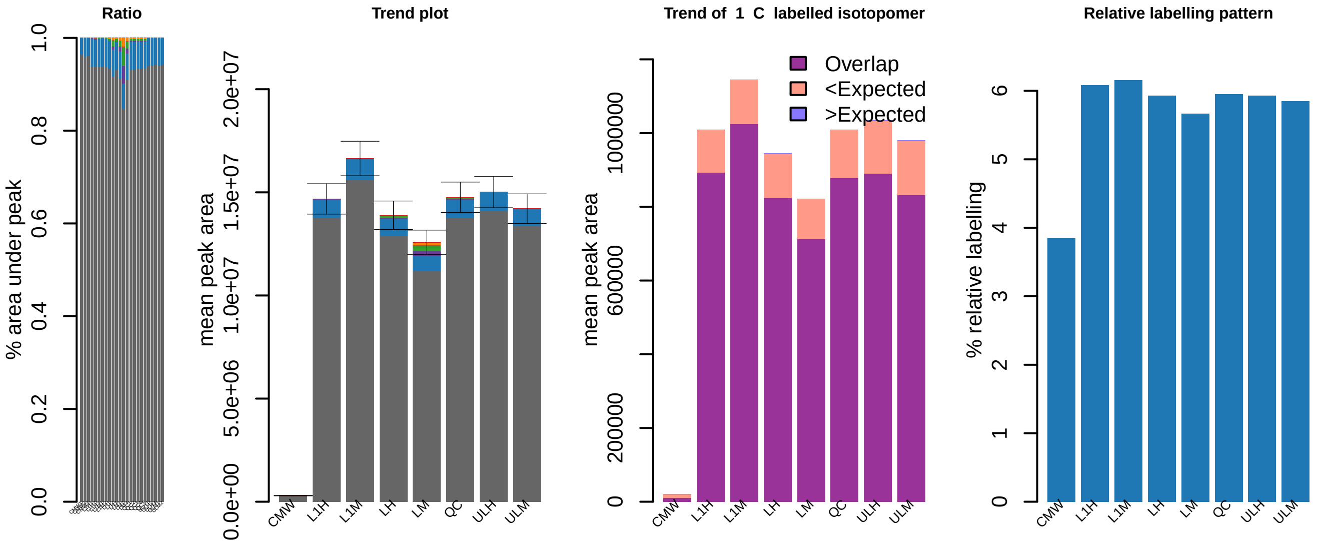

(S)−Malate

Formula: $C_4H_6O_5$ Mass: 134.022 Std.RT: 1023.5170872 Ion: NEG

G1

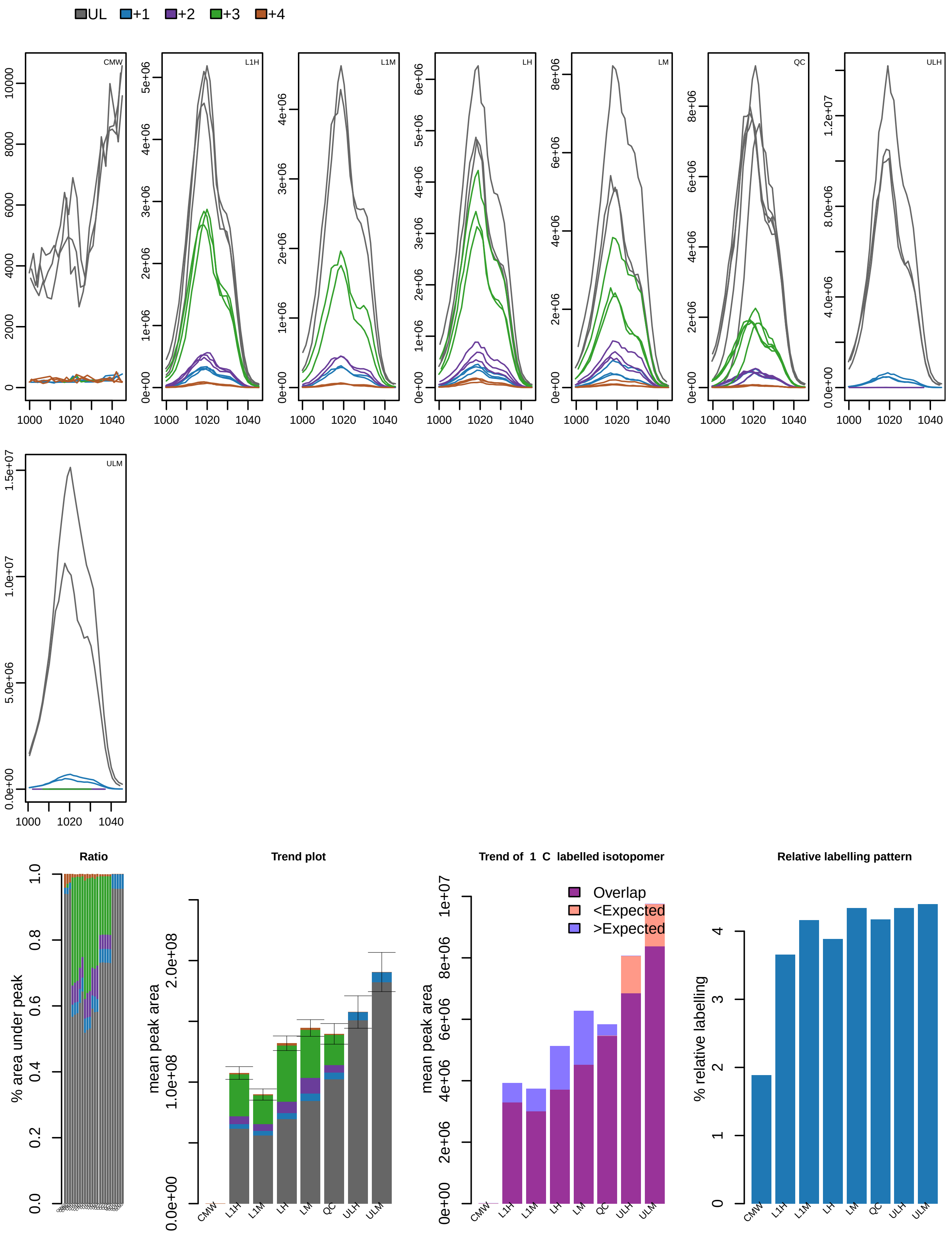

(S)−Malate

G2

Formula: C4H6O5 Mass: 134.022 Std.RT: 1023.5170872 Ion: NEG

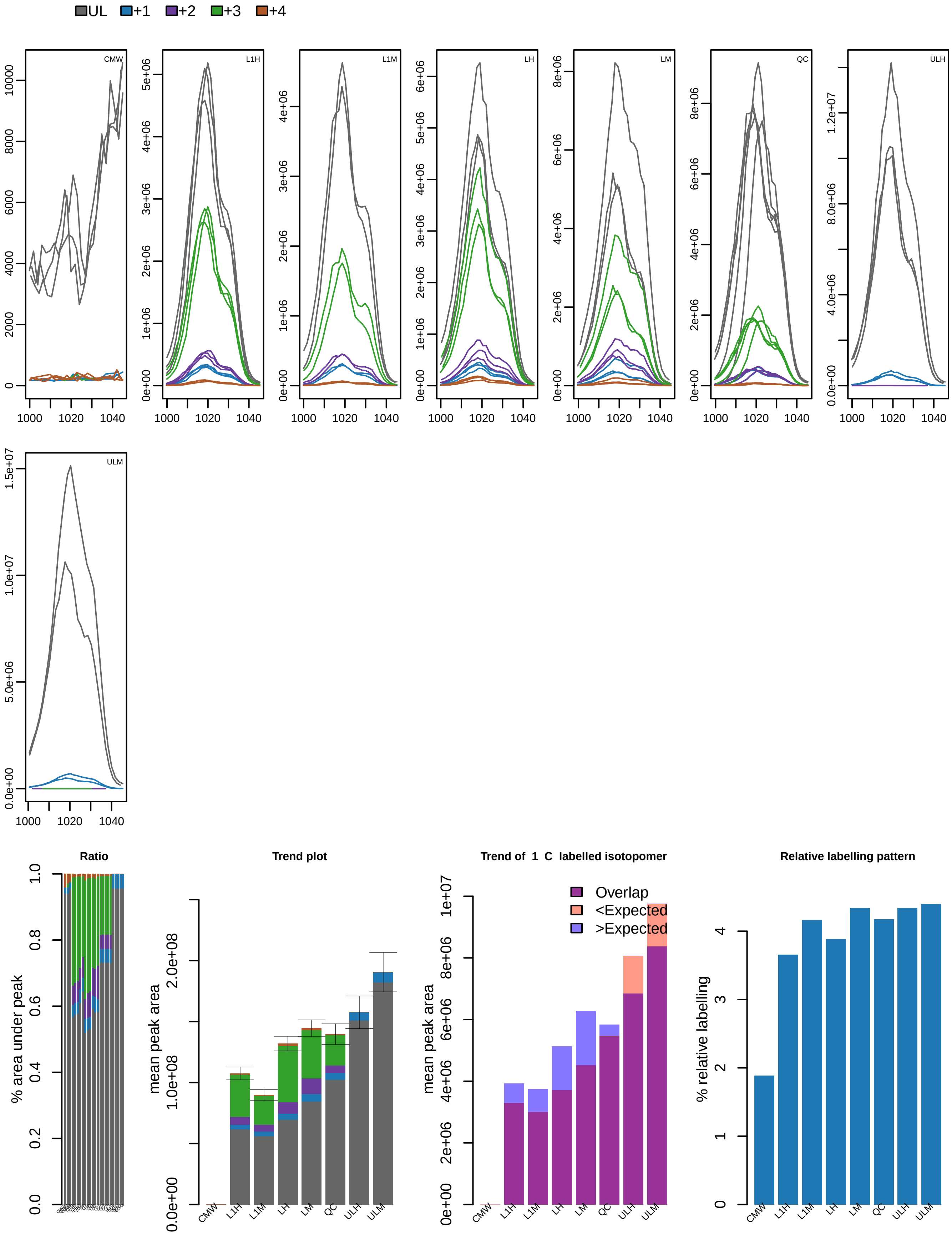

cis−Aconitate

Formula: C6H6O6 Mass: 174.016 Std.RT: 1136.7508392 Ion: NEG

G1

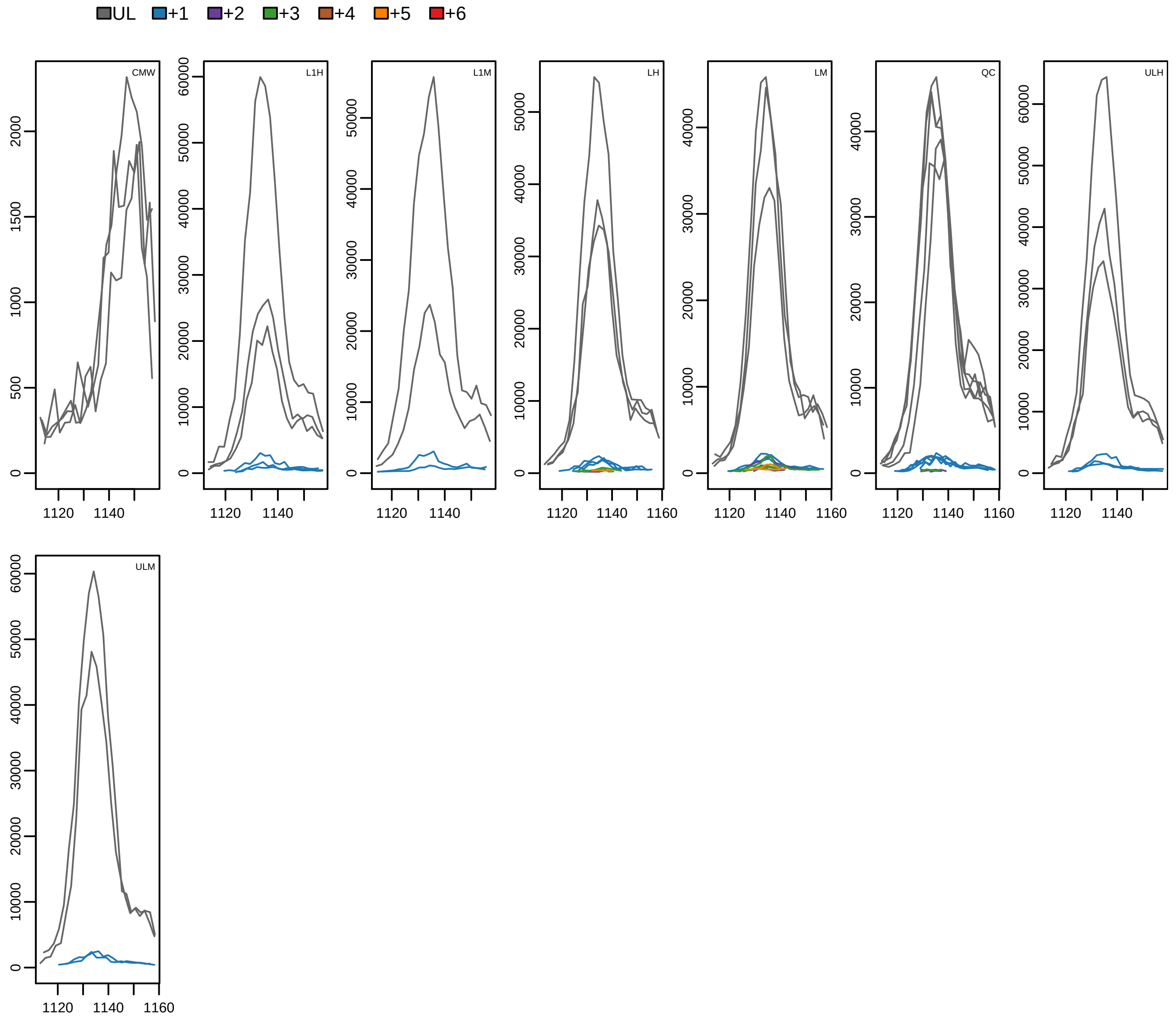

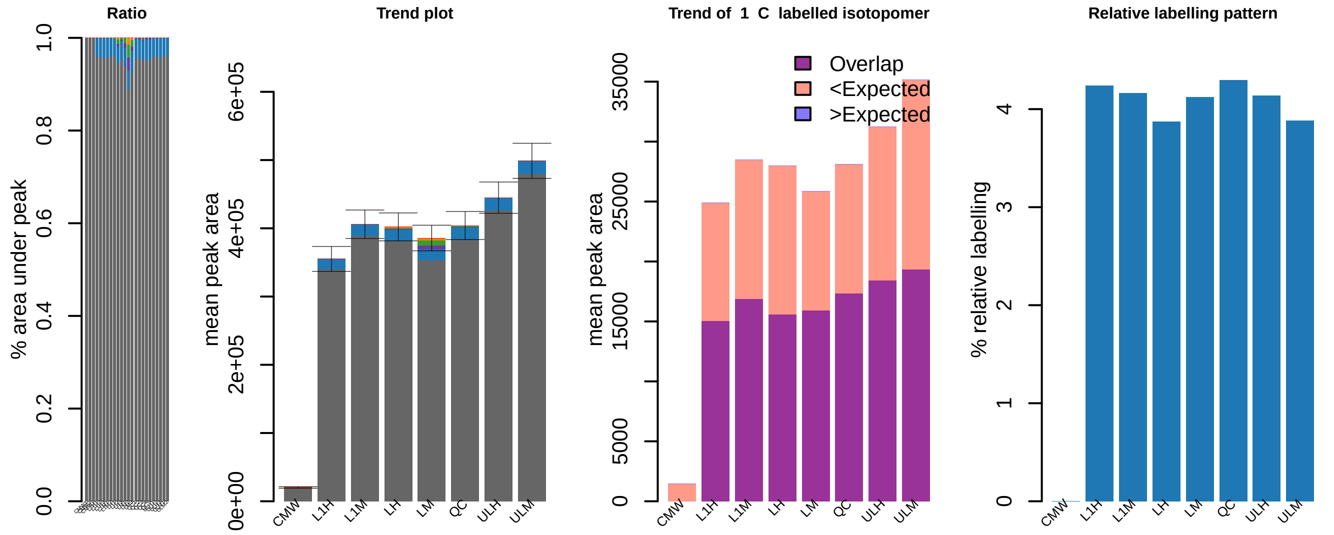

Succinate

Formula: $C_4H_6O_4$ Mass: 118.027 Std.RT: 967.3782504 Ion: NEG

G1

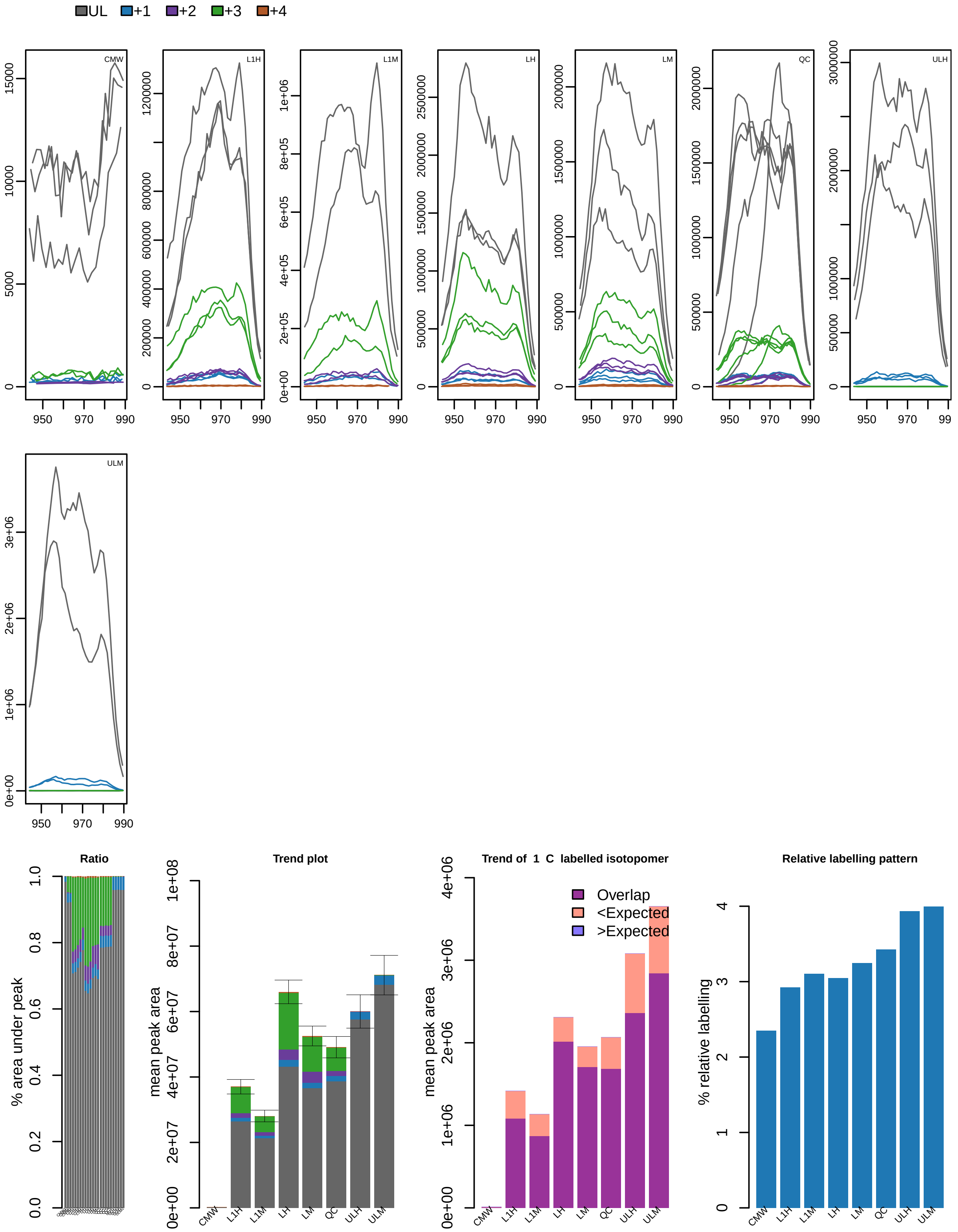

Succinate

Formula: C4H6O4 Mass: 118.027 Std.RT: 967.3782504 Ion: NEG

G2

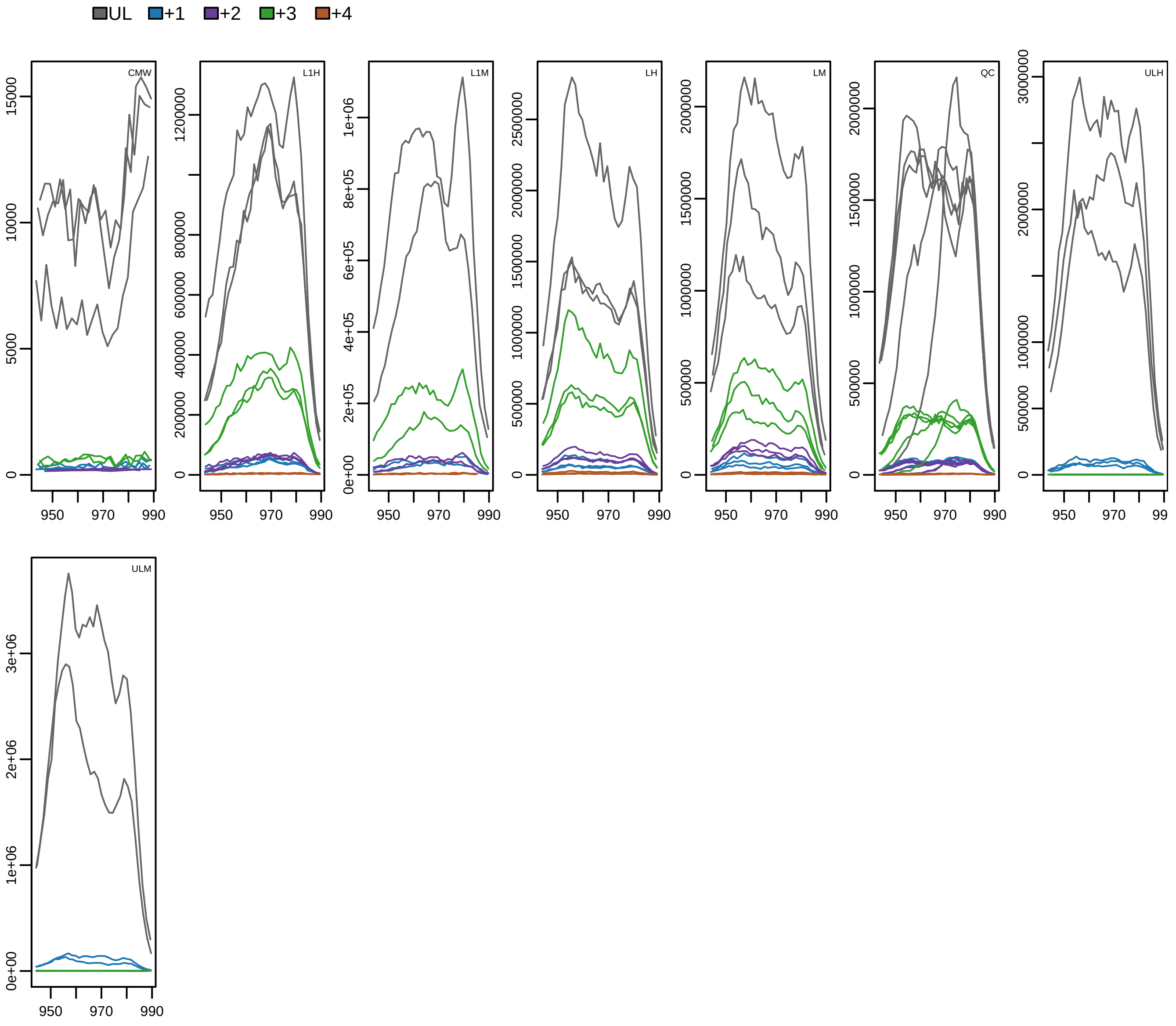

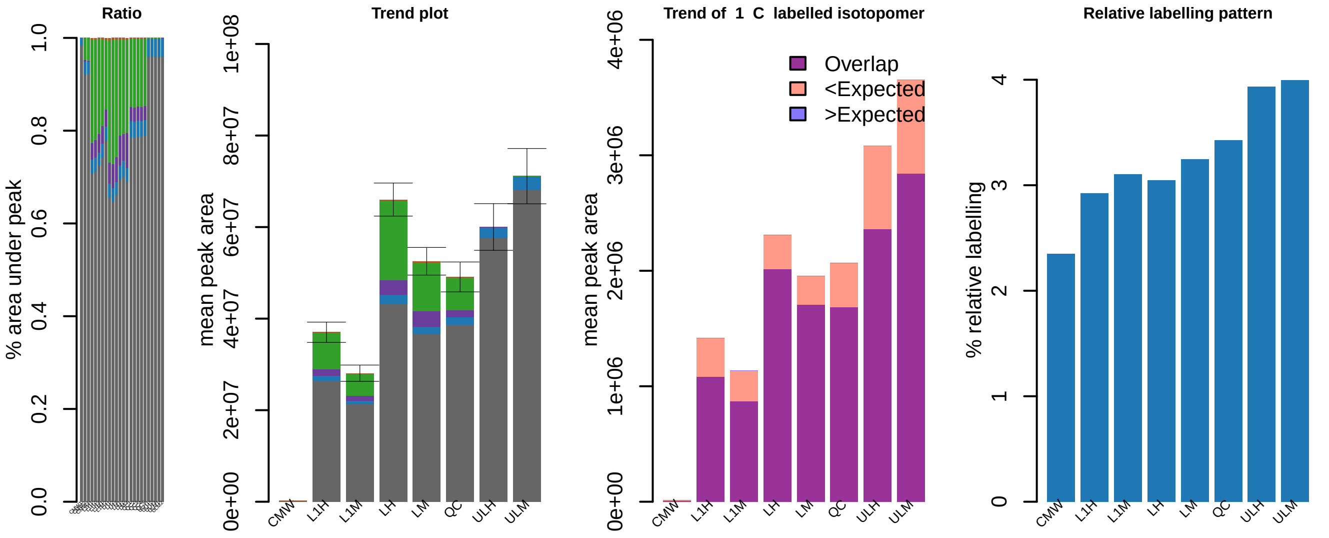

GDP−mannose

G1

Formula: C16H25N5O16P2 Mass: 605.077 Std.RT: 1168.4676162 Ior

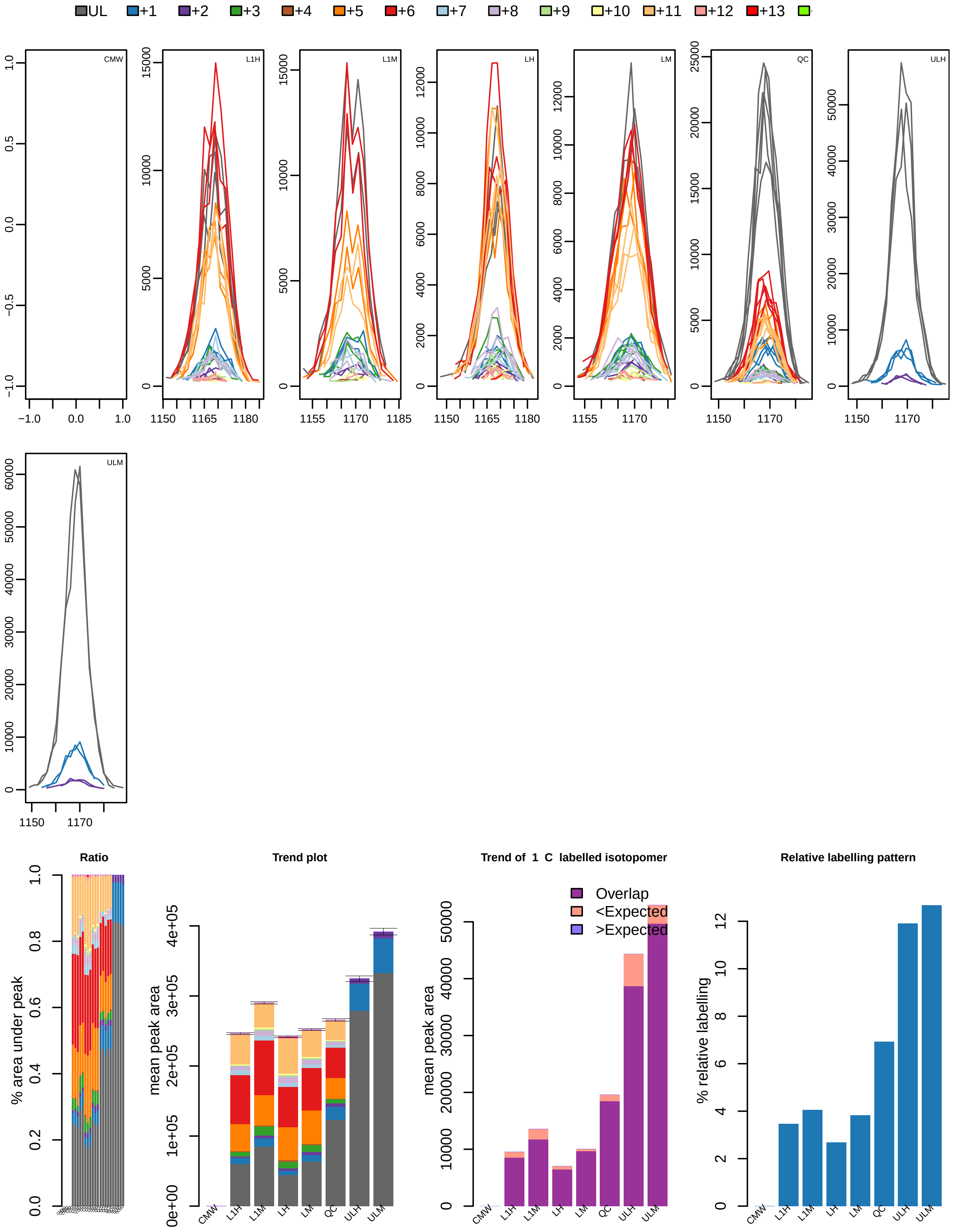

D−Sorbitol

Formula: C6H14O6 Mass: 182.079 Std.RT: 902.0710836 Ion: NEG

G1

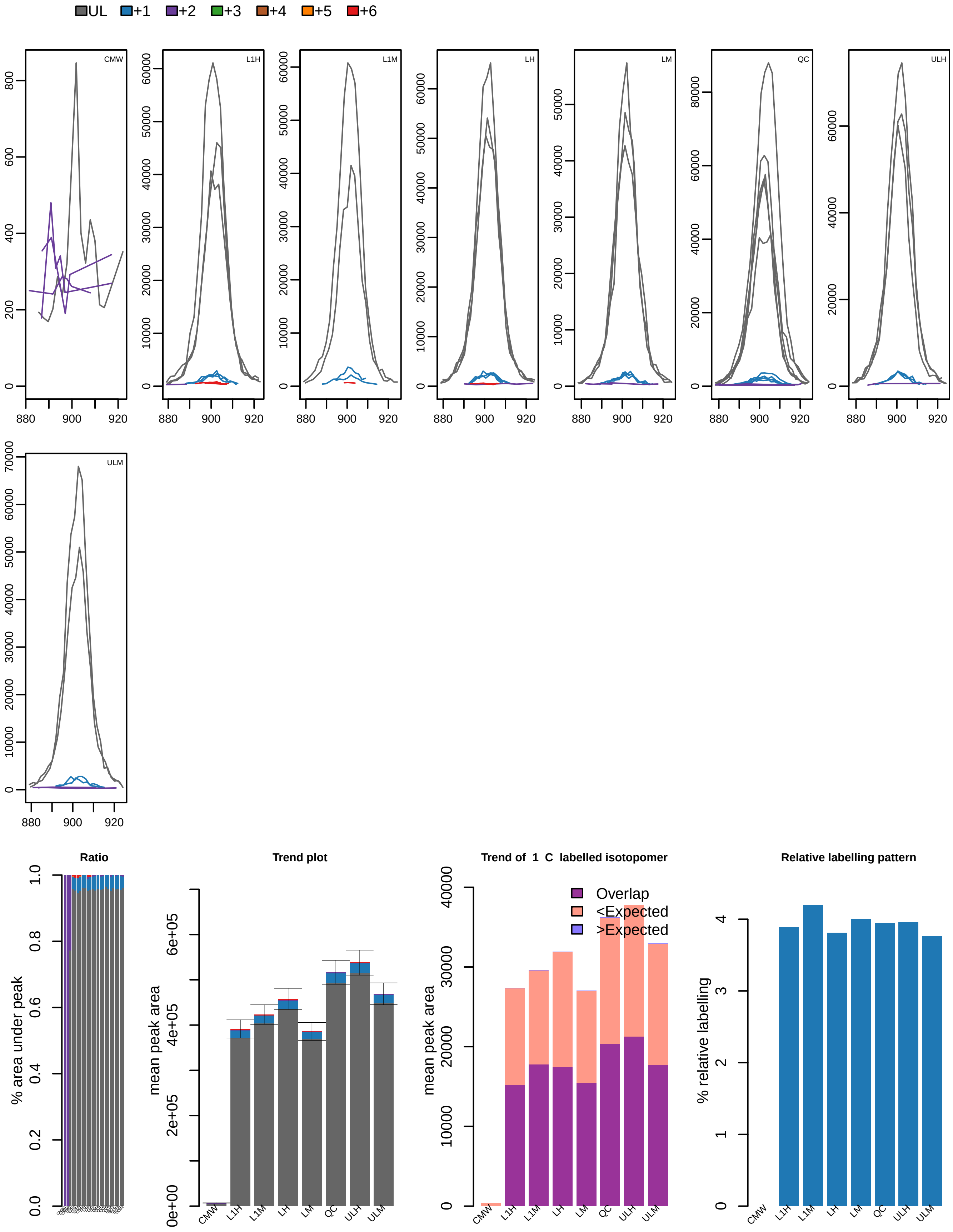

D−Fructose

Formula: $C_6H_{12}O_6$ Mass: 180.063 Std.RT: 870.685257 Ion: NEG

G1

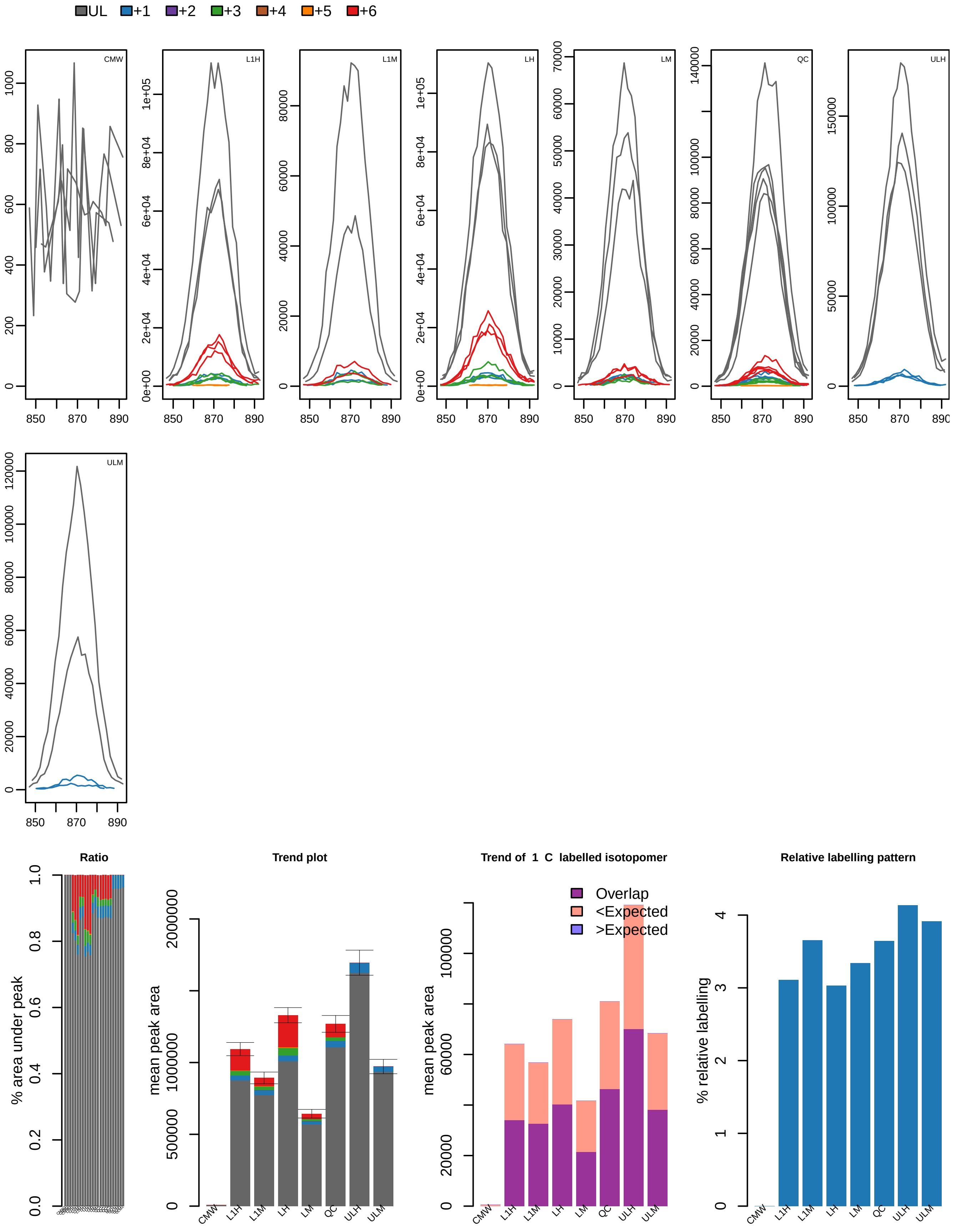

D−Galactosamine

Formula: C6H13NO5 Mass: 179.079 Std.RT: 723.239751 Ion: NEG

G1

UL +1 +2 +3 +4 +5 +6

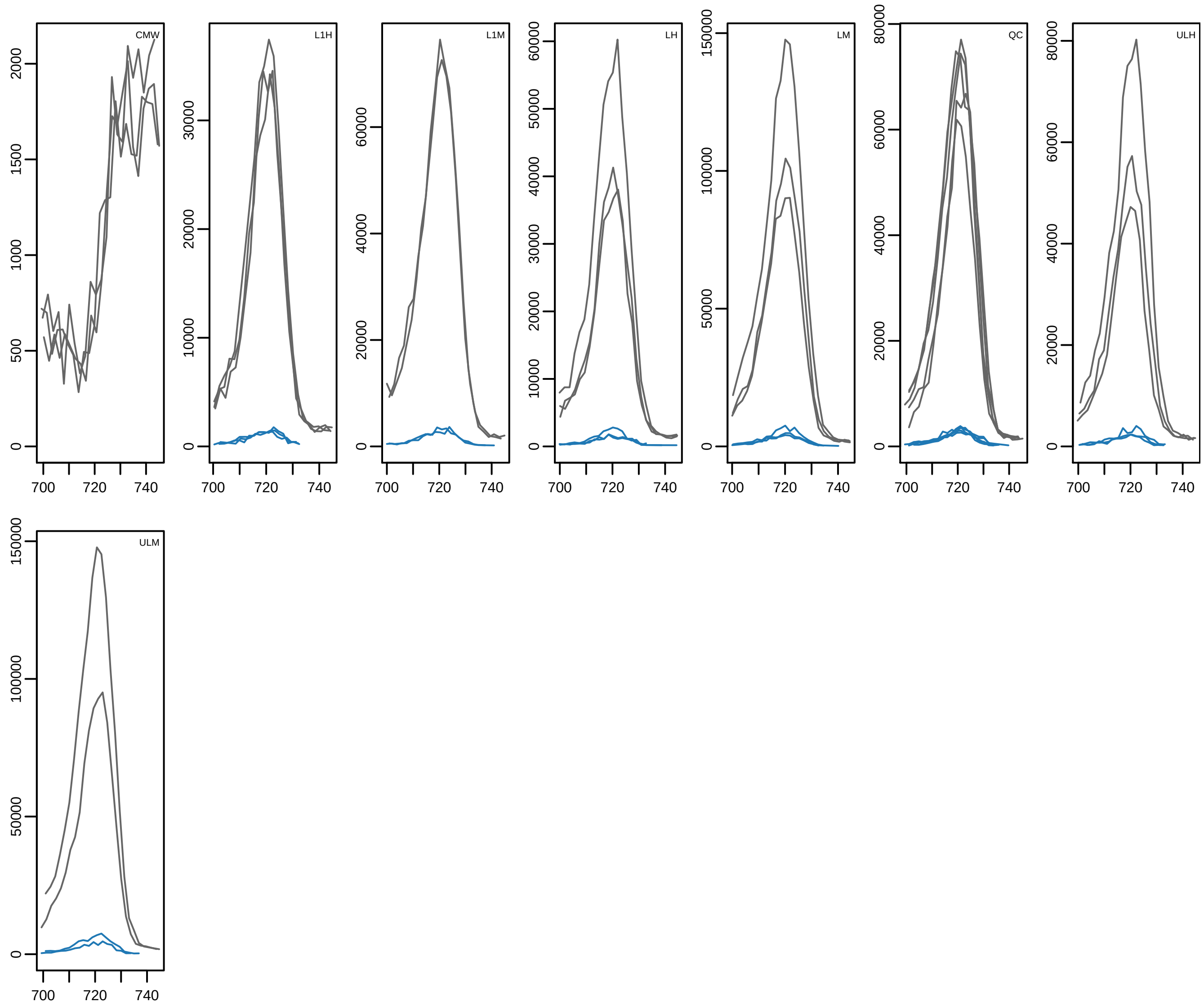

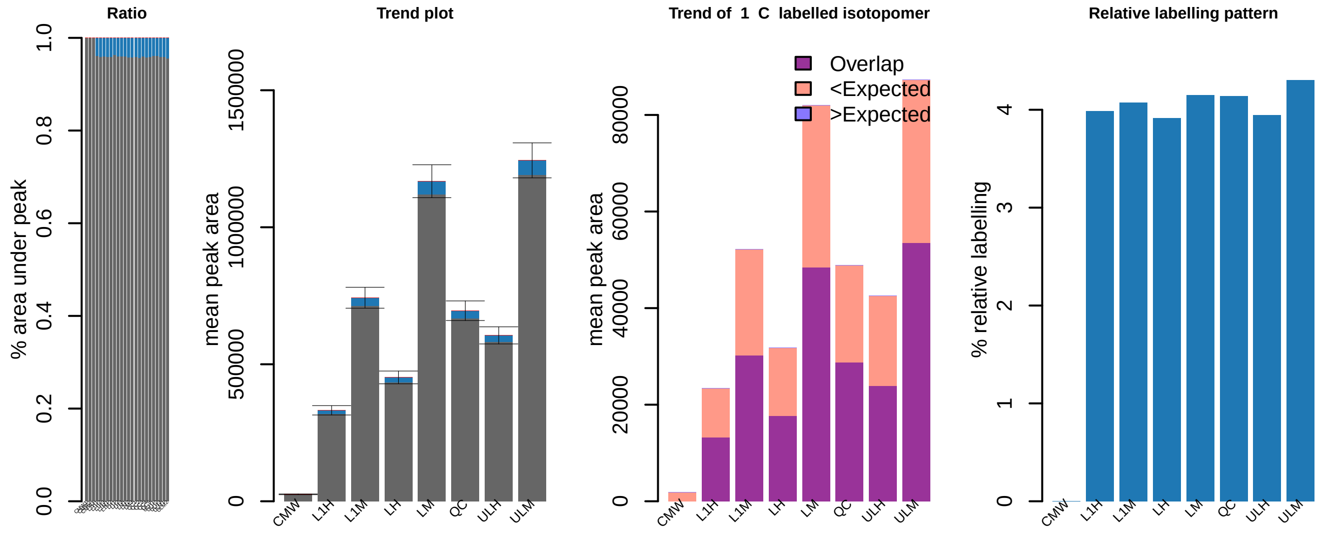

Glycerol

Formula: C3H8O3 Mass: 92.047 Std.RT: 639.969498 Ion: NEG

G1

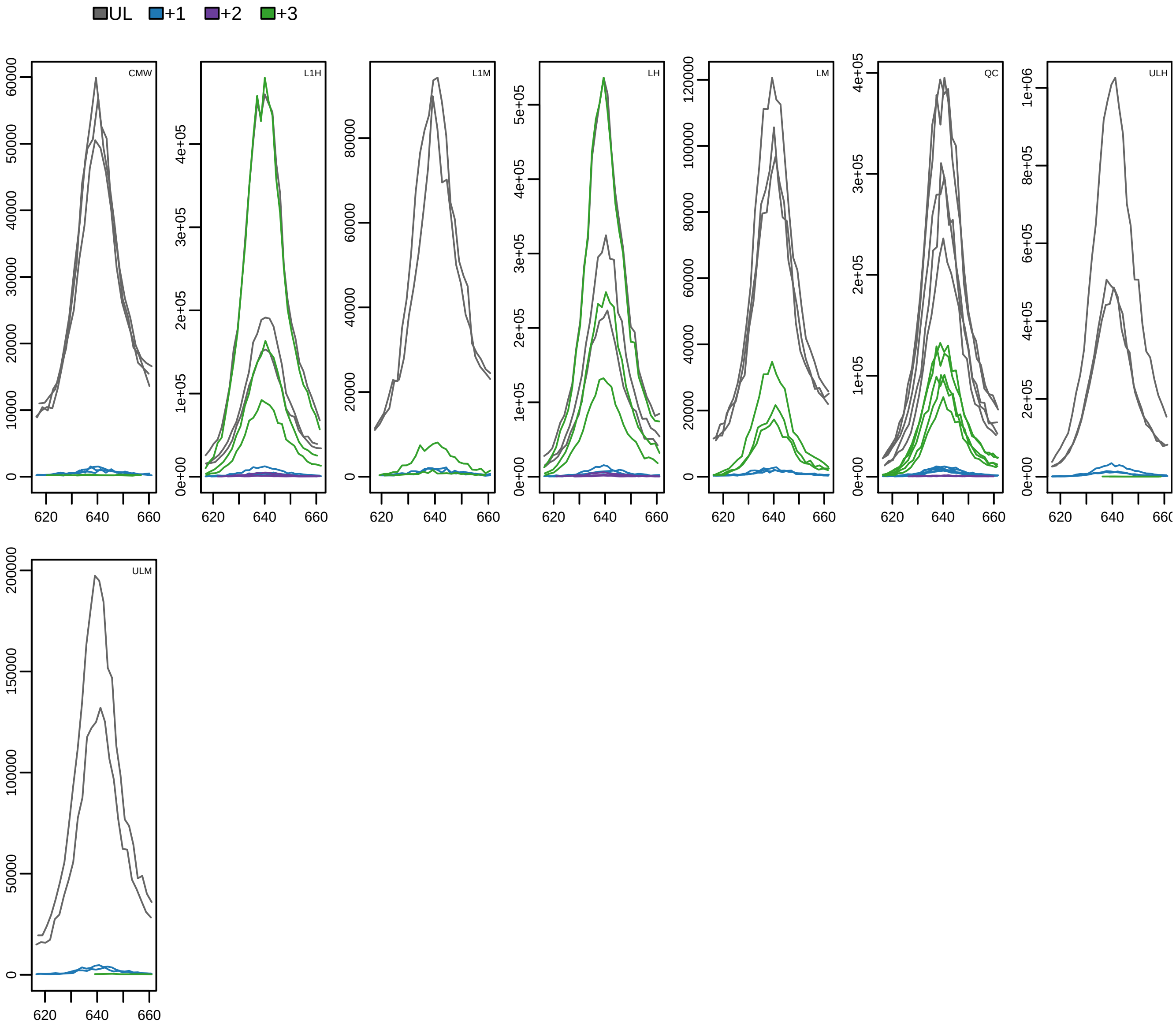

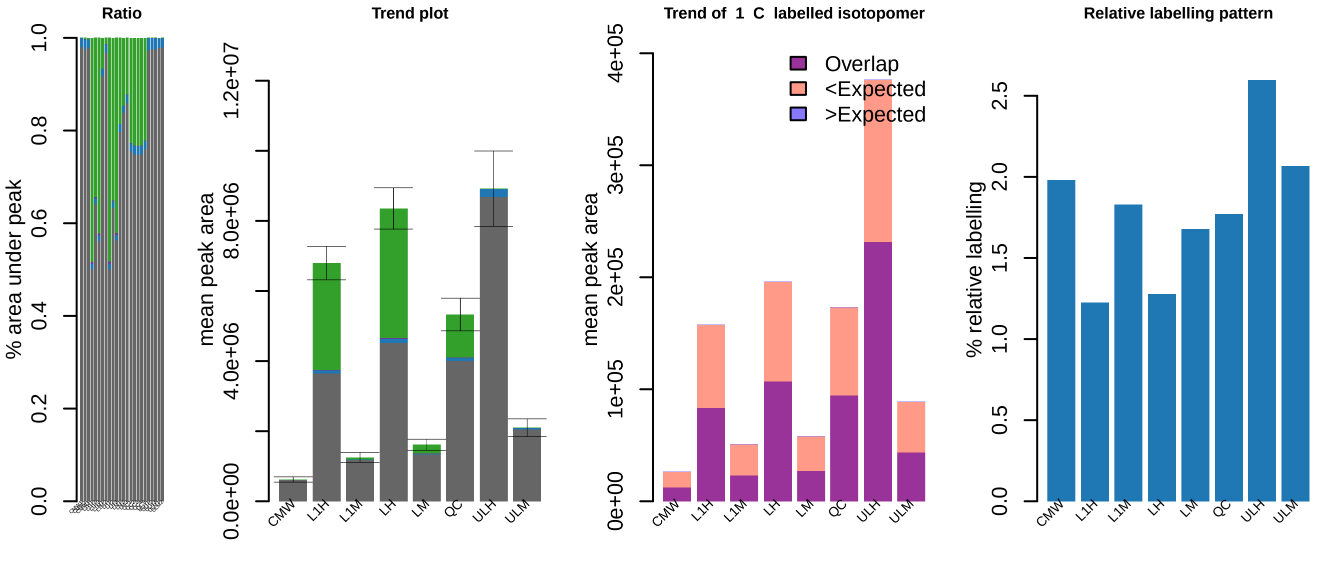

Sucrose

Formula: C12H22O11 Mass: 342.116 Std.RT: 988.8873366 Ion: NEG

G1

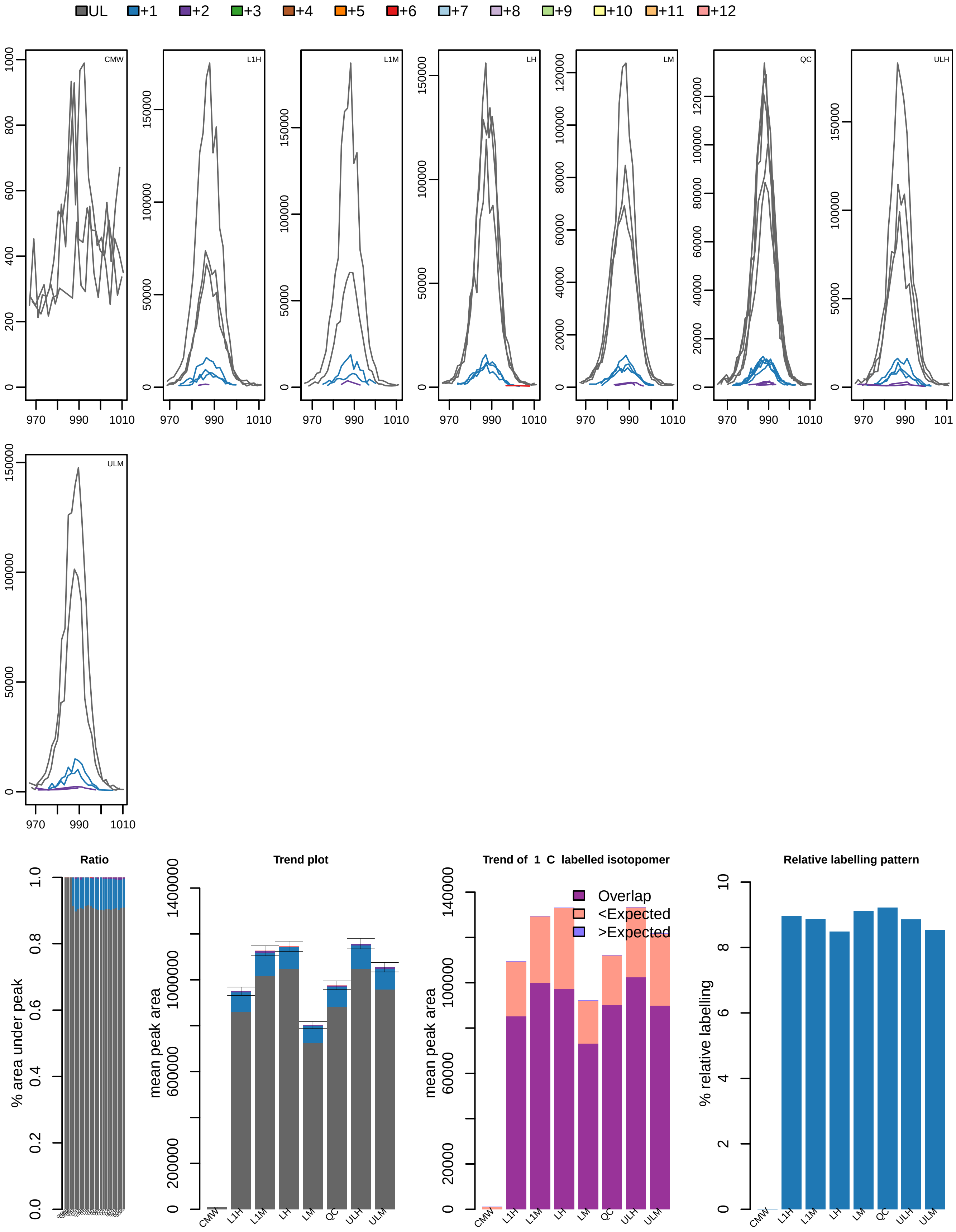

D−Glycerate

Formula: C3H6O4 Mass: 106.027 Std.RT: 737.4837522 Ion: NEG

G1

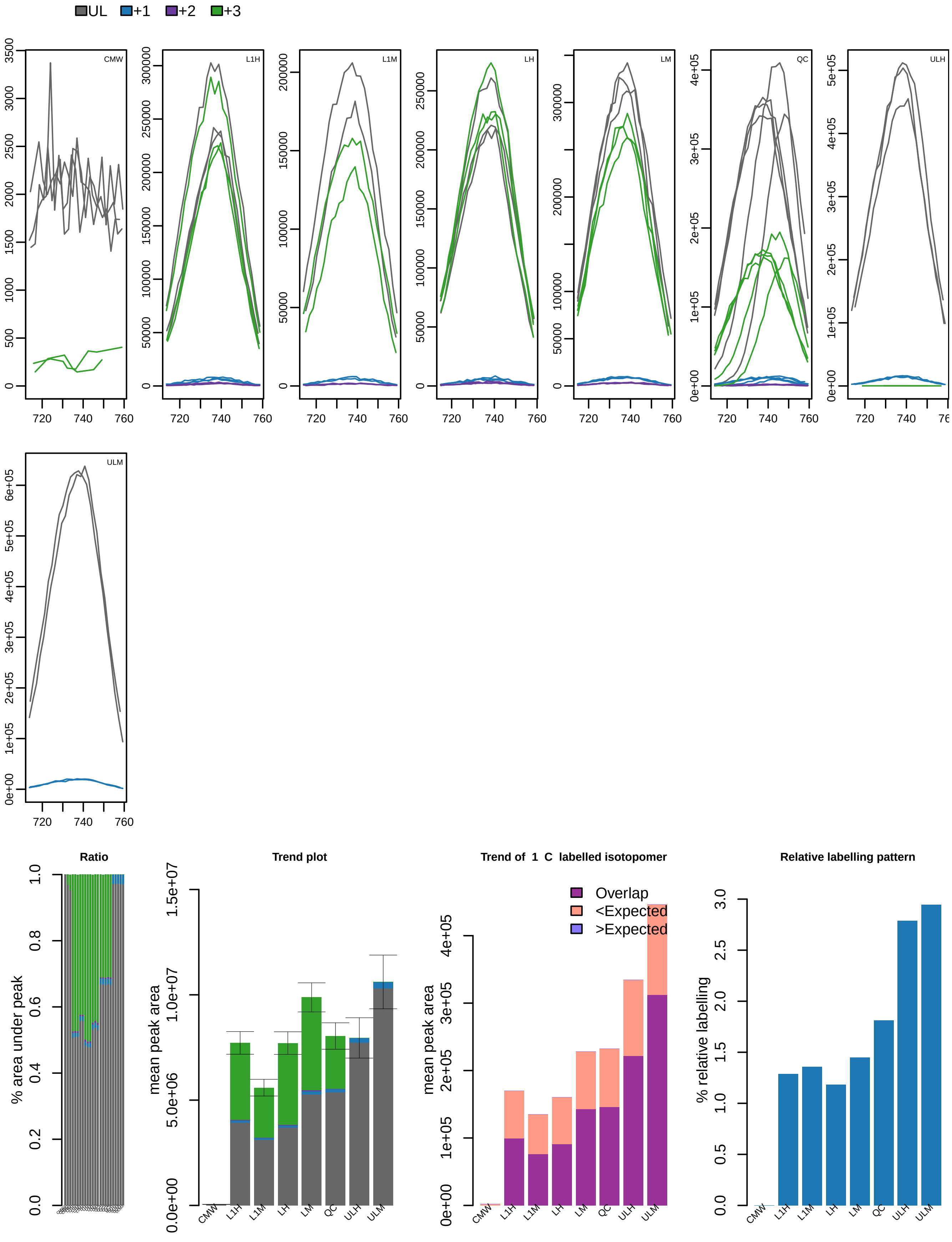

Maltotriose

G1

Formula: C18H32O16 Mass: 504.169 Std.RT: 1097.4861828 Ion: NE

UL +1 +2 +3 +4 +5 +6 +7 +8 +9 +10 +11 +12 +13

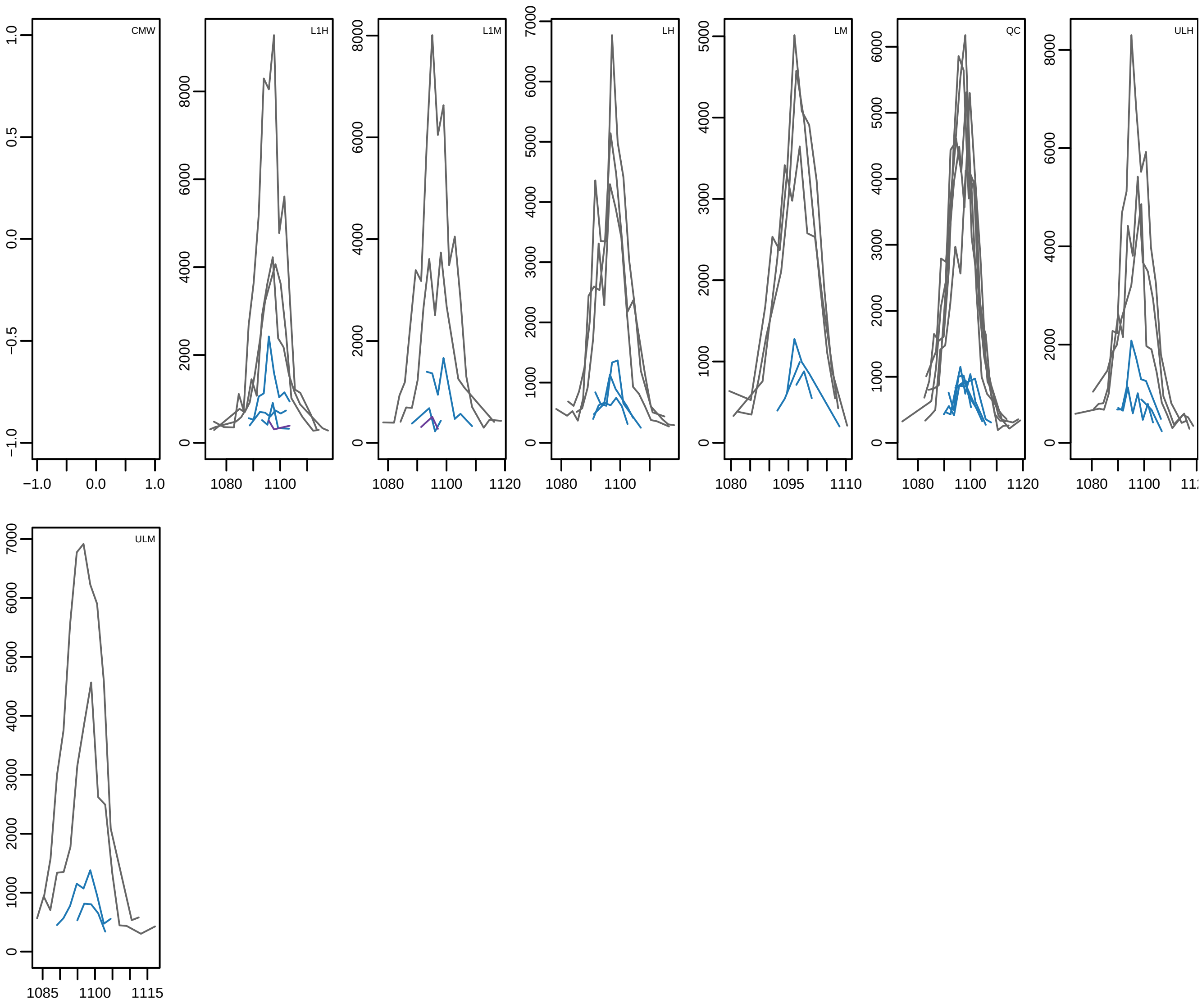

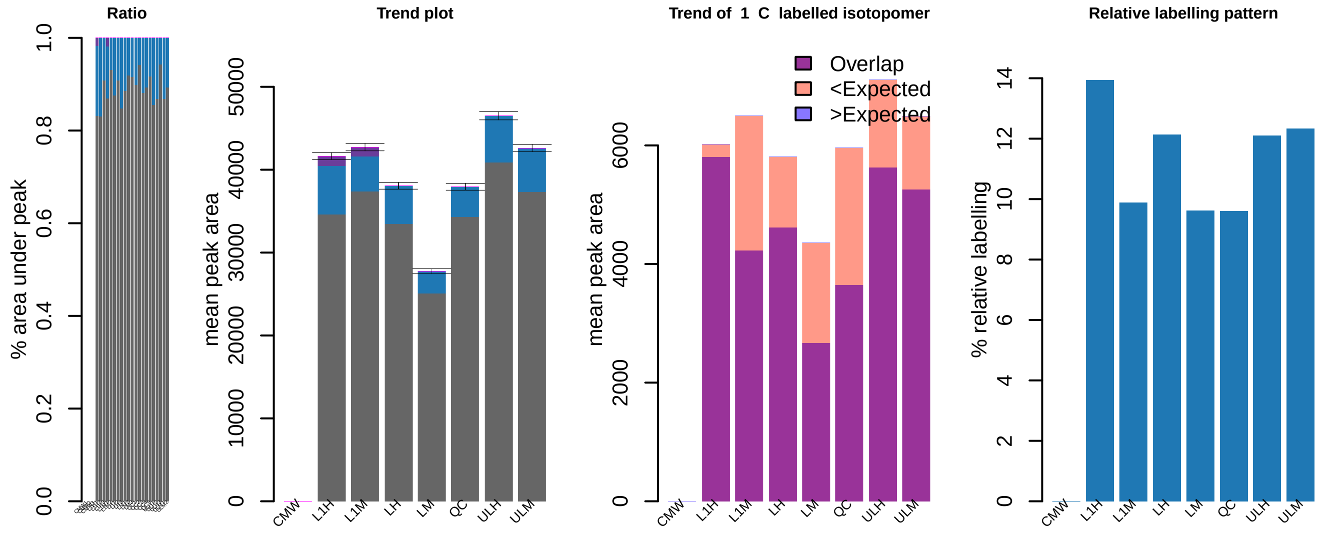

Pyruvate

Formula: C3H4O3 Mass: 88.016 Std.RT: 435.8467089 Ion: NEG

G1

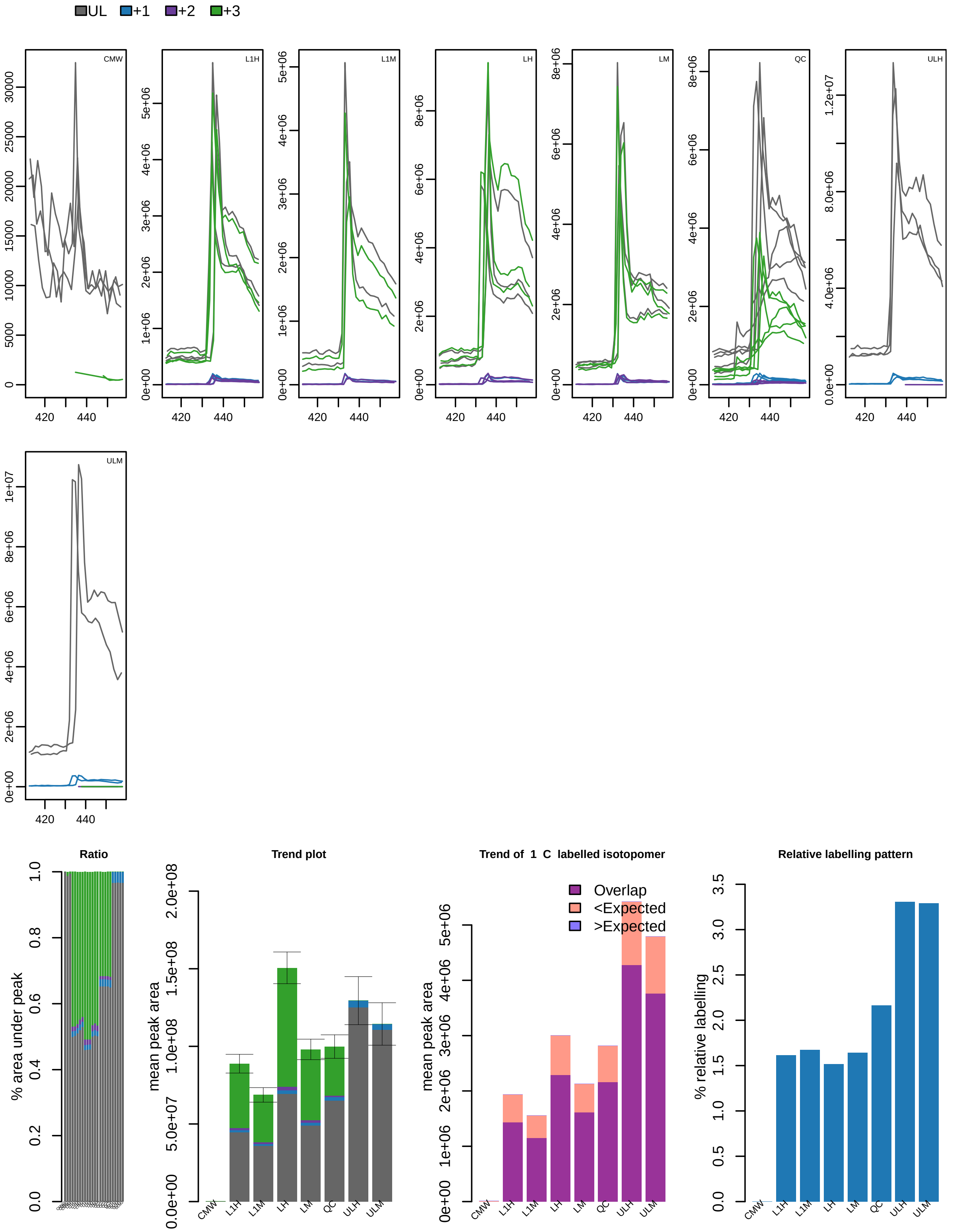

Pyruvate

Formula: C3H4O3 Mass: 88.016 Std.RT: 435.8467089 Ion: NEG

G2

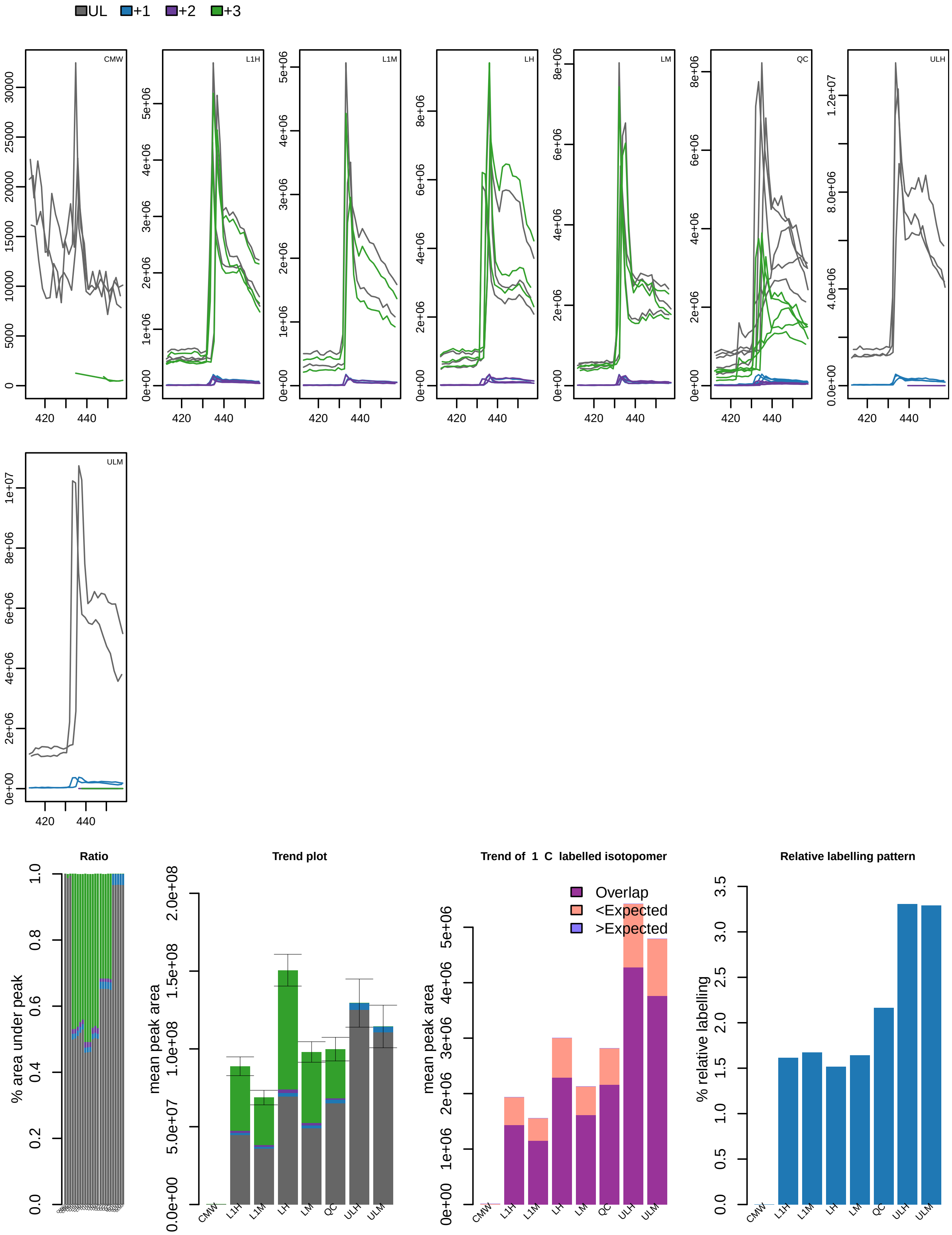

Phosphoenolpyruvate

G1

Formula: C3H5O6P Mass: 167.982 Std.RT: 1117.66476 Ion: NEG

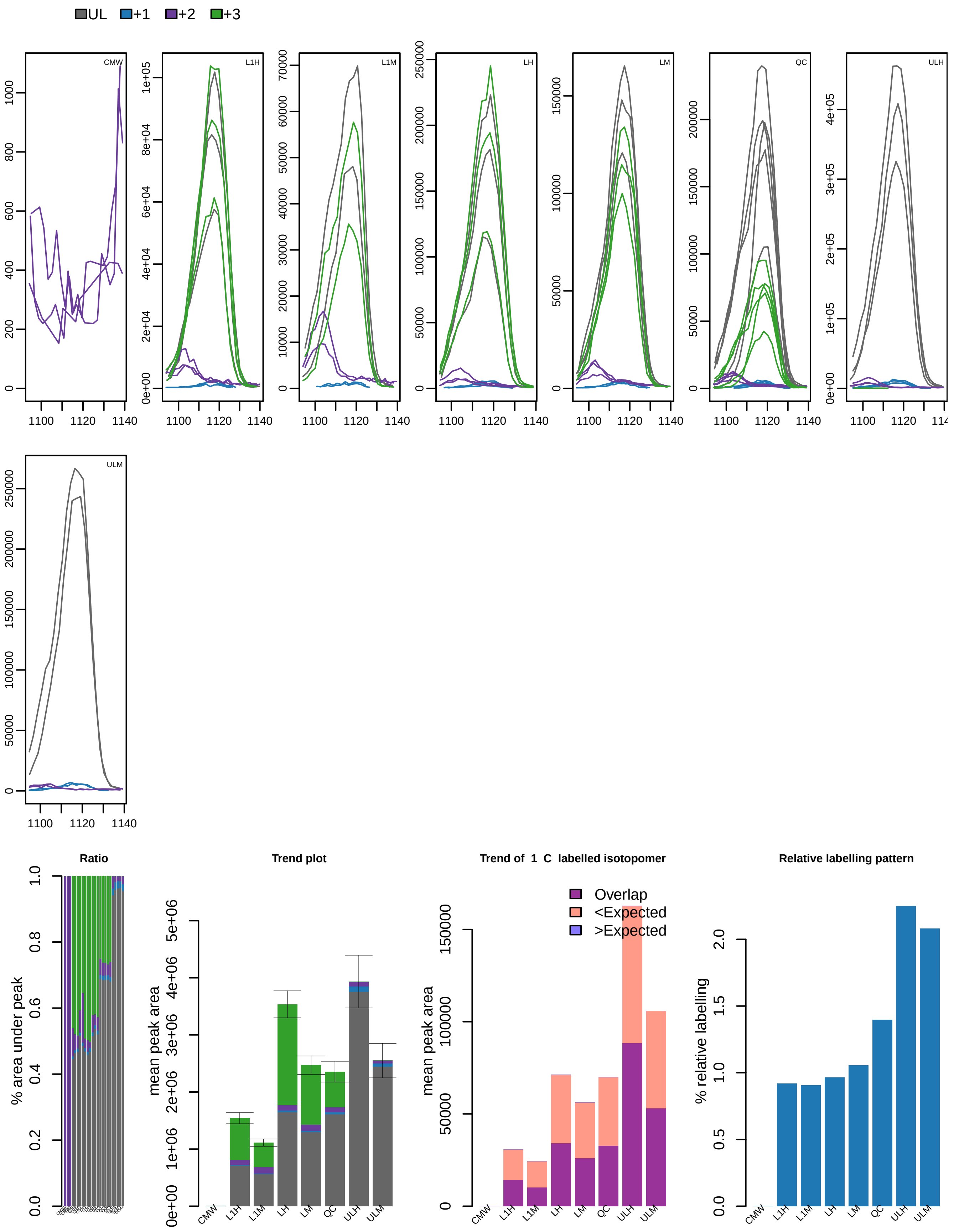

3−Phospho−D−glycerate

Formula: C3H7O7P Mass: 185.993 Std.RT: 1084.4990532 Ion: NEG

G1

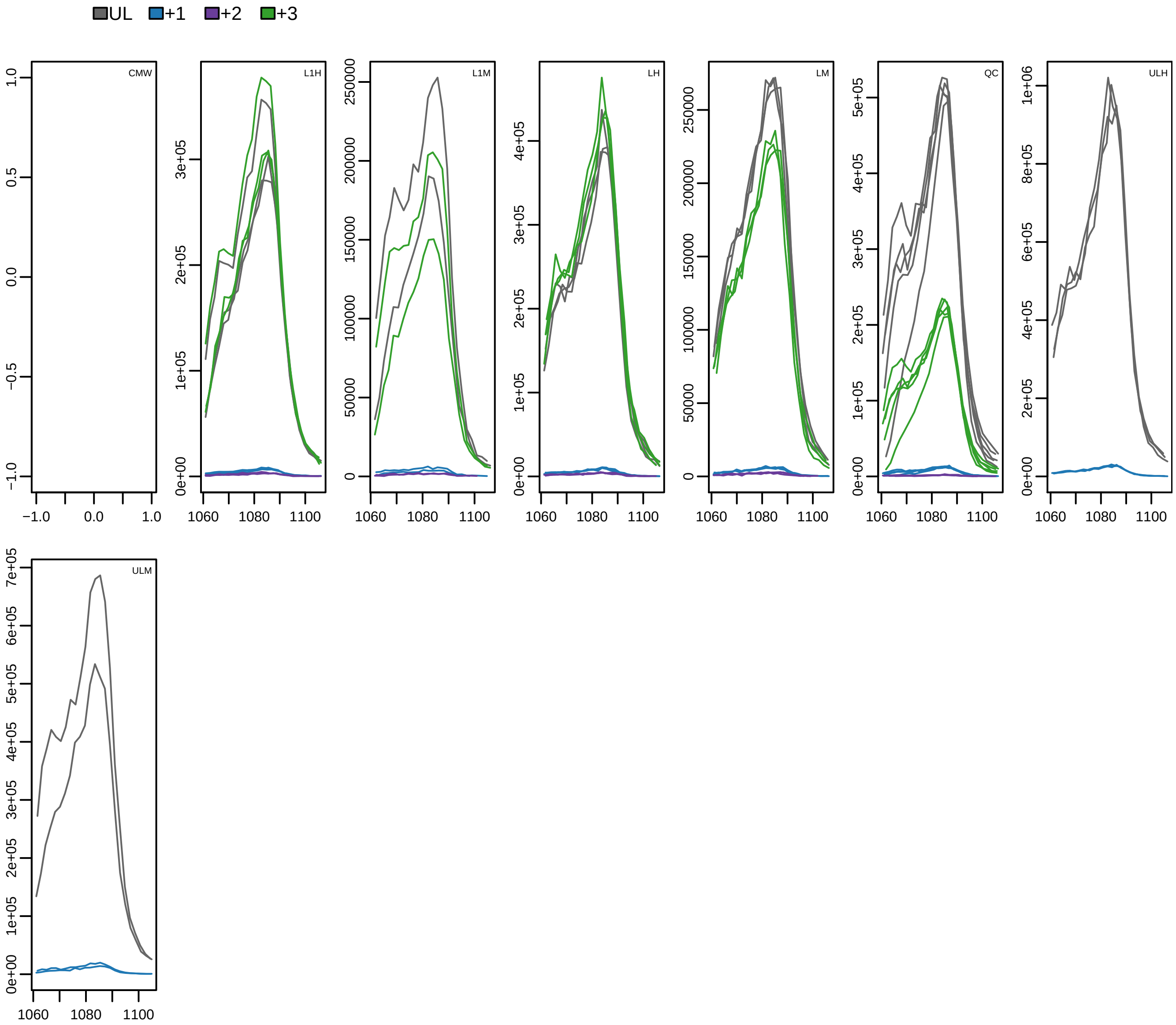

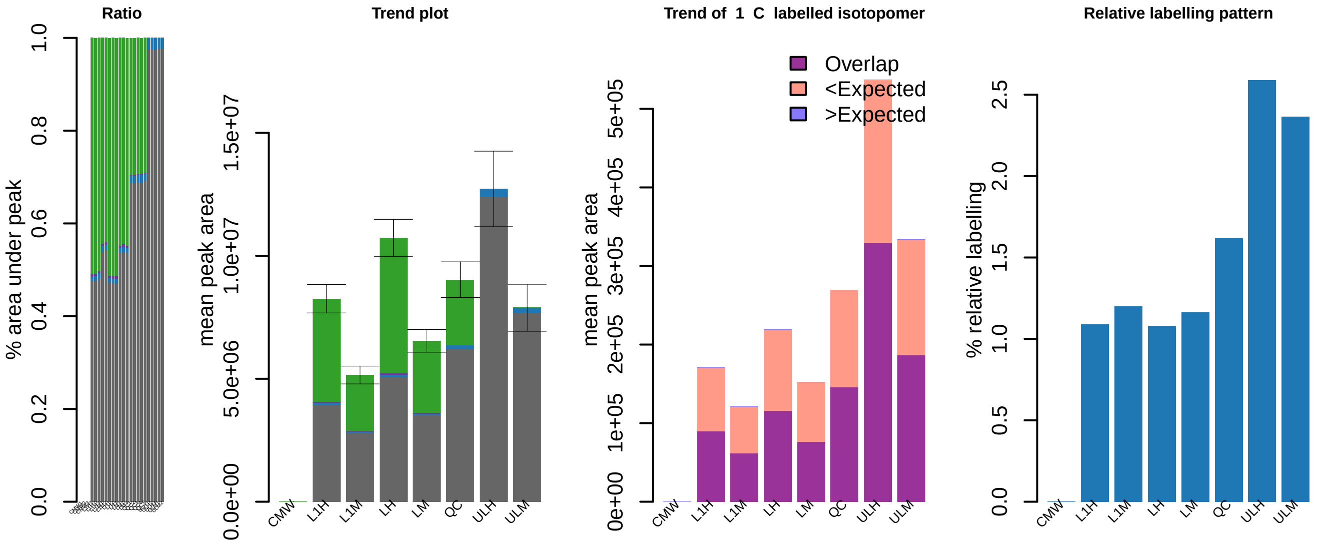

D−Glucose

Formula: $C6H12O6$ Mass: 180.063 Std.RT: 956.453247 Ion: NEG

G1

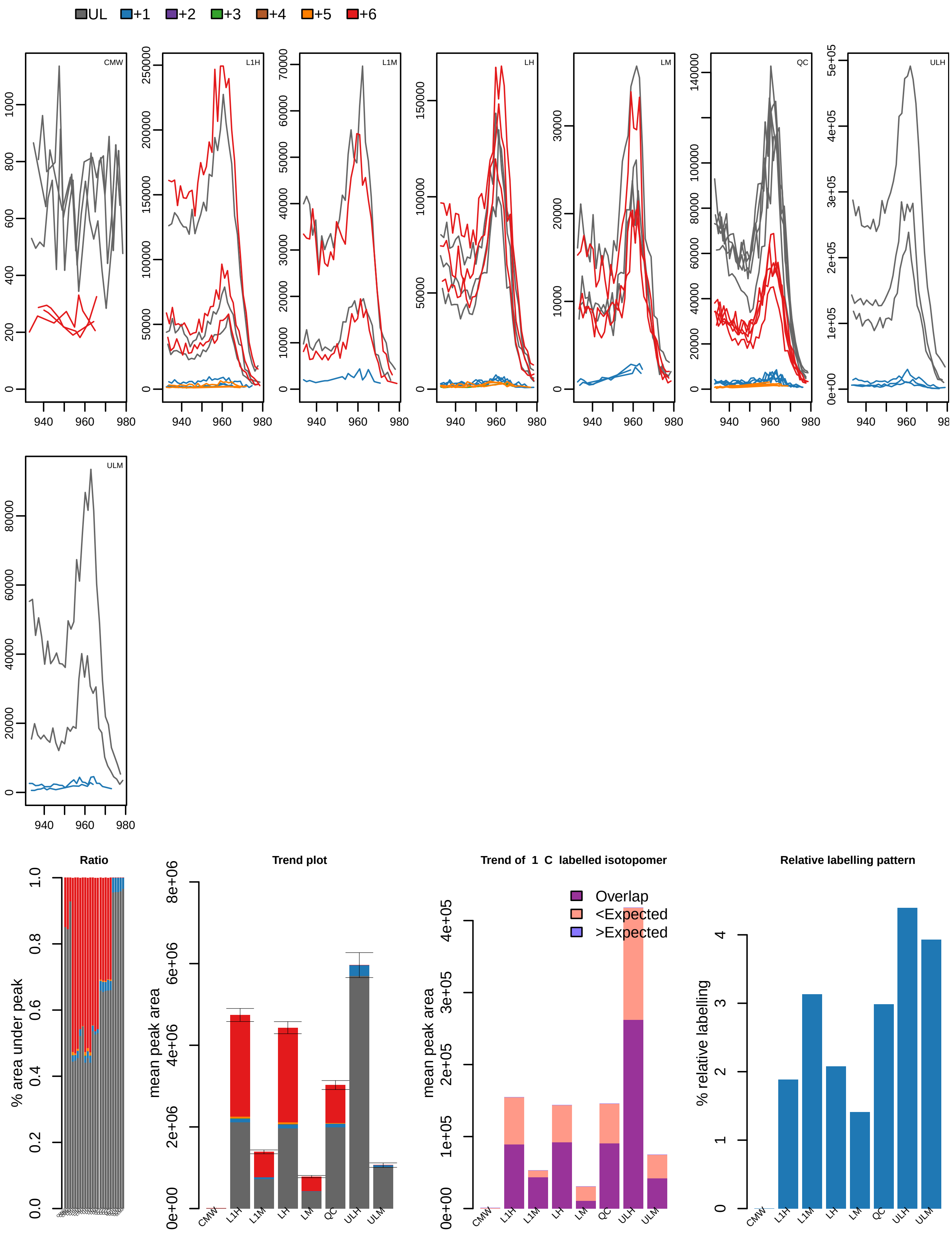

D−Glyceraldehyde 3−phosphate

Formula: C3H7O6P Mass: 169.998 Std.RT: 995.5217172 Ion: NEG

G1

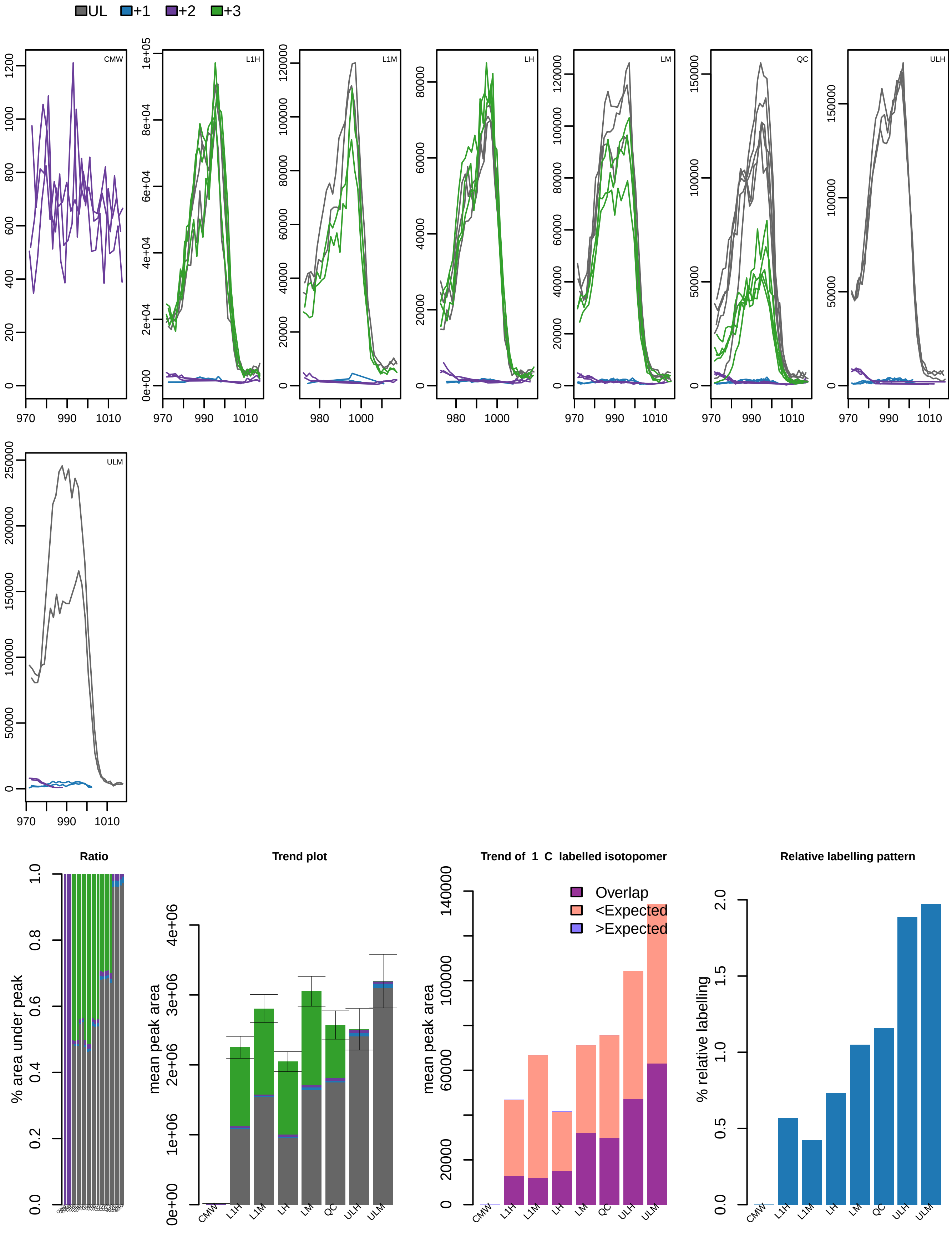

D−Glyceraldehyde 3−phosphate

Formula: C3H7O6P Mass: 169.998 Std.RT: 995.5217172 Ion: NEG

G2

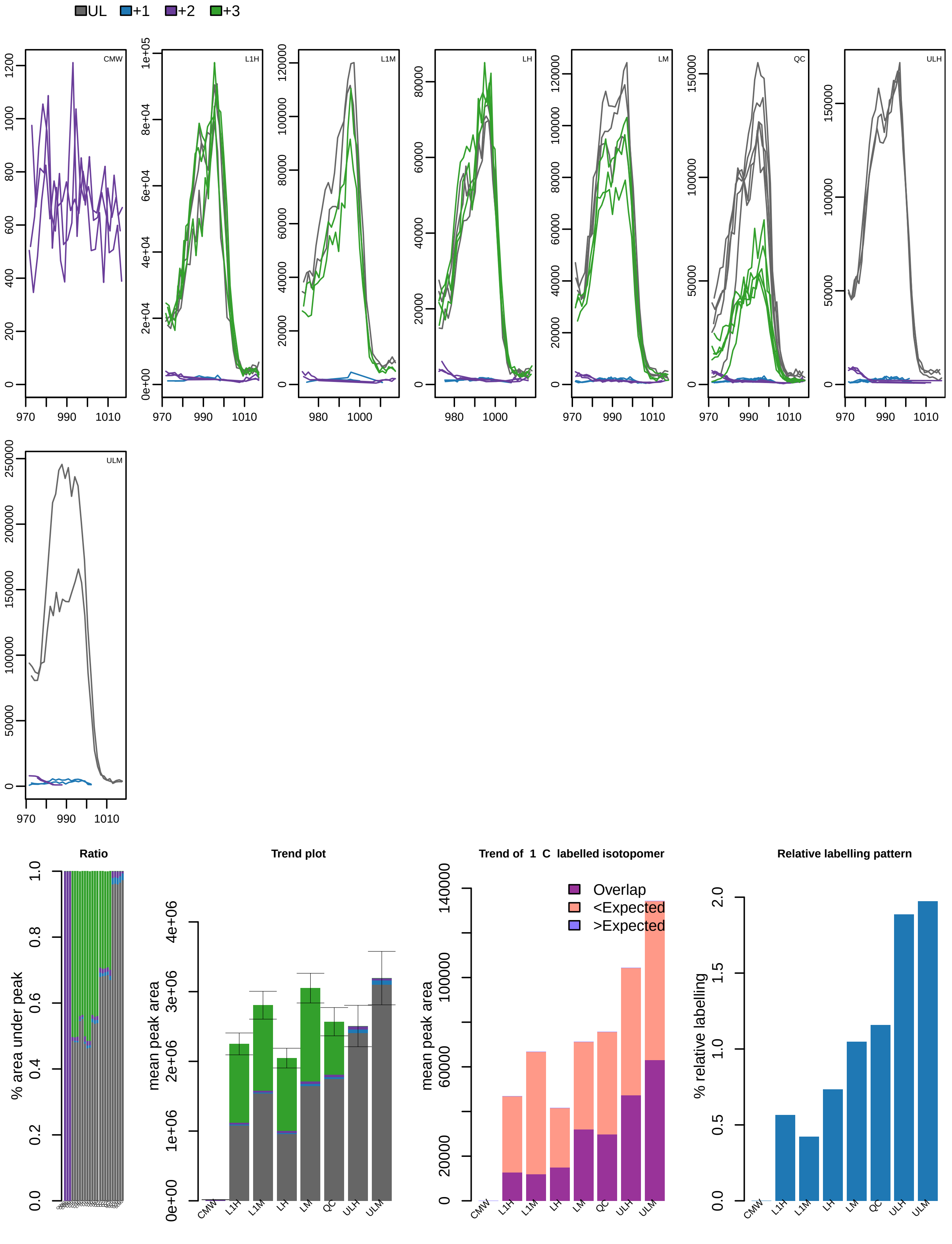

myo−Inositol

Formula: C6H12O6 Mass: 180.063 Std.RT: 1113.964167 Ion: NEG

G1

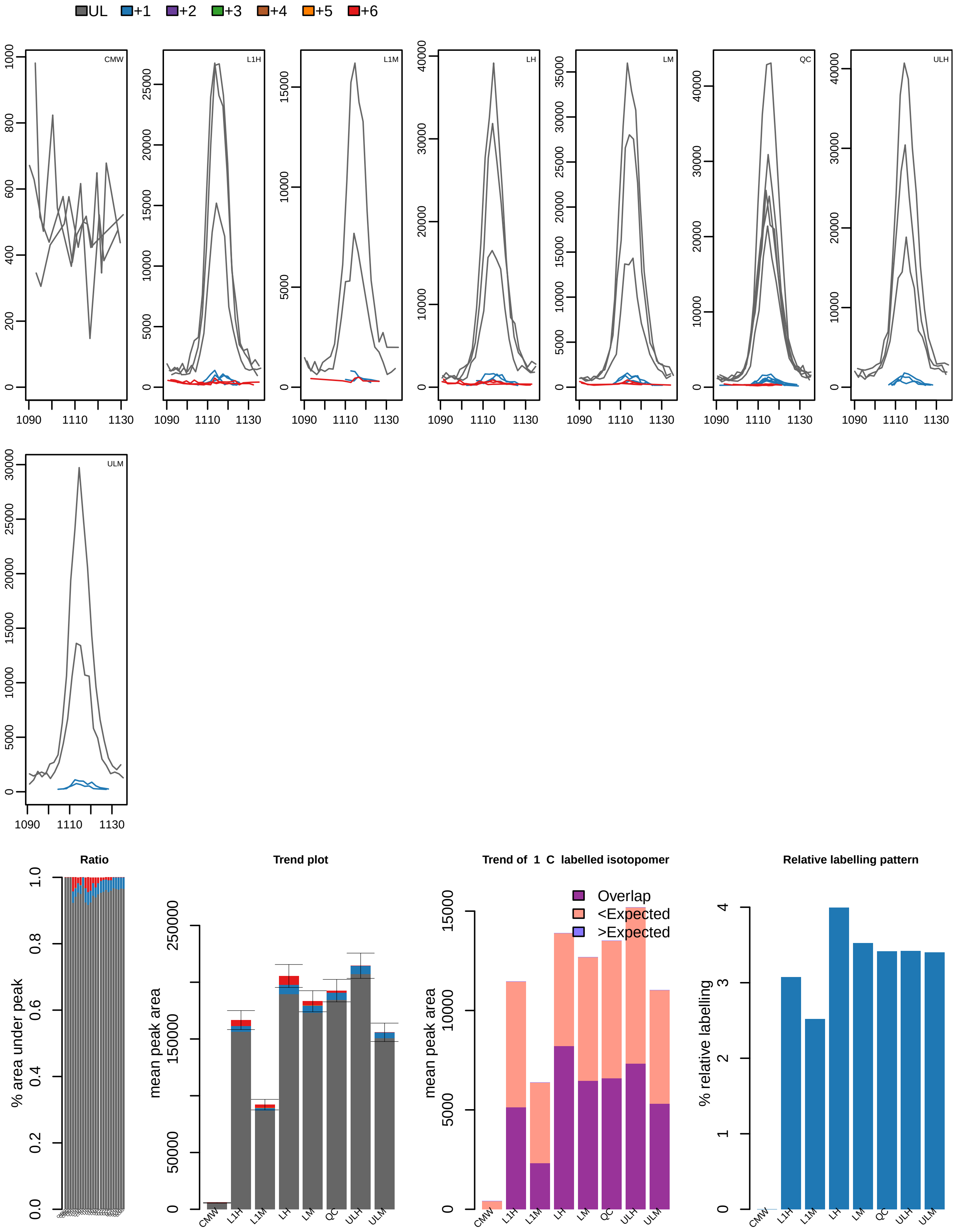

1−O−Methyl−myo−inositol

Formula: C7H14O6 Mass: 194.079 Std.RT: 853.5622506 Ion: NEG

G1

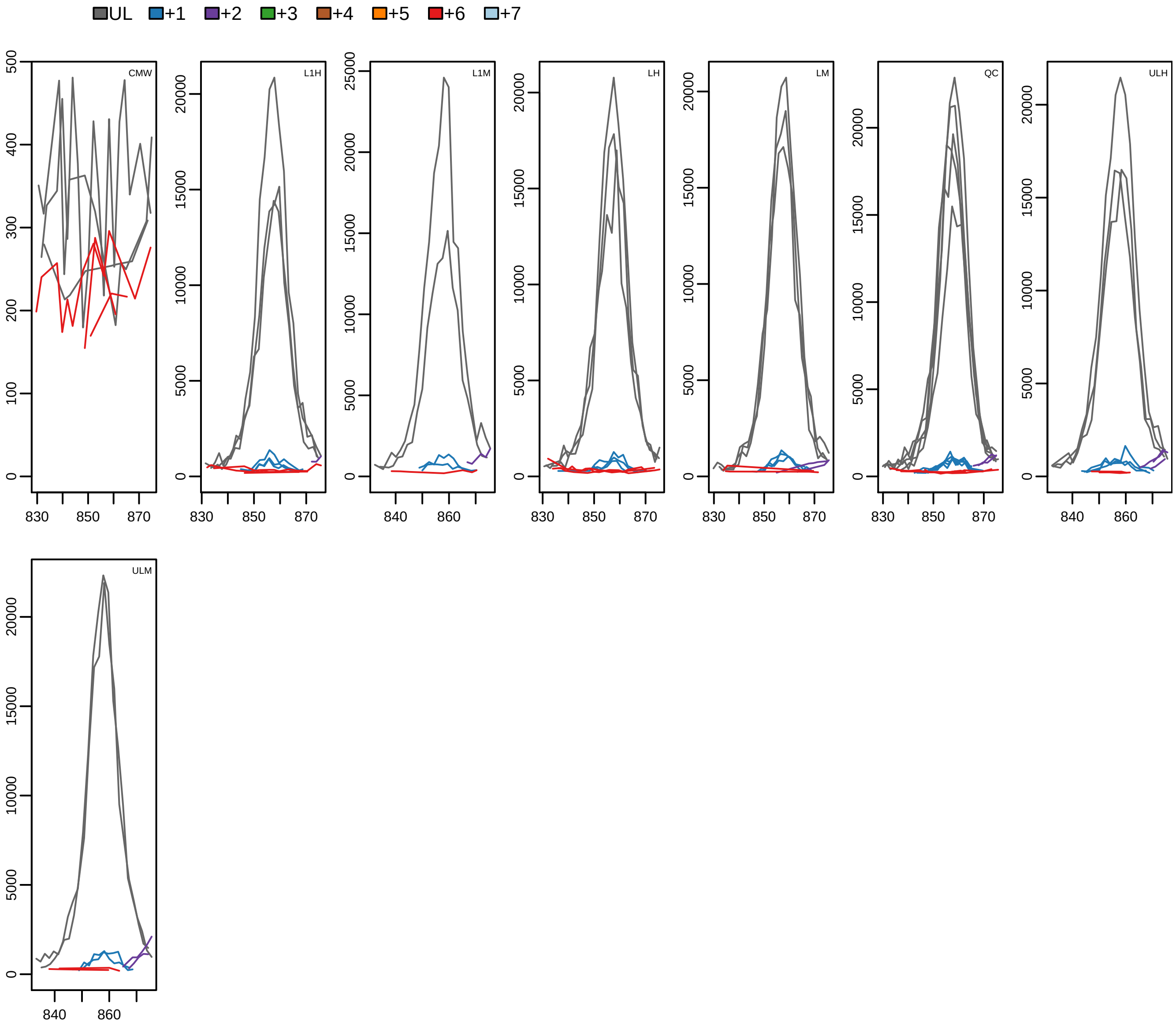

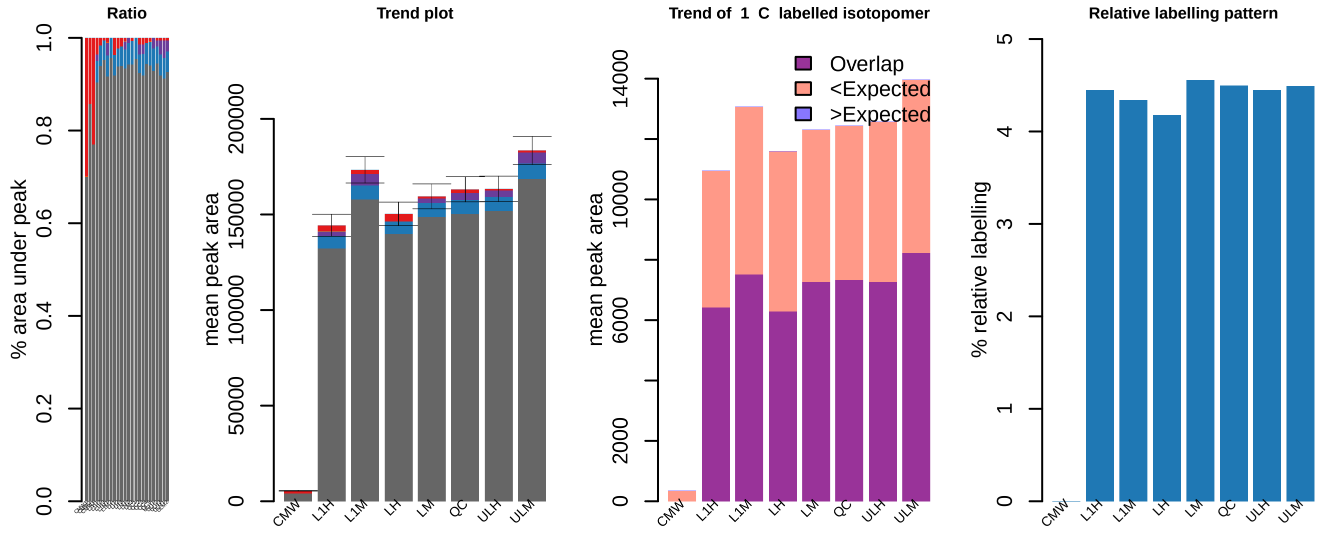

D−Ribulose

Formula: $C_5H_{10}O_5$ Mass: 150.053 Std.RT: 693.3349992 Ion: NEG

G1

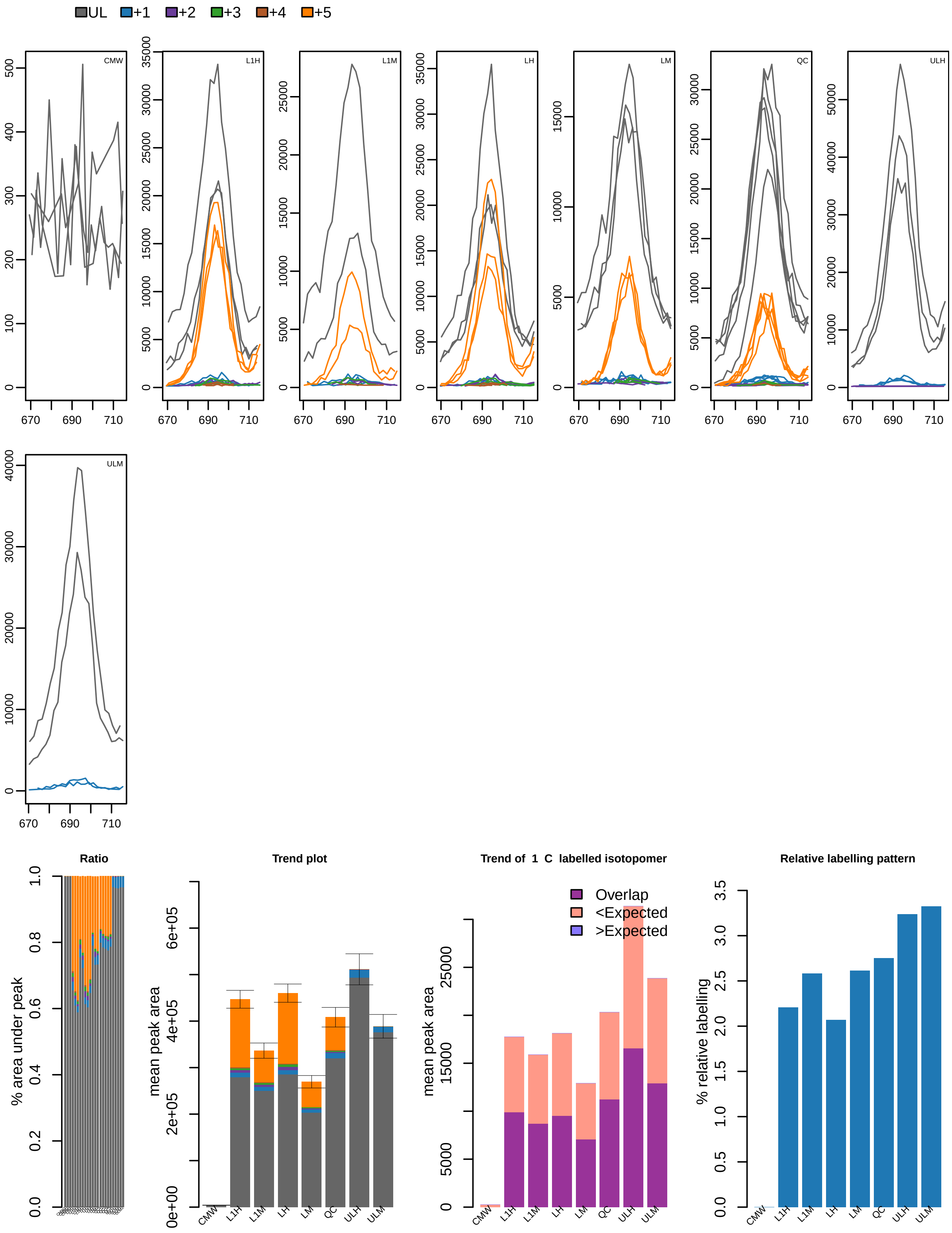

D−Ribulose

Formula: $C_5H_{10}O_5$ Mass: 150.053 Std.RT: 693.3349992 Ion: NEG

G2

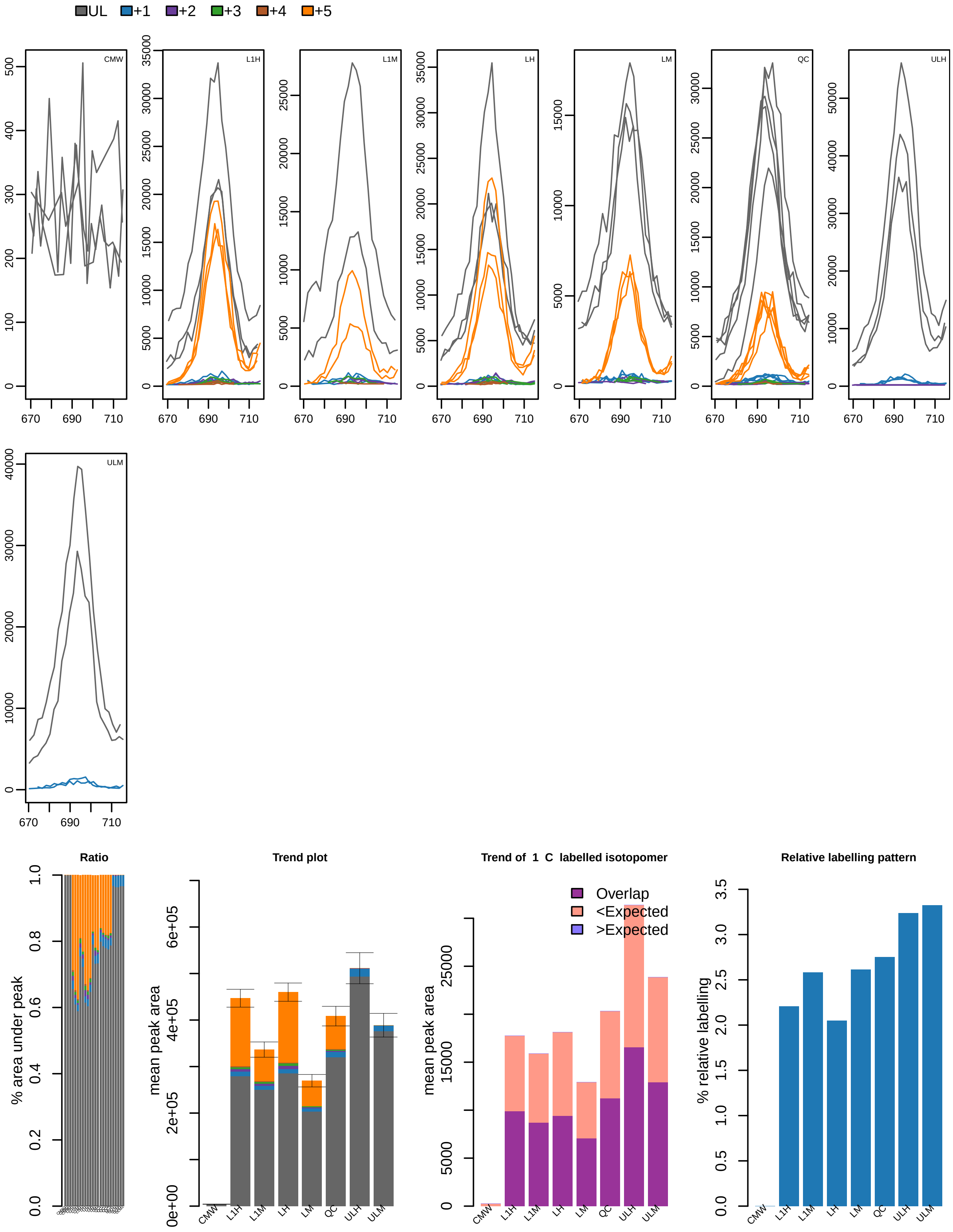

Xylitol

Formula: C5H12O5 Mass: 152.068 Std.RT: 832.1345412 Ion: NEG

G1

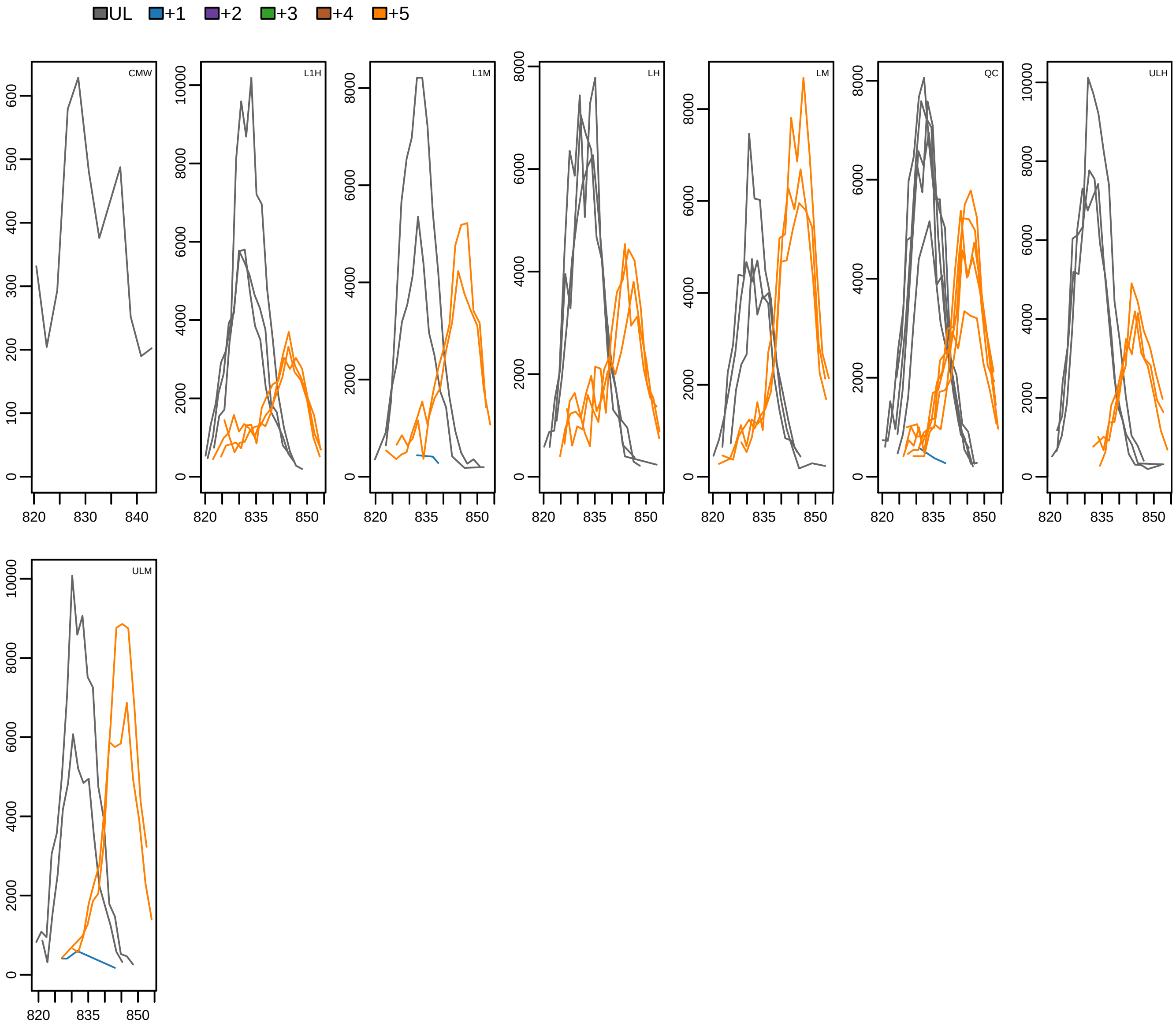

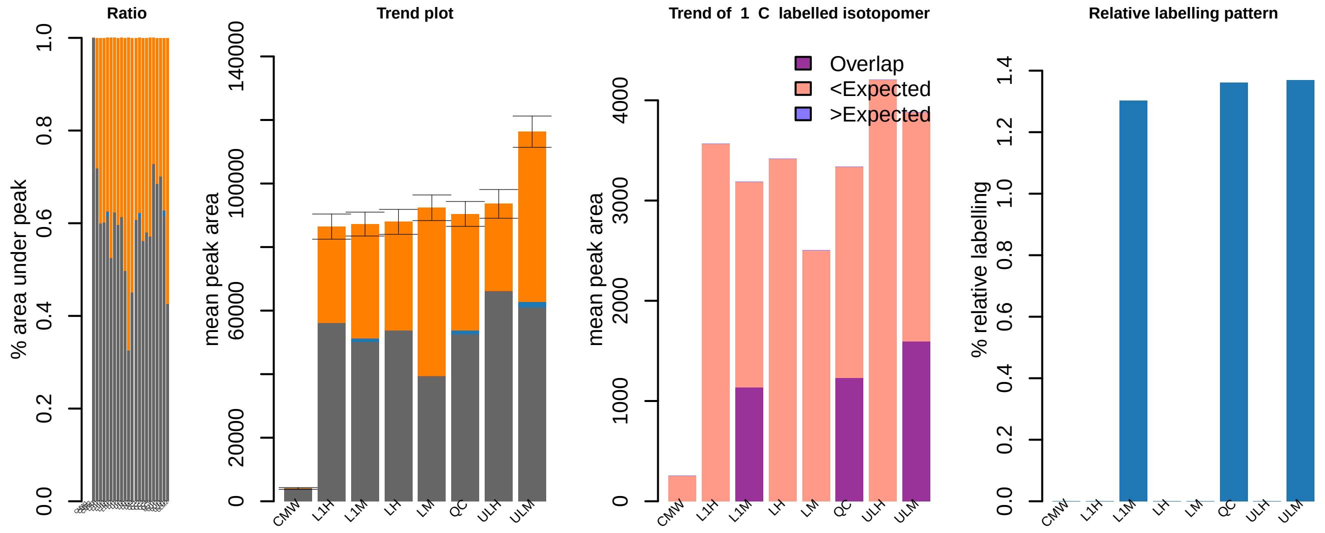

D−Glucuronate

Formula: C6H10O7 Mass: 194.043 Std.RT: 921.3669996 Ion: NEG

G1

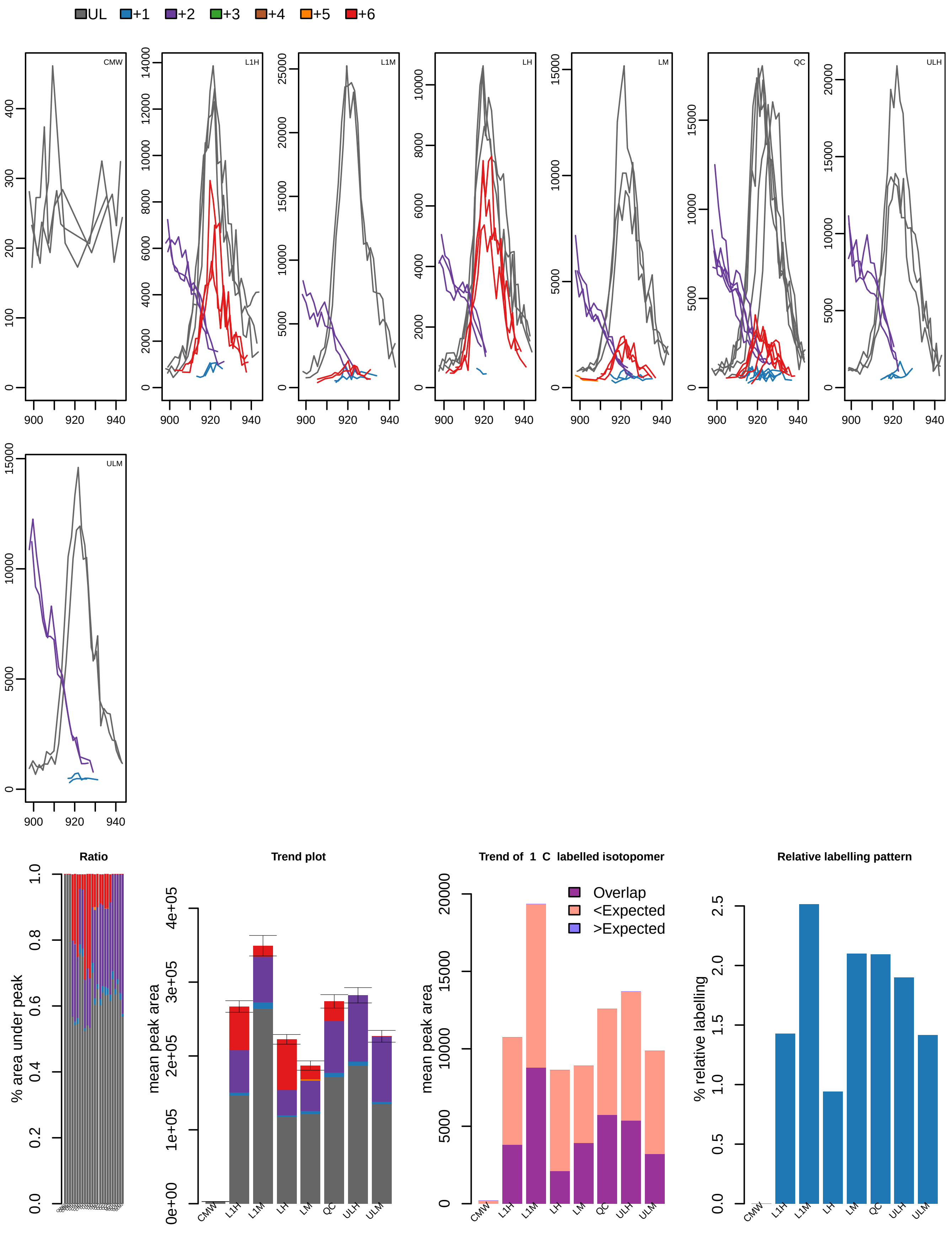

UDP−glucose

Formula: C15H24N2O17P2 Mass: 566.055 Std.RT: 1055.8433262 Ior

G1

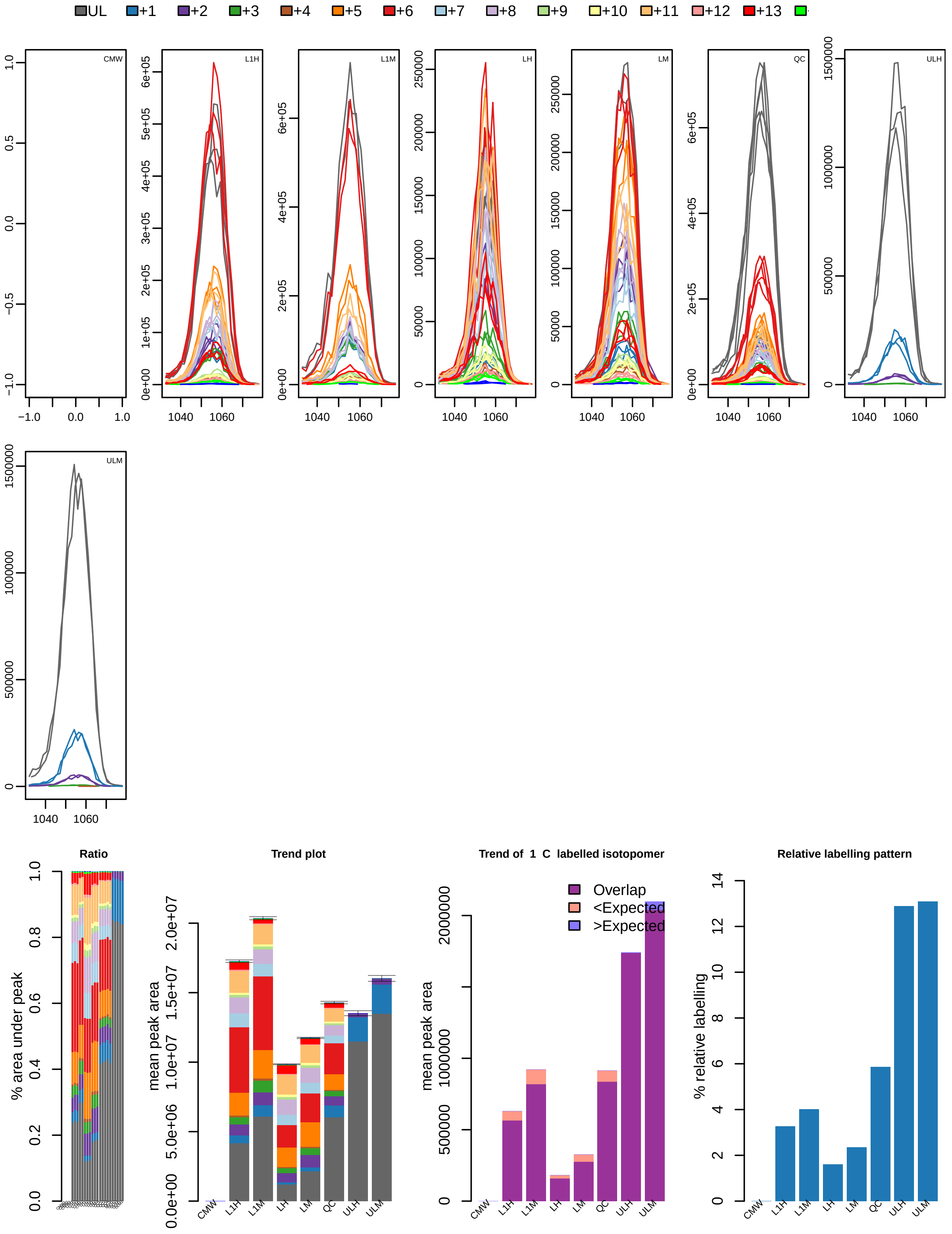

Deoxyribose

Formula: C5H10O4 Mass: 134.058 Std.RT: 476.93912508 Ion: NEG

G1

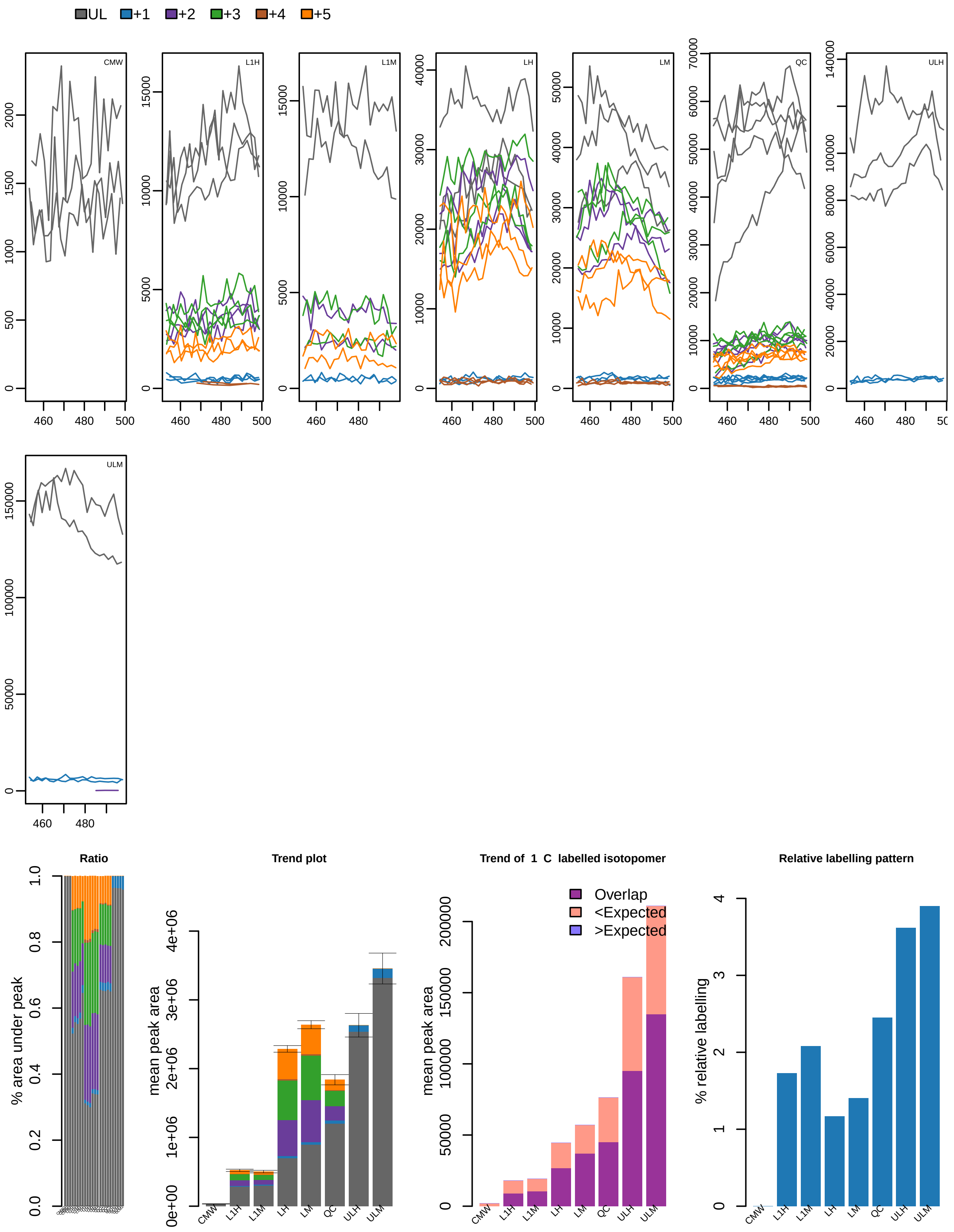

6−Phospho−D−gluconate

Formula: C6H13O10P Mass: 276.025 Std.RT: 1130.2852488 Ion: NE

G1

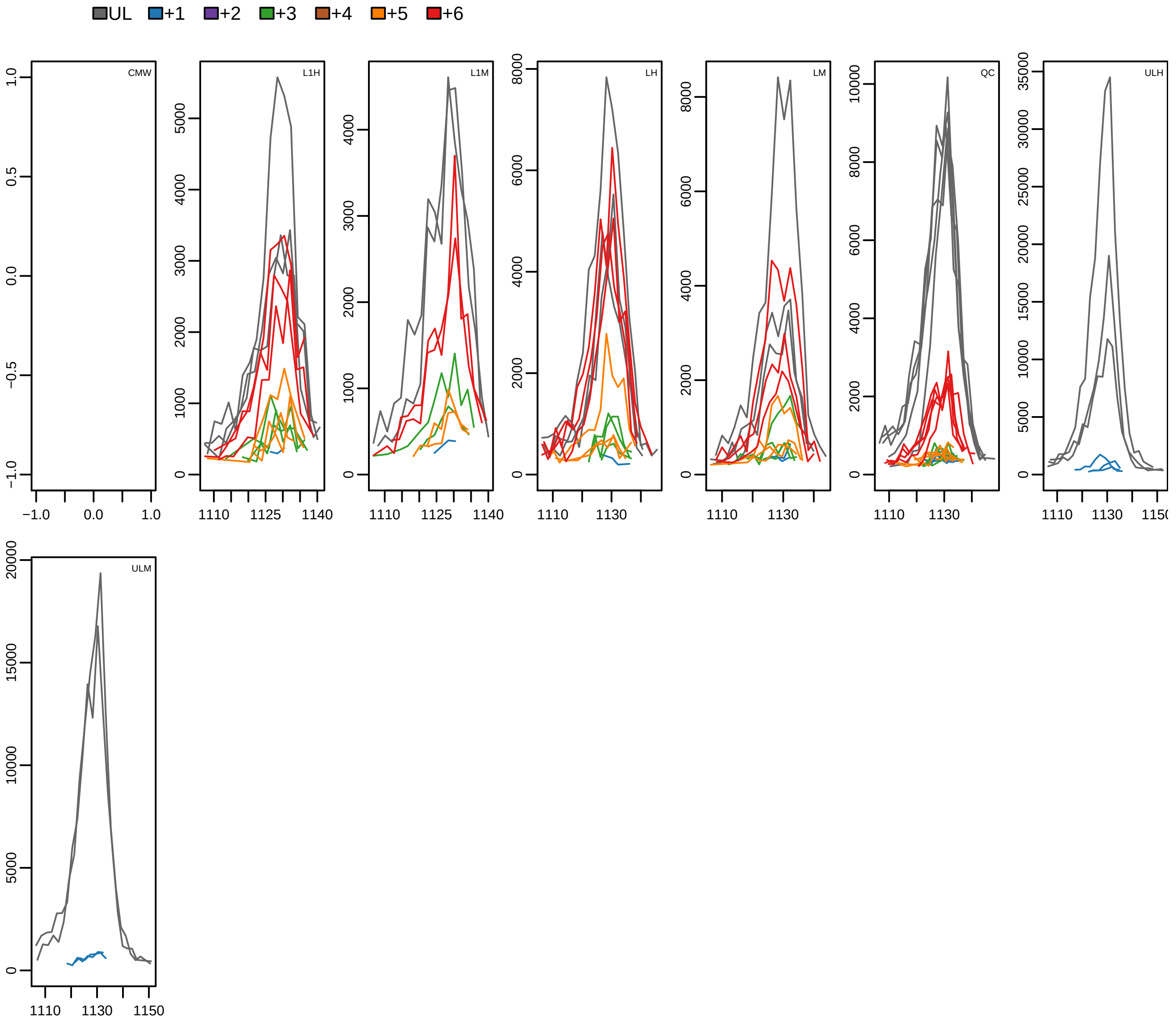

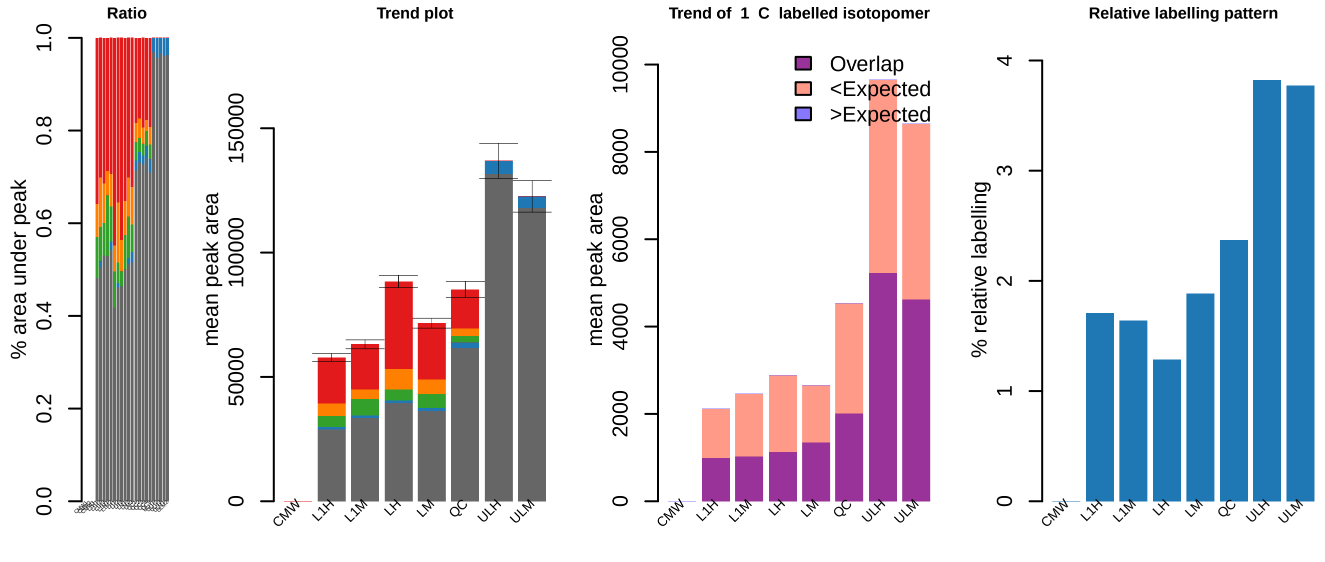

D−Gluconic acid

Formula: $C_6H_{12}O_7$ Mass: 196.058 Std.RT: 893.998497 Ion: NEG

G1

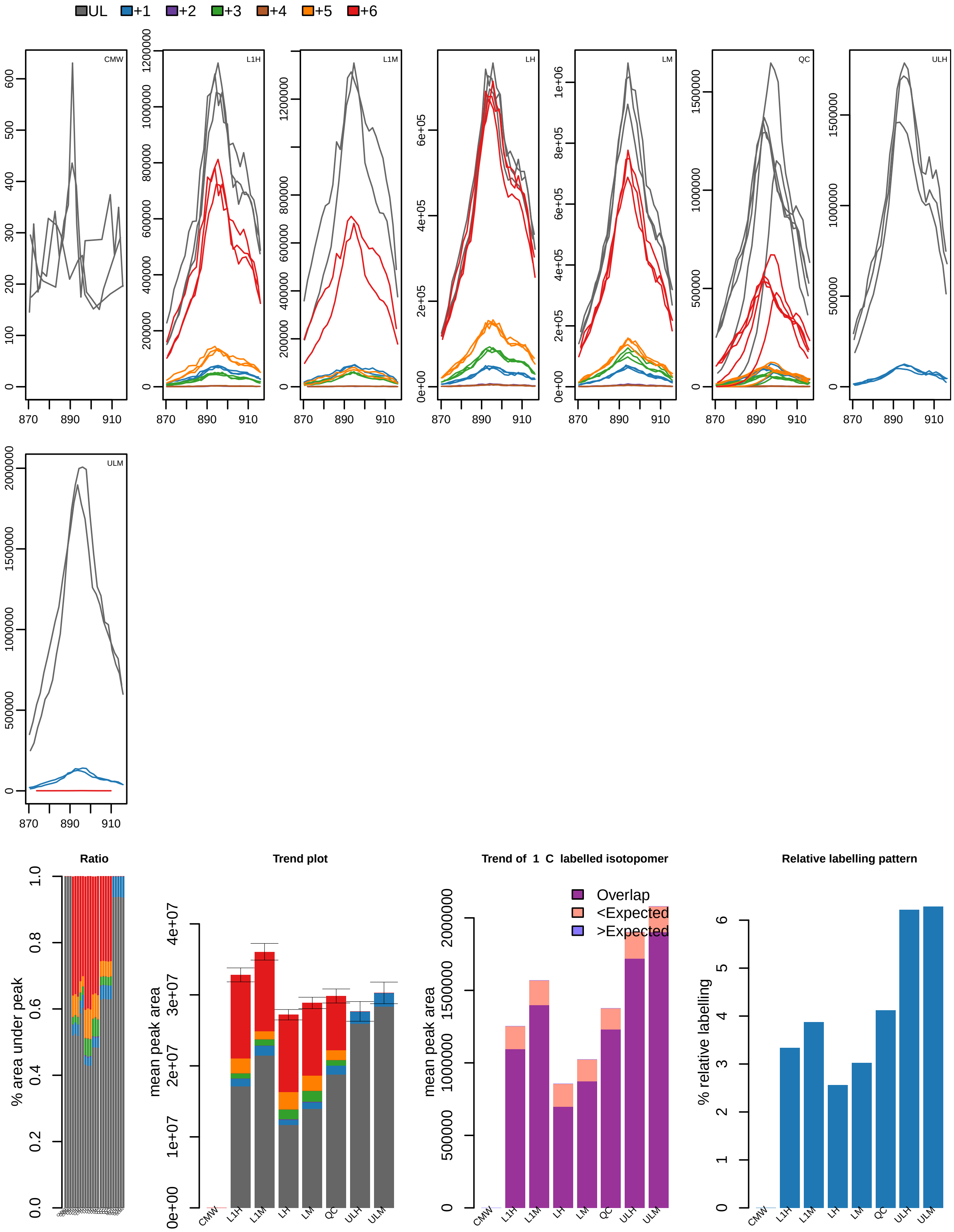

D−Ribose

Formula: $C5H10O5$ Mass: 150.053 Std.RT: 717.3402456 Ion: NEG

G1

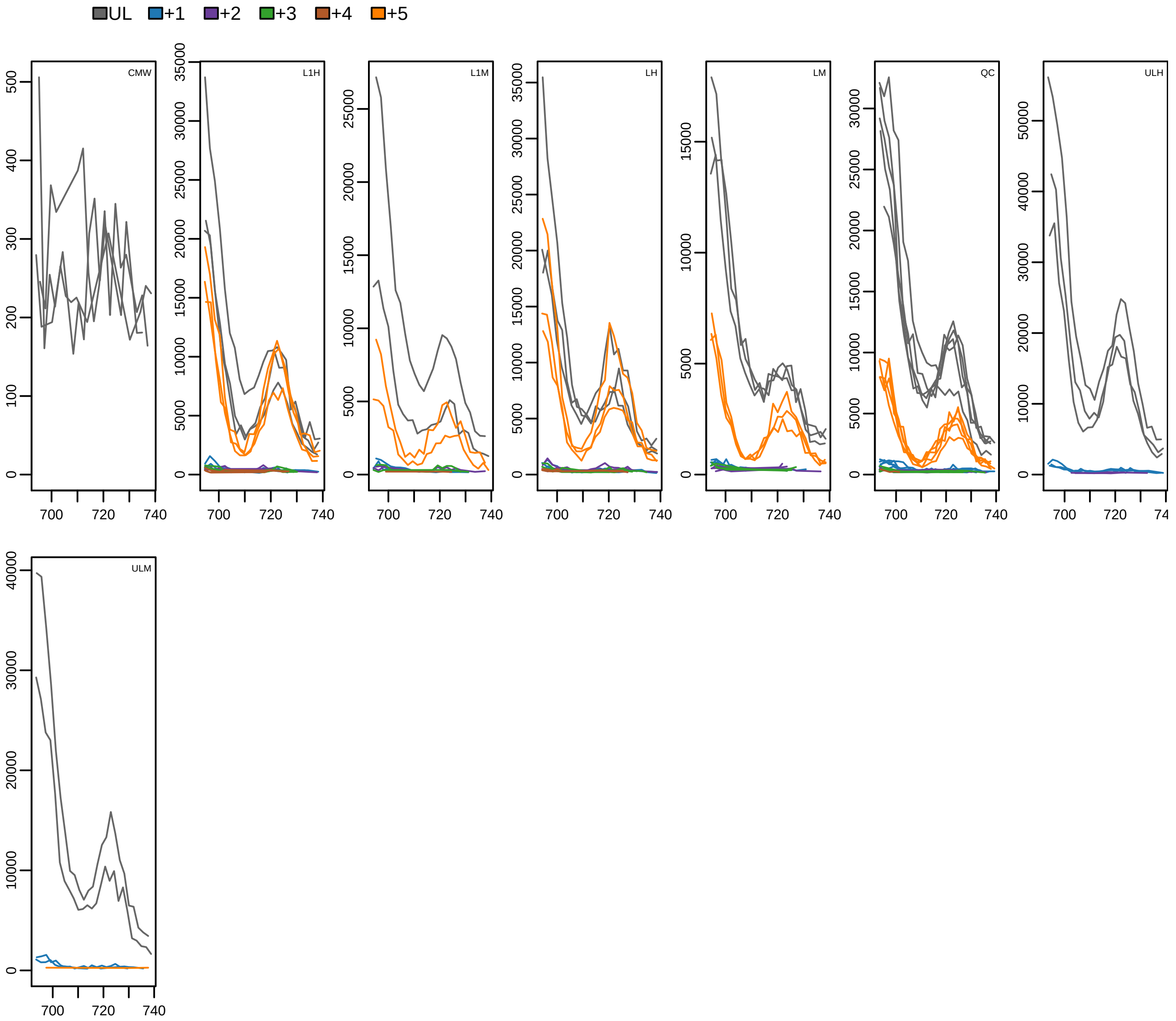

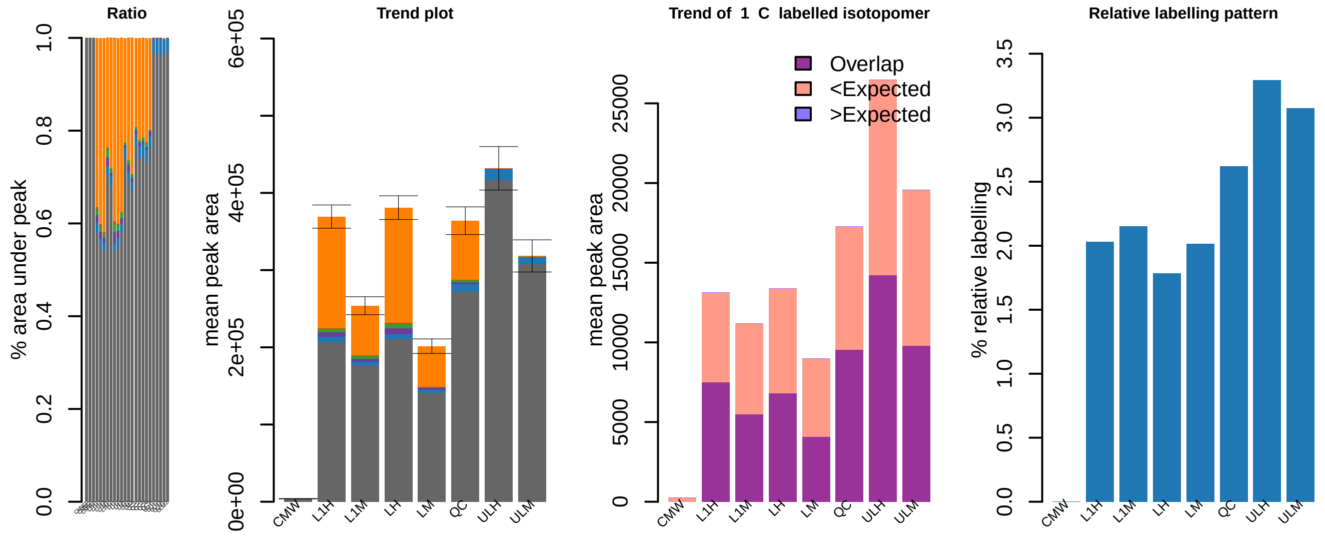

D−Ribose

Formula: C5H10O5 Mass: 150.053 Std.RT: 717.3402456 Ion: NEG

G2

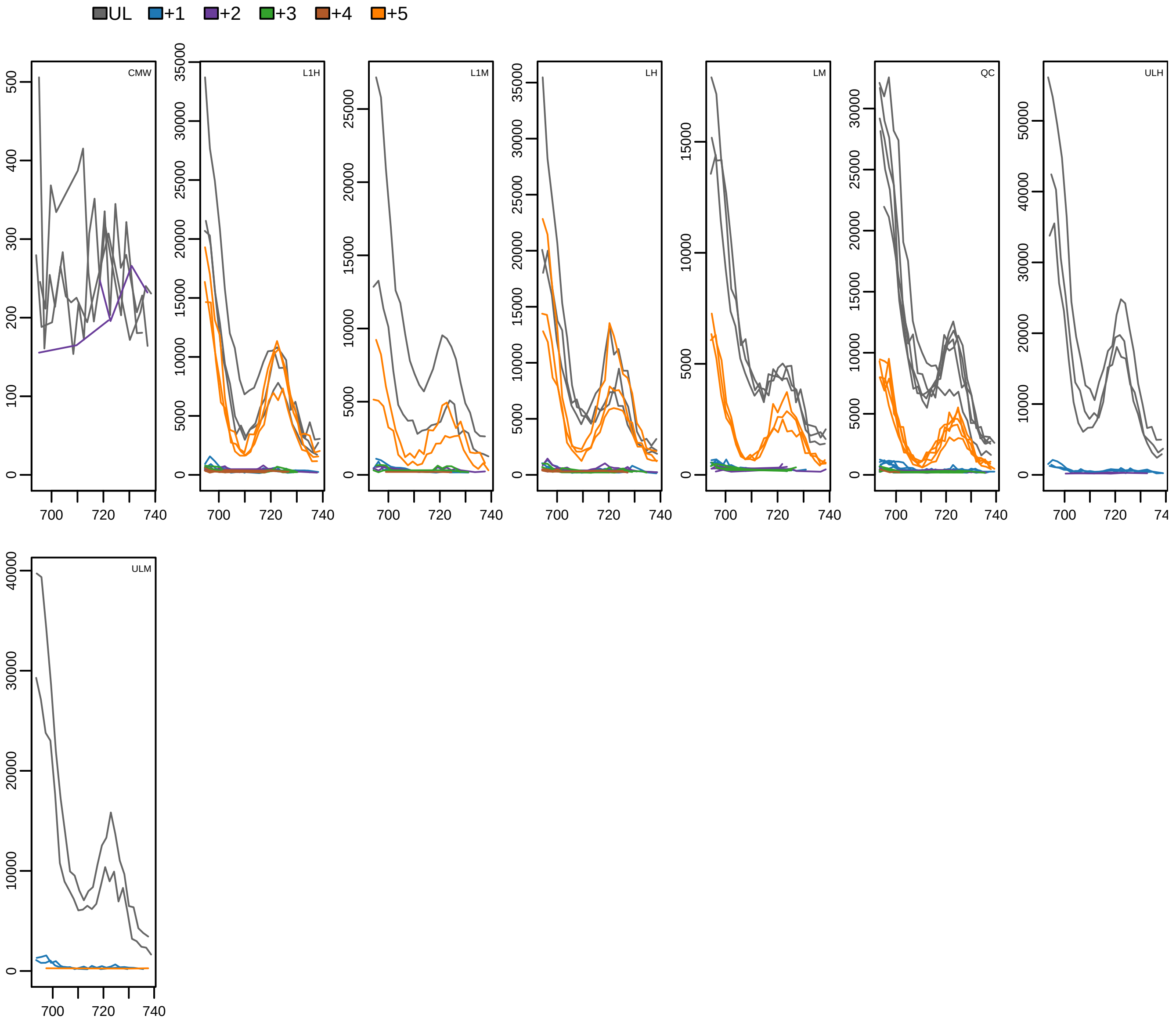

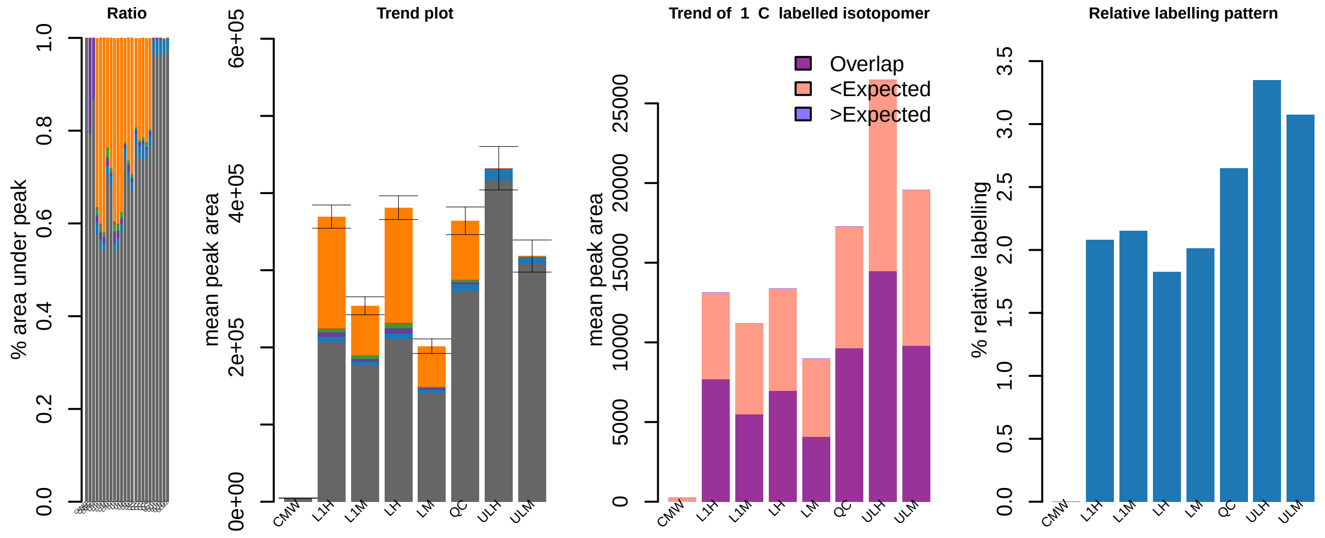

2−Deoxy−D−ribose 5−phosphate

Formula: C5H11O7P Mass: 214.024 Std.RT: 948.676569 Ion: NEG

G1

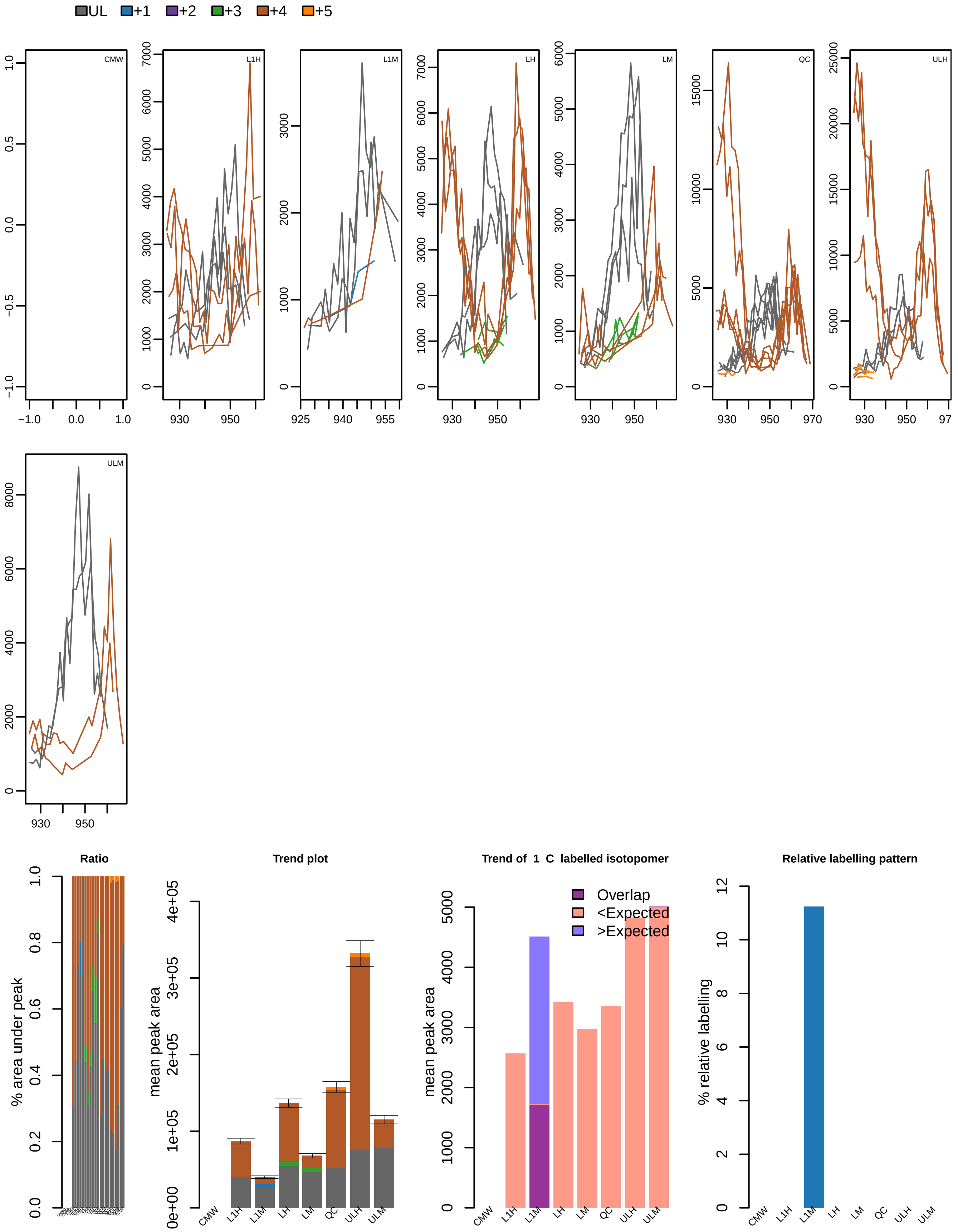

D−Sedoheptulose 7−phosphate

Formula: C7H15O10P Mass: 290.04 Std.RT: 954.5970078 Ion: NEG

G1

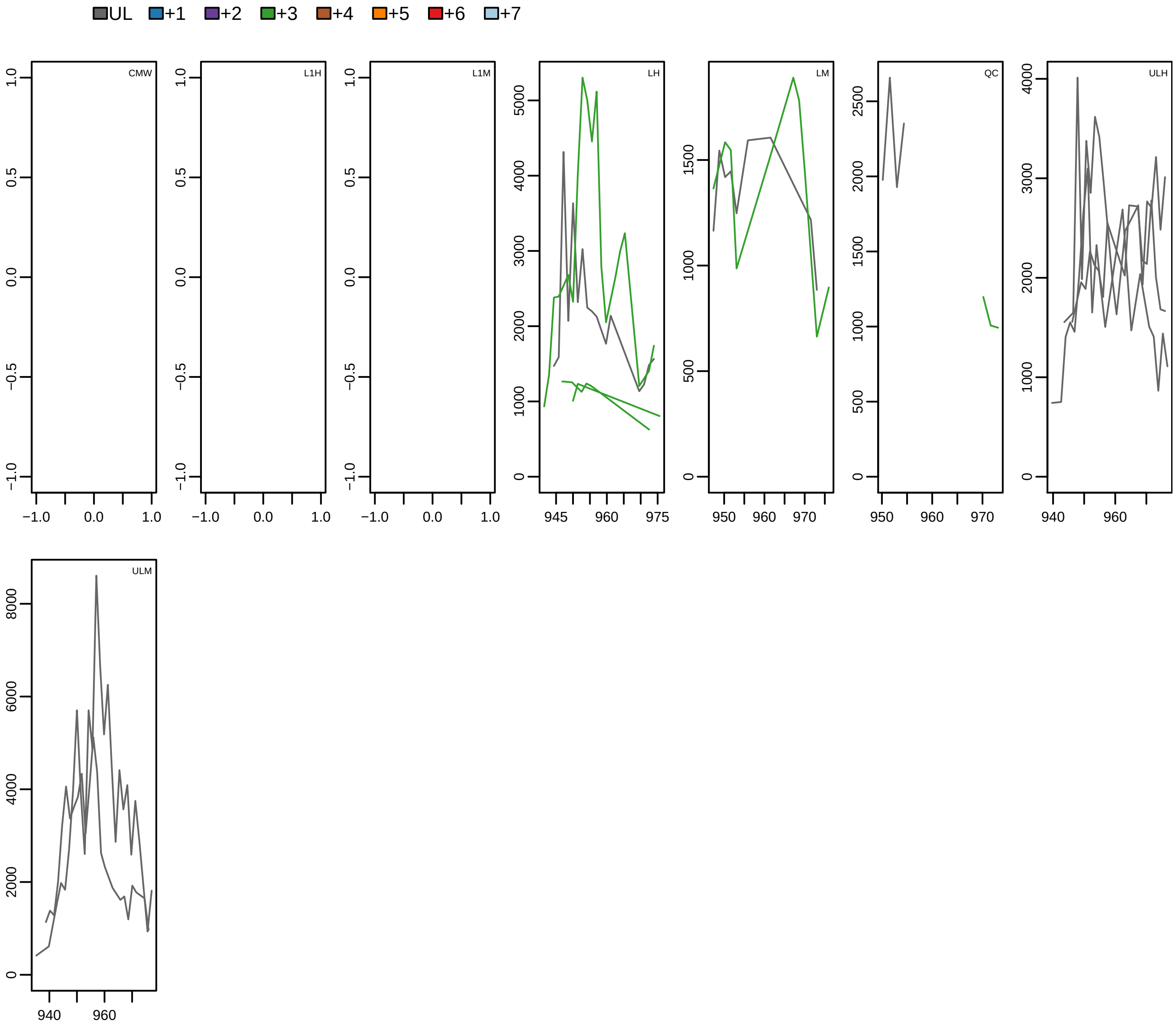

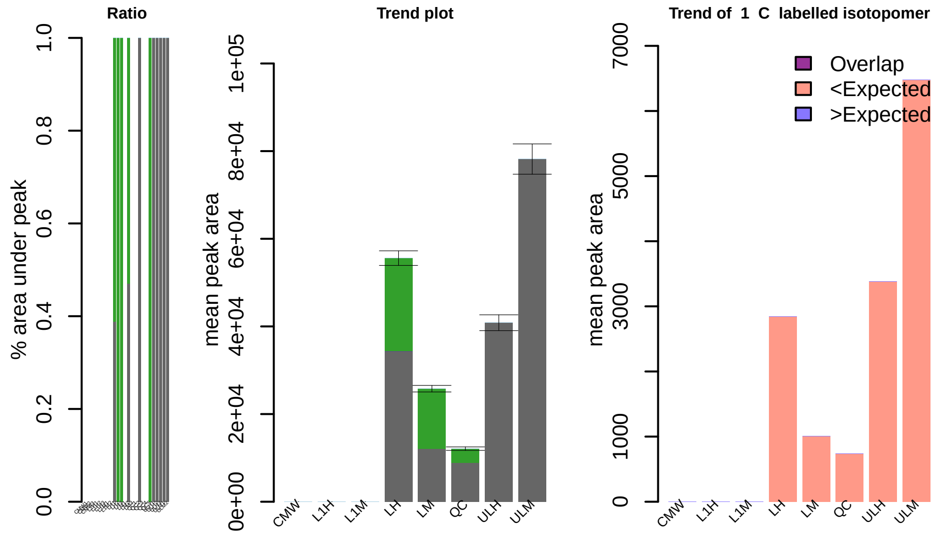

2−Dehydro−3−deoxy−D−gluconate

Formula: C6H10O6 Mass: 178.048 Std.RT: 660.3712488 Ion: NEG

G1

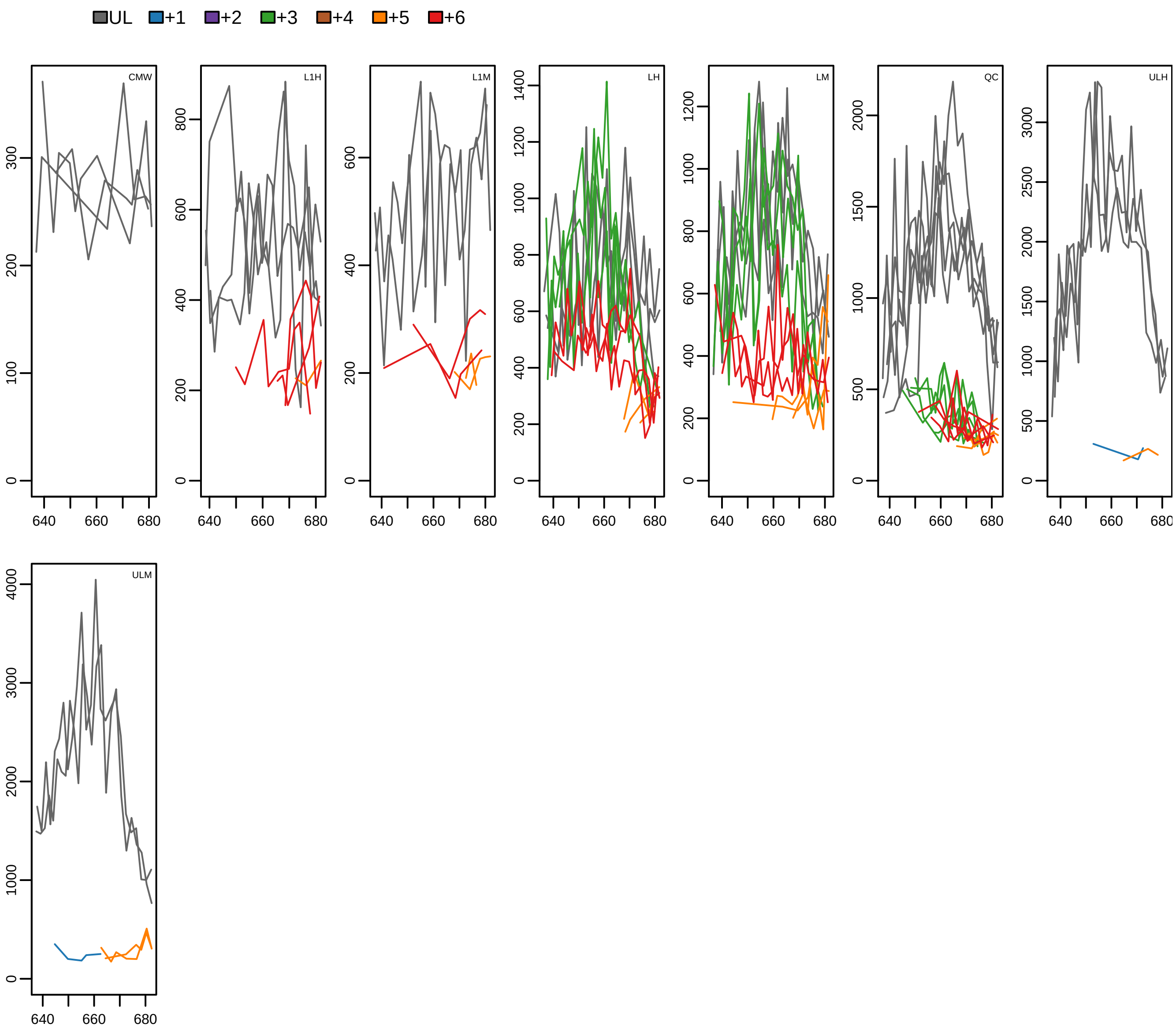

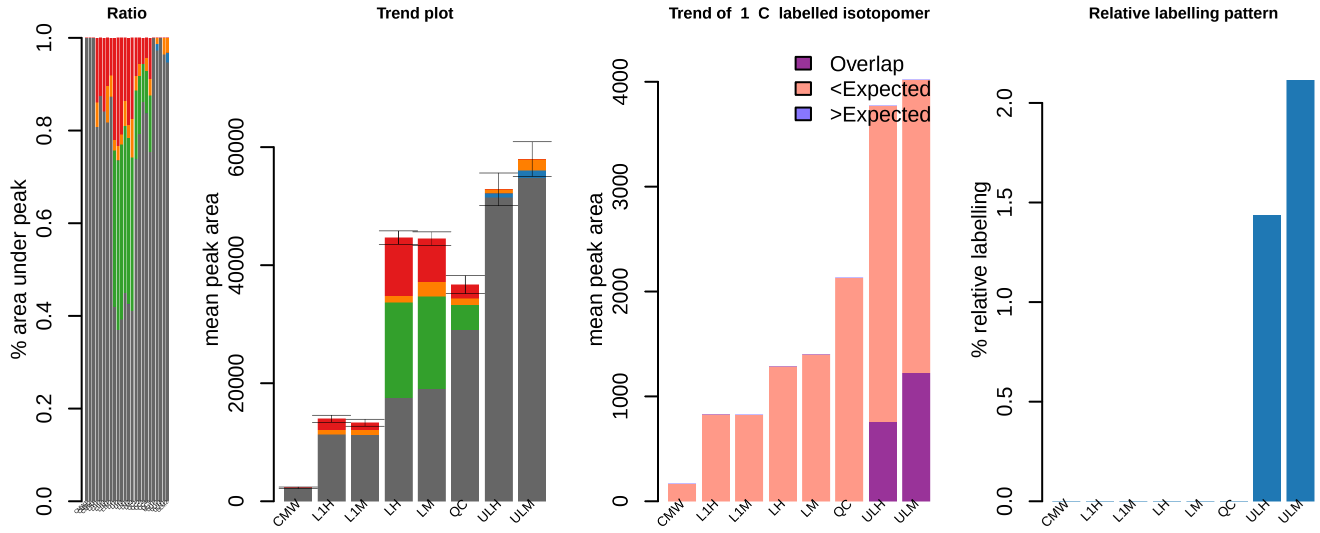

D−Fructose 6−phosphate

Formula: C6H13O9P Mass: 260.03 Std.RT: 1037.8975068 Ion: NEG

G1

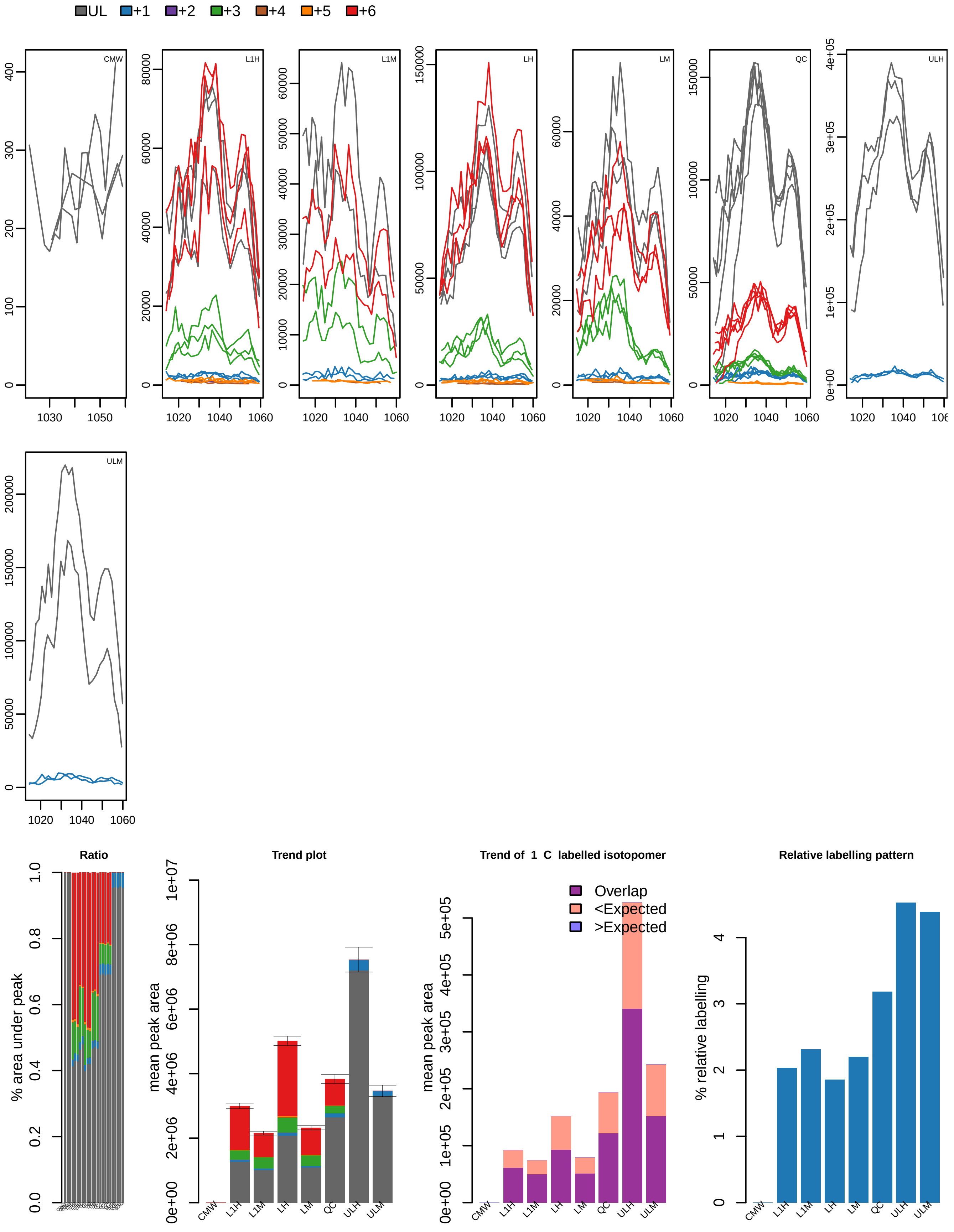

D−Fructose 6−phosphate

Formula: C6H13O9P Mass: 260.03 Std.RT: 1037.8975068 Ion: NEG

G2

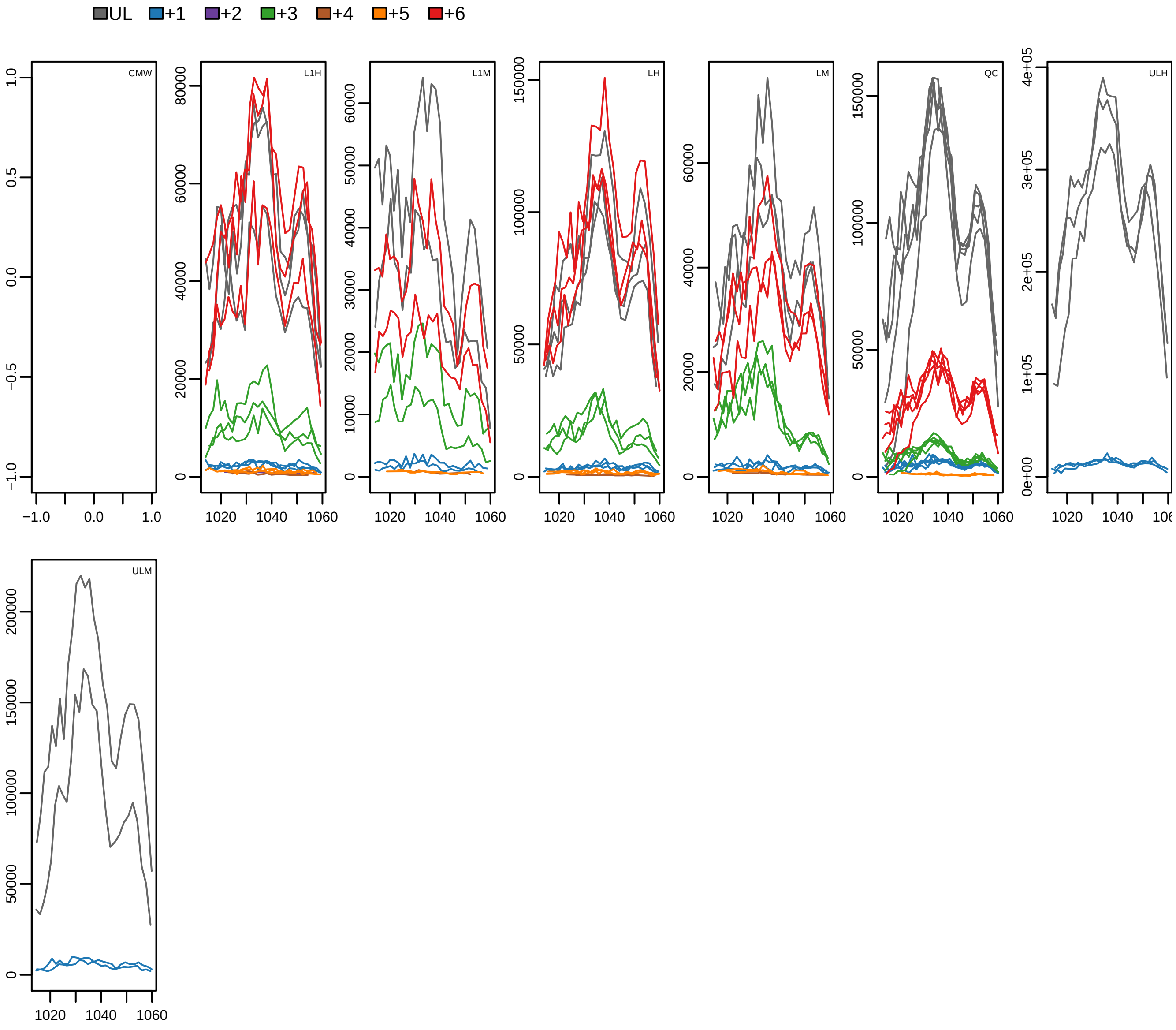

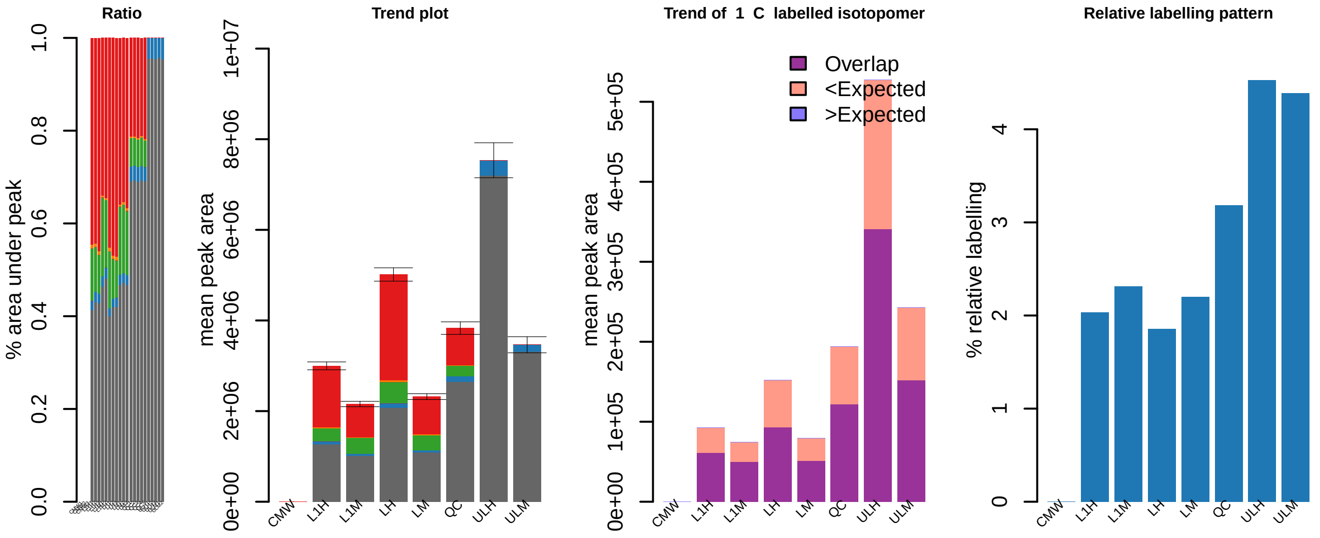

D−Ribose 5−phosphate

Formula: C5H11O8P Mass: 230.019 Std.RT: 1013.046189 Ion: NEG

G1

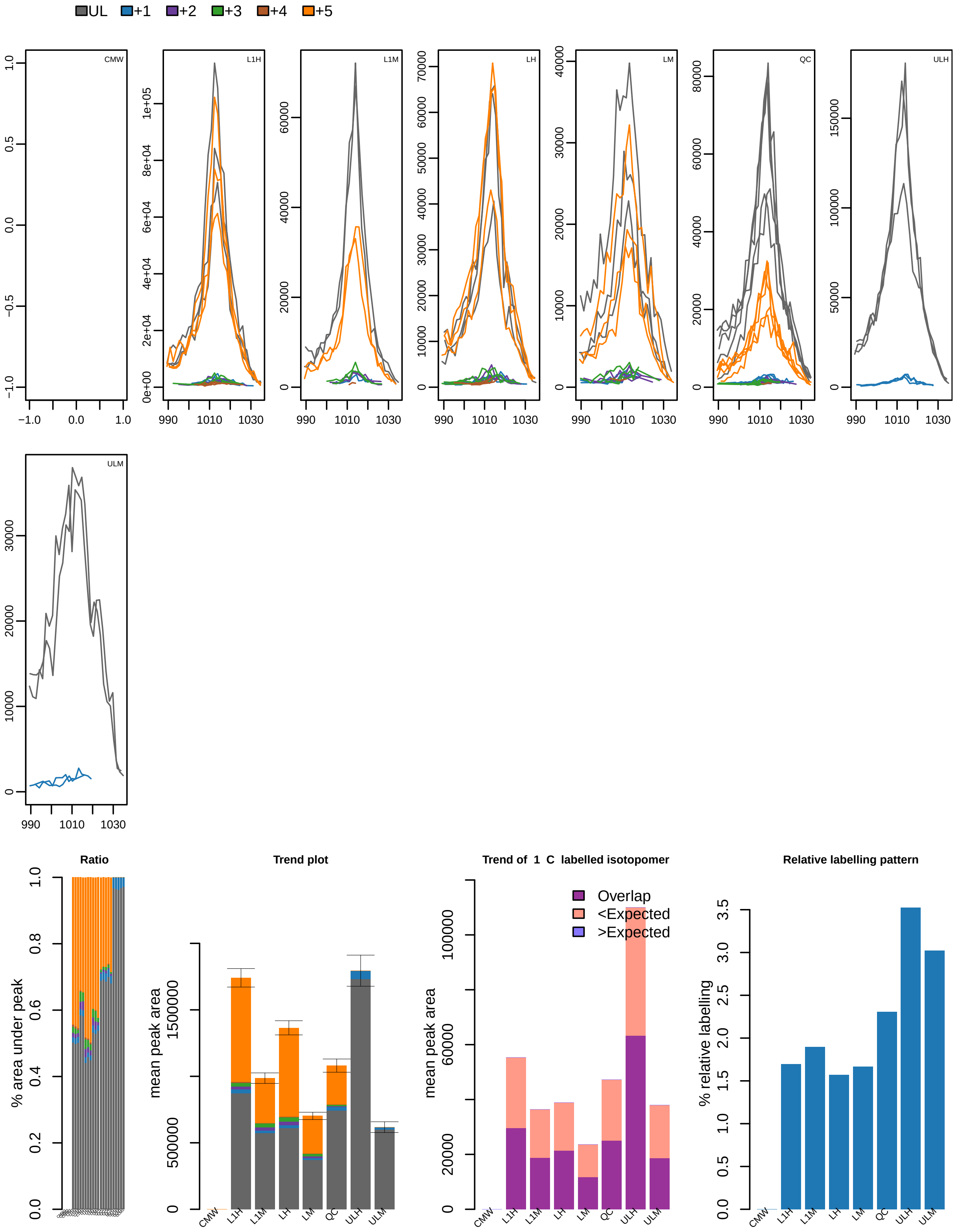

2−Methylcitrate

Formula: C7H10O7 Mass: 206.043 Std.RT: 1109.390823 Ion: NEG

G1

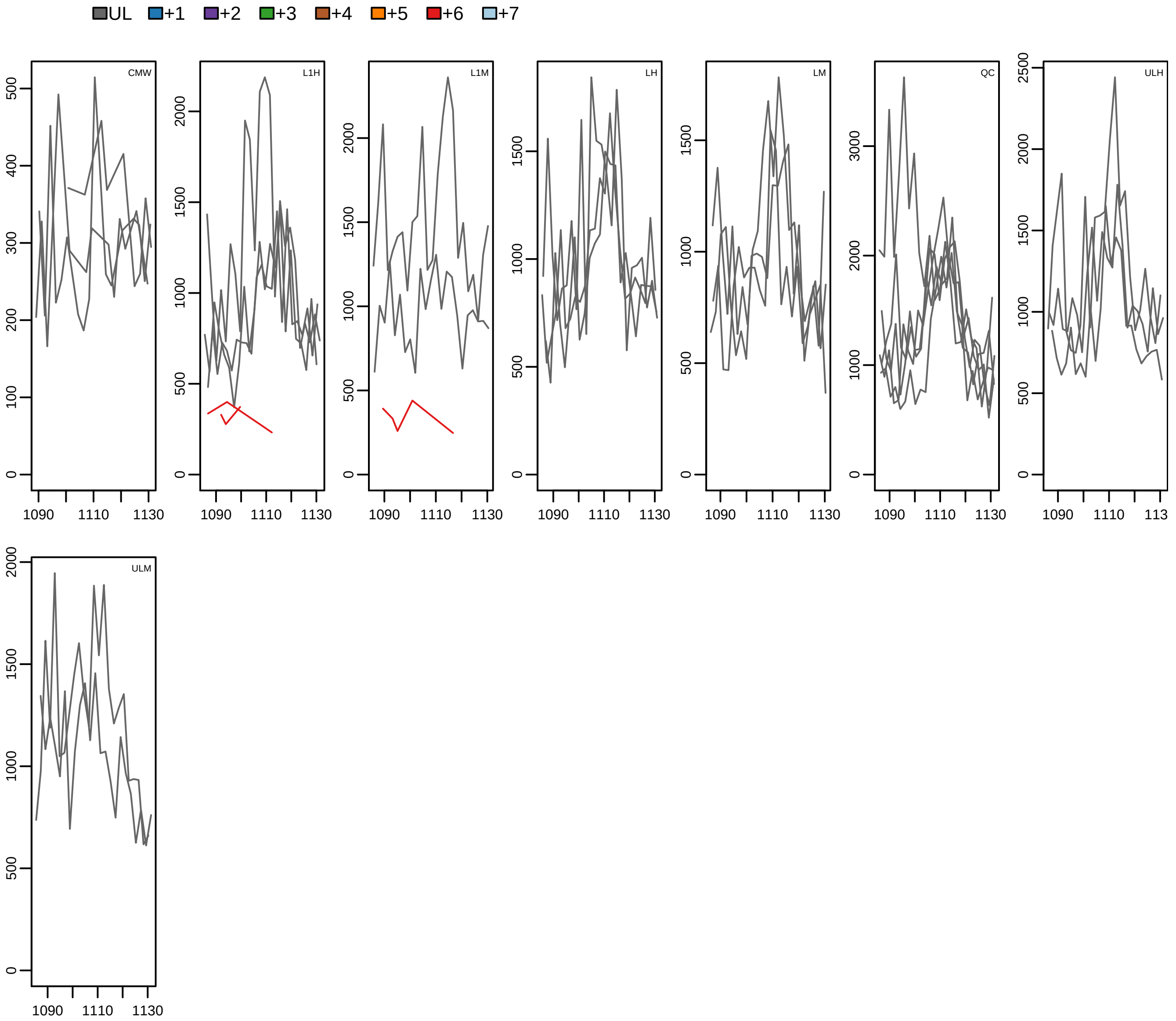

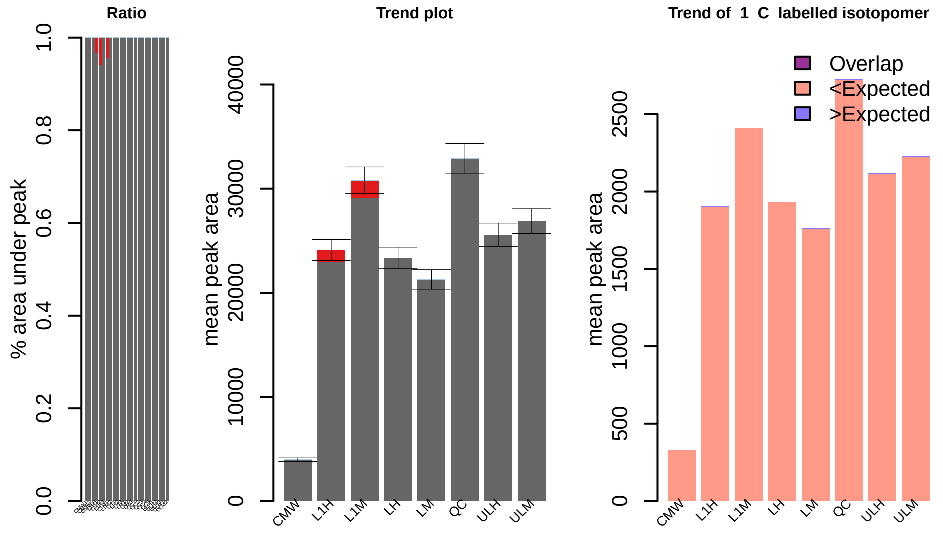

(R)−Lactate

Formula: C3H6O3 Mass: 90.032 Std.RT: 552.58095552 Ion: NEG

G1

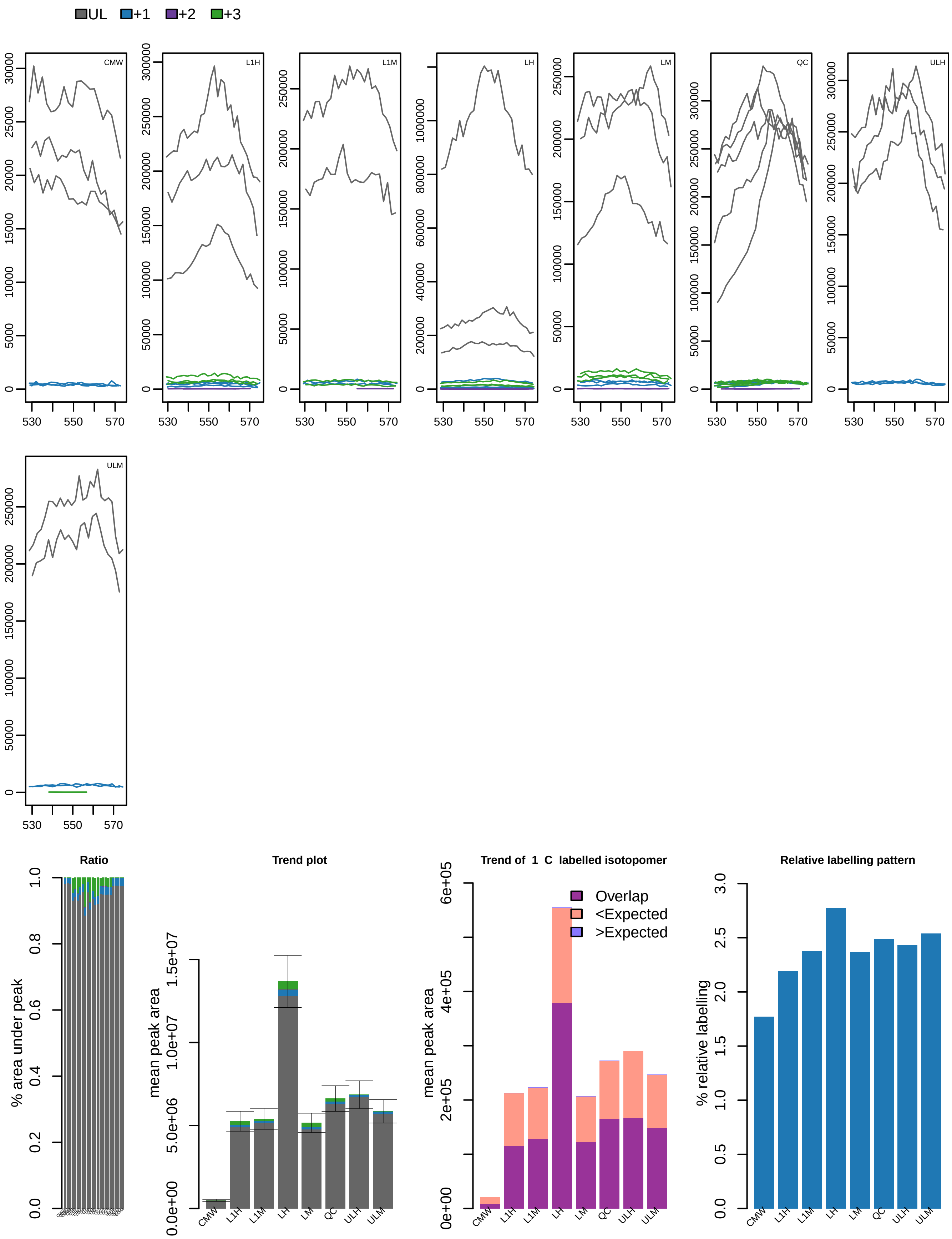

D−Glucose 6−phosphate

Formula: C6H13O9P Mass: 260.03 Std.RT: 1091.6683452 Ion: NEG

G1

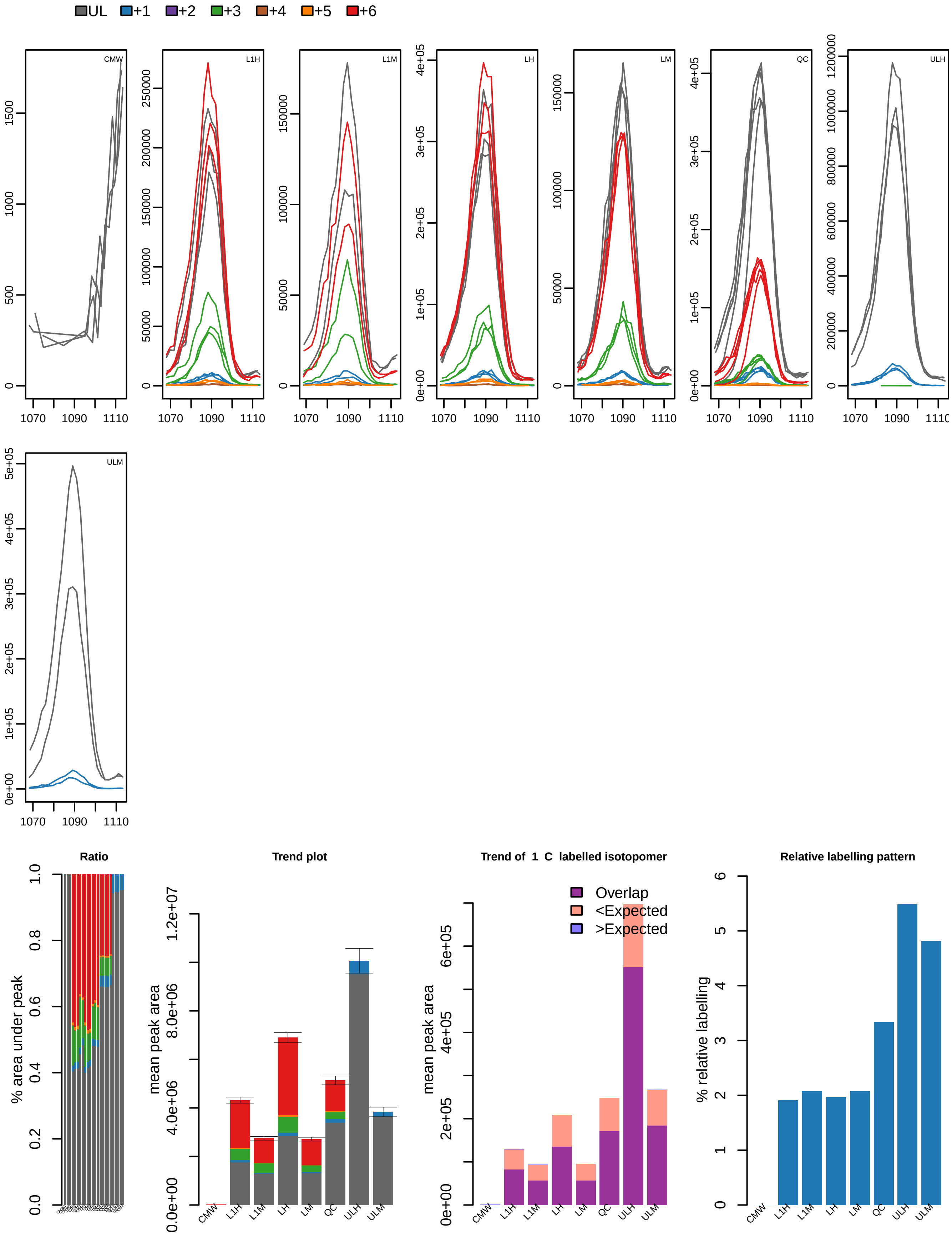

Glycogen

G1

Formula: C24H42O21 Mass: 666.222 Std.RT: 1182.698556 Ion: NEG

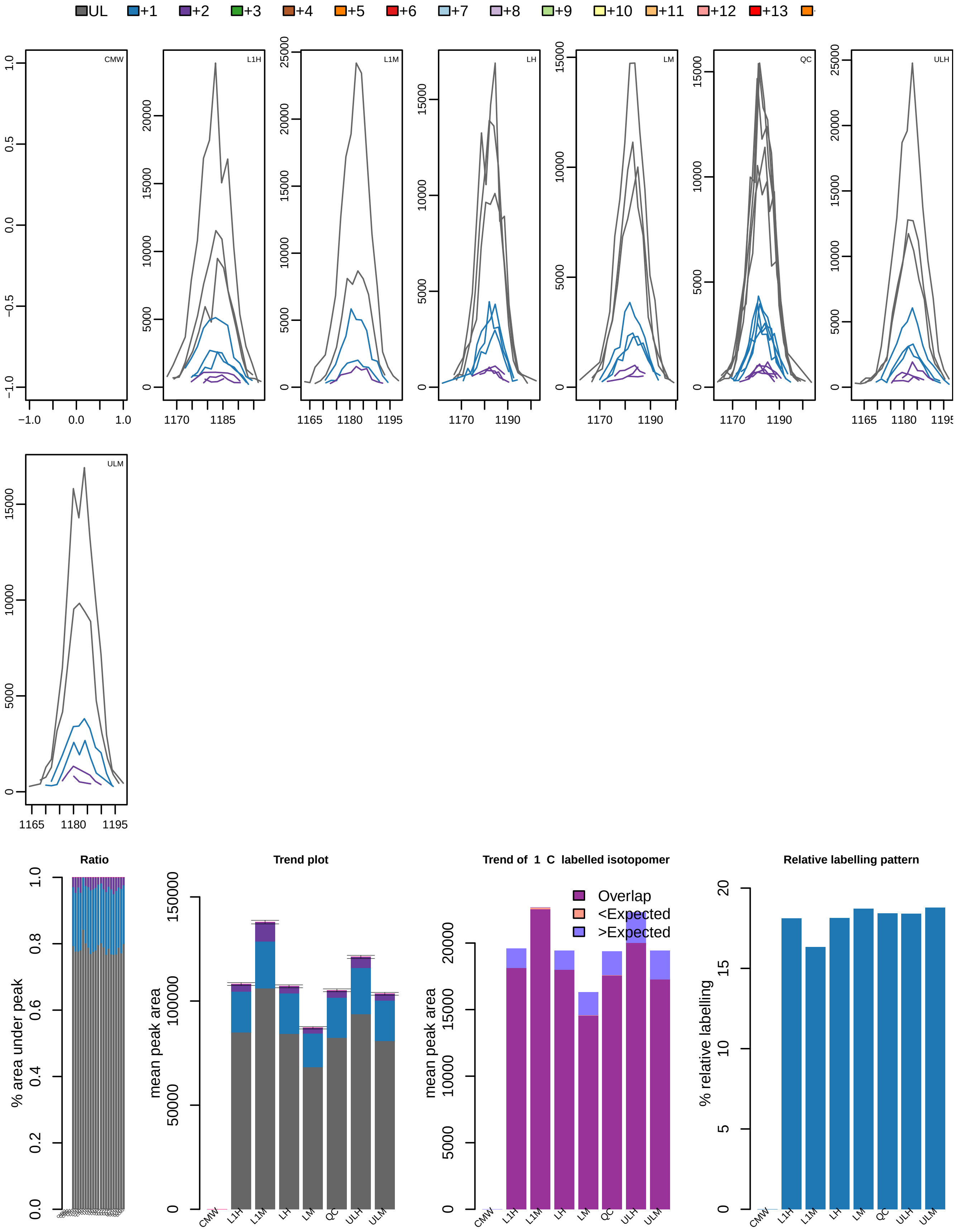

D−Fructose 1,6−bisphosphate

Formula: C6H14O12P2 Mass: 339.996 Std.RT: 1156.223319 Ion: NE

G1

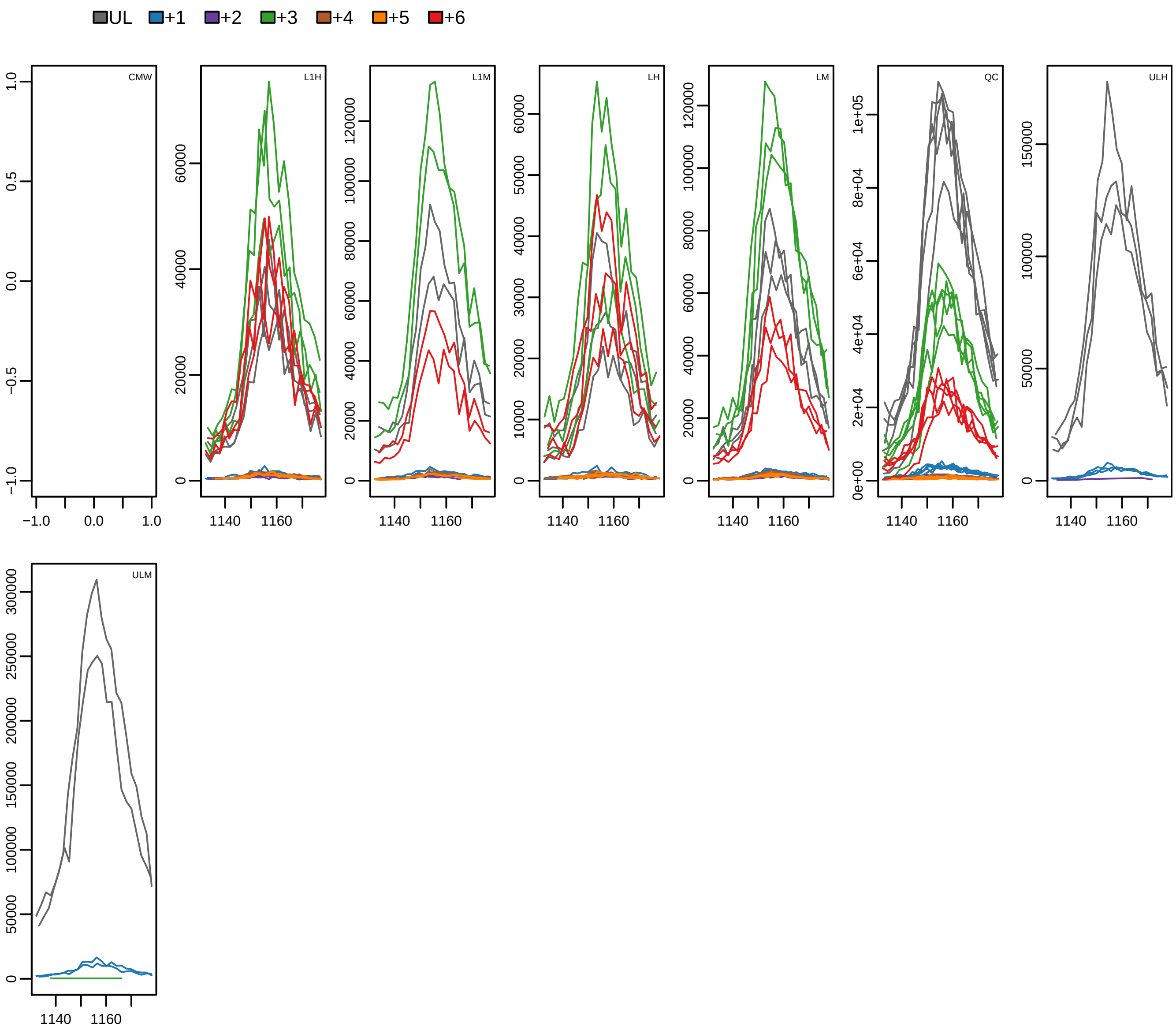

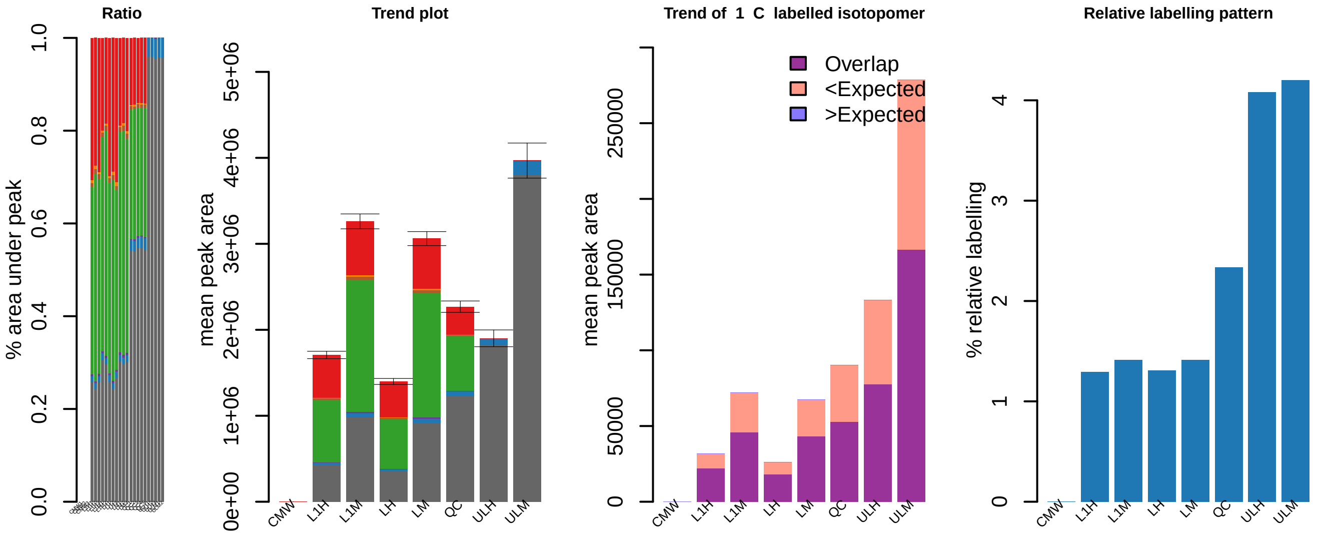

ATP

G1

Formula: C10H16N5O13P3 Mass: 506.996 Std.RT: 1062.654774 Ion:

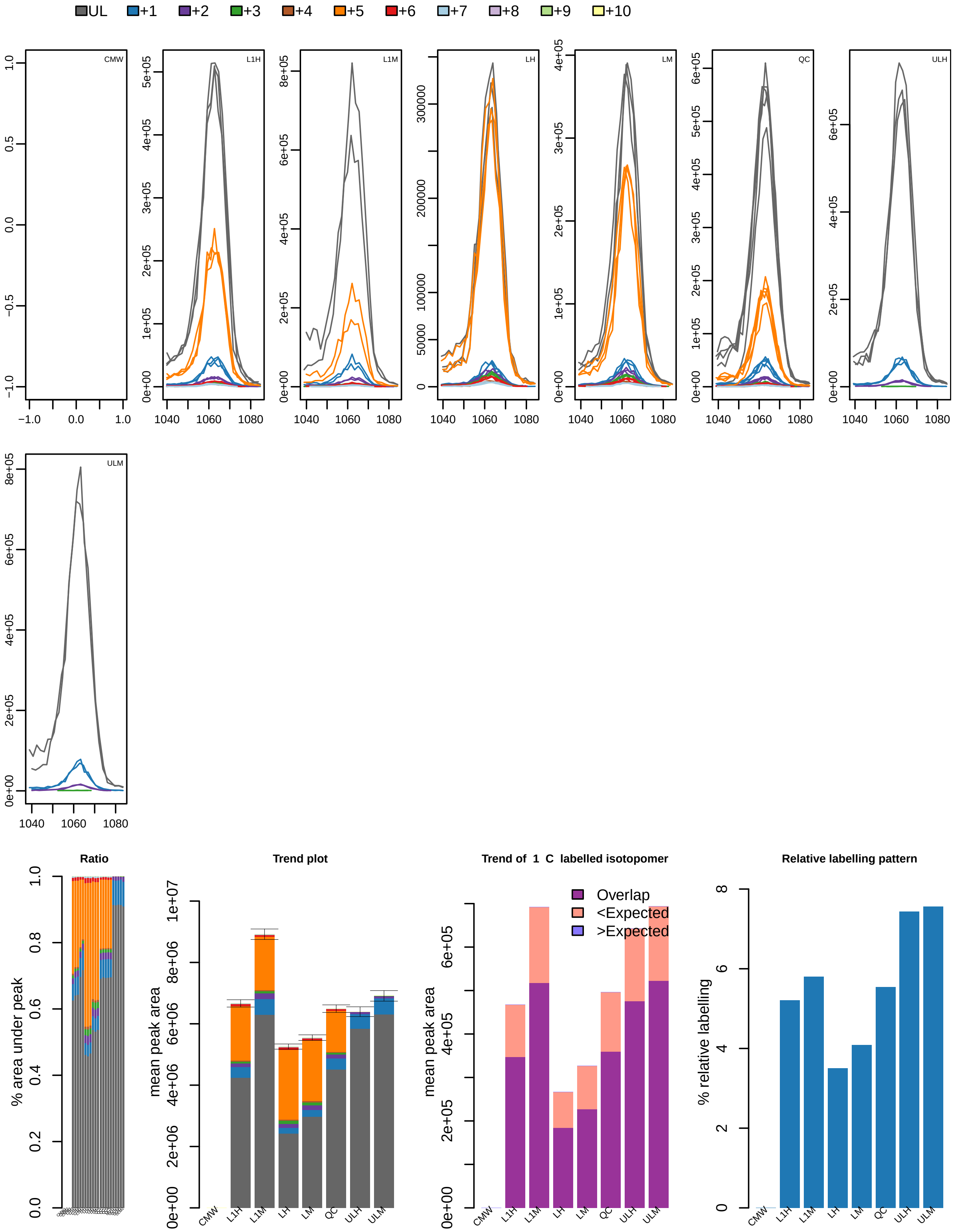

ADP

Formula: C10H15N5O10P2 Mass: 427.029 Std.RT: 981.412287 Ion:

G1

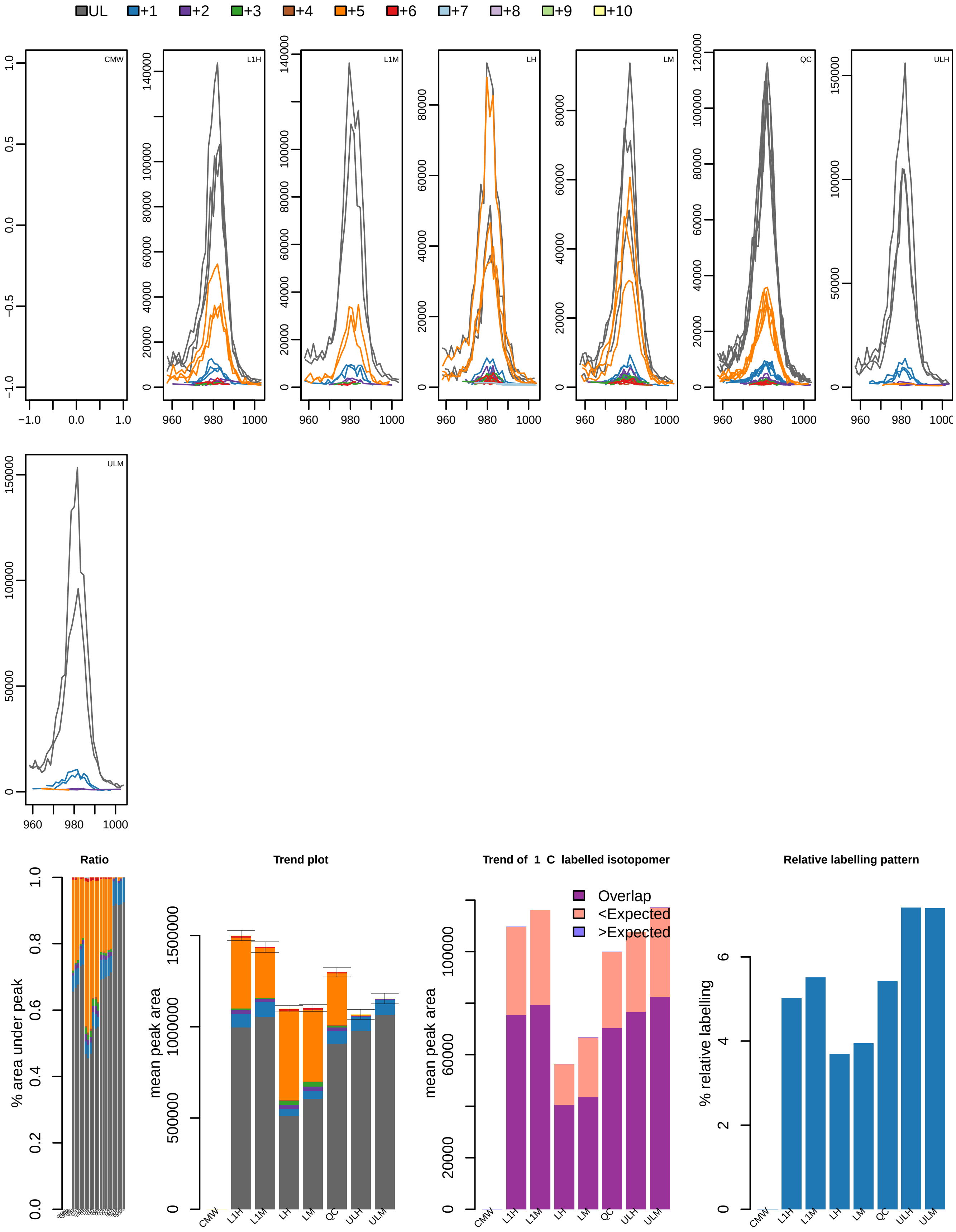

Glycodeoxycholate

Formula: C26H43NO5 Mass: 449.314 Std.RT: 250.0330956 Ion: NEC

G1

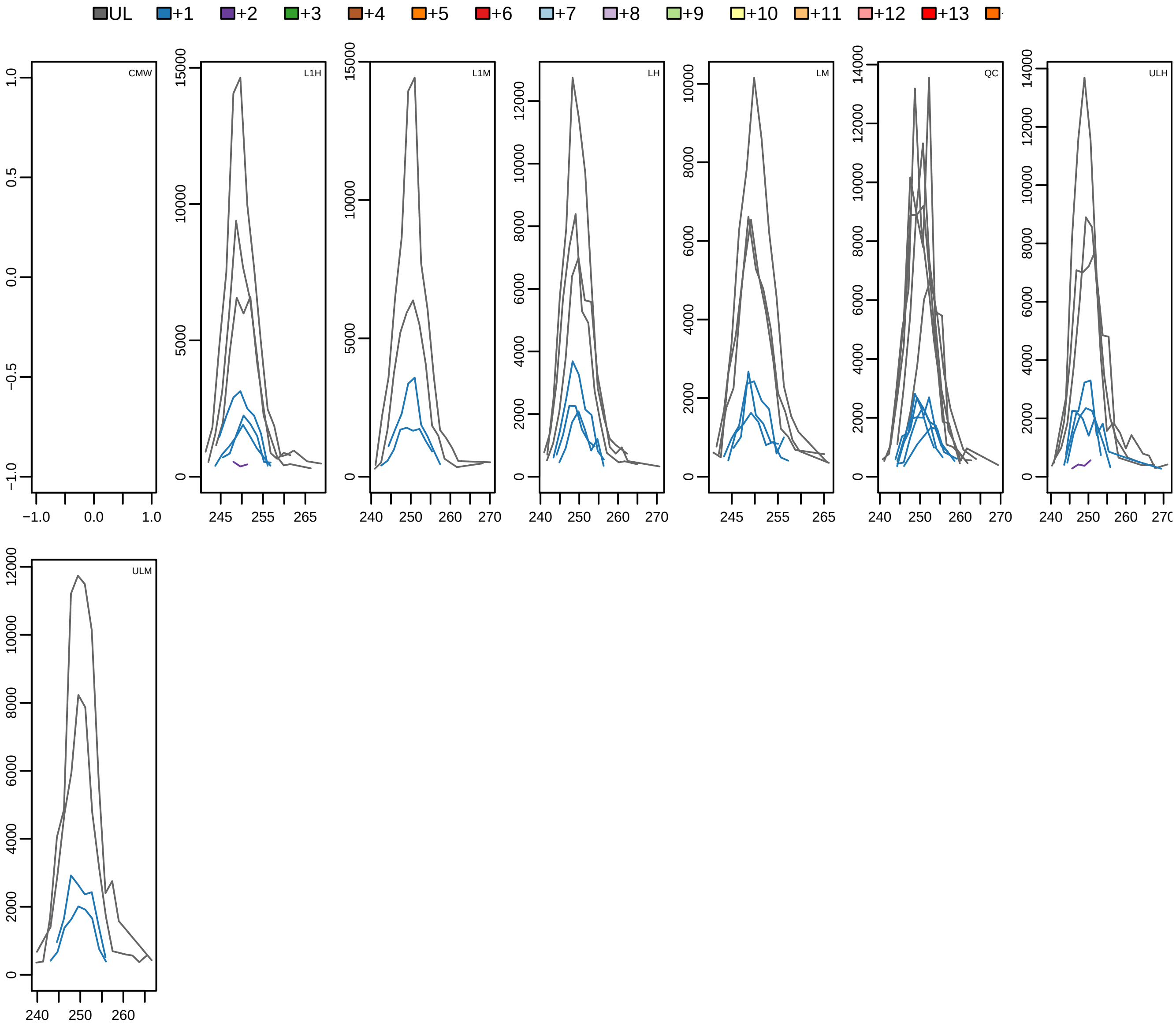

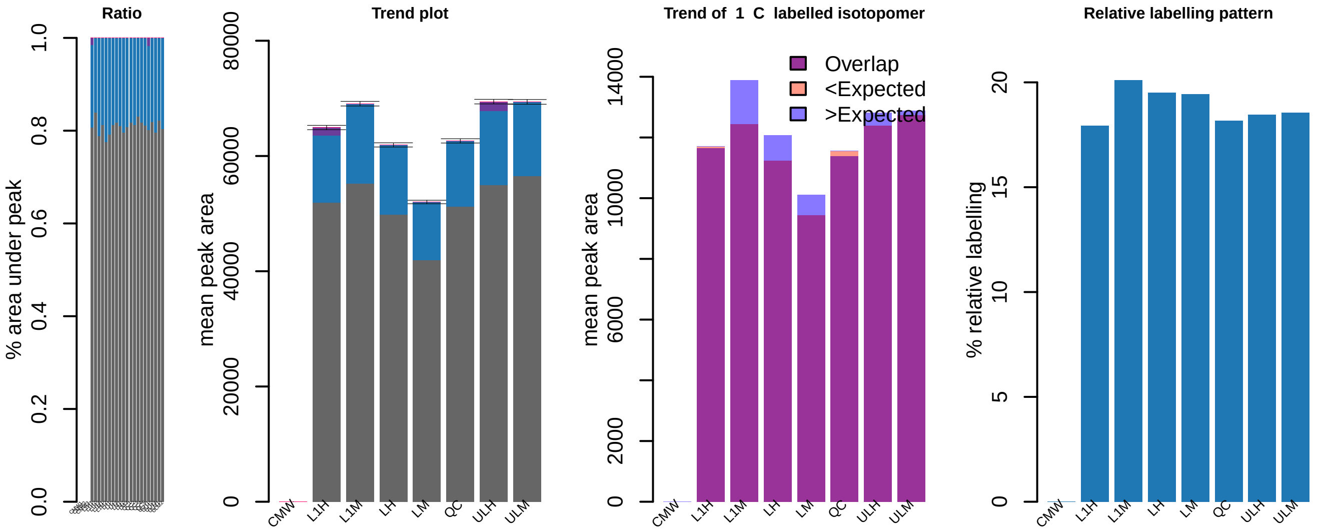

Taurine

Formula: C2H7NO3S Mass: 125.015 Std.RT: 950.2777188 Ion: NEG

G1

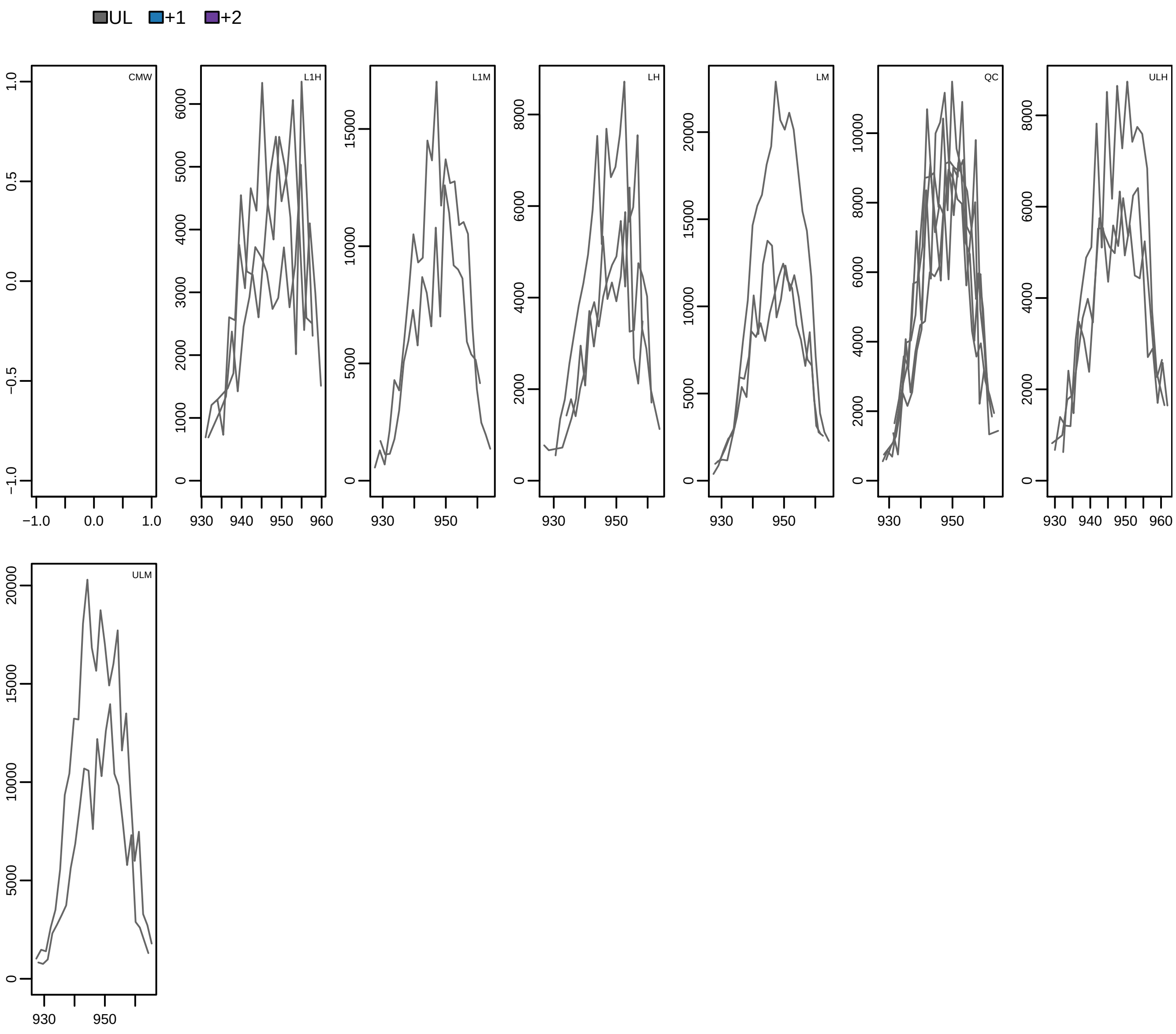

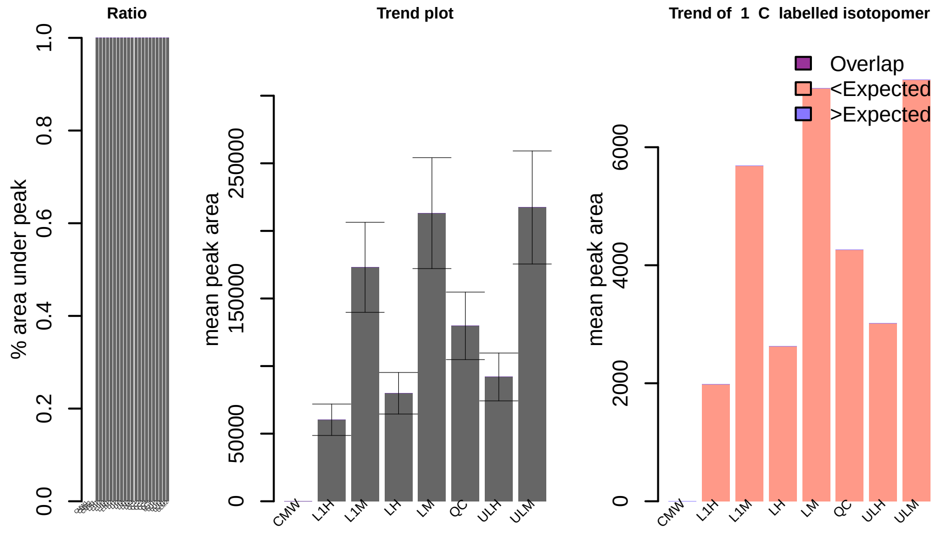

Icosadienoic acid

Formula: C20H36O2 Mass: 308.272 Std.RT: 211.79662002 Ion: NEG

G1

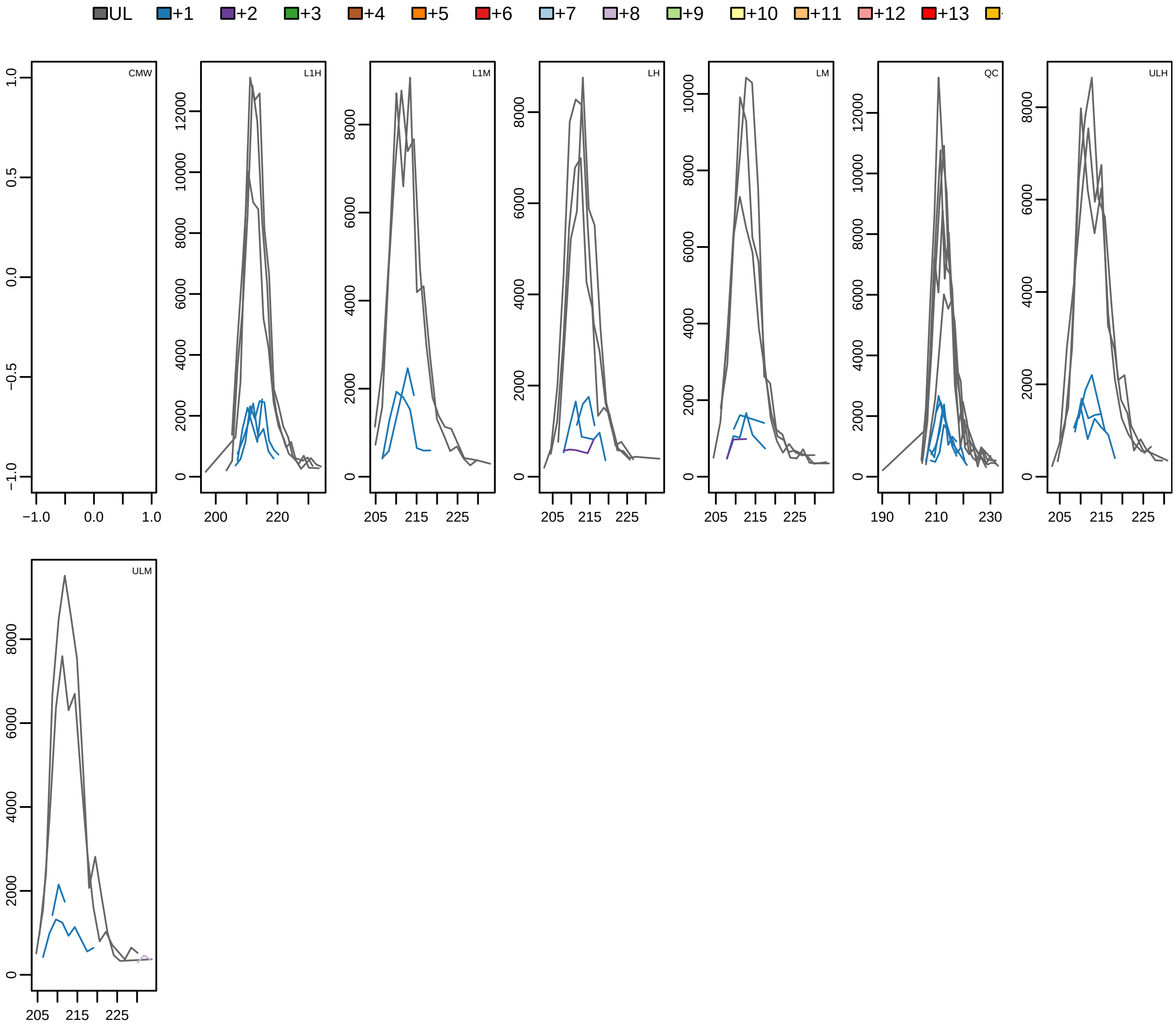

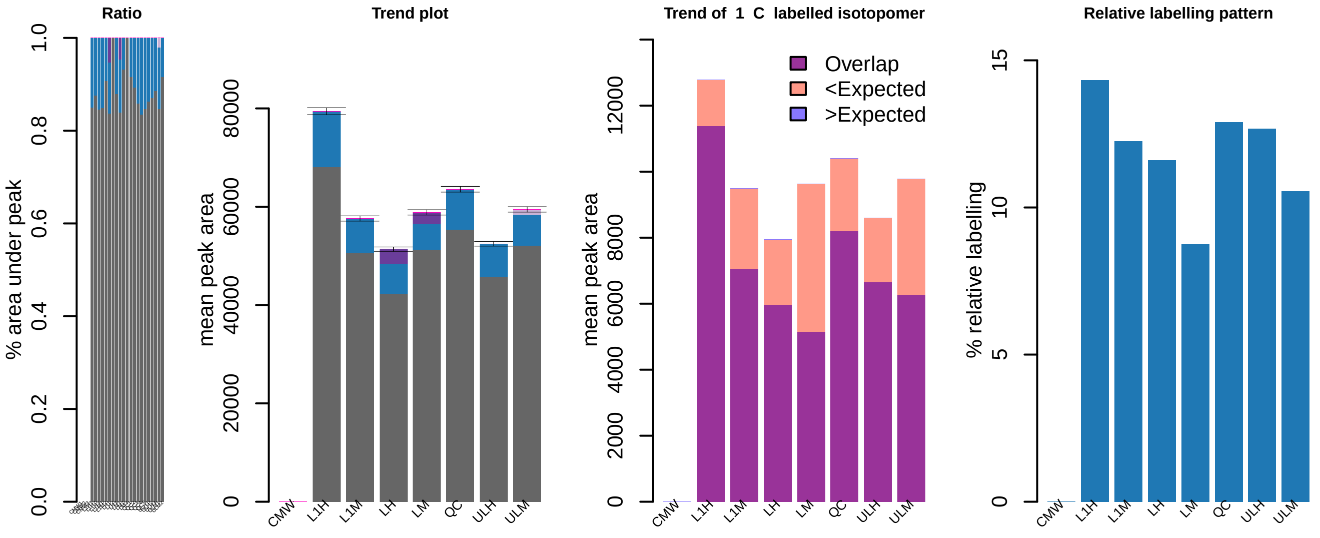

Tetracosanoic acid

Formula: C24H48O2 Mass: 368.365 Std.RT: 209.76437442 Ion: NEG

G1

UL +1 +2 +3 +4 +5 +6 +7 +8 +9 +10 +11 +12 +13

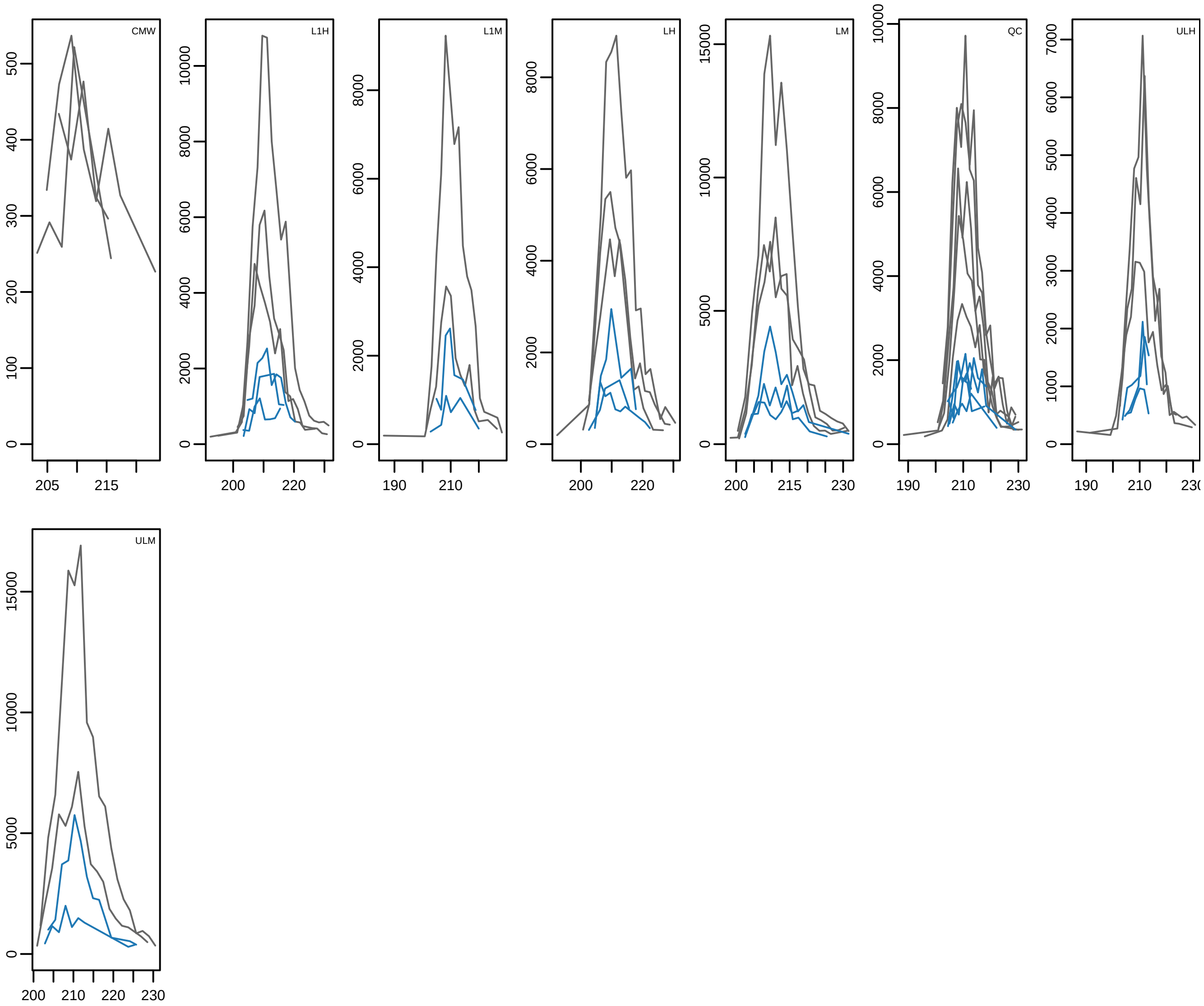

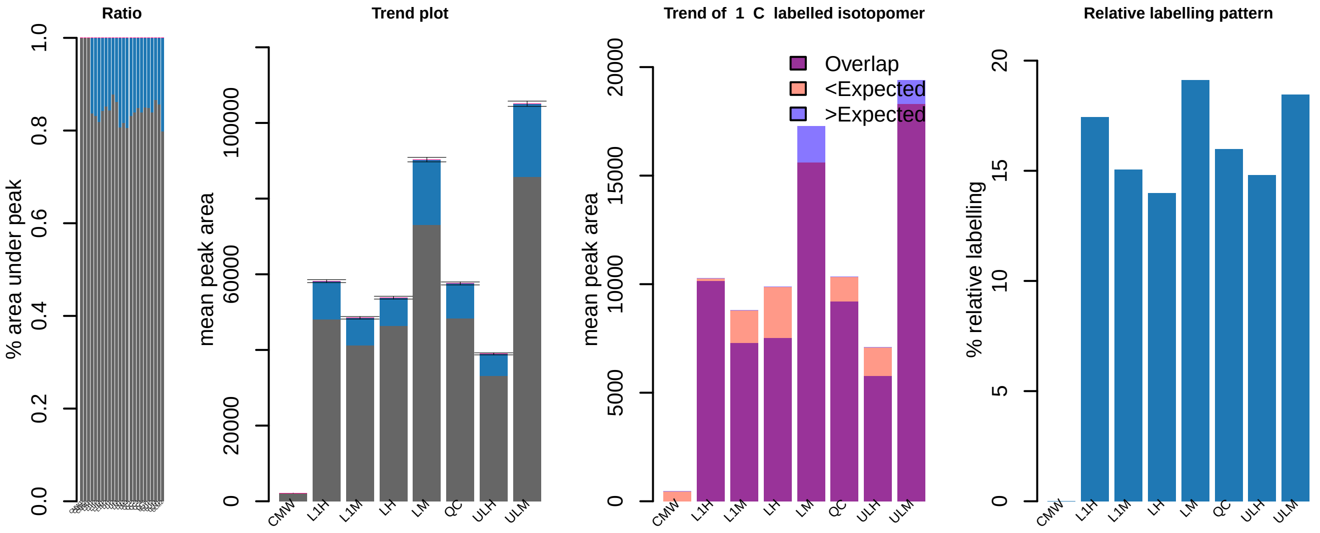

Docosanoic acid

Formula: C22H44O2 Mass: 340.334 Std.RT: 210.67795692 Ion: NEG

G1

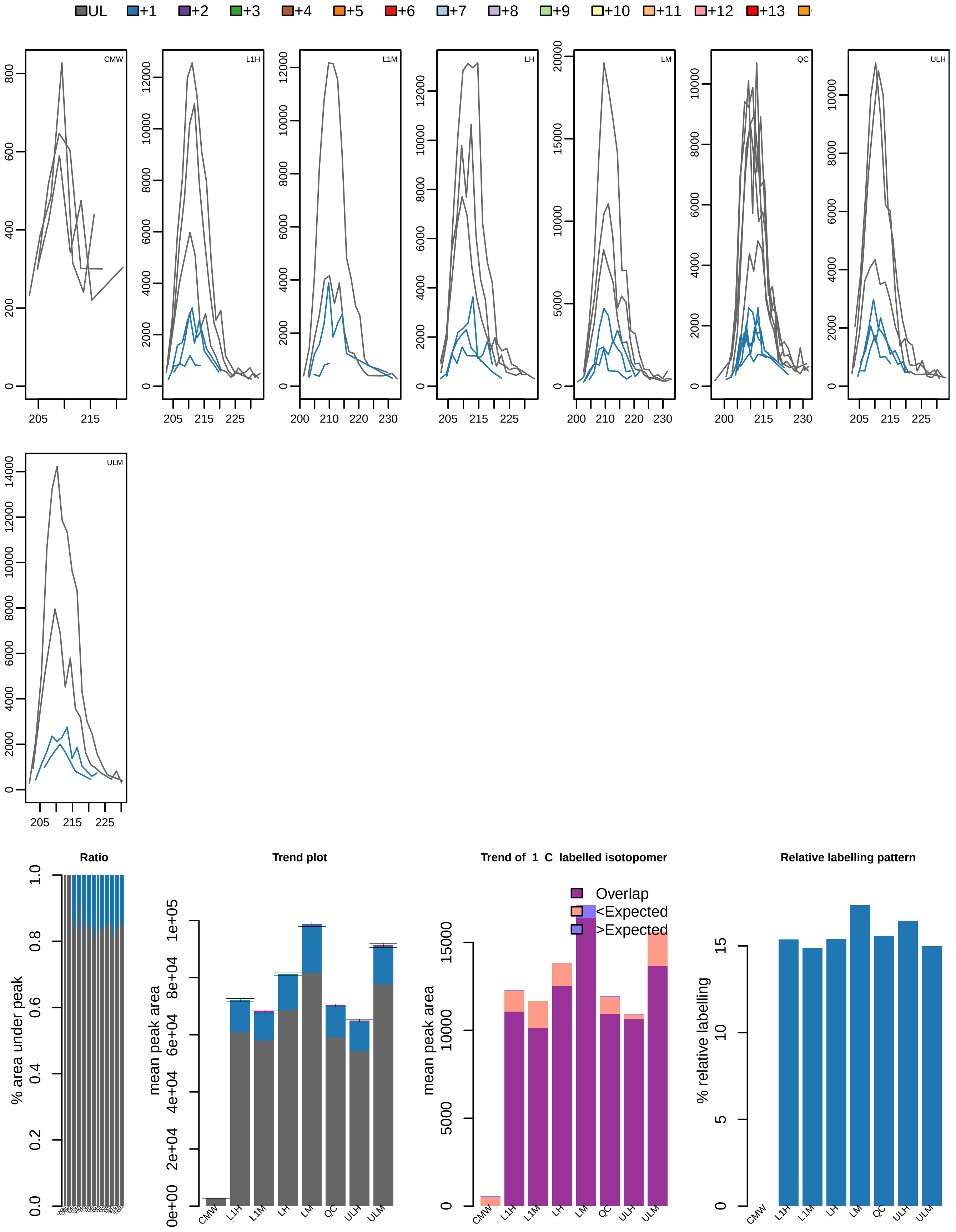

Dodecanoic acid

Formula: $C_{12}H_{24}O_2$ Mass: 200.178 Std.RT: 221.5577901 Ion: NEG

G1

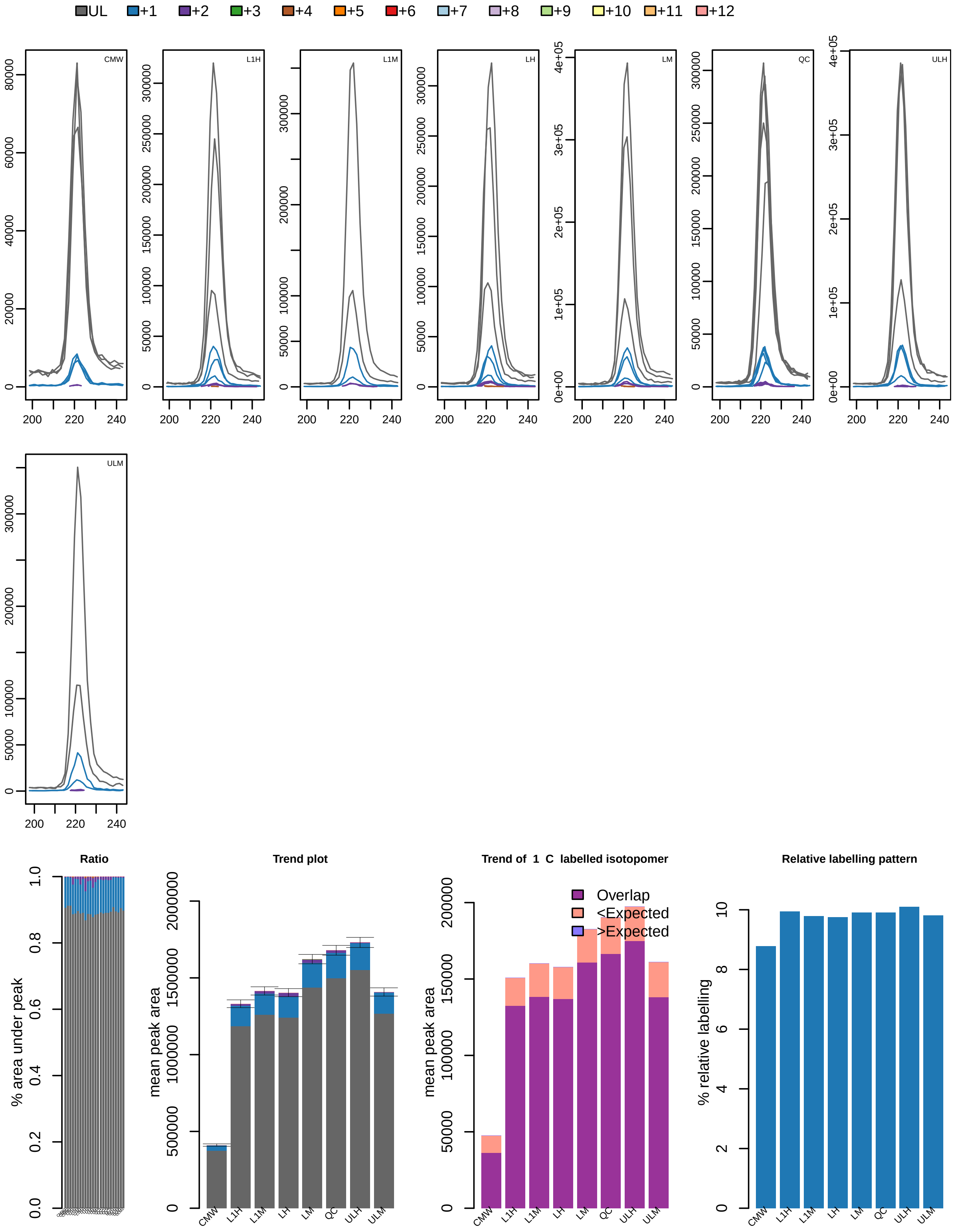

(9Z)−Hexadecenoic acid

Formula: C16H30O2 Mass: 254.225 Std.RT: 215.313165 Ion: NEG

G1

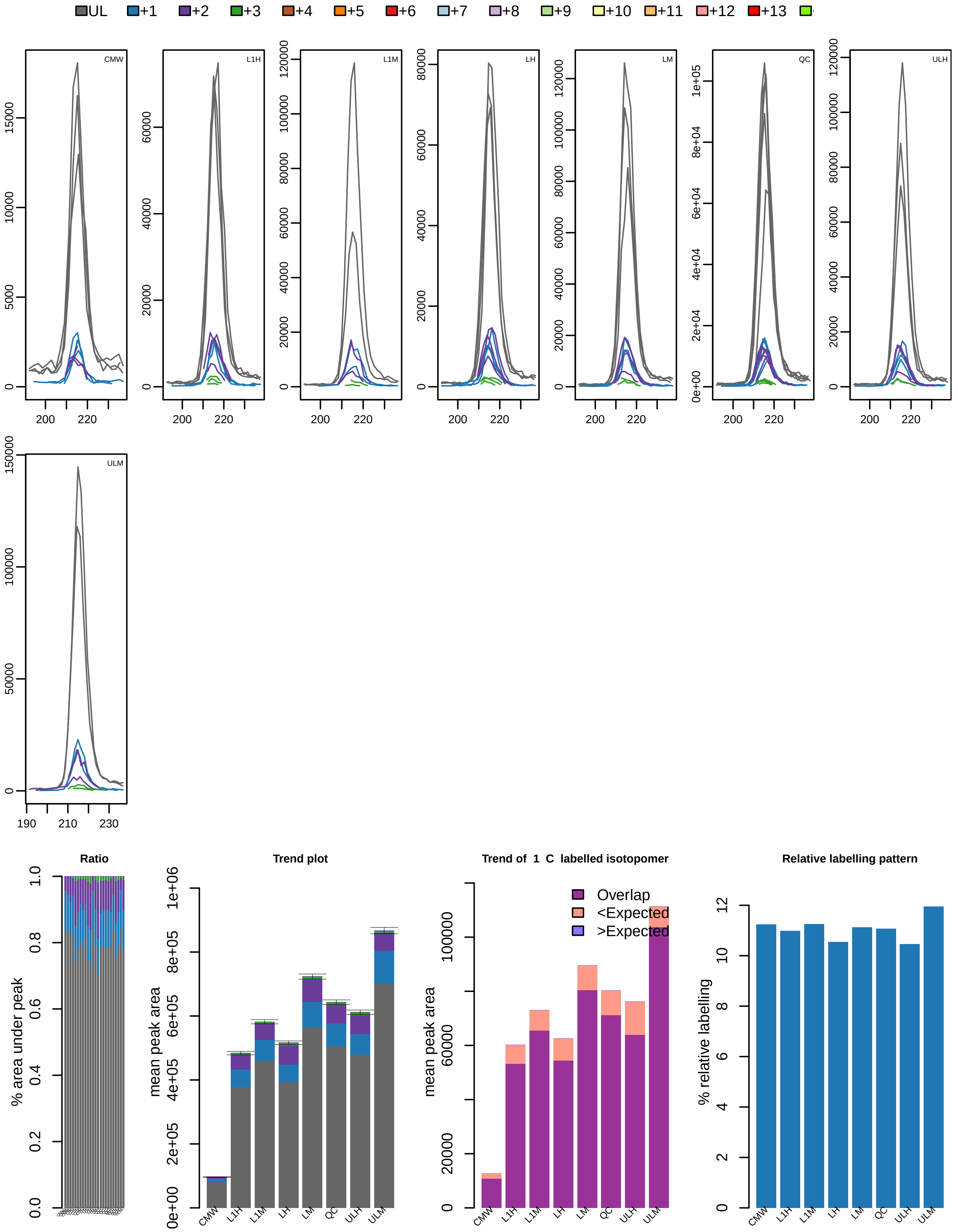

Tetradecanoic acid

Formula: C14H28O2 Mass: 228.209 Std.RT: 217.23320898 Ion: NEG

G1

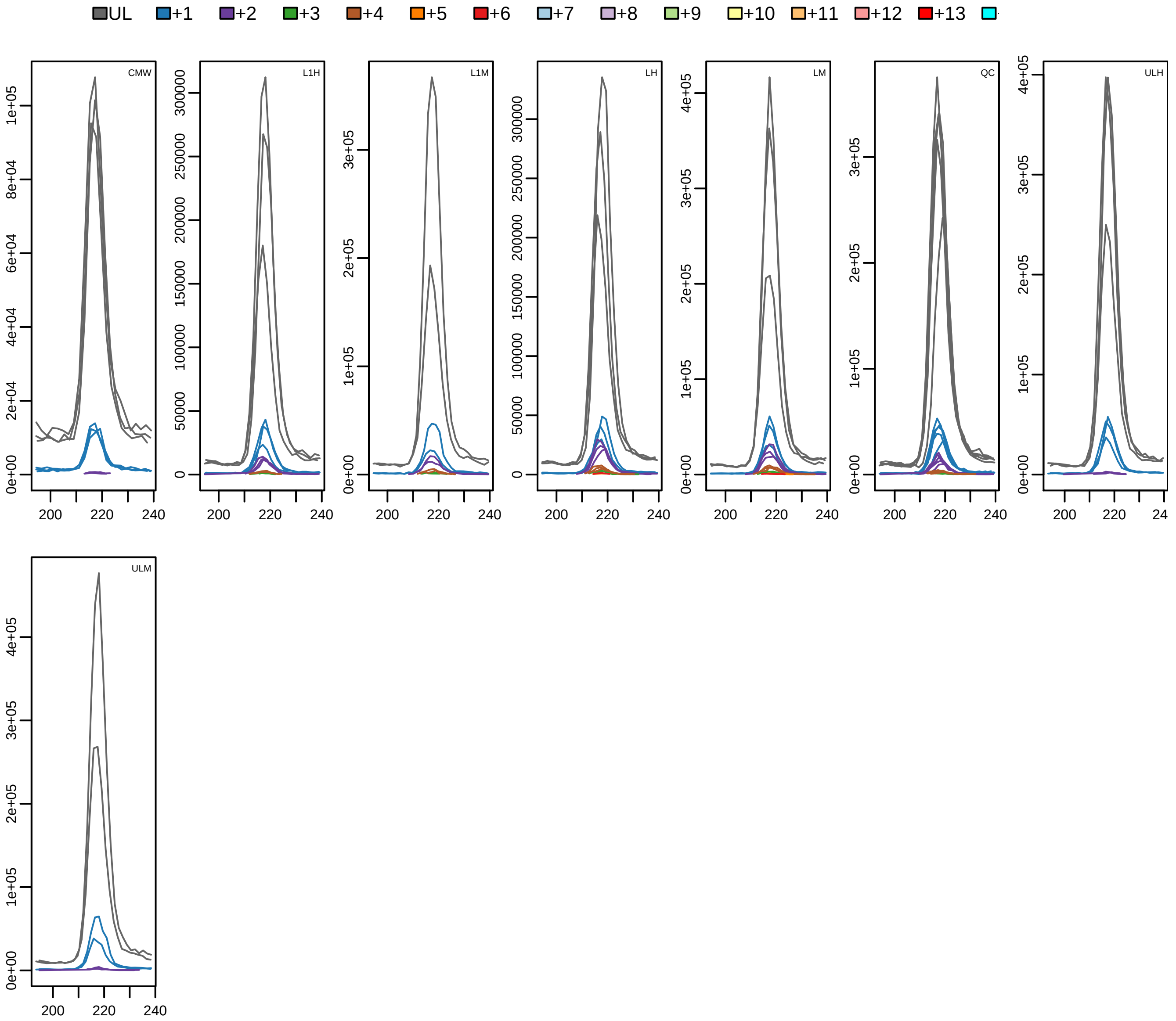

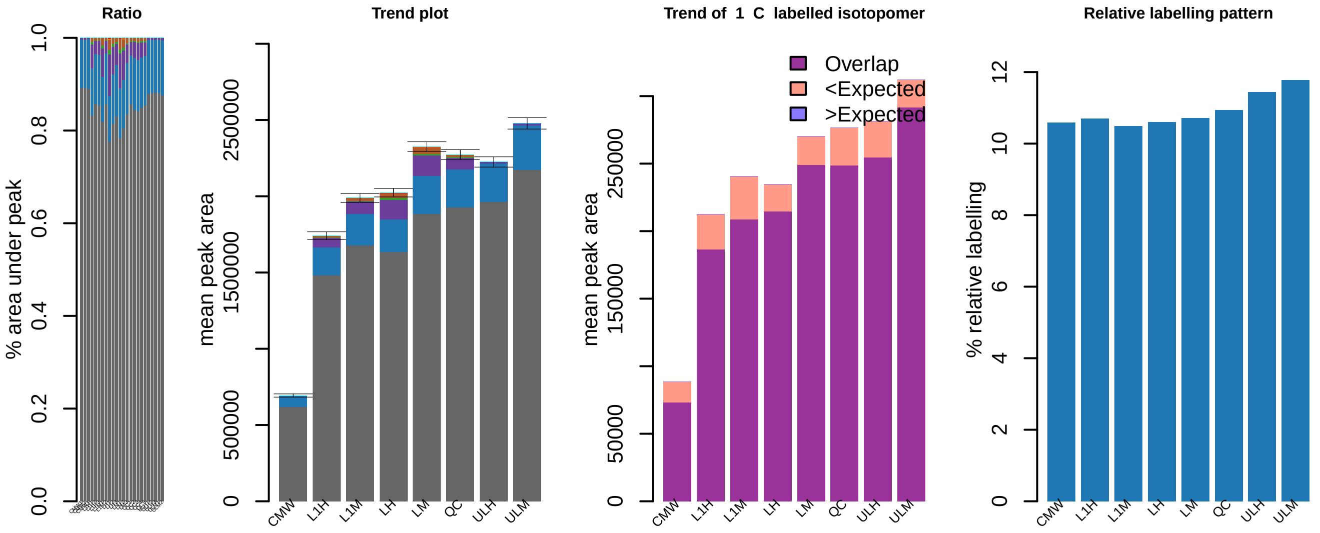

Hexadecanoic acid

Formula: C16H32O2 Mass: 256.24 Std.RT: 214.69370778 Ion: NEG

G1

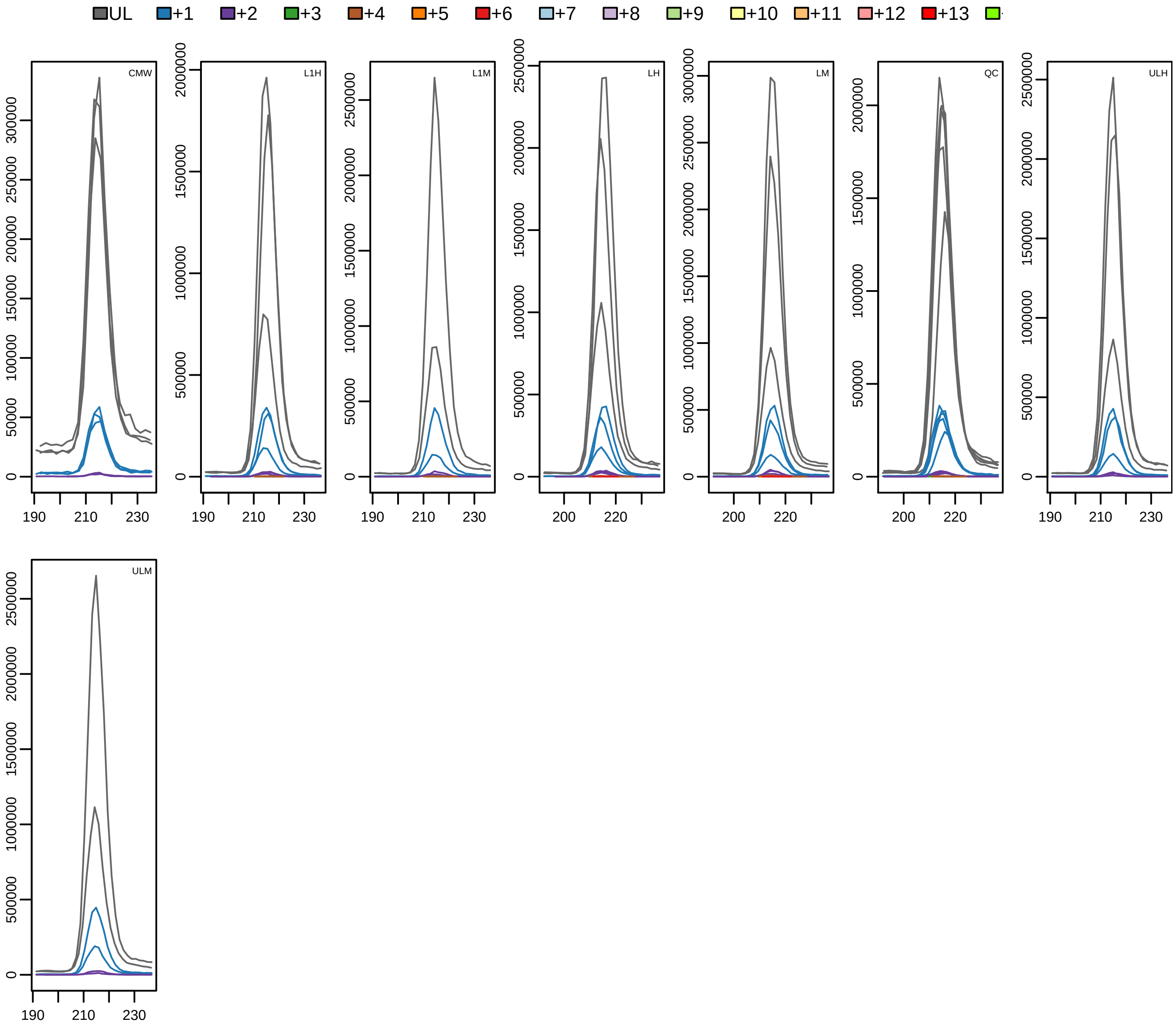

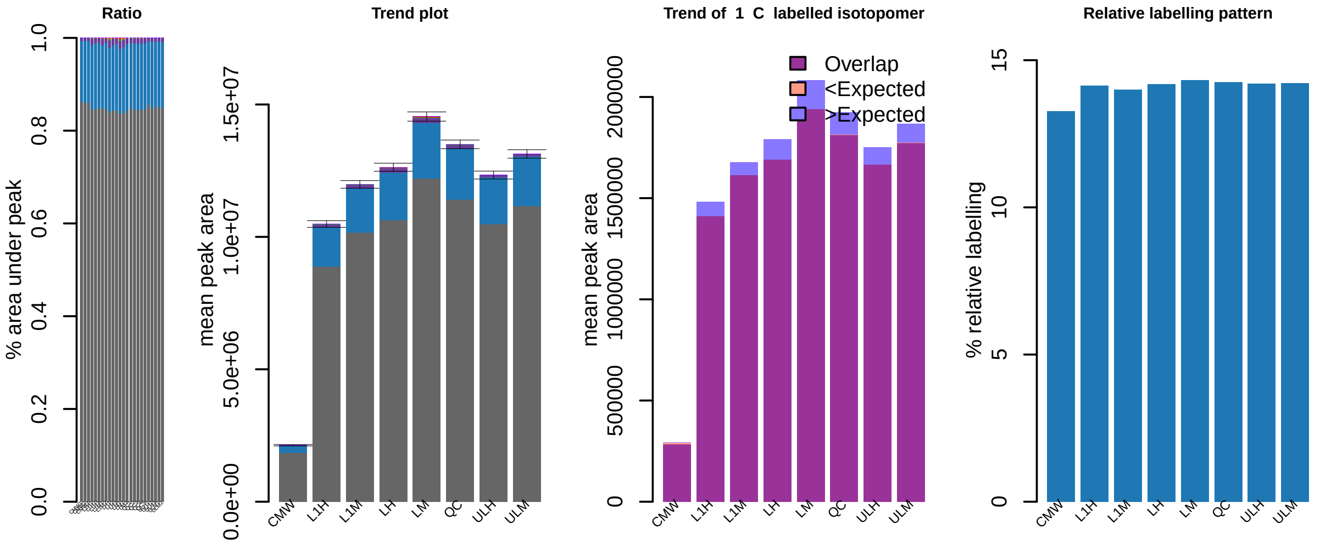

Tetradecanoyl−CoA

Formula: C35H62N7O17P3S Mass: 977.314 Std.RT: 267.67452282 I[illegible]

G1

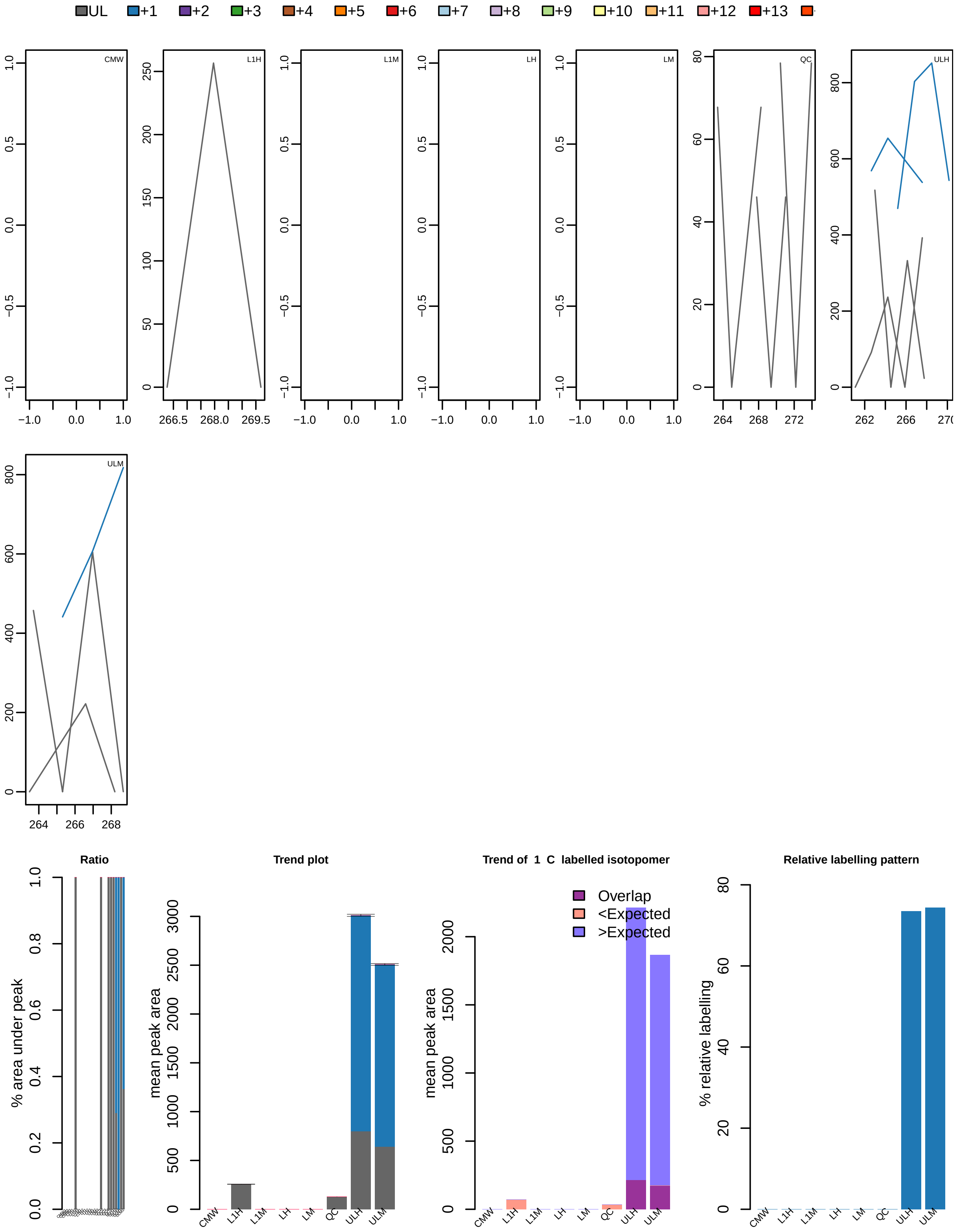

sn−Glycerol 3−phosphate

Formula: C3H9O6P Mass: 172.014 Std.RT: 960.9317526 Ion: NEG

G1

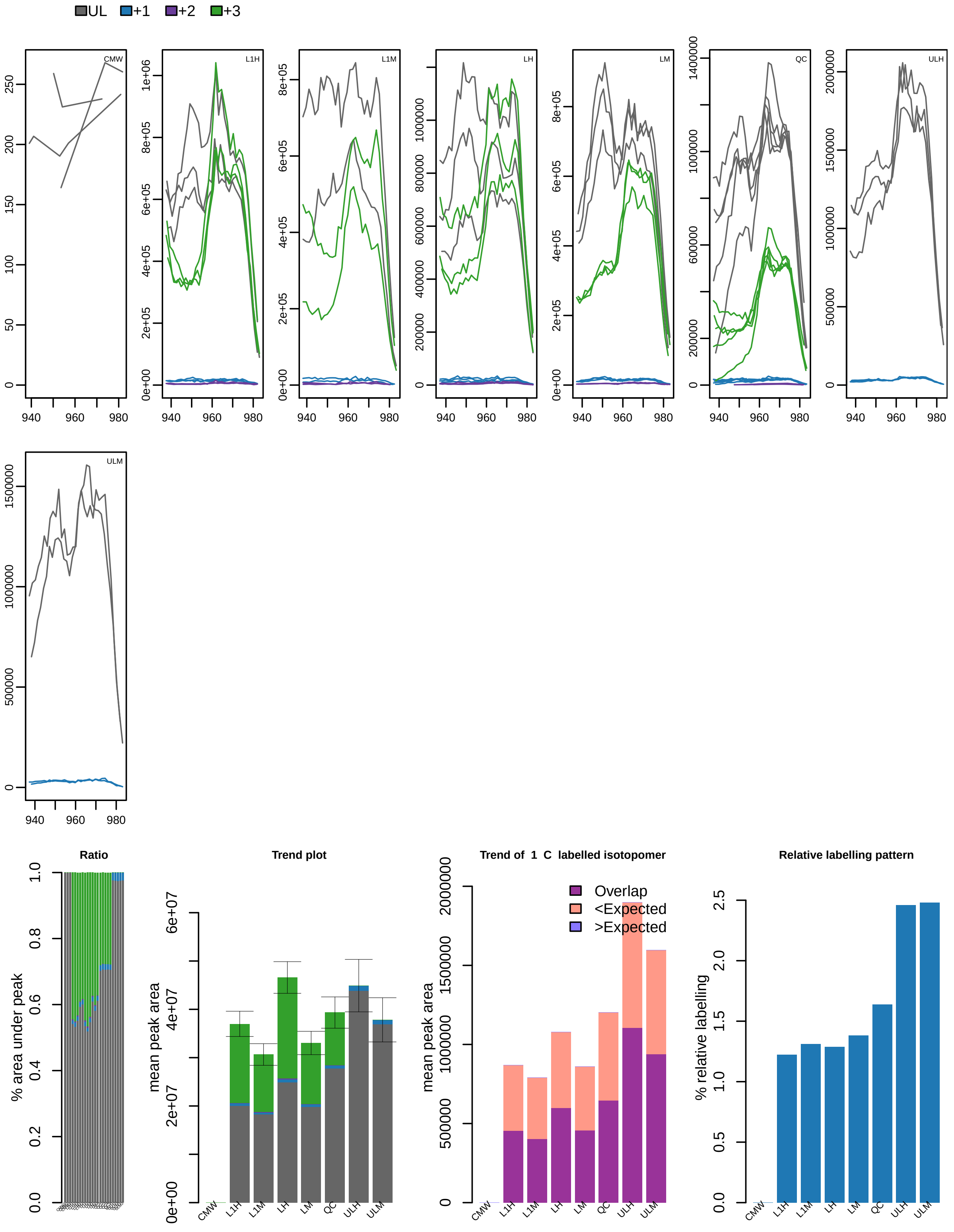

LPA(0:0/18:2(9Z,12Z))

Formula: C21H39O7P Mass: 434.243 Std.RT: 258.96819048 Ion: NE

G1

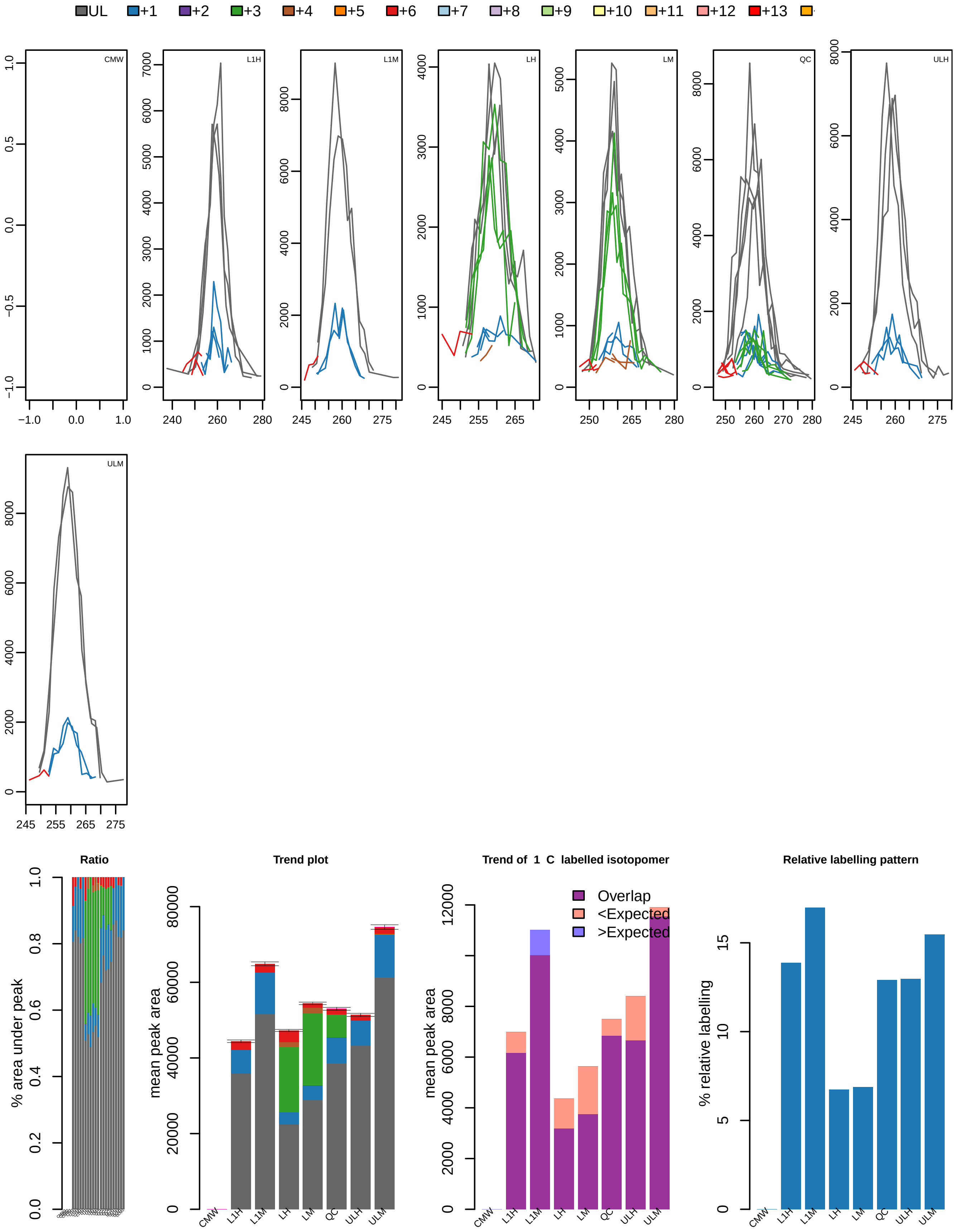

CDP−ethanolamine

Formula: C11H20N4O11P2 Mass: 446.06 Std.RT: 1062.9890538 Ion:

G1

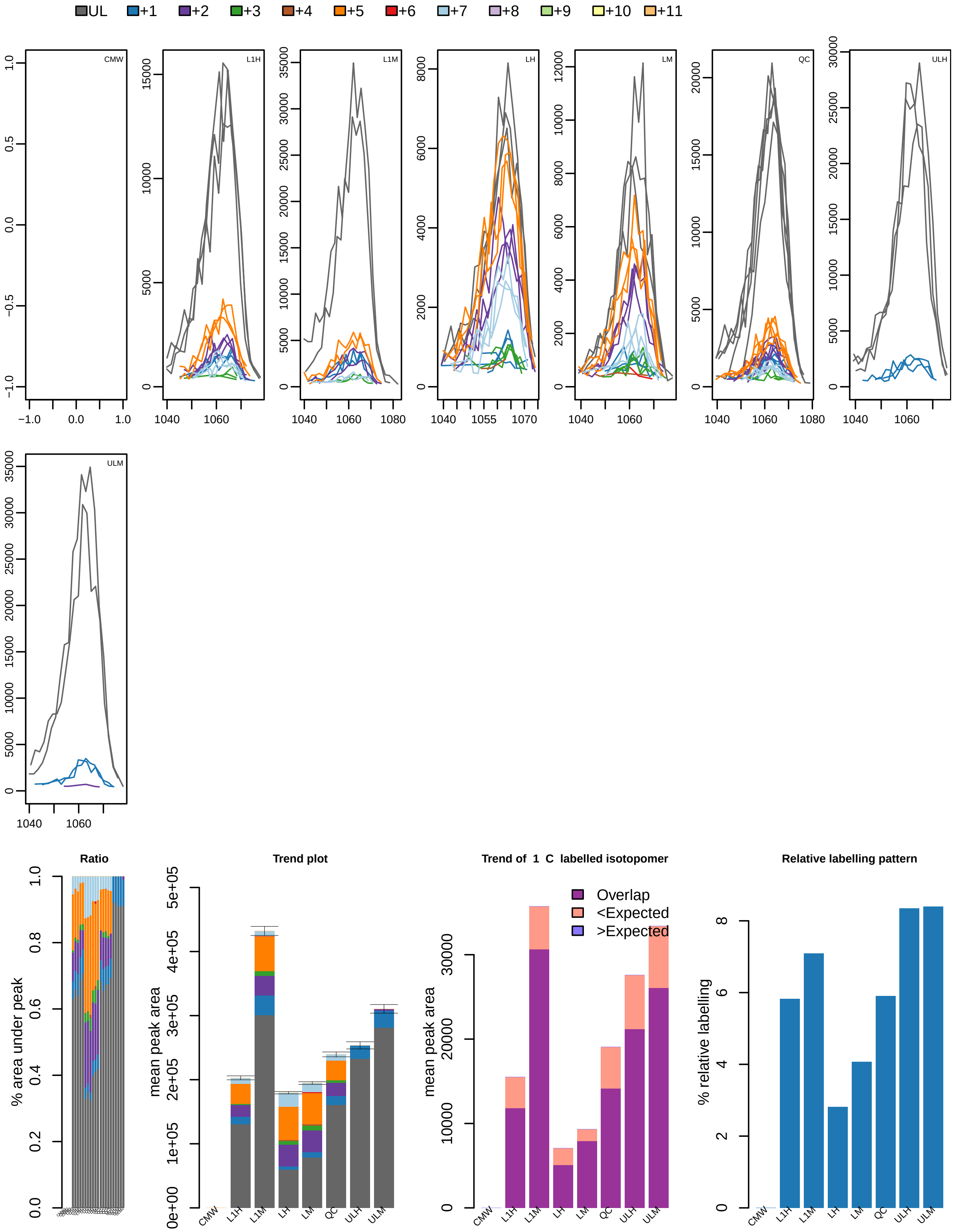

12−OPDA

Formula: $C_{18}H_{28}O_3$ Mass: 292.204 Std.RT: 230.68683312 Ion: NEG

G1

UL +1 +2 +3 +4 +5 +6 +7 +8 +9 +10 +11 +12 +13

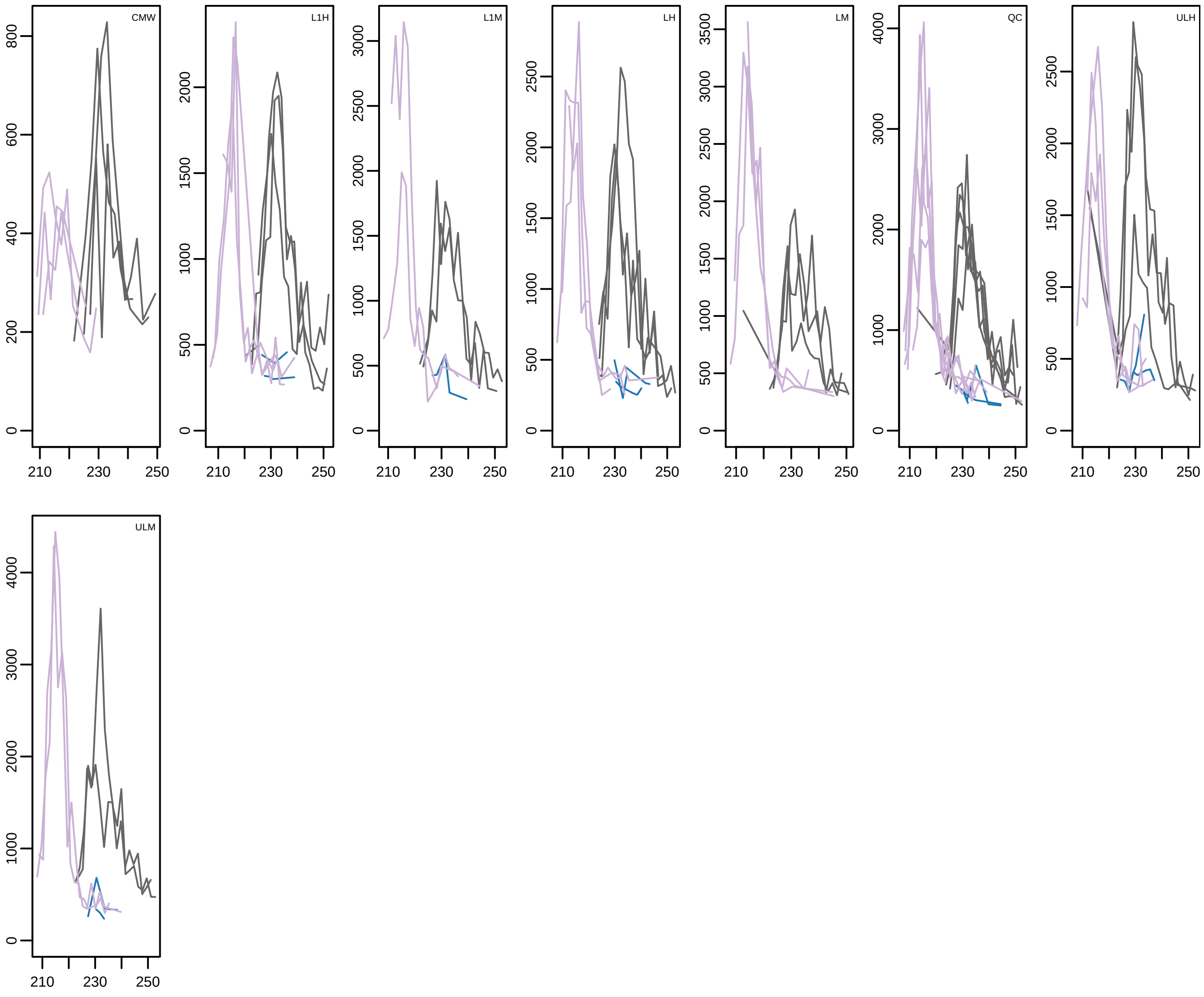

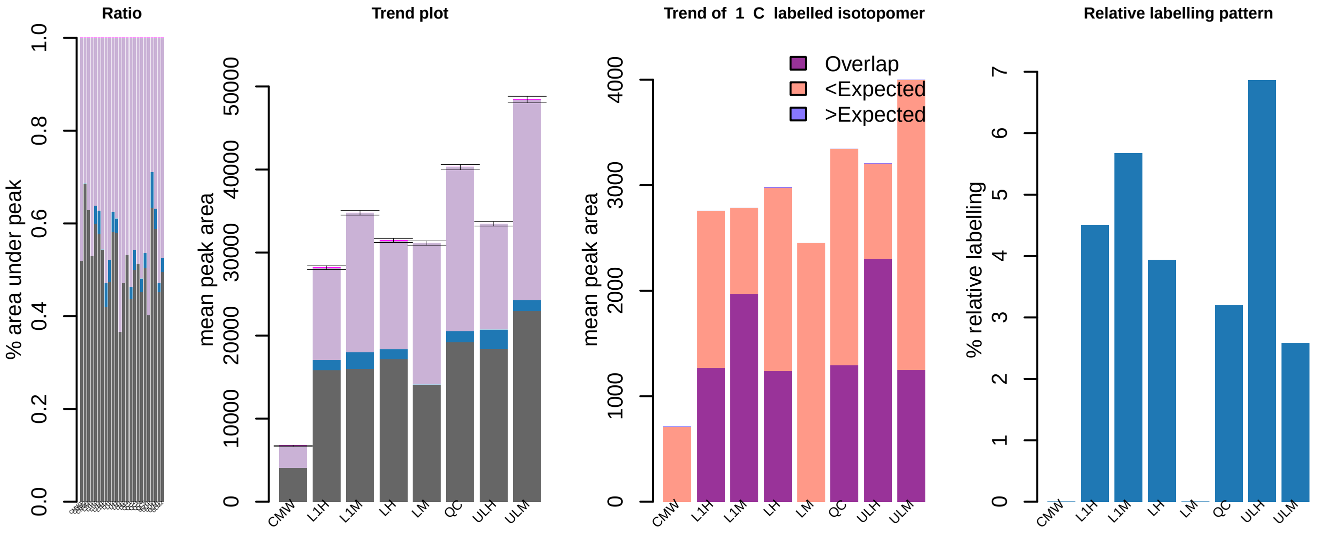

[FA (18:3)] 9Z,12Z,15Z−octadecatrienoic acid

Formula: C18H30O2 Mass: 278.225 Std.RT: 214.21220778 Ion: NEG

G1

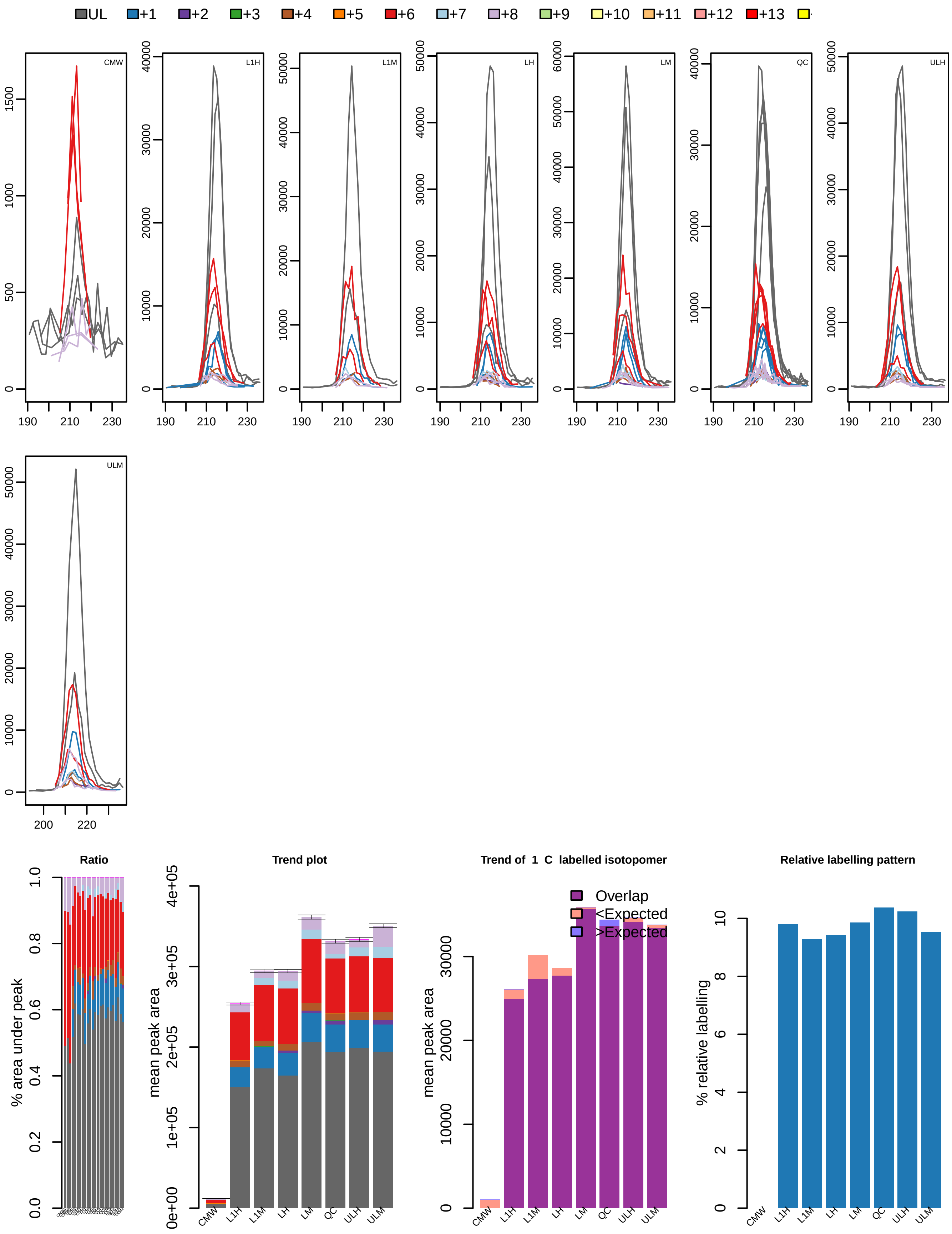

[FA (20:0)] 11Z−eicosenoic acid

Formula: C20H38O2 Mass: 310.287 Std.RT: 211.53029118 Ion: NEG

G1

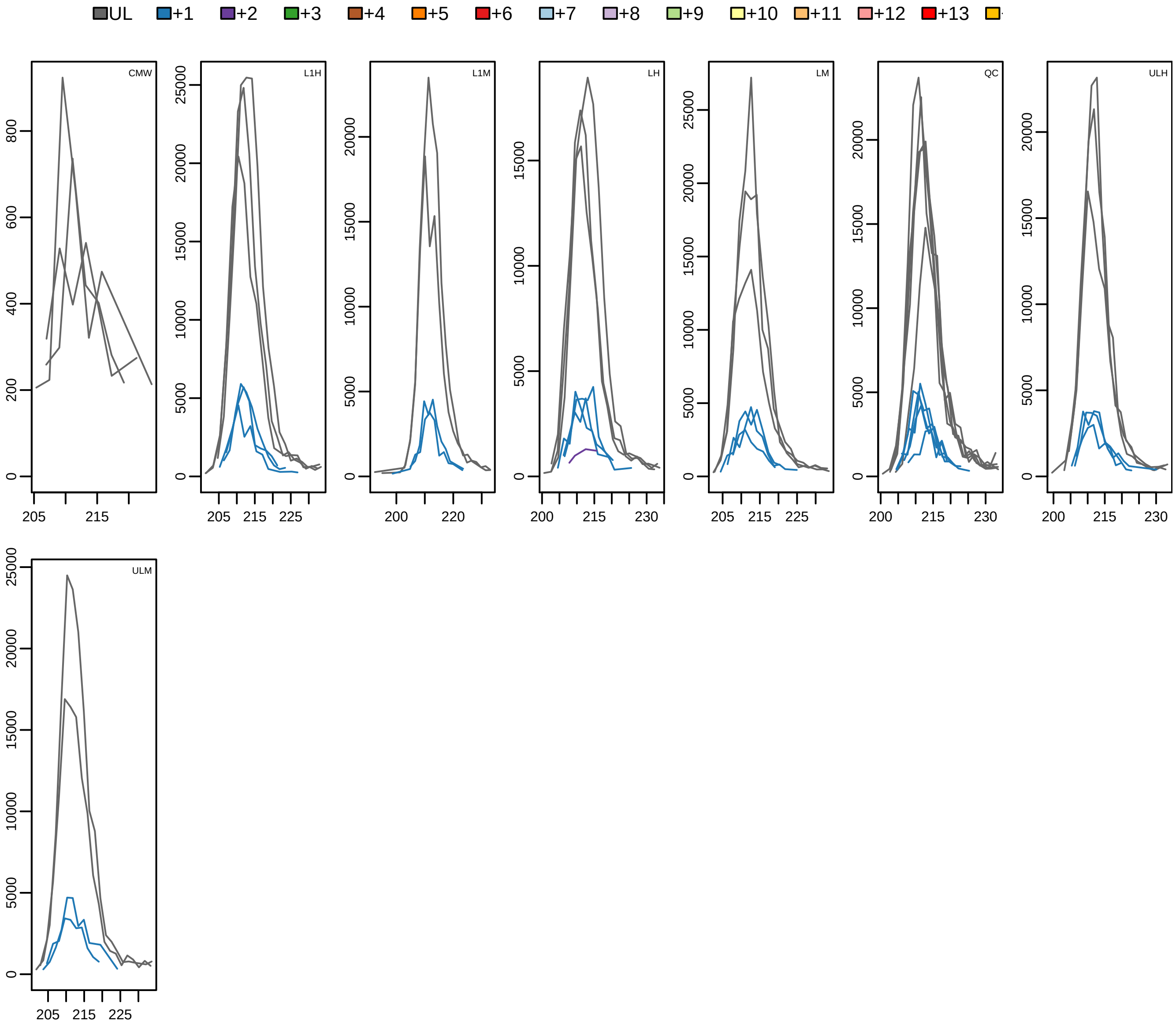

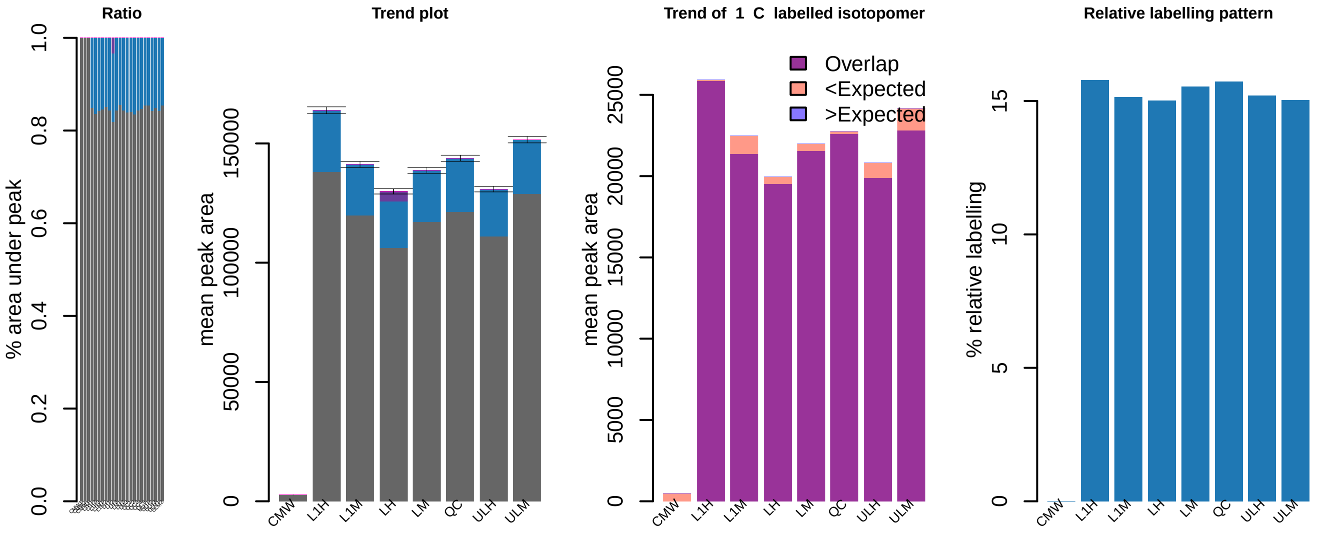

Docosahexaenoicacid

Formula: C22H32O2 Mass: 328.24 Std.RT: 211.1214759 Ion: NEG

G1

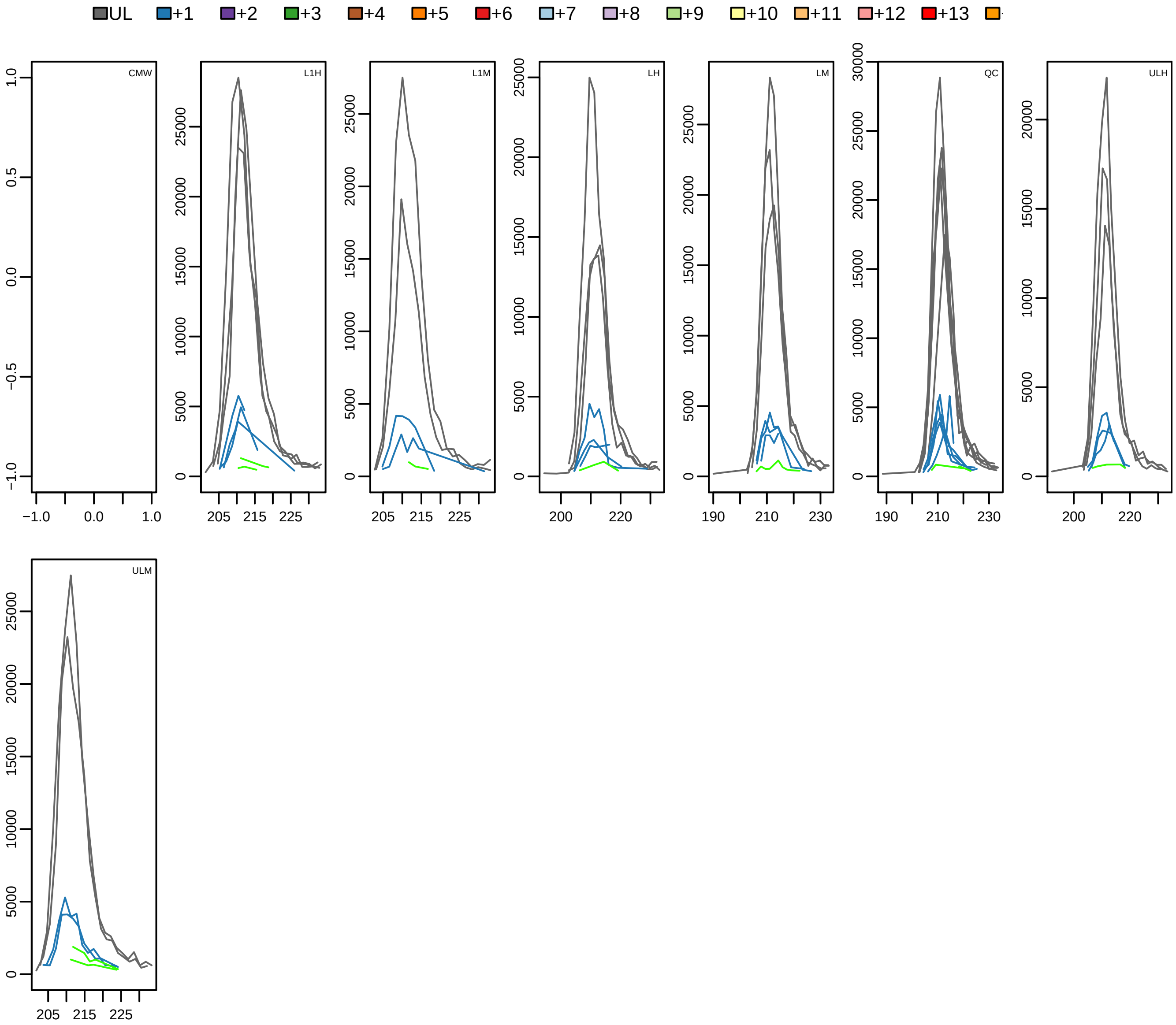

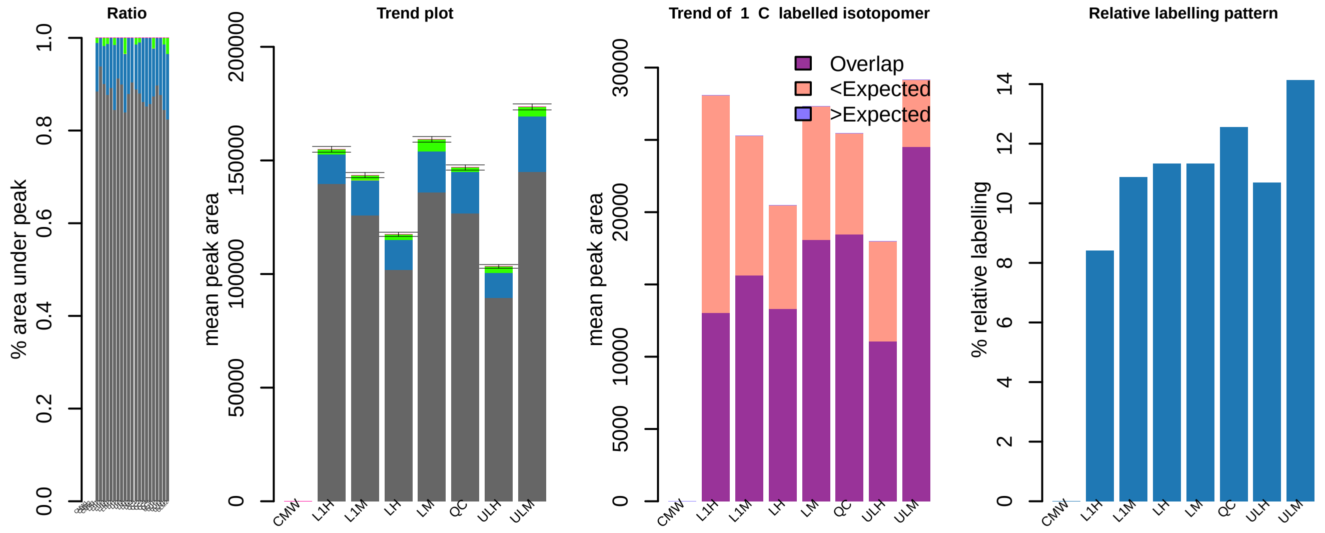

[FA (20:5)] 5Z,8Z,11Z,14Z,17Z−eicosapentaenoic acid

G1

Formula: C20H30O2 Mass: 302.225 Std.RT: 215.61043518 Ion: NEG

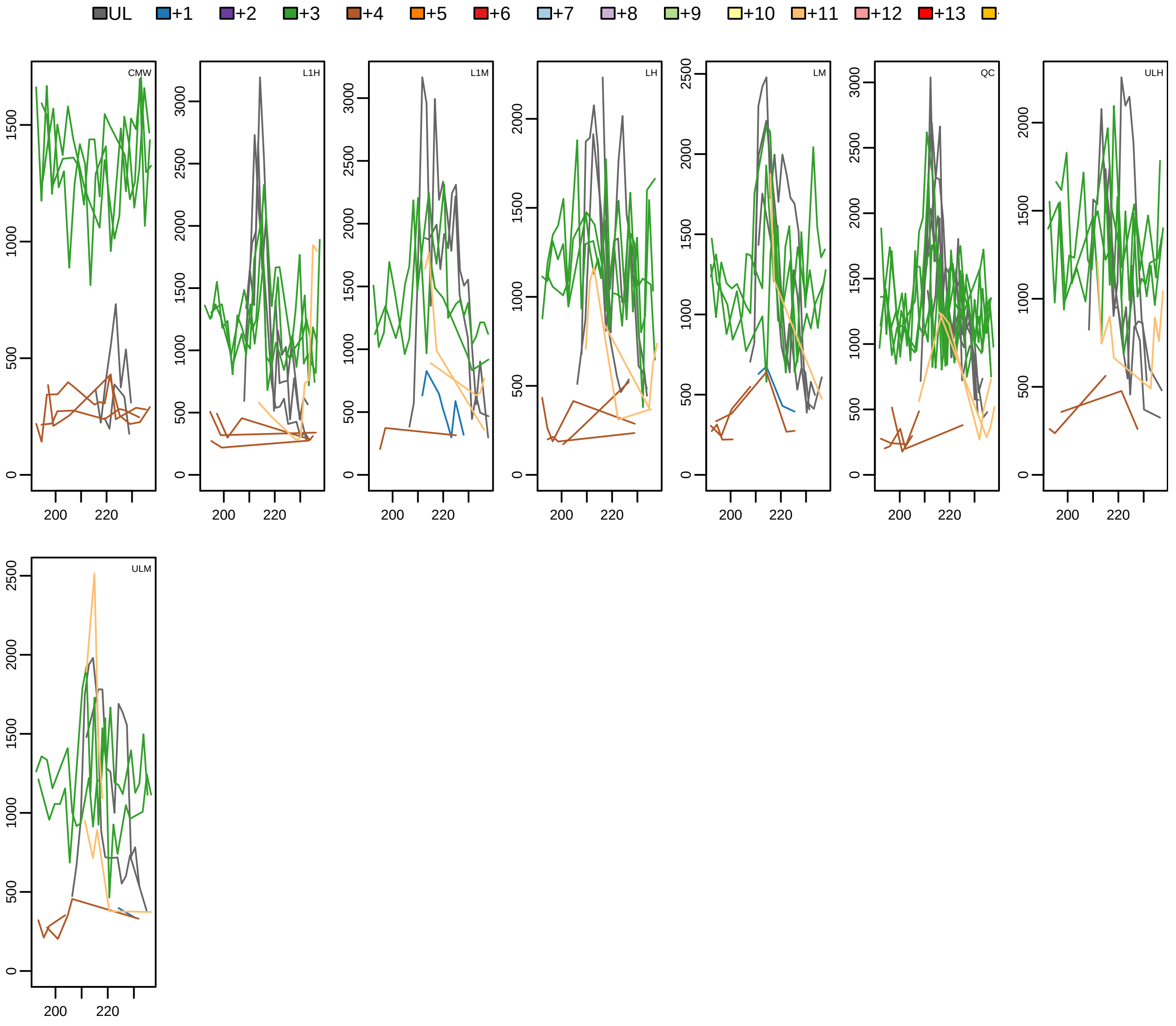

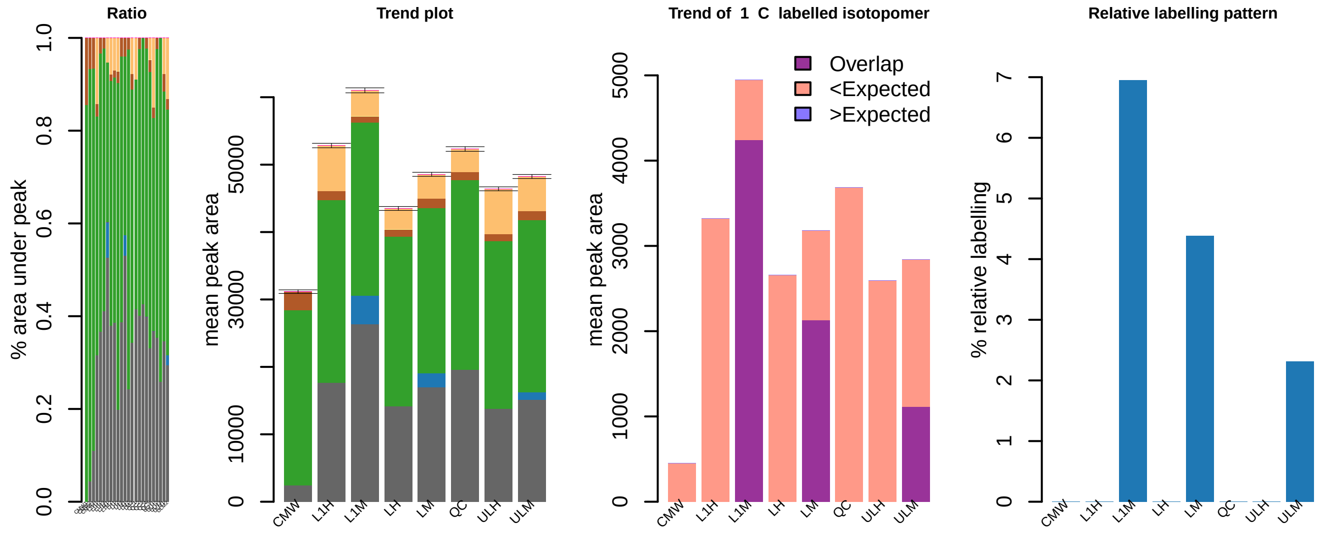

[FA (22:5)] 7Z,10Z,13Z,16Z,19Z−docosapentaenoic aci

Formula: C22H34O2 Mass: 330.256 Std.RT: 211.11042858 Ion: NEG

G1

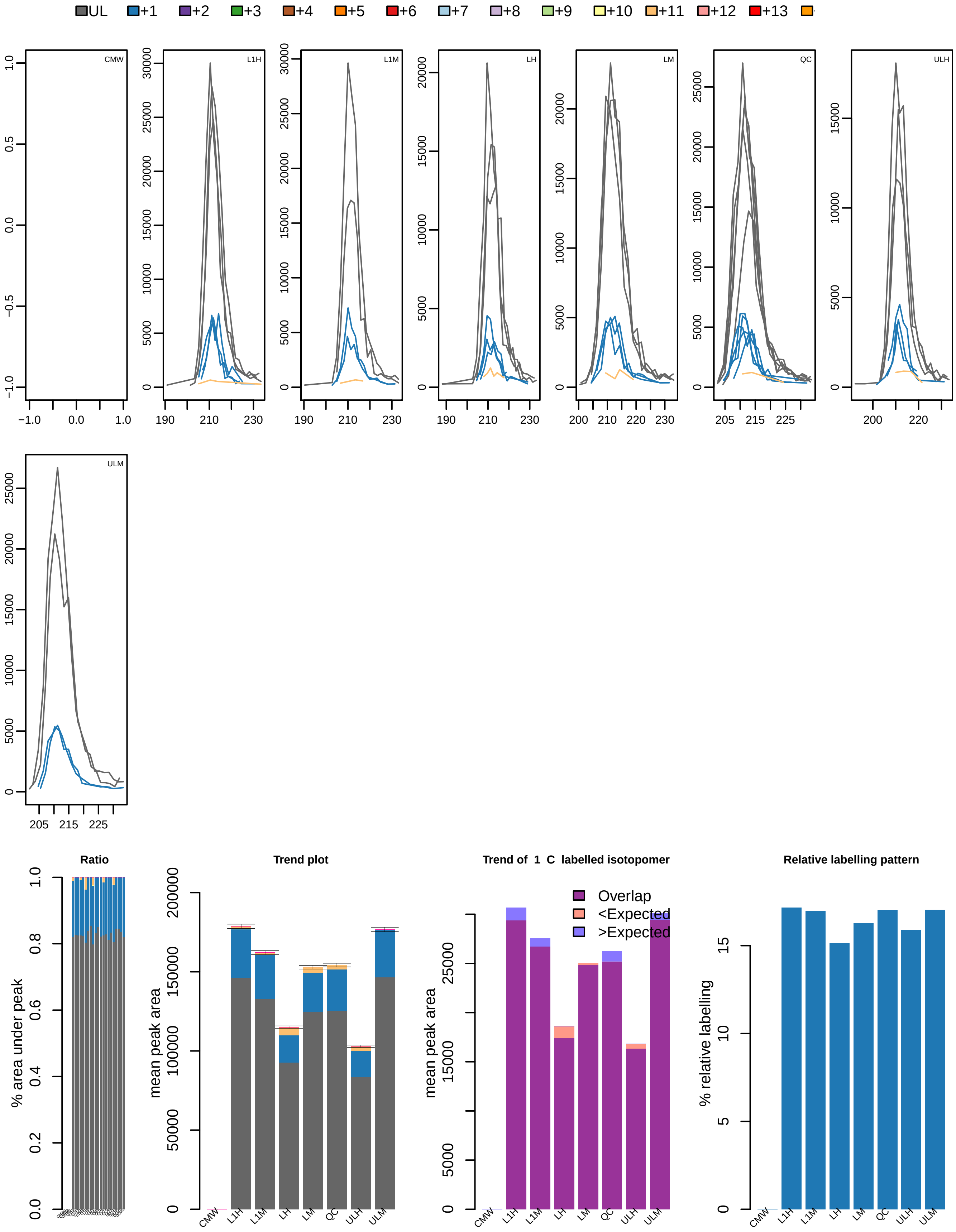

[FA (22:0)] 13Z−docosenoic acid

Formula: C22H42O2 Mass: 338.318 Std.RT: 210.4329522 Ion: NEG

G1

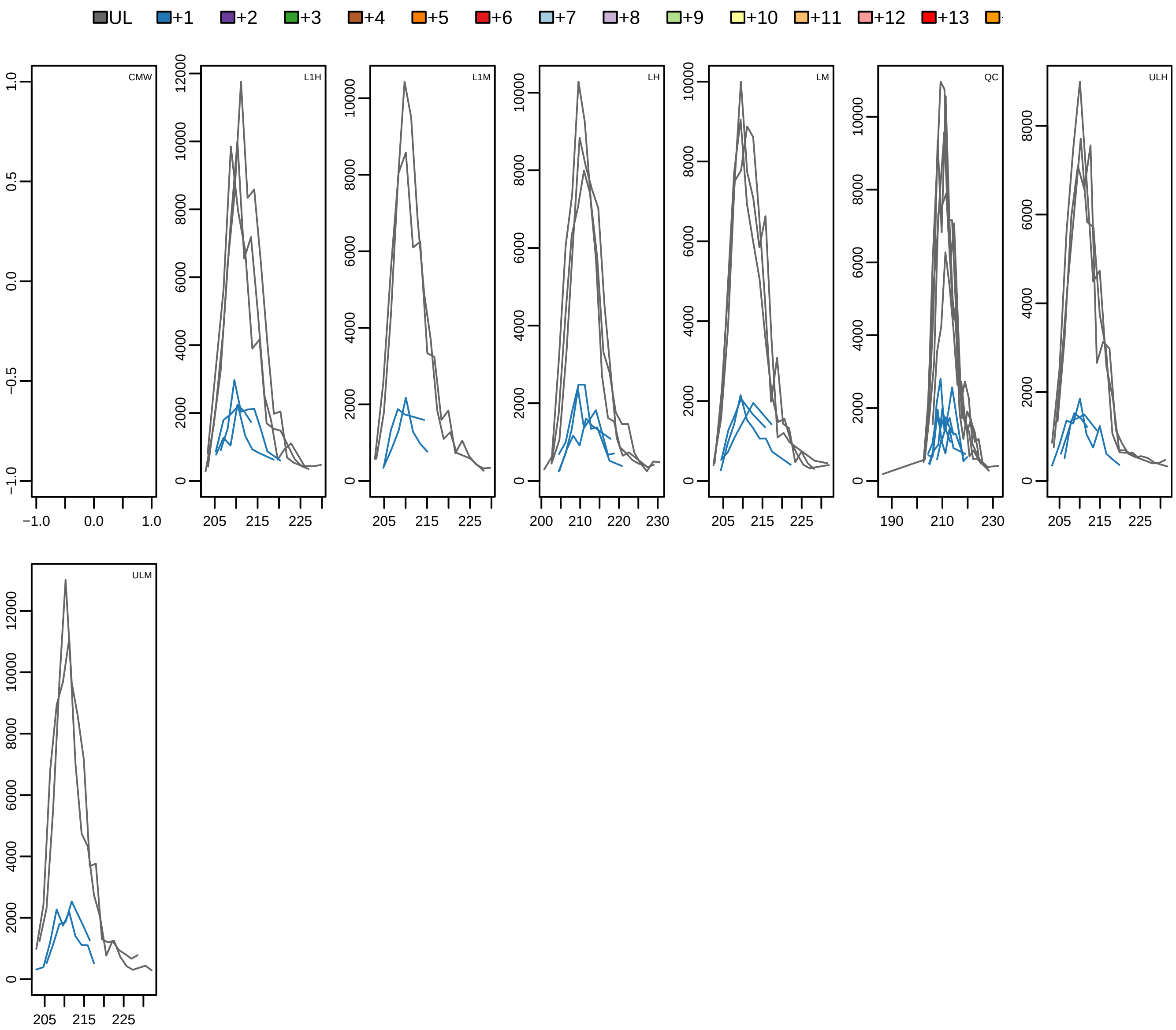

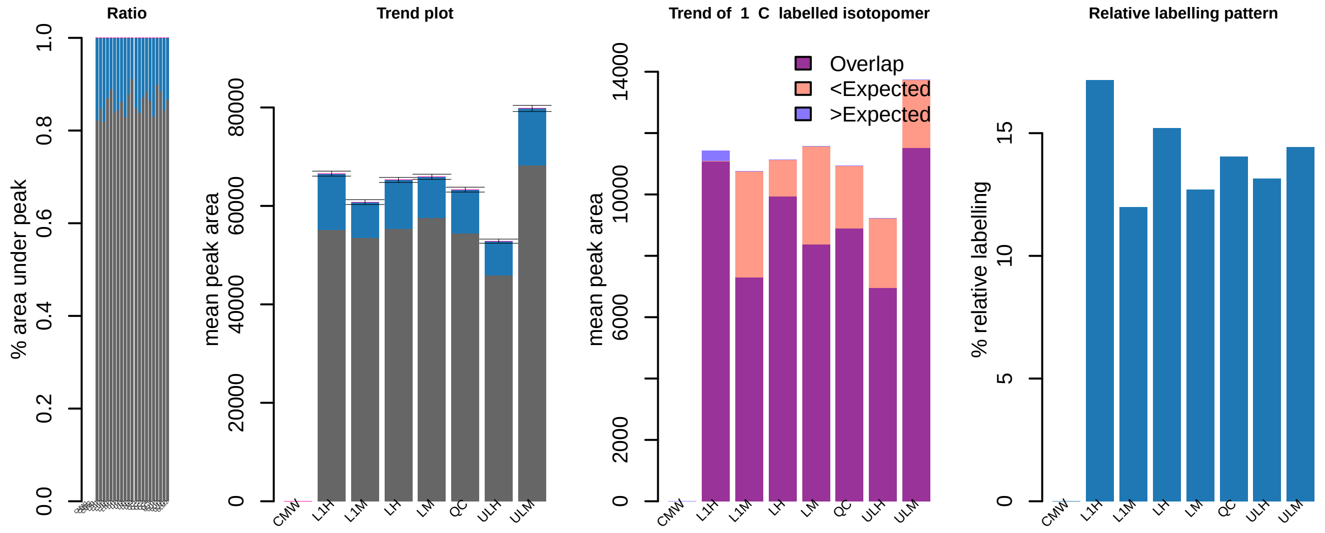

Adipate

Formula: C6H10O4 Mass: 146.058 Std.RT: 890.7314964 Ion: NEG

G1

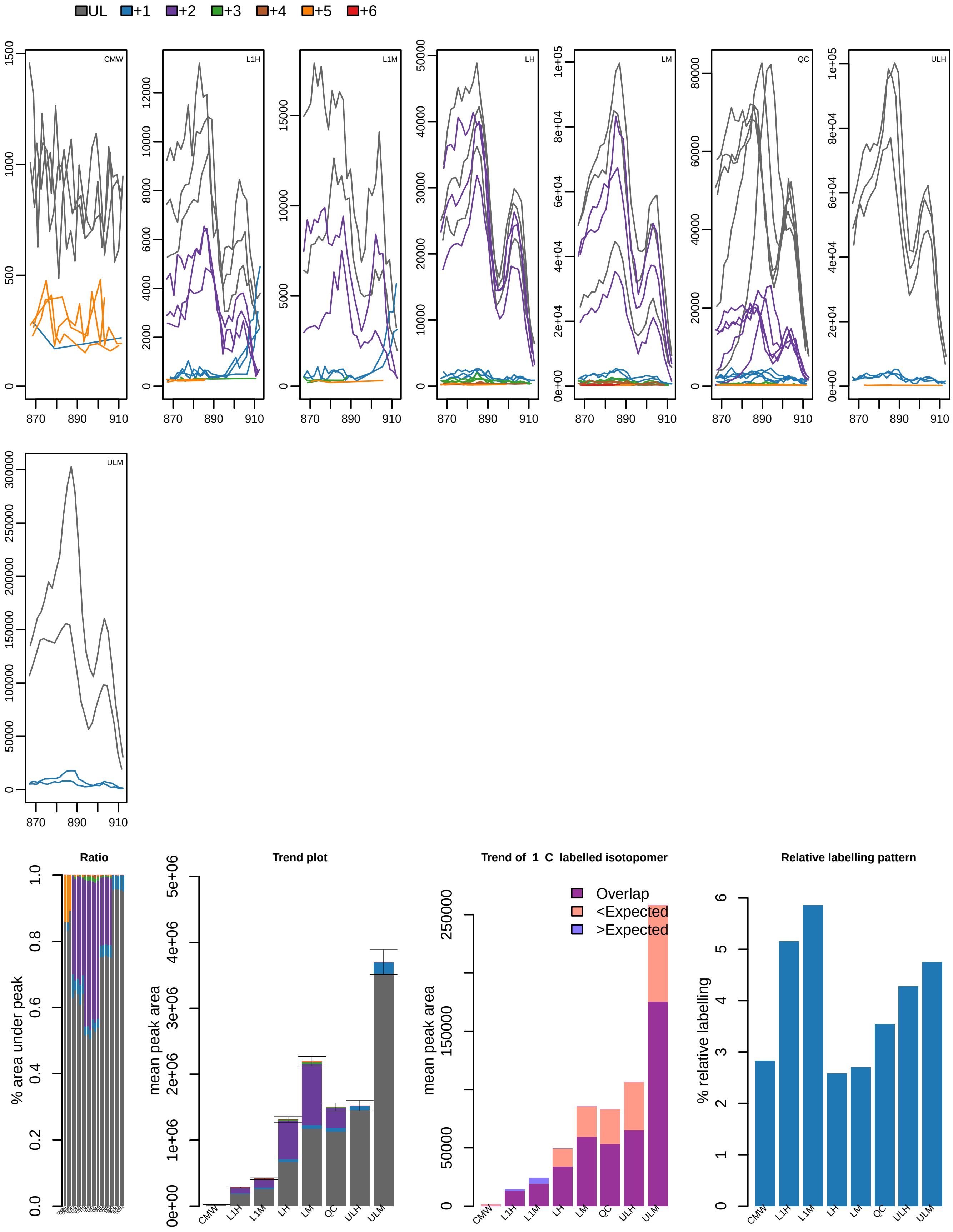

Adipate

Formula: C6H10O4 Mass: 146.058 Std.RT: 890.7314964 Ion: NEG

G2

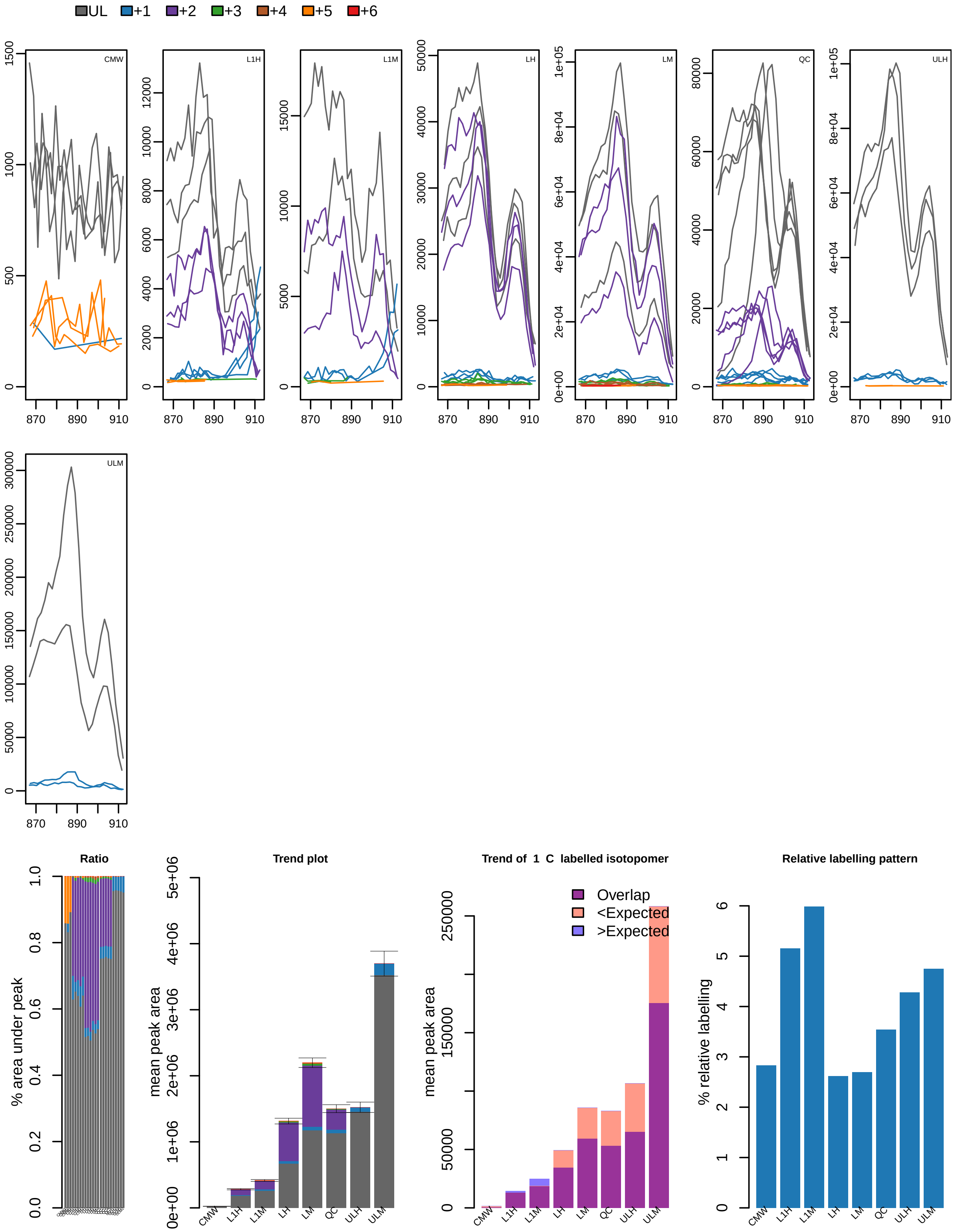

[FA hydroxy,trihydroxy(3:0/2:0)] N−(1,3−dihydroxypropa

Formula: C23H41NO6 Mass: 427.293 Std.RT: 465.1741257 Ion: NEC

G1

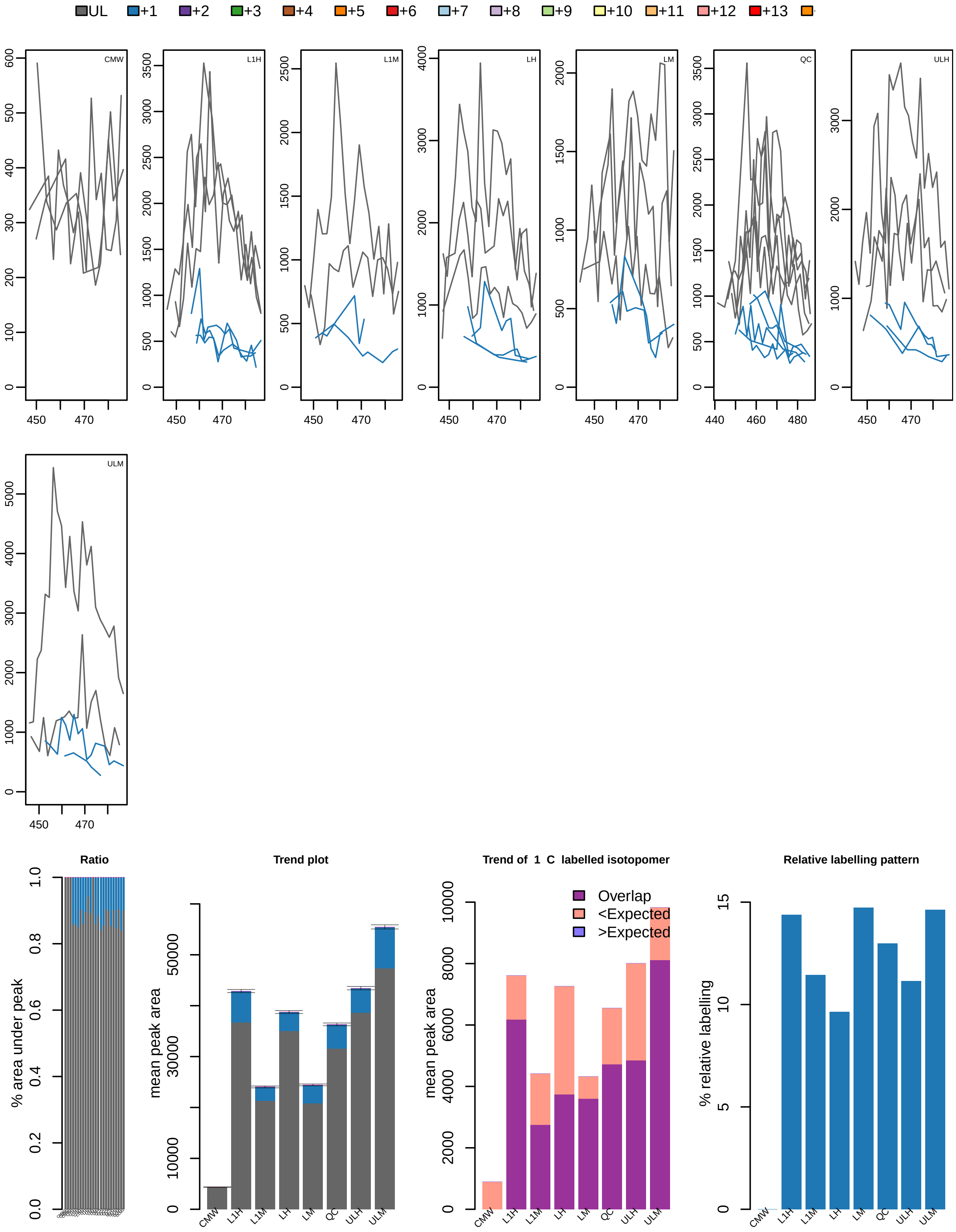

[FA (18:1)] 9Z−octadecenoic acid

Formula: C18H34O2 Mass: 282.256 Std.RT: 213.21608478 Ion: NEG

G1

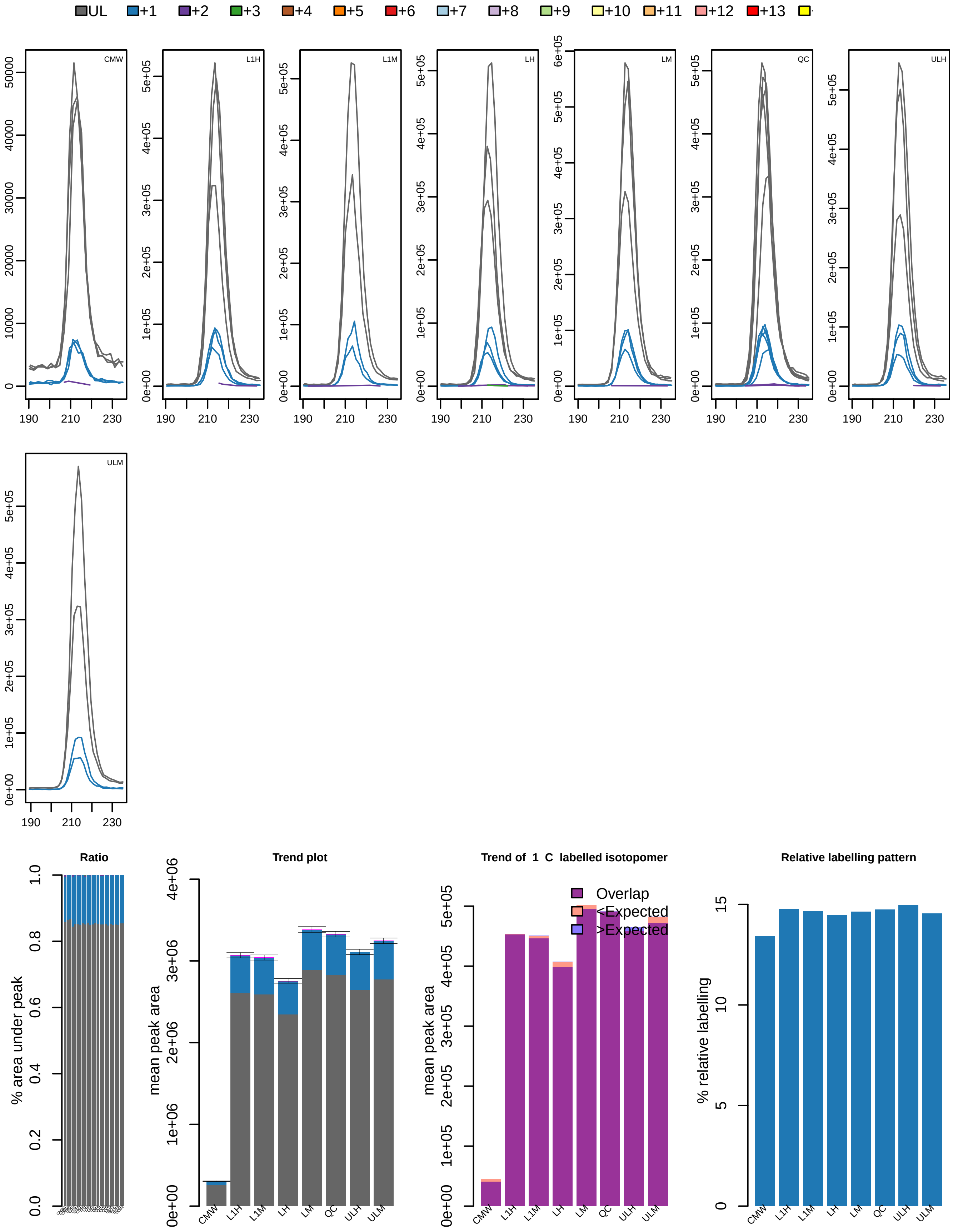

[FA trihydroxy(4:0)] 2,2,4−trihydroxy−butanoic acid

G1

Formula: C4H8O5 Mass: 136.037 Std.RT: 642.2094984 Ion: NEG

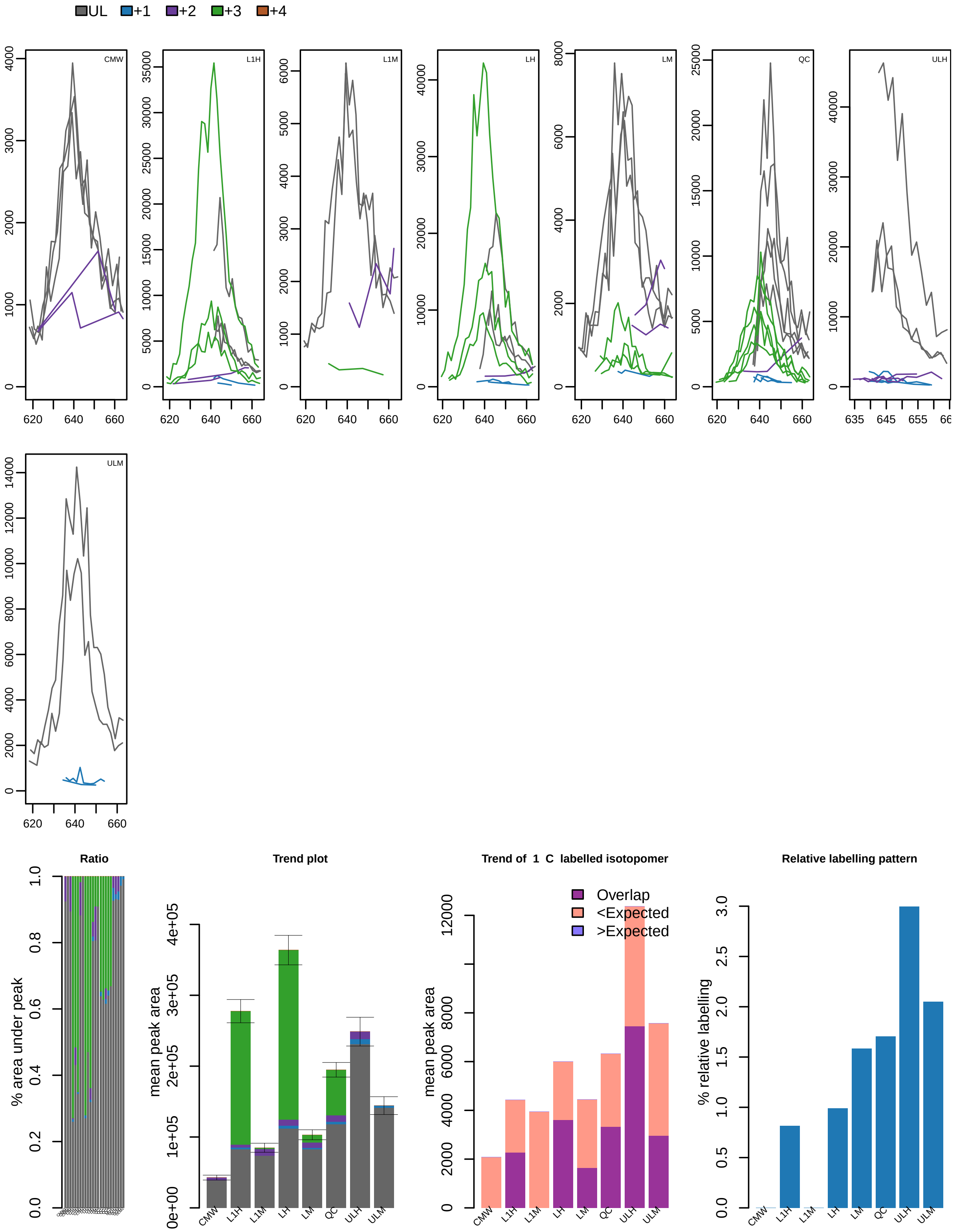

[FA dimethyl(13:0)] 2,5−dimethyl−2E−tridecenoic acid

G1

Formula: $C_{15}H_{28}O_2$ Mass: 240.209 Std.RT: 216.46937502 Ion: NEG

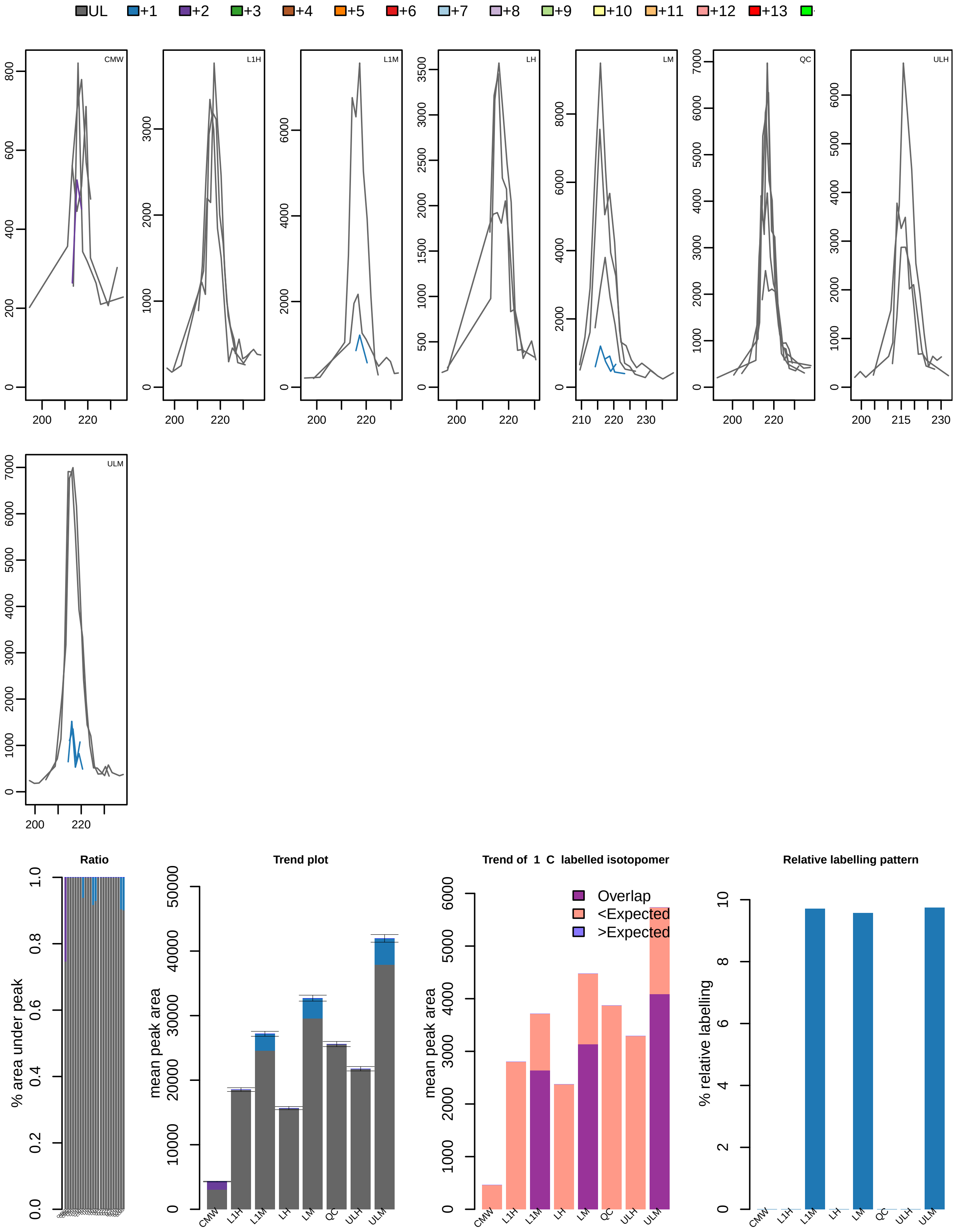

2S−Hydroxytetradecanoic acid

Formula: C14H28O3 Mass: 244.204 Std.RT: 217.6950003 Ion: NEG

G1

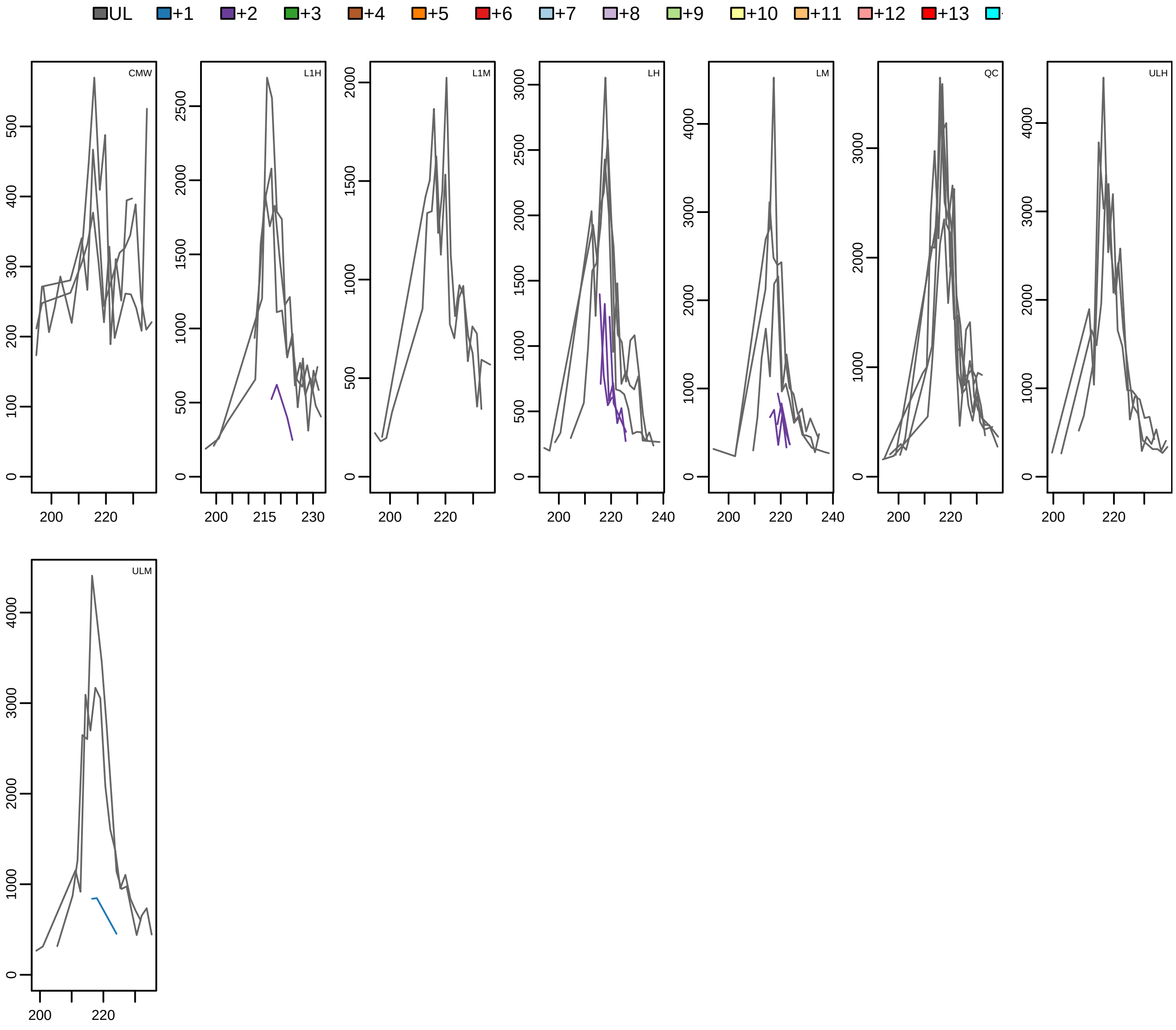

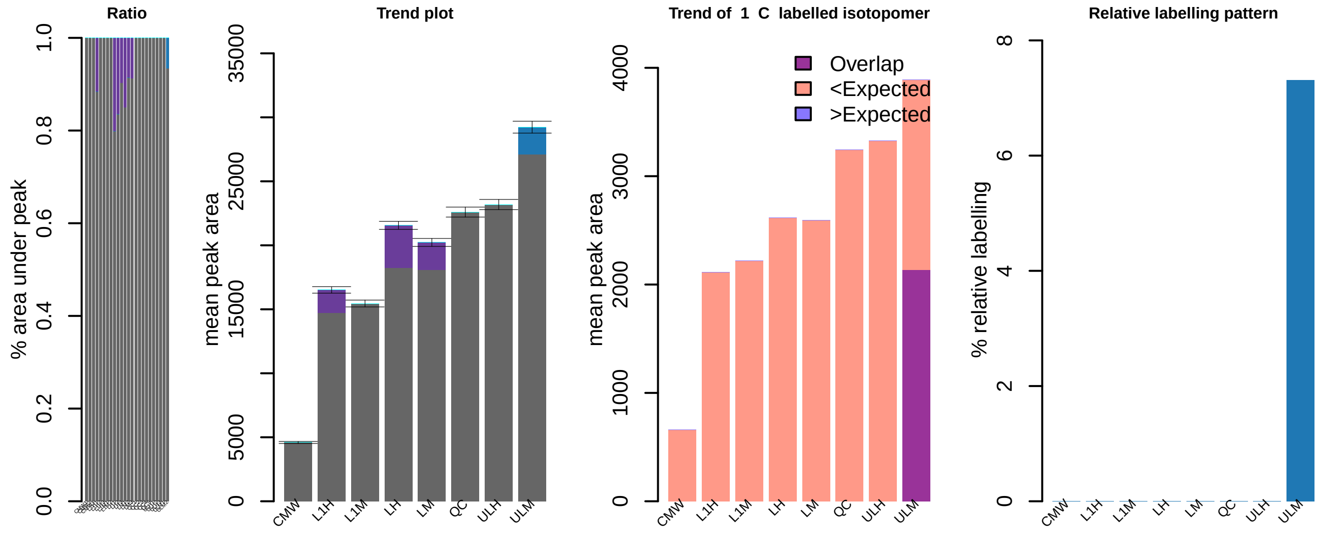

12−Hydroxydodecanoic acid

Formula: C12H24O3 Mass: 216.173 Std.RT: 224.5535844 Ion: NEG

G1

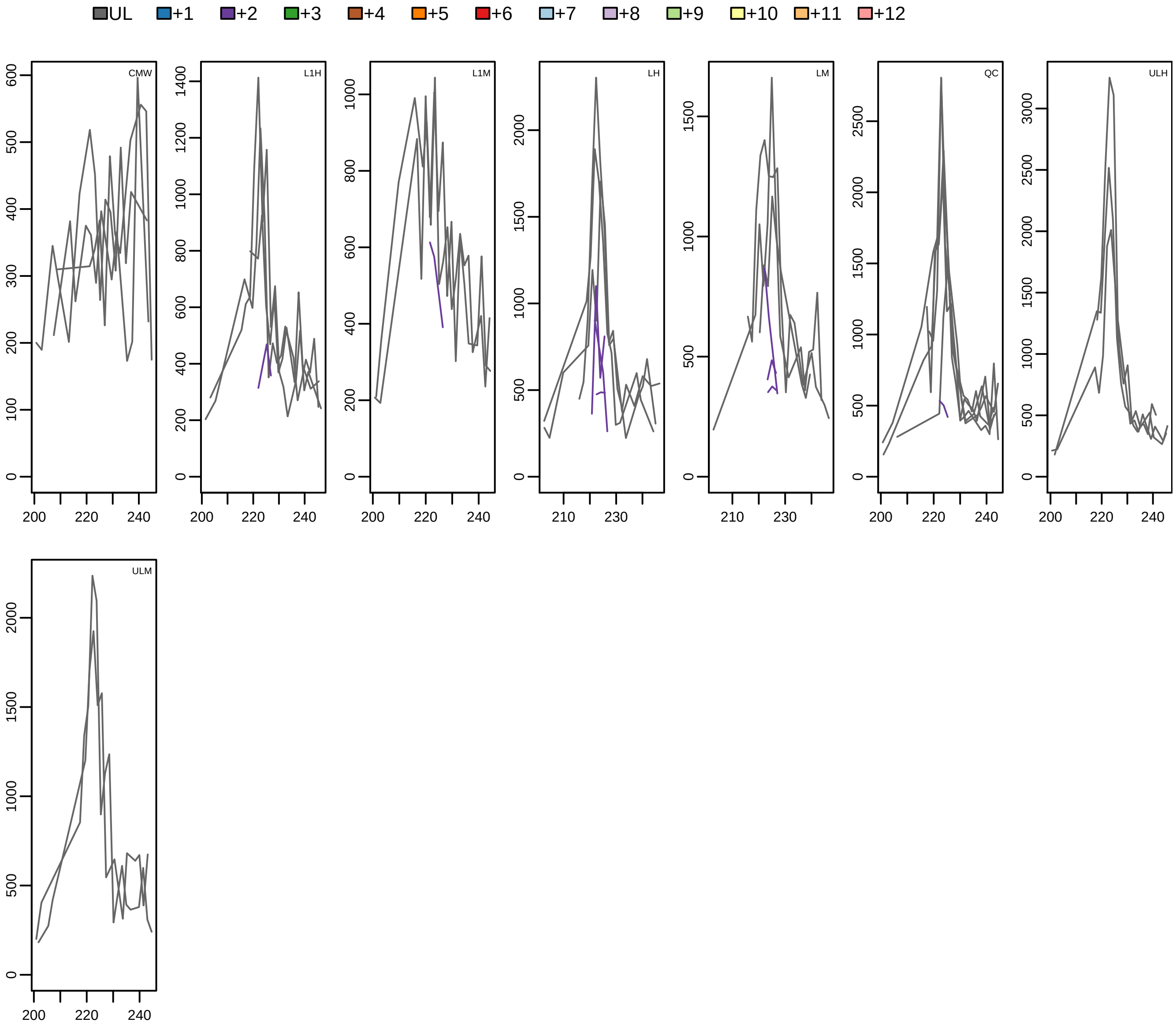

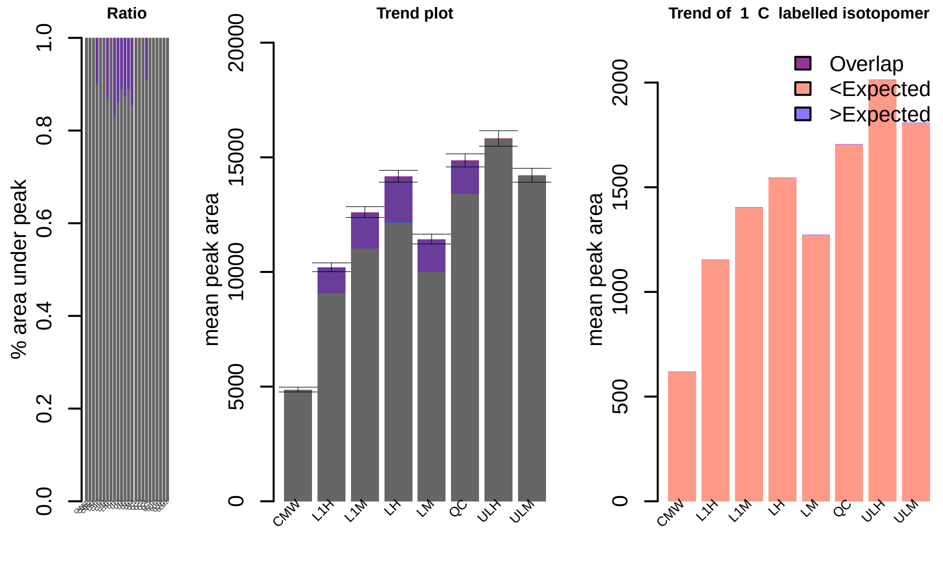

(9Z)−Tetradecenoic acid

Formula: $C_{14}H_{26}O_2$ Mass: 226.193 Std.RT: 218.37366738 Ion: NEG

G1

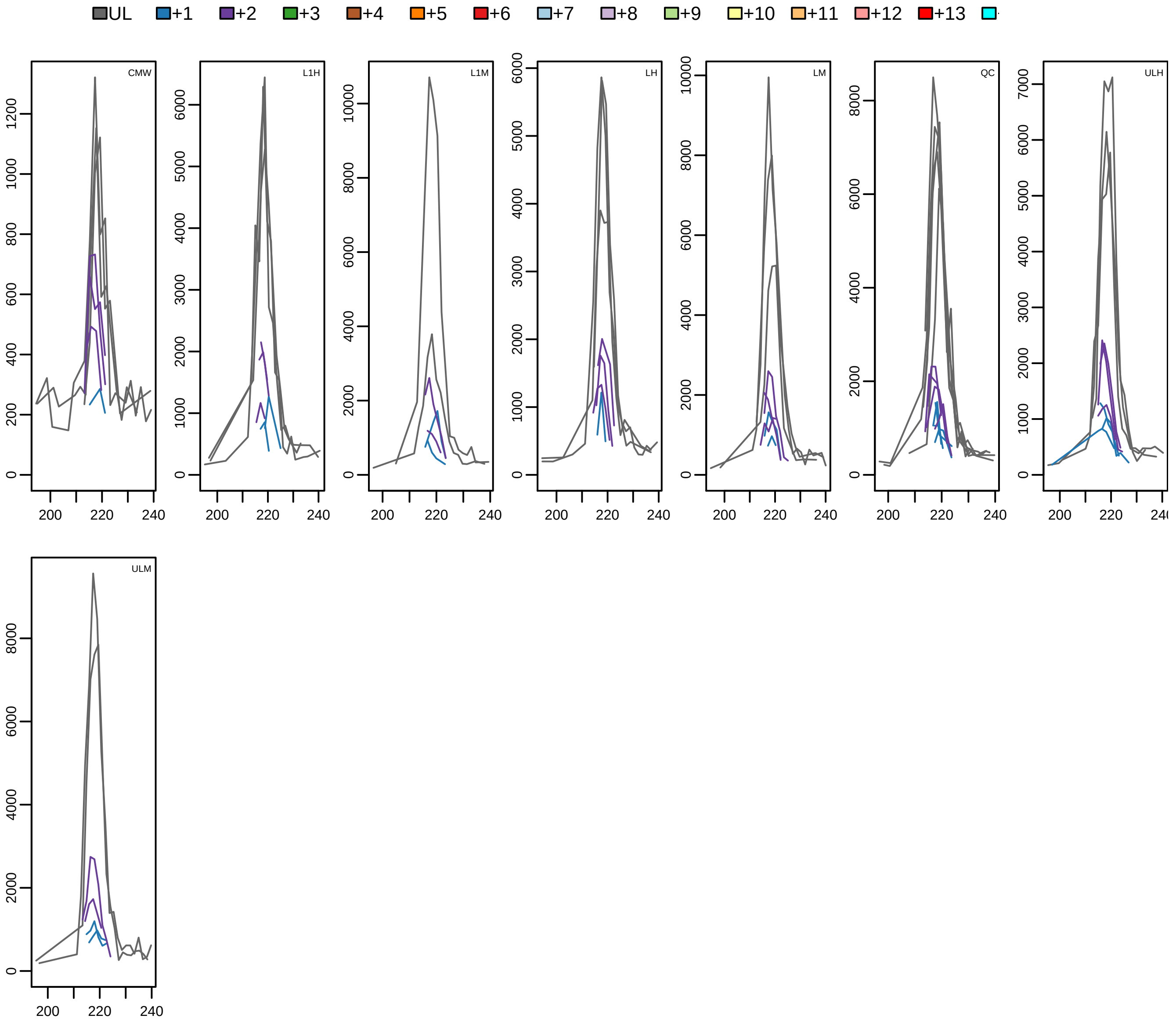

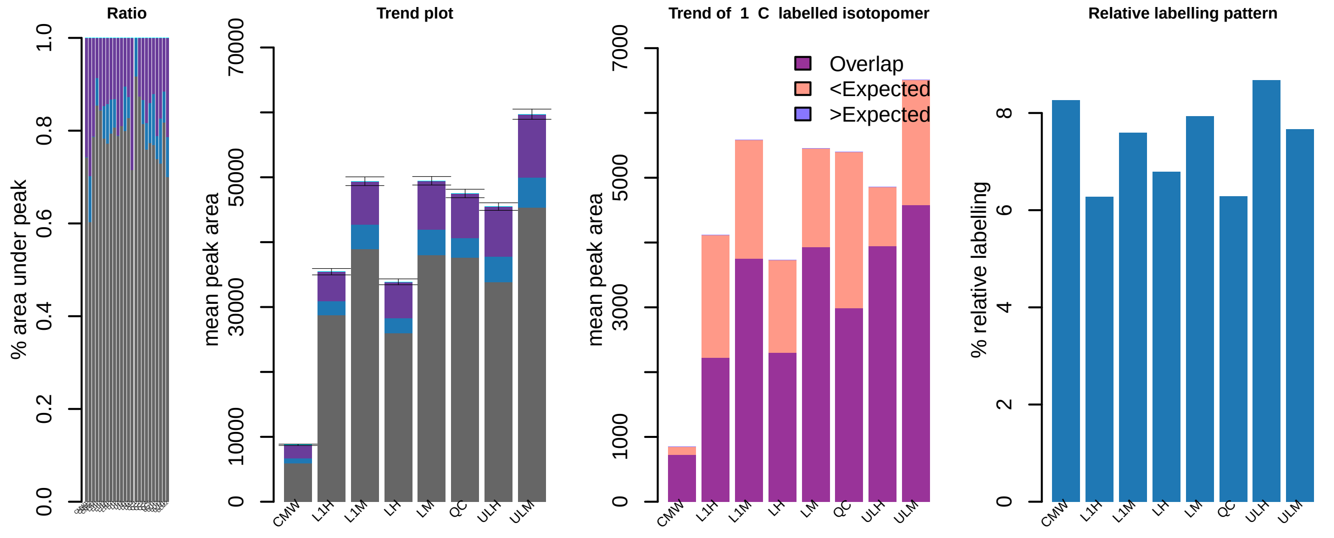

13,16,19−Docosatrienoic acid

Formula: $C_{22}H_{38}O_2$ Mass: 334.287 Std.RT: 210.97061958 Ion: NEG

G1

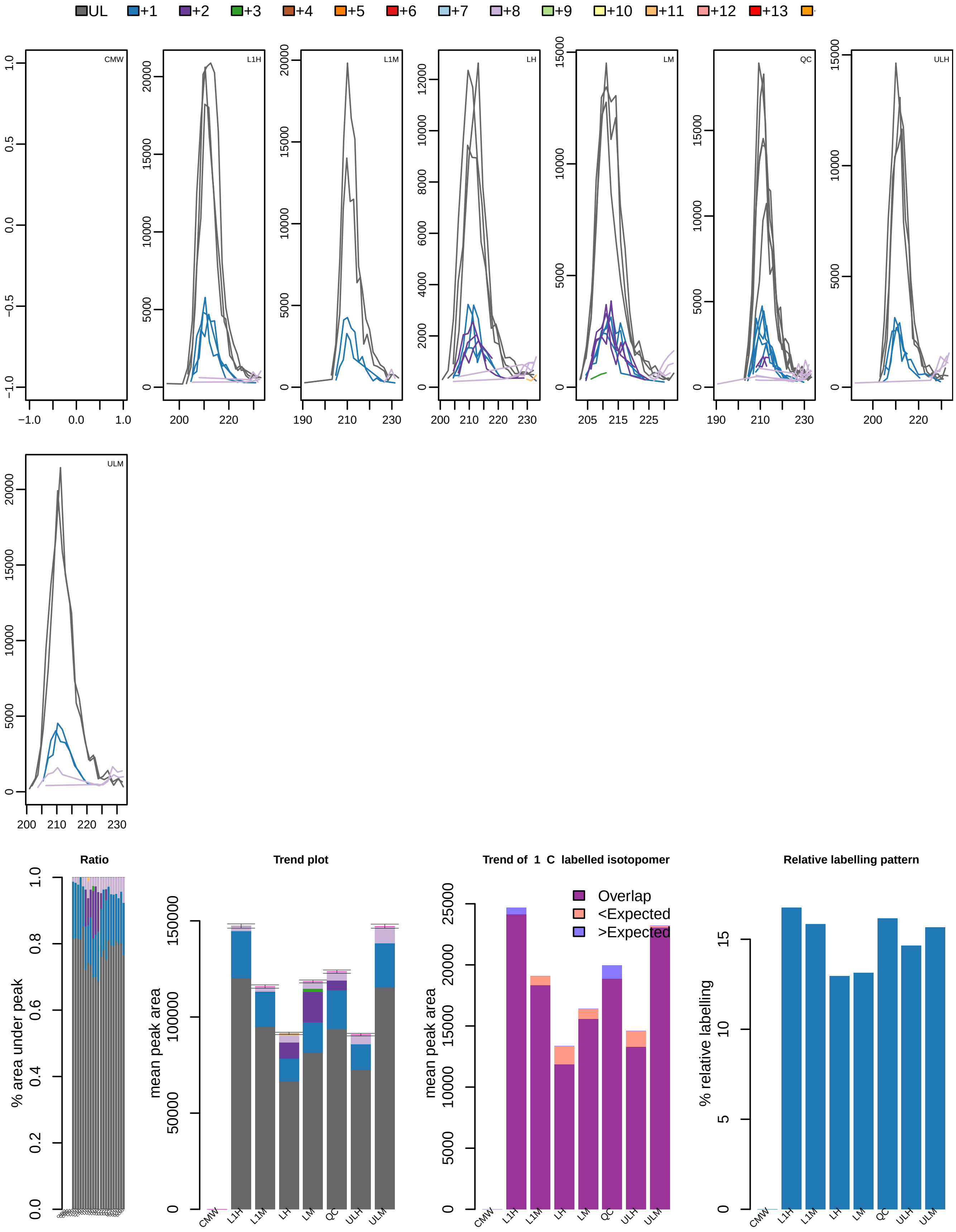

[FA hydroxy(17:2)] 7−hydroxy−10E,16−heptadecadien−

Formula: C17H26O3 Mass: 278.188 Std.RT: 218.43391668 Ion: NEG

G1

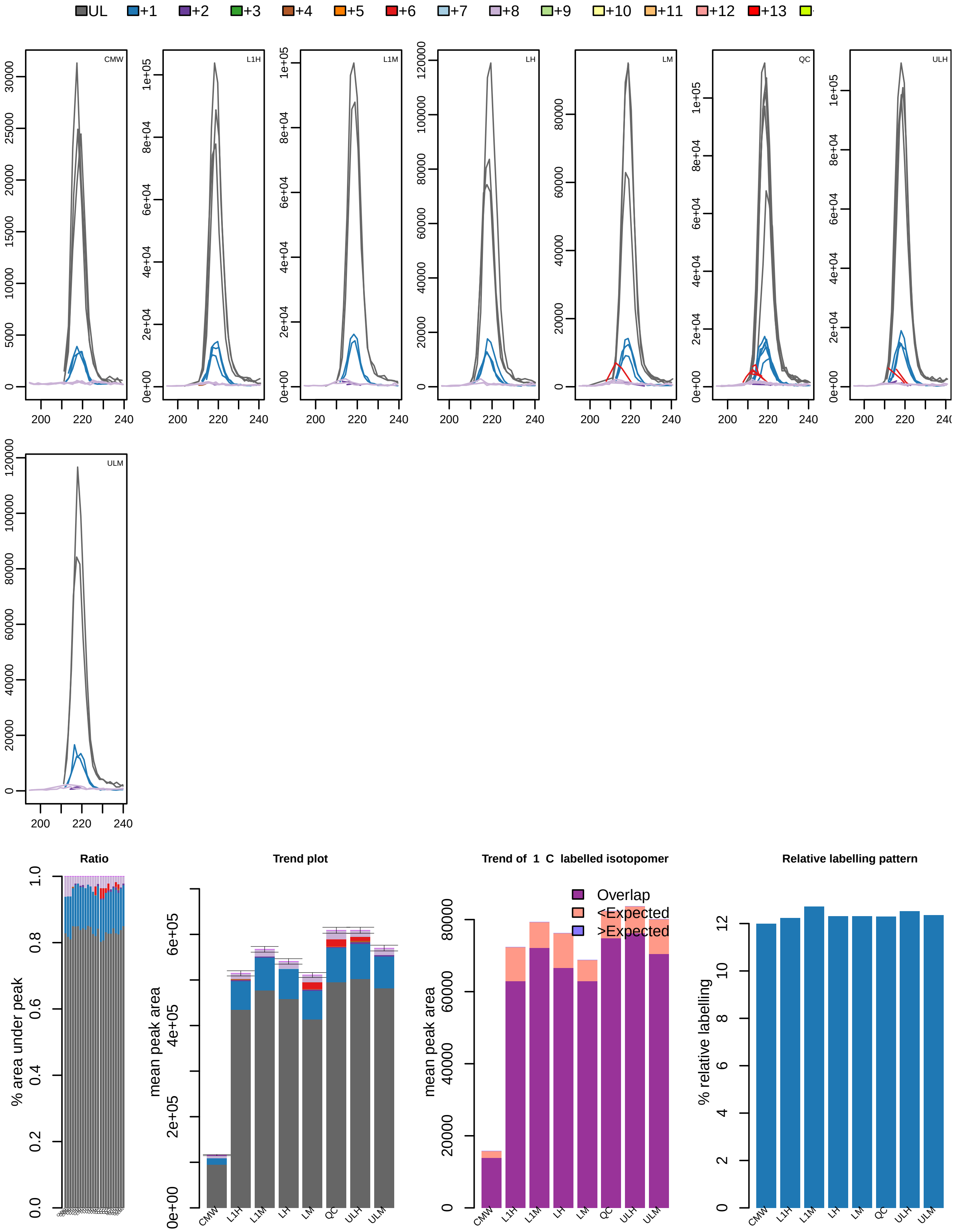

[FA (22:4)] 7Z,10Z,13Z,16Z−docosatetraenoic acid

Formula: C22H36O2 Mass: 332.272 Std.RT: 211.03933278 Ion: NEG

G1

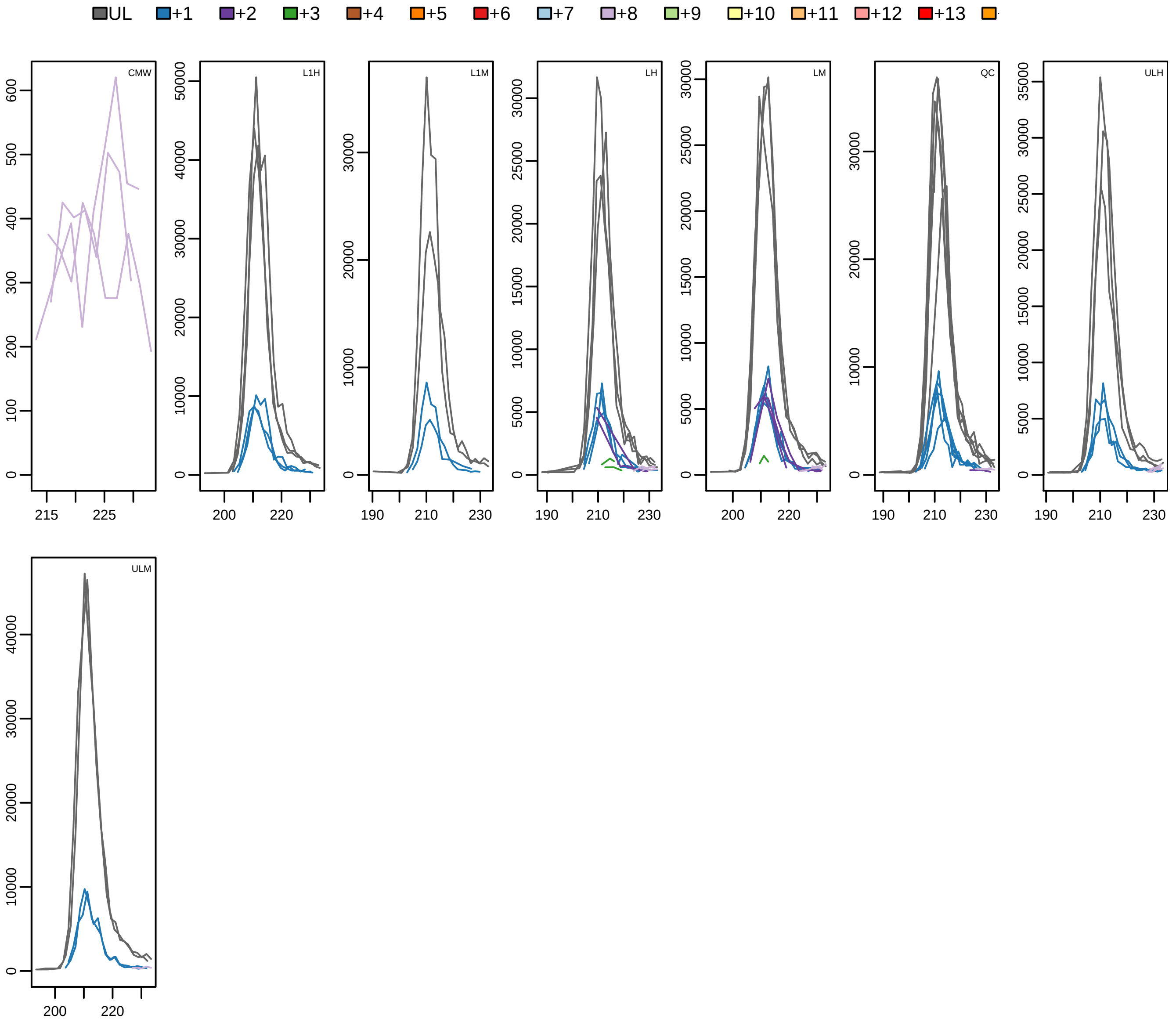

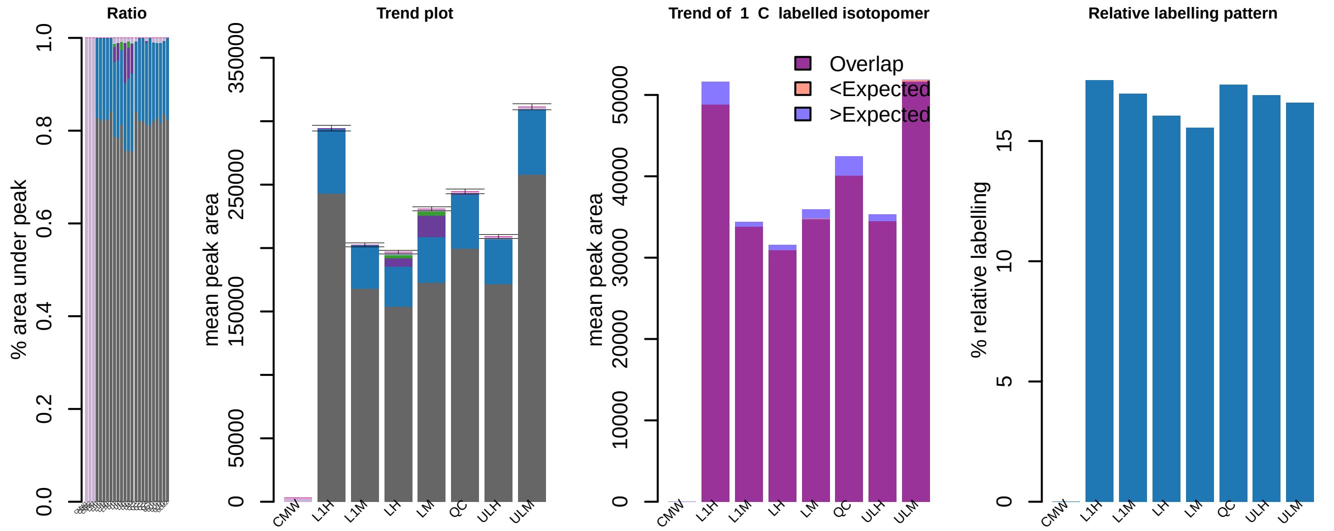

[FA hydroxy(18:0)] 9,10−dihydroxy−octadecanoic acid

Formula: C18H36O4 Mass: 316.261 Std.RT: 223.9164168 Ion: NEG

G1

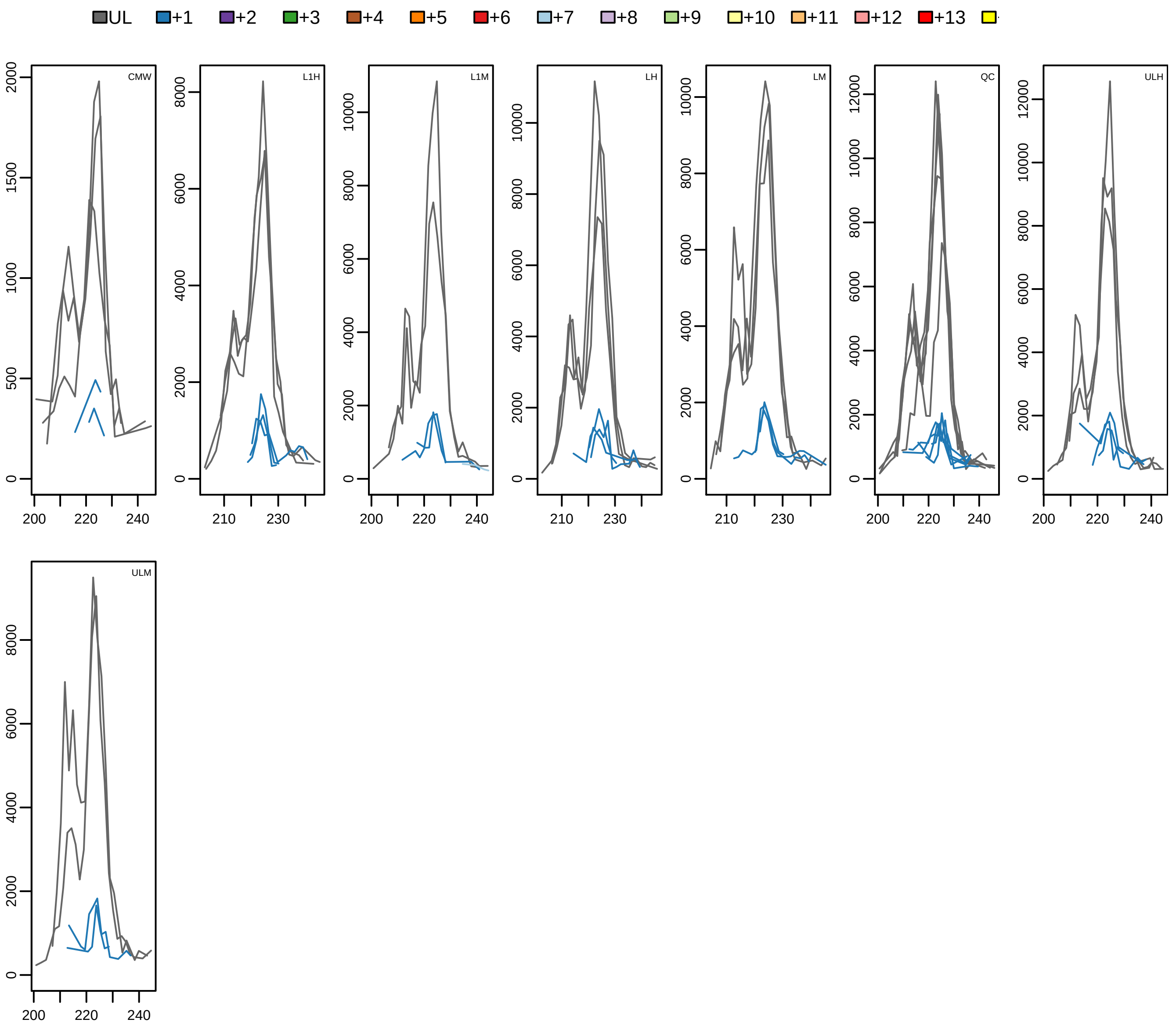

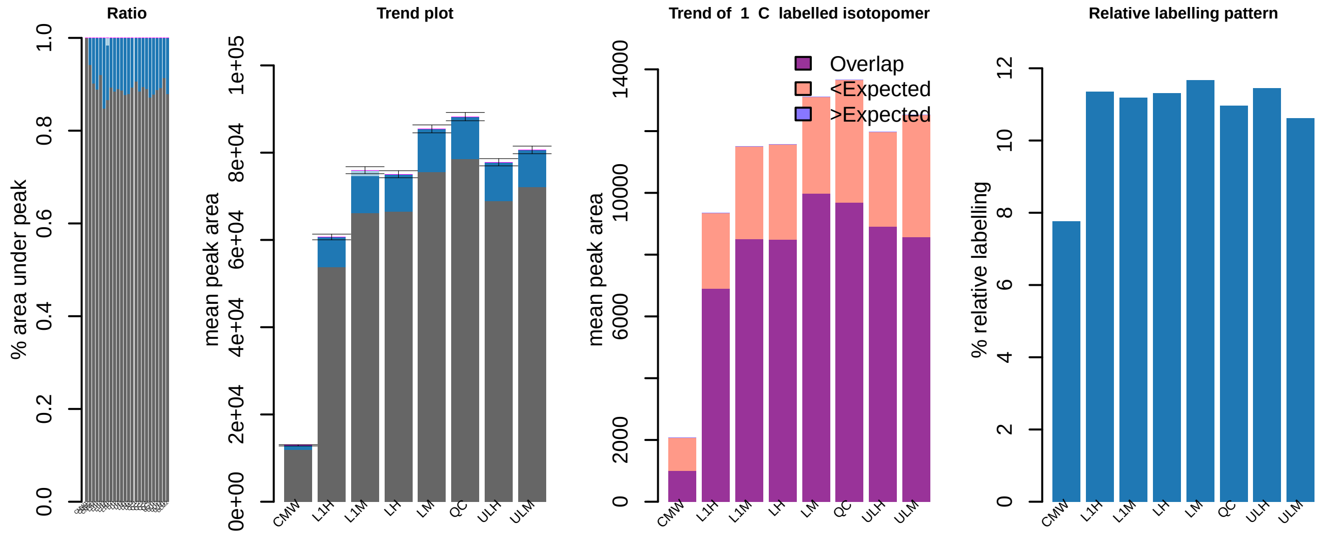

[FA (24:6)] 6,9,12,15,18,21−Tetracosahexaynoic acid

Formula: C24H24O2 Mass: 344.178 Std.RT: 235.8086664 Ion: NEG

G1

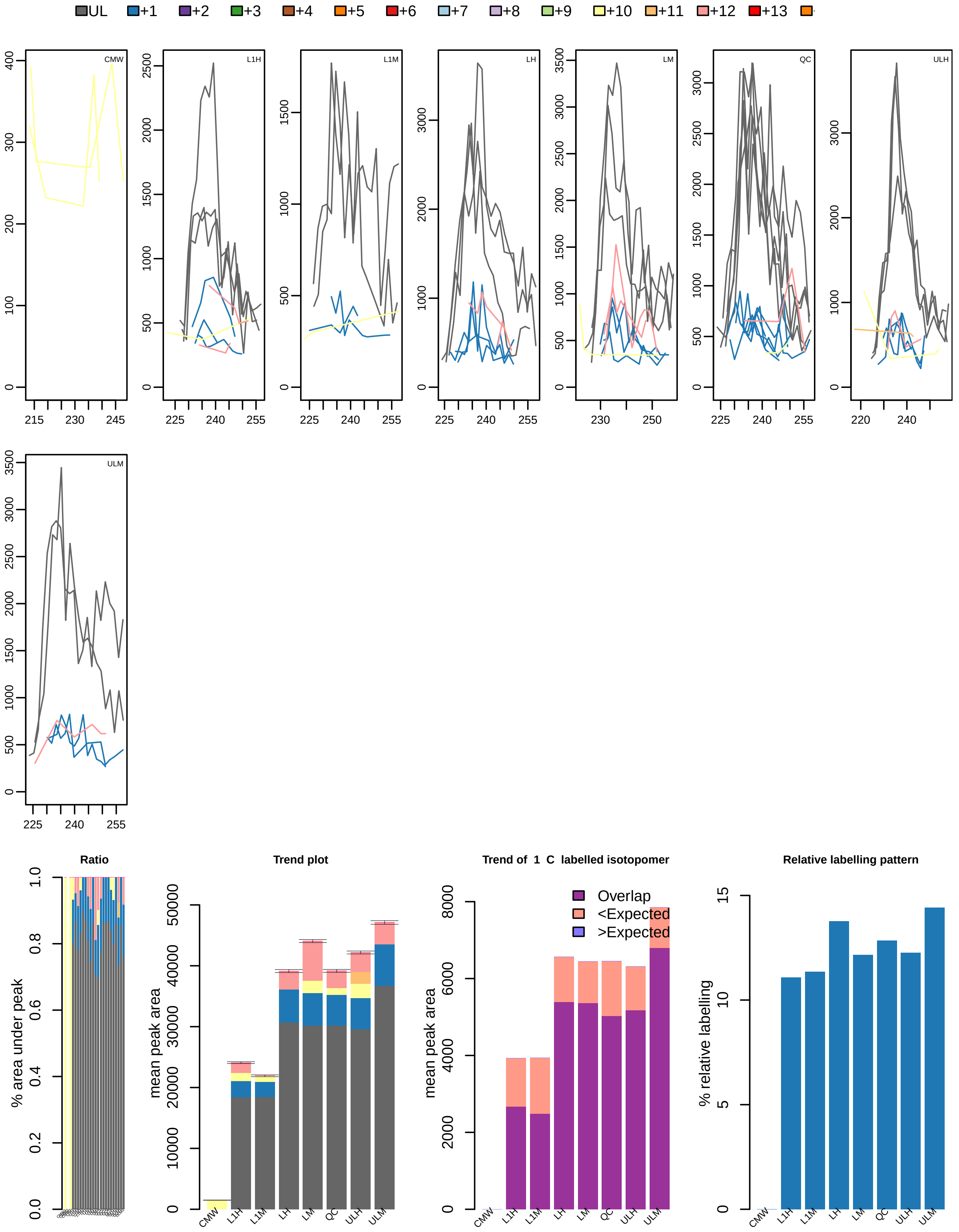

omega−Cyclohexylundecanoic acid

Formula: C17H32O2 Mass: 268.24 Std.RT: 214.33874958 Ion: NEG

G1

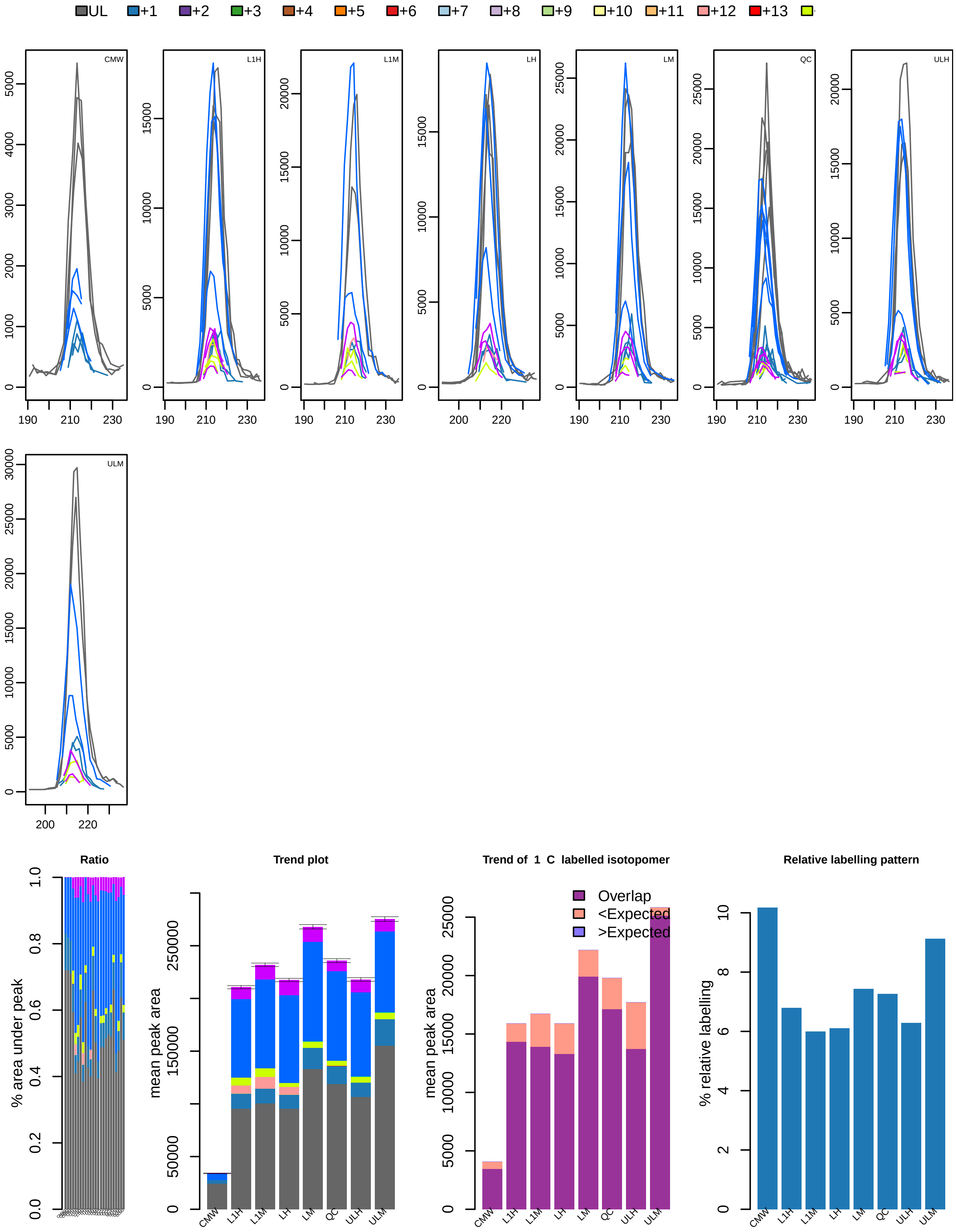

[FA methyl(18:0)] 11R,12S−methylene−octadecanoic a

G1

Formula: C19H36O2 Mass: 296.272 Std.RT: 212.8600419 Ion: NEG

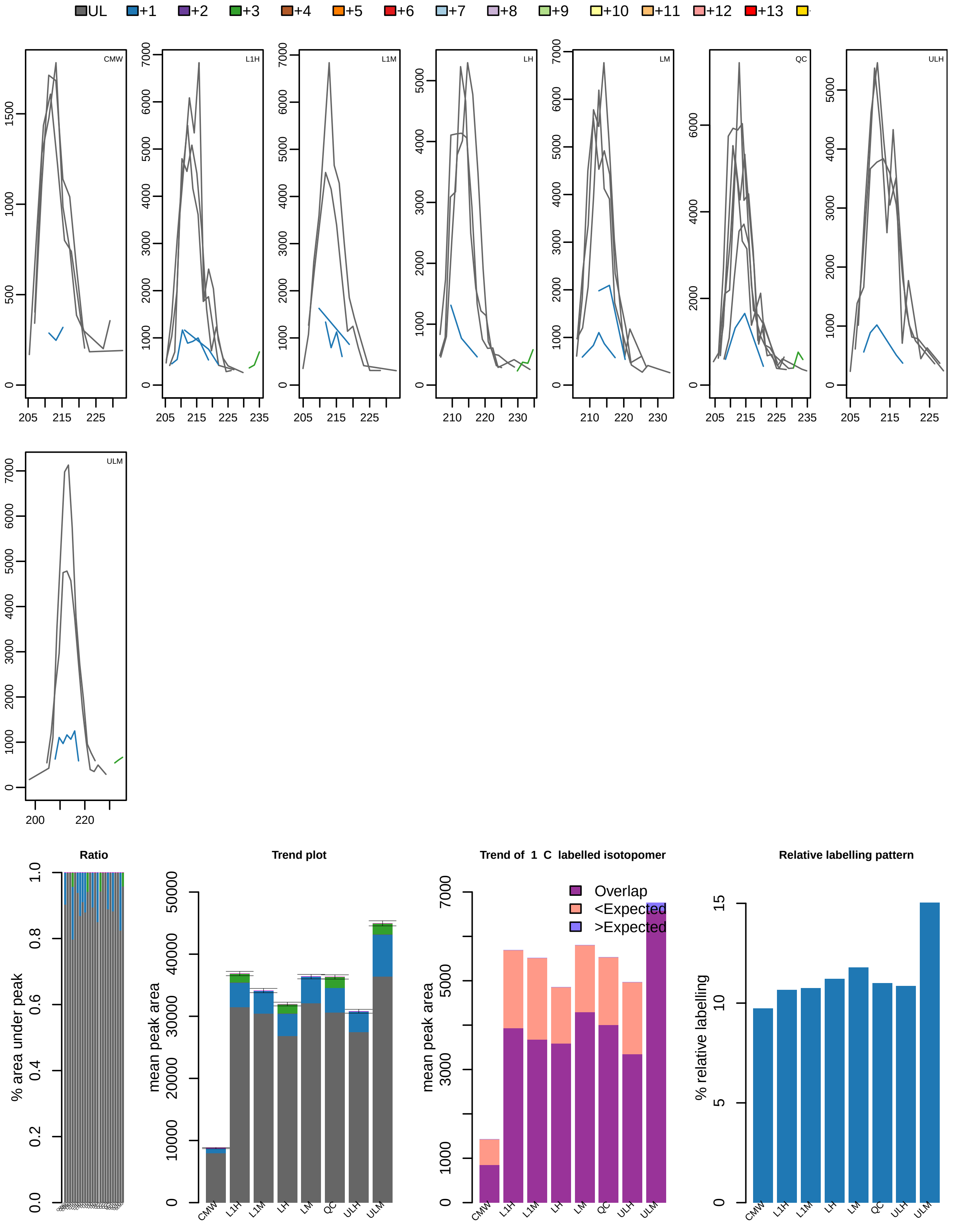

[FA hydroxy(24:0)] 2−hydroxy−15−tetracosenoic acid

G1

Formula: C24H46O3 Mass: 382.345 Std.RT: 207.98609202 Ion: NEG

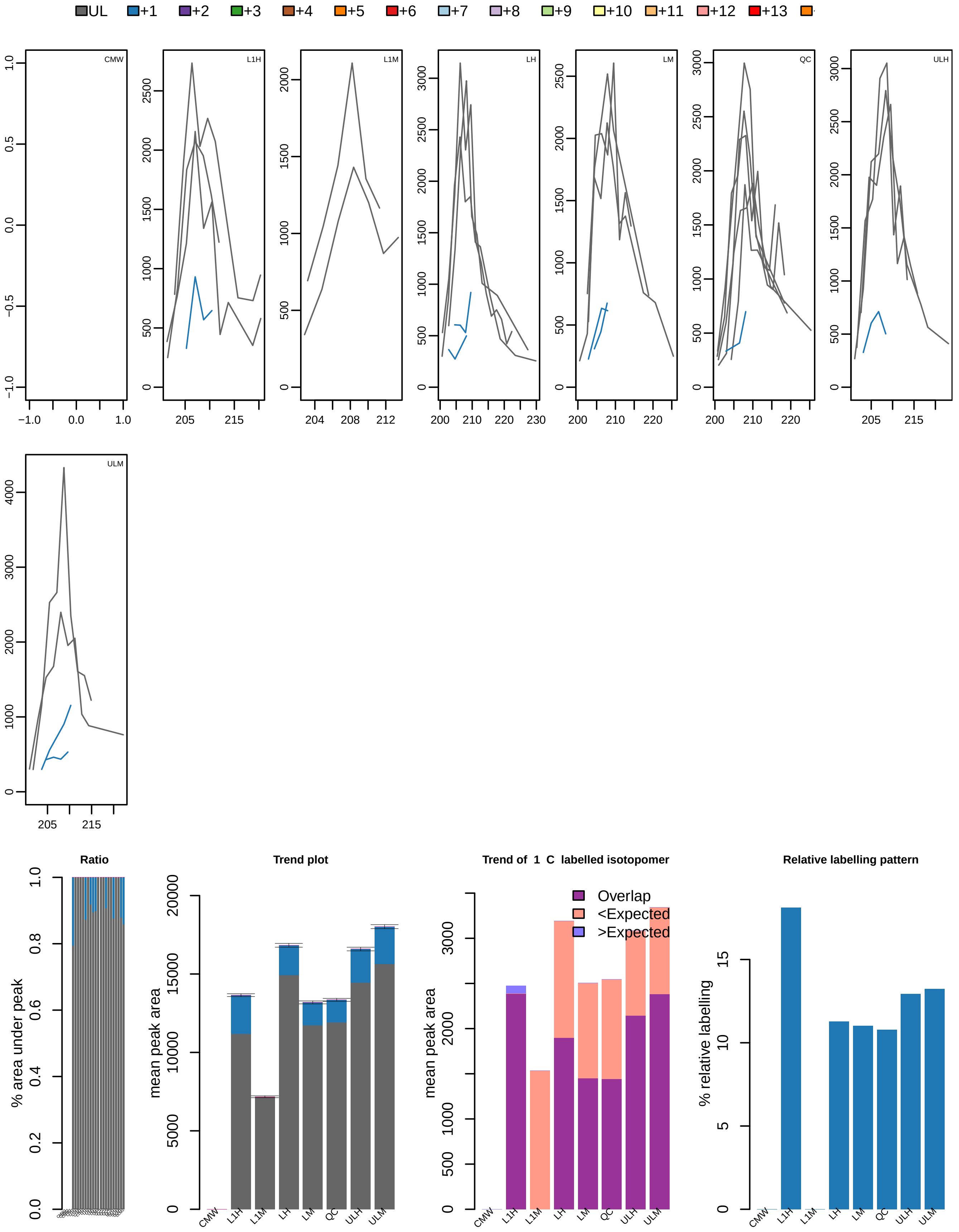

[FA (17:0)] heptadecanoic acid

Formula: C17H34O2 Mass: 270.256 Std.RT: 213.8024991 Ion: NEG

G1

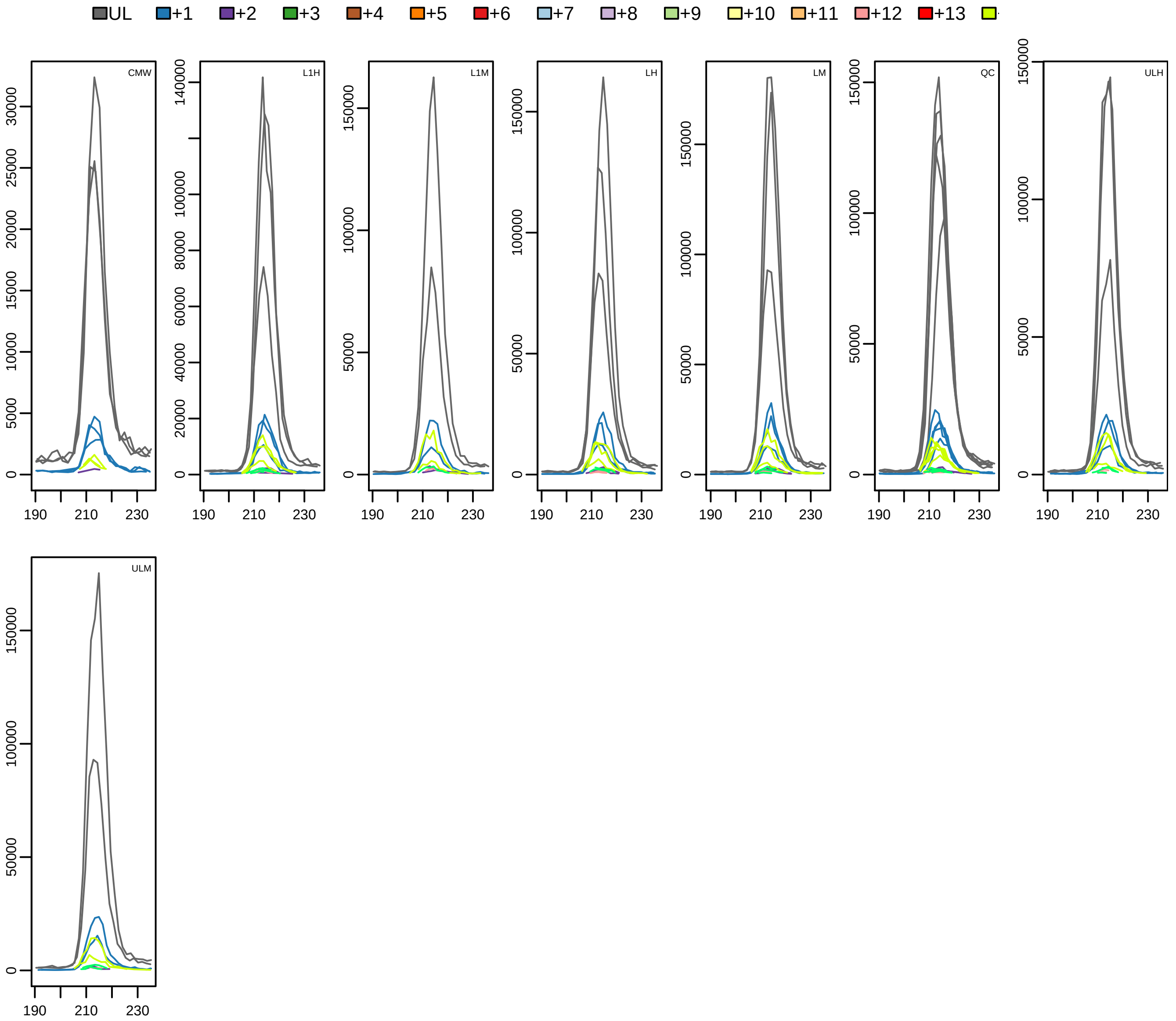

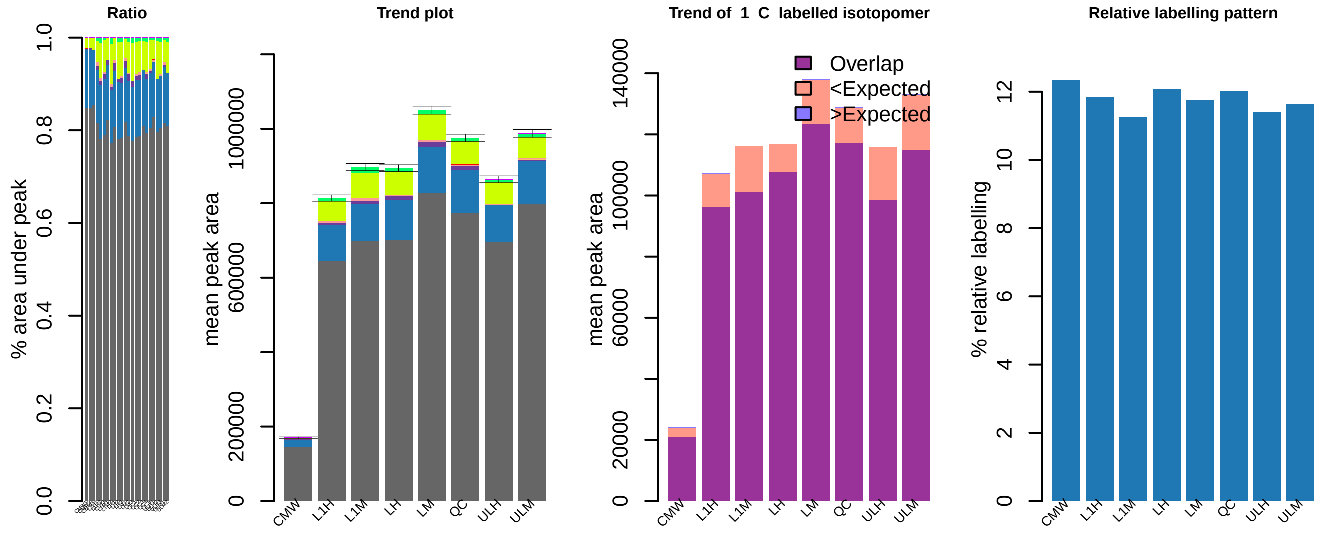

[FA hydroxy(18:0)] 2S−hydroxy−octadecanoic acid

Formula: C18H36O3 Mass: 300.266 Std.RT: 214.50229068 Ion: NEG

G1

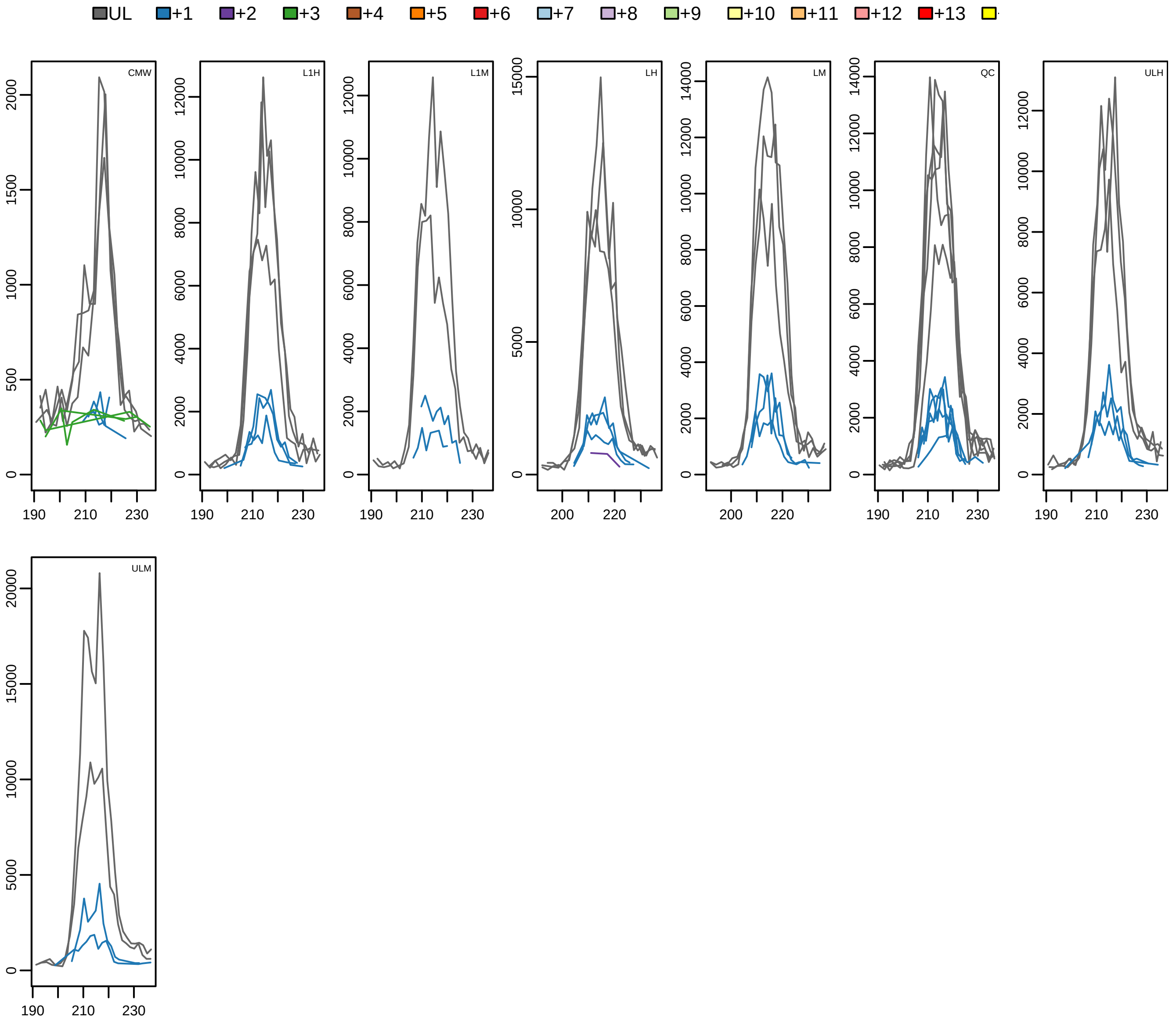

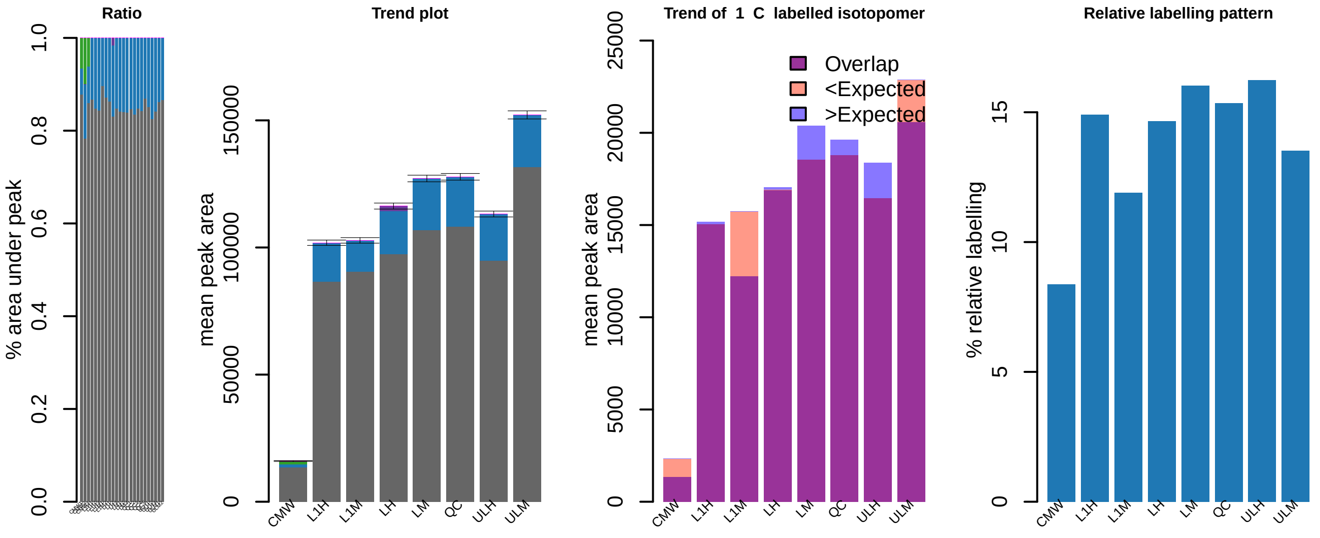

[FA (20:3)] 8Z,11Z,14Z−eicosatrienoic acid

Formula: C20H34O2 Mass: 306.256 Std.RT: 212.31052578 Ion: NEG

G1

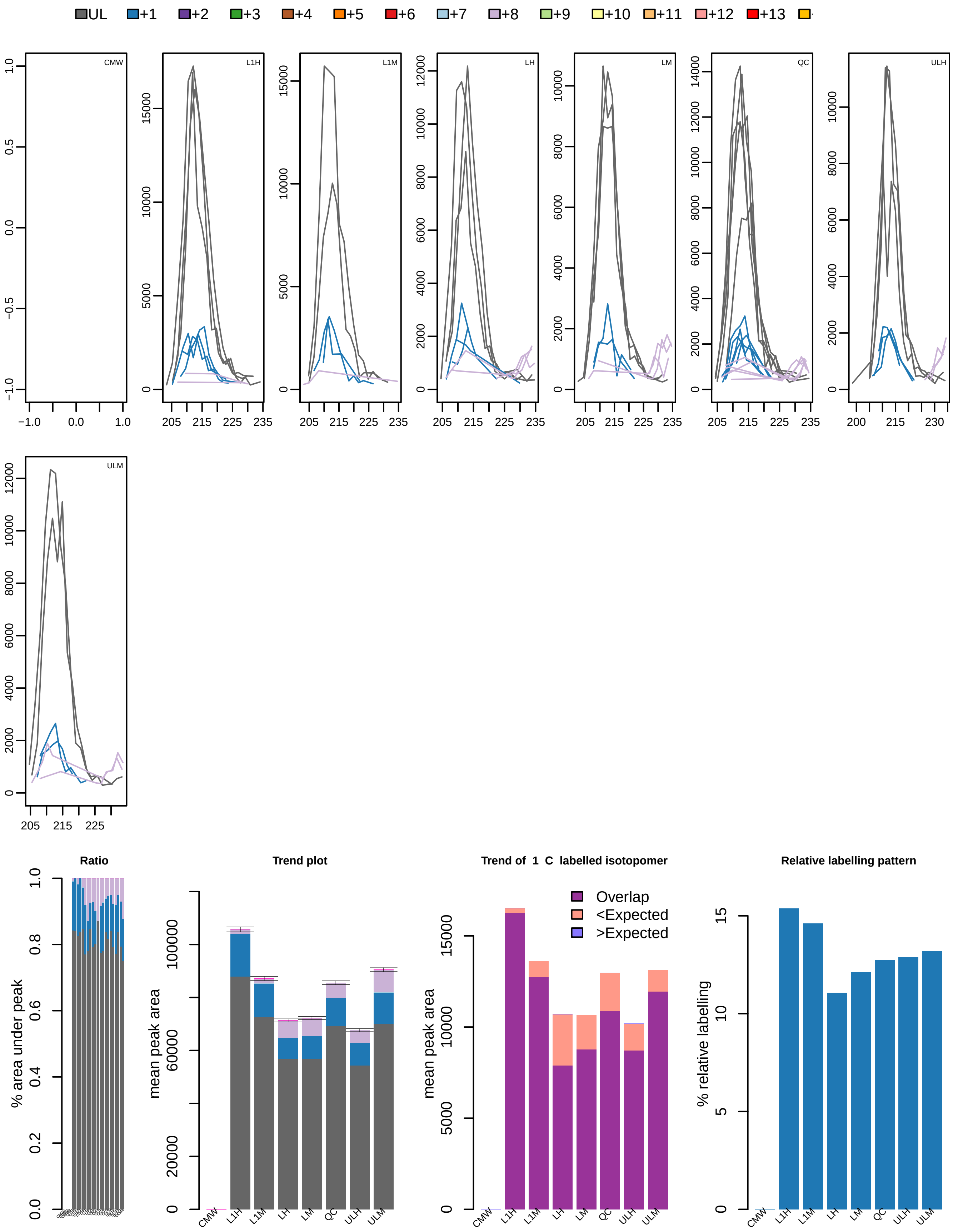

[FA hydroxy(4:0/18:0)] 9,10,12,13−tetrahydroxy−octade

Formula: C18H36O6 Mass: 348.251 Std.RT: 253.342809 Ion: NEG

G1

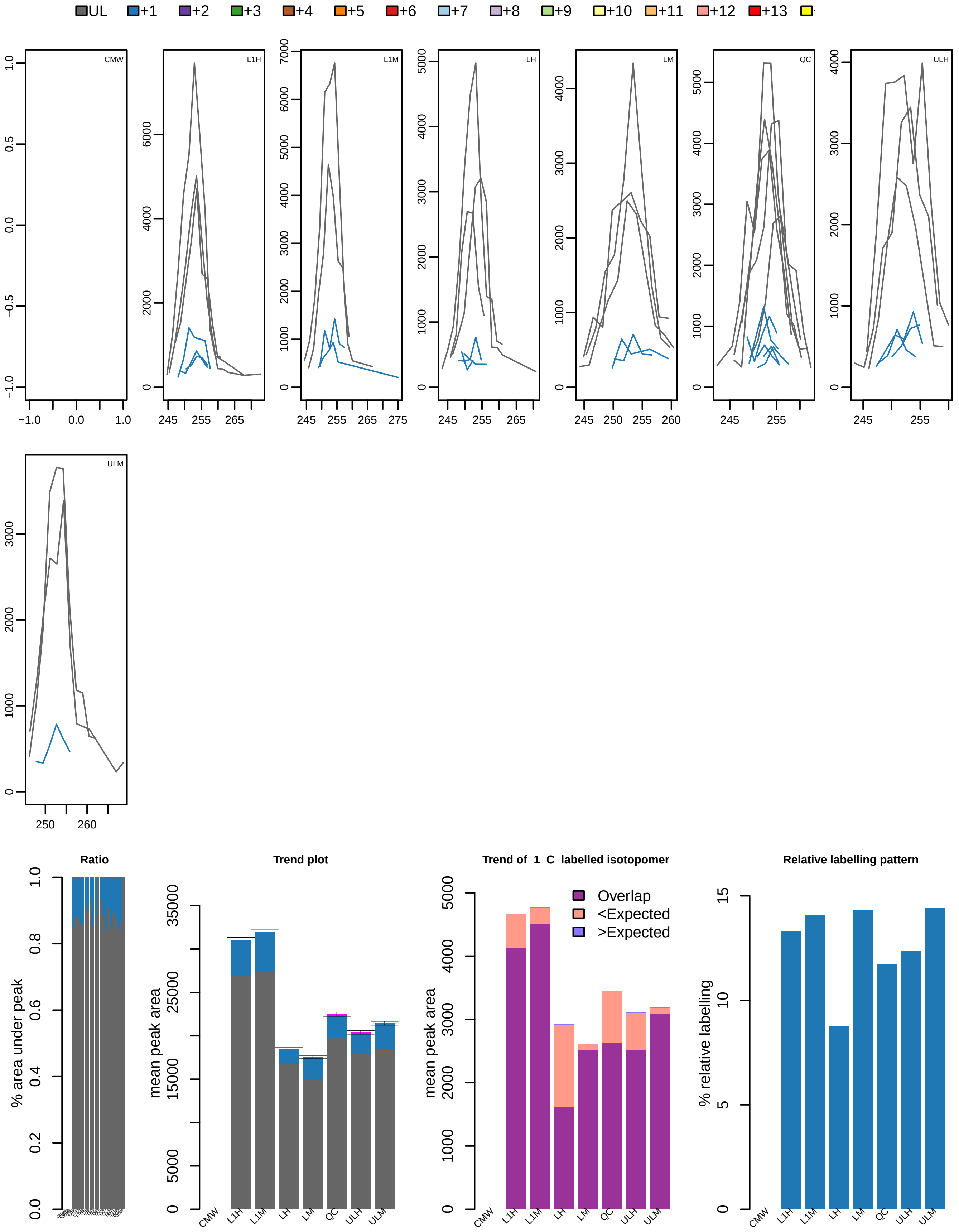

Nonadecanoicacid

Formula: C19H38O2 Mass: 298.287 Std.RT: 212.10966678 Ion: NEG

G1

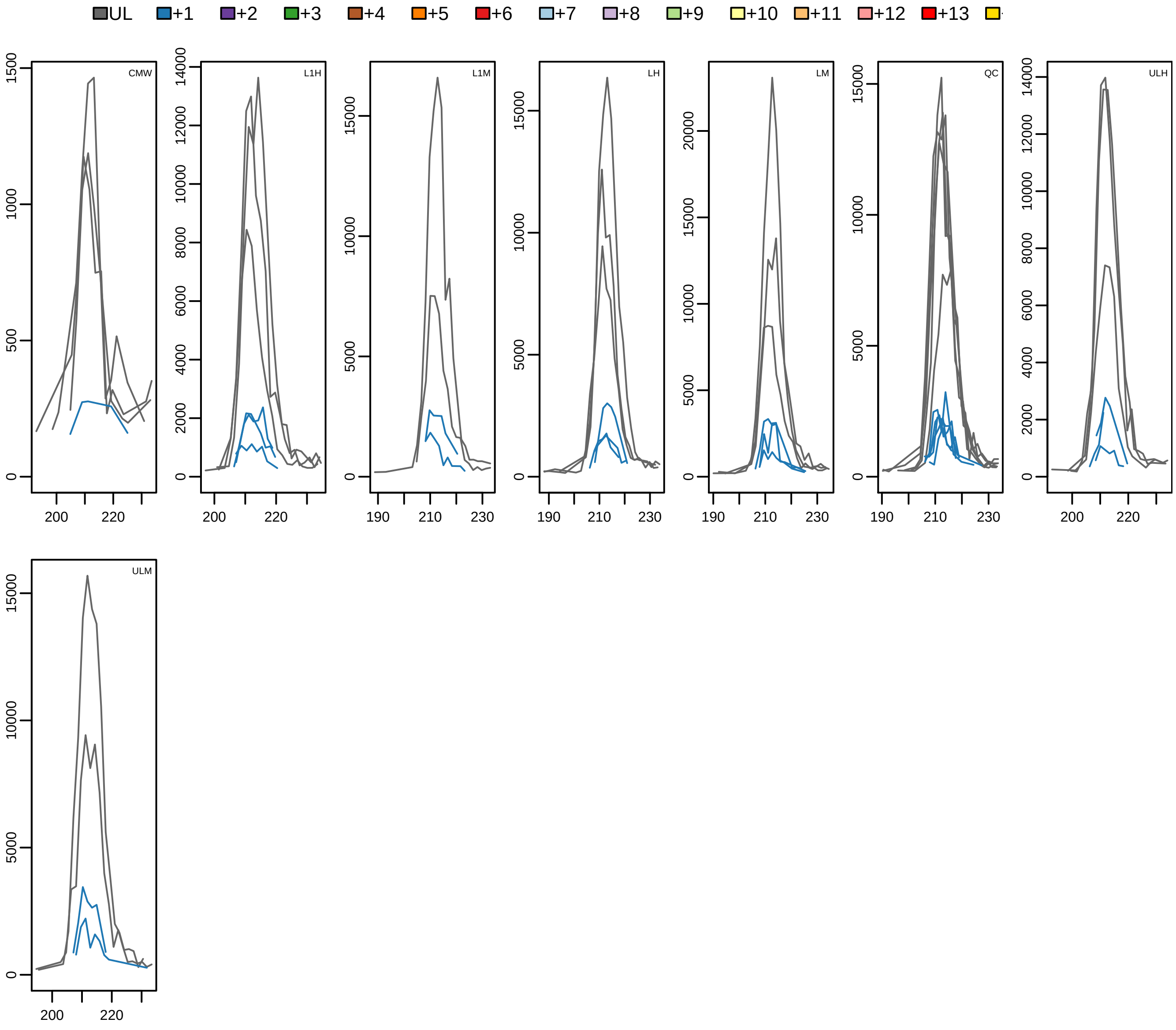

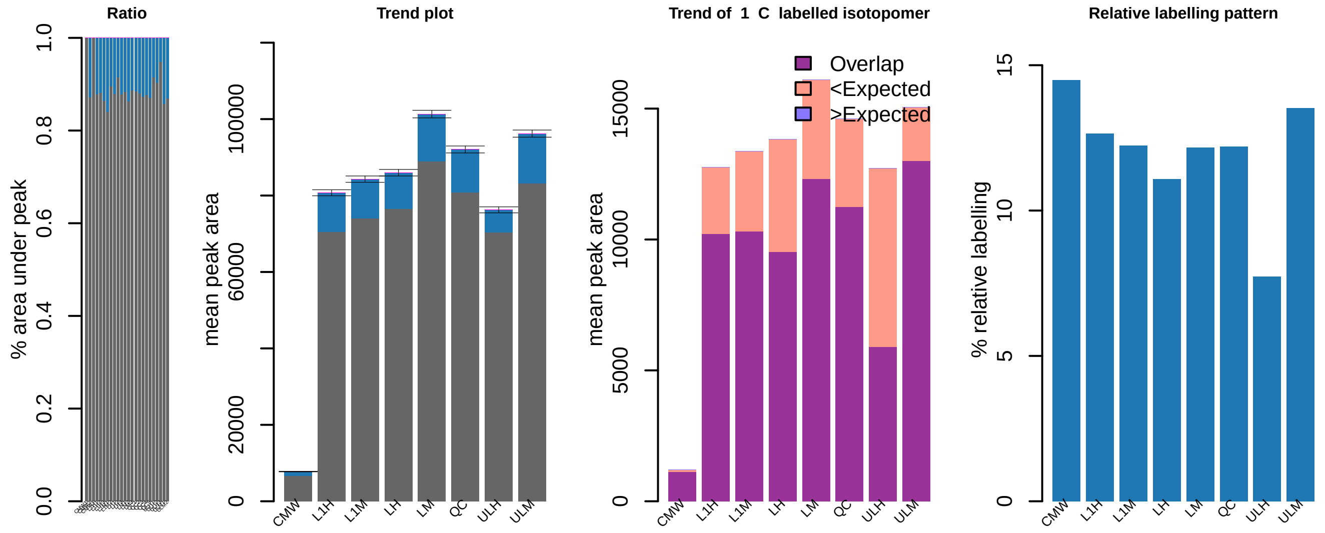

[FA hydroxy(24:0)] 2−hydroxy−tetracosanoic acid

Formula: $C_{24}H_{48}O_3$ Mass: 384.36 Std.RT: 208.40058972 Ion: NEG

G1

UL +1 +2 +3 +4 +5 +6 +7 +8 +9 +10 +11 +12 +13

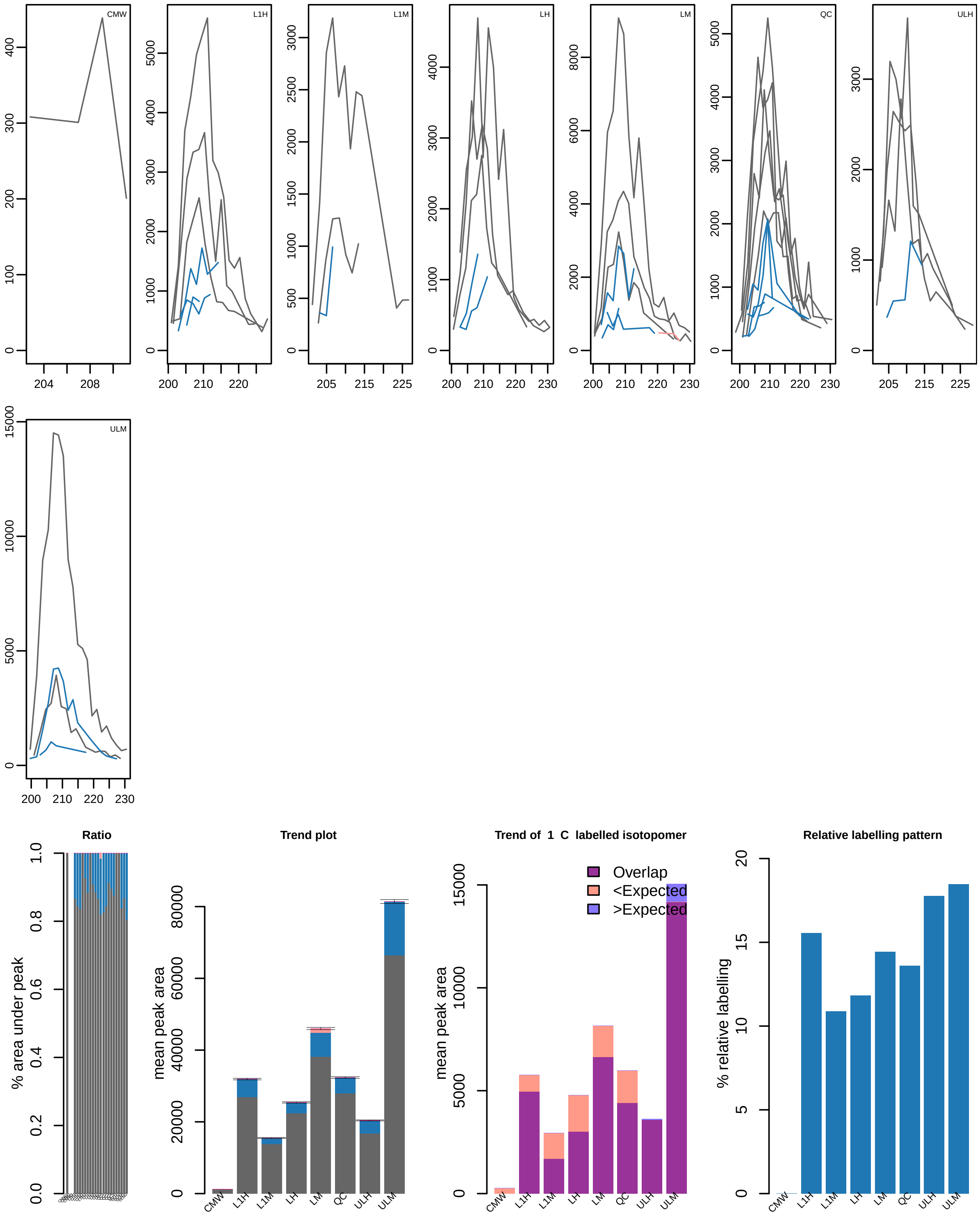

[FA (20:4)] 5Z,8Z,11Z,14Z−eicosatetraenoic acid

Formula: C20H32O2 Mass: 304.24 Std.RT: 212.01971508 Ion: NEG

G1

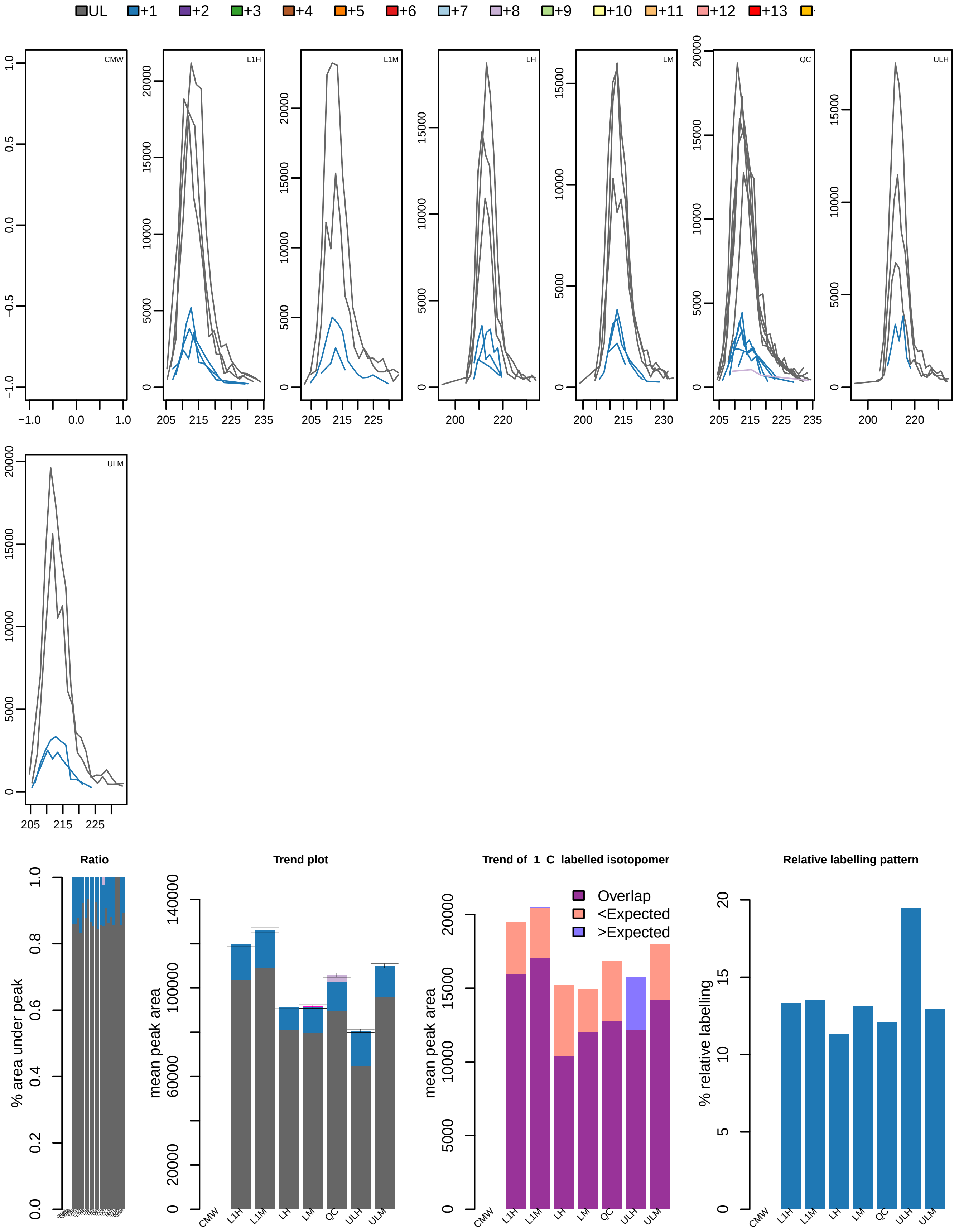

[FA hydroxy(22:0)] 13−hydroxy−docosanoic acid

G1

Formula: C22H44O3 Mass: 356.329 Std.RT: 210.12357078 Ion: NEG

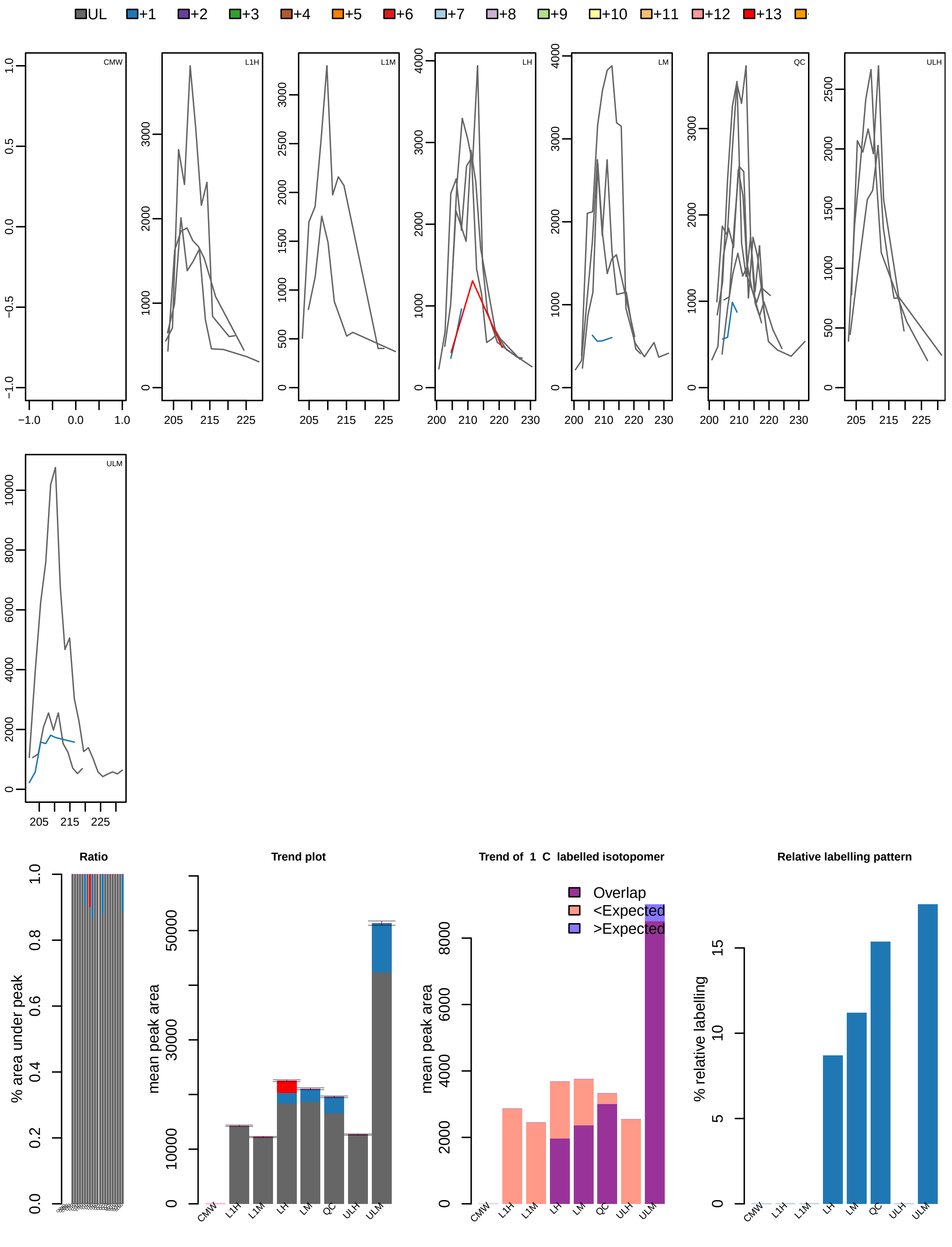

[FA (24:0/2:0)] Tetracosanedioic acid

Formula: C24H46O4 Mass: 398.34 Std.RT: 208.45028322 Ion: NEG

G1

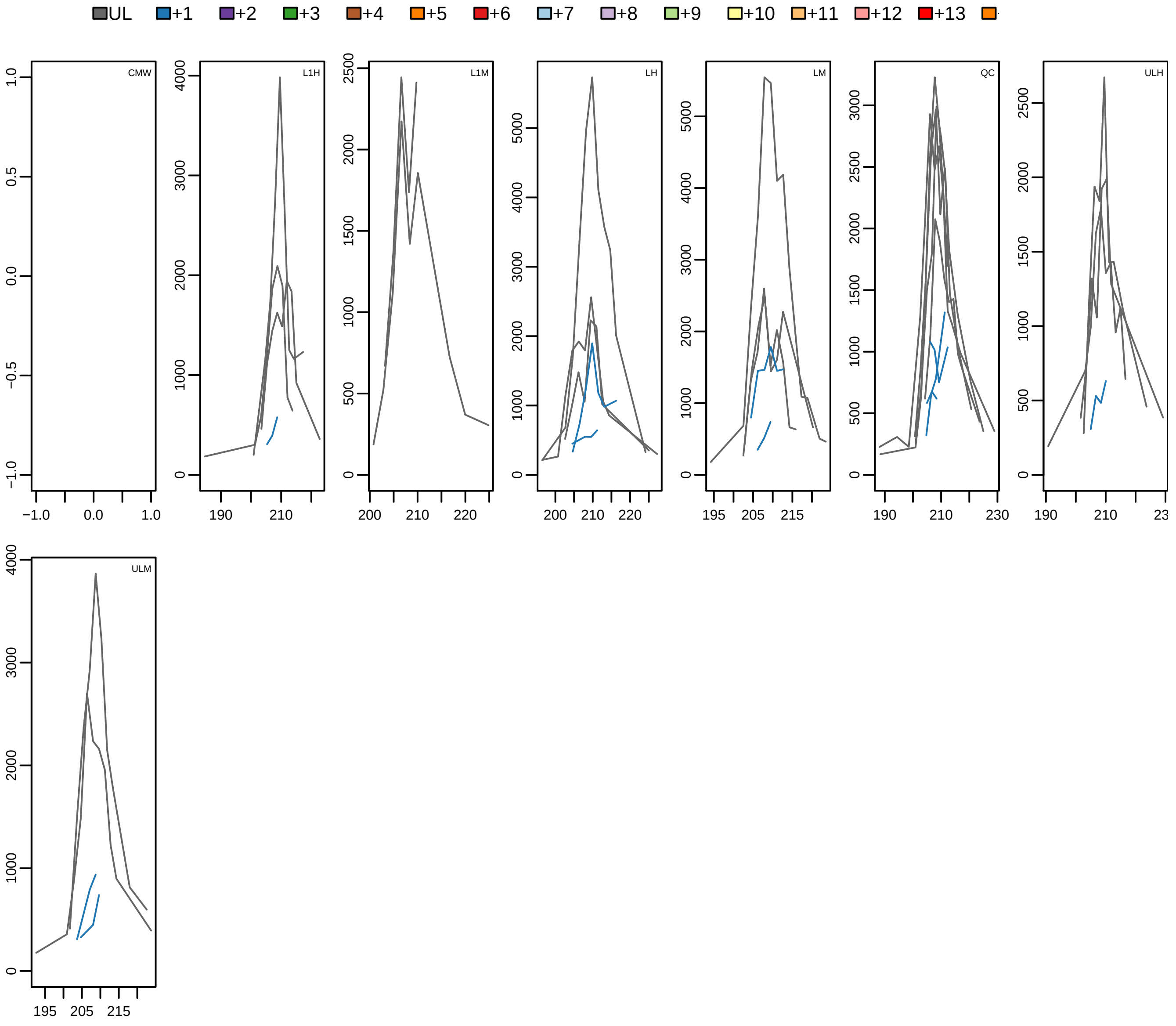

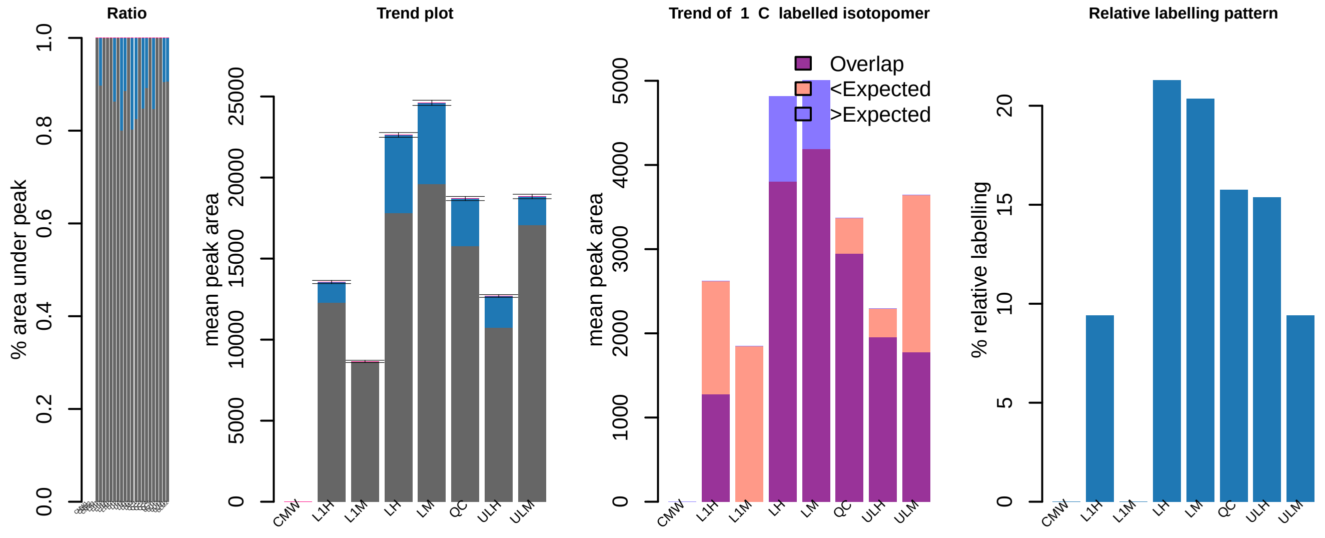

[FA hydroxy(6:0)] 4−hydroxy−hexanoic acid

Formula: C6H12O3 Mass: 132.079 Std.RT: 269.84358468 Ion: NEG

G1

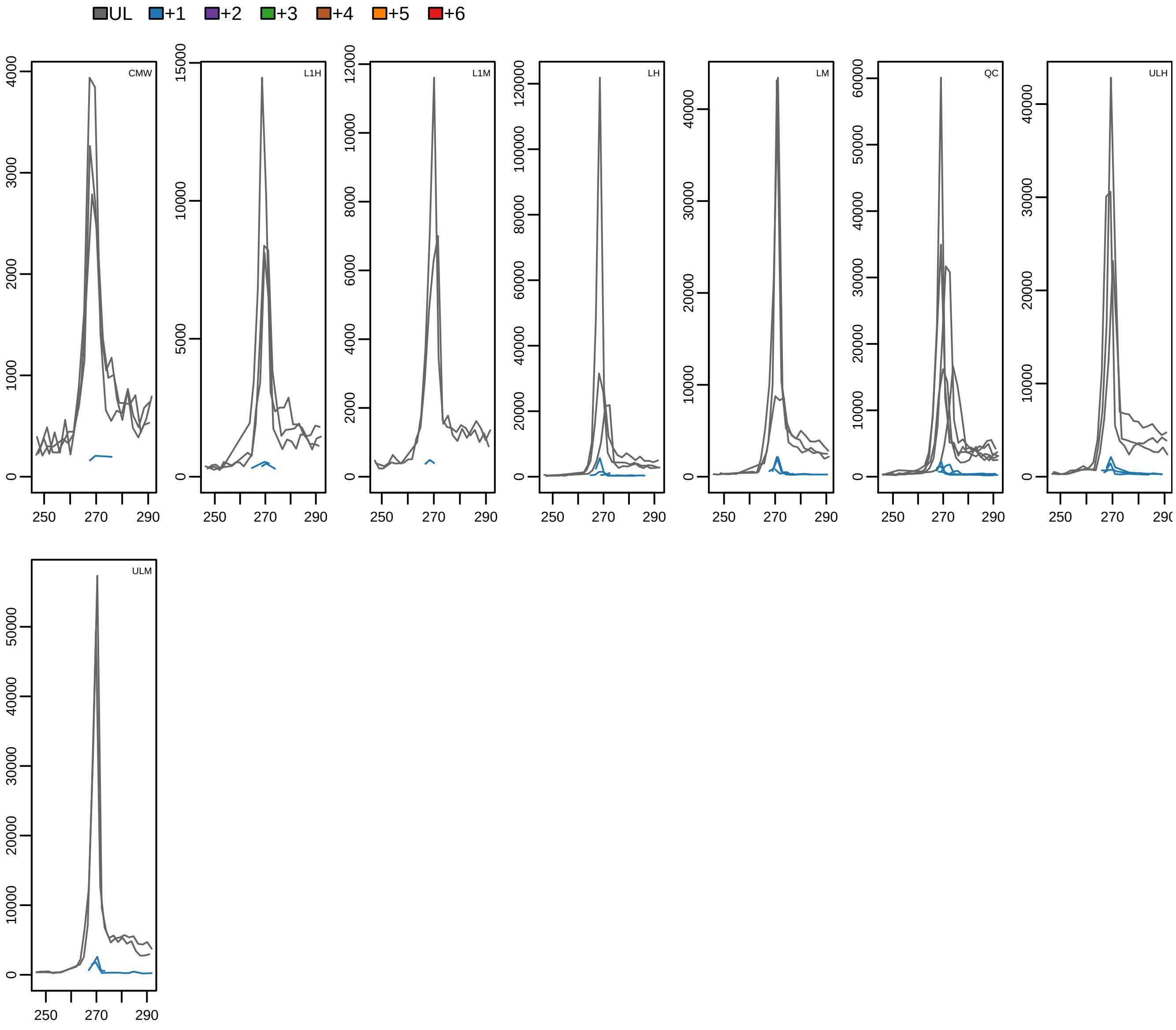

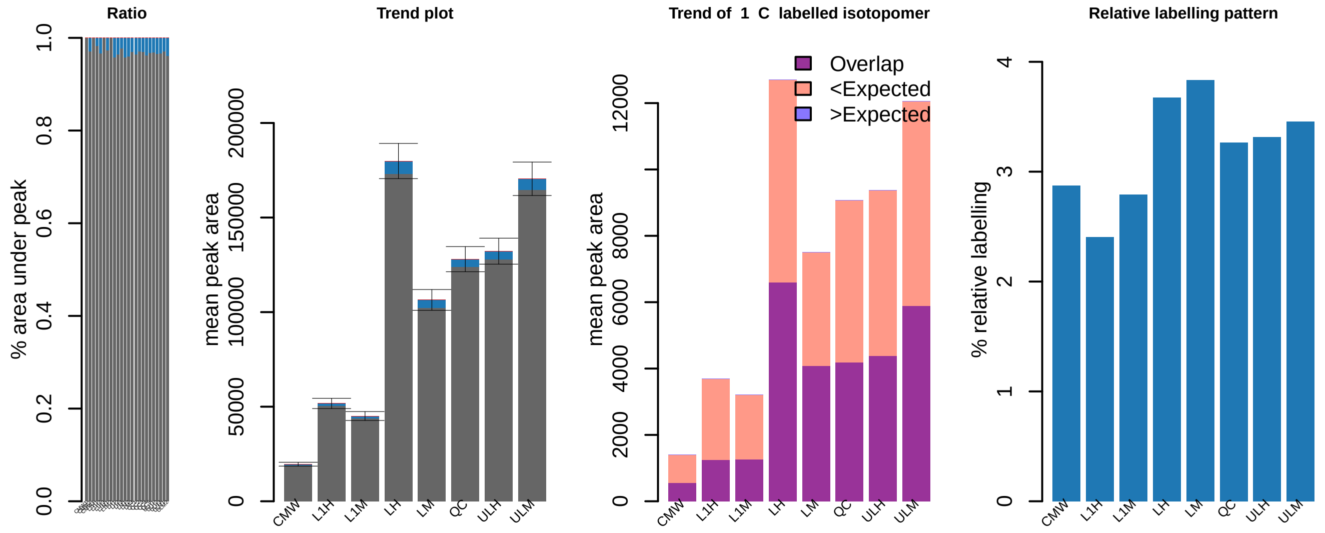

[FA (18:2)] 9S−hydroperoxy−10E,12Z−octadecadienoic

G1

Formula: C18H32O4 Mass: 312.23 Std.RT: 218.203083 Ion: NEG

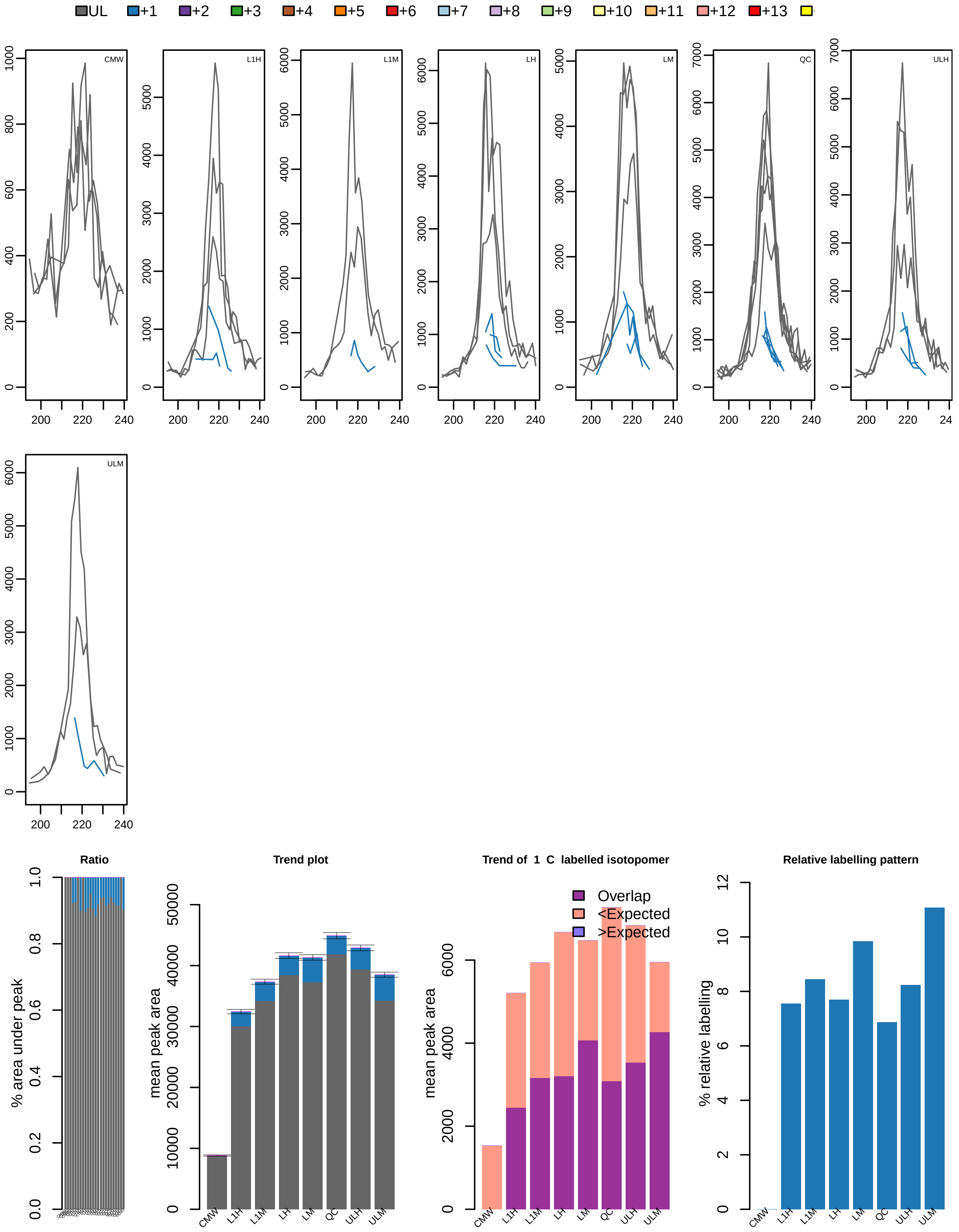

[FA trihydroxy(18:1)] 9S,12S,13S−trihydroxy−10E−octa

Formula: C18H34O5 Mass: 330.241 Std.RT: 233.33816778 Ion: NEG

G1

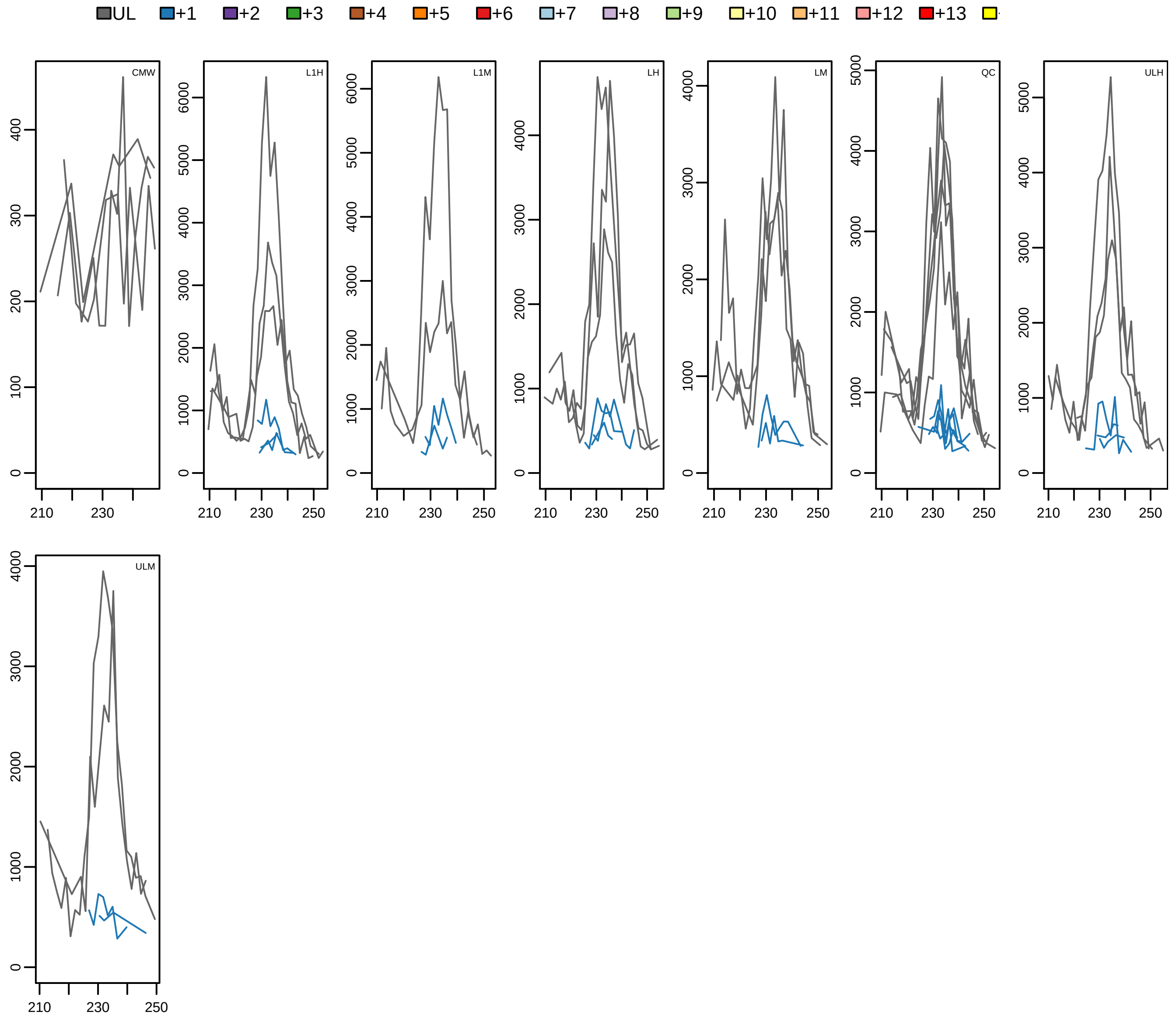

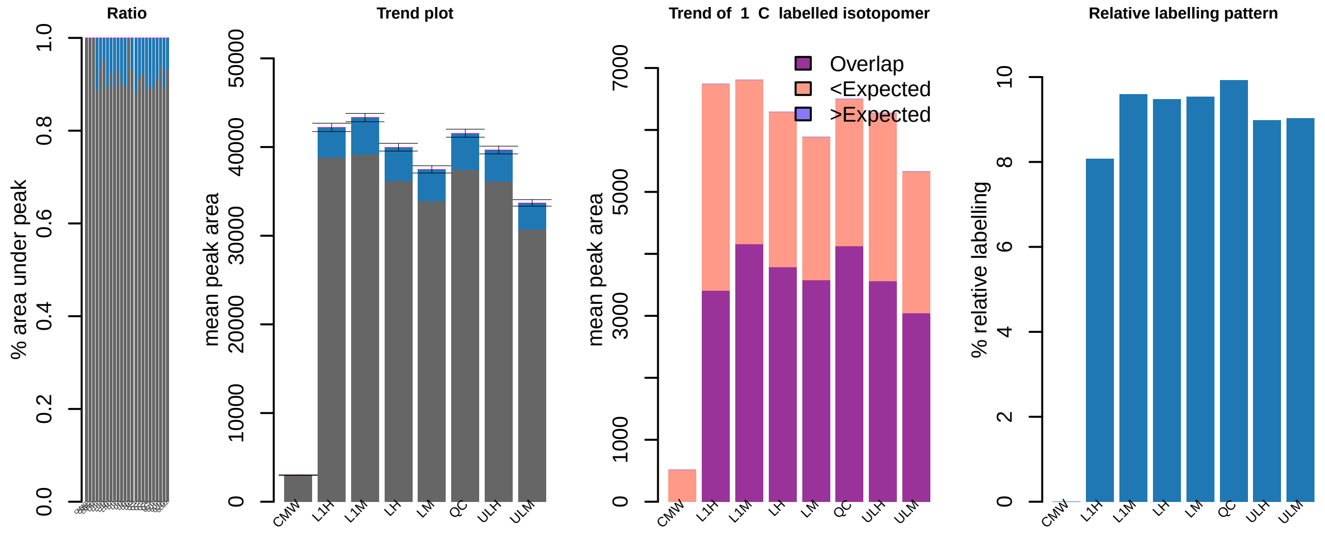

[GP (18:0/18:0)] 1−octadecanoyl−2−(9Z−octadecenoyl) G1

Formula: C43H82NO10P Mass: 803.568 Std.RT: 232.946523 Ion: NE

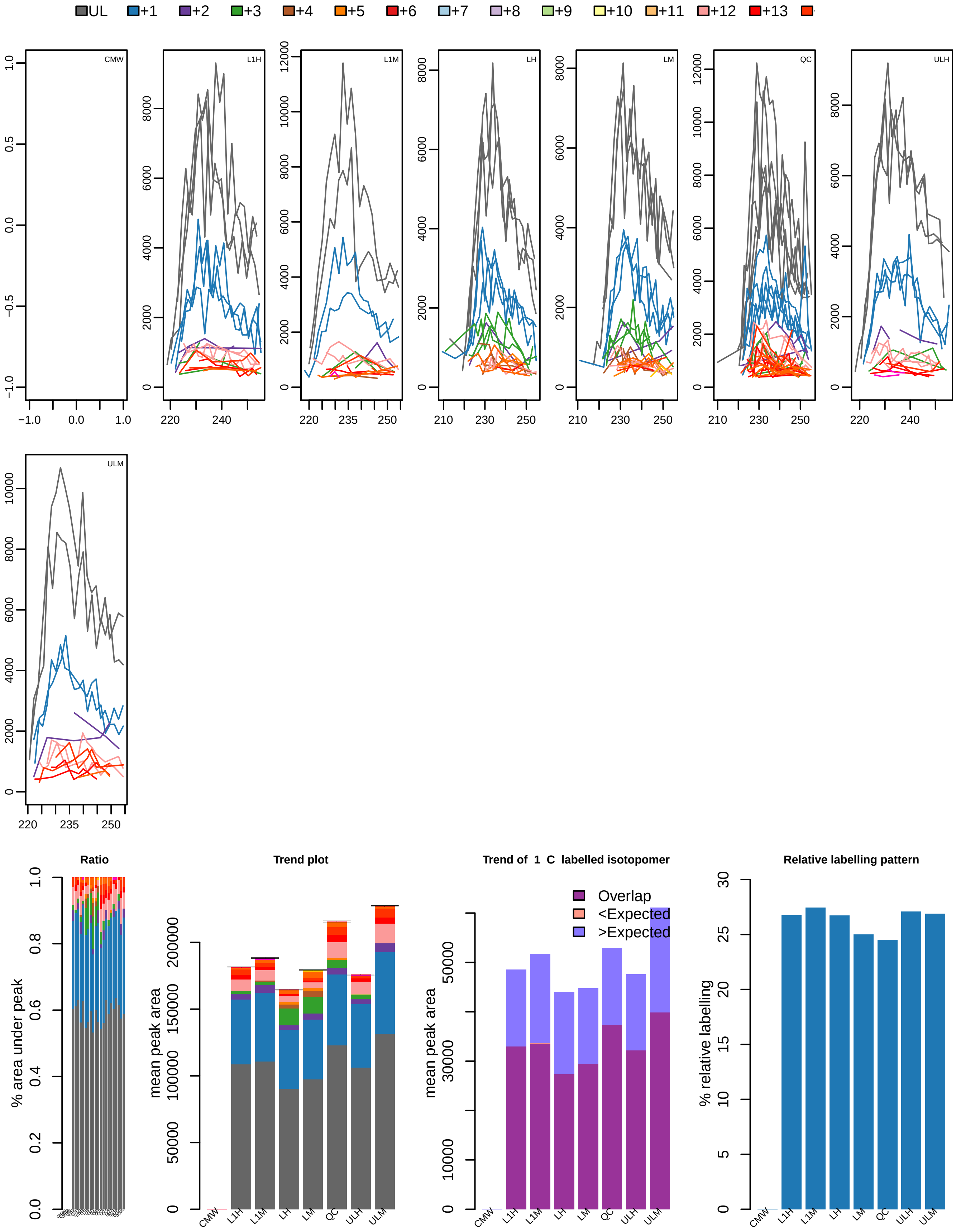

[GP (18:2/18:2)] 1,2−di−(9Z,12Z−octadecadienoyl)−sn−

G1

Formula: C39H69O8P Mass: 696.473 Std.RT: 207.5612379 Ion: NEG

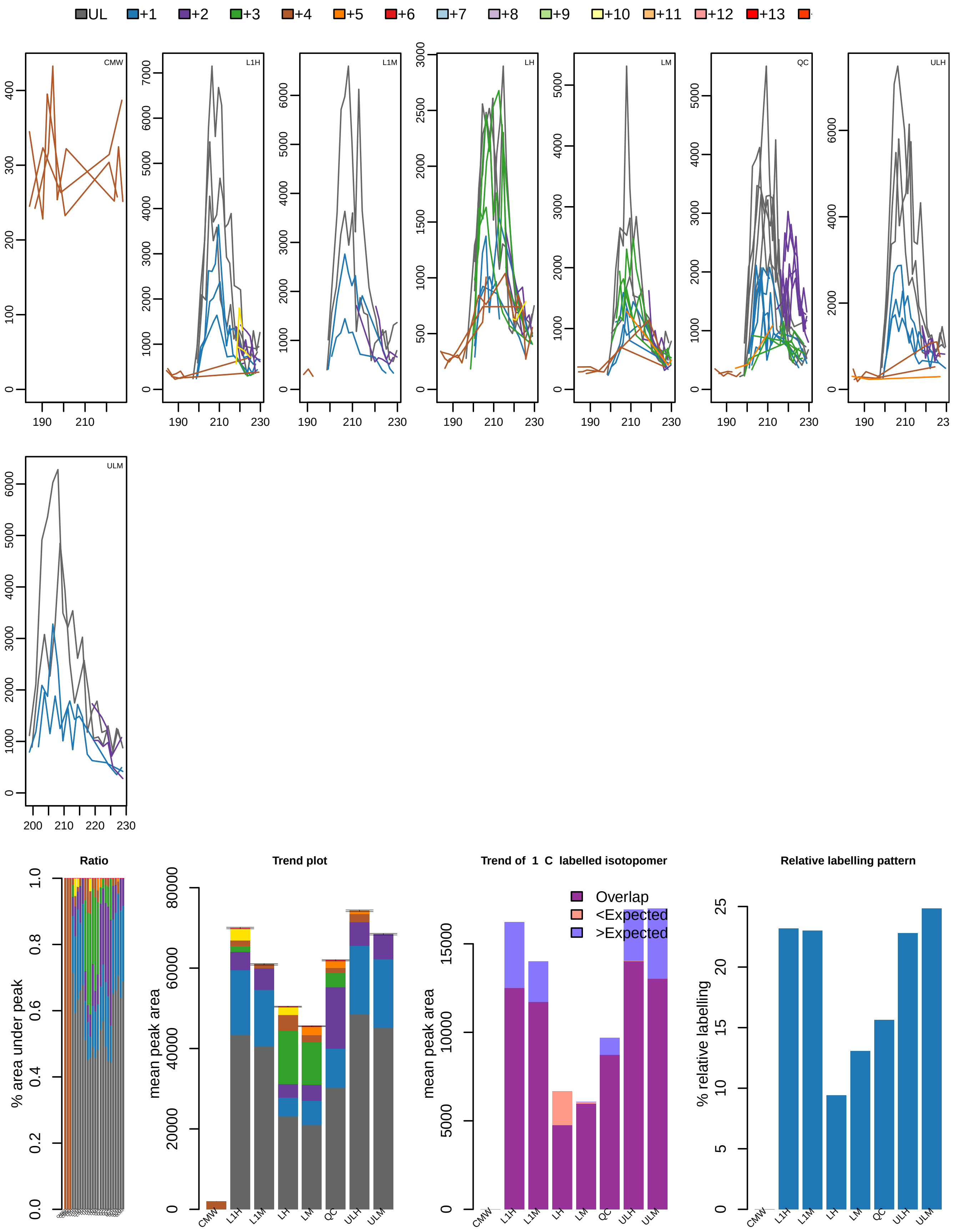

[GP (18:0/18:2)] 1−octadecanoyl−2−(9Z,12Z−octadeca

Formula: C39H73O8P Mass: 700.504 Std.RT: 210.4846656 Ion: NE[illegible]

G1

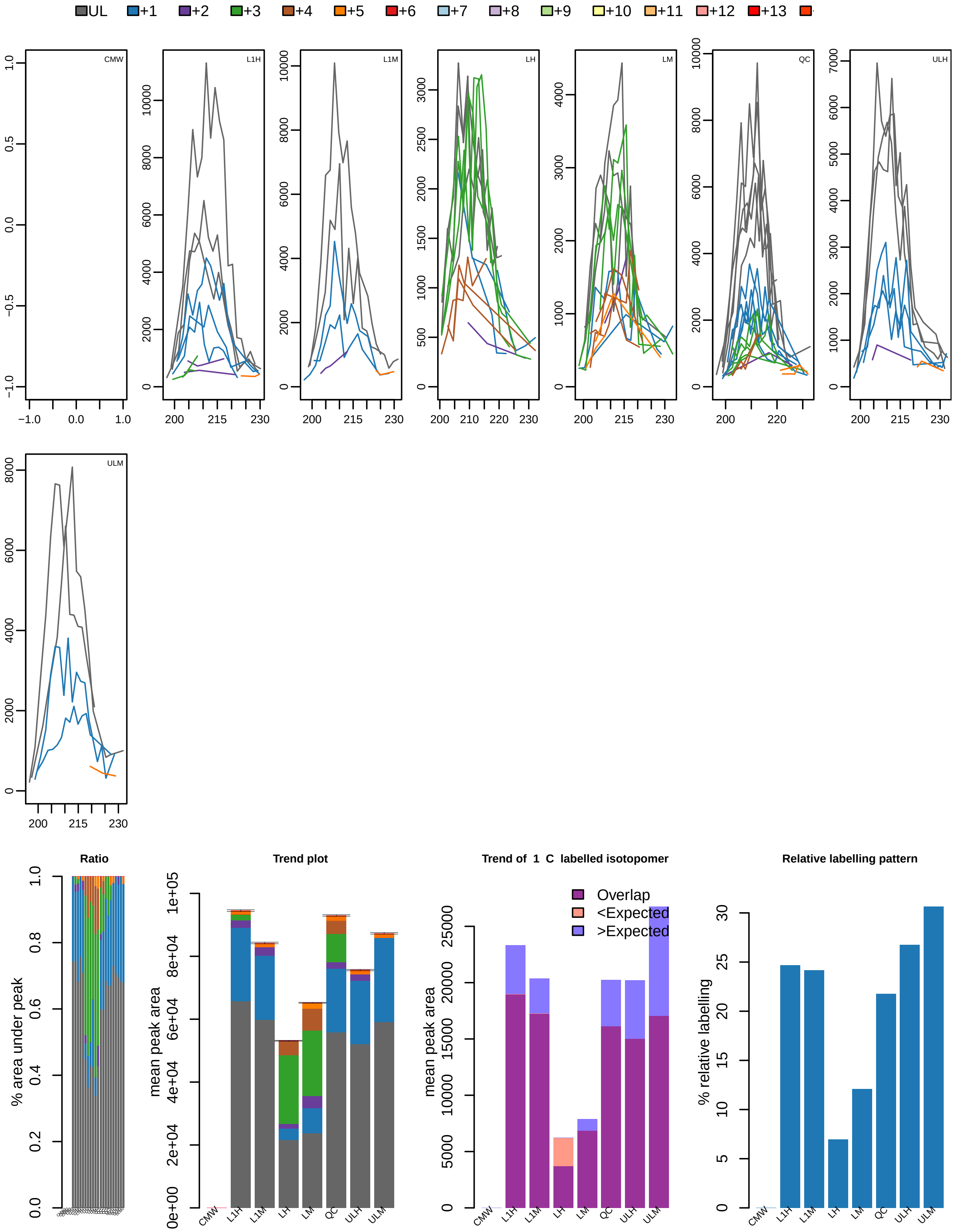

[PC (17:1)] 1−(1Z−heptadecenyl)−sn−glycero−3−phosp
Formula: C25H52NO6P Mass: 493.353 Std.RT: 245.23366662 Ion: N

G1

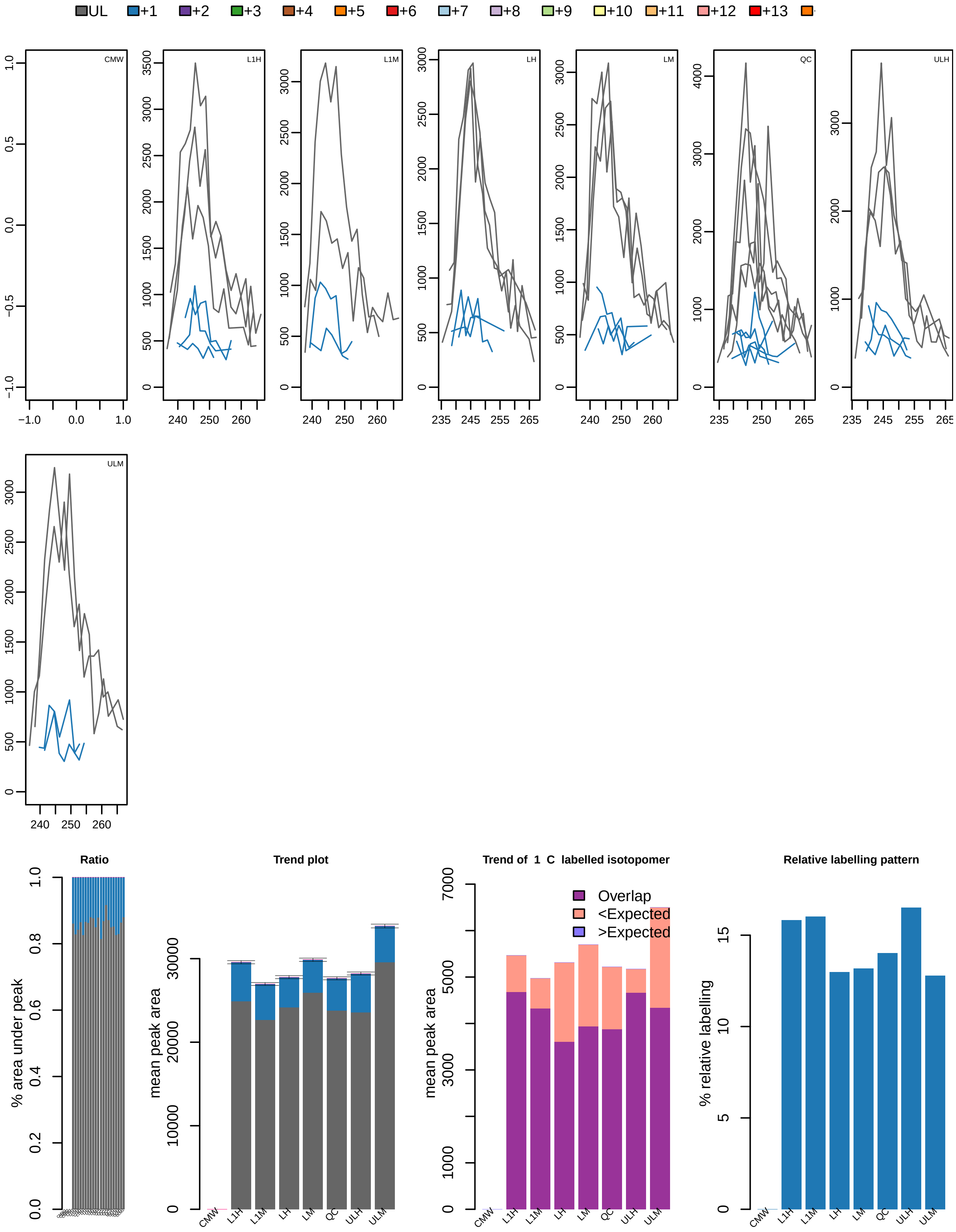

[PE (18:0/18:2)] 1−octadecanoyl−2−(9Z,12Z−octadeca

Formula: C41H78NO8P Mass: 743.547 Std.RT: 223.14013068 Ion: N

G1

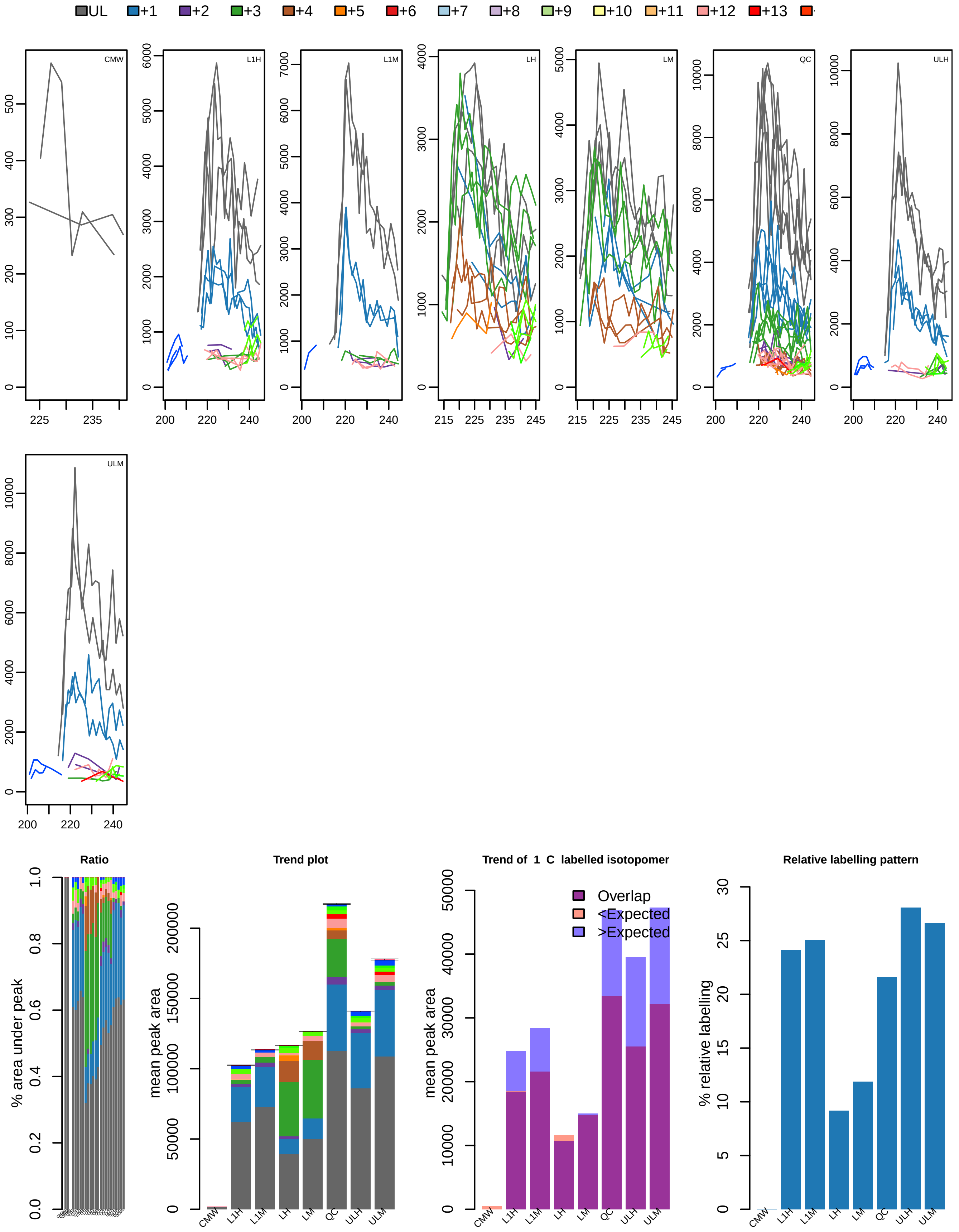

LysoPE(0:0/22:4(7Z,10Z,13Z,16Z))

Formula: C27H48NO7P Mass: 529.317 Std.RT: 246.60999918 Ion: N

G1

UL +1 +2 +3 +4 +5 +6 +7 +8 +9 +10 +11 +12 +13

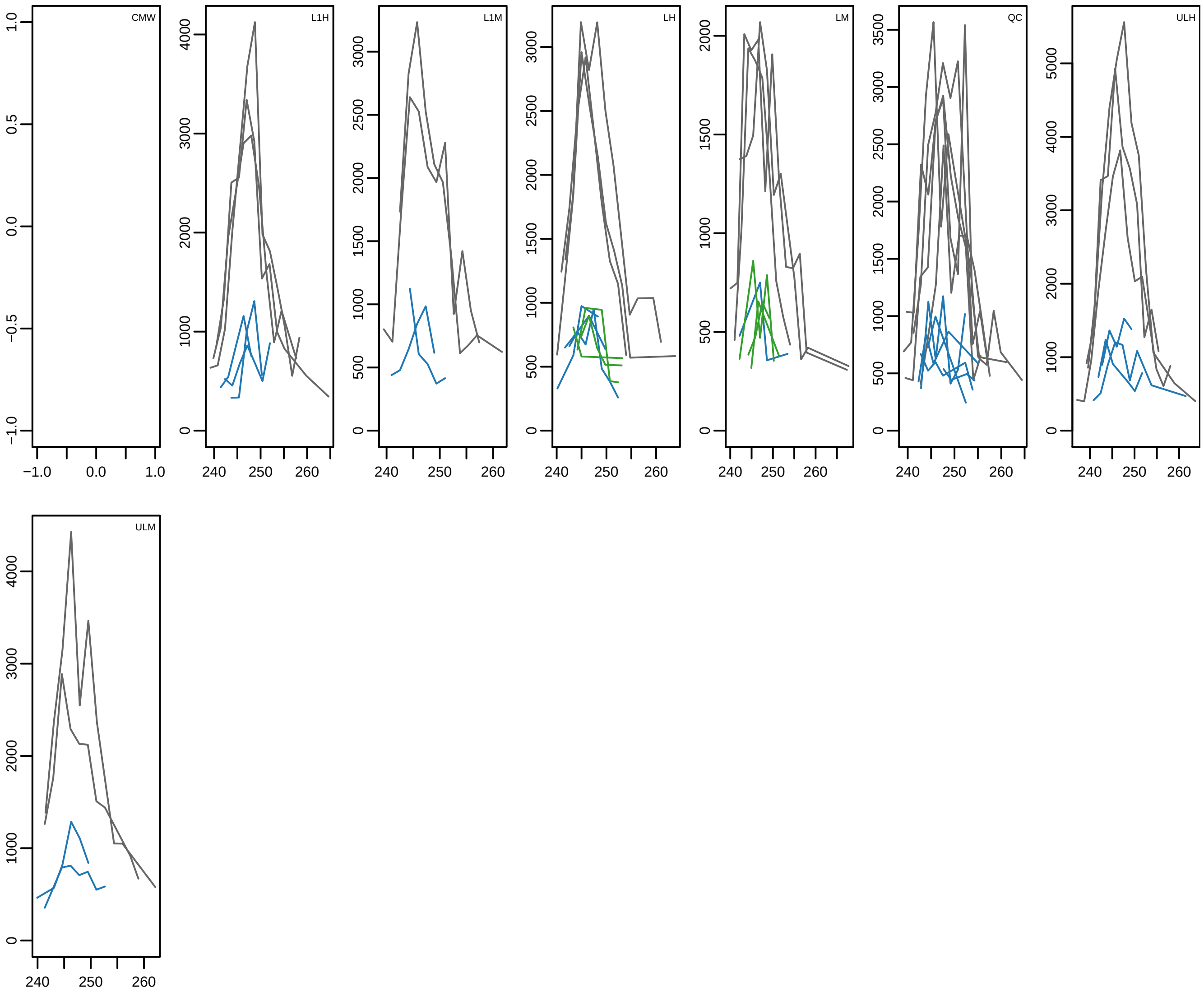

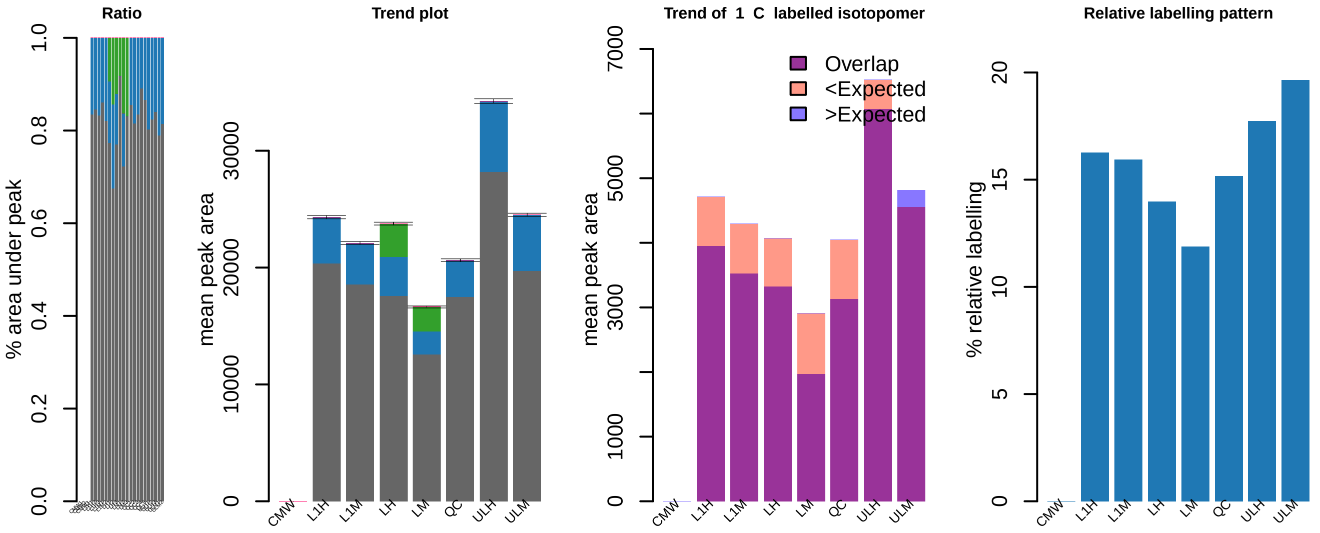

LysoPE(0:0/22:6(4Z,7Z,10Z,13Z,16Z,19Z))

Formula: C27H44NO7P Mass: 525.286 Std.RT: 248.7933807 Ion: NE

G1

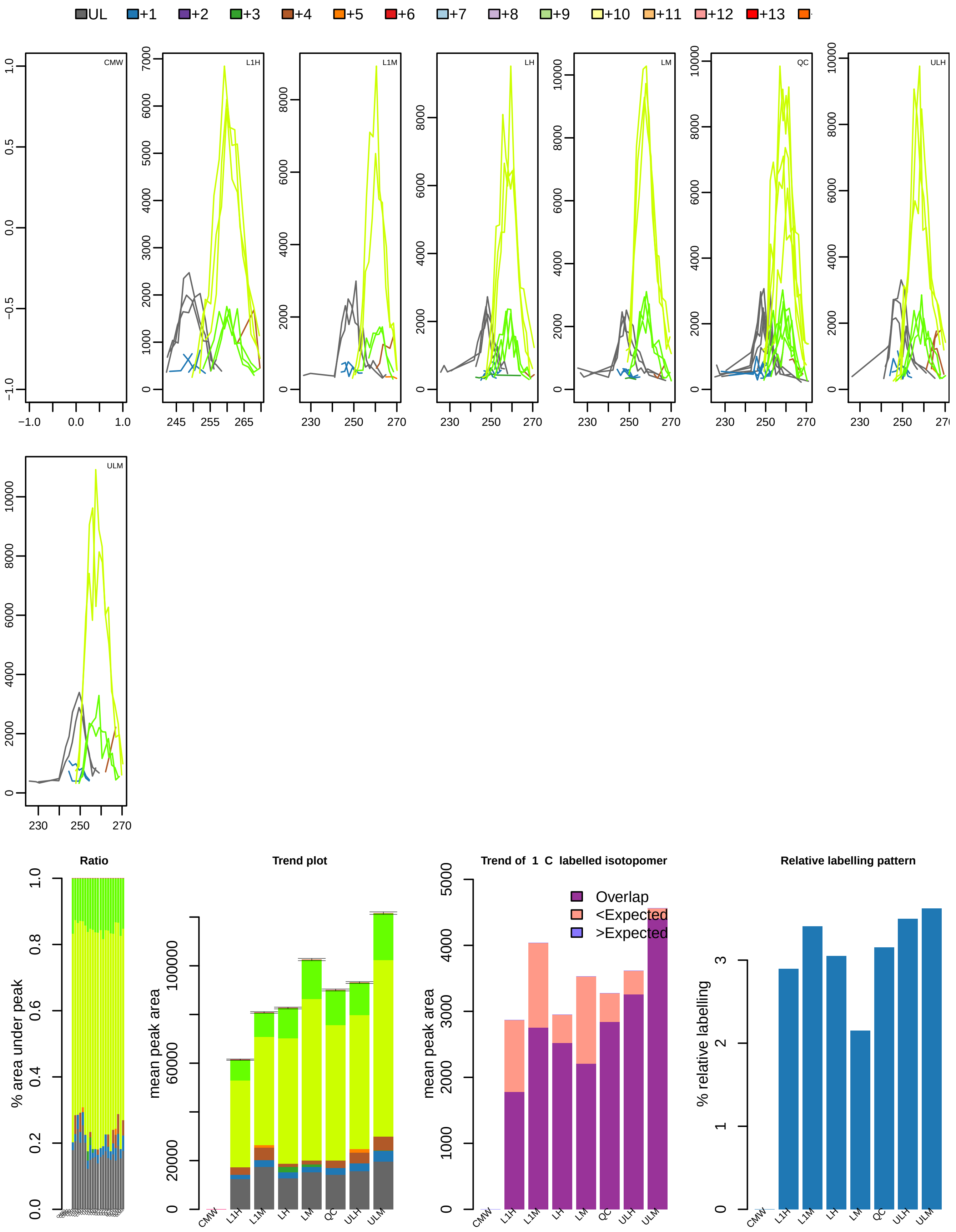

[PG (18:1/18:1)] 1,2−di−(9Z−octadecenoyl)−sn−glycerc

Formula: C42H79O10P Mass: 774.541 Std.RT: 204.40899882 Ion: N

G1

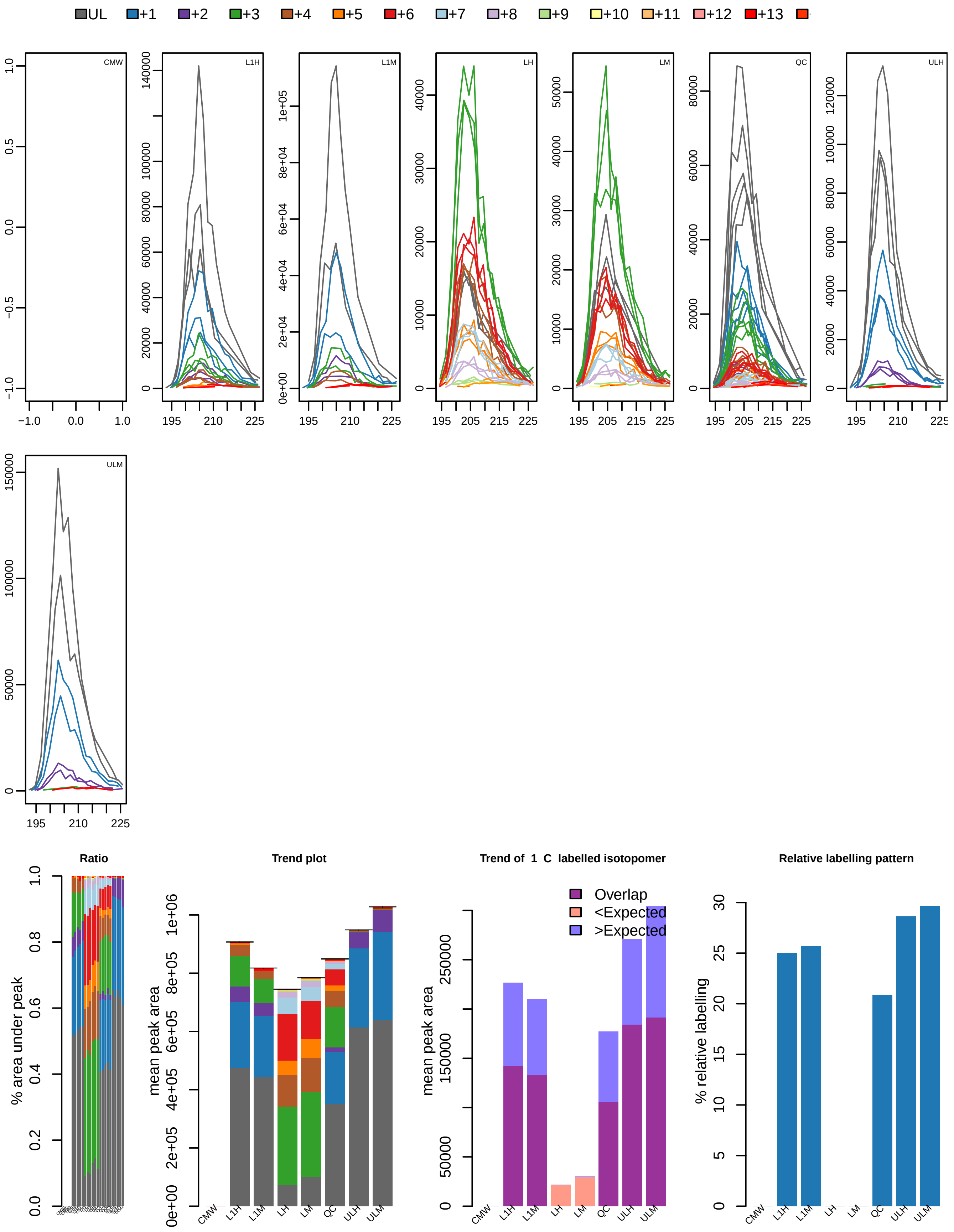

PG(16:0/20:3(5Z,8Z,11Z))

Formula: C42H77O10P Mass: 772.525 Std.RT: 204.30438162 Ion: N

G1

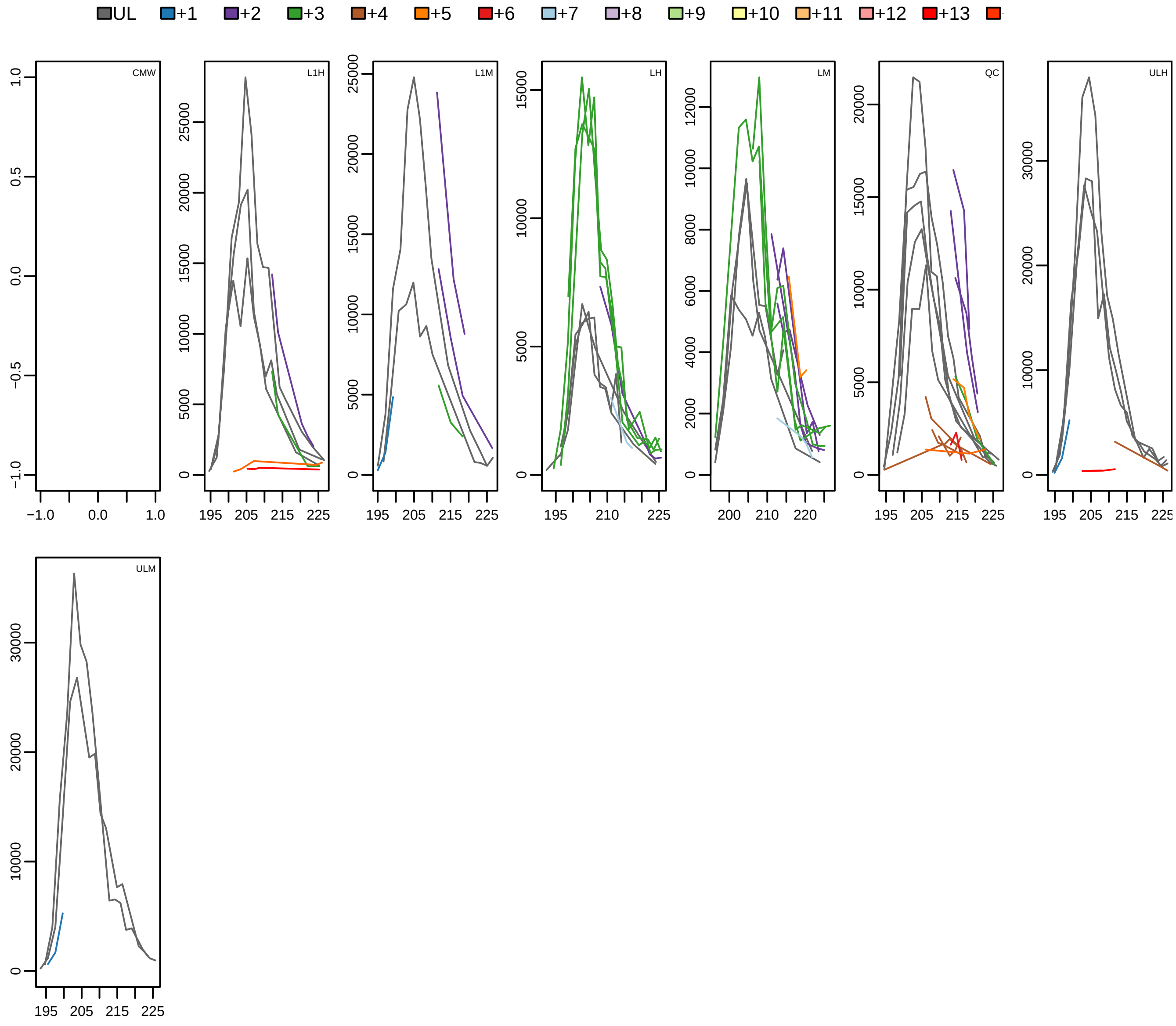

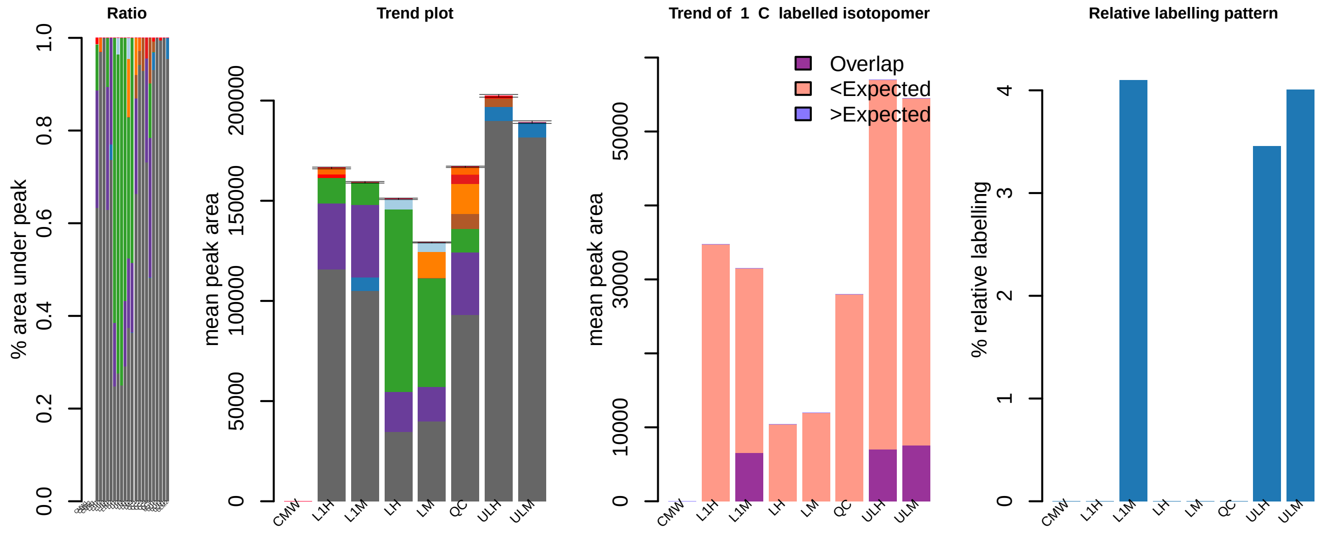

PG(18:0/22:4(7Z,10Z,13Z,16Z))

Formula: C46H83O10P Mass: 826.572 Std.RT: 204.57181002 Ion: N

G1

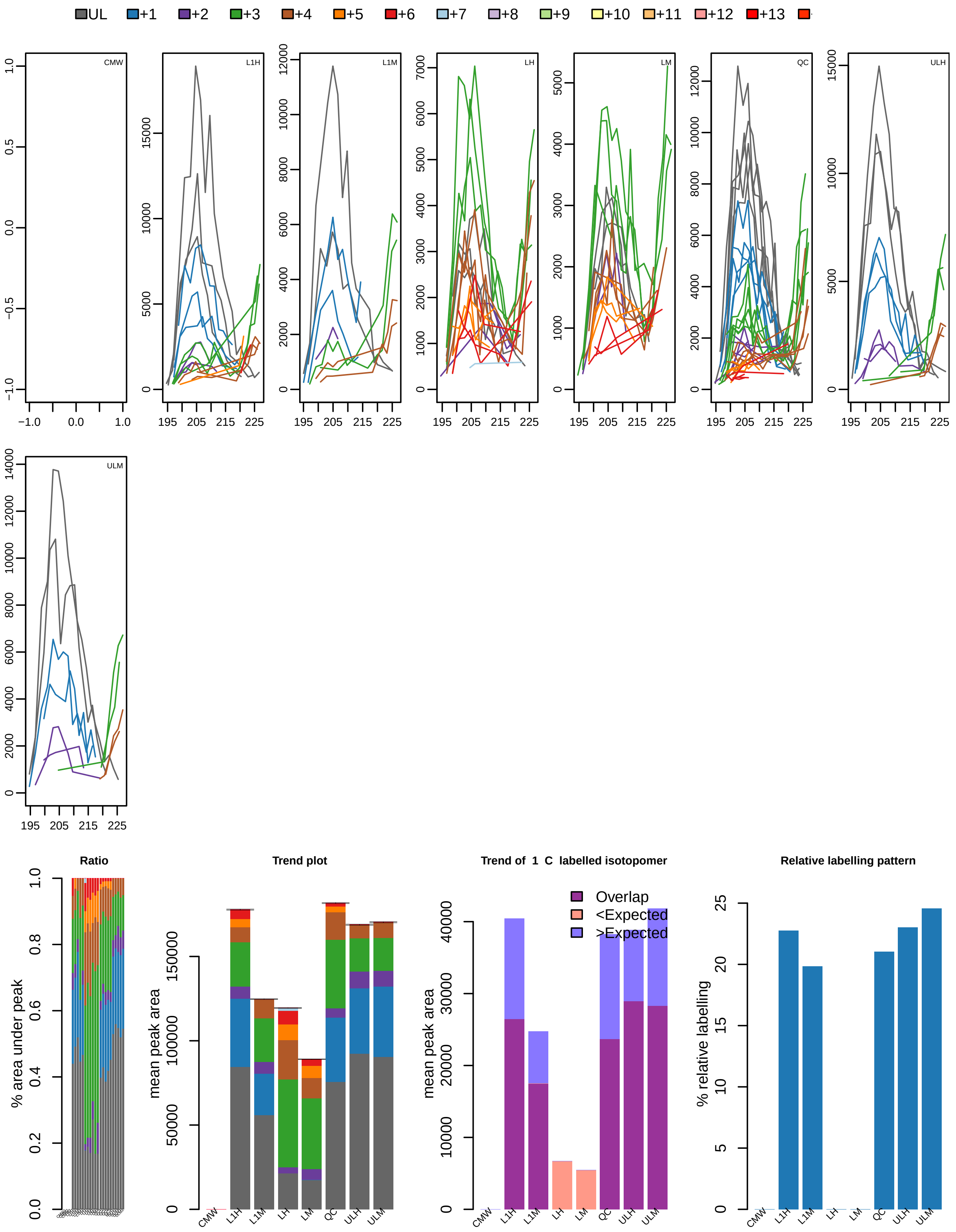

PG(18:0/22:5(4Z,7Z,10Z,13Z,16Z))

Formula: C46H81O10P Mass: 824.557 Std.RT: 203.90114898 Ion: N

G1

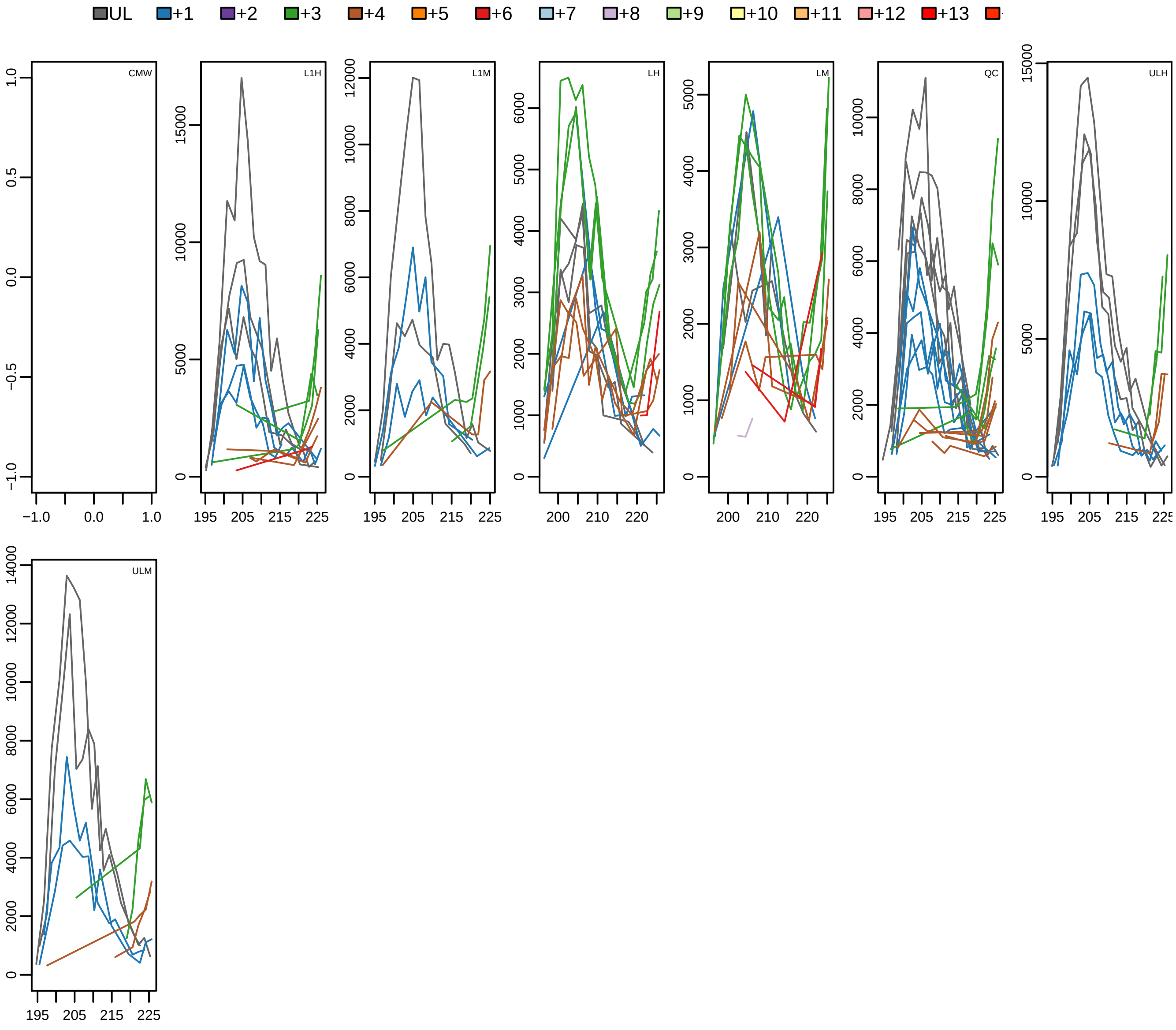

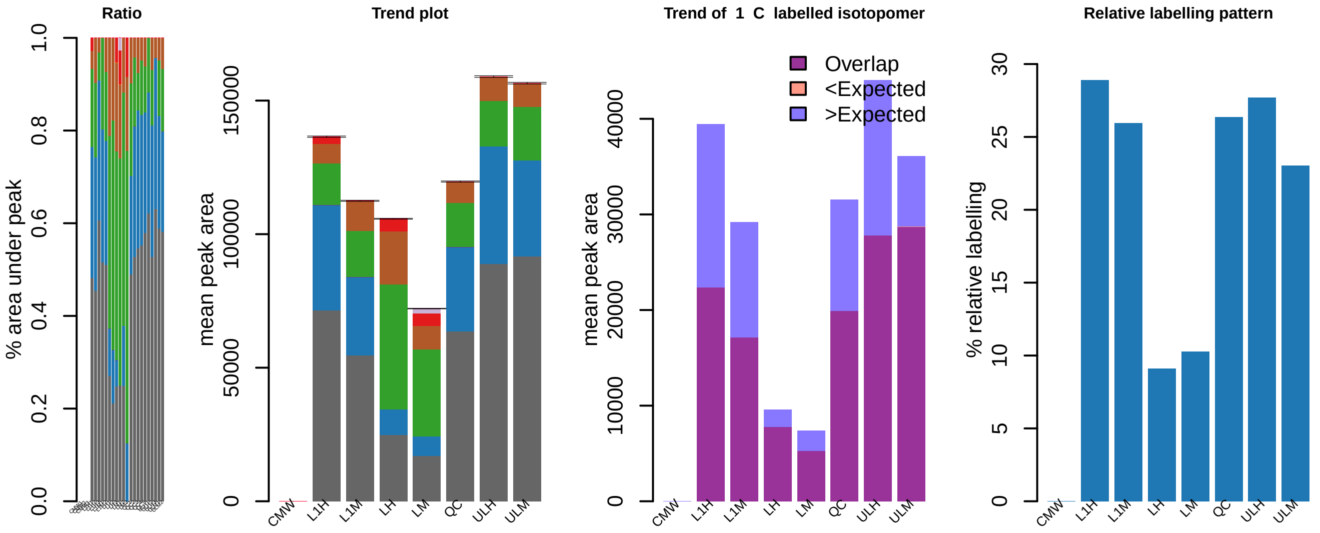

PG(18:2(9Z,12Z)/22:6(4Z,7Z,10Z,13Z,16Z,19Z))

Formula: C46H75O10P Mass: 818.51 Std.RT: 204.18290352 Ion: NE

G1

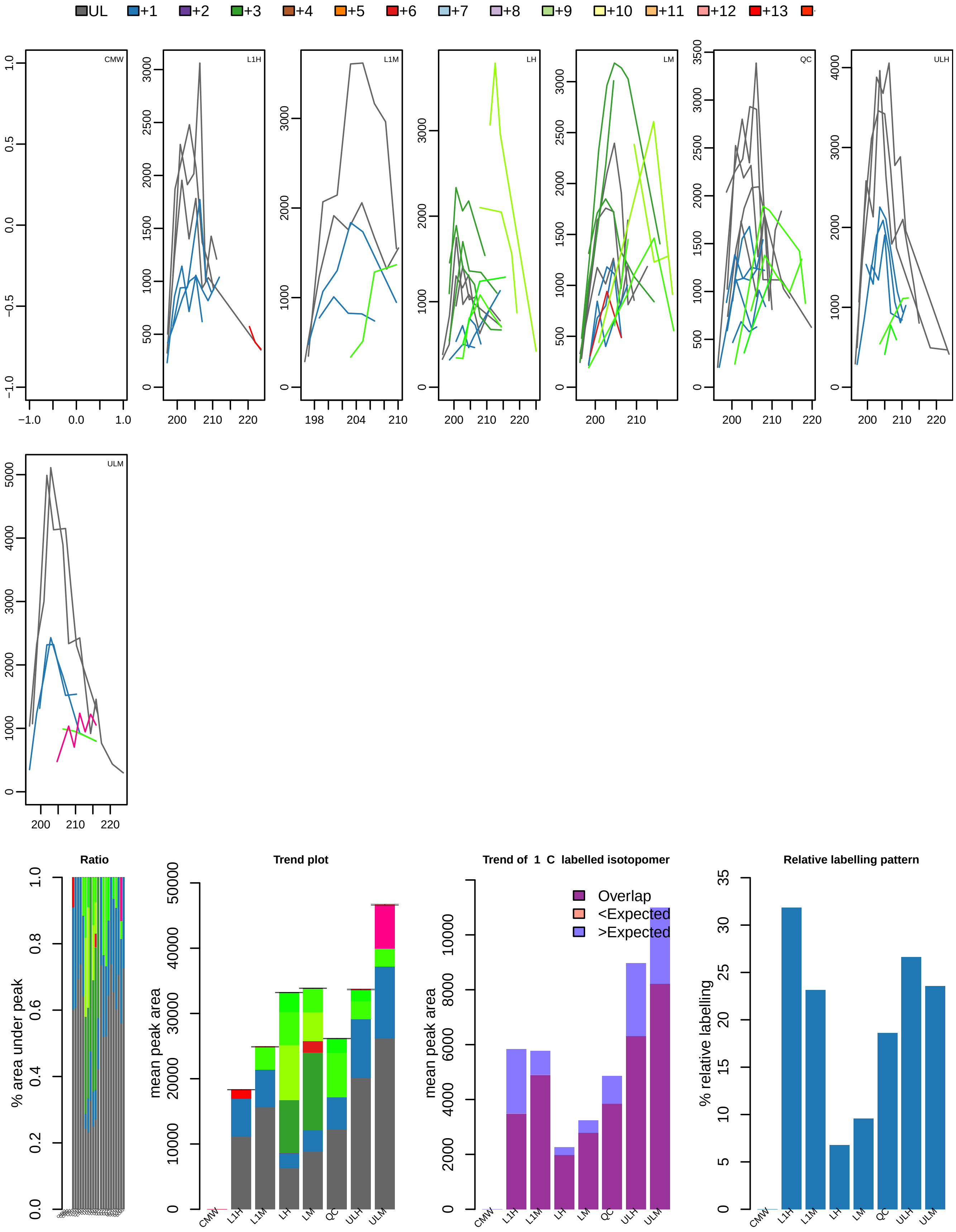

PIP(16:0/18:2(9Z,12Z))

Formula: C43H80O16P2 Mass: 914.492 Std.RT: 220.3564713 Ion: N

G1

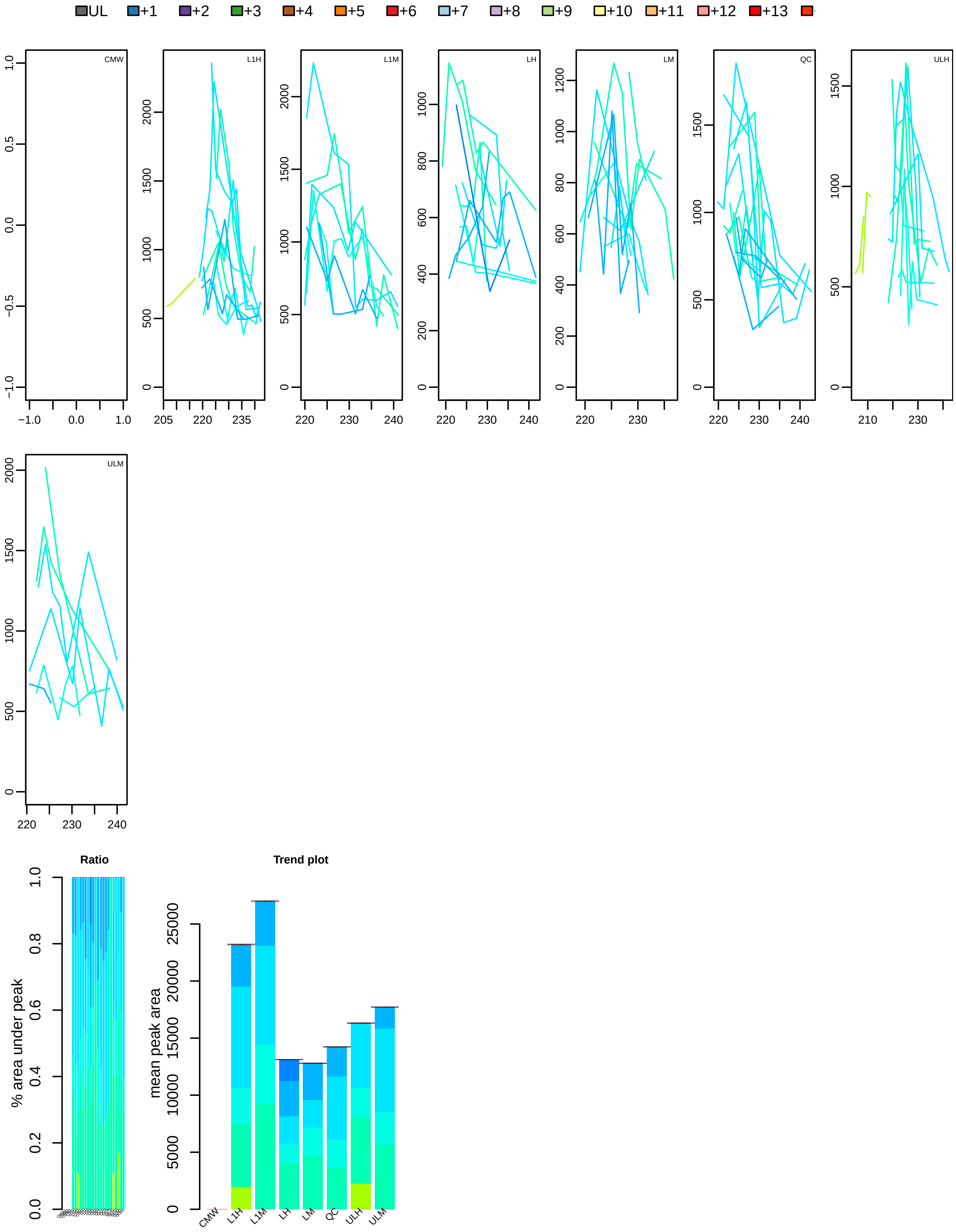

PIP(16:0/20:2(11Z,14Z))

Formula: C45H84O16P2 Mass: 942.523 Std.RT: 219.59257362 Ion: [illegible]

G1

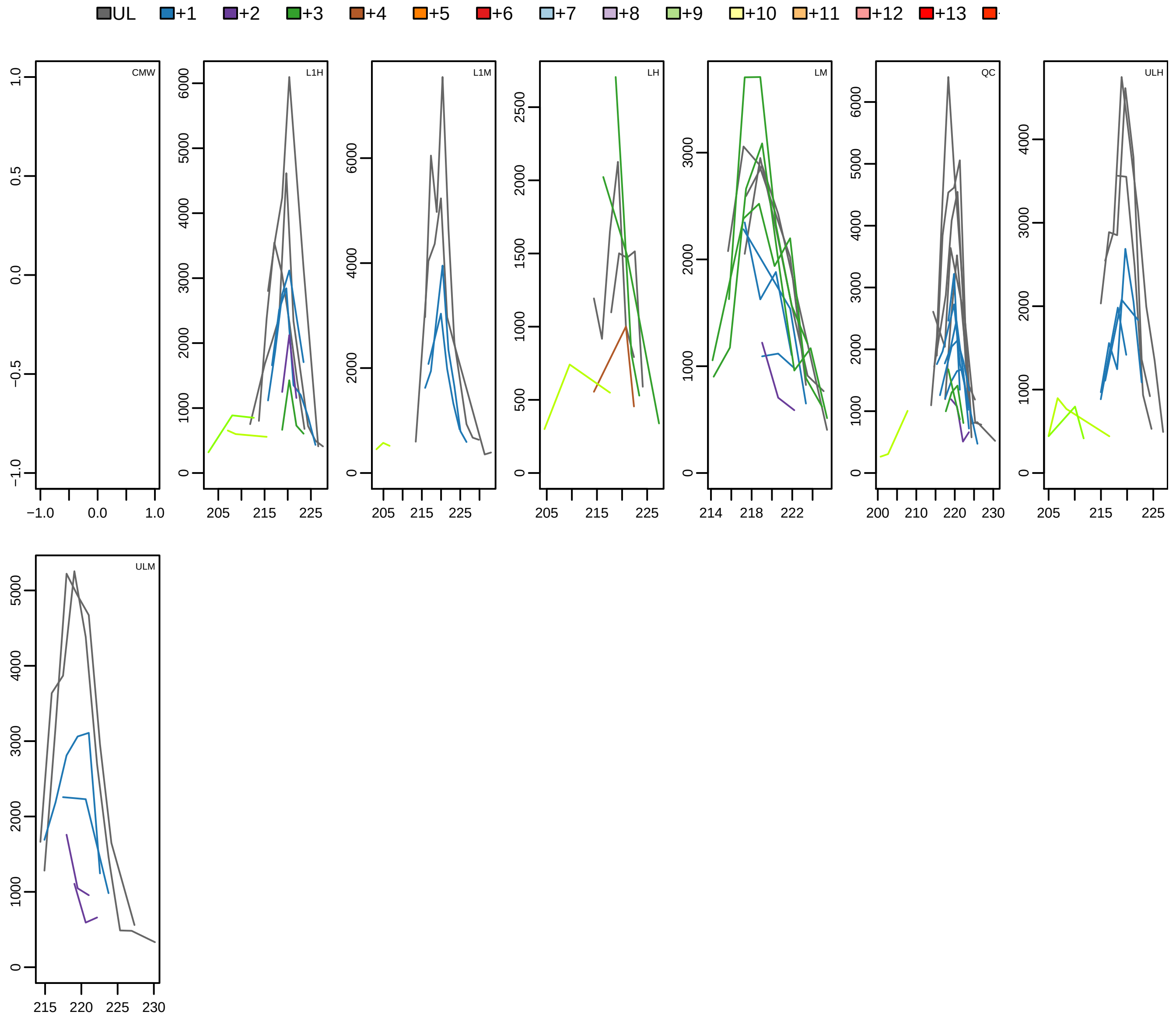

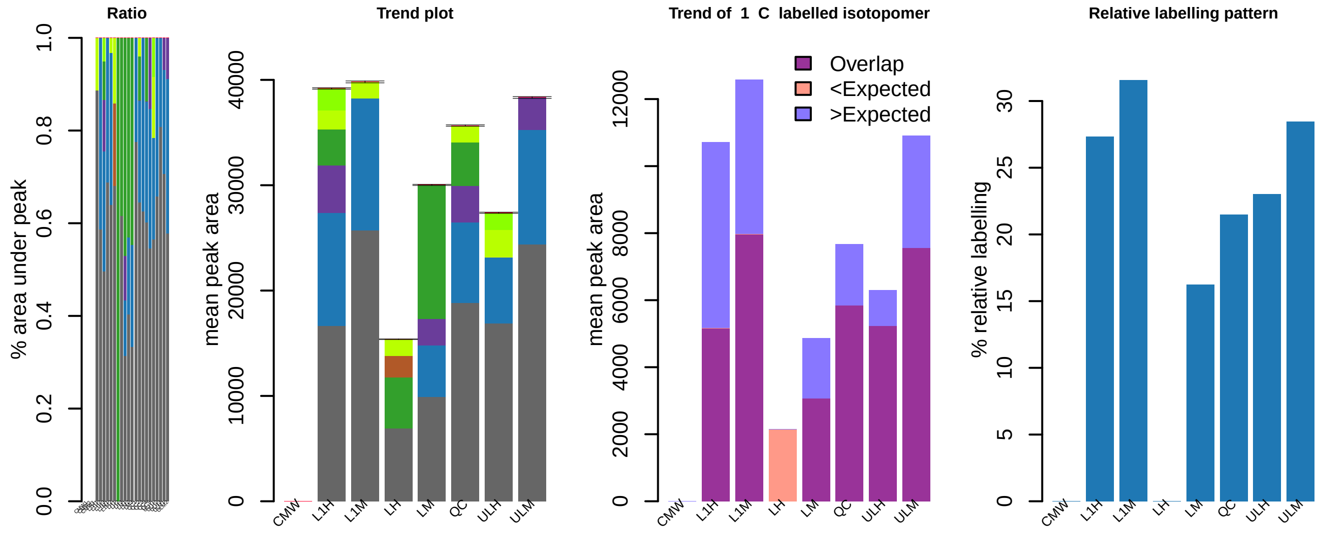

[PI (18:0/18:0)] 1,2−di−(9Z−octadecenoyl)−sn−glycero−

Formula: C45H83O13P Mass: 862.557 Std.RT: 211.03242858 Ion: N

G1

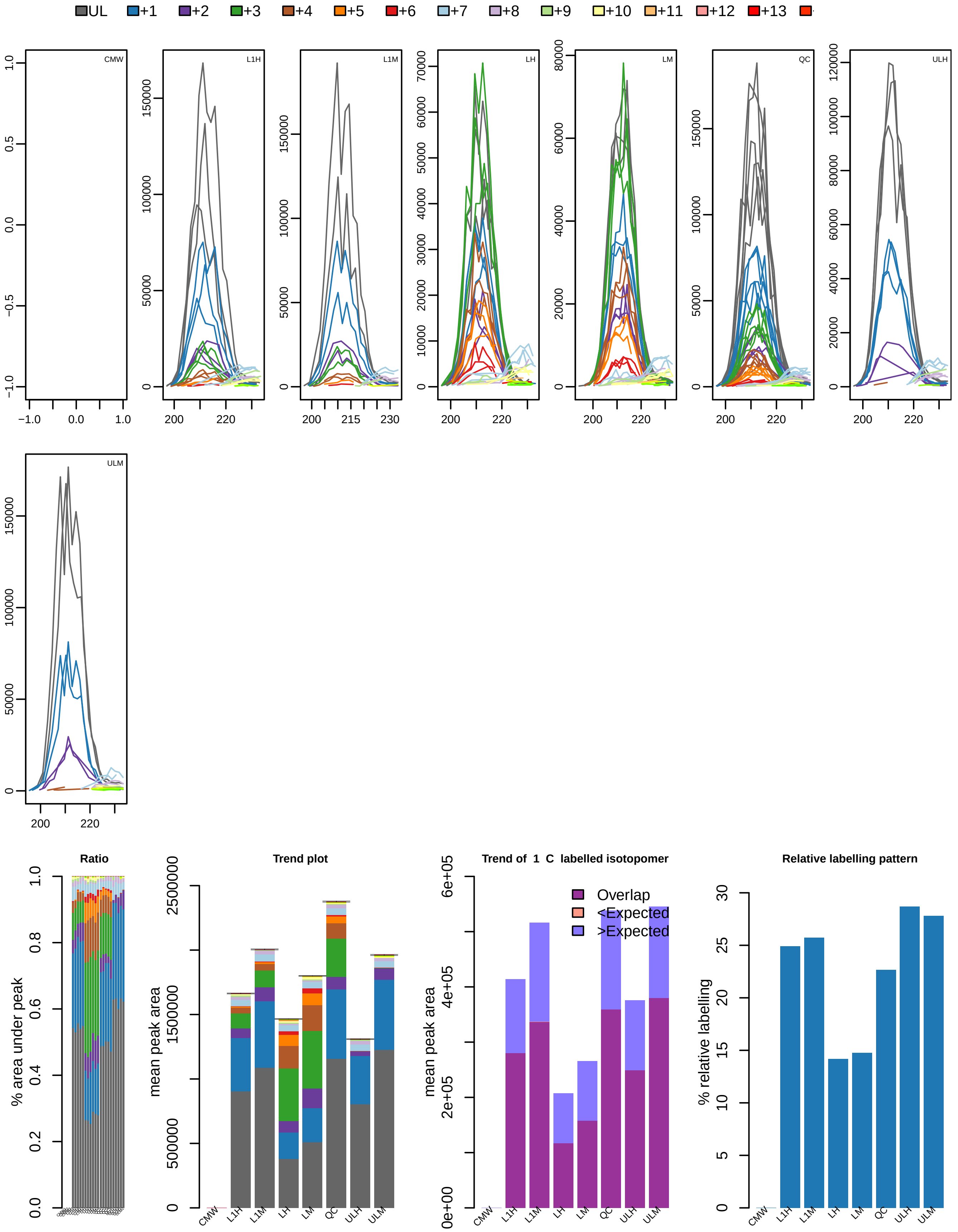

PI(16:0/20:4(5Z,8Z,11Z,14Z))

Formula: $C_{45}H_{79}O_{13}P$ Mass: 858.526 Std.RT: 210.81842772 Ion: N

G1

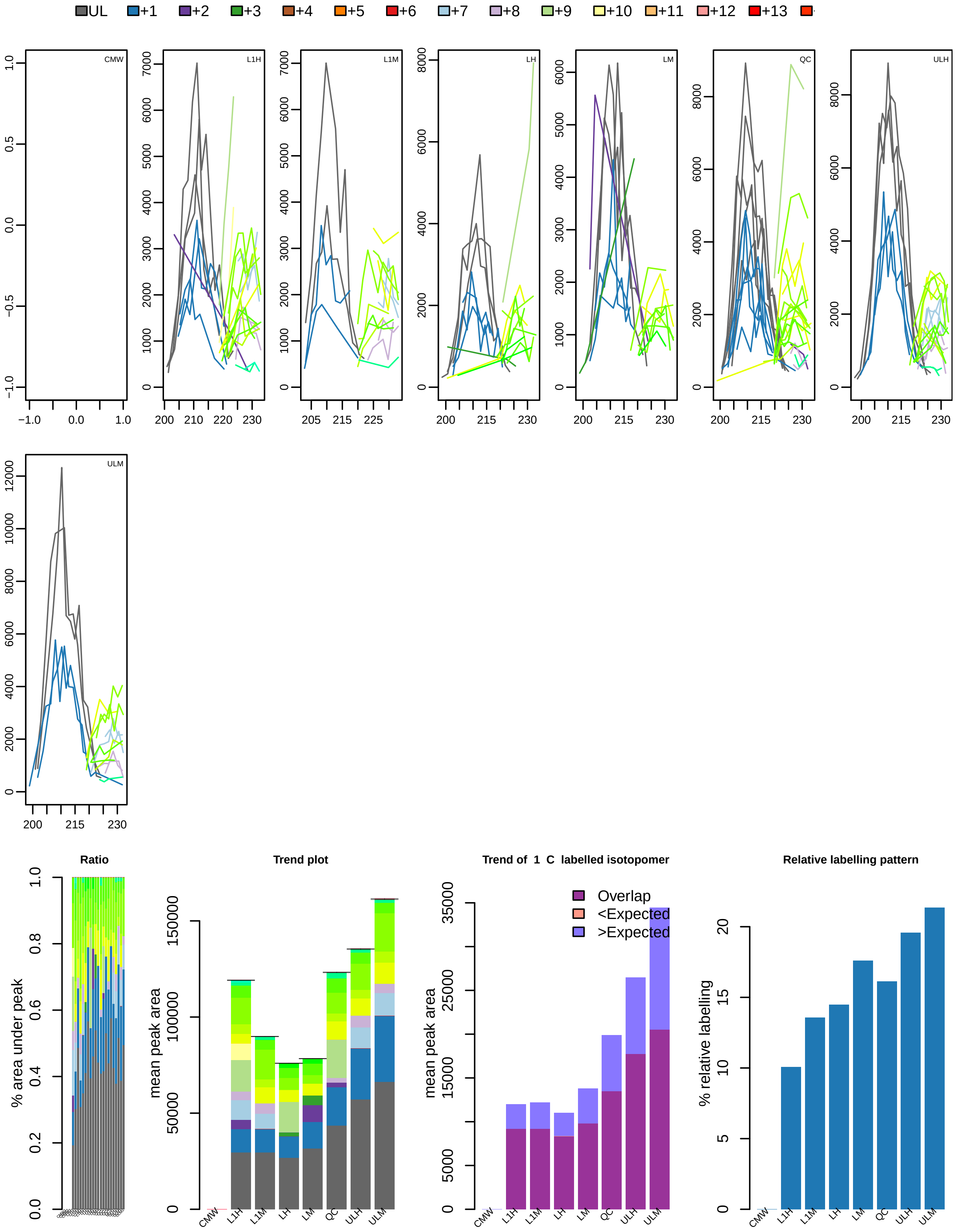

PI(16:0/20:3(5Z,8Z,11Z))

Formula: C45H81O13P Mass: 860.541 Std.RT: 210.80304612 Ion: N

G1

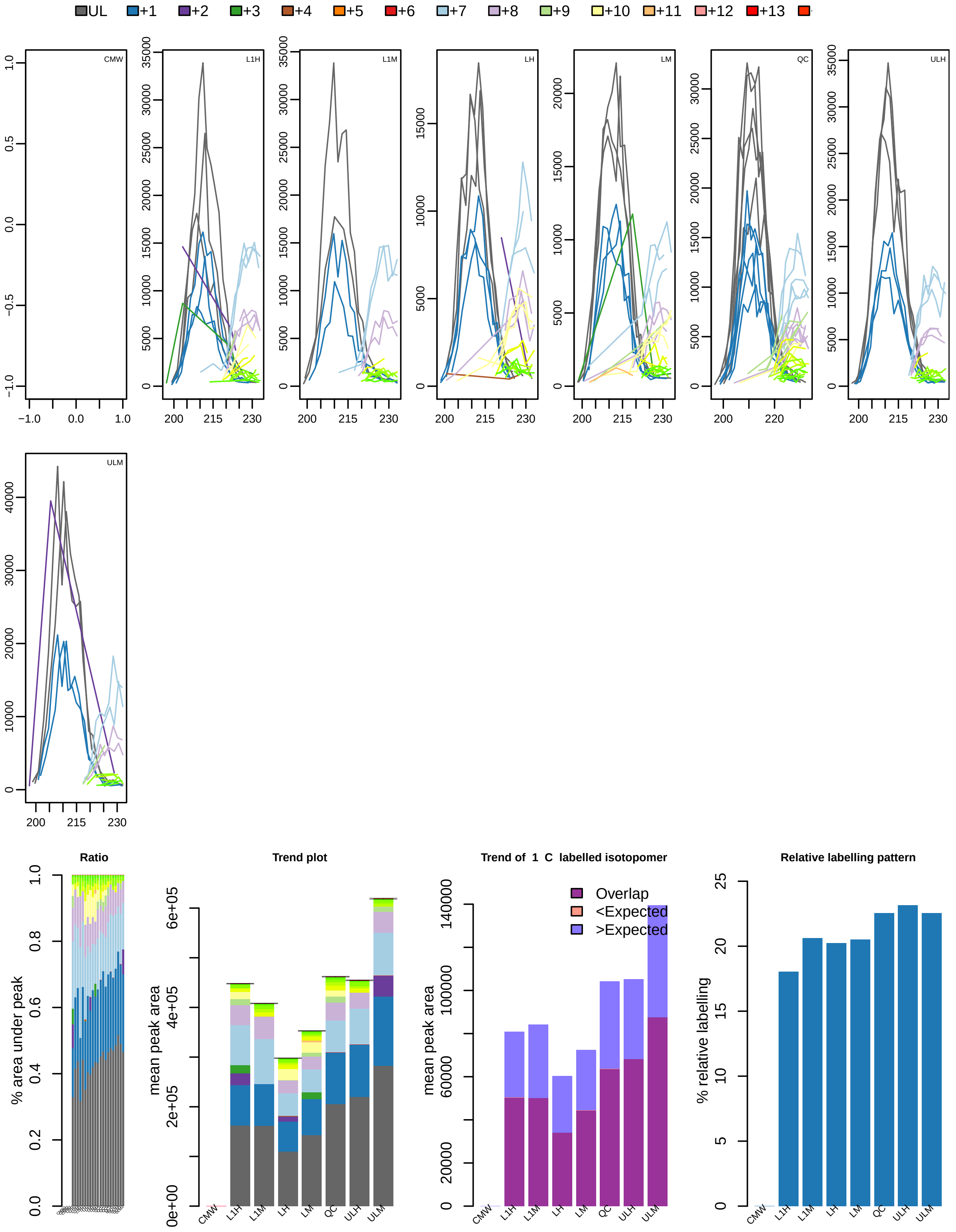

PI(18:0/22:6(4Z,7Z,10Z,13Z,16Z,19Z))

G1

Formula: C49H83O13P Mass: 910.557 Std.RT: 210.3555225 Ion: NE

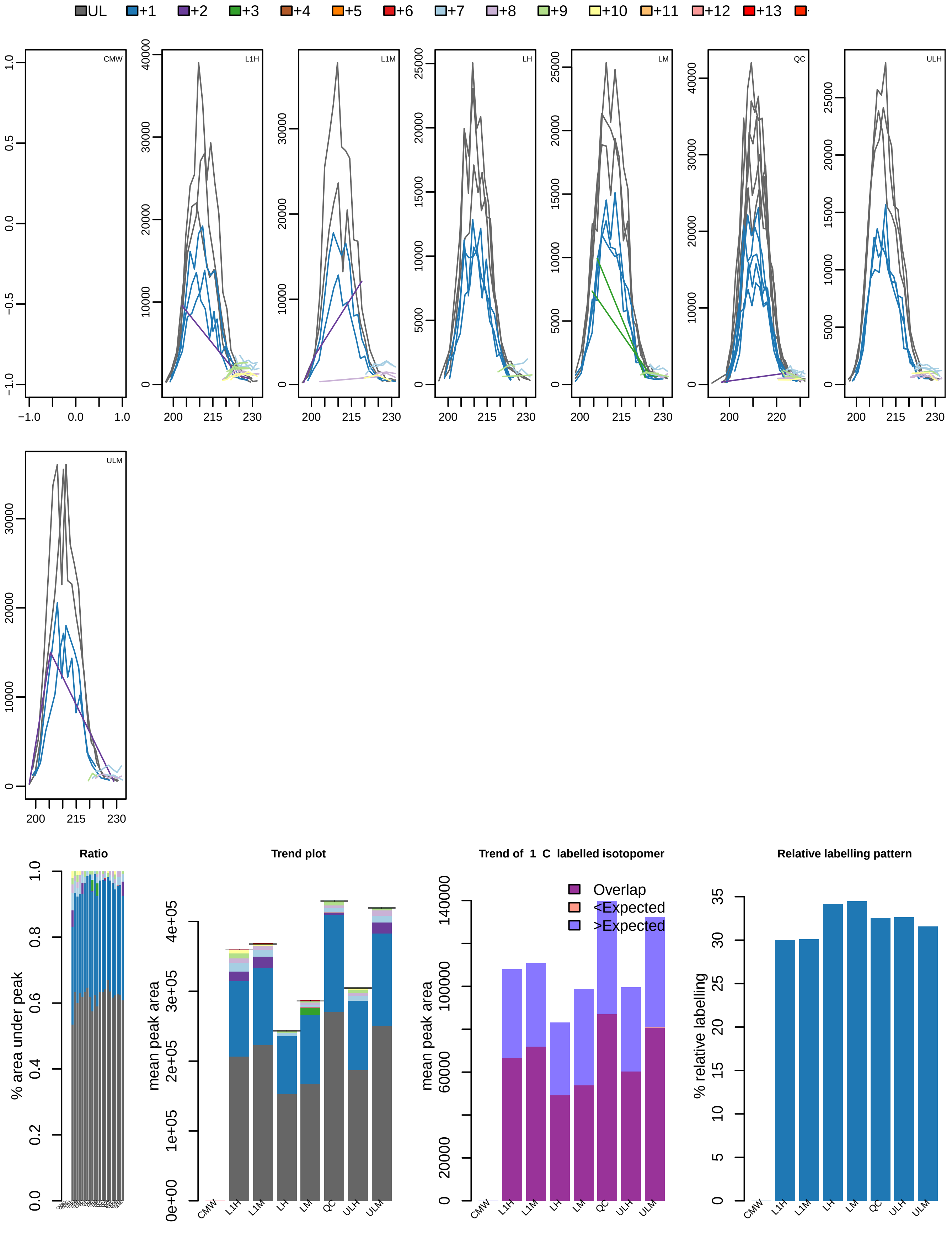

[SP (16:0)] N−(hexadecanoyl)−sphing−4−enine−1−pho[illegible]
Formula: C34H68NO6P Mass: 617.478 Std.RT: 243.92056932 Ion: [illegible]

G1

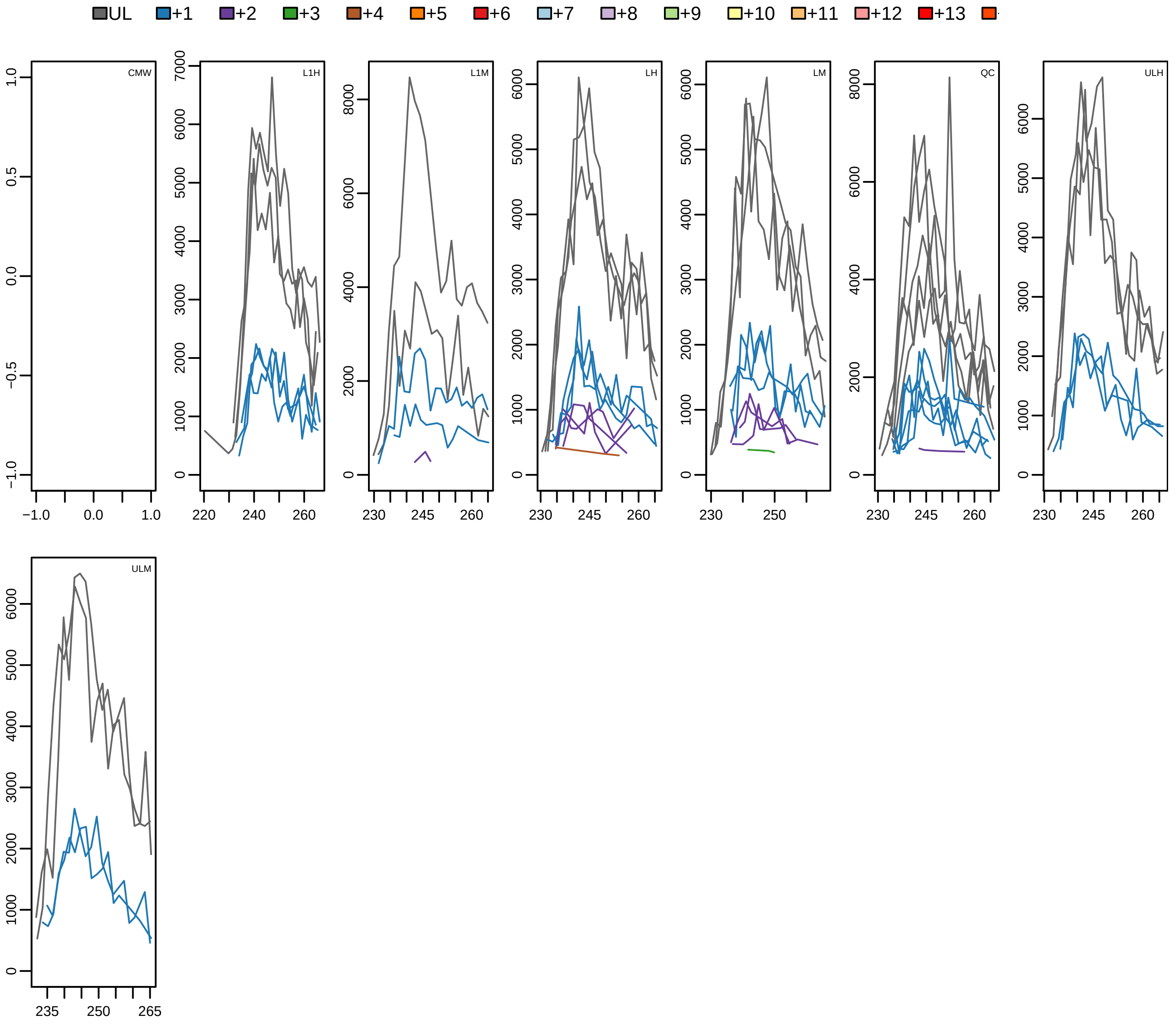

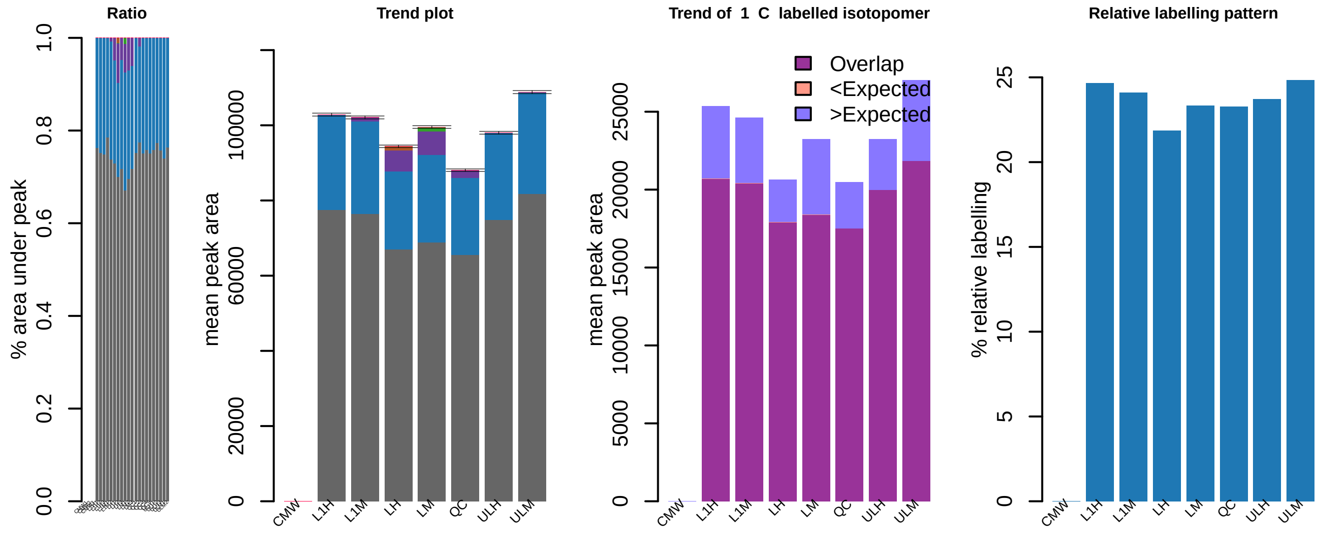

Cholesterolsulfate

Formula: C27H46O4S Mass: 466.312 Std.RT: 205.347096 Ion: NEG

G1

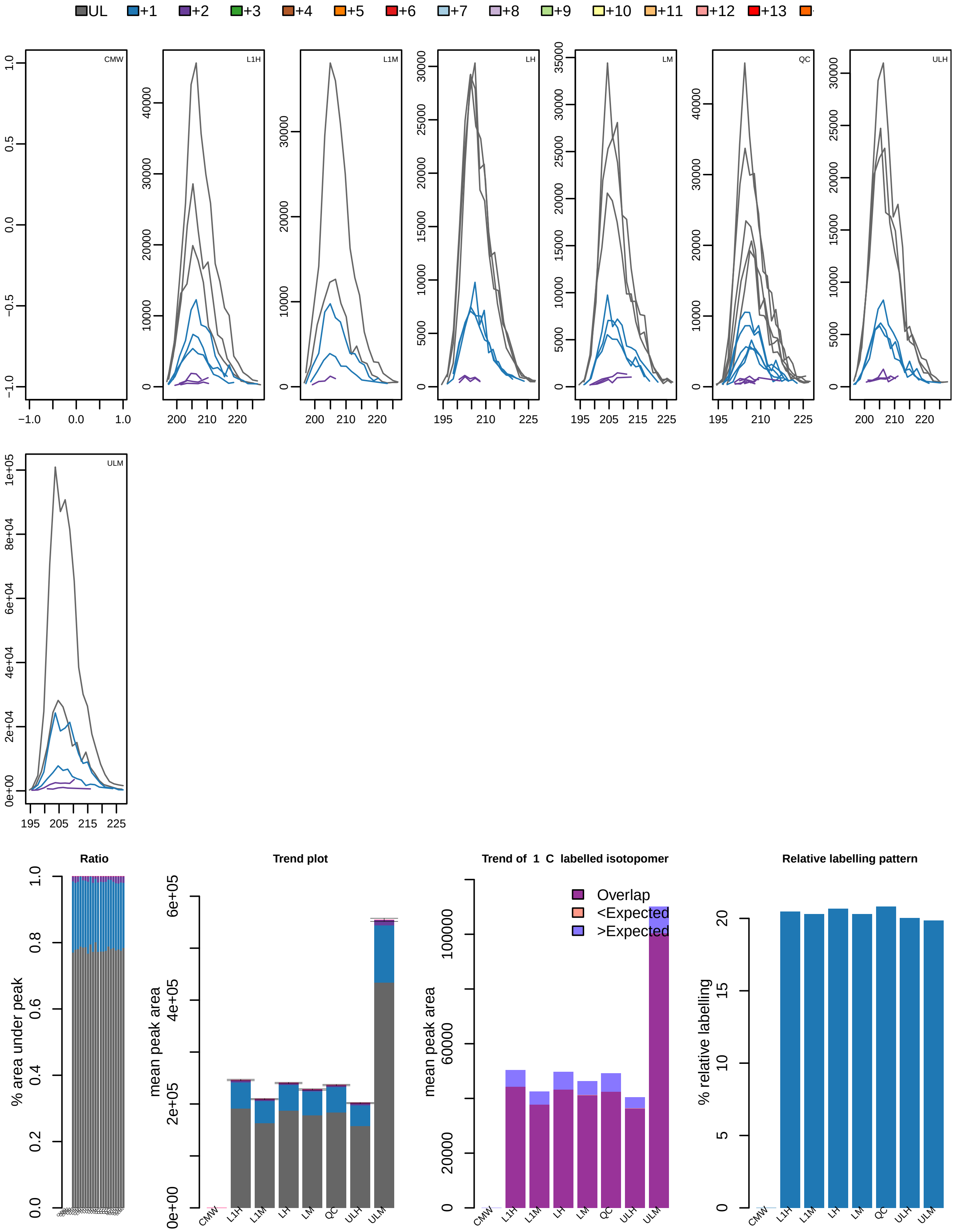

[ST hydrox] N−(3alpha,7alpha−dihydroxy−5beta−cholar

G1

Formula: C26H45NO6S Mass: 499.297 Std.RT: 234.46376178 Ion: N

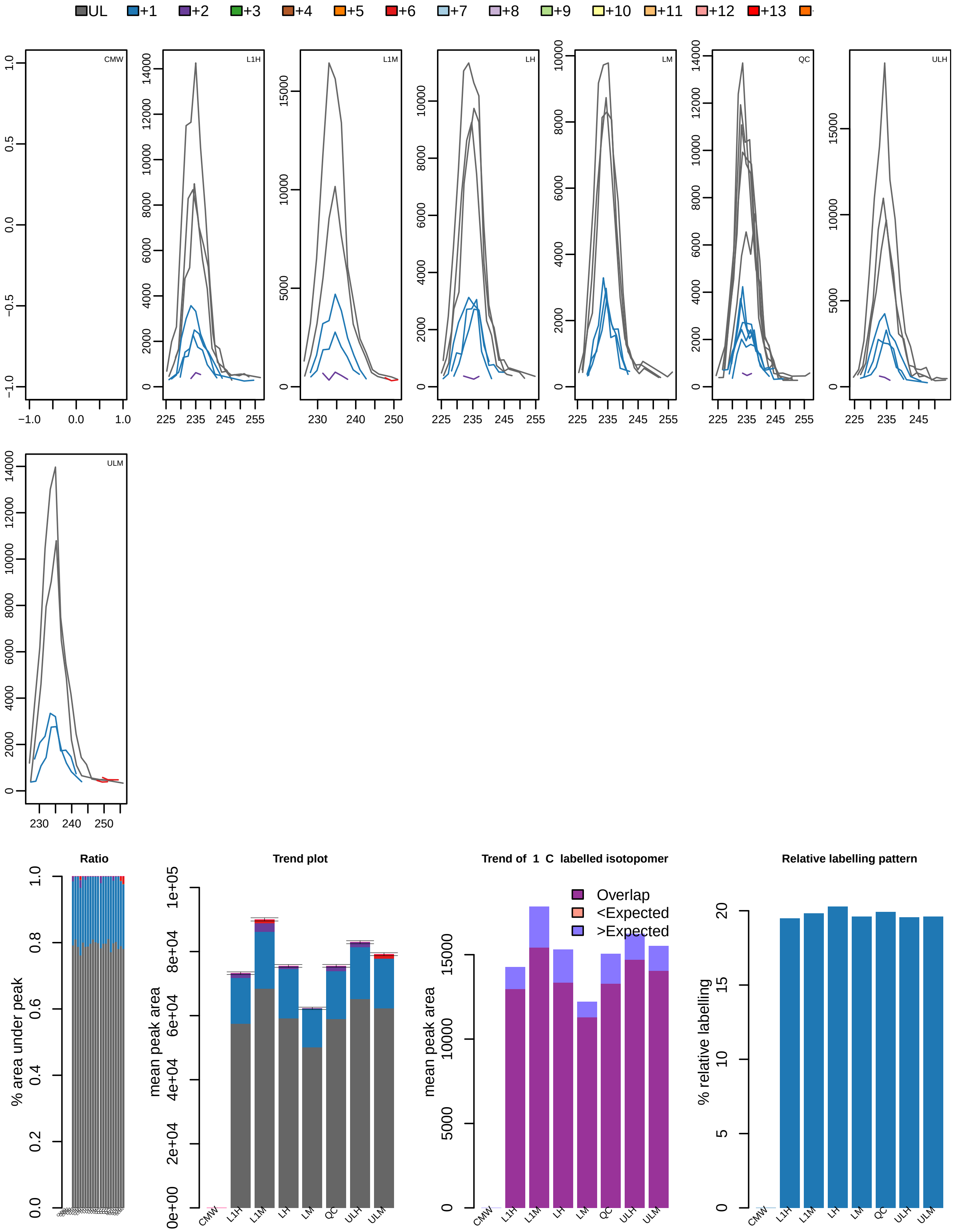

Cholate

Formula: C24H40O5 Mass: 408.288 Std.RT: 267.42790728 Ion: NEG

G1

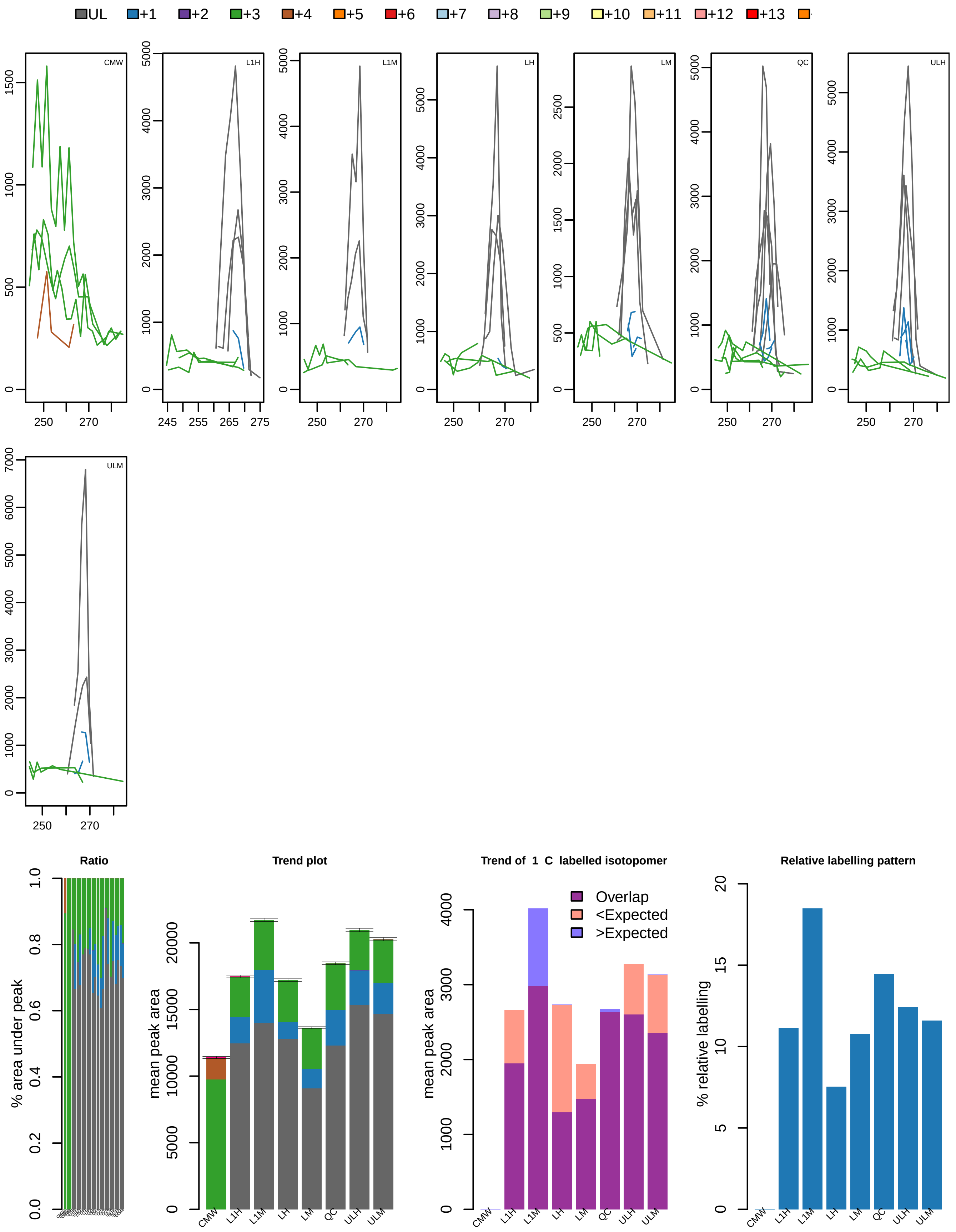

a Cysteine adduct

Formula: C6H9NO4S Mass: 191.025 Std.RT: 961.7219994 Ion: NEG

G1

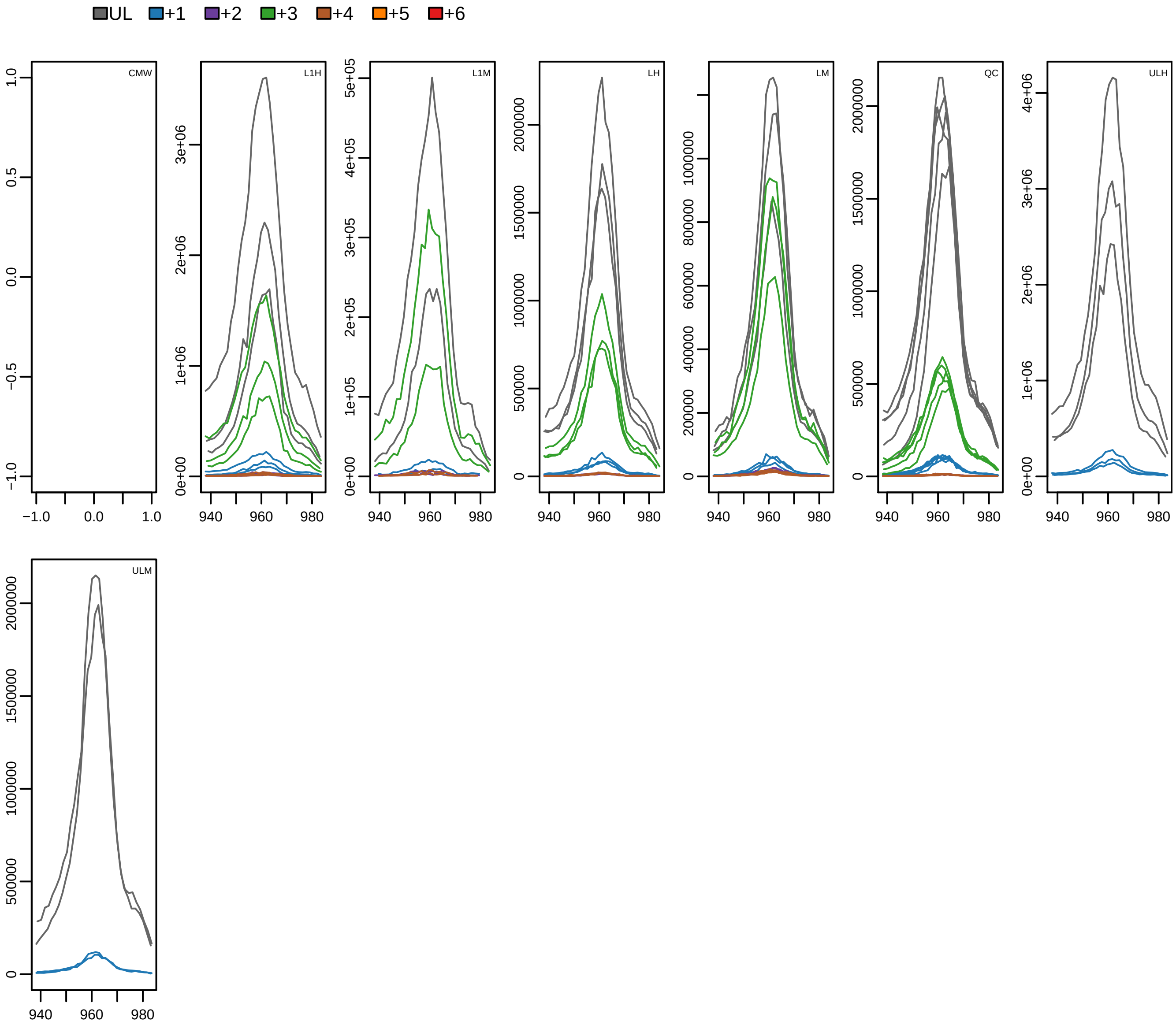

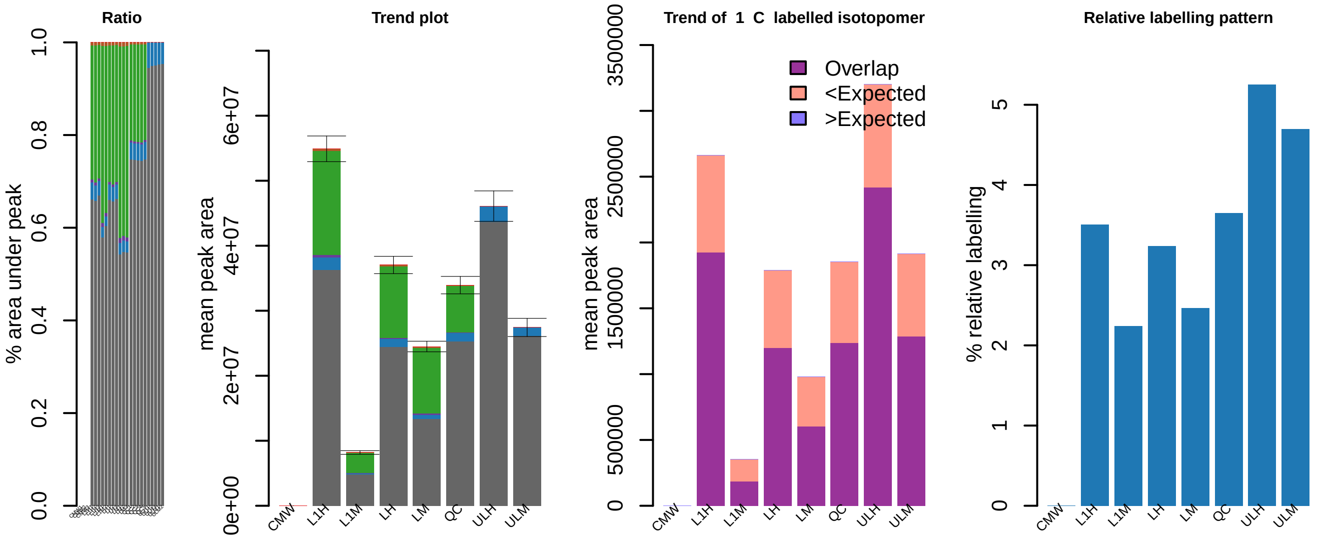

2,3,6−Trihydroxypyridine

Formula: C5H5NO3 Mass: 127.027 Std.RT: 633.1613796 Ion: NEG

G1

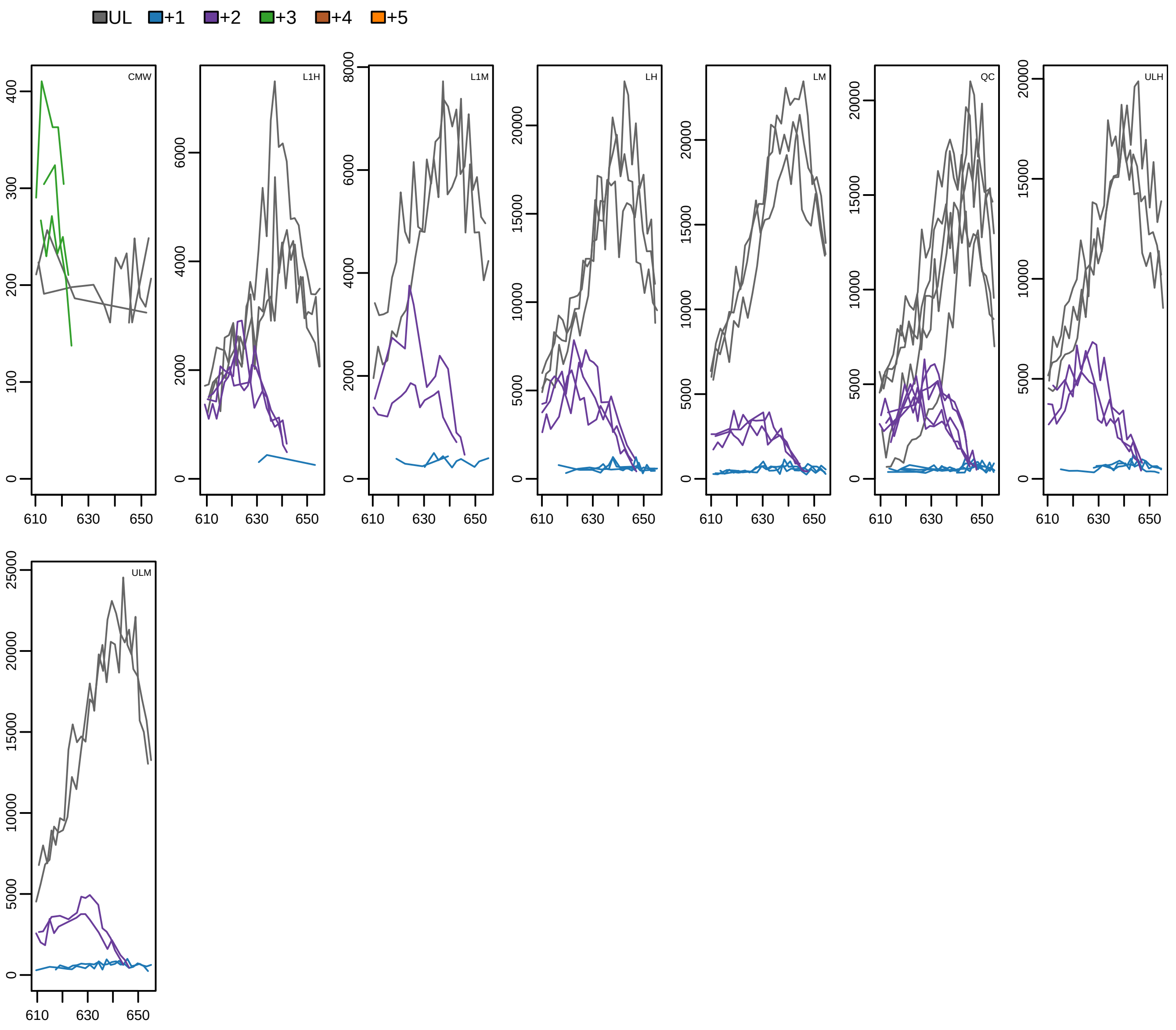

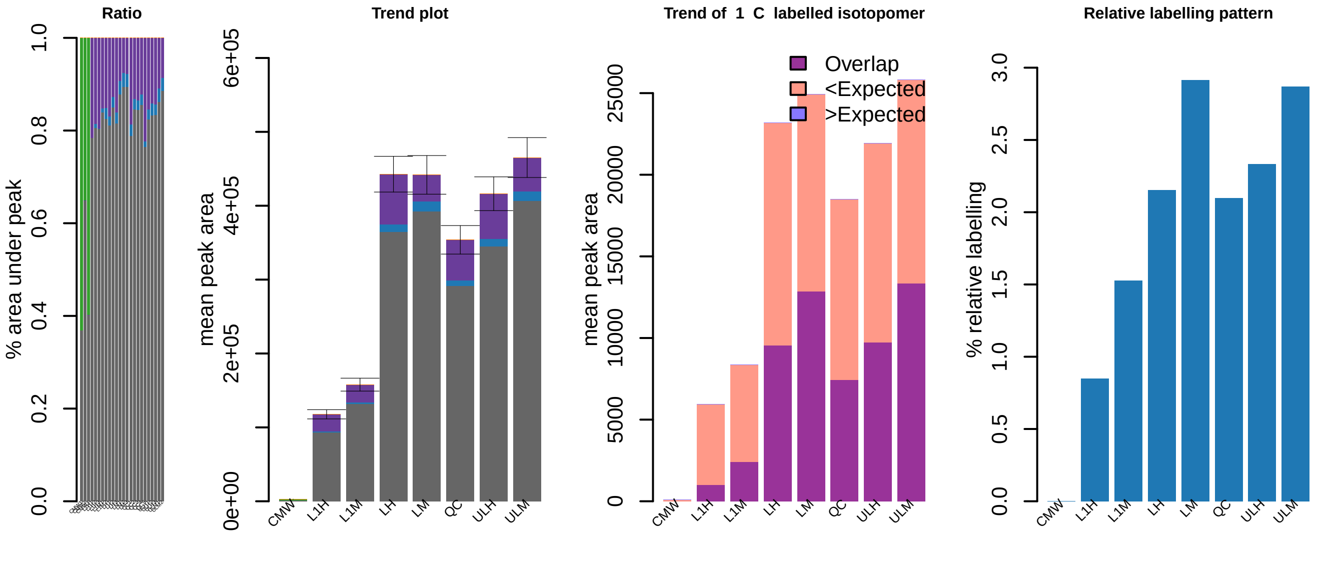

Folate

Formula: C19H19N7O6 Mass: 441.14 Std.RT: 1070.5807152 Ion: NE

G1

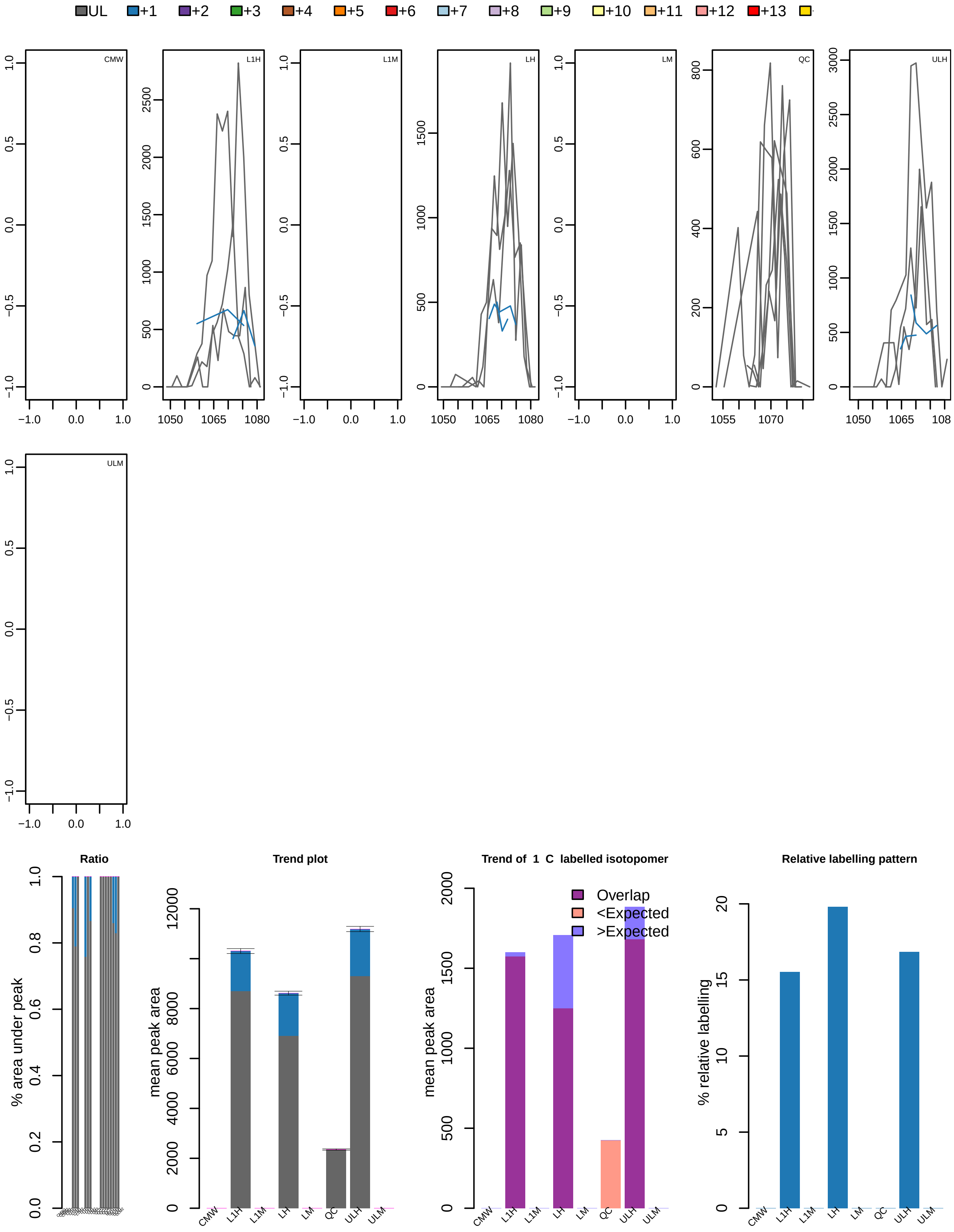

Allantoin

Formula: C4H6N4O3 Mass: 158.044 Std.RT: 879.030291 Ion: NEG

G1

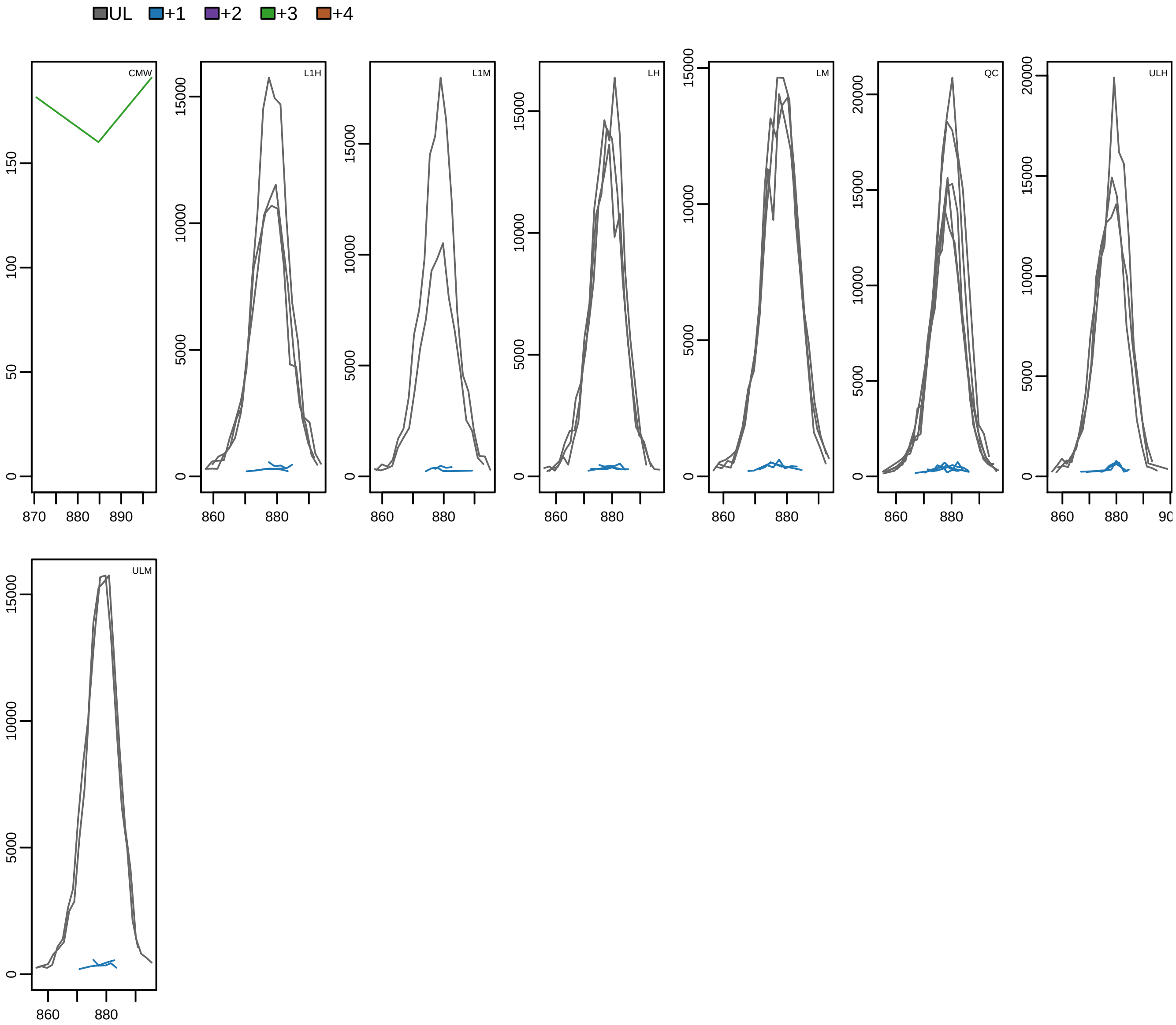

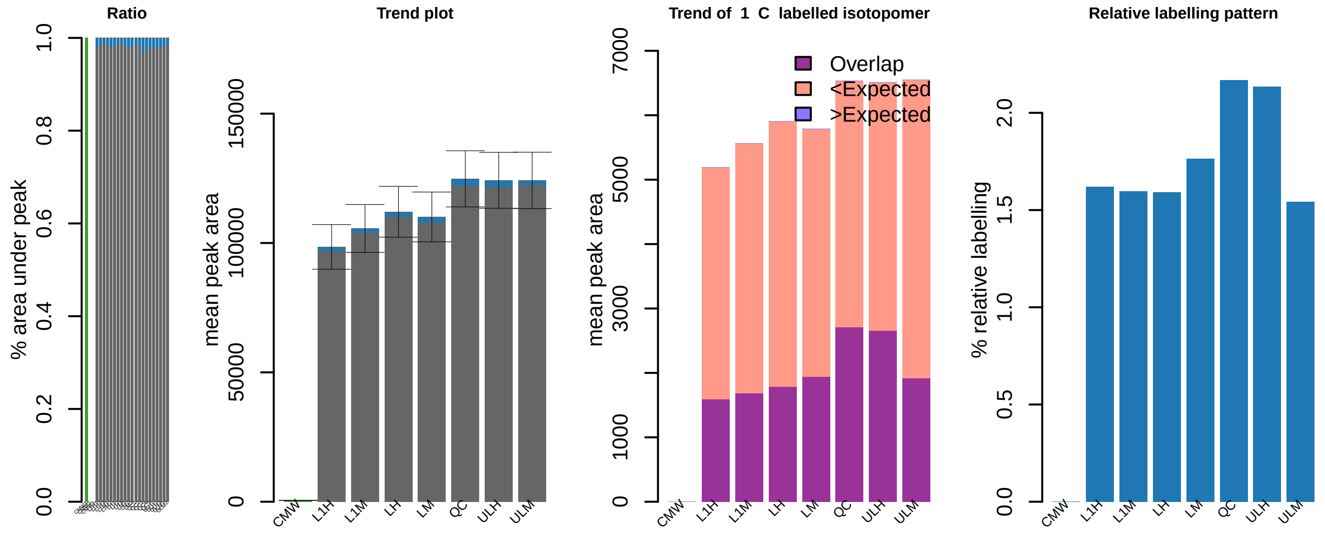

GDP

Formula: C10H15N5O11P2 Mass: 443.024 Std.RT: 1148.0095158 Ior

G1

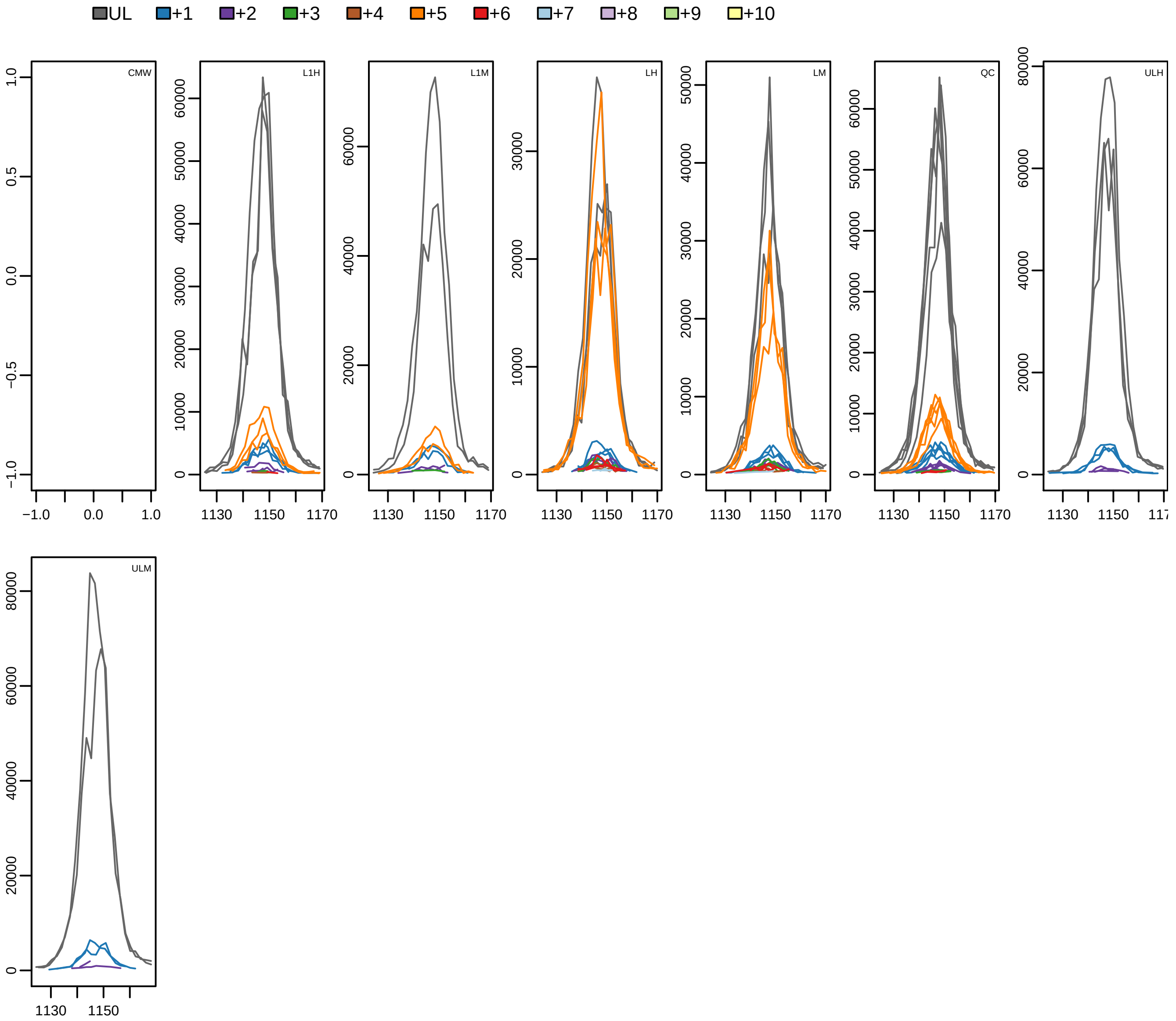

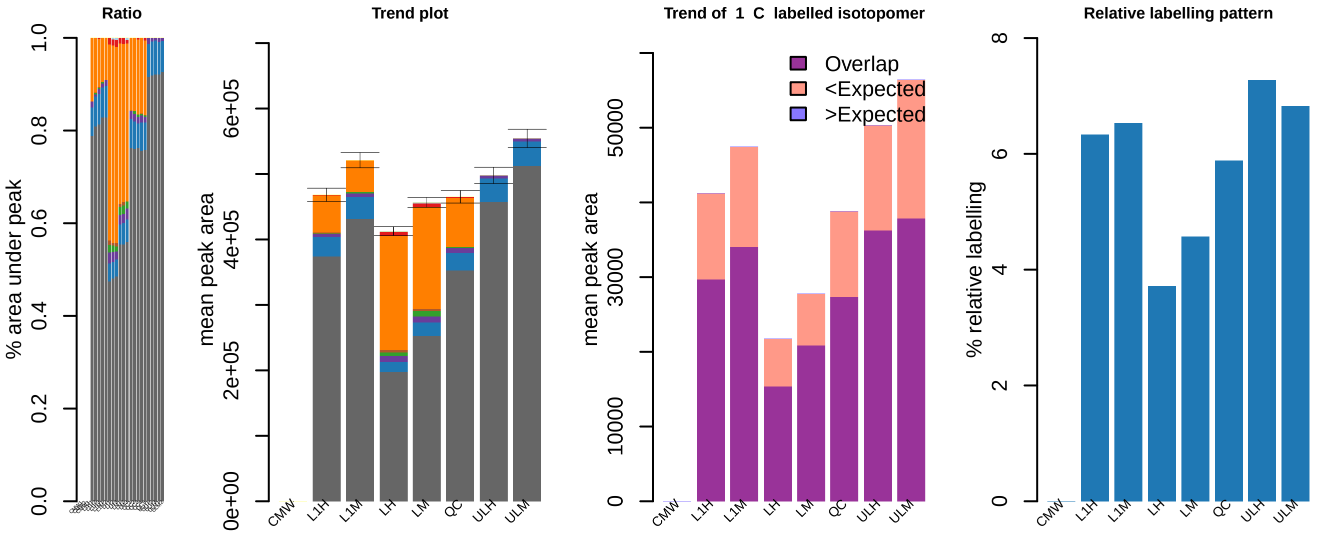

Urate

Formula: C5H4N4O3 Mass: 168.028 Std.RT: 790.9528548 Ion: NEG

G1

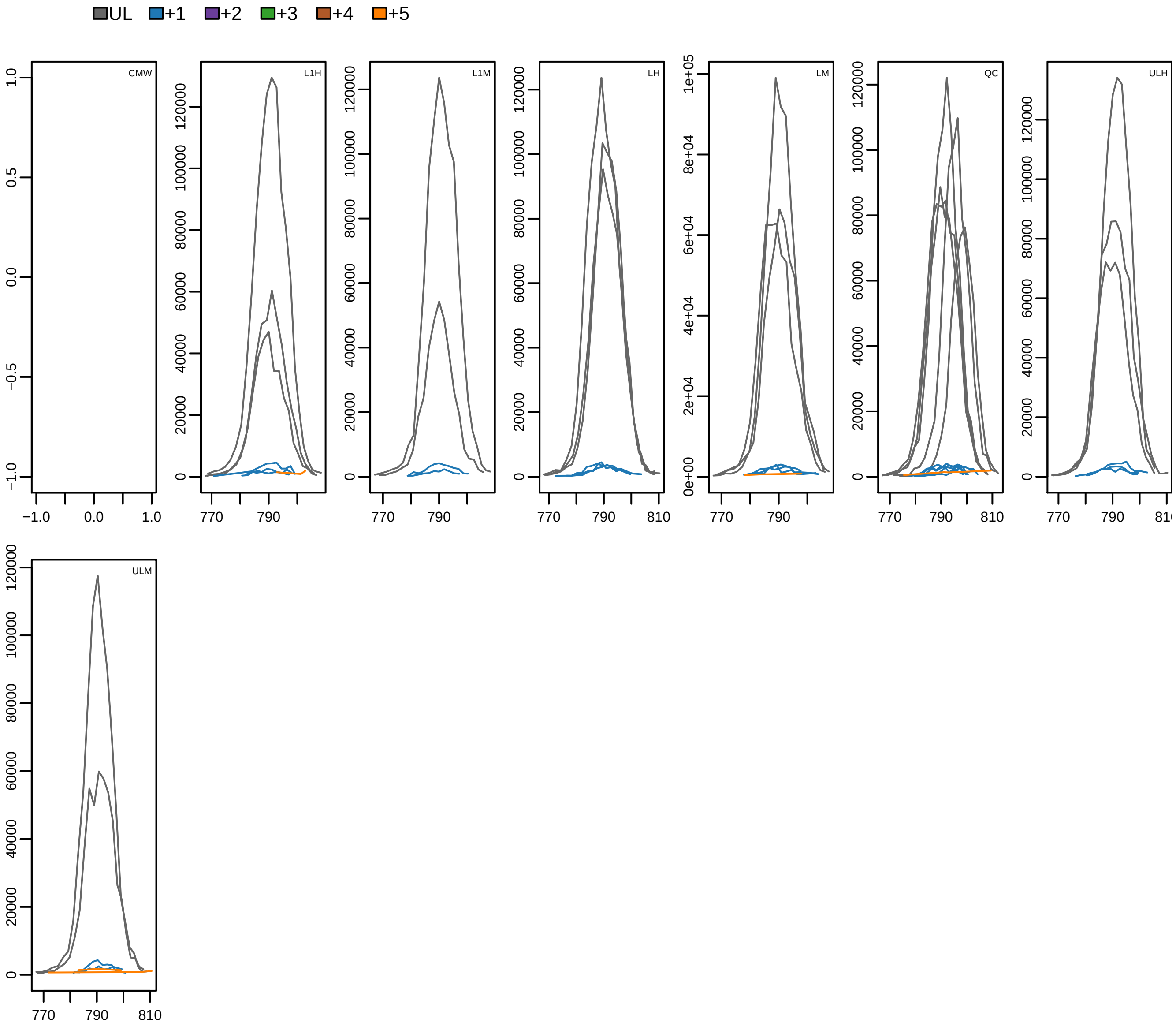

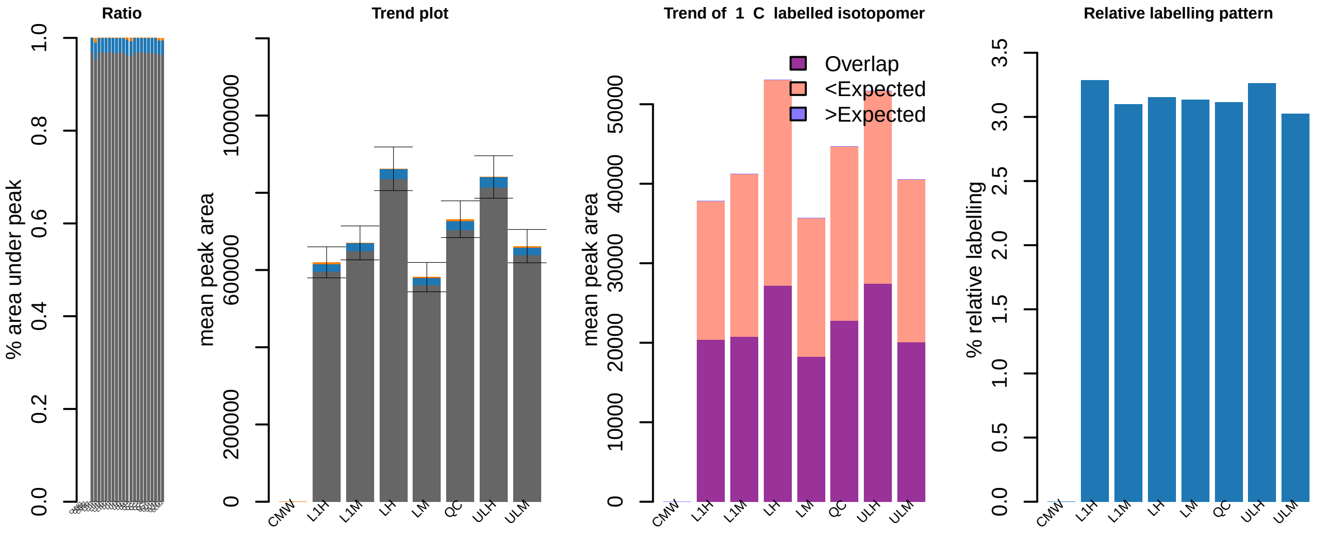

N6−(1,2−Dicarboxyethyl)−AMP

Formula: C14H18N5O11P Mass: 463.074 Std.RT: 1158.1461942 Ion:

G1

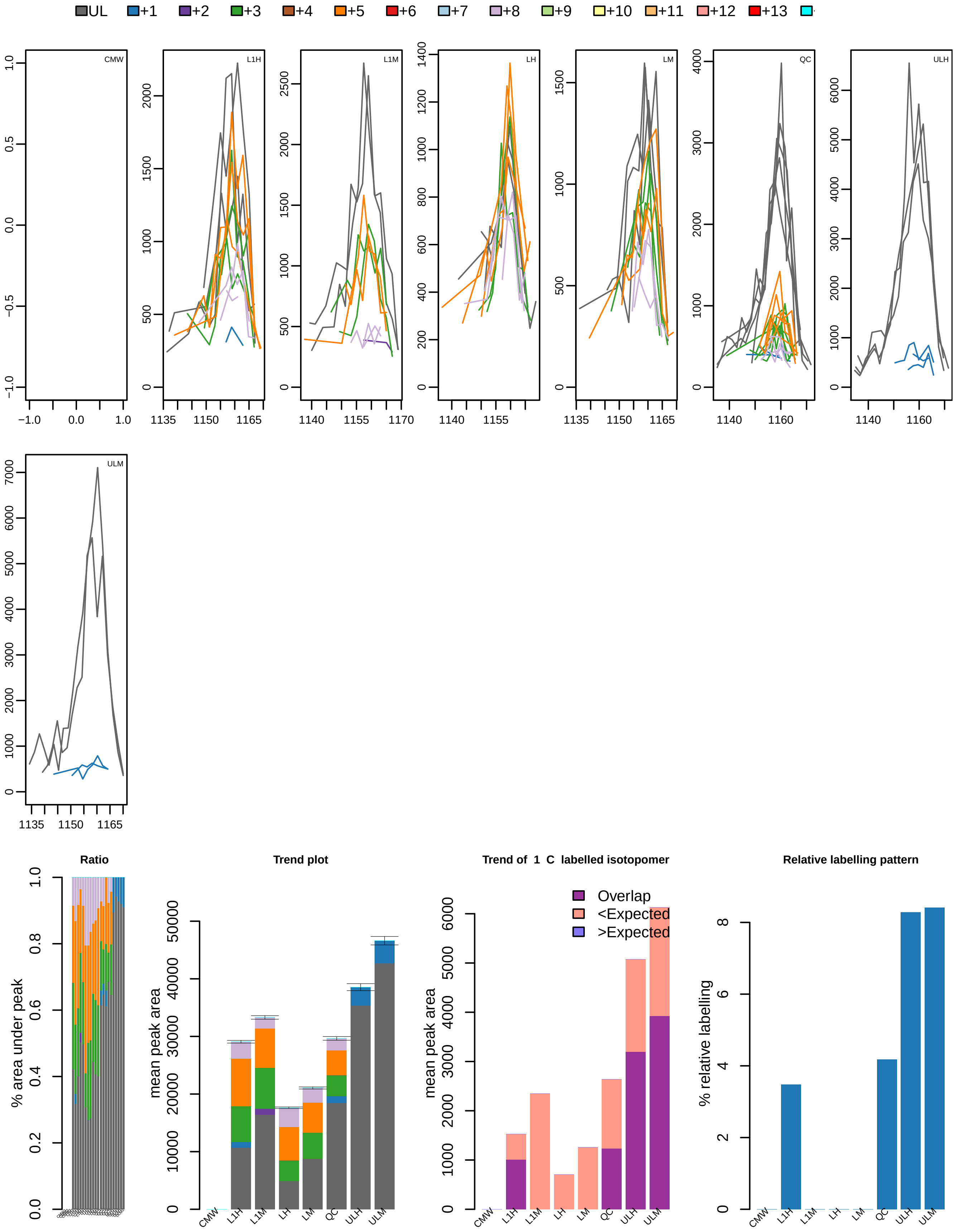

Xanthine

Formula: C5H4N4O2 Mass: 152.033 Std.RT: 714.4911384 Ion: NEG

G1

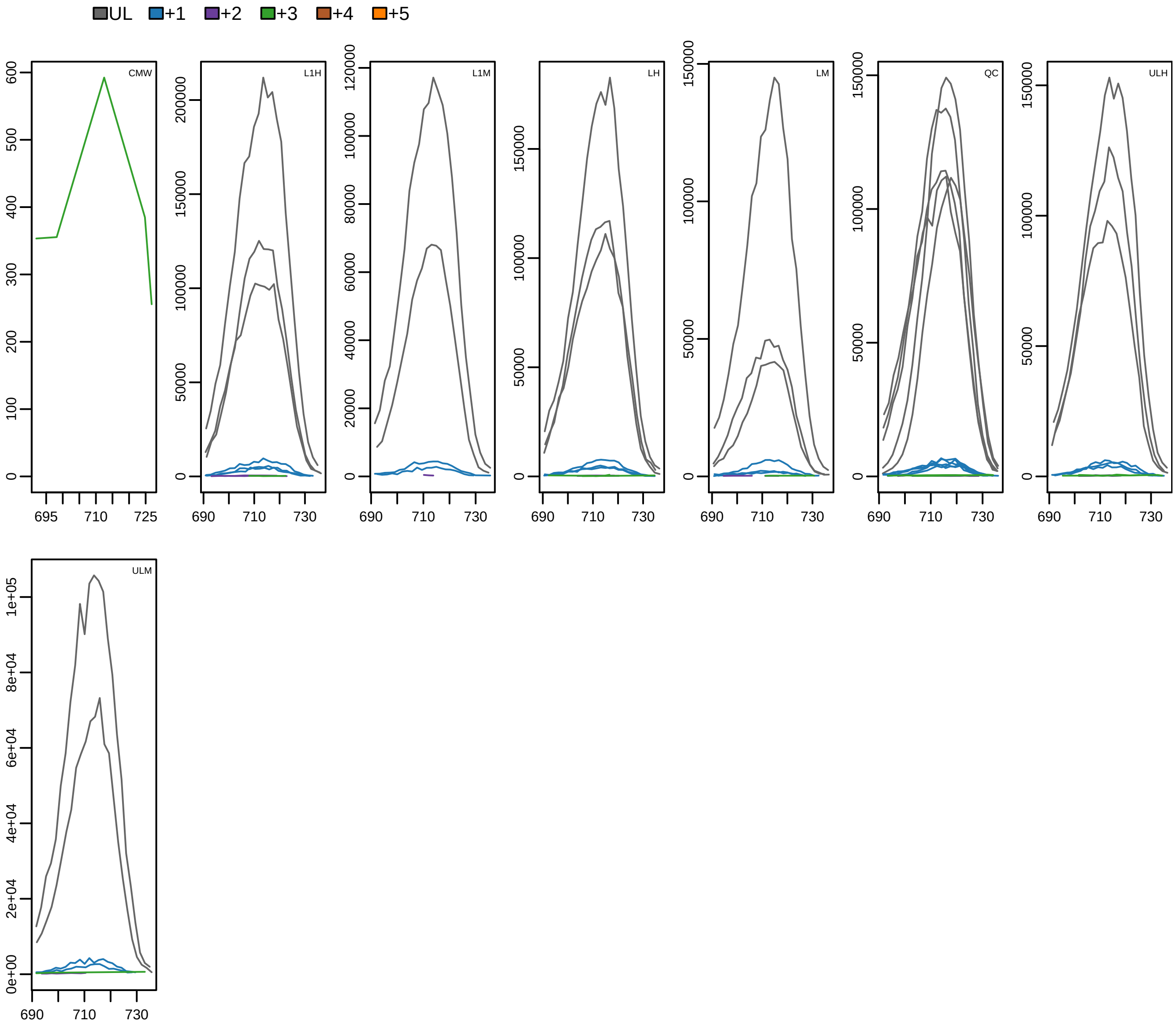

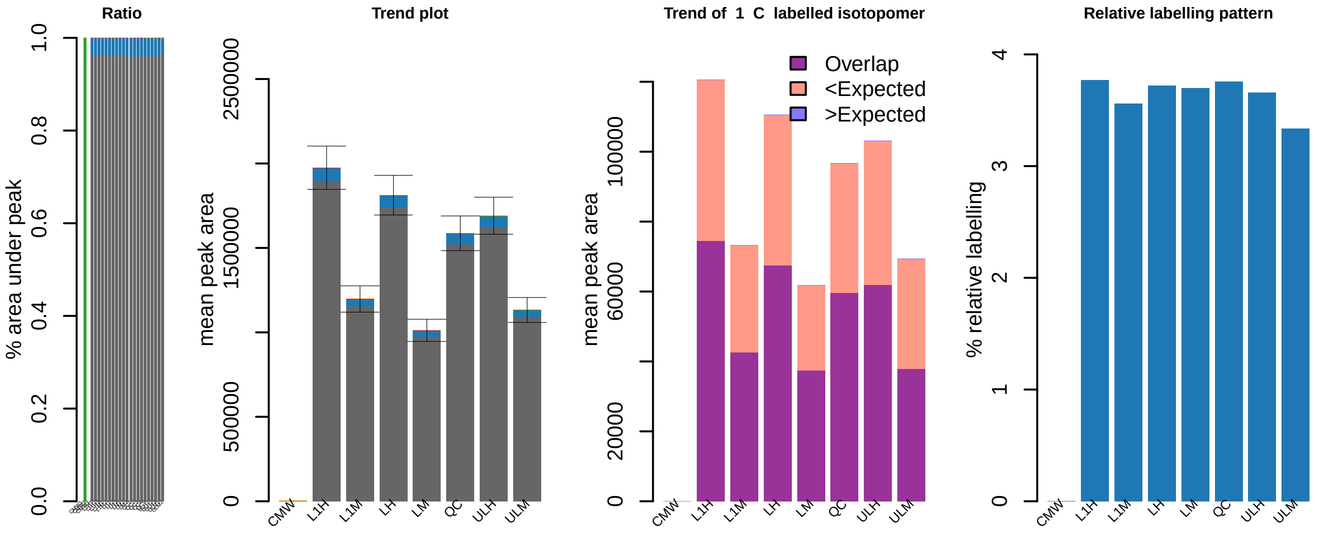

Glyoxylate

Formula: $C2H2O3$ Mass: 74 Std.RT: 1016.6023332 Ion: NEG

G1

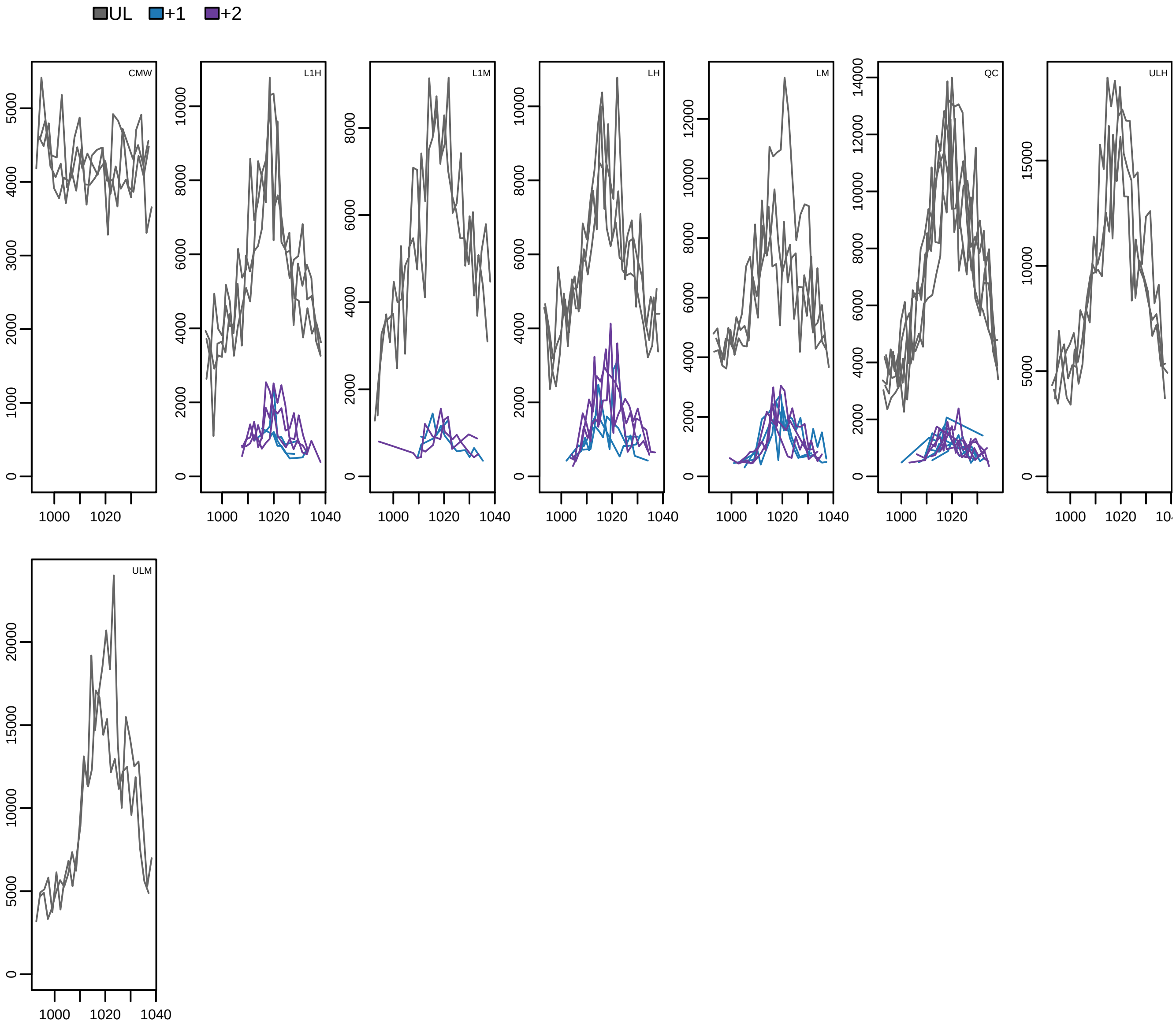

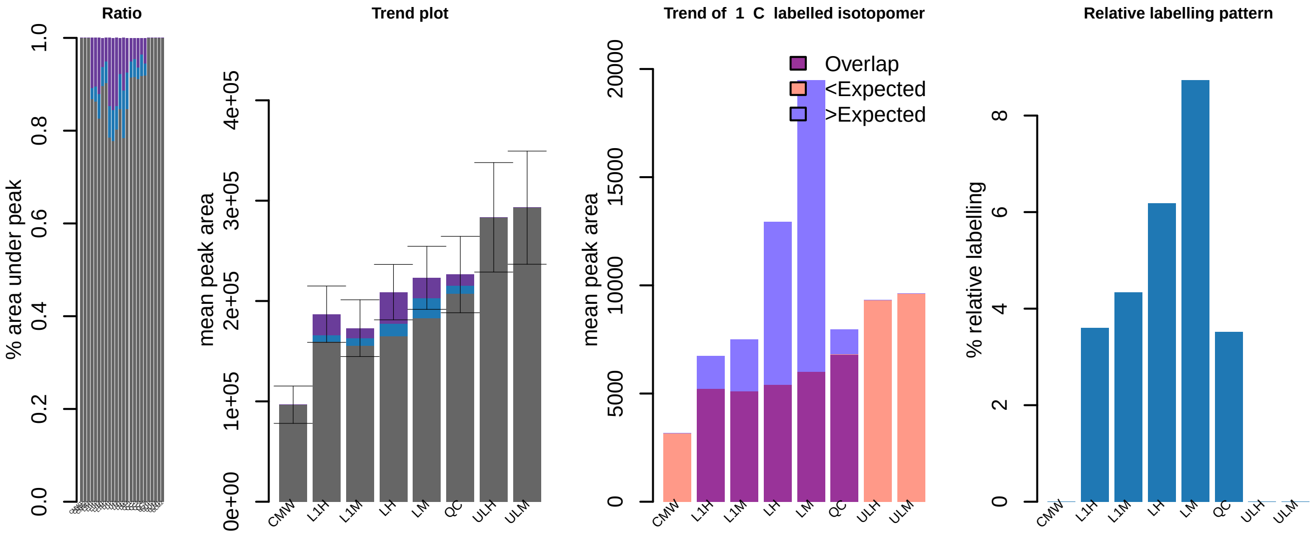

GMP

G1

Formula: C10H14N5O8P Mass: 363.058 Std.RT: 1078.4147538 Ion:

UL +1 +2 +3 +4 +5 +6 +7 +8 +9 +10

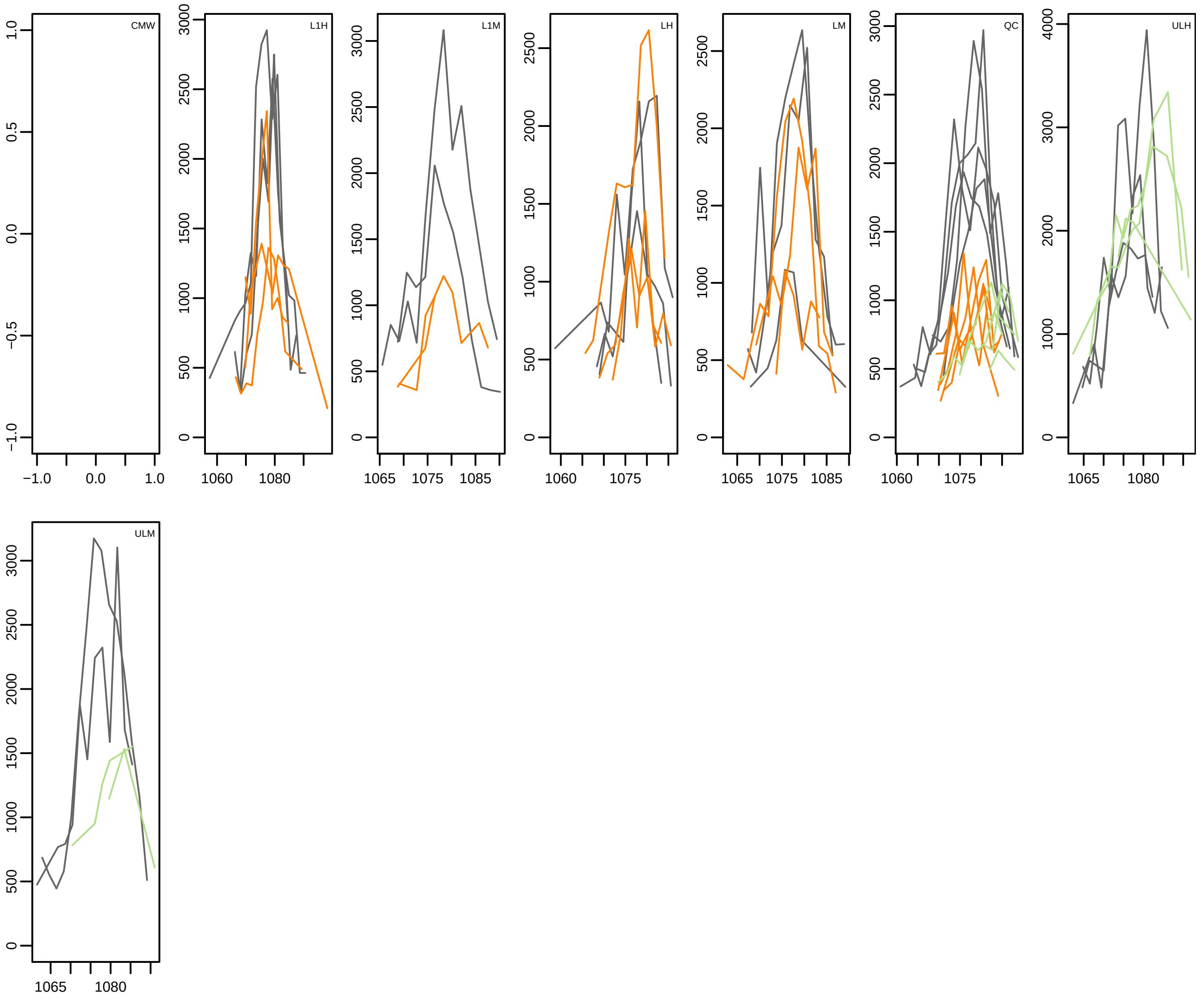

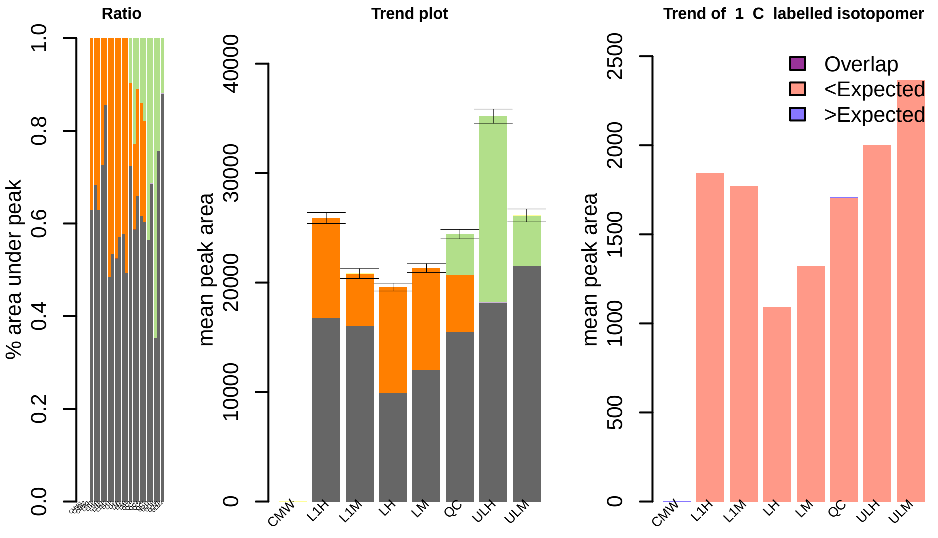

GTP

Formula: C10H16N5O14P3 Mass: 522.991 Std.RT: 1226.054757 Ion:

G1

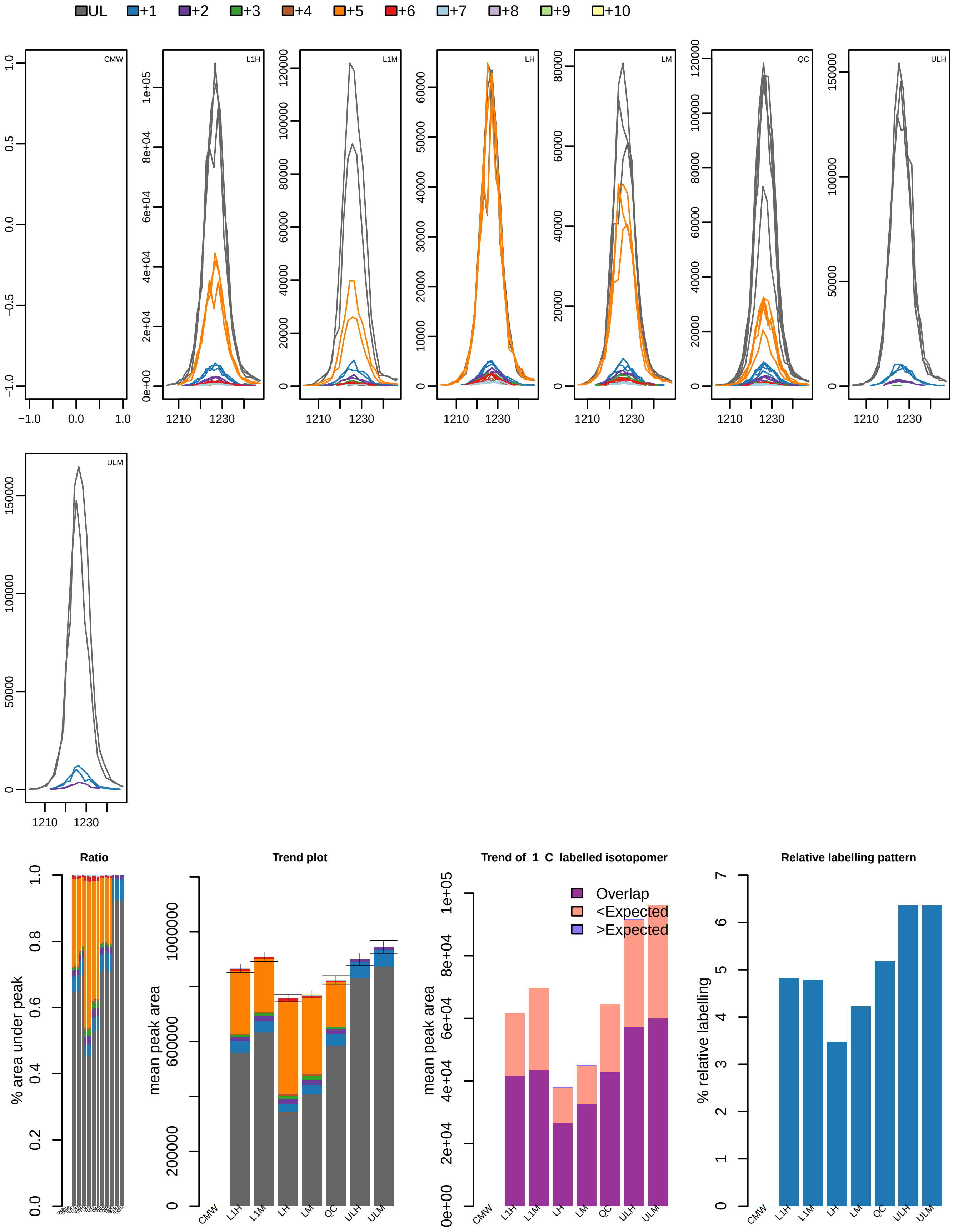

dCTP

G1

Formula: C9H16N3O13P3 Mass: 466.99 Std.RT: 1120.344279 Ion: N

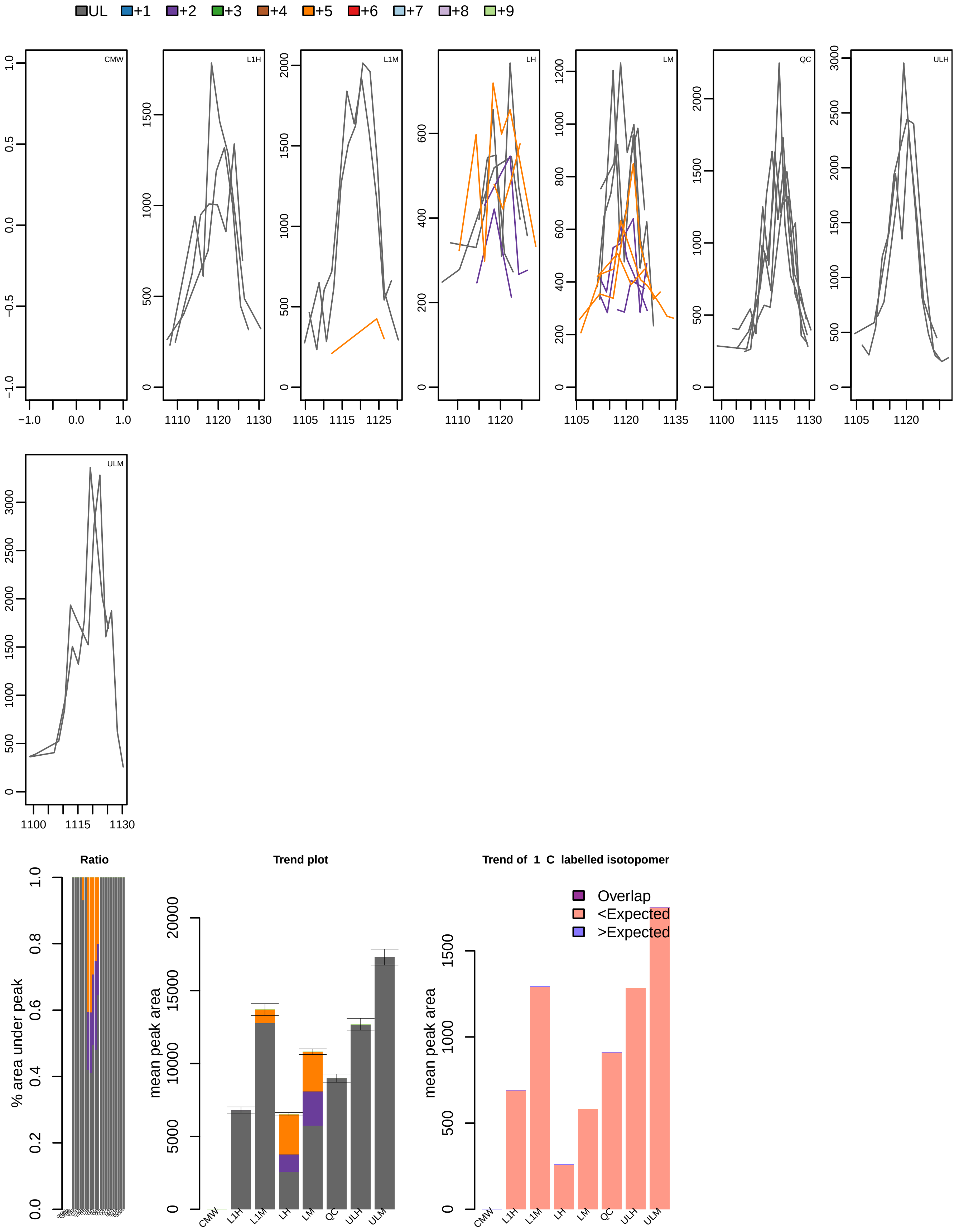

UTP

Formula: C9H15N2O15P3 Mass: 483.969 Std.RT: 1142.1523902 Ion:

G1

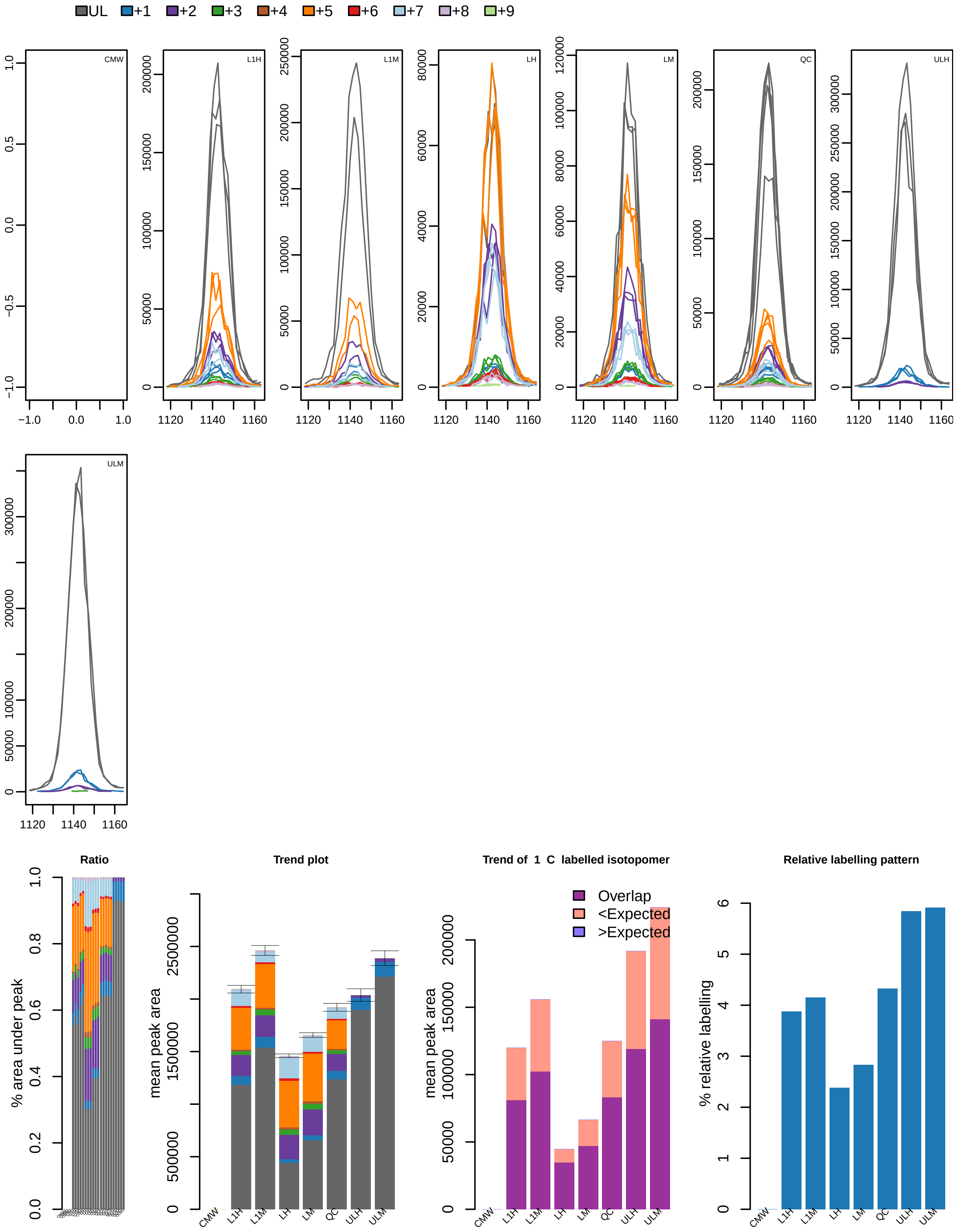

CTP

Formula: C9H16N3O14P3 Mass: 482.985 Std.RT: 1173.117153 Ion:

G1

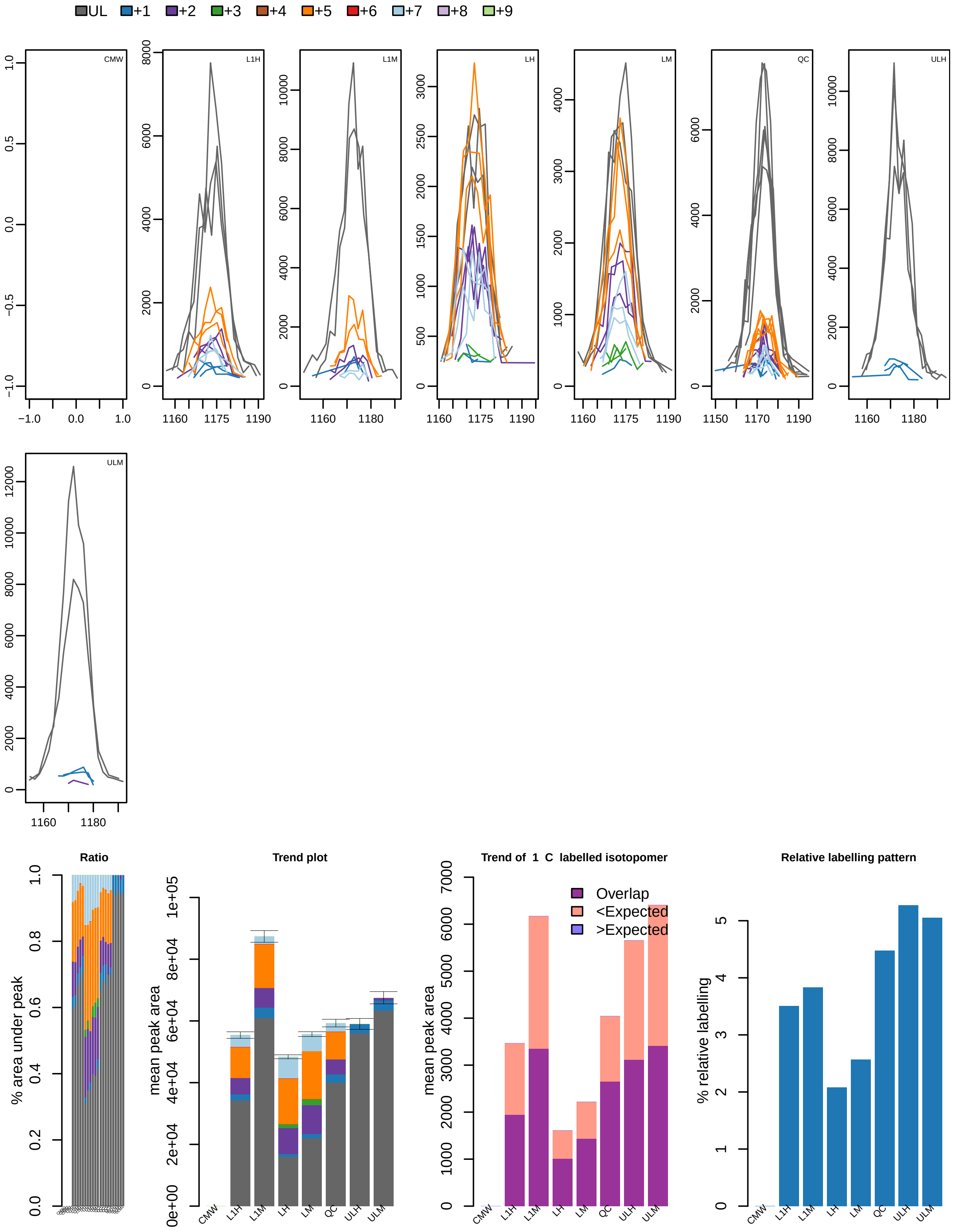

Deoxyuridine

Formula: C9H12N2O5 Mass: 228.075 Std.RT: 480.0595224 Ion: NEC

G1

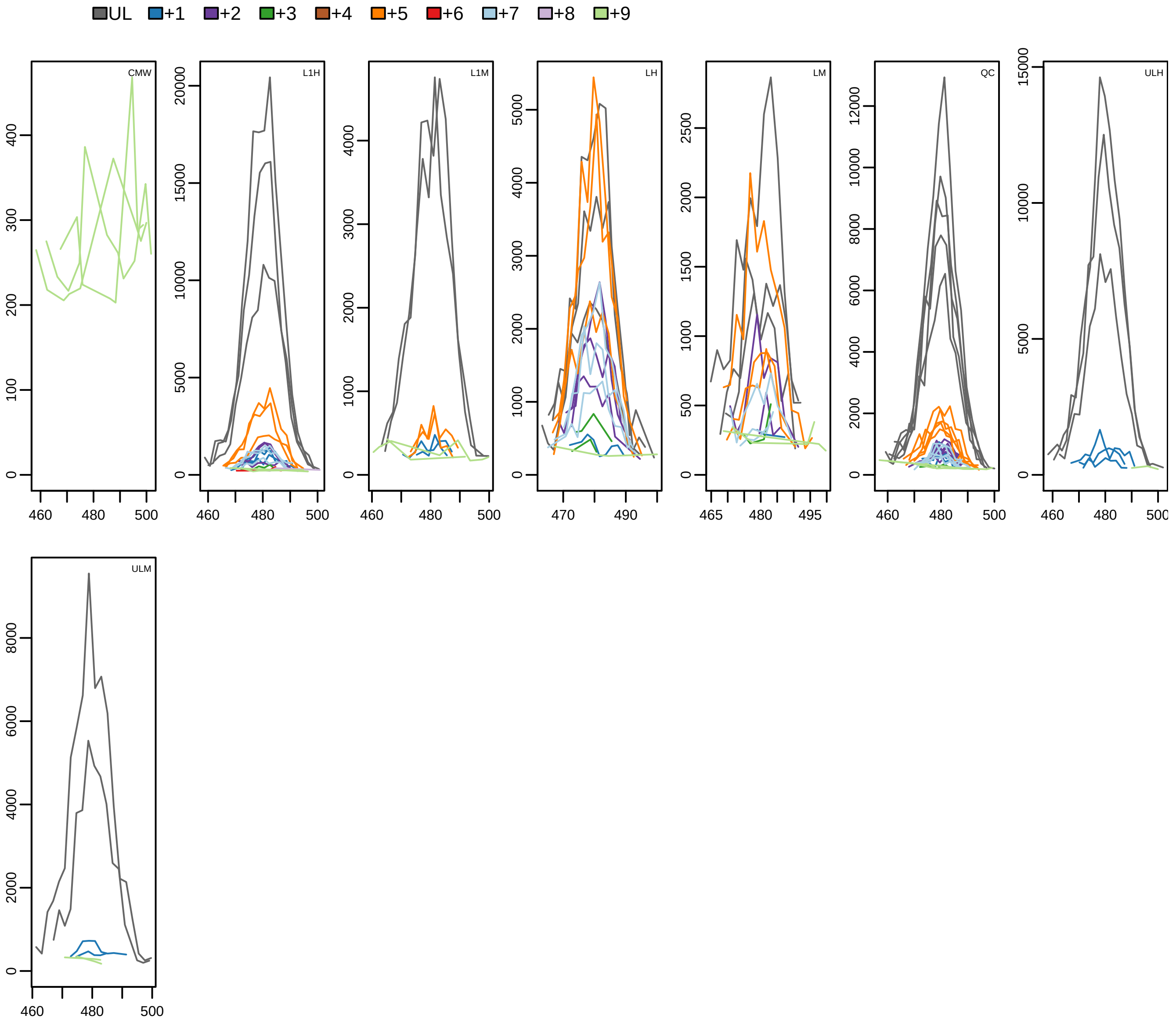

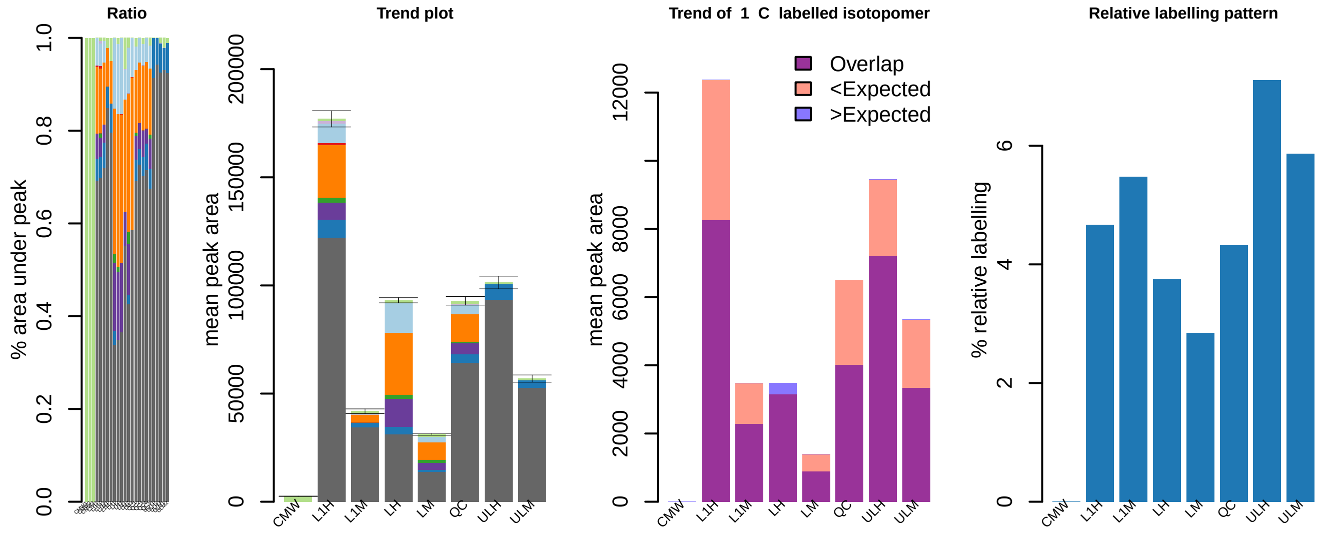

Uridine

Formula: C9H12N2O6 Mass: 244.07 Std.RT: 606.5479998 Ion: NEG

G1

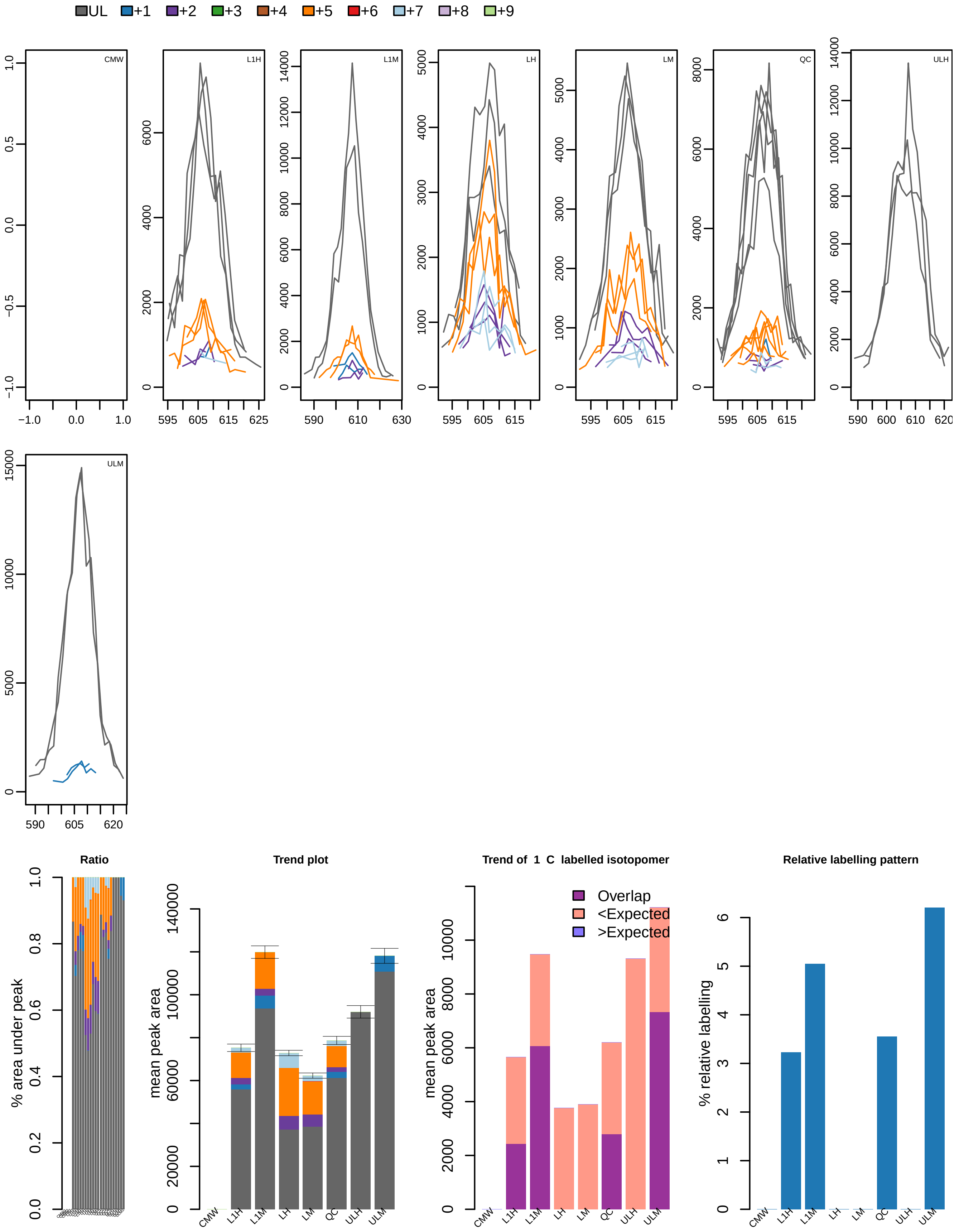

(S)−Dihydroorotate

Formula: C5H6N2O4 Mass: 158.033 Std.RT: 684.5408616 Ion: NEG

G1

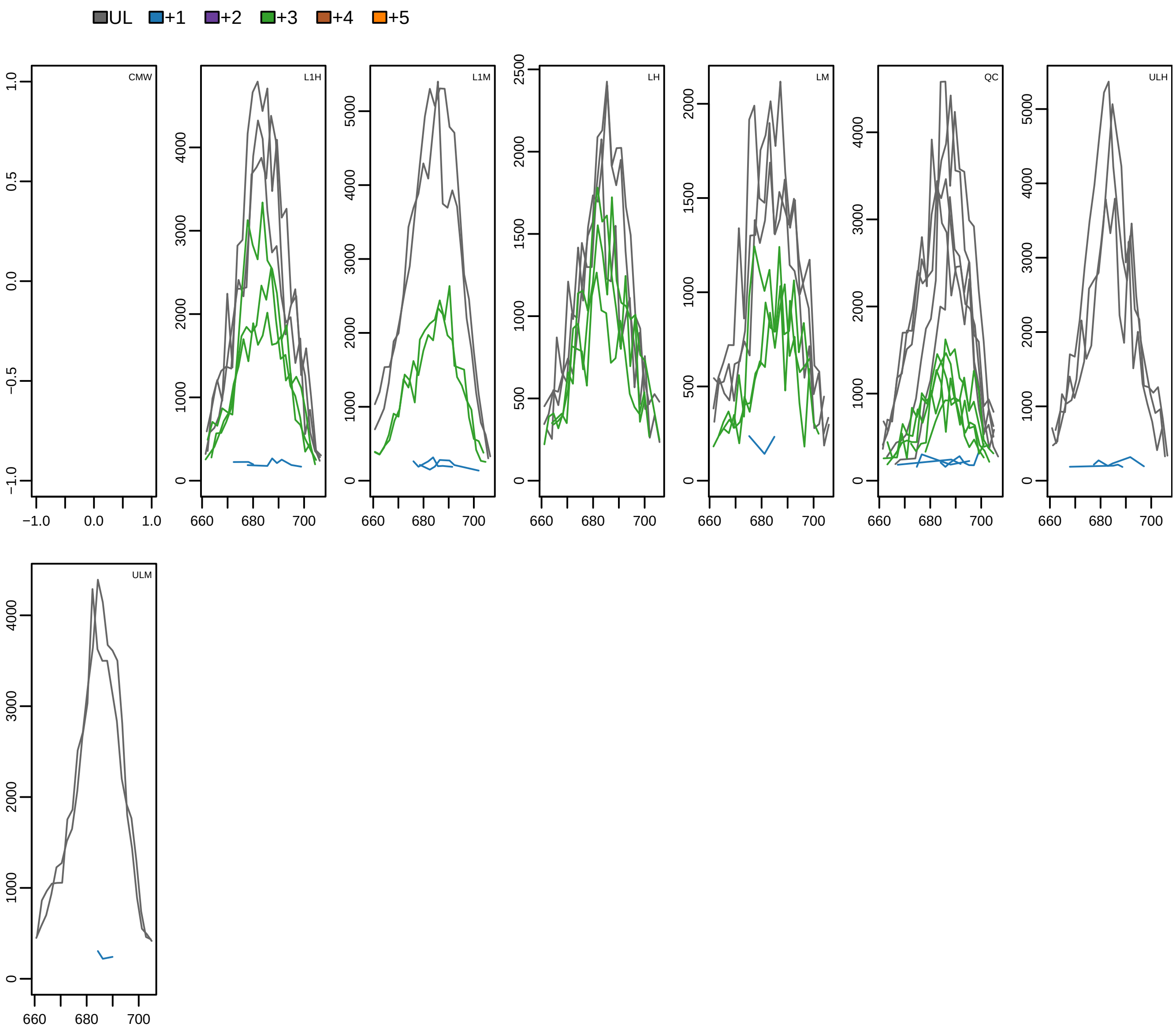

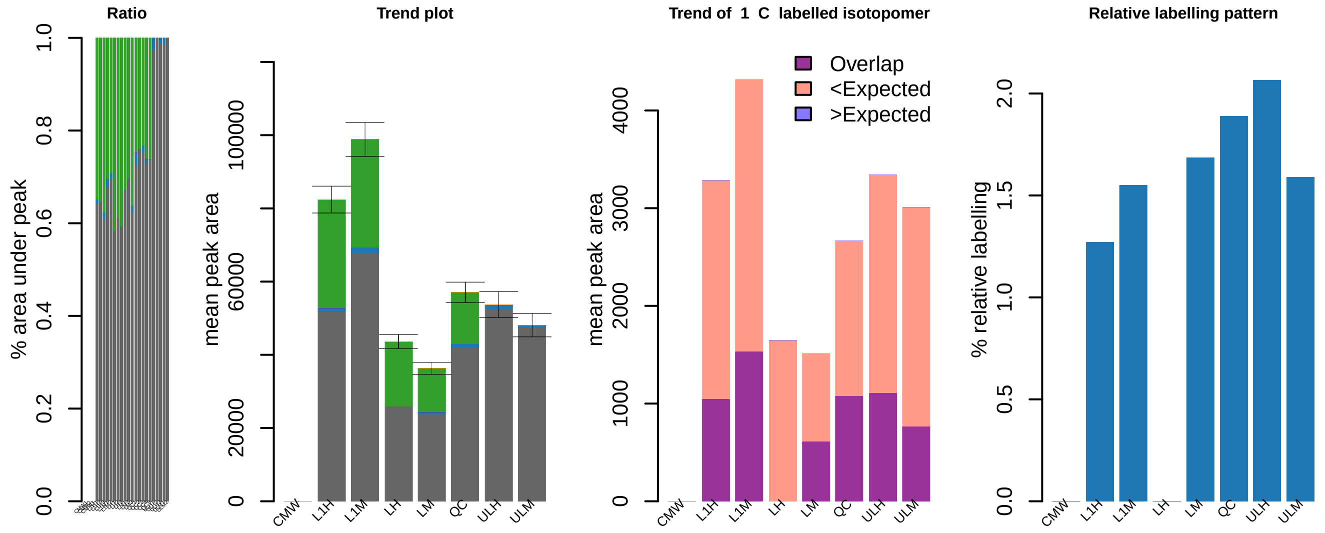

Orotate

Formula: C5H4N2O4 Mass: 156.017 Std.RT: 604.2105246 Ion: NEG

G1

UL +1 +2 +3 +4 +5

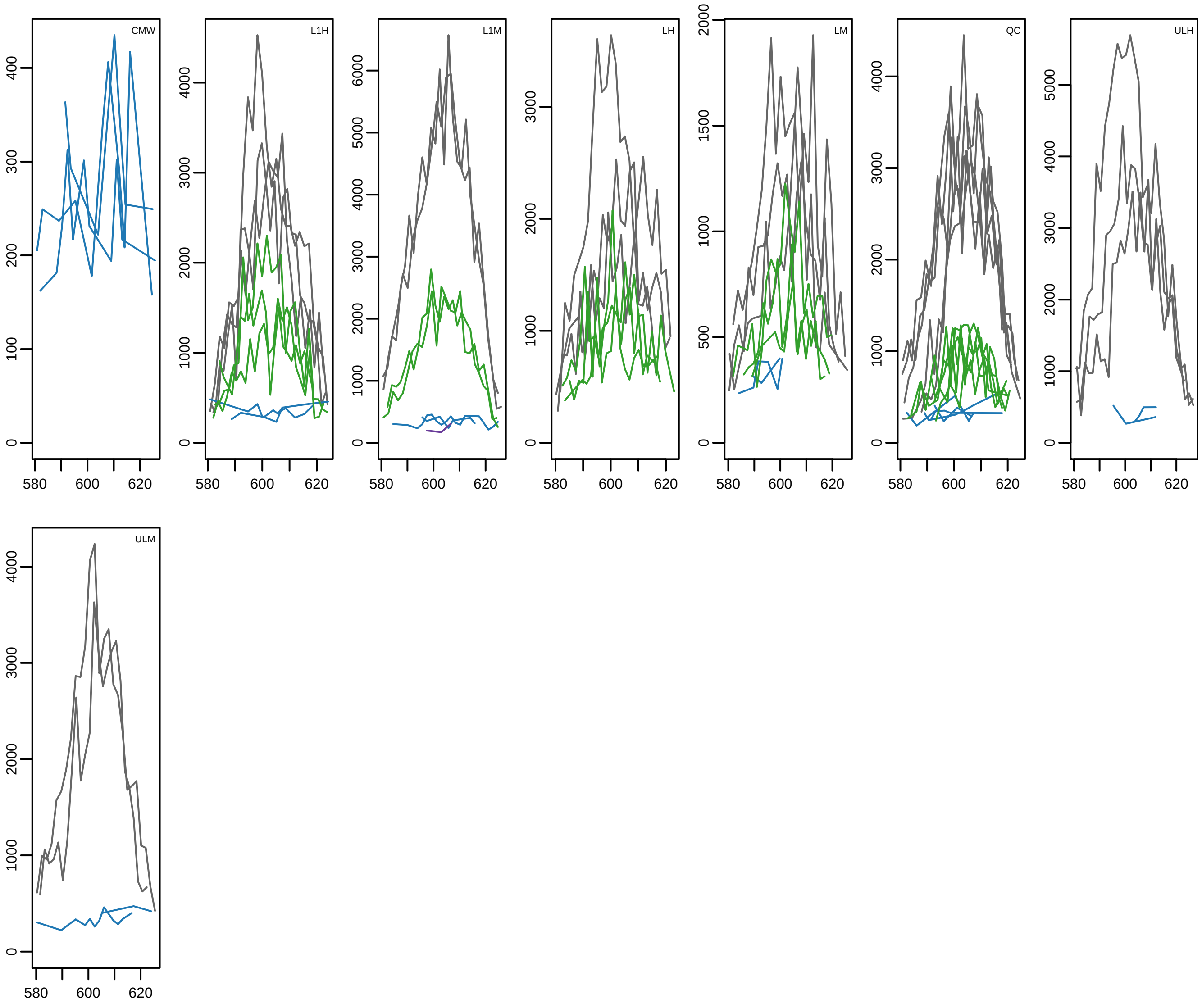

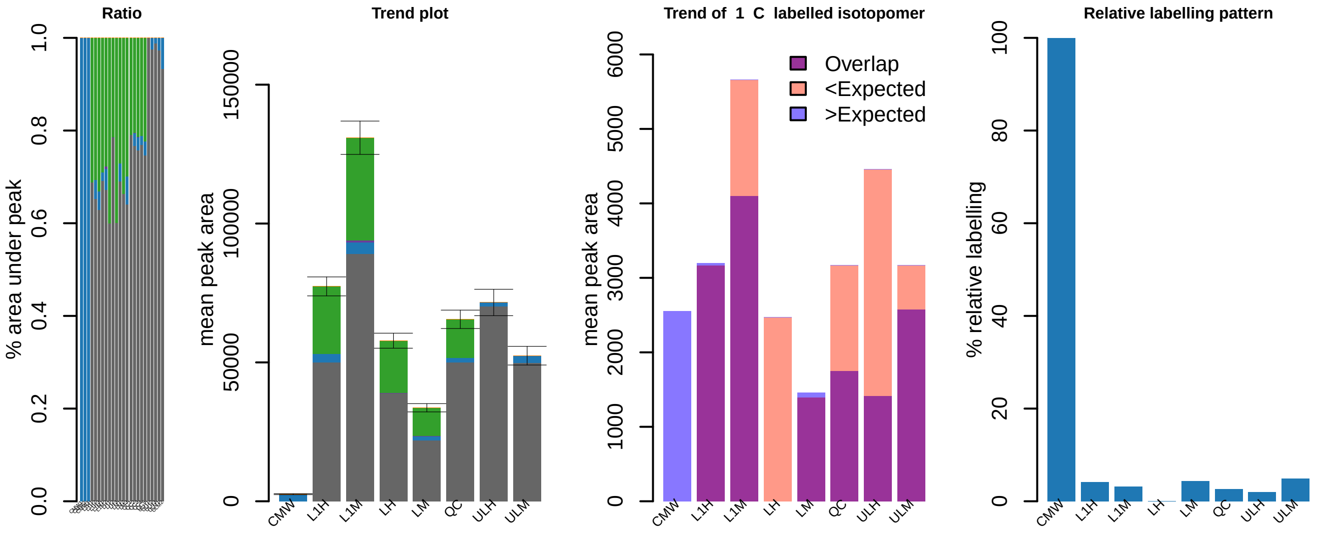

dTMP

Formula: C10H15N2O8P Mass: 322.057 Std.RT: 802.2165702 Ion: N

G1

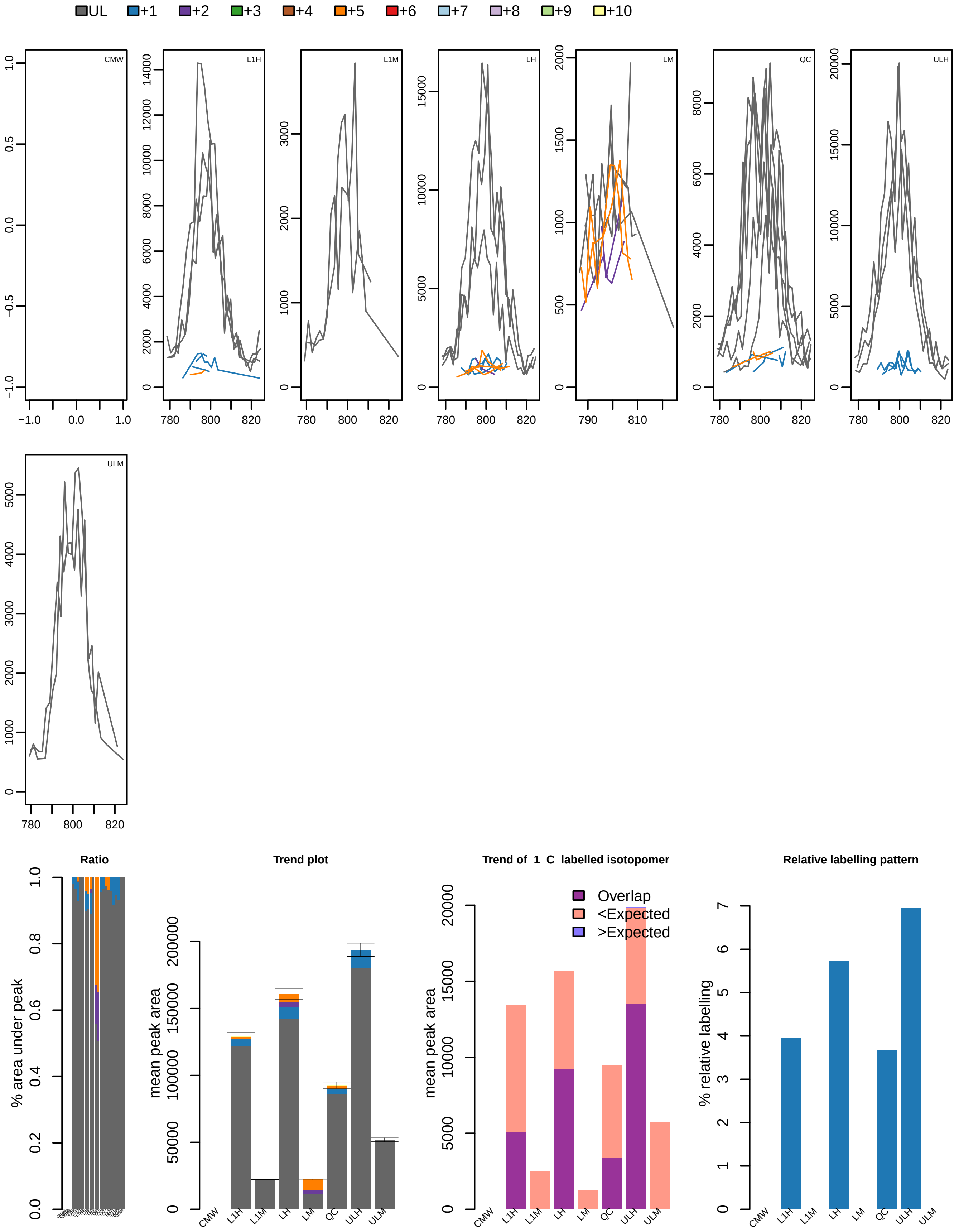

Thymidine

Formula: $C10H14N2O5$ Mass: 242.09 Std.RT: 433.26193242 Ion: NE

G1

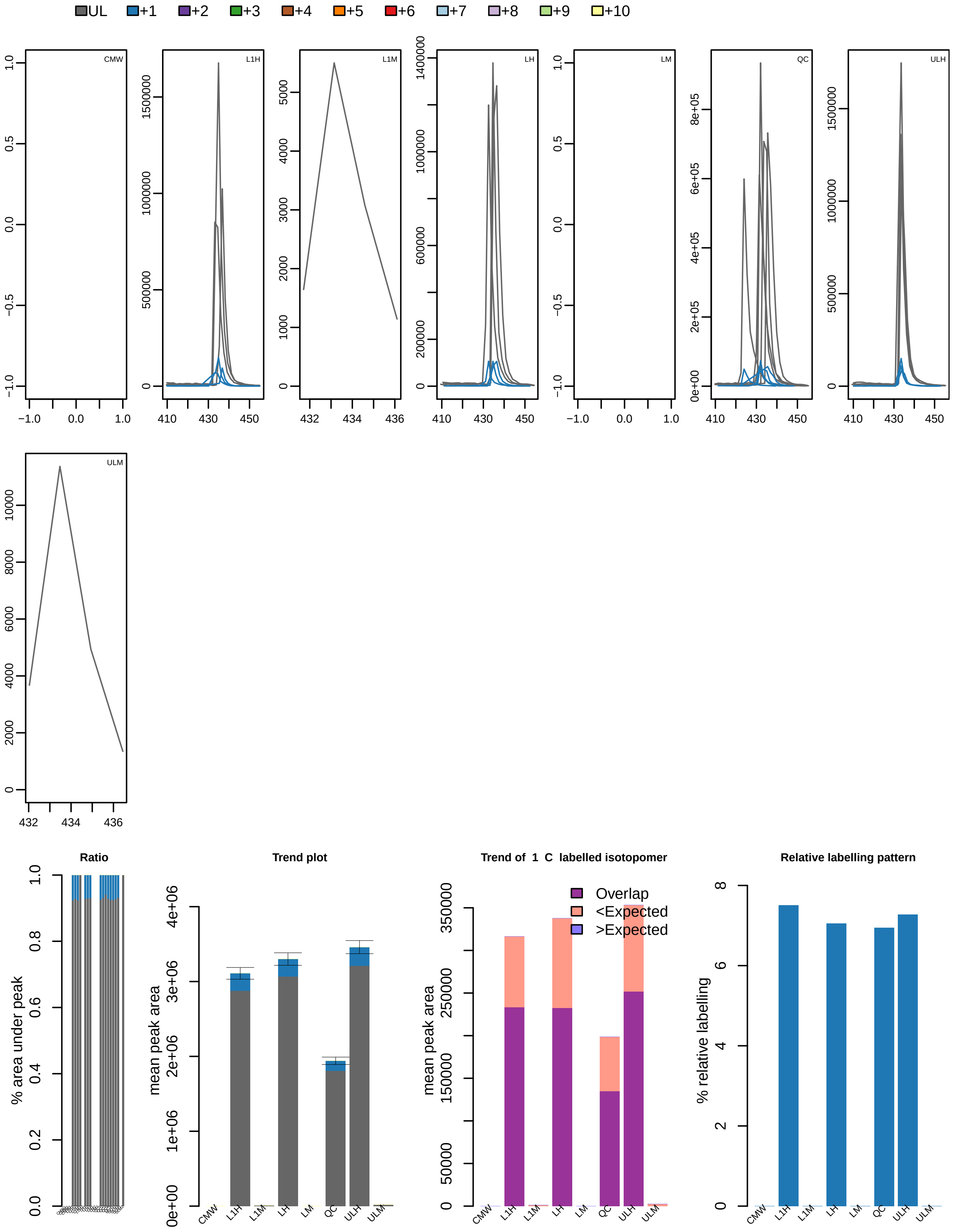

Thymidine

Formula: $C10H14N2O5$ Mass: 242.09 Std.RT: 433.26193242 Ion: NE

G2

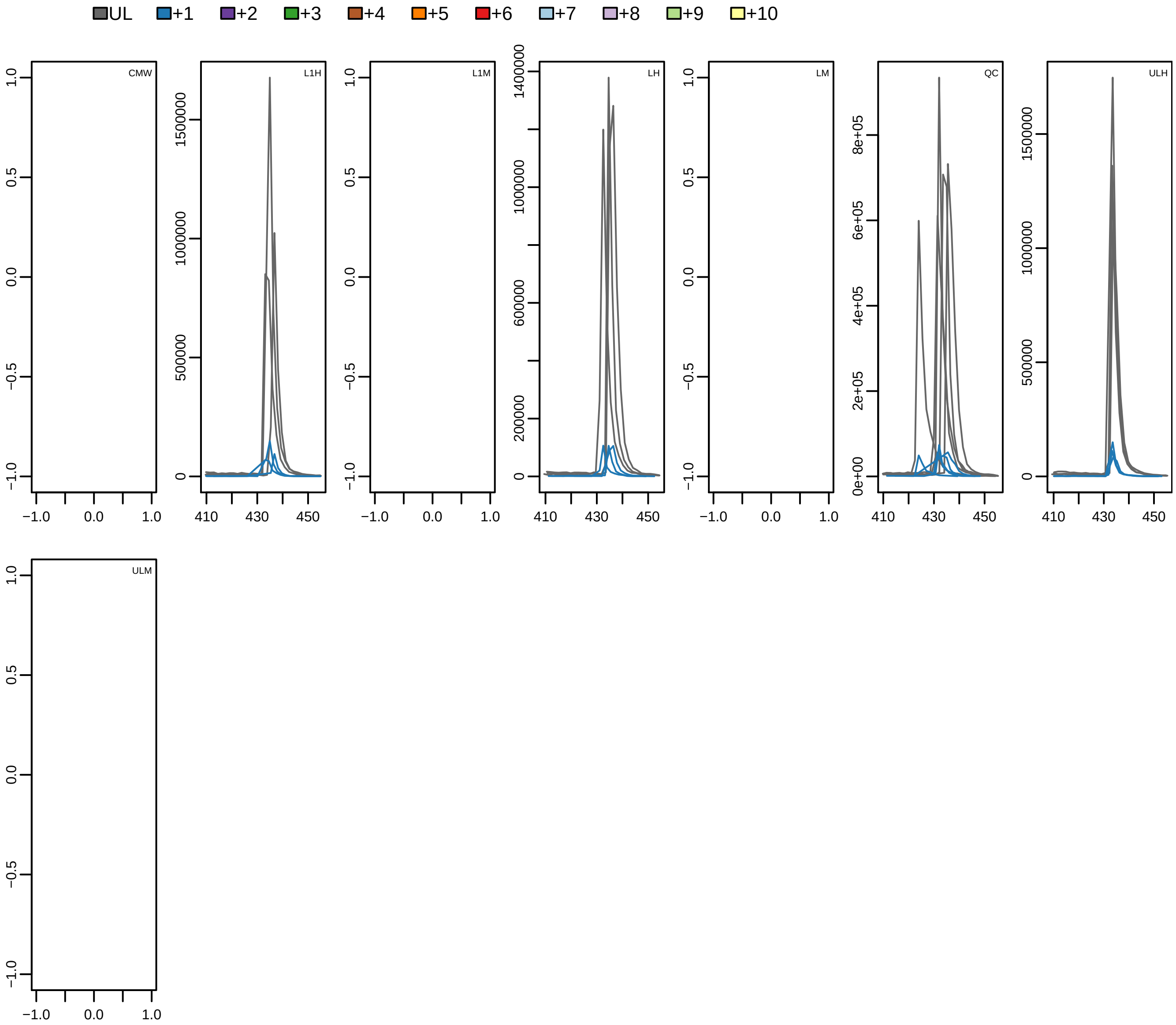

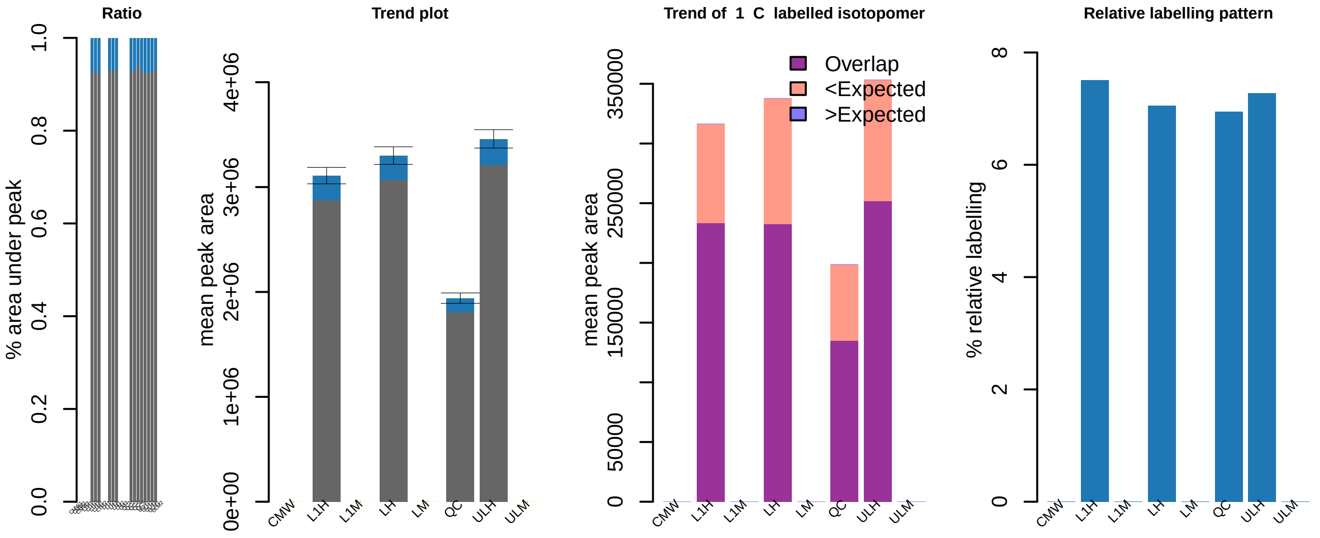

dTTP

Formula: C10H17N2O14P3 Mass: 481.989 Std.RT: 1033.464297 Ion:

G1

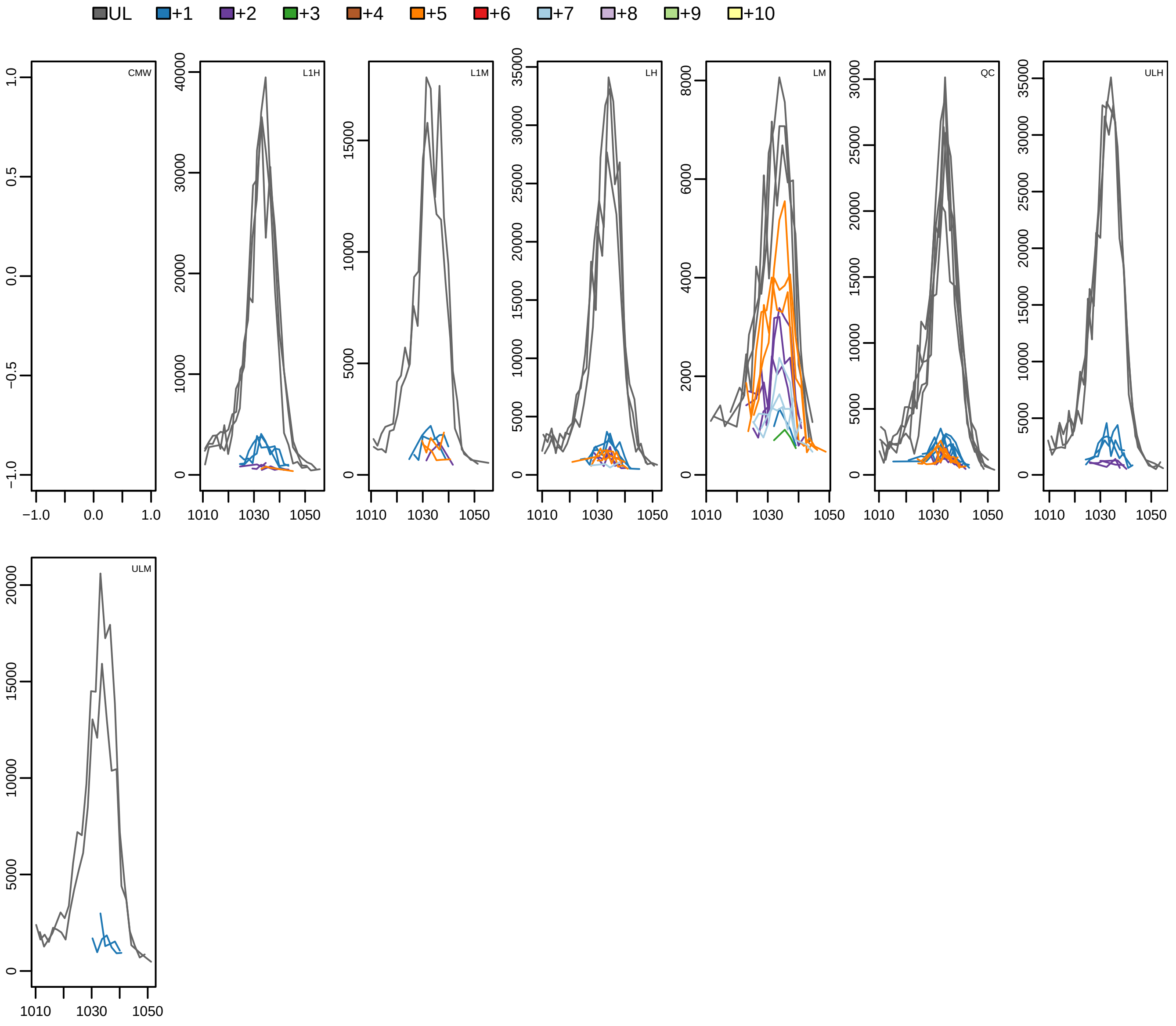

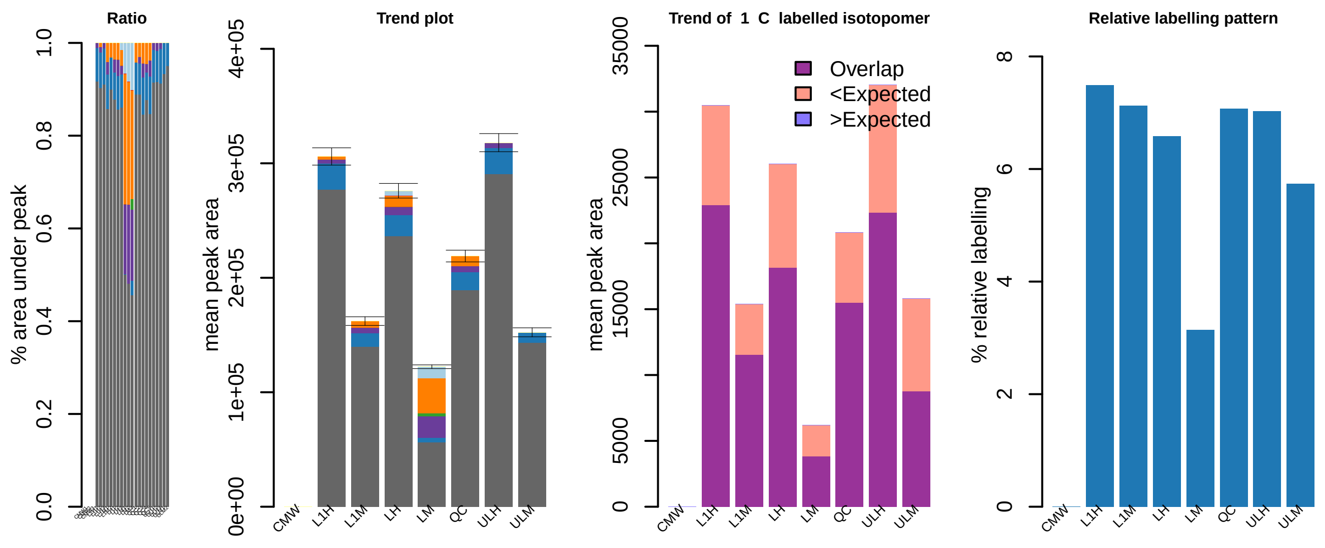

5,6−Dihydrouracil

Formula: C4H6N2O2 Mass: 114.043 Std.RT: 458.37375258 Ion: NE(

G1

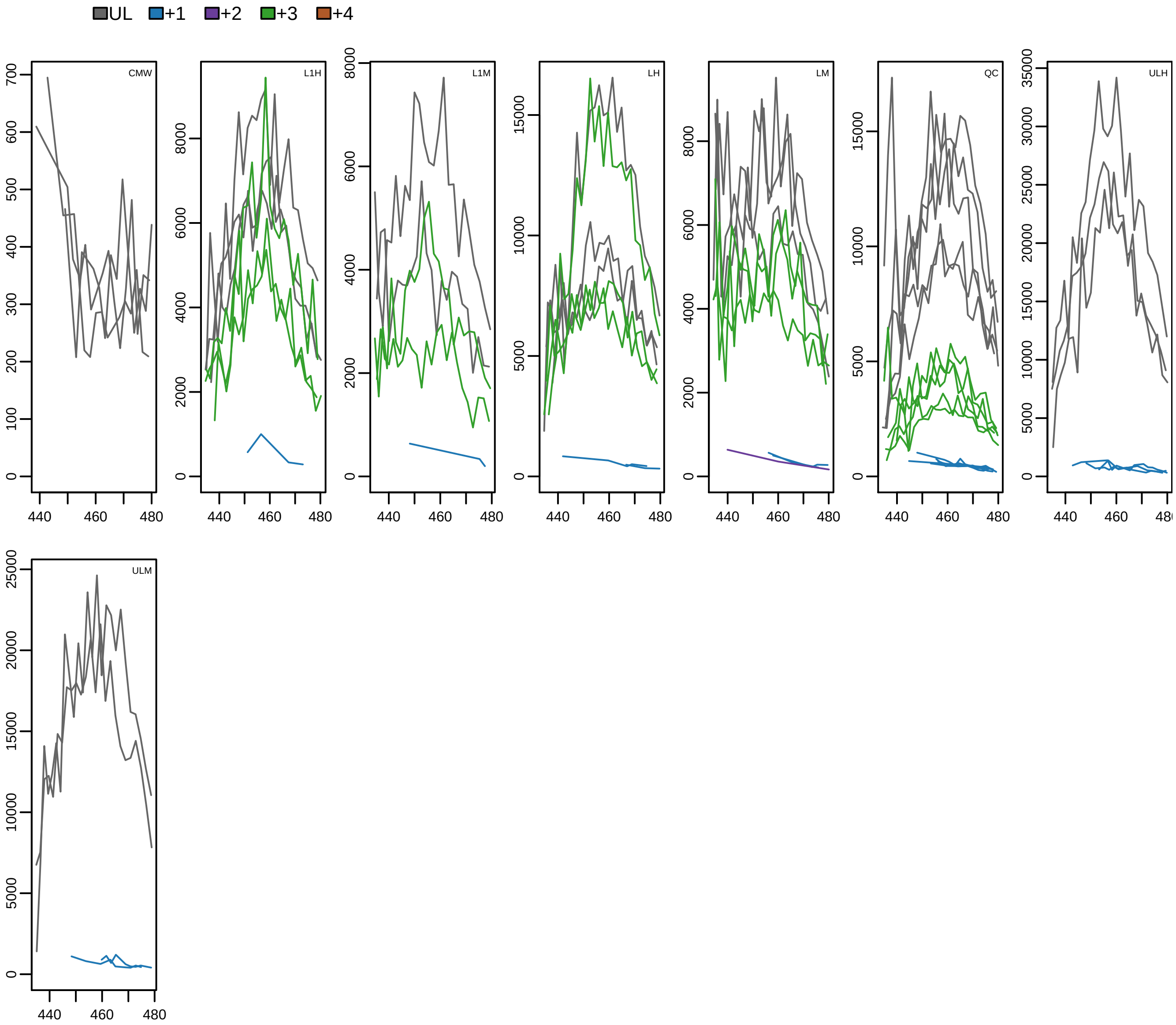

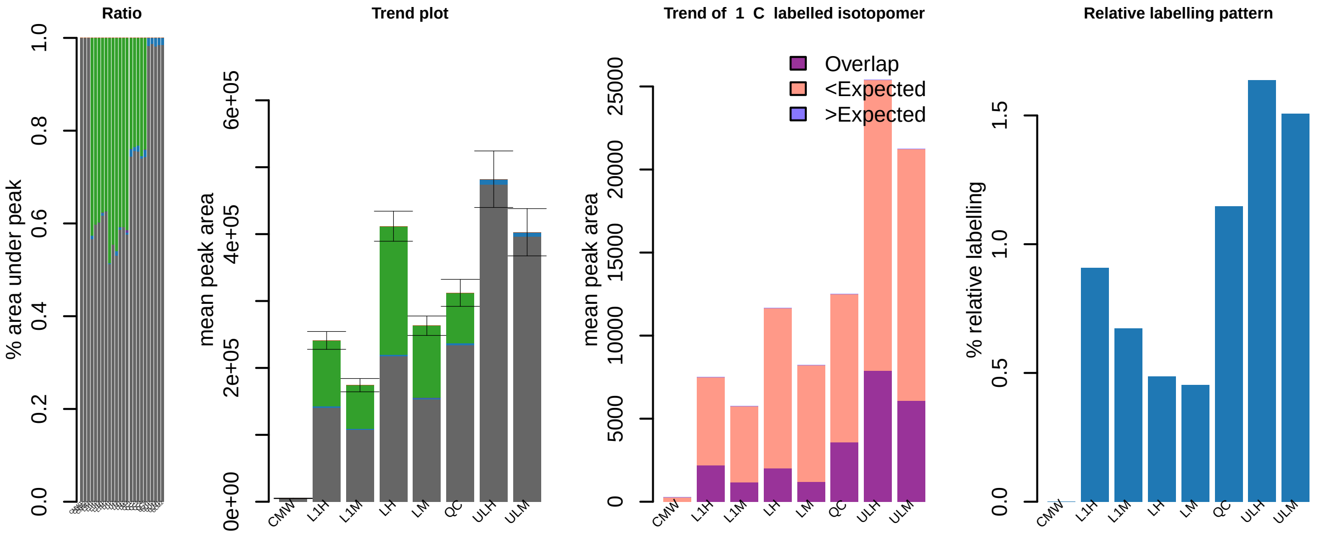

Uracil

Formula: C4H4N2O2 Mass: 112.027 Std.RT: 499.1936607 Ion: NEG

G1

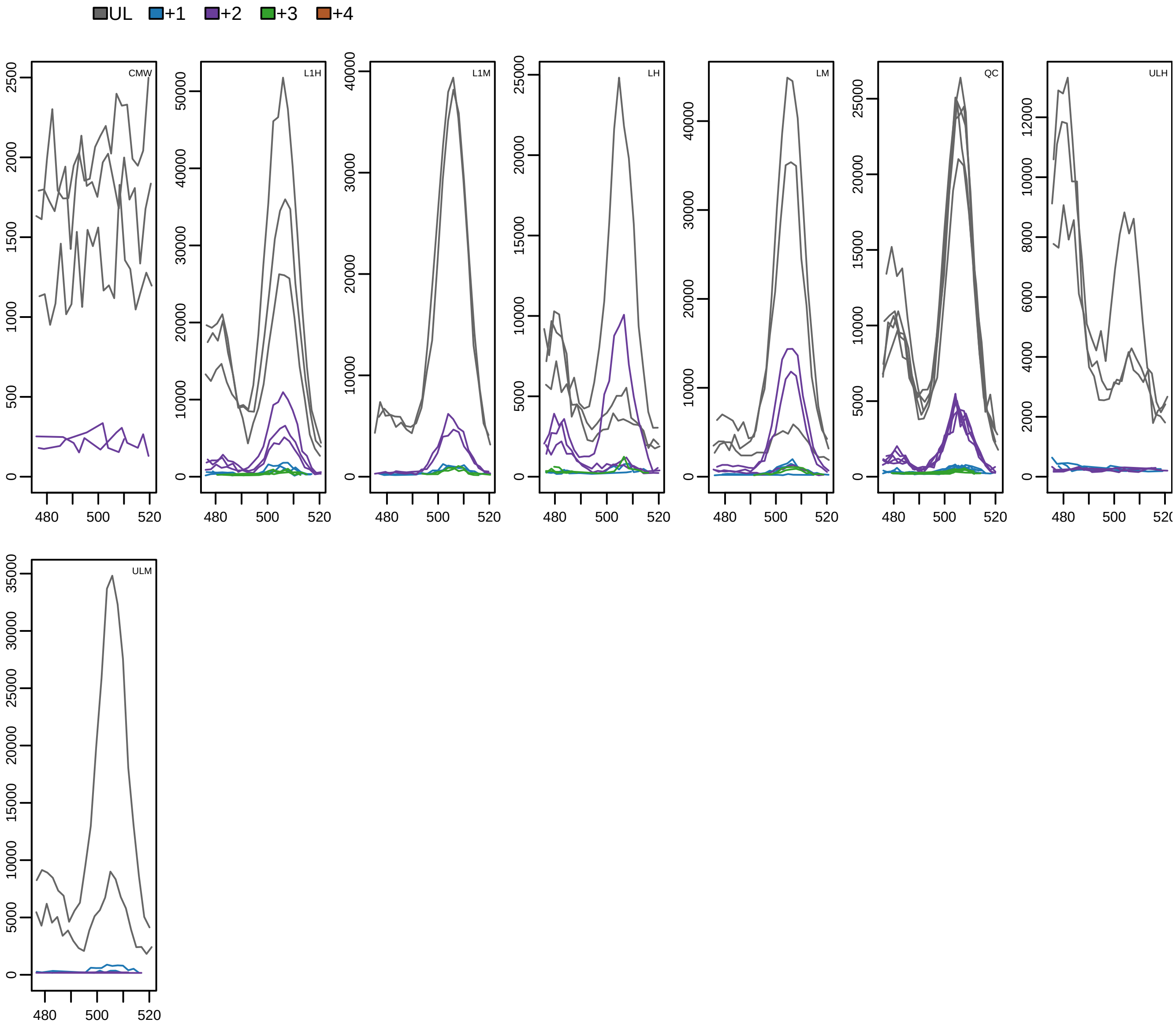

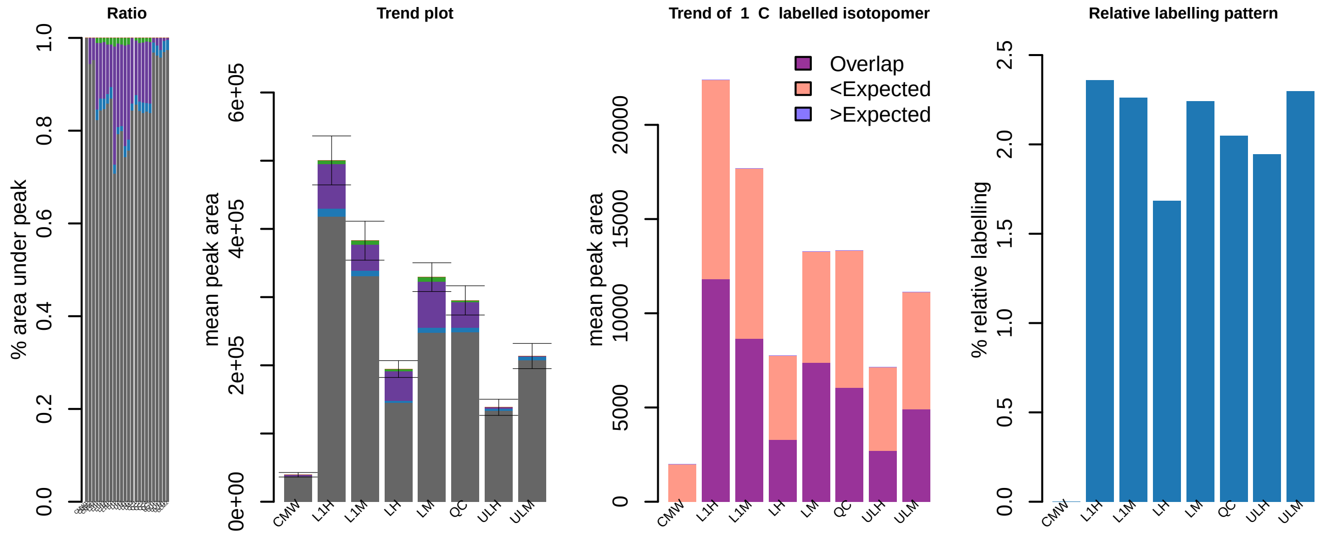

Uracil

Formula: C4H4N2O2 Mass: 112.027 Std.RT: 499.1936607 Ion: NEG

G2

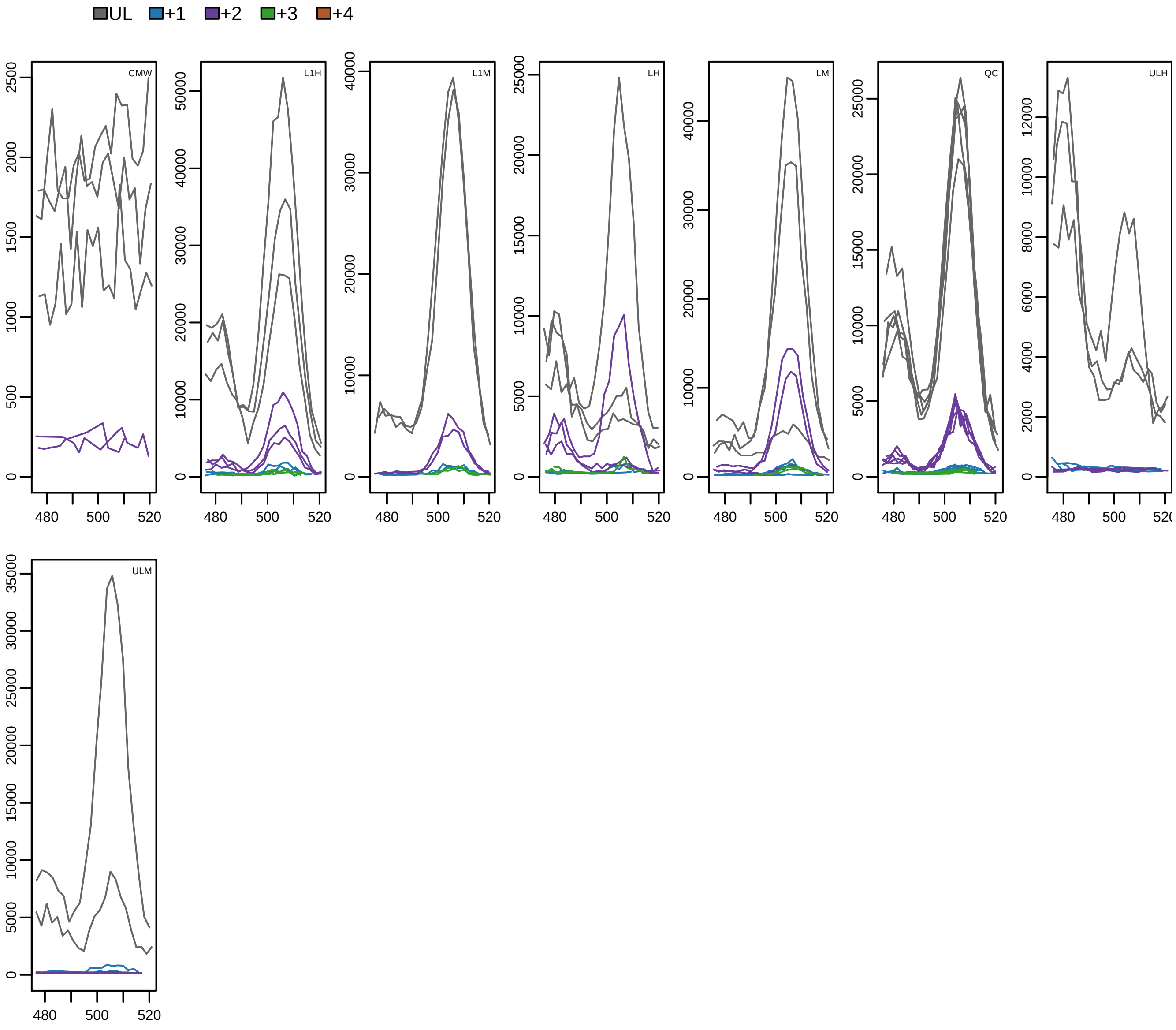

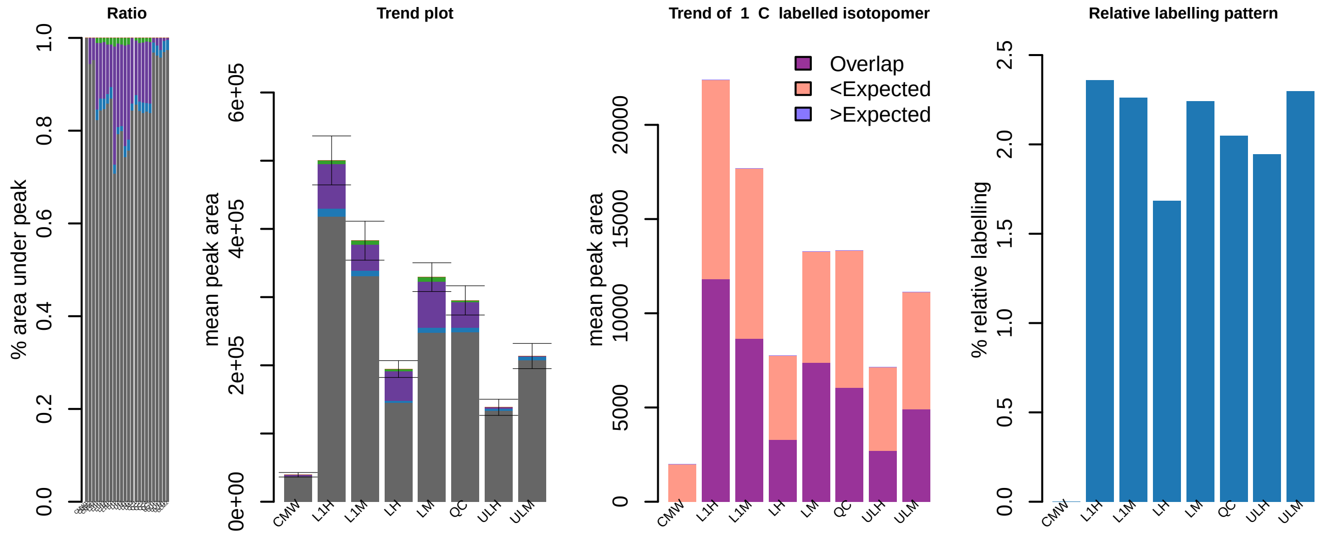

UMP

Formula: C9H13N2O9P Mass: 324.036 Std.RT: 981.9428988 Ion: NE

G1

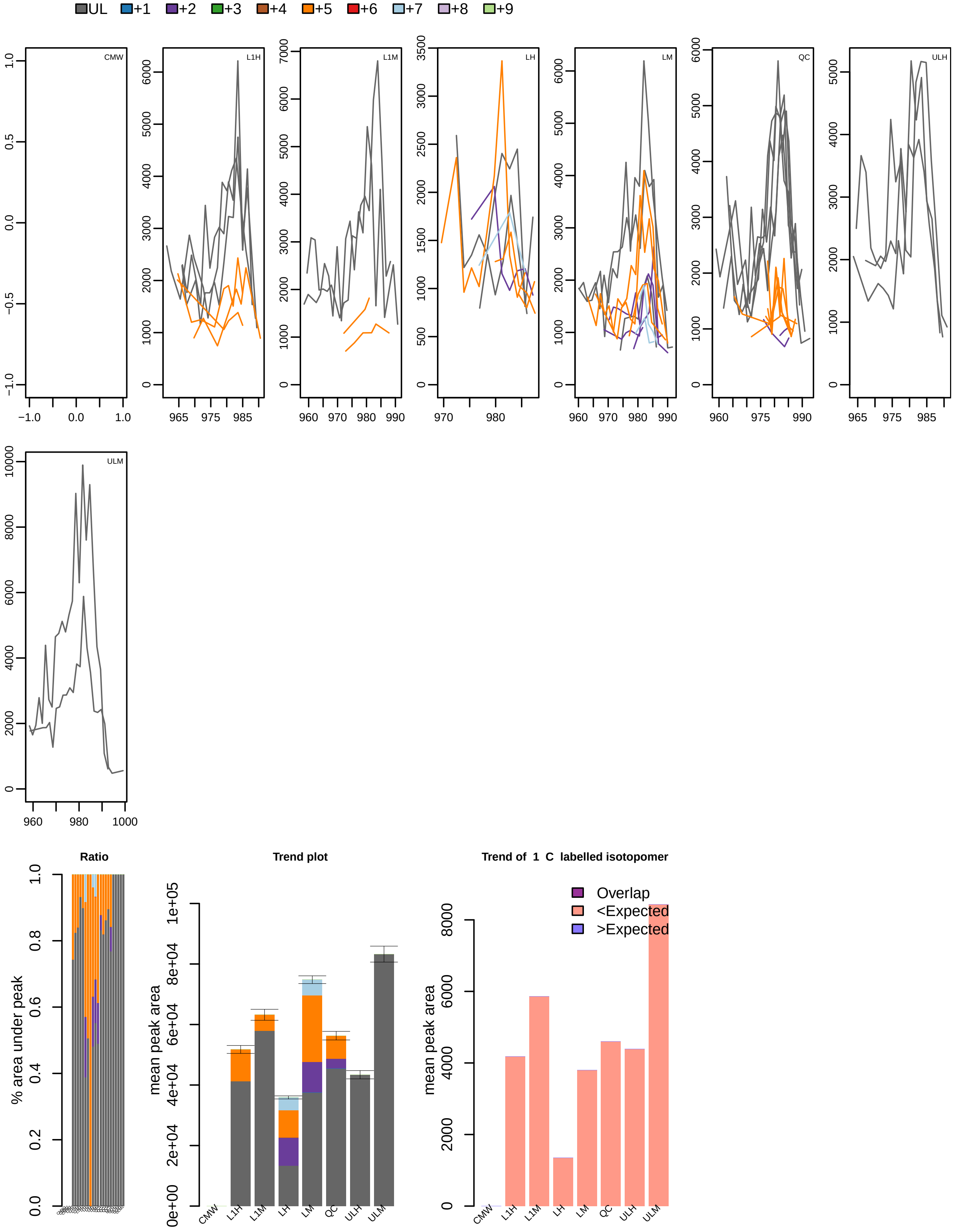

UDP

Formula: C9H14N2O12P2 Mass: 404.002 Std.RT: 1060.66047 Ion: N

G1

UL +1 +2 +3 +4 +5 +6 +7 +8 +9

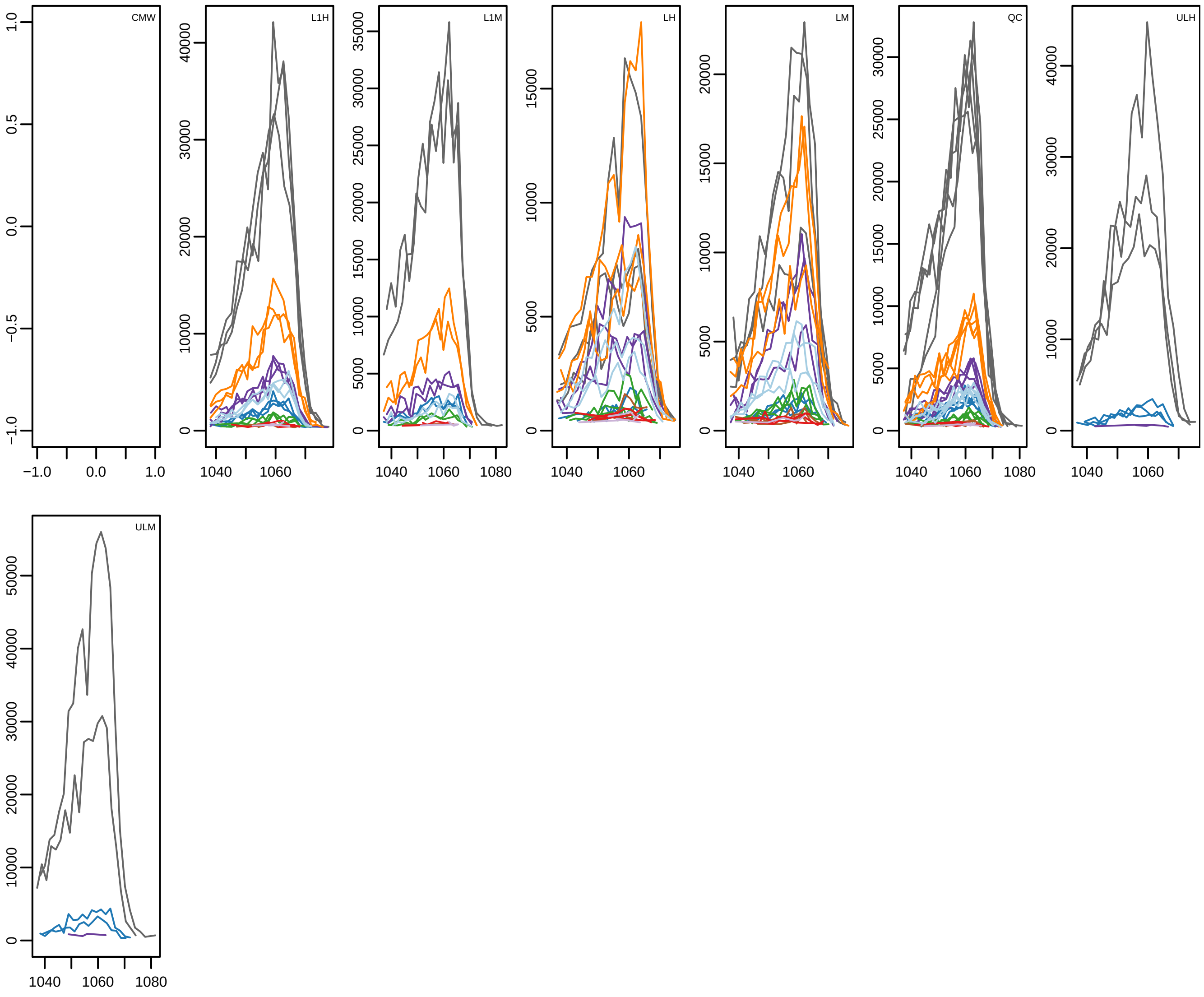

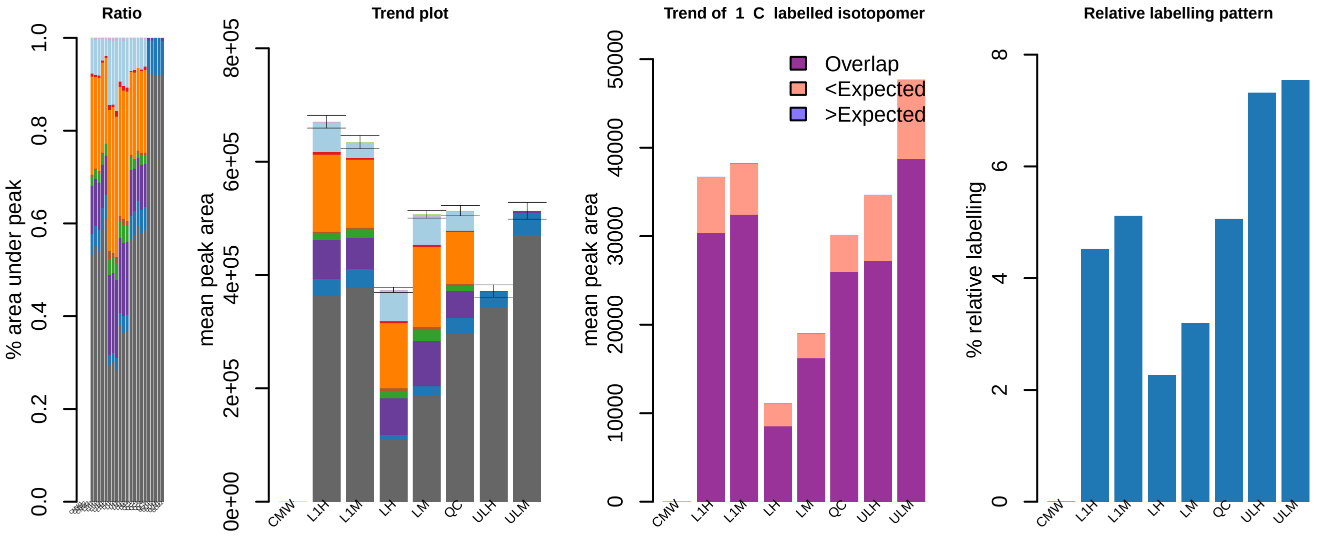

Aspartyl−L−proline

Formula: C9H14N2O5 Mass: 230.09 Std.RT: 804.7165674 Ion: NEG

G1

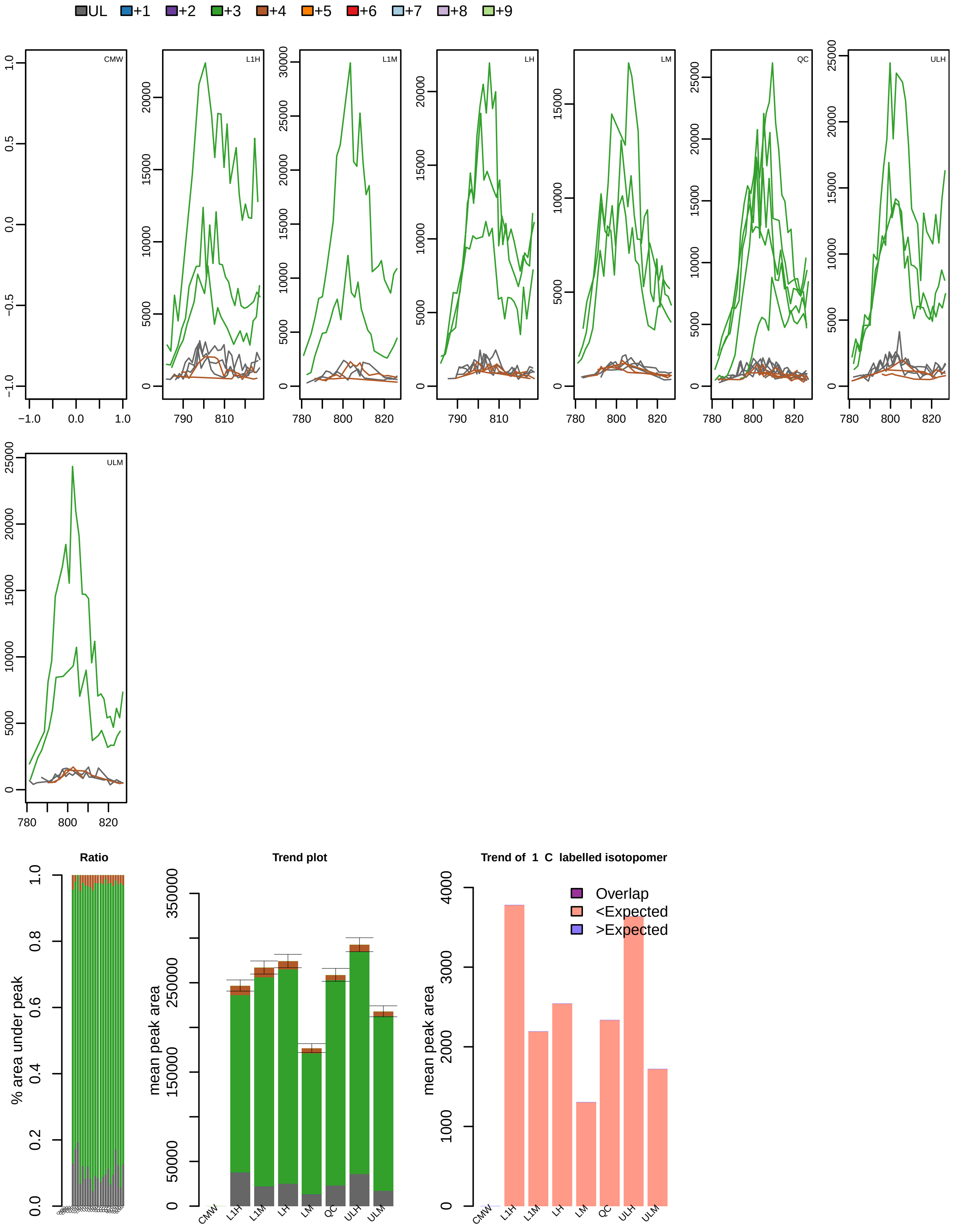

Glu−Gly

Formula: C7H12N2O5 Mass: 204.075 Std.RT: 987.9877146 Ion: NEC

G1

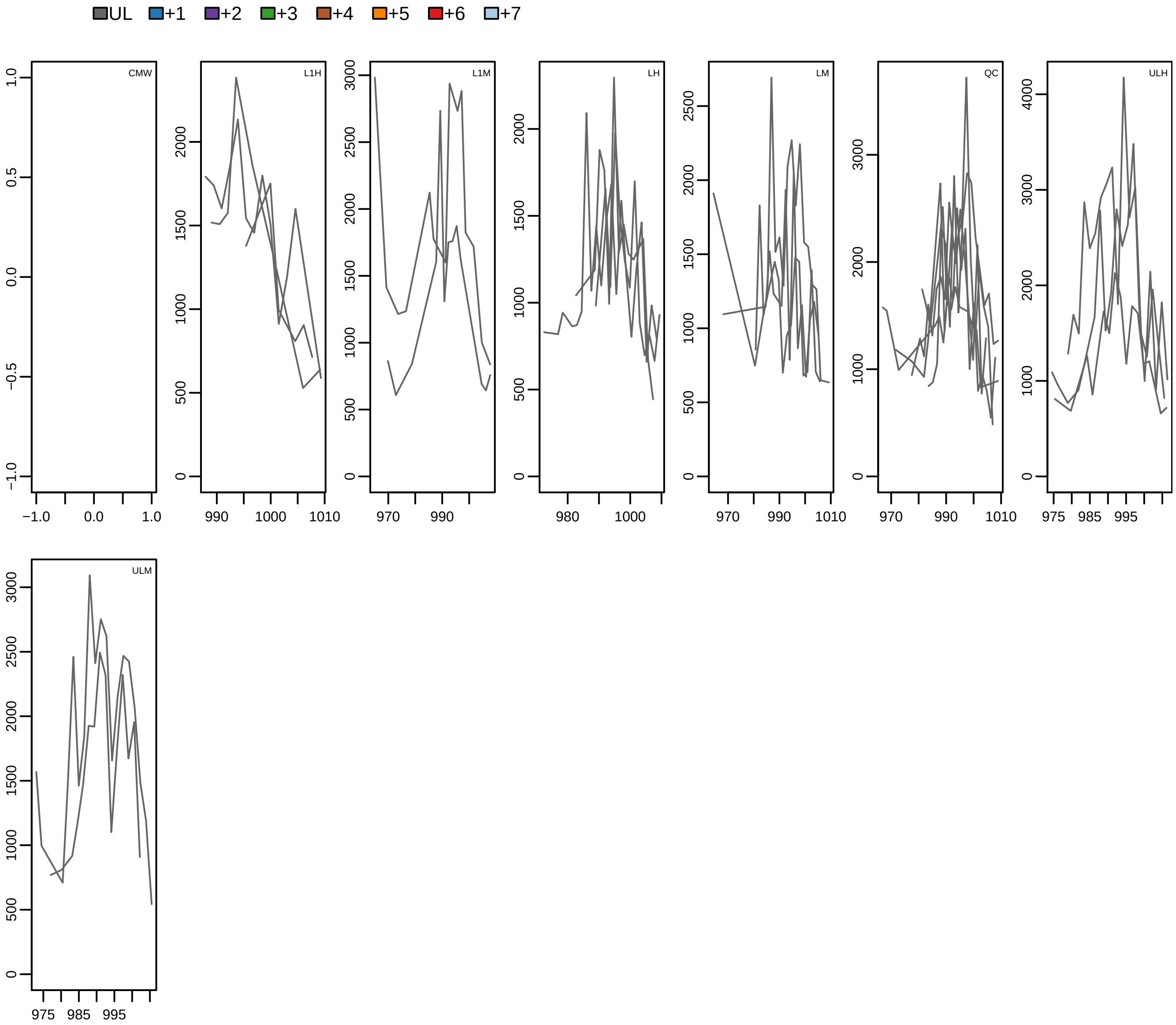

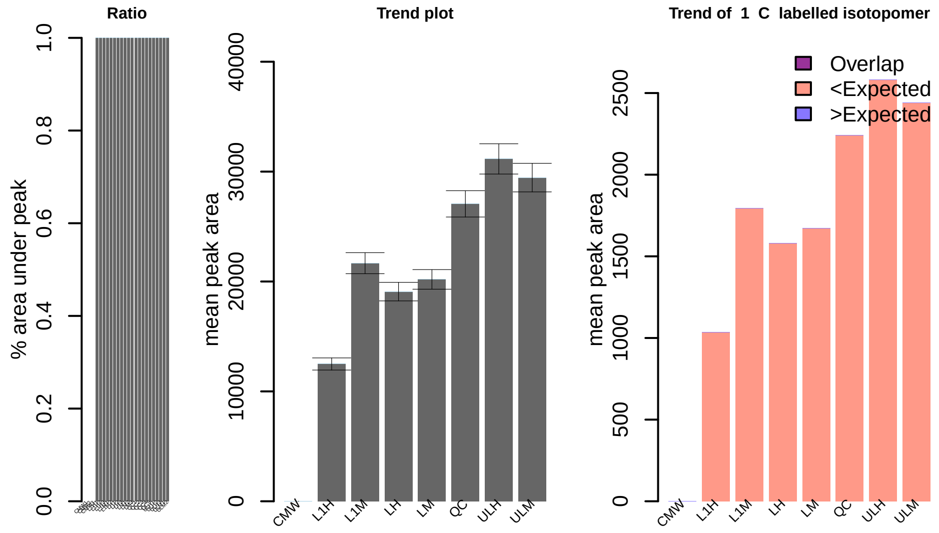

Glu−Ser

Formula: C8H14N2O6 Mass: 234.085 Std.RT: 1005.663432 Ion: NE(

G1

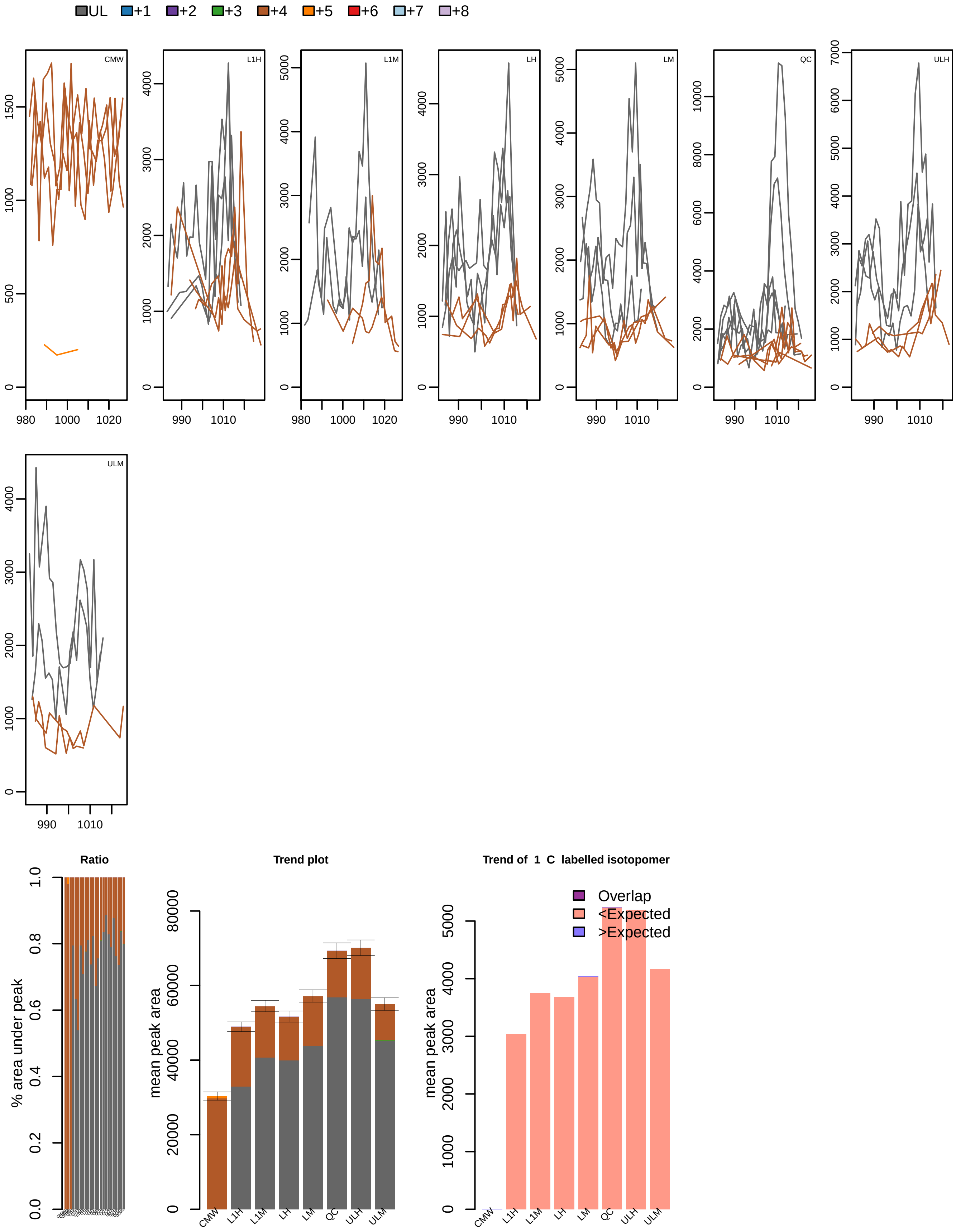

Asp−Ser

Formula: C7H12N2O6 Mass: 220.07 Std.RT: 1014.3390468 Ion: NEC

G1

UL +1 +2 +3 +4 +5 +6 +7

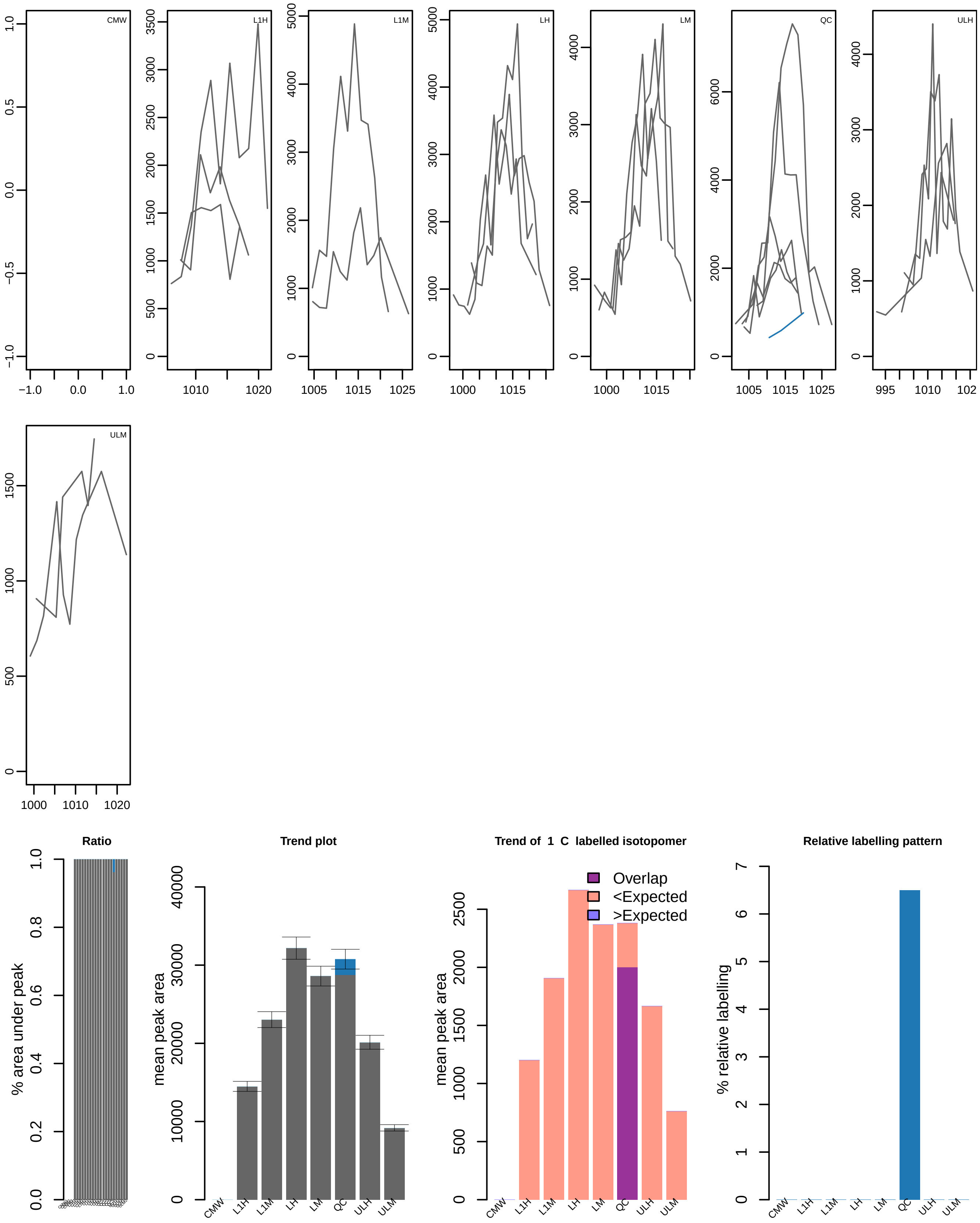

Glu−Met

Formula: C10H18N2O5S Mass: 278.094 Std.RT: 798.9294318 Ion: N

G1

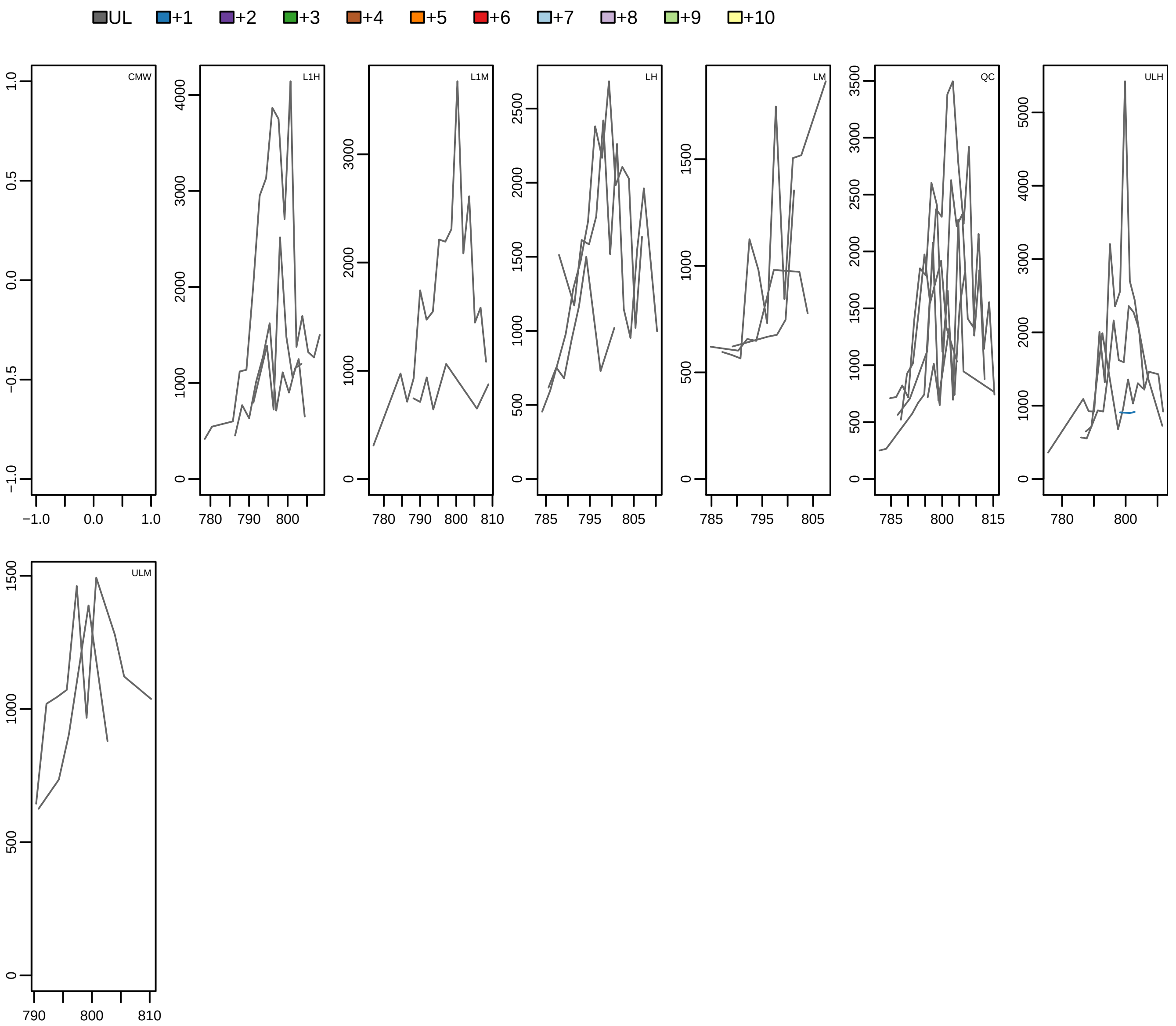

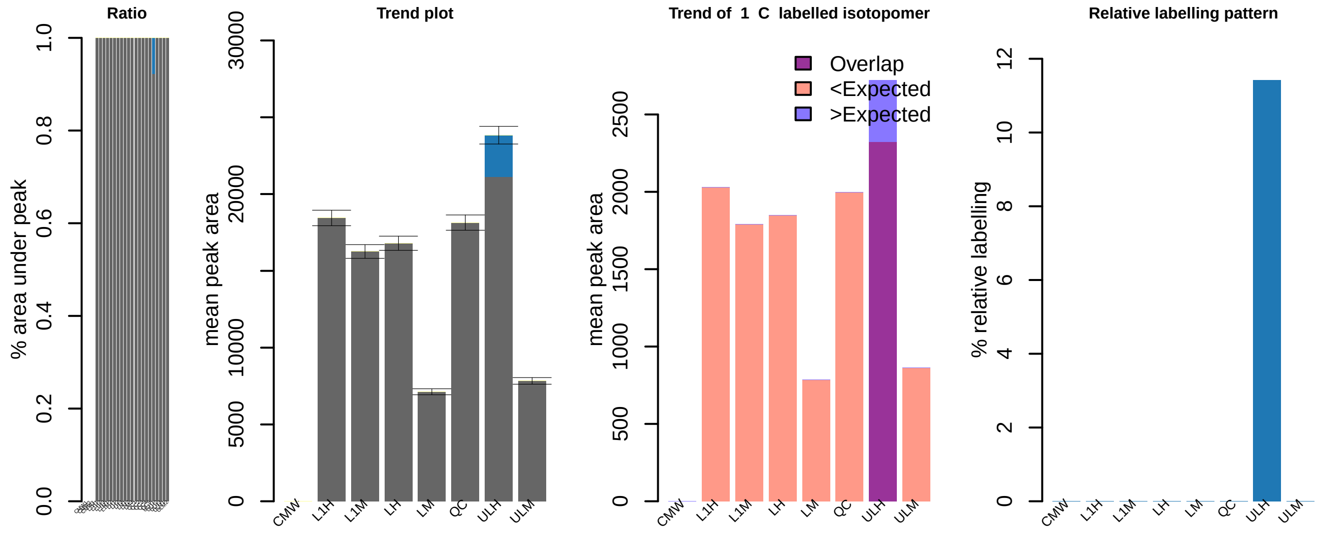

Glu−Val

G1

Formula: C10H18N2O5 Mass: 246.122 Std.RT: 799.4142864 Ion: NE

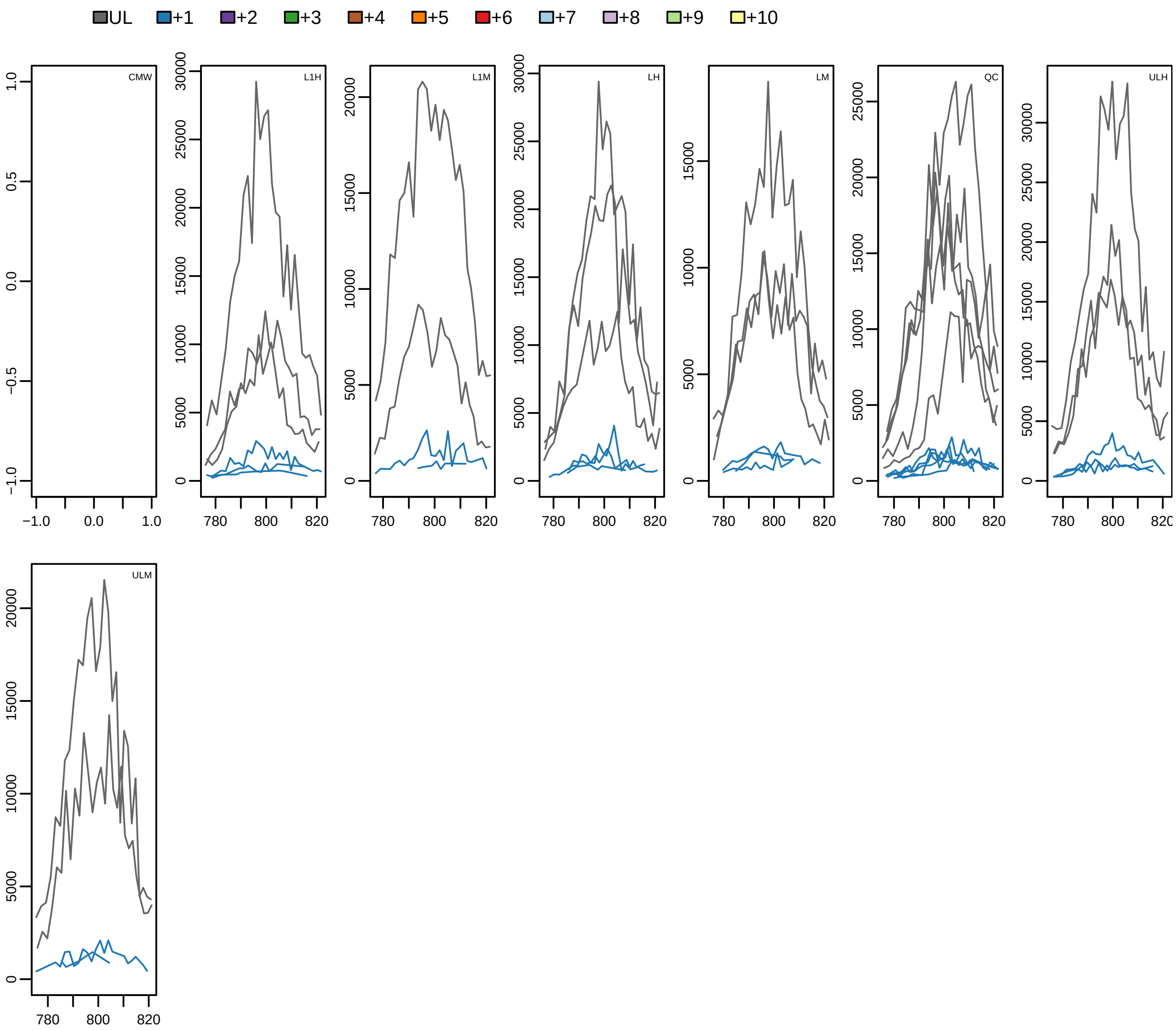

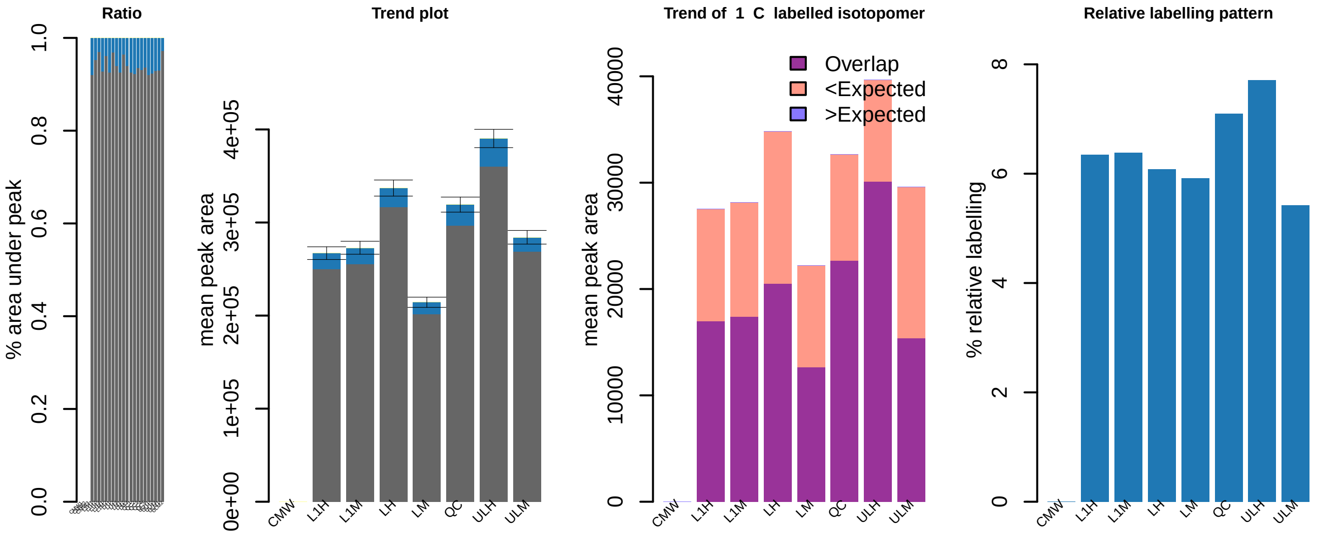

Glu−Val

G2

Formula: C10H18N2O5 Mass: 246.122 Std.RT: 799.4142864 Ion: NE

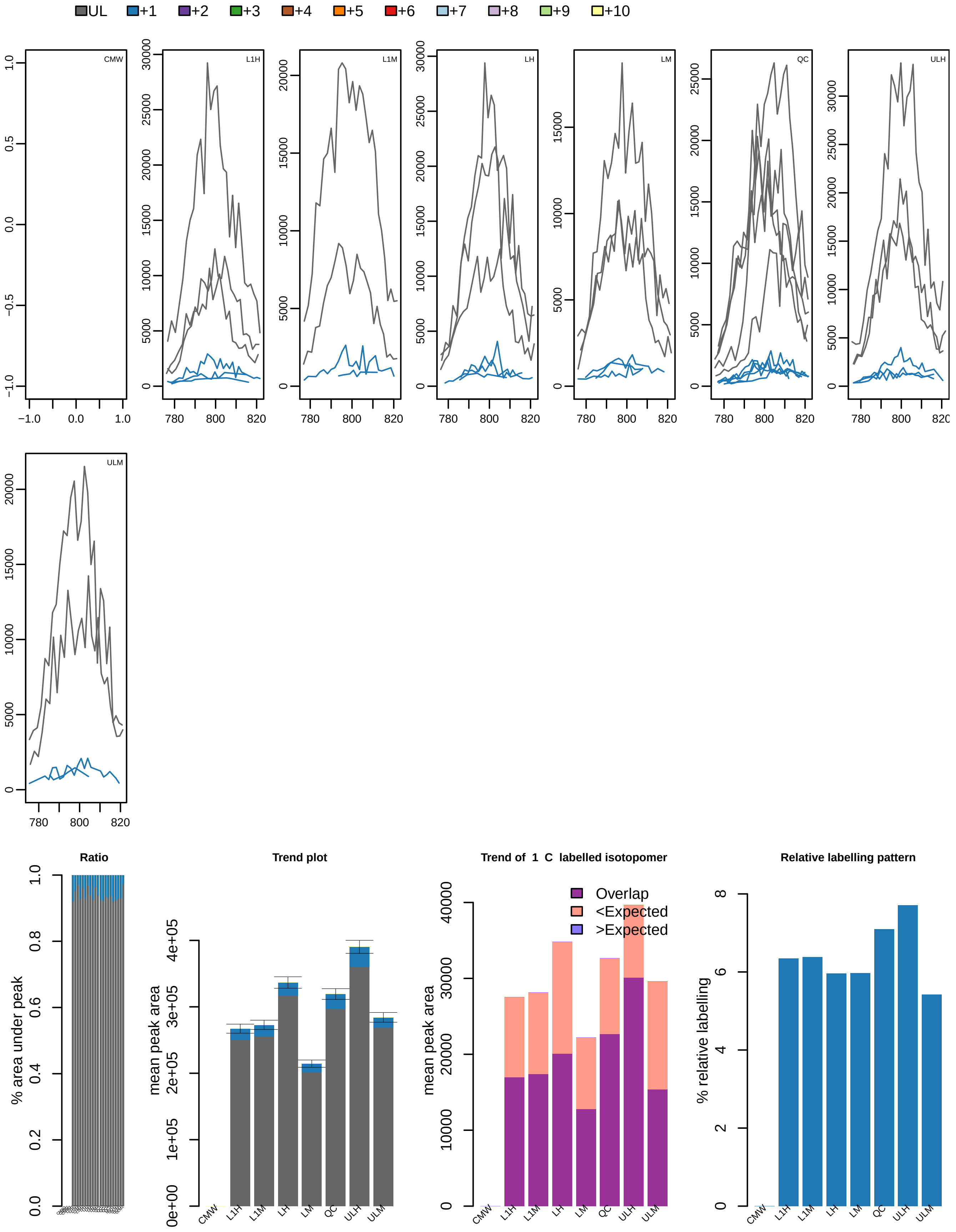

Ala−Asn

Formula: C7H13N3O4 Mass: 203.091 Std.RT: 823.980858 Ion: NEG

G1

UL +1 +2 +3 +4 +5 +6 +7

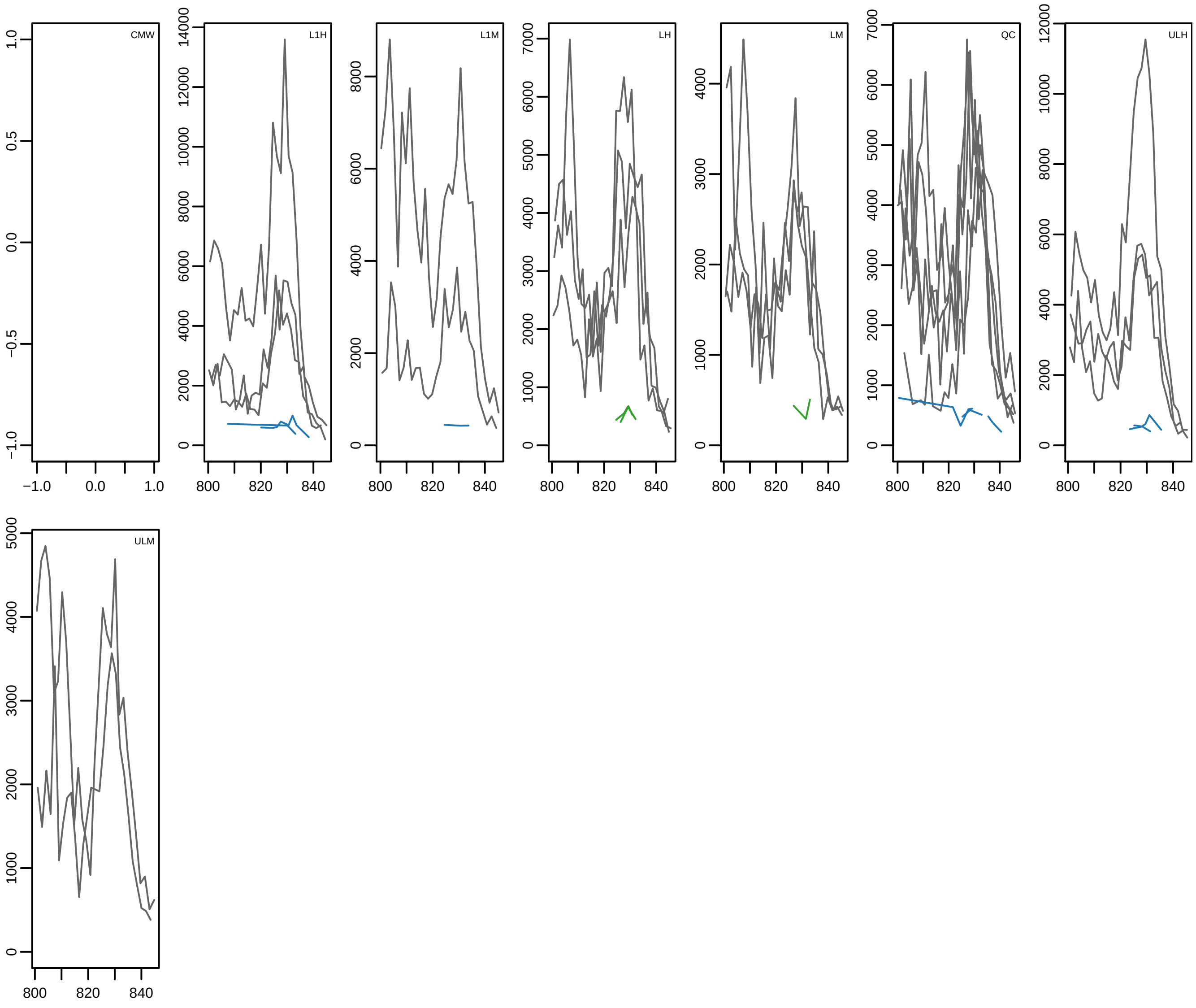

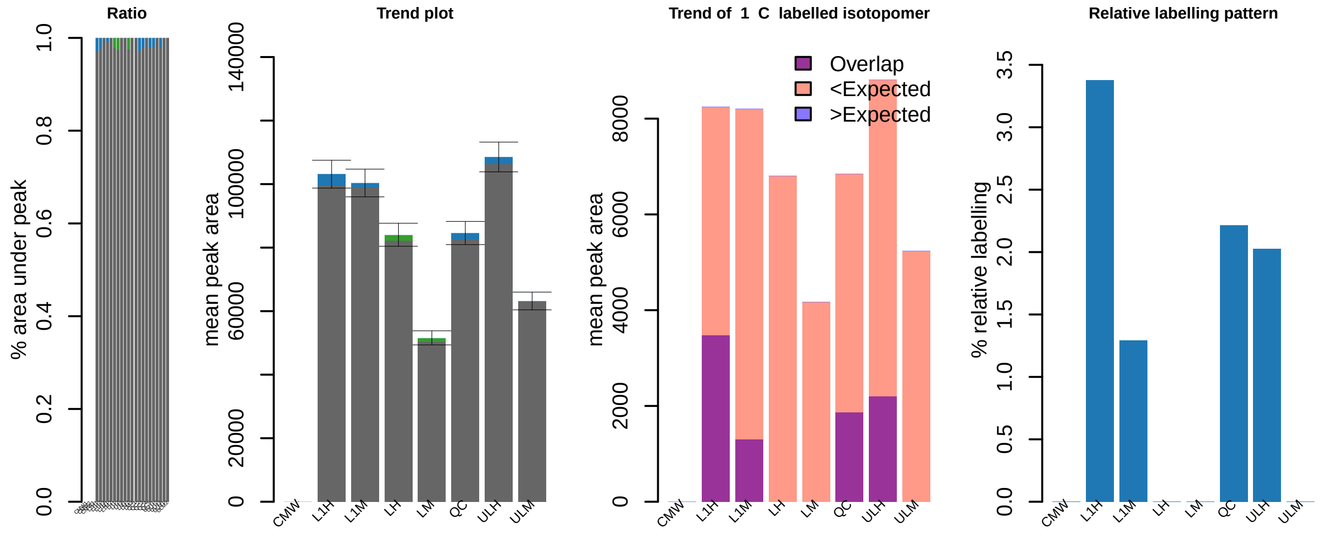

Ala−Asn

Formula: $C7H13N3O4$ Mass: 203.091 Std.RT: 823.980858 Ion: NEG

G2

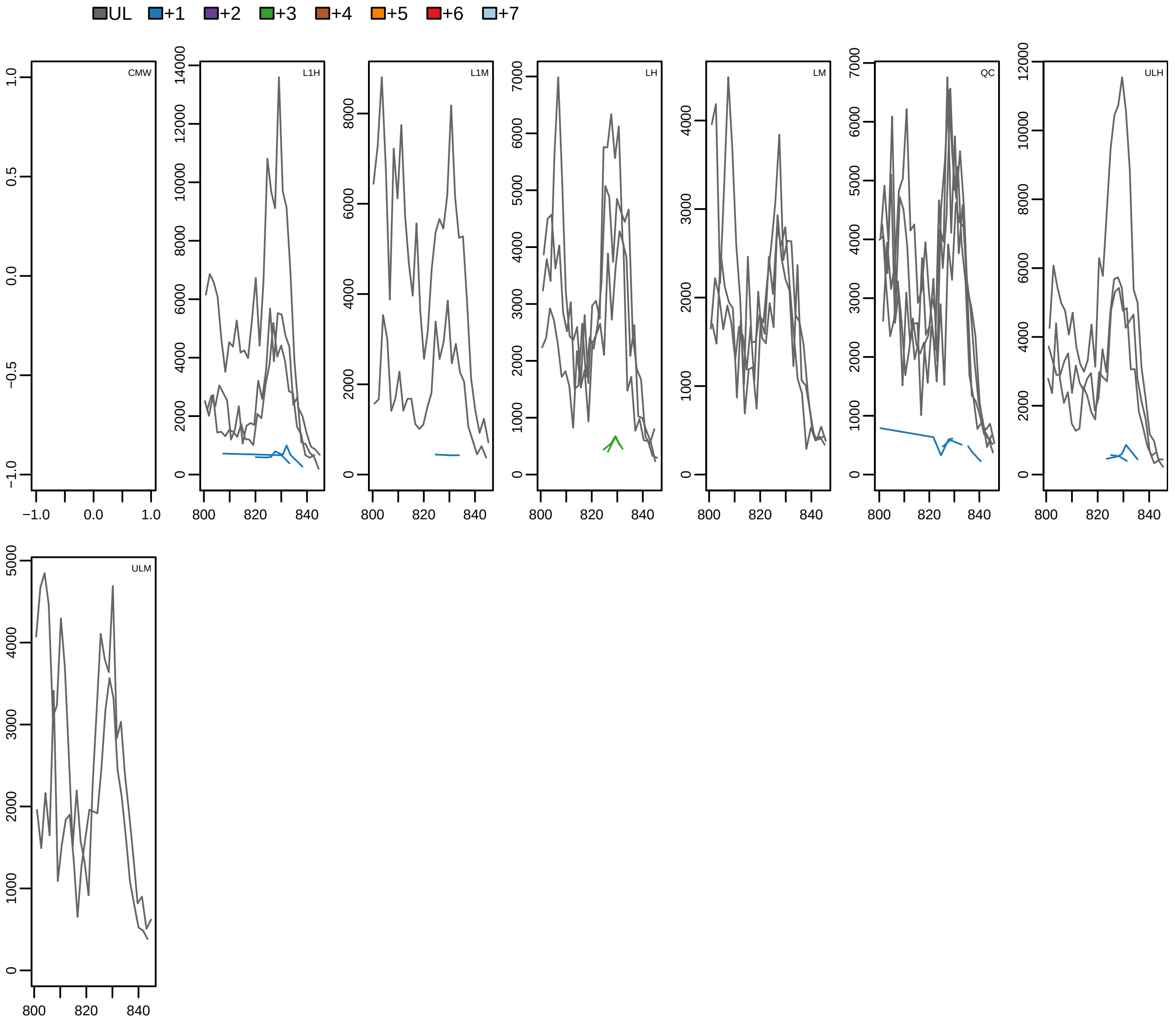

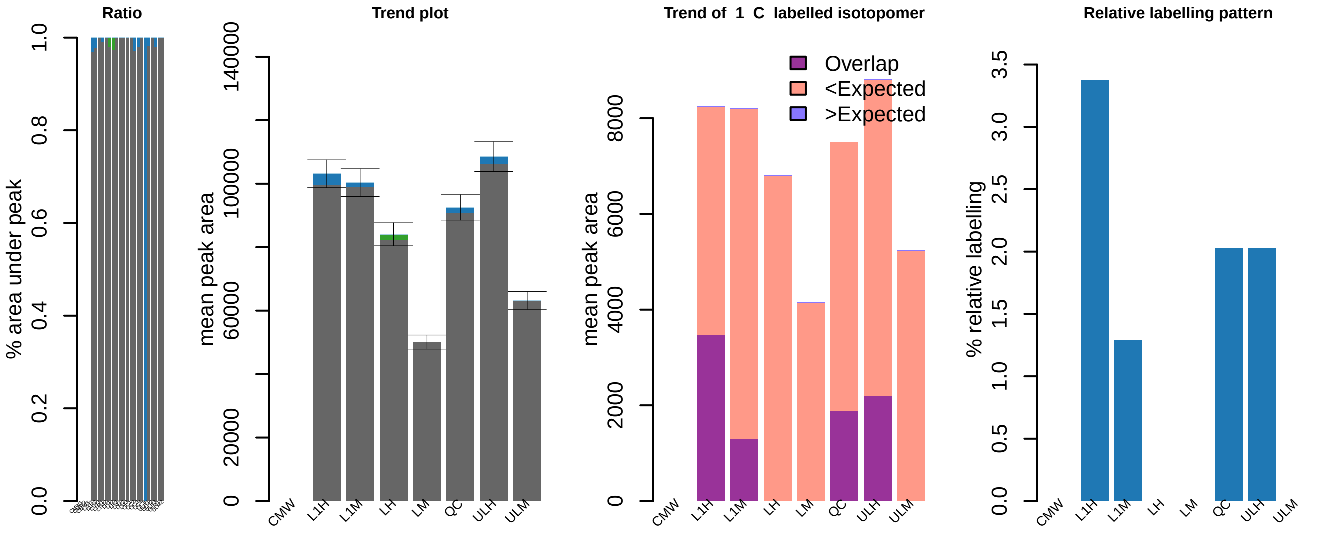

Ala−Ser

Formula: C6H12N2O4 Mass: 176.08 Std.RT: 793.912005 Ion: NEG

G1

UL +1 +2 +3 +4 +5 +6

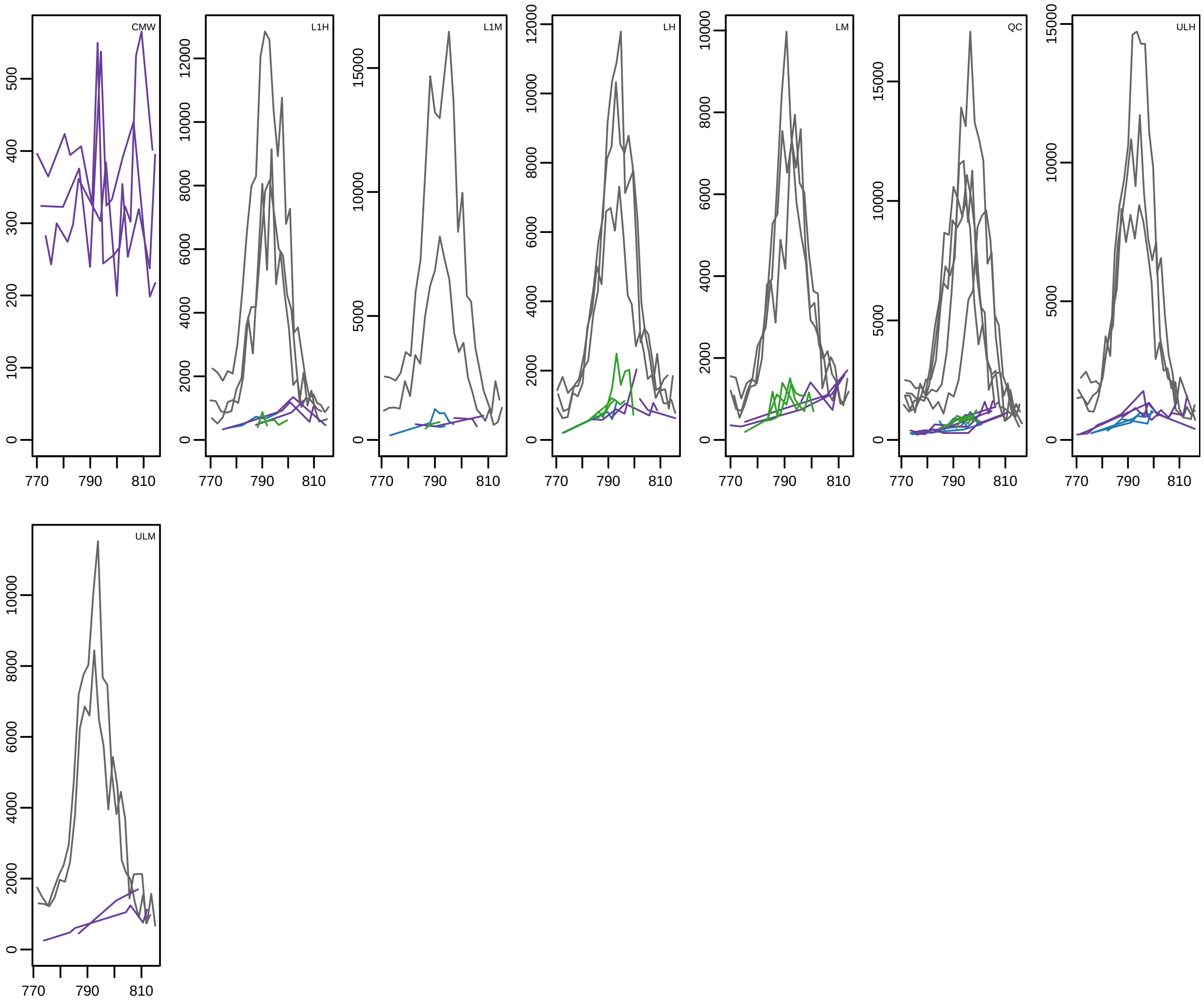

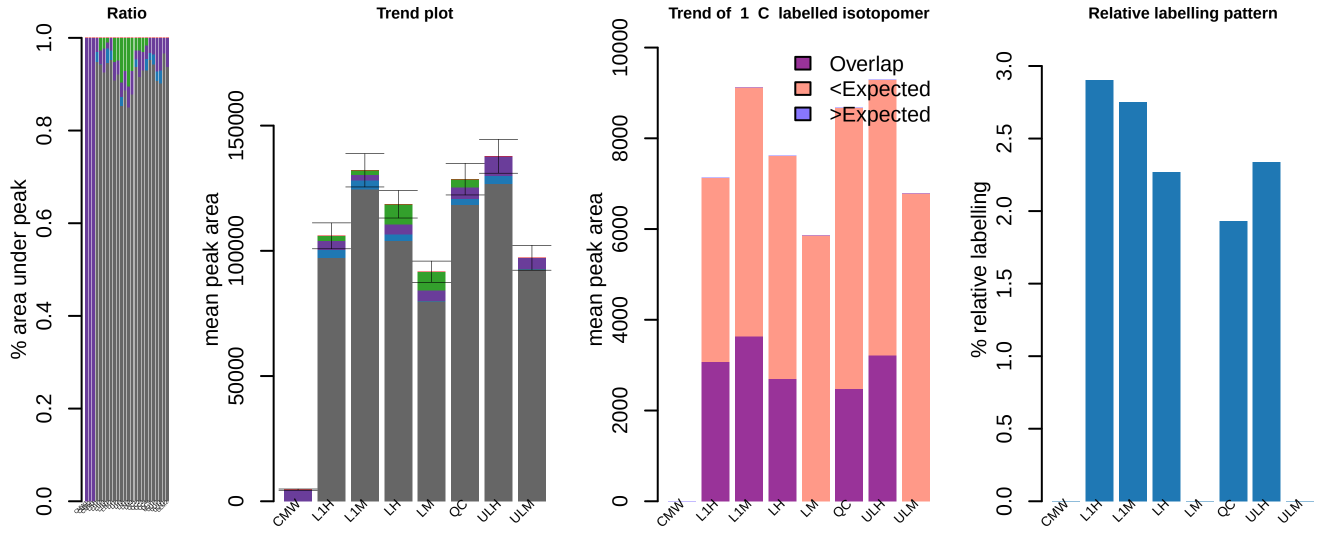

Thr−Ser

Formula: C7H14N2O5 Mass: 206.09 Std.RT: 793.0005786 Ion: NEG

G1

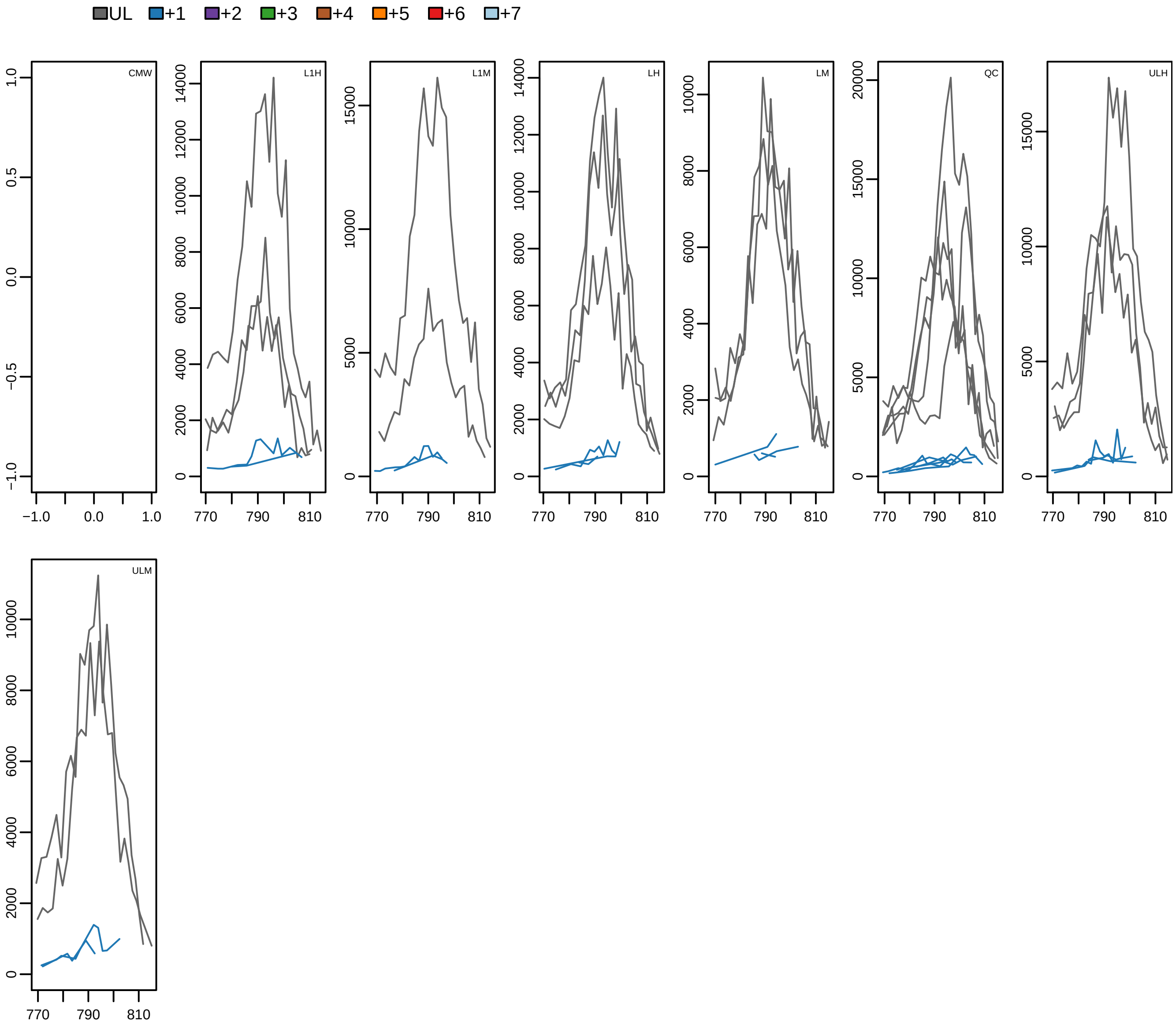

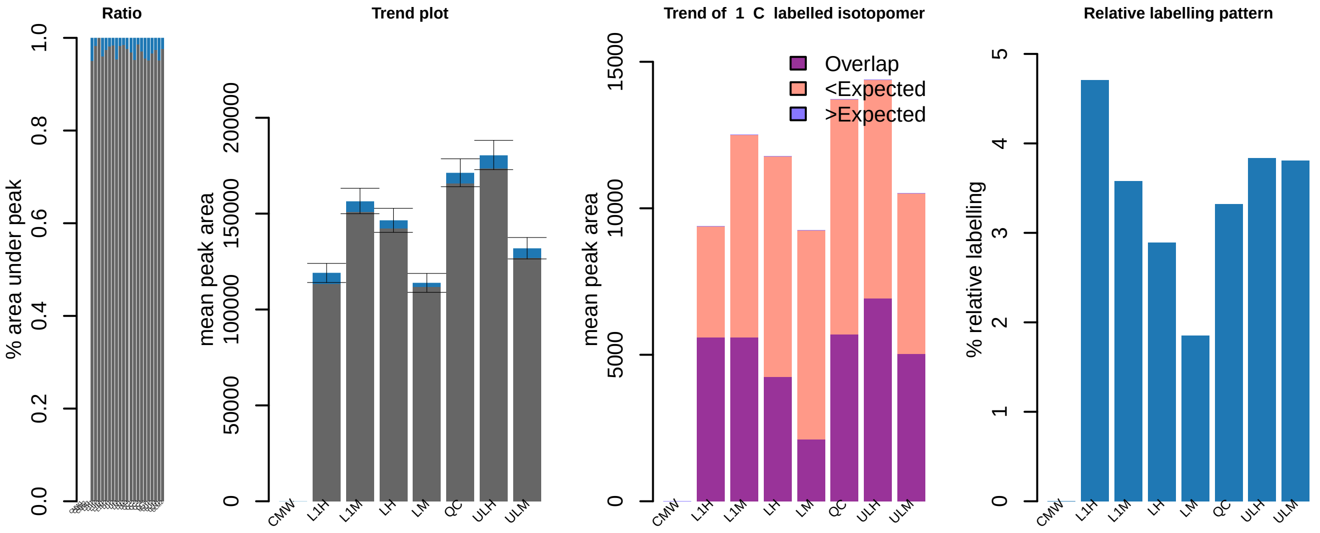

Ser−Ser

Formula: C6H12N2O5 Mass: 192.075 Std.RT: 834.8697102 Ion: NEG

G1

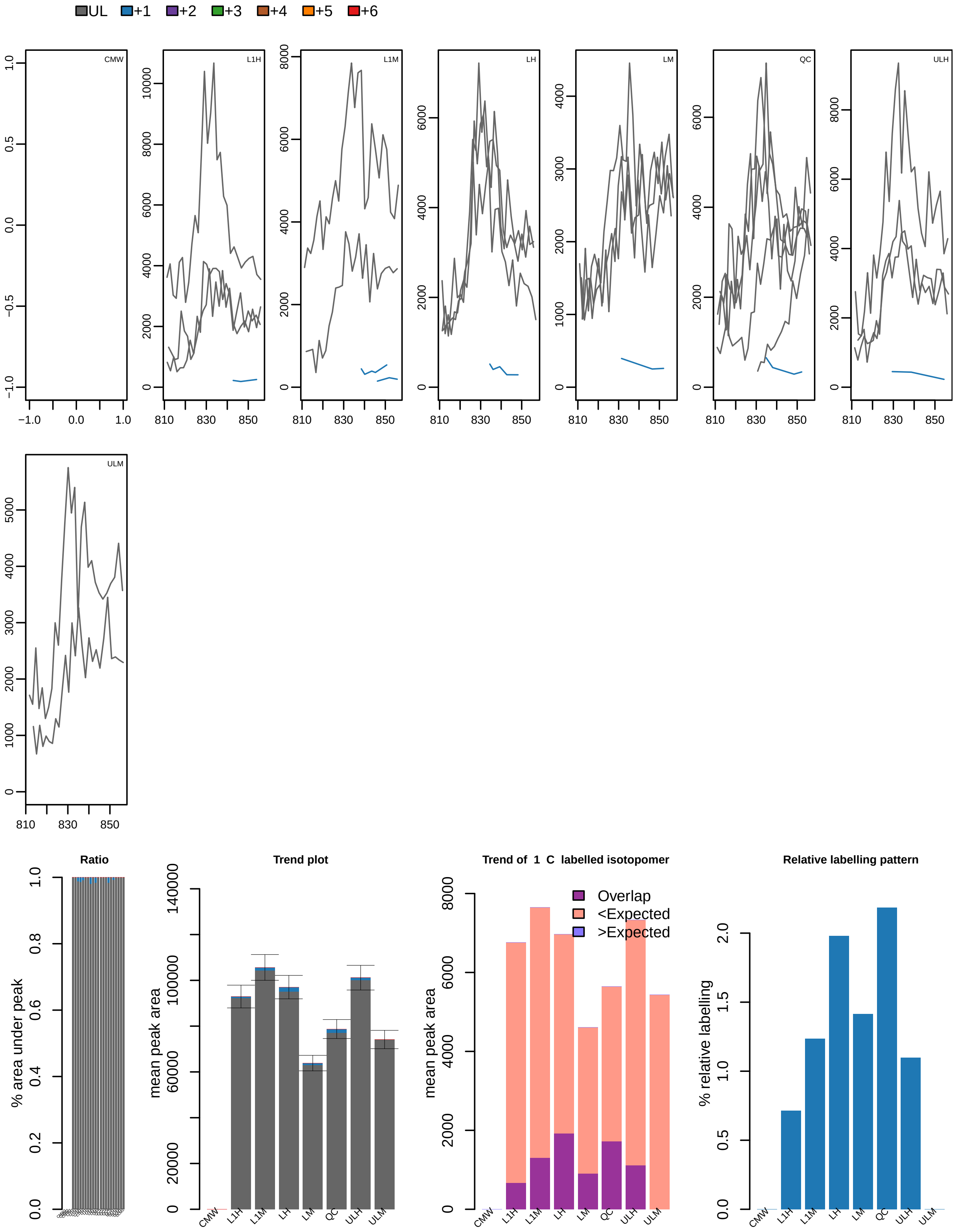

Ser−Ser

Formula: C6H12N2O5 Mass: 192.075 Std.RT: 834.8697102 Ion: NEG

G2

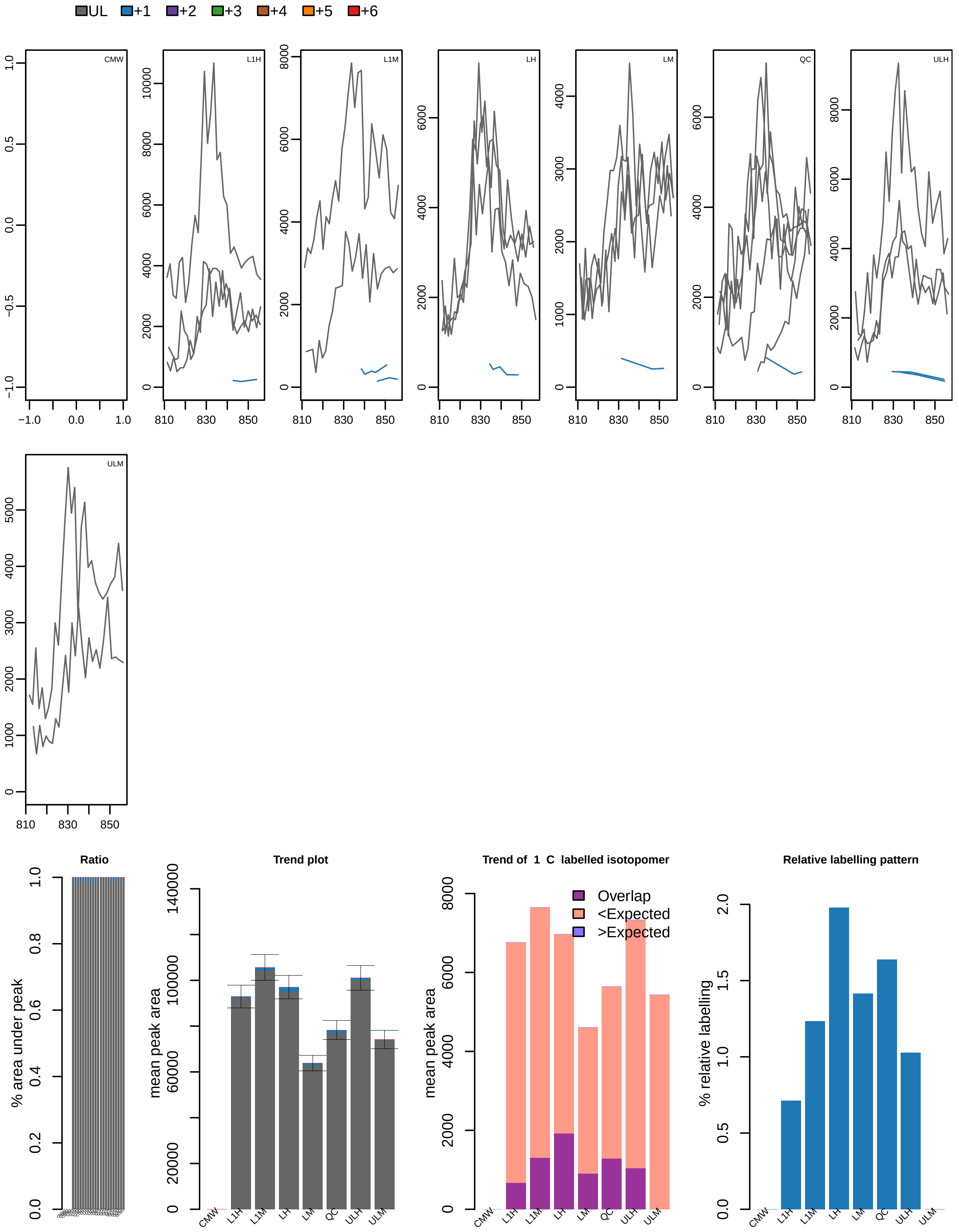

Gly−Ser

Formula: C5H10N2O4 Mass: 162.064 Std.RT: 806.5217196 Ion: NEG

G1

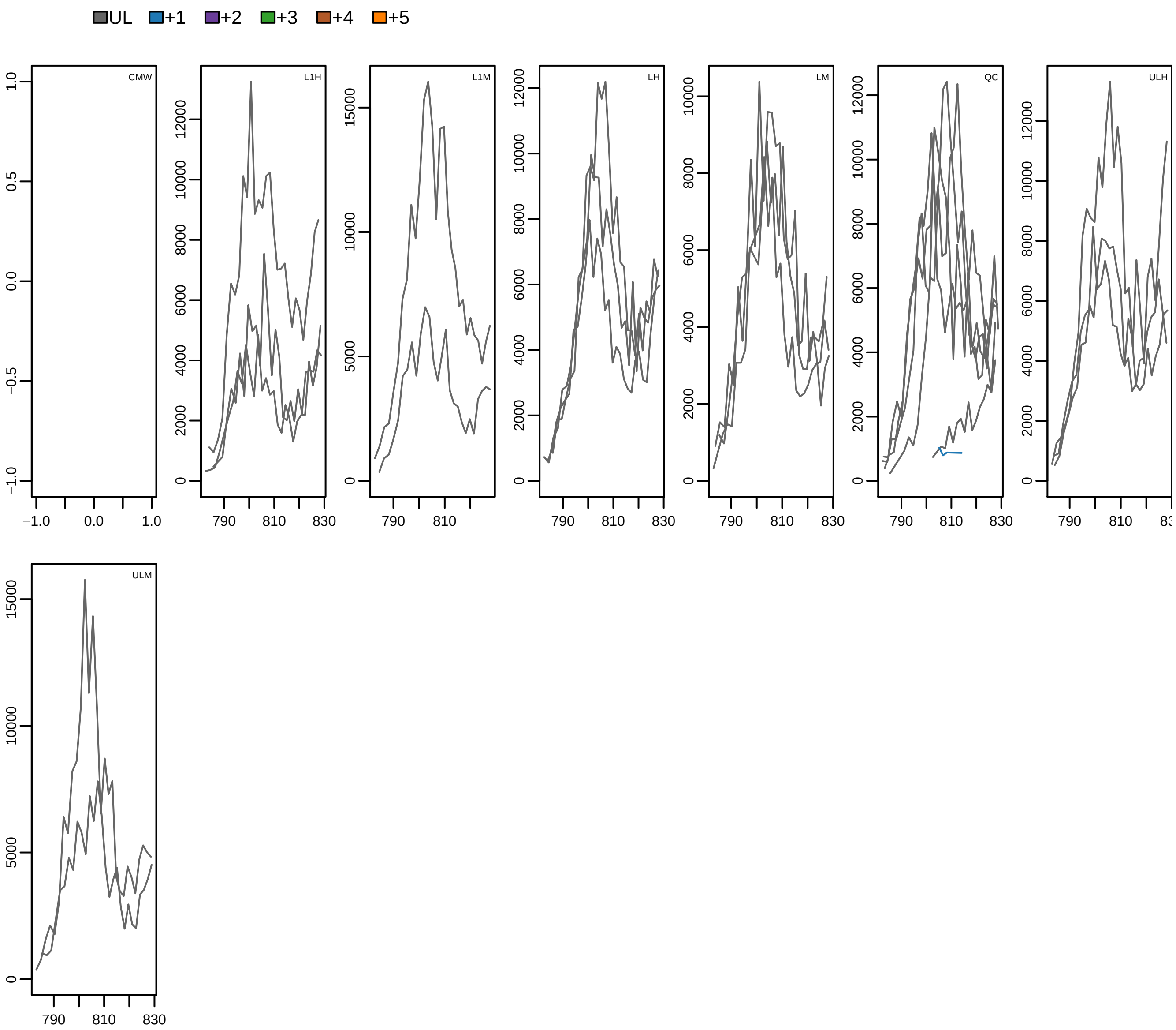

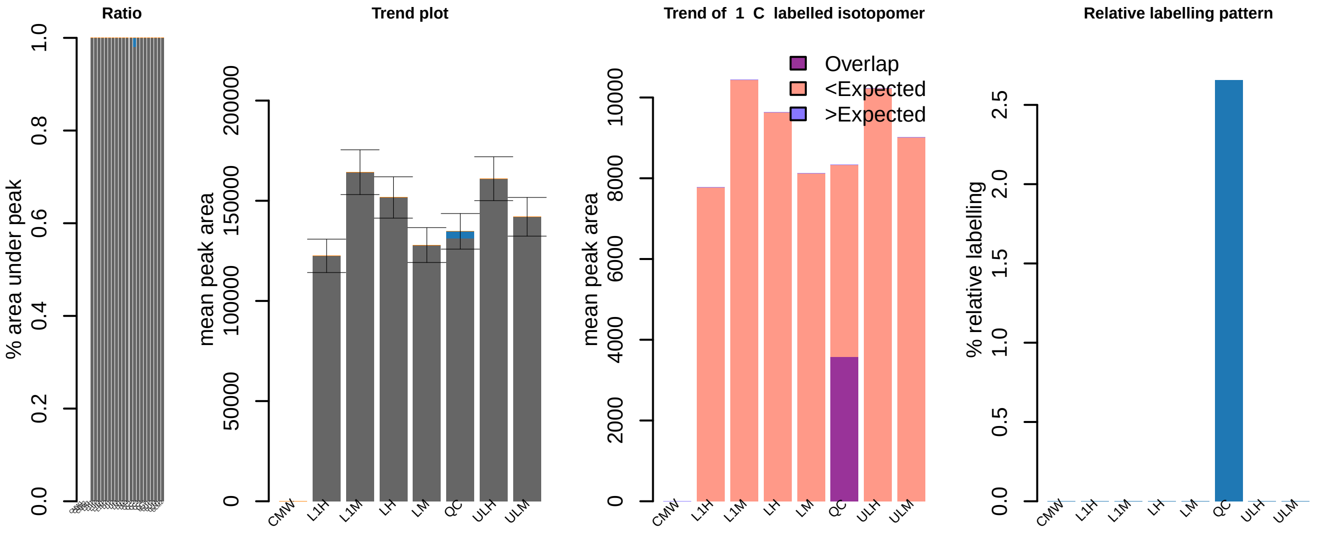

Gly−Ser

Formula: C5H10N2O4 Mass: 162.064 Std.RT: 806.5217196 Ion: NEC

G2

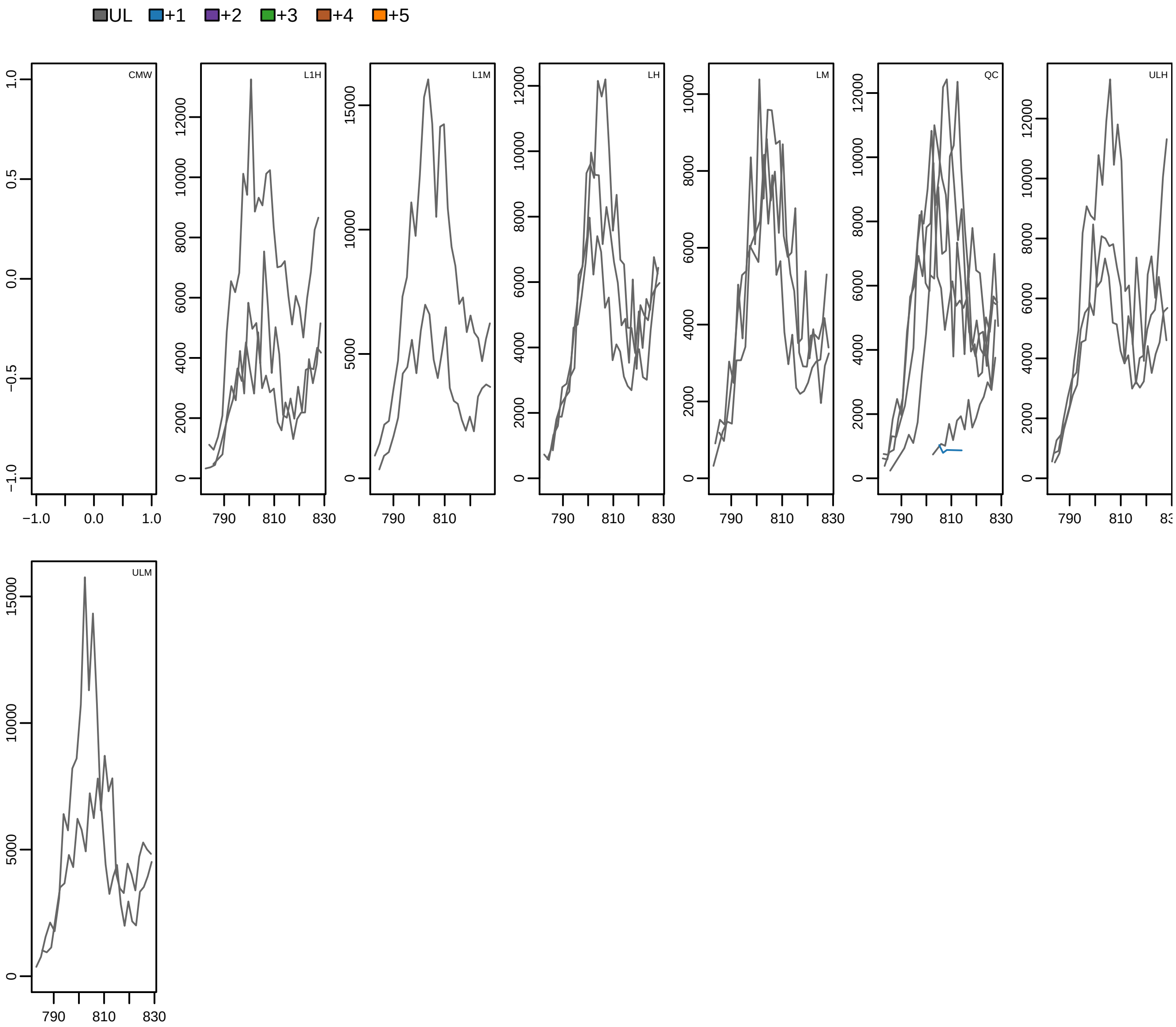

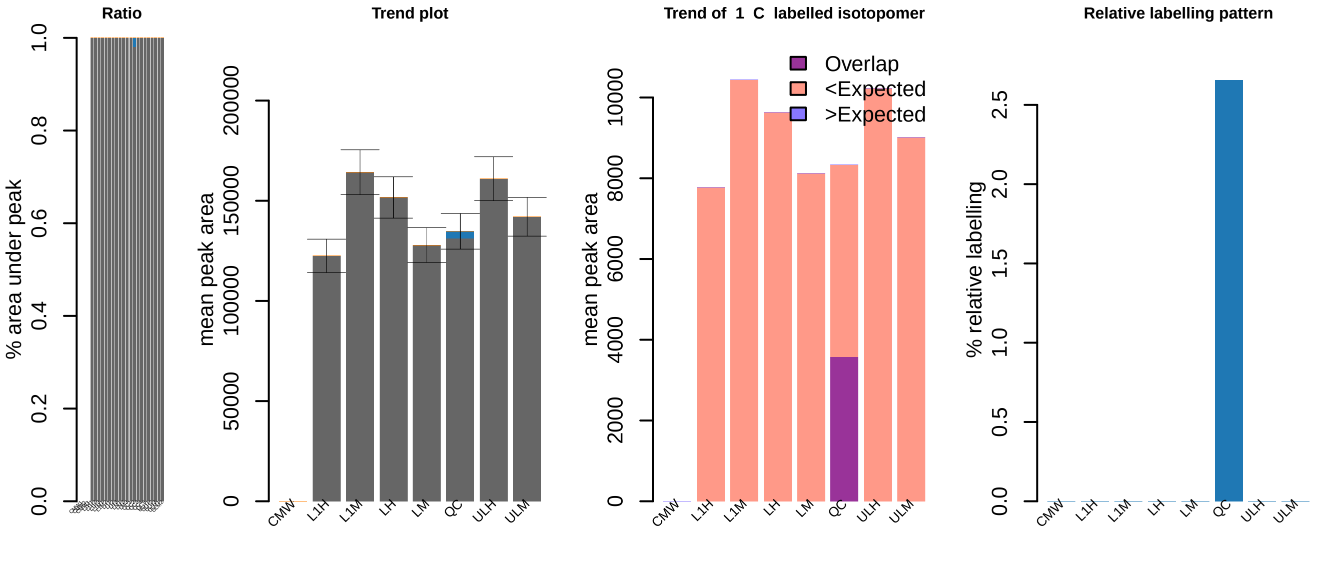

Asp−Met−Thr−Thr

Formula: C17H30N4O9S Mass: 466.173 Std.RT: 482.18185122 Ion:

G1

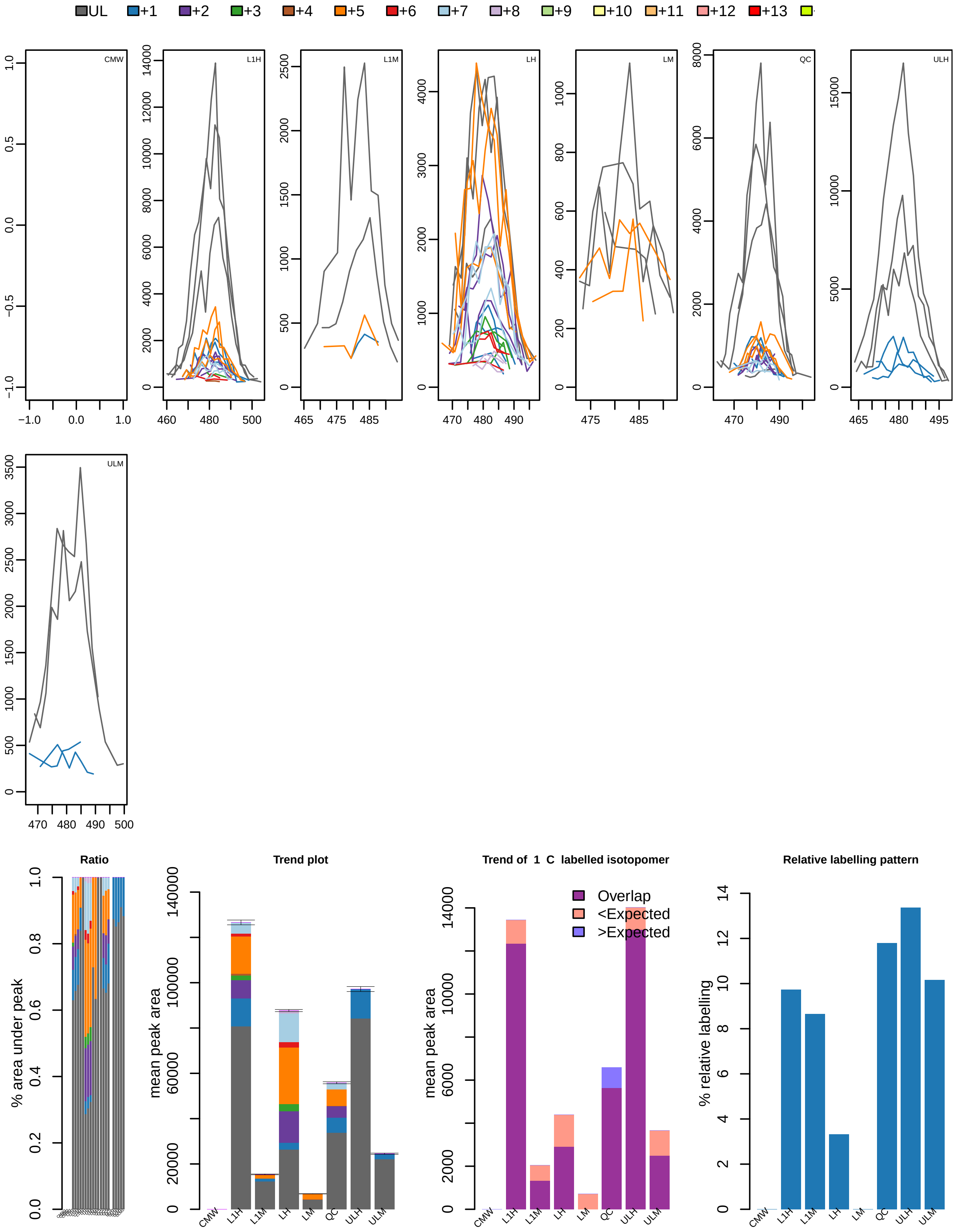

Ala−Gly−Ser

Formula: C8H15N3O5 Mass: 233.101 Std.RT: 806.8540008 Ion: NEG

G1

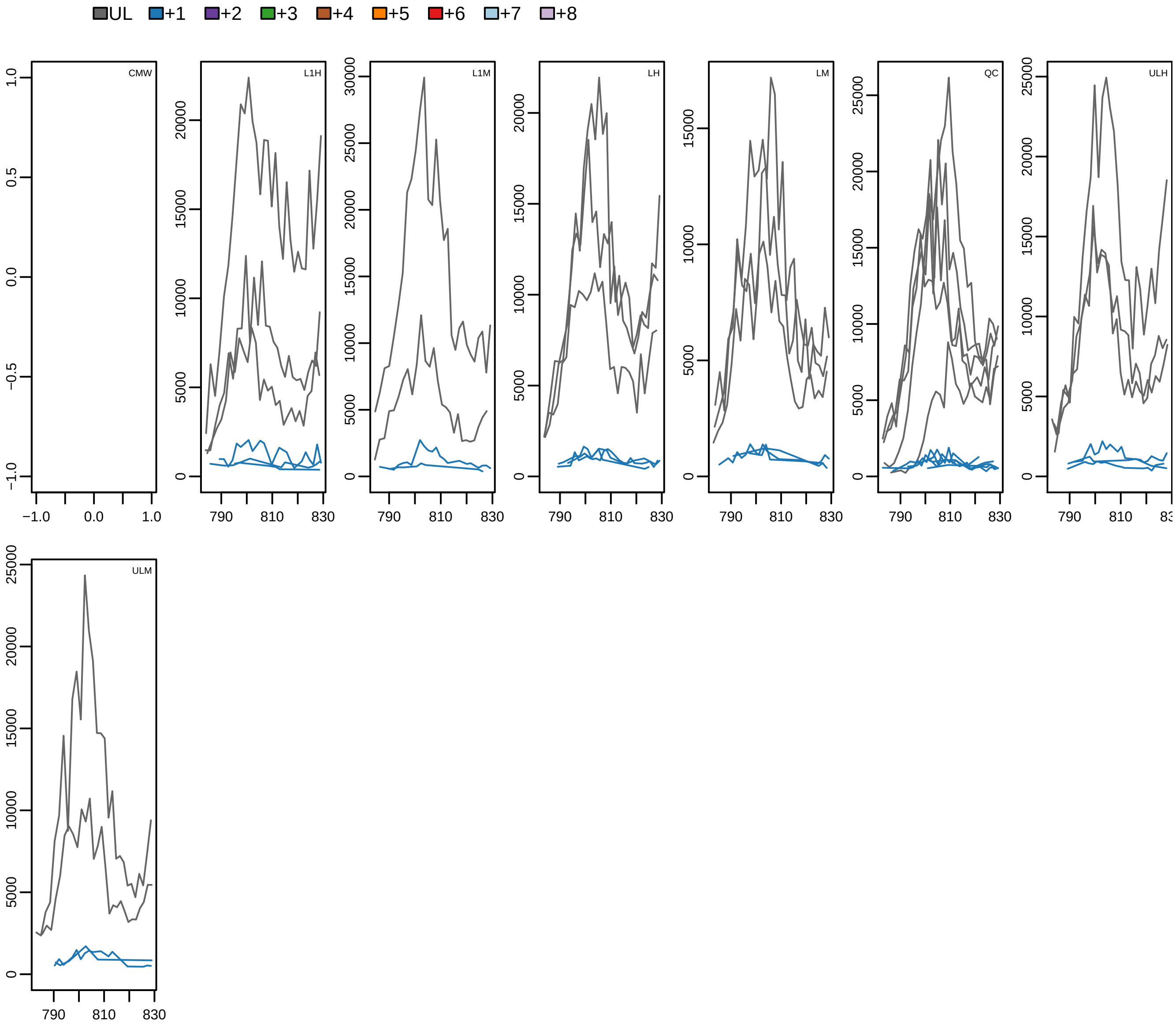

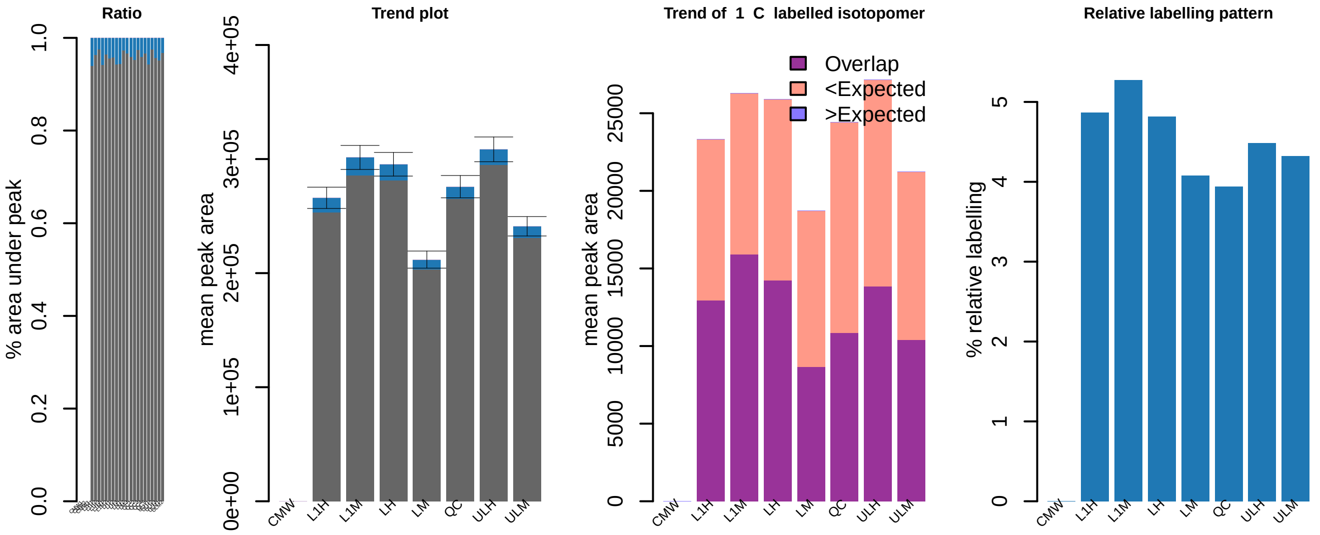

Parapyruvate

Formula: C6H8O6 Mass: 176.032 Std.RT: 966.0884982 Ion: NEG

G1

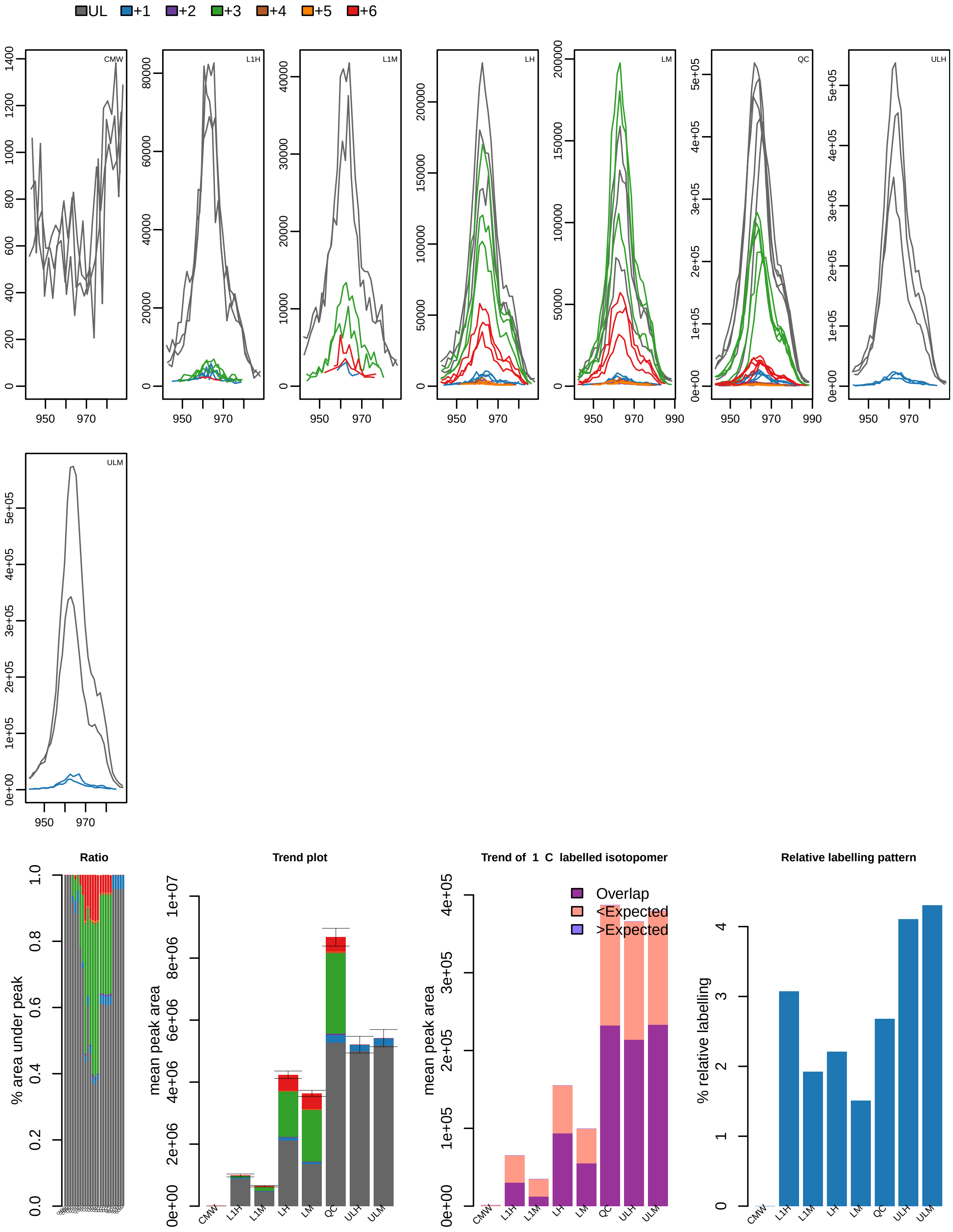

2−Maleylacetate

Formula: $C_6H_6O_5$ Mass: 158.022 Std.RT: 914.7904992 Ion: NEG

G1

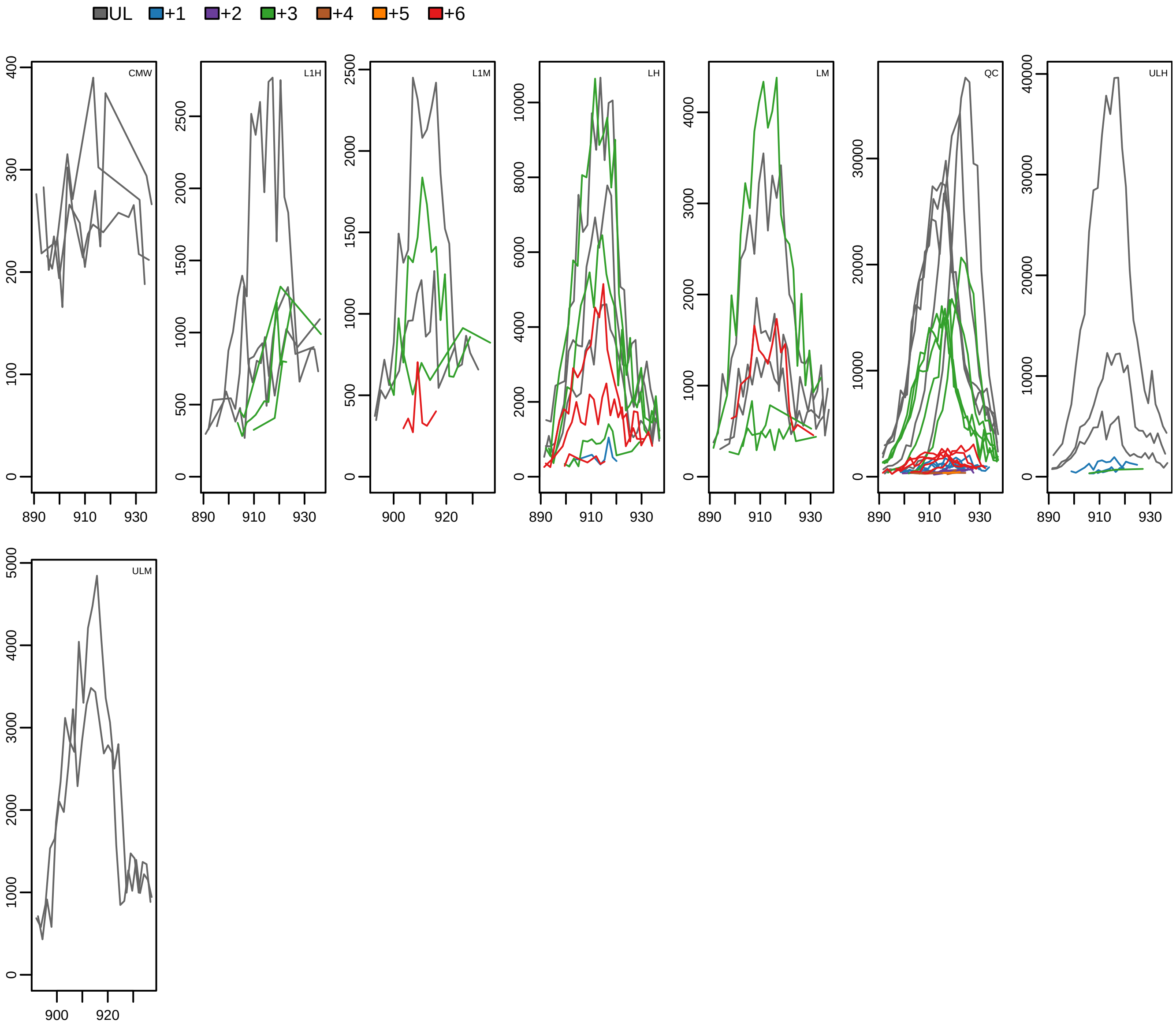

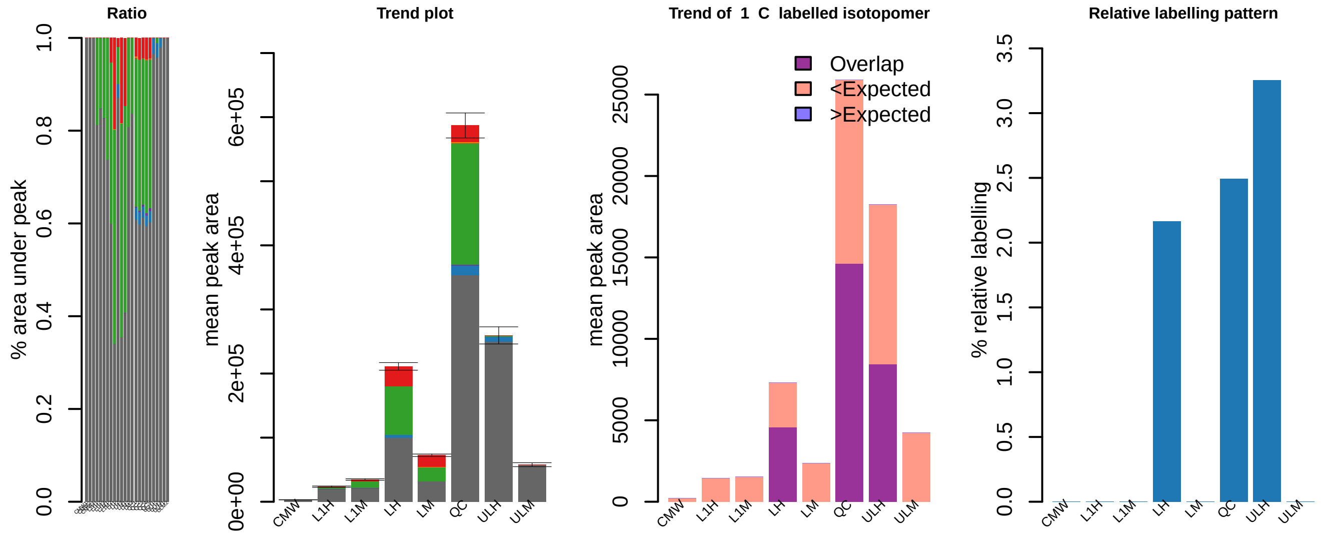

Phosphophosphinate

Formula: C6H15NO7P2 Mass: 275.032 Std.RT: 1021.1307612 Ion: N

G1

UL +1 +2 +3 +4 +5 +6

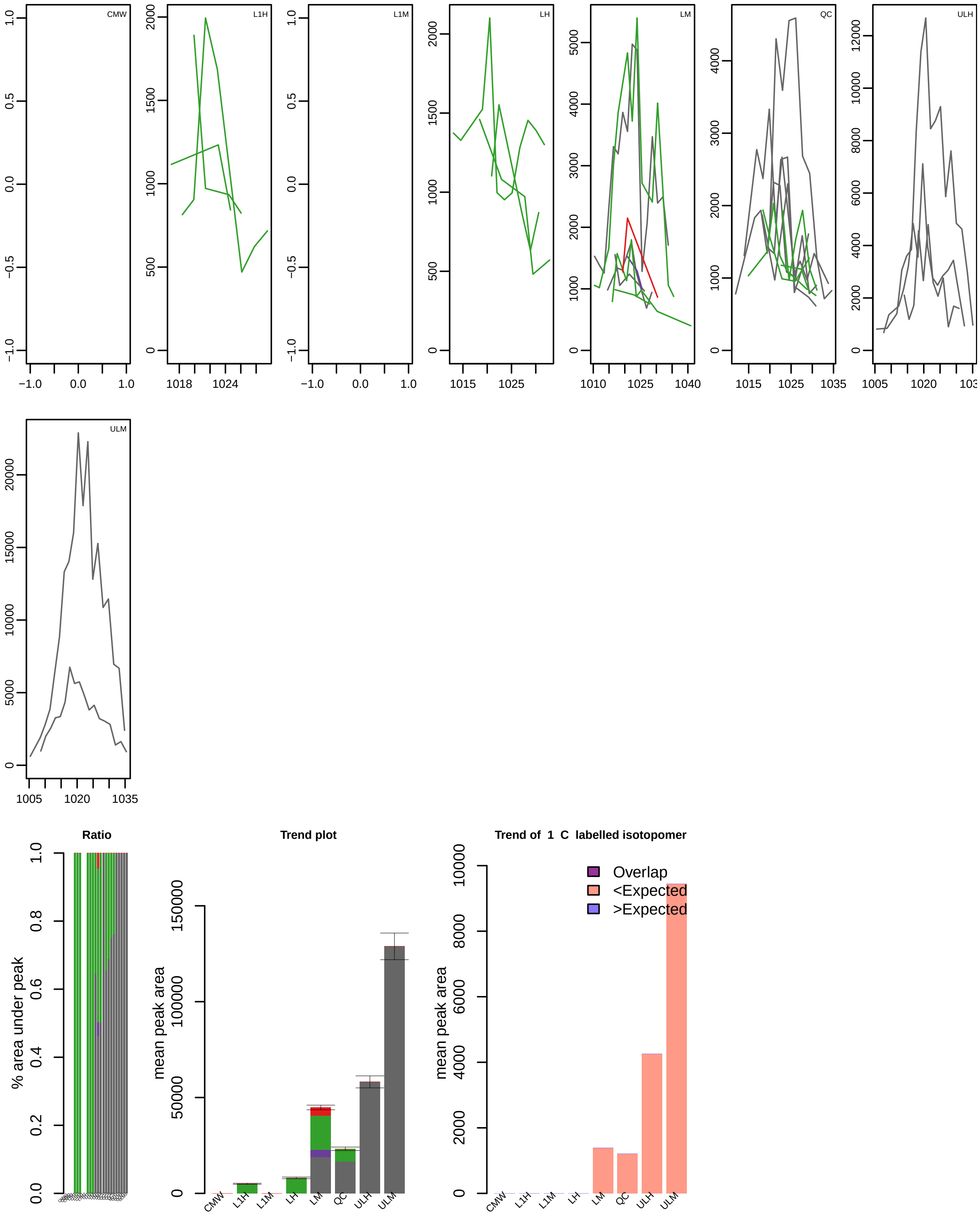

1−18:2−lysophosphatidylglycerol

Formula: C24H45O9P Mass: 508.28 Std.RT: 214.36029318 Ion: NEC

G1

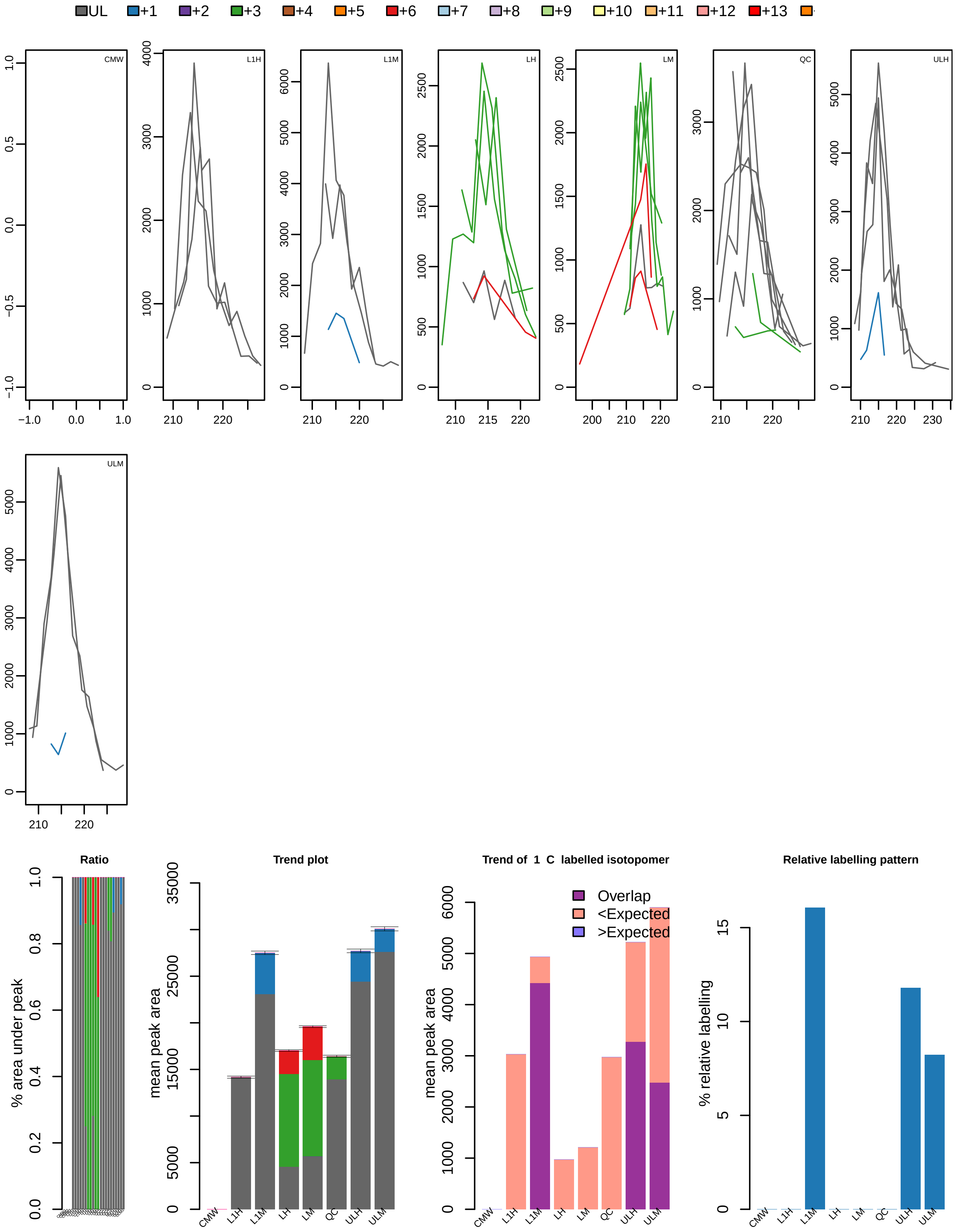

octulose 8−phosphate

Formula: C8H17O11P Mass: 320.051 Std.RT: 1079.8638042 Ion: NE

G1

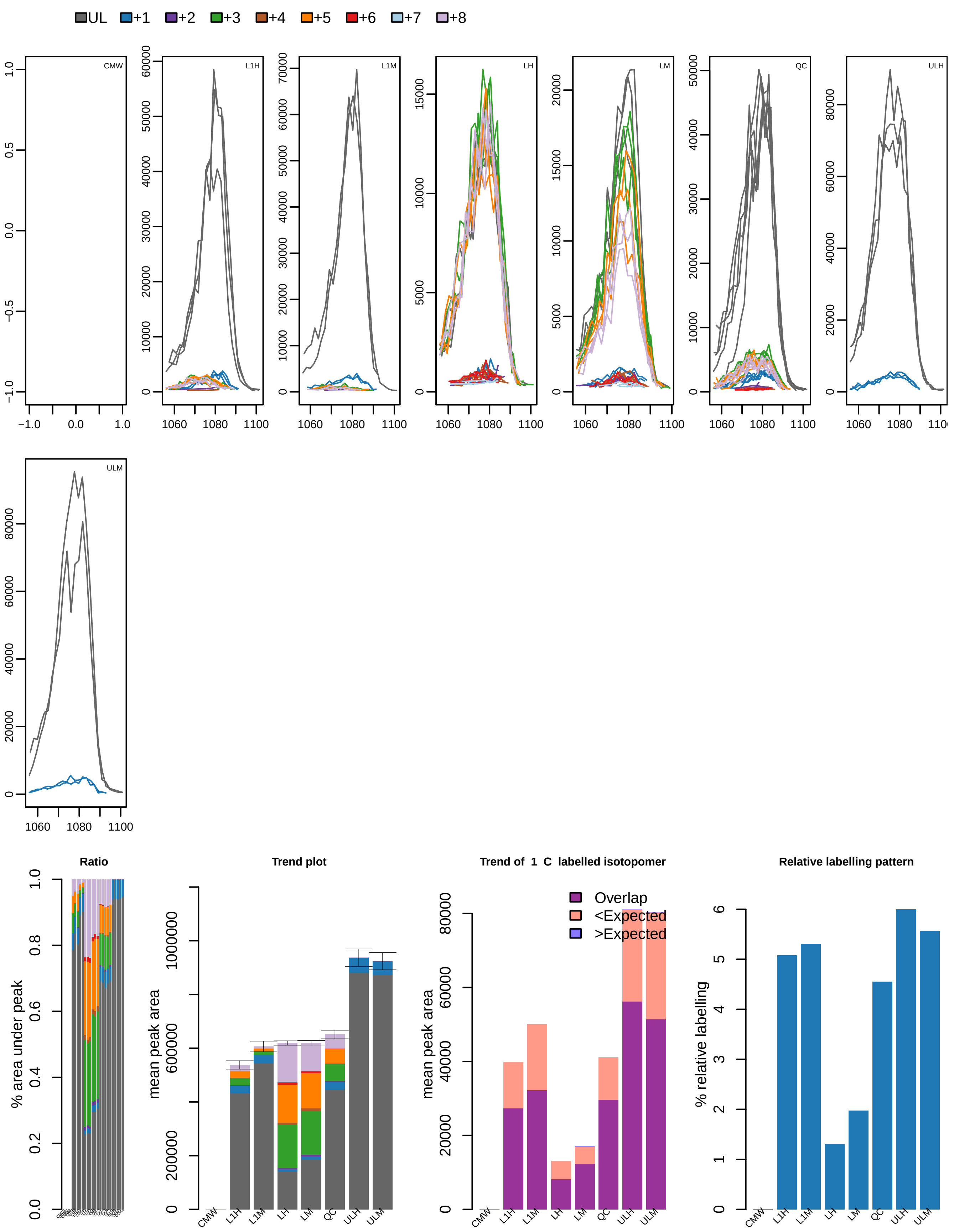

nonulose 9−phosphate

Formula: C9H19O12P Mass: 350.061 Std.RT: 1088.133336 Ion: NEC

G1

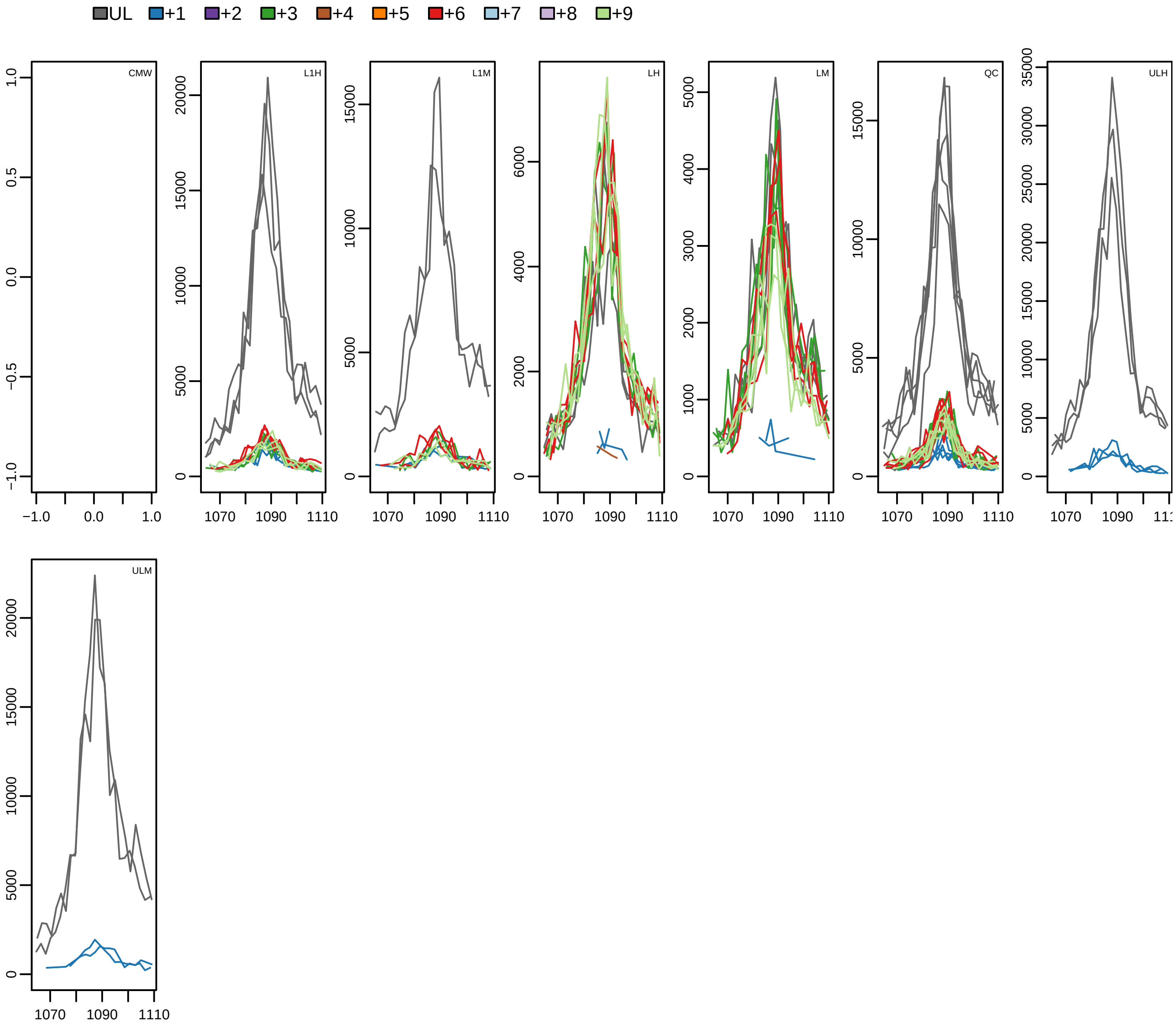

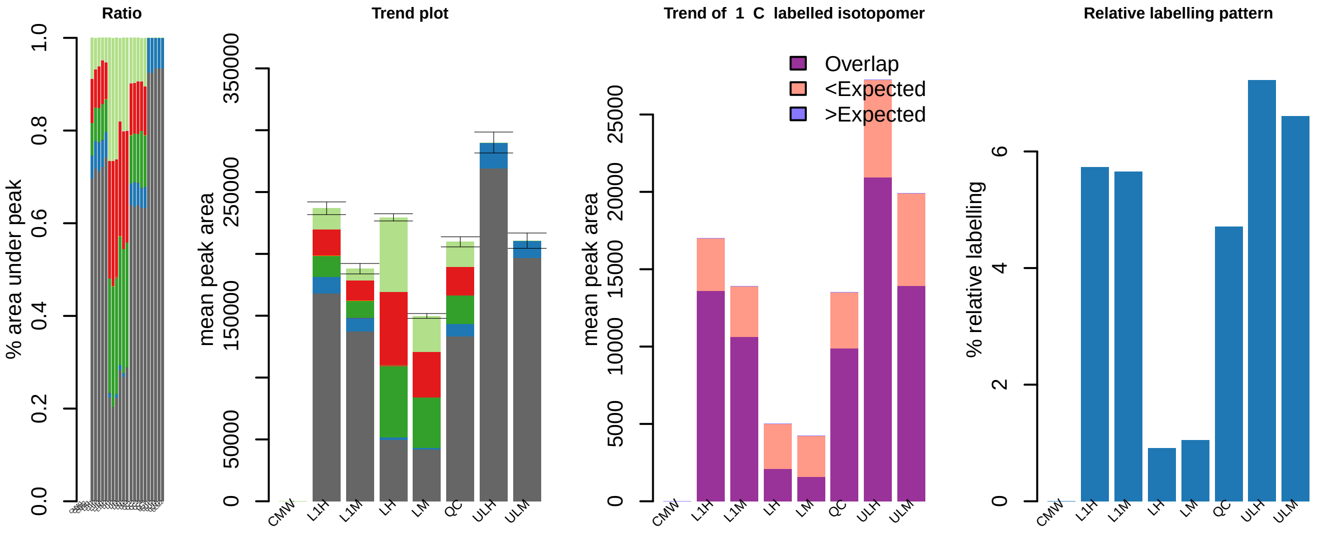

Glycerophosphoglycerol

Formula: C6H15O8P Mass: 246.05 Std.RT: 811.0402776 Ion: NEG

G1

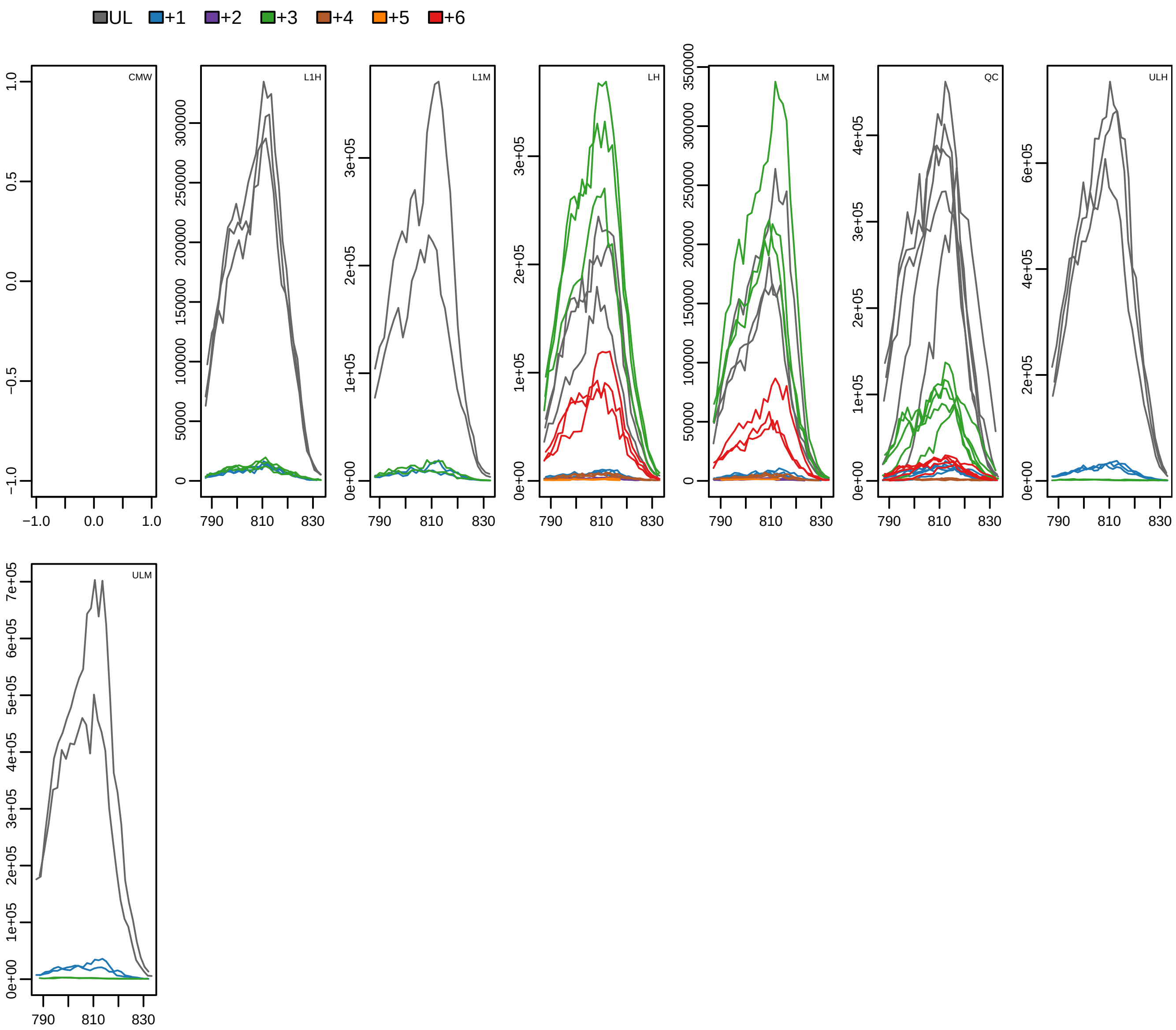

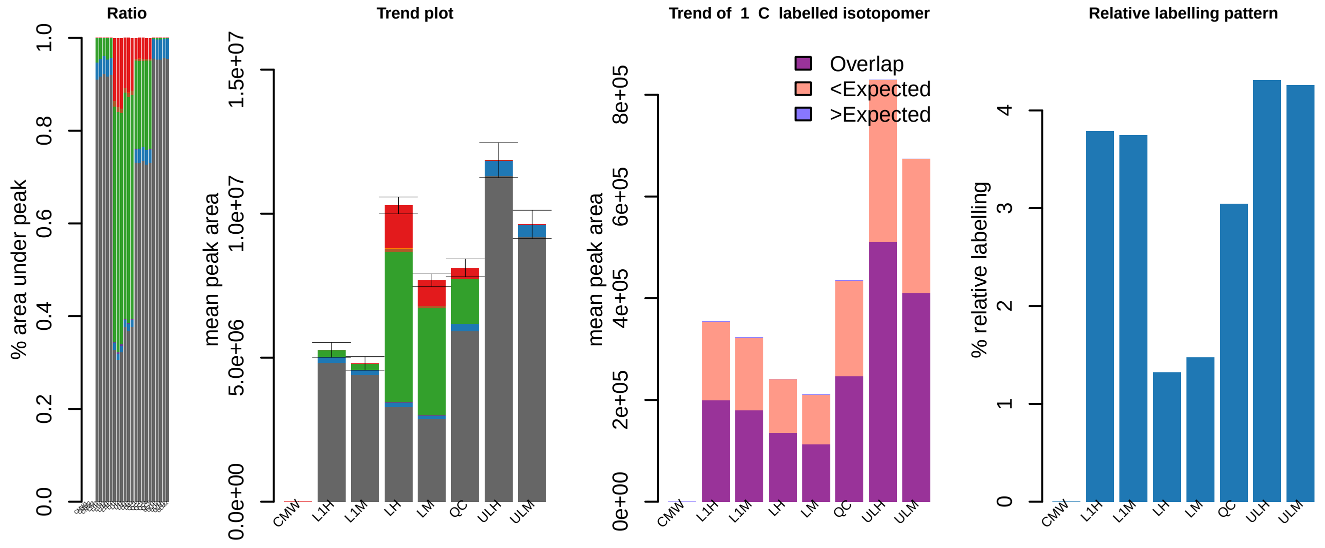

P−DPD

Formula: C5H9O7P Mass: 212.009 Std.RT: 797.7542814 Ion: NEG

G1

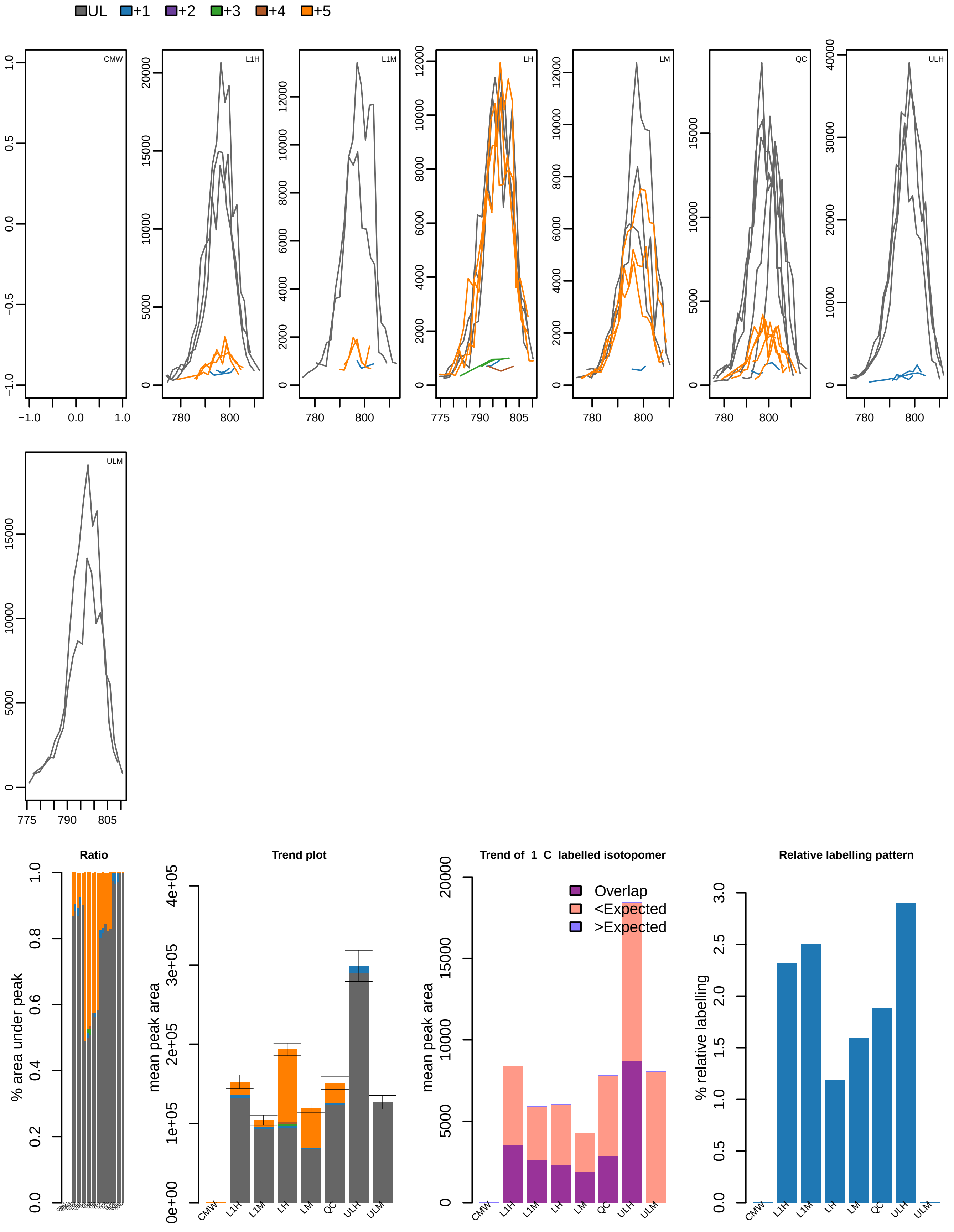

Orotidine(fragment)

G1

Formula: C9H12N2O6 Mass: 244.07 Std.RT: 764.687997 Ion: NEG

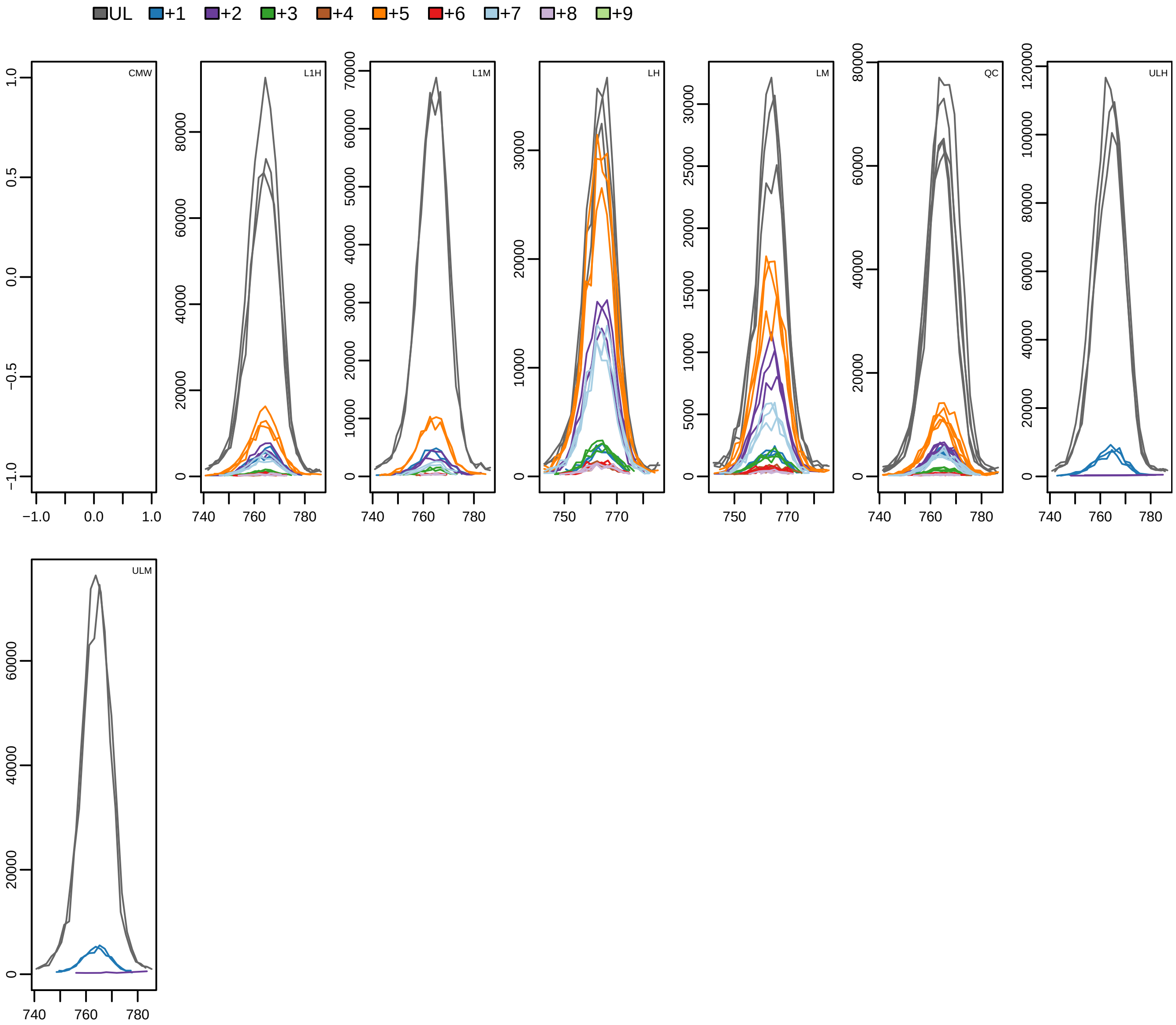

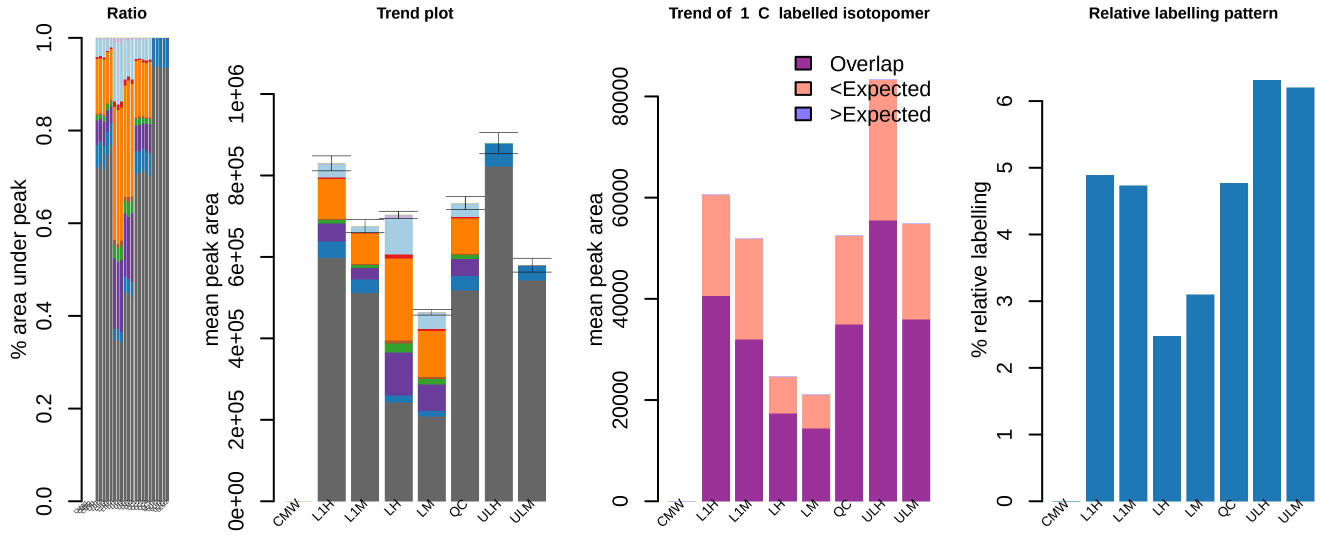

5−6−Dihydrouridine

Formula: C9H14N2O6 Mass: 246.085 Std.RT: 622.7971398 Ion: NEG

G1

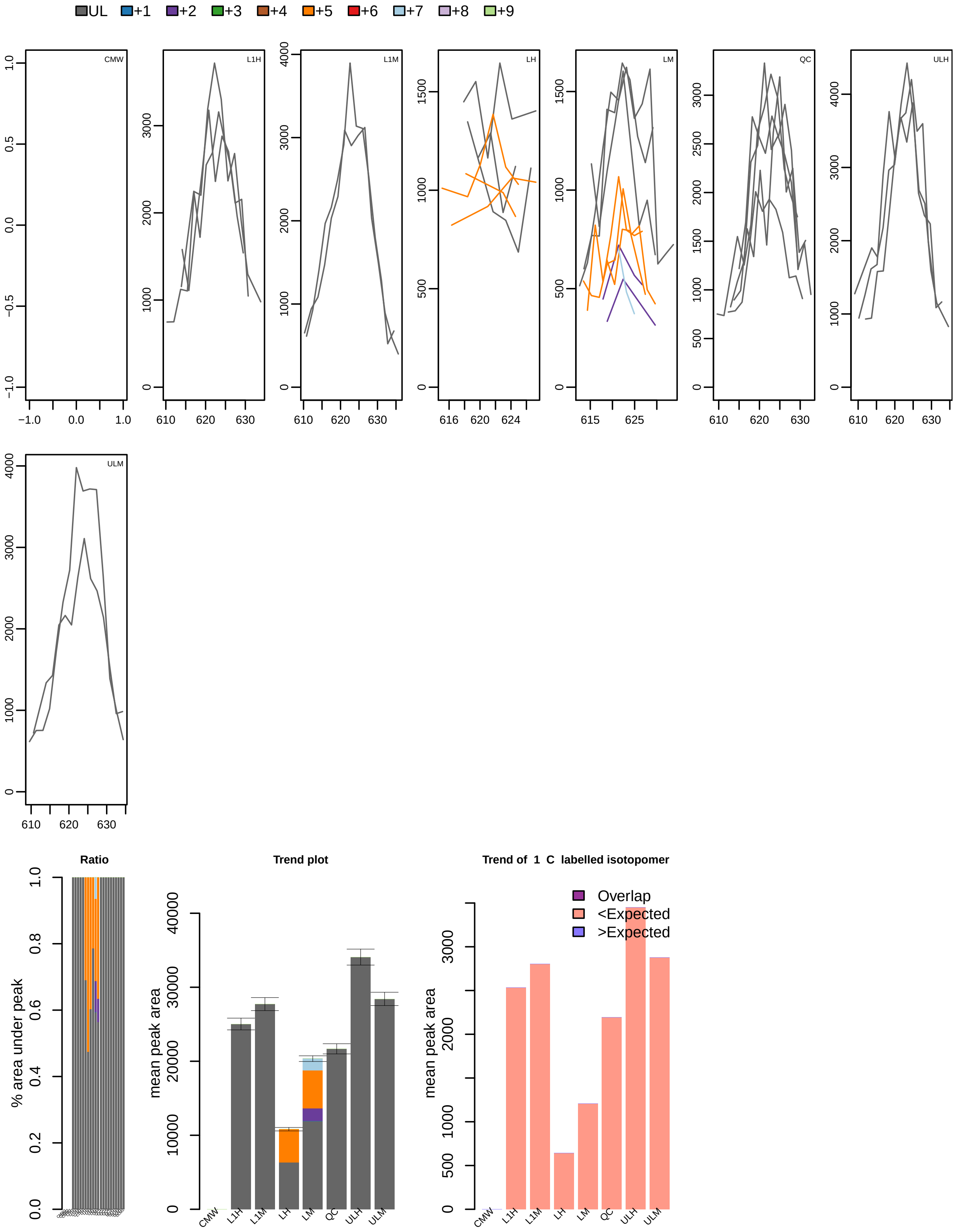

nocardicin G

Formula: $C_{19}H_{19}N_3O_6$ Mass: 385.127 Std.RT: 904.9671426 Ion: NE

G1

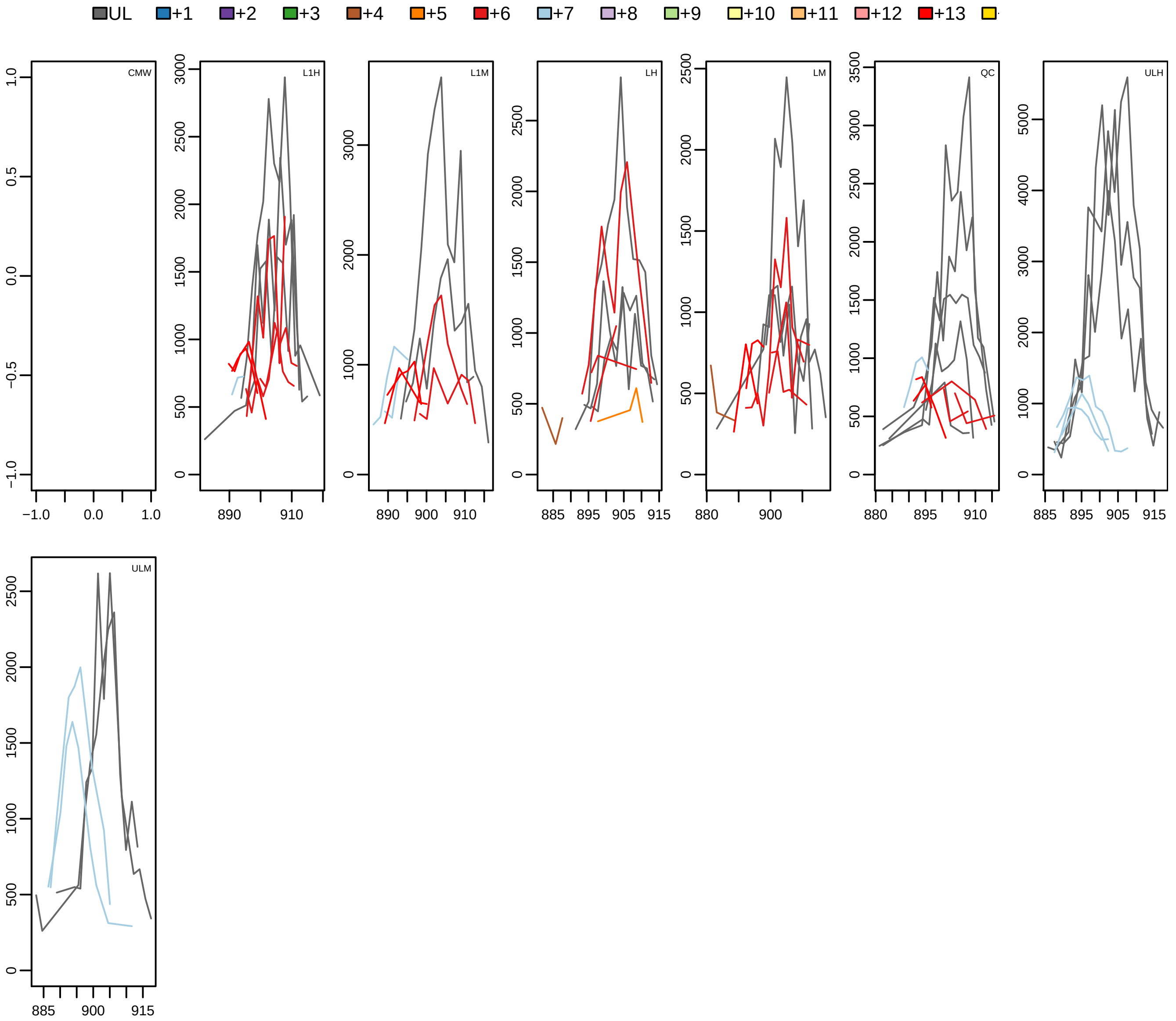

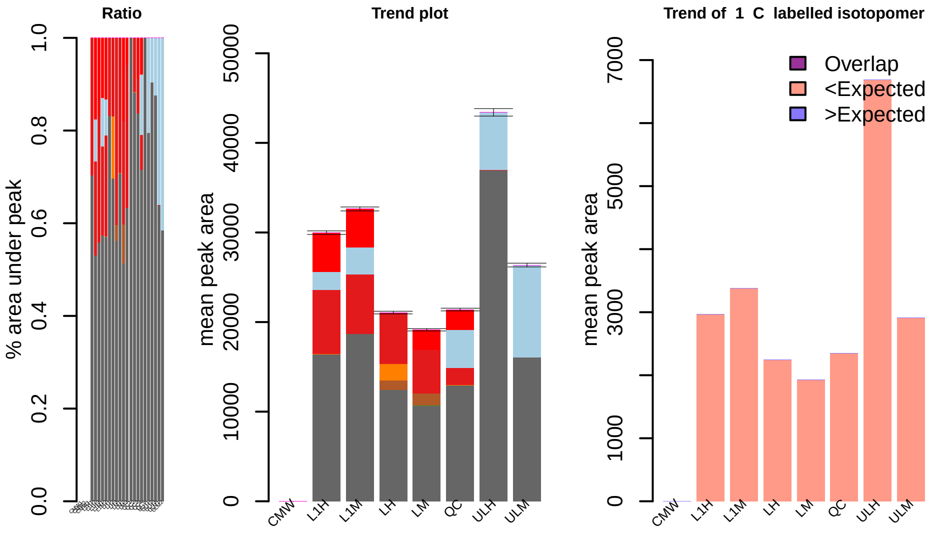

1−18:0−2−18:3−phosphatidate

Formula: C39H71O8P Mass: 698.489 Std.RT: 208.26554952 Ion: NE

G1

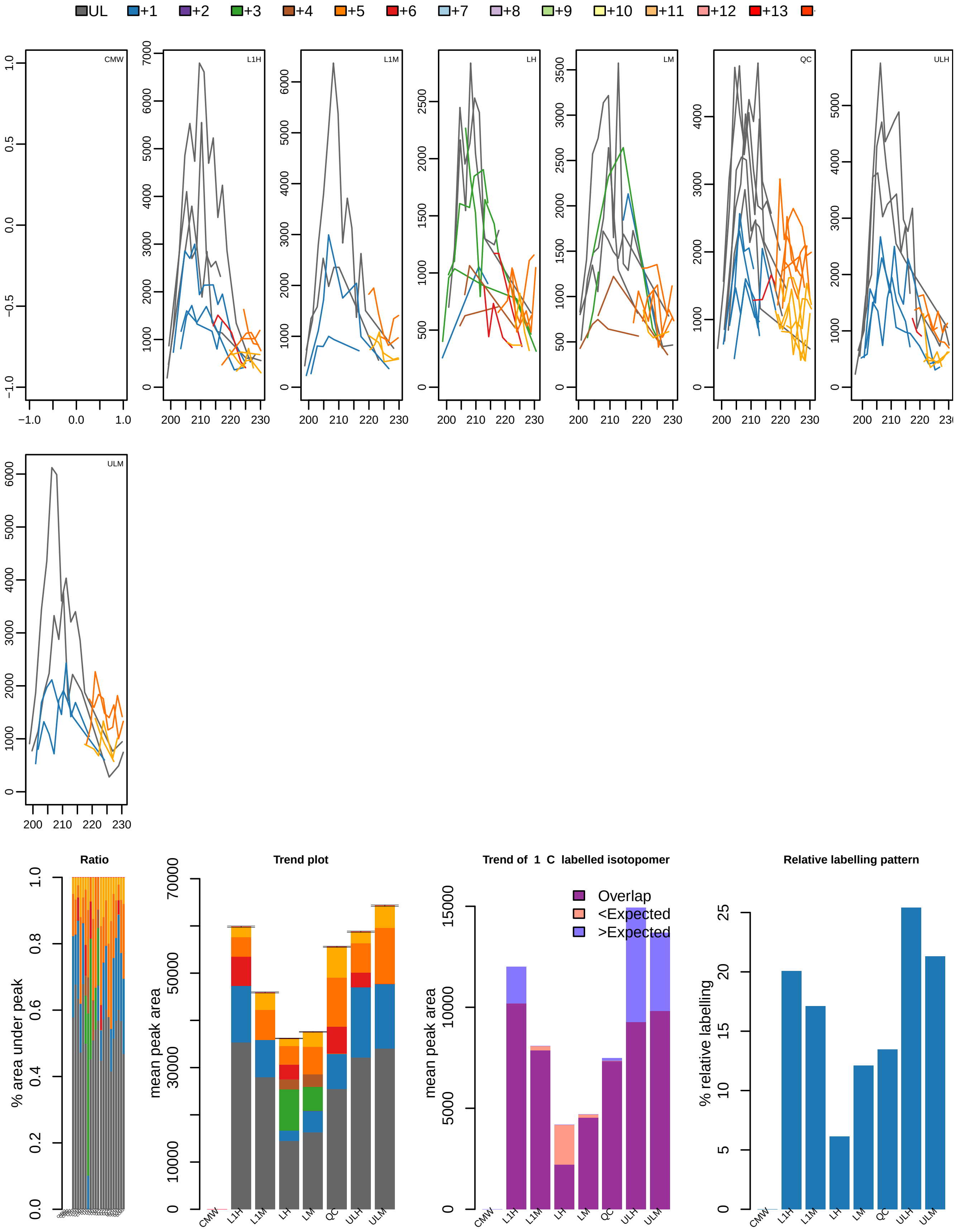

Muramic acid

Formula: C9H17NO7 Mass: 251.101 Std.RT: 795.5601372 Ion: NEG

G1

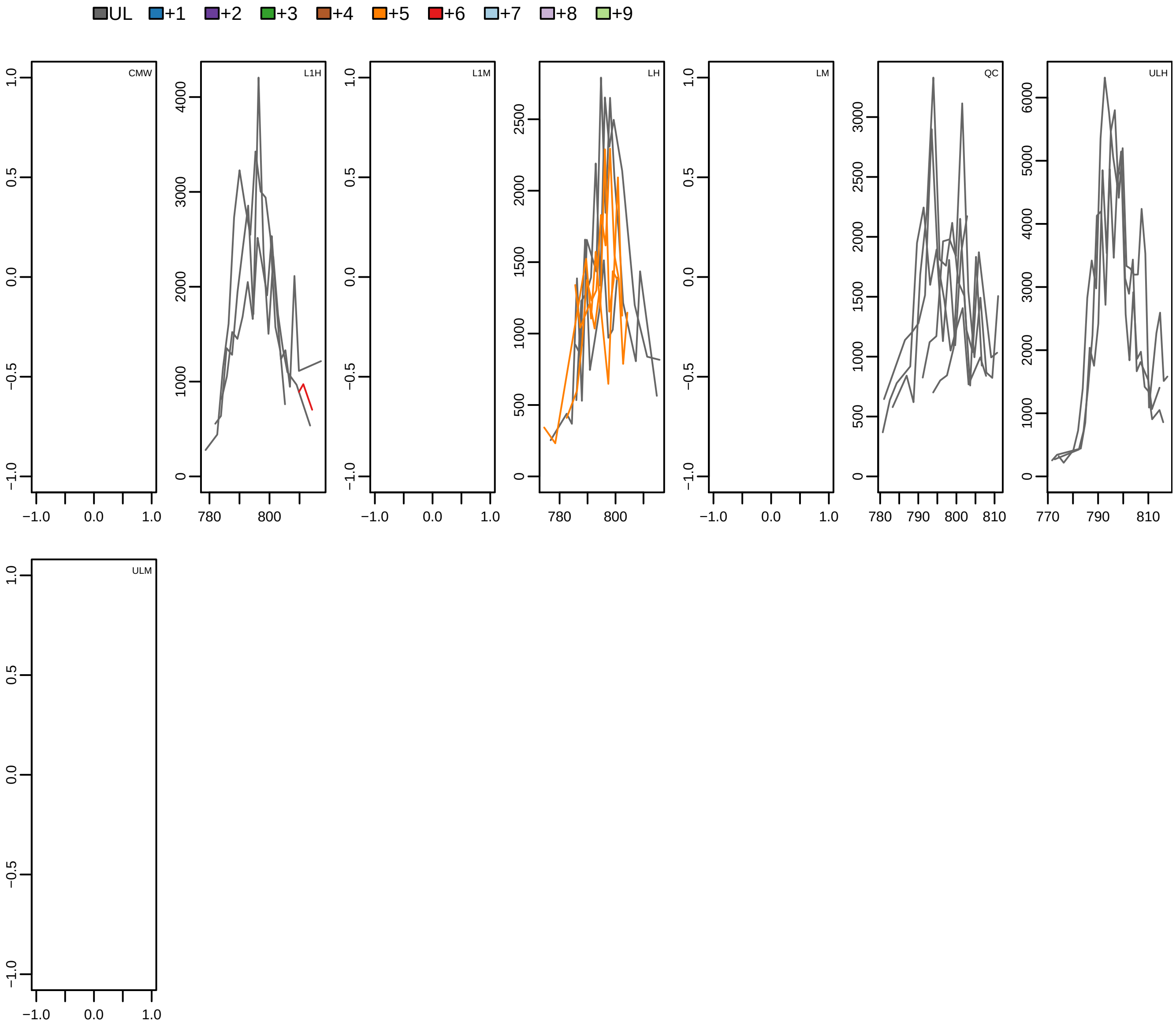

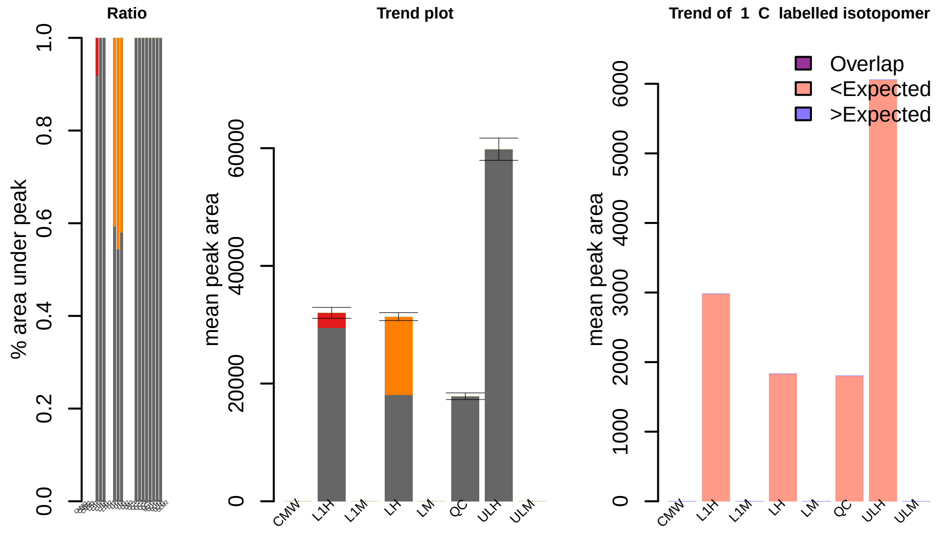

L−alpha−glutamyl−L−hydroxyproline

Formula: C10H16N2O6 Mass: 260.101 Std.RT: 819.7797156 Ion: NE

G1

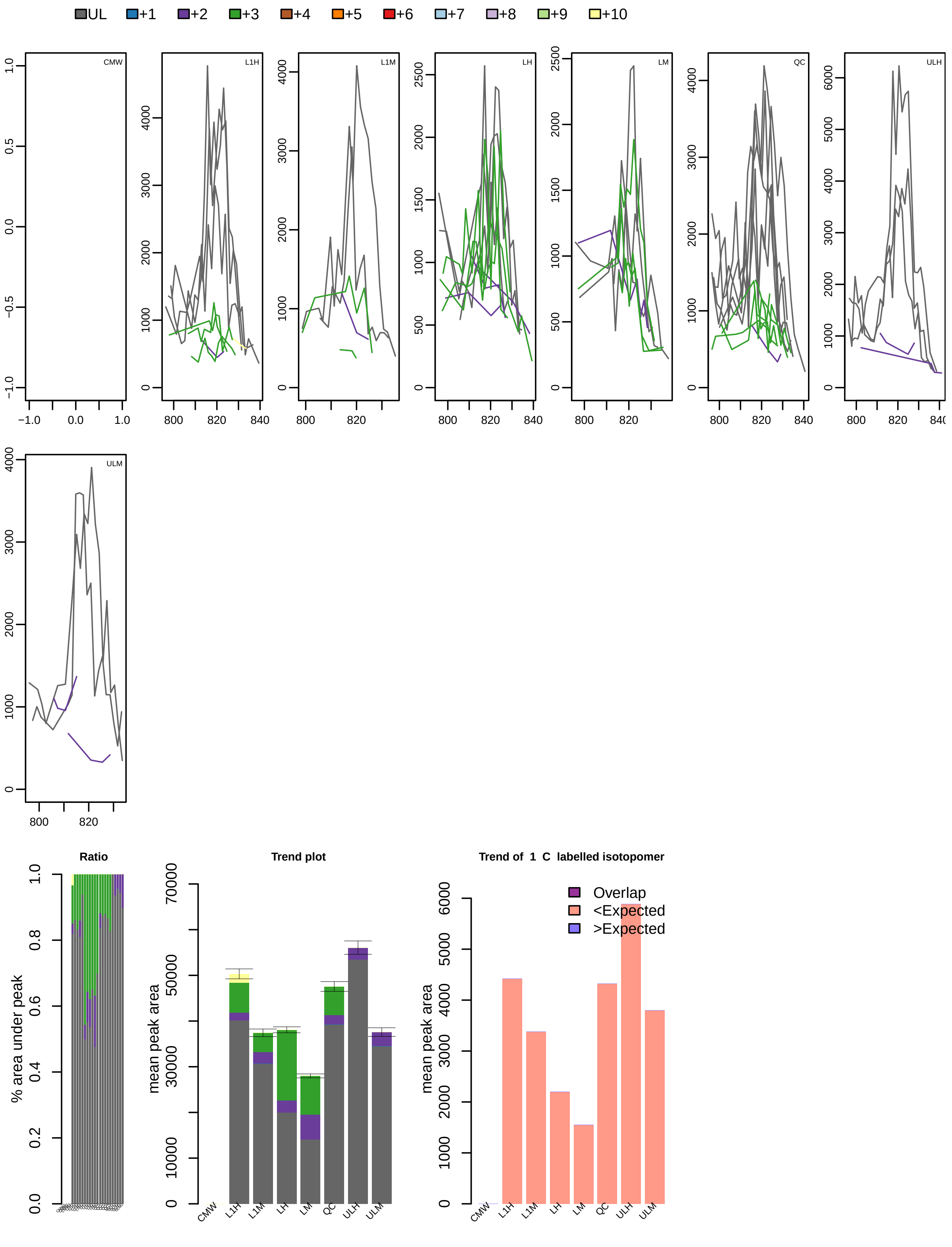

N−Glycolyl−D−mannosamine 6−phosphate

G1

Formula: C8H16NO10P Mass: 317.051 Std.RT: 960.726309 Ion: NE(

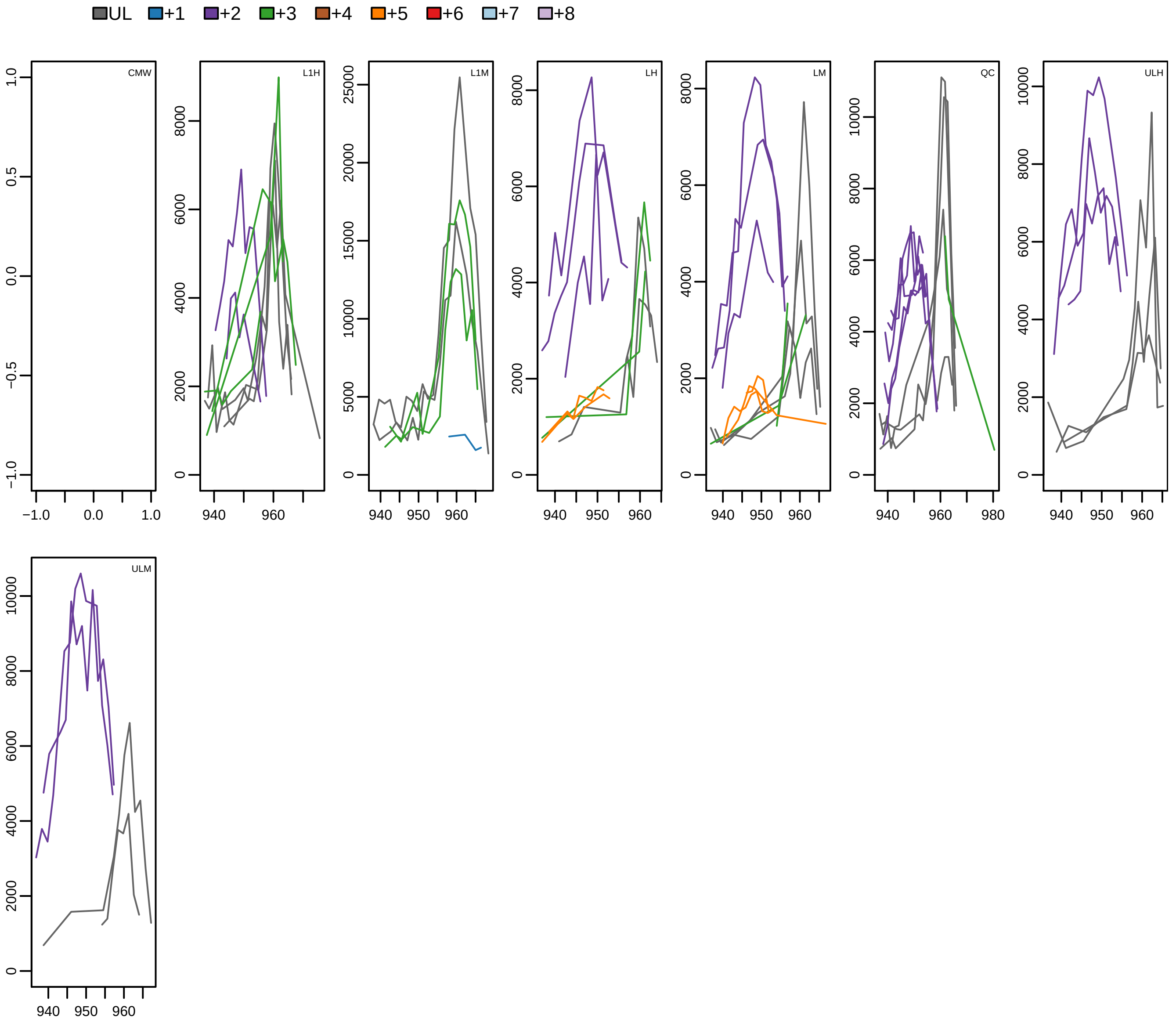

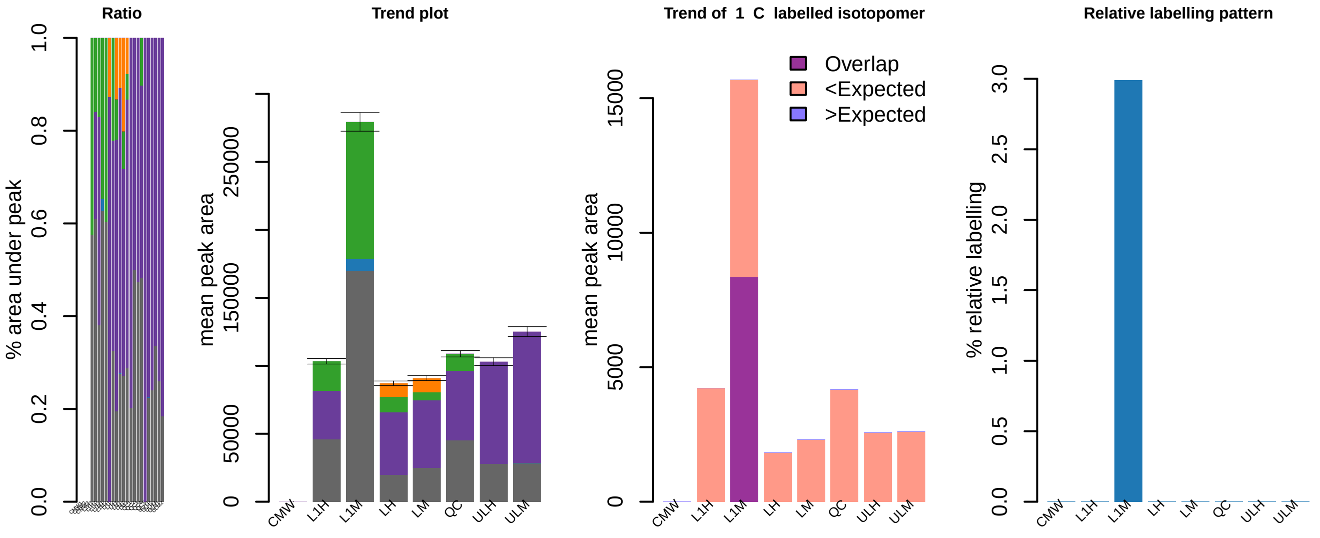

N3−fumaramoyl−L−2,3−diaminopropanoate

Formula: C7H10N2O5 Mass: 202.059 Std.RT: 921.7731498 Ion: NEG

G1

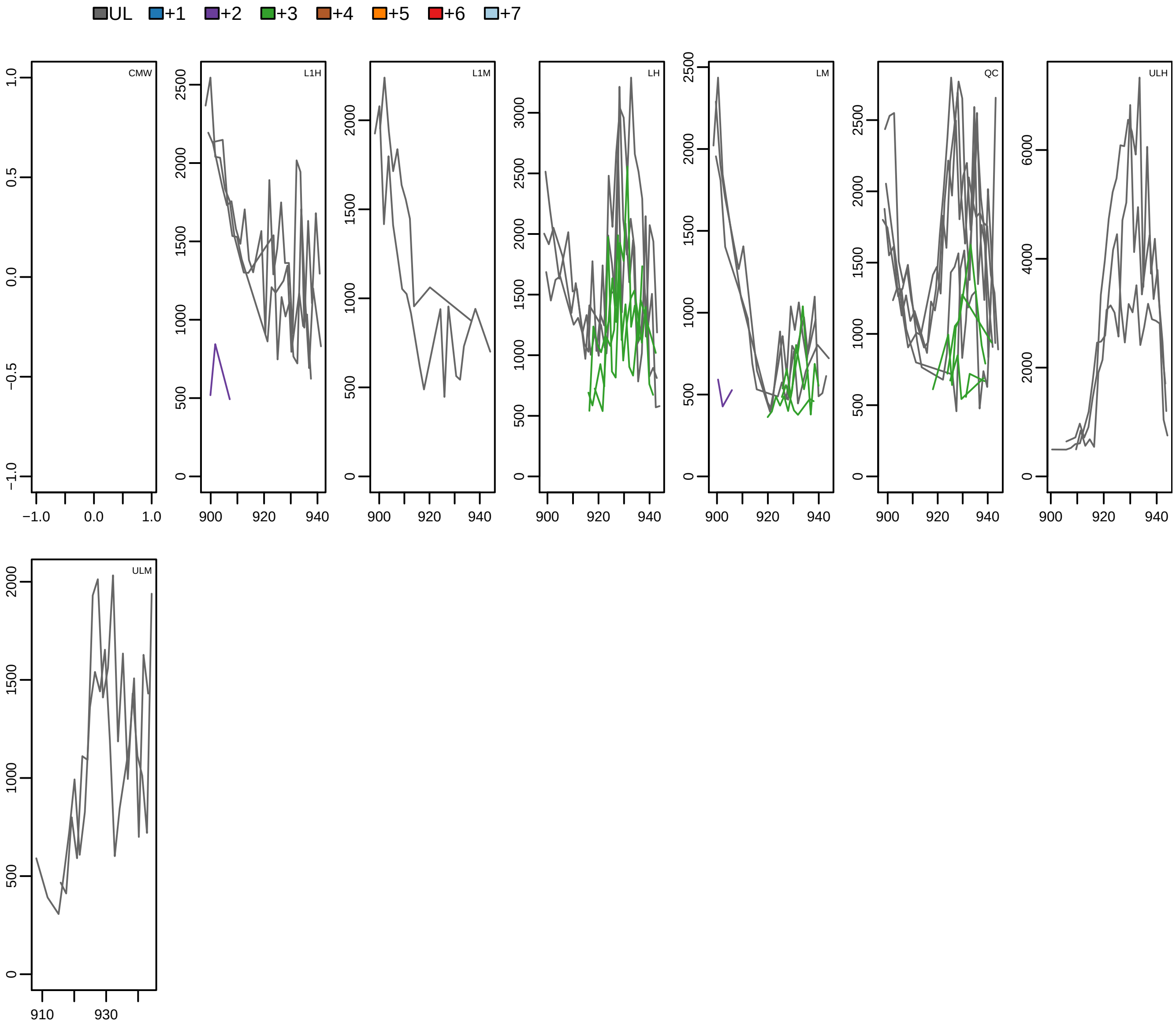

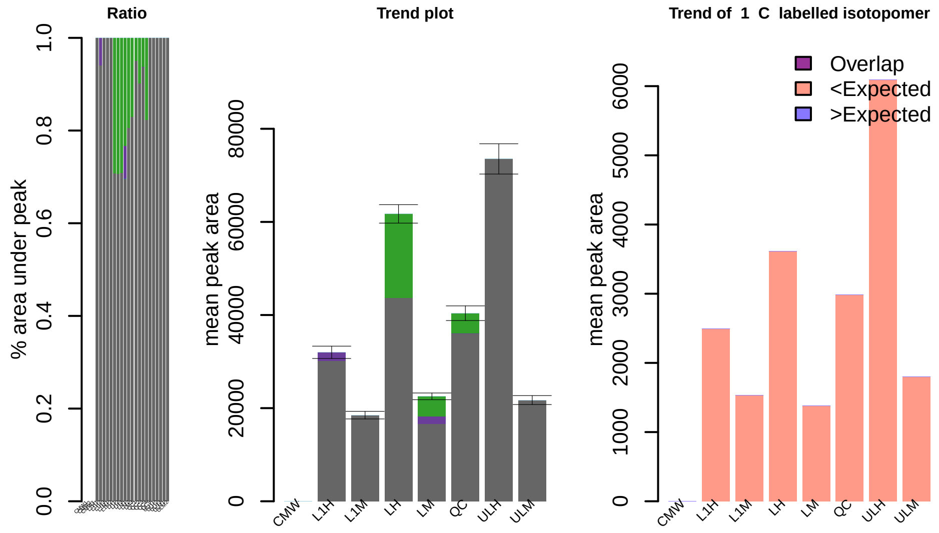

2,7−Anhydro−alpha−N−acetylneuraminic acid

Formula: C11H17NO8 Mass: 291.095 Std.RT: 798.8148516 Ion: NEC

G1

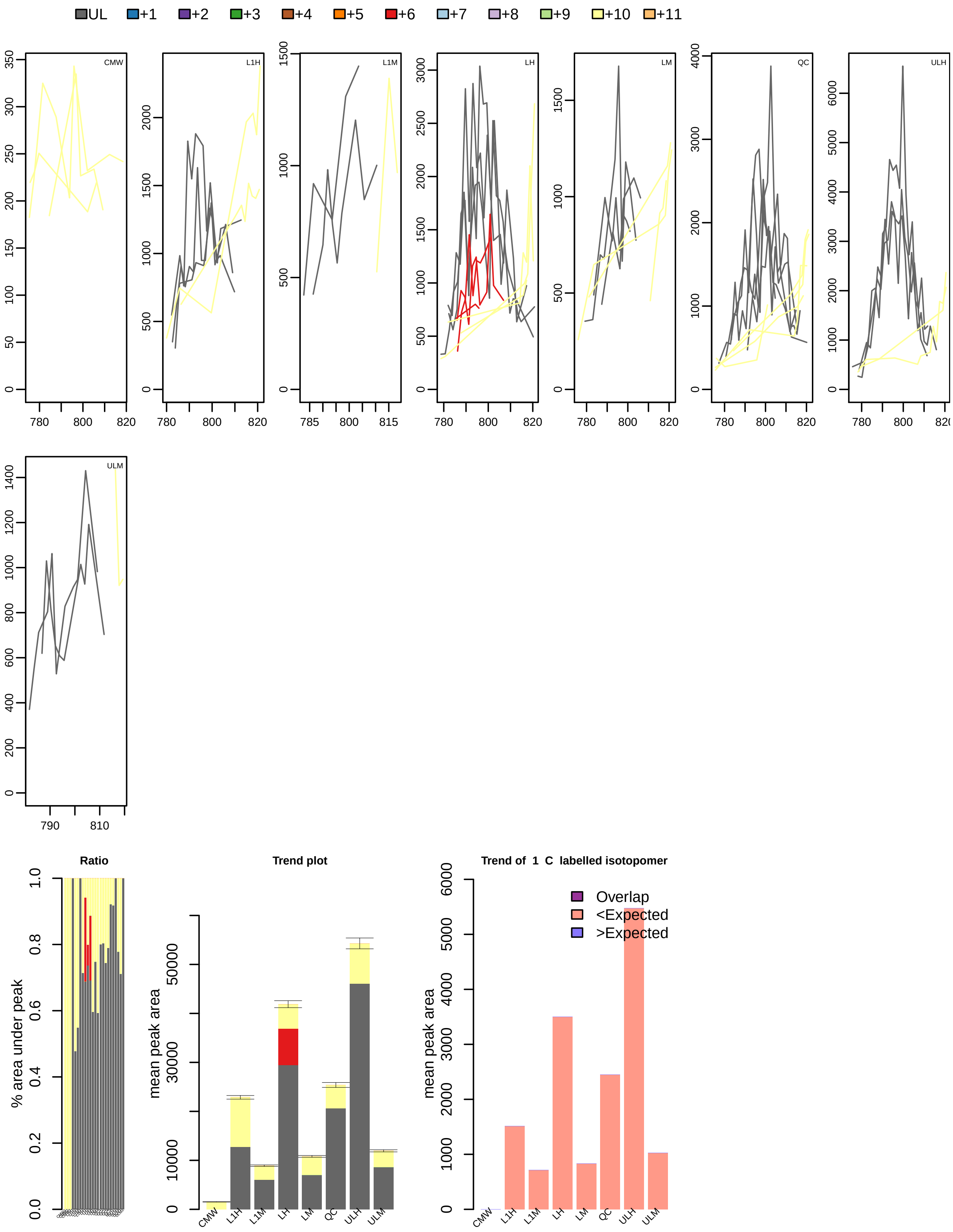

sn−glycero−3−Phospho−1−inositol

Formula: $C_9H_{19}O_{11}P$ Mass: 334.066 Std.RT: 1045.093314 Ion: NEC

G1

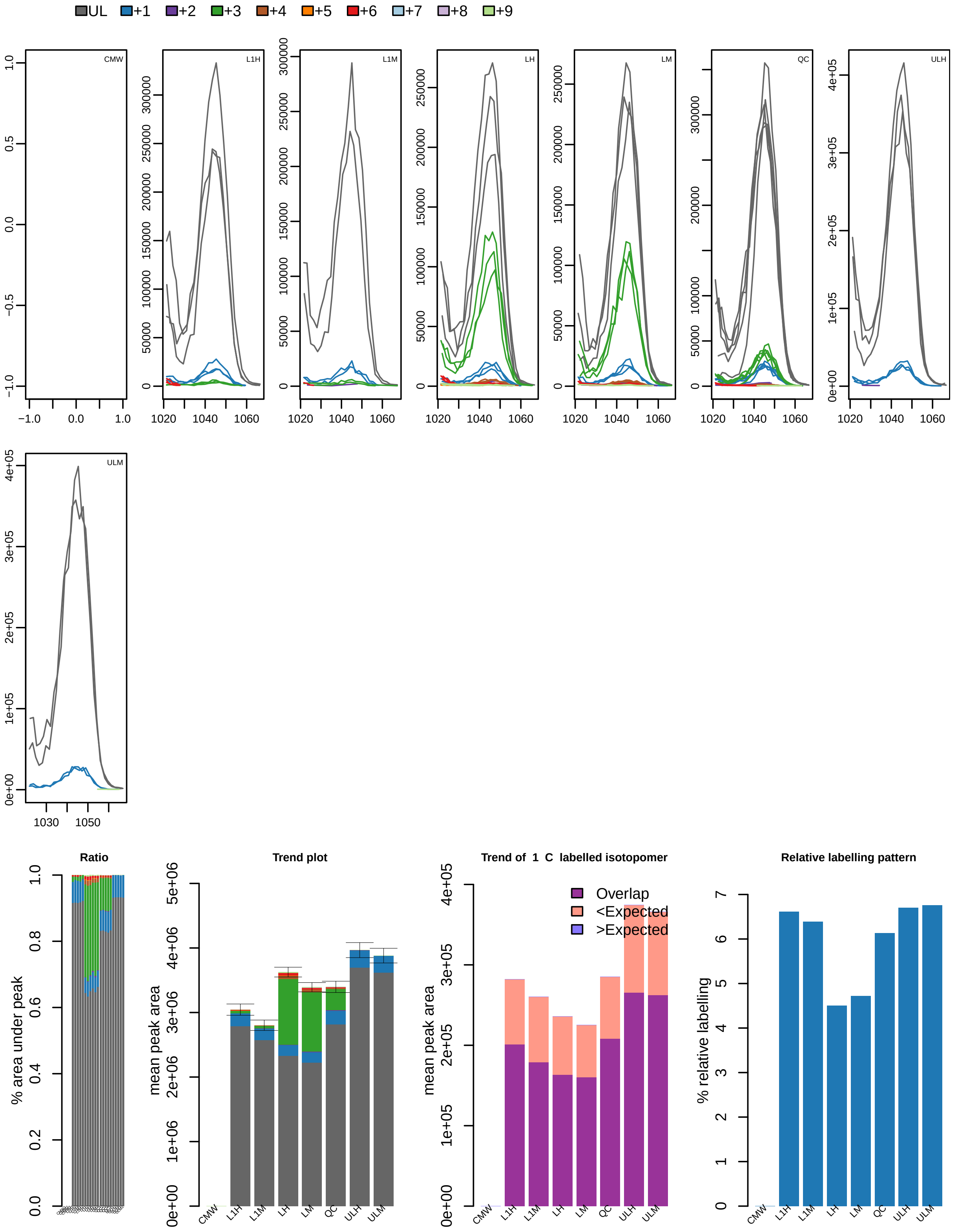

sn−glycero−3−Phospho−1−inositol

Formula: C9H19O11P Mass: 334.066 Std.RT: 1045.093314 Ion: NEC

G2

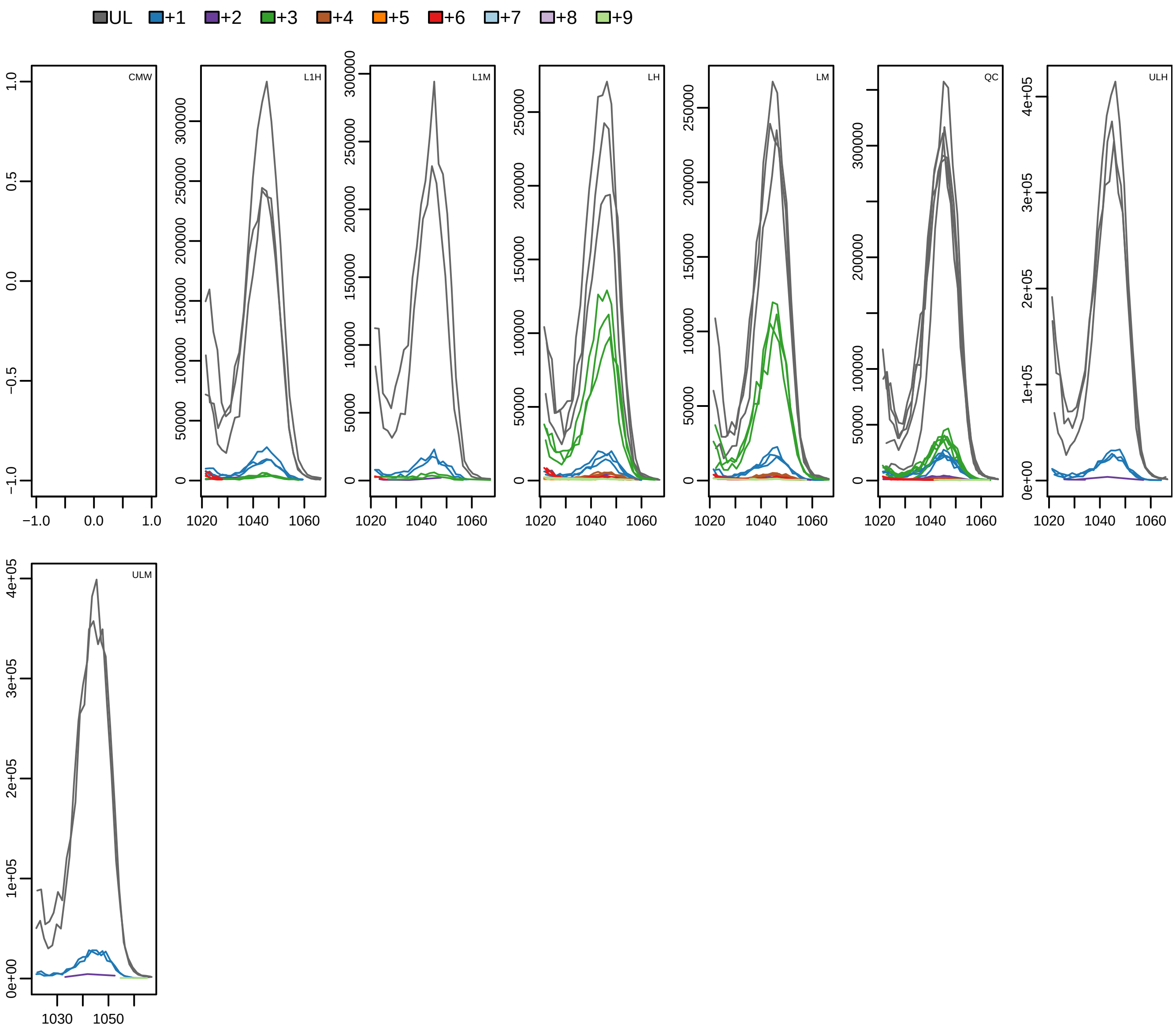

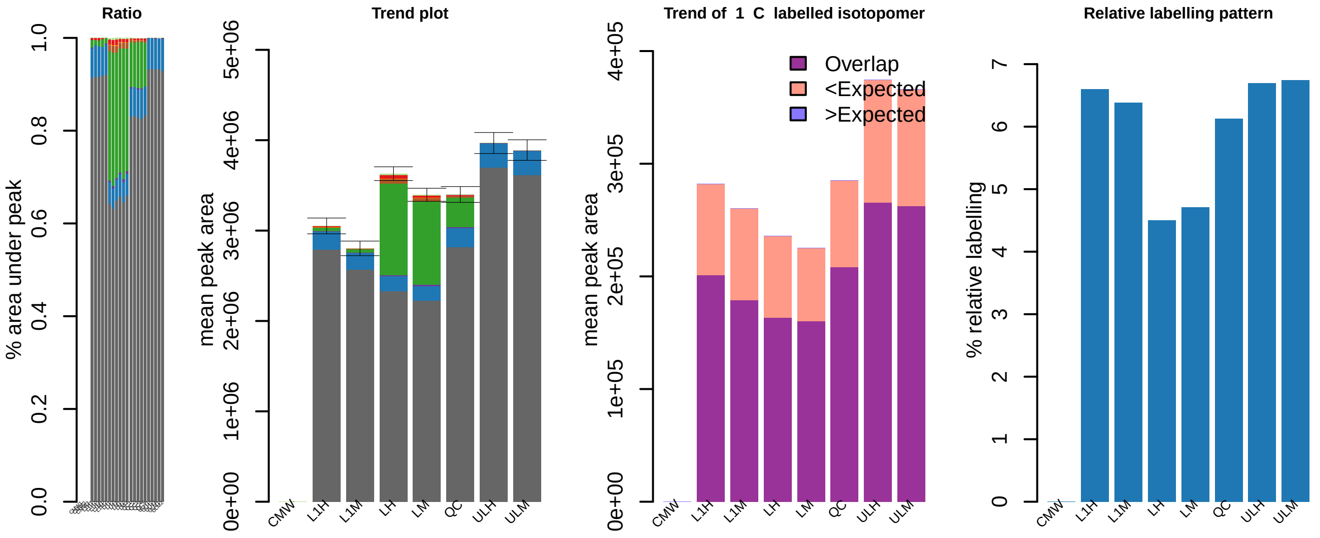

L−thiazolidine−4−carboxylate

Formula: C4H7NO2S Mass: 133.02 Std.RT: 455.85323808 Ion: NEG

G1

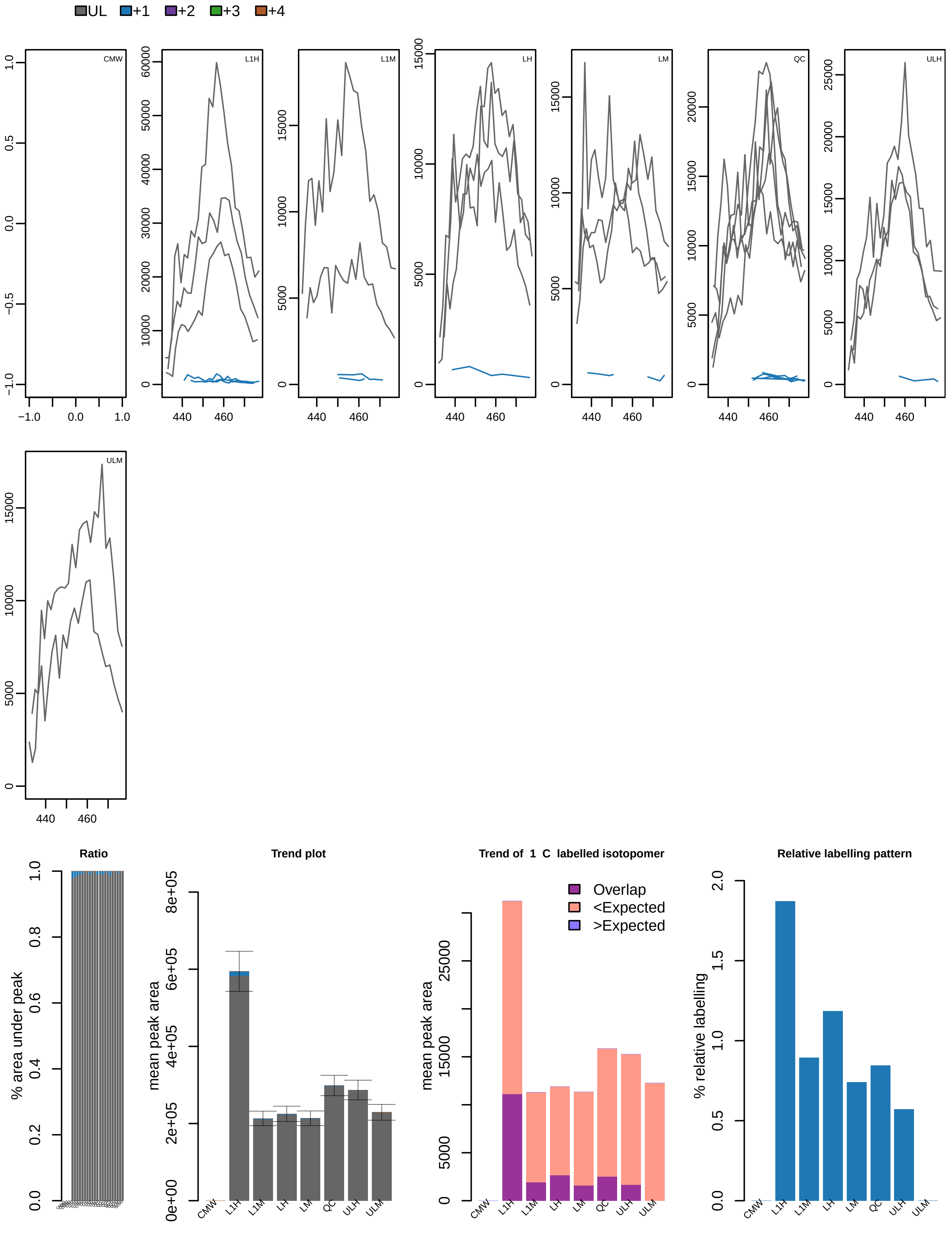

2−Dehydro−D−xylonate

Formula: C5H8O6 Mass: 164.032 Std.RT: 812.4240006 Ion: NEG

G1

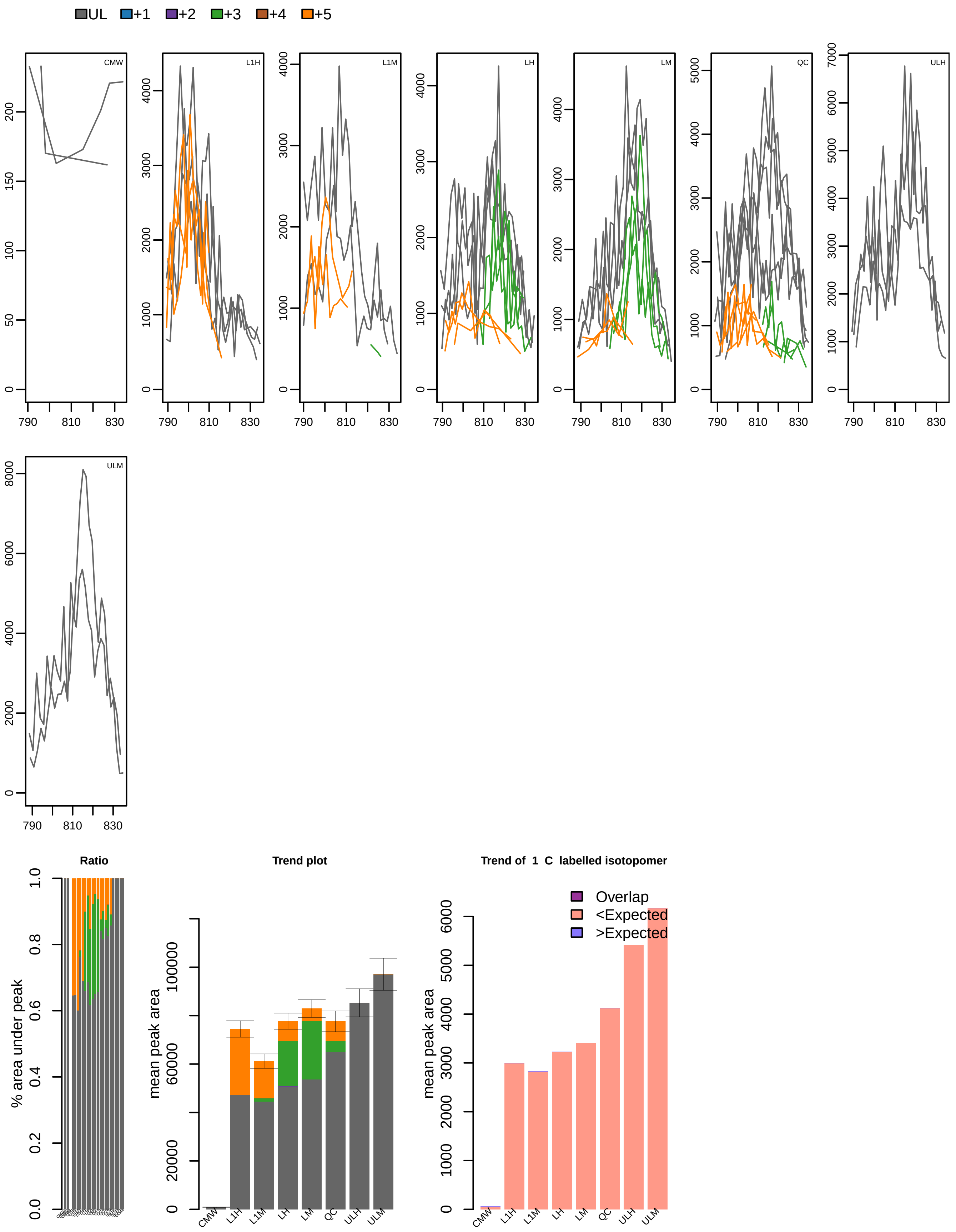

sodium dodecyl sulfate

Formula: C12H26O4S Mass: 266.155 Std.RT: 208.0859559 Ion: NEC

G1

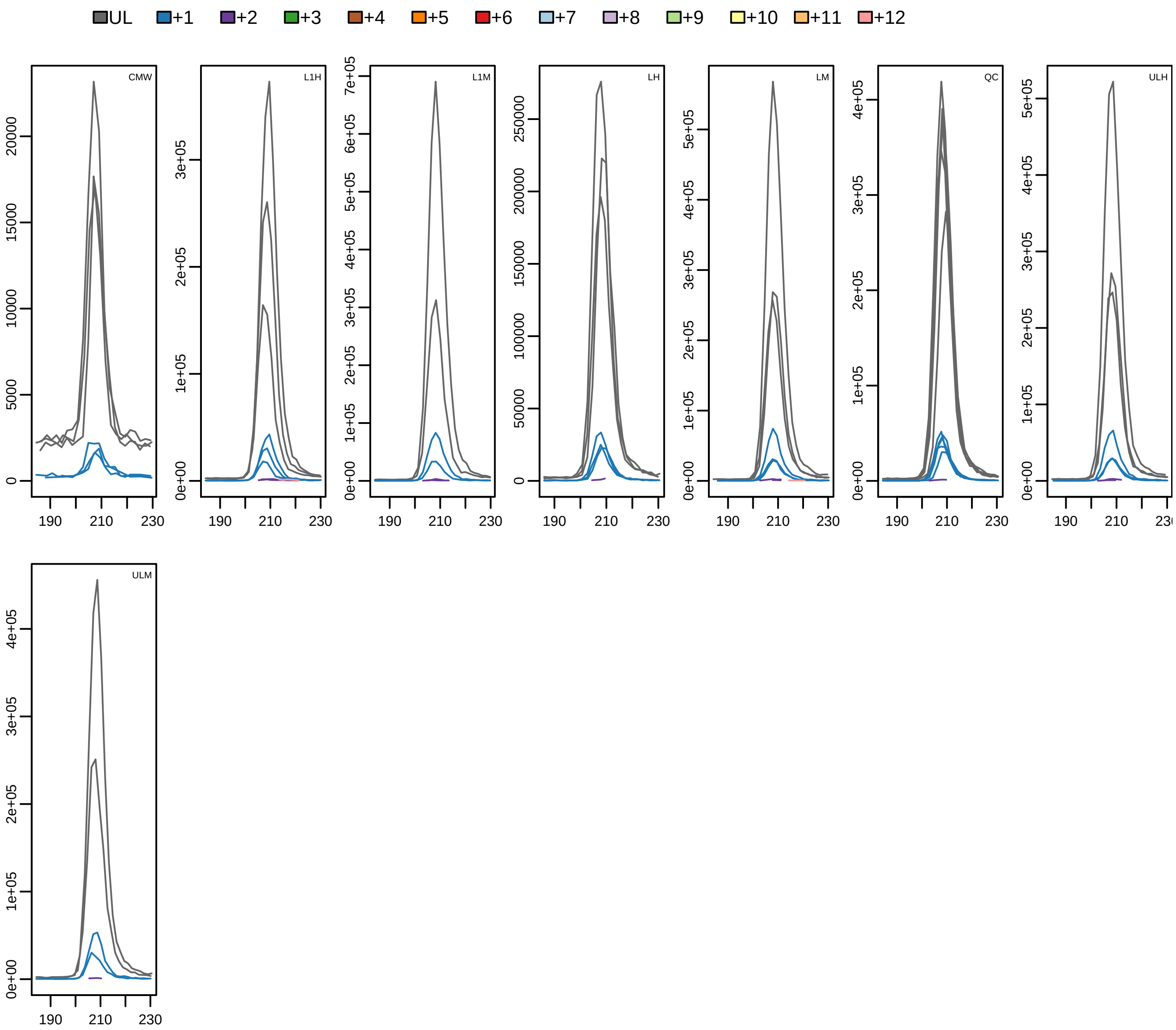

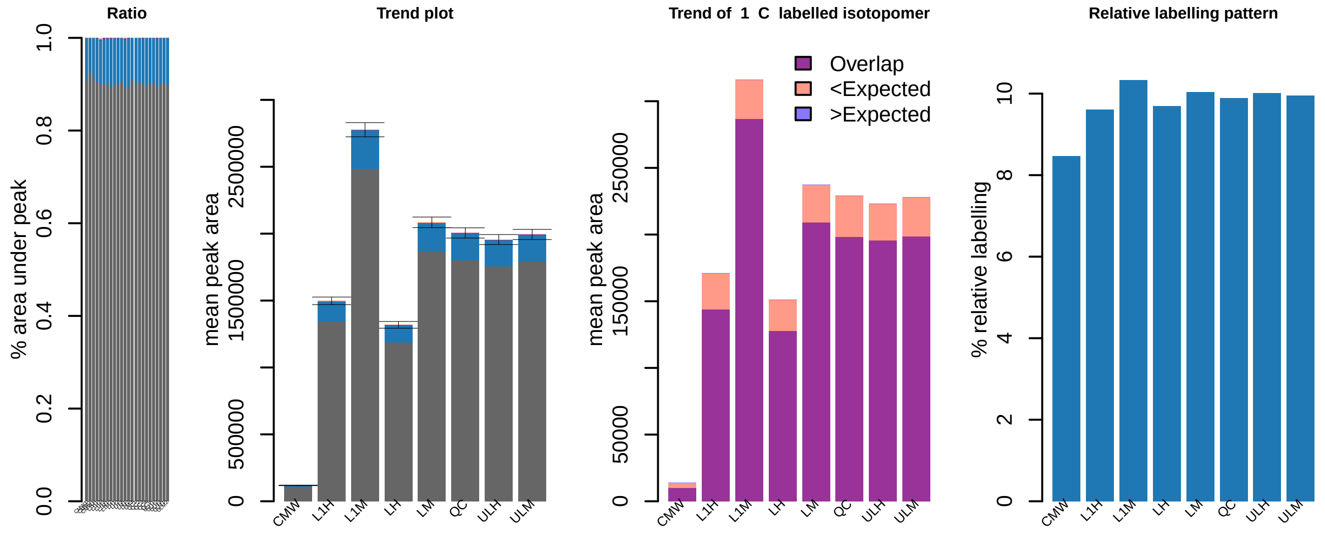

L−Glutamate methylester

Formula: C6H11NO4 Mass: 161.069 Std.RT: 564.2868729 Ion: NEG

G1

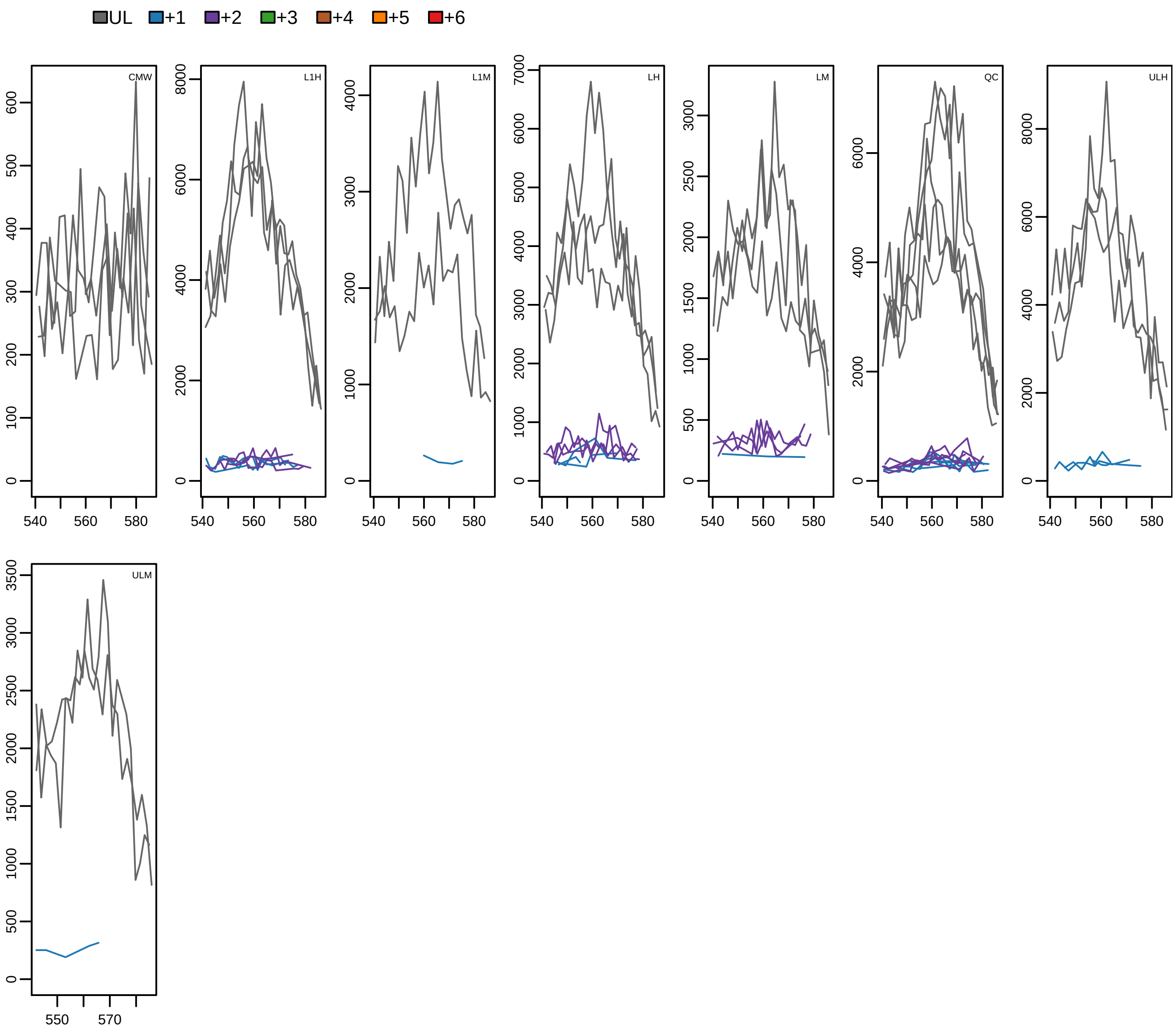

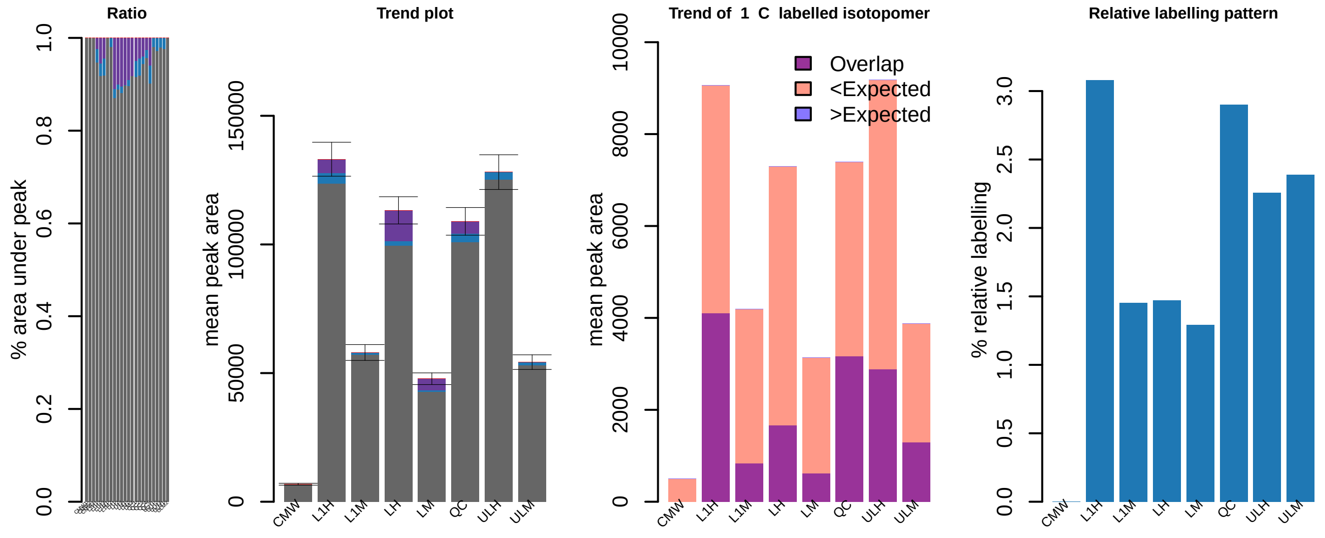

3−sulfopropanoate

Formula: C3H6O5S Mass: 153.994 Std.RT: 1048.5345972 Ion: NEG

G1

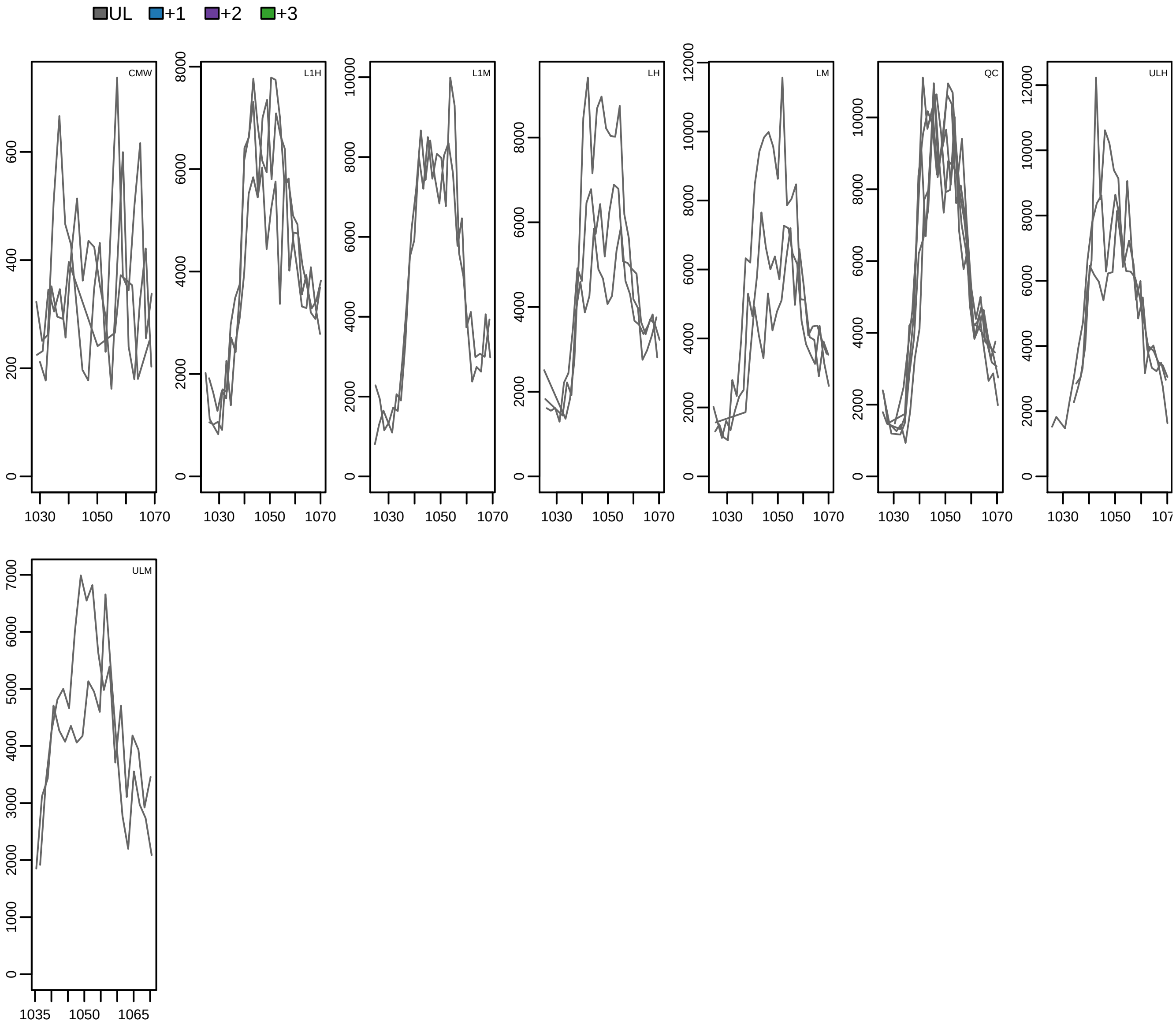

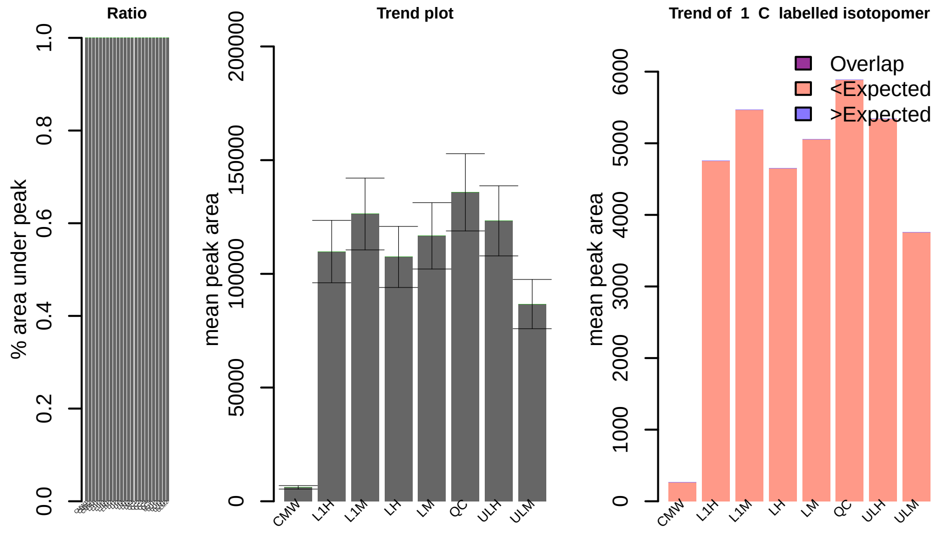

Hydroxyacetone phosphate

G1

Formula: C3H7O5P Mass: 154.003 Std.RT: 709.812573 Ion: NEG

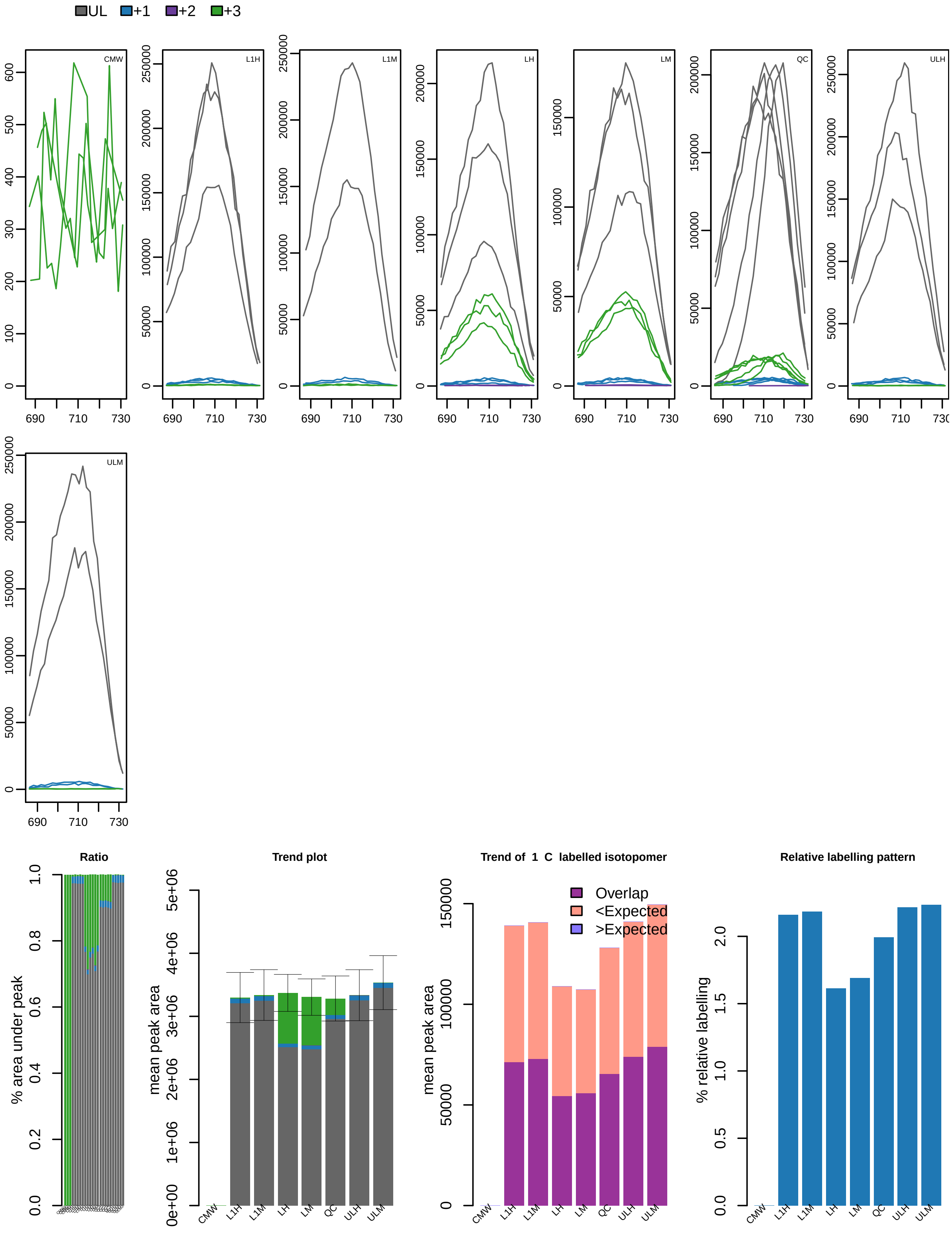

1−deoxynojirimycin

Formula: C6H13NO4 Mass: 163.084 Std.RT: 721.7427468 Ion: NEG

G1

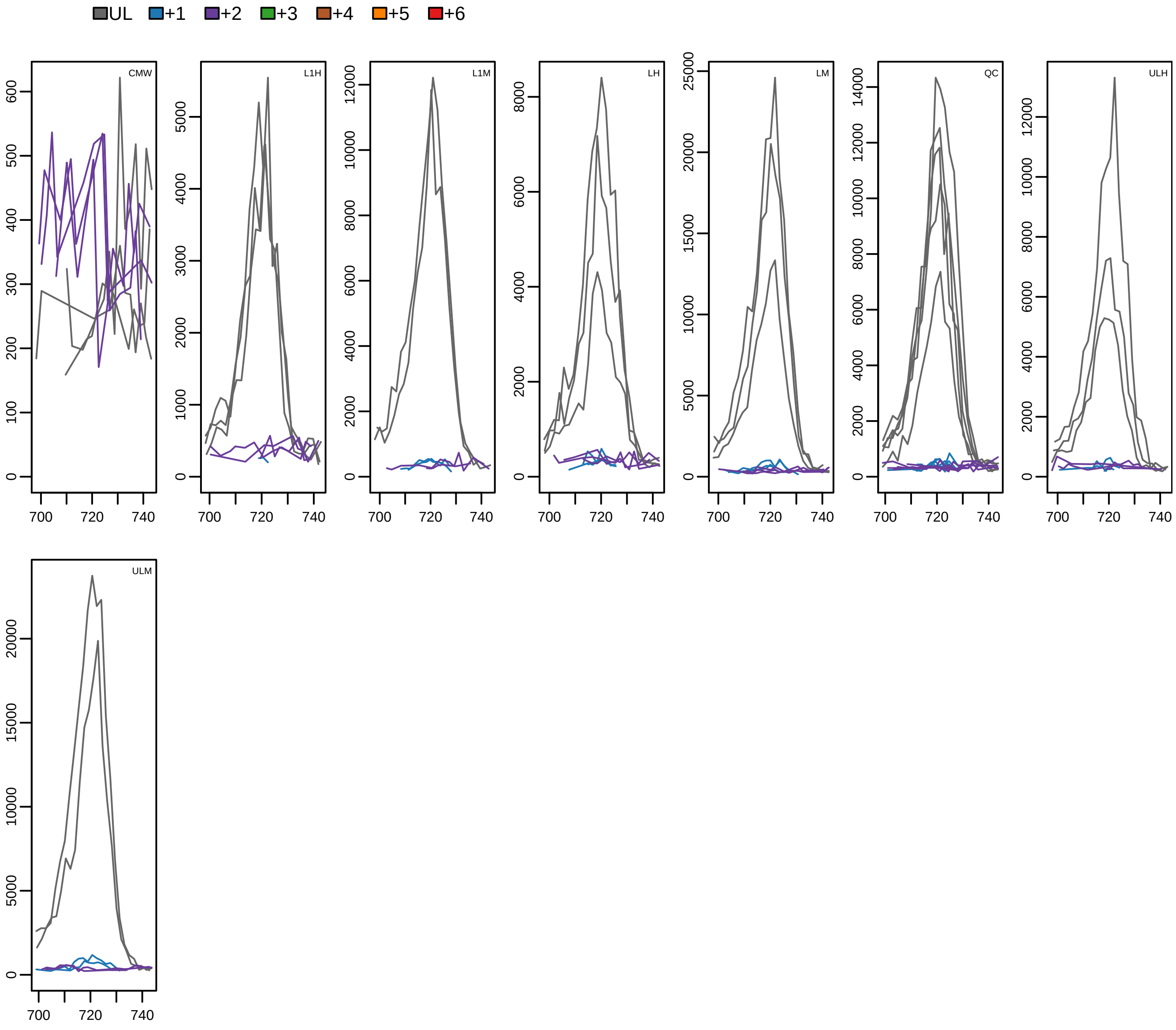

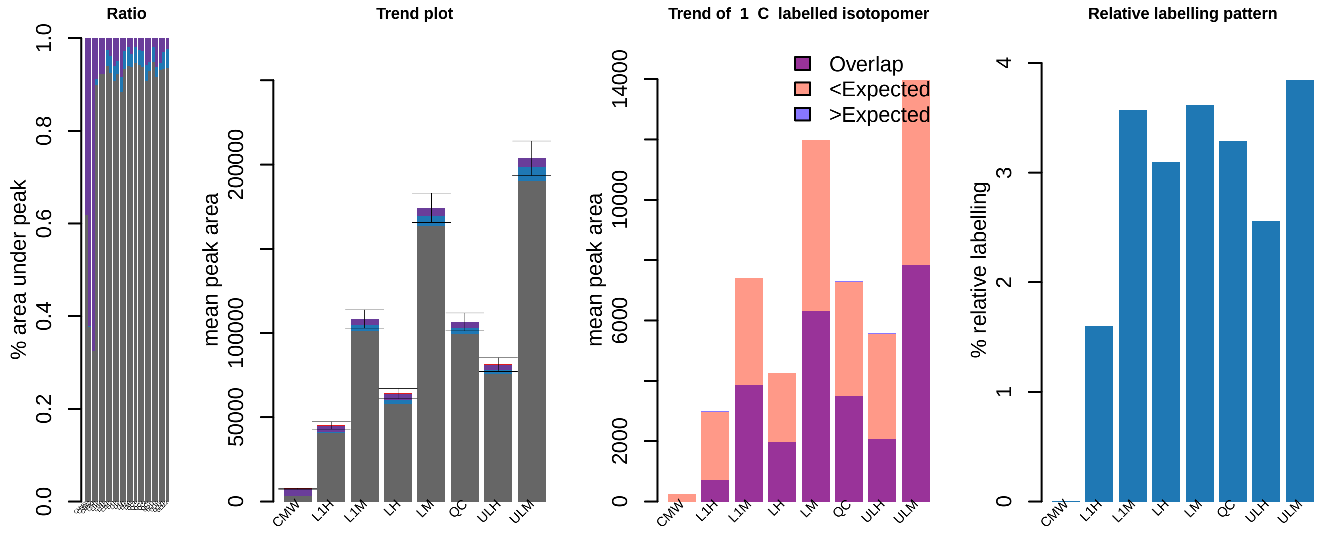

Vinylacetylglycine

Formula: C6H9NO3 Mass: 143.058 Std.RT: 956.6002476 Ion: NEG

G1

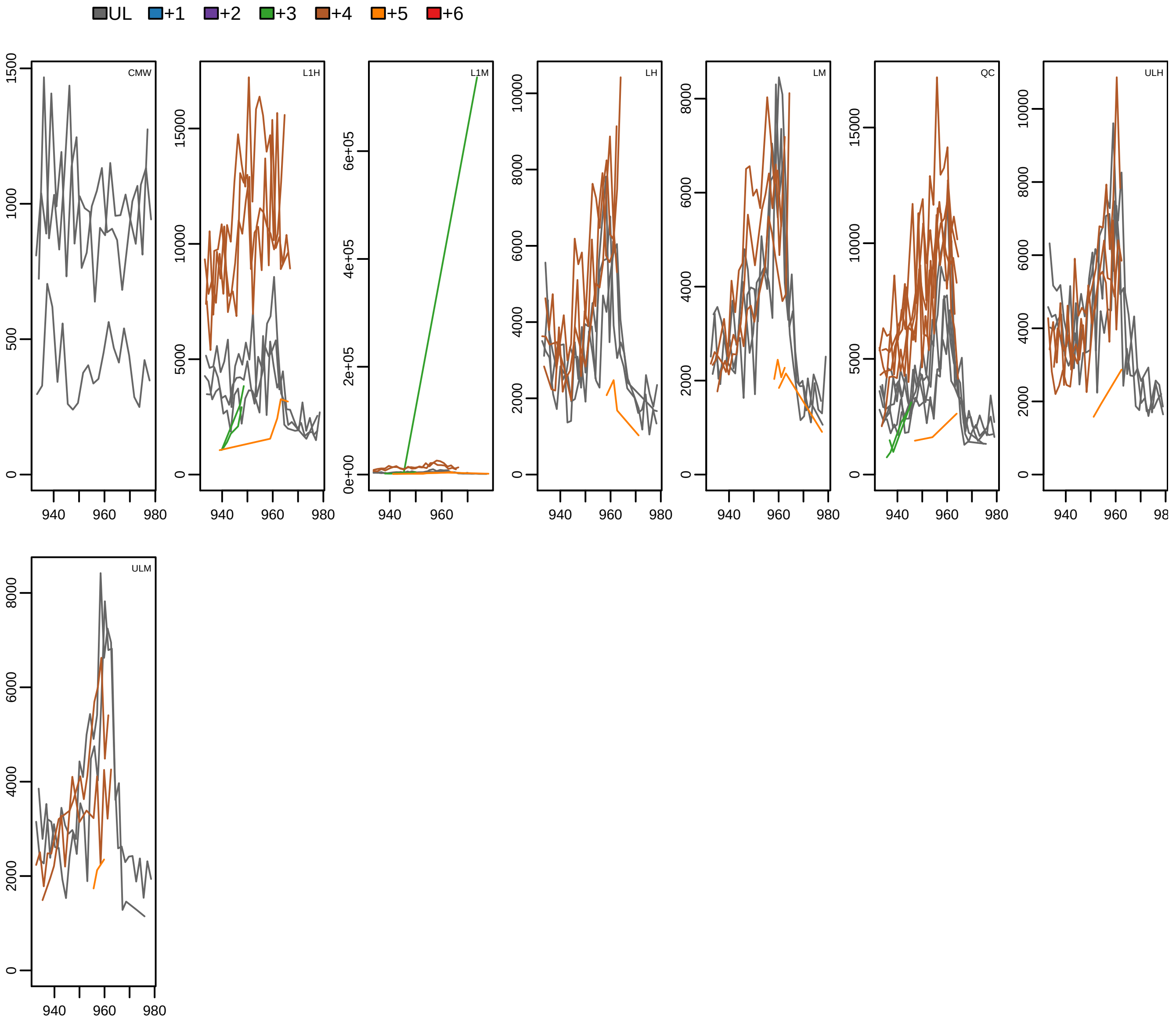

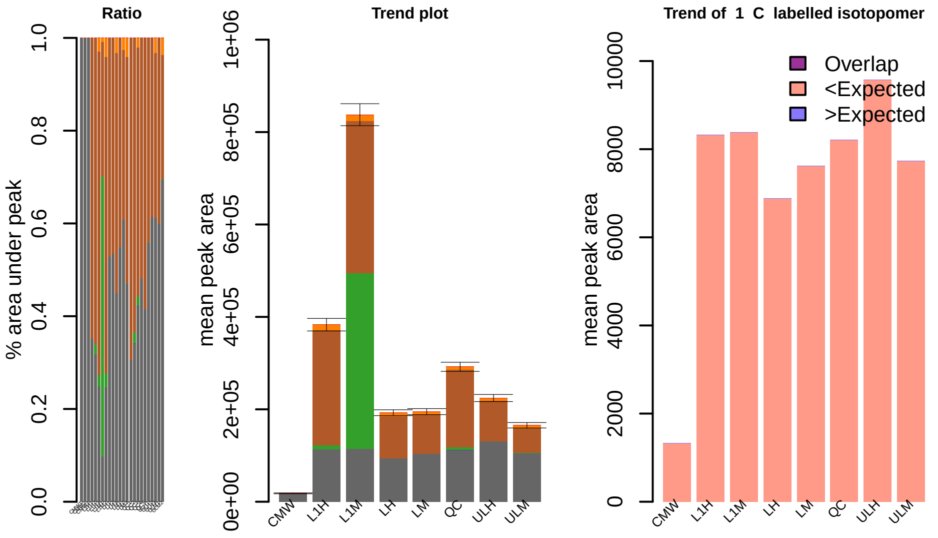

N3−(4−methoxyfumaroyl)−L−2,3−diaminopropanoate

G1

Formula: C8H12N2O5 Mass: 216.075 Std.RT: 800.5400016 Ion: NEG

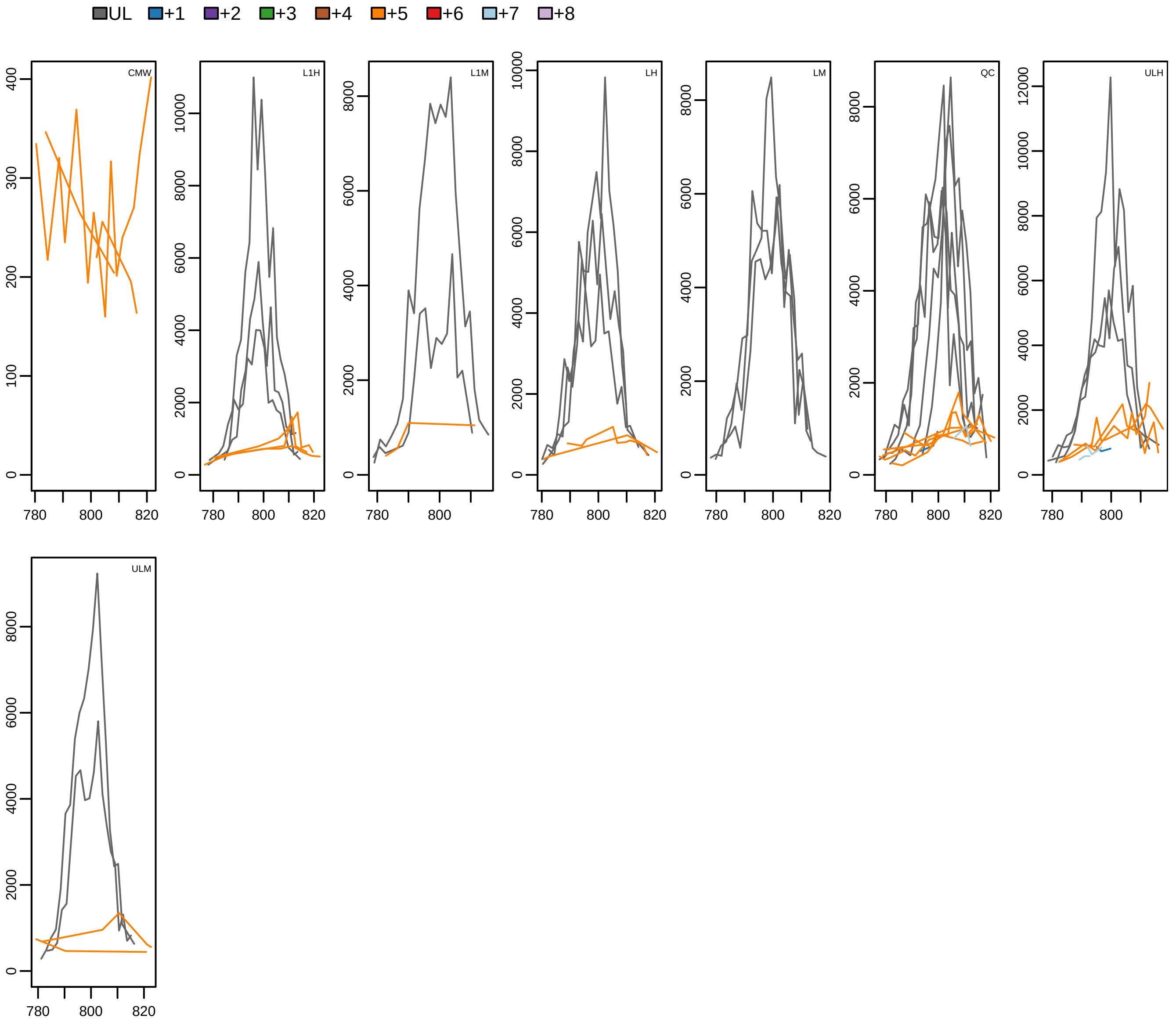

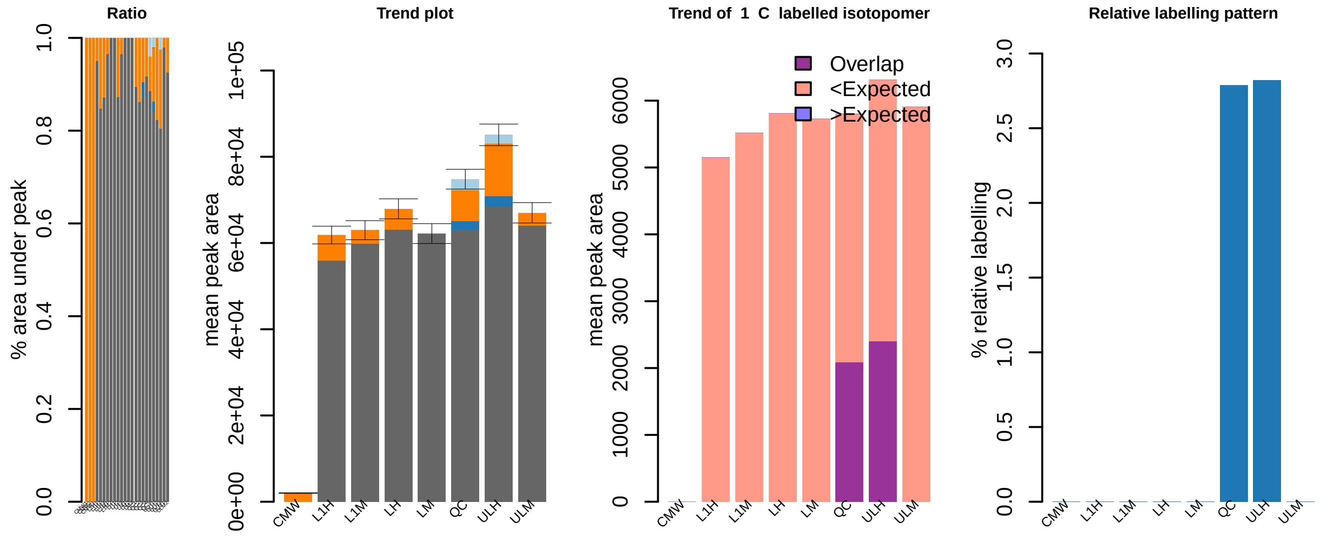

4,4'−Sulfonyldiphenol

Formula: C12H10O4S Mass: 250.03 Std.RT: 314.06238078 Ion: NEC

G1

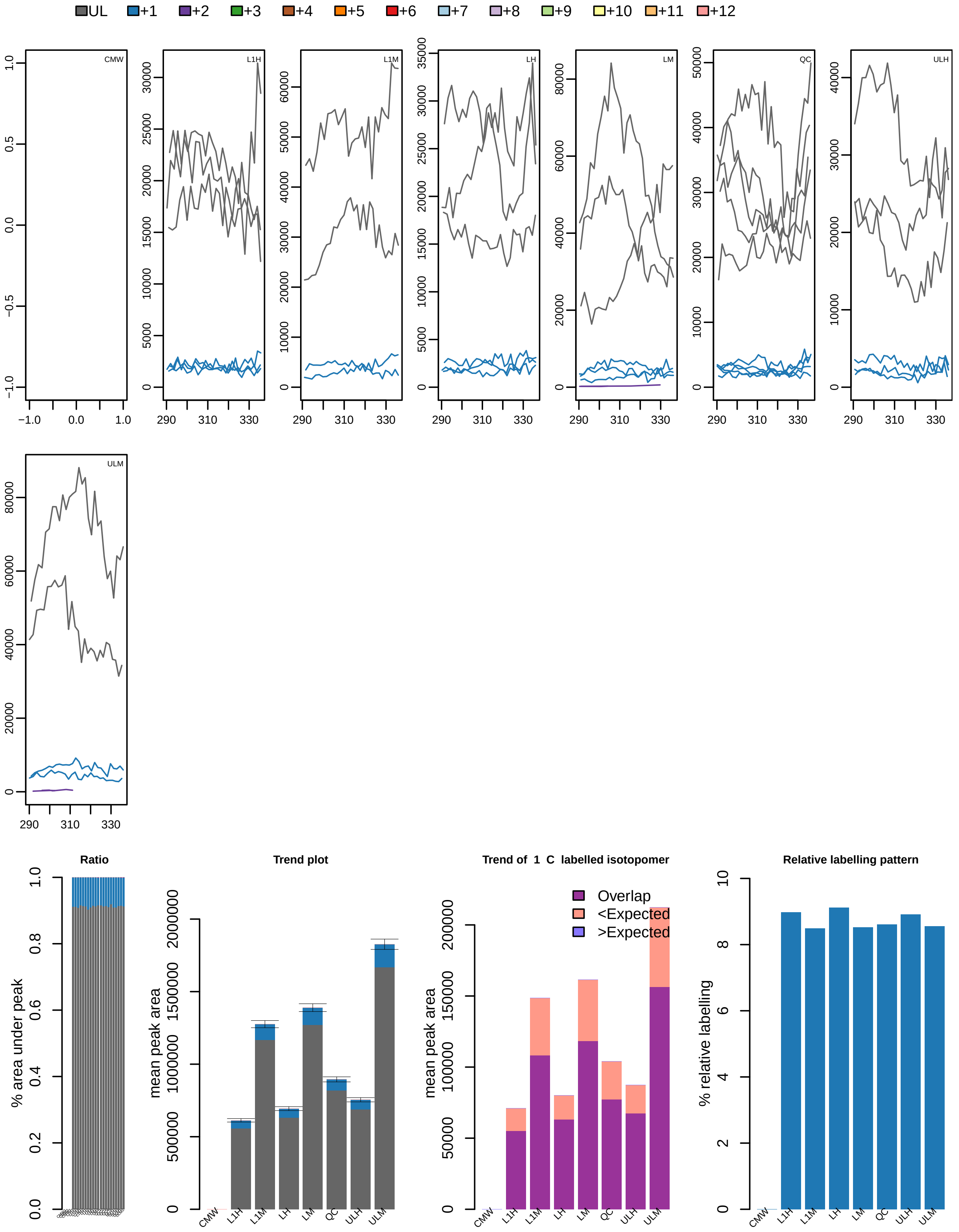

D−myo−Inositol 1,2−cyclic phosphate

Formula: C6H11O8P Mass: 242.019 Std.RT: 1099.4171382 Ion: NEC

G1

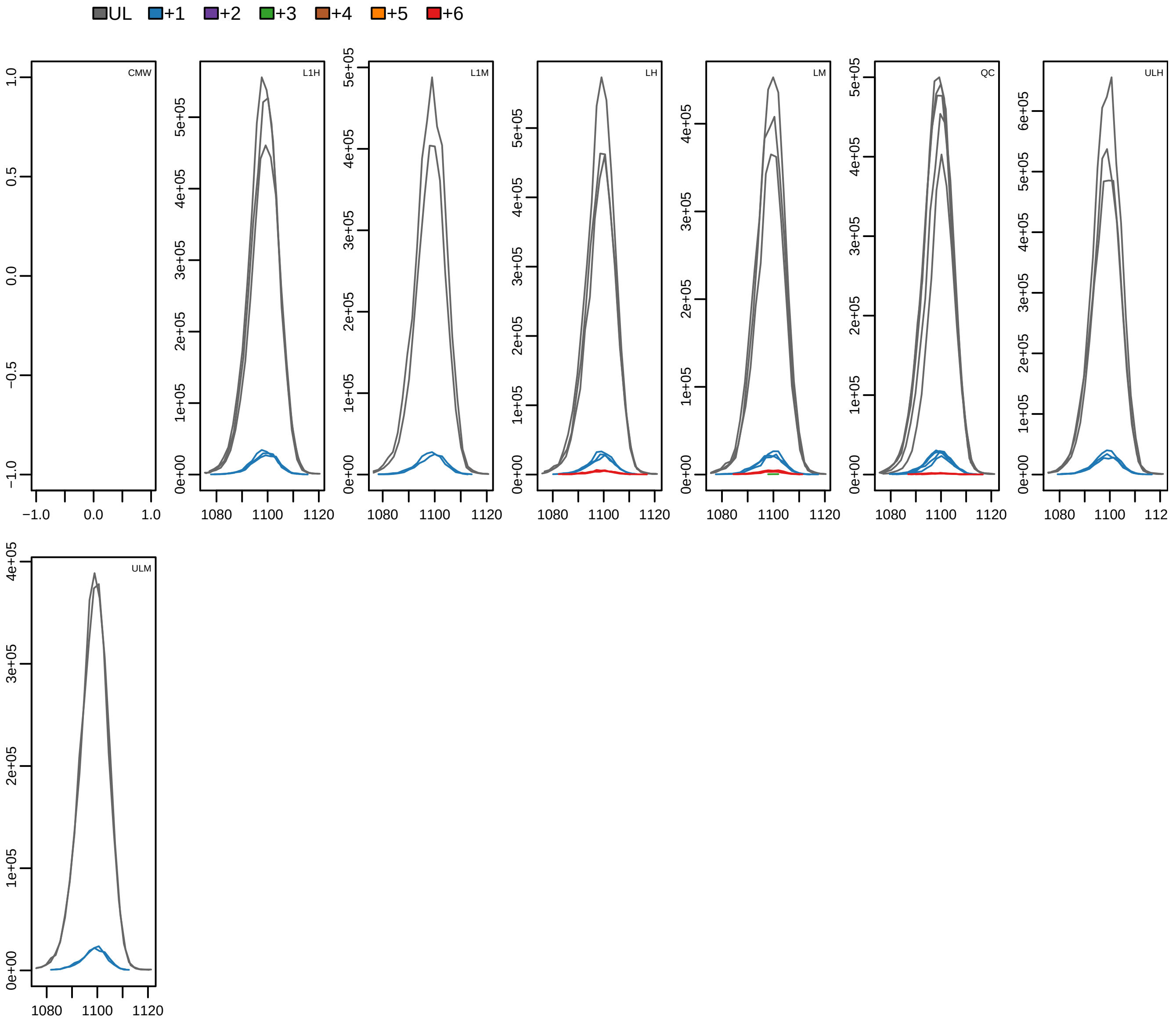

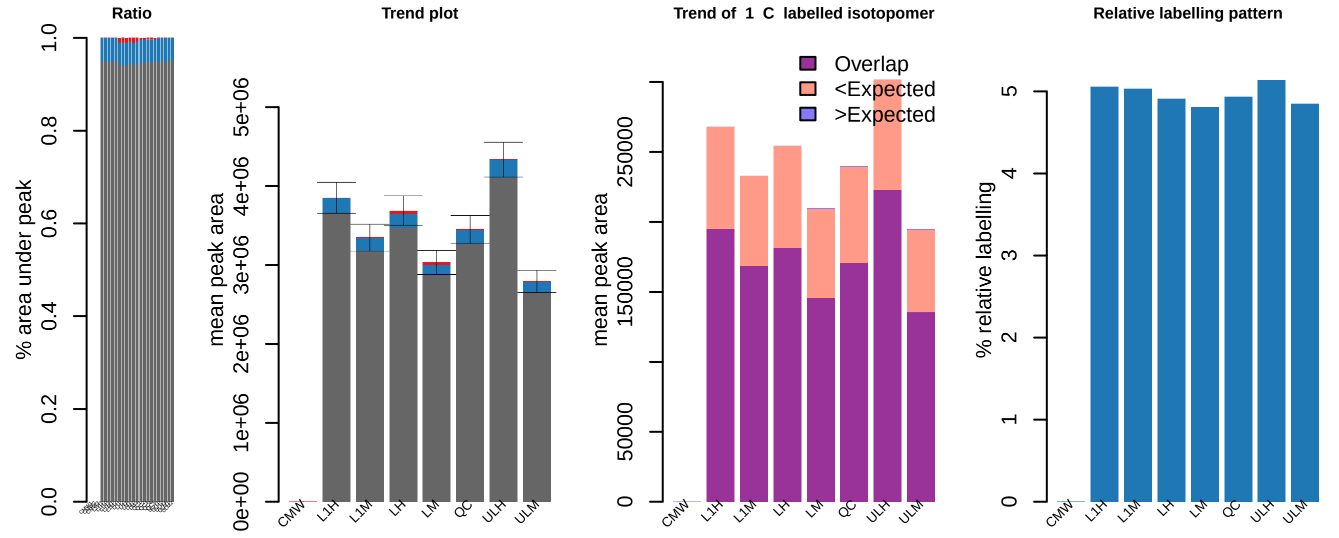

L−Azetidine 2−carboxylic acid

Formula: C4H7NO2 Mass: 101.048 Std.RT: 903.313497 Ion: NEG

G1

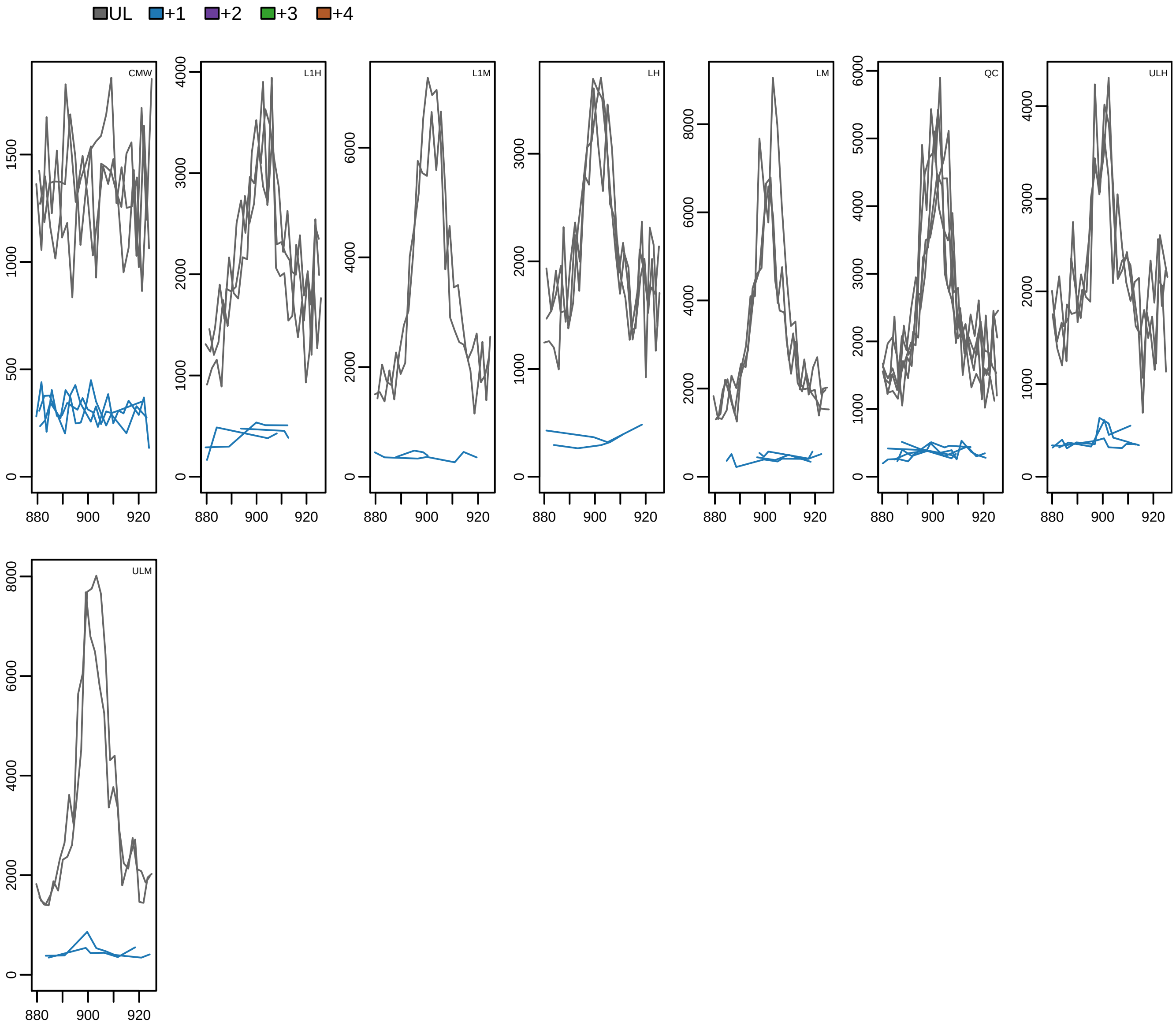

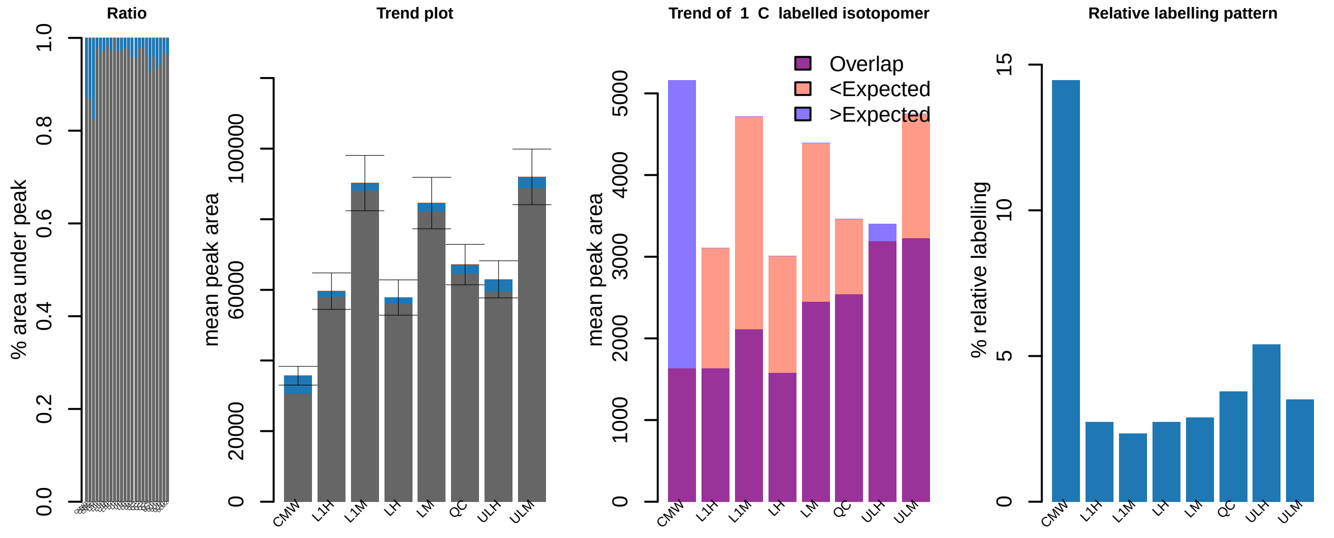

(S)−AMPA

Formula: C7H10N2O4 Mass: 186.064 Std.RT: 653.1720024 Ion: NEG

G1

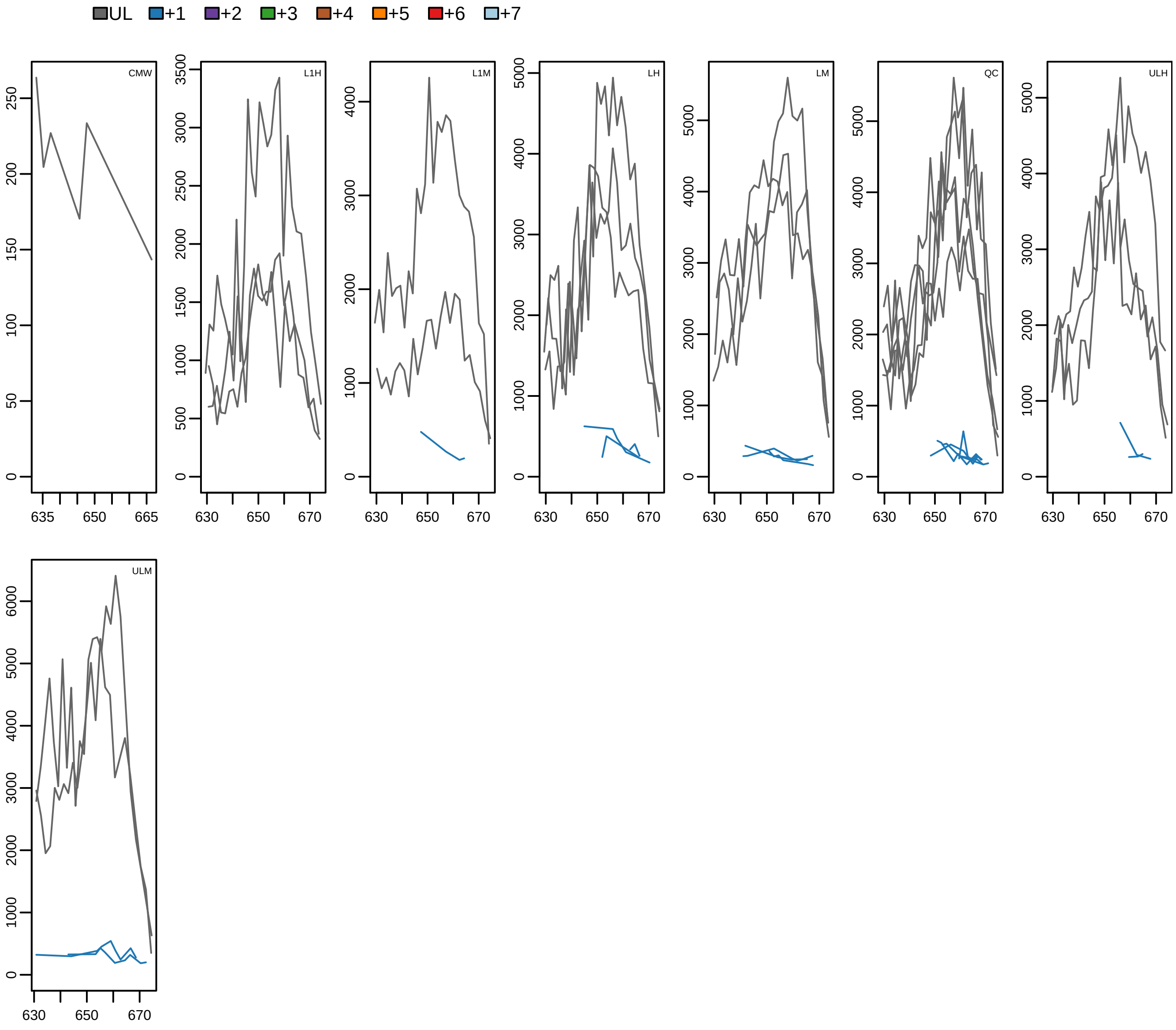

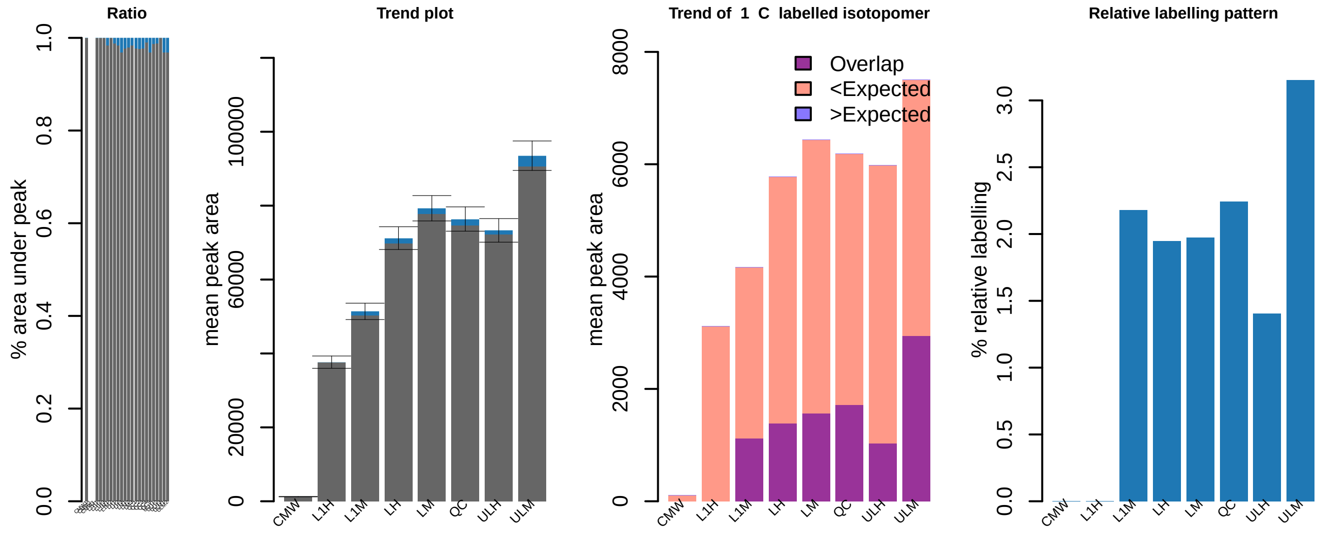

DL−Methionine sulfone

G1

Formula: C5H11NO4S Mass: 181.041 Std.RT: 737.292267 Ion: NEG

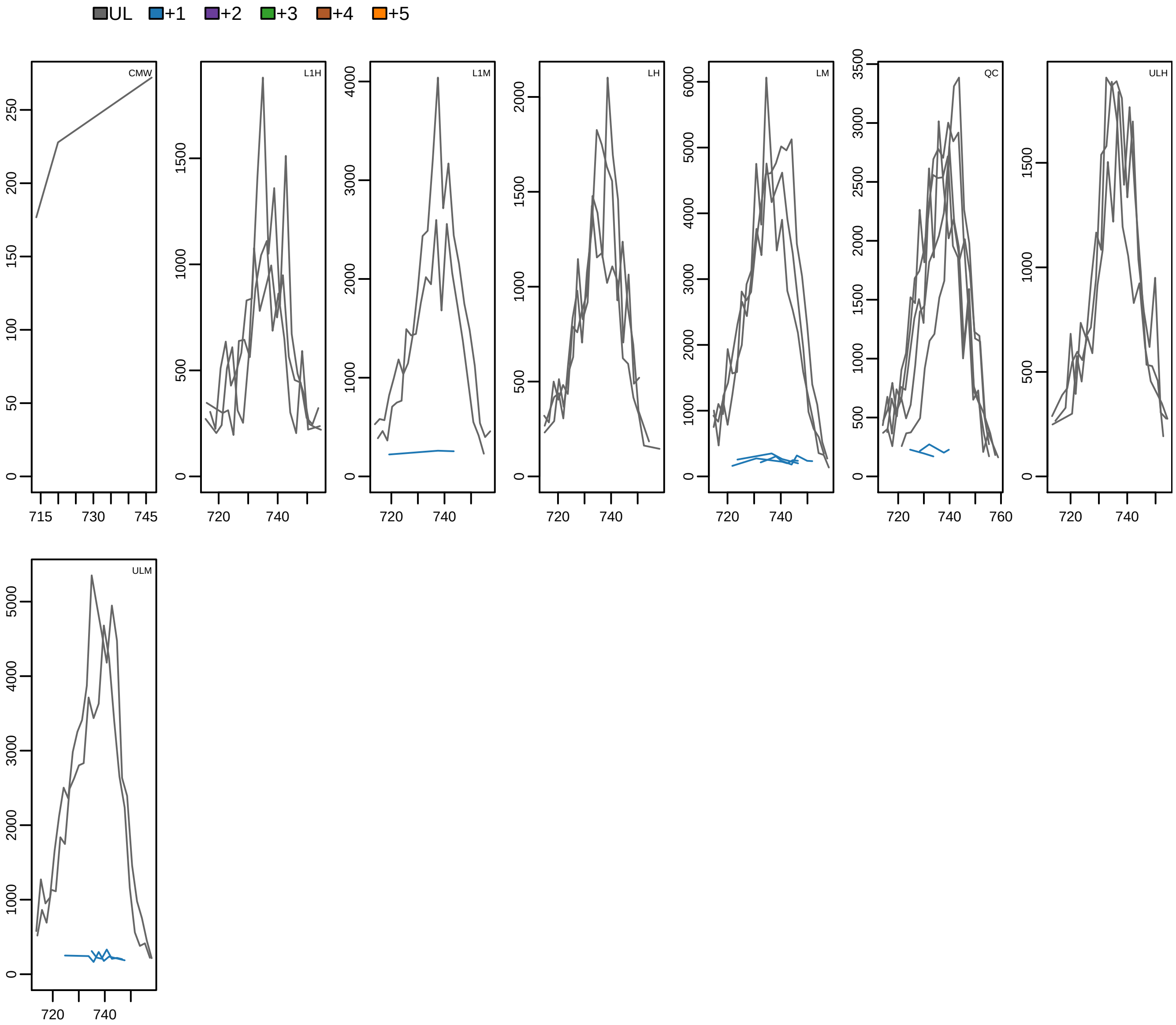

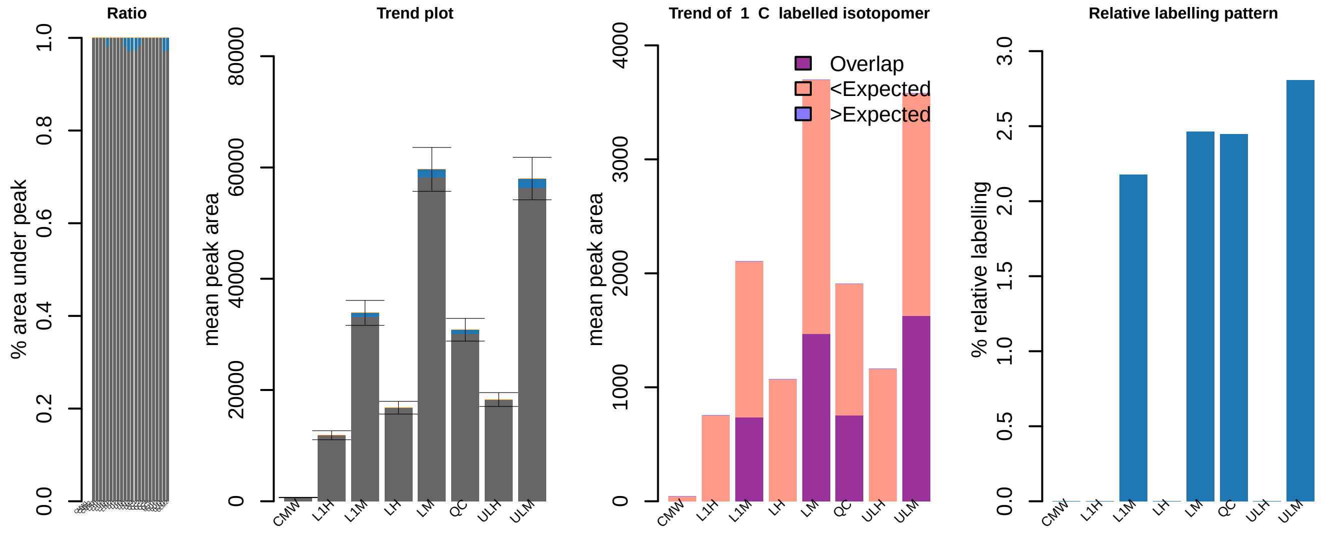

2−Aminobutan−4−olide

G1

Formula: C4H7NO2 Mass: 101.048 Std.RT: 966.4559124 Ion: NEG

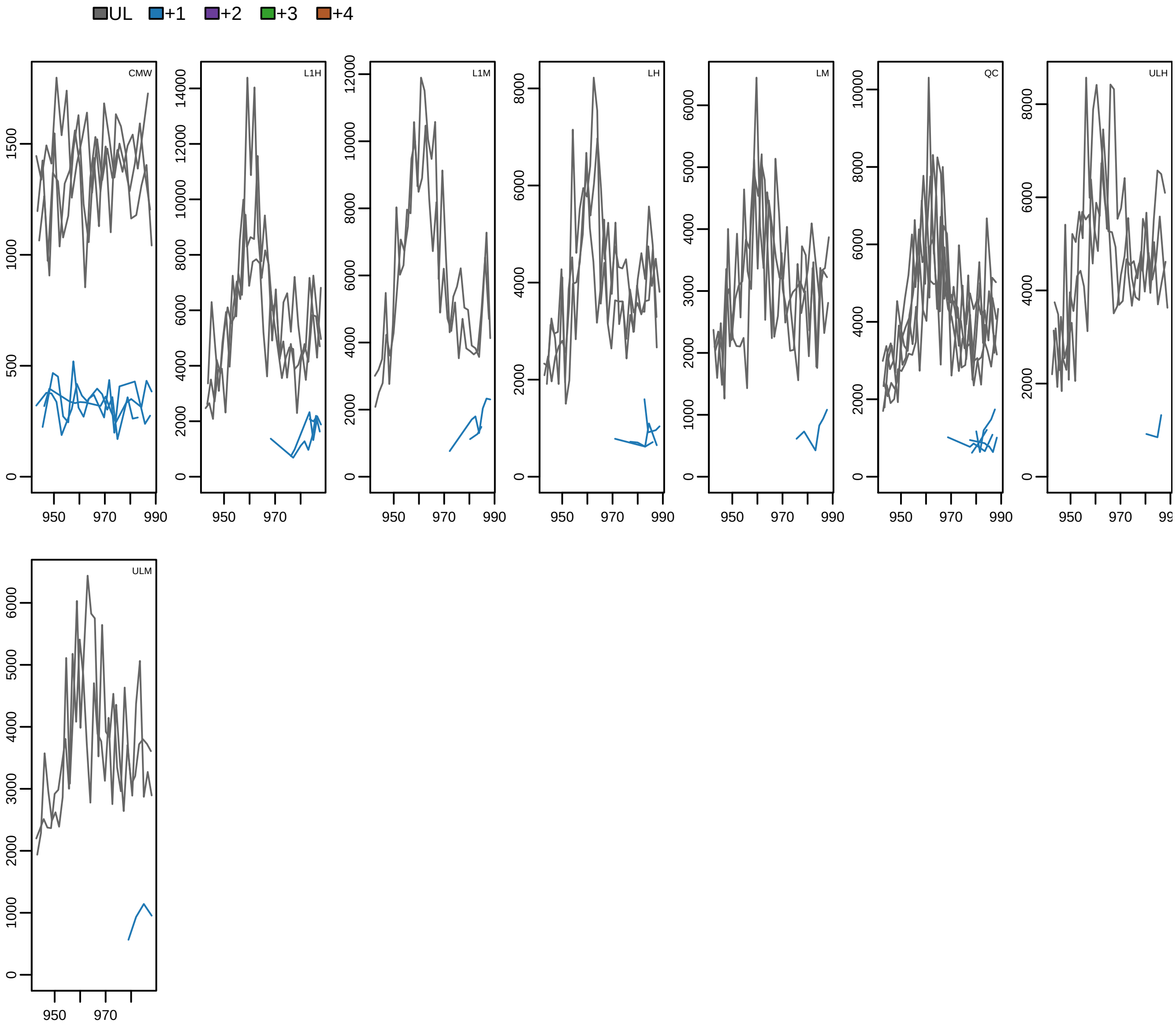

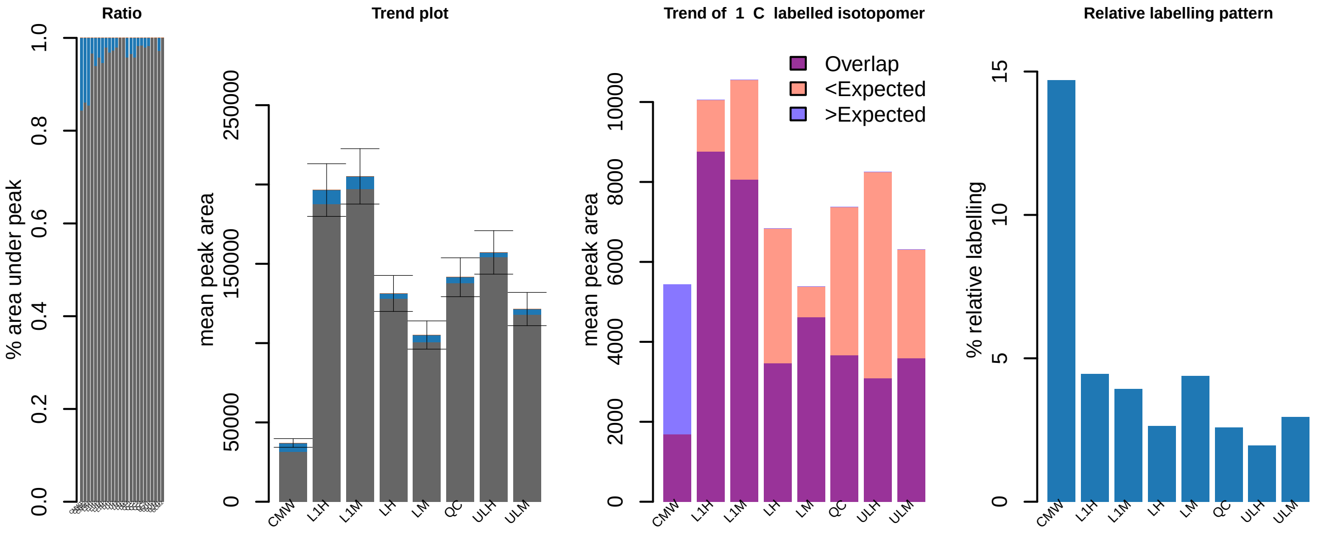

3−Sulfolactate

Formula: C3H6O6S Mass: 169.989 Std.RT: 1102.3691562 Ion: NEG

G1

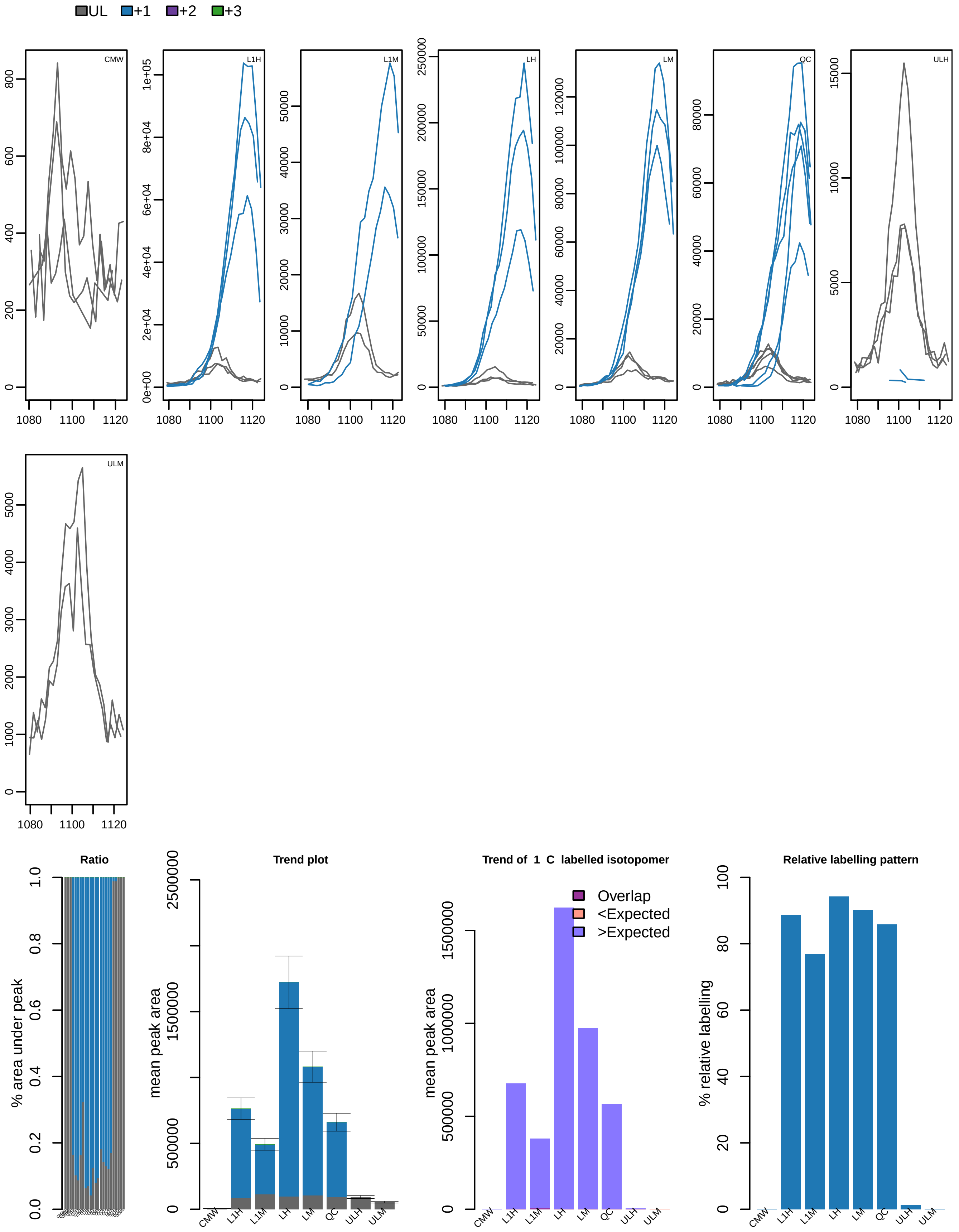

N−Glycosyl−L−asparagine

G1

Formula: C10H18N2O8 Mass: 294.106 Std.RT: 945.832287 Ion: NEC

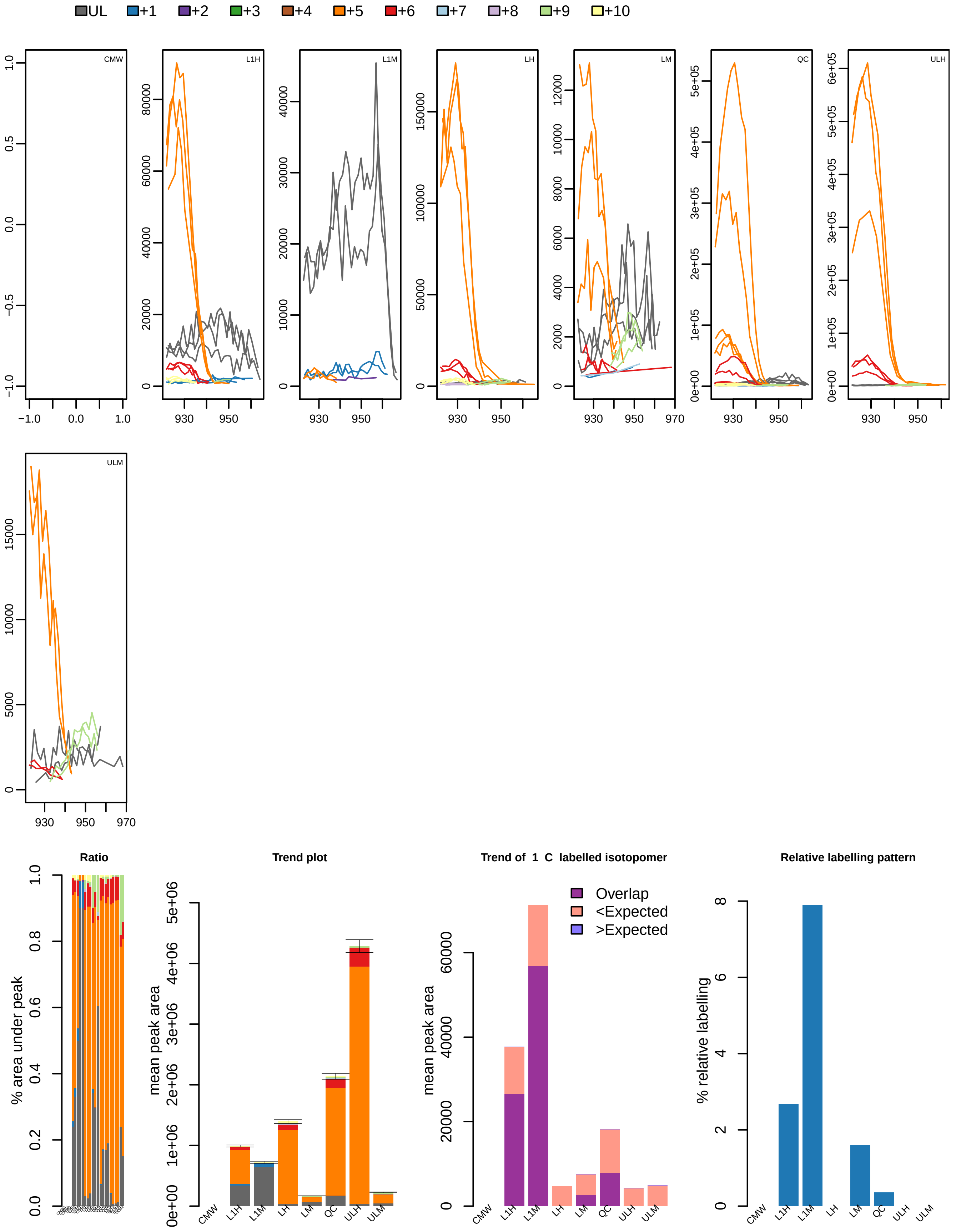

N−formylmaleamate

Formula: $C_5H_5NO_4$ Mass: 143.022 Std.RT: 810.5354994 Ion: NEG

G1

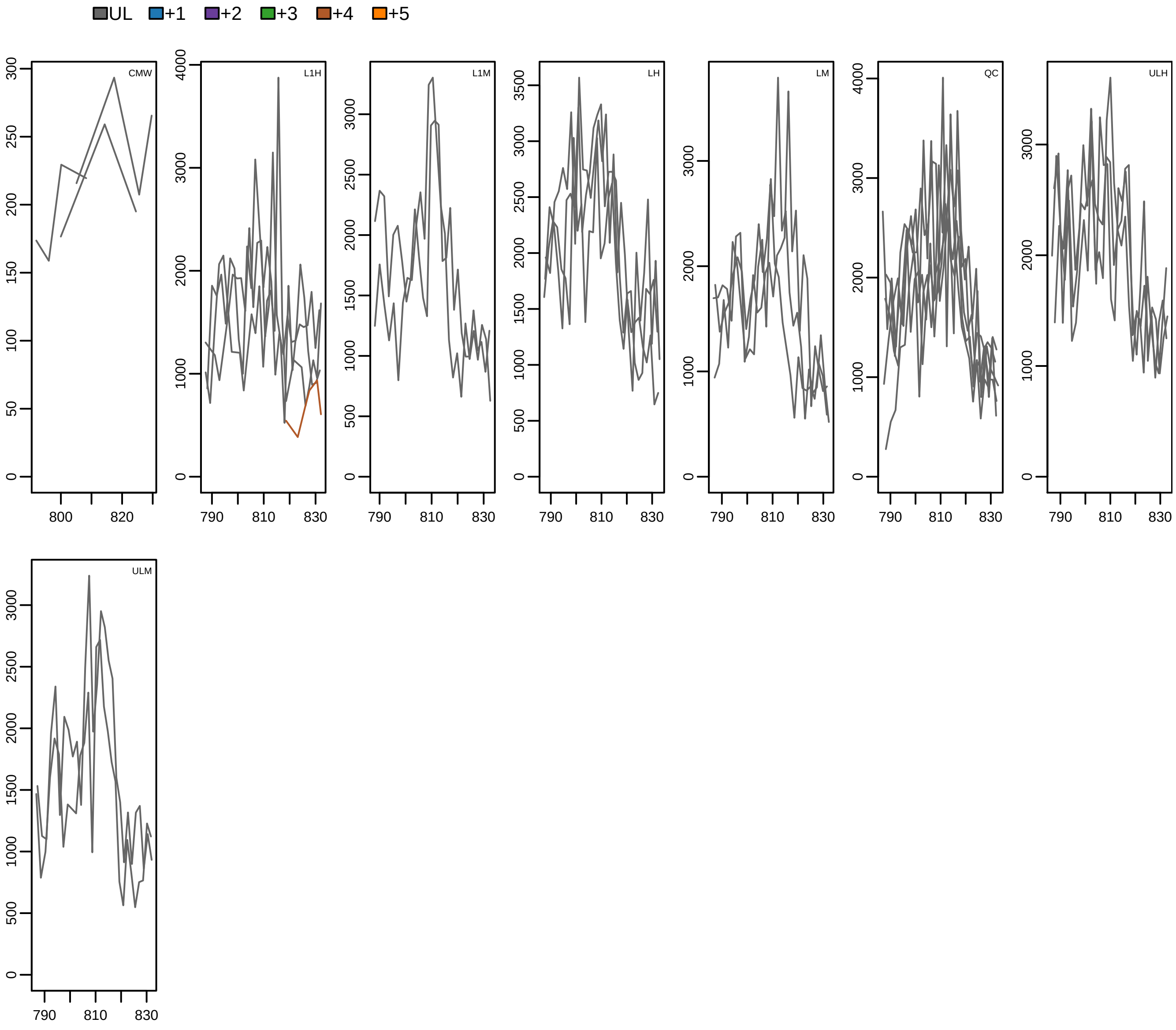

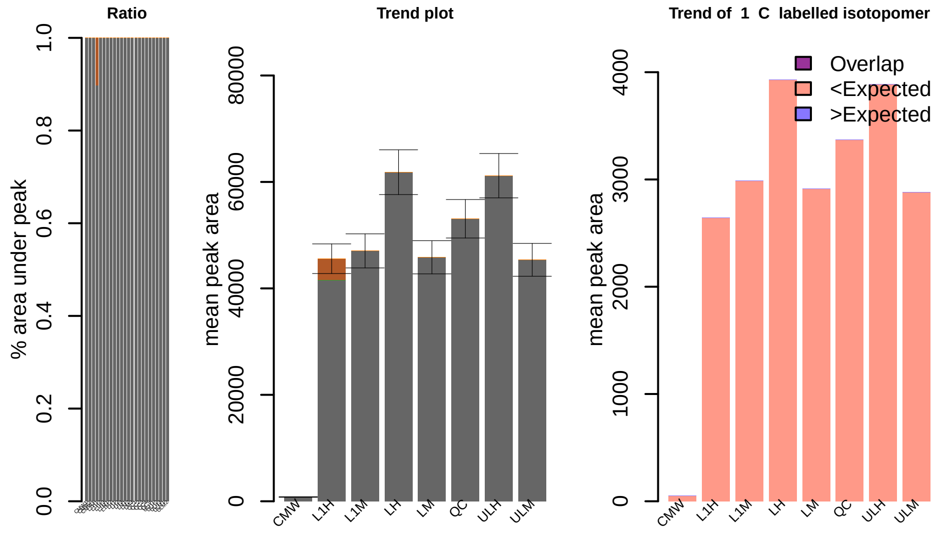

N−acetyl −D− glucosaminitol

Formula: $C_8H_{17}NO_6$ Mass: 223.106 Std.RT: 867.2077146 Ion: NEG

G1

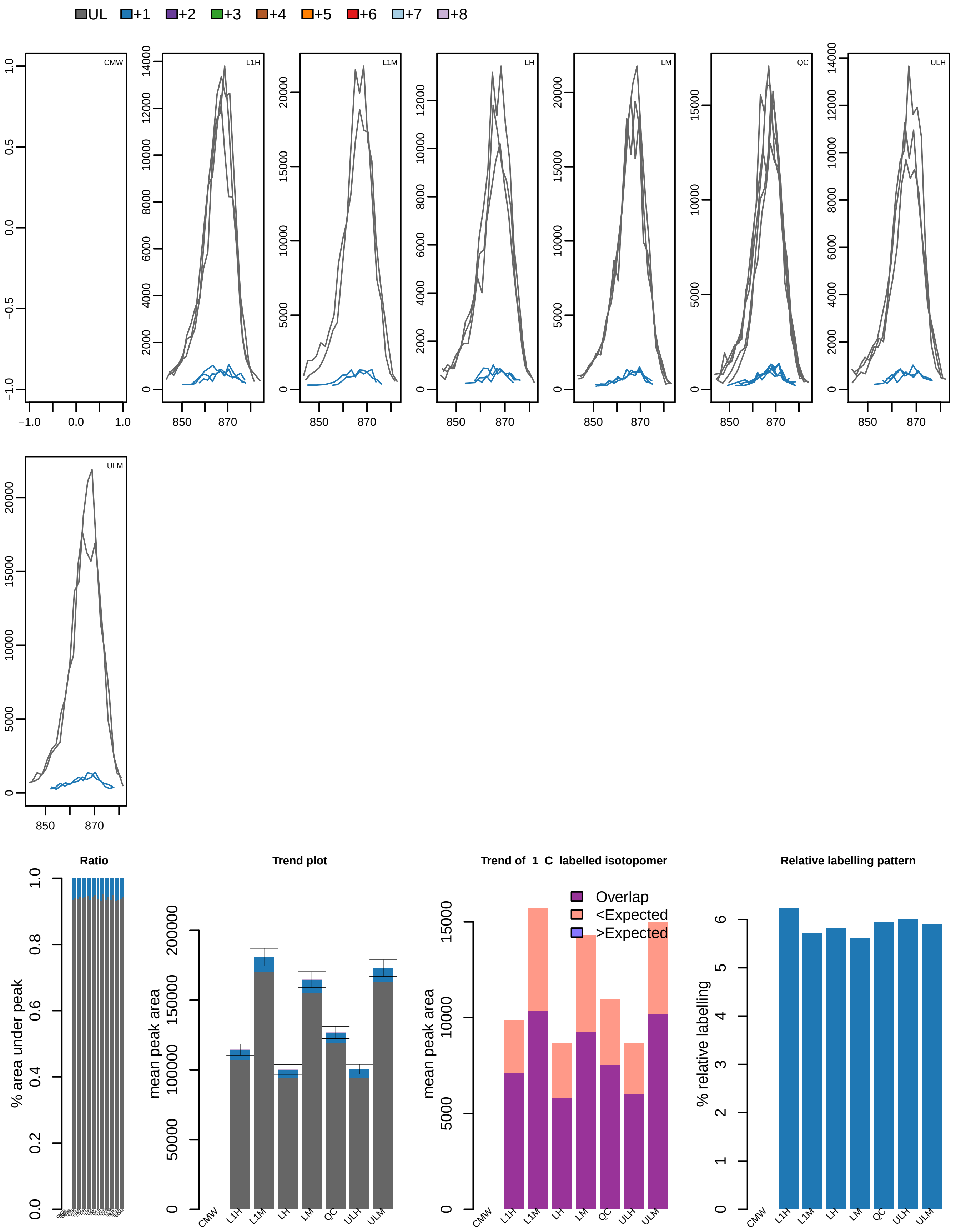

Norepinephrinesulfate

Formula: C8H11NO6S Mass: 249.031 Std.RT: 1197.13047 Ion: NEG

G1

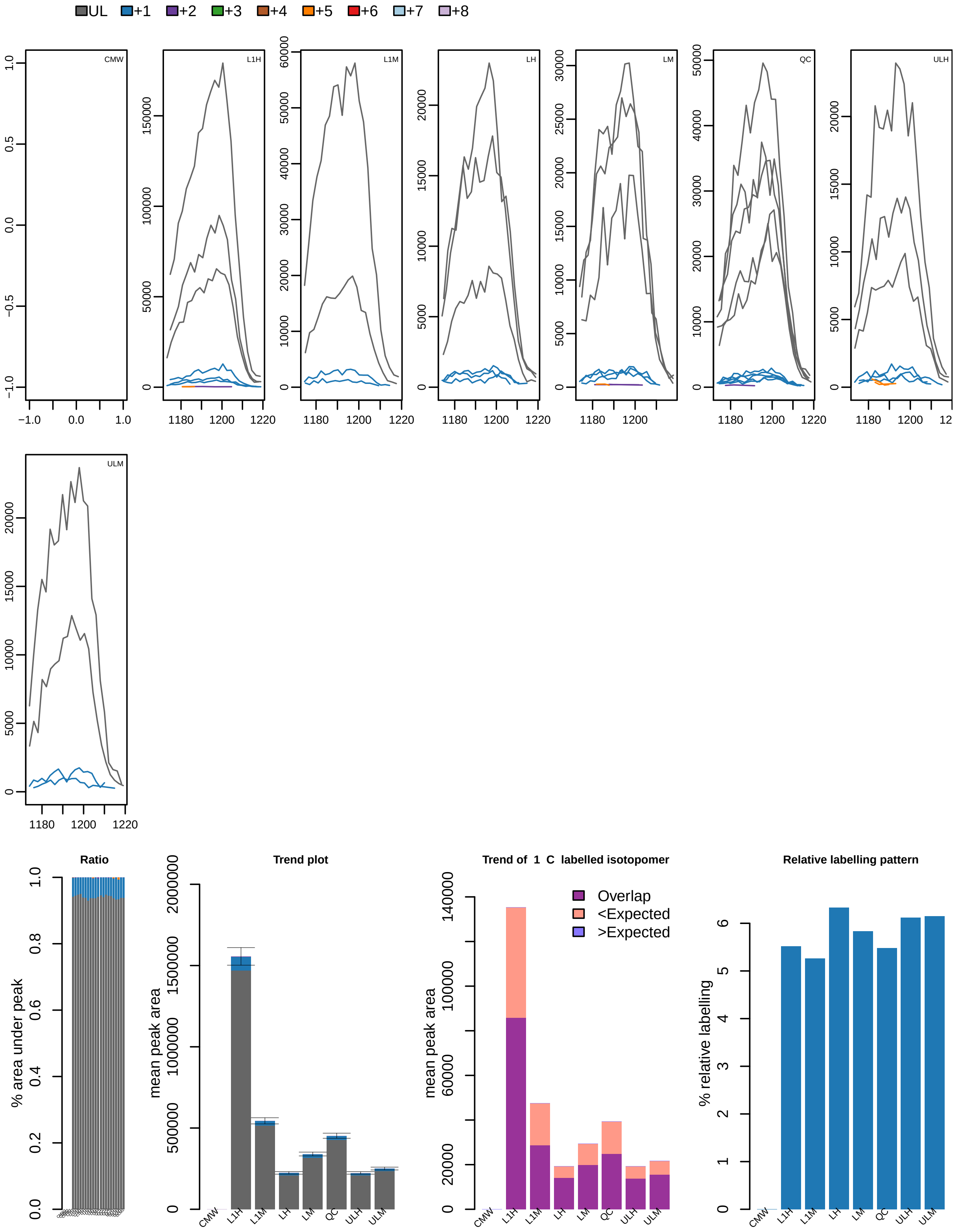

(+/−)−6−Acetonyldihydrochelerythrine

Formula: C24H23NO5 Mass: 405.158 Std.RT: 216.88416738 Ion: NE

G1

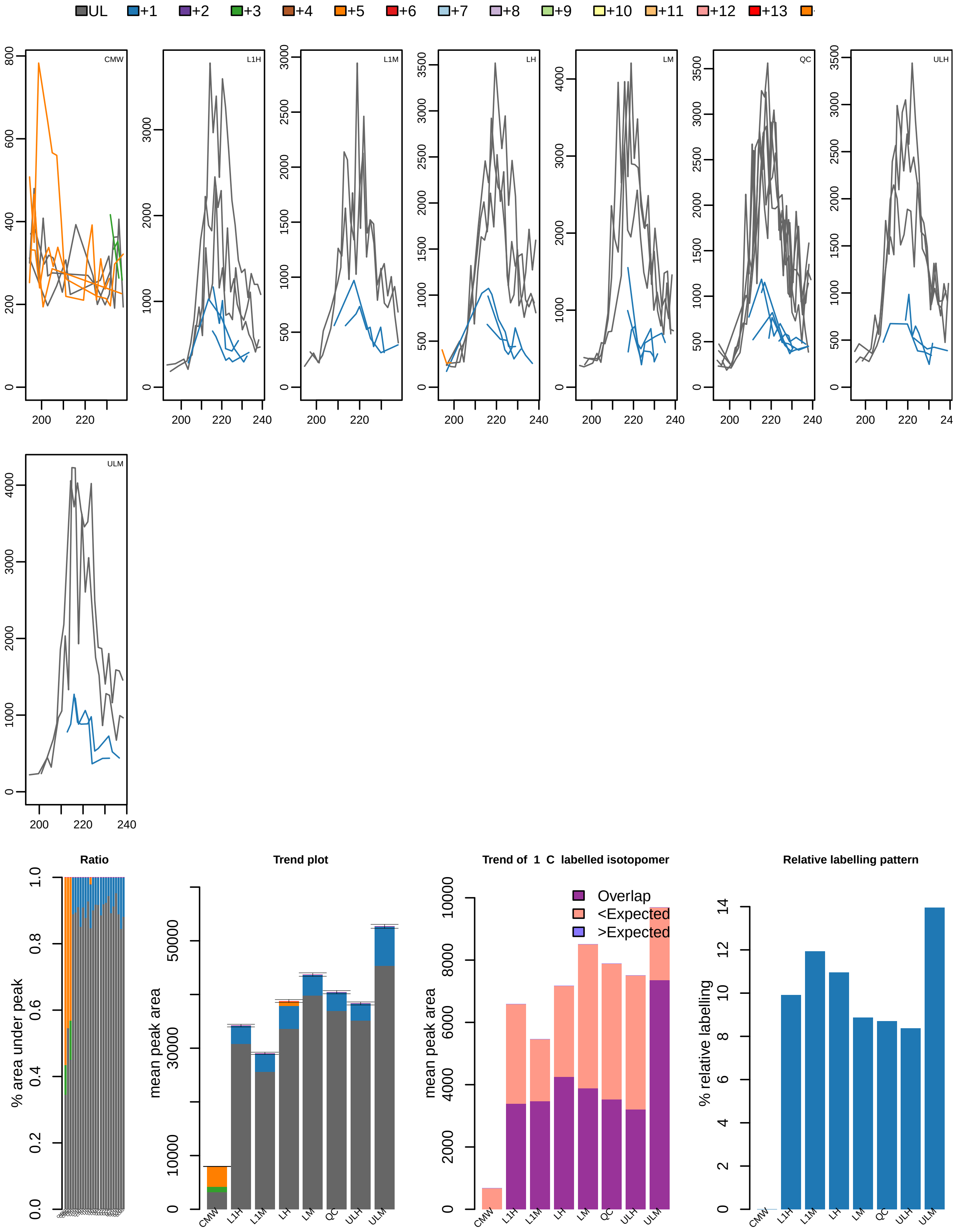

1,4−Bis(2−ethylhexyl) sulfosuccinate

Formula: C20H38O7S Mass: 422.234 Std.RT: 205.01136498 Ion: NE

G1

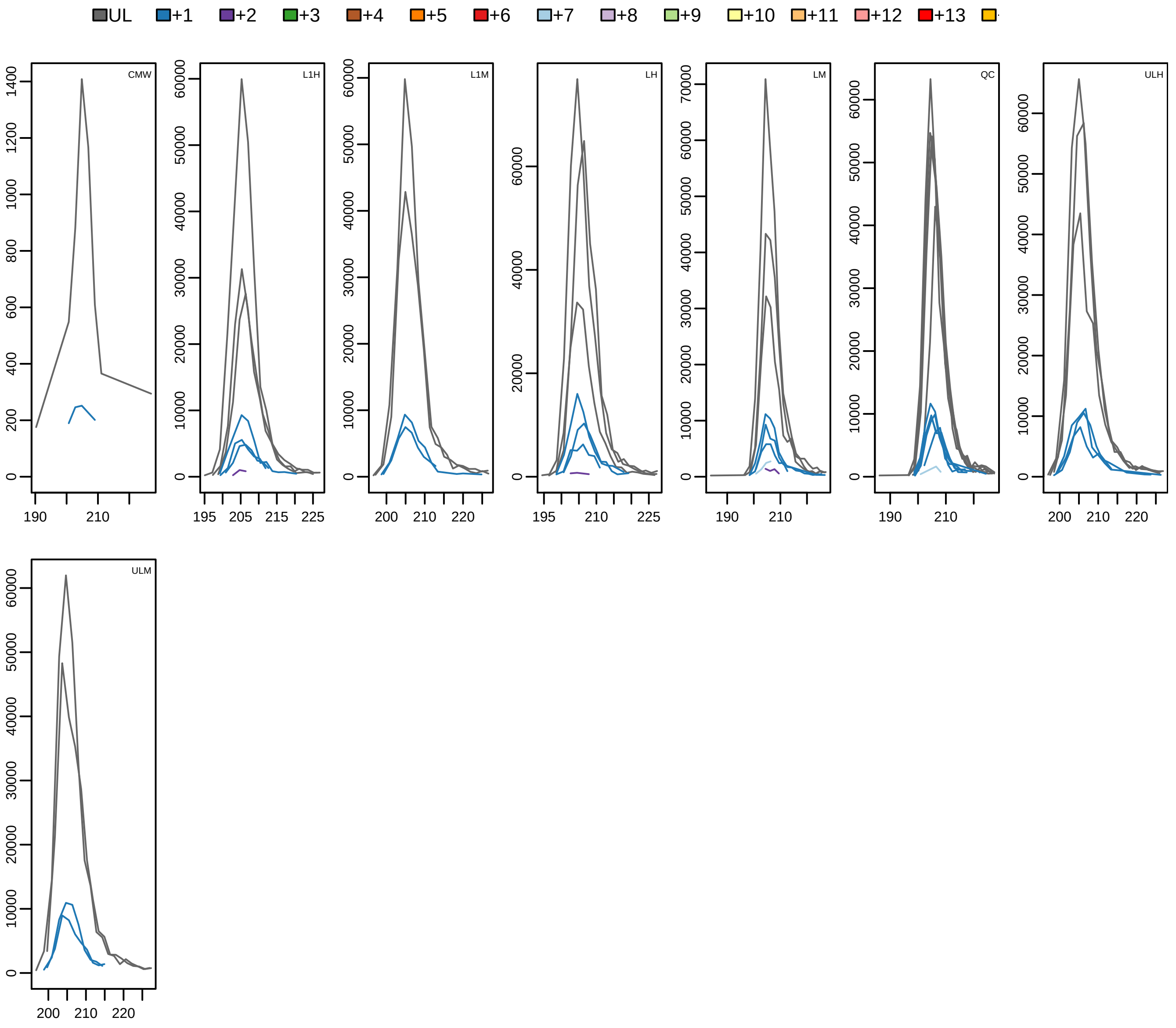

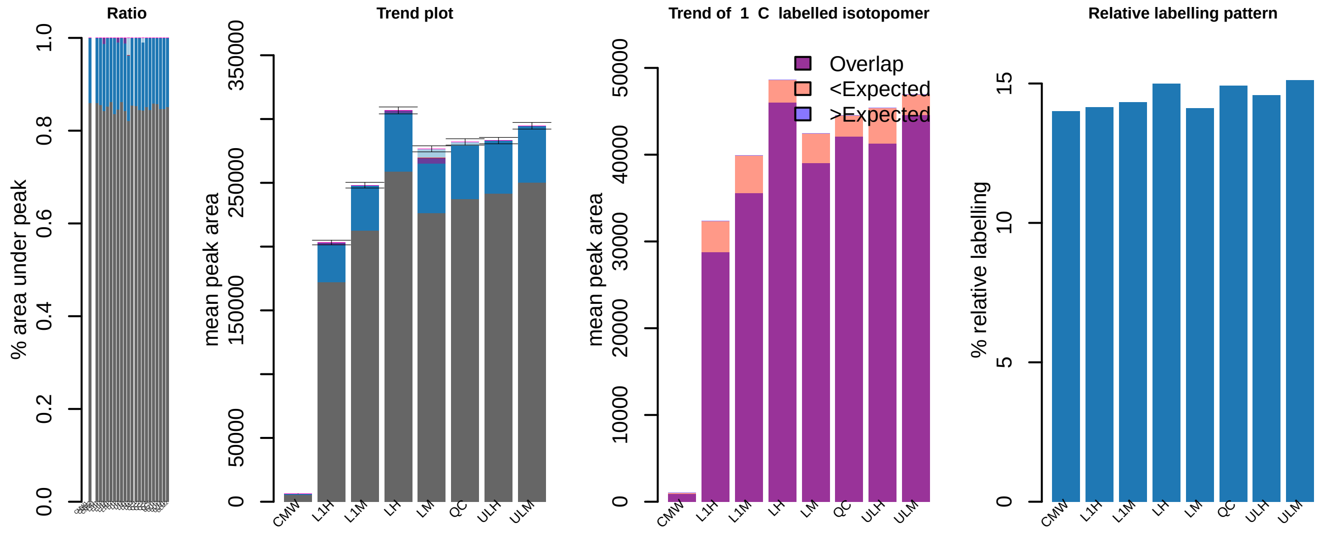

4−hydroperoxyoctadeca−t−2−nonenal

Formula: C9H16O3 Mass: 172.11 Std.RT: 212.2257093 Ion: NEG

G1

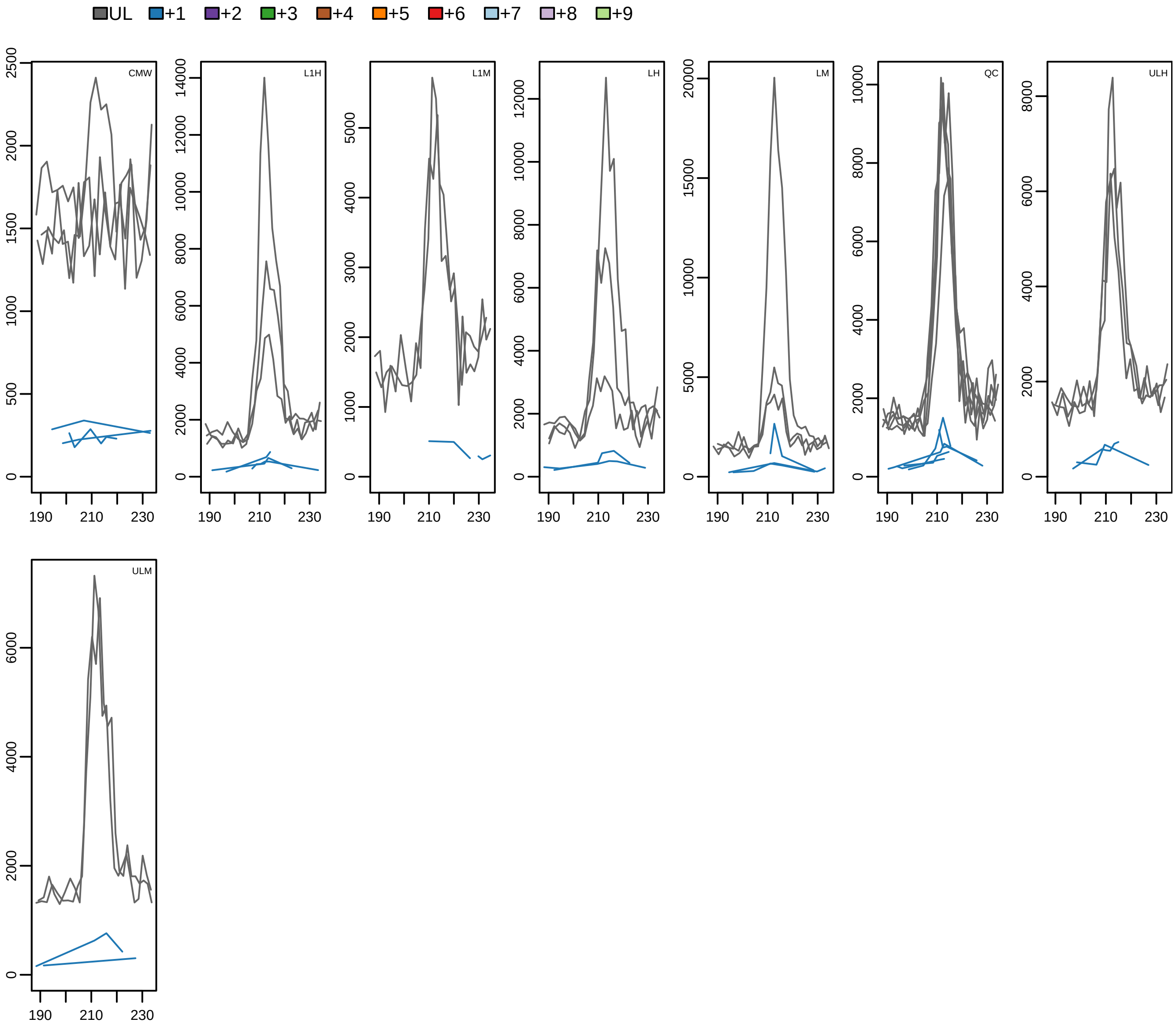

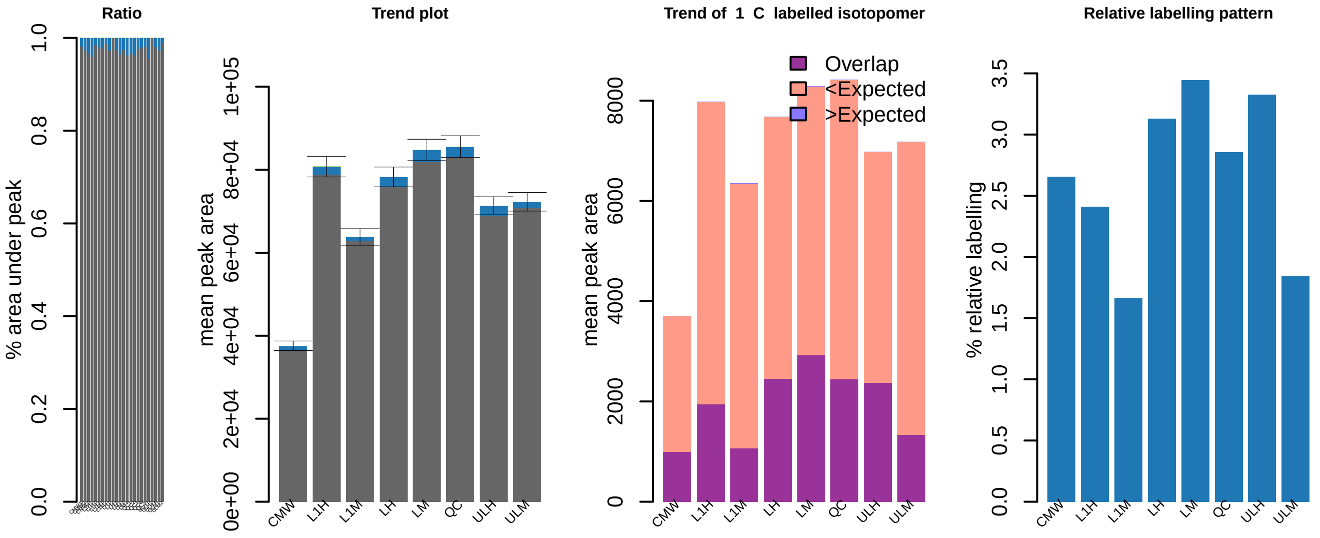

4−hydroperoxyoctadeca−t−2−nonenal

Formula: C9H16O3 Mass: 172.11 Std.RT: 212.2257093 Ion: NEG

G2

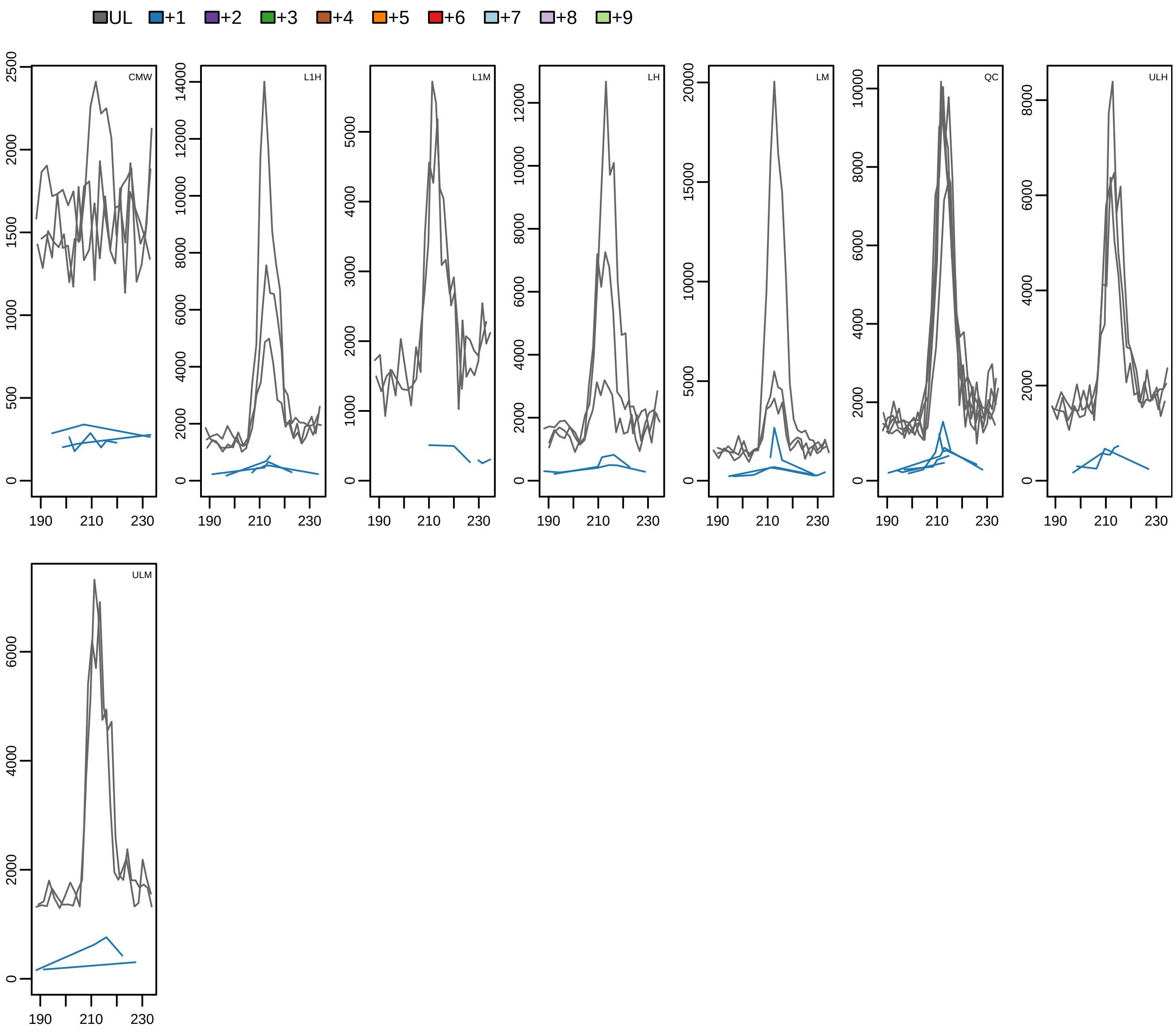

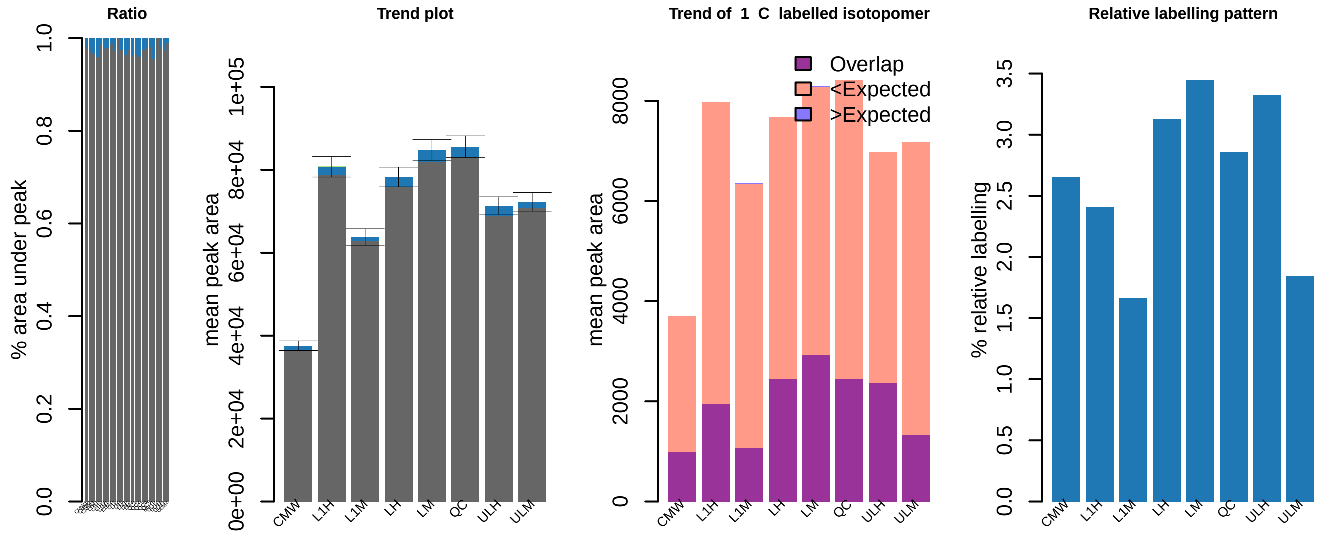

N−Ethylglycocyamine

Formula: C8H17NO5 Mass: 207.111 Std.RT: 867.4571448 Ion: NEG

G1

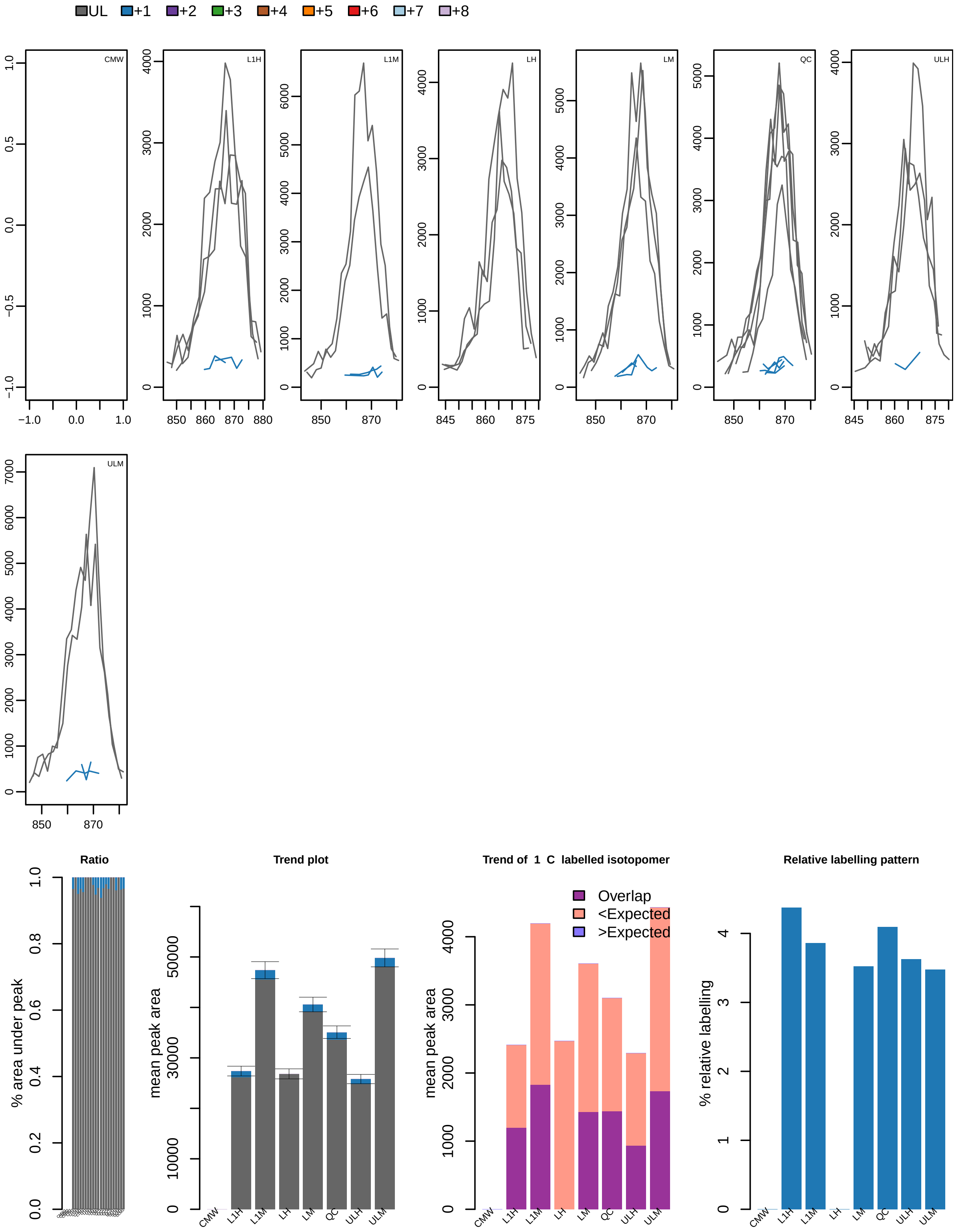

parabanate

Formula: C3H2N2O3 Mass: 114.007 Std.RT: 811.5734964 Ion: NEG

G1

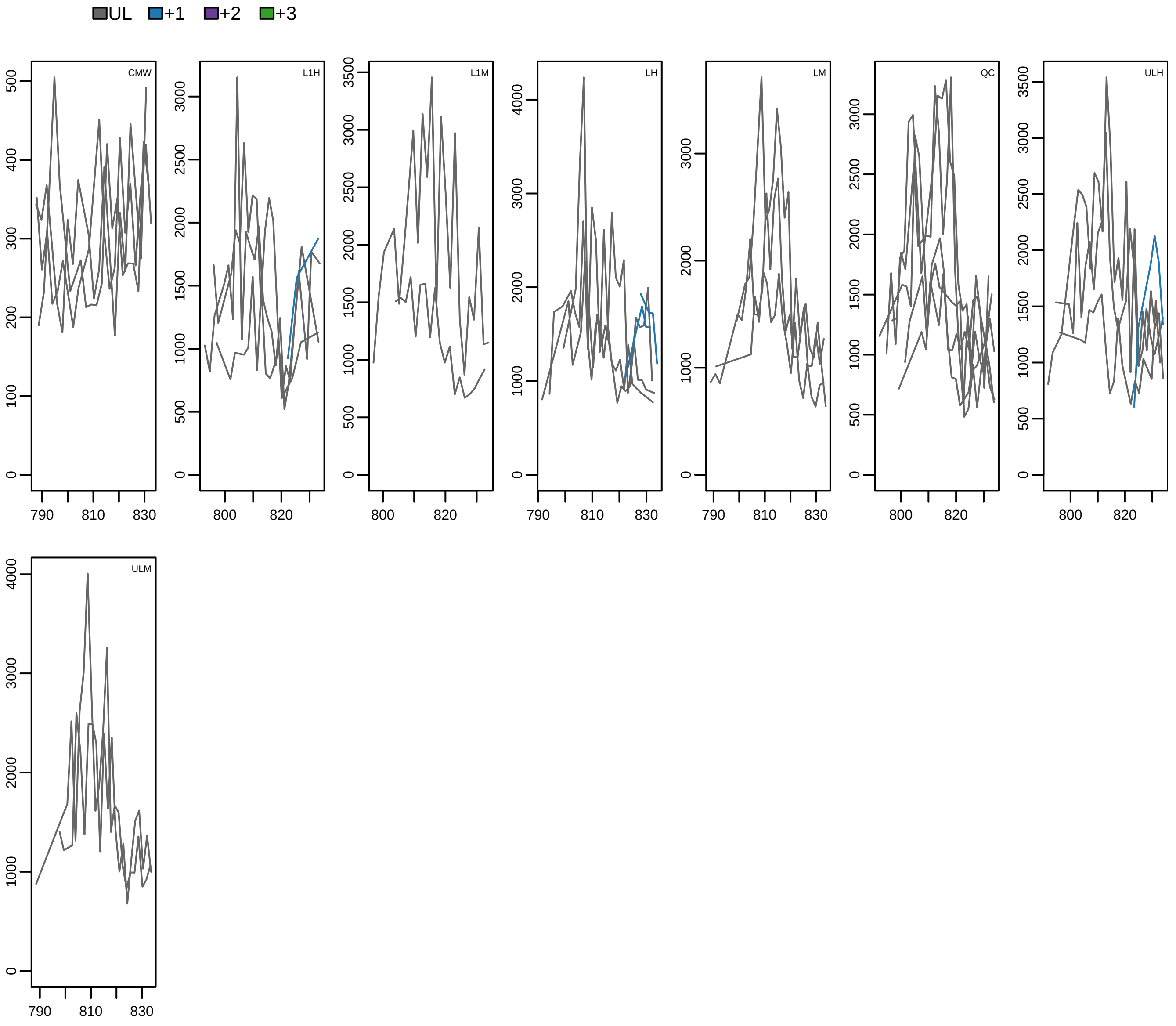

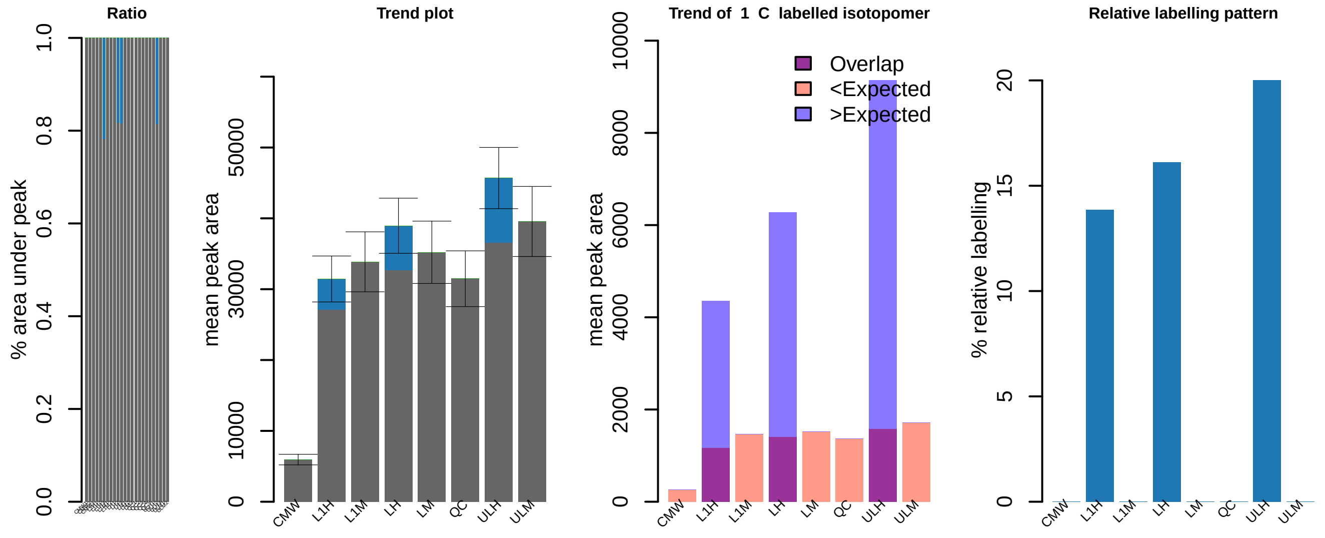

5D−5−O−Methyl−2,3,5/4,6−pentahydroxycyclohexanon

G1

Formula: C7H12O6 Mass: 192.063 Std.RT: 803.6734902 Ion: NEG

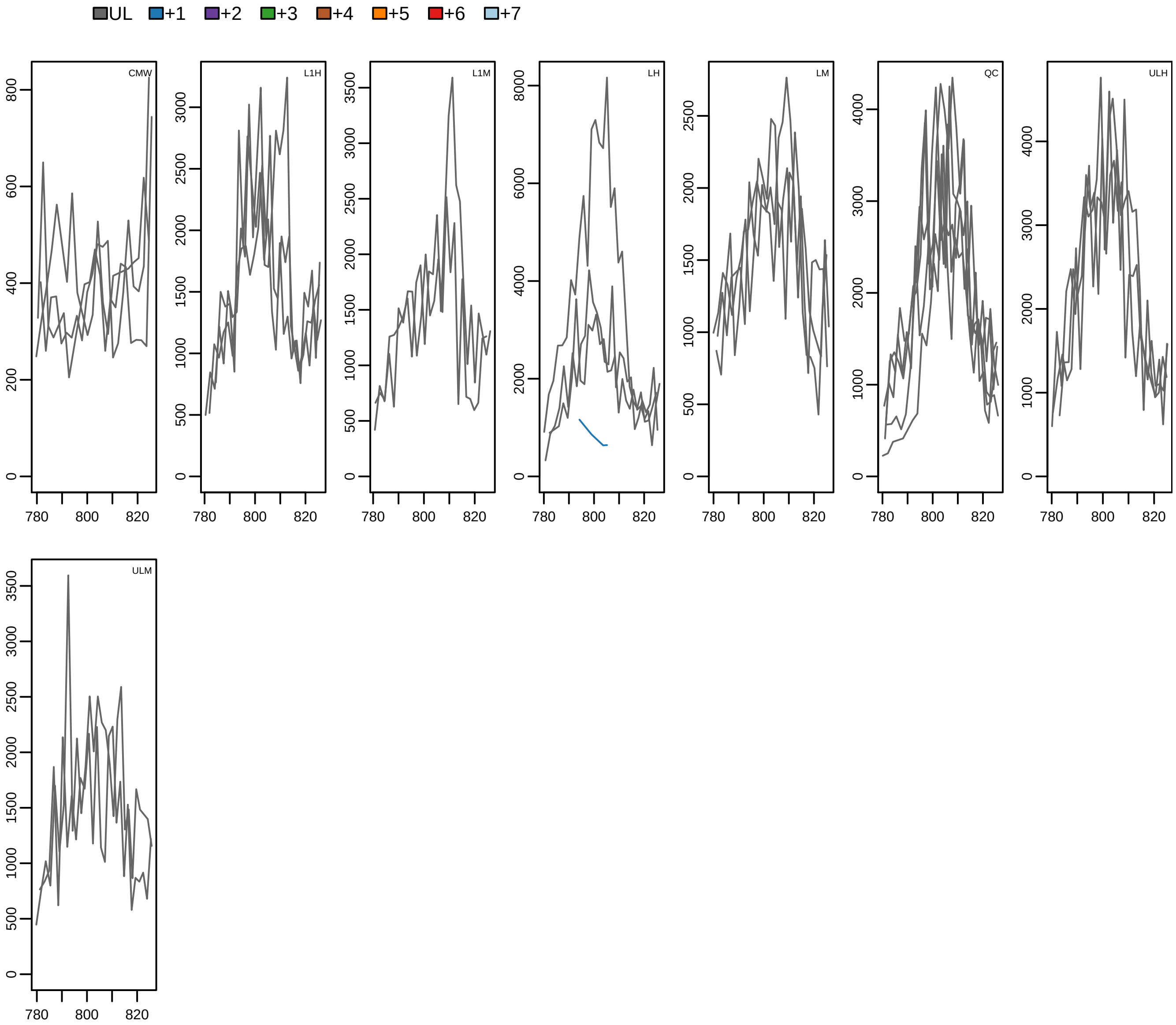

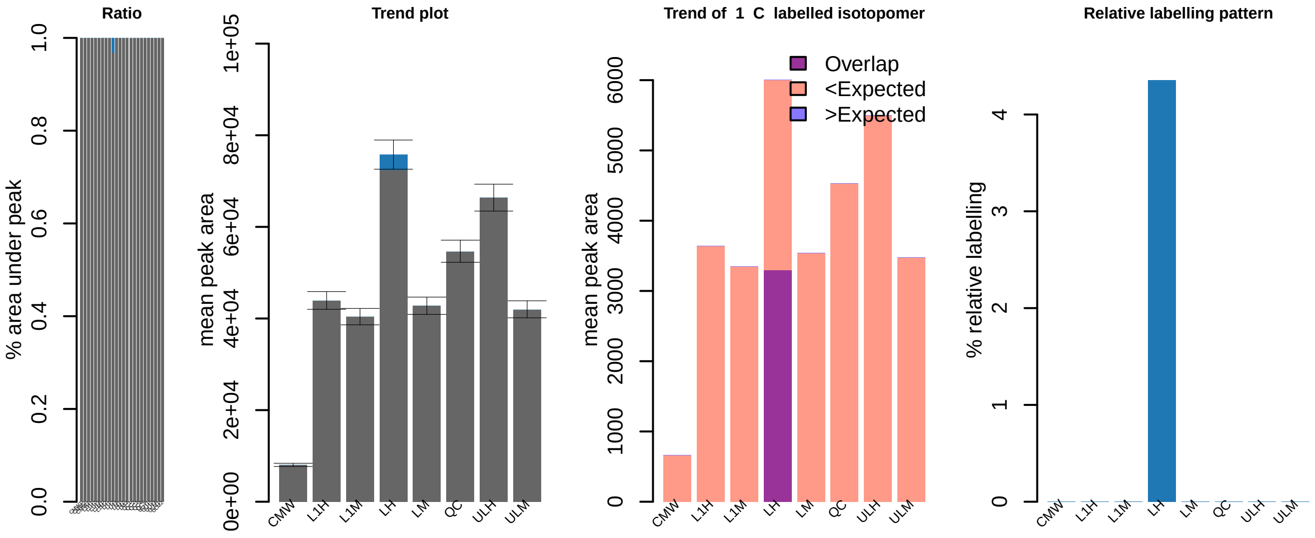

Propane−1,2−diol 1−phosphate

Formula: C3H9O5P Mass: 156.019 Std.RT: 717.8616492 Ion: NEG

G1

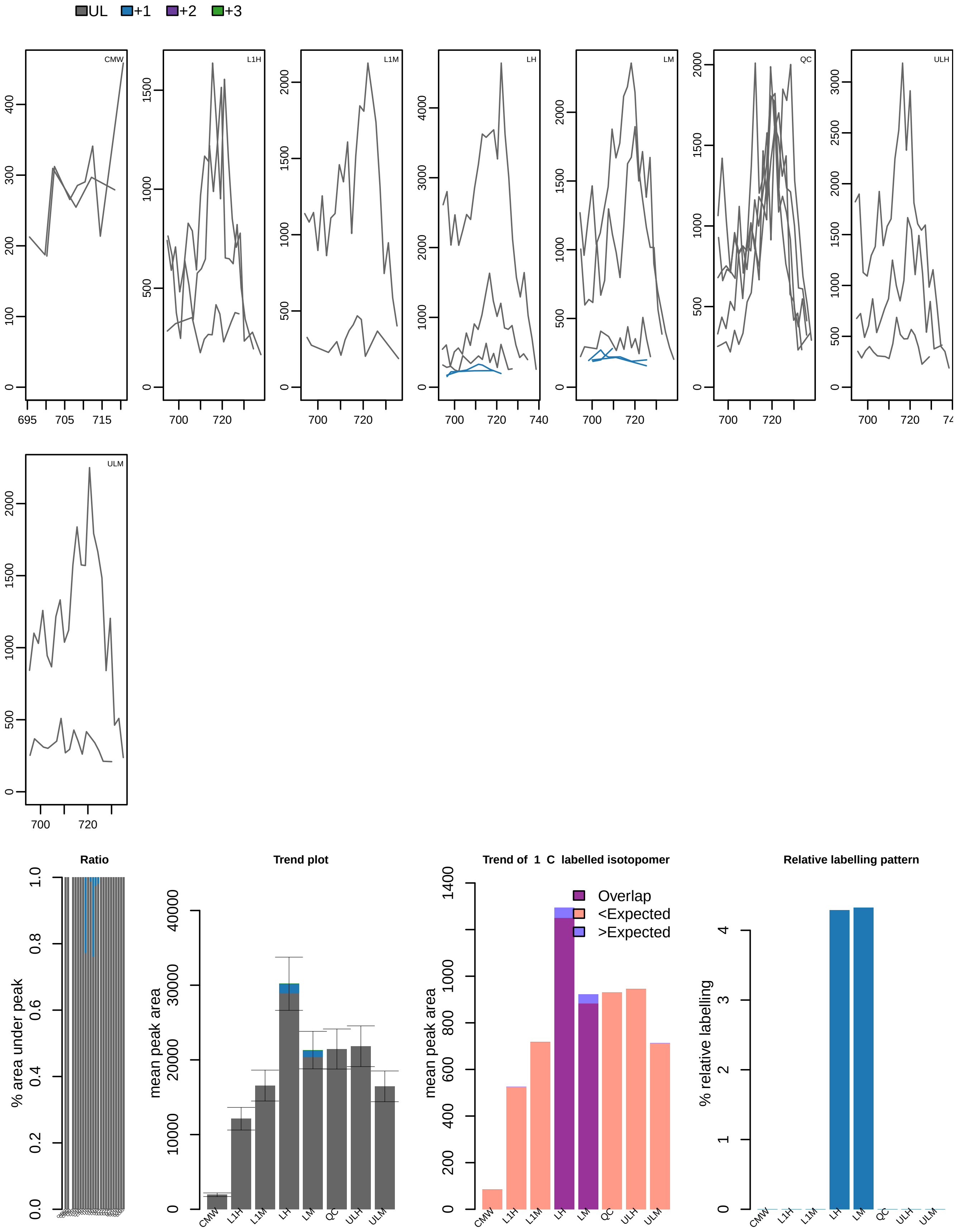

2−oxobut−3−enanoate

Formula: C4H4O3 Mass: 100.016 Std.RT: 982.8730038 Ion: NEG

G1

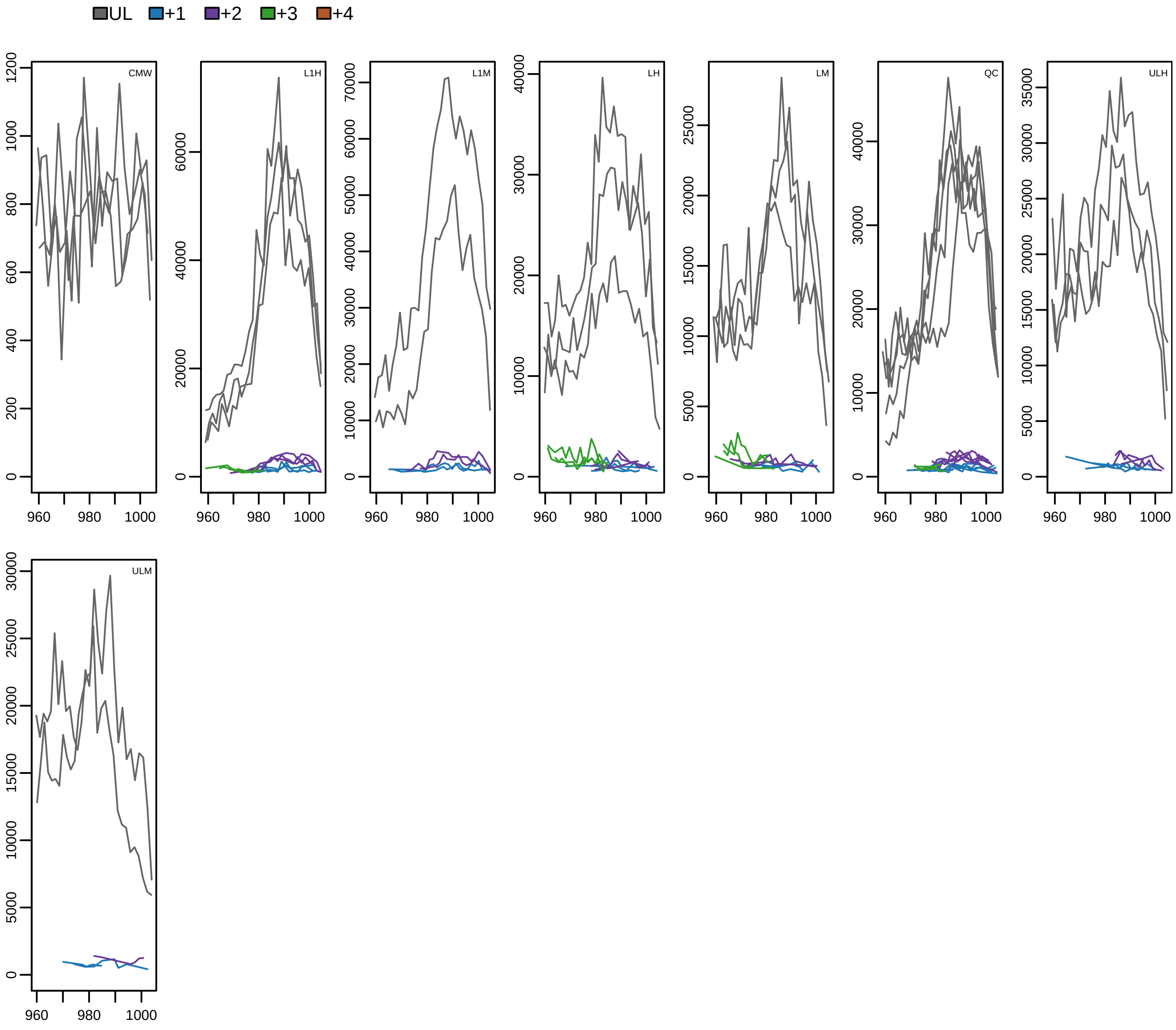

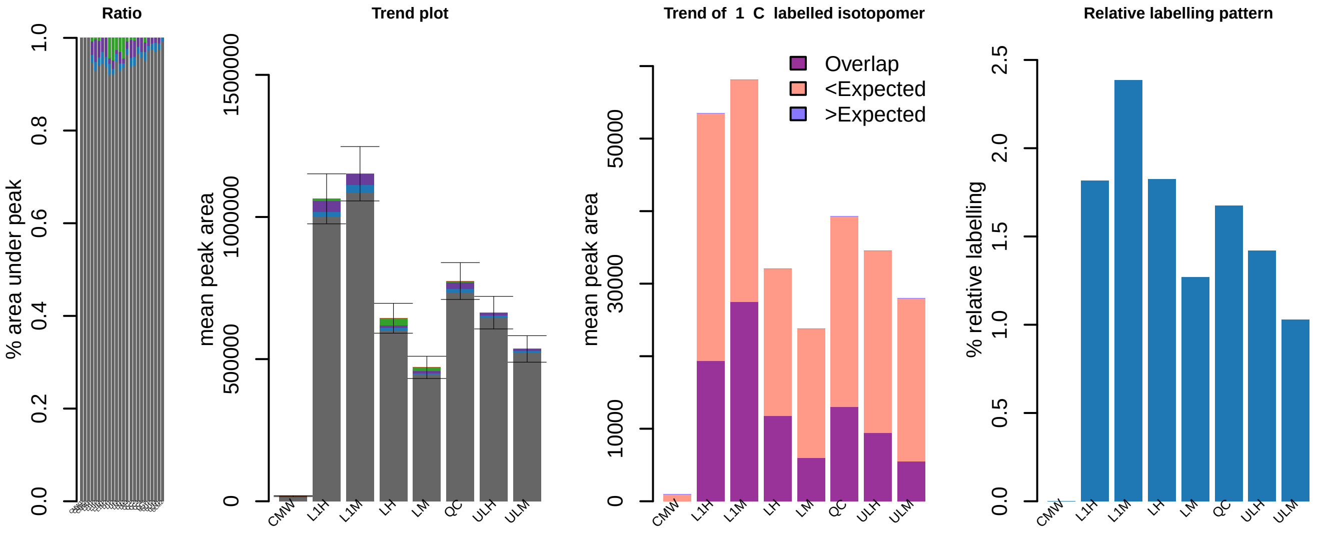

2−oxobut−3−enanoate

Formula: C4H4O3 Mass: 100.016 Std.RT: 982.8730038 Ion: NEG

G2

UL +1 +2 +3 +4

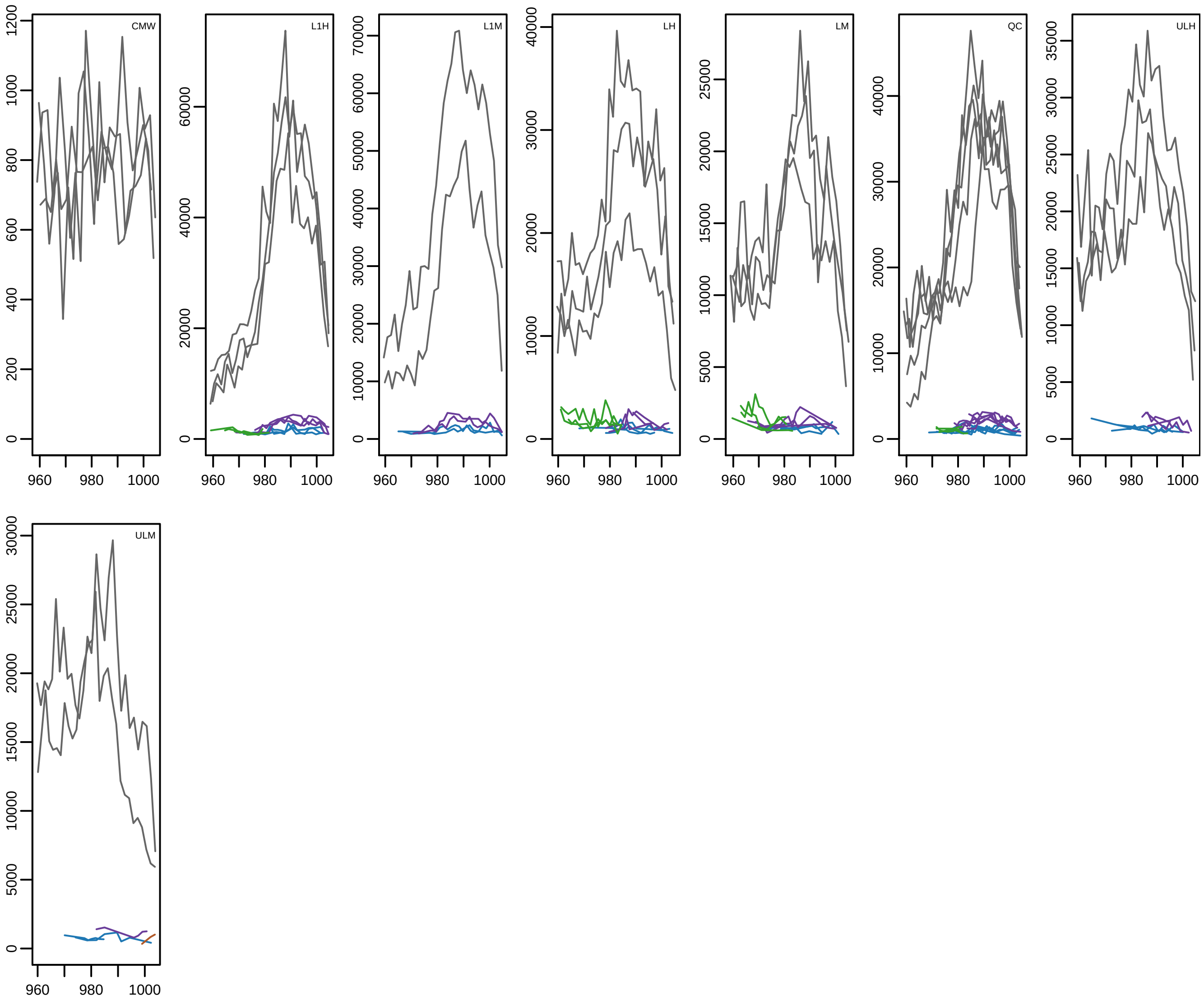

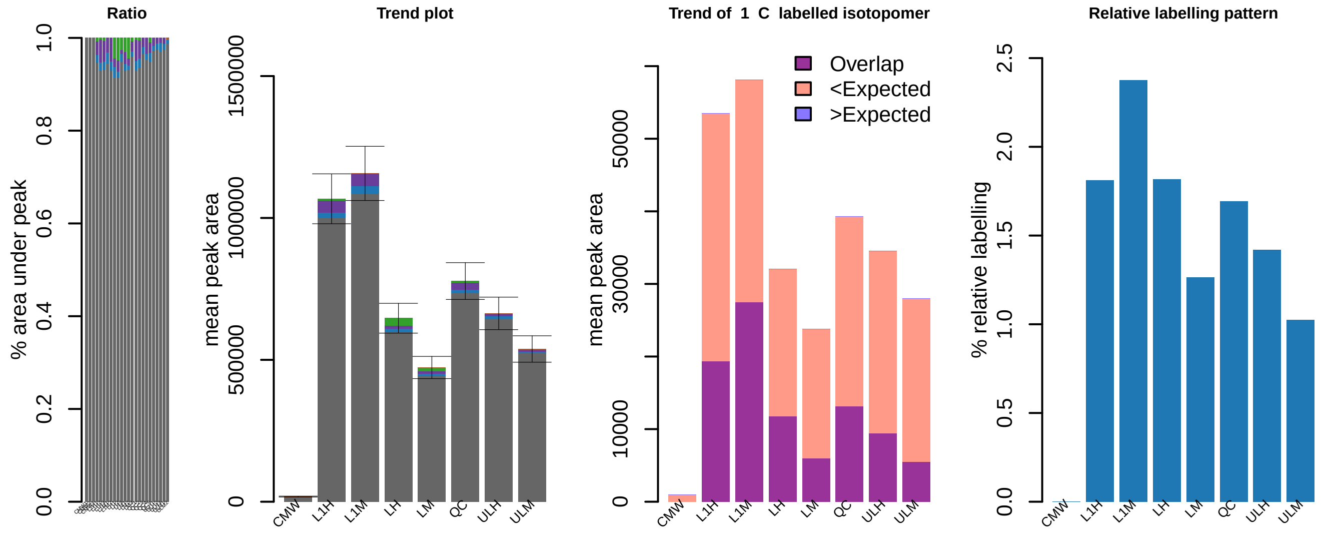

16−hydroxypalmitate

Formula: C16H32O3 Mass: 272.235 Std.RT: 213.50387568 Ion: NEG

G1

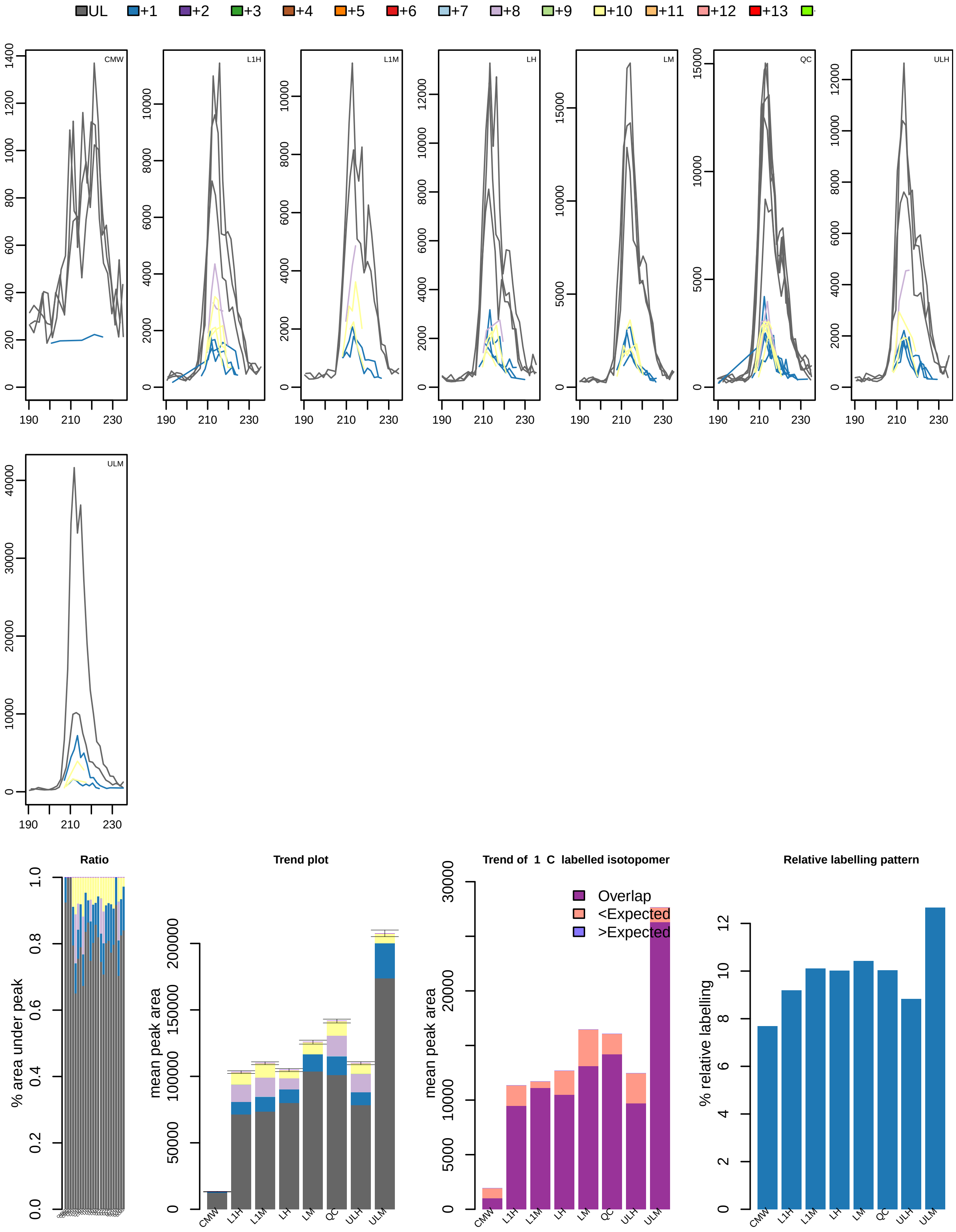

Acetone cyanohydrin

Formula: C4H7NO Mass: 85.053 Std.RT: 961.2041046 Ion: NEG

G1

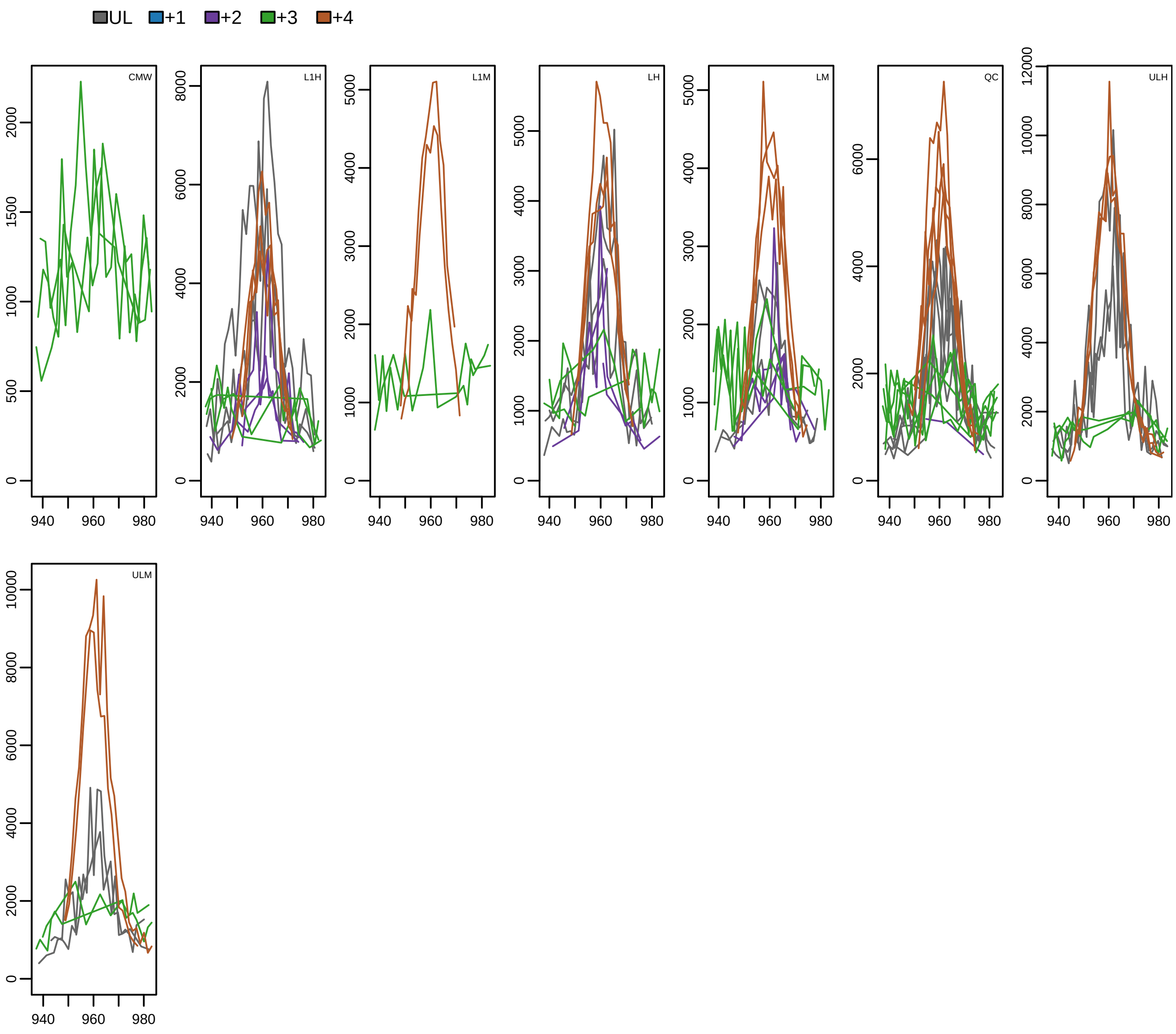

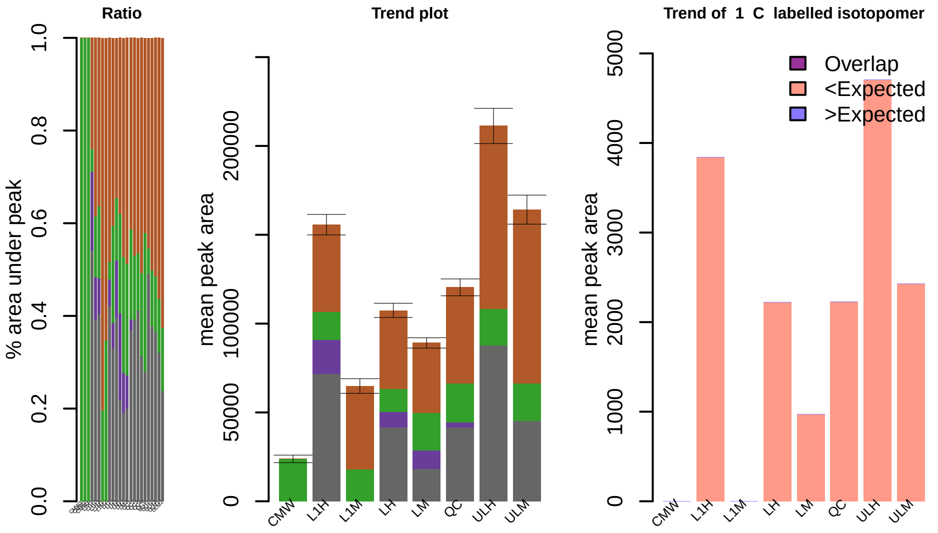

Supplement: S2 Fig — (PDF) [file ppat.1004689.s008.pdf]
