# Supplementary material for: Probing the Metabolic Network in Bloodstream-Form Trypanosoma brucei Using Untargeted Metabolomics with Stable Isotope Labelled Glucose
Source: PLoS Pathog. 2015 Mar 16;11(3):e1004689. doi: 10.1371/journal.ppat.1004689 (PMC4361558; doi:10.1371/journal.ppat.1004689)

Homoarginine

G1

Formula: C7H16N4O2 Mass: 188.127 Std.RT: 1451.0716602 Ion: PC

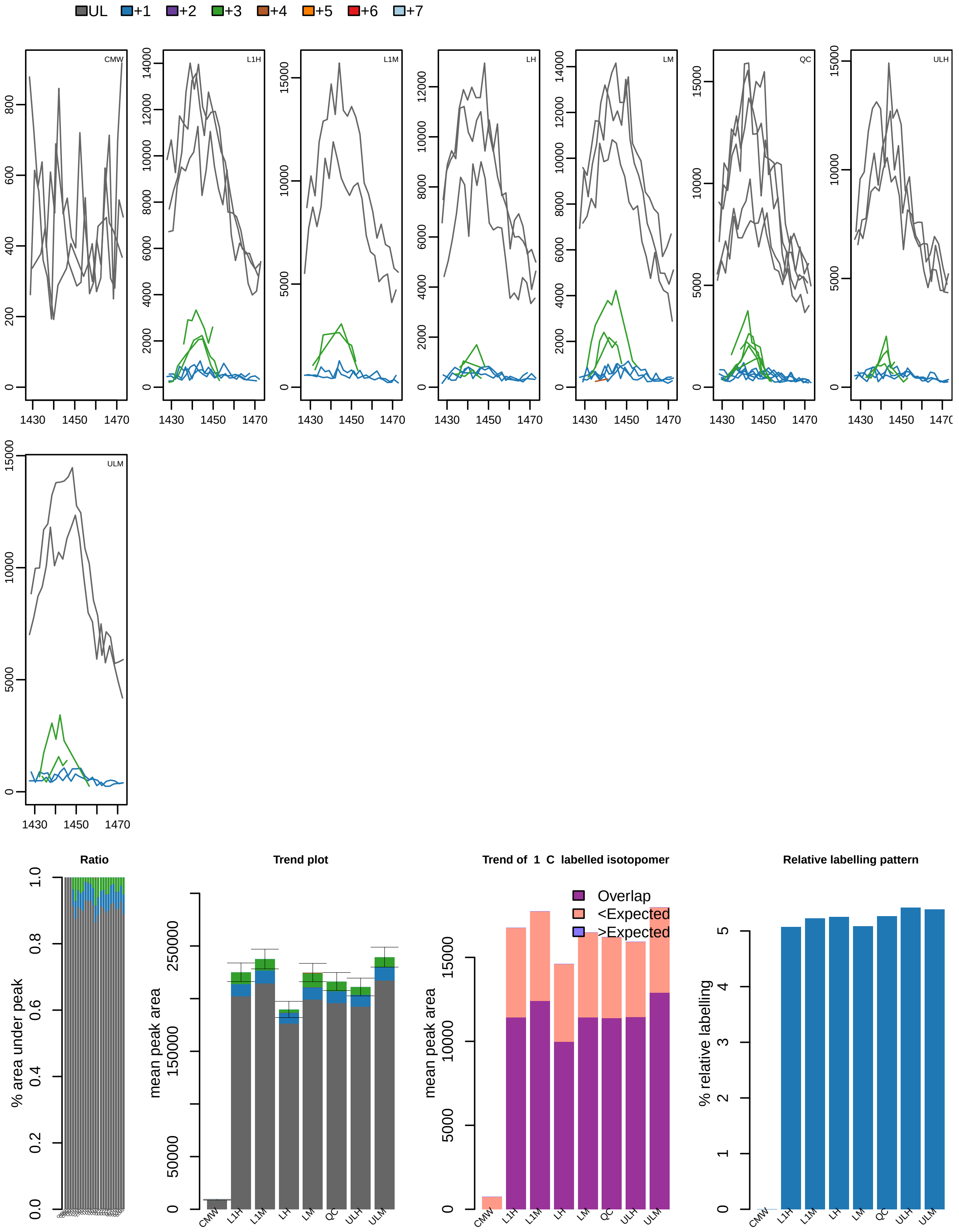

N6−Methyl−L−lysine

Formula: C7H16N2O2 Mass: 160.121 Std.RT: 1504.6417398 Ion: PC

G1

UL +1 +2 +3 +4 +5 +6 +7

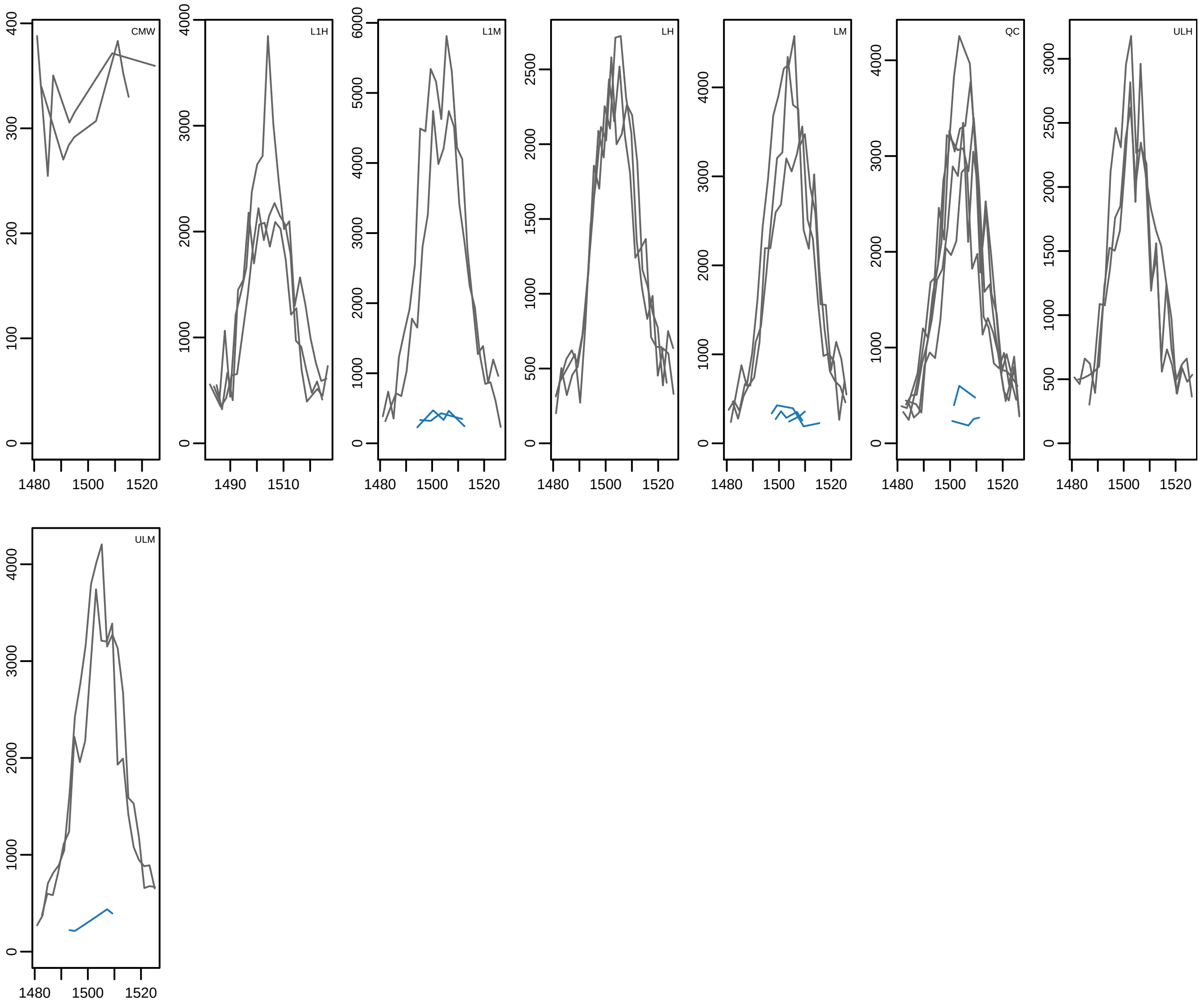

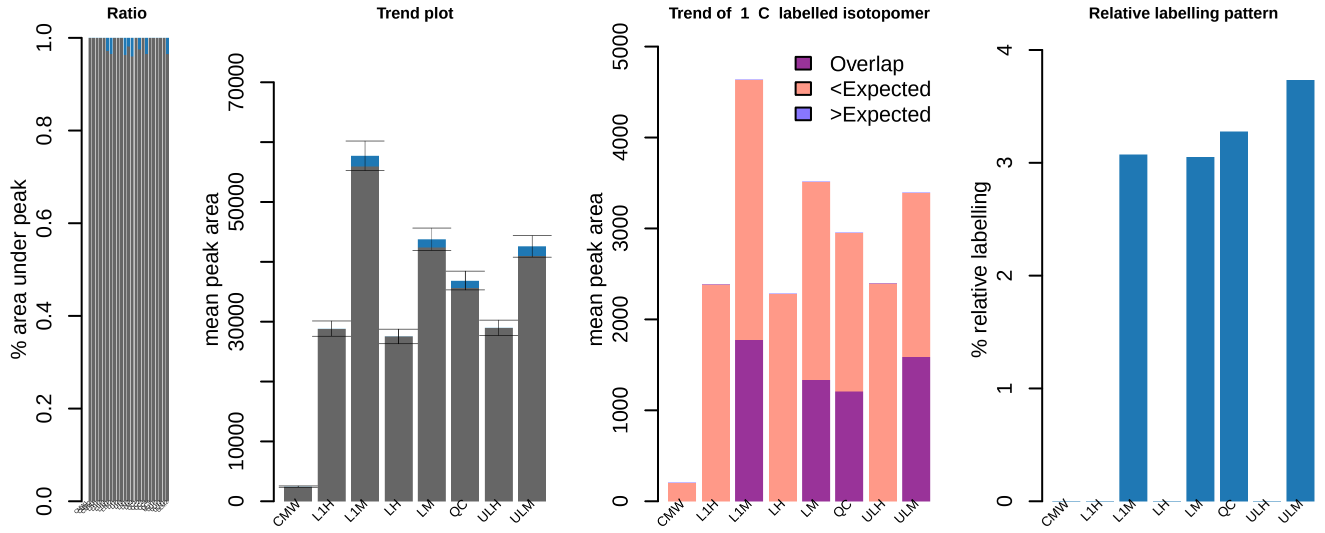

L−Lysine 1,6−lactam

Formula: C6H12N2O Mass: 128.095 Std.RT: 774.1562526 Ion: POS

G1

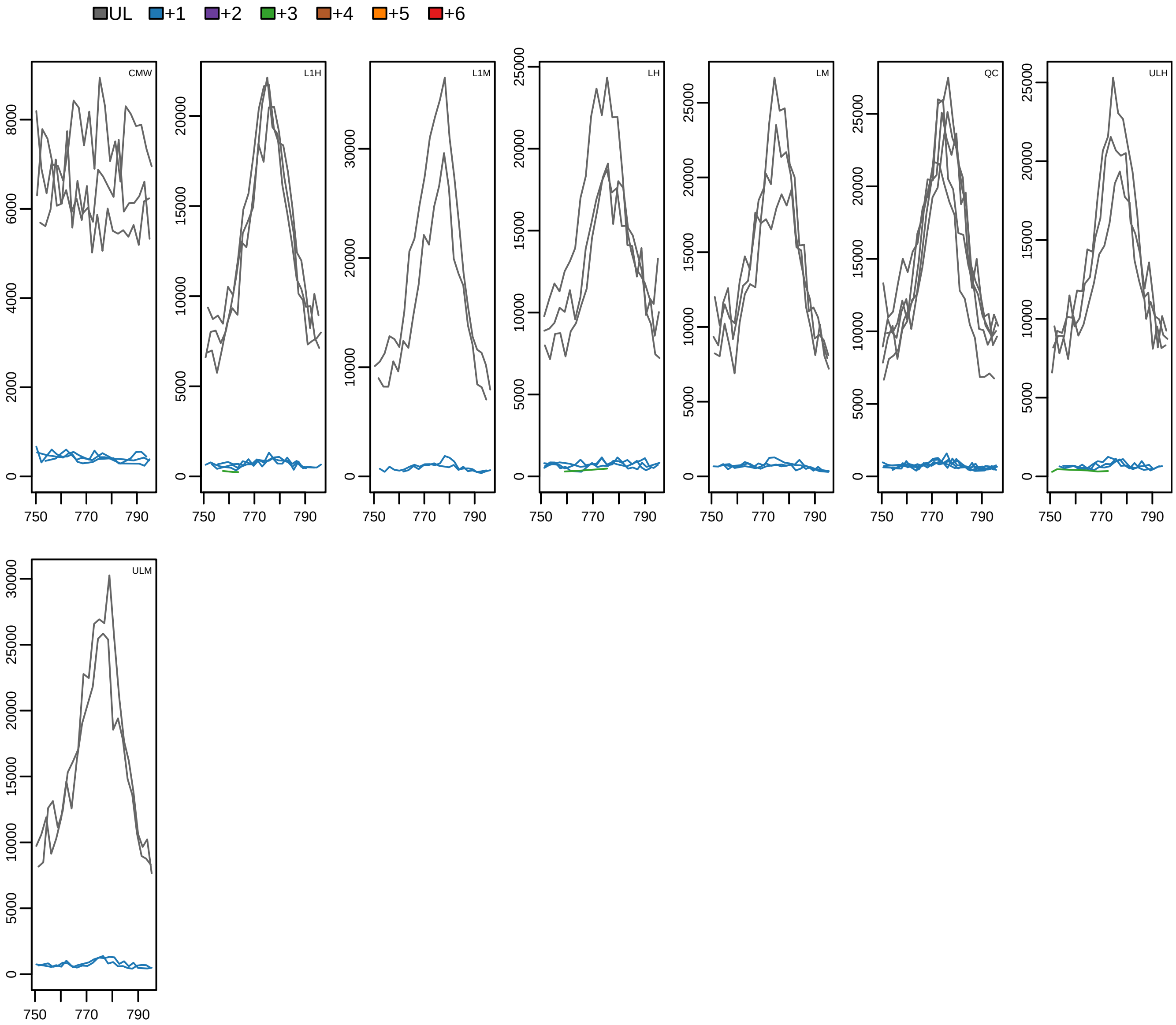

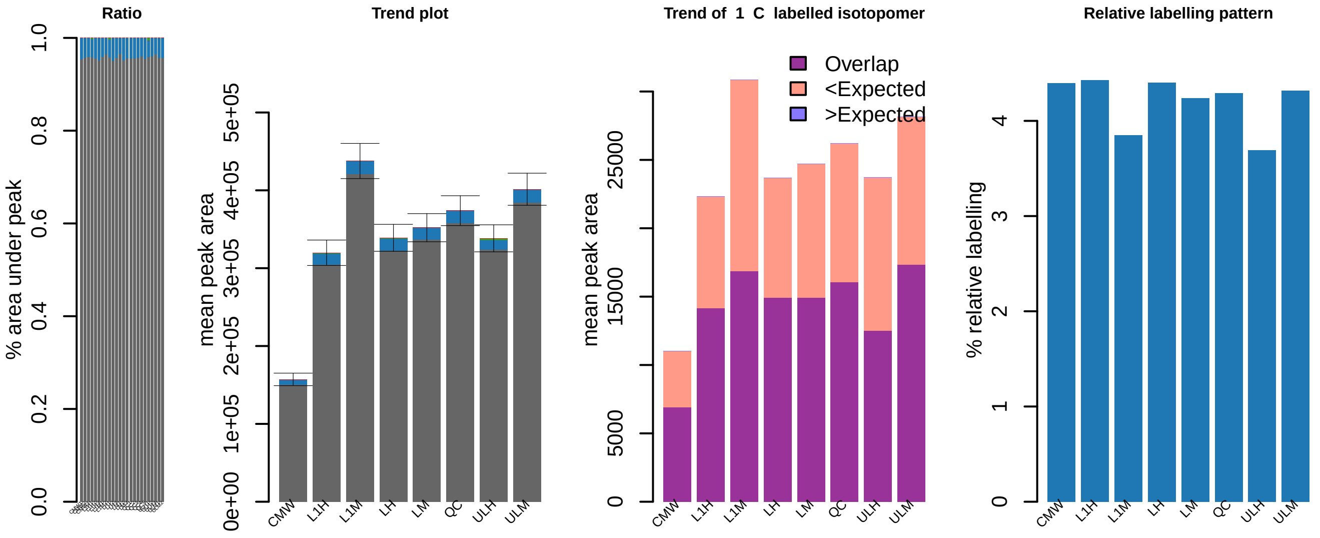

CysteineMercaptoethanol disulfide

Formula: C5H11NO3S2 Mass: 197.018 Std.RT: 712.2511482 Ion: PC

G1

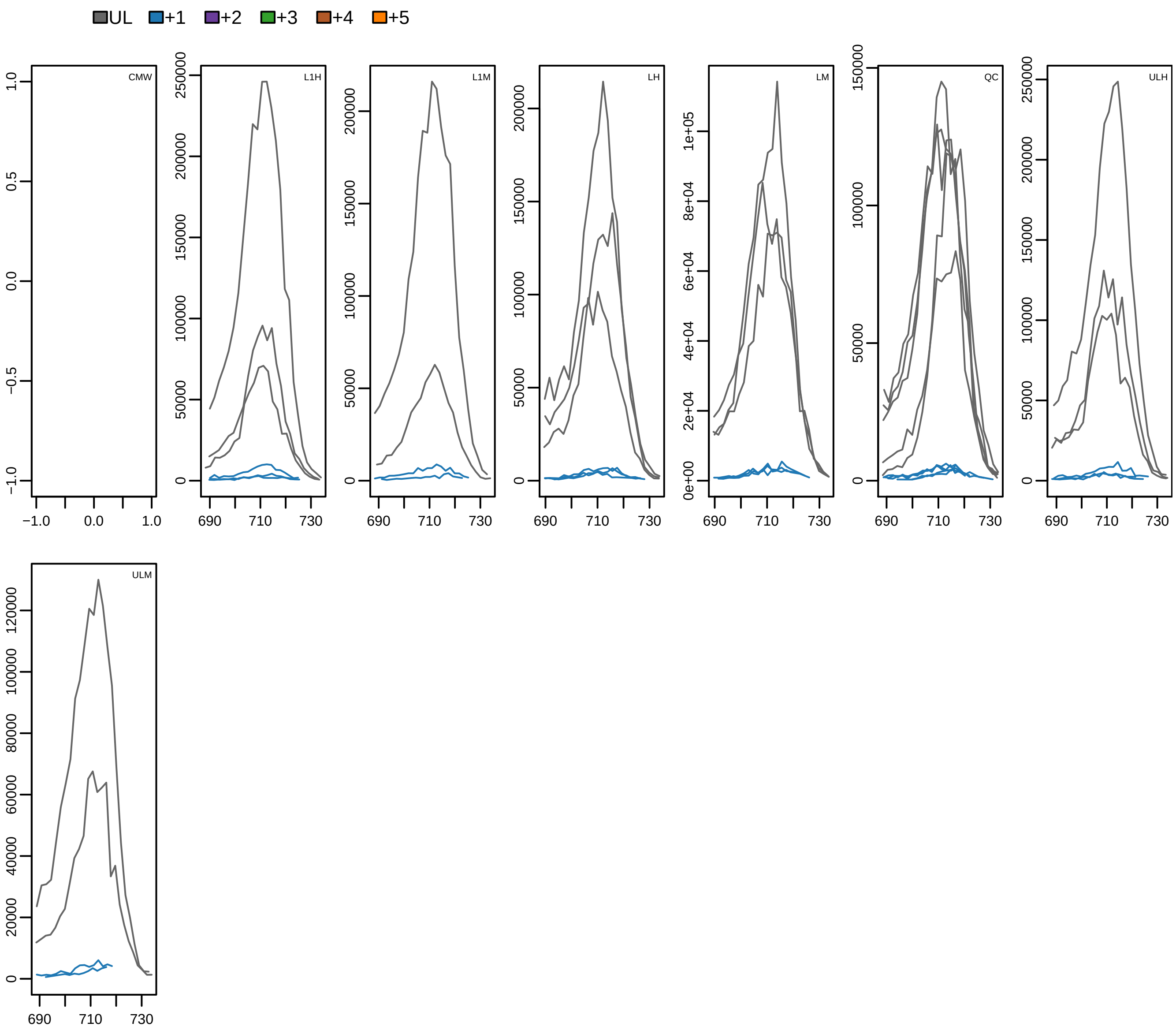

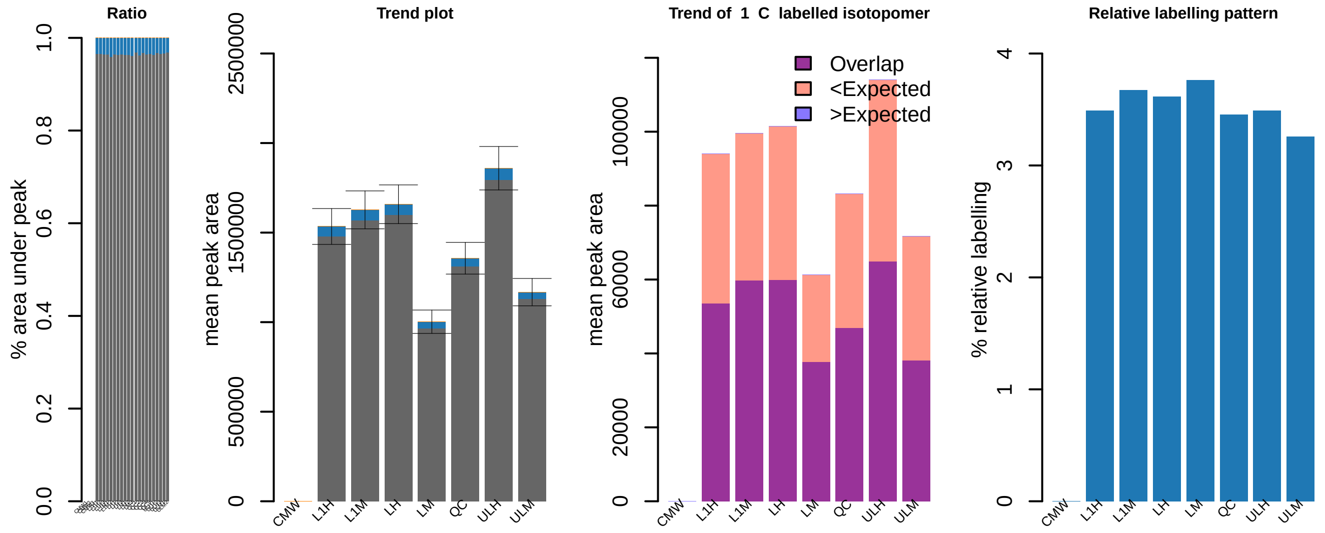

O−Acetylcarnitine

G1

Formula: C9H17NO4 Mass: 203.116 Std.RT: 710.9115018 Ion: POS

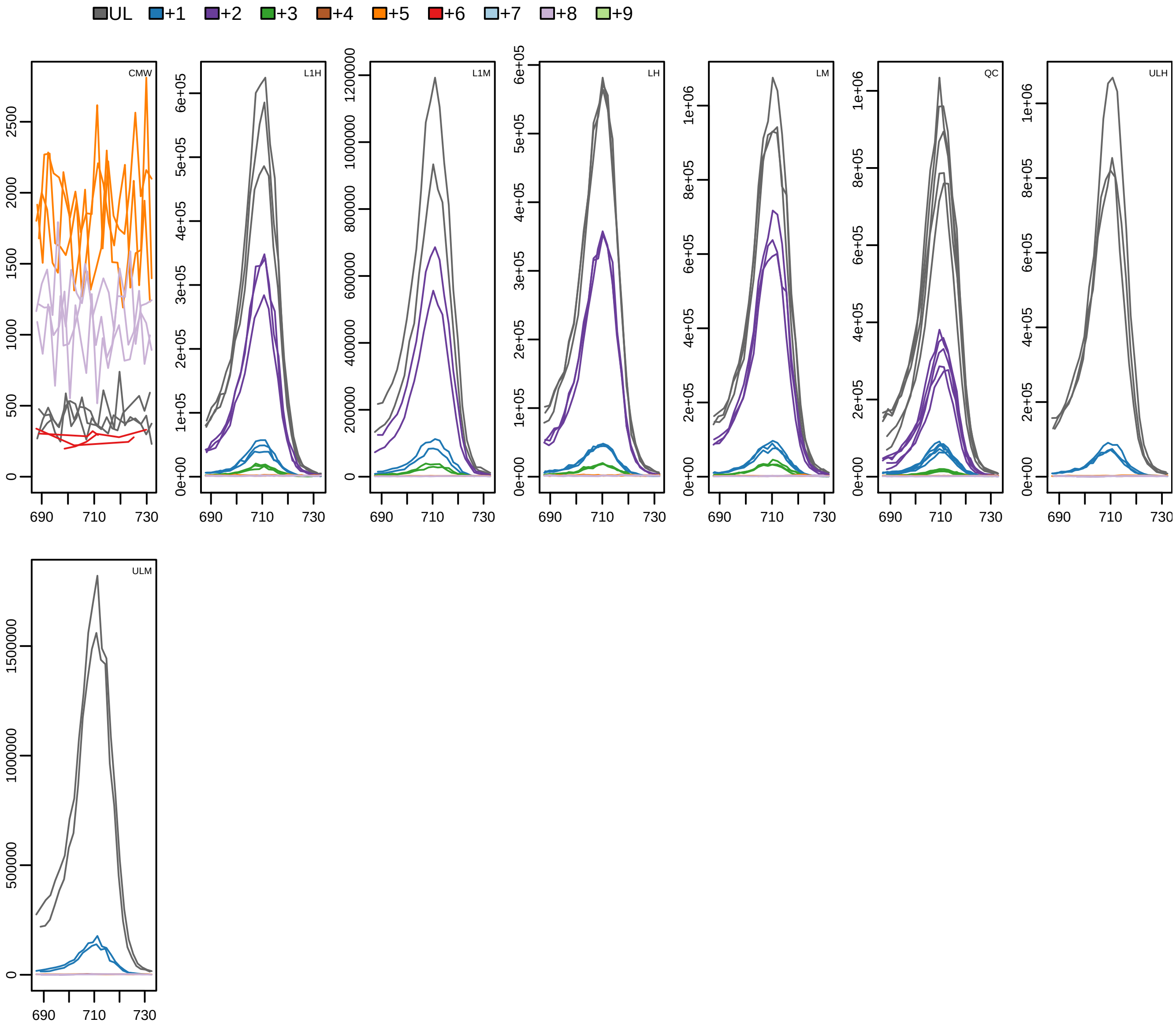

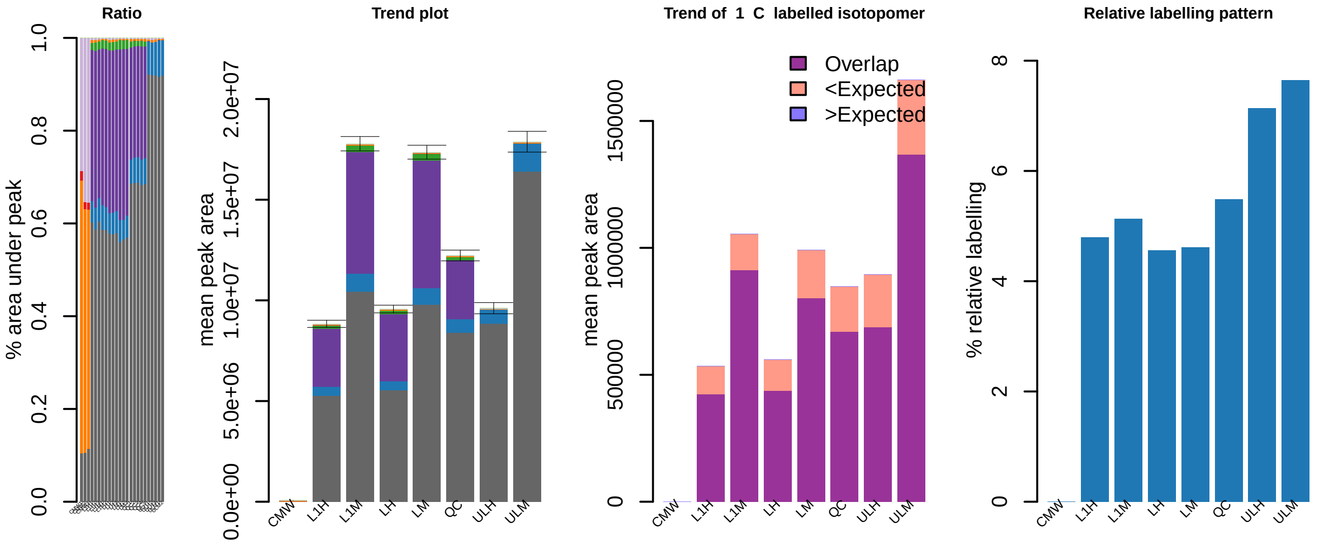

L−Alanine

Formula: C3H7NO2 Mass: 89.048 Std.RT: 959.4942474 Ion: POS

G1

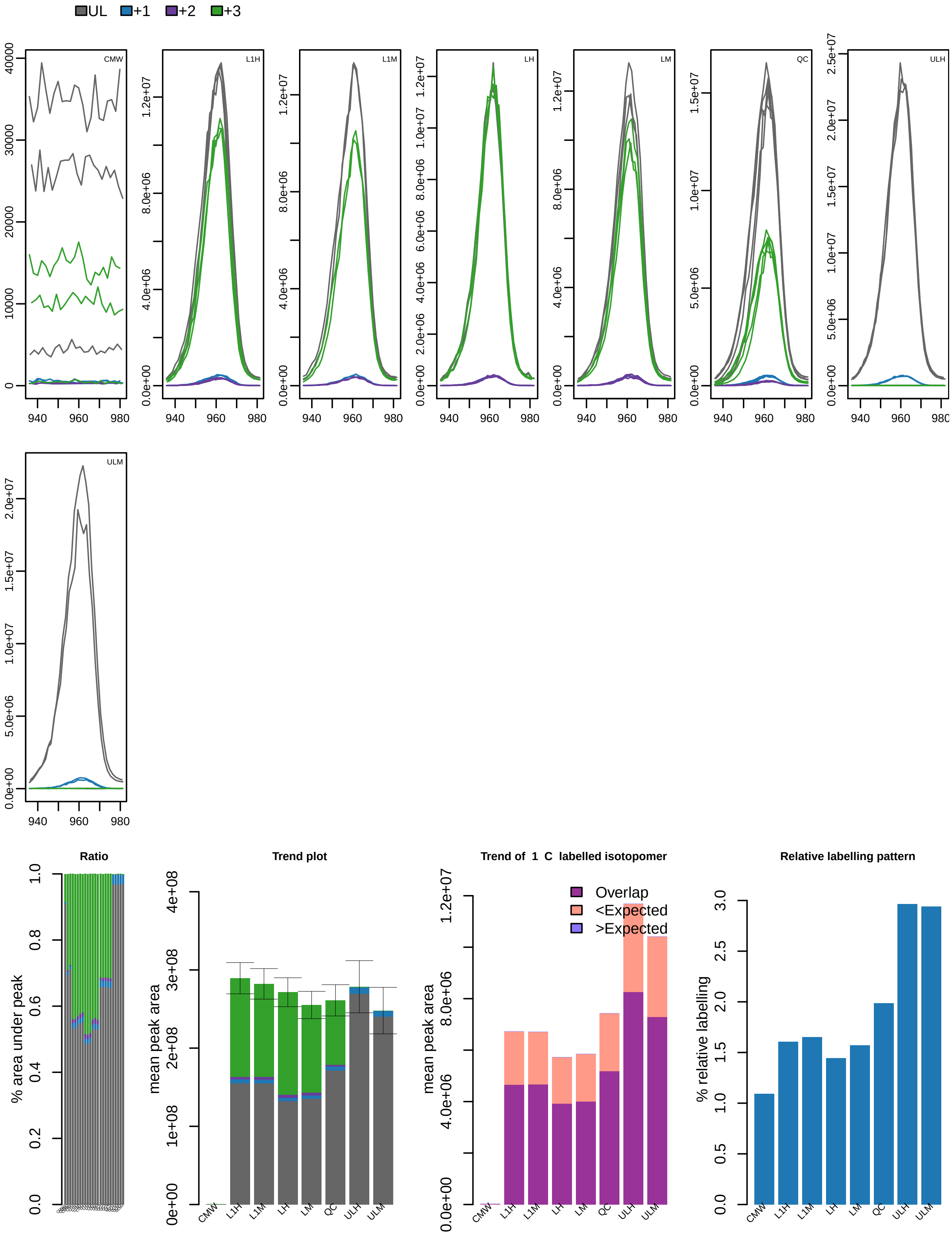

Carnosine

G1

Formula: C9H14N4O3 Mass: 226.107 Std.RT: 696.8317206 Ion: POS

UL +1 +2 +3 +4 +5 +6 +7 +8 +9

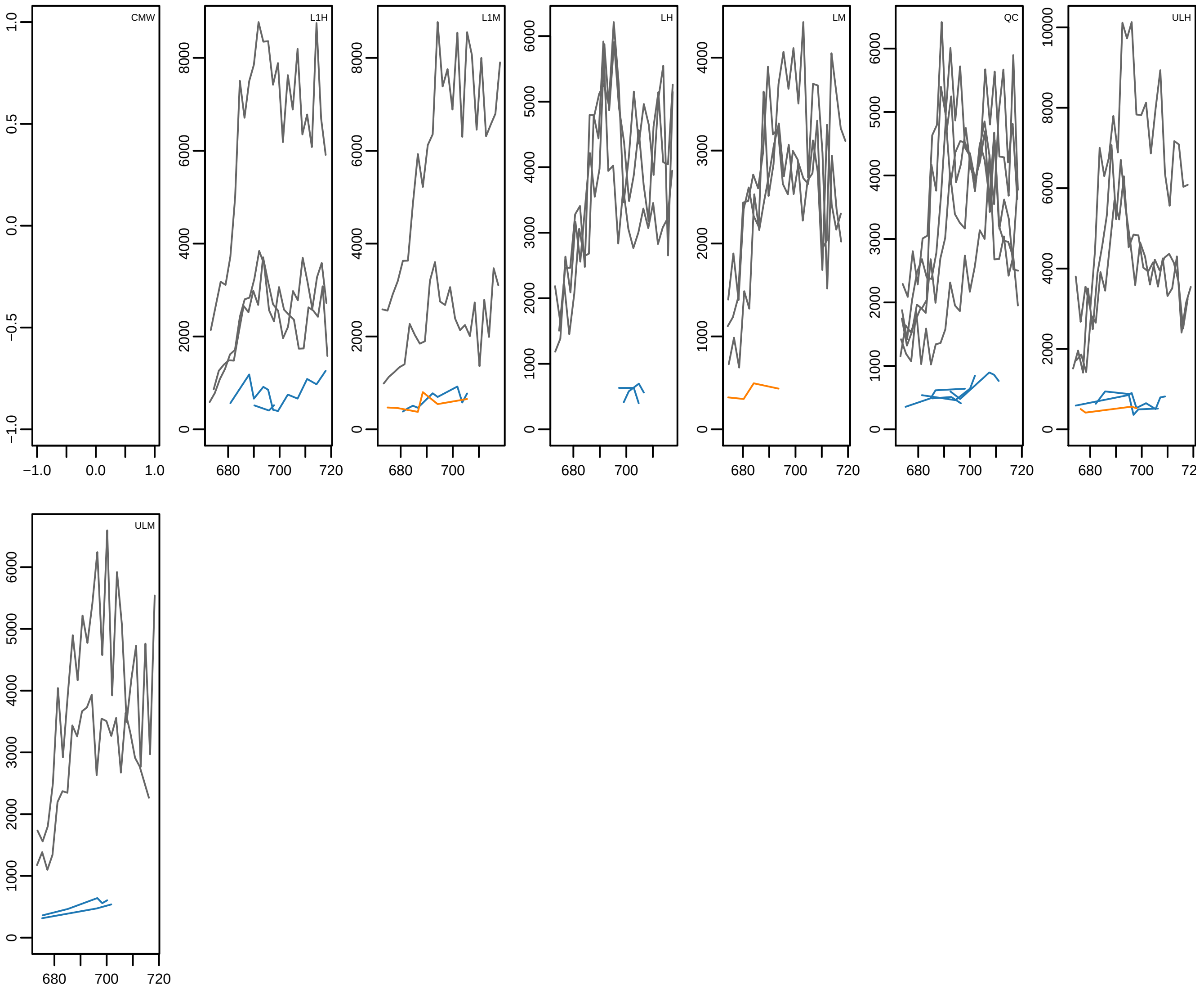

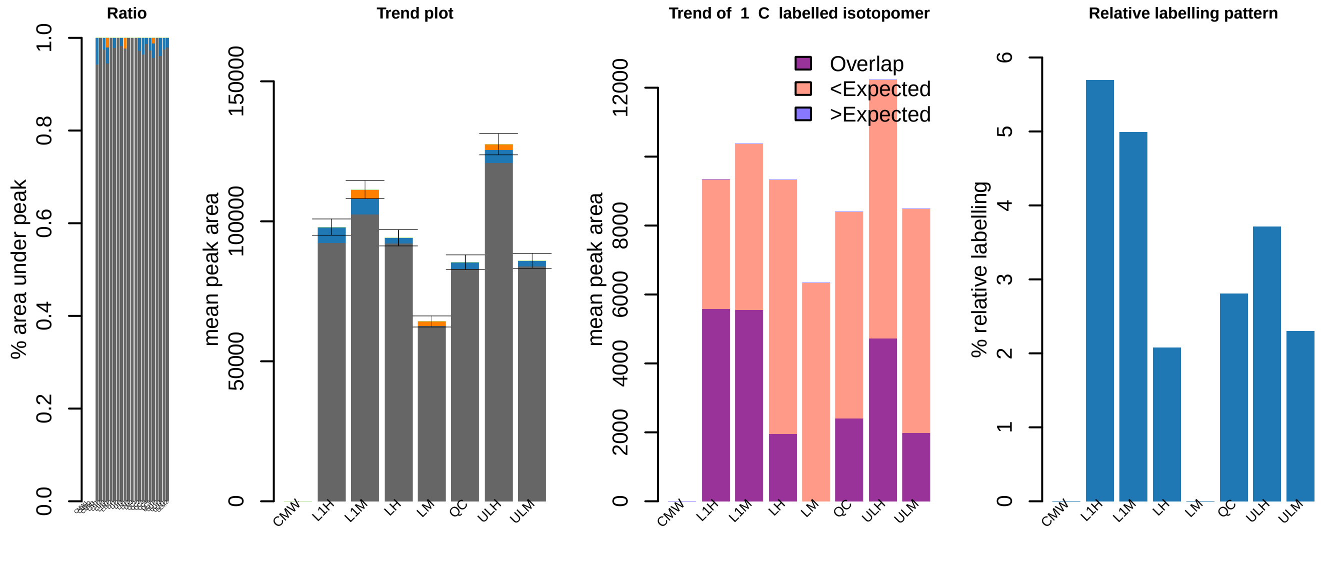

L−Asparagine

Formula: C4H8N2O3 Mass: 132.053 Std.RT: 984.7294284 Ion: POS

G1

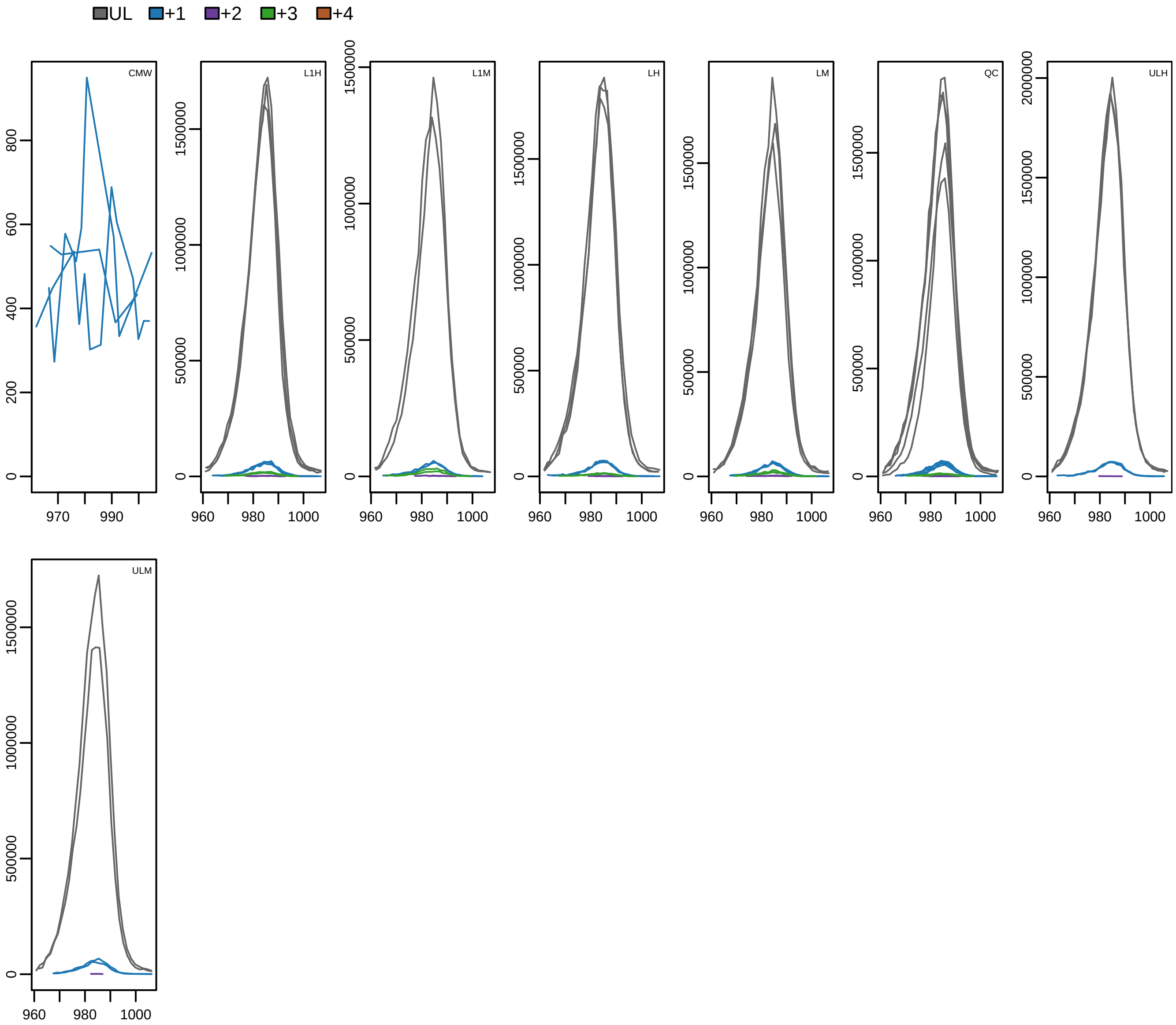

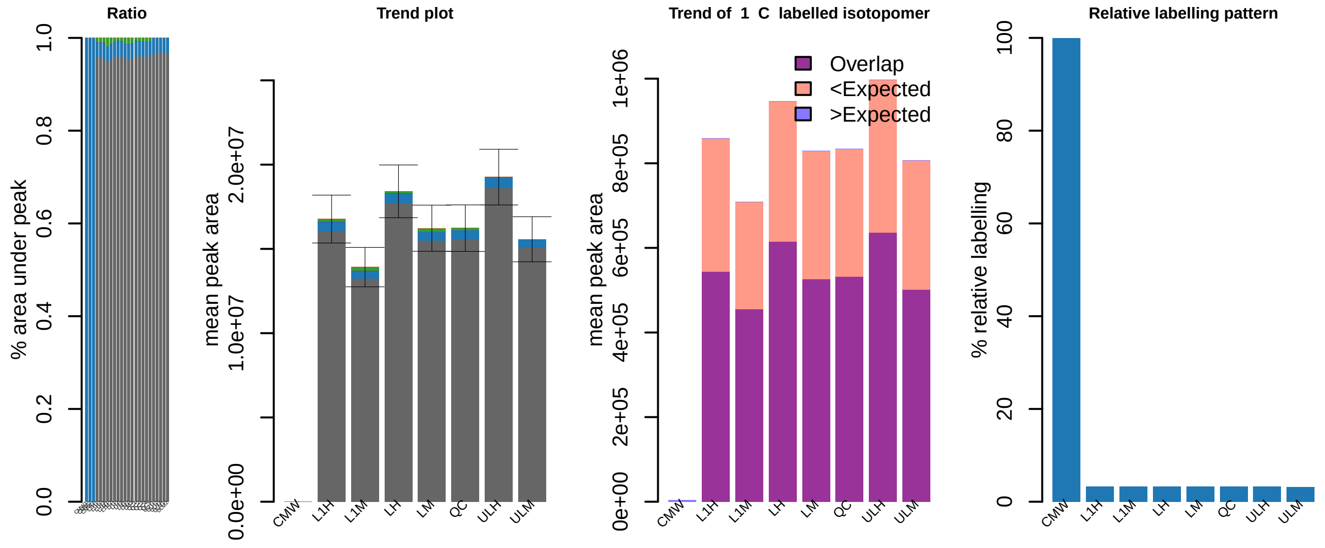

4−Acetamidobutanoate

Formula: C6H11NO3 Mass: 145.074 Std.RT: 487.06949742 Ion: POS

G1

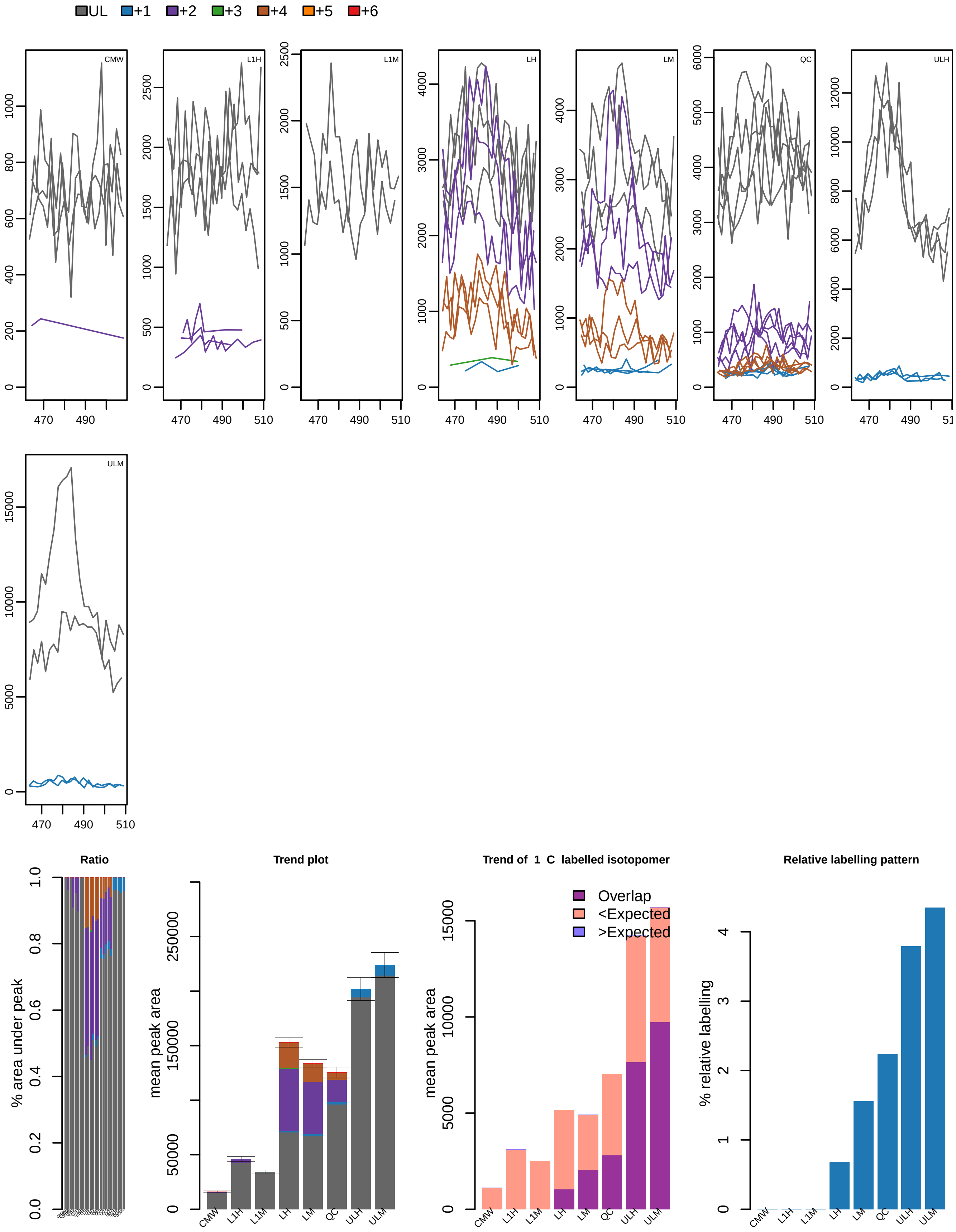

4−Acetamidobutanoate

Formula: C6H11NO3 Mass: 145.074 Std.RT: 487.06949742 Ion: POS

G2

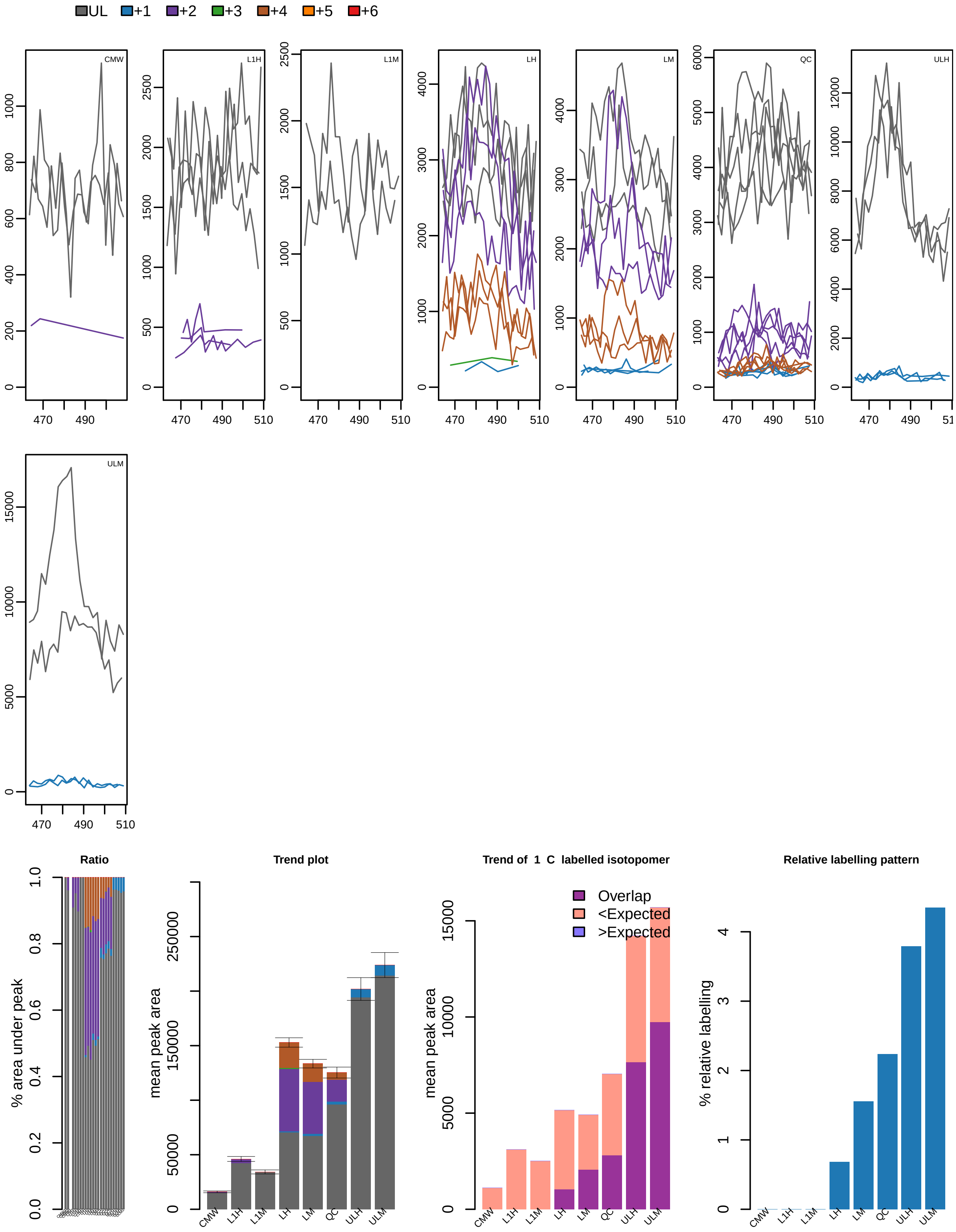

N2−(D−1−Carboxyethyl)−L−arginine

Formula: C9H18N4O4 Mass: 246.133 Std.RT: 1113.18954 Ion: POS

G1

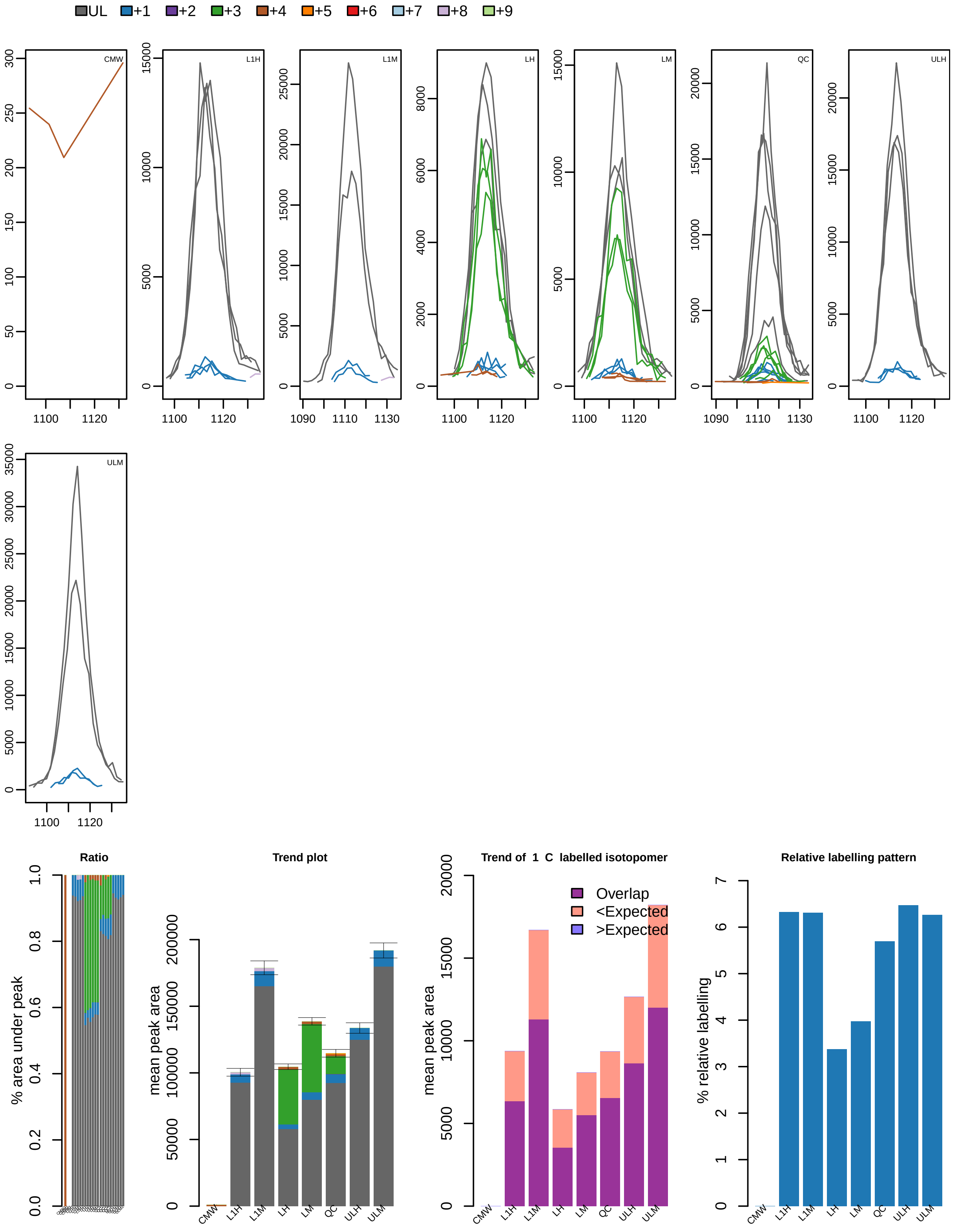

N−Acetylornithine

Formula: C7H14N2O3 Mass: 174.1 Std.RT: 874.6892418 Ion: POS

G1

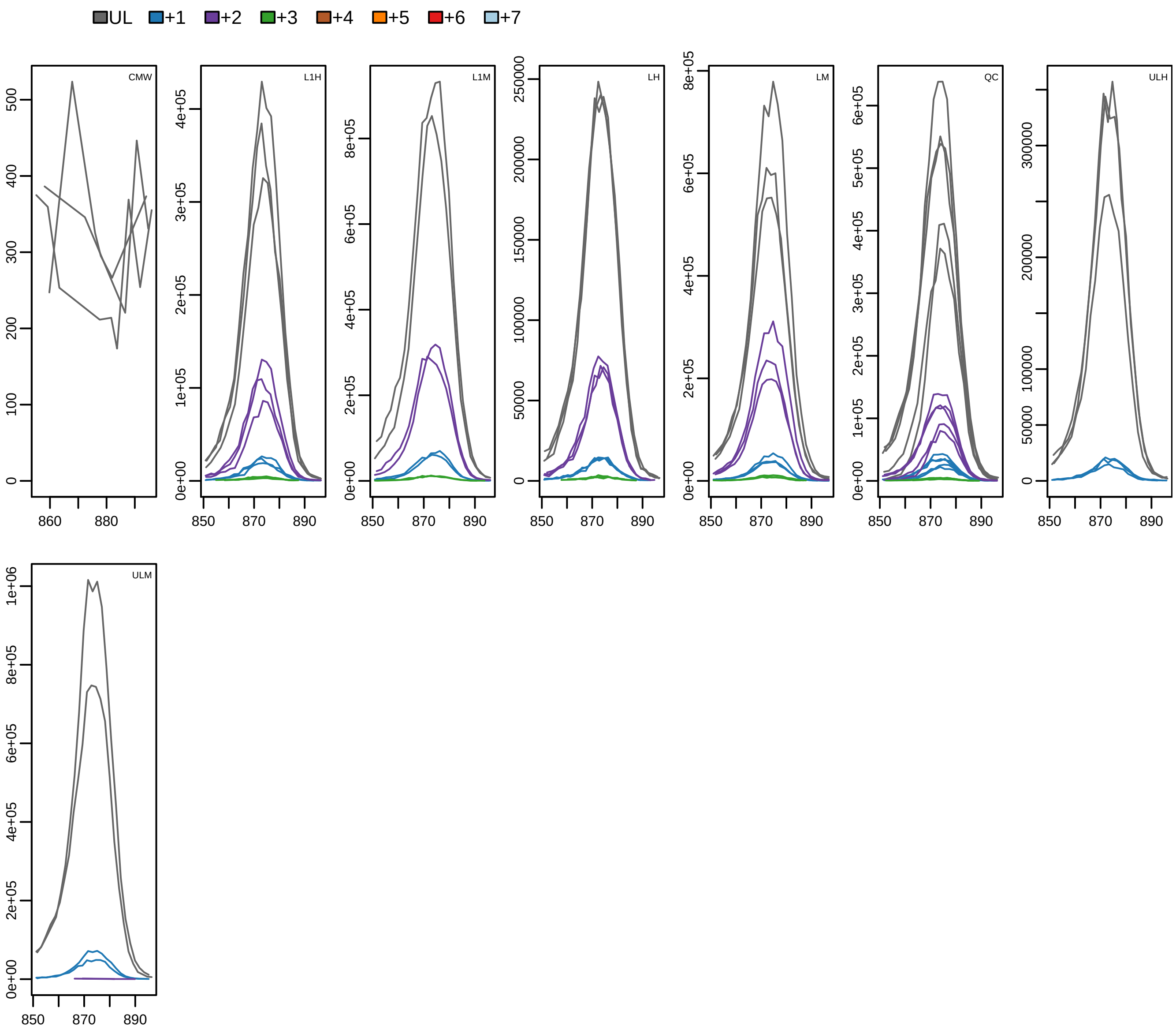

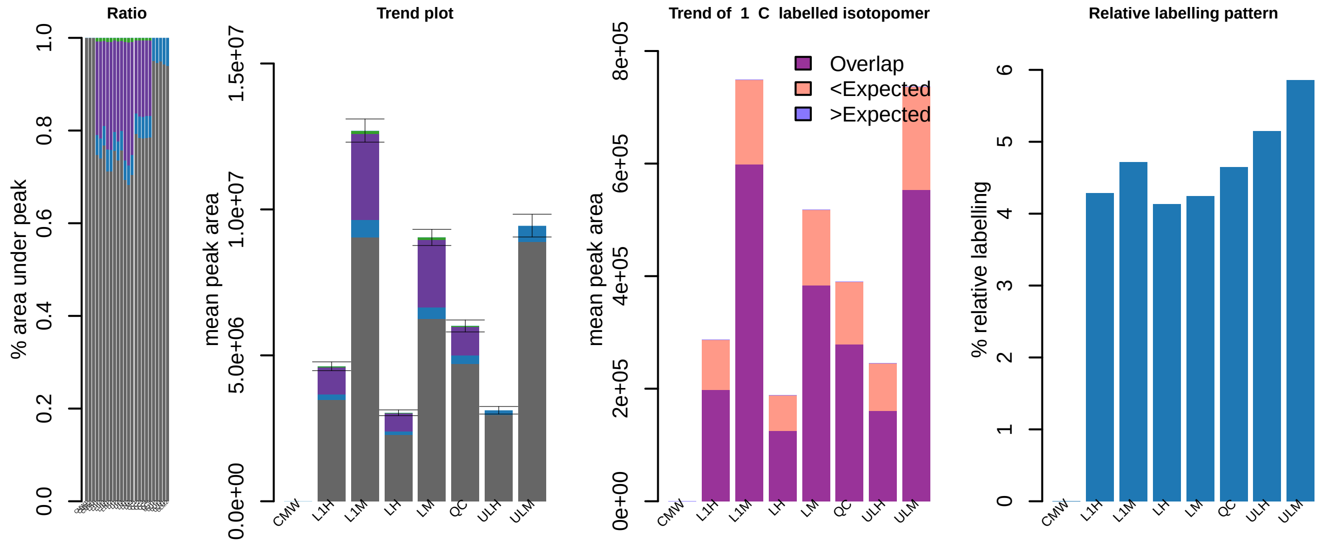

gamma−Glutamyl−gamma−aminobutyraldehyde

Formula: C9H16N2O4 Mass: 216.111 Std.RT: 722.9132514 Ion: POS

G1

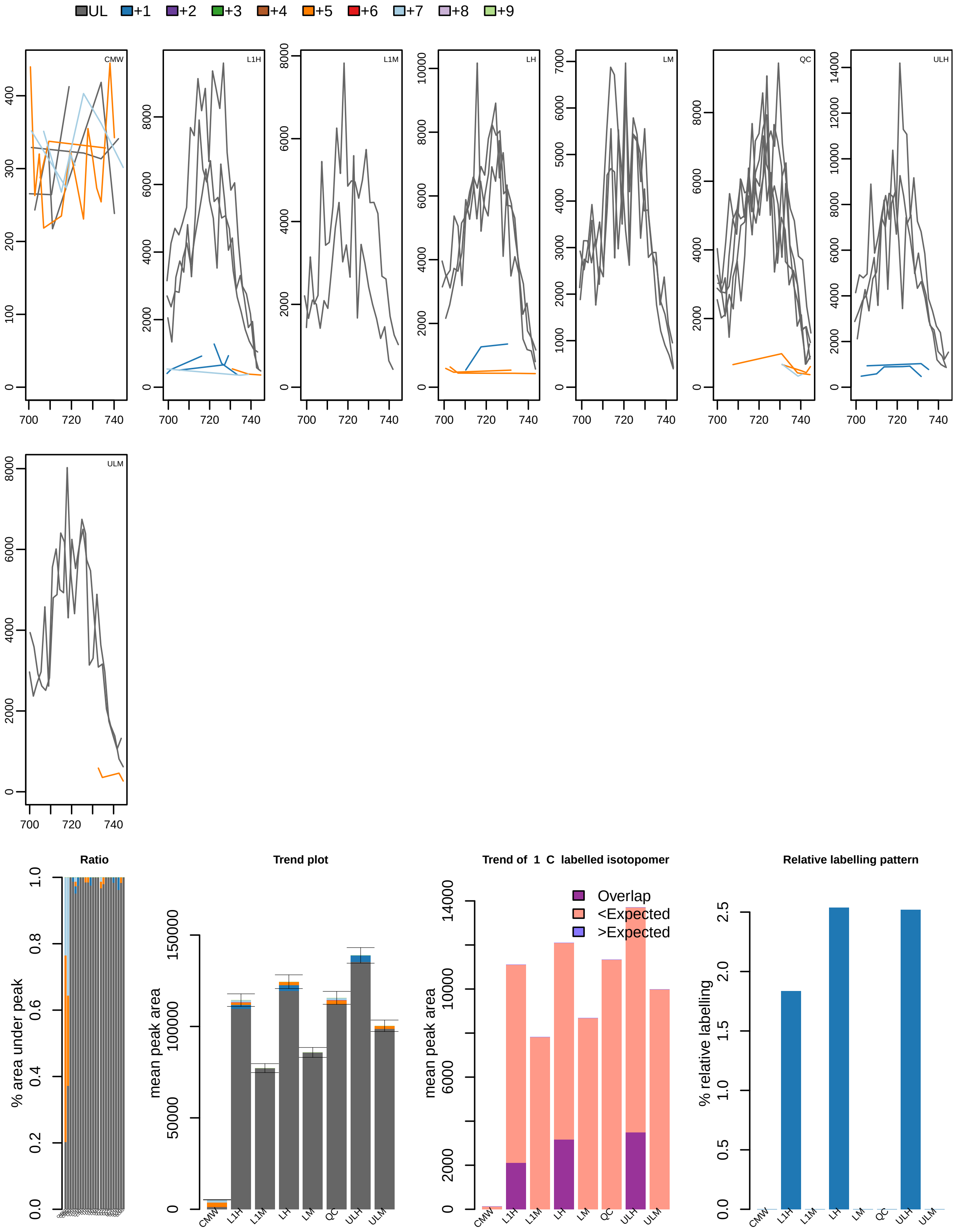

L−Glutamate 5−semialdehyde

Formula: C5H9NO3 Mass: 131.058 Std.RT: 937.8152442 Ion: POS

G1

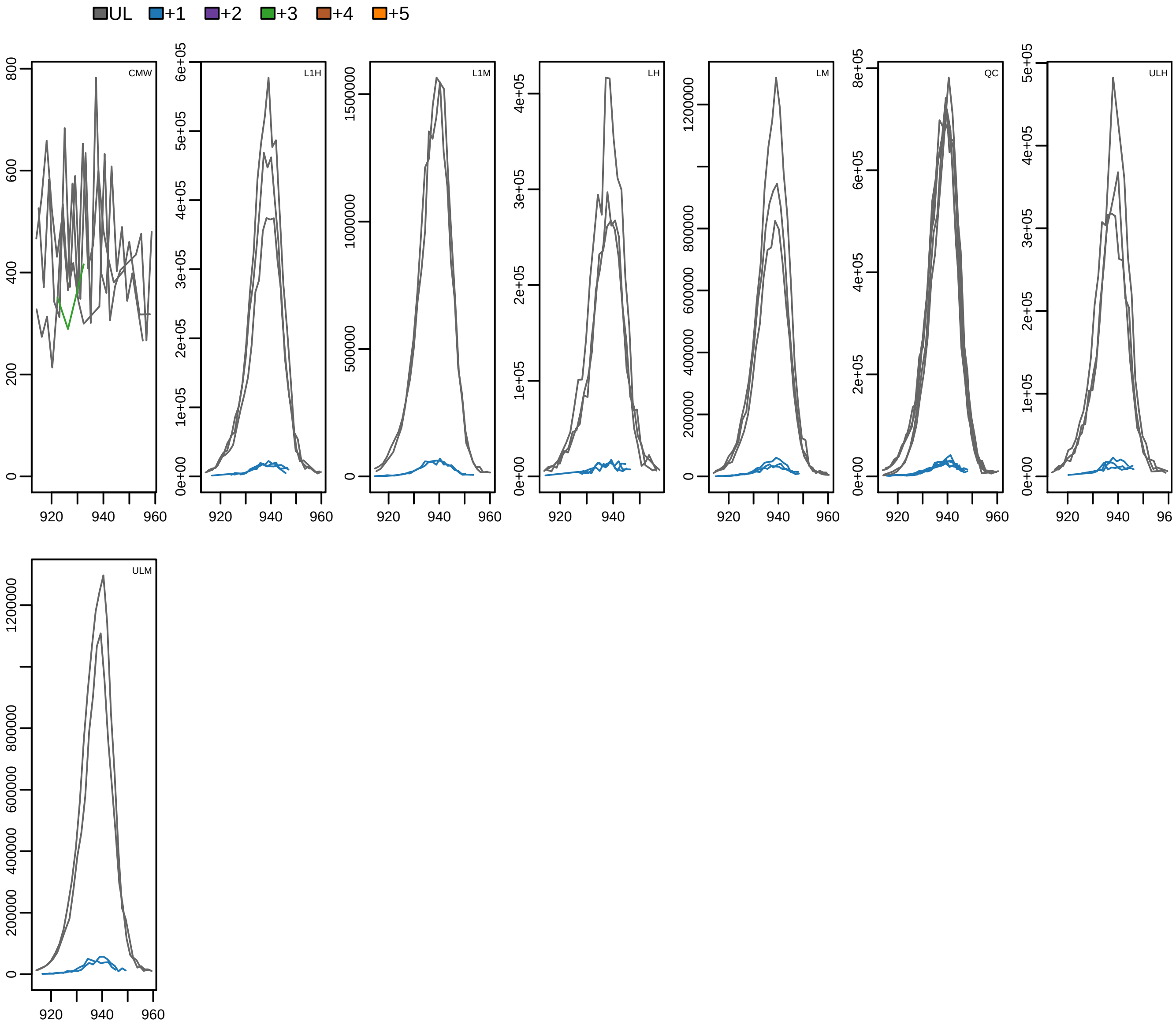

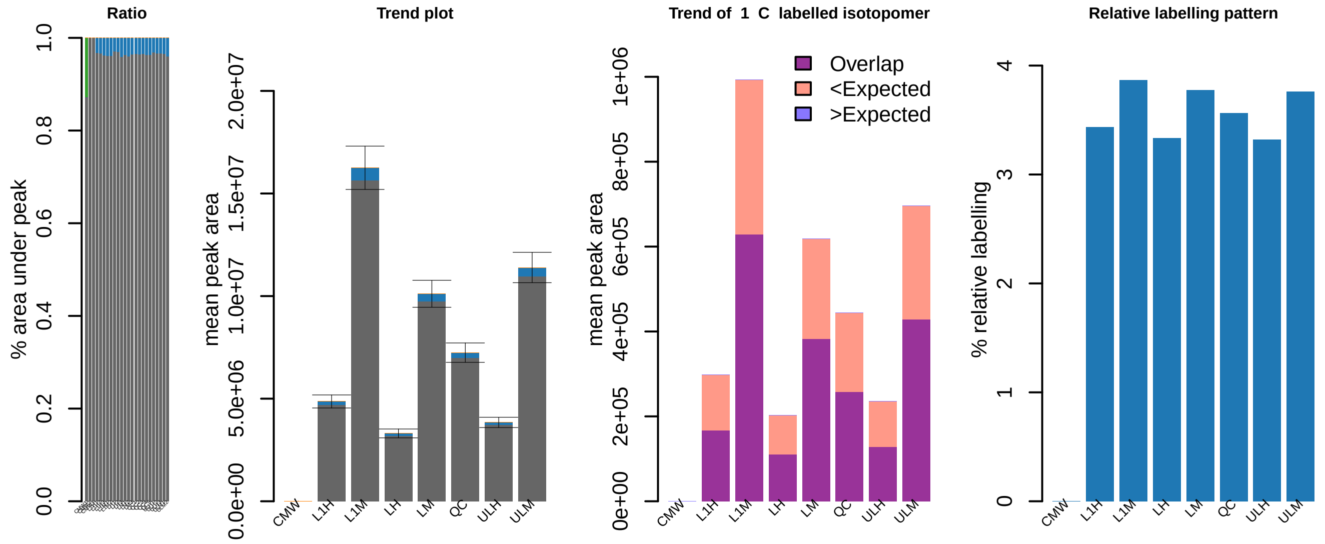

N4−Acetylaminobutanal

Formula: C6H11NO2 Mass: 129.079 Std.RT: 432.94133382 Ion: POS

G1

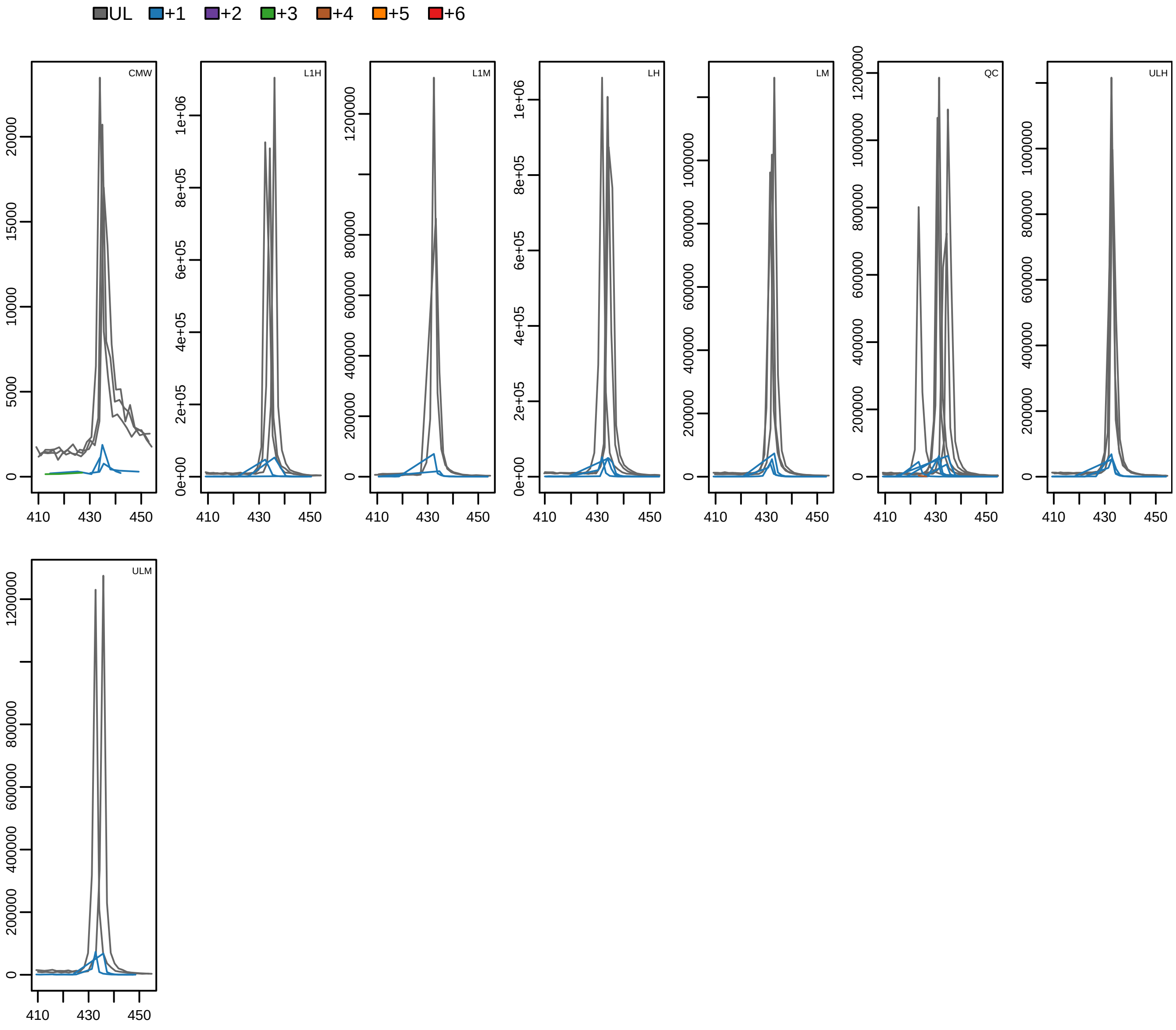

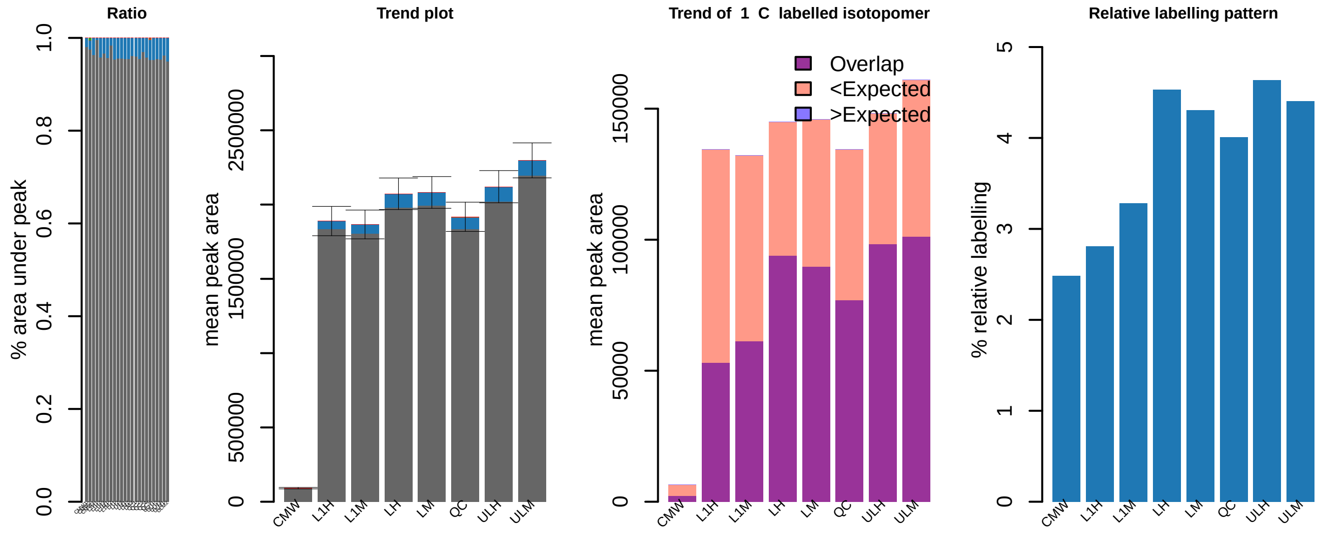

Creatinine

Formula: C4H7N3O Mass: 113.059 Std.RT: 599.92988082 Ion: POS

G1

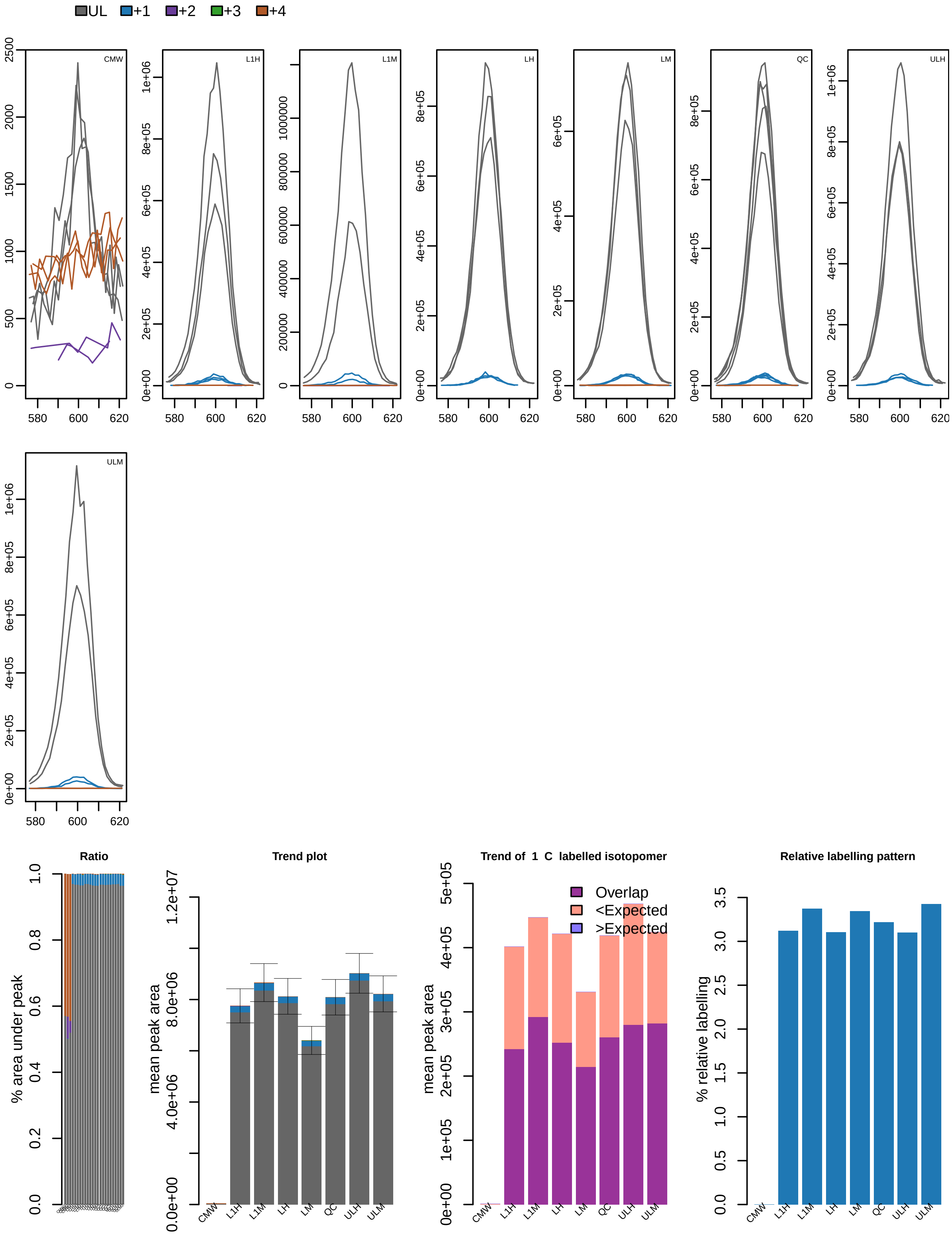

4−Guanidinobutanoate

G1

Formula: $C_5H_{11}N_3O_2$ Mass: 145.085 Std.RT: 992.1194994 Ion: POS

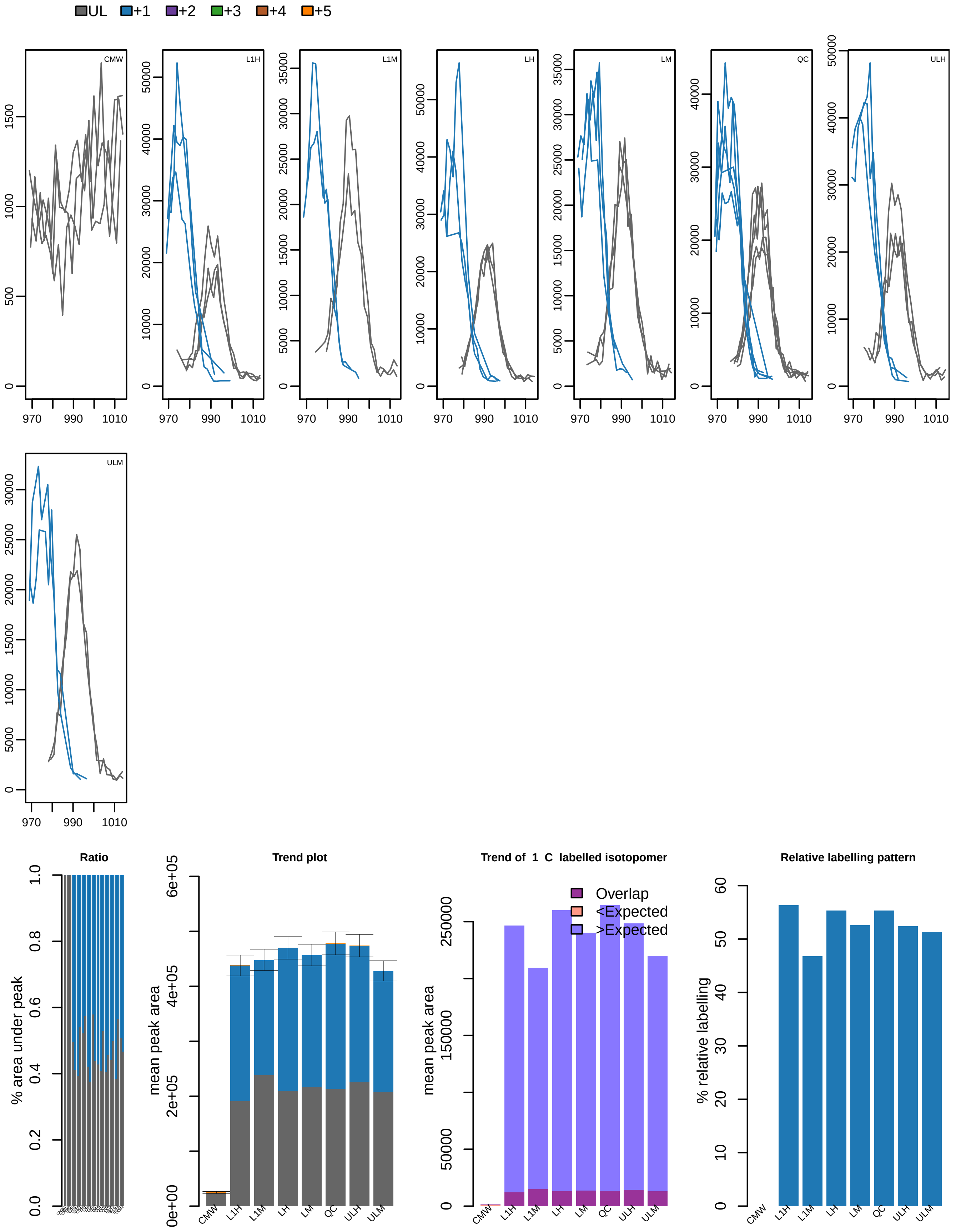

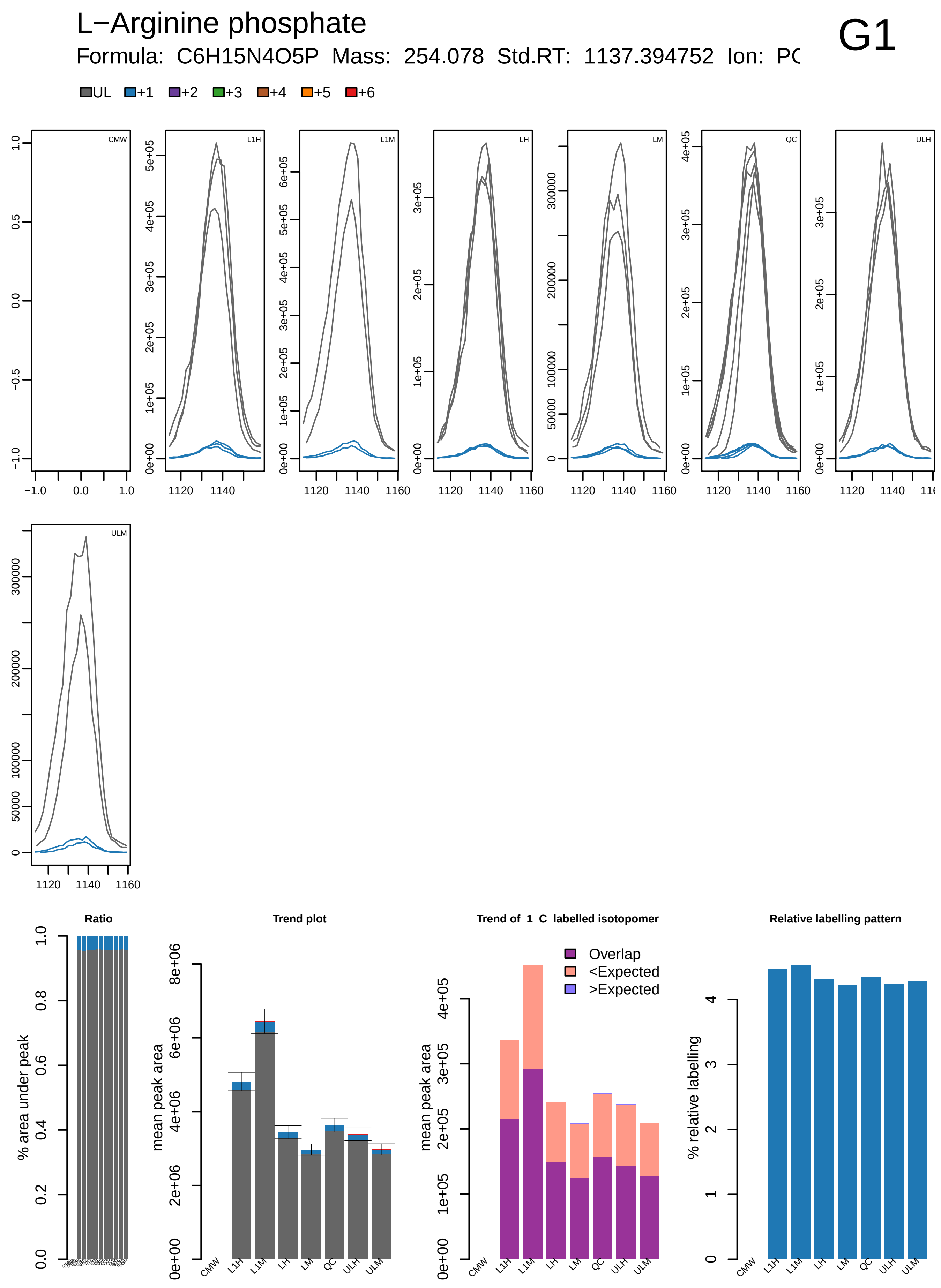

L−Arginine phosphate
Formula: C6H15N4O5P Mass: 254.078 Std.RT: 1137.394752 Ion: PC
G1
UL +1 +2 +3 +4 +5 +6
CMW
L1H
L1M
LH
LM
QC
ULH
ULM
Ratio
% area under peak
Trend plot
mean peak area
Trend of 1 C labelled isotopomer
Overlap
<Expected
>Expected
Relative labelling pattern
% relative labelling

N−Acetylputrescine

Formula: C6H14N2O Mass: 130.111 Std.RT: 1182.8579202 Ion: POS

G1

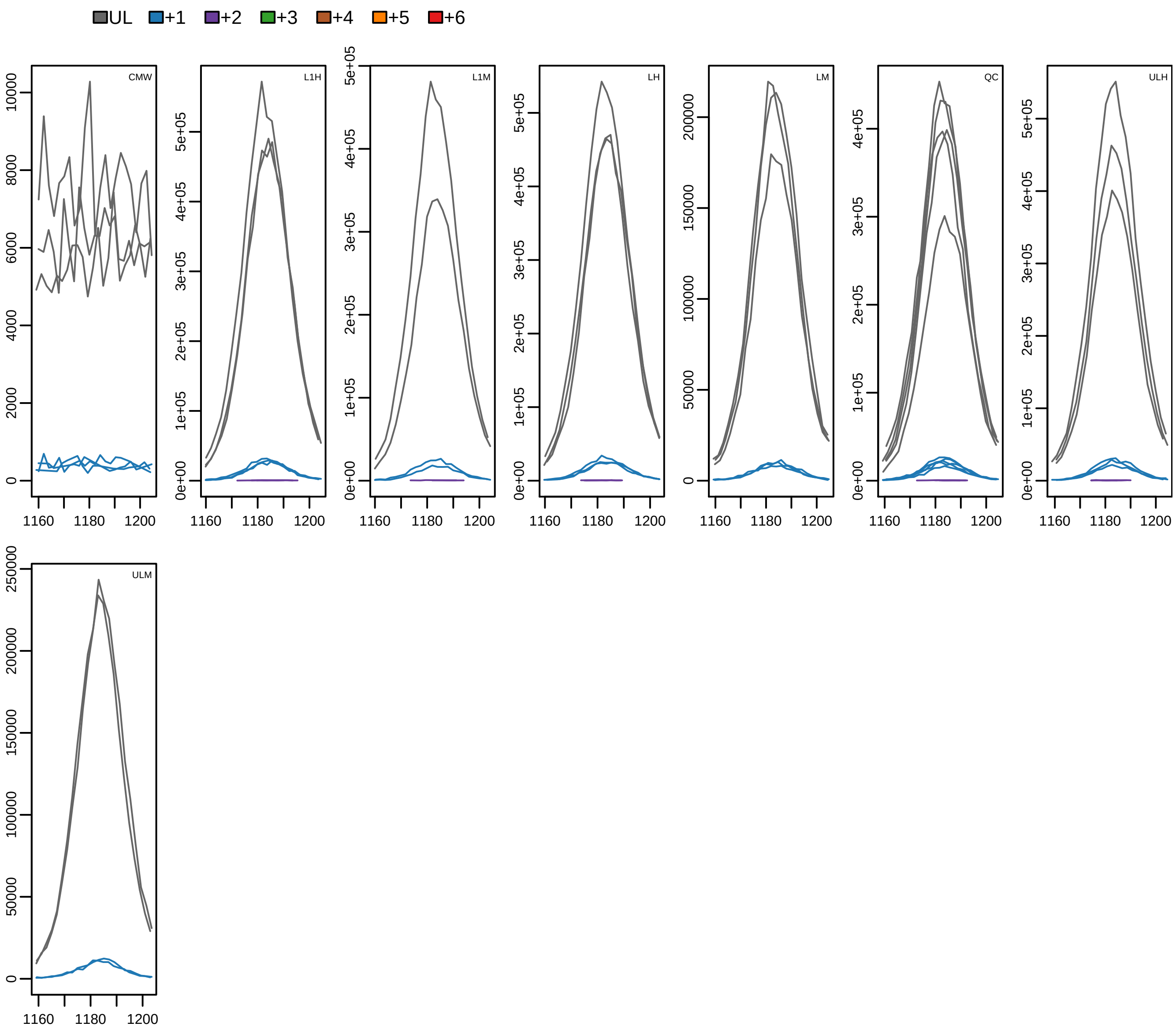

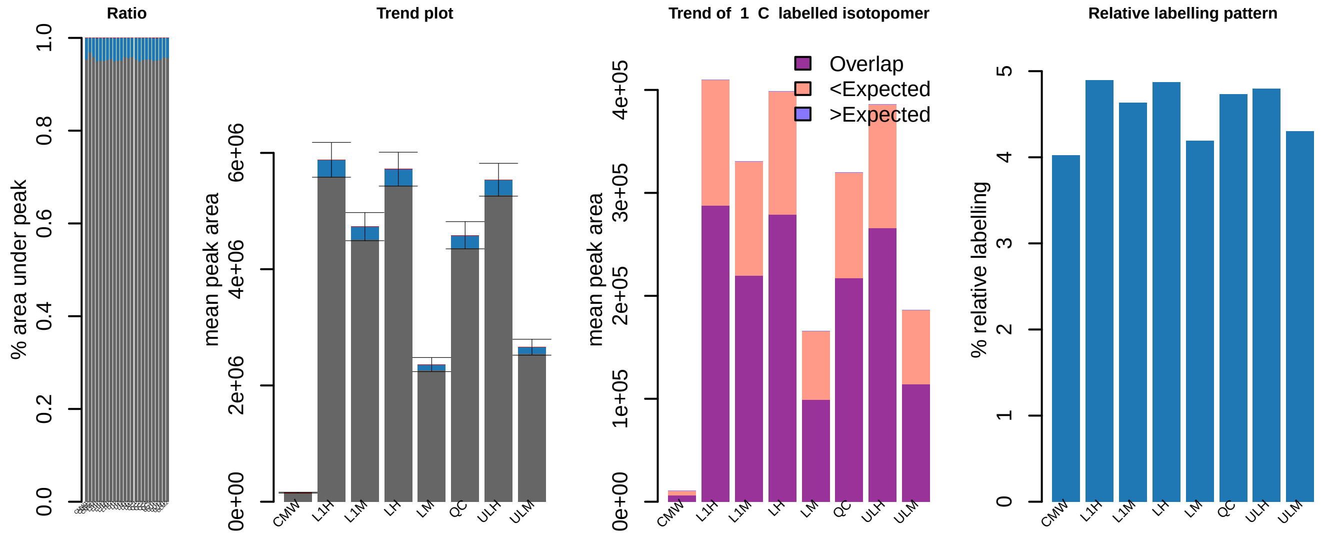

L−Citrulline

Formula: C6H13N3O3 Mass: 175.096 Std.RT: 1031.117394 Ion: POS

G1

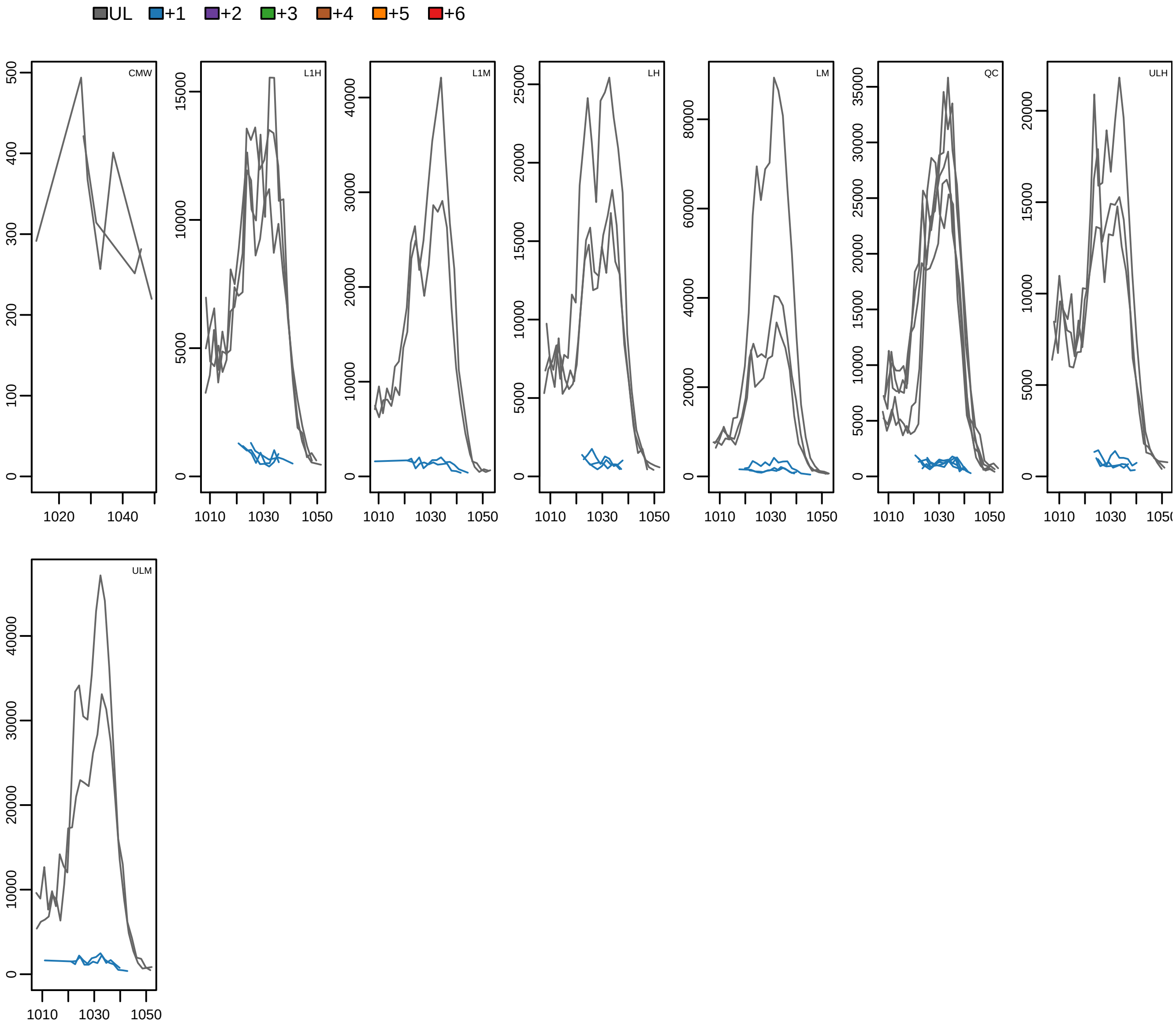

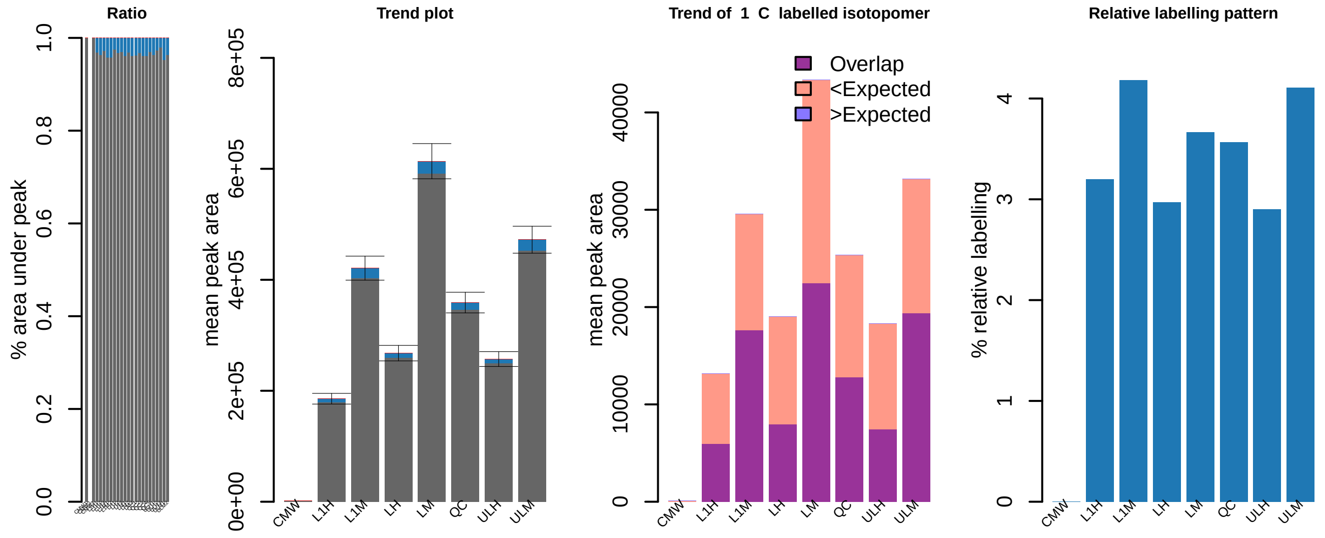

N−Succinyl−L−glutamate

Formula: C9H13NO7 Mass: 247.069 Std.RT: 1044.143334 Ion: POS

G1

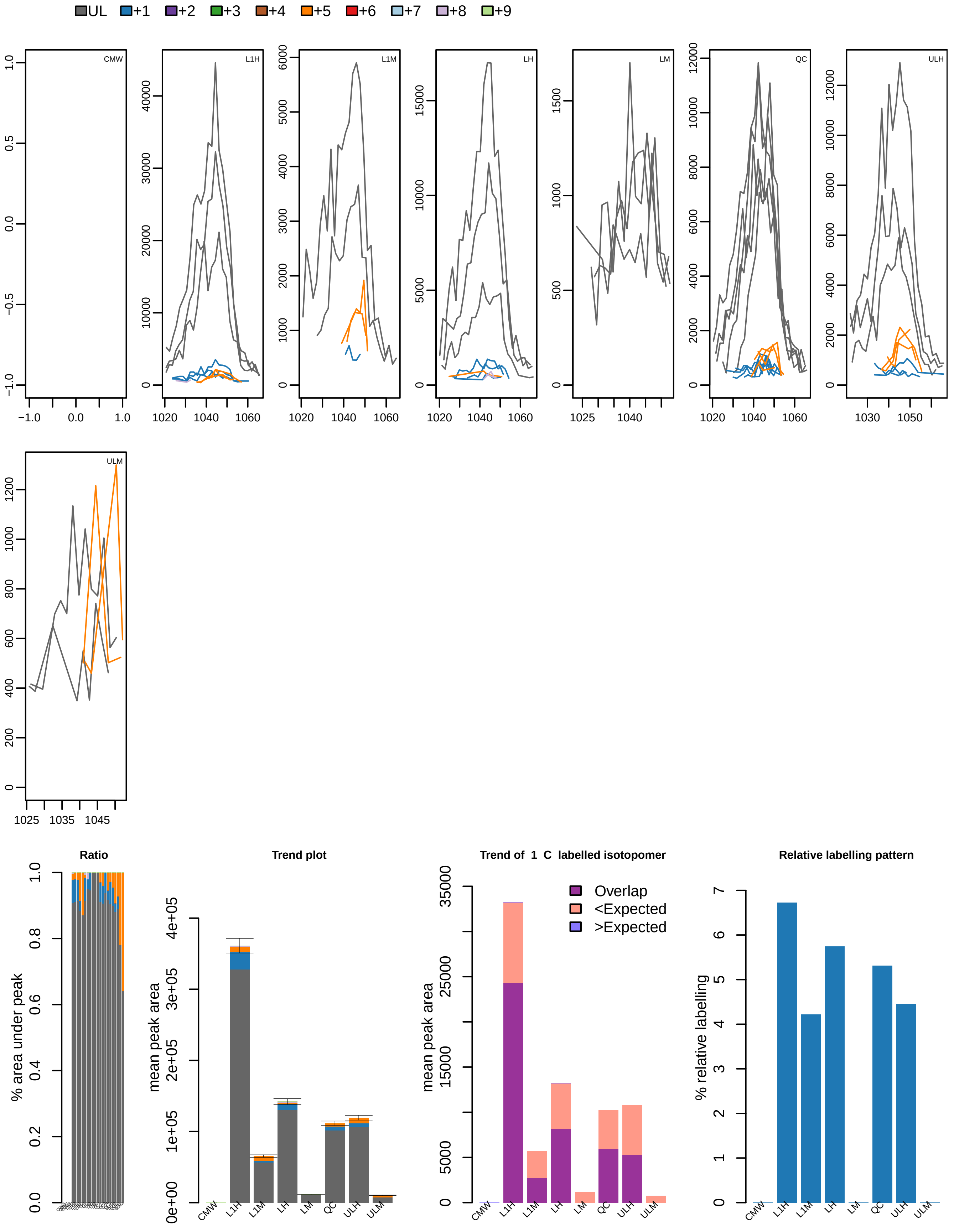

L−Arginine

Formula: C6H14N4O2 Mass: 174.112 Std.RT: 1663.7383368 Ion: PC

G1

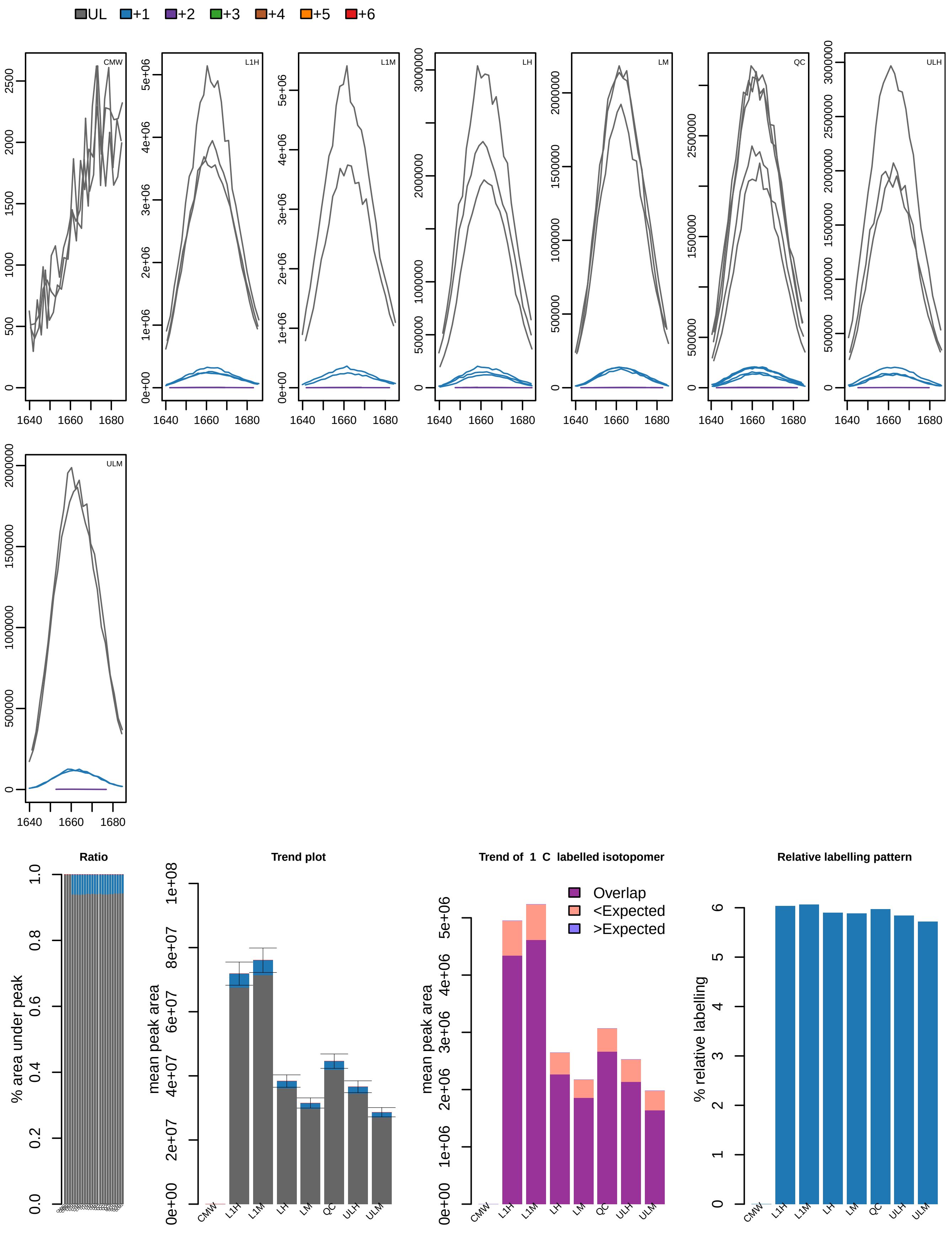

L−Ornithine

Formula: C5H12N2O2 Mass: 132.09 Std.RT: 1441.7858382 Ion: POS

G1

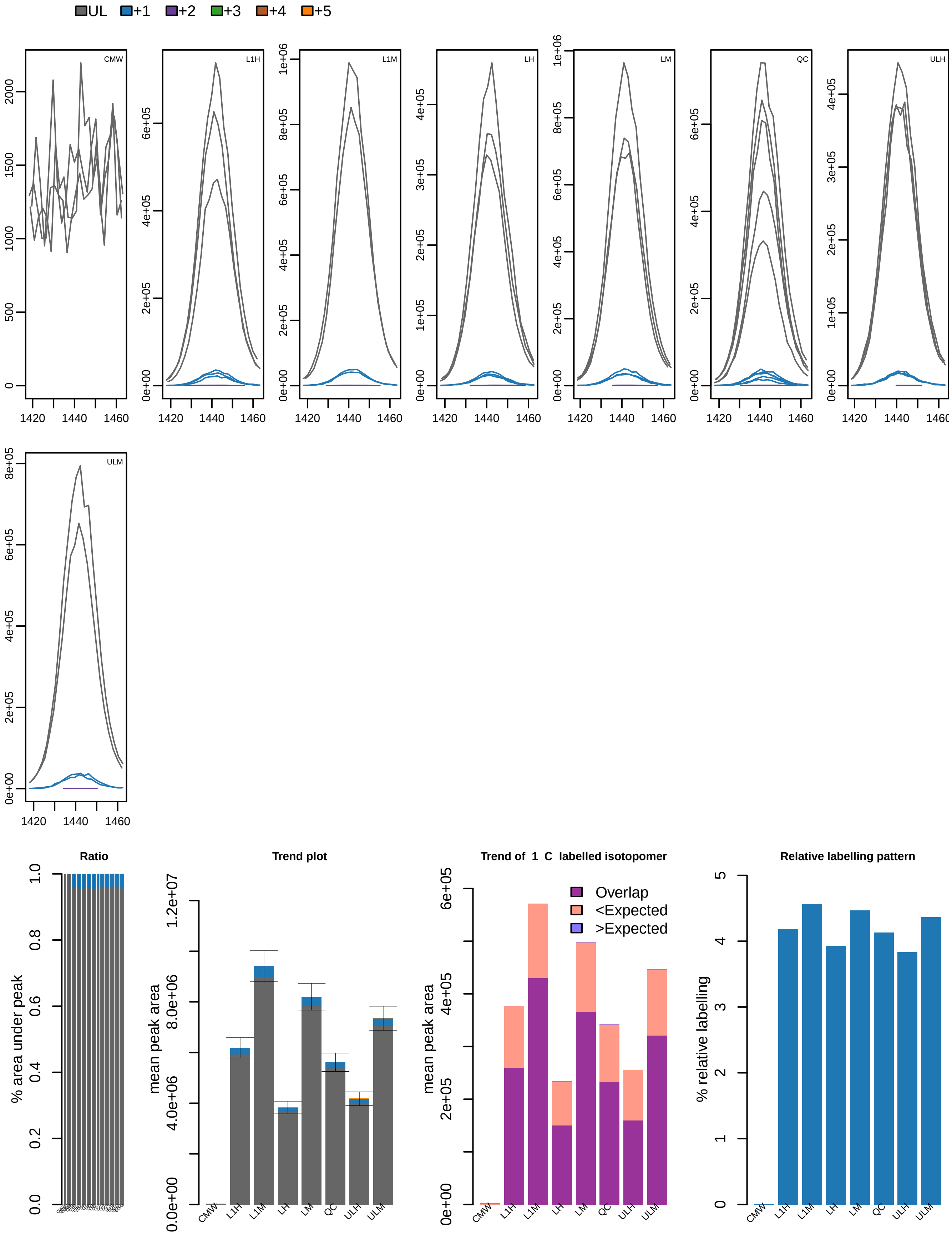

4−Aminobutanoate

Formula: C4H9NO2 Mass: 103.063 Std.RT: 1005.050664 Ion: POS

G1

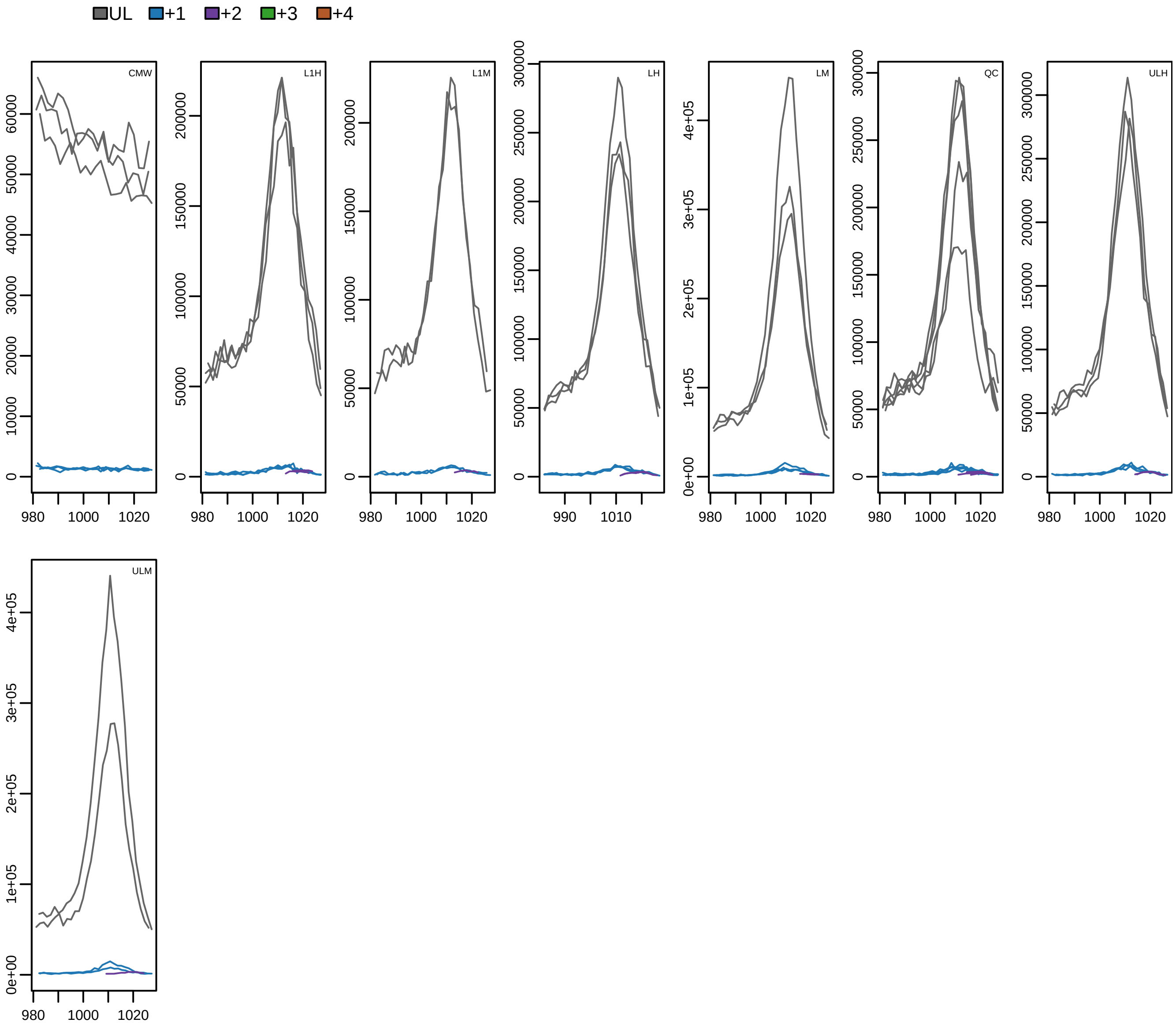

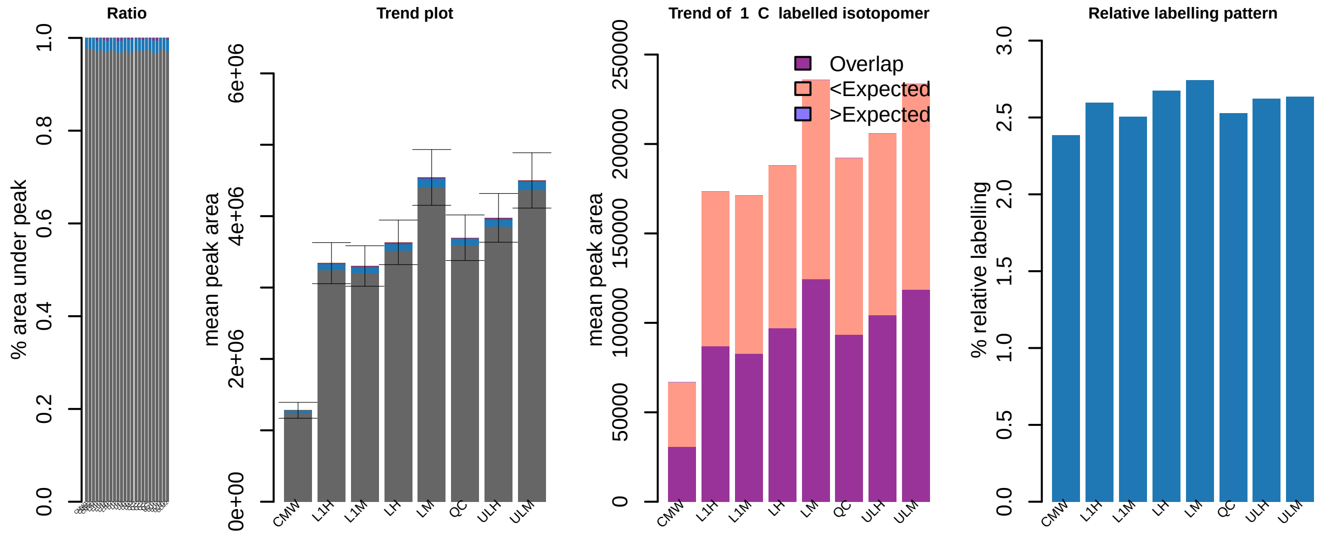

S−Adenosyl−L−methionine

Formula: C15H22N6O5S Mass: 398.137 Std.RT: 1055.9685702 Ion:

G1

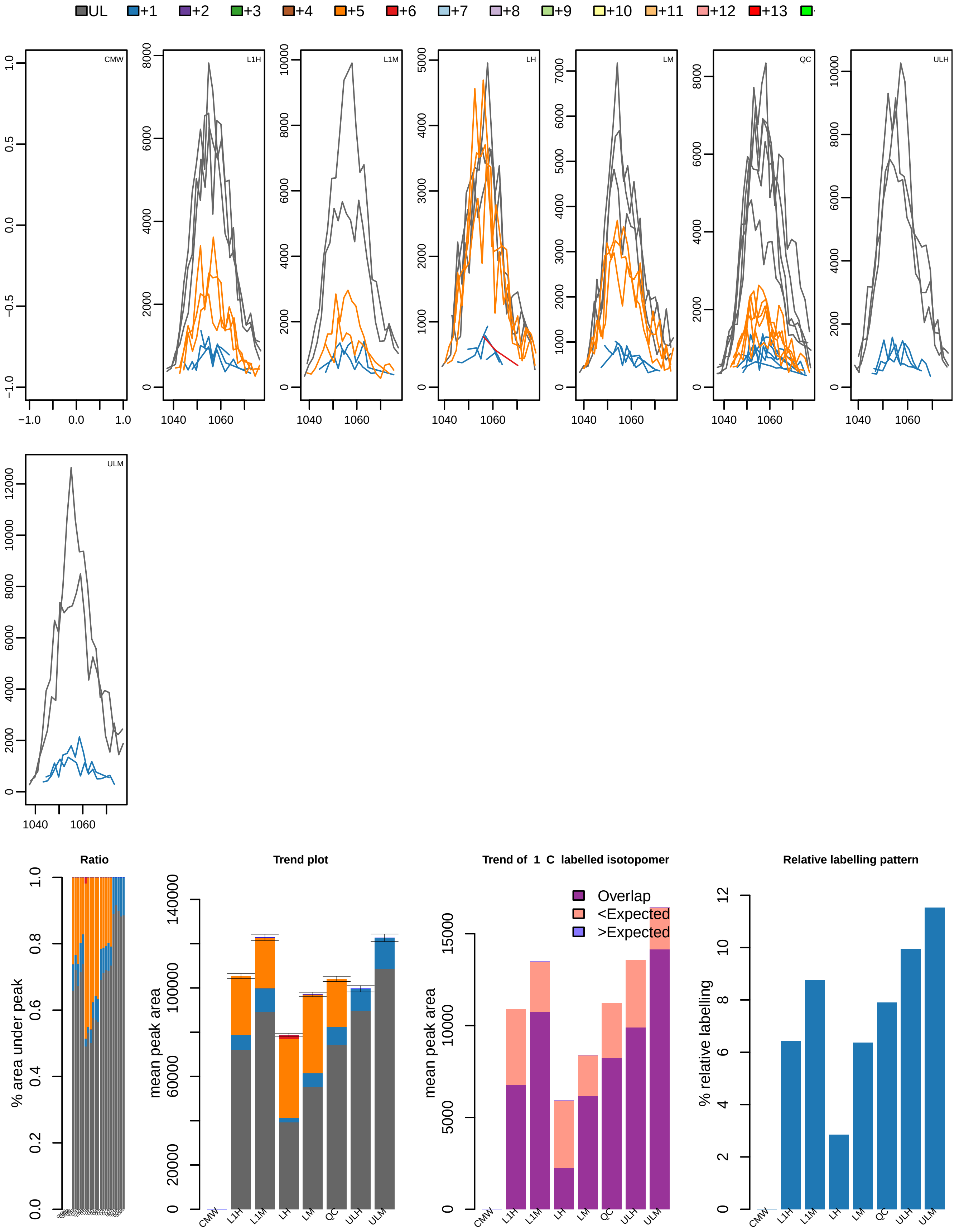

5−Methylthioadenosine

Formula: C11H15N5O3S Mass: 297.09 Std.RT: 433.23409308 Ion: P

G1

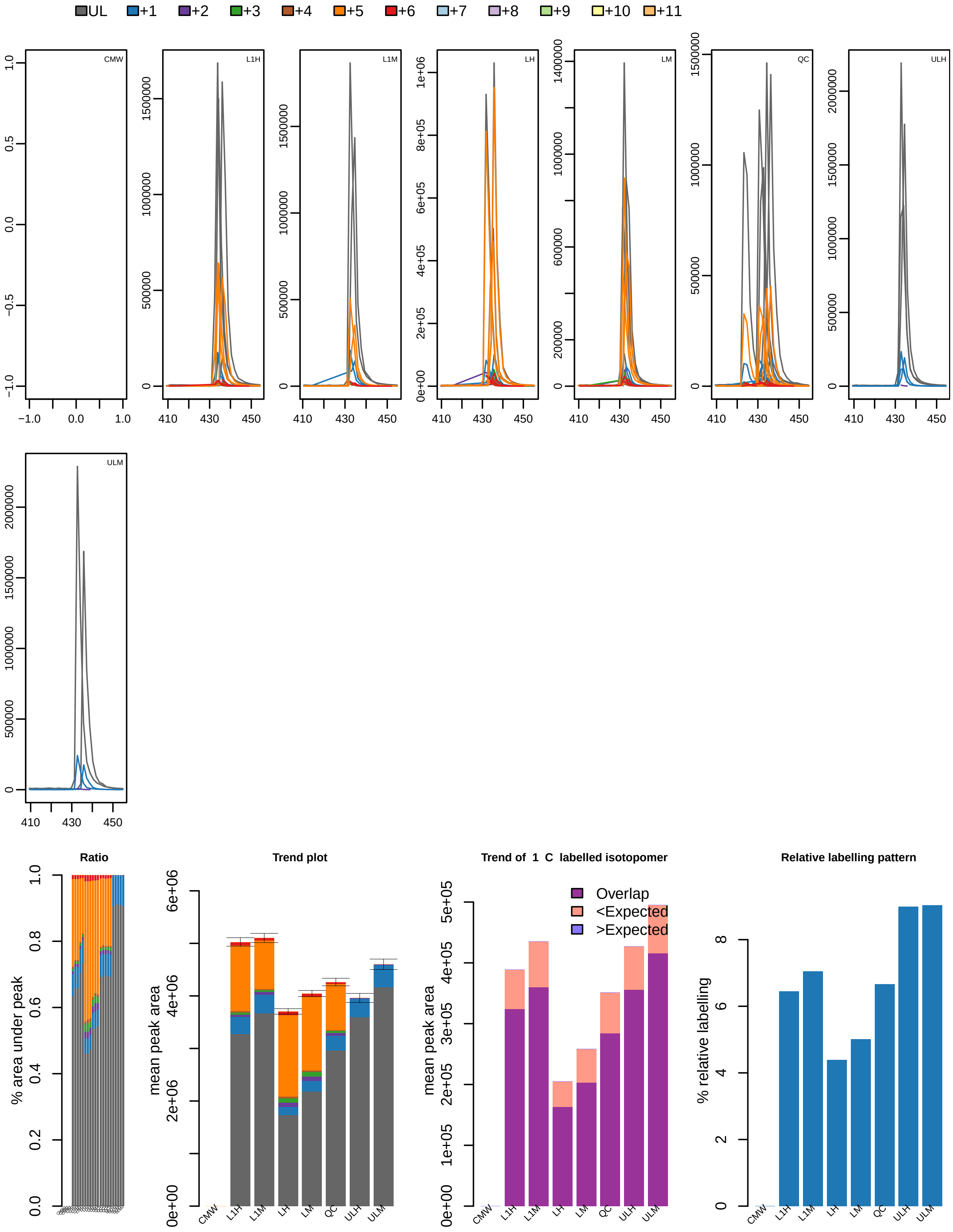

L−Proline

Formula: C5H9NO2 Mass: 115.063 Std.RT: 828.6907476 Ion: POS

G1

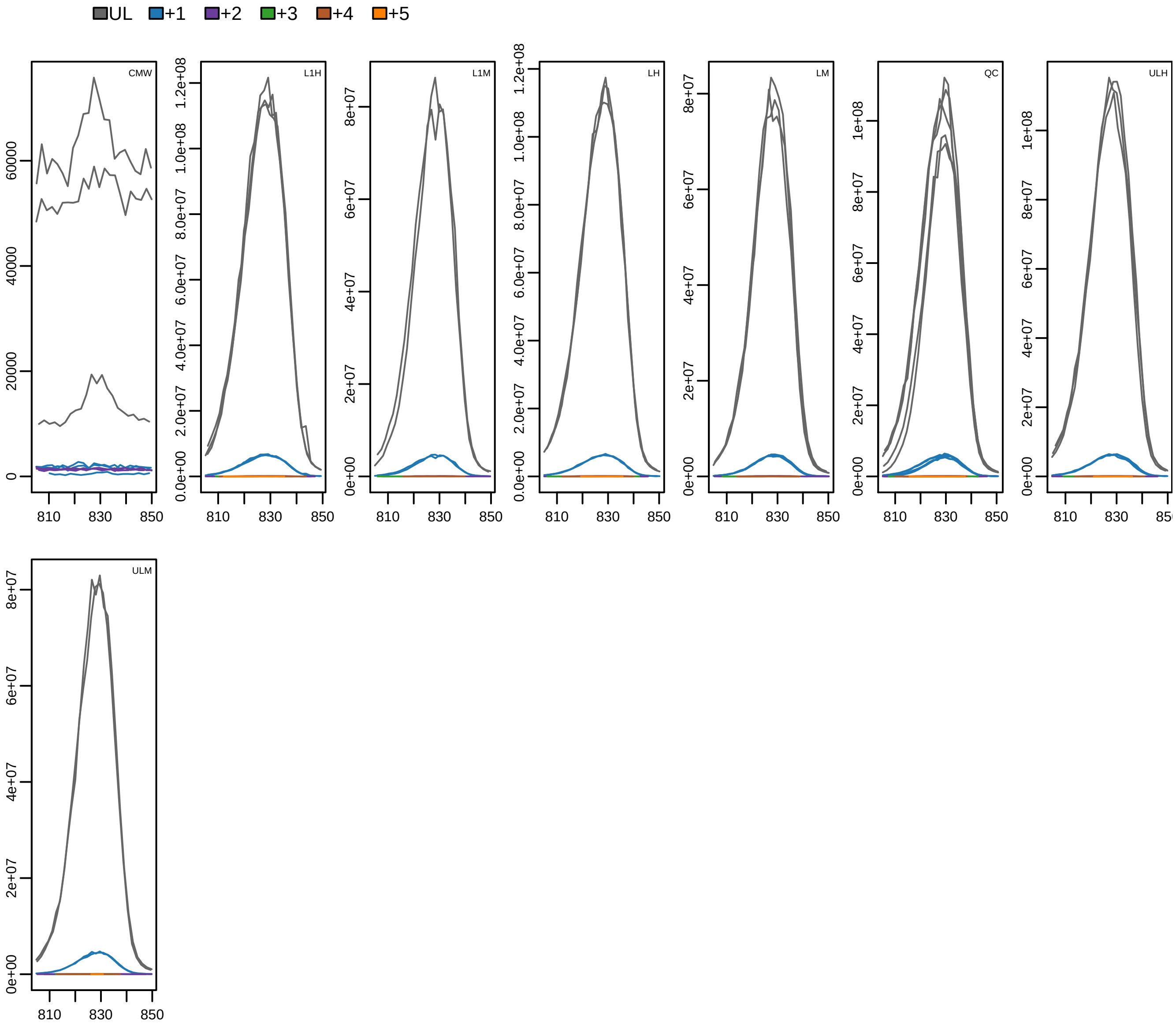

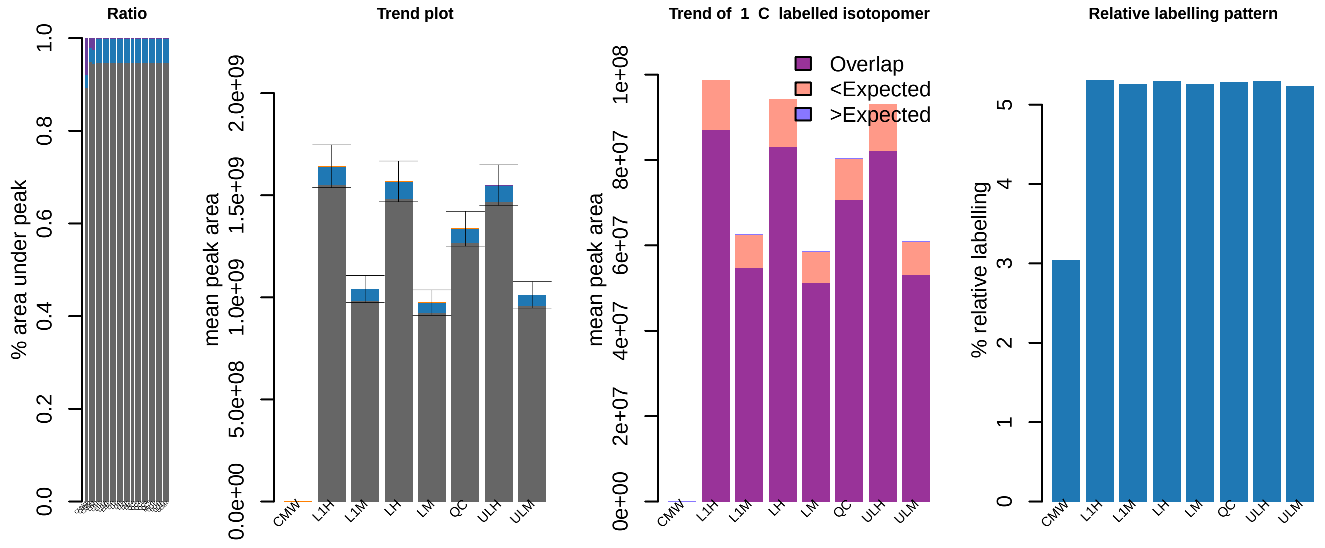

N−Acetyl−L−citrulline

Formula: C8H15N3O4 Mass: 217.106 Std.RT: 774.403146 Ion: POS

G1

UL +1 +2 +3 +4 +5 +6 +7 +8

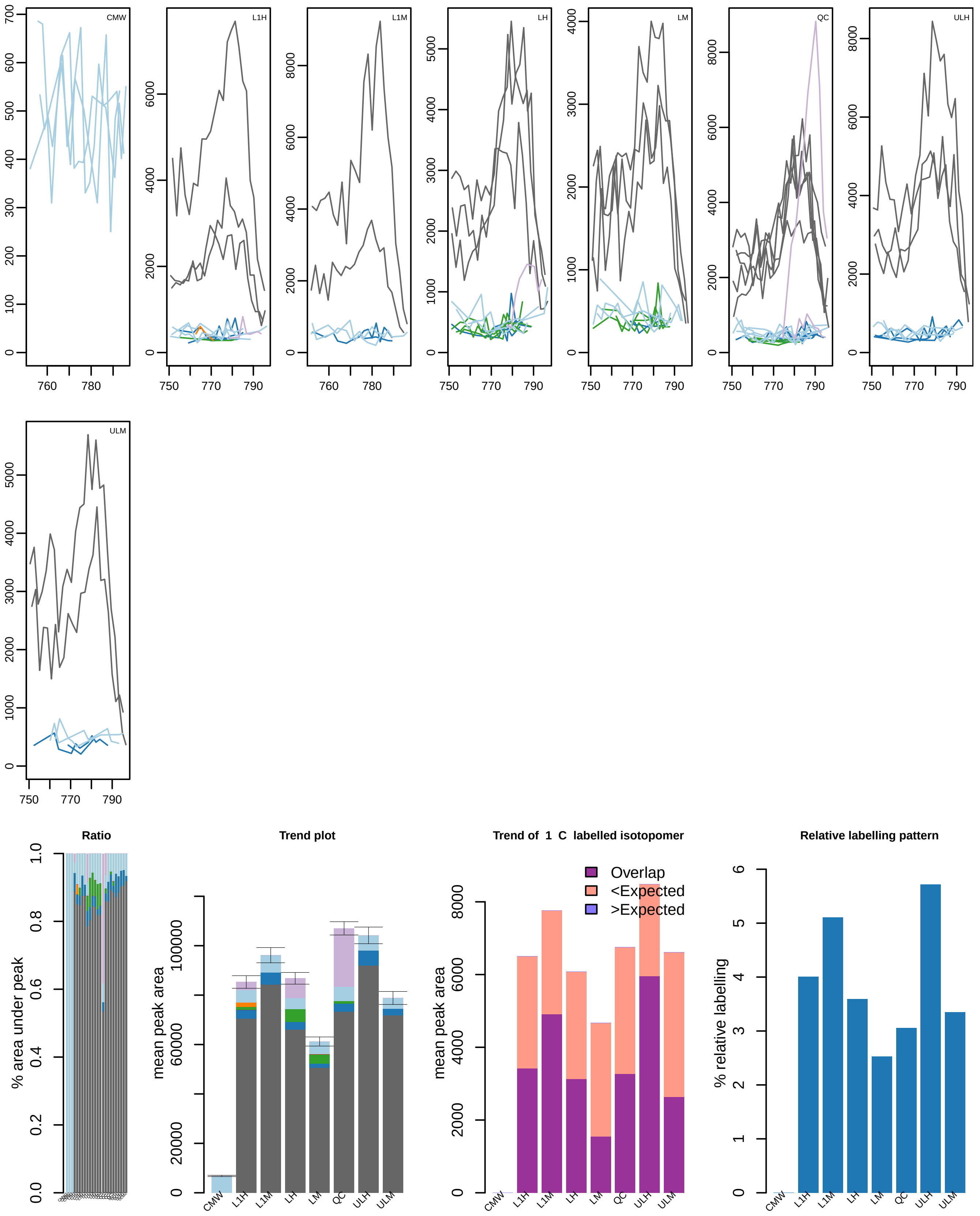

N−Acetyl−L−citrulline

Formula: C8H15N3O4 Mass: 217.106 Std.RT: 774.403146 Ion: POS

G2

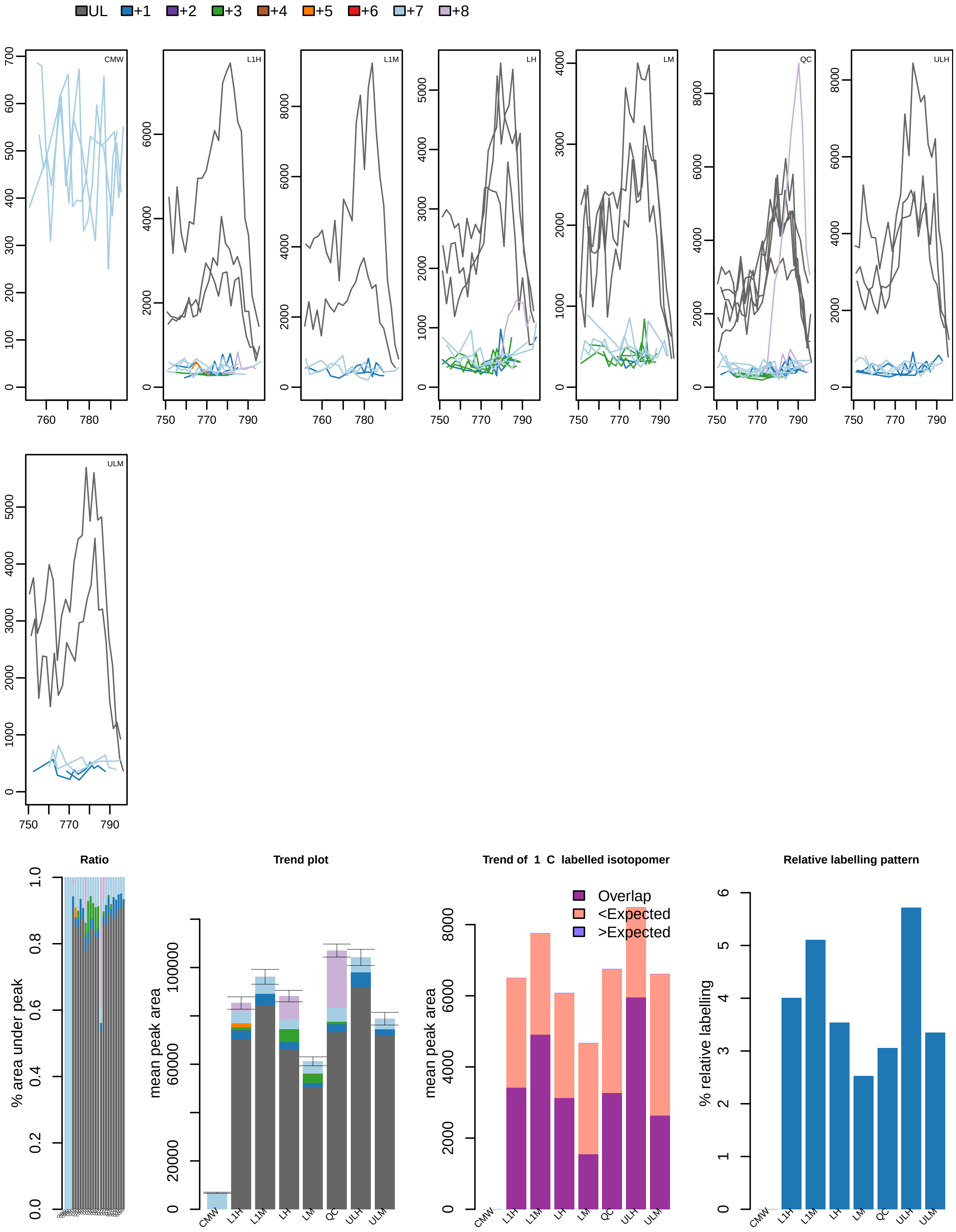

1−5−diazabicyclononane

Formula: C7H14N2 Mass: 126.116 Std.RT: 842.0227512 Ion: POS

G1

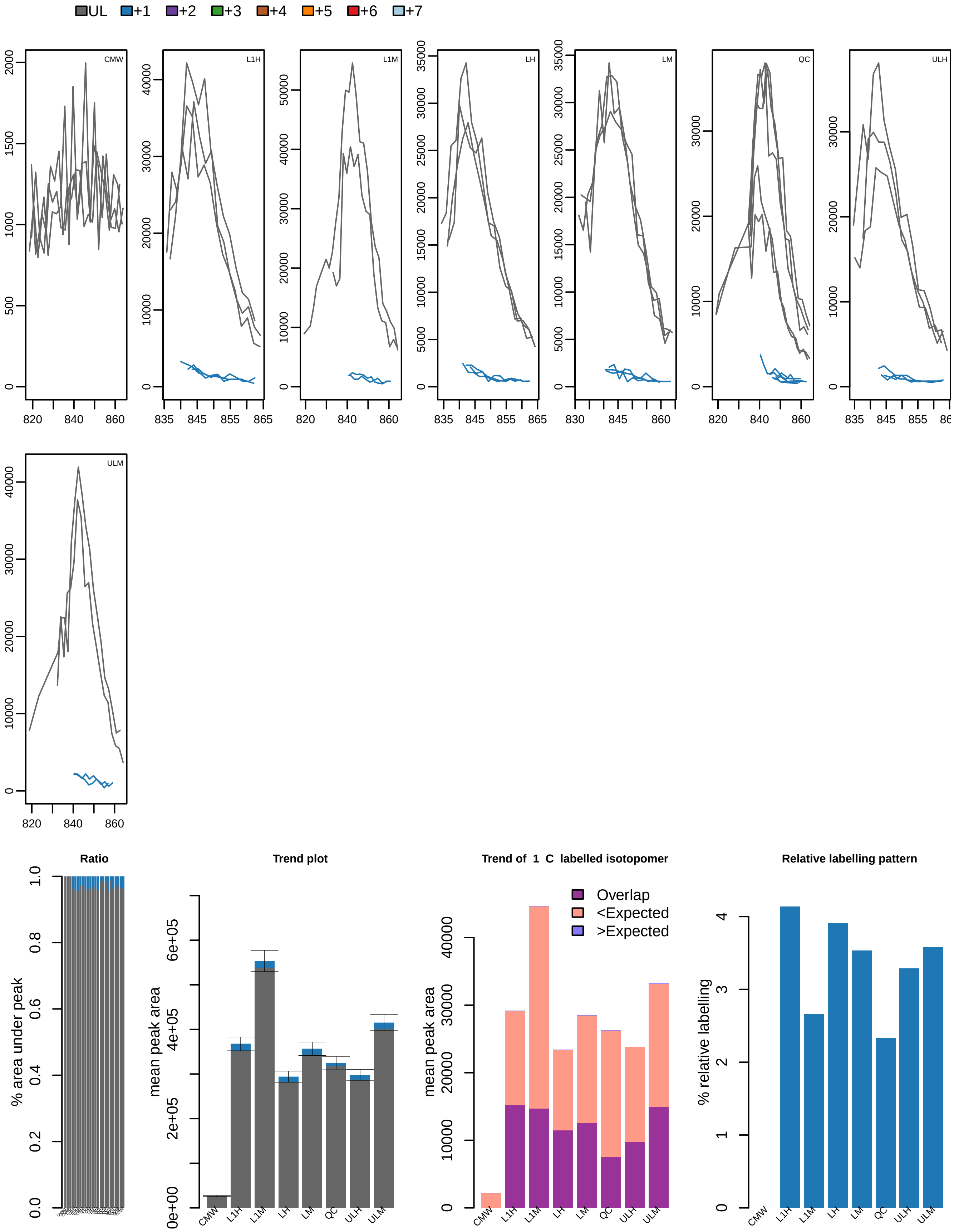

Pantothenate

Formula: C9H17NO5 Mass: 219.111 Std.RT: 491.4513741 Ion: POS

G1

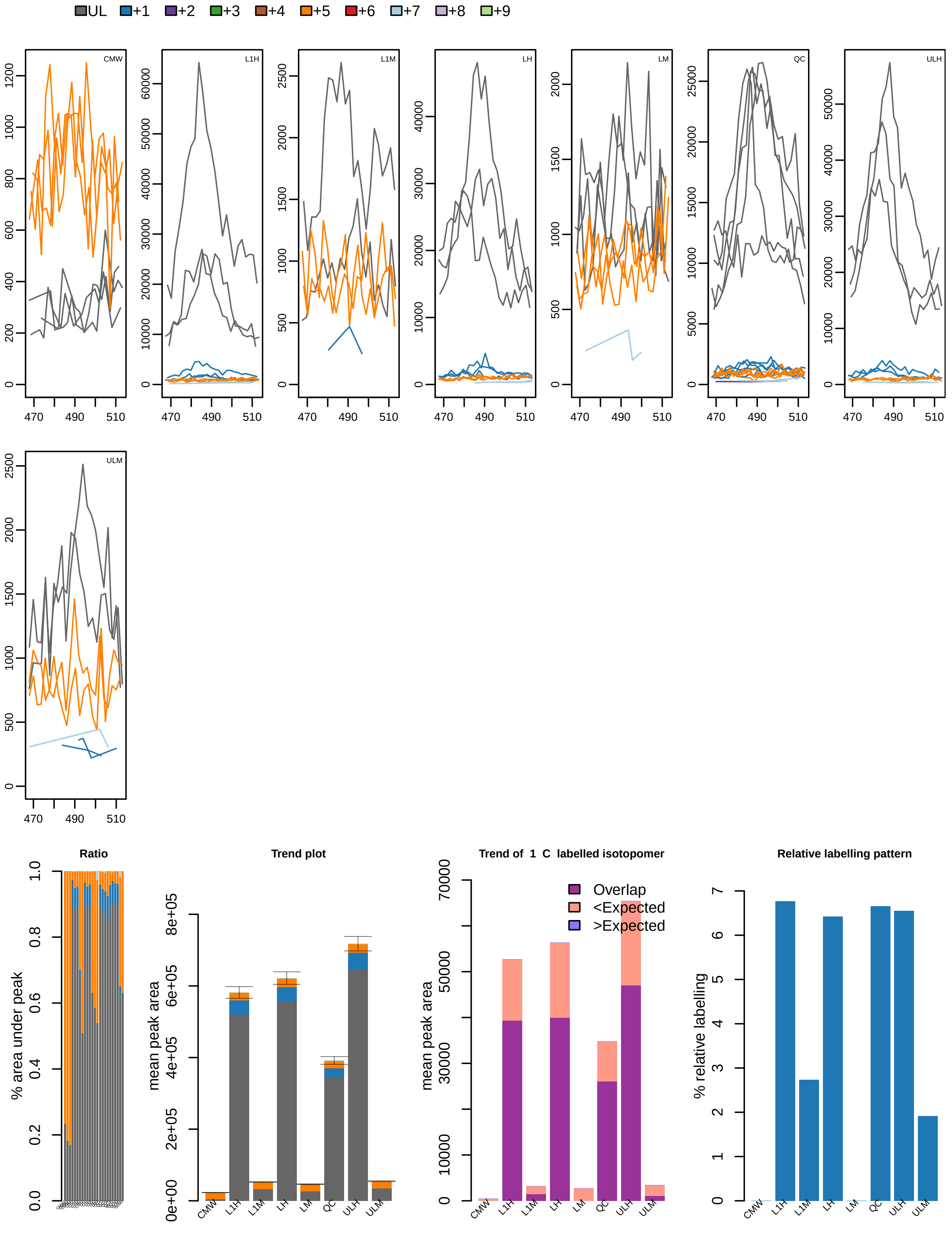

gamma−Amino−gamma−cyanobutanoate

Formula: $C_5H_8N_2O_2$ Mass: 128.059 Std.RT: 966.9915036 Ion: POS

G1

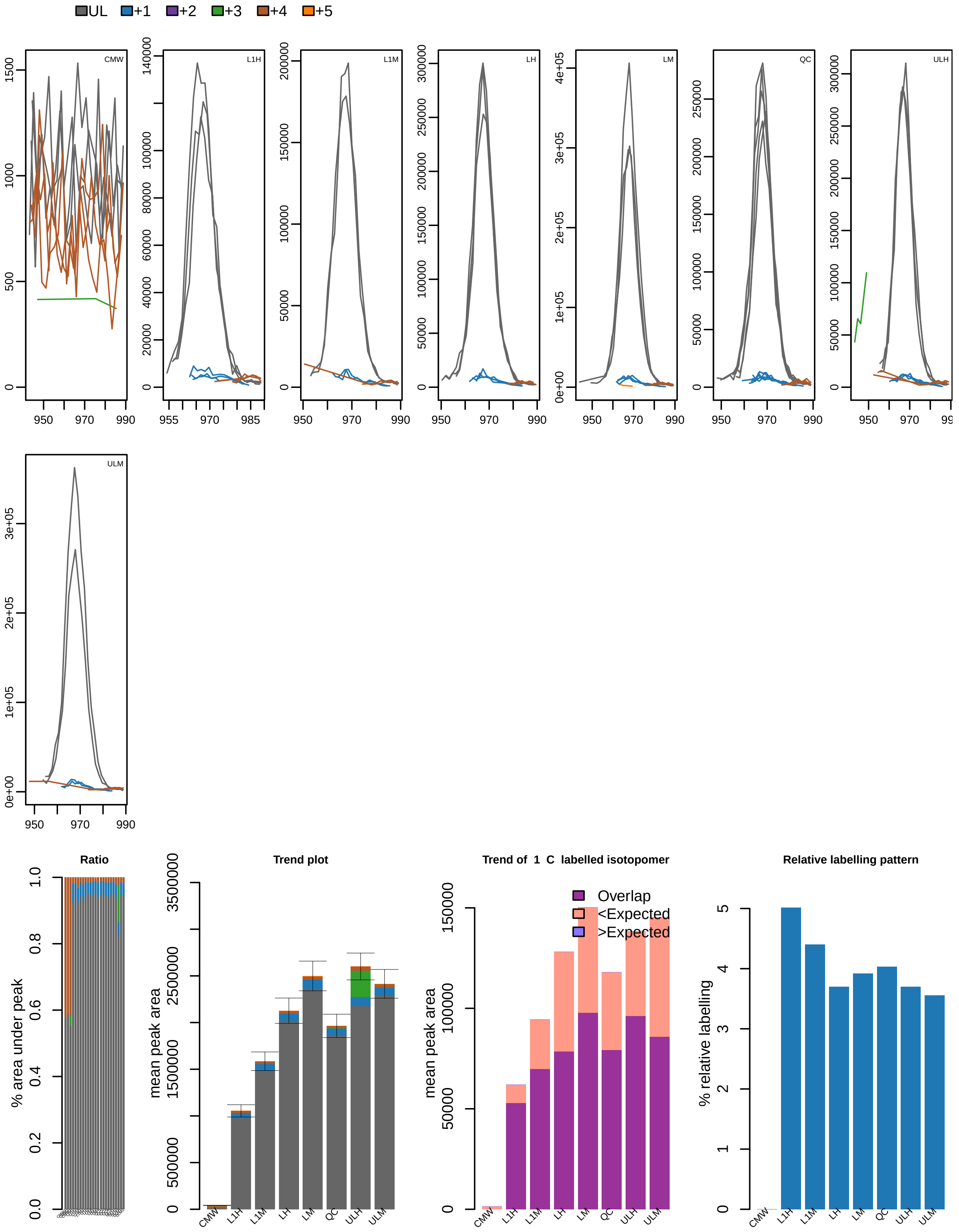

L−Cystine

Formula: C6H12N2O4S2 Mass: 240.024 Std.RT: 1039.1999922 Ion:

G1

UL +1 +2 +3 +4 +5 +6

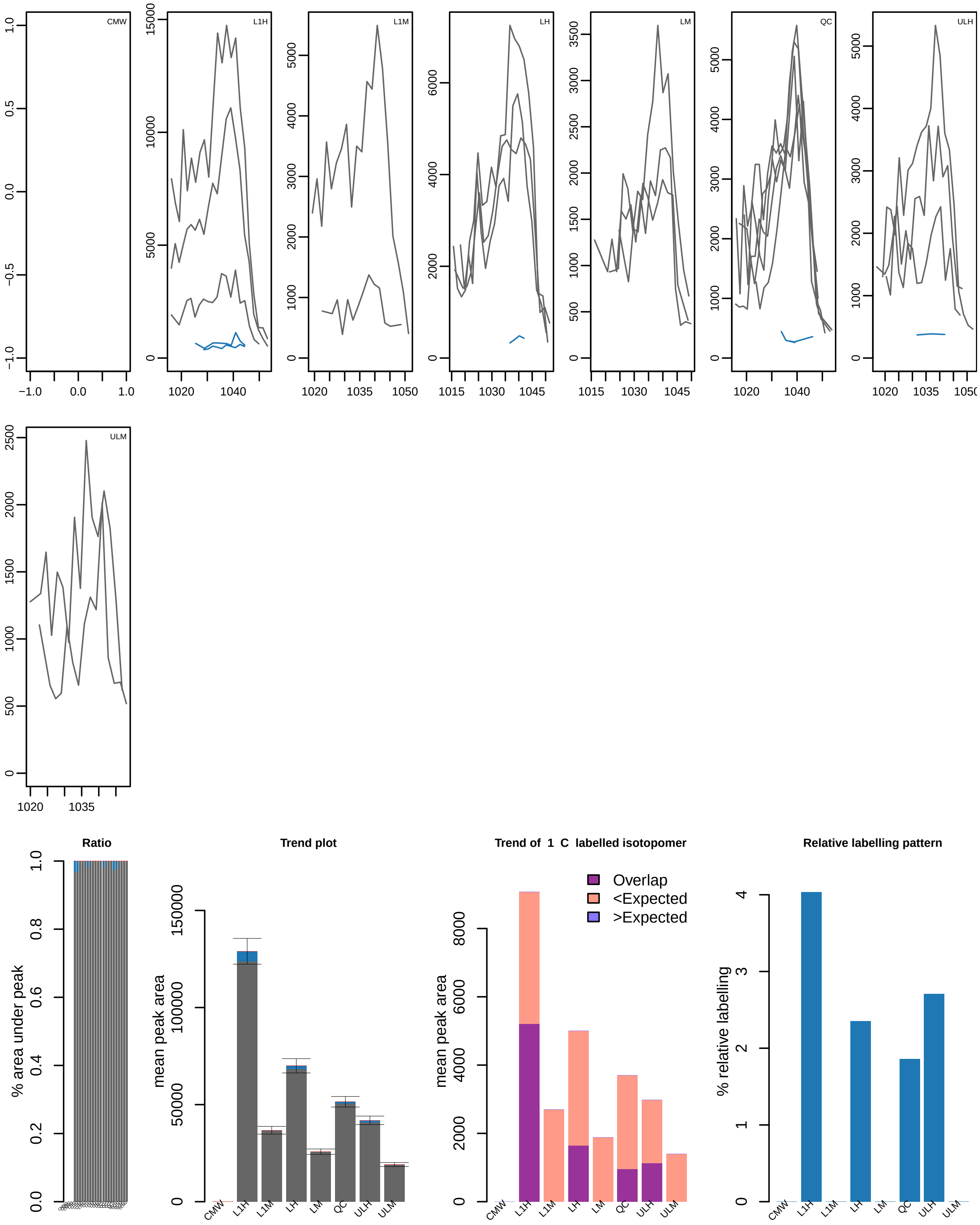

D−Alanyl−D−alanine

Formula: C6H12N2O3 Mass: 160.085 Std.RT: 685.4119974 Ion: POS

G1

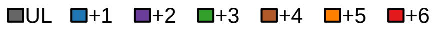

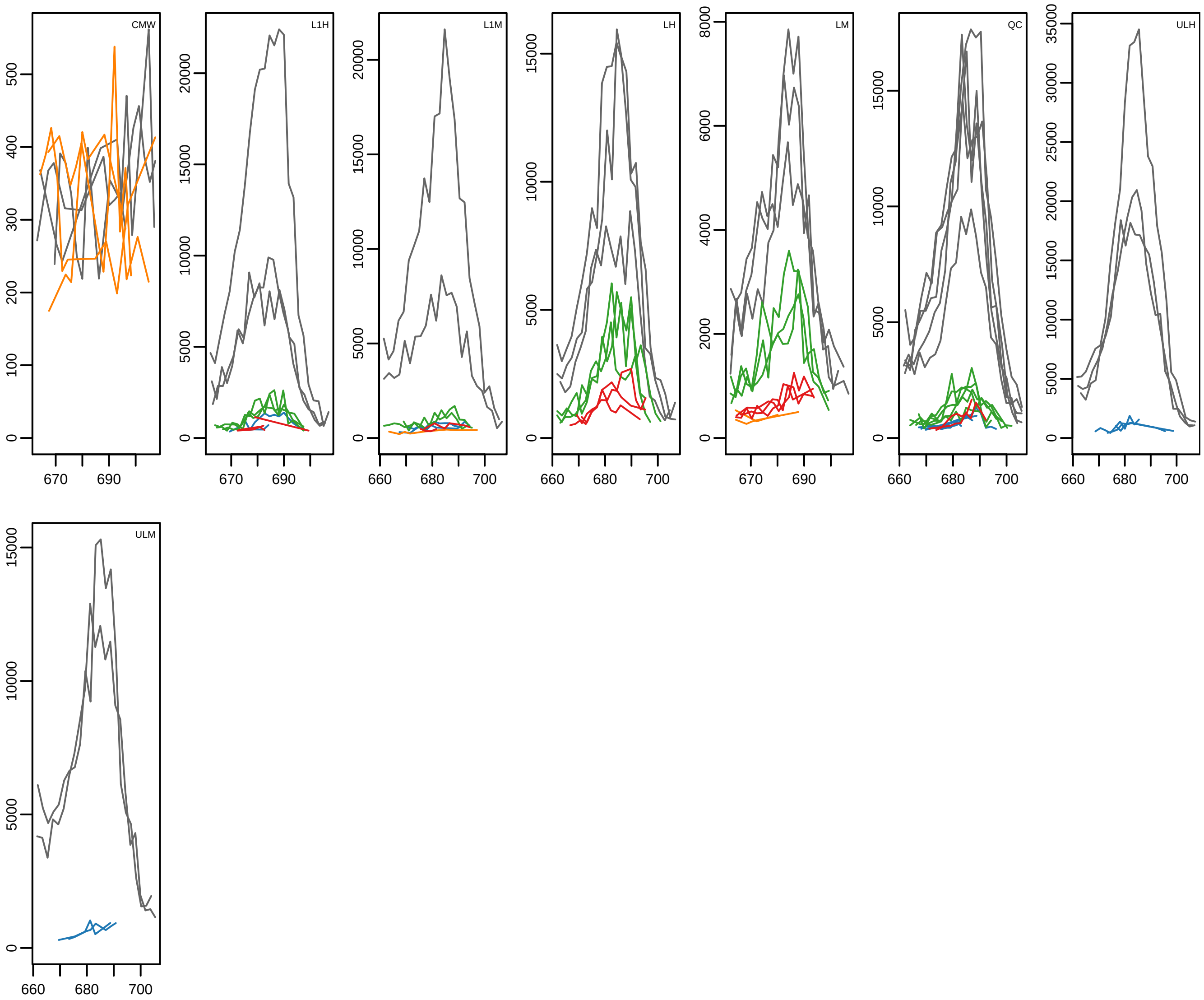

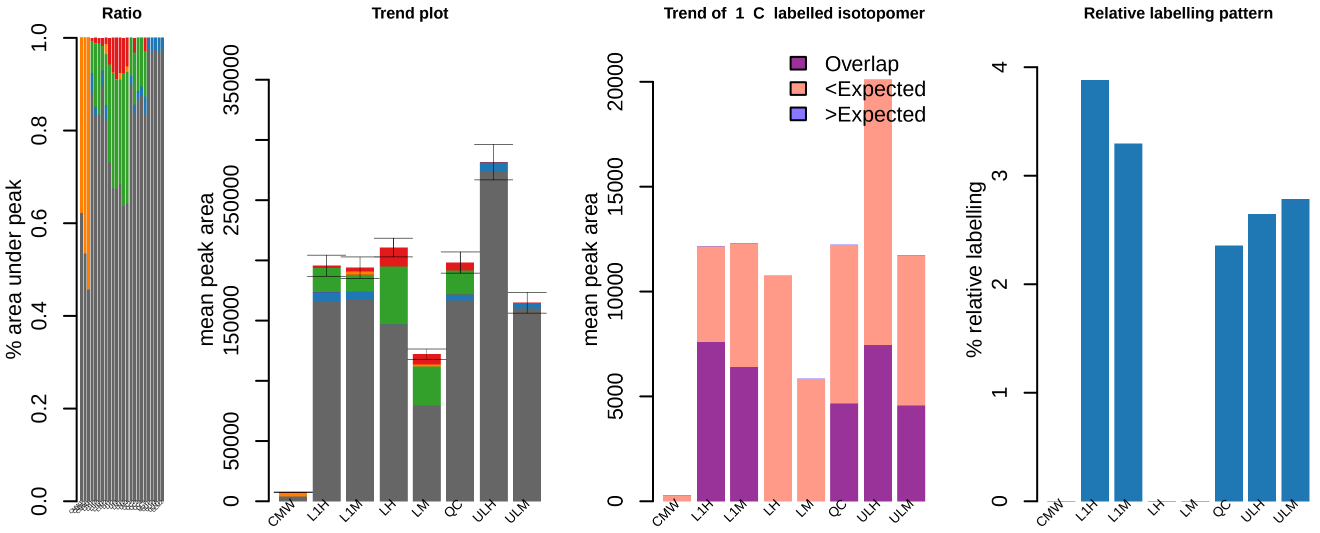

5−Guanidino−2−oxopentanoate

Formula: C6H11N3O3 Mass: 173.08 Std.RT: 926.4054564 Ion: POS

G1

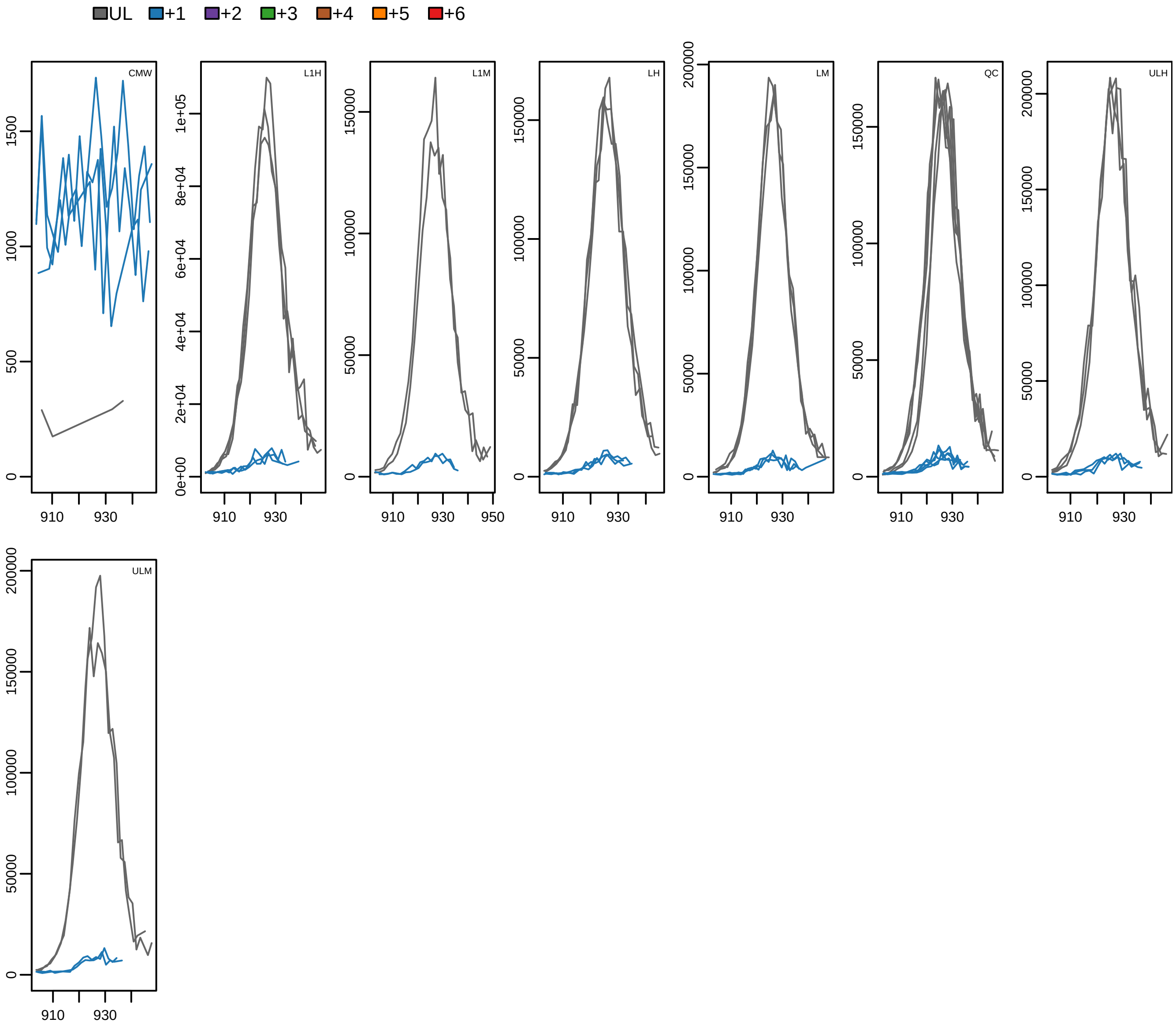

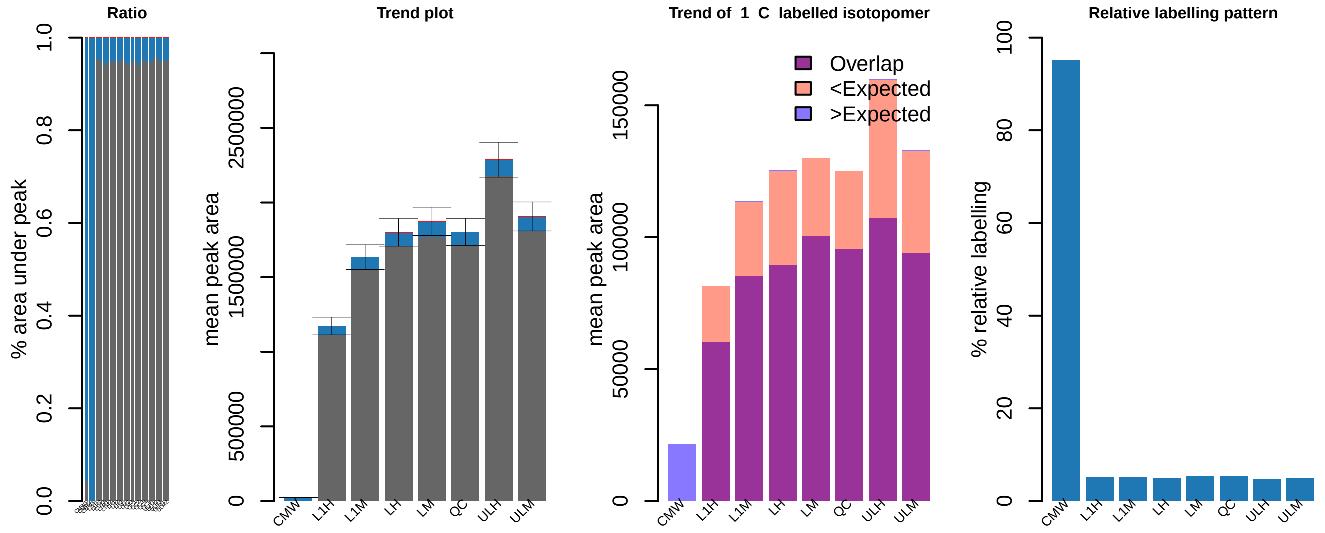

N−Acetyl−D−glucosamine

Formula: C8H15NO6 Mass: 221.09 Std.RT: 756.5660052 Ion: POS

G1

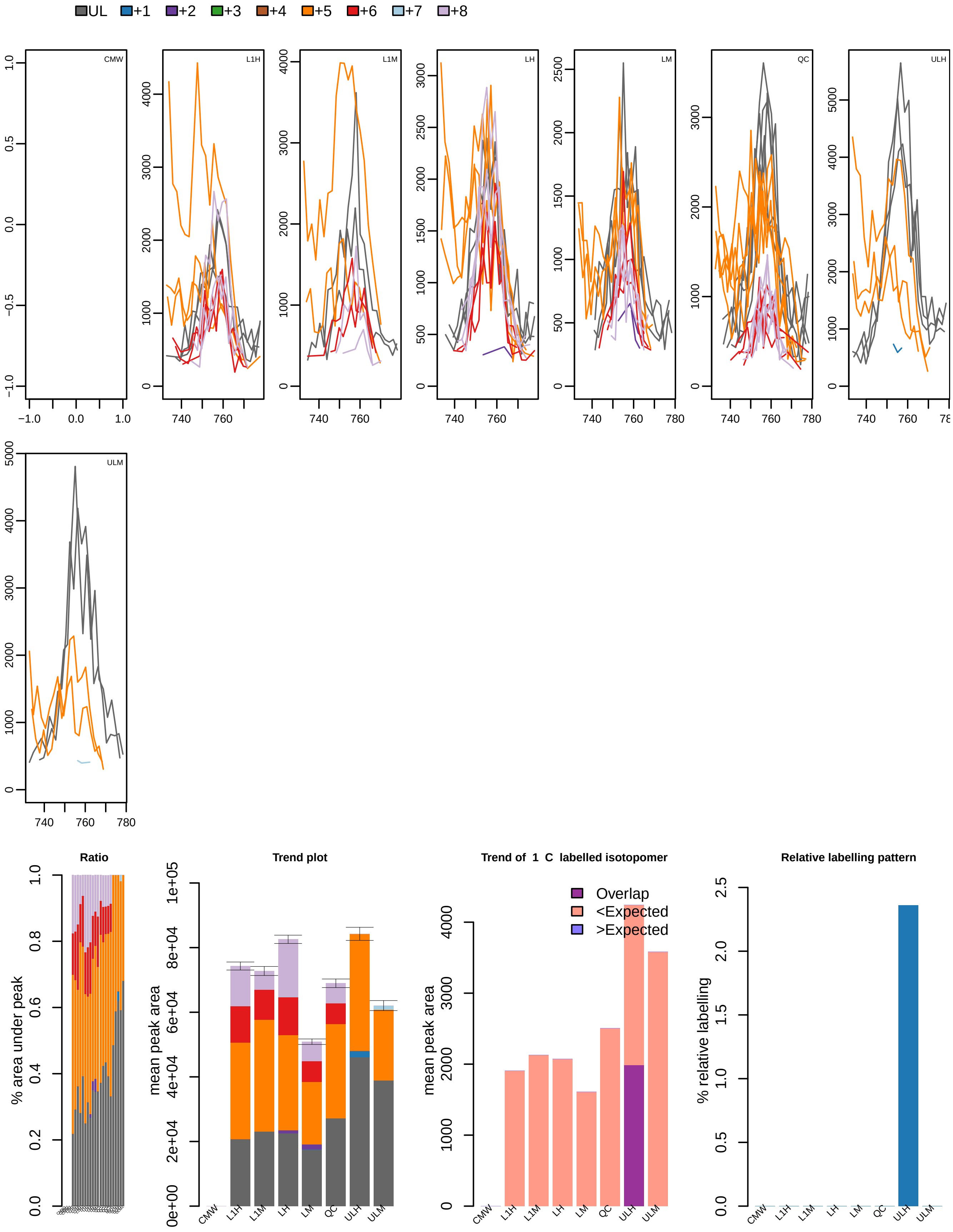

Glutathione

Formula: $C10H17N3O6S$ Mass: 307.084 Std.RT: 930.9508638 Ion: P

G1

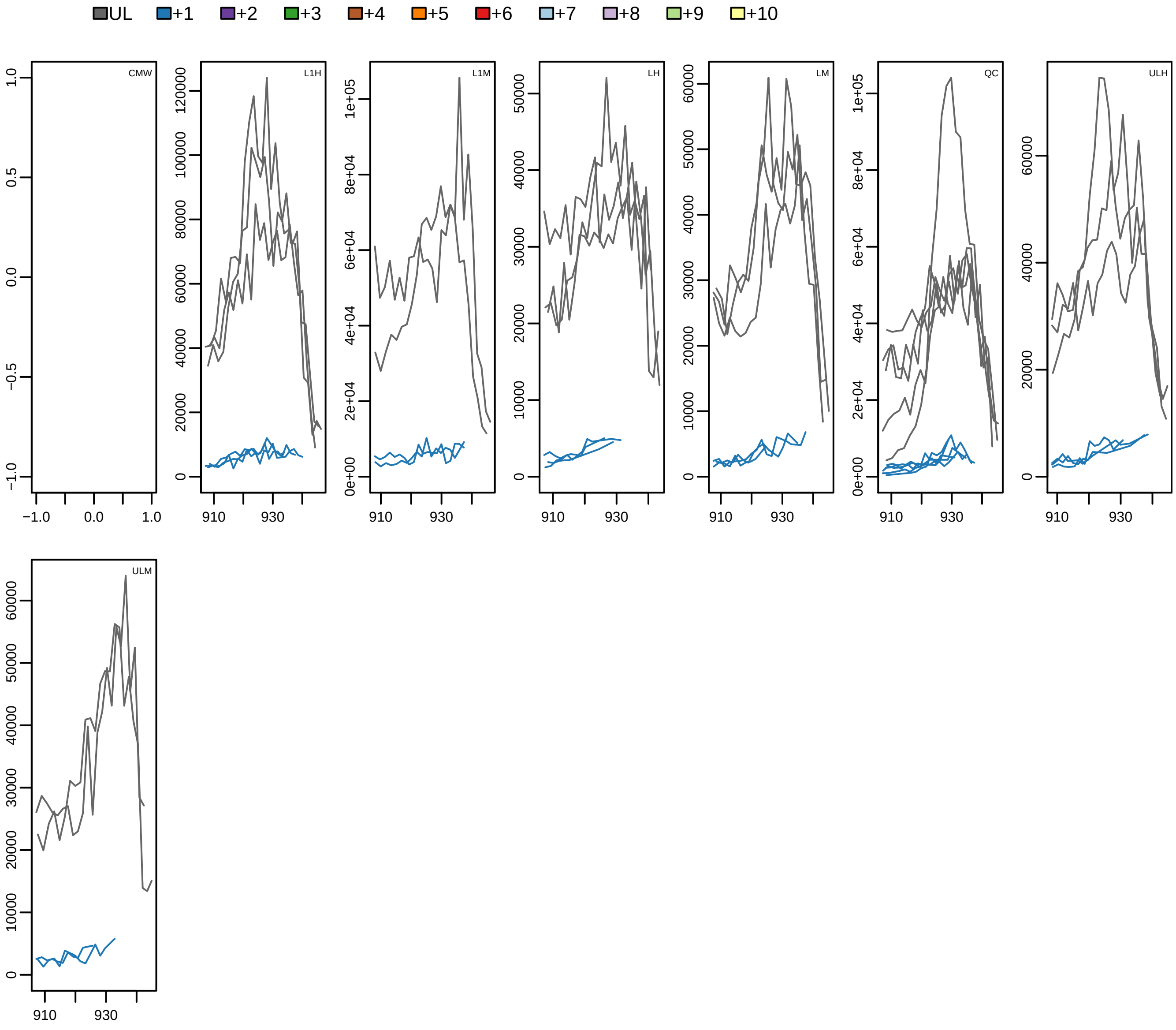

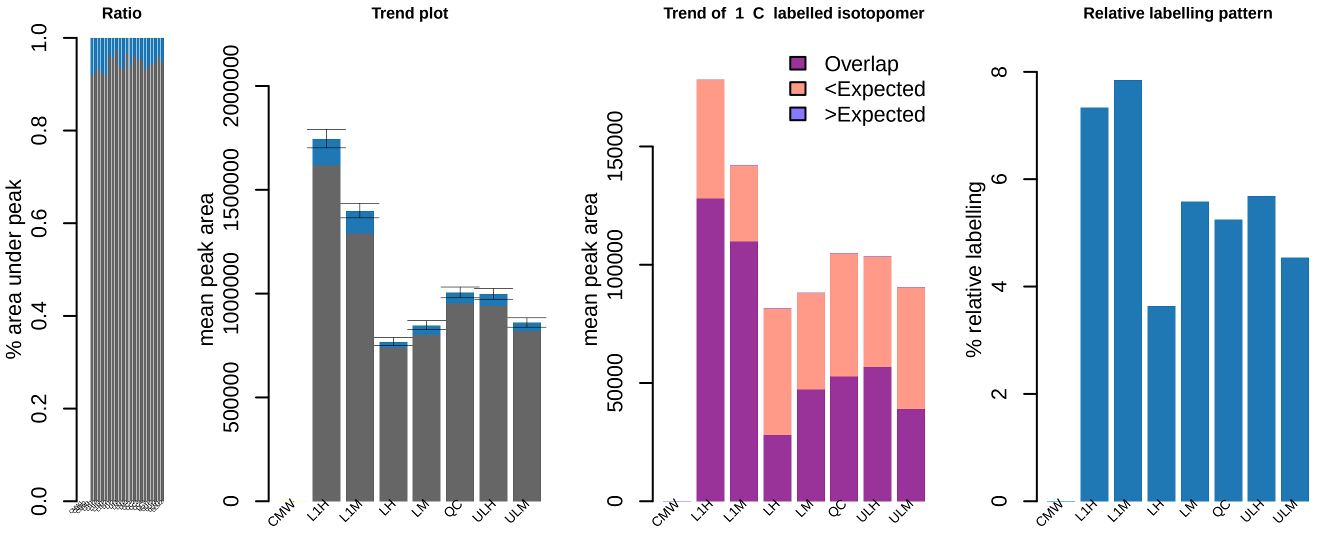

L−Glutamine

Formula: C5H10N2O3 Mass: 146.069 Std.RT: 975.577497 Ion: POS

G1

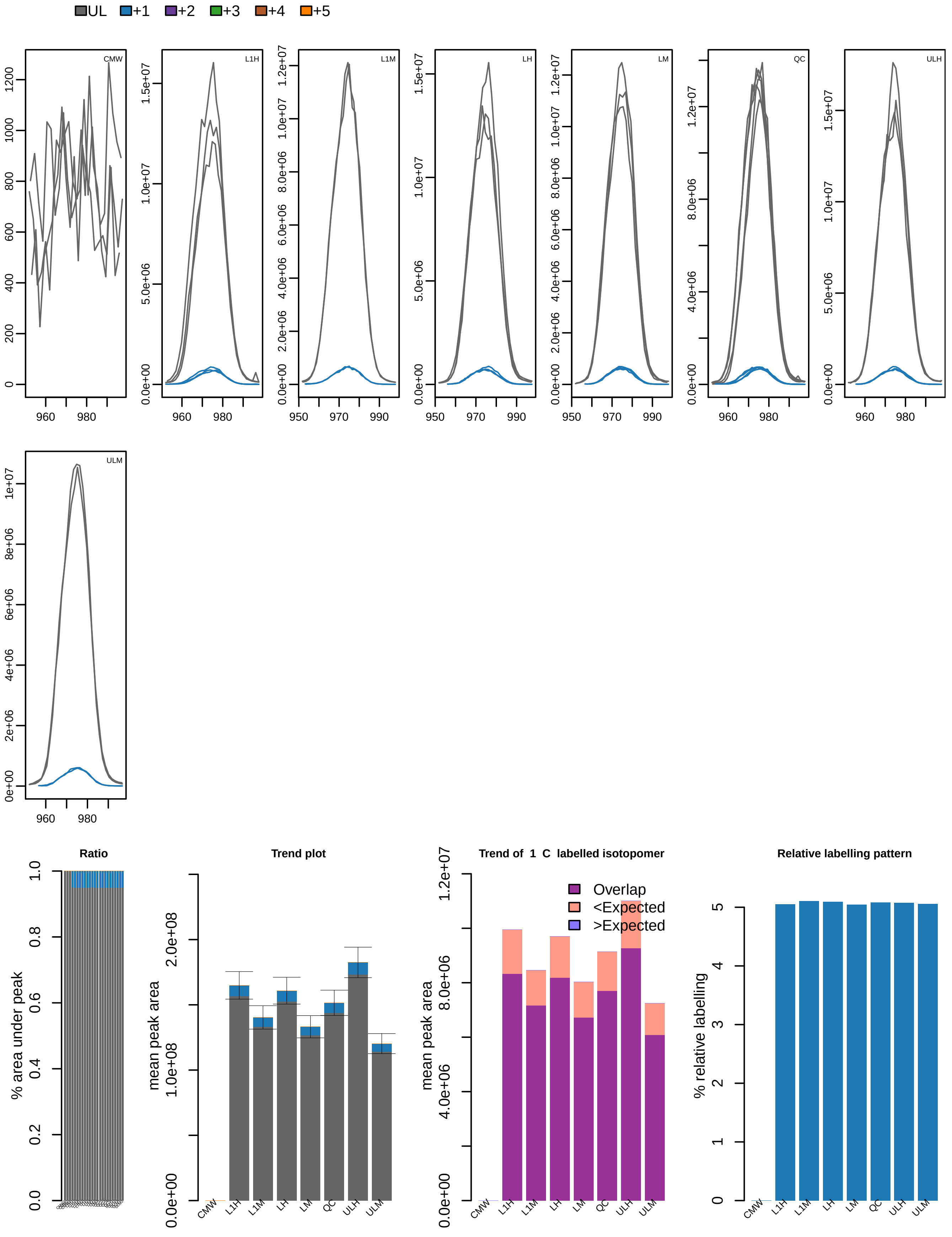

Trypanothione disulfide

Formula: C27H47N9O10S2 Mass: 721.289 Std.RT: 1274.986524 Ion:

G1

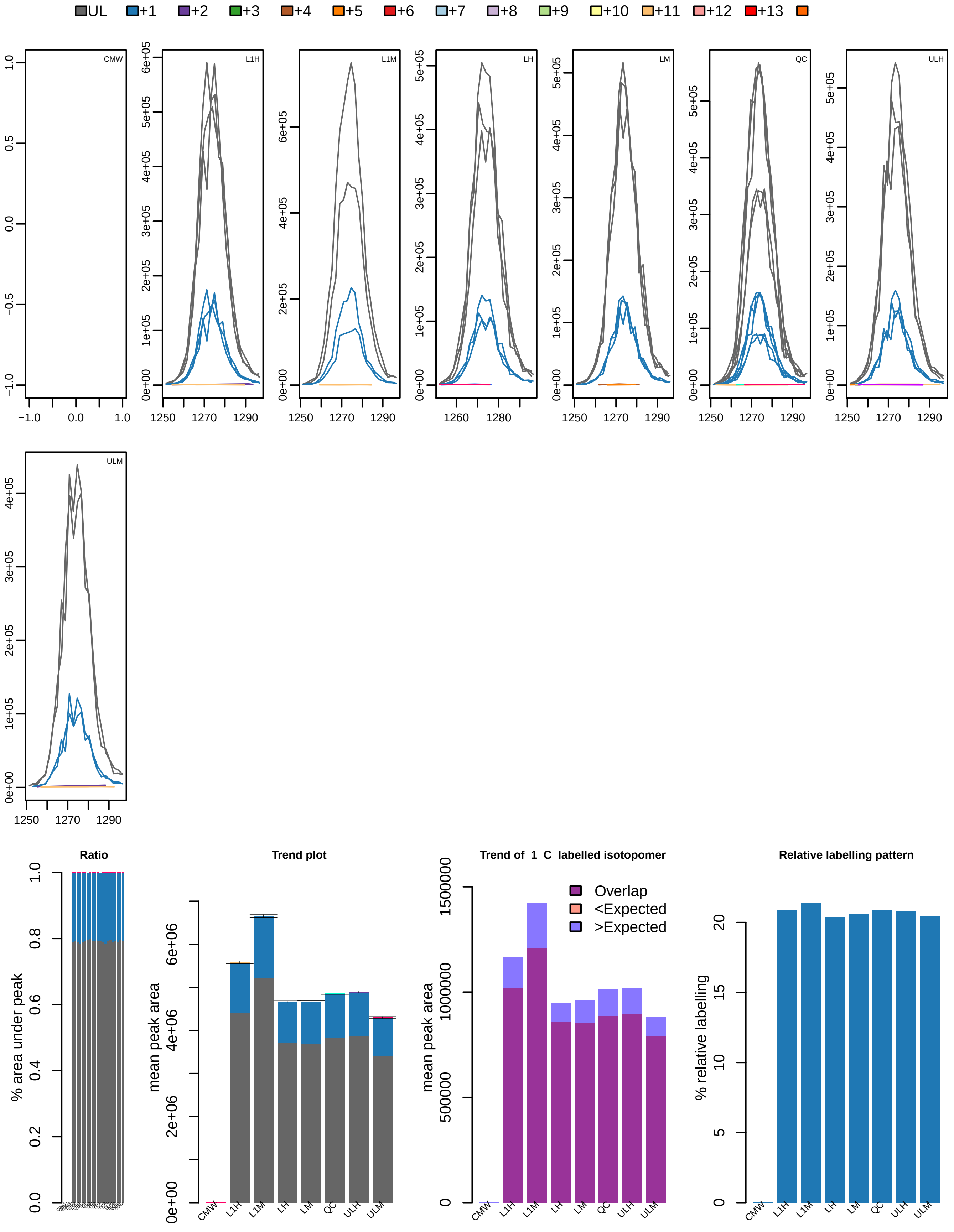

N,N−Dimethylglycine

Formula: C4H9NO2 Mass: 103.063 Std.RT: 786.826749 Ion: POS

G1

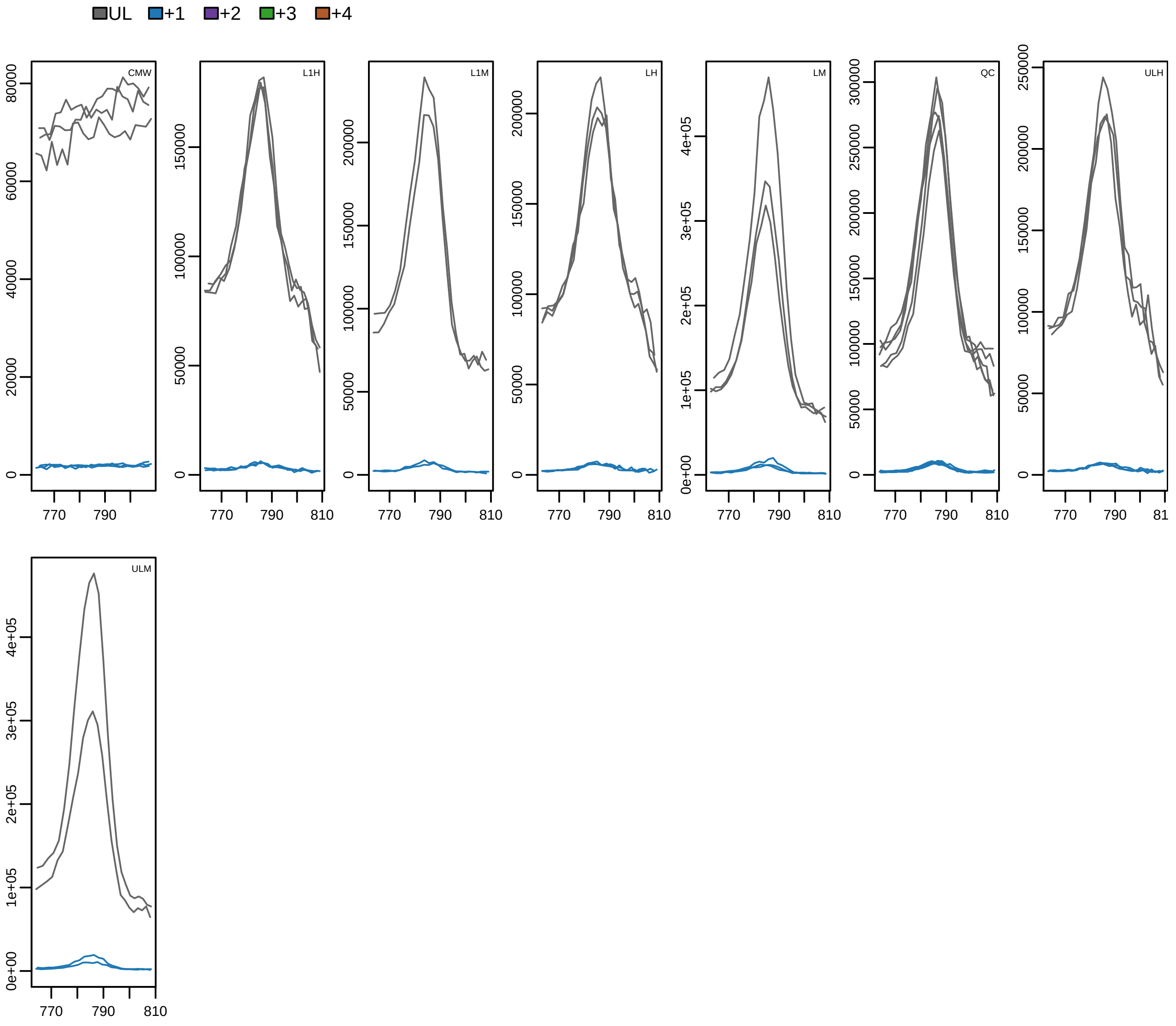

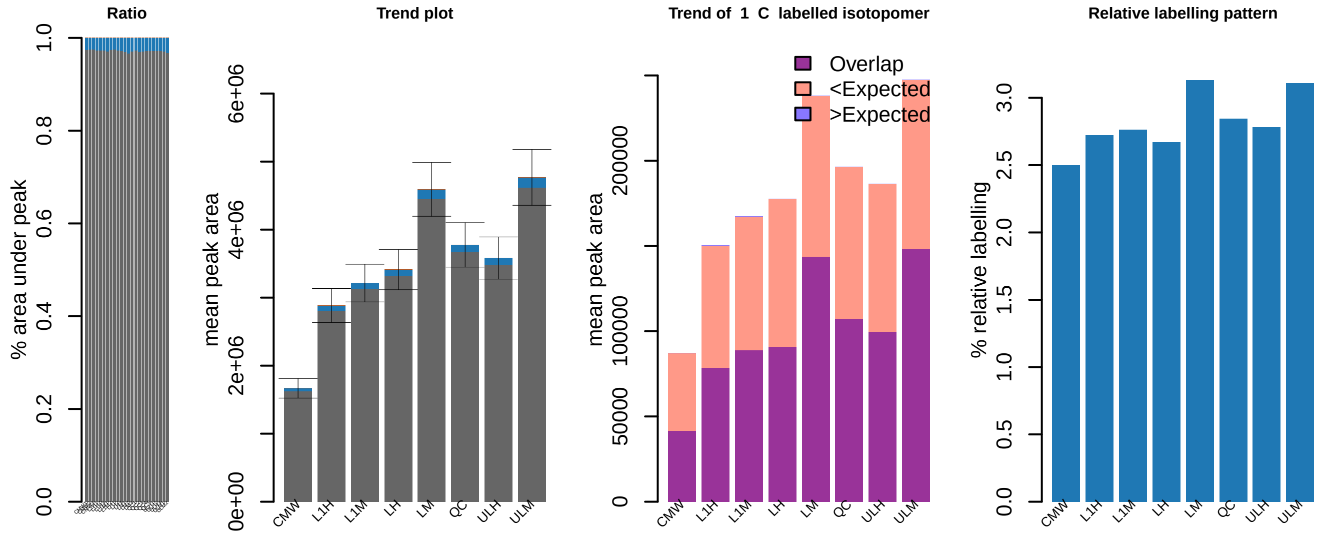

Ectoine

Formula: C6H10N2O2 Mass: 142.074 Std.RT: 851.7295074 Ion: POS

G1

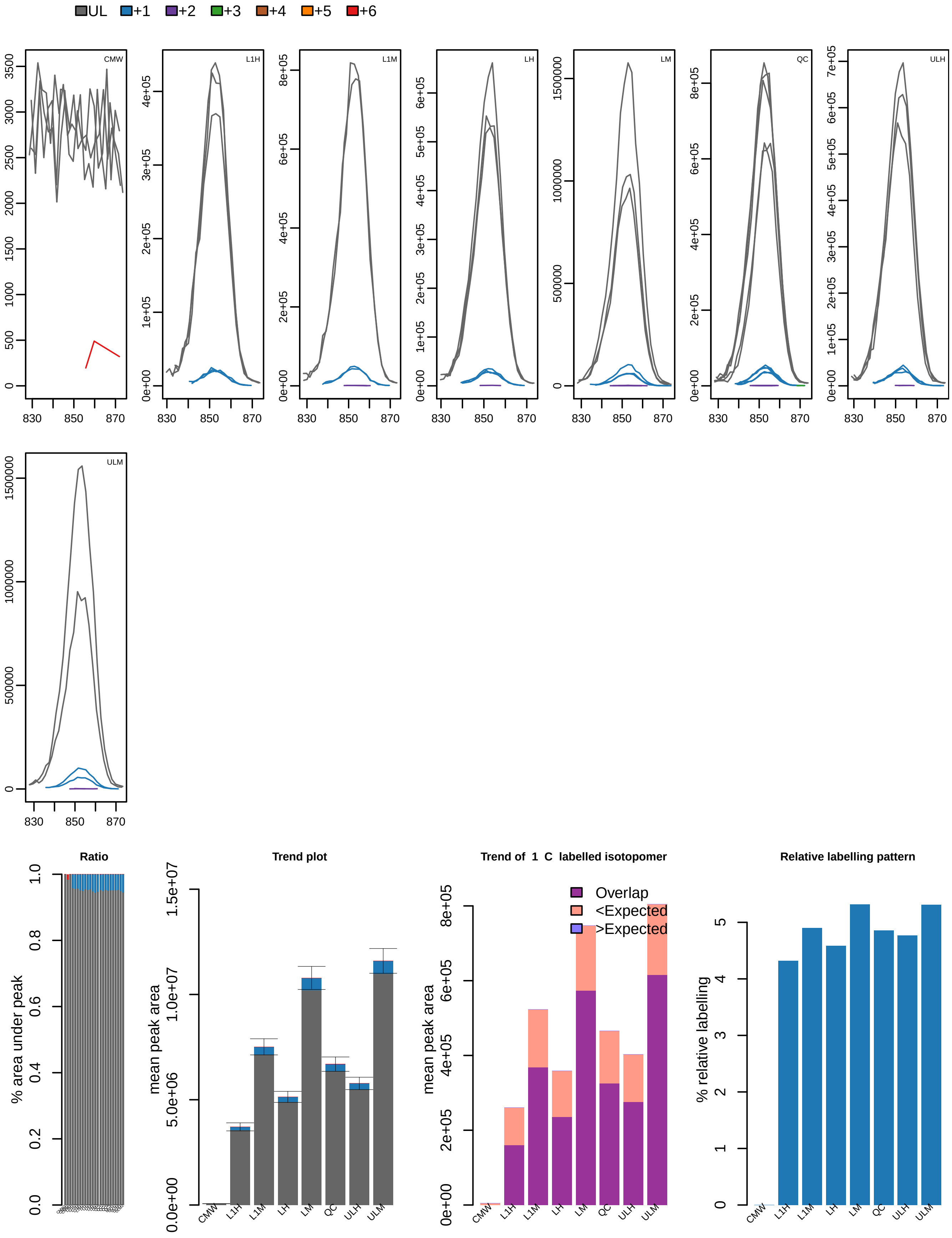

Guanidinoacetate

G1

Formula: C3H7N3O2 Mass: 117.054 Std.RT: 1034.5316622 Ion: POS

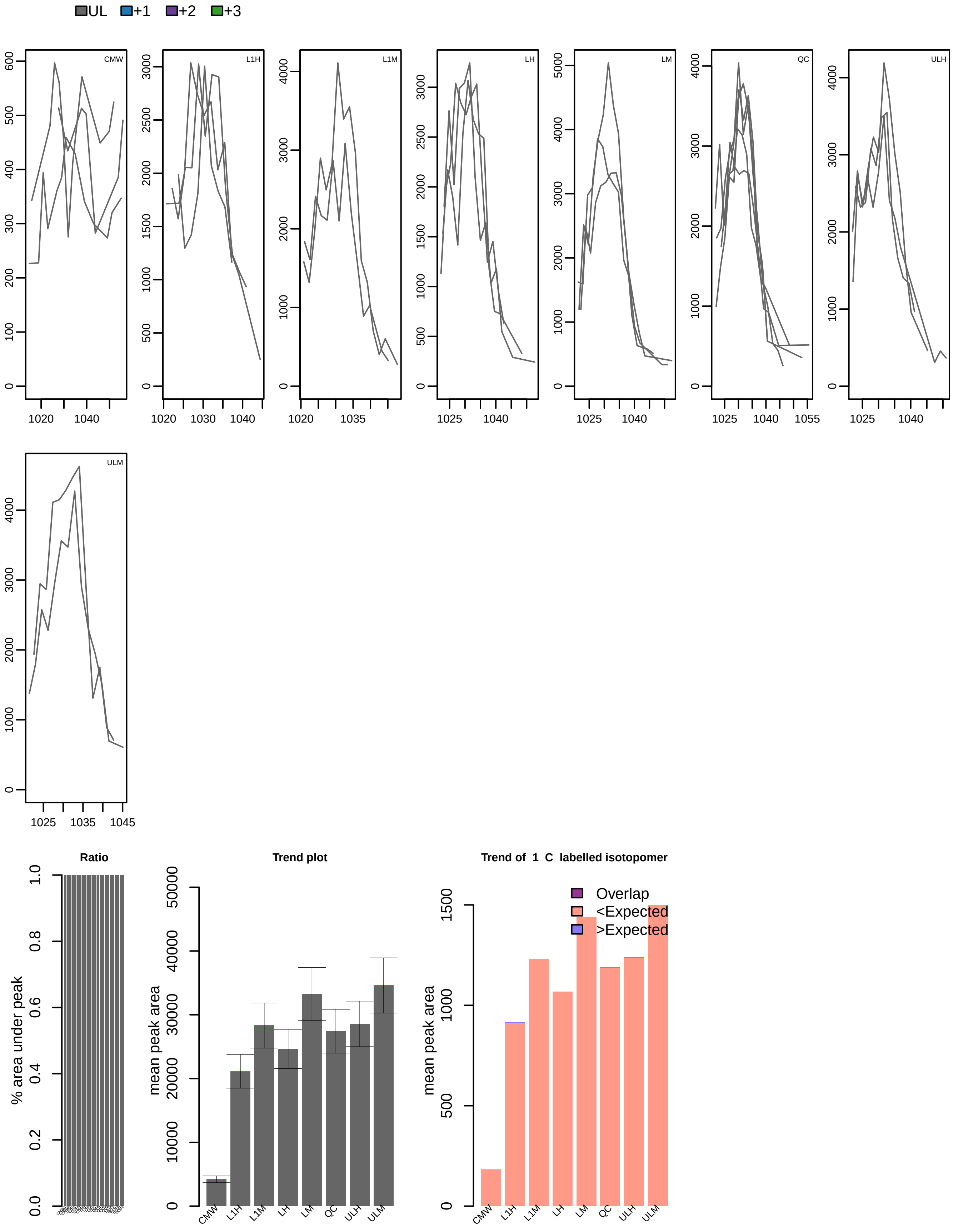

Creatine

Formula: C4H9N3O2 Mass: 131.069 Std.RT: 961.8774972 Ion: POS

G1

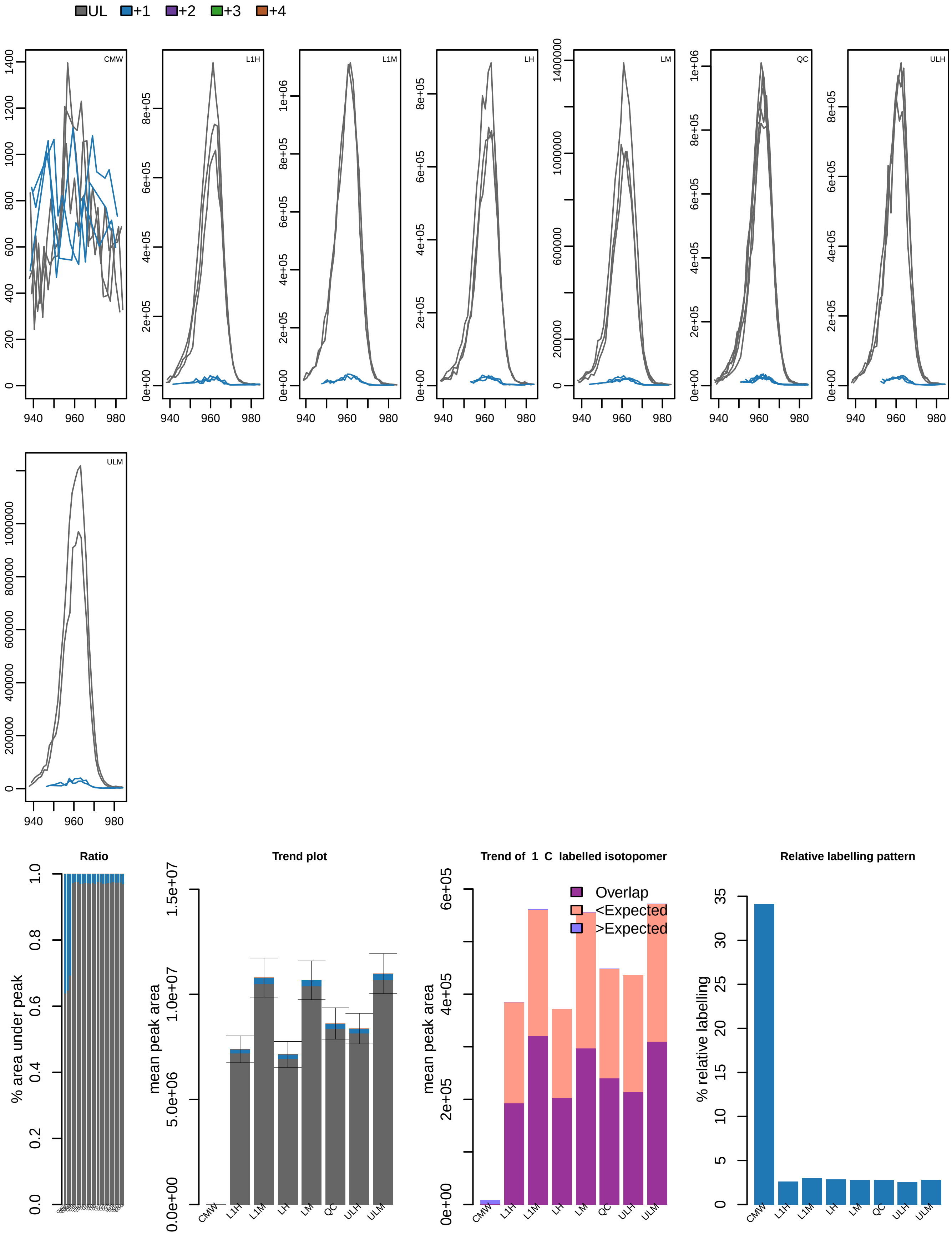

L−Cystathionine

Formula: C7H14N2O4S Mass: 222.067 Std.RT: 1093.525245 Ion: PC

G1

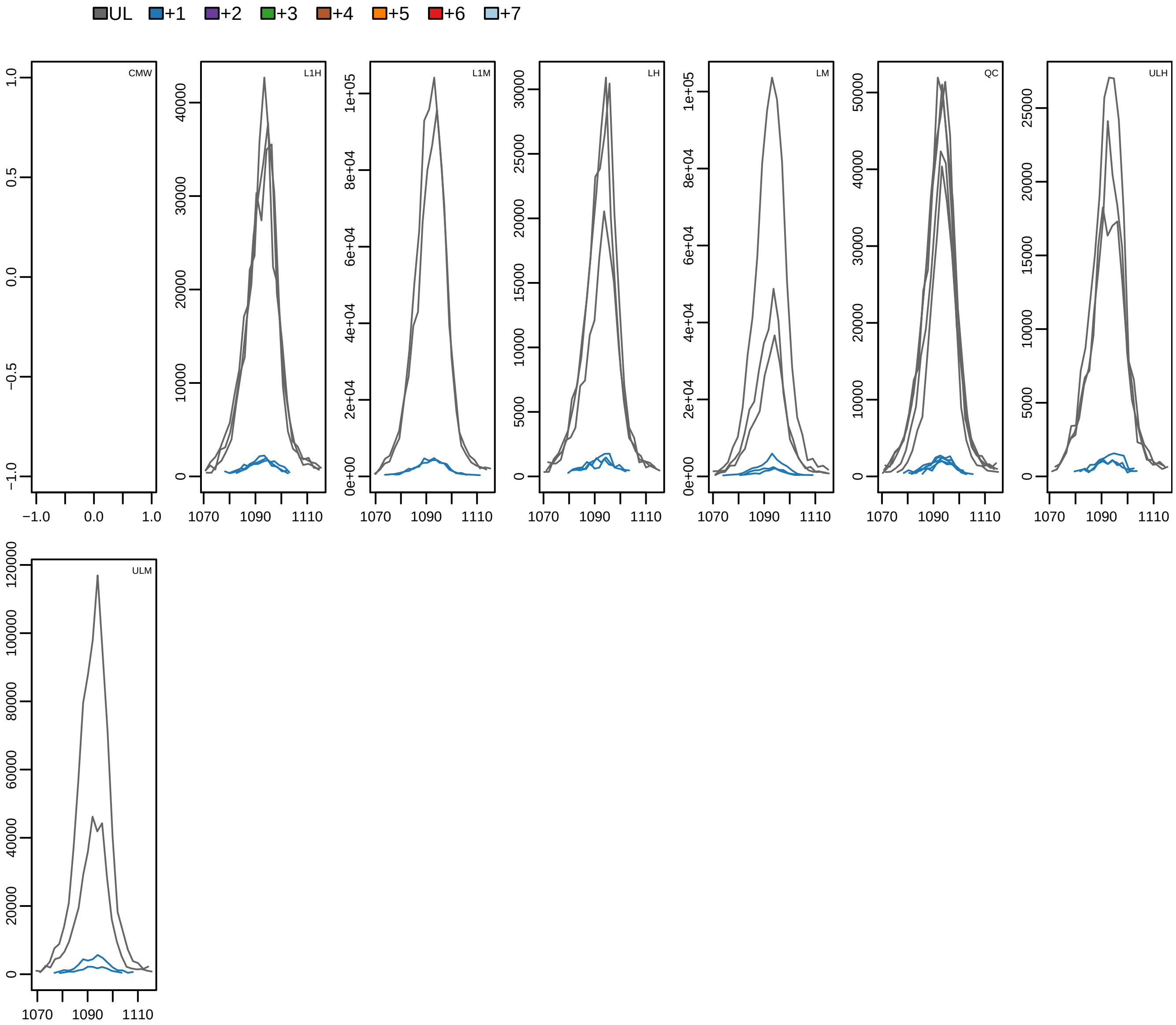

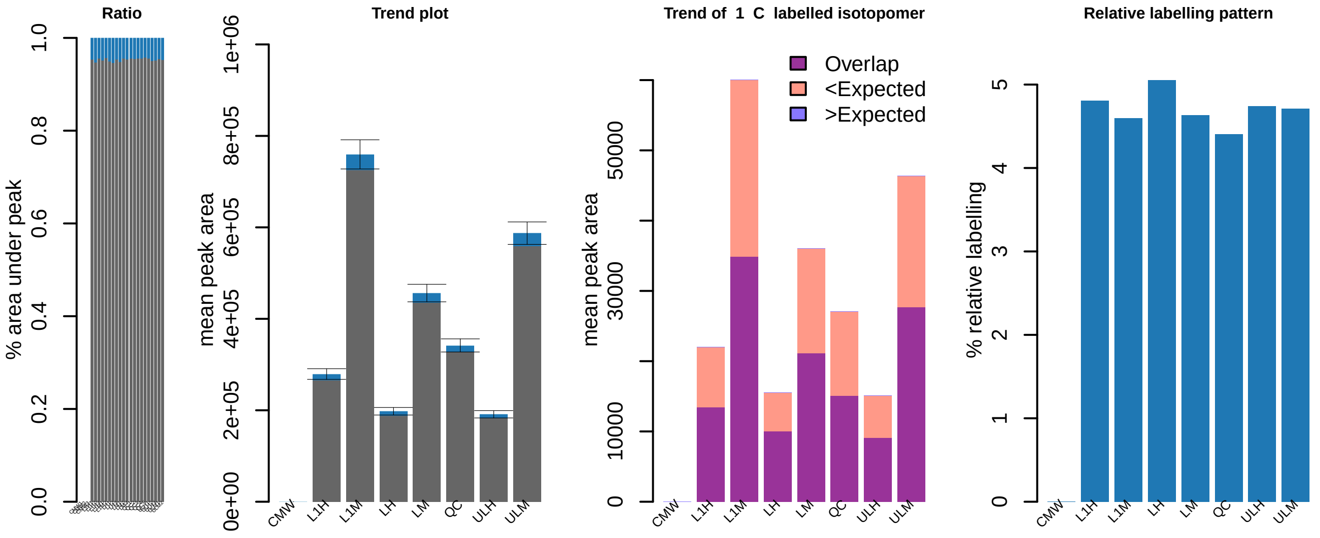

L−Serine

Formula: C3H7NO3 Mass: 105.043 Std.RT: 1018.818756 Ion: POS

G1

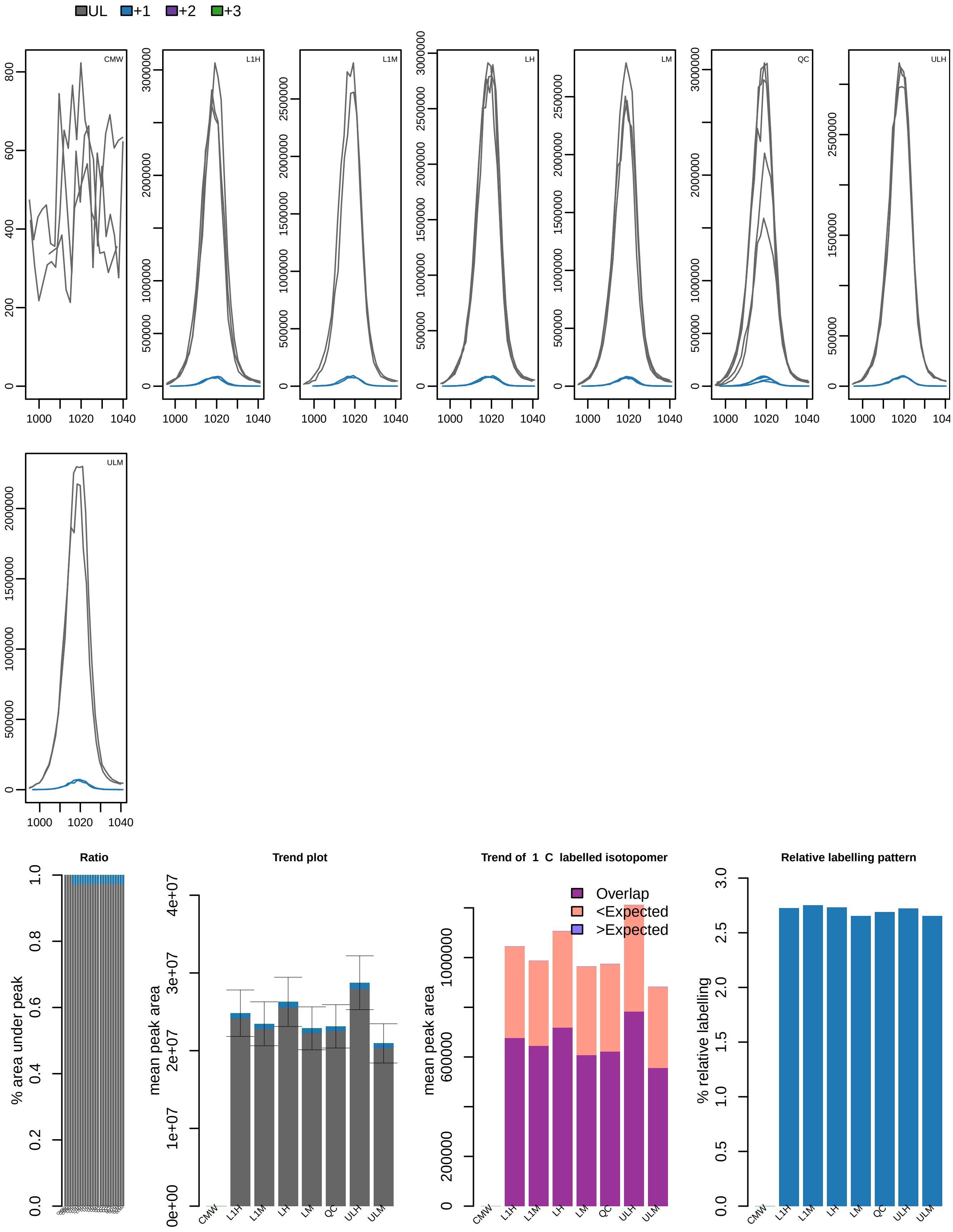

L−Threonine

Formula: C4H9NO3 Mass: 119.058 Std.RT: 929.1547548 Ion: POS

G1

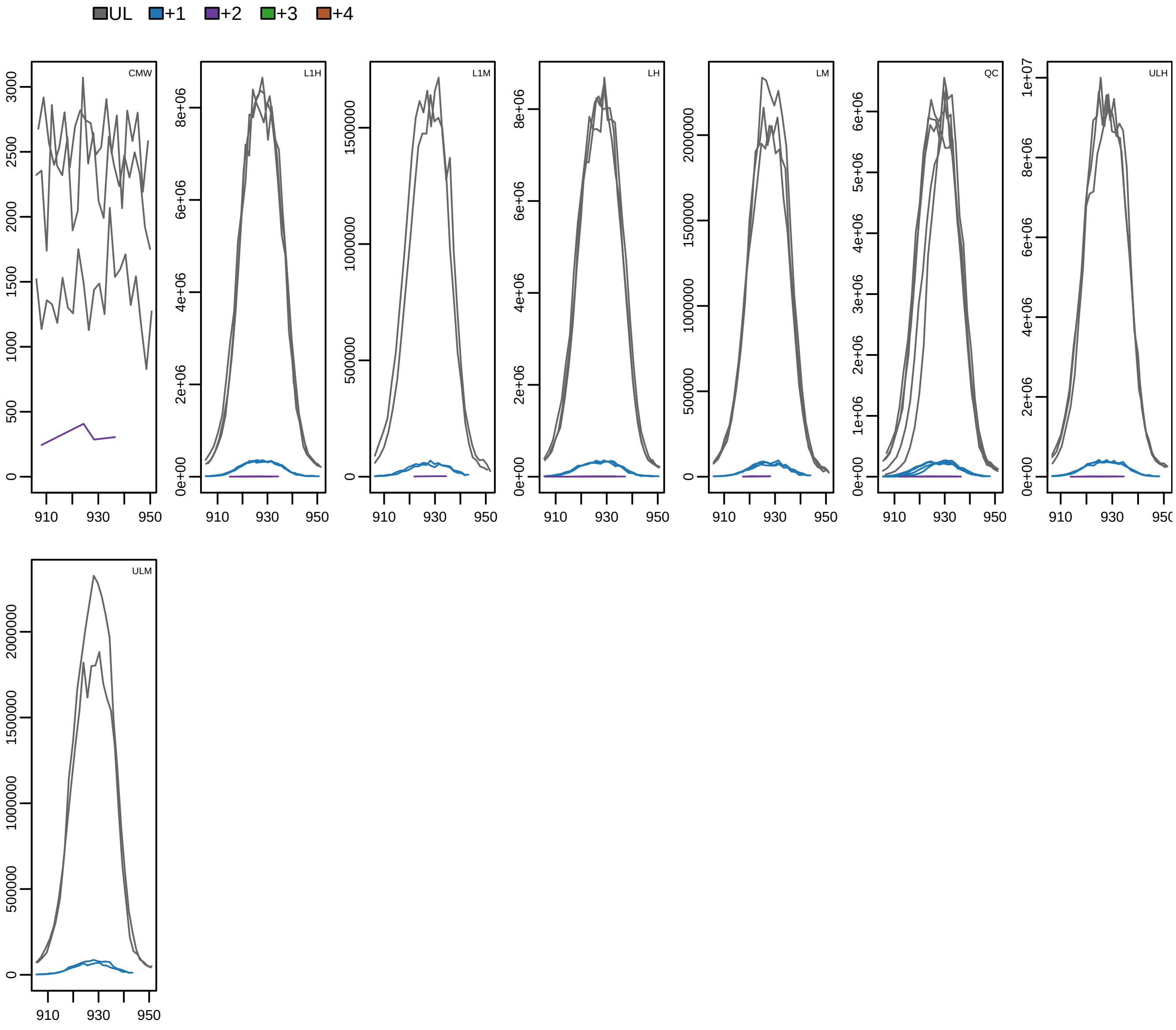

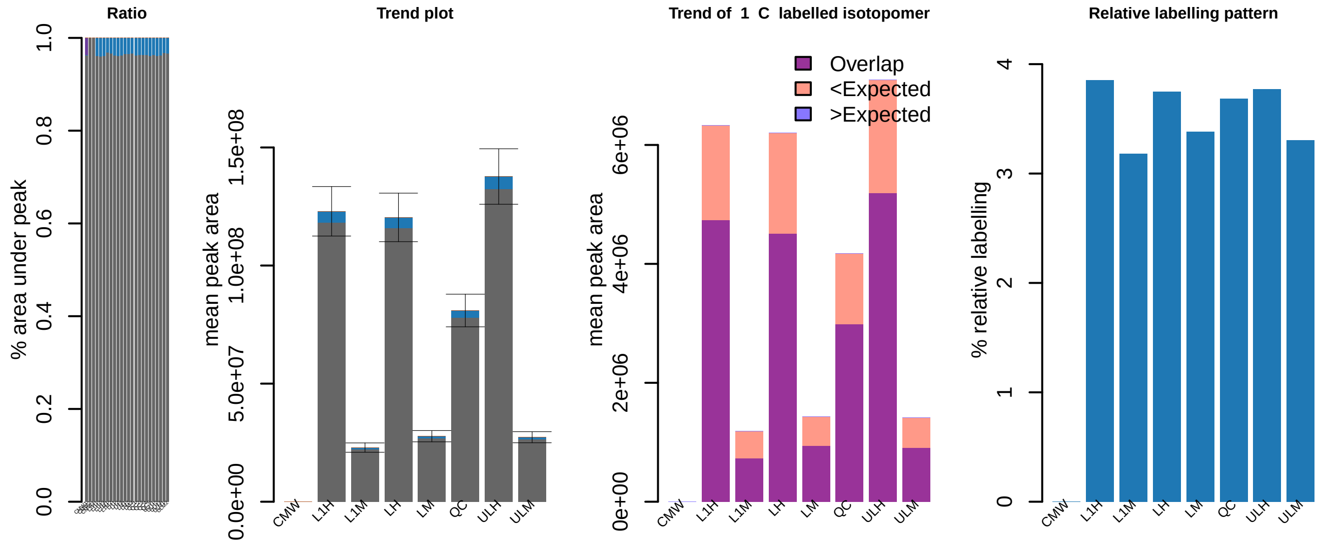

N(pi)−Methyl−L−histidine

Formula: C7H11N3O2 Mass: 169.085 Std.RT: 810.5020014 Ion: POS

G1

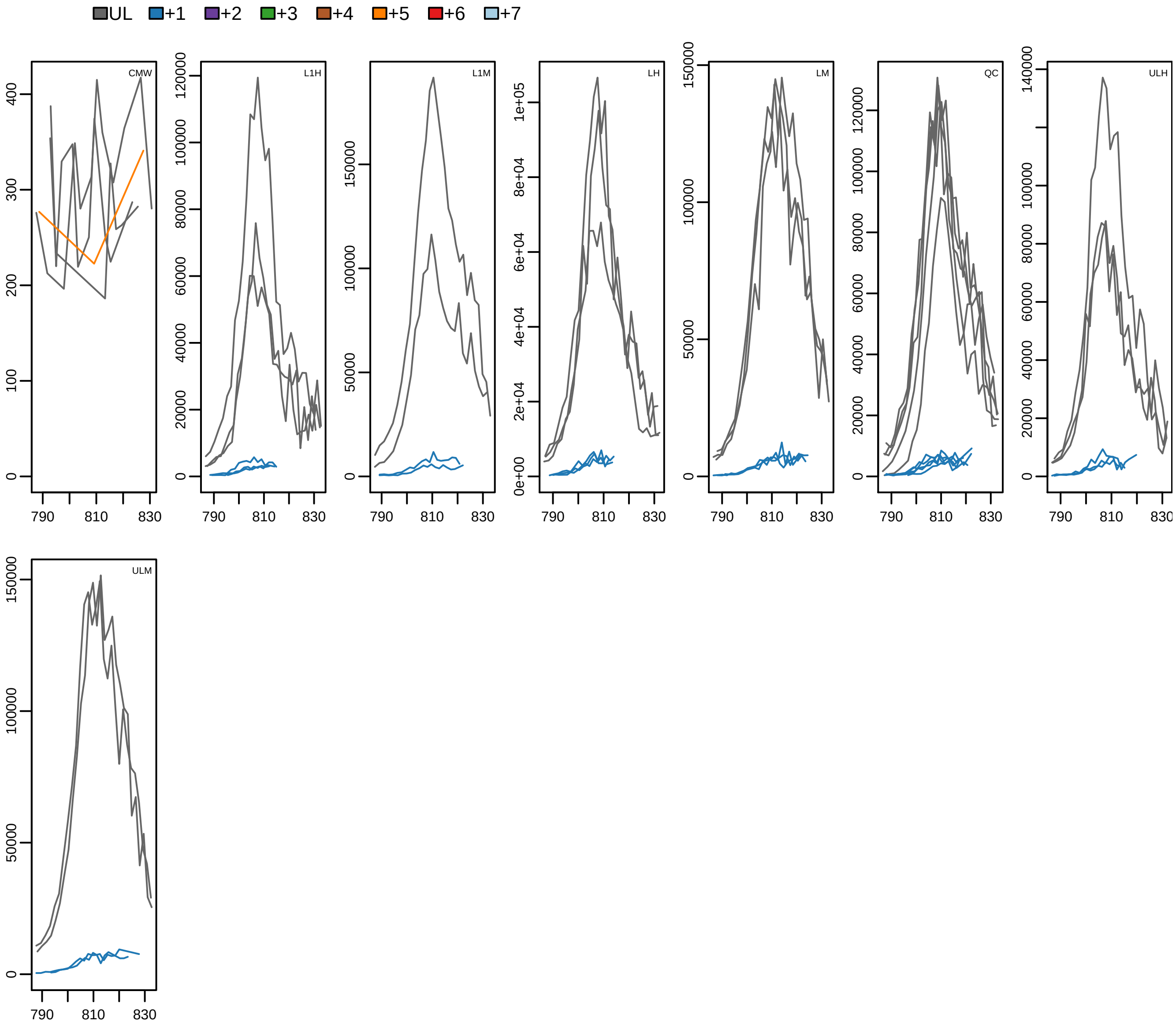

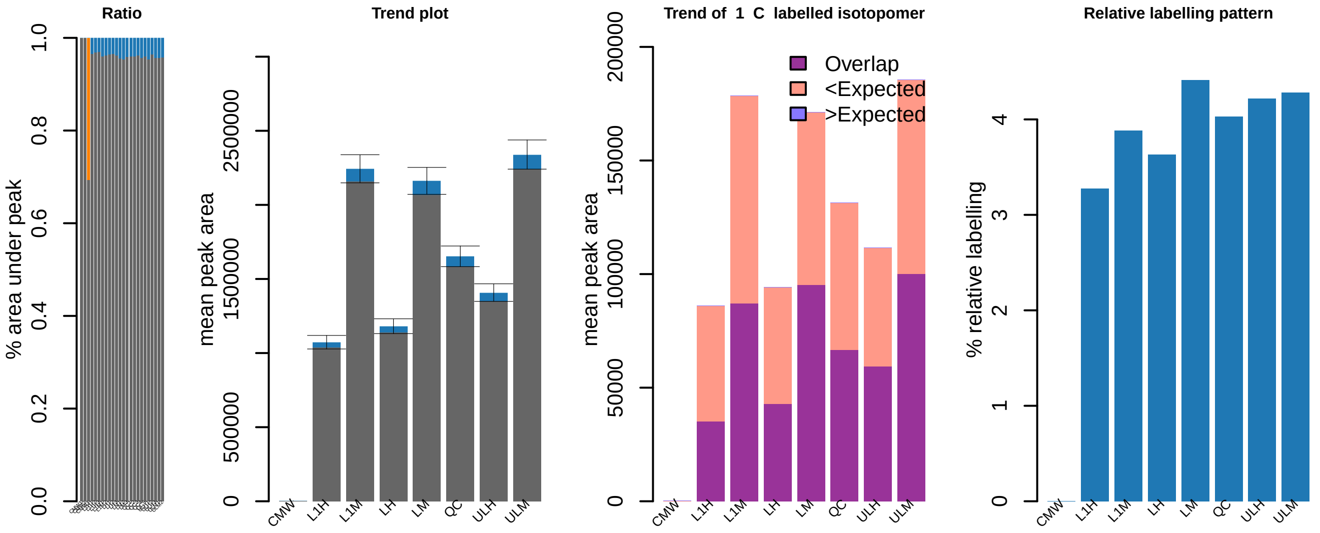

Hydantoin−5−propionate

Formula: C6H8N2O4 Mass: 172.048 Std.RT: 629.045718 Ion: POS

G1

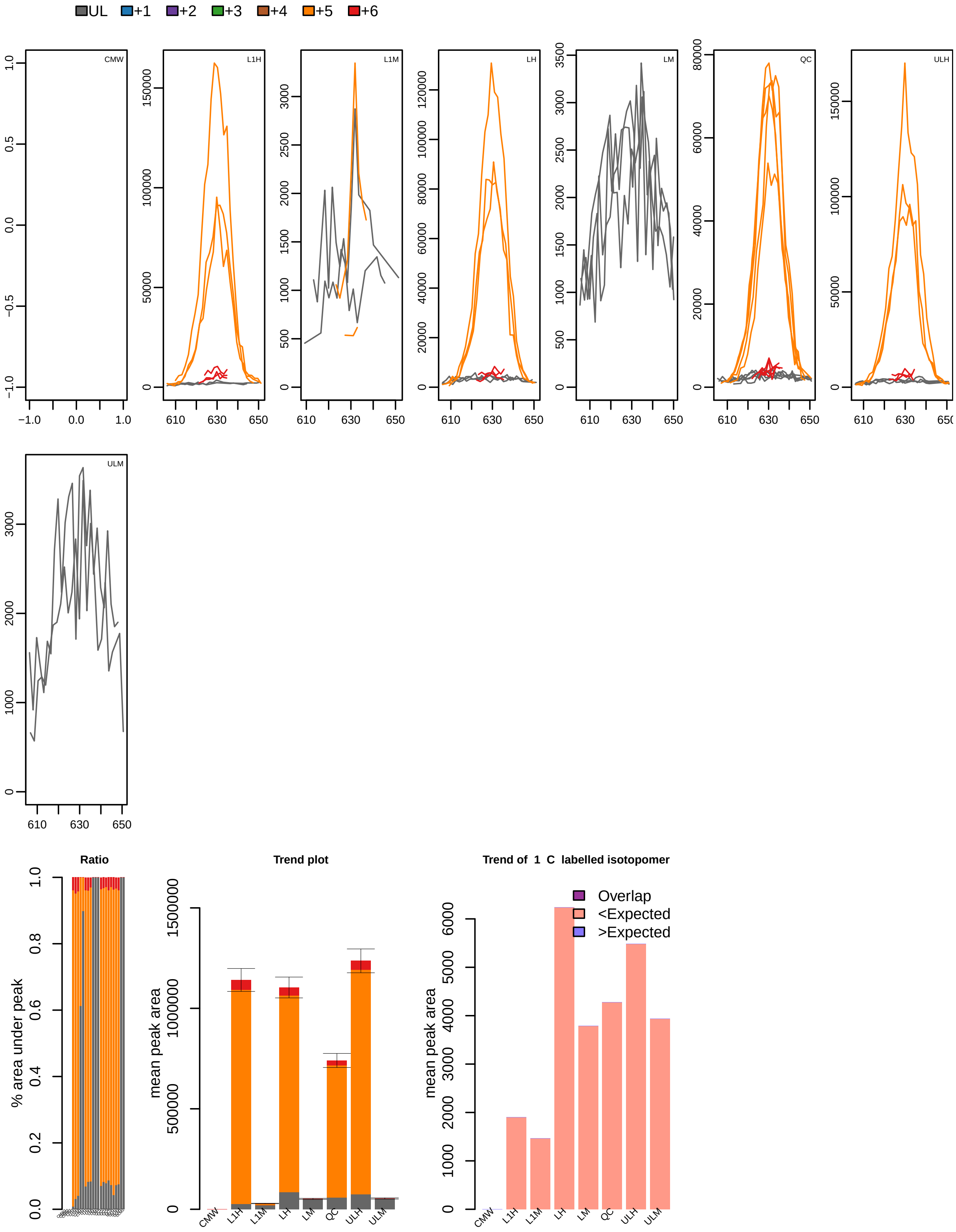

Imidazol−5−yl−pyruvate

Formula: C6H6N2O3 Mass: 154.038 Std.RT: 625.1954316 Ion: POS

G1

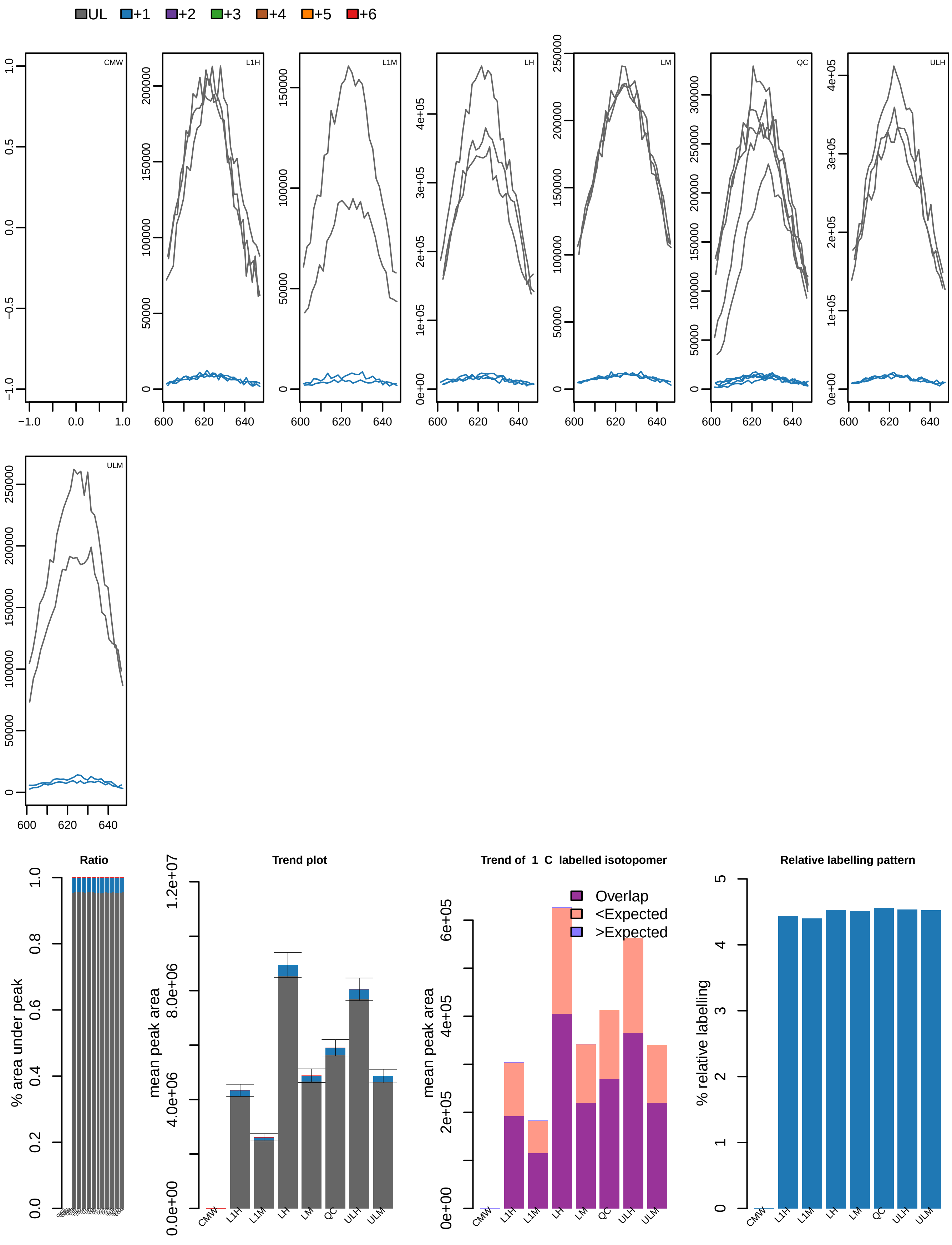

4−Imidazolone−5−propanoate

Formula: C6H8N2O3 Mass: 156.053 Std.RT: 685.9757166 Ion: POS

G1

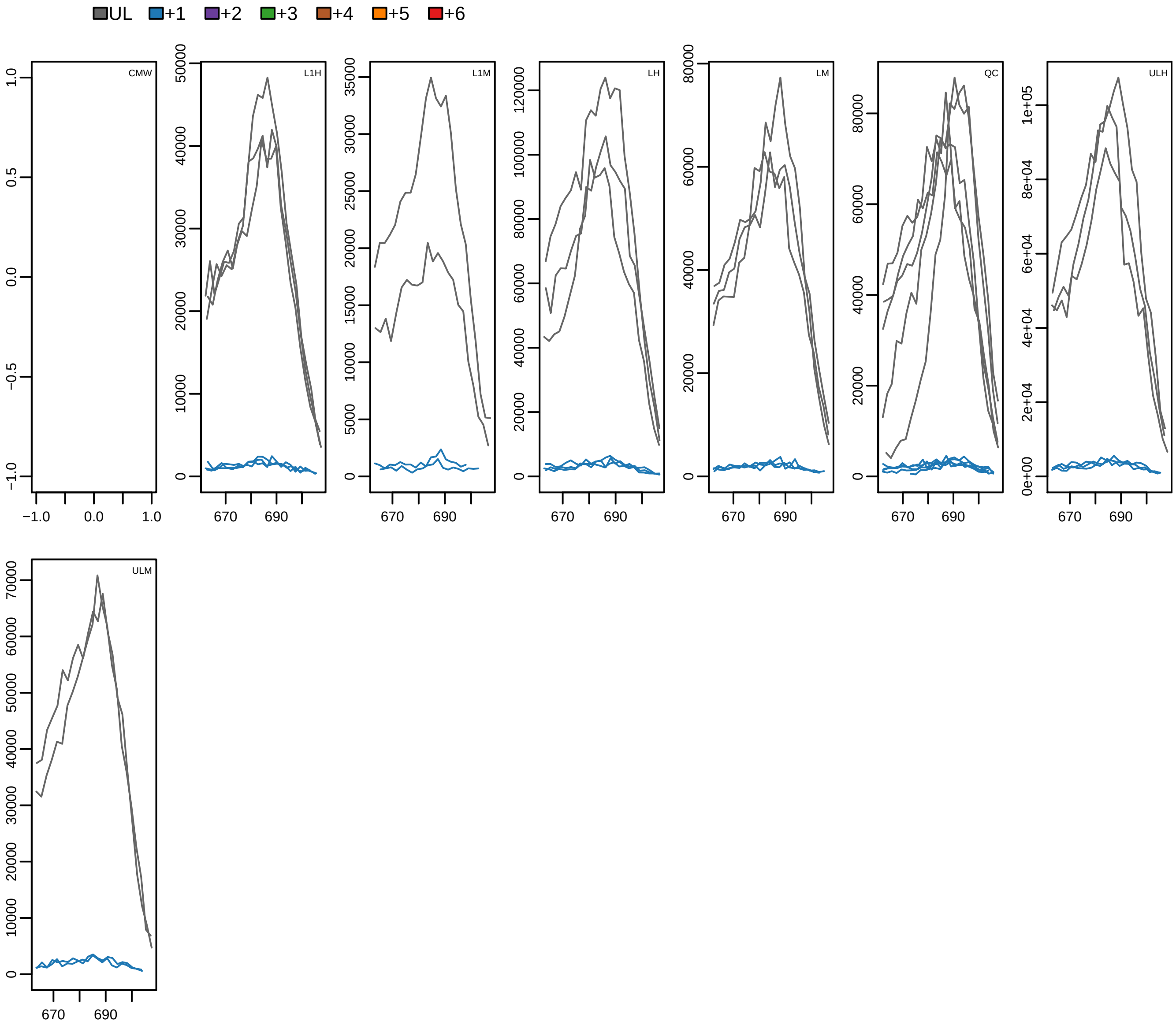

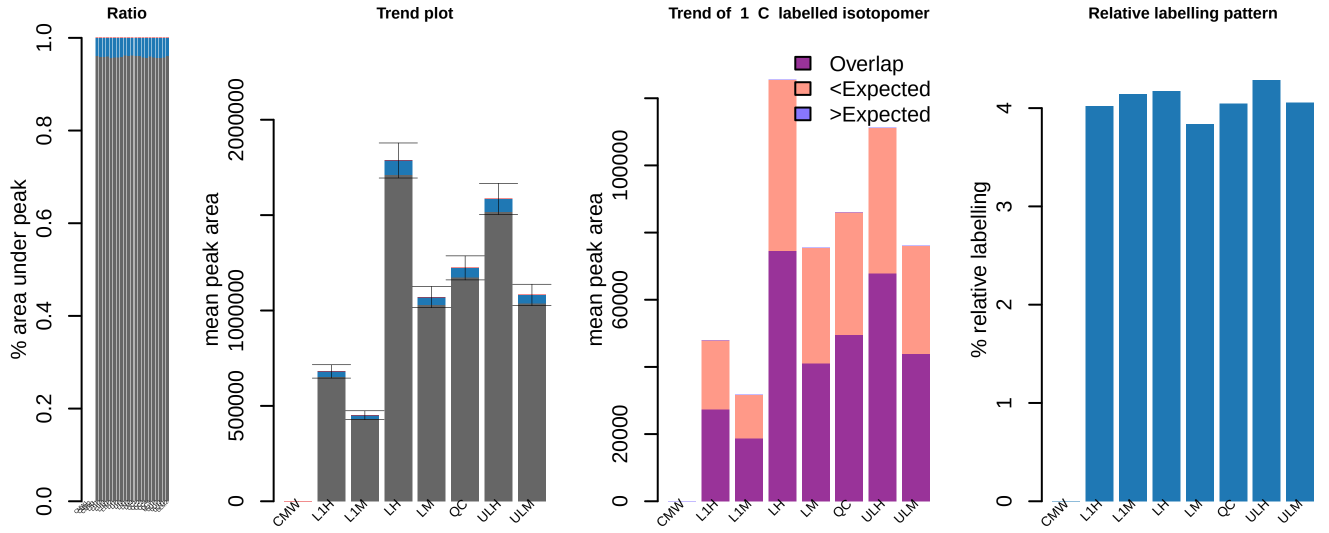

Imidazole−4−acetate

Formula: C5H6N2O2 Mass: 126.043 Std.RT: 718.4215014 Ion: POS

G1

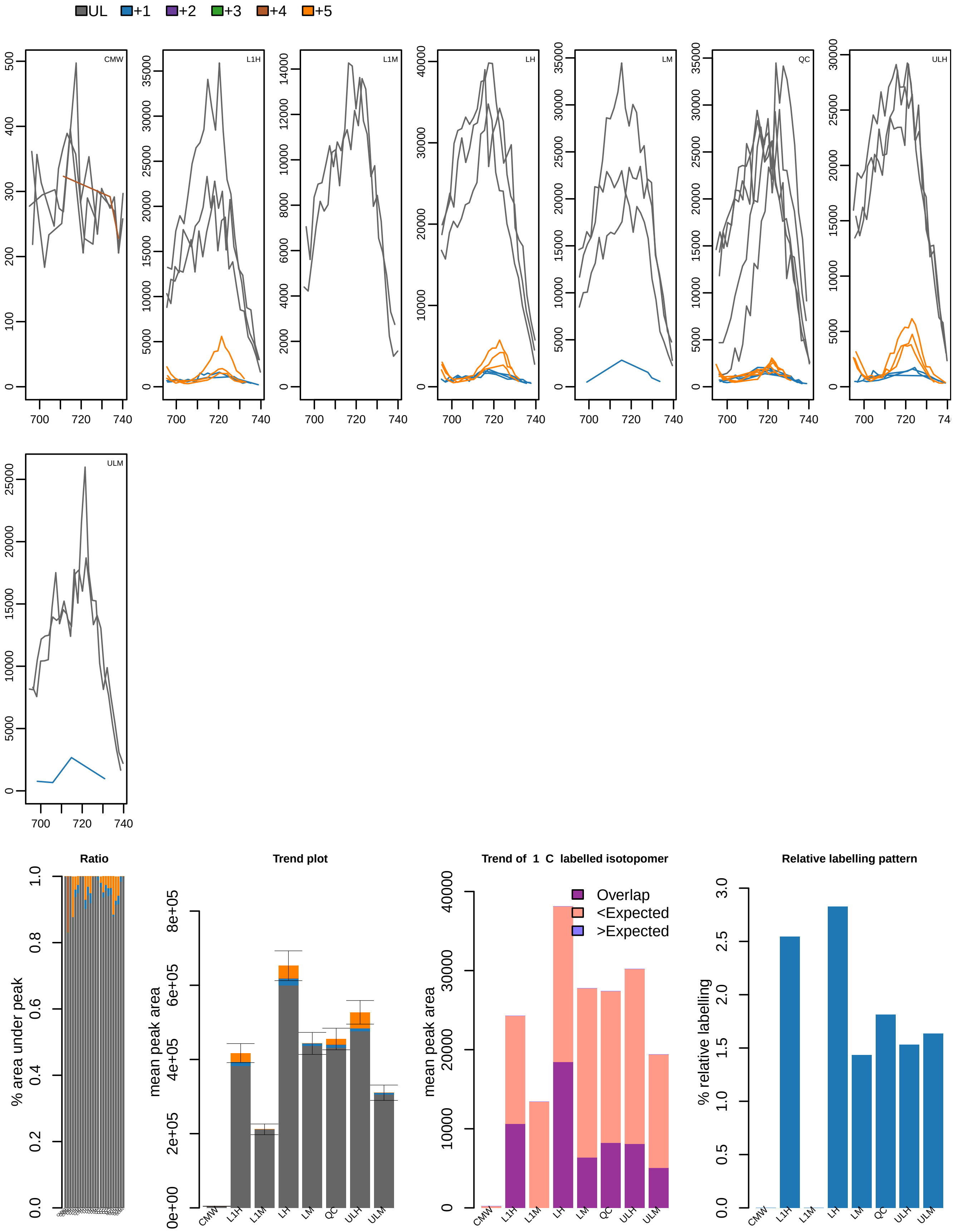

Urocanate

Formula: C6H6N2O2 Mass: 138.043 Std.RT: 433.48599882 Ion: POS

G1

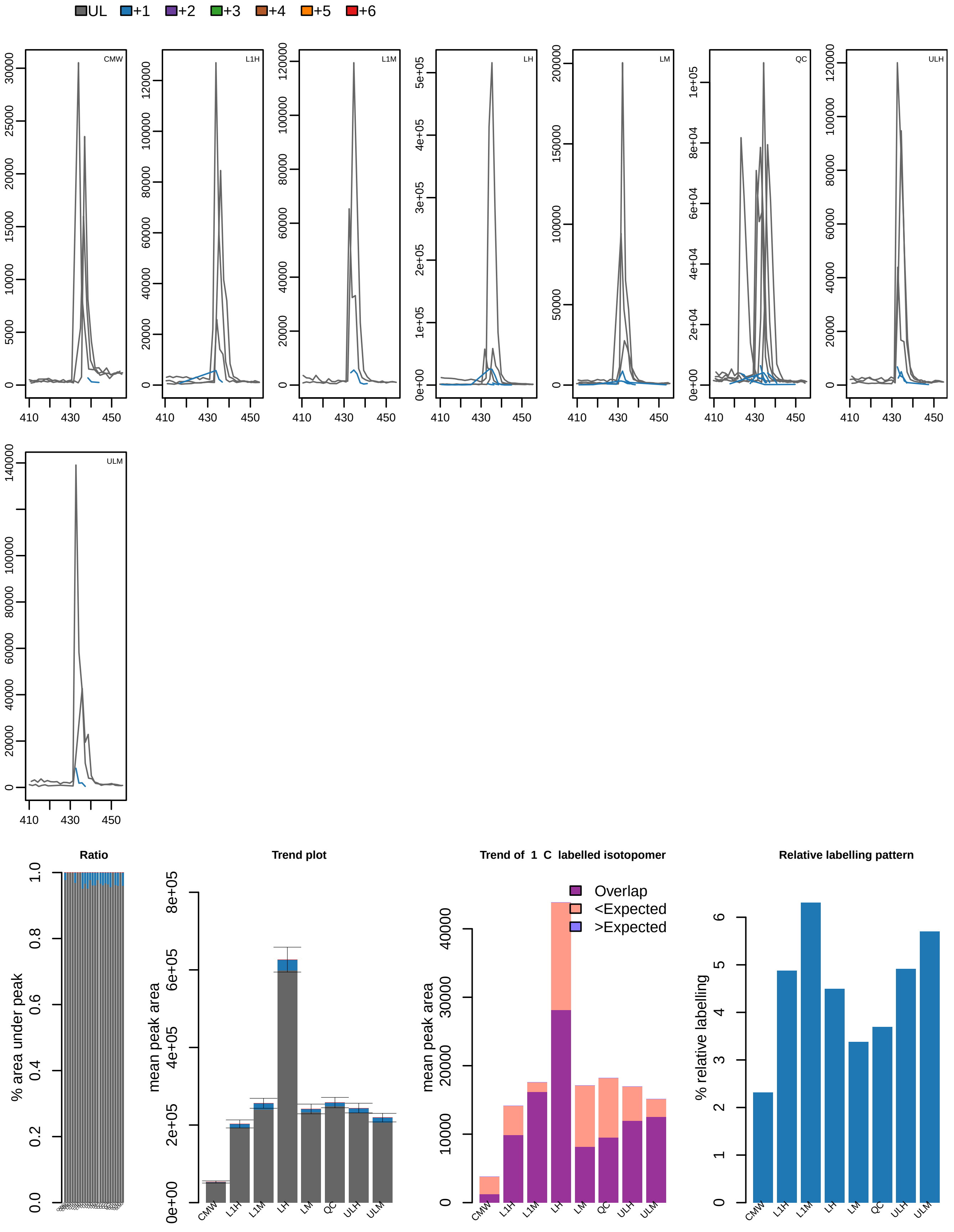

L−Histidine

Formula: C6H9N3O2 Mass: 155.069 Std.RT: 960.2597454 Ion: POS

G1

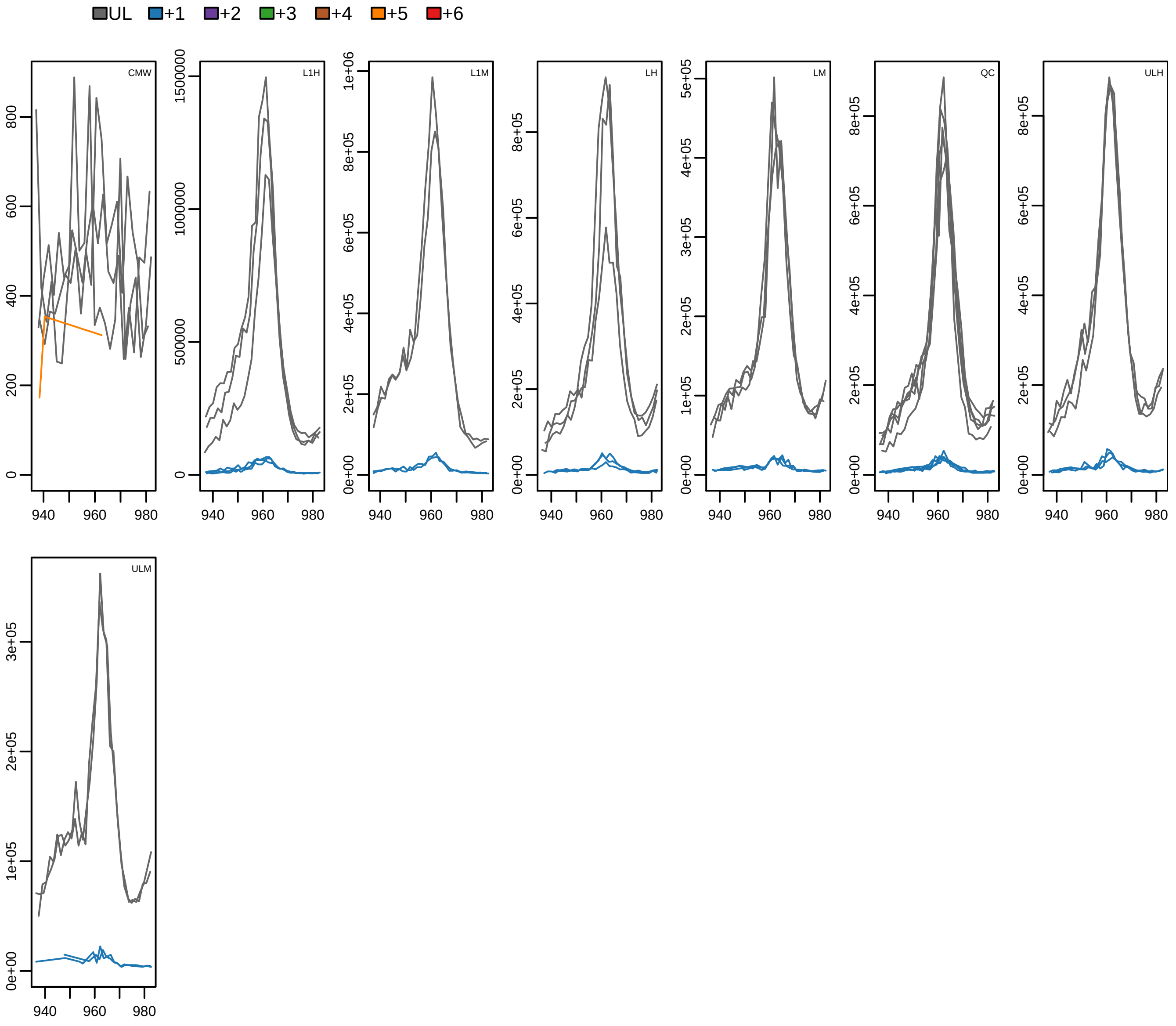

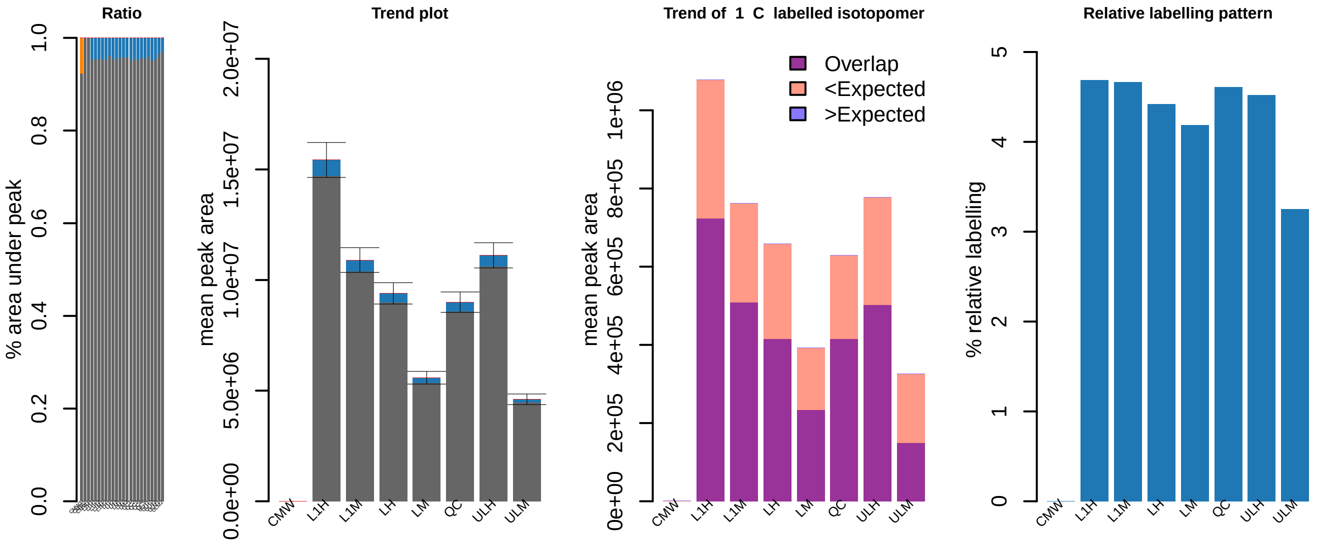

L−Histidine

Formula: C6H9N3O2 Mass: 155.069 Std.RT: 960.2597454 Ion: POS

G2

UL +1 +2 +3 +4 +5 +6

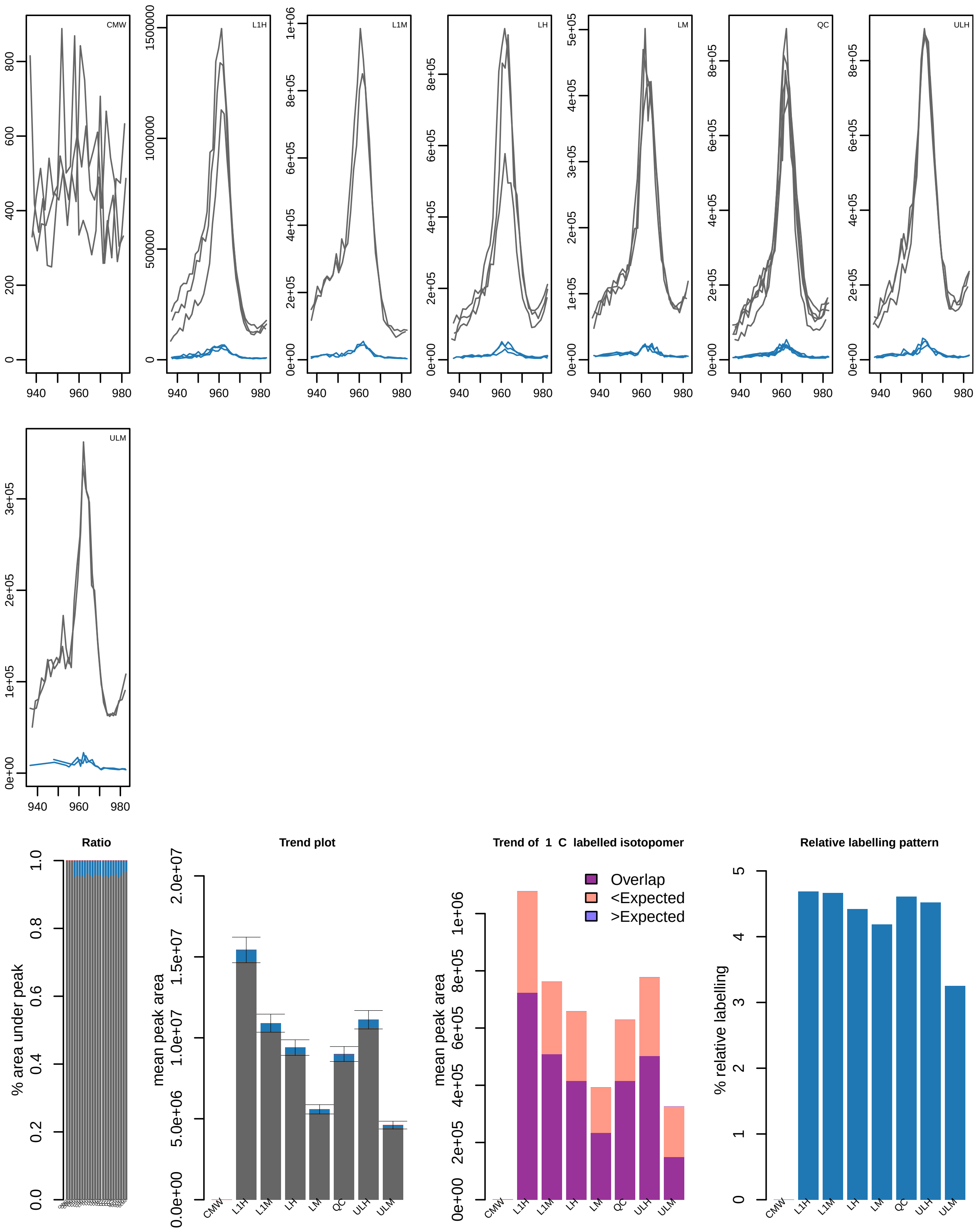

N2−Acetyl−L−aminoadipate

Formula: C8H13NO5 Mass: 203.079 Std.RT: 978.2703246 Ion: POS

G1

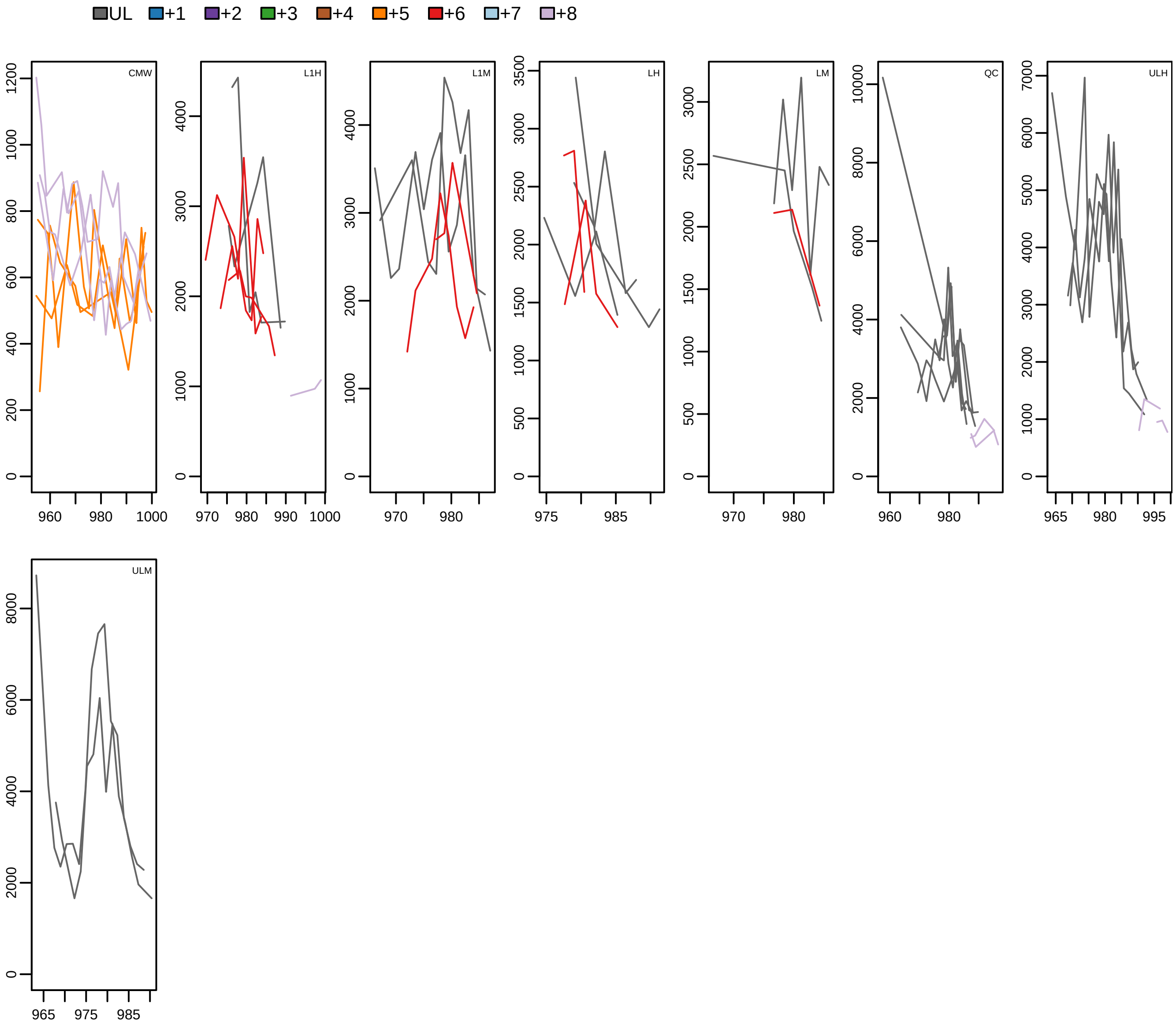

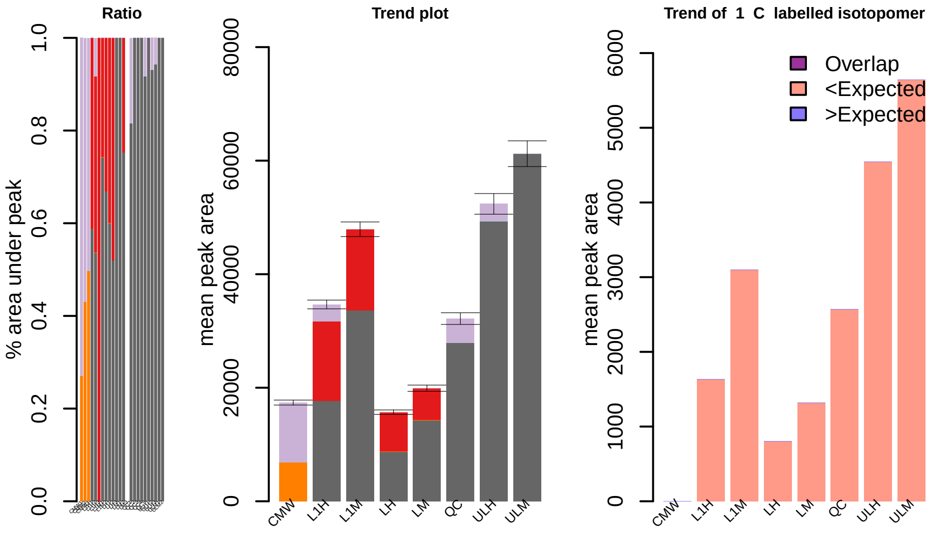

L−Lysine

Formula: $C6H14N2O2$ Mass: 146.106 Std.RT: 1569.58875 Ion: POS

G1

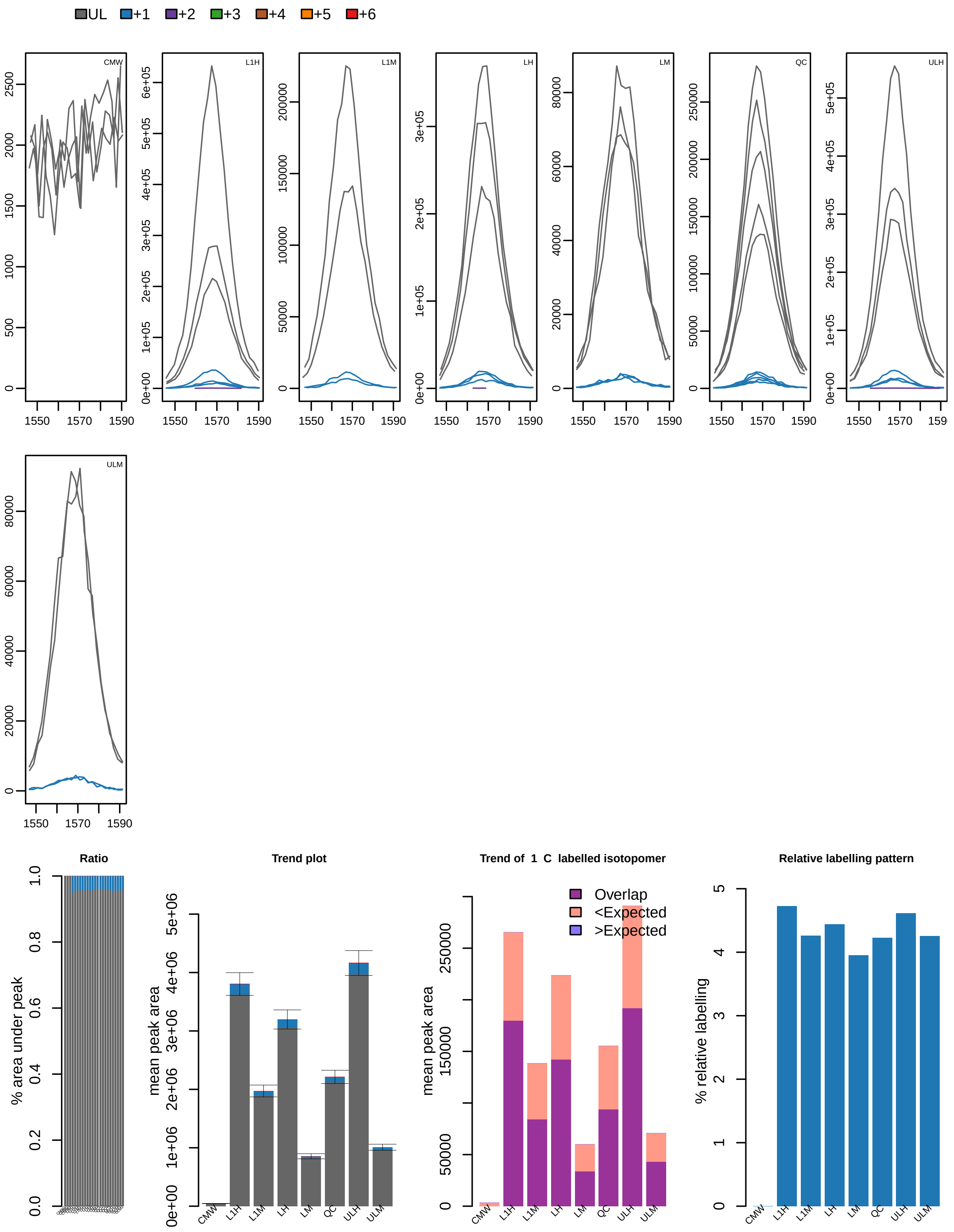

5−Acetamidopentanoate

Formula: C7H13NO3 Mass: 159.09 Std.RT: 437.85529068 Ion: POS

G1

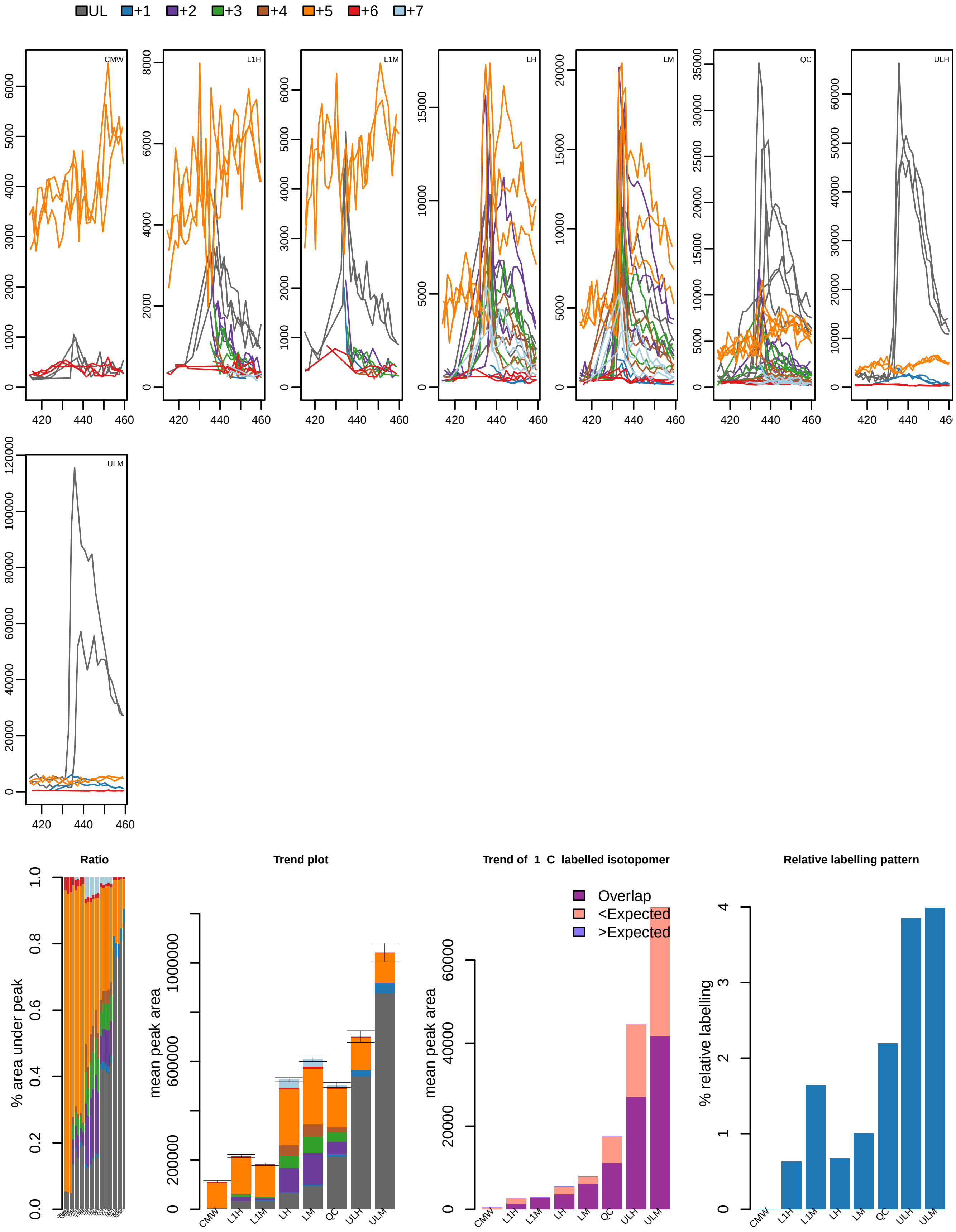

N6−Acetyl−N6−hydroxy−L−lysine

Formula: C8H16N2O4 Mass: 204.111 Std.RT: 1071.351429 Ion: POS

G1

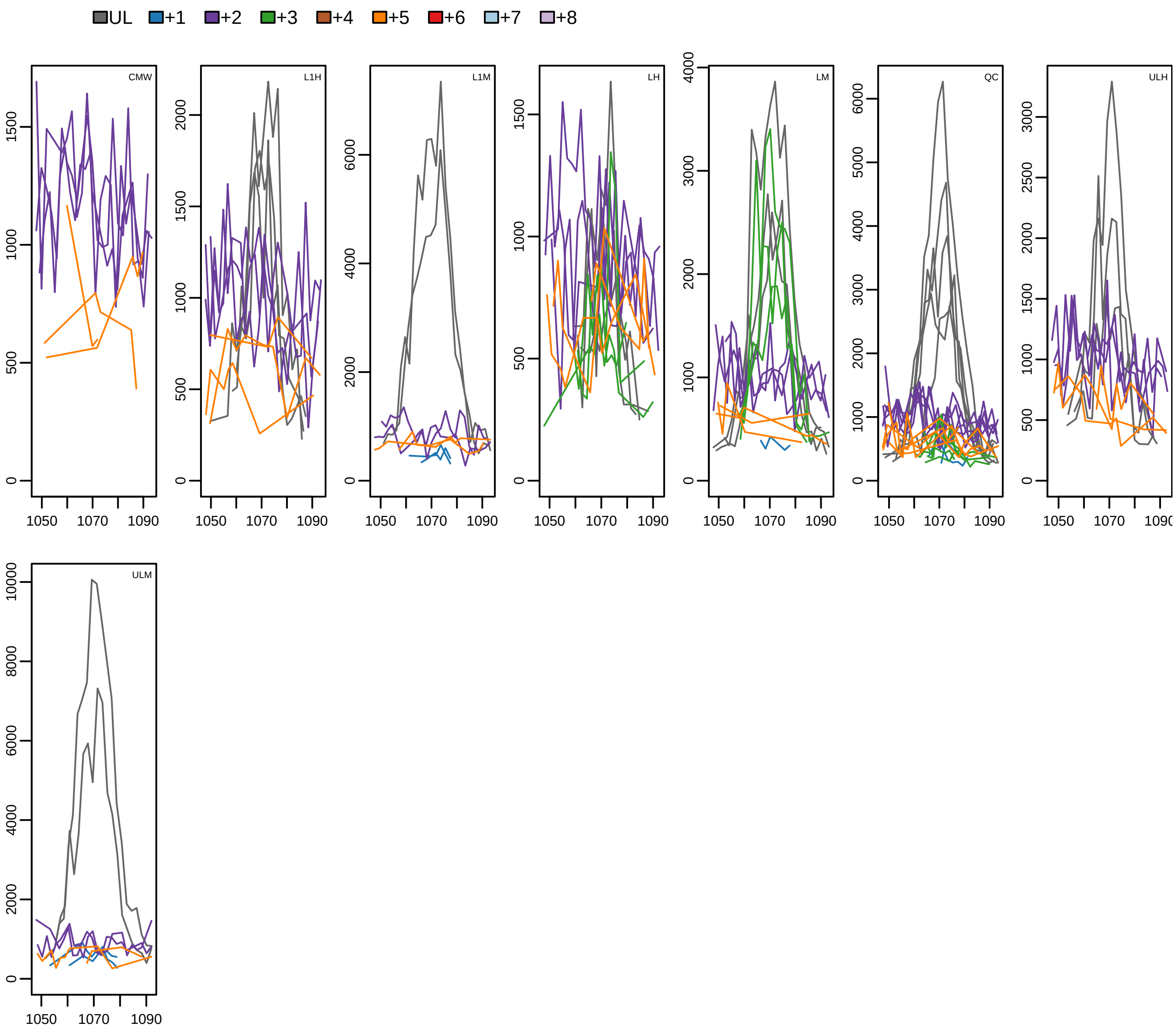

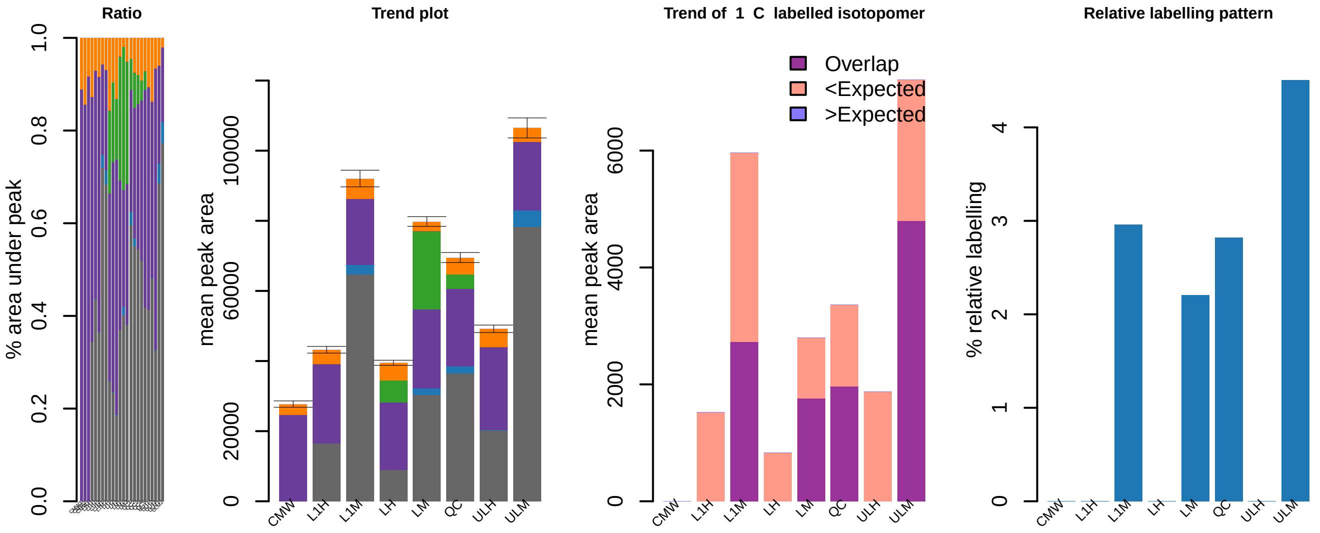

N6−Acetyl−L−lysine

Formula: C8H16N2O3 Mass: 188.116 Std.RT: 836.3650434 Ion: POS

G1

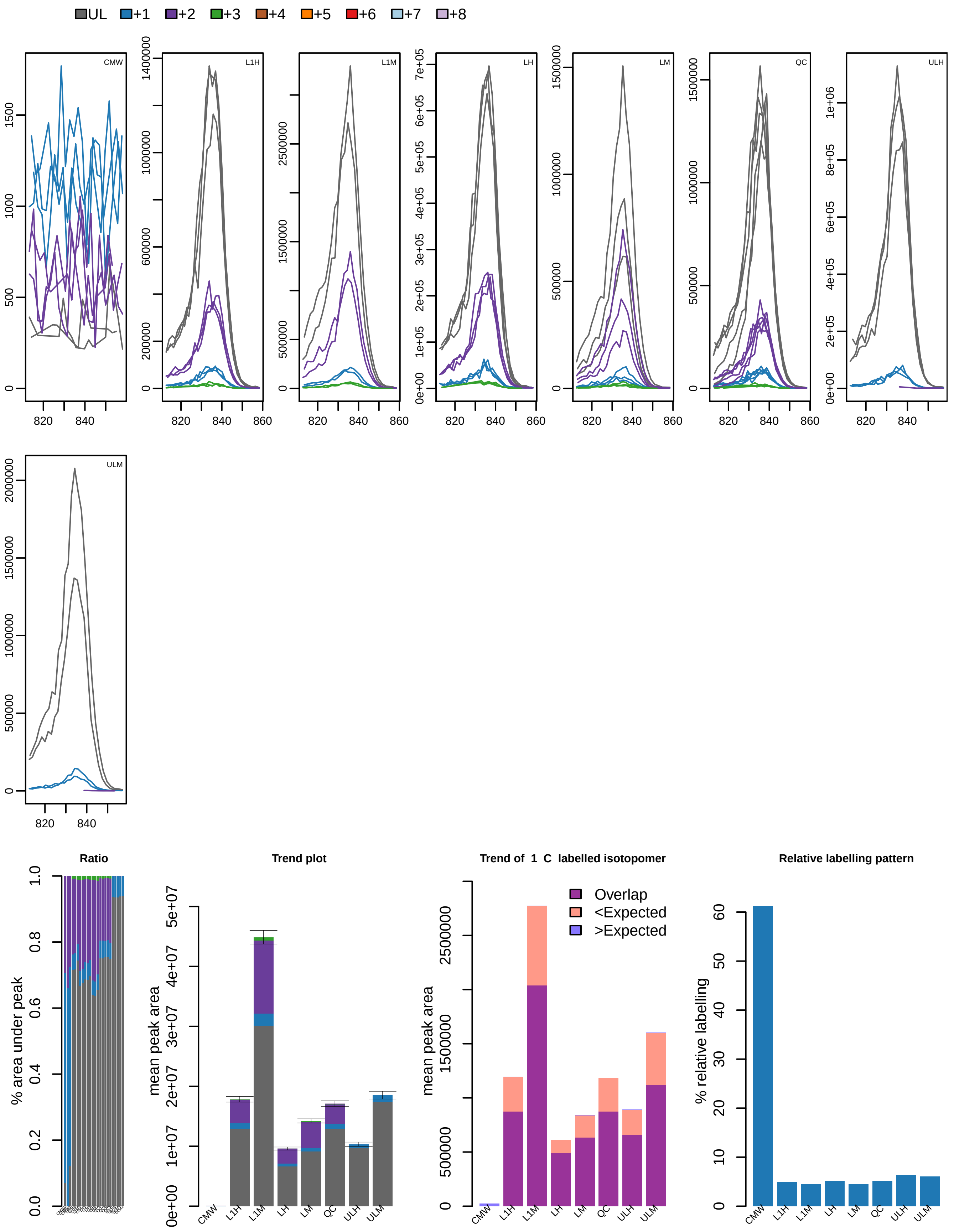

6−Amino−2−oxohexanoate

Formula: C6H11NO3 Mass: 145.074 Std.RT: 688.274007 Ion: POS

G1

UL +1 +2 +3 +4 +5 +6

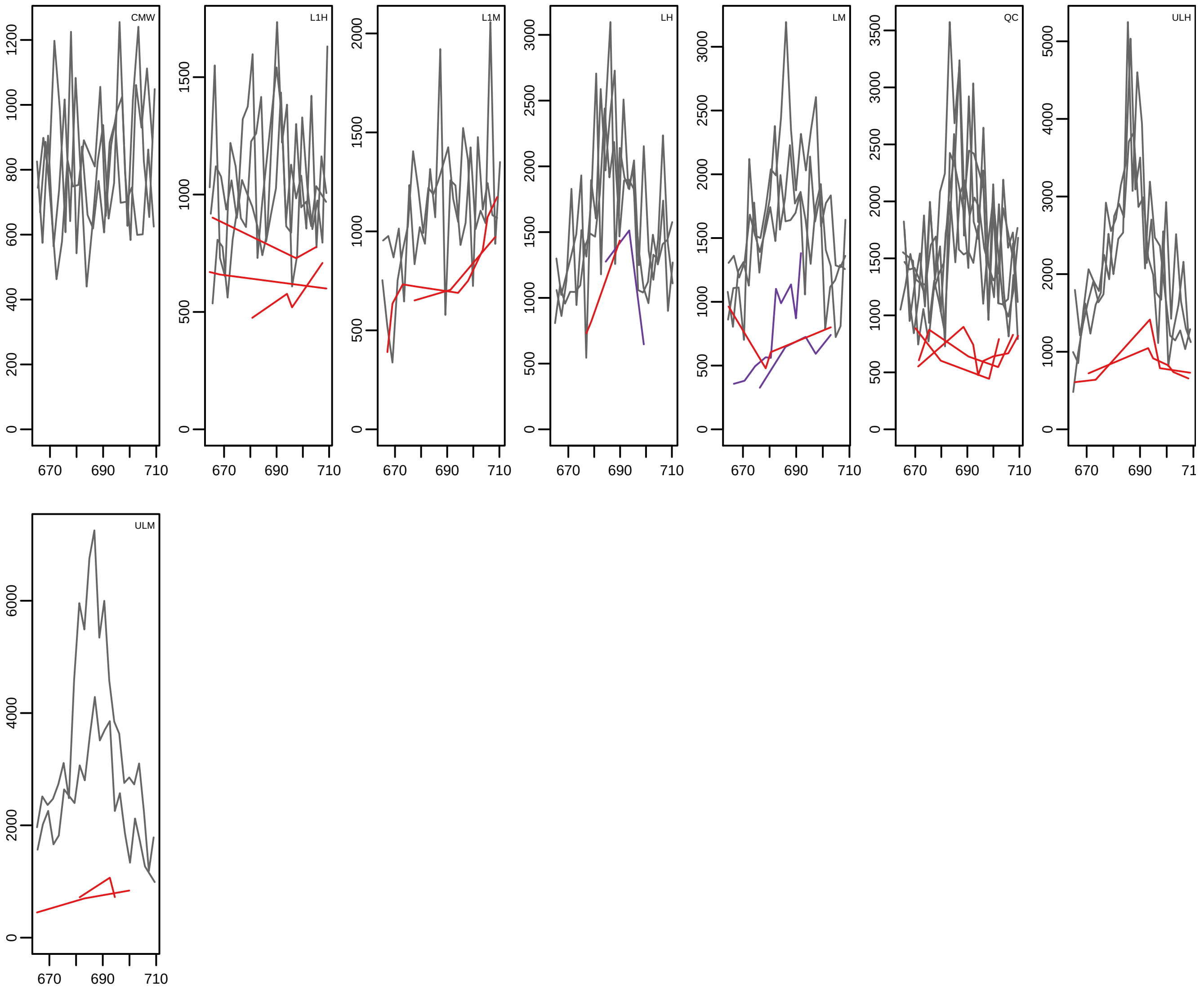

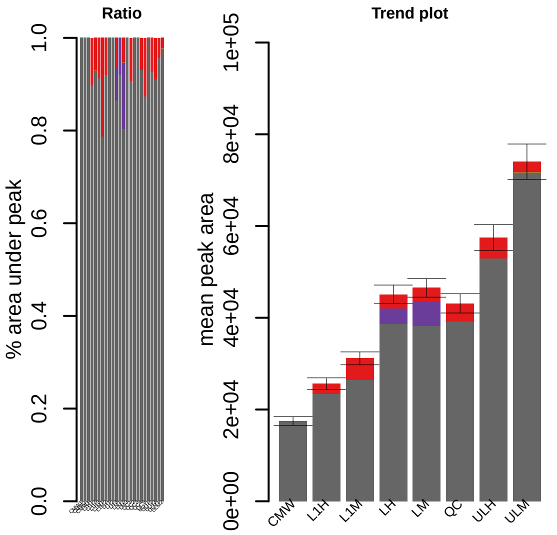

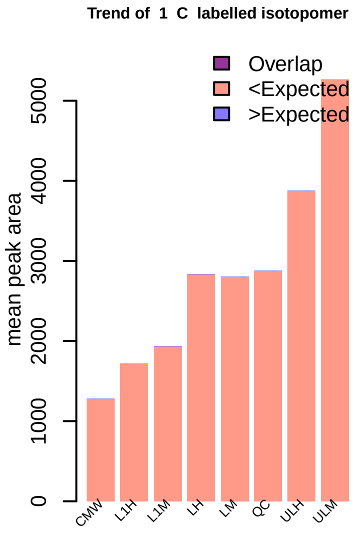

N6,N6,N6−Trimethyl−L−lysine

Formula: C9H20N2O2 Mass: 188.152 Std.RT: 1428.9509448 Ion: PC

G1

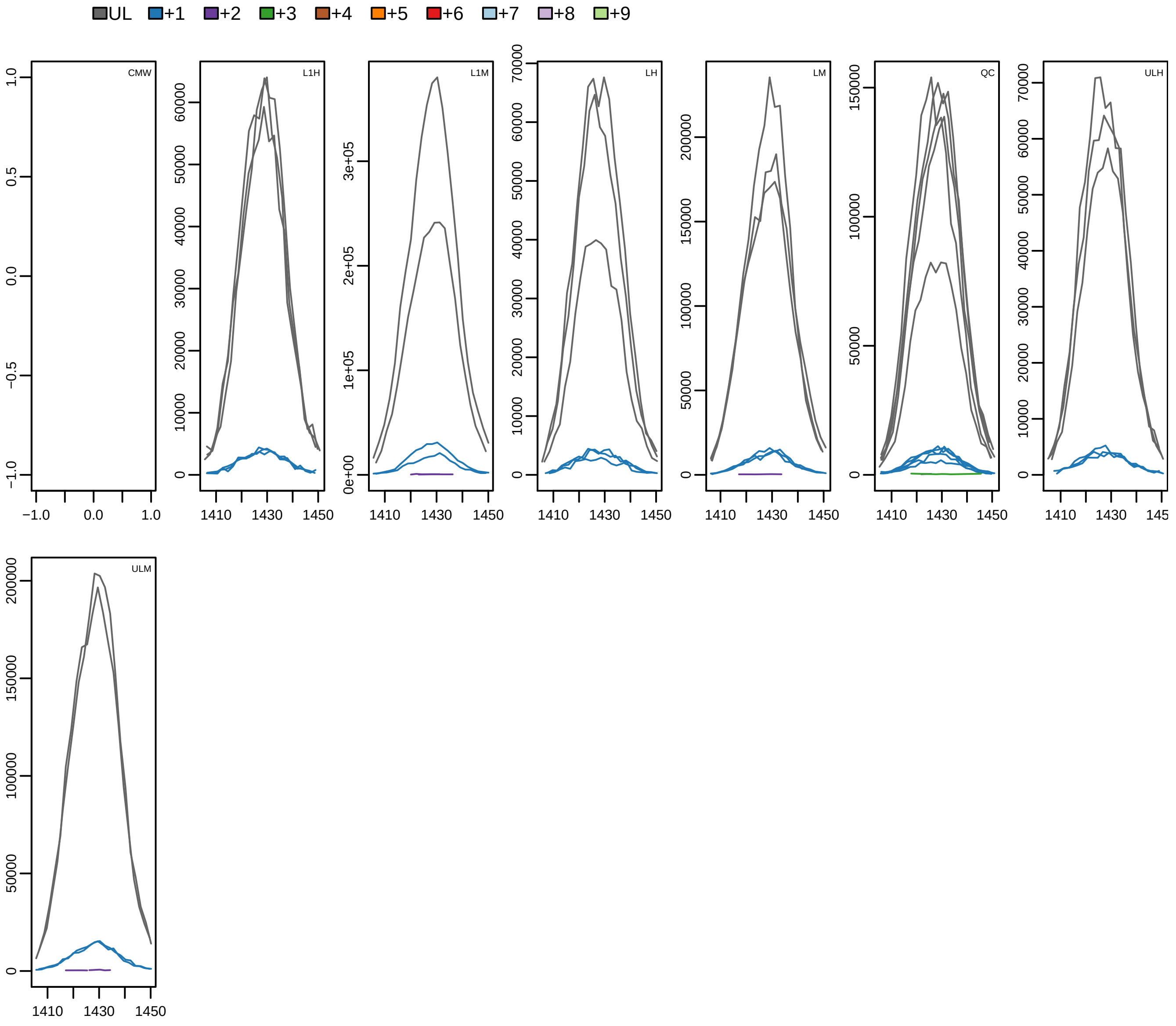

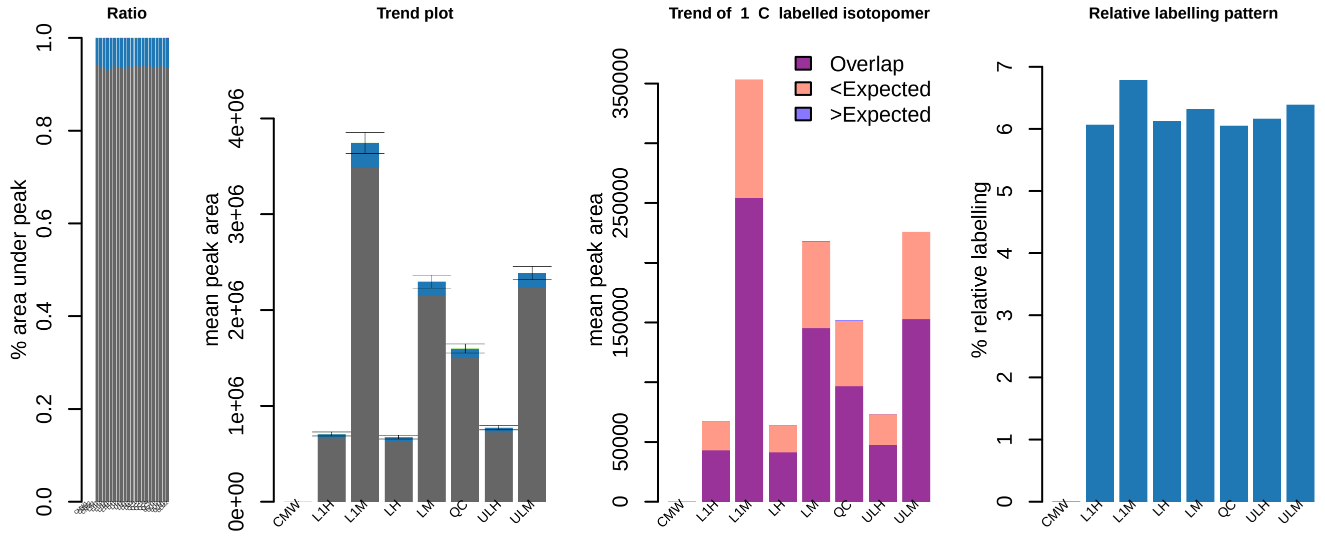

L−Carnitine

Formula: C7H15NO3 Mass: 161.105 Std.RT: 867.4442562 Ion: POS

G1

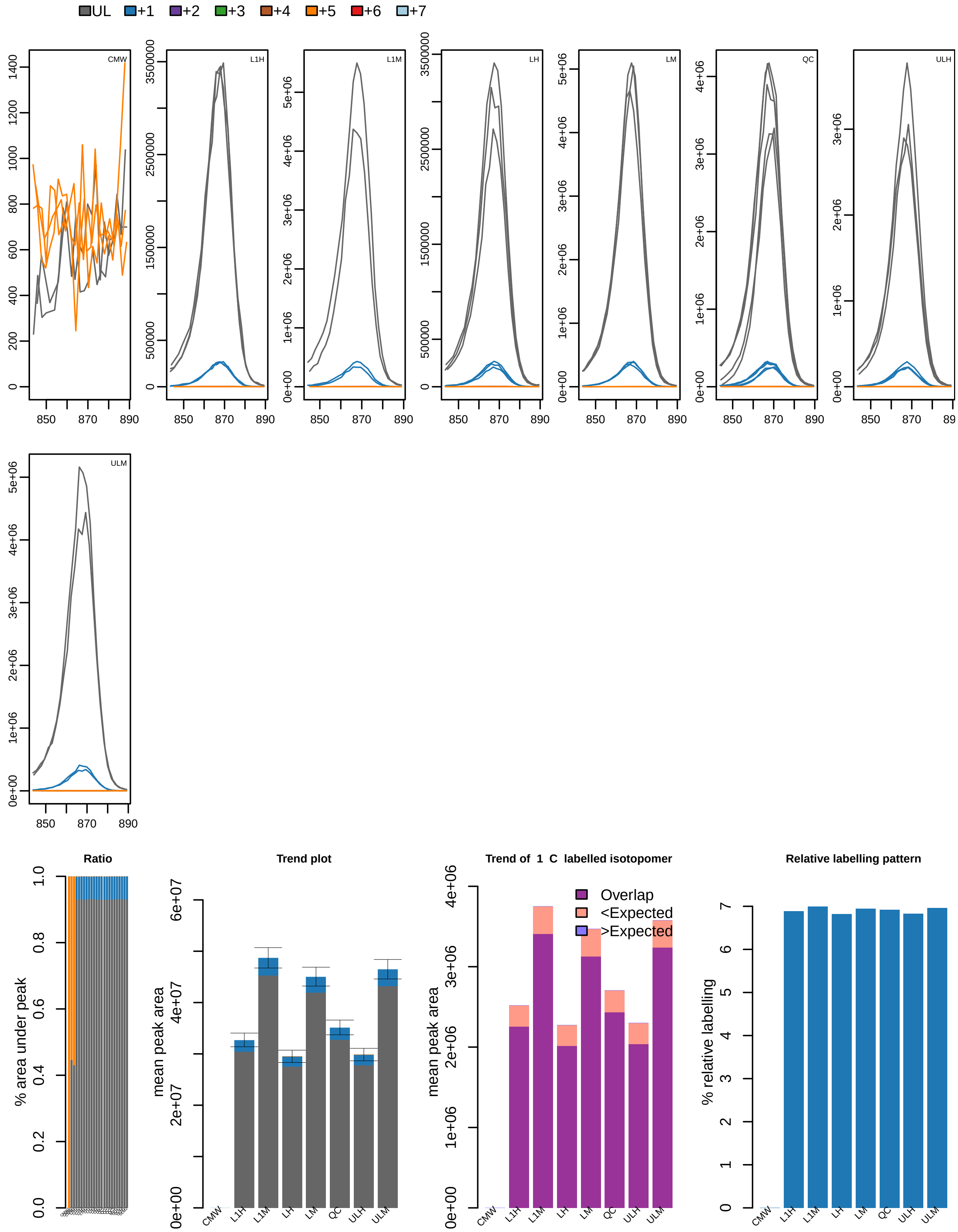

4−Trimethylammoniobutanoate

Formula: $C_7H_{15}NO_2$ Mass: 145.11 Std.RT: 872.8437474 Ion: POS

G1

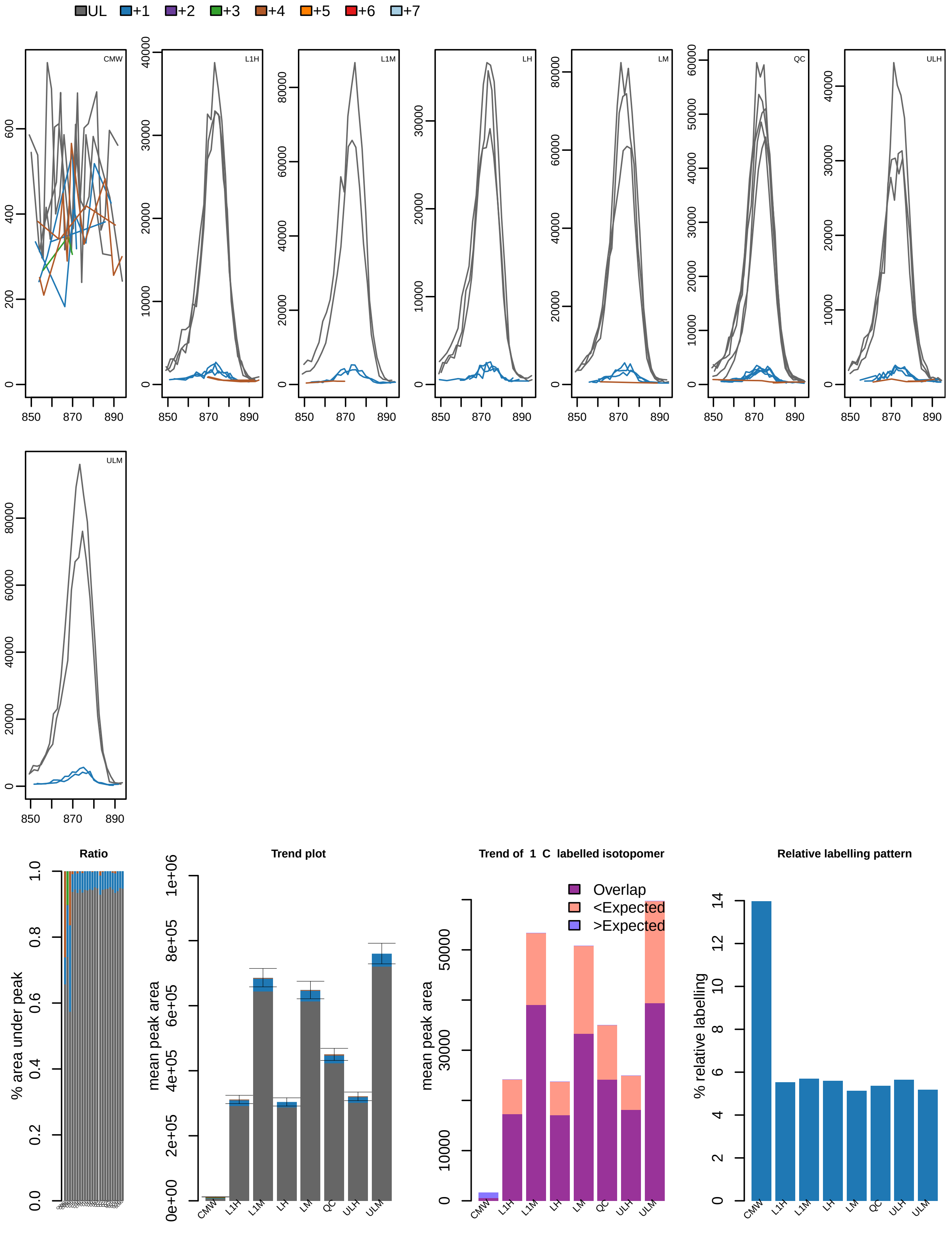

2,3,4,5−Tetrahydropyridine−2−carboxylate

Formula: C6H9NO2 Mass: 127.063 Std.RT: 853.1842524 Ion: POS

G1

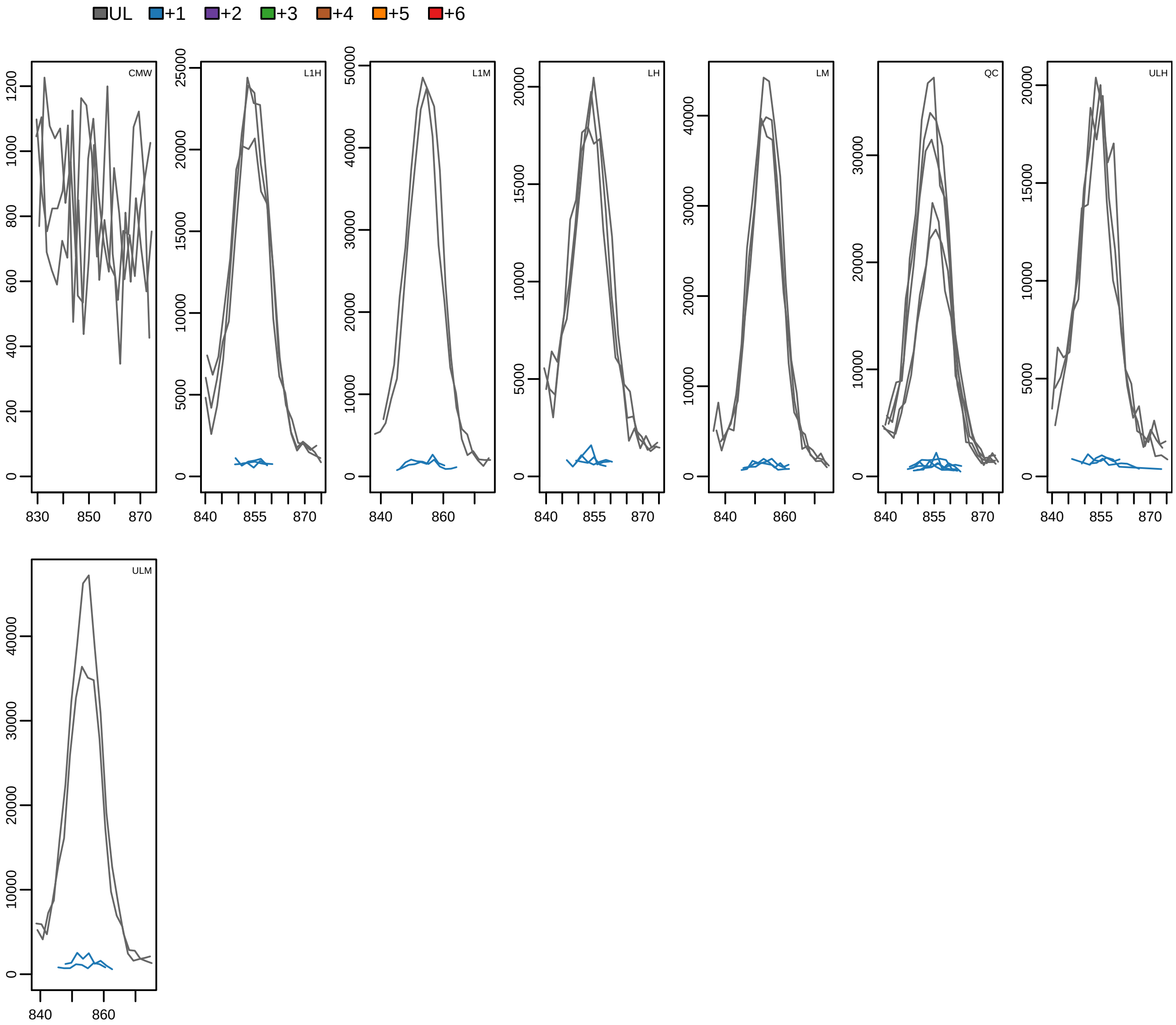

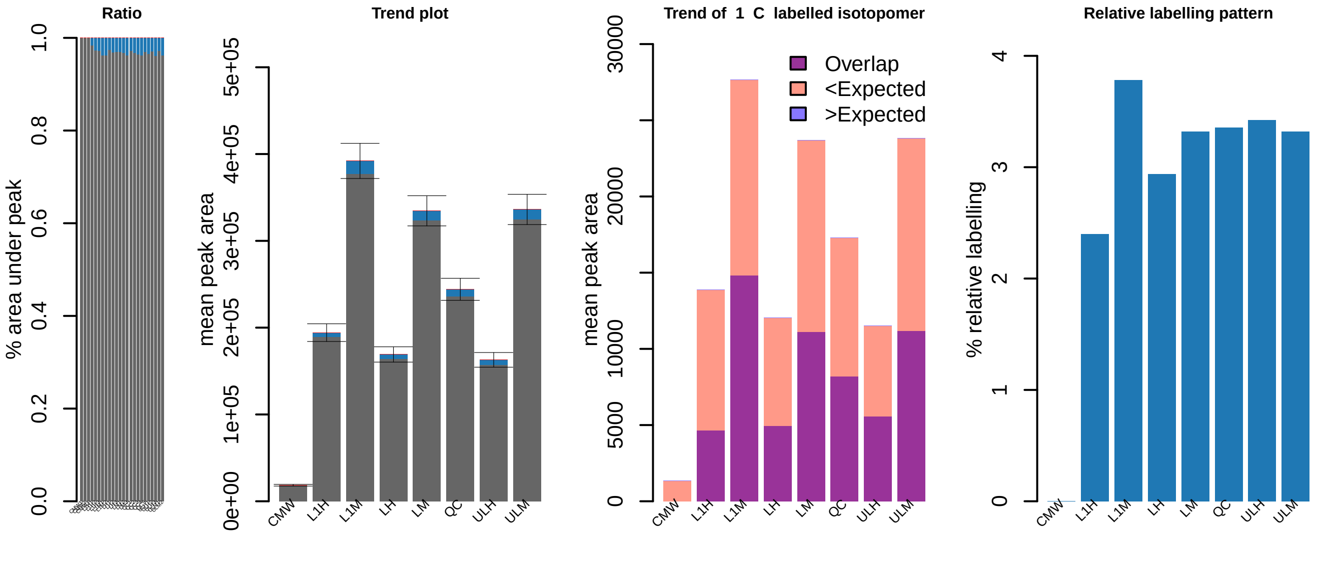

[FA oxo,amino(6:0)] 3−oxo−5S−amino−hexanoic acid

Formula: C6H11NO3 Mass: 145.074 Std.RT: 852.8172534 Ion: POS

G1

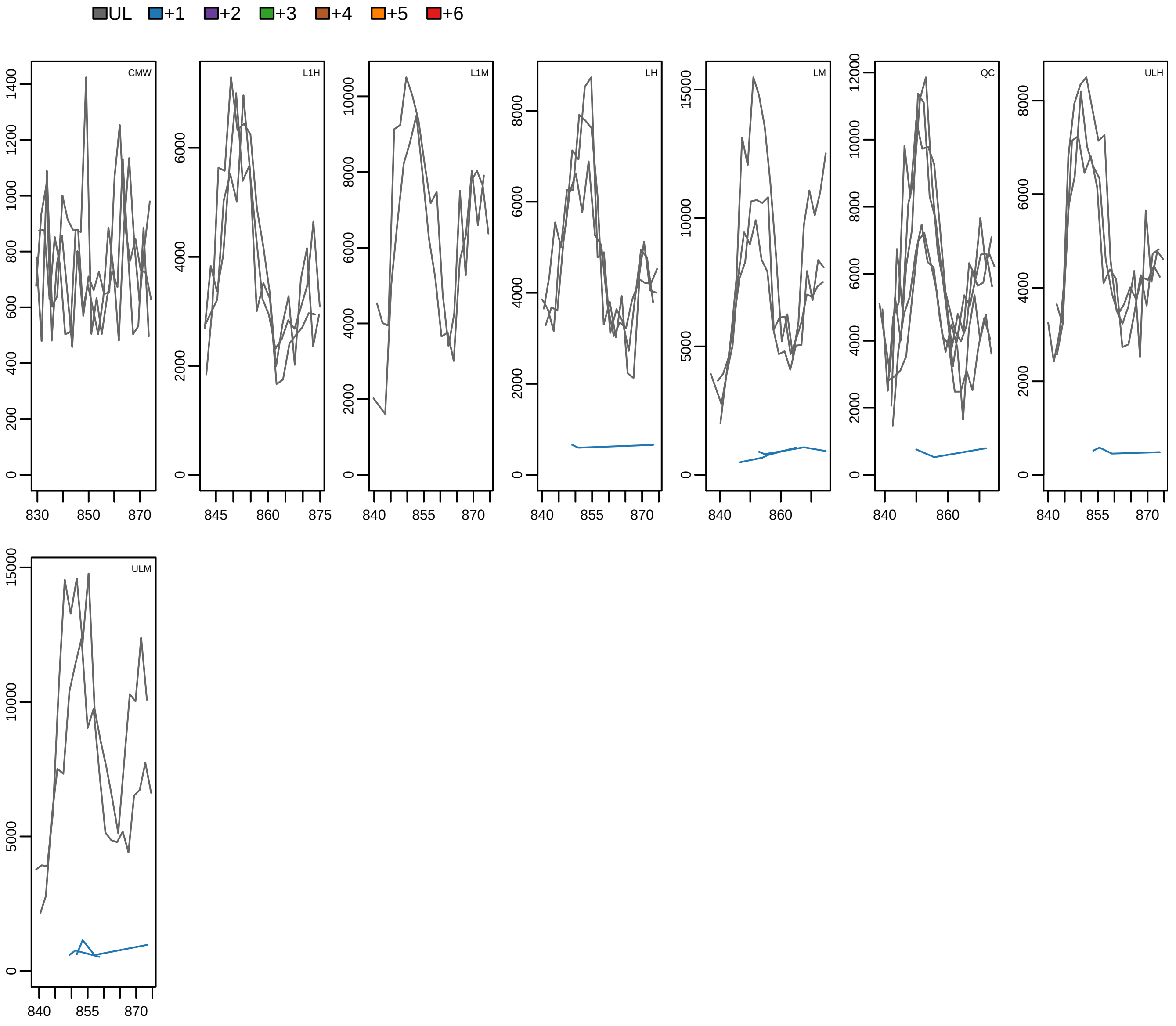

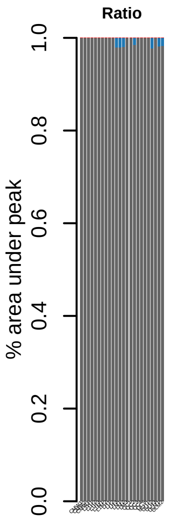

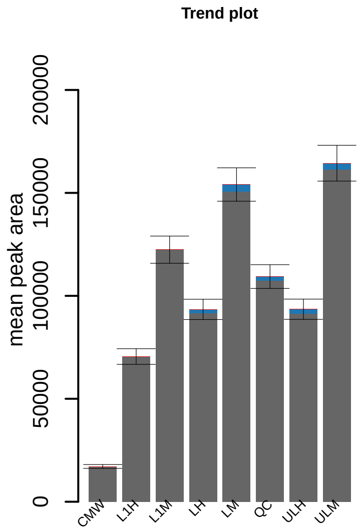

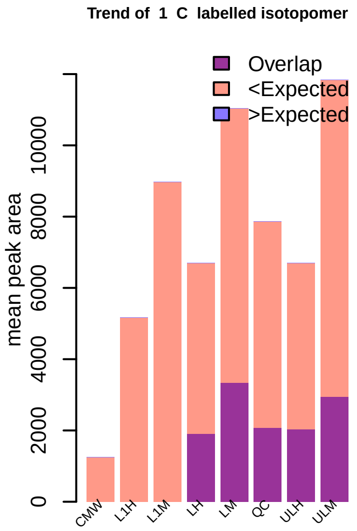

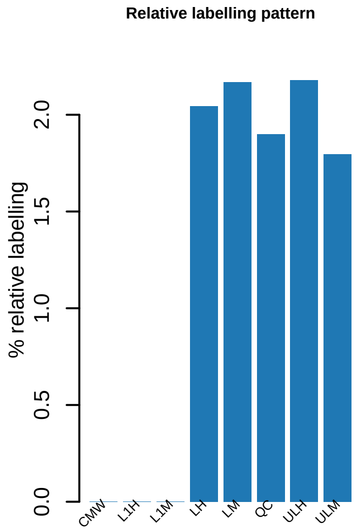

[FA oxo,amino(6:0)] 3−oxo−5S−amino−hexanoic acid

Formula: C6H11NO3 Mass: 145.074 Std.RT: 852.8172534 Ion: POS

G2

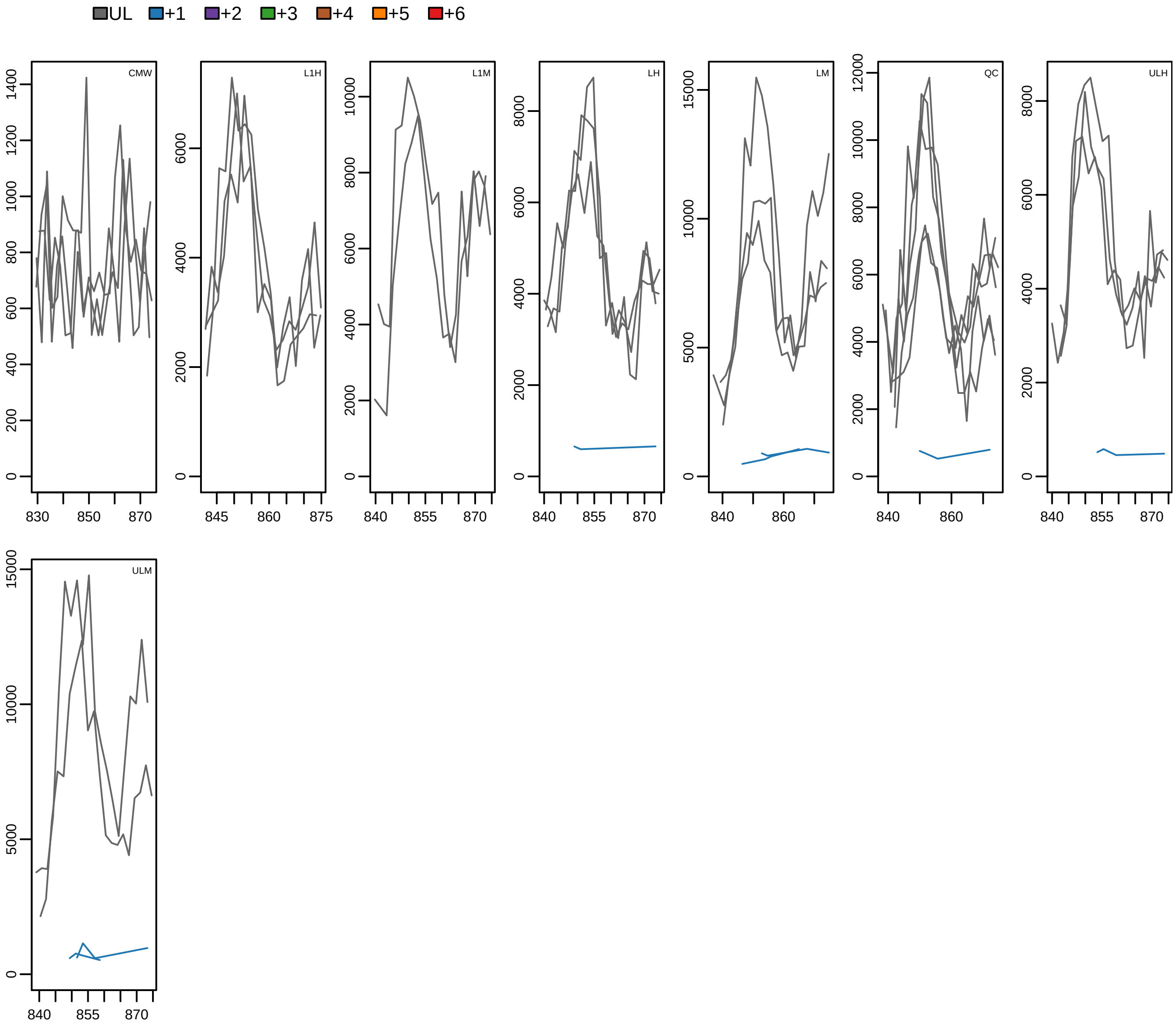

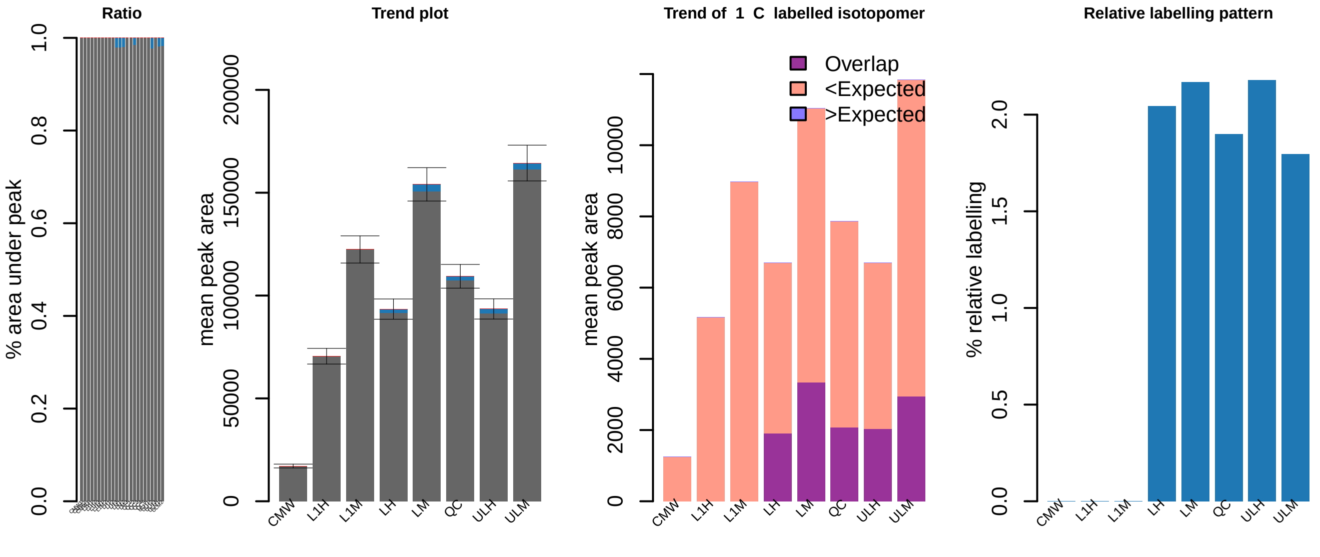

[FA oxo,amino(6:0)] 3−oxo−5S−amino−hexanoic acid

Formula: $C_6H_{11}NO_3$ Mass: 145.074 Std.RT: 852.8172534 Ion: POS

G3

UL +1 +2 +3 +4 +5 +6

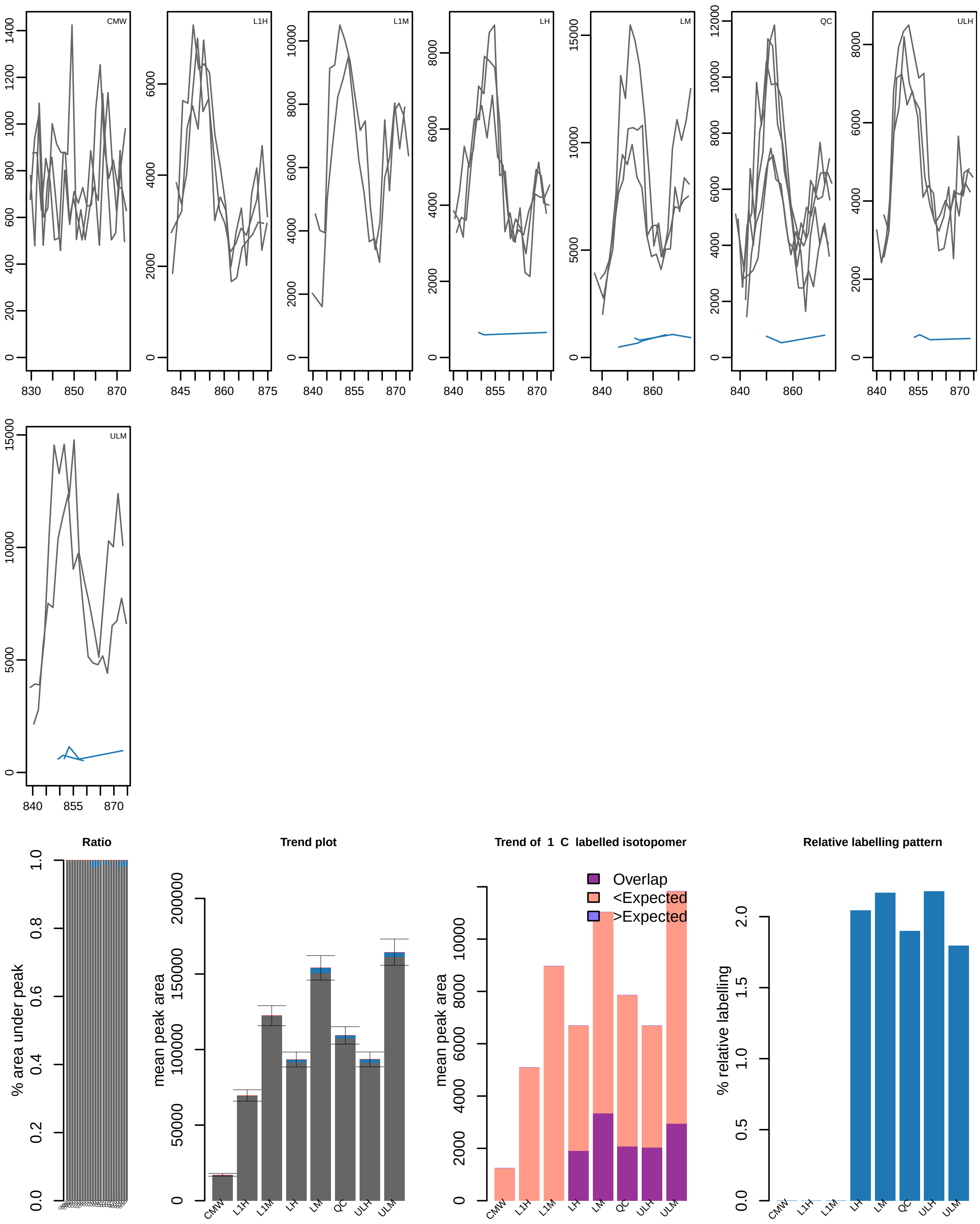

L−Pipecolate

Formula: C6H11NO2 Mass: 129.079 Std.RT: 801.4722546 Ion: POS

G1

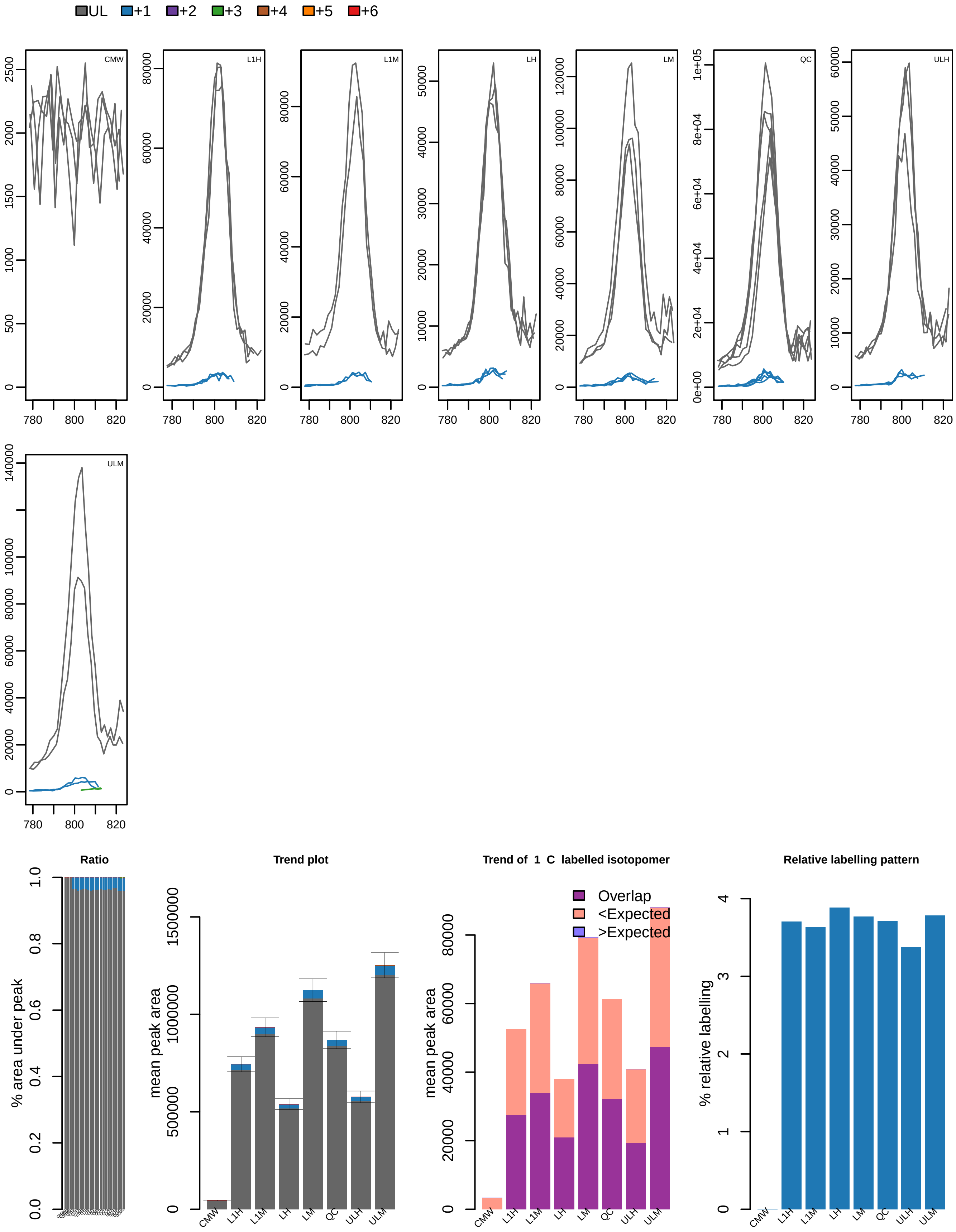

L−Pipecolate

Formula: $C_6H_{11}NO_2$ Mass: 129.079 Std.RT: 801.4722546 Ion: POS

G2

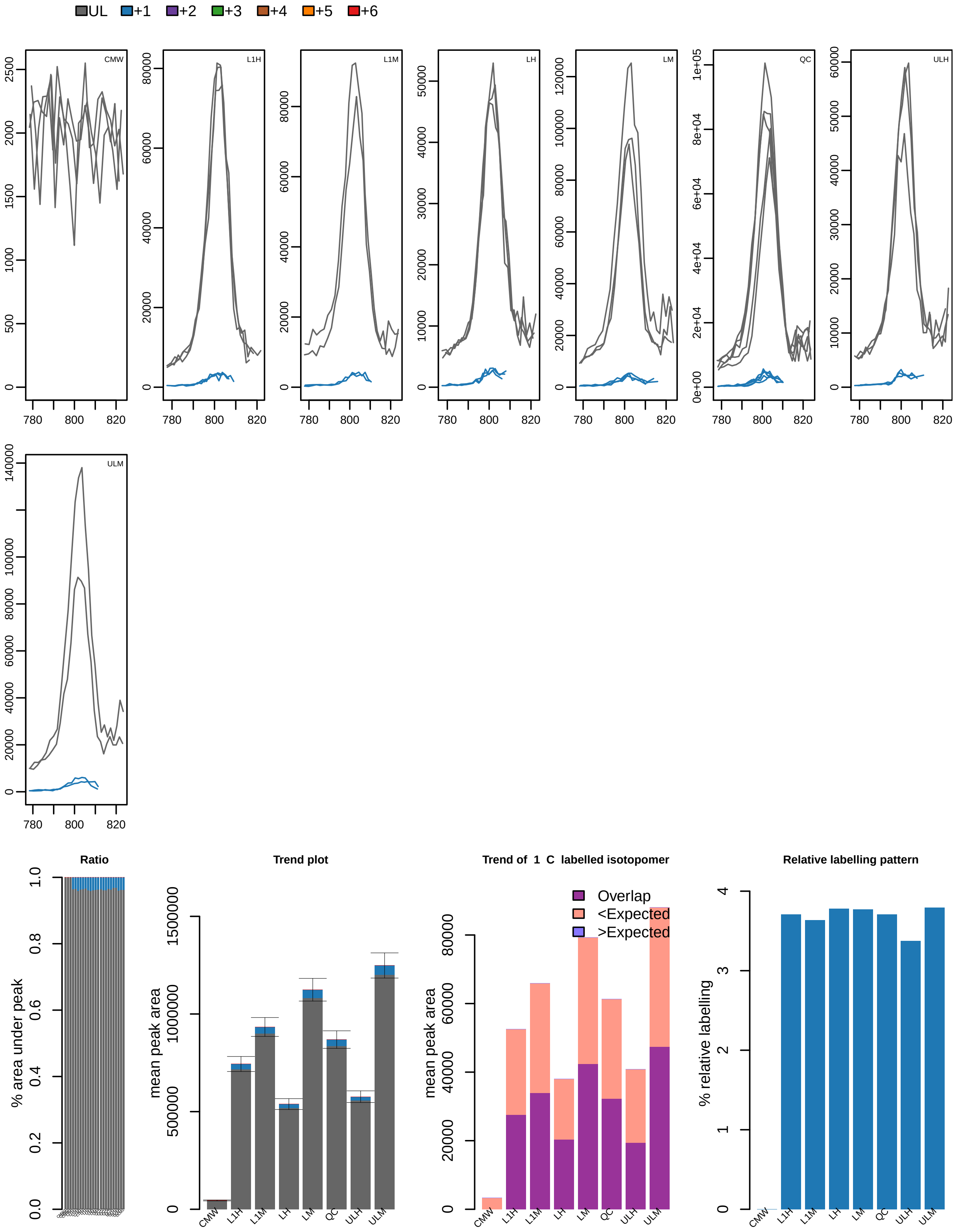

S−Adenosyl−L−homocysteine

Formula: C14H20N6O5S Mass: 384.122 Std.RT: 876.589716 Ion: PC

G1

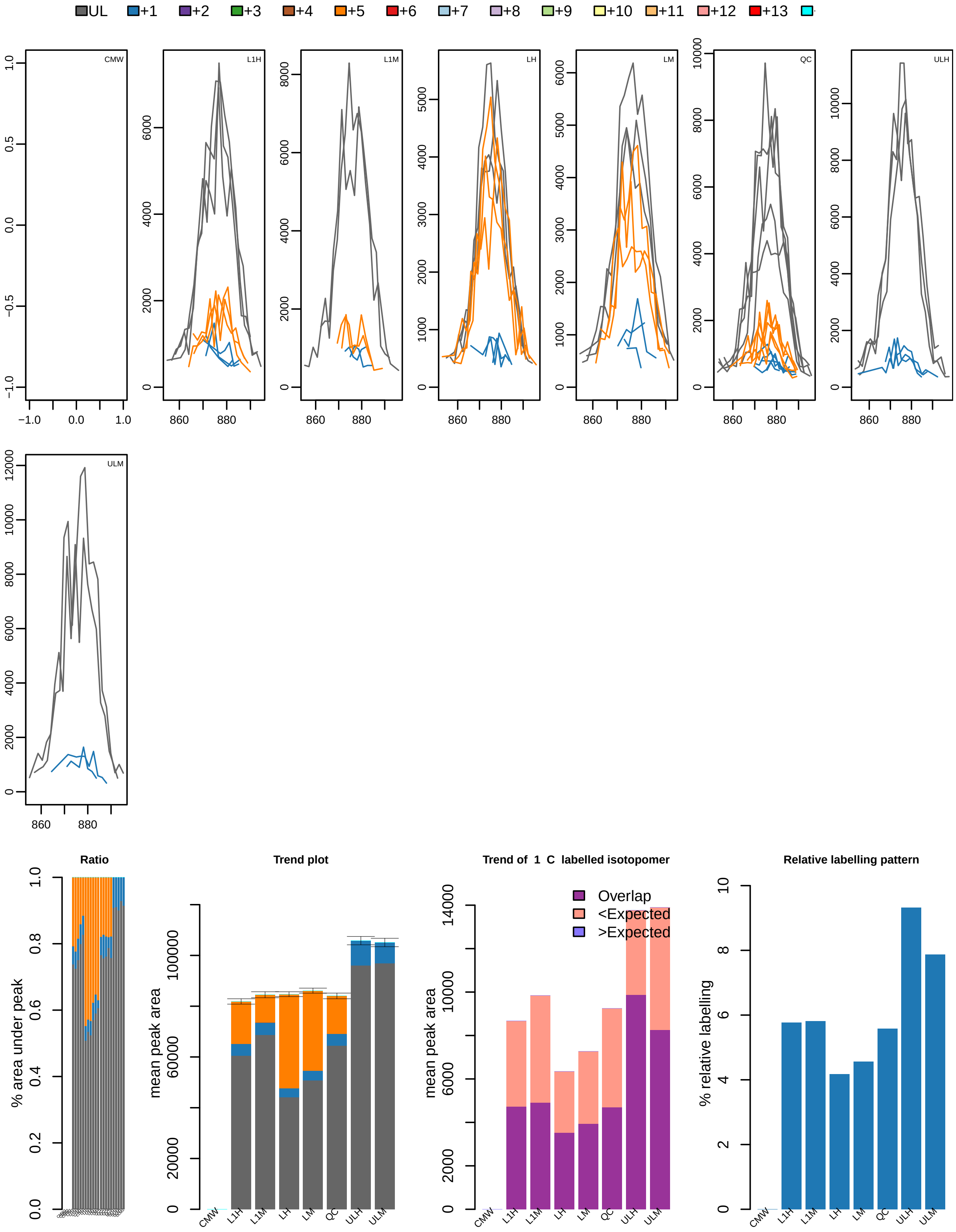

L−Methionine

Formula: C5H11NO2S Mass: 149.051 Std.RT: 731.1920016 Ion: PO[illegible]

G1

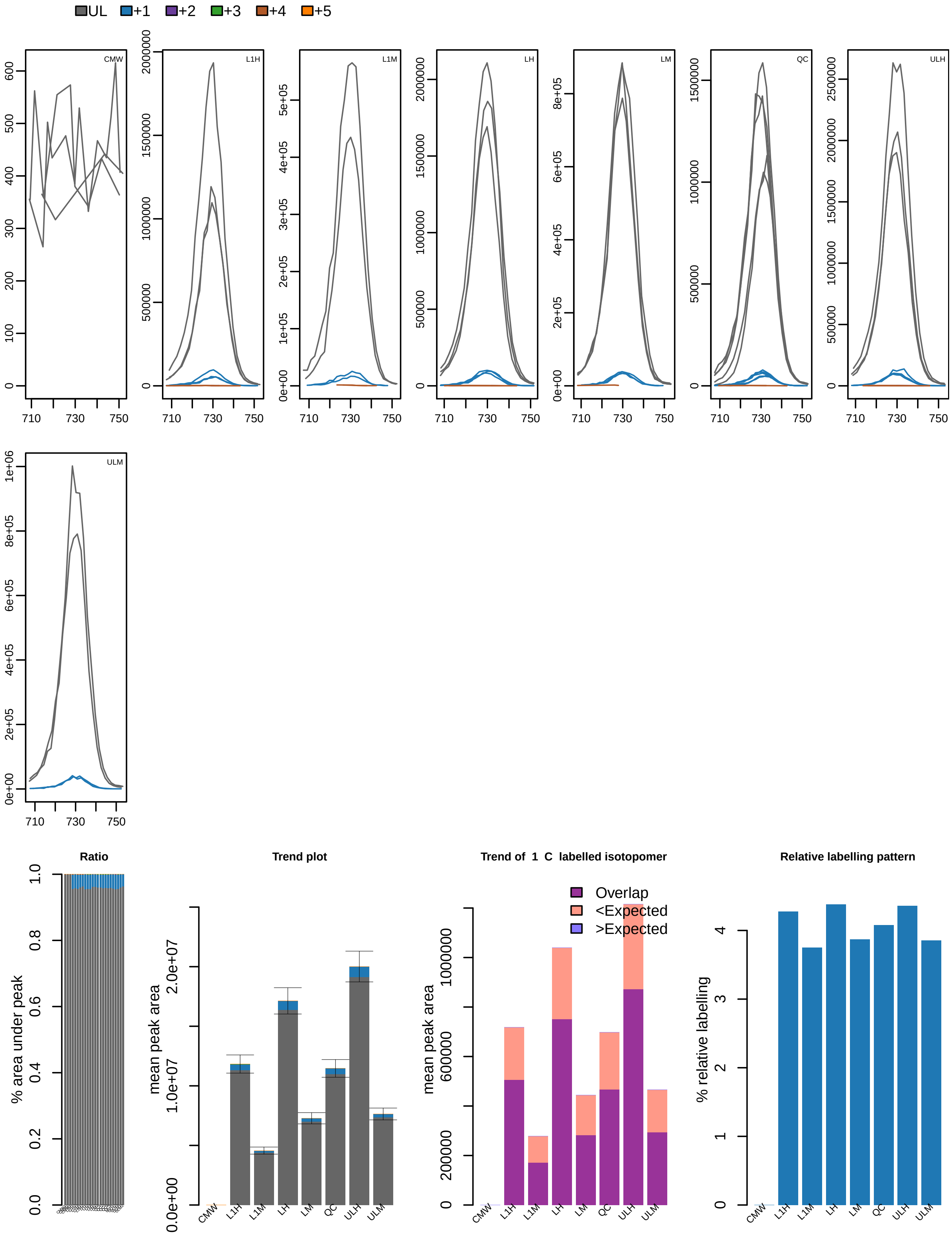

L−Methionine S−oxide

Formula: C5H11NO3S Mass: 165.046 Std.RT: 851.756859 Ion: POS

G1

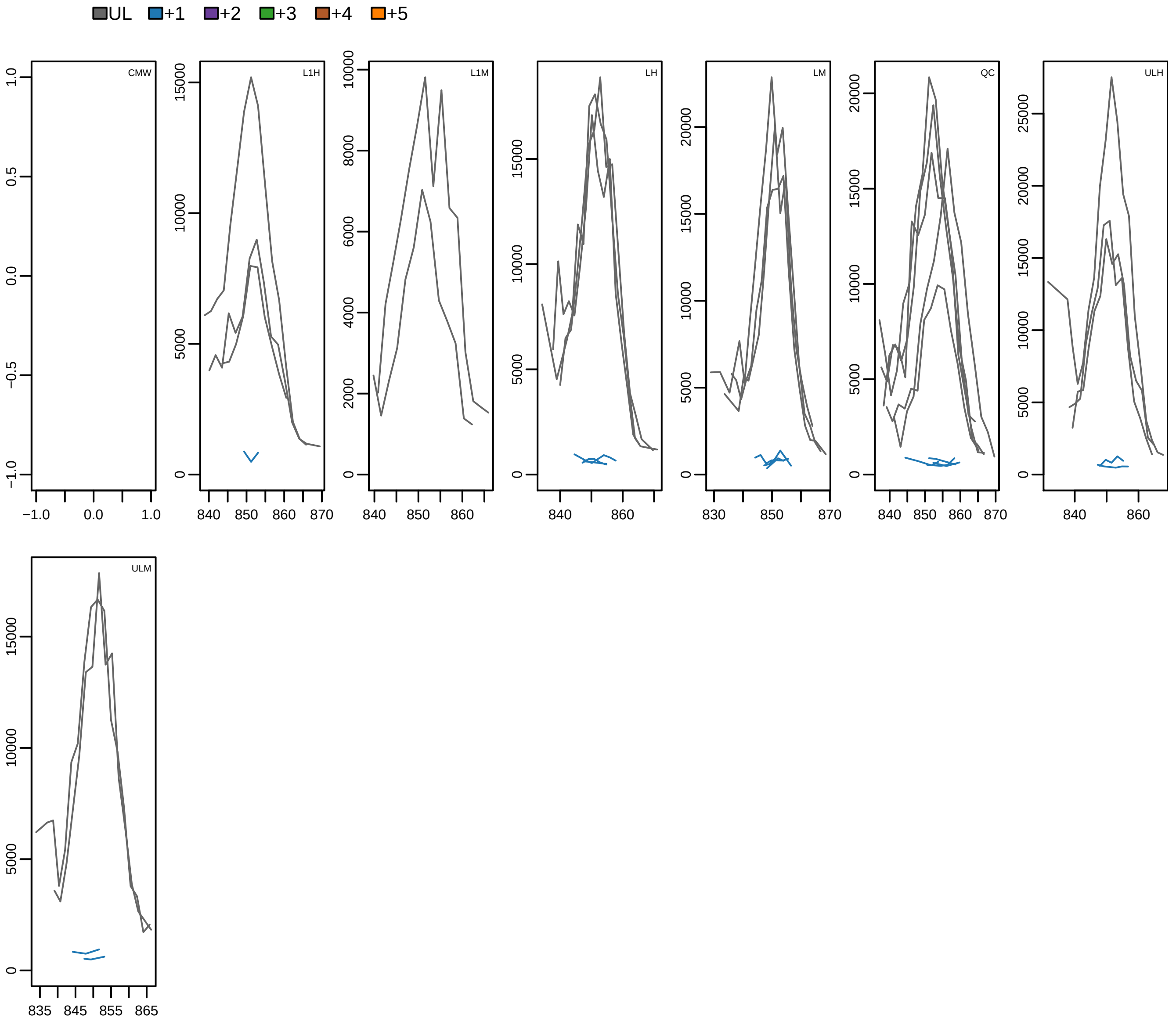

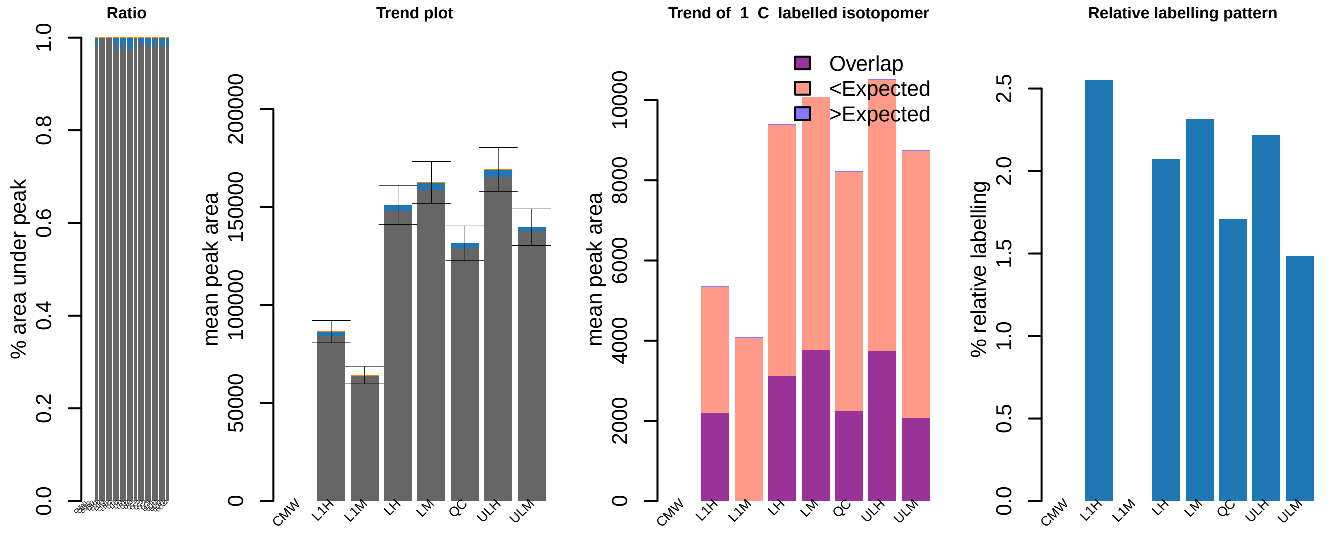

O−Acetyl−L−homoserine

Formula: C6H11NO4 Mass: 161.069 Std.RT: 694.876254 Ion: POS

G1

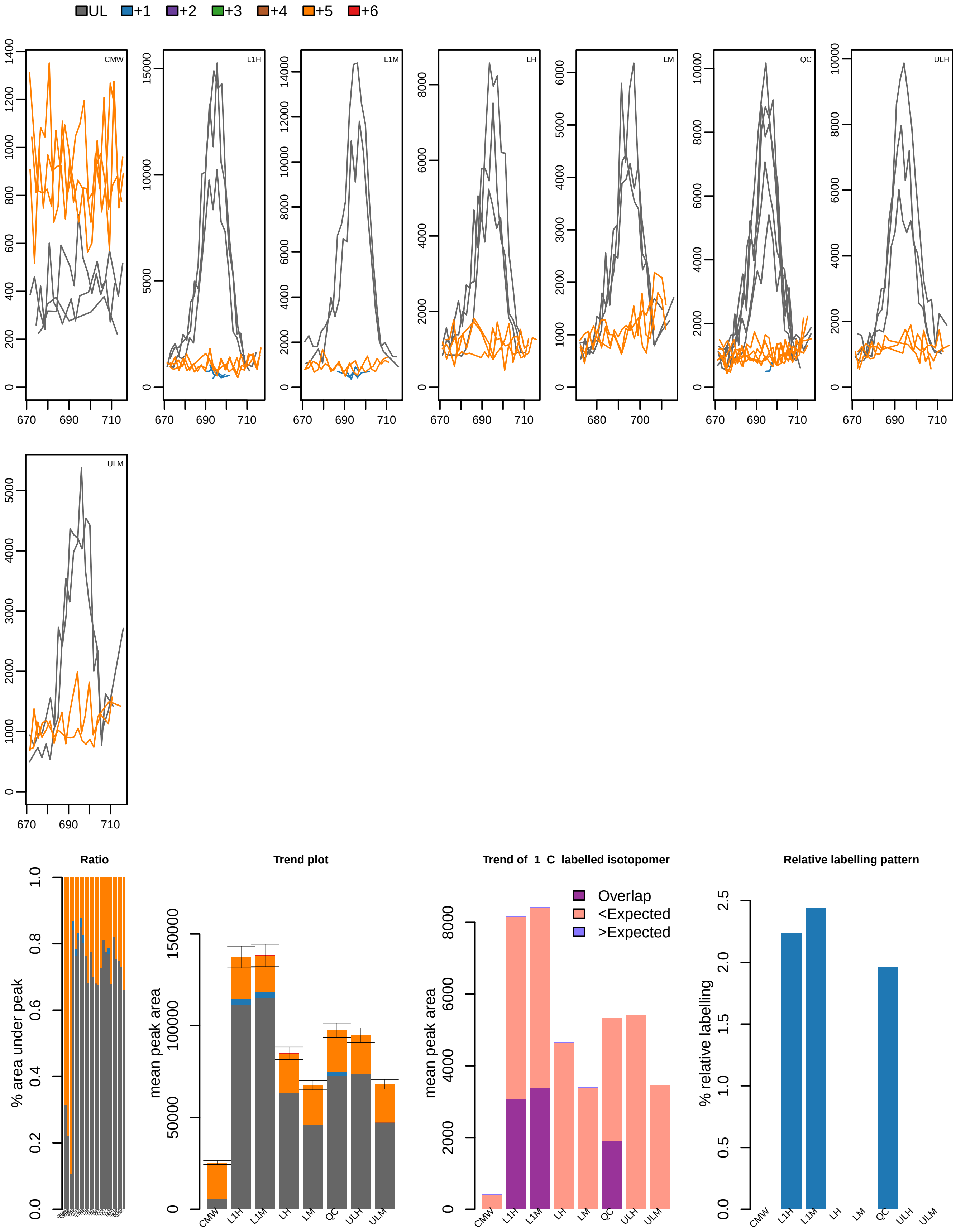

L−Phenylalanine

Formula: C9H11NO2 Mass: 165.079 Std.RT: 637.4000934 Ion: POS

G1

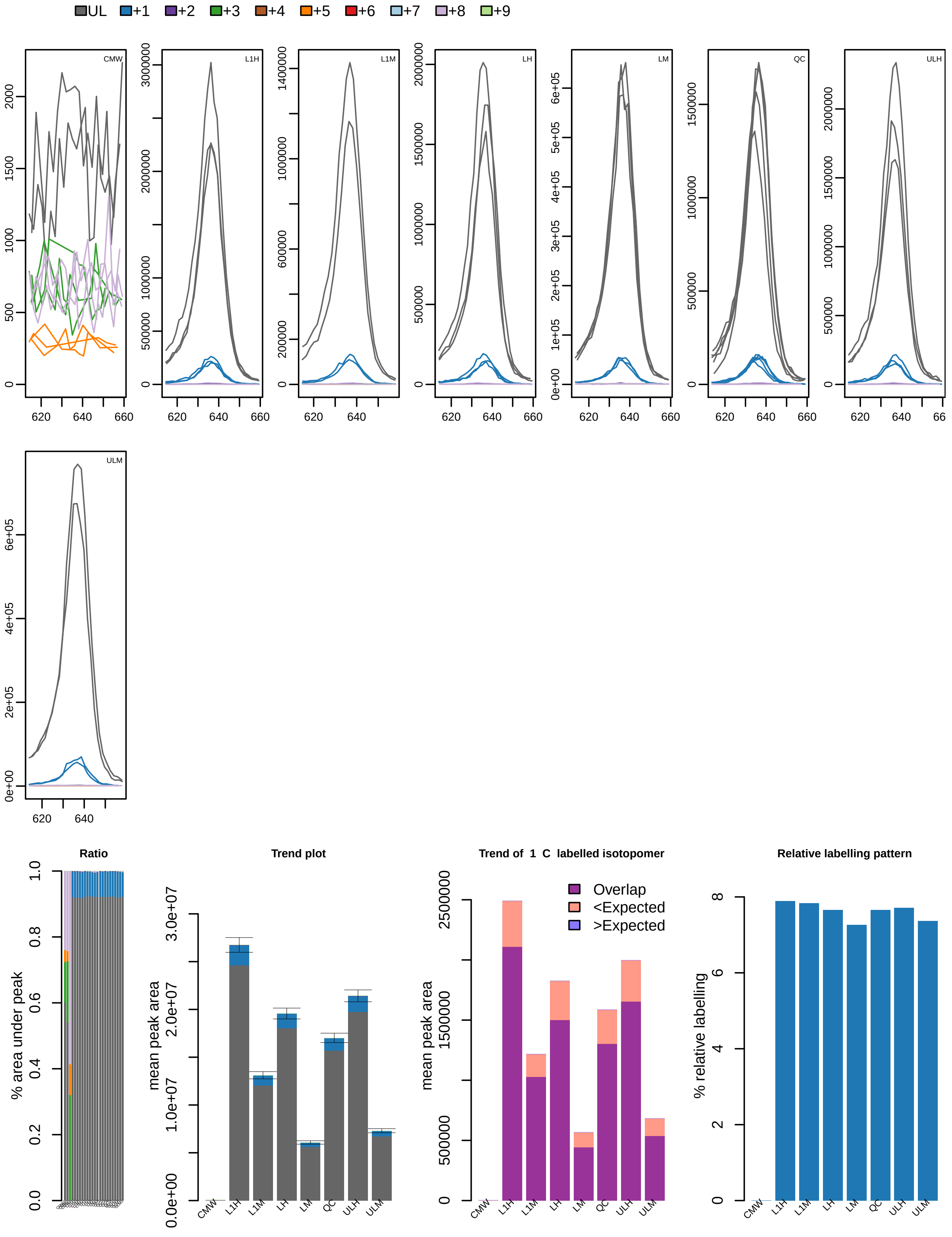

Hypotaurine

Formula: C2H7NO2S Mass: 109.02 Std.RT: 972.6488616 Ion: POS

G1

UL +1 +2

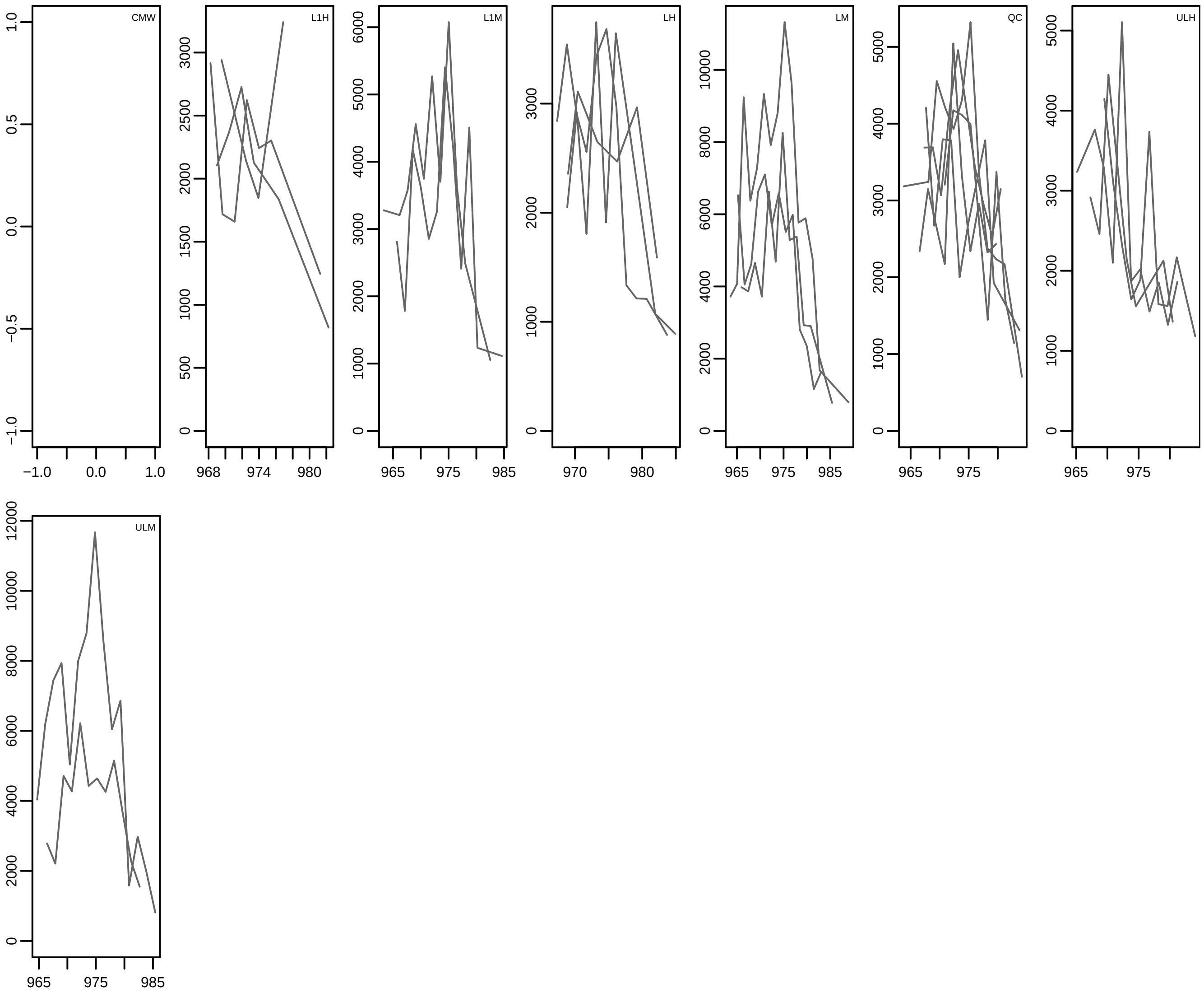

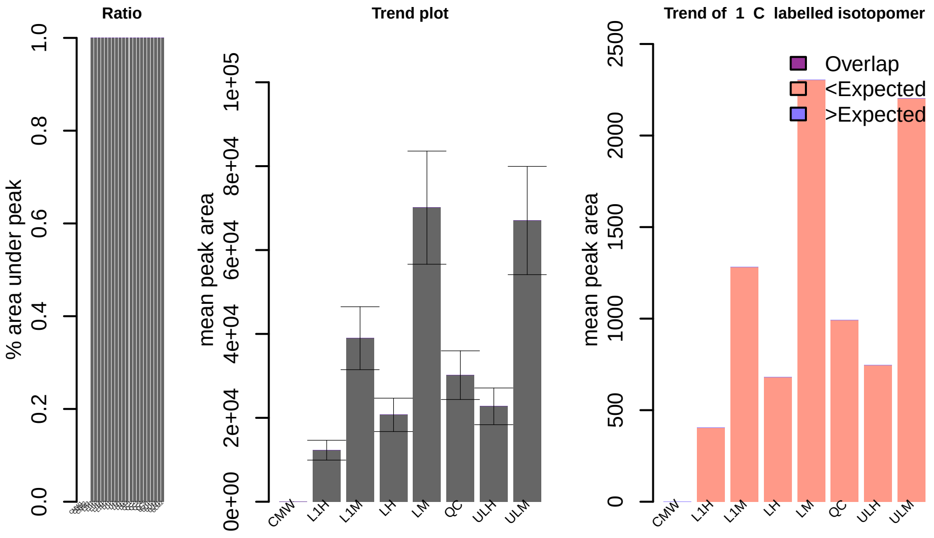

L−Kynurenine

Formula: C10H12N2O3 Mass: 208.085 Std.RT: 685.7217174 Ion: PC

G1

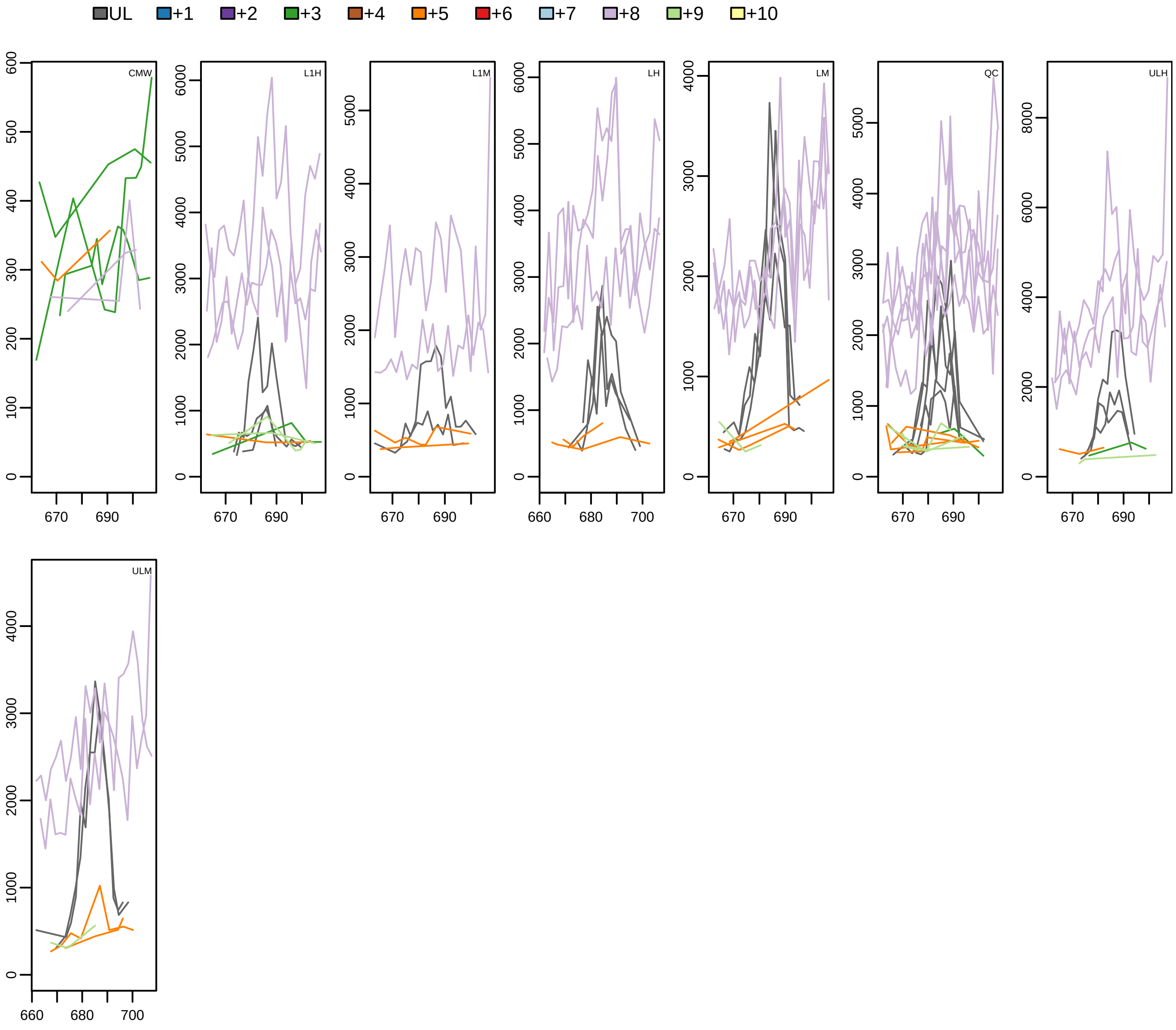

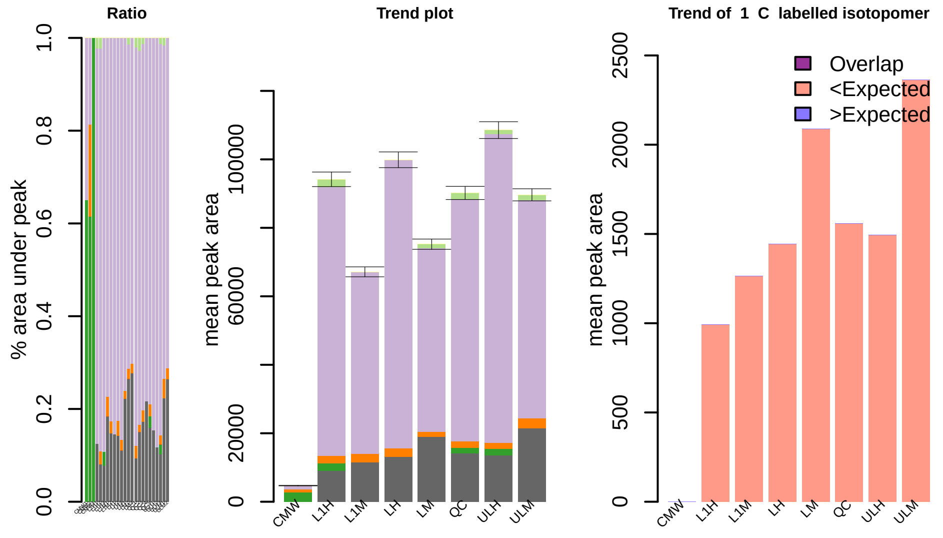

L−Tryptophan

Formula: C11H12N2O2 Mass: 204.09 Std.RT: 737.1331338 Ion: POS

G1

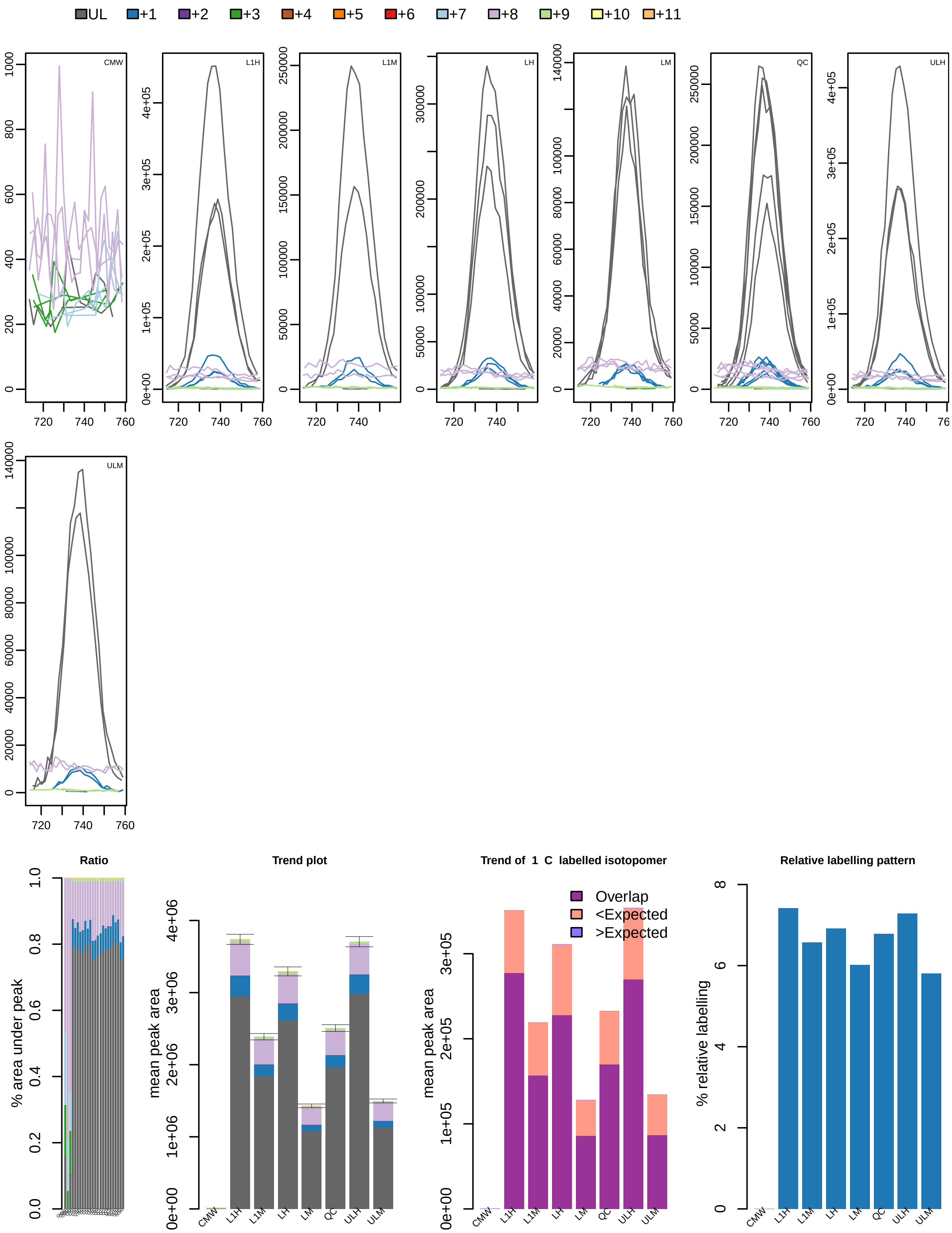

L−Tyrosine

Formula: C9H11NO3 Mass: 181.074 Std.RT: 831.4772442 Ion: POS

G1

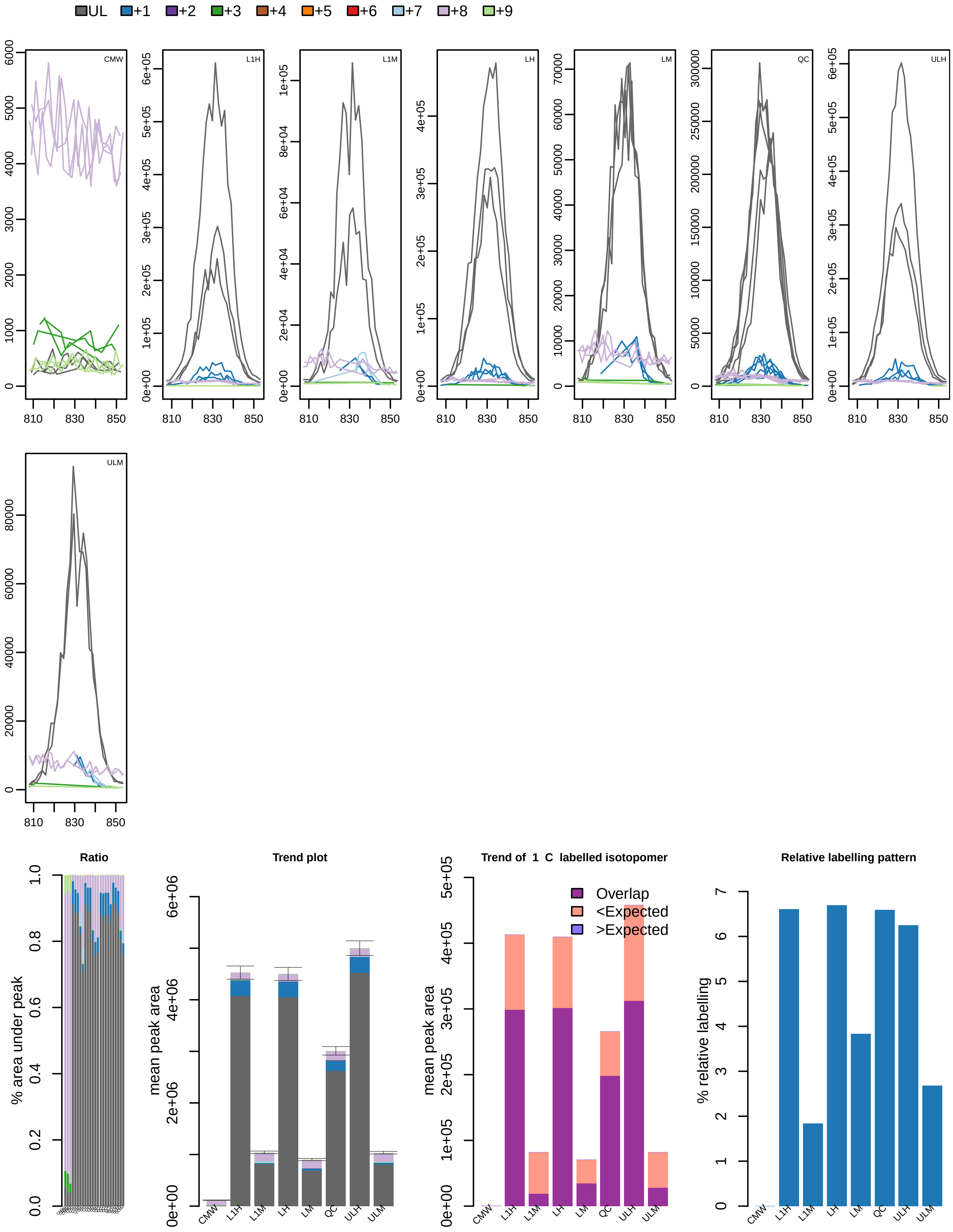

L−3−Amino−isobutanoate

Formula: C4H9NO2 Mass: 103.063 Std.RT: 887.5964964 Ion: POS

G1

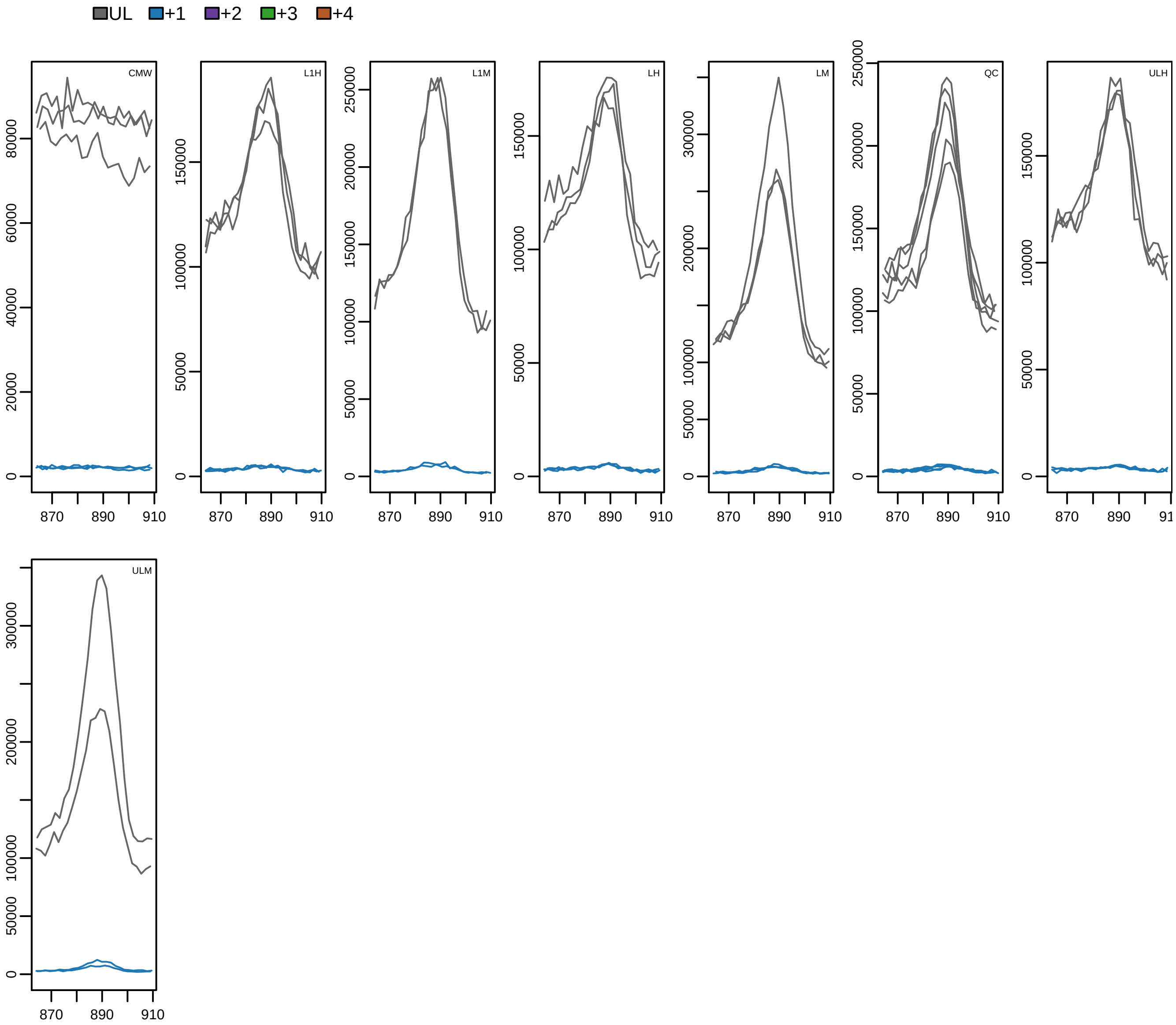

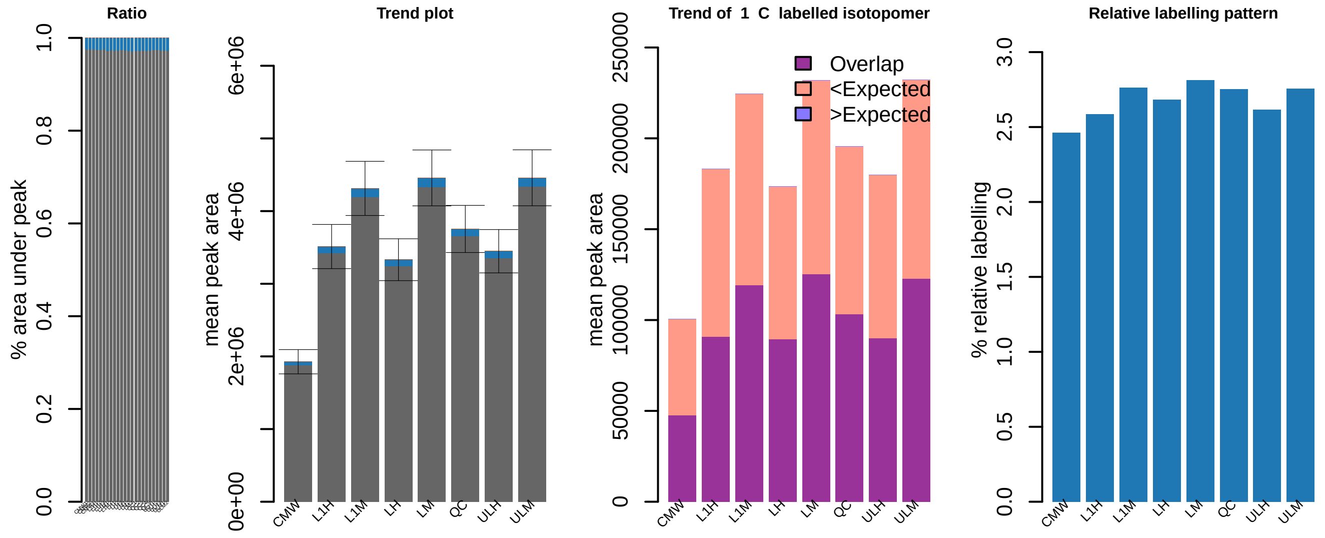

L−Leucine

Formula: C6H13NO2 Mass: 131.095 Std.RT: 688.5995028 Ion: POS

G1

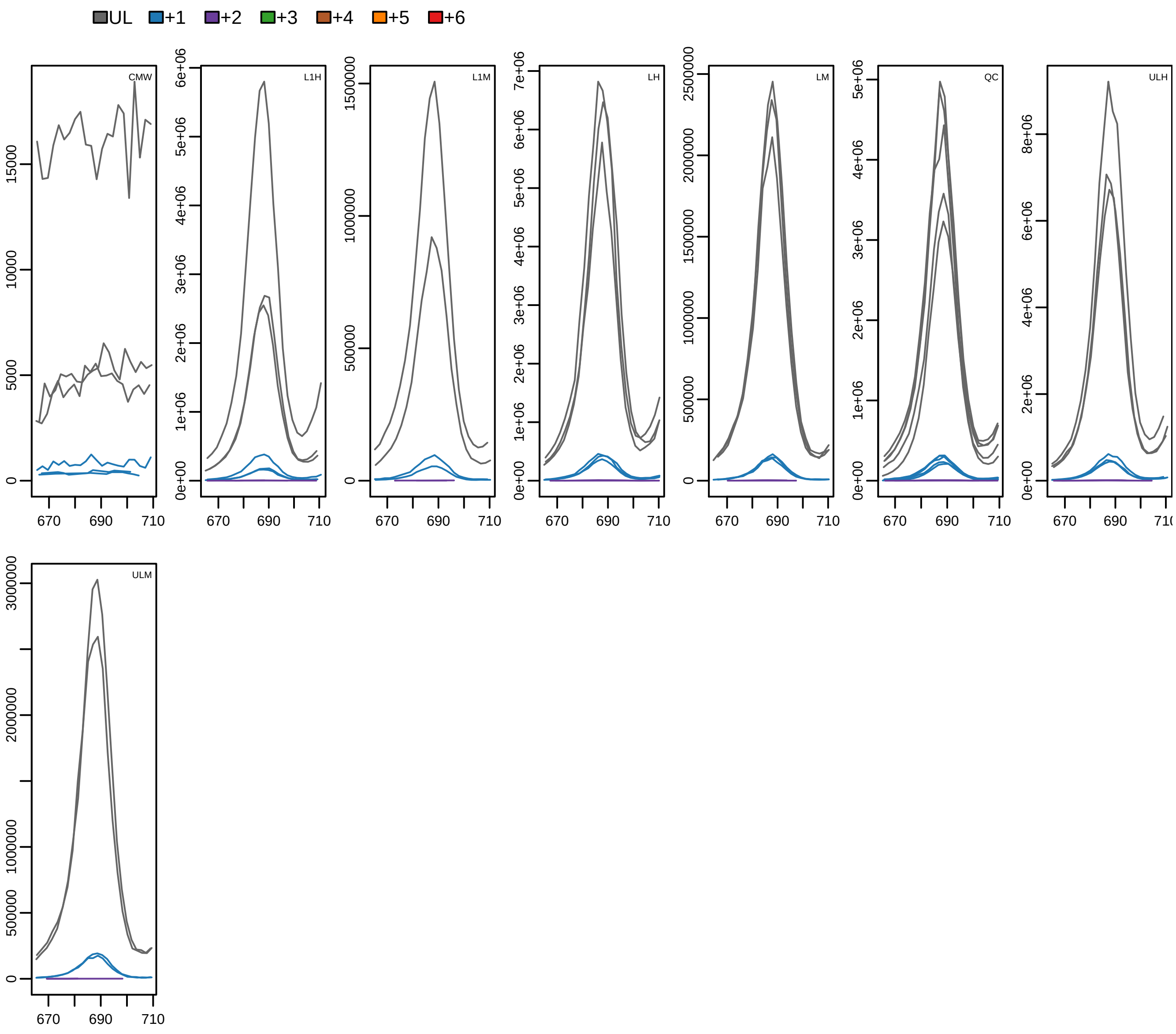

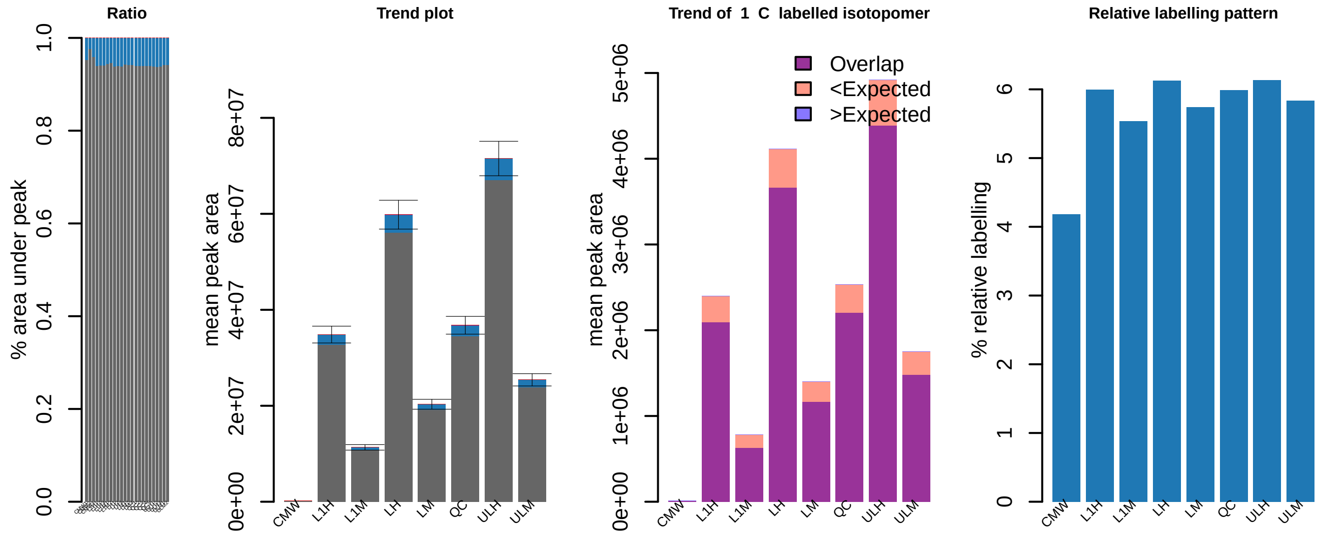

L−Leucine

Formula: C6H13NO2 Mass: 131.095 Std.RT: 688.5995028 Ion: POS

G2

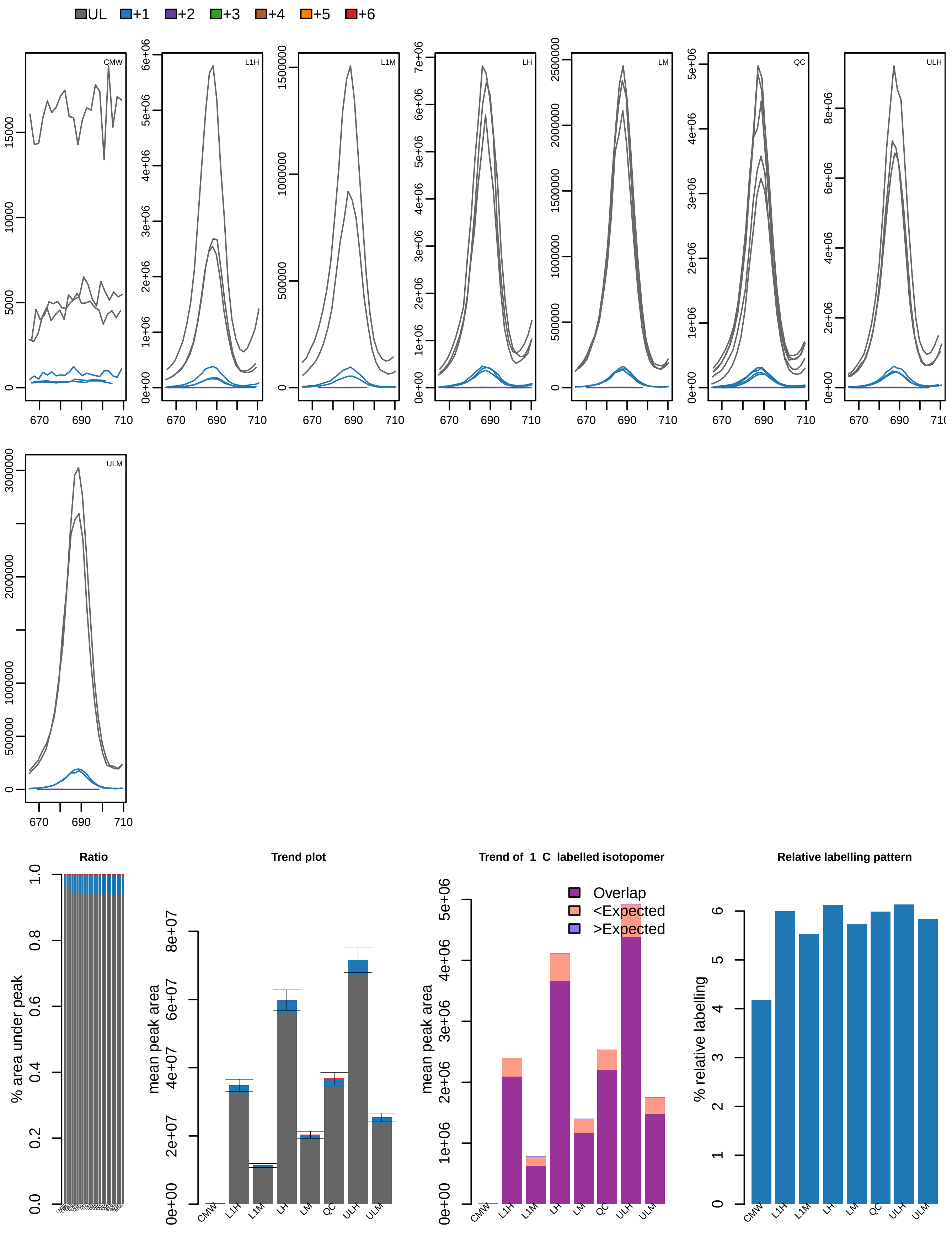

L−Isoleucine

Formula: C6H13NO2 Mass: 131.095 Std.RT: 720.5872524 Ion: POS

G1

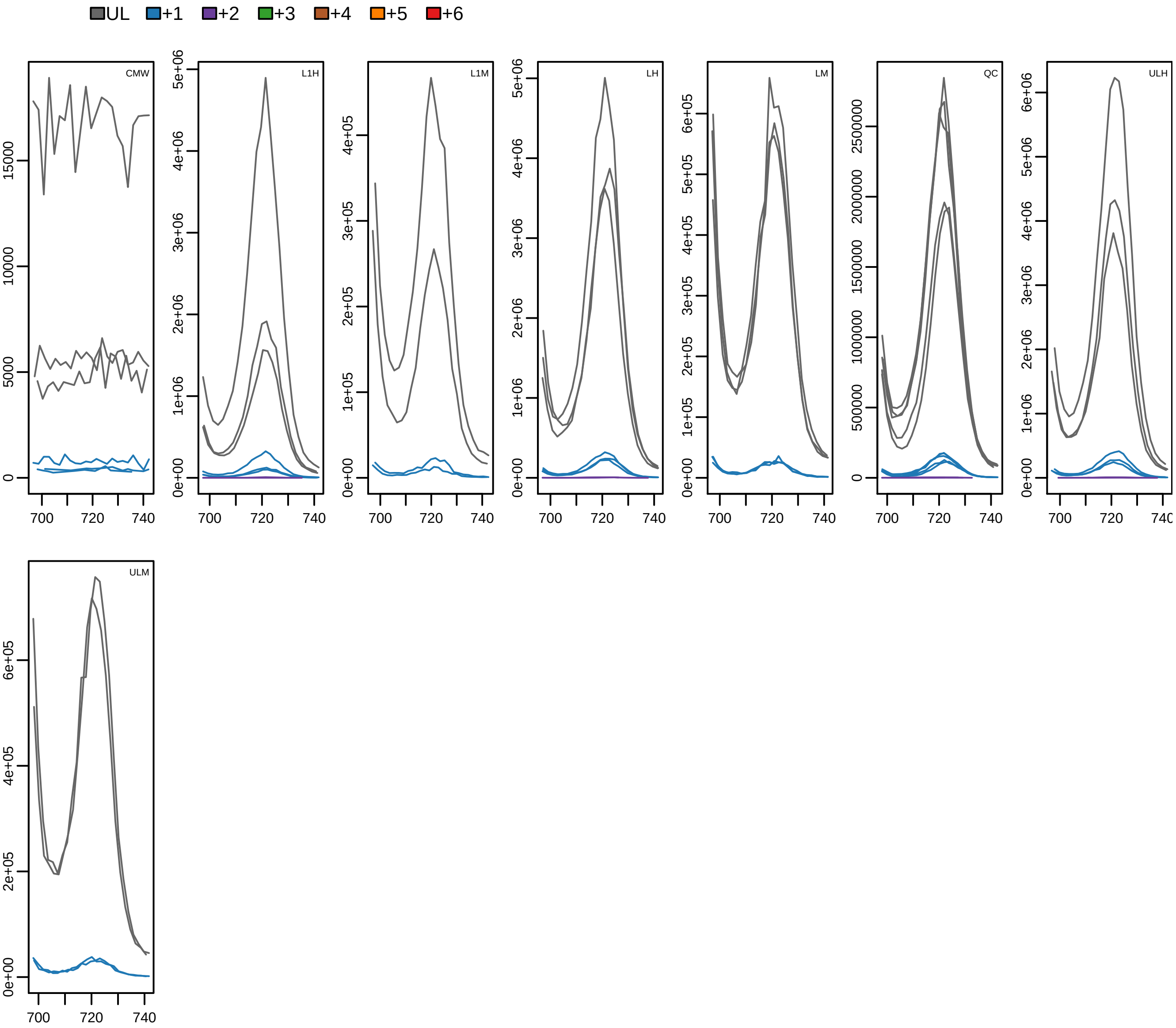

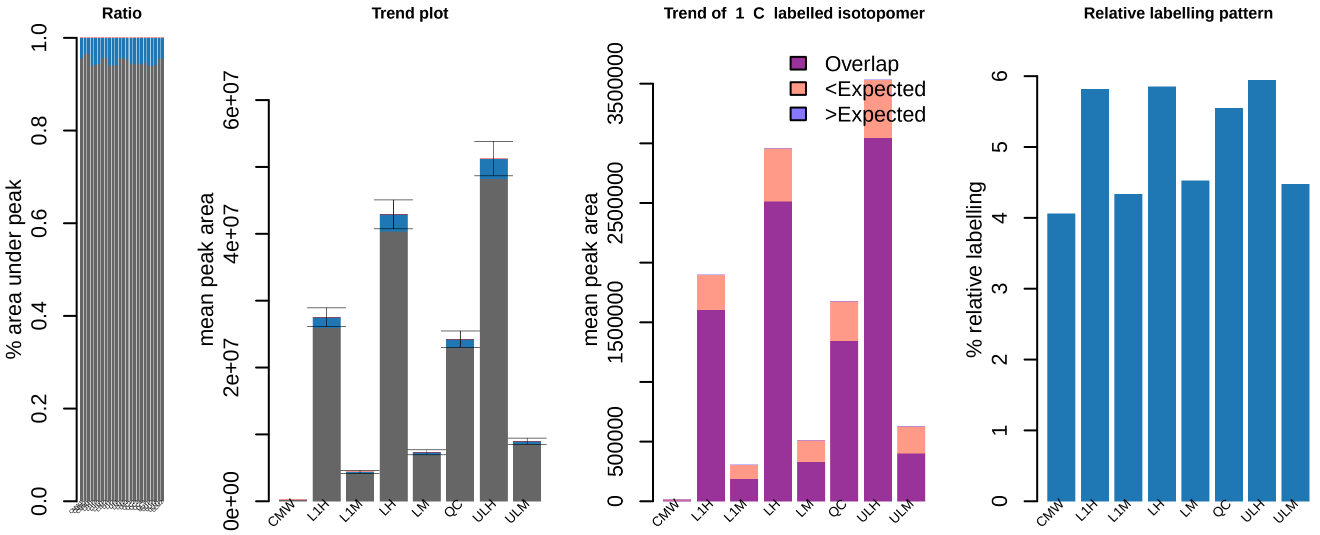

L−Isoleucine

Formula: C6H13NO2 Mass: 131.095 Std.RT: 720.5872524 Ion: POS

G2

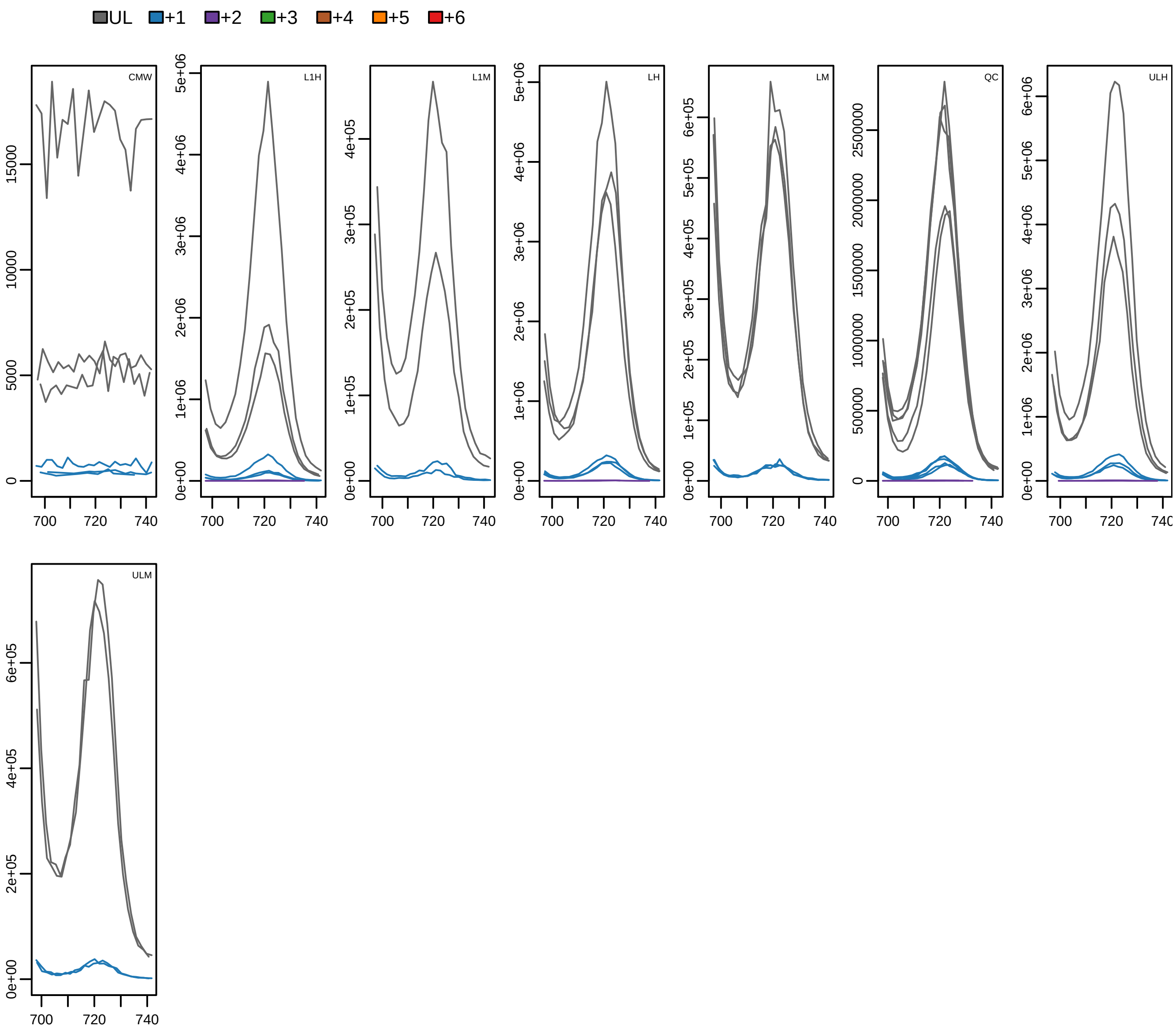

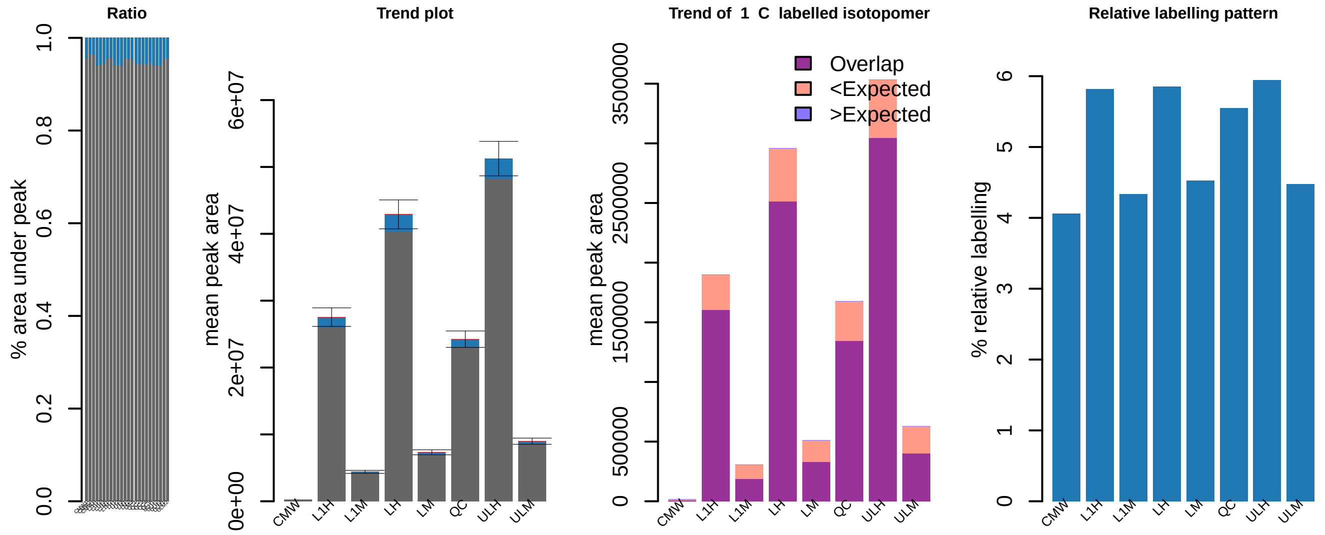

Slaframine

Formula: C12H20N2O3 Mass: 240.147 Std.RT: 635.7227532 Ion: PC

G1

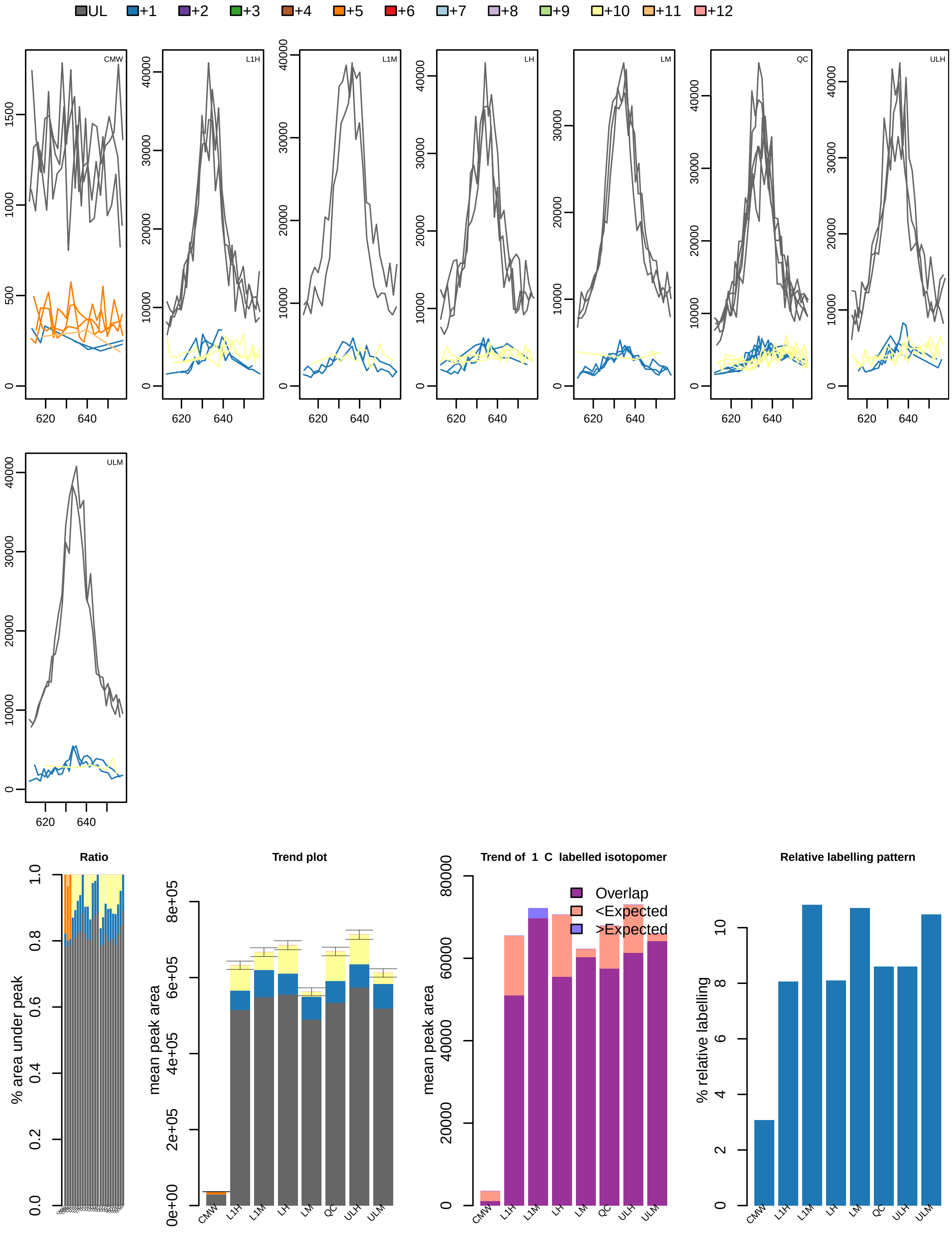

Caffeine

Formula: C8H10N4O2 Mass: 194.08 Std.RT: 657.5930022 Ion: POS

G1

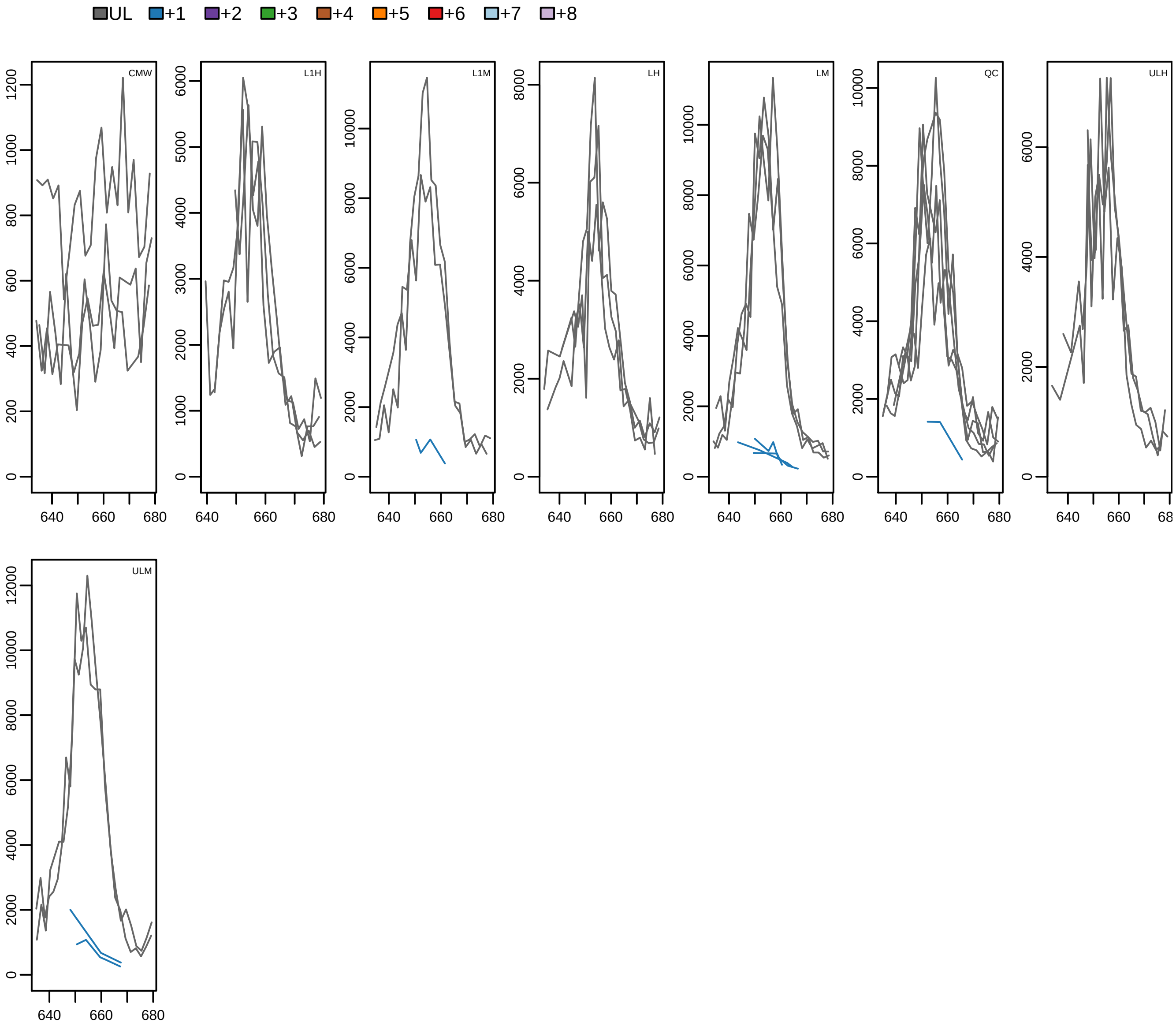

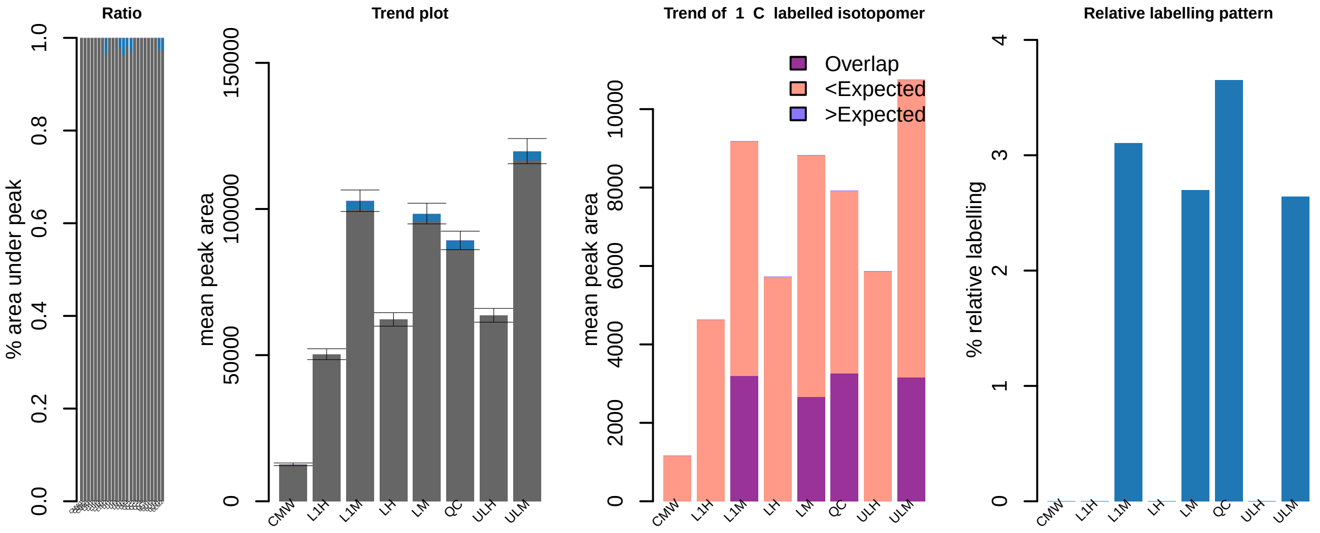

Proclavaminic acid

Formula: C8H14N2O4 Mass: 202.095 Std.RT: 811.7669958 Ion: POS

G1

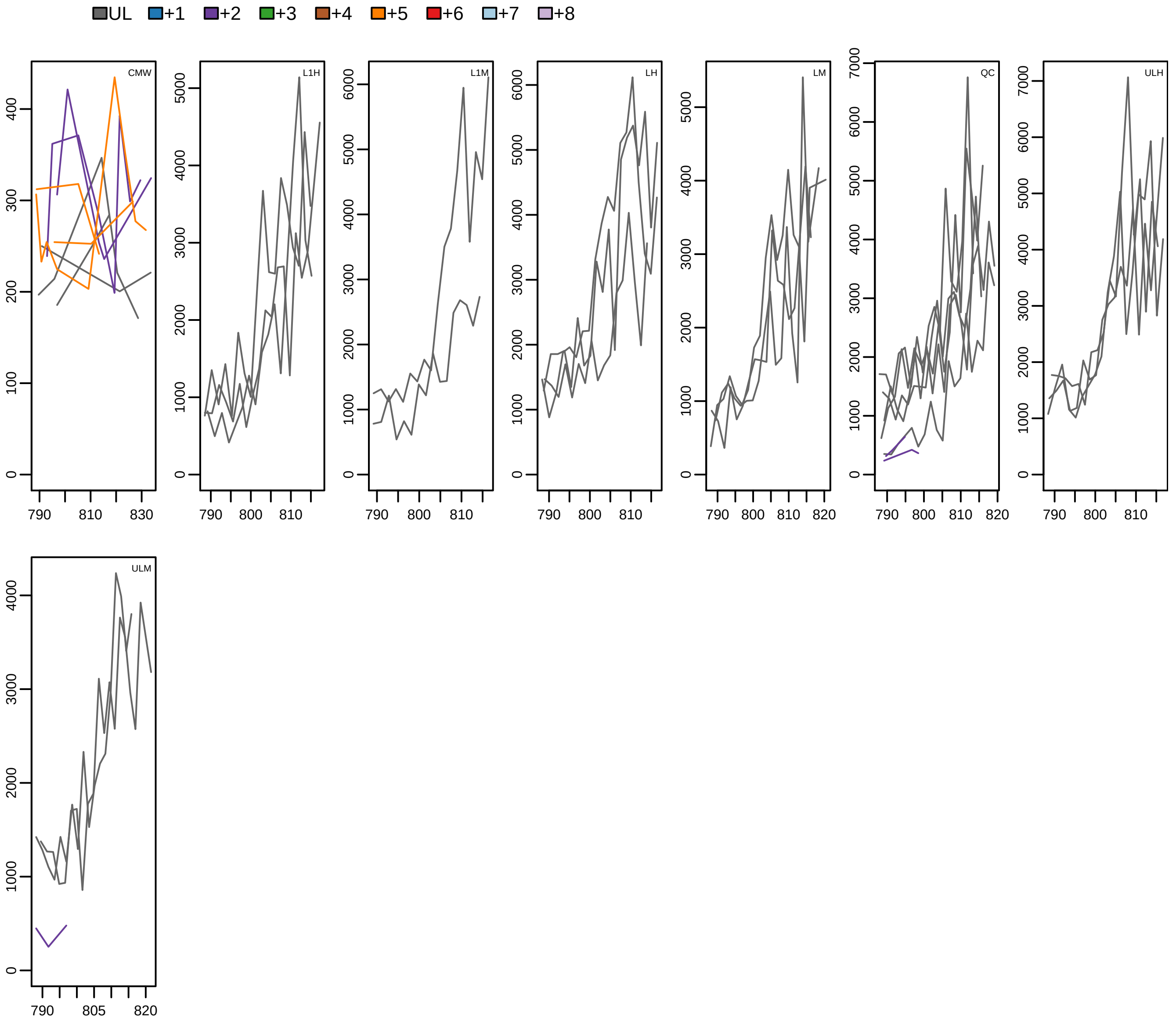

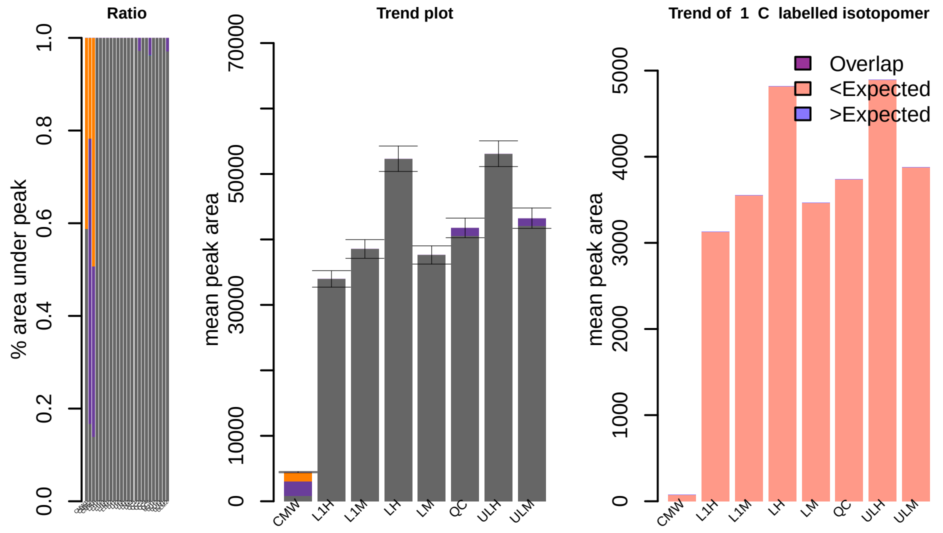

Biochanin A

Formula: $C16H12O5$ Mass: 284.068 Std.RT: 427.66761852 Ion: POS

G1

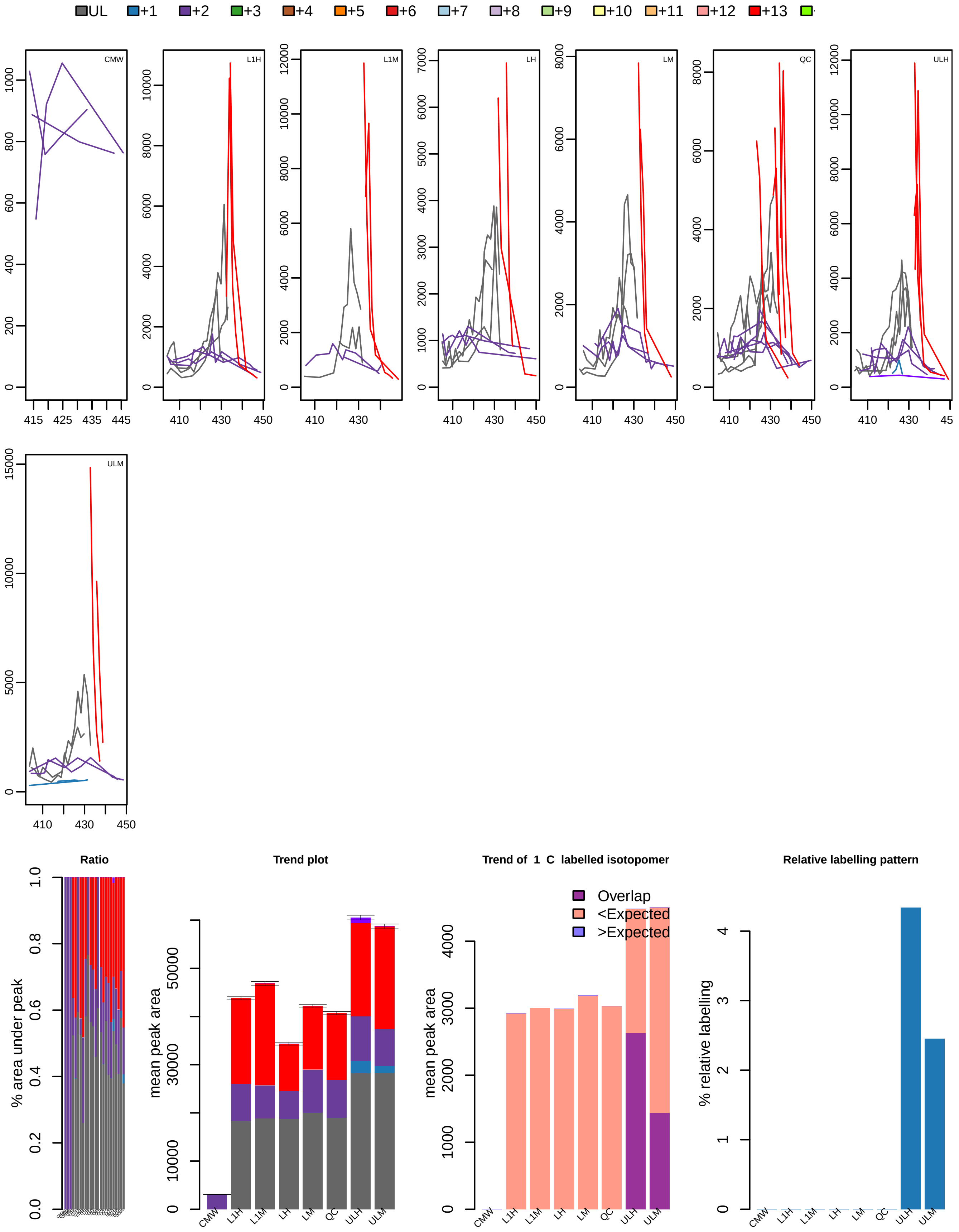

Glycitein

Formula: C16H12O5 Mass: 284.068 Std.RT: 362.65152558 Ion: POS

G1

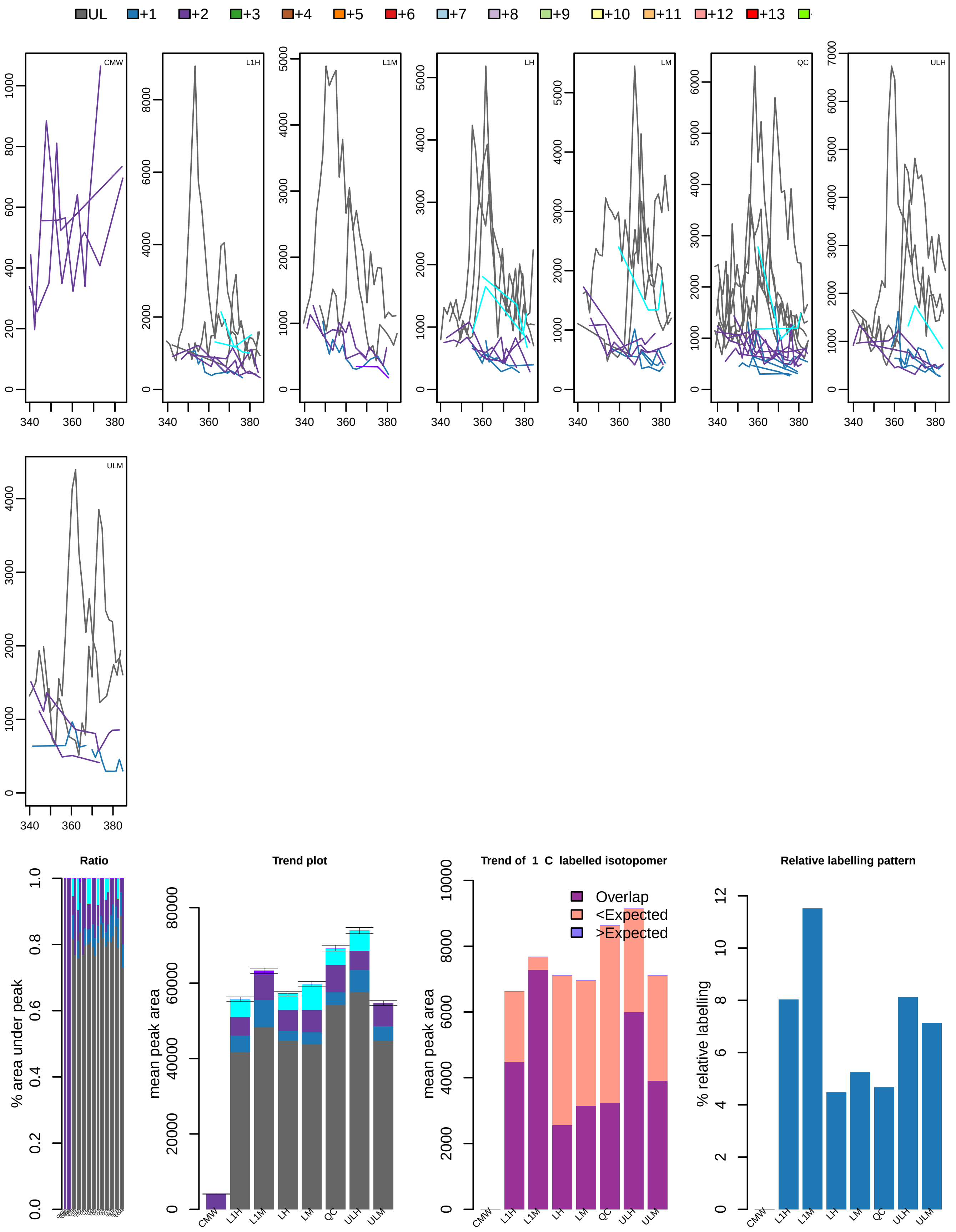

Daidzein

Formula: C15H10O4 Mass: 254.058 Std.RT: 365.47523862 Ion: POS

G1

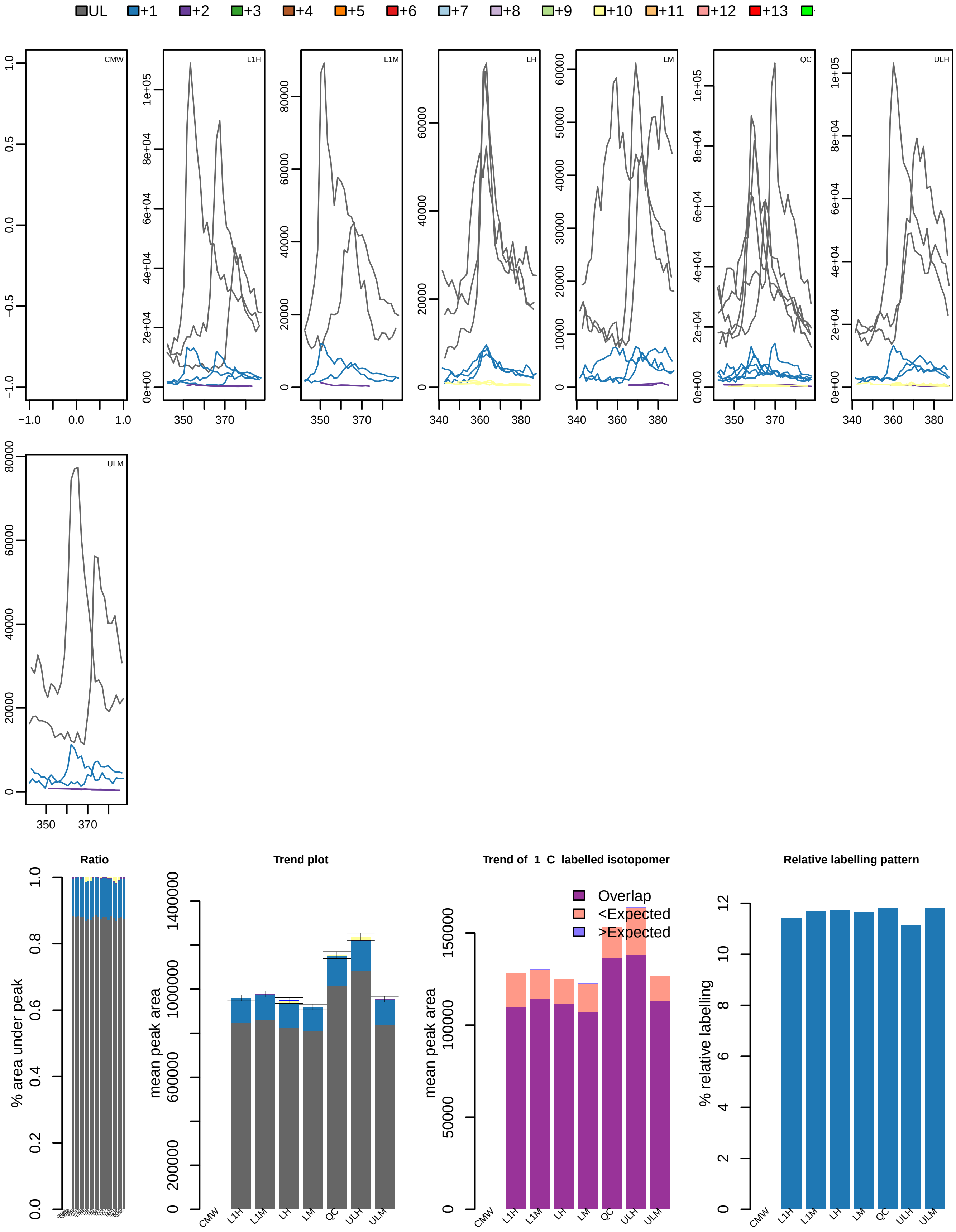

Daidzein

Formula: C15H10O4 Mass: 254.058 Std.RT: 365.47523862 Ion: POS

G2

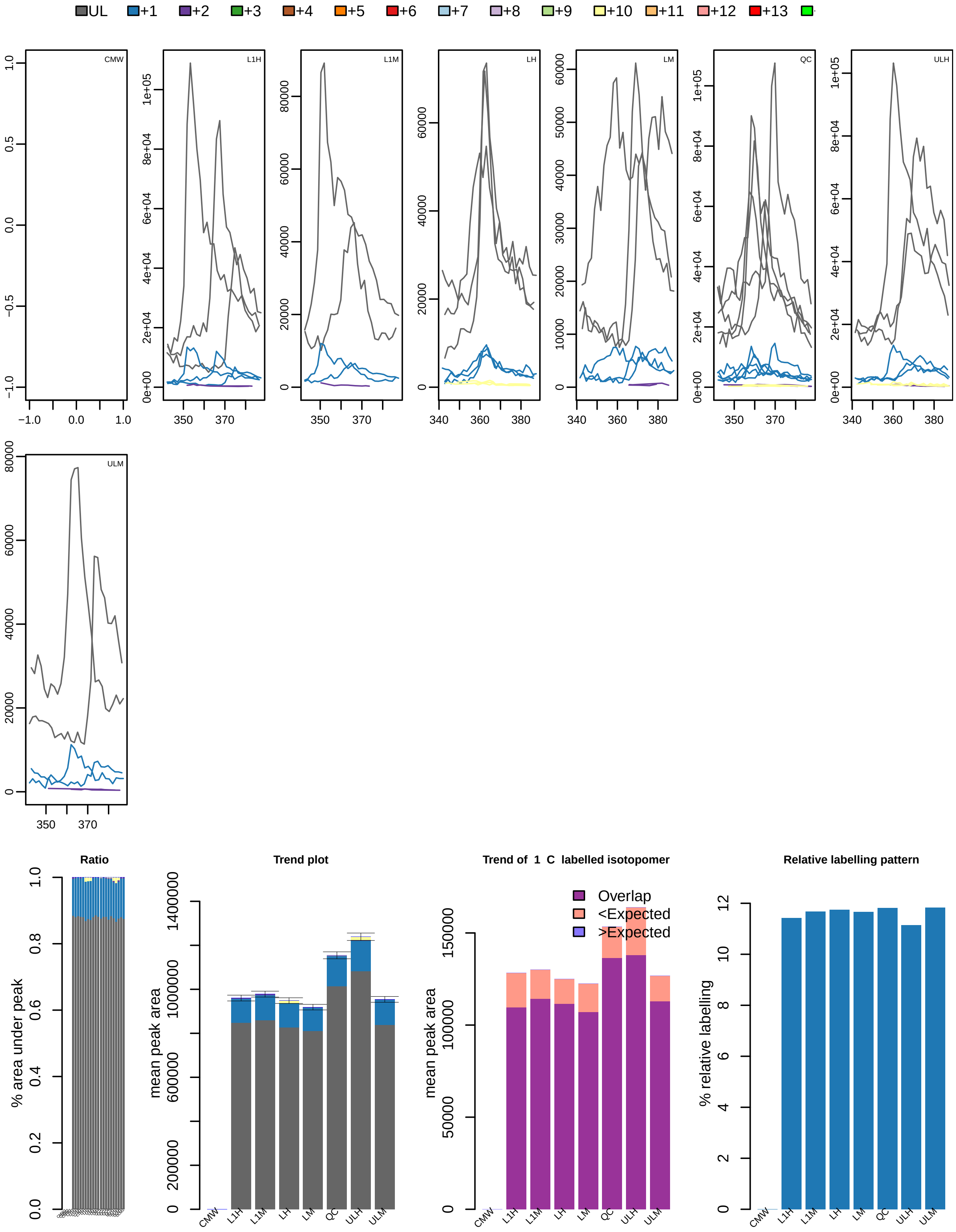

p−Coumaroyl quinic acid

Formula: C16H18O8 Mass: 338.1 Std.RT: 989.6677128 Ion: POS

G1

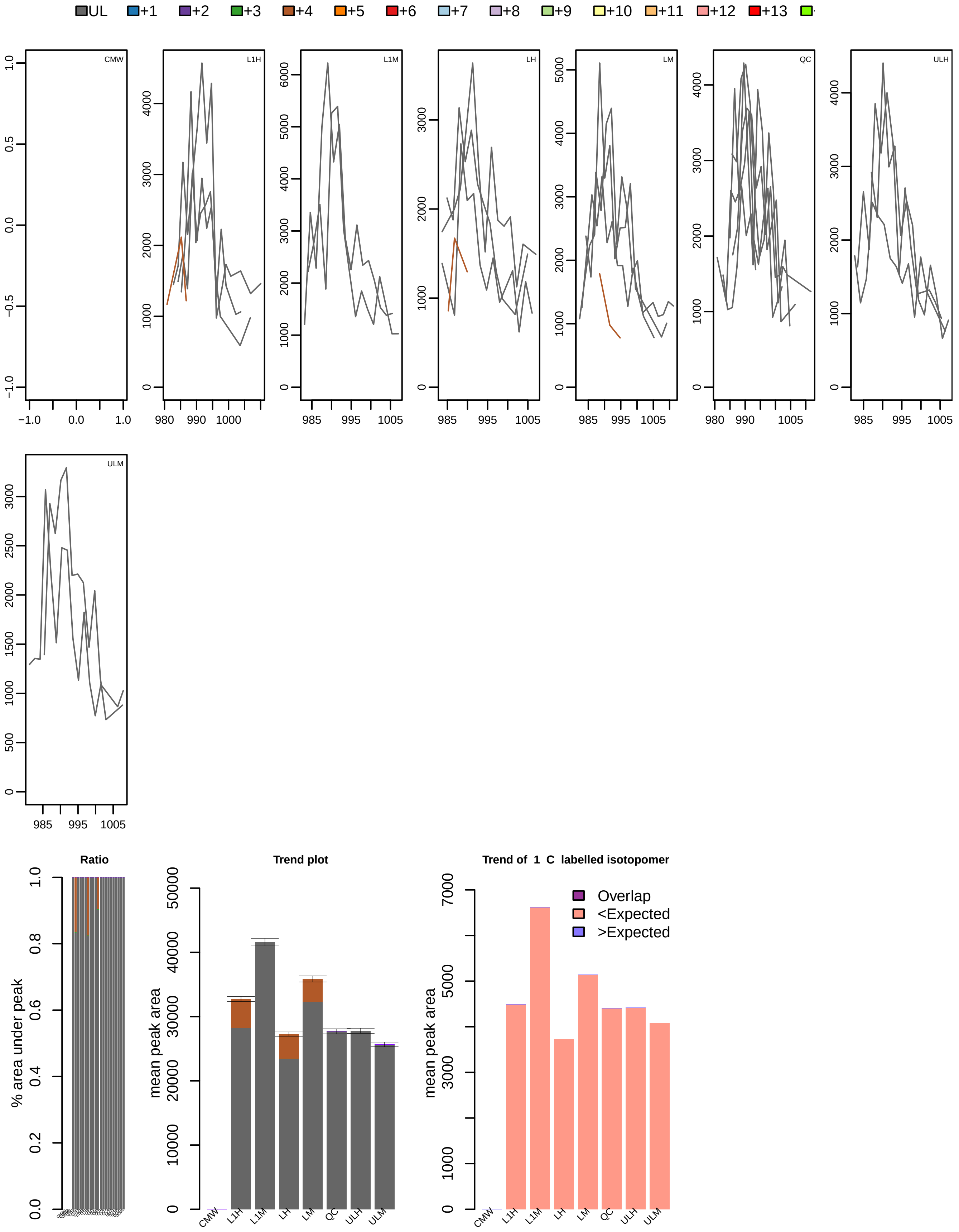

D−Glucosamine

Formula: C6H13NO5 Mass: 179.079 Std.RT: 873.843498 Ion: POS

G1

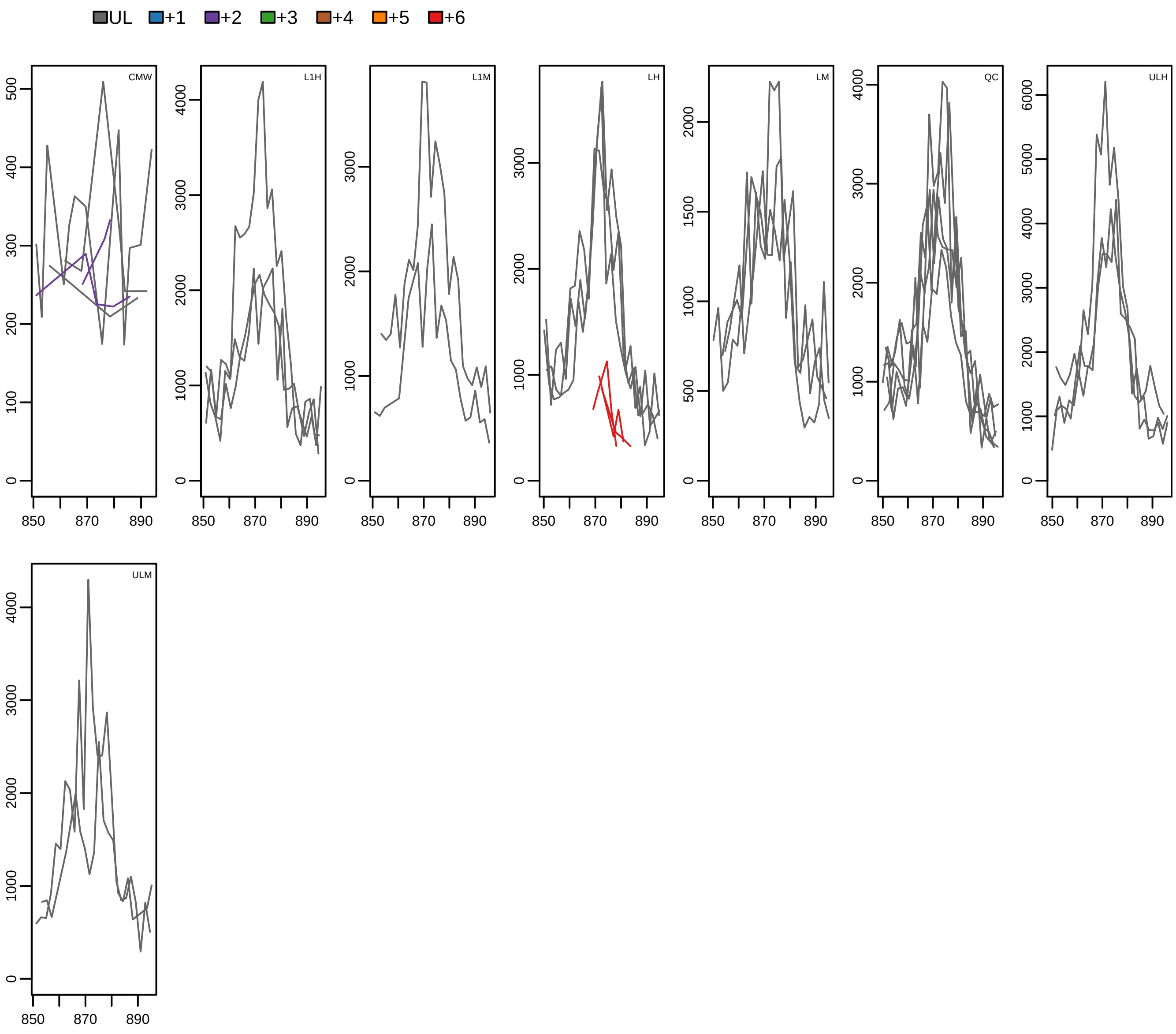

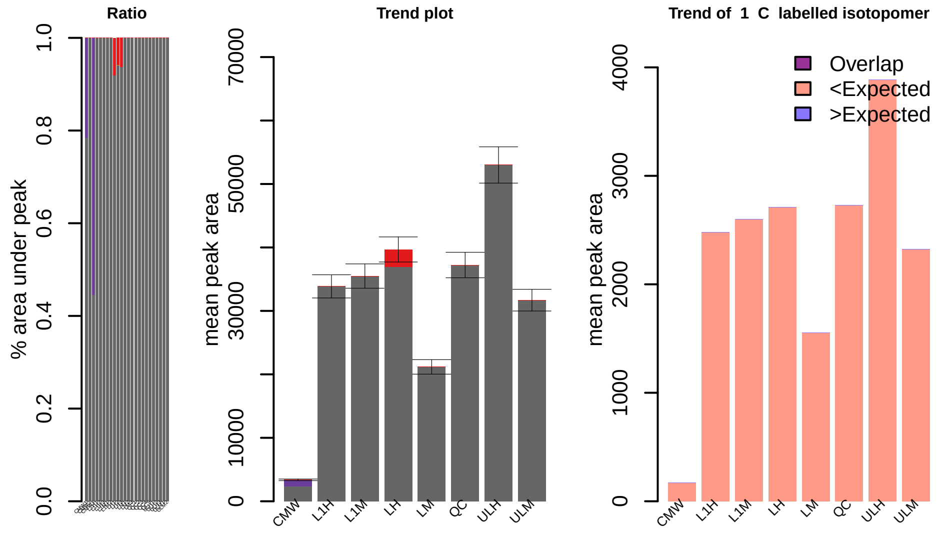

4−Hydroxy−4−methylglutamate

Formula: C6H11NO5 Mass: 177.064 Std.RT: 844.9997154 Ion: POS

G1

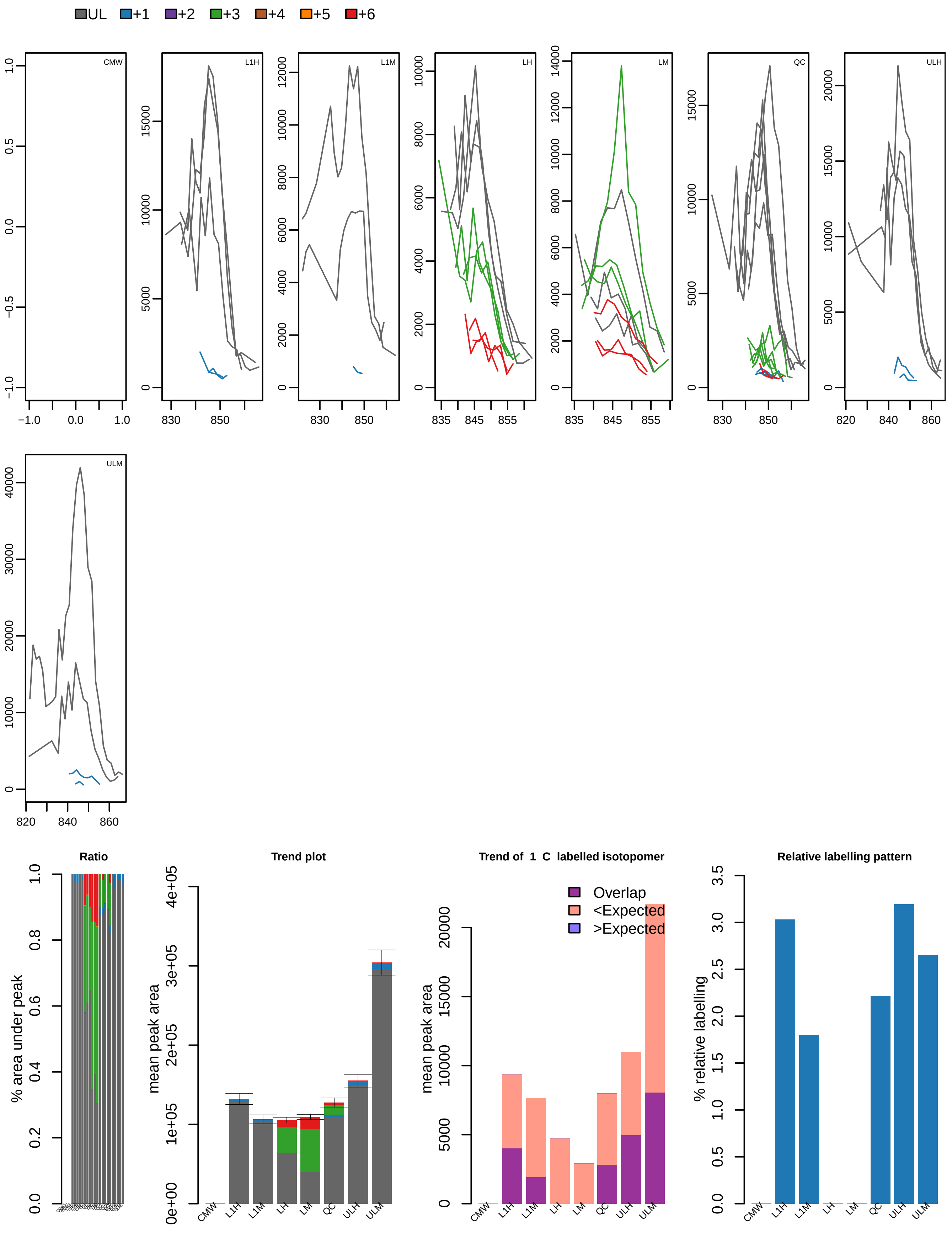

2−(alpha−Hydroxyethyl)thiamine diphosphate

Formula: C14H22N4O8P2S Mass: 468.063 Std.RT: 930.1617084 Ion:

G1

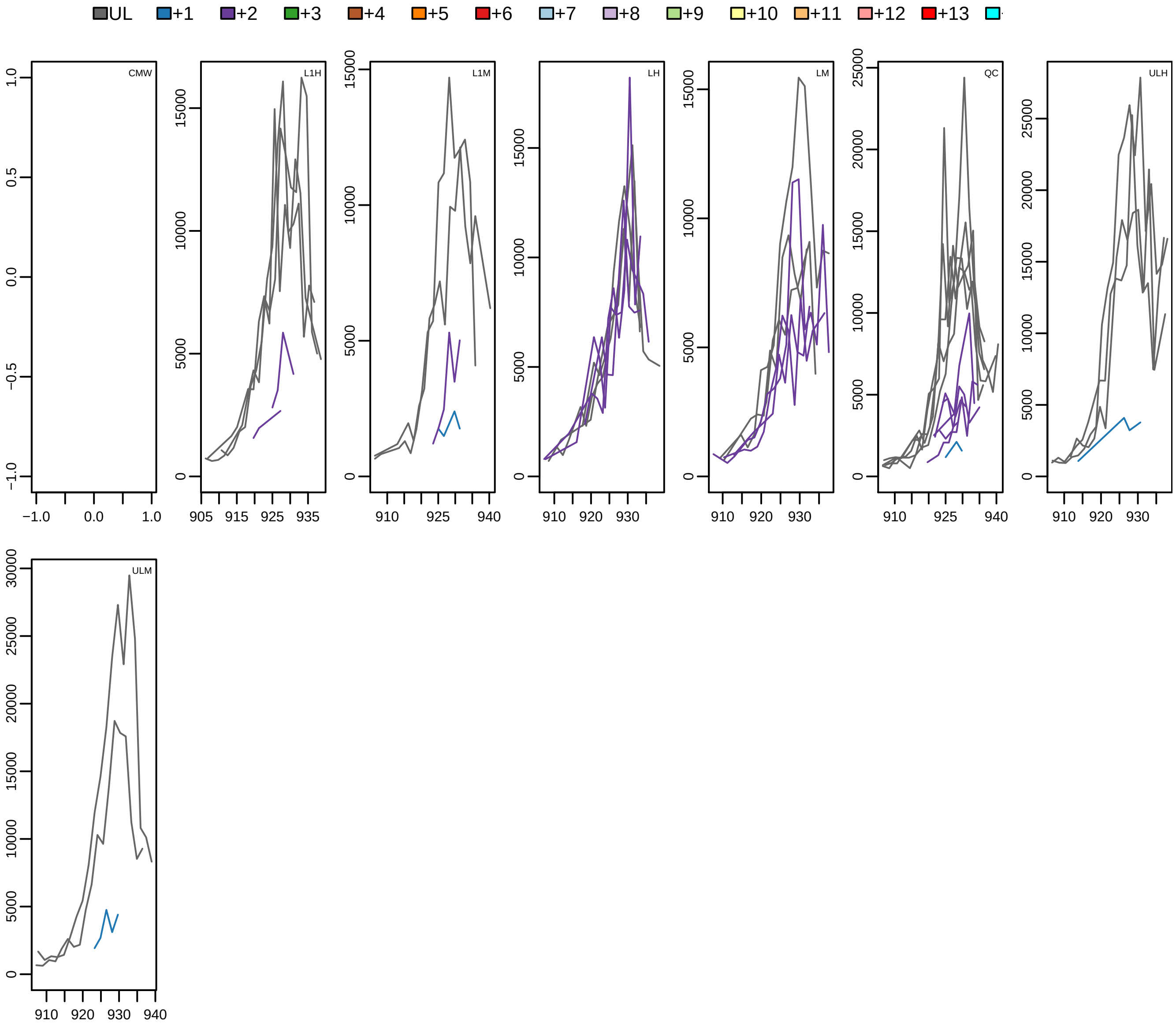

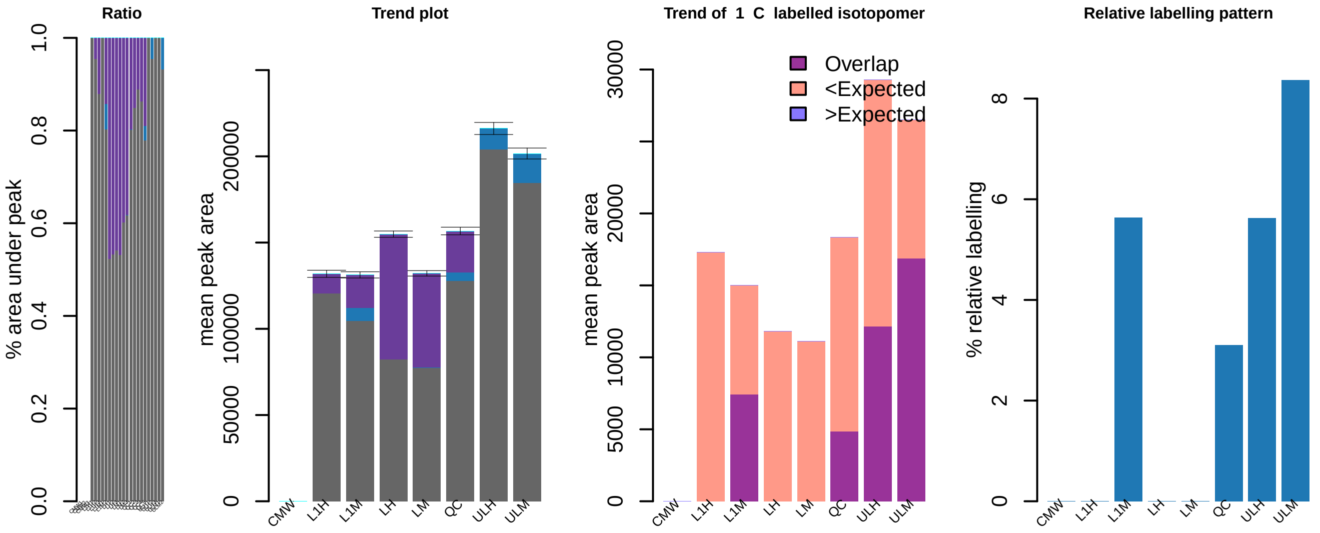

NADH

Formula: C21H29N7O14P2 Mass: 665.125 Std.RT: 868.395174 Ion:

G1

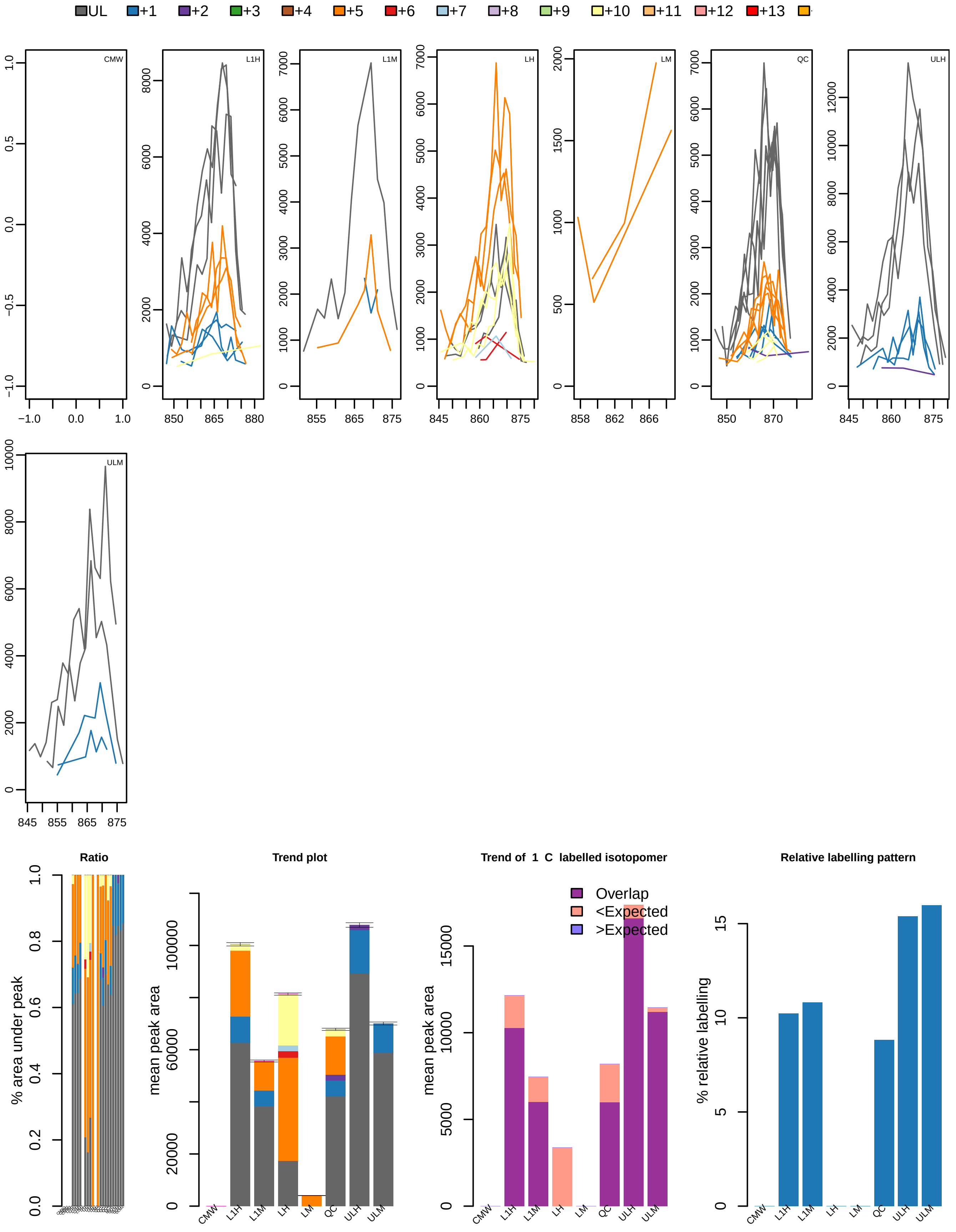

NAD+

Formula: C21H27N7O14P2 Mass: 663.109 Std.RT: 929.2991418 Ion:

G1

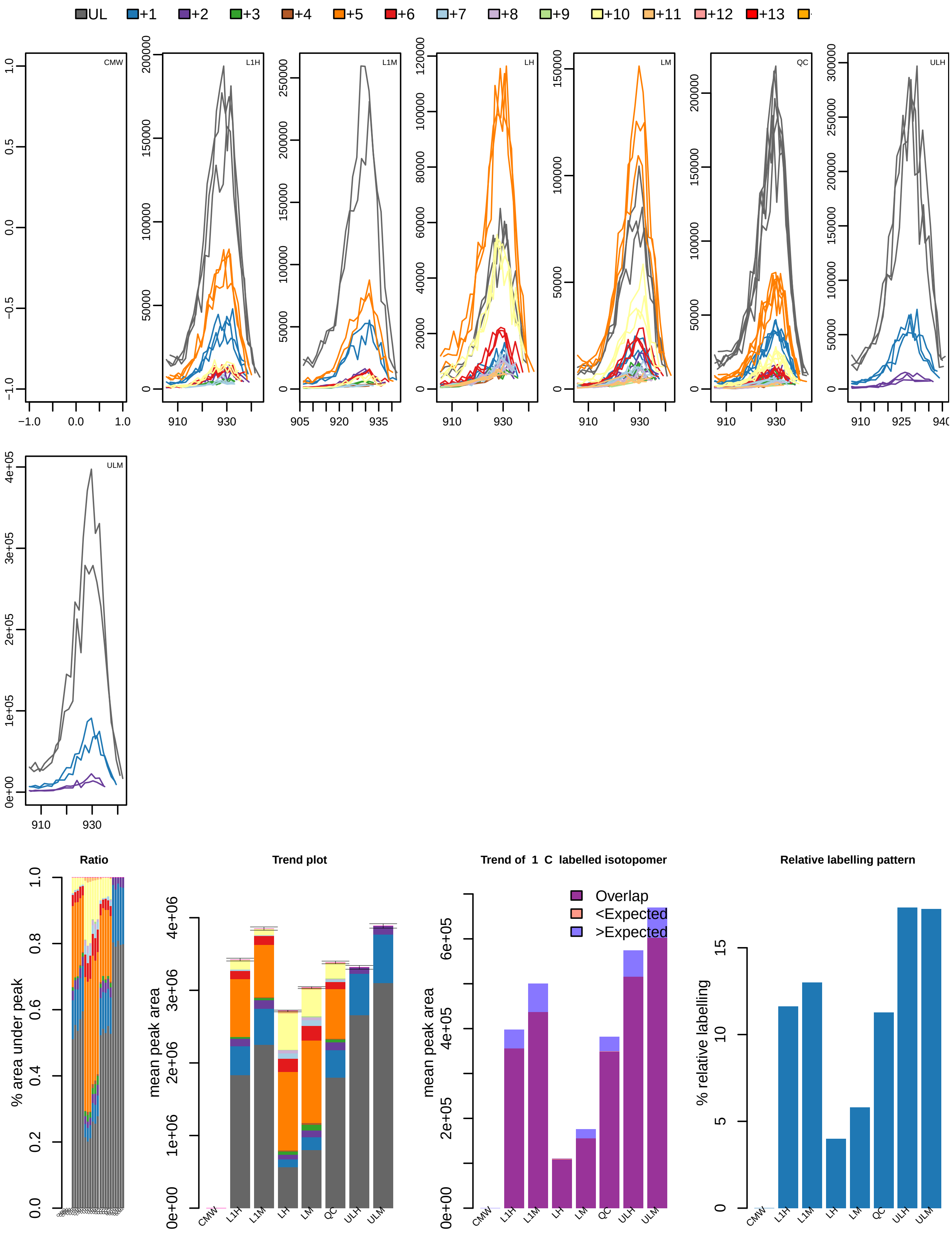

CDP−choline

Formula: C14H26N4O11P2 Mass: 488.107 Std.RT: 1004.436666 Ion:

G1

UL +1 +2 +3 +4 +5 +6 +7 +8 +9 +10 +11 +12 +13

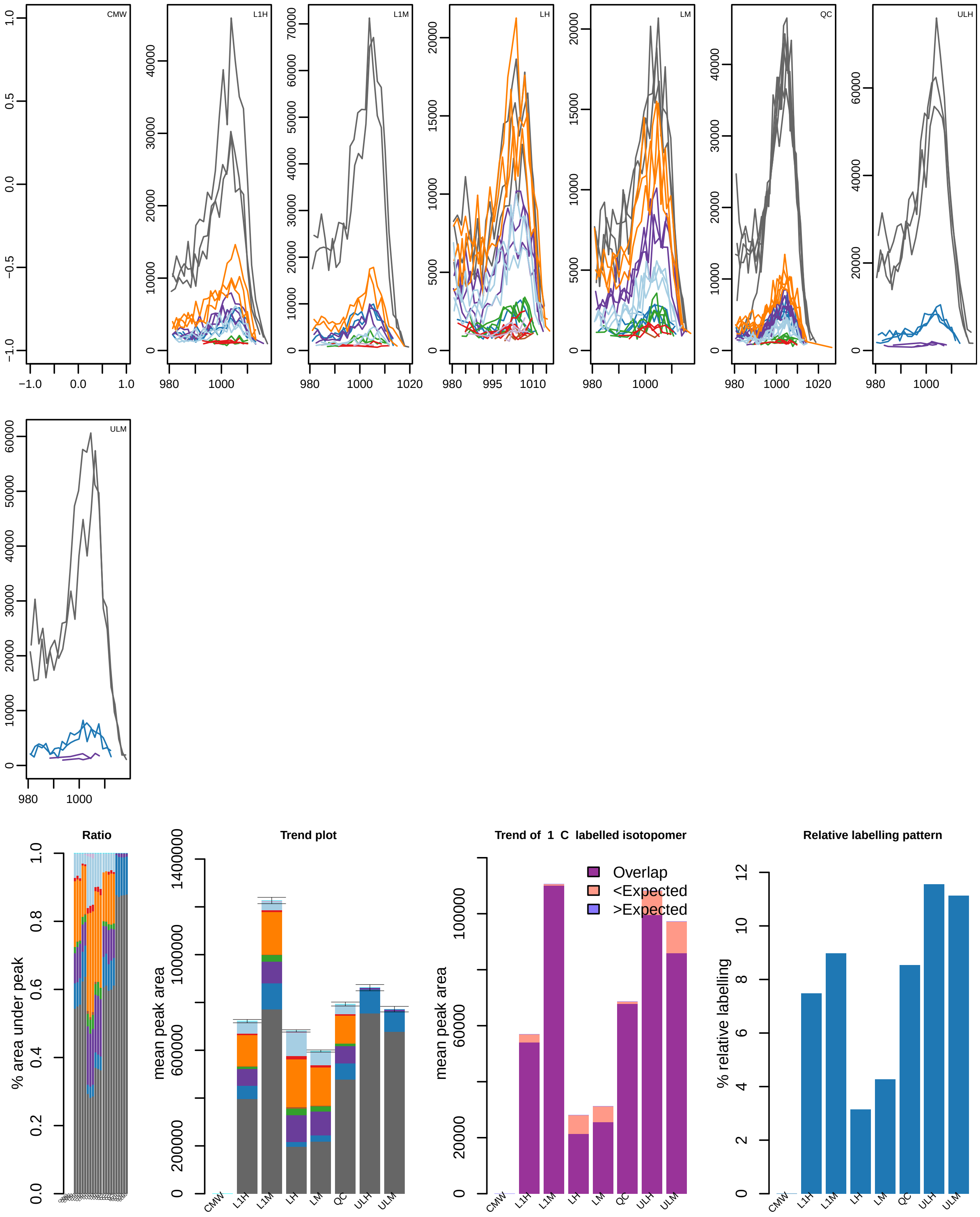

Phosphodimethylethanolamine

Formula: C4H12NO4P Mass: 169.05 Std.RT: 925.9777164 Ion: POS

G1

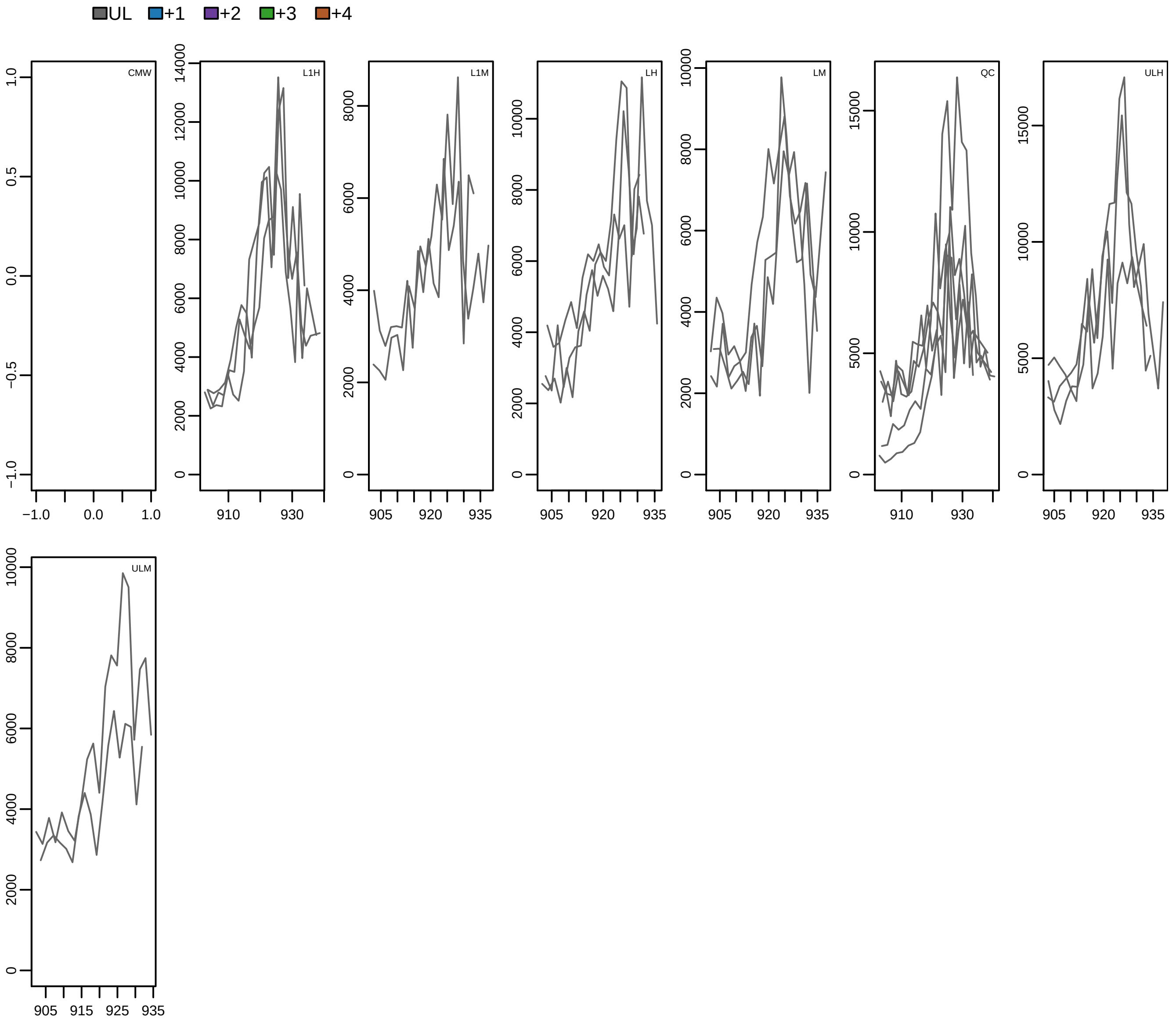

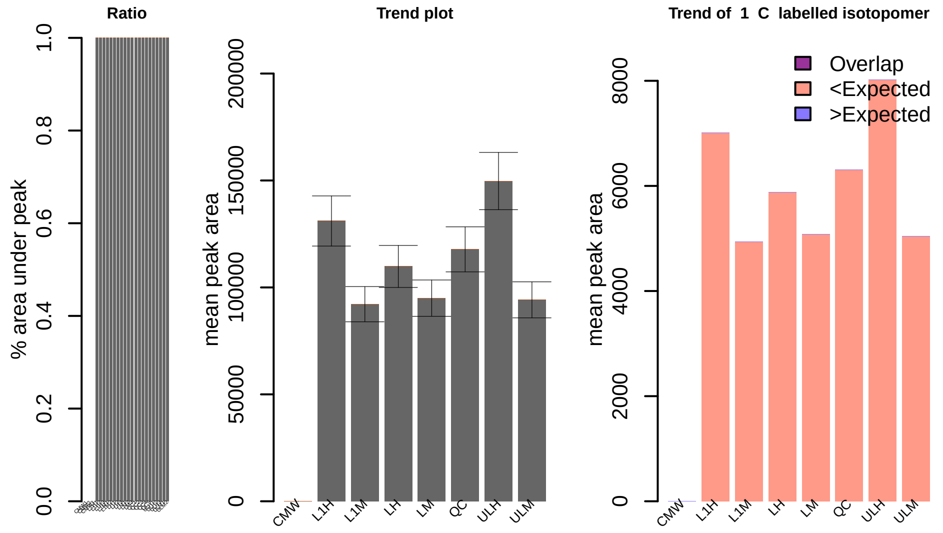

Phosphodimethylethanolamine

G2

Formula: C4H12NO4P Mass: 169.05 Std.RT: 925.9777164 Ion: POS

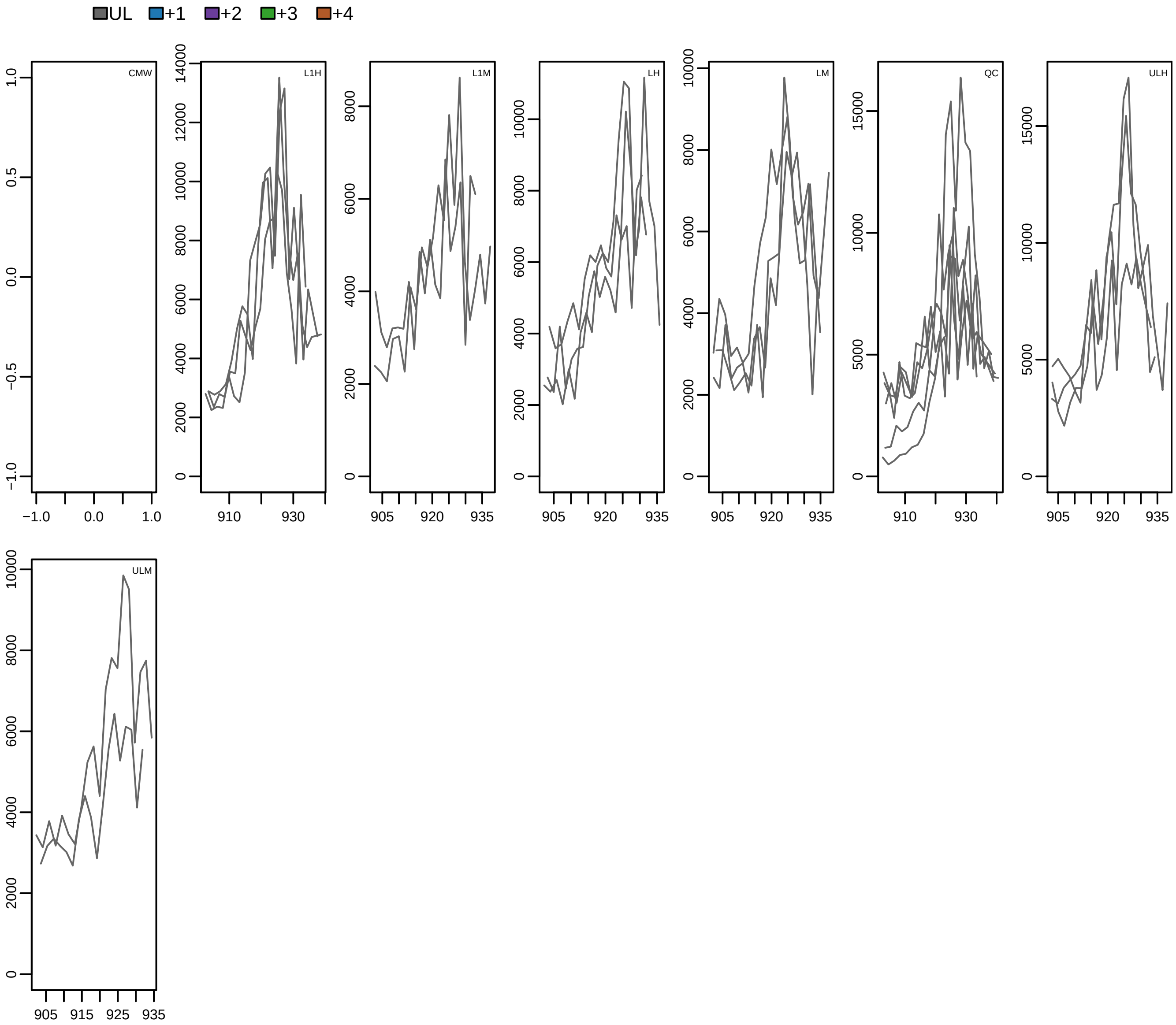

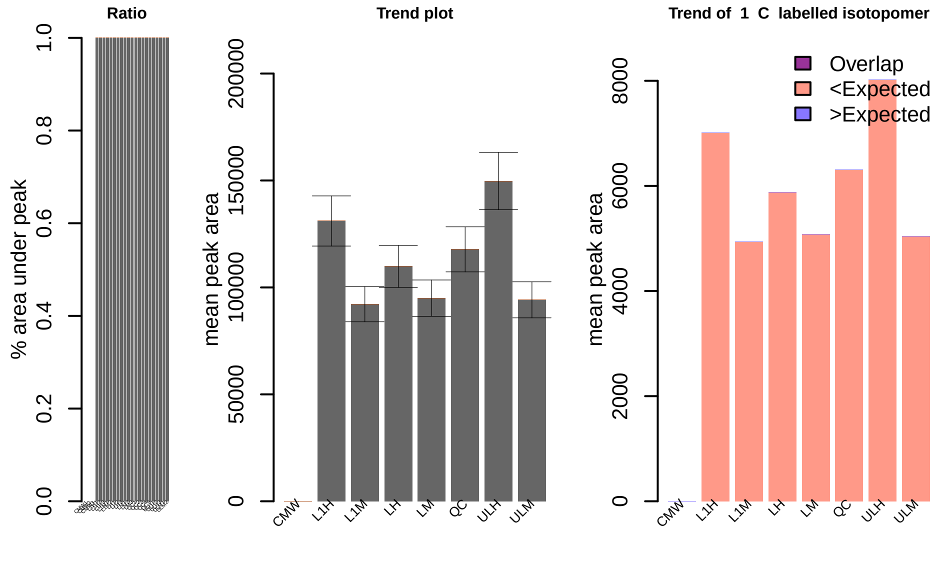

N−Methylethanolamine phosphate

Formula: C3H10NO4P Mass: 155.035 Std.RT: 984.875427 Ion: POS

G1

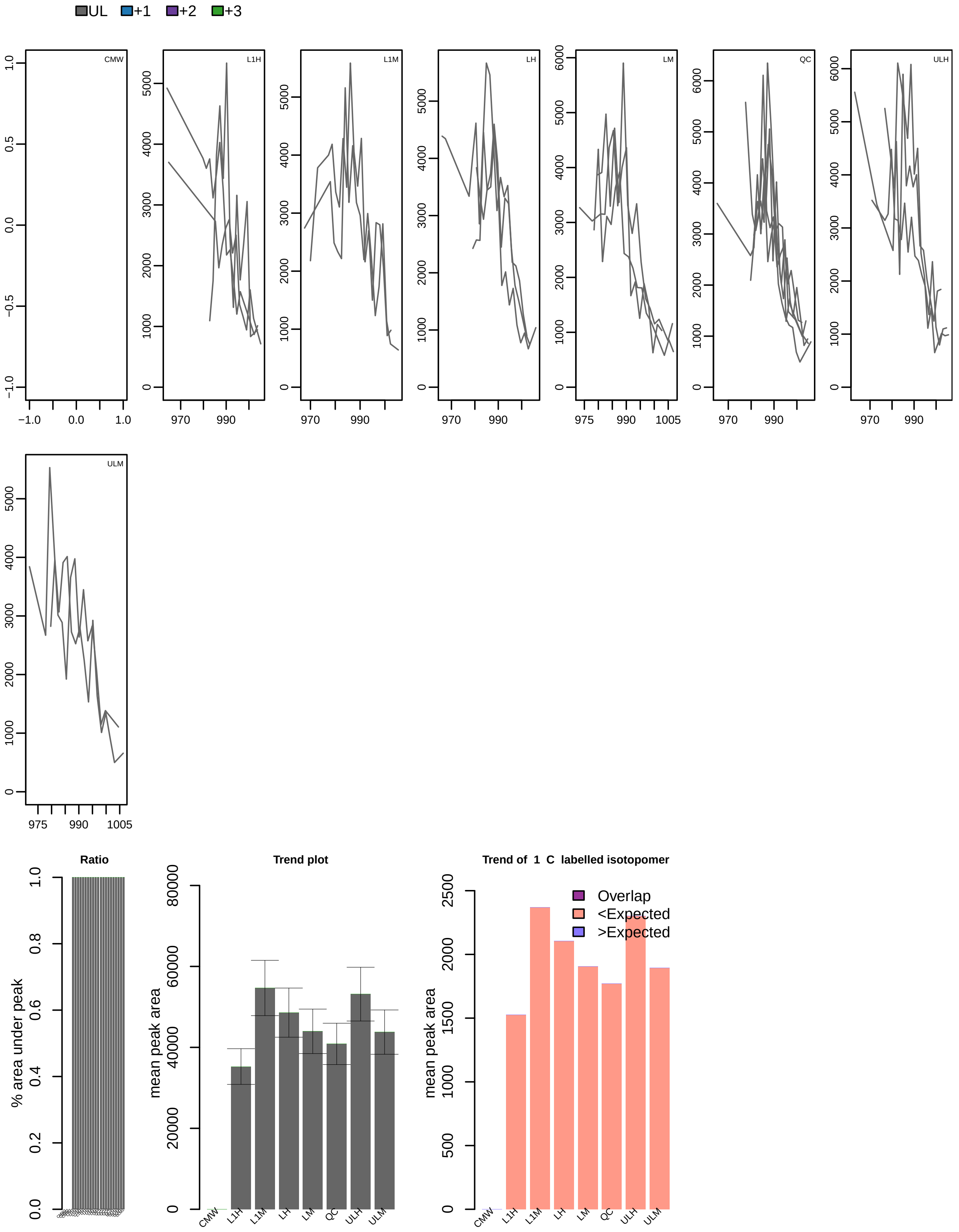

Triethanolamine

G1

Formula: C6H15NO3 Mass: 149.105 Std.RT: 568.03507998 Ion: POS

UL +1 +2 +3 +4 +5 +6

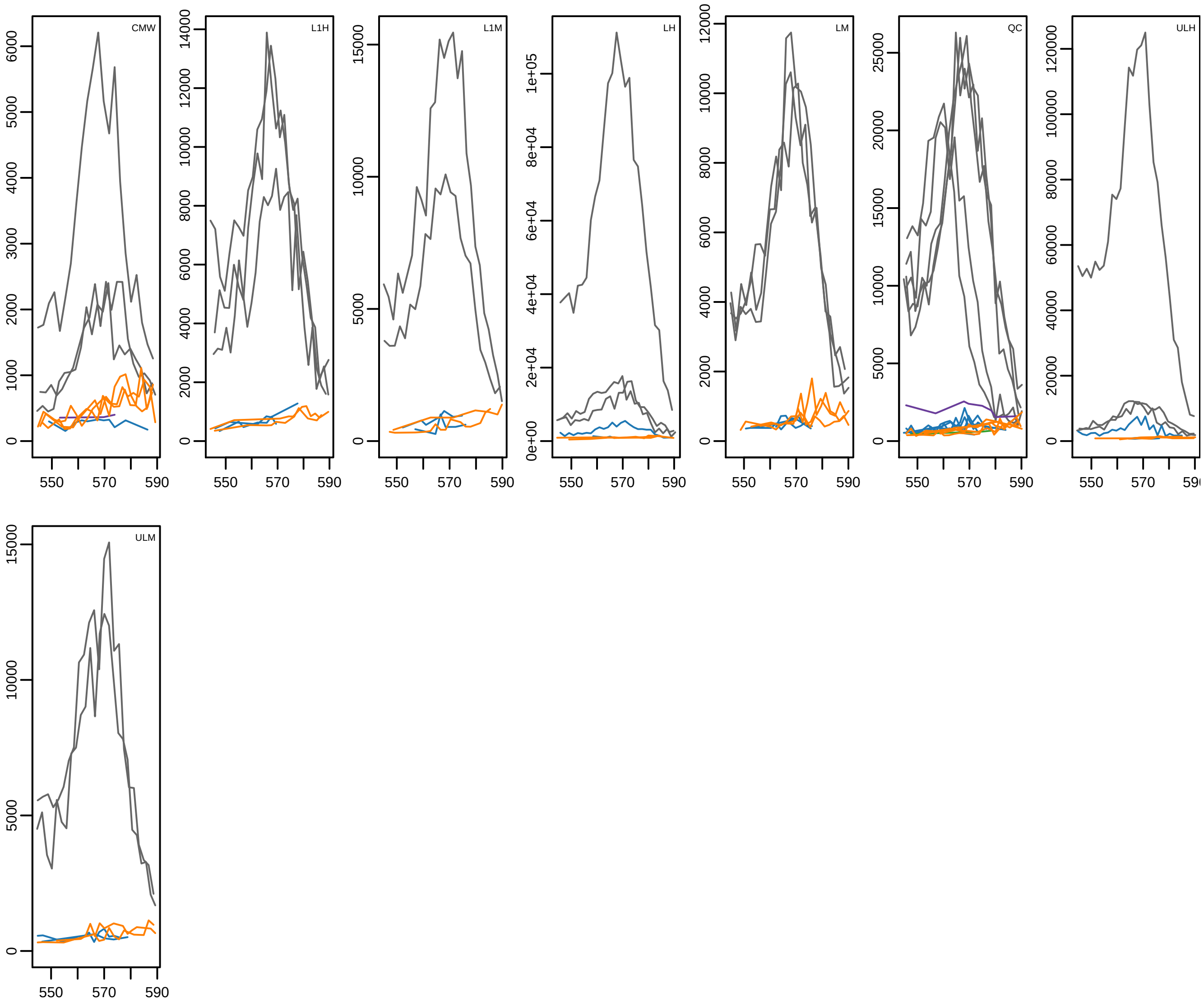

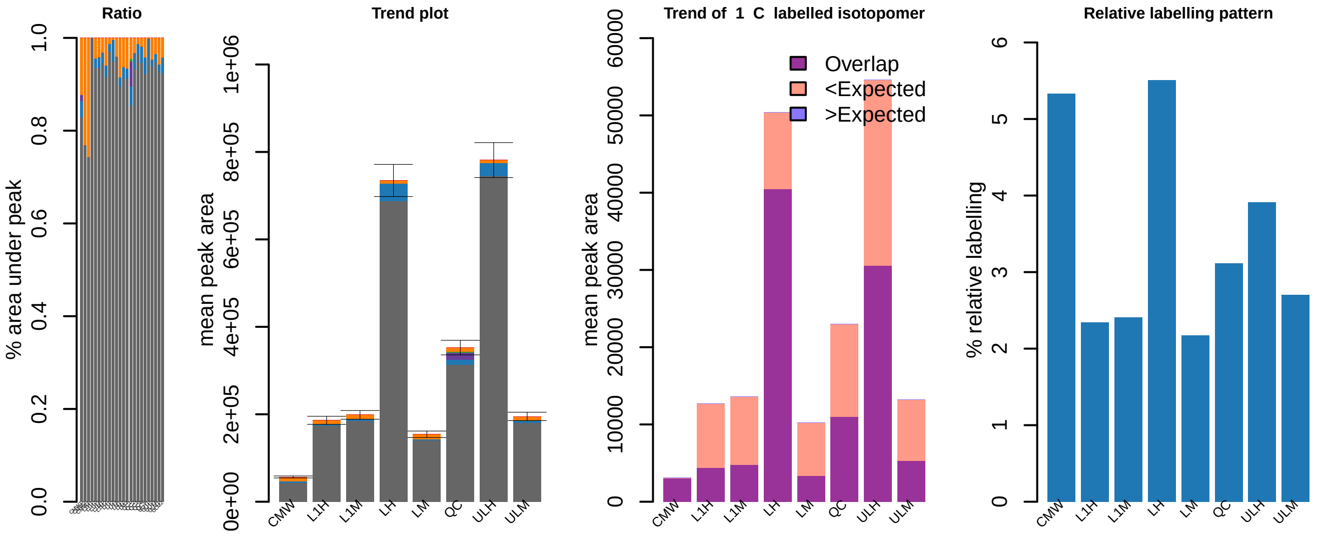

Triethanolamine

Formula: C6H15NO3 Mass: 149.105 Std.RT: 568.03507998 Ion: POS

G2

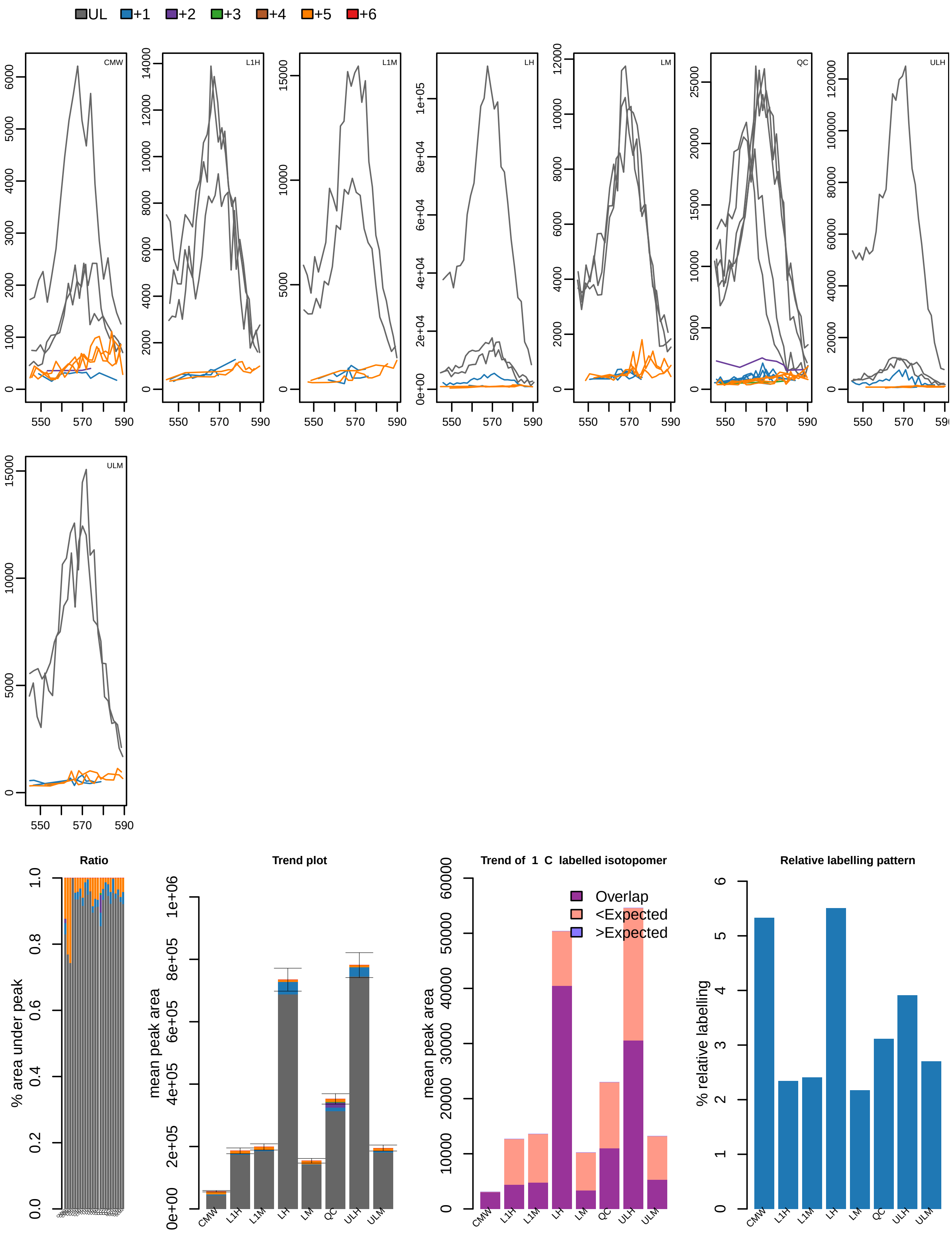

Acetylcholine

Formula: C7H15NO2 Mass: 145.11 Std.RT: 1005.5698068 Ion: POS

G1

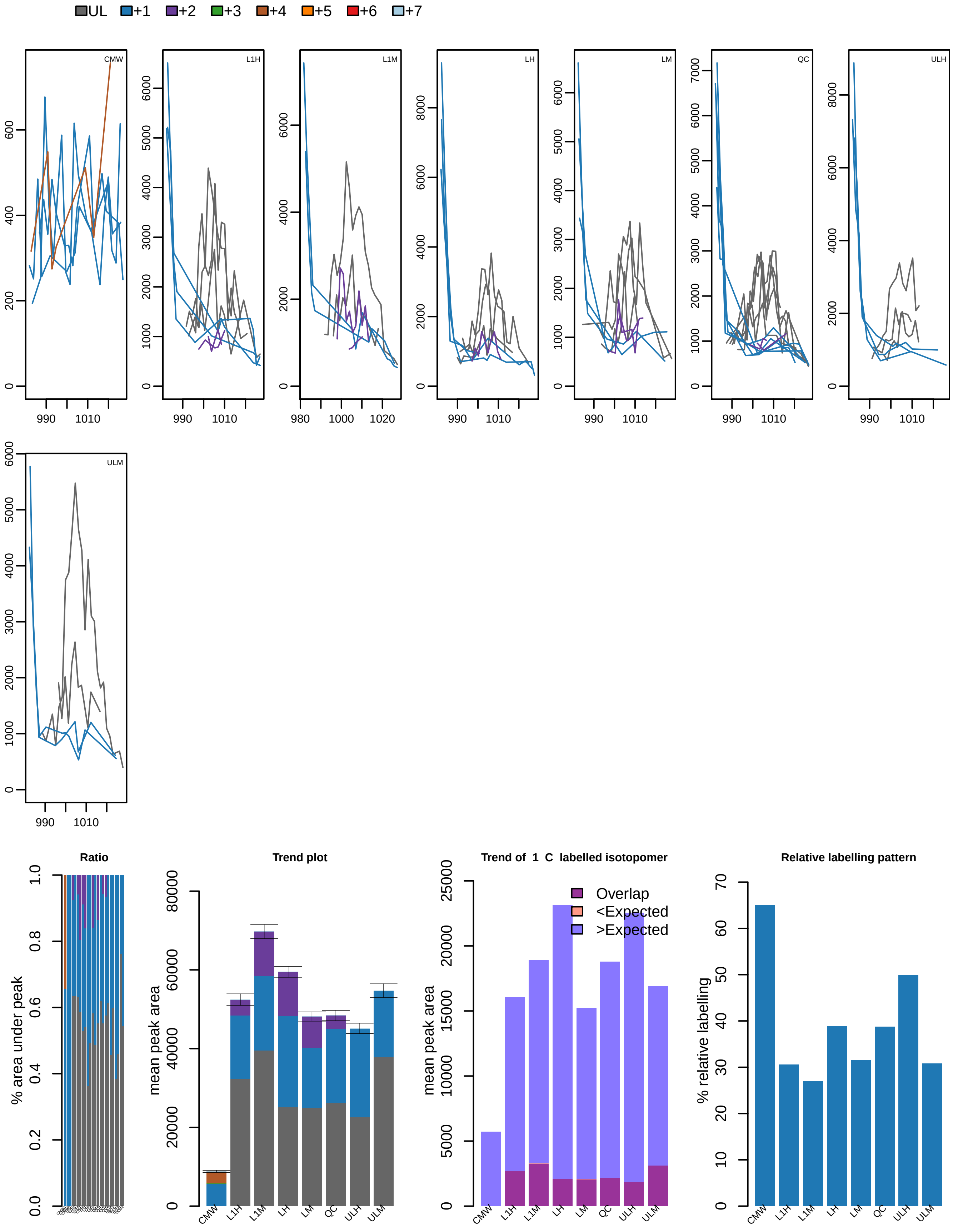

sn−glycero−3−Phosphoethanolamine

Formula: C5H14NO6P Mass: 215.056 Std.RT: 1015.3776192 Ion: PC

G1

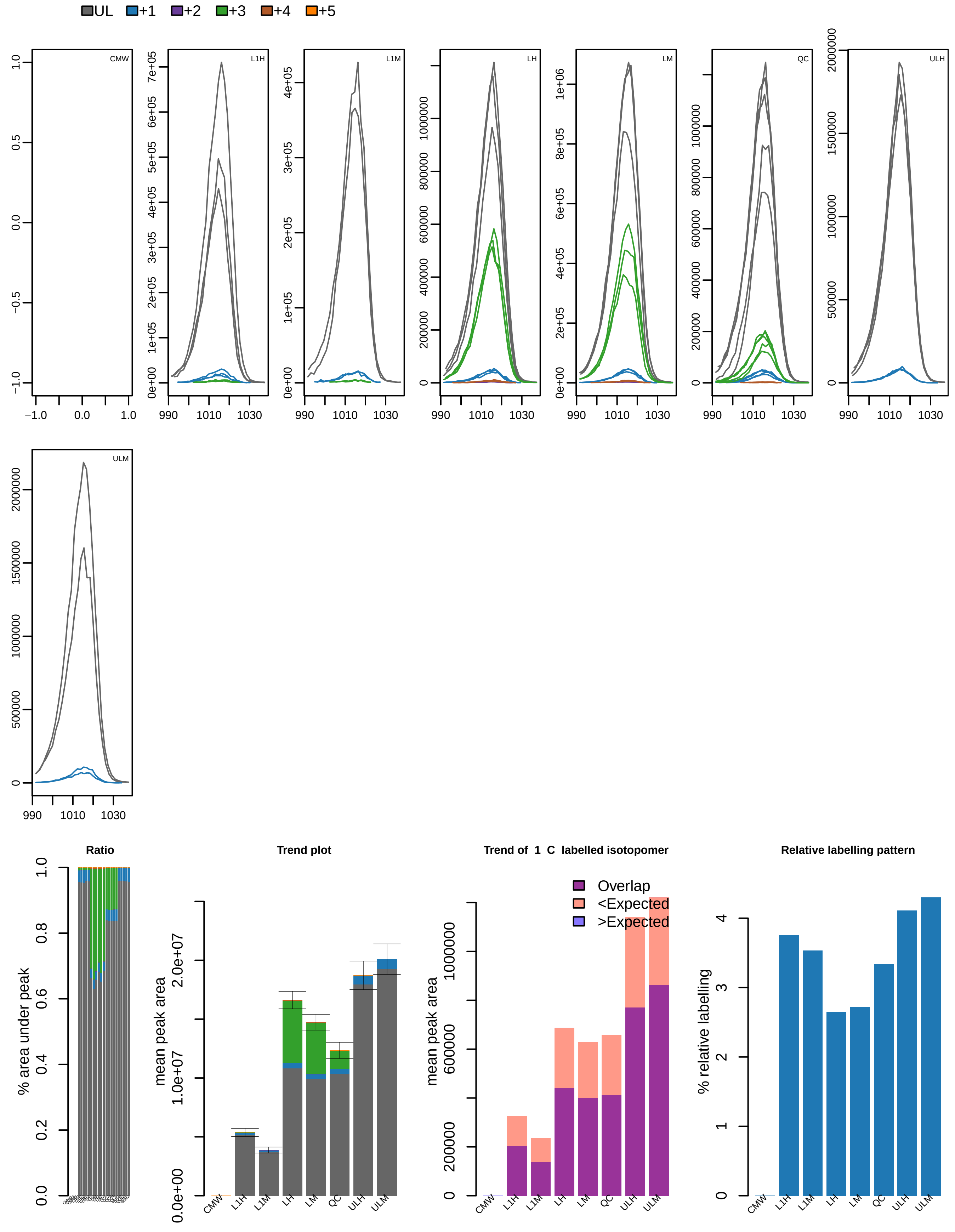

sn−glycero−3−Phosphocholine

G1

Formula: C8H20NO6P Mass: 257.103 Std.RT: 948.5170008 Ion: PO[illegible]

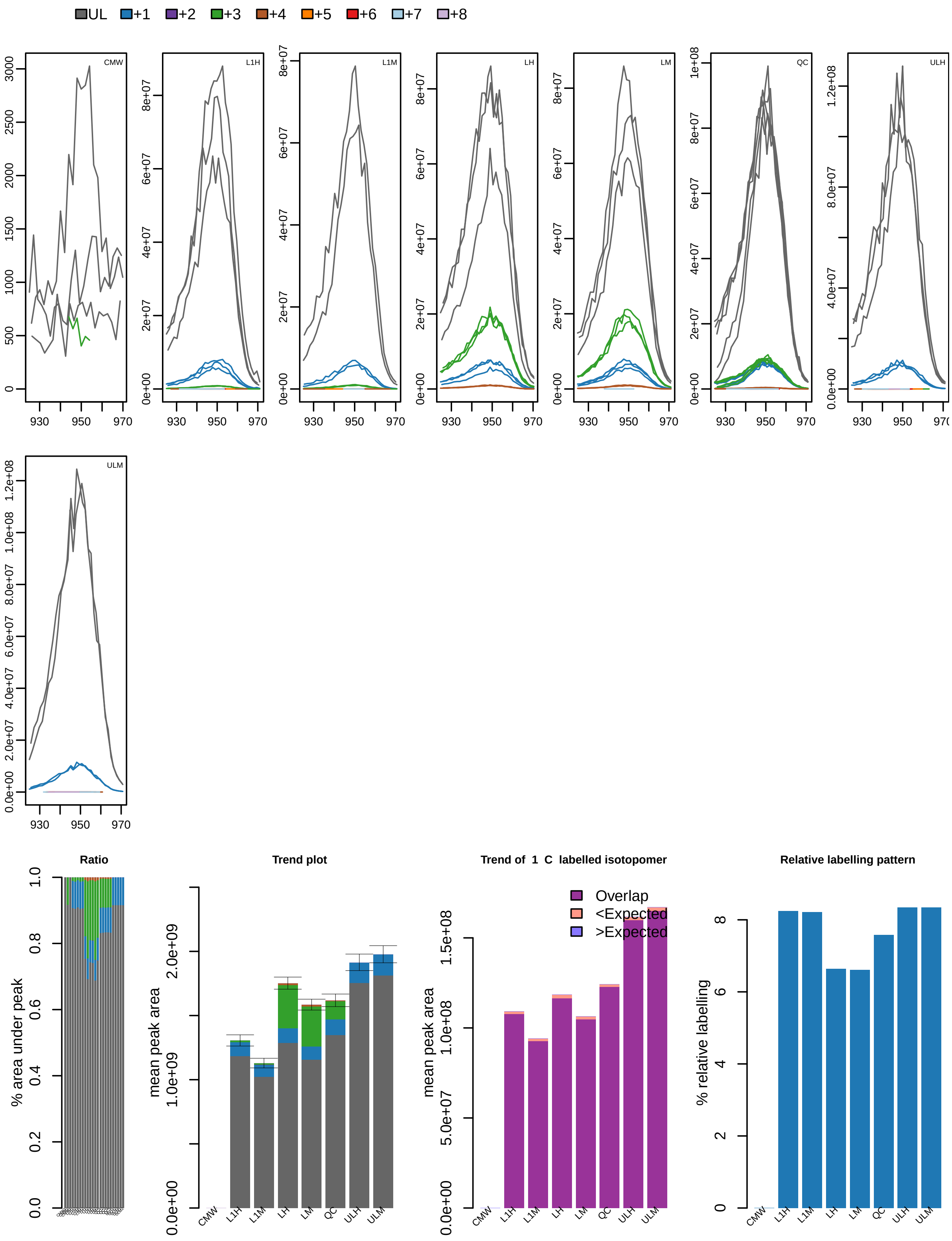

Choline phosphate

Formula: C5H14NO4P Mass: 183.066 Std.RT: 1009.3339128 Ion: PC

G1

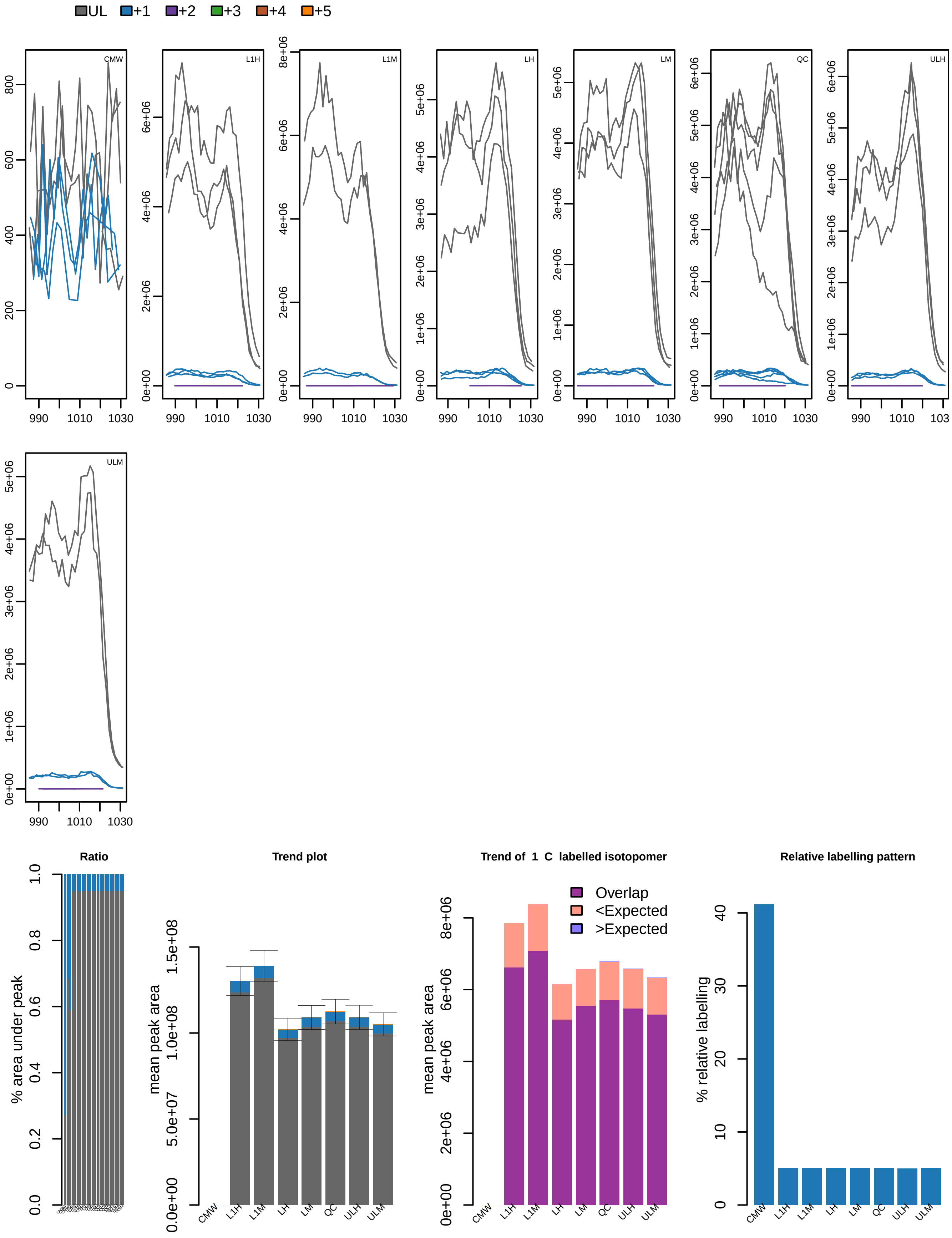

4−8dimethylnonanoylcarnitine

Formula: C18H35NO4 Mass: 329.257 Std.RT: 220.18637532 Ion: PC

G1

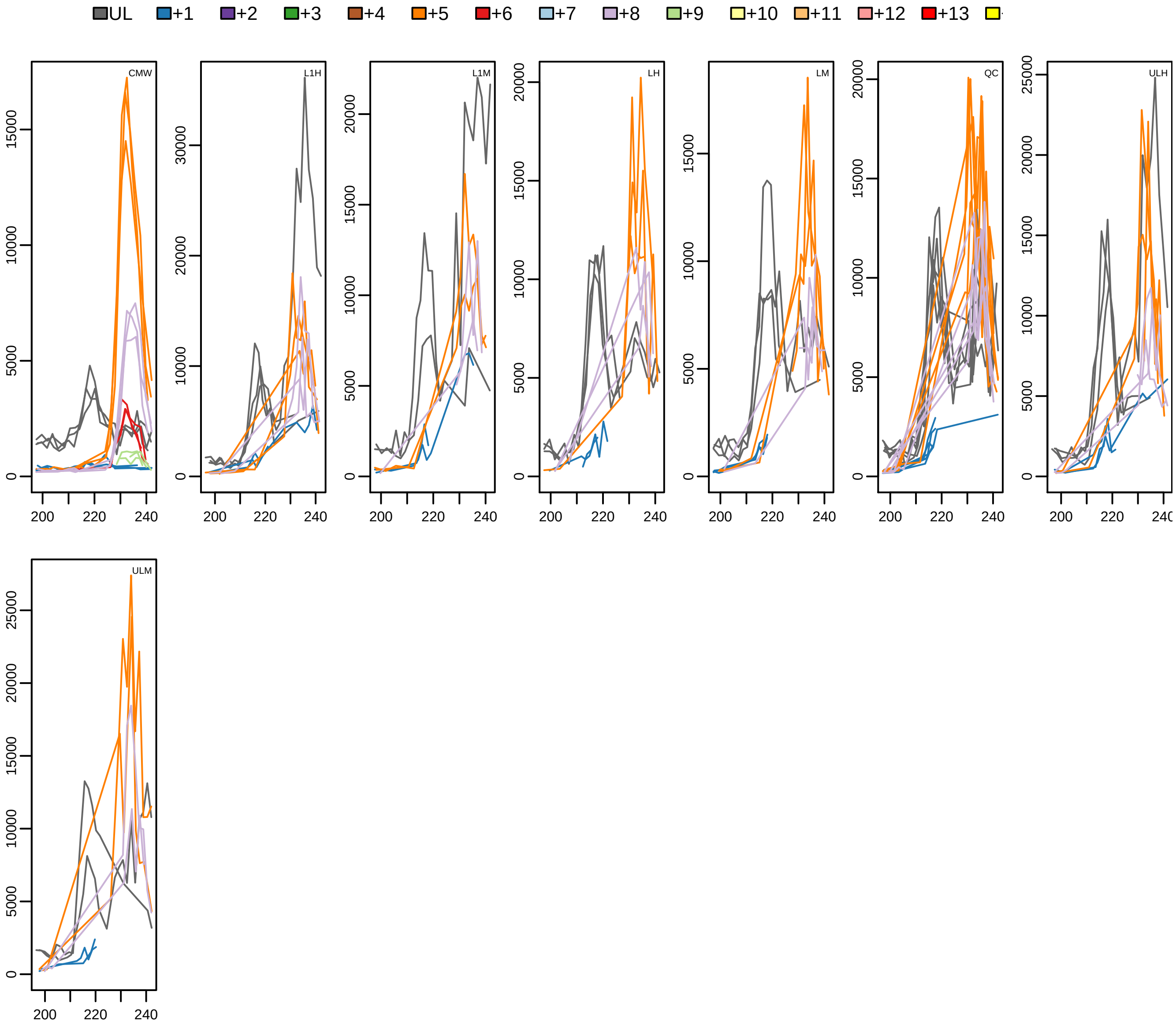

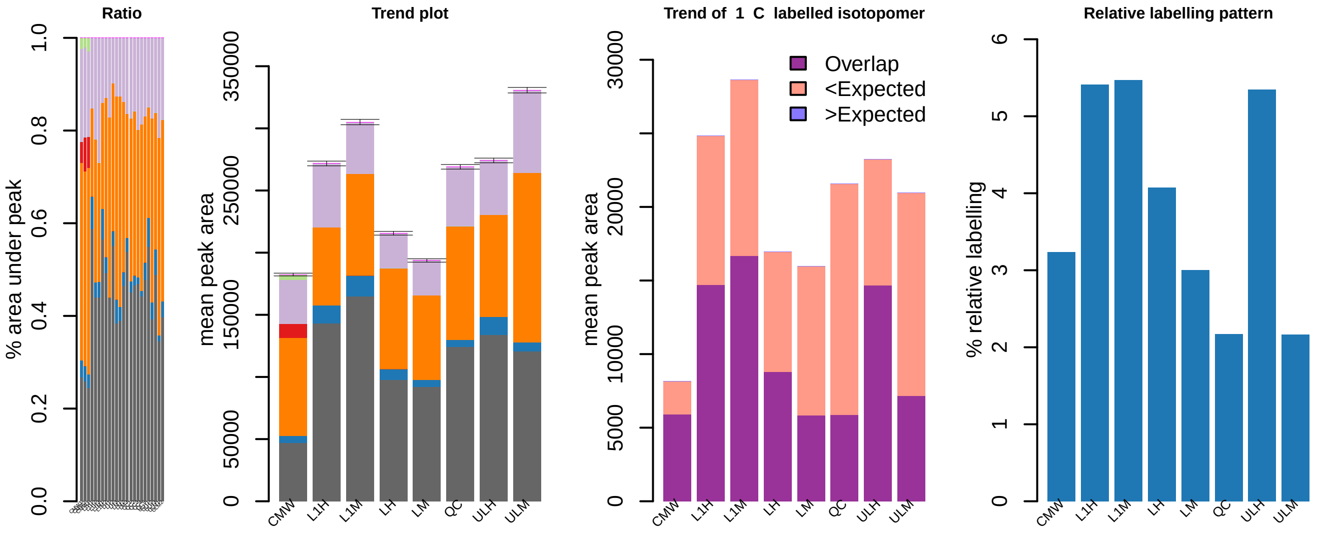

2−Butenoate

Formula: $C_4H_6O_2$ Mass: 86.037 Std.RT: 901.0027542 Ion: POS

G1

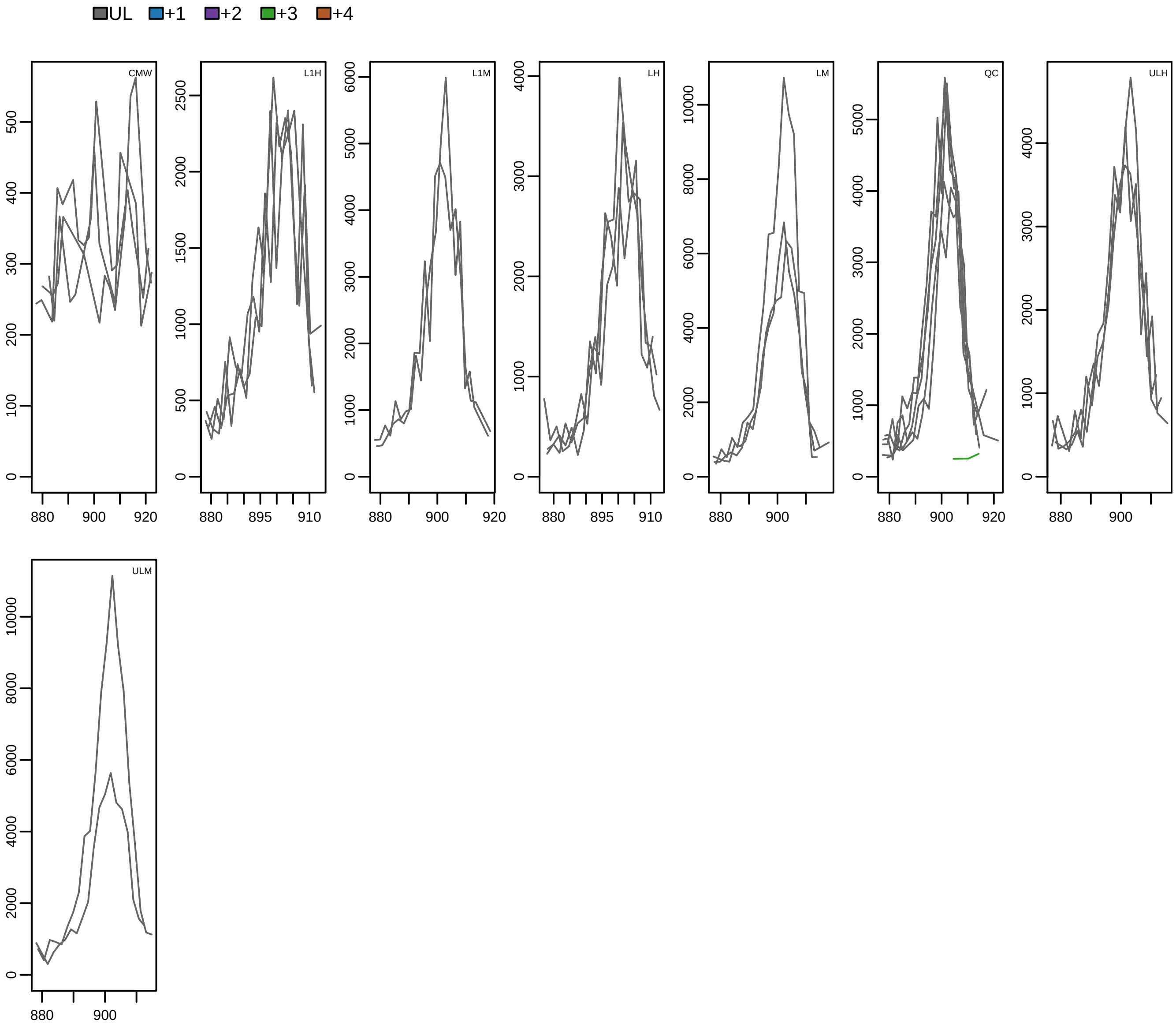

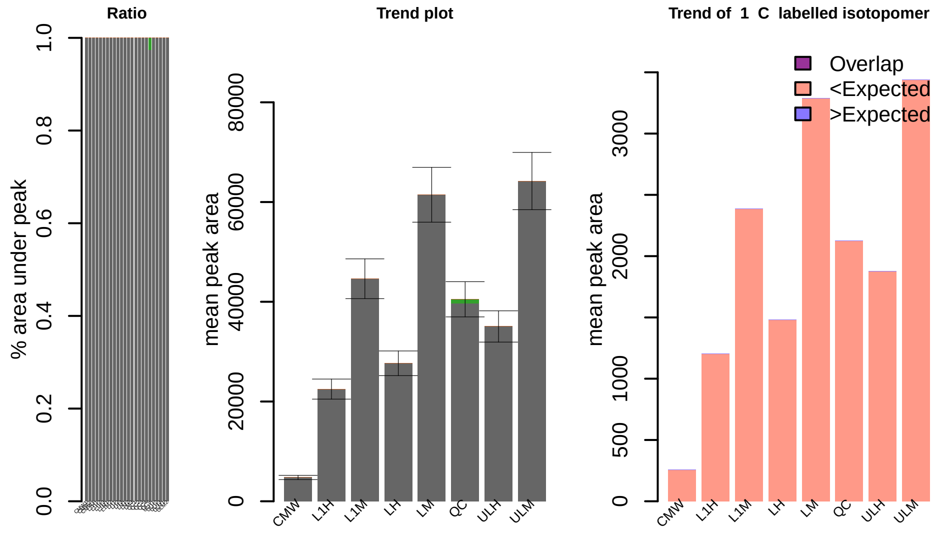

O−Butanoylcarnitine

Formula: C11H21NO4 Mass: 231.147 Std.RT: 530.12354412 Ion: PC

G1

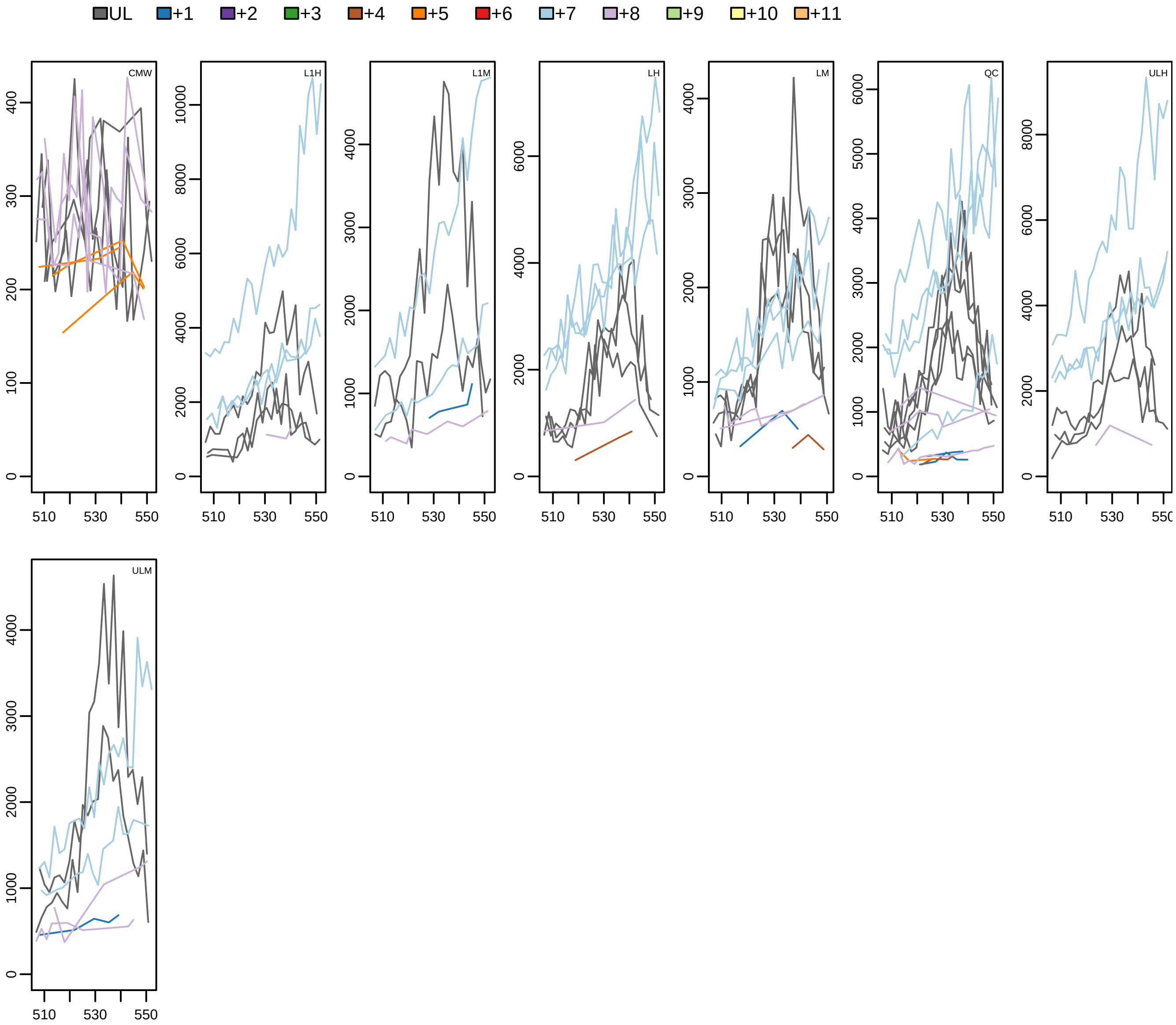

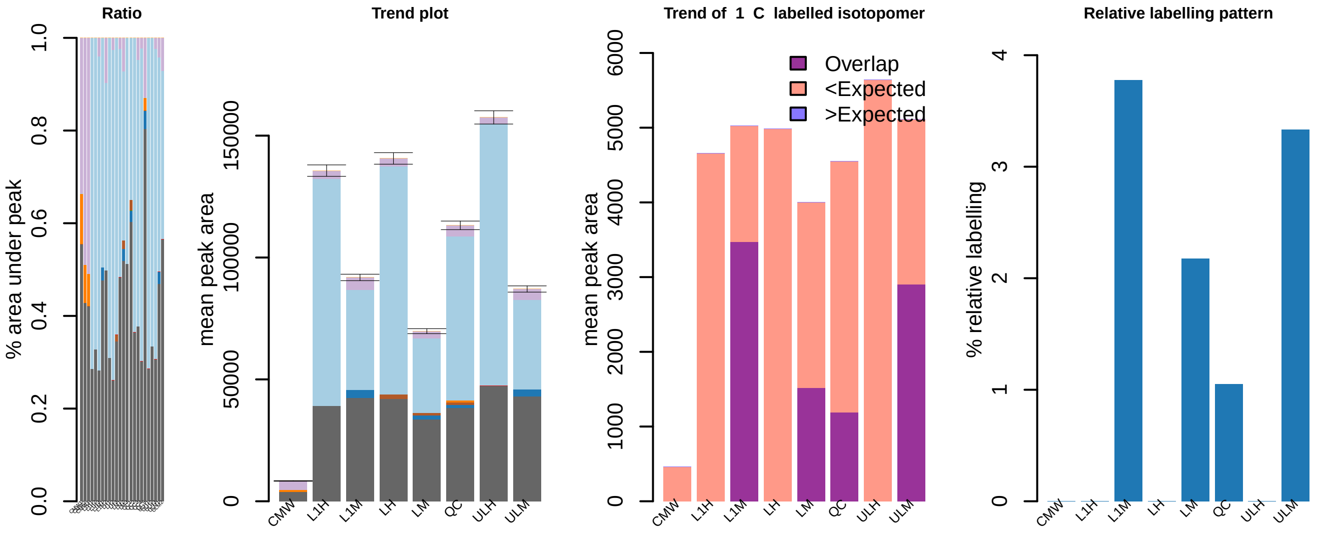

[FA (20:0)] N−(11Z−eicosaenoyl)−ethanolamine

Formula: C22H43NO2 Mass: 353.329 Std.RT: 233.07379092 Ion: PC

G1

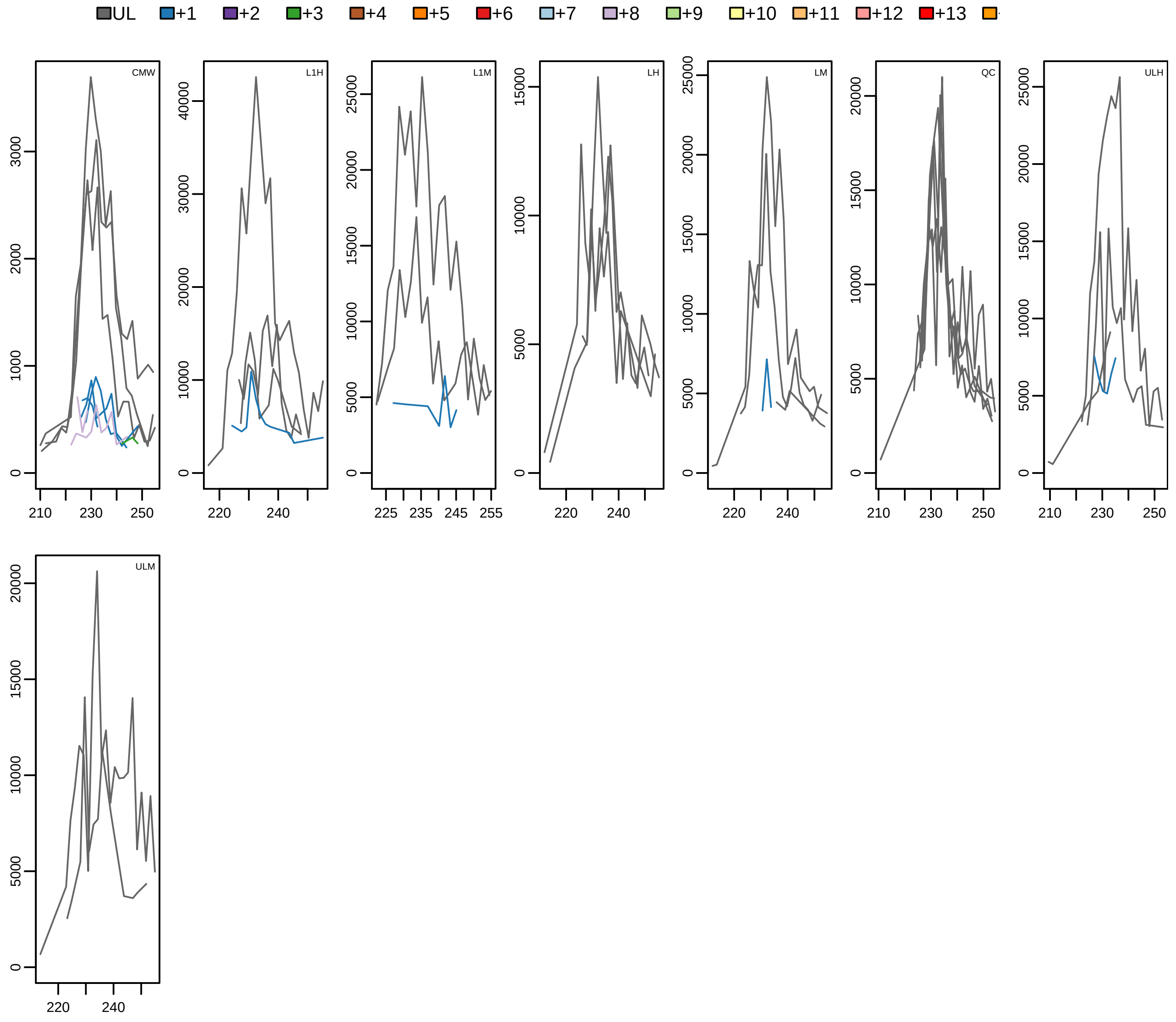

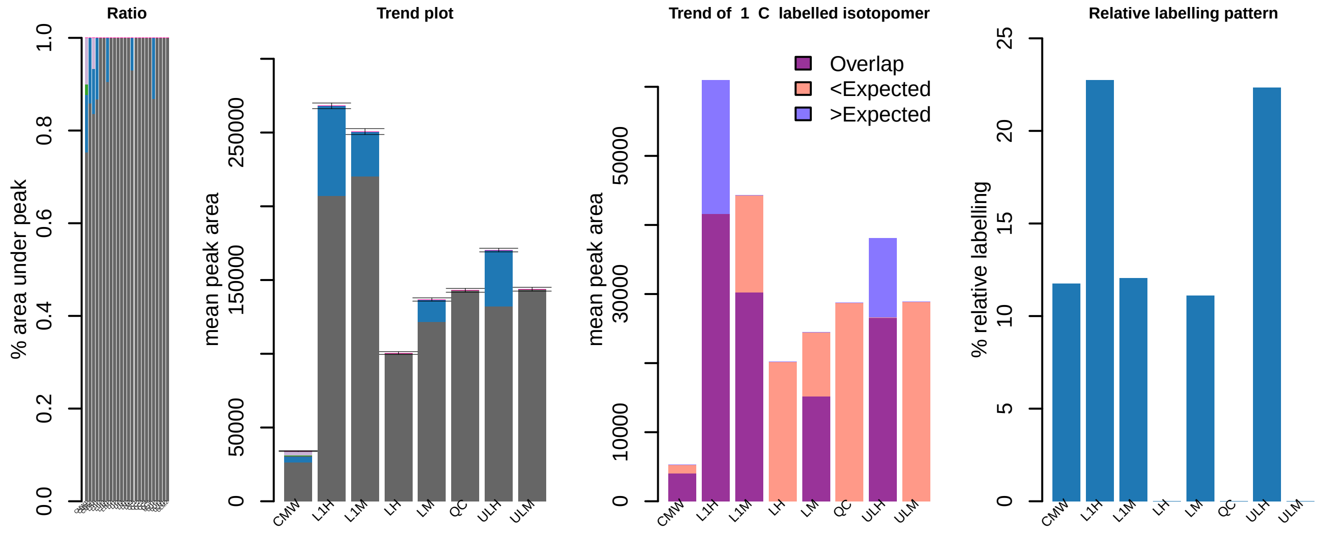

[FA (20:2)] N−(11Z,14Z−eicosadienoyl)−ethanolamine

G1

Formula: C22H41NO2 Mass: 351.314 Std.RT: 232.39837458 Ion: PC

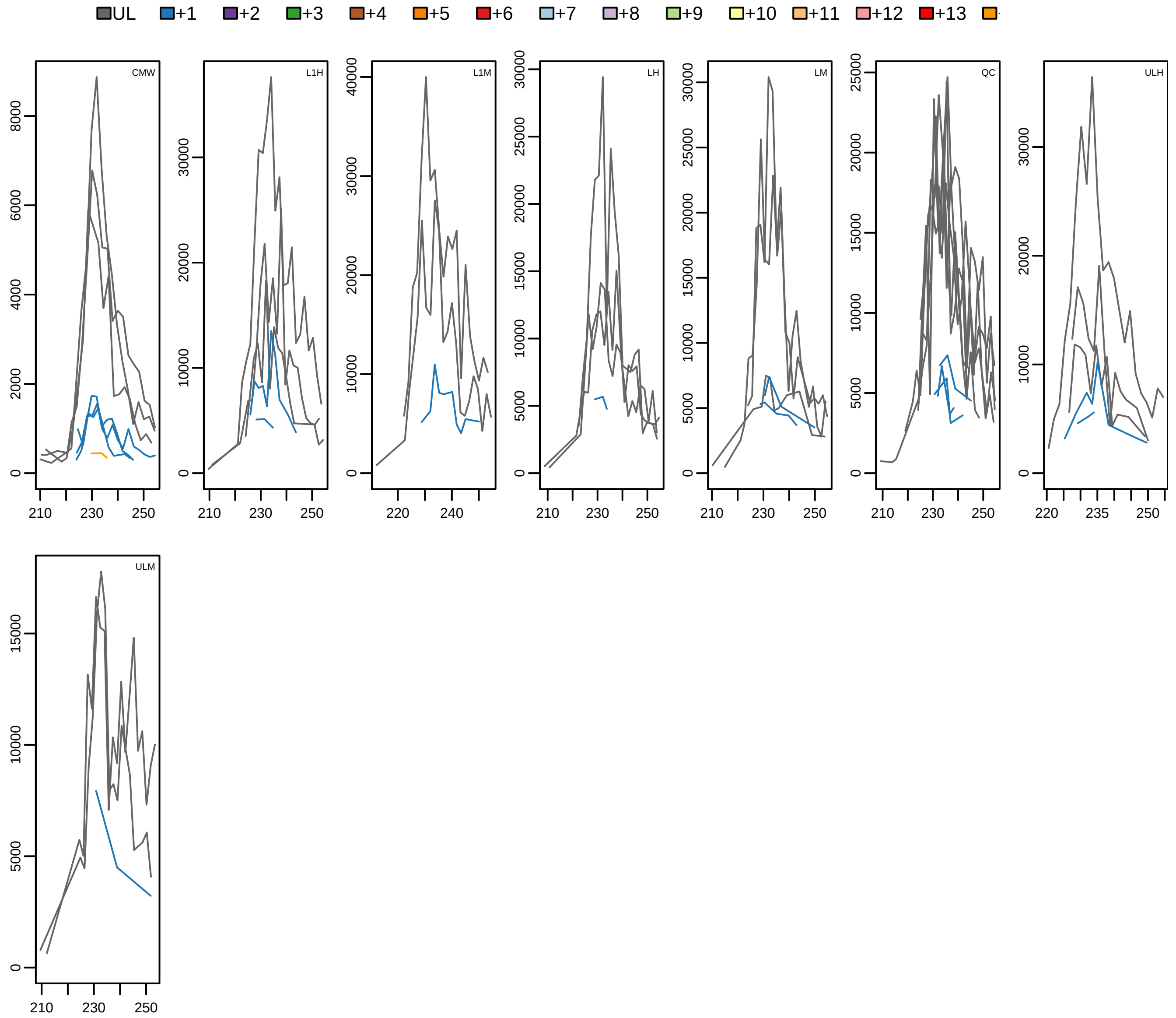

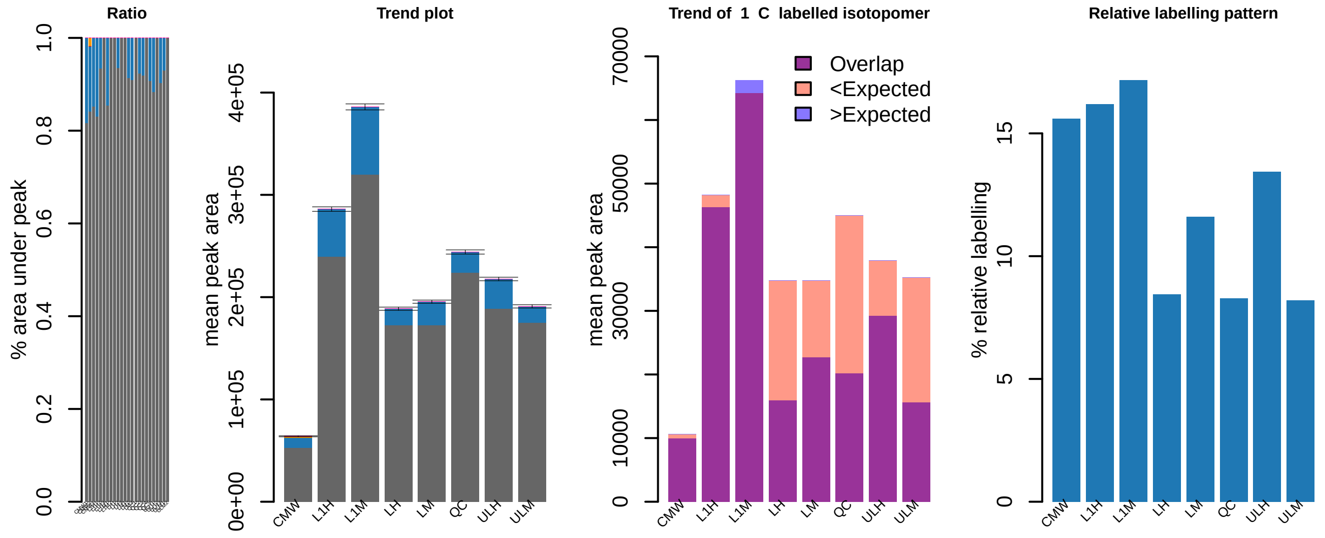

O−Propanoylcarnitine

Formula: C10H19NO4 Mass: 217.131 Std.RT: 617.034327 Ion: POS

G1

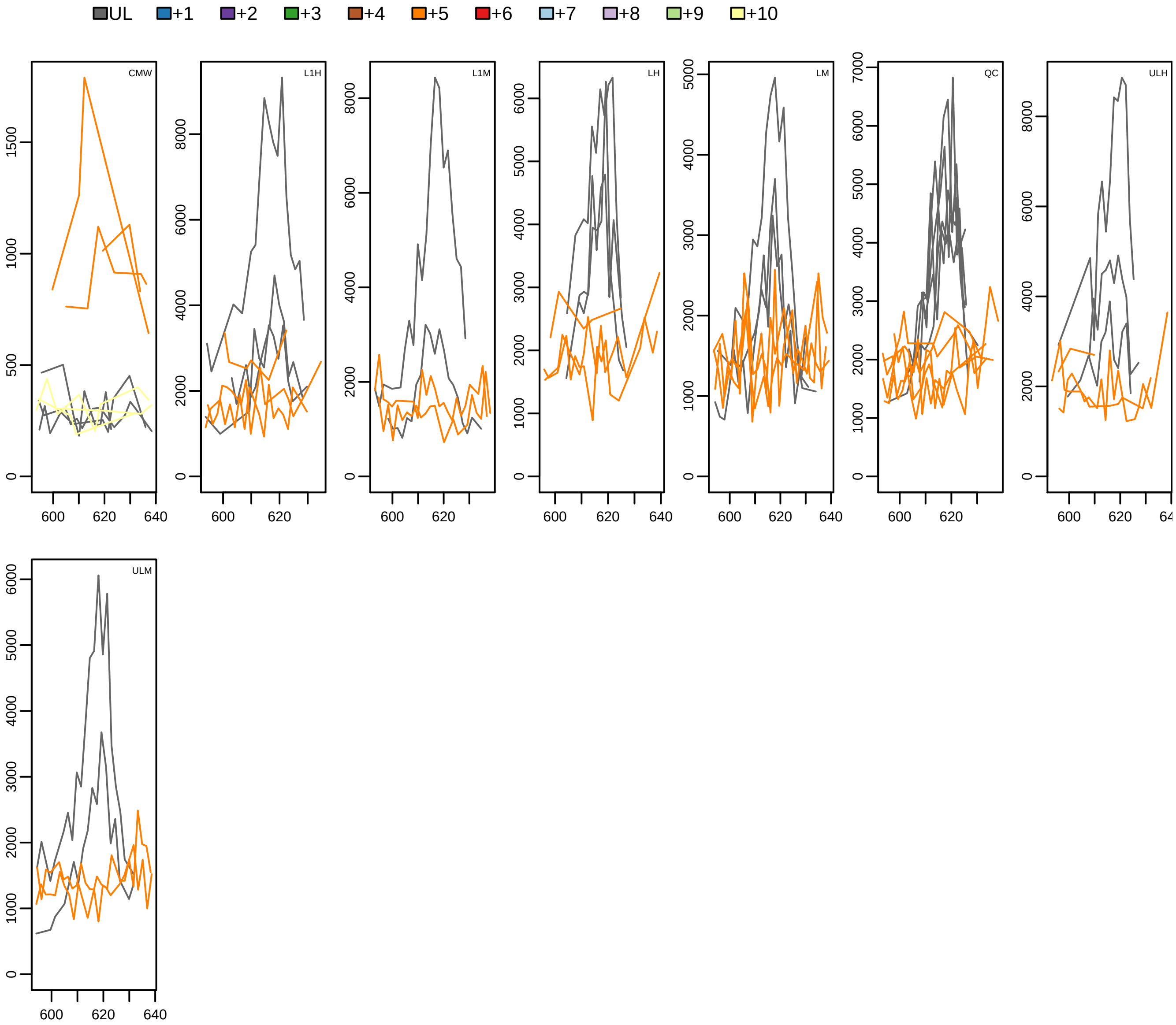

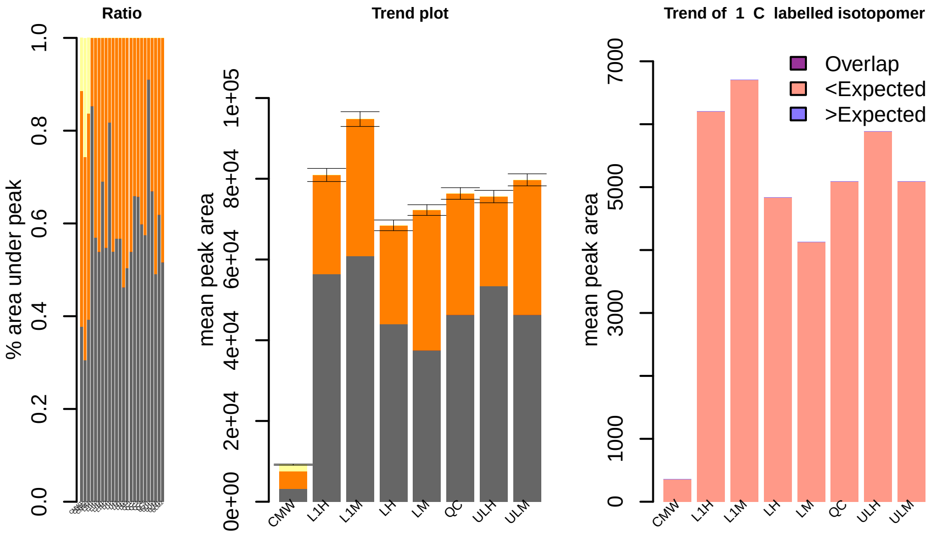

[GL methyl(15:0/8:0)] 1−(14−methyl−pentadecanoyl)−2

Formula: C39H70O4 Mass: 602.527 Std.RT: 208.5582864 Ion: POS

G1

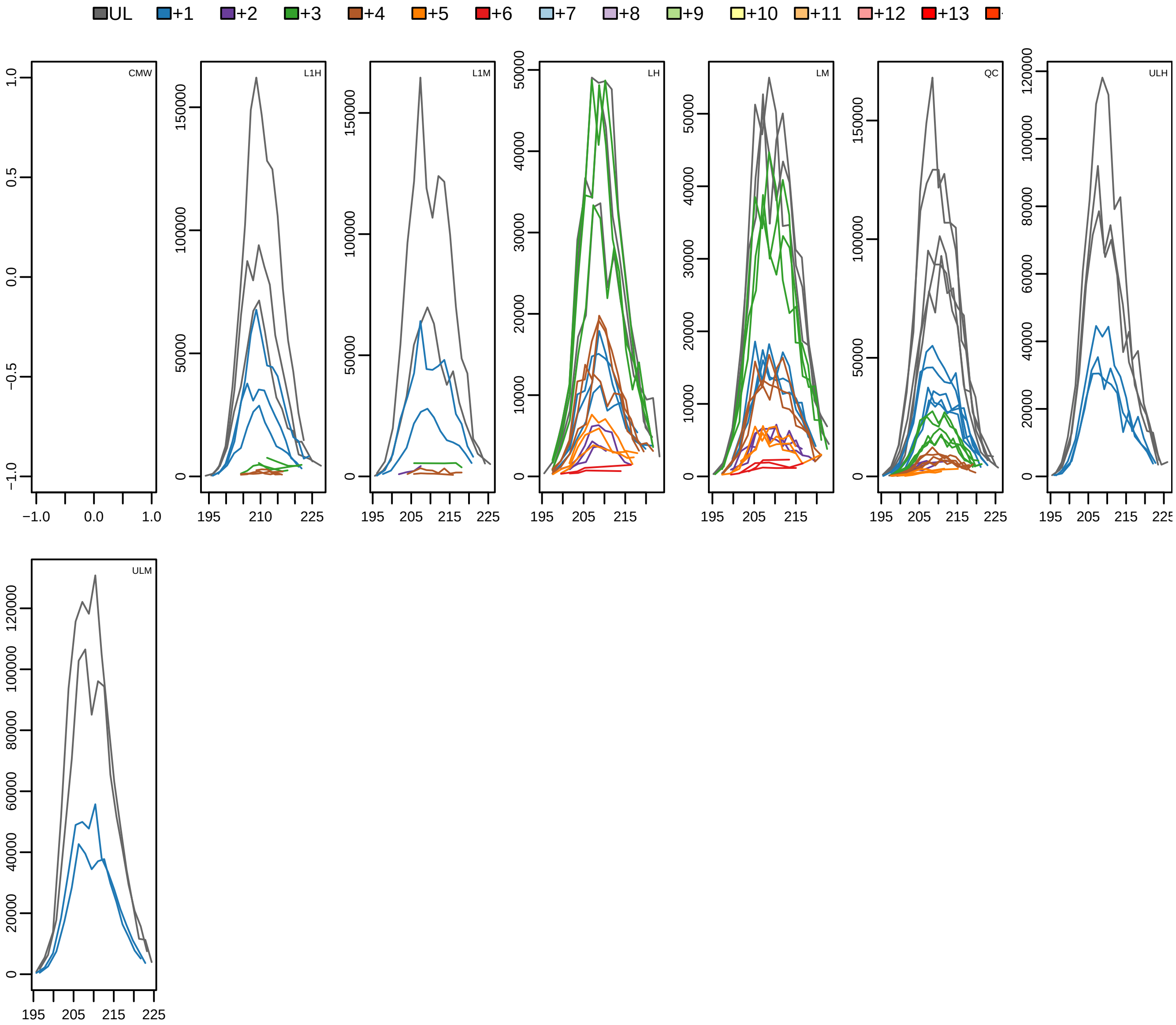

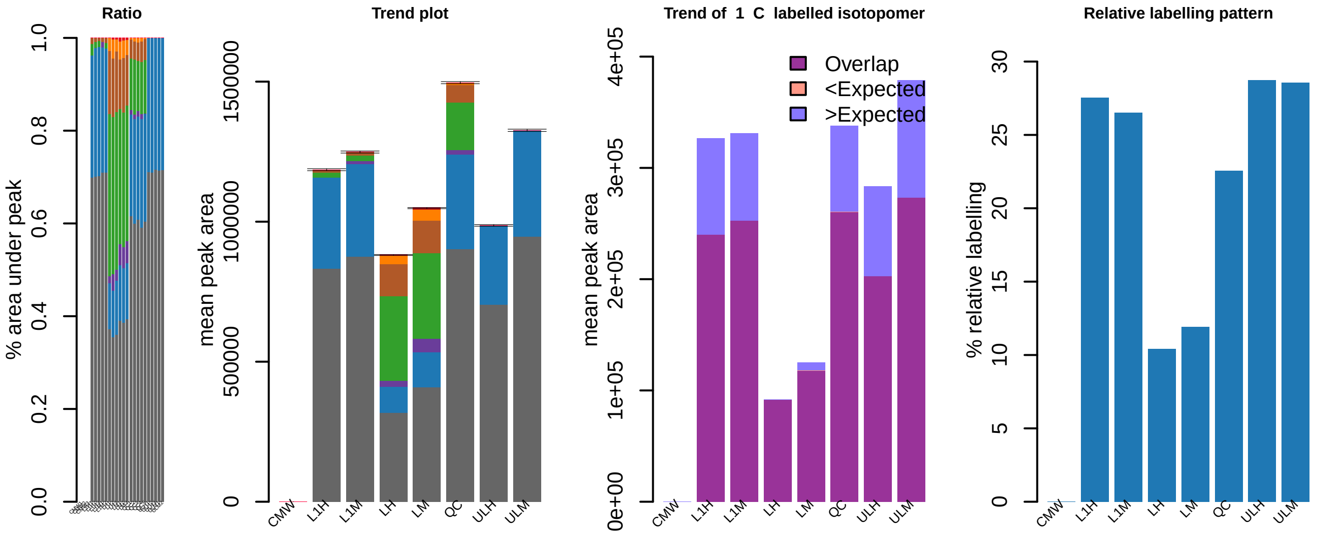

[GL methyl(15:0/8:0)] 1−(14−methyl−pentadecanyl)−2−

Formula: C39H72O3 Mass: 588.548 Std.RT: 208.0051902 Ion: POS

G1

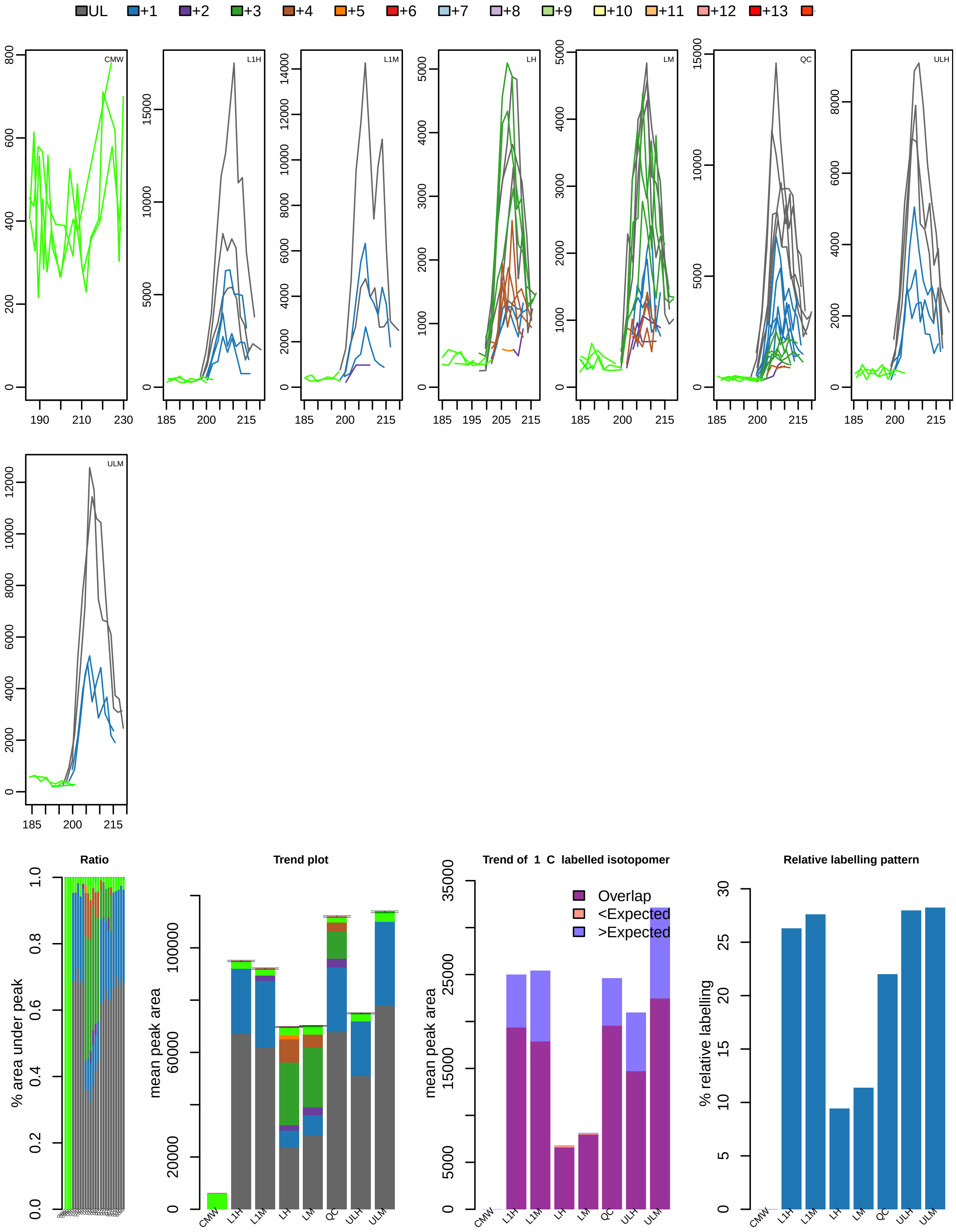

[GL (8:0/8:0)] 1−(8−[5]−ladderane−octanoyl)−2−(8−[3]−

Formula: C43H68O4 Mass: 648.512 Std.RT: 207.73733382 Ion: POS

G1

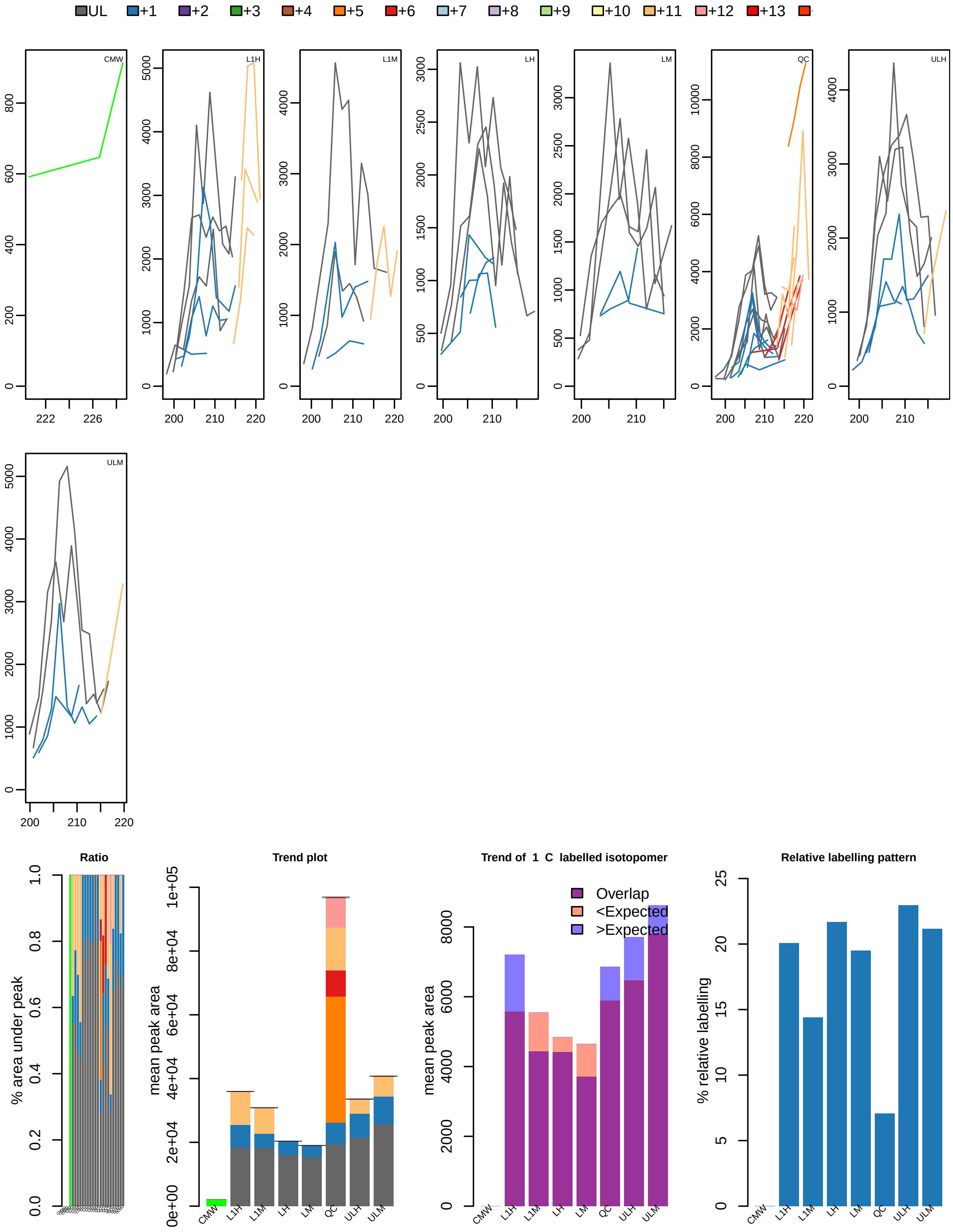

[GL (8:0/8:0)] 1−(8−[3]−ladderane−octanoyl−2−(8−[3]−l

Formula: C43H70O4 Mass: 650.527 Std.RT: 208.31071398 Ion: POS

G1

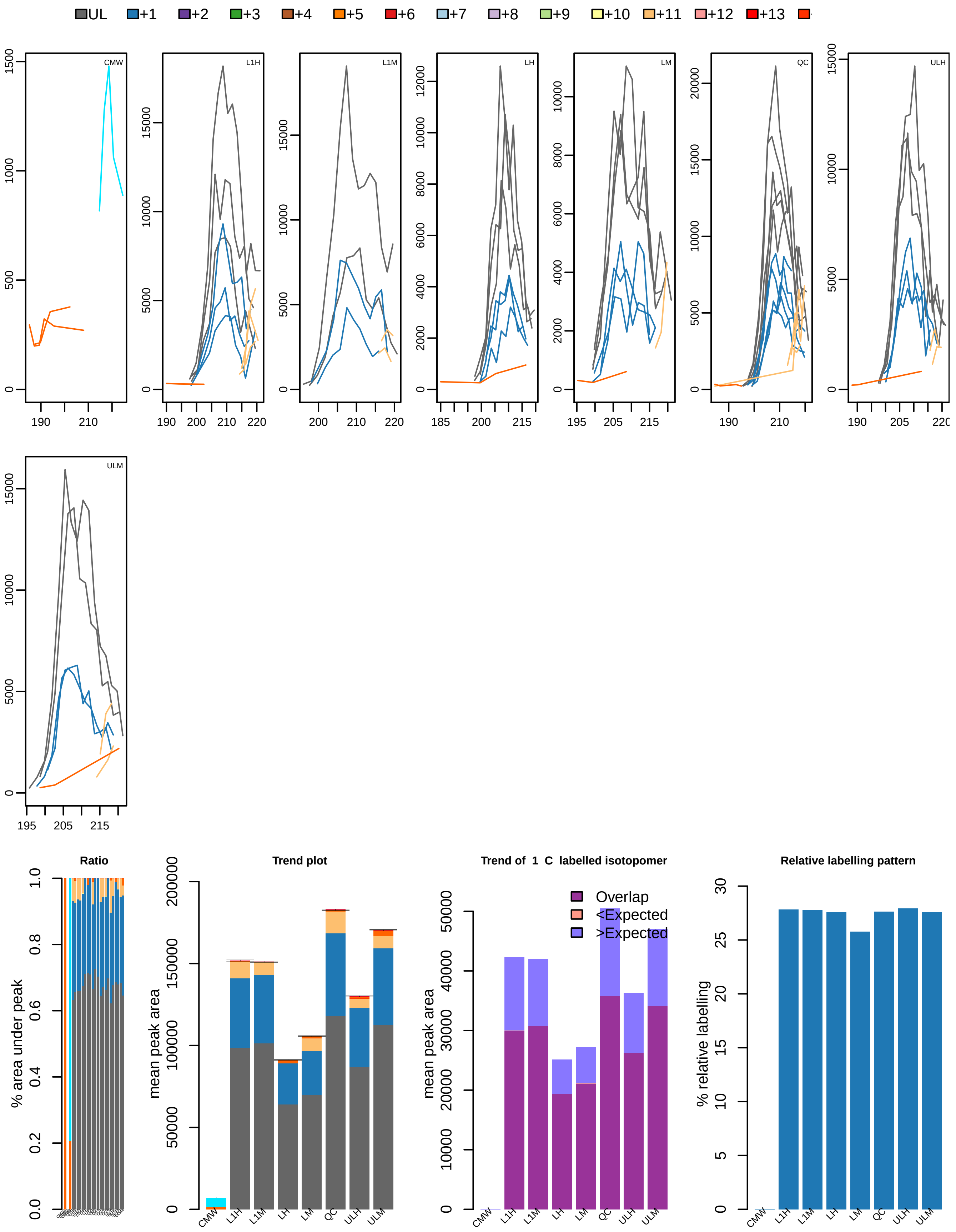

[GL (22:1/22:2)] 1−(13Z−docosenoyl)−2−(13Z,16Z−doc

G1

Formula: C47H86O5 Mass: 730.648 Std.RT: 418.95914418 Ion: POS

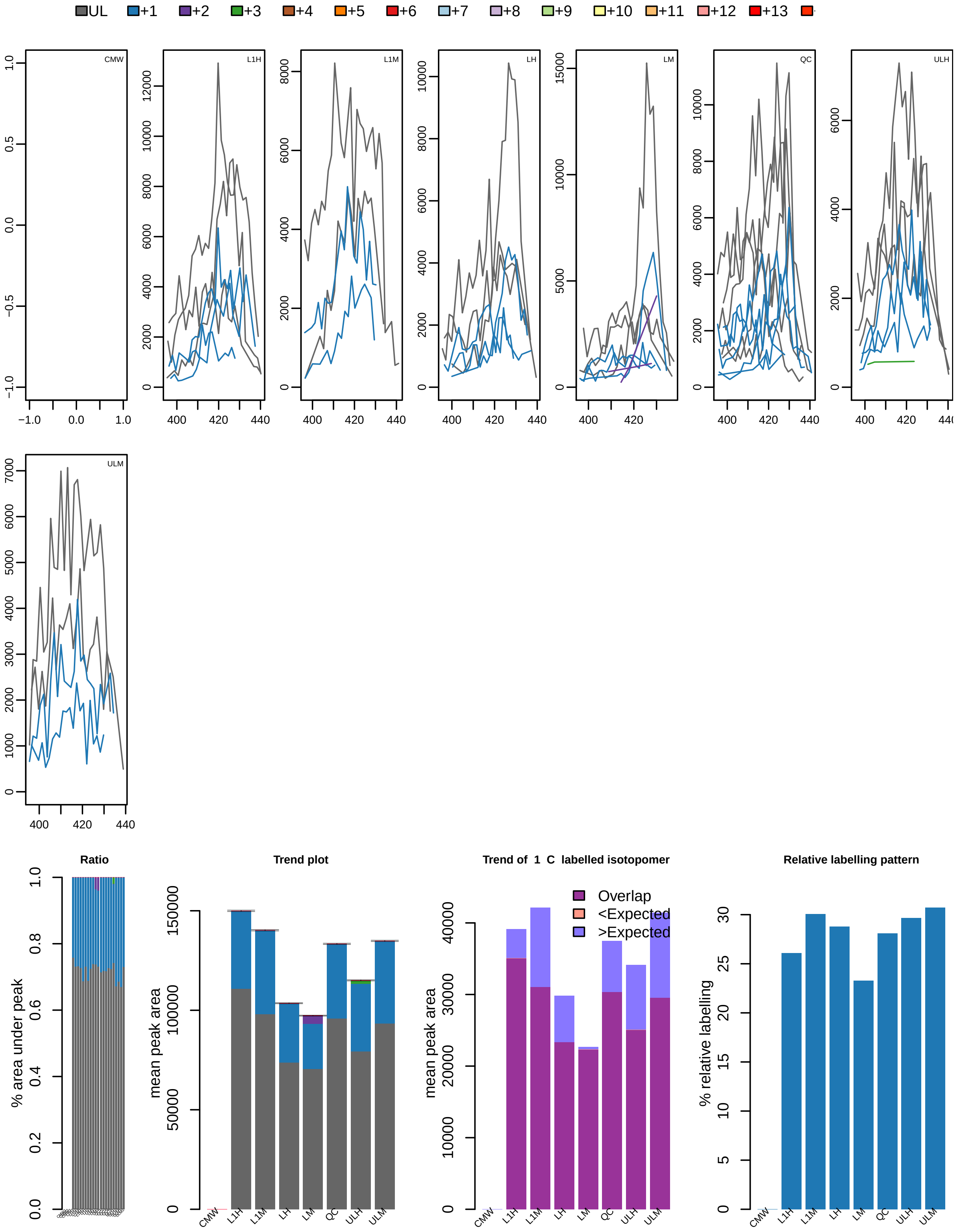

[GL (21:0/22:3)] 1−heneicosanoyl−2−(10Z,13Z,16Z−do

Formula: C46H84O5 Mass: 716.632 Std.RT: 421.32152298 Ion: POS

G1

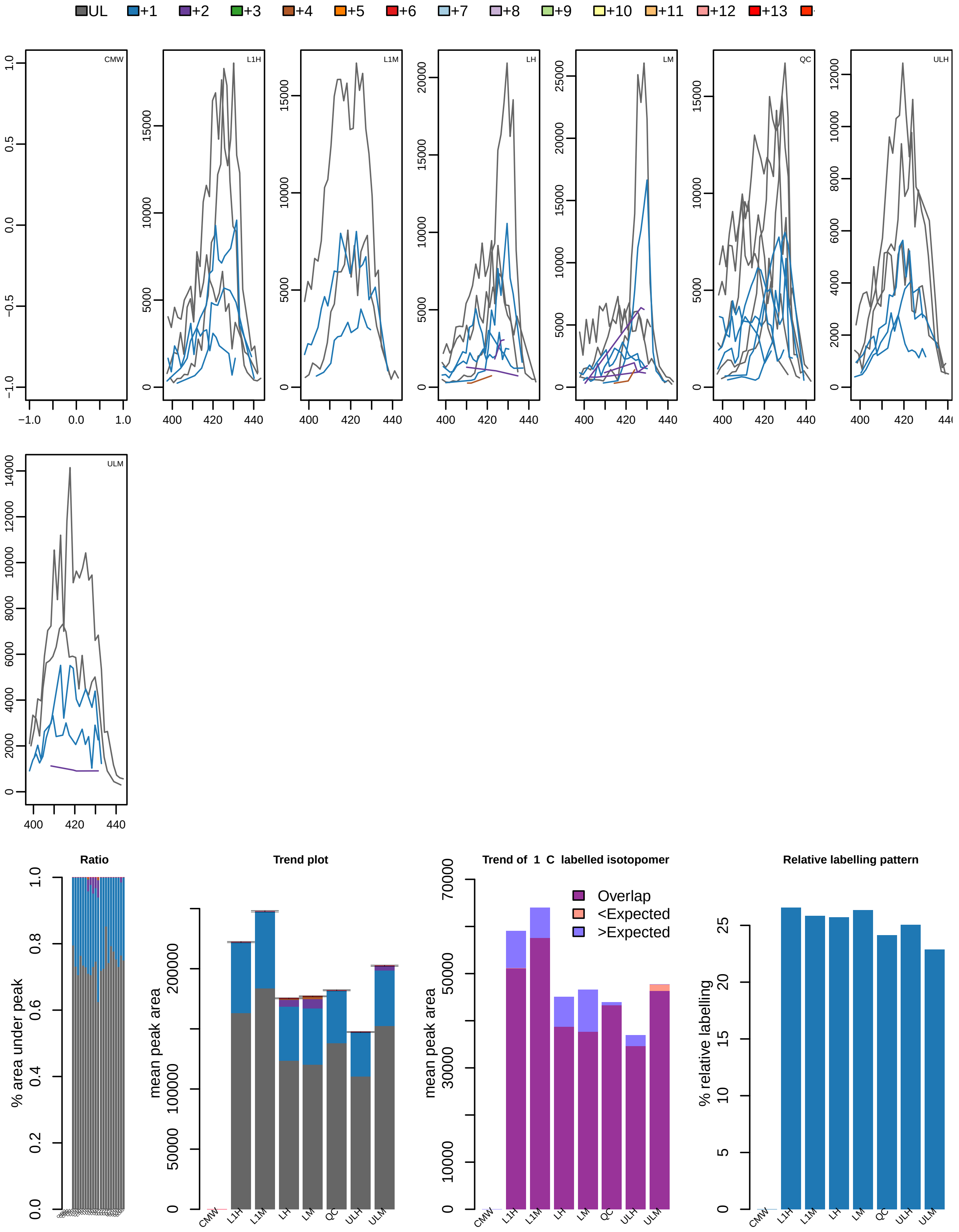

PC(14:0/14:1(9Z))

Formula: C36H70NO8P Mass: 675.484 Std.RT: 234.71611188 Ion: P

G1

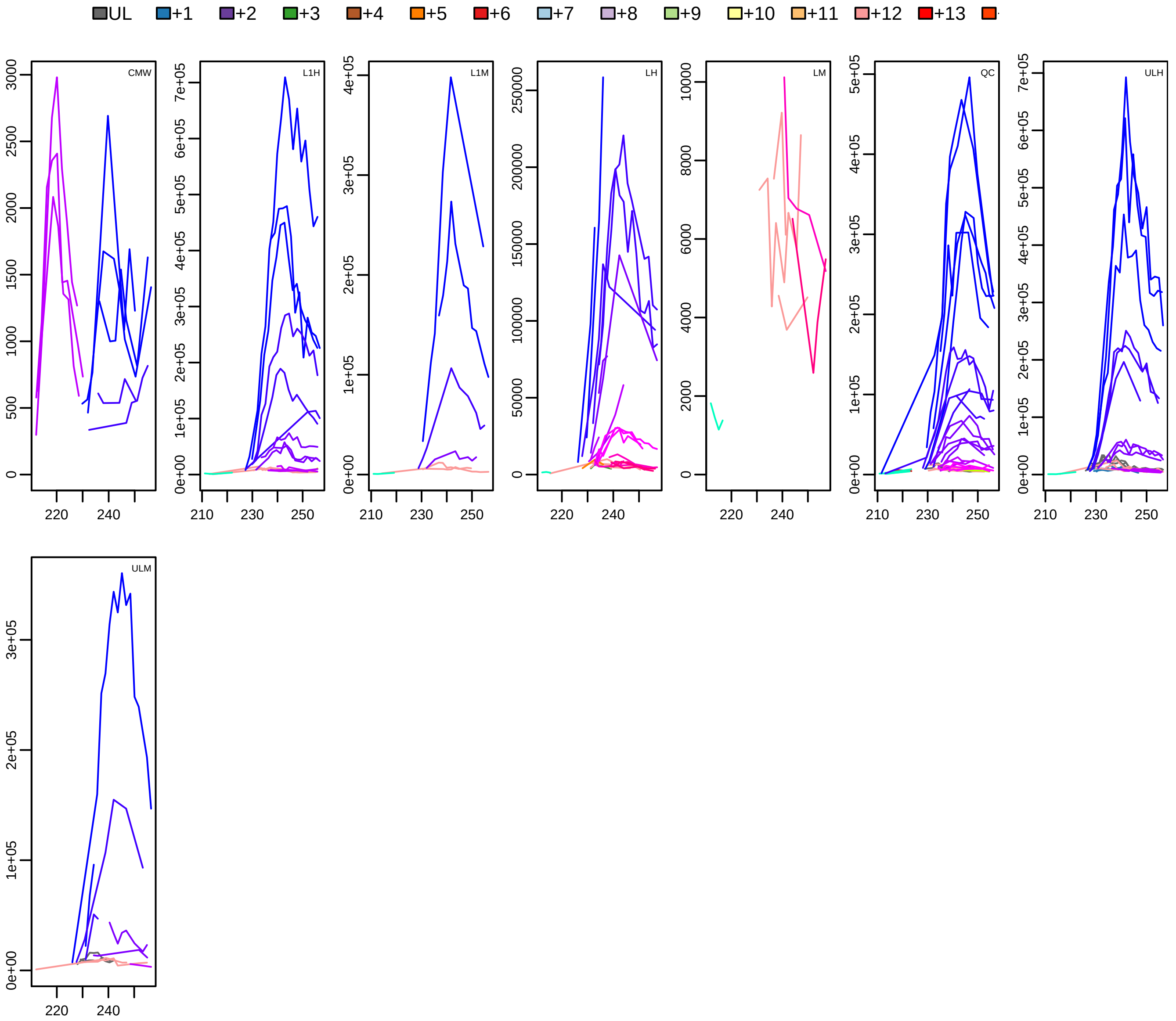

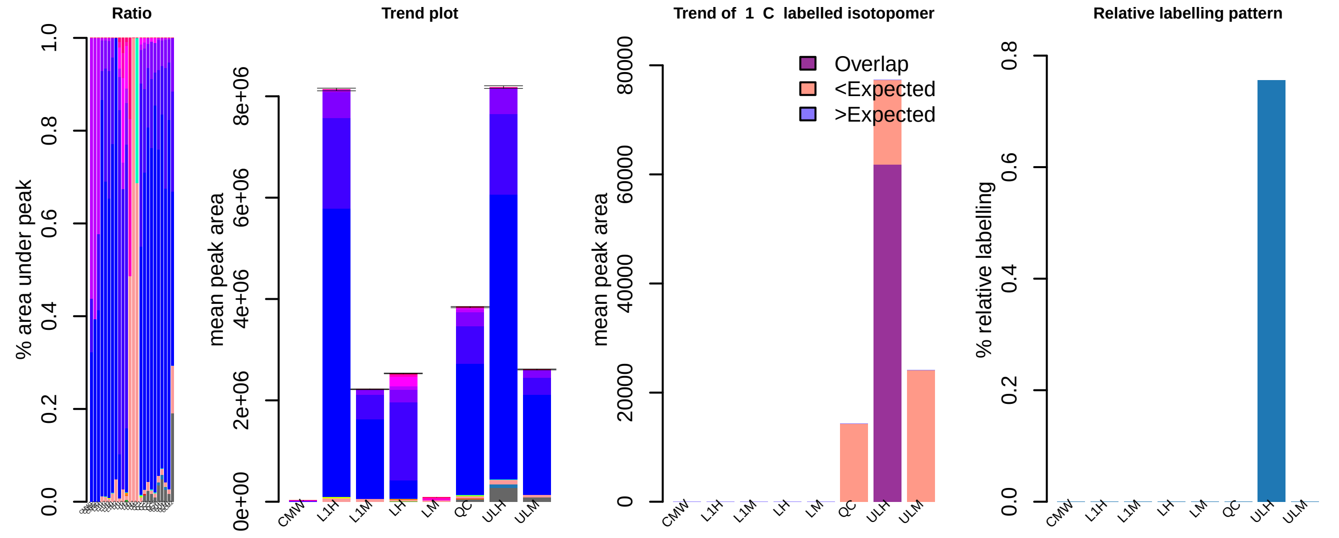

[PC (14:0/18:1)] 1−tetradecanoyl−2−(11Z−octadecenoy

Formula: C40H78NO8P Mass: 731.547 Std.RT: 237.99899952 Ion: P

G1

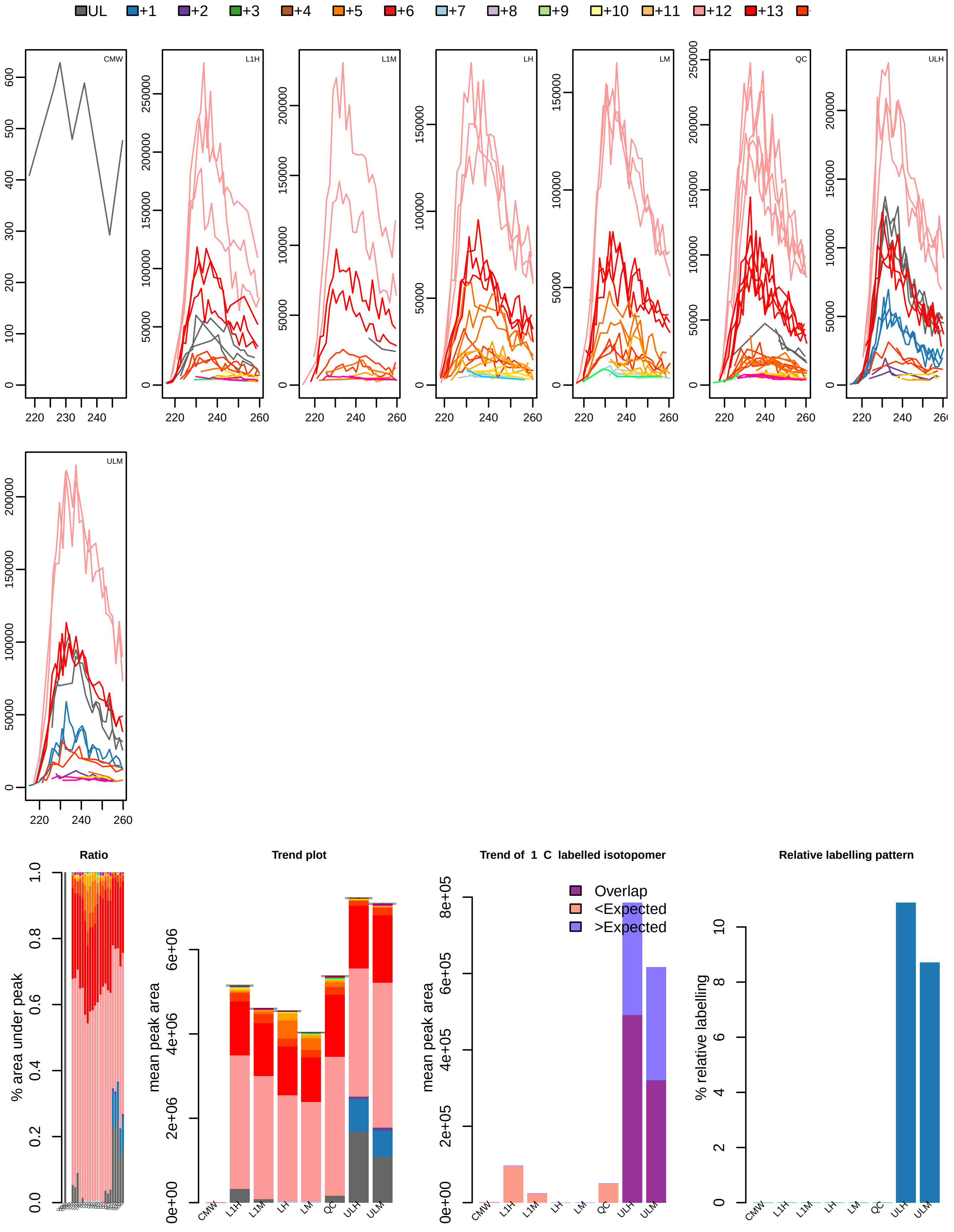

[PC (16:0/16:0)] 1−hexadecanoyl−2−hexadecanoyl−sn-

Formula: C40H80NO8P Mass: 733.562 Std.RT: 272.034201 Ion: POS

G1

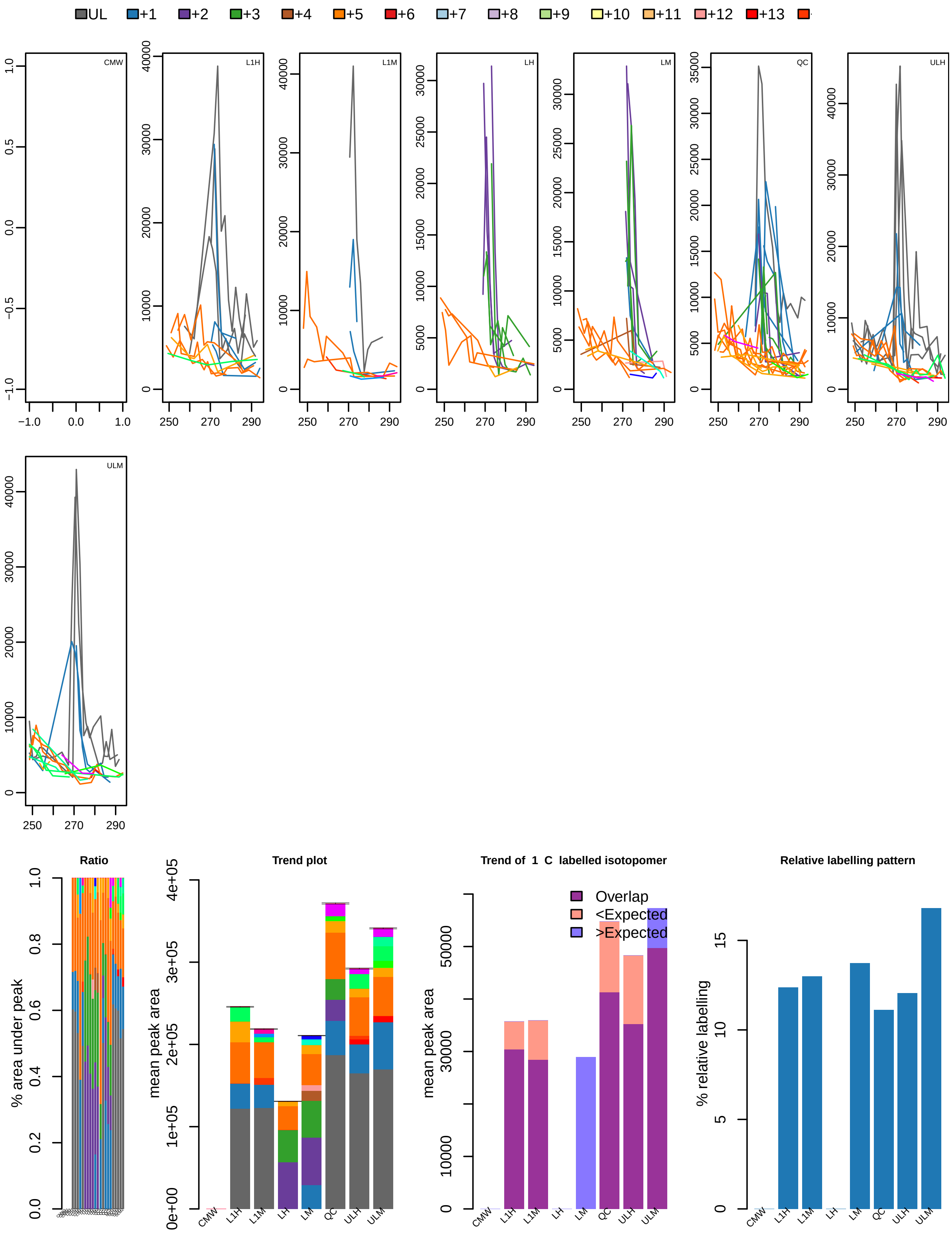

[PC (14:0/18:2)] 1−tetradecanoyl−2−(9Z,12Z−octadeca

Formula: C40H76NO8P Mass: 729.531 Std.RT: 232.86533322 Ion: P

G1

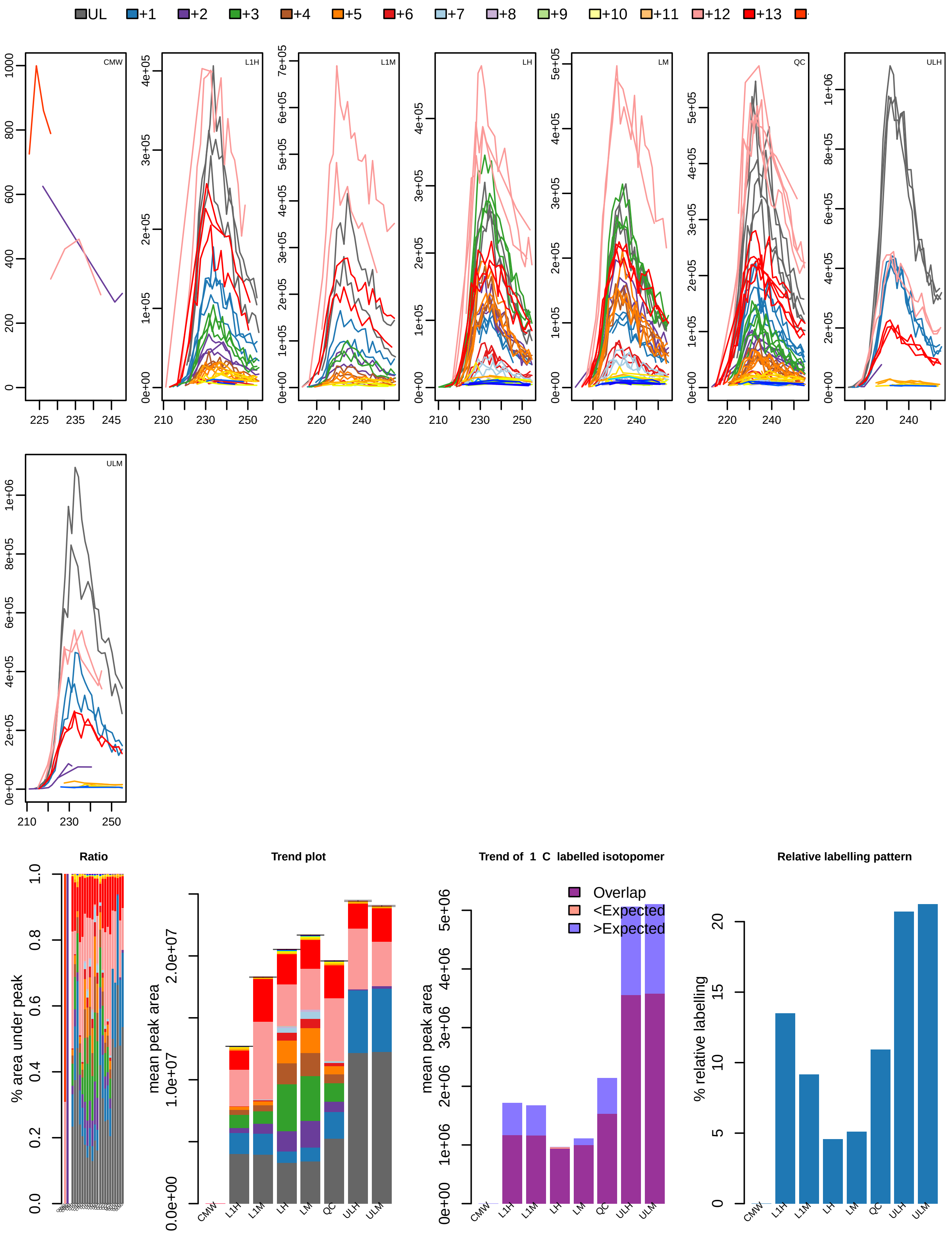

PC(14:1(9Z)/16:1(9Z))

Formula: C38H72NO8P Mass: 701.5 Std.RT: 233.85728532 Ion: PO[illegible]

G1

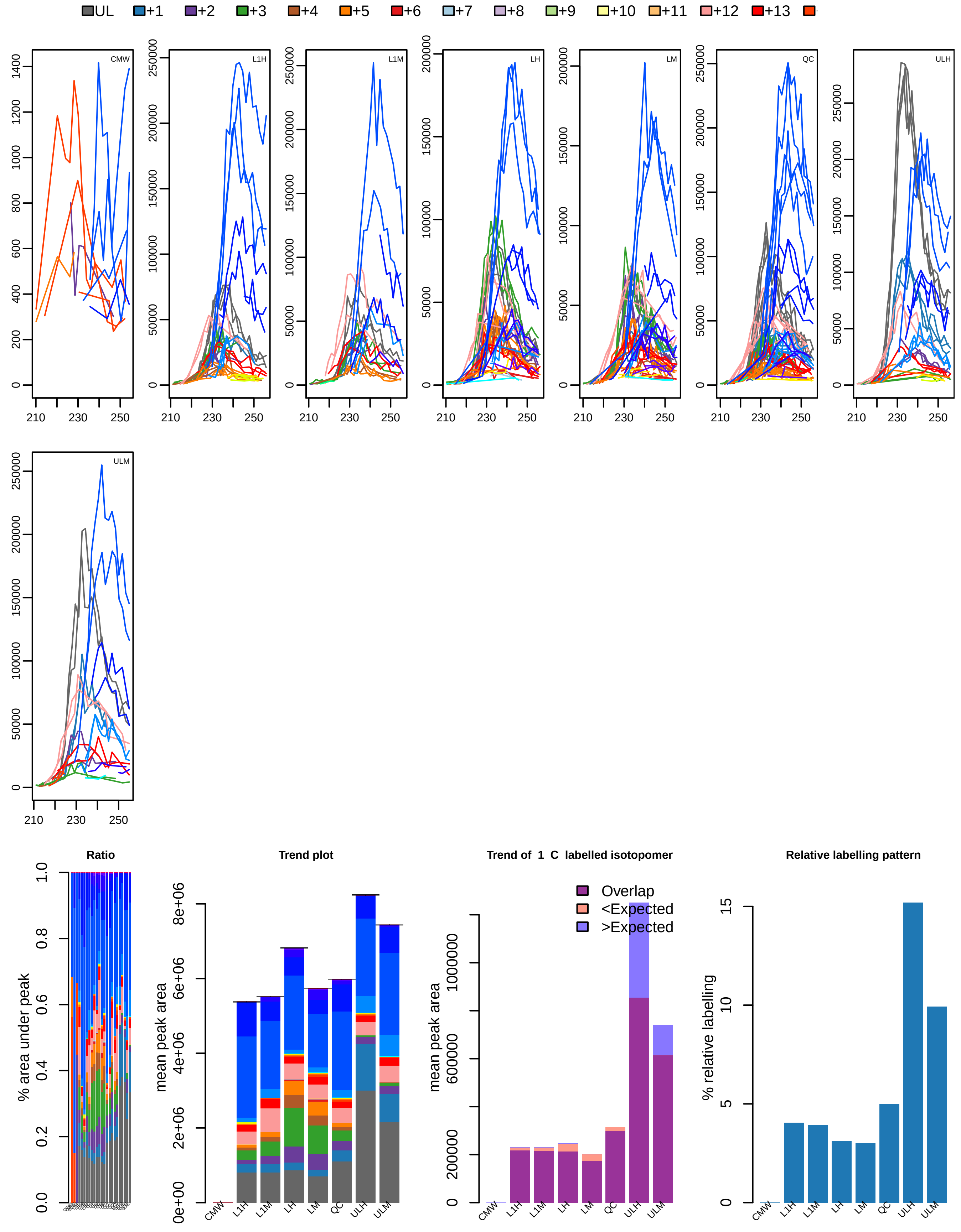

[PC methyl(14:2)] 2−methyl−3−tetradecyl−sn−glycero−[illegible]

Formula: C23H50NO6P Mass: 467.338 Std.RT: 258.005778 Ion: PO[illegible]

G1

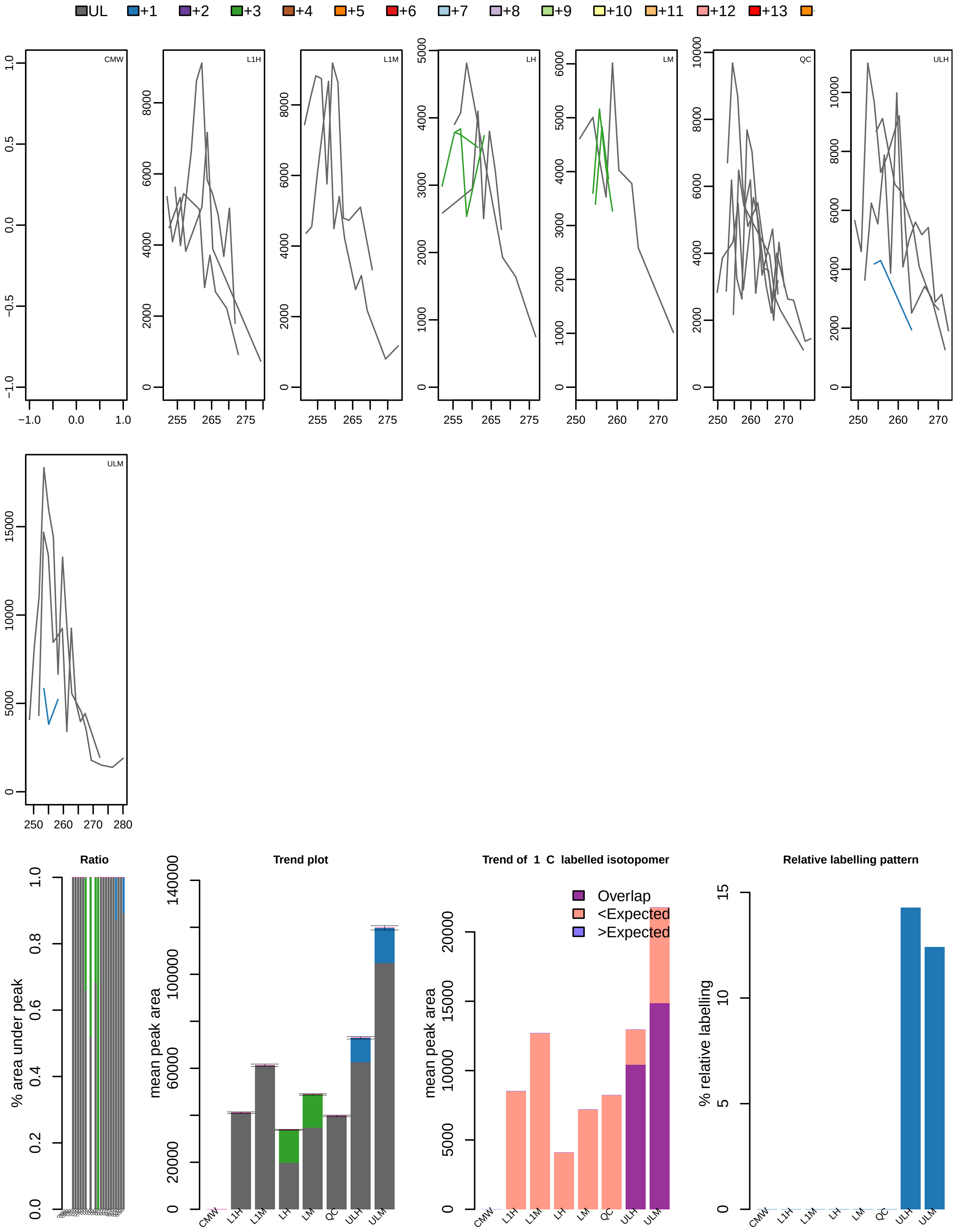

[PC (14:0)] 1−tetradecanoyl−sn−glycero−3−phosphoch

Formula: C22H46NO7P Mass: 467.301 Std.RT: 266.5785246 Ion: PC

G1

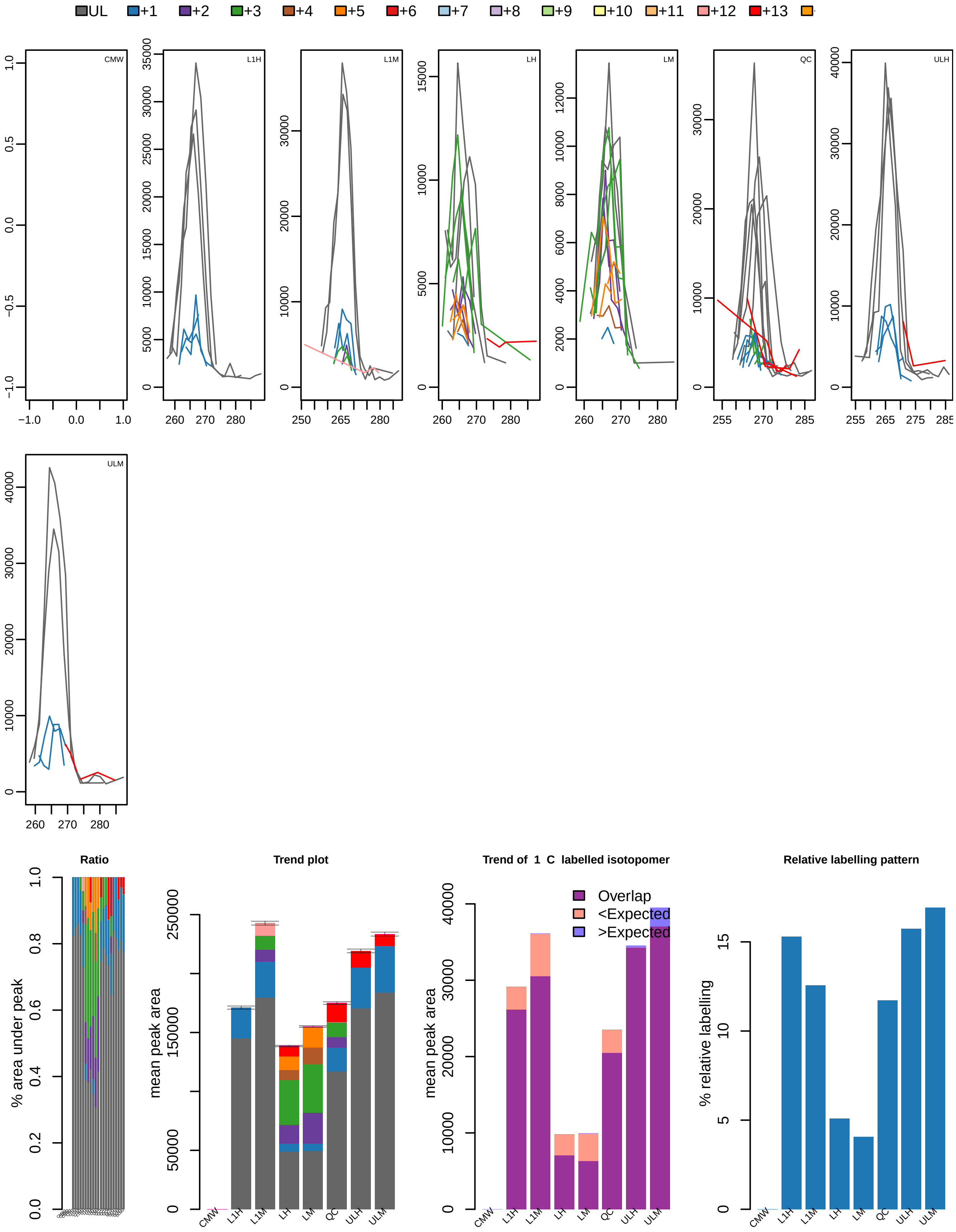

[PC (14:1/14:1)] 1,2−di−(9Z−tetradecenoyl)−sn−glycerc

Formula: C36H68NO8P Mass: 673.468 Std.RT: 235.43837988 Ion: P

G1

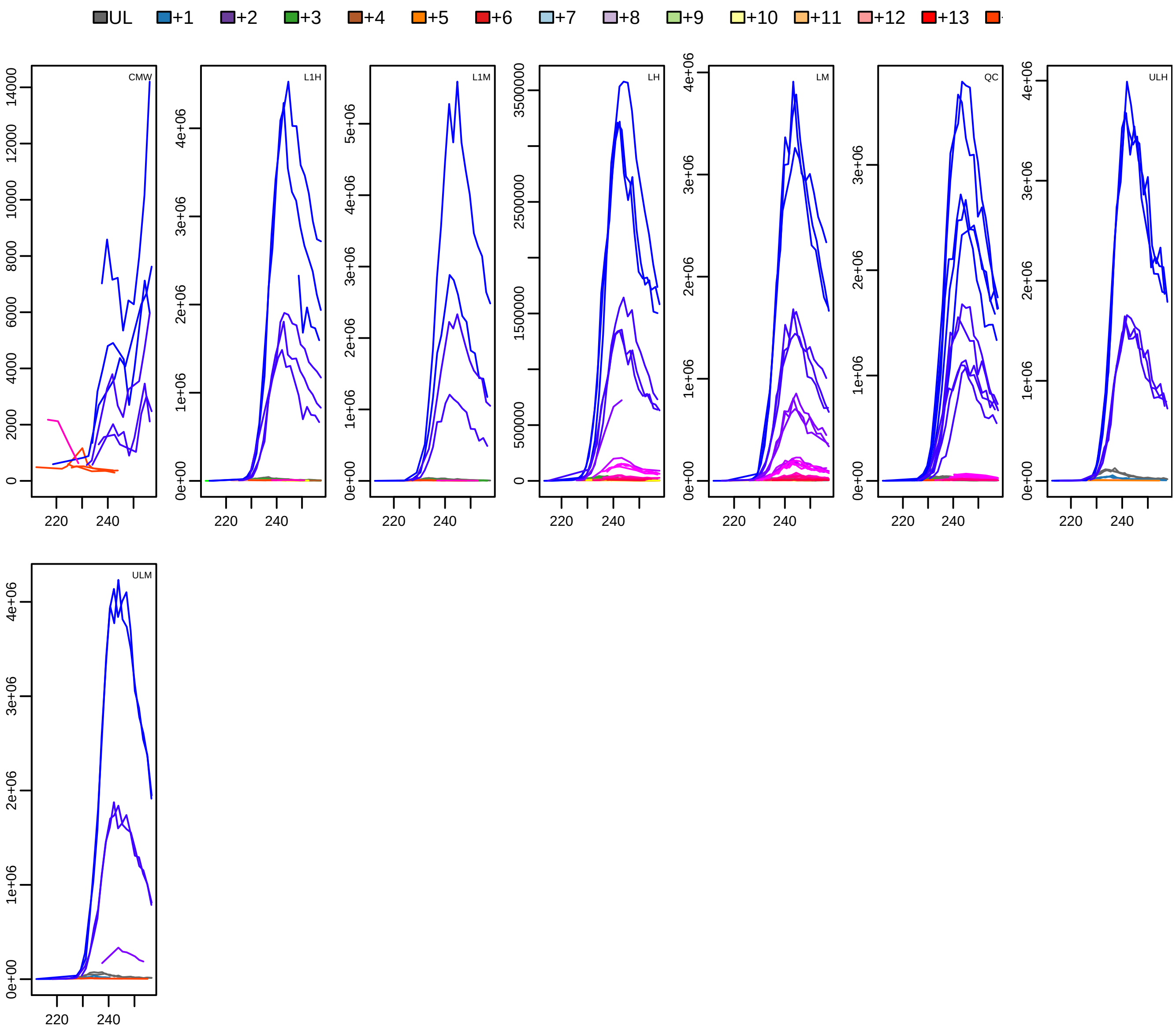

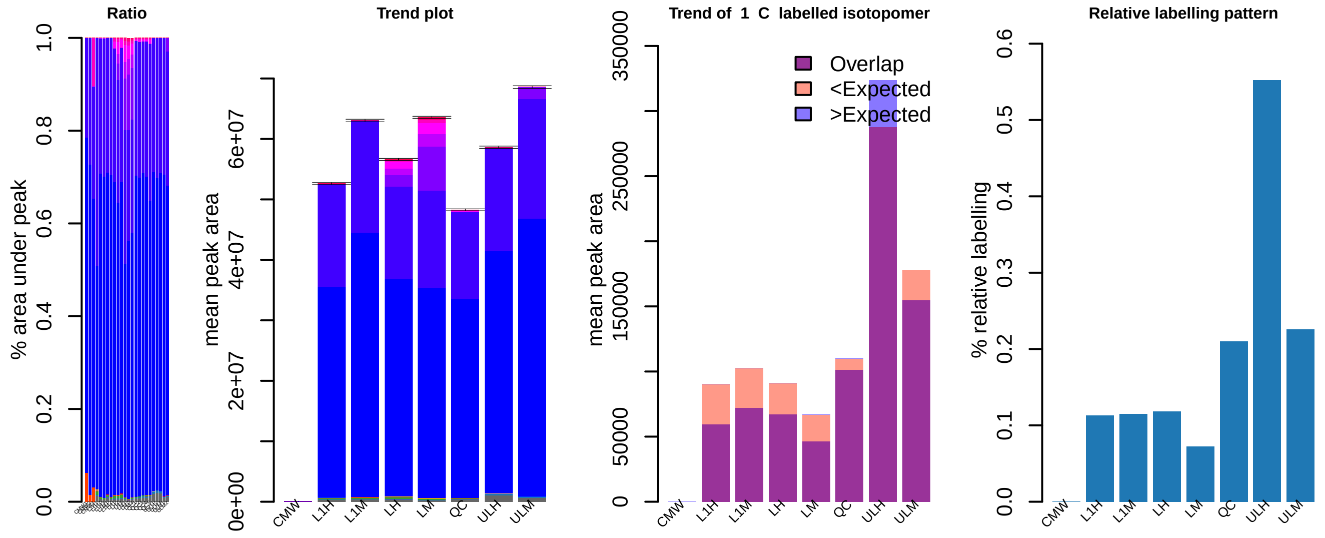

[PC (14:0/18:3)] 1−tetradecanoyl−2−(9Z,12Z,15Z−octa[illegible]

Formula: C40H74NO8P Mass: 727.515 Std.RT: 232.6464771 Ion: PC[illegible]

G1

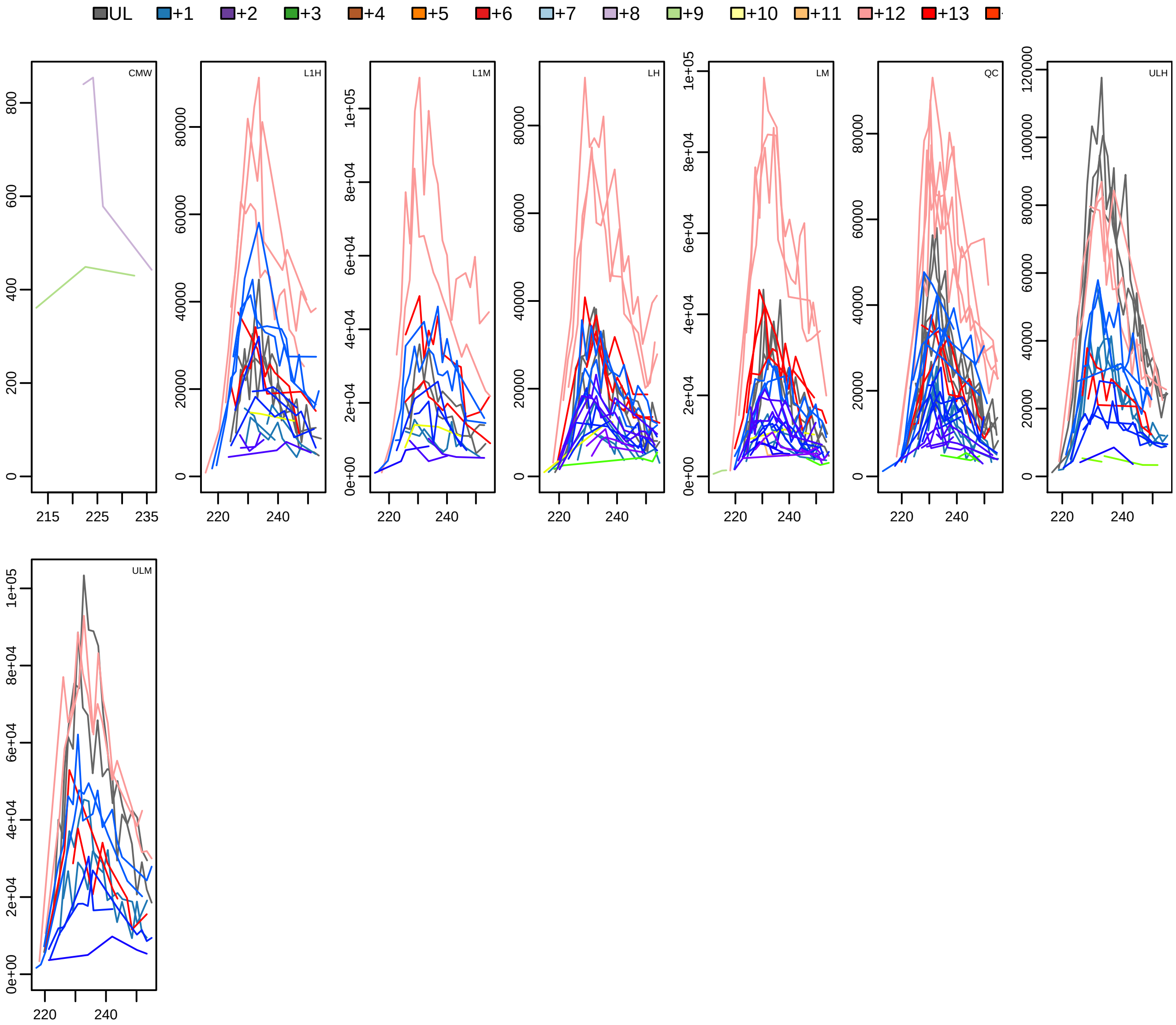

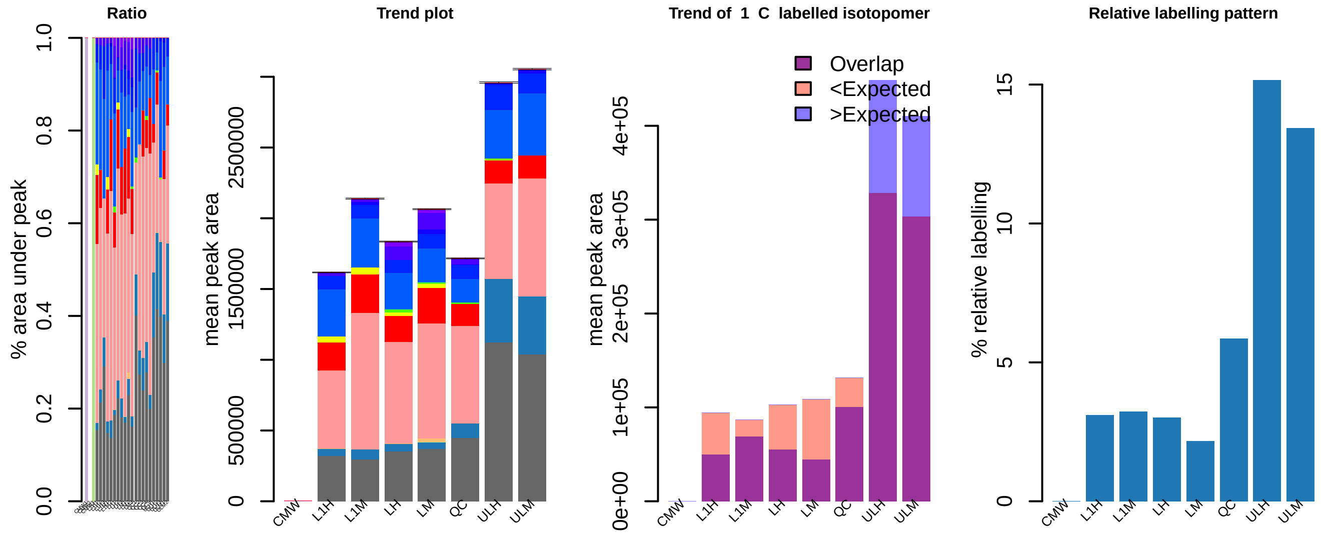

[PC (18:1/18:1)] 1−(9Z−octadecenoyl)−2−(9Z−octadece

Formula: C44H84NO8P Mass: 785.593 Std.RT: 234.1080831 Ion: PC

G1

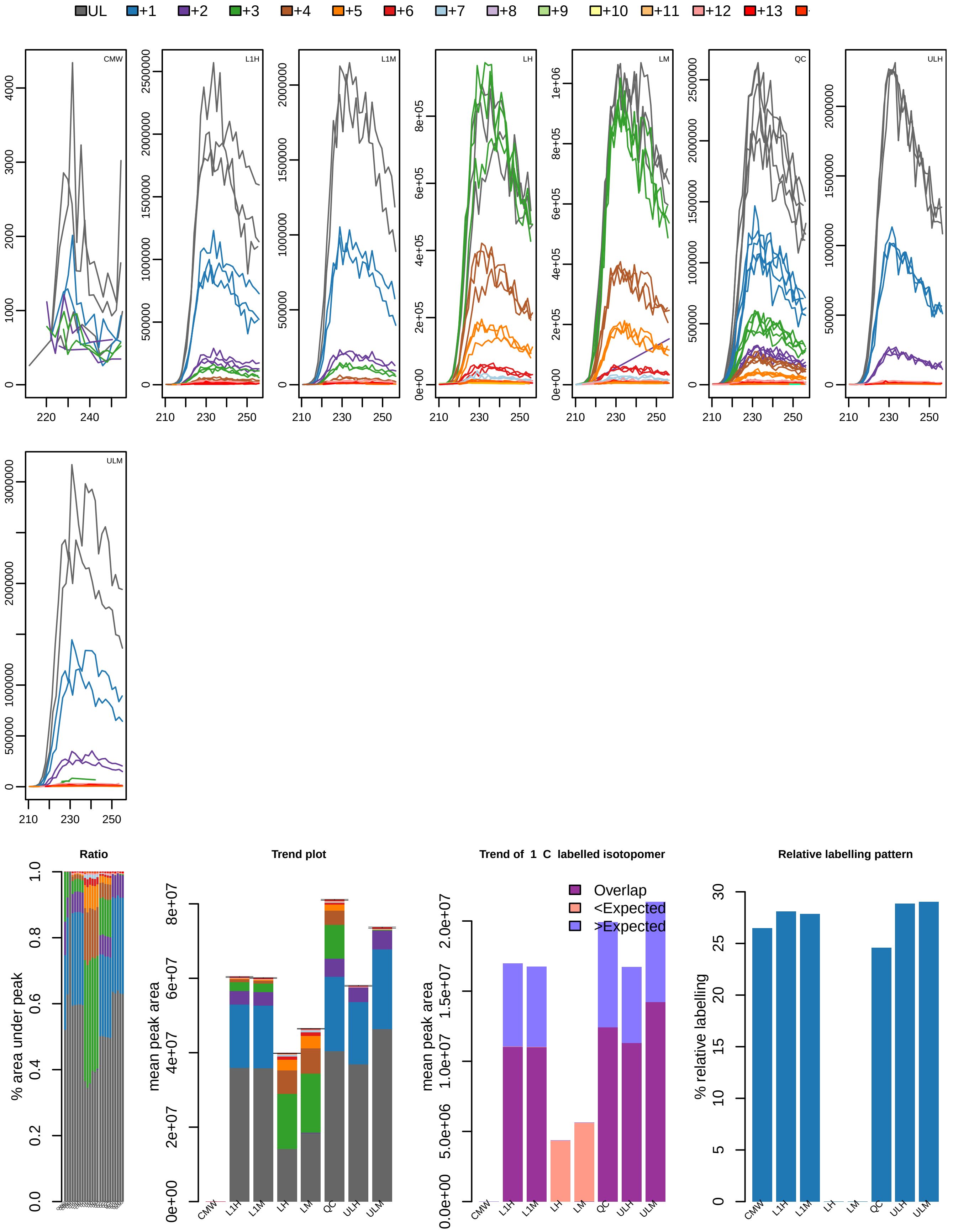

[PC (18:1/18:1)] 1−(9Z−octadecenoyl)−2−(9Z−octadece

Formula: C44H84NO8P Mass: 785.593 Std.RT: 234.1080831 Ion: PC

G2

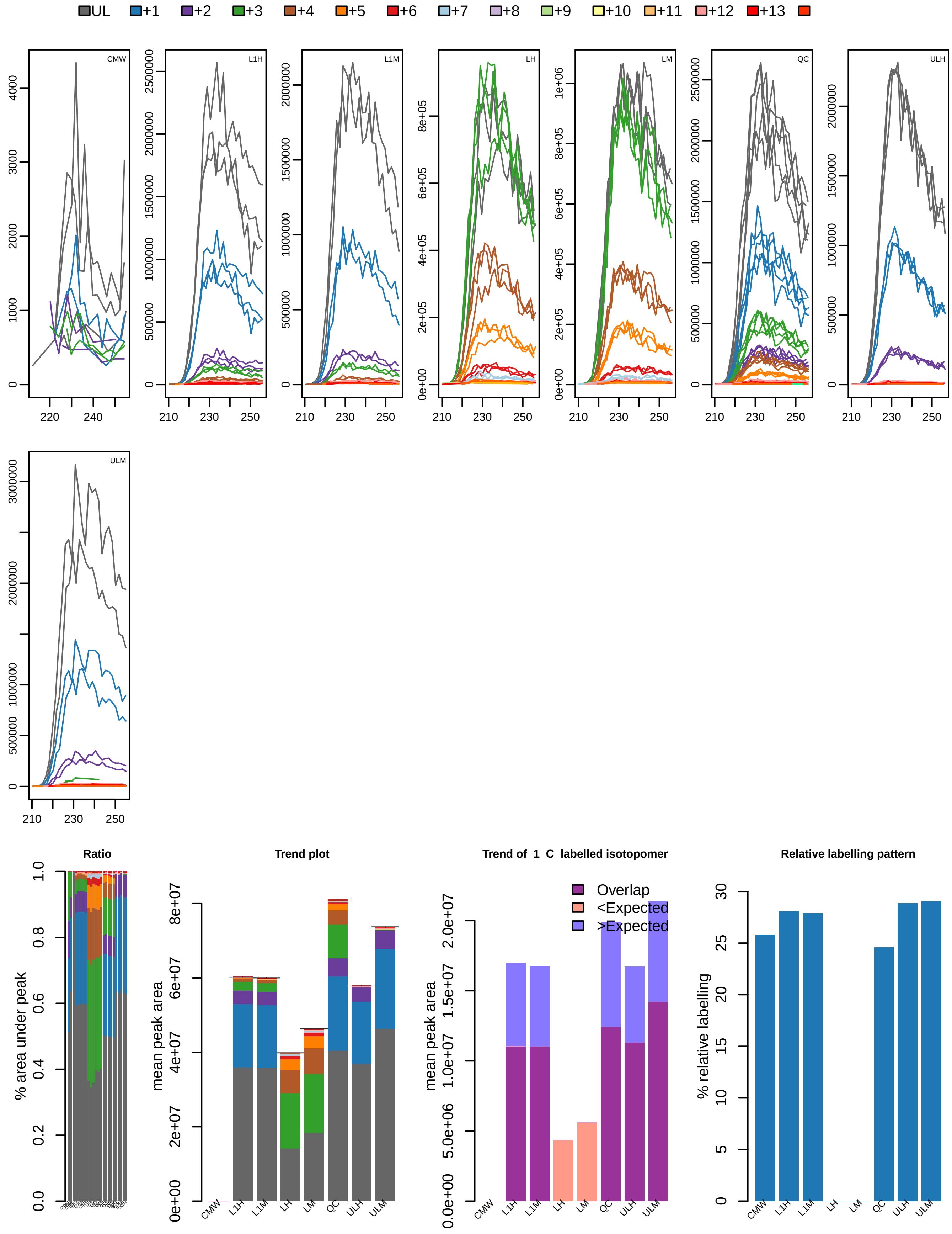

PC(14:1(9Z)/20:5(5Z,8Z,11Z,14Z,17Z))

Formula: C42H72NO8P Mass: 749.5 Std.RT: 232.80485682 Ion: PO[illegible]

G1

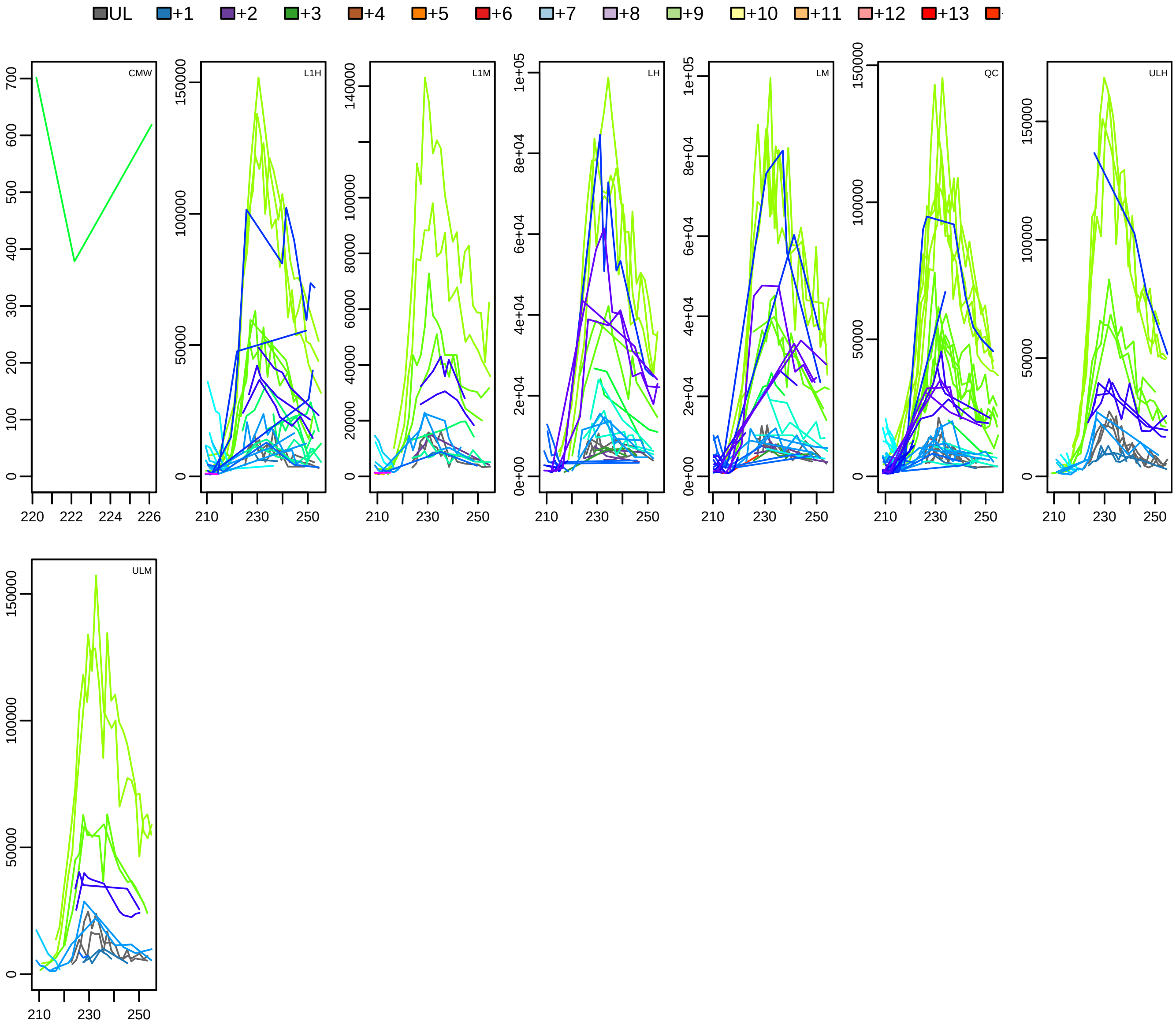

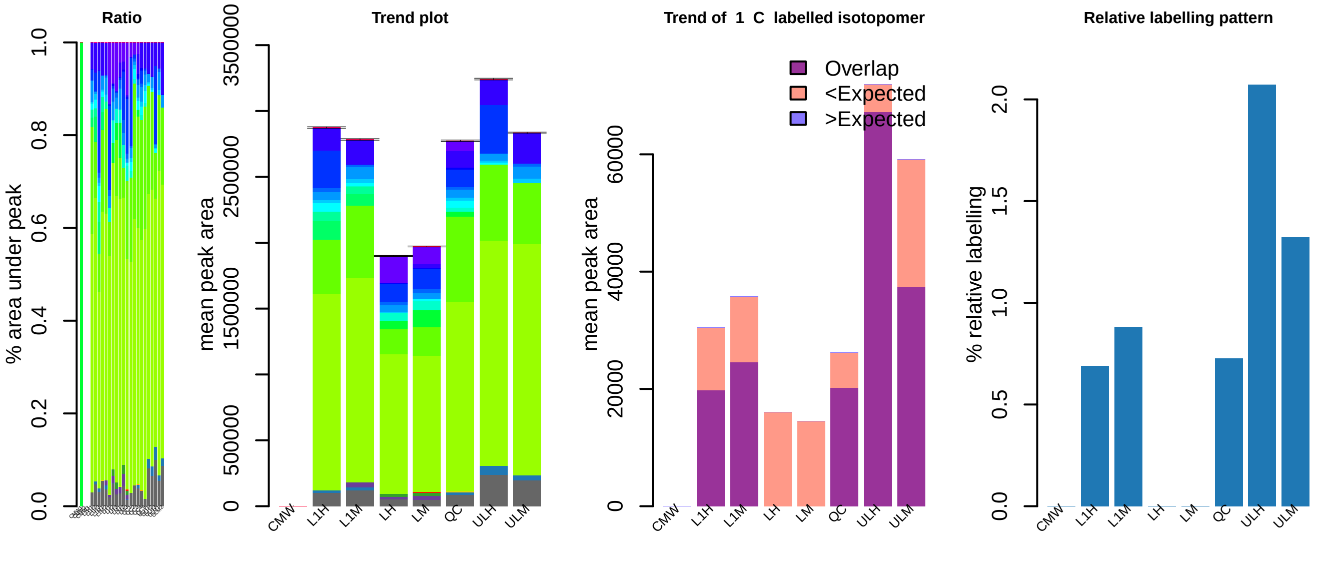

[PC (18:1/18:0)] 1−(1Z−octadecenyl)−2−(9Z−octadecer

Formula: C44H86NO7P Mass: 771.614 Std.RT: 233.60554122 Ion: P

G1

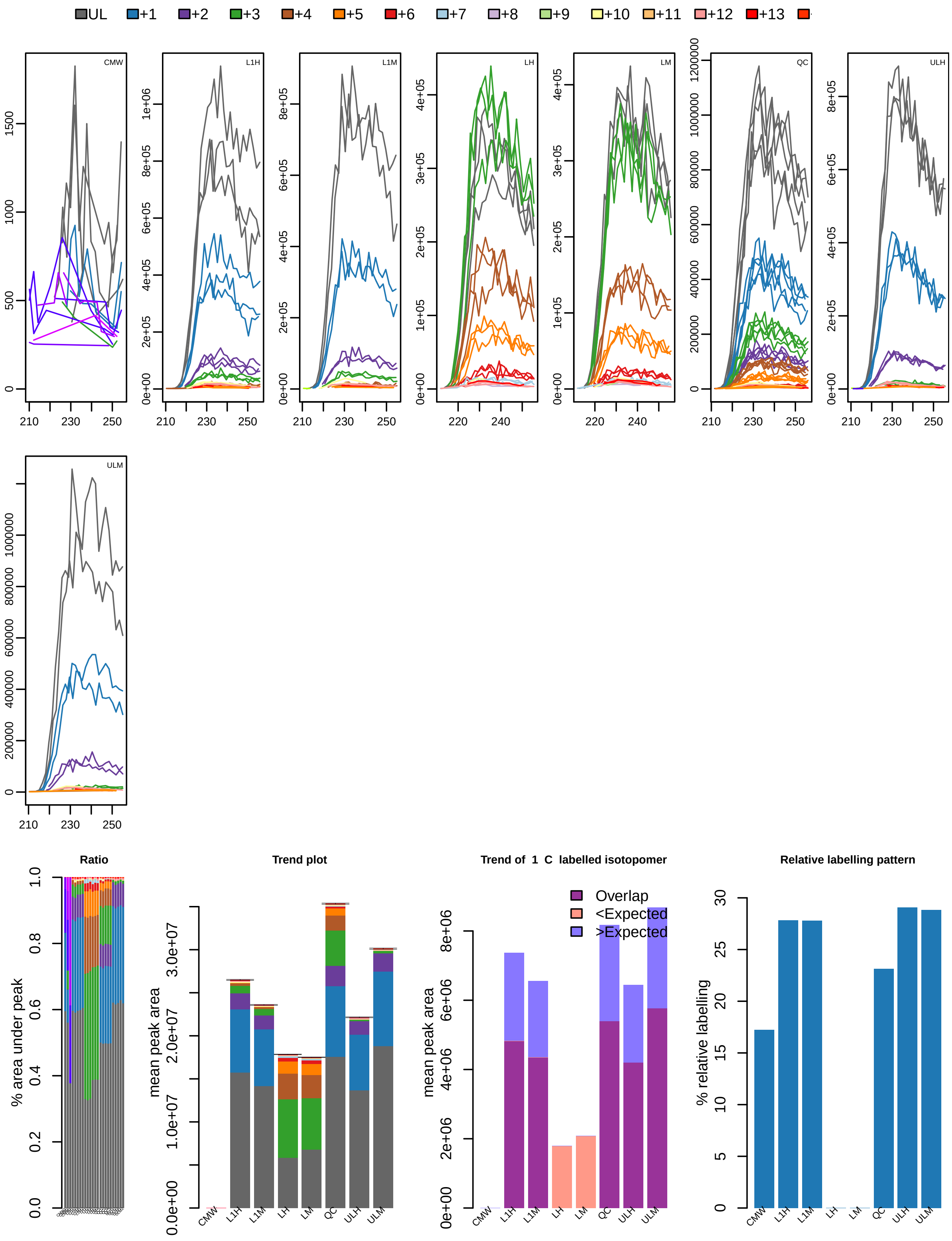

[PC (18:1/18:0)] 1−(1Z−octadecenyl)−2−(9Z−octadecer

Formula: C44H86NO7P Mass: 771.614 Std.RT: 233.60554122 Ion: P

G2

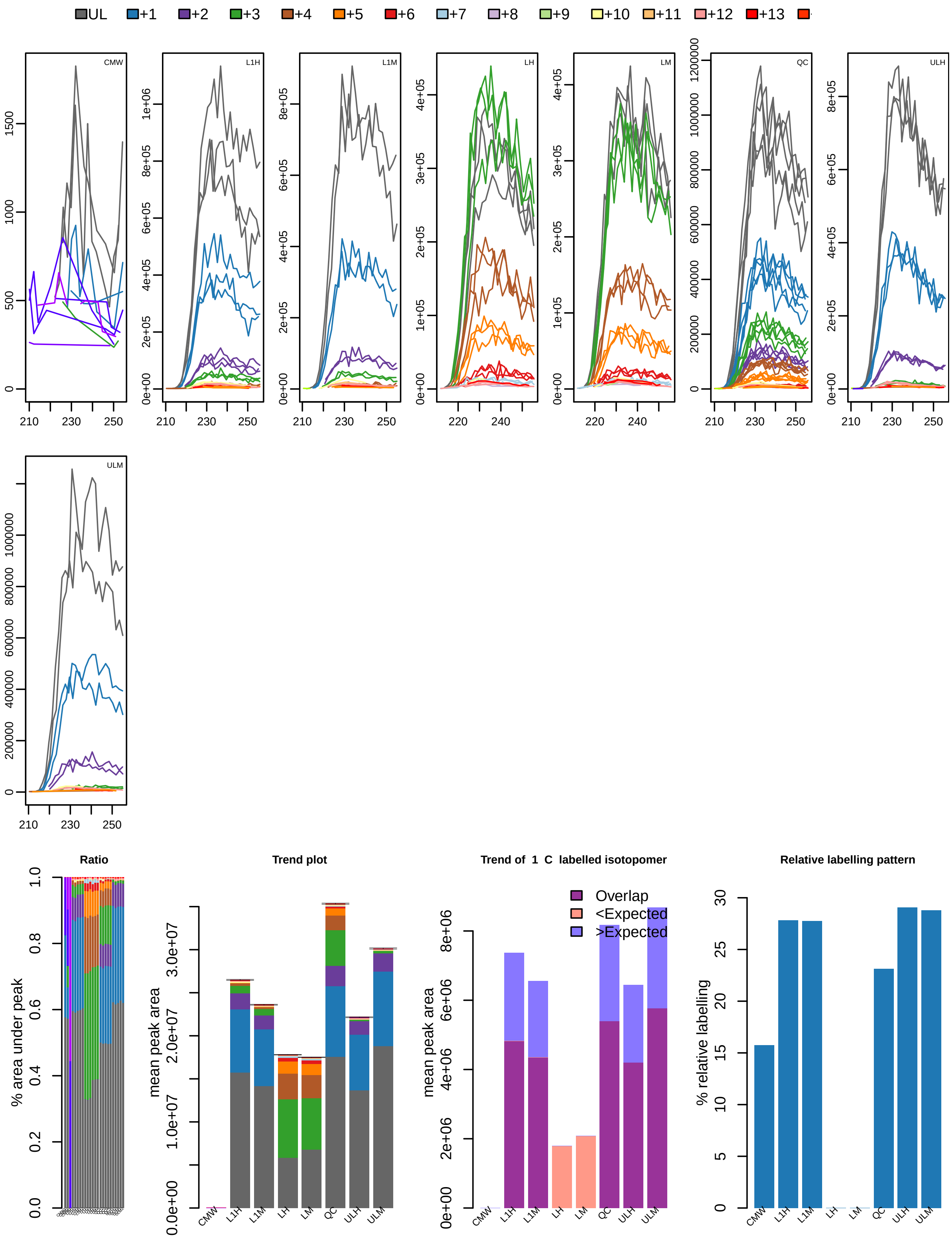

PC(18:2(9Z,12Z)/18:1(9Z))

Formula: C44H82NO8P Mass: 783.578 Std.RT: 230.79287472 Ion: P

G1

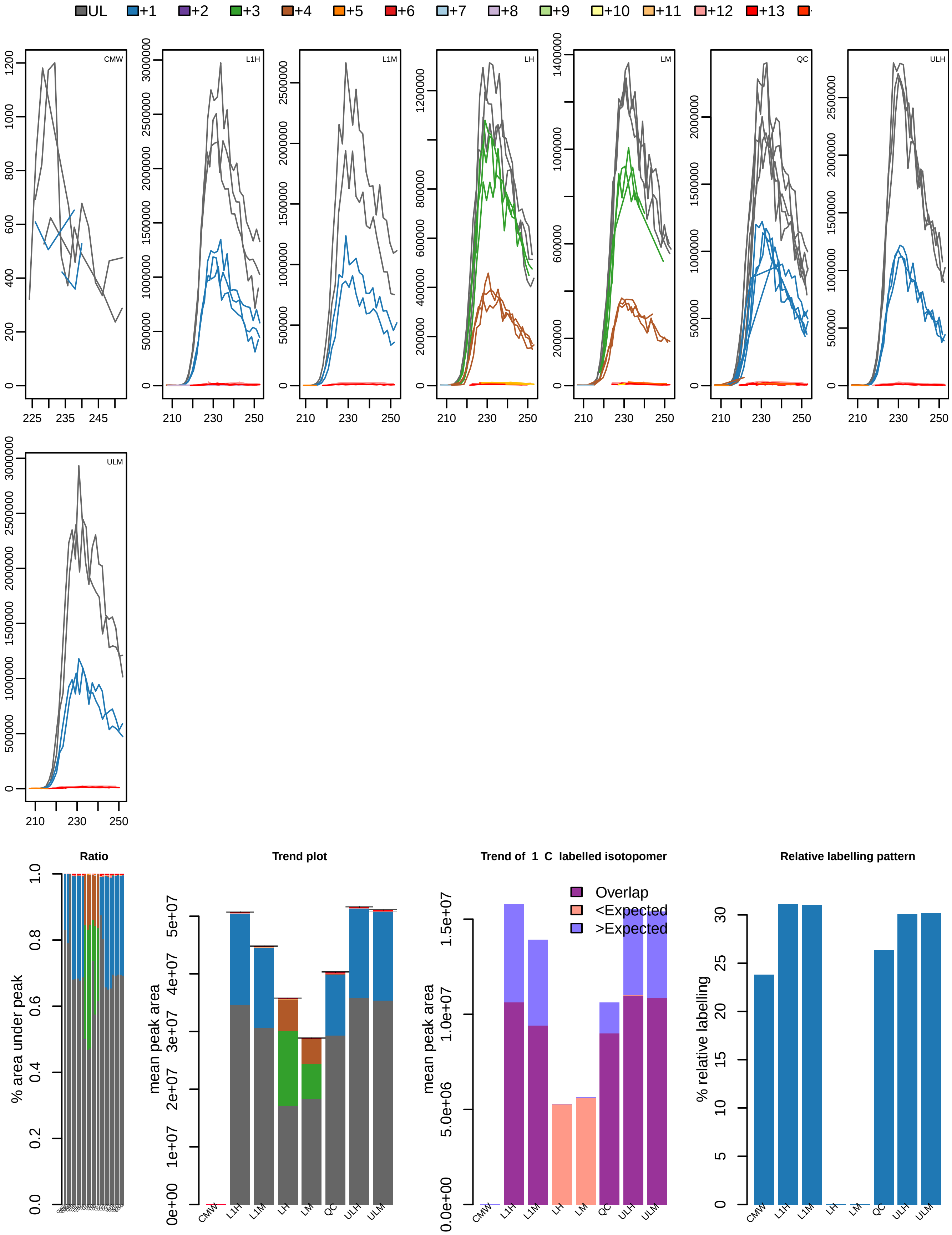

PC(14:1(9Z)/15:0)

Formula: C37H72NO8P Mass: 689.5 Std.RT: 208.44261678 Ion: PO[illegible]

G1

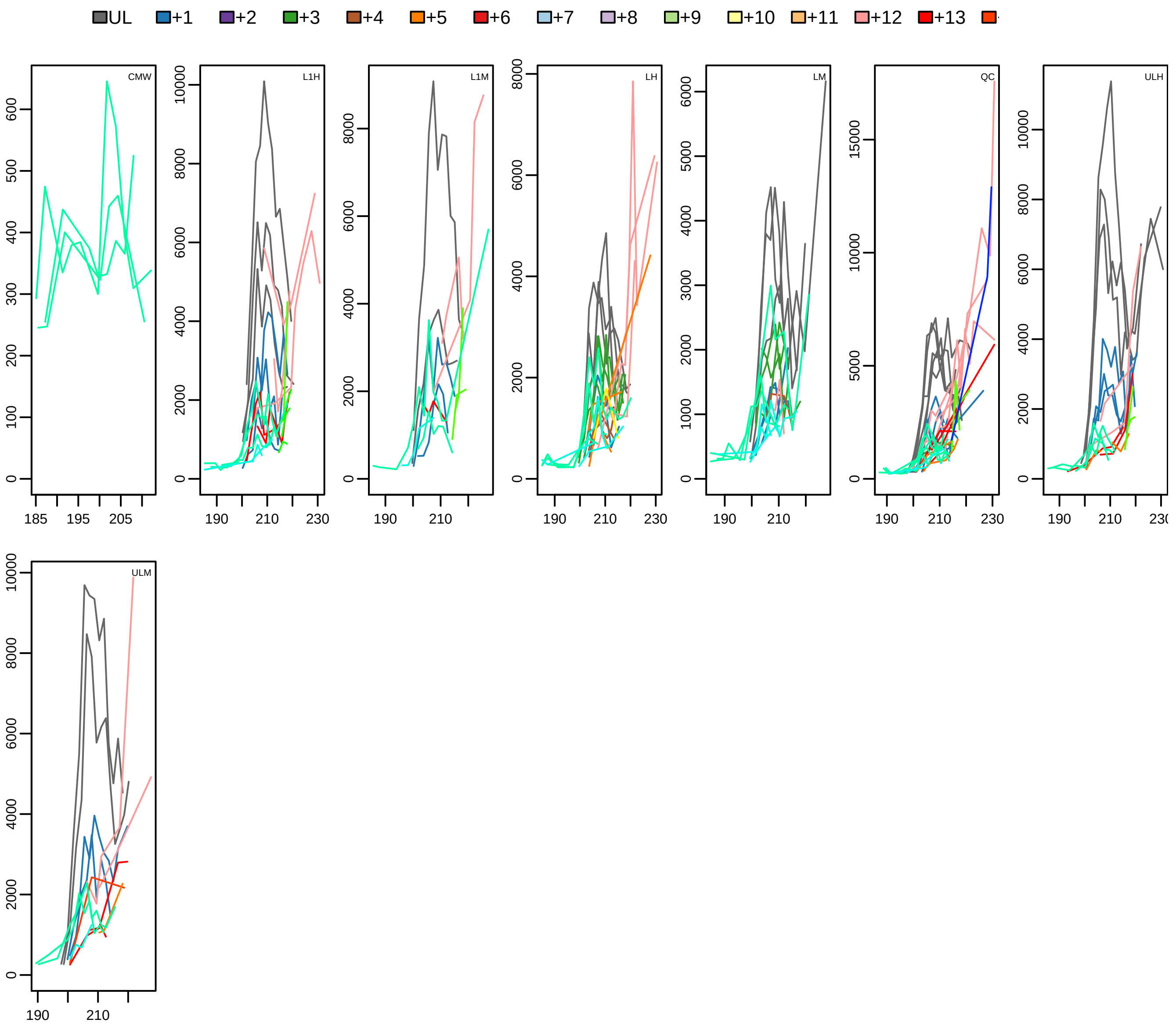

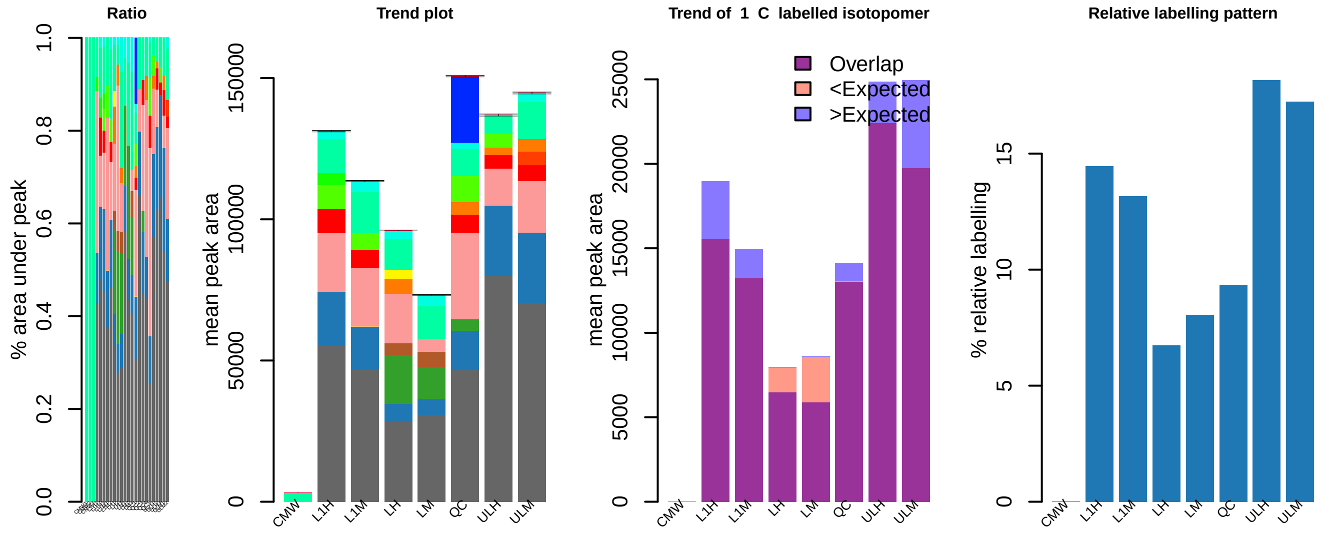

[PC (14:0/20:4)] 1−tetradecanoyl−2−(5Z,8Z,11Z,14Z−e

Formula: C42H76NO8P Mass: 753.531 Std.RT: 231.58095372 Ion: P

G1

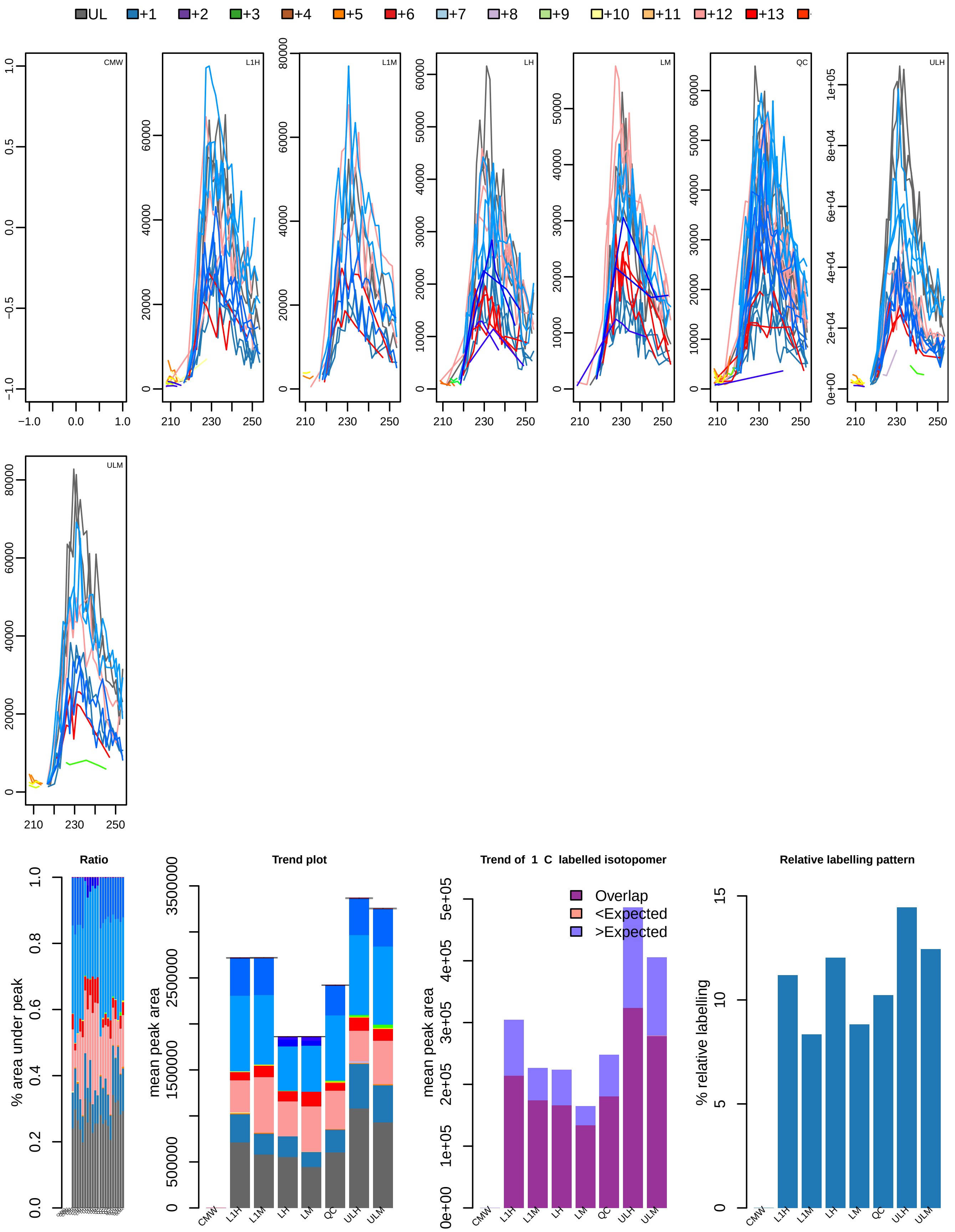

[PC (16:0/20:4)] 1−hexadecanoyl−2−(5Z,8Z,11Z,14Z−e

Formula: C44H80NO8P Mass: 781.562 Std.RT: 230.58539022 Ion: P

G1

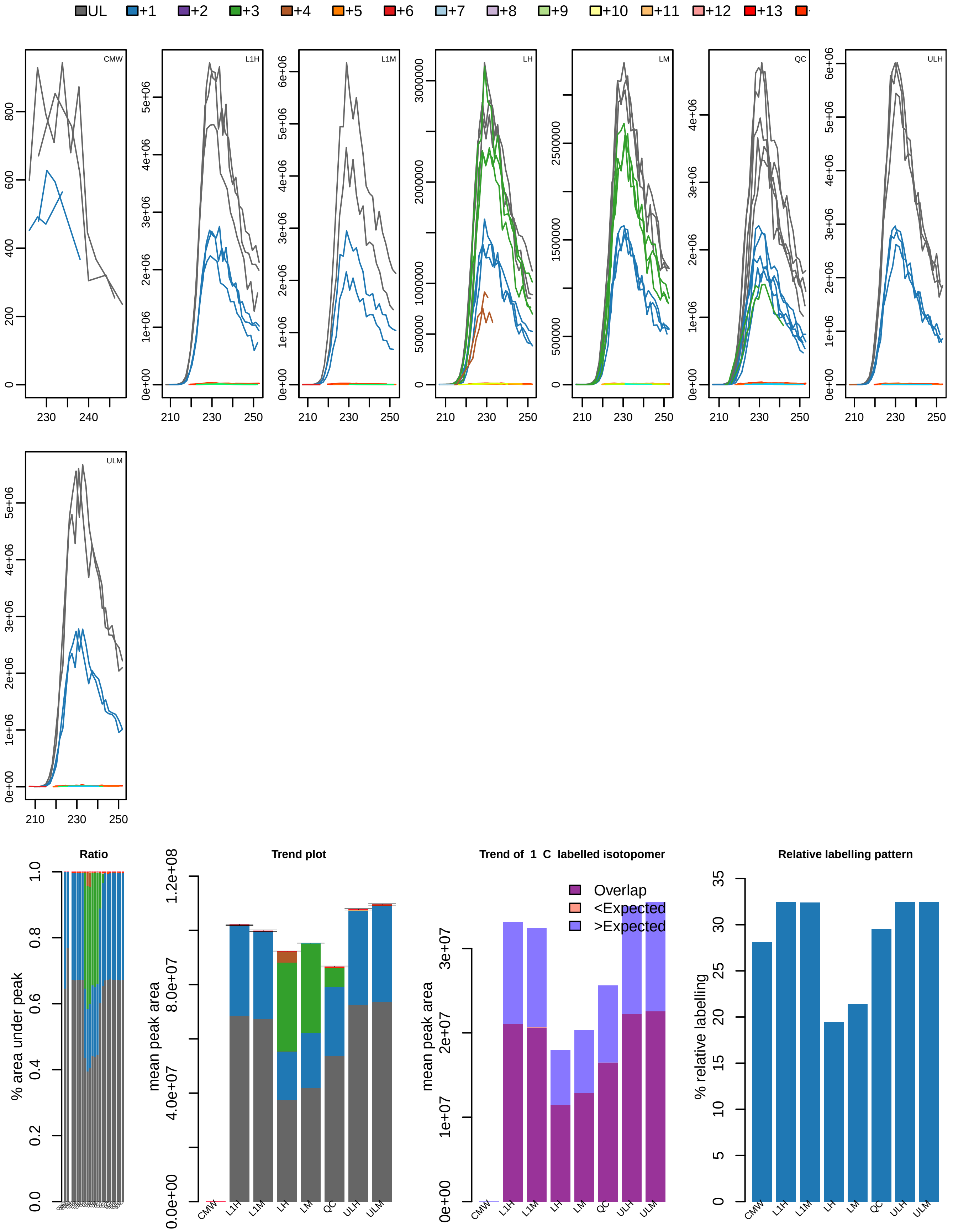

[PC (16:0/18:2)] 1−hexadecanoyl−2−(9Z,12Z−octadeca

Formula: C42H80NO8P Mass: 757.562 Std.RT: 232.6014576 Ion: PC

G1

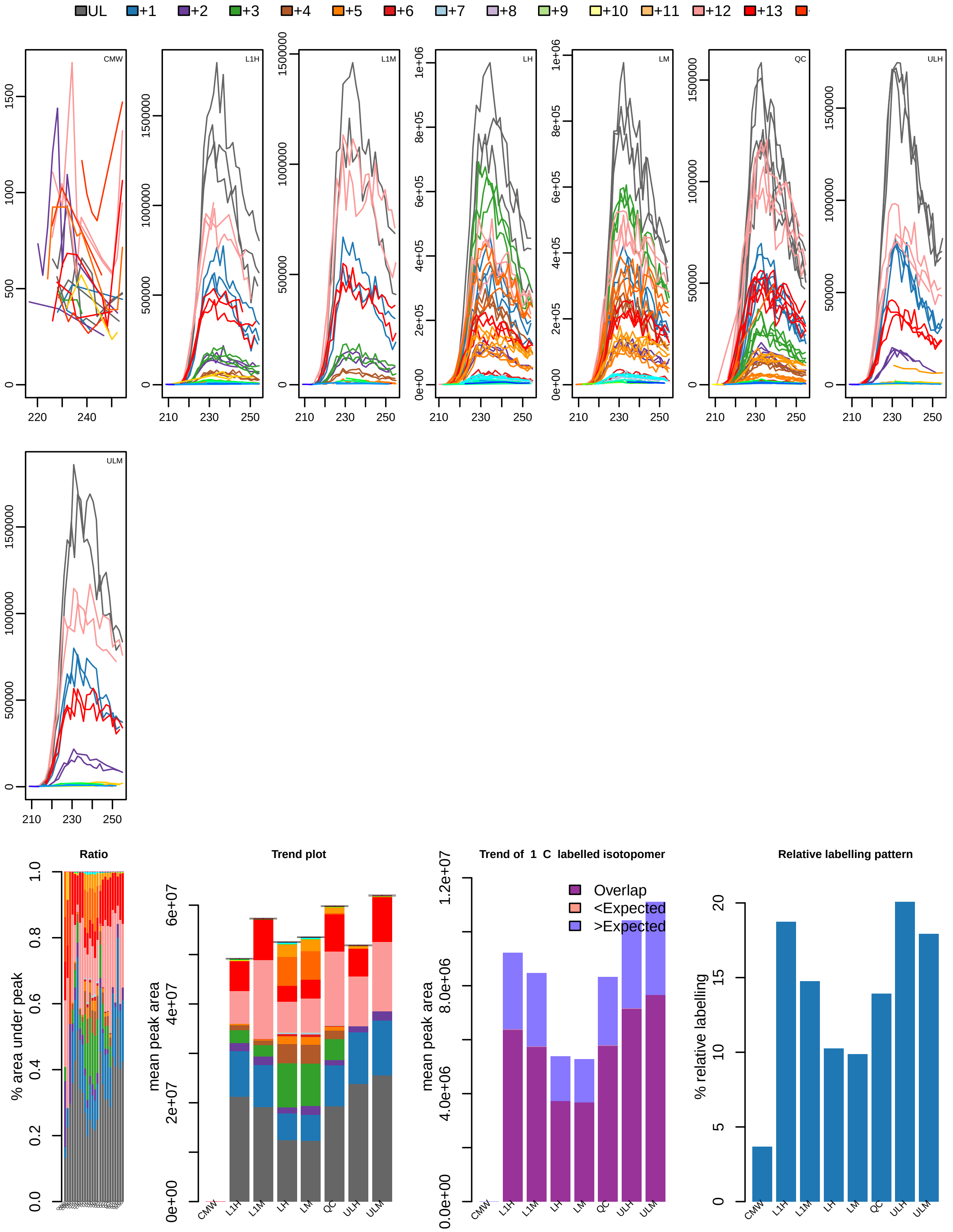

PC(18:1(11Z)/P−18:1(11Z))

G1

Formula: C44H84NO7P Mass: 769.599 Std.RT: 233.59238928 Ion: P

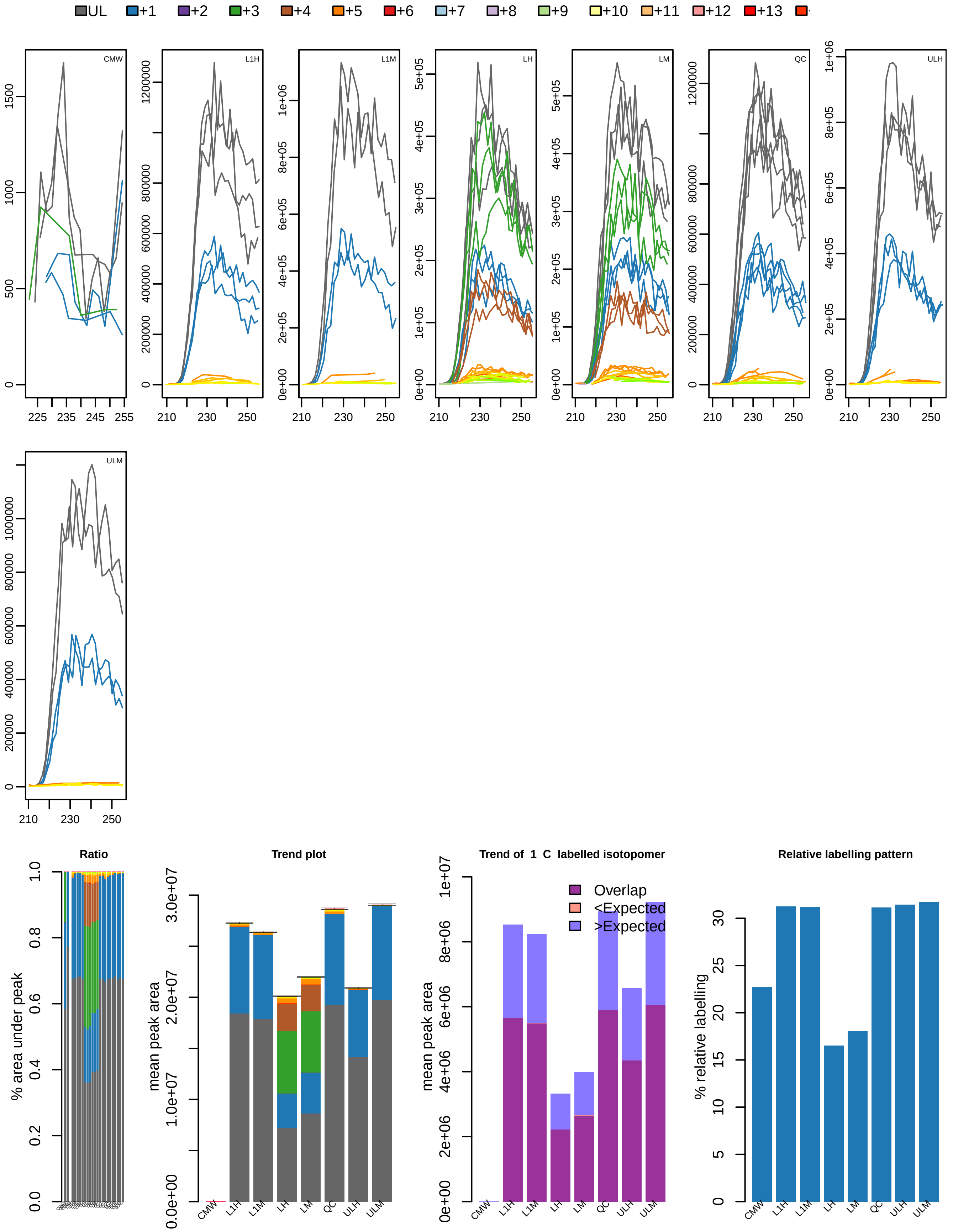

[PC (18:0/22:4)] 1−octadecanoyl−2−(7Z,10Z,13Z,16Z−

Formula: C48H88NO8P Mass: 837.625 Std.RT: 230.9043318 Ion: PC

G1

UL +1 +2 +3 +4 +5 +6 +7 +8 +9 +10 +11 +12 +13

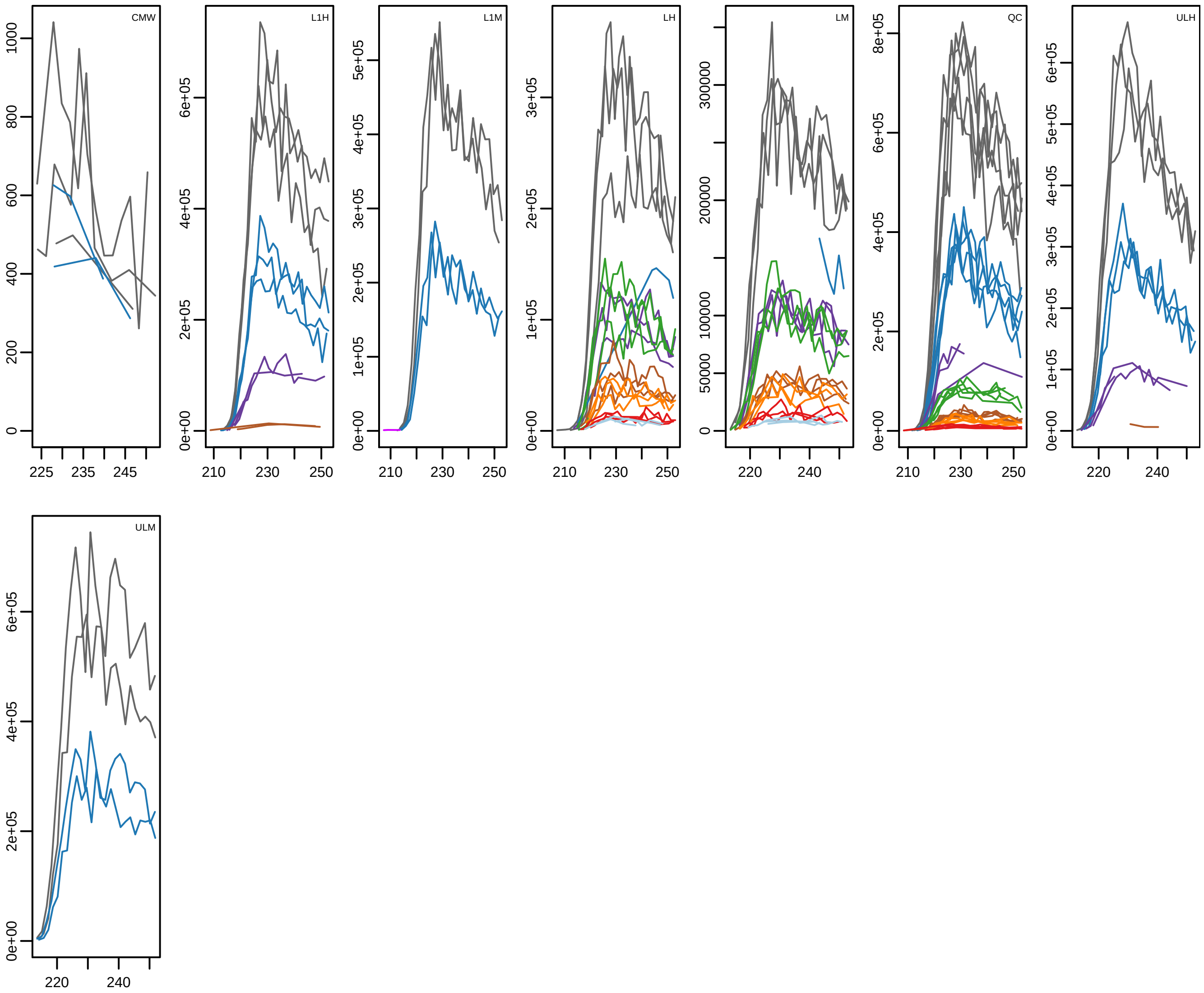

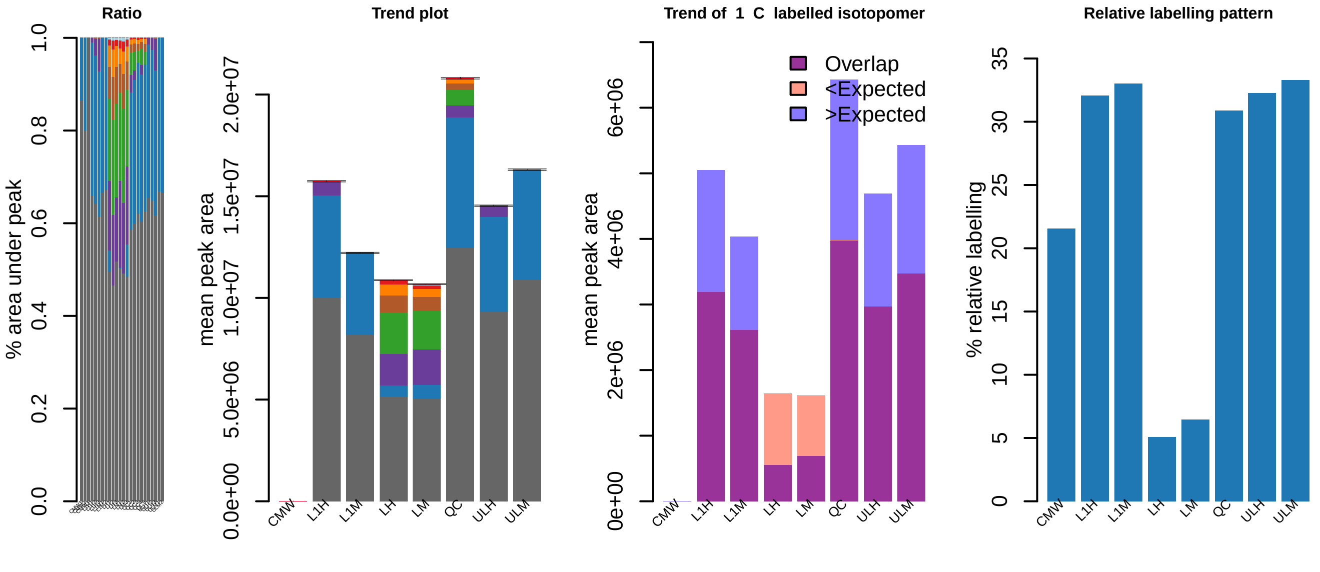

[PC (18:2)] 1−(9Z,12Z−octadecadienoyl)−sn−glycero−3

Formula: C26H50NO7P Mass: 519.332 Std.RT: 258.3183327 Ion: PC

G1

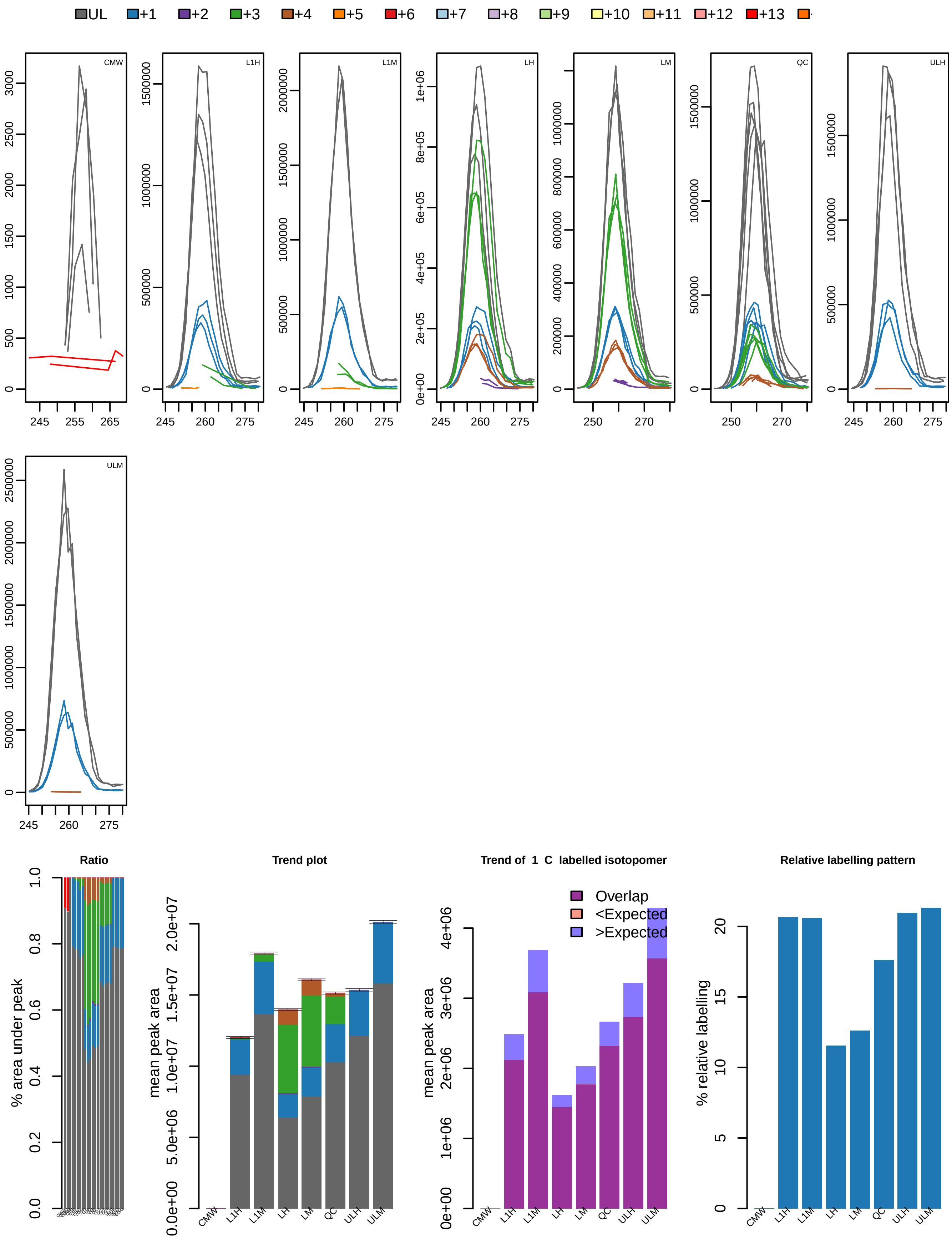

[PC (18:2)] 1−(9Z,12Z−octadecadienoyl)−sn−glycero−3

Formula: C26H50NO7P Mass: 519.332 Std.RT: 258.3183327 Ion: PC

G2

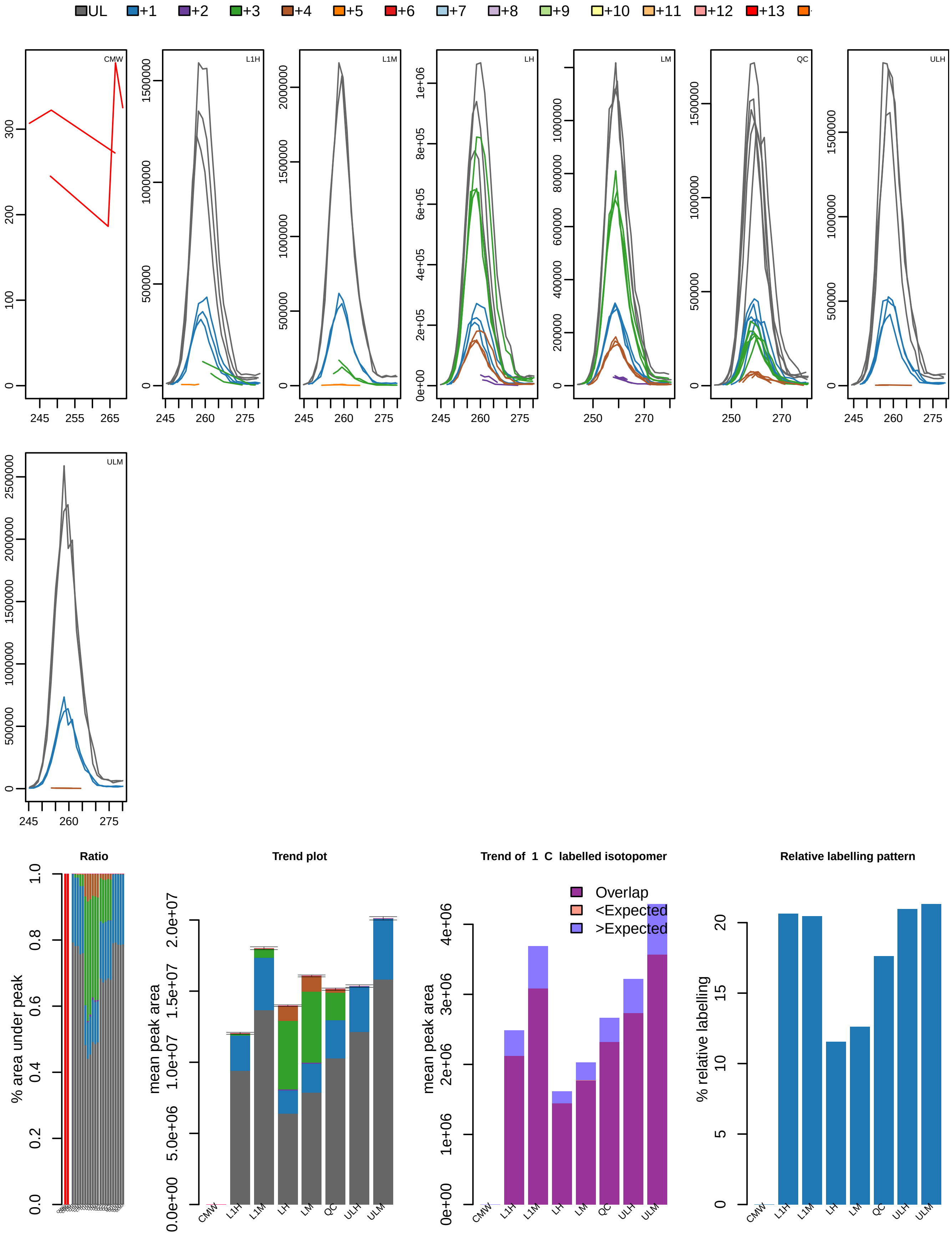

[PC (18:0/20:2)] 1−octadecanoyl−2−(11Z,14Z−eicosadi

Formula: C46H88NO8P Mass: 813.625 Std.RT: 233.0885235 Ion: PC

G1

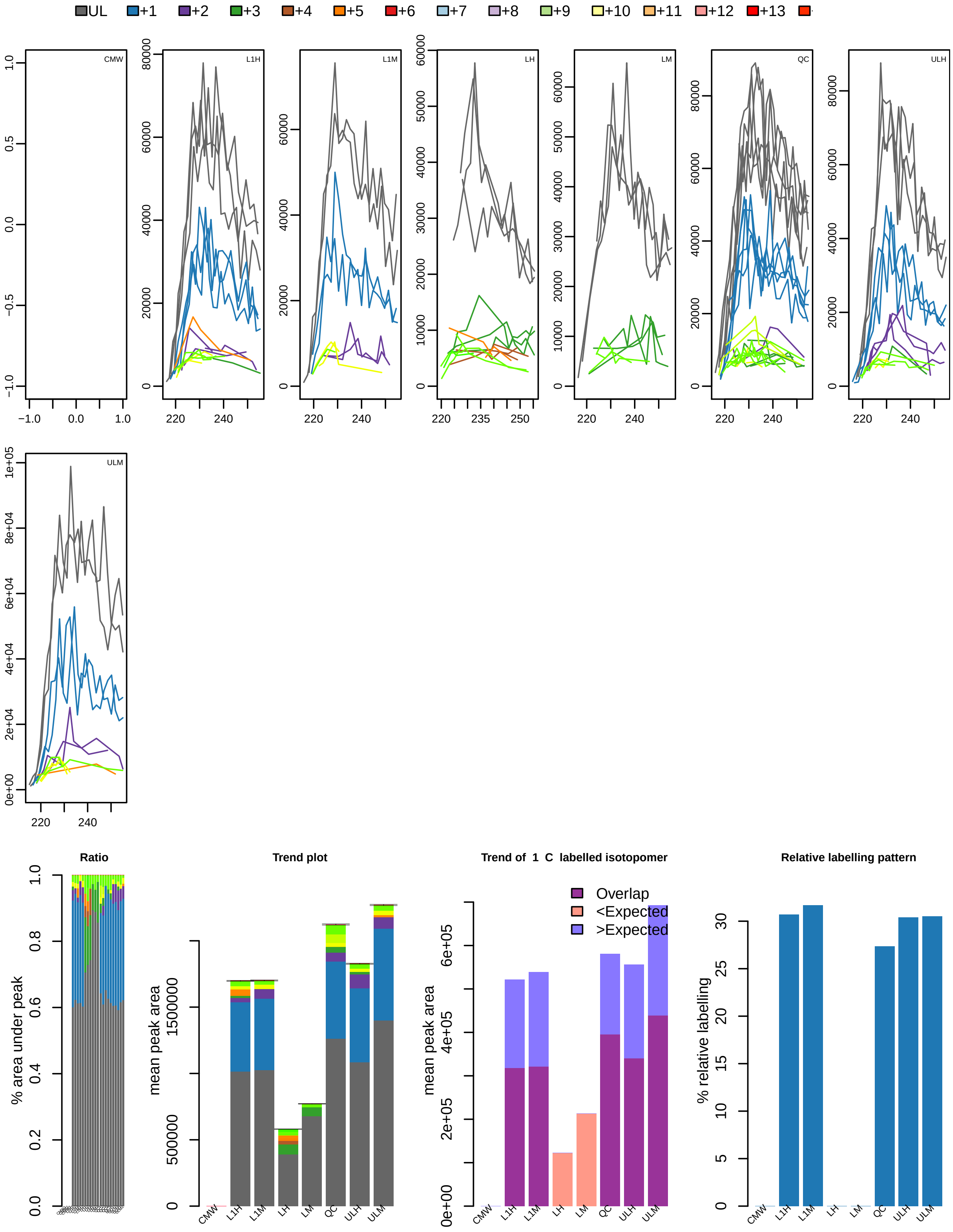

PC(15:0/20:3(5Z,8Z,11Z))

Formula: C43H80NO8P Mass: 769.562 Std.RT: 231.73147578 Ion: P

G1

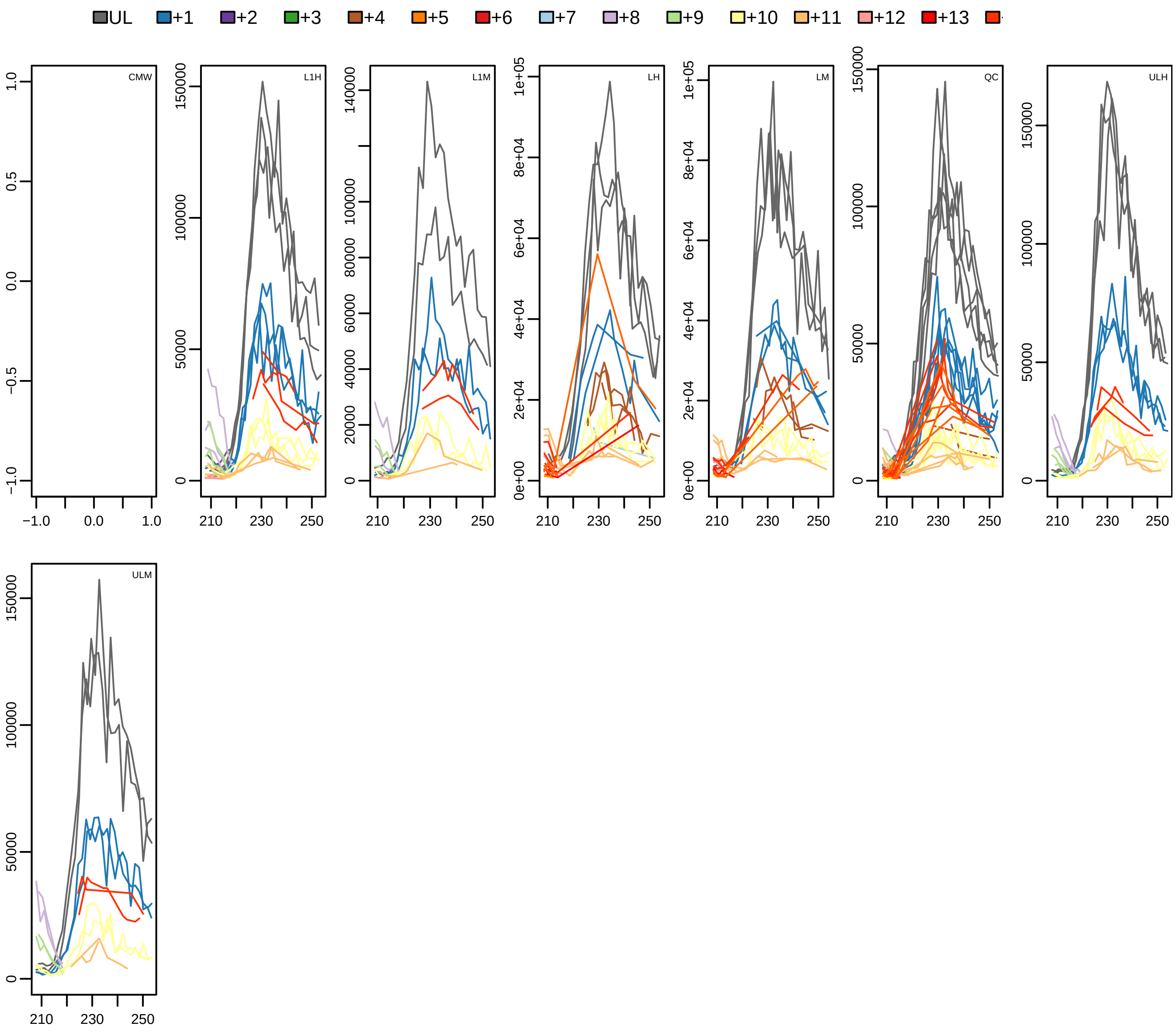

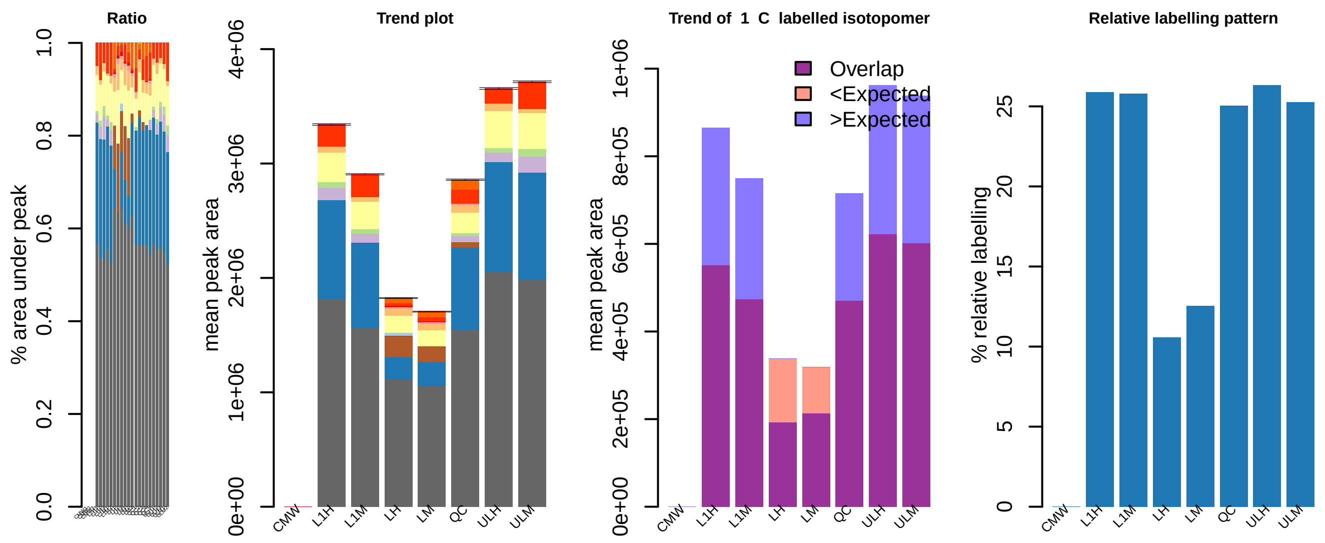

[PC (16:0/18:3)] 1−hexadecanoyl−2−(9Z,12Z,15Z−octa

Formula: C42H78NO8P Mass: 755.547 Std.RT: 231.58028592 Ion: P

G1

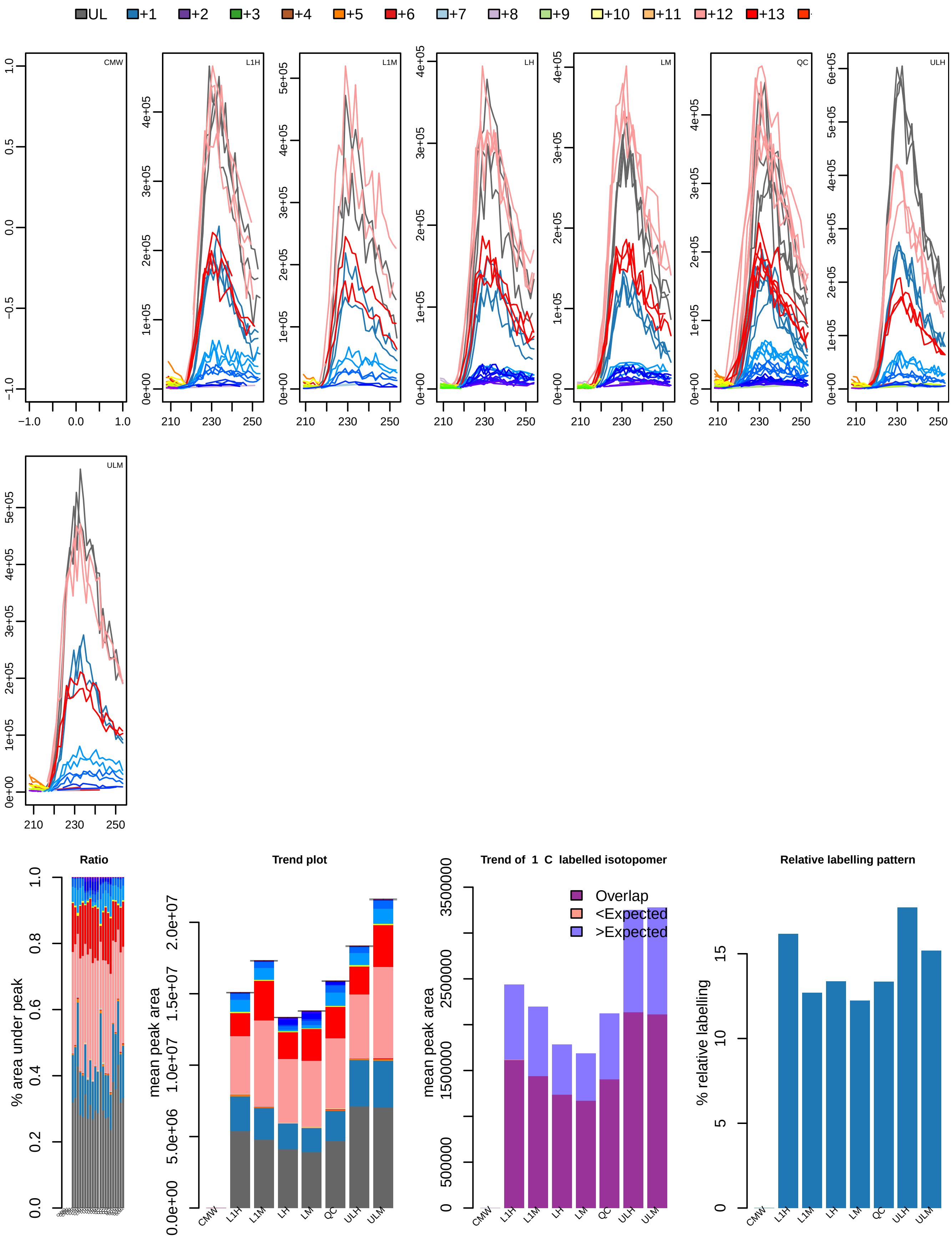

[PC (18:1/22:5)] 1−(11Z−octadecenoyl)−2−(7Z,10Z,13Z

Formula: C48H84NO8P Mass: 833.593 Std.RT: 308.96223522 Ion: P

G1

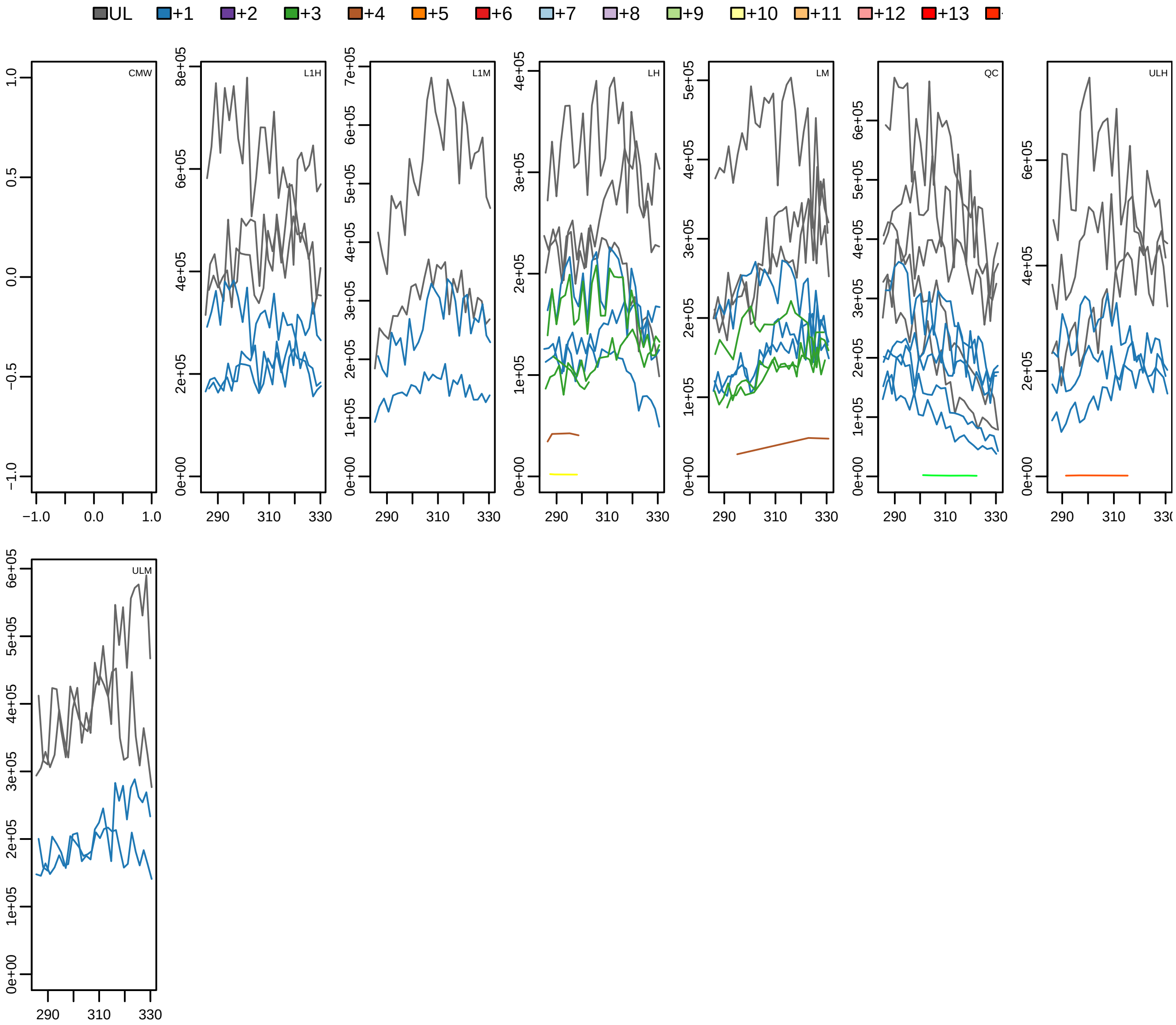

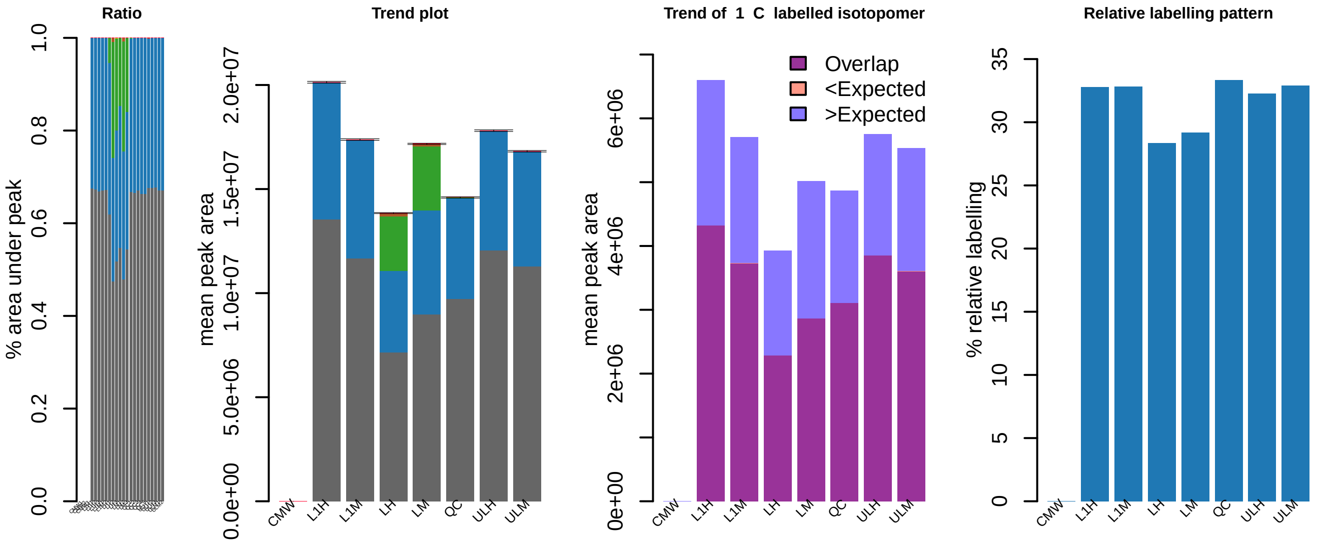

[PC (18:1/22:5)] 1−(11Z−octadecenoyl)−2−(7Z,10Z,13Z

Formula: C48H84NO8P Mass: 833.593 Std.RT: 308.96223522 Ion: P

G2

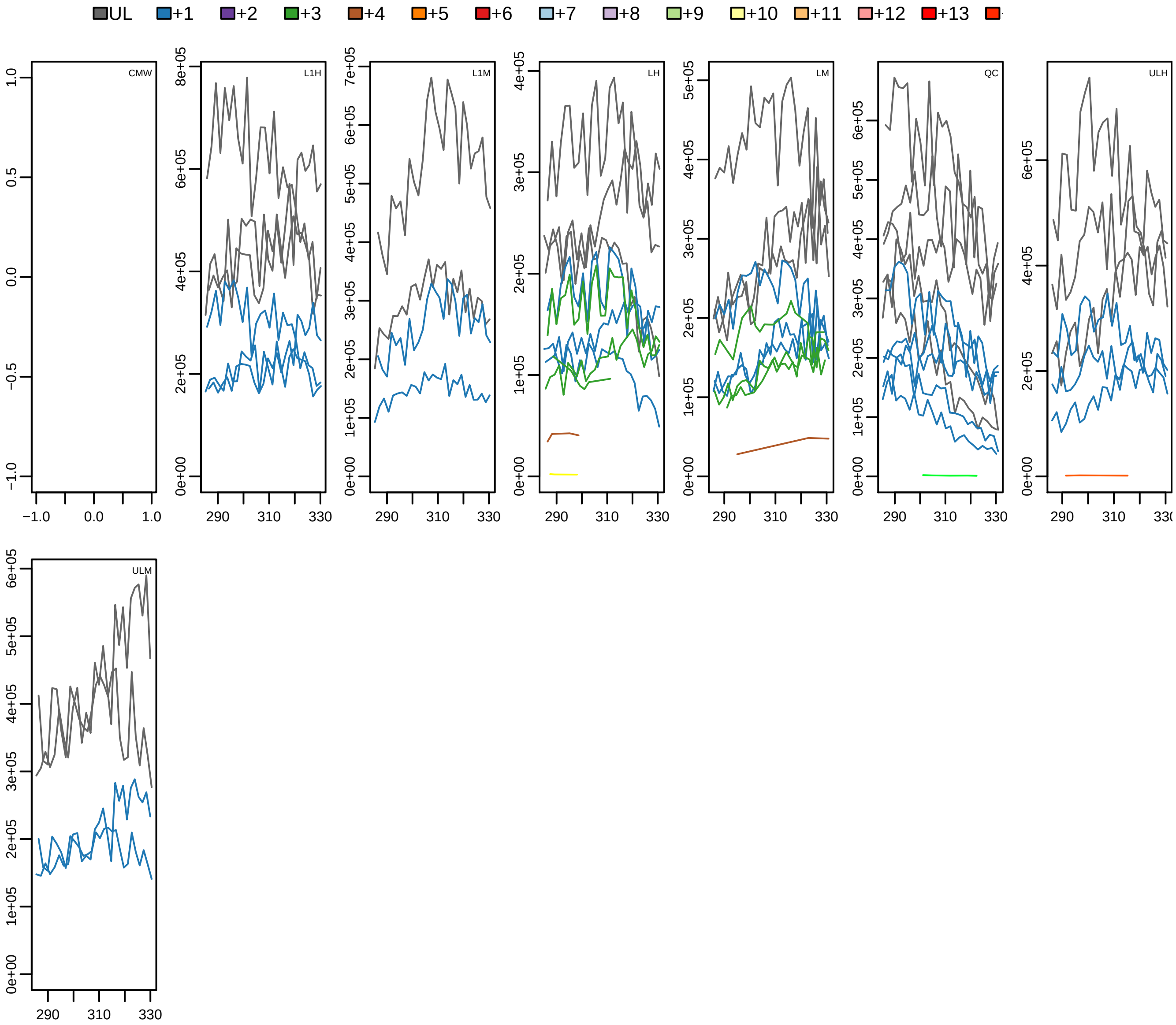

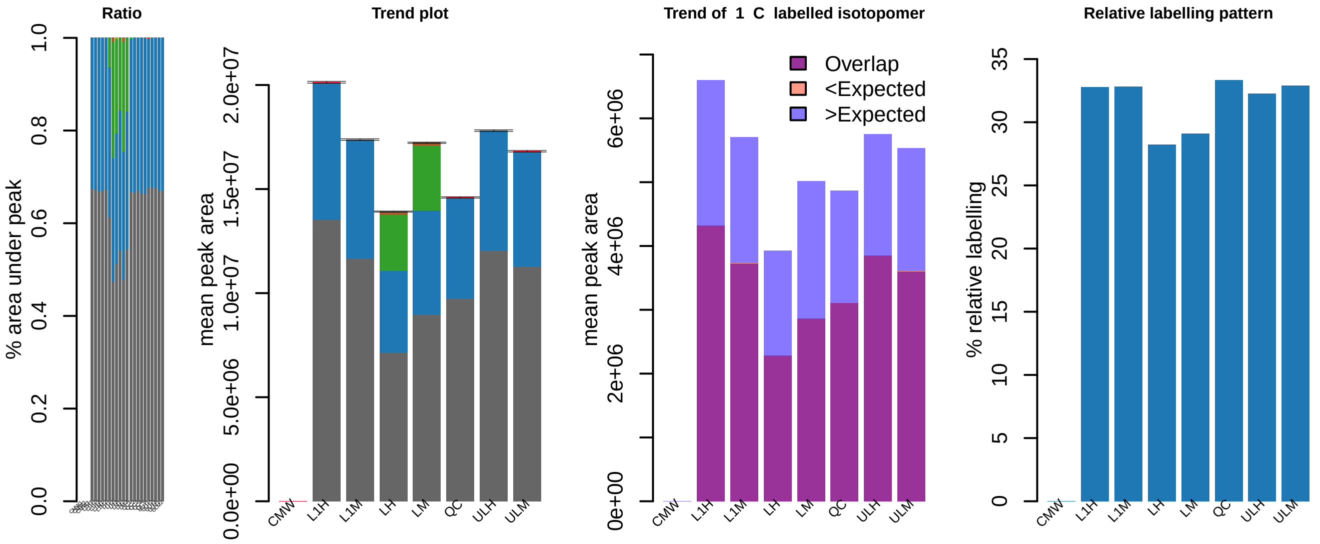

LysoPC(O−18:0)

Formula: C26H56NO6P Mass: 509.385 Std.RT: 258.77652852 Ion: P

G1

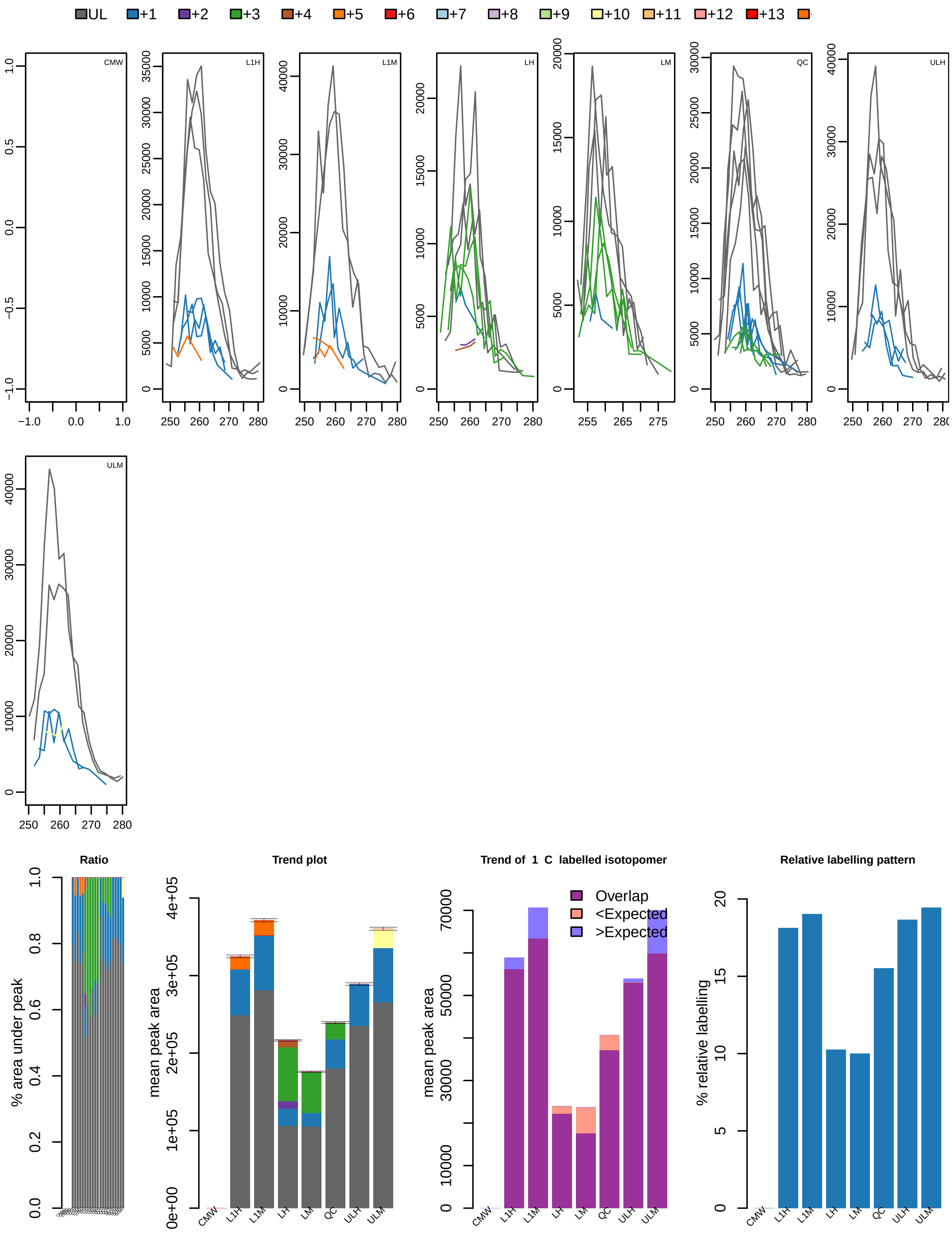

[PC (17:0)] 1−(10Z−heptadecenoyl)−sn−glycero−3−pho

Formula: C25H50NO7P Mass: 507.332 Std.RT: 259.65630108 Ion: P

G1

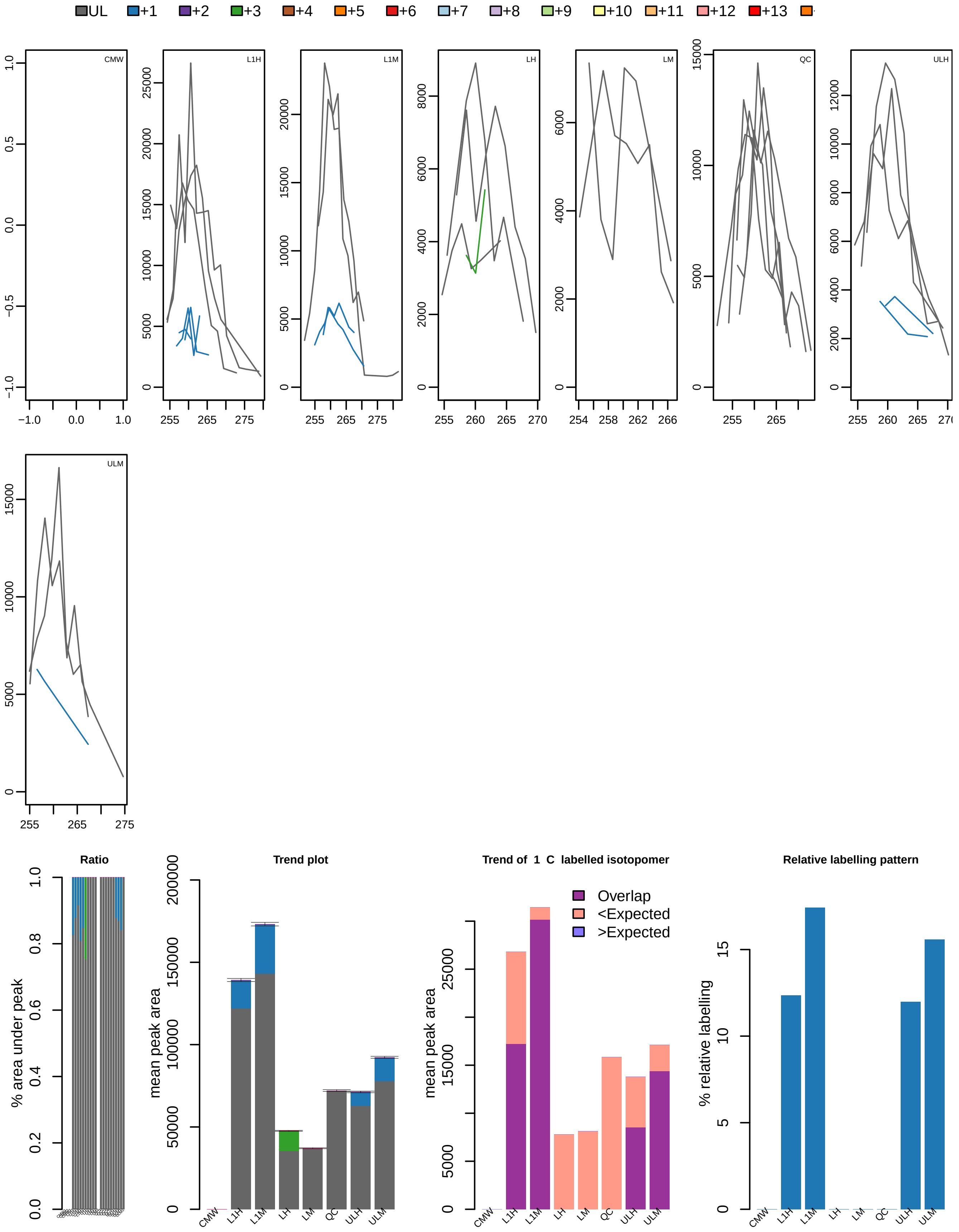

[PC (18:3)] 1−(9Z,12Z,15Z−octadecatrienoyl)−sn−glyce

Formula: C26H48NO7P Mass: 517.317 Std.RT: 259.8078105 Ion: PC

G1

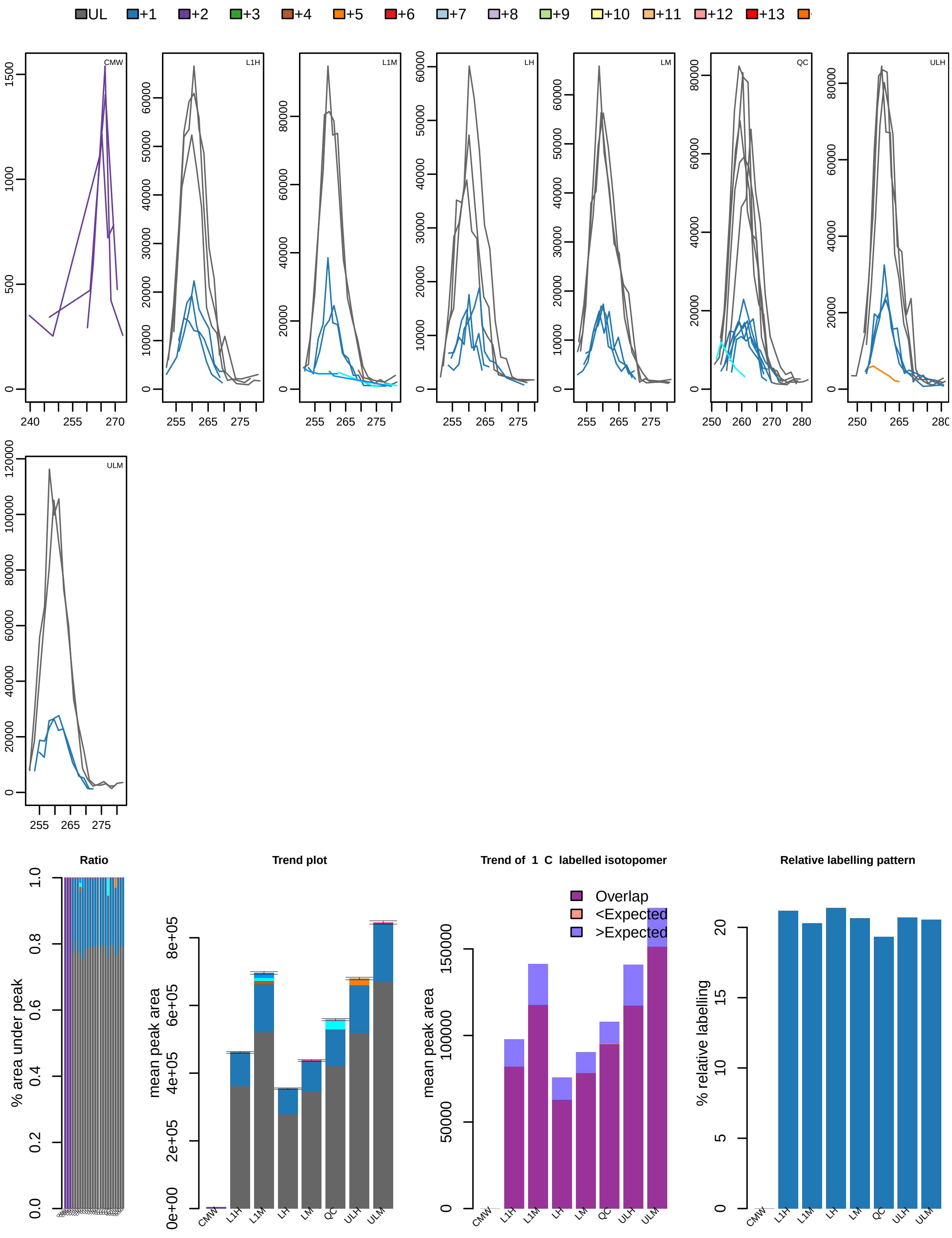

LysoPC(22:4(7Z,10Z,13Z,16Z))

Formula: C30H54NO7P Mass: 571.364 Std.RT: 251.038572 Ion: PO[illegible]

G1

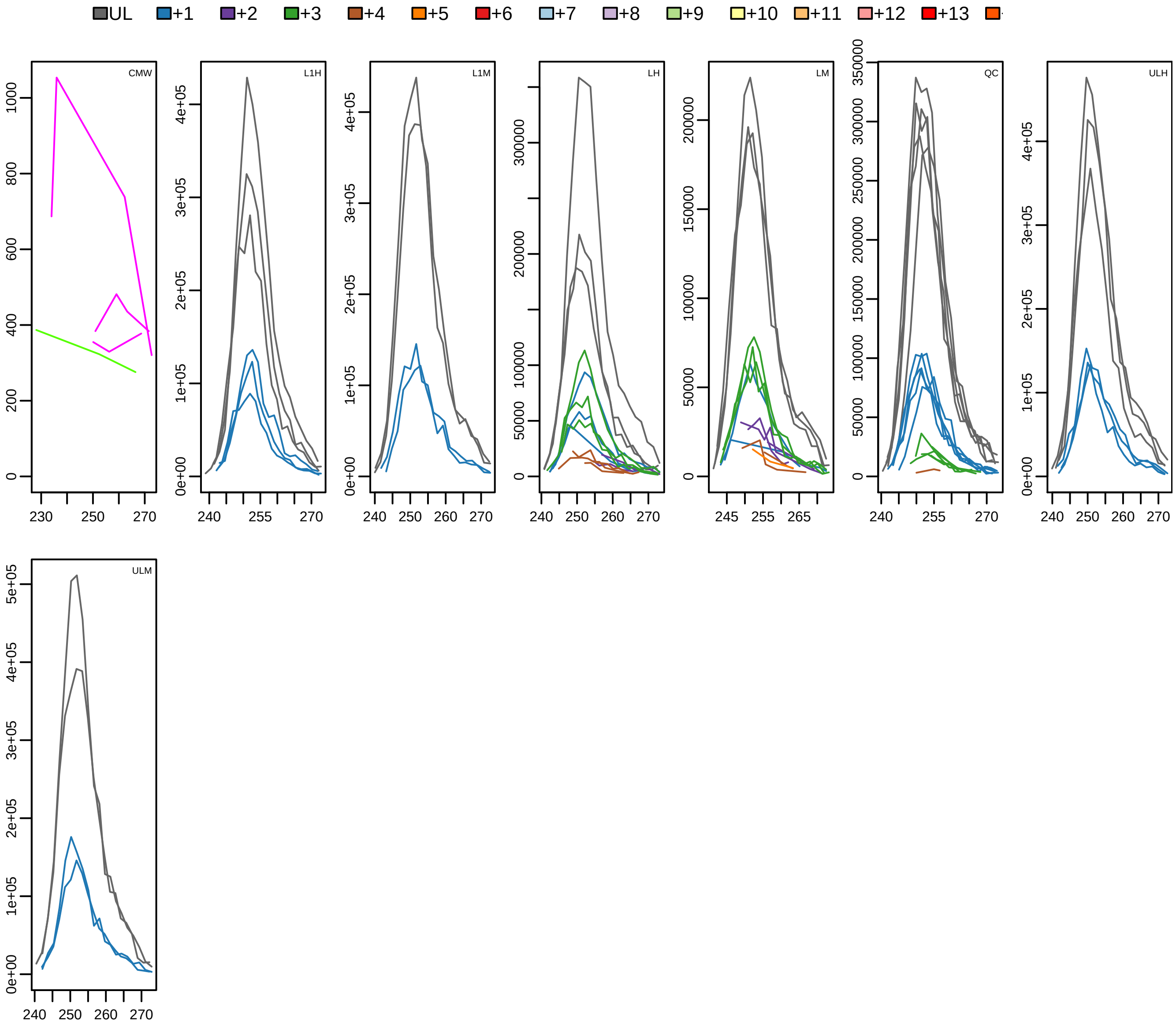

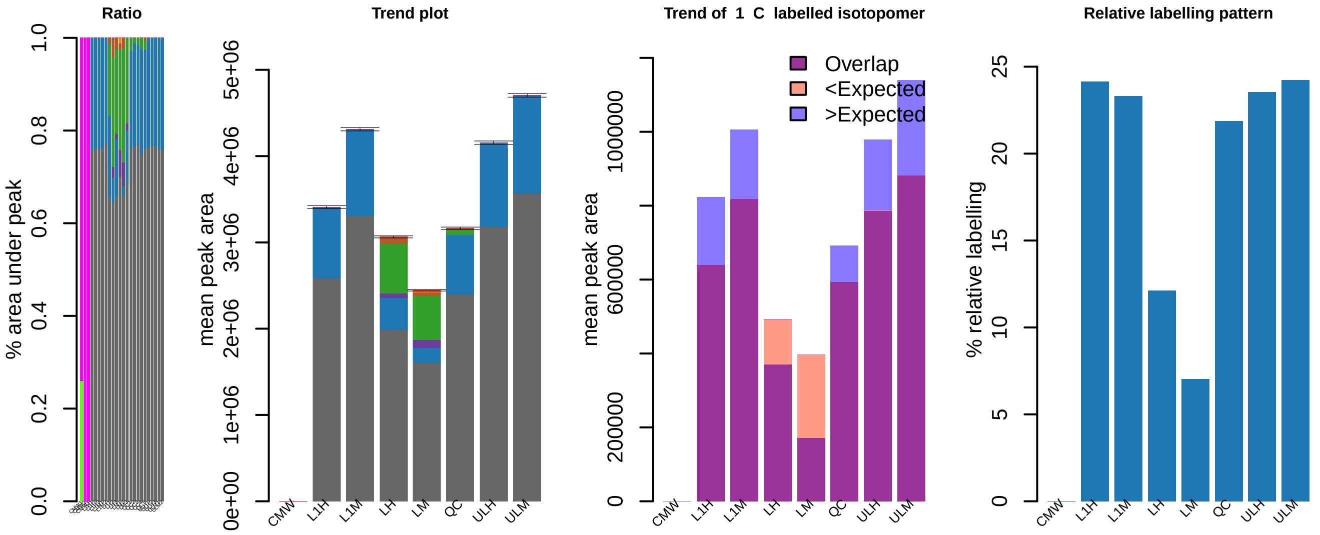

[PC (18:1/20:3)] 1−(9Z−octadecenoyl)−2−(5Z,8Z,11Z−e

Formula: C46H84NO8P Mass: 809.593 Std.RT: 229.77931632 Ion: P

G1

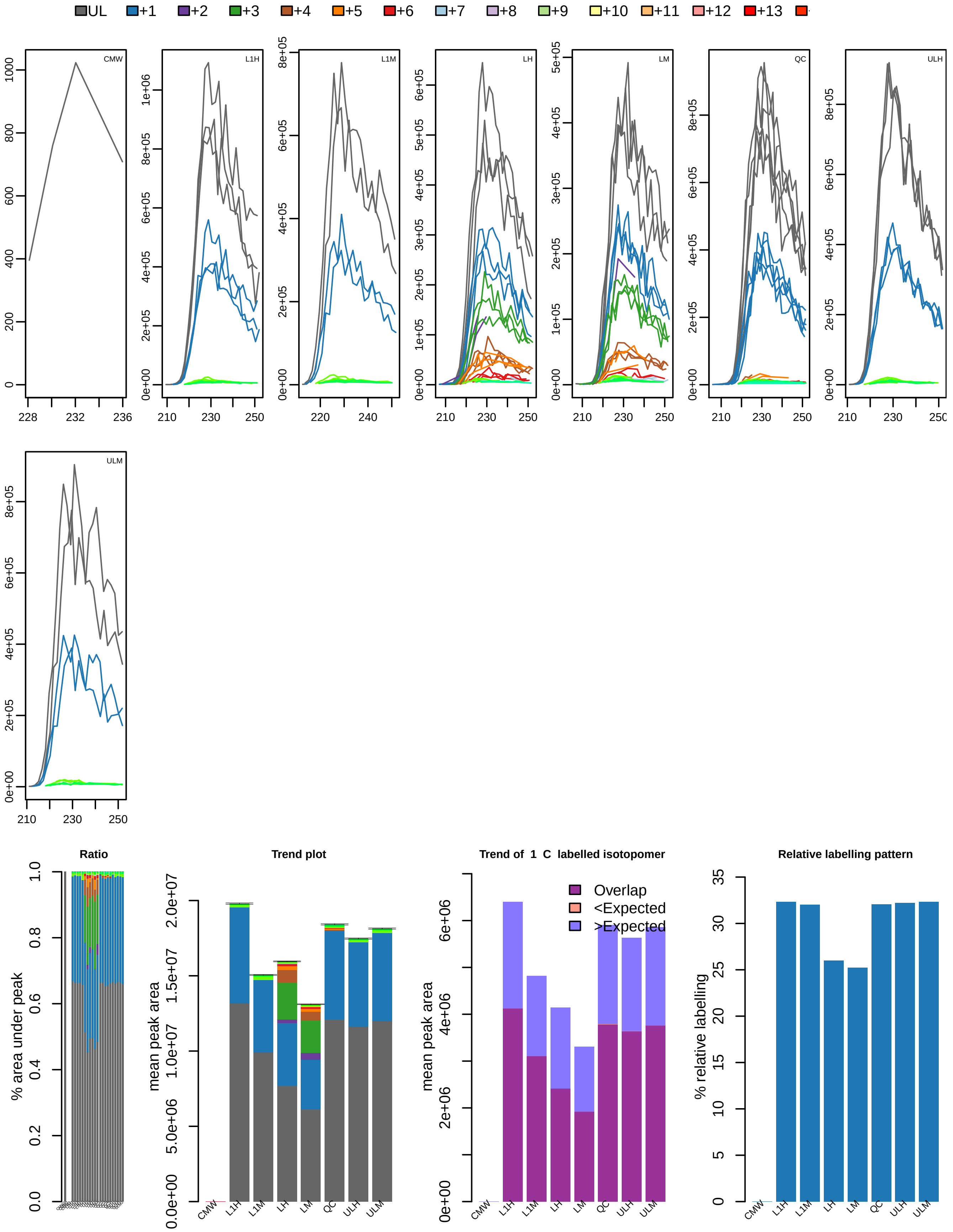

LysoPC(20:1(11Z))

Formula: C28H56NO7P Mass: 549.379 Std.RT: 253.4102631 Ion: PC

G1

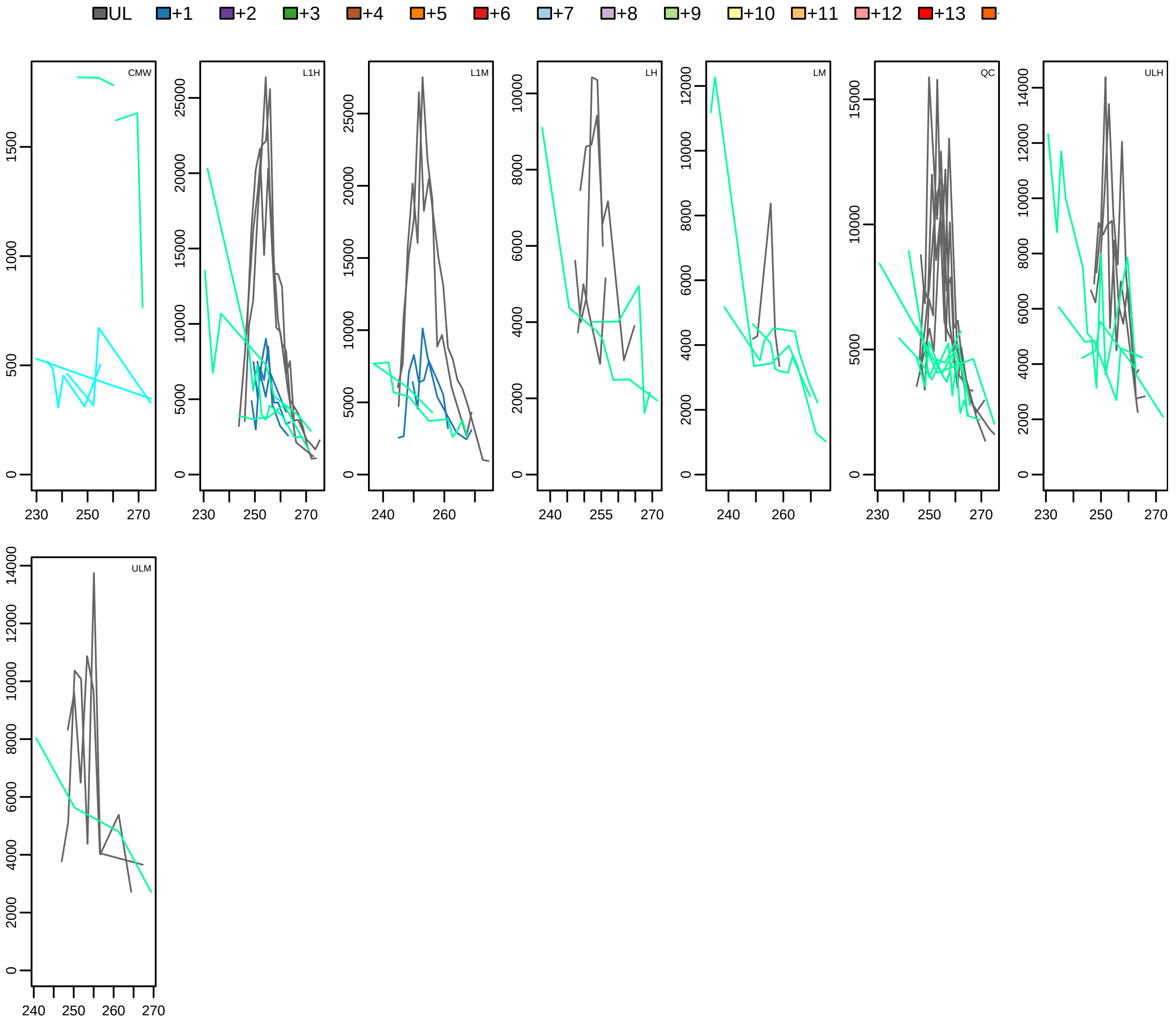

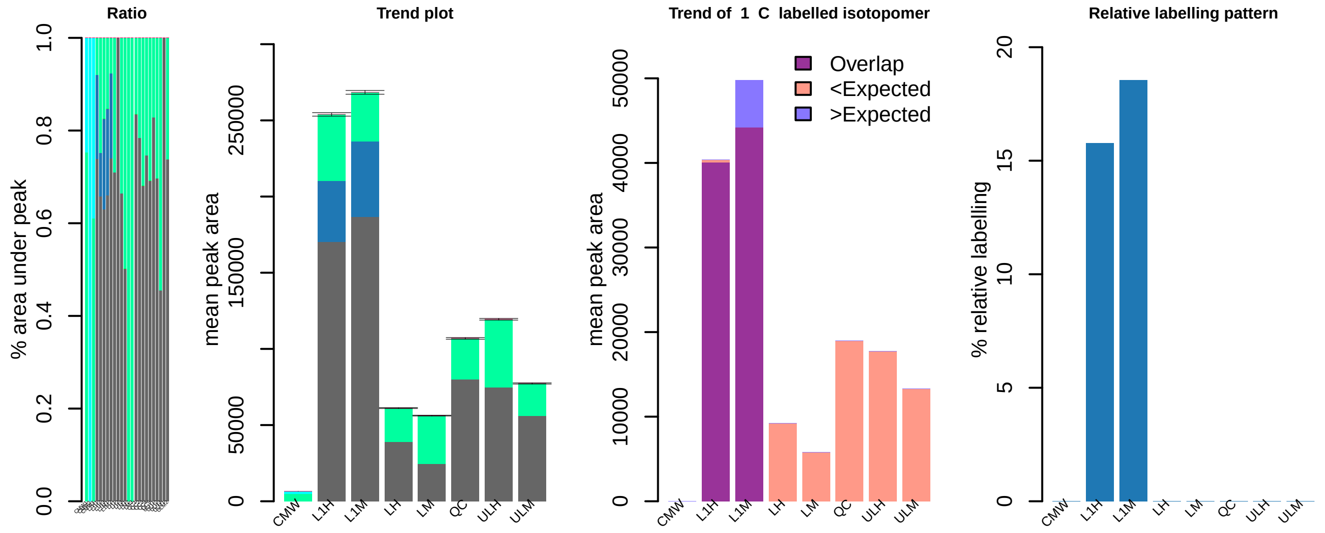

[PC (15:0)] 1−pentadecanoyl−sn−glycero−3−phosphoc

Formula: C23H48NO7P Mass: 481.317 Std.RT: 262.29871548 Ion: P

G1

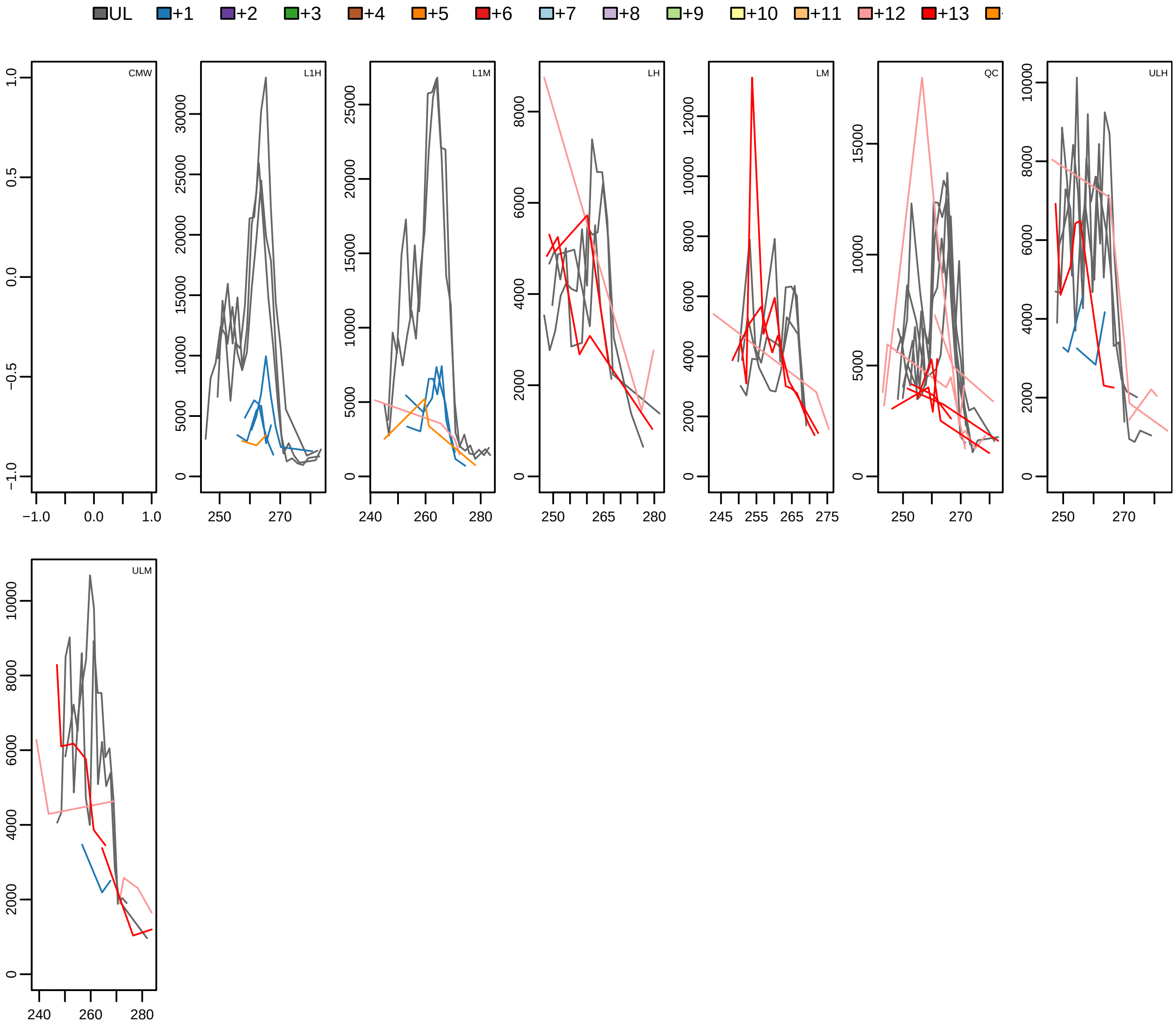

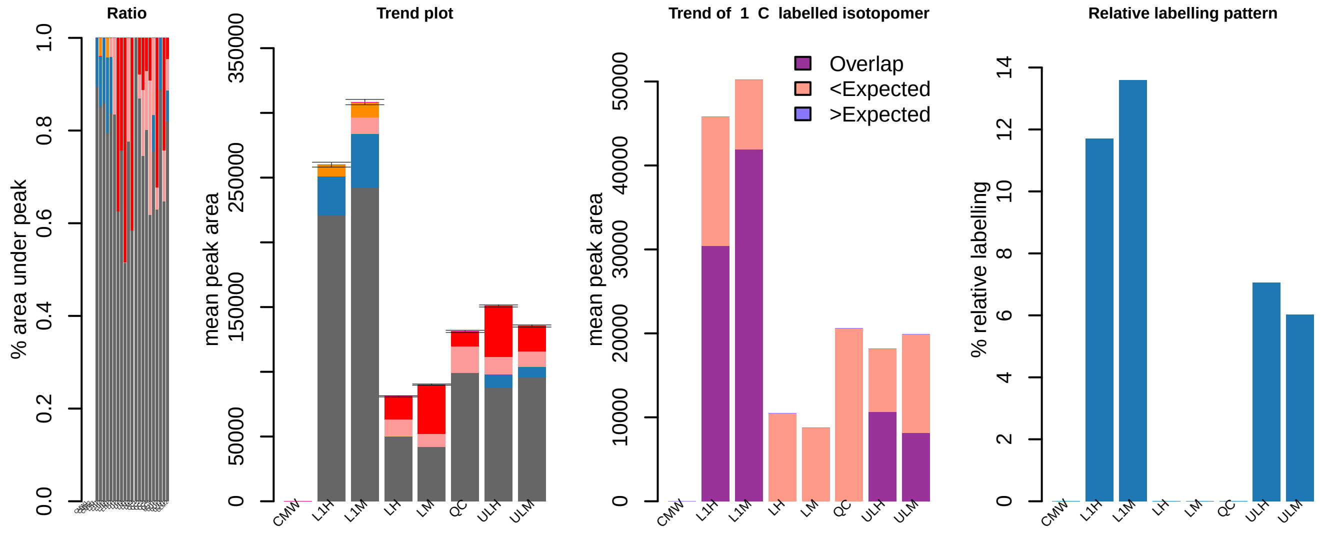

[PC (14:1)] 1−(1E−tetradecenyl)−sn−glycero−3−phosph

Formula: C22H46NO6P Mass: 451.306 Std.RT: 262.7313507 Ion: PC

G1

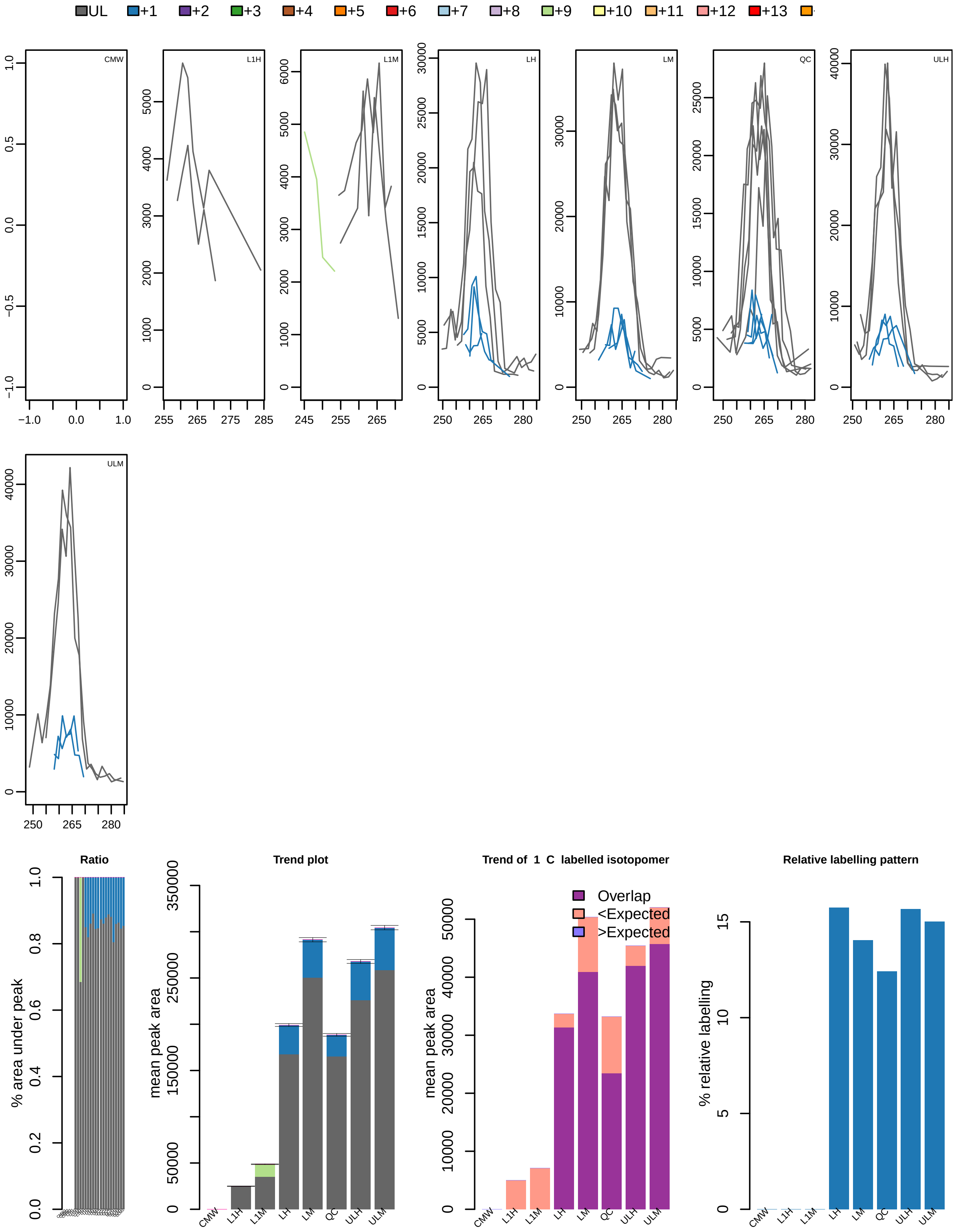

[PC (18:2/22:6)] 1−(9Z,12Z−octadecadienoyl)−2−(4Z,7Z

Formula: C48H80NO8P Mass: 829.562 Std.RT: 227.69966418 Ion: P

G1

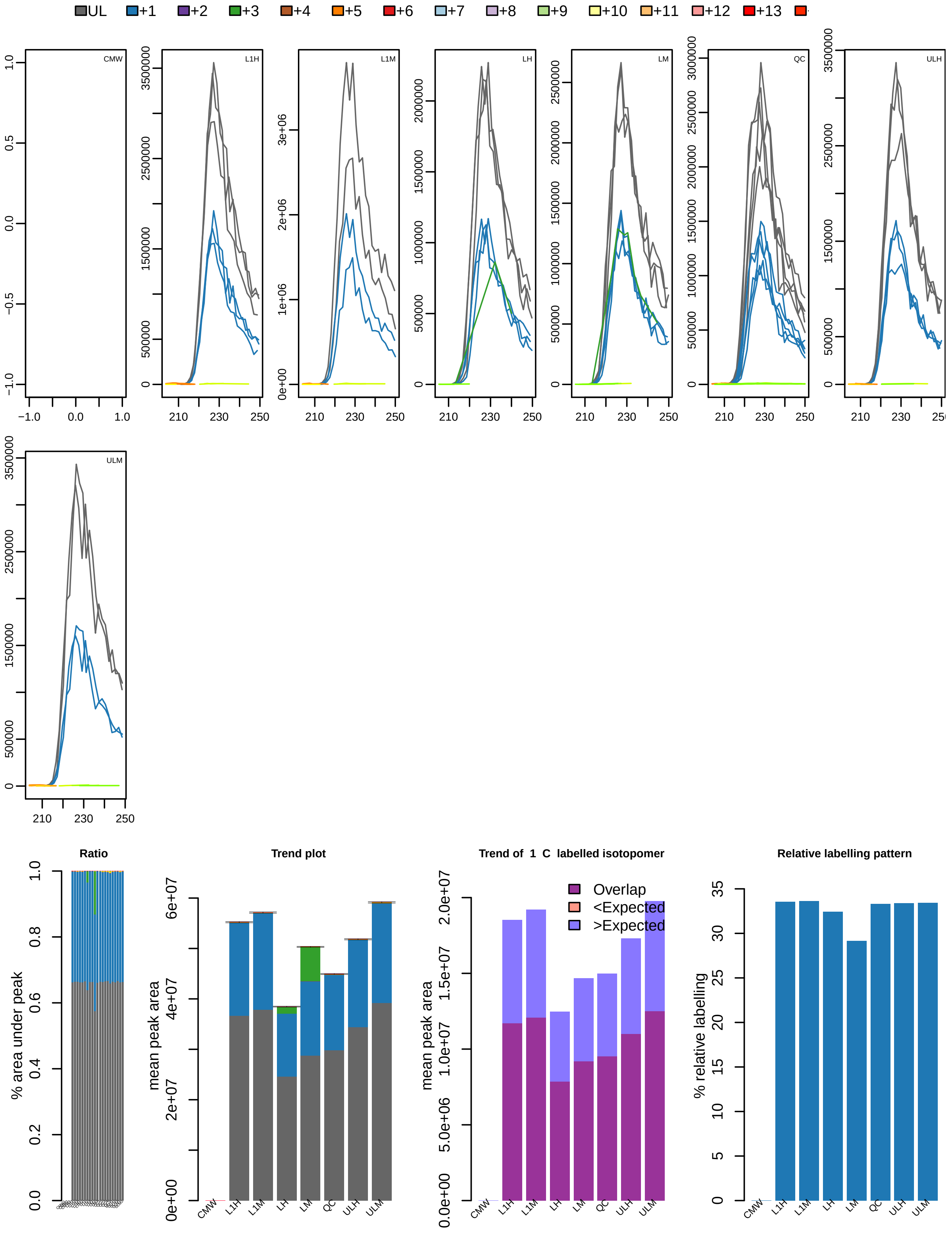

[PC (16:1)] 1−(1Z−hexadecenyl)−sn−glycero−3−phospl

Formula: C24H50NO6P Mass: 479.338 Std.RT: 257.4415218 Ion: PC

G1

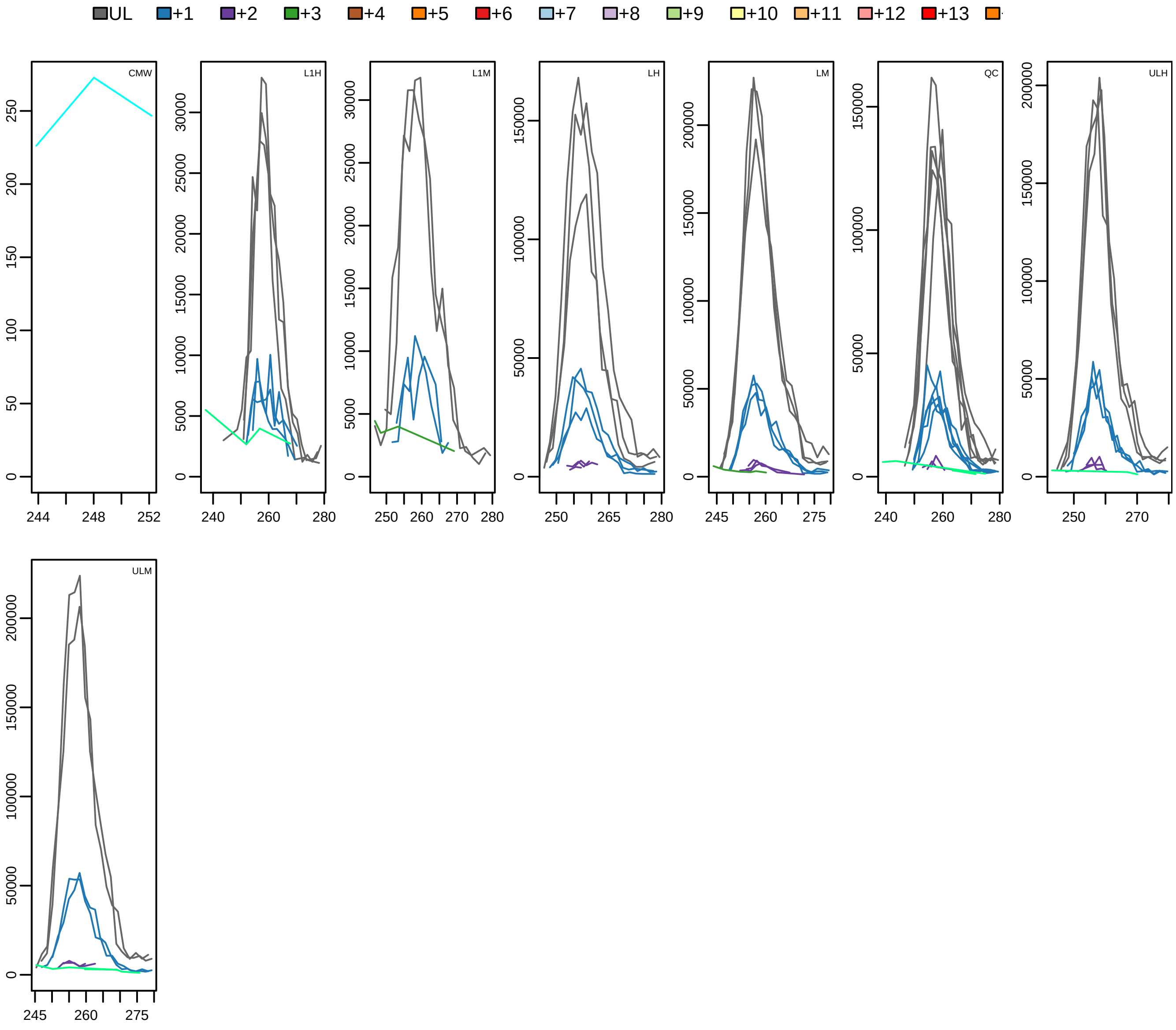

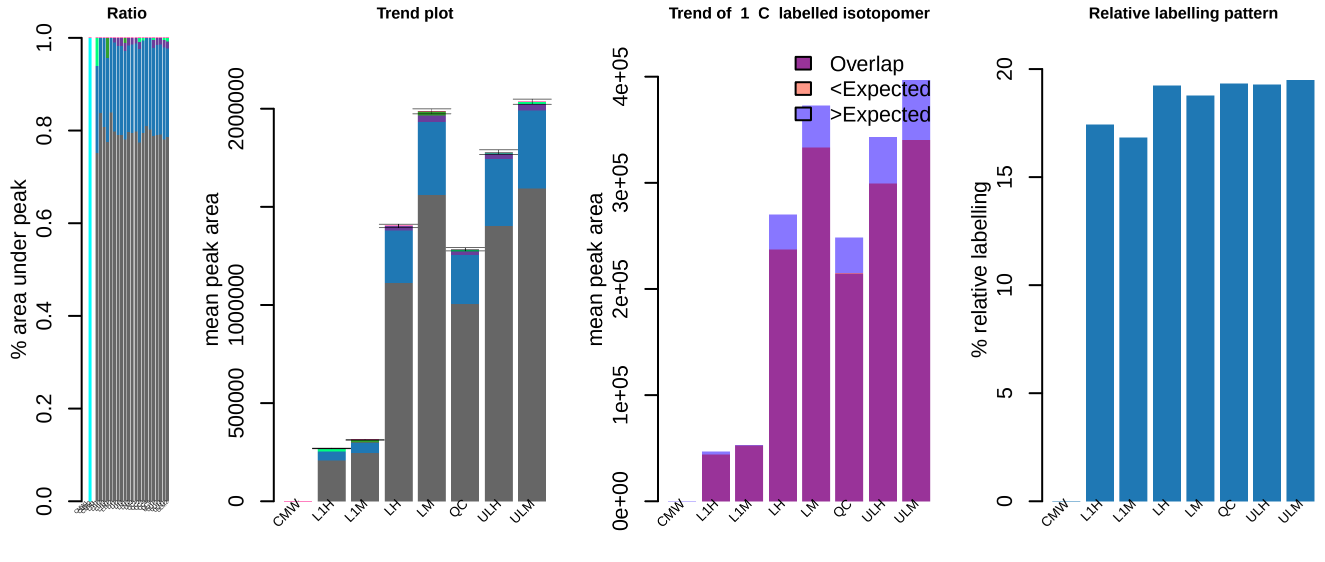

[PC (18:1)] 1−(11Z−octadecenyl)−sn−glycero−3−phosp

Formula: C26H54NO6P Mass: 507.369 Std.RT: 253.11009582 Ion: P

G1

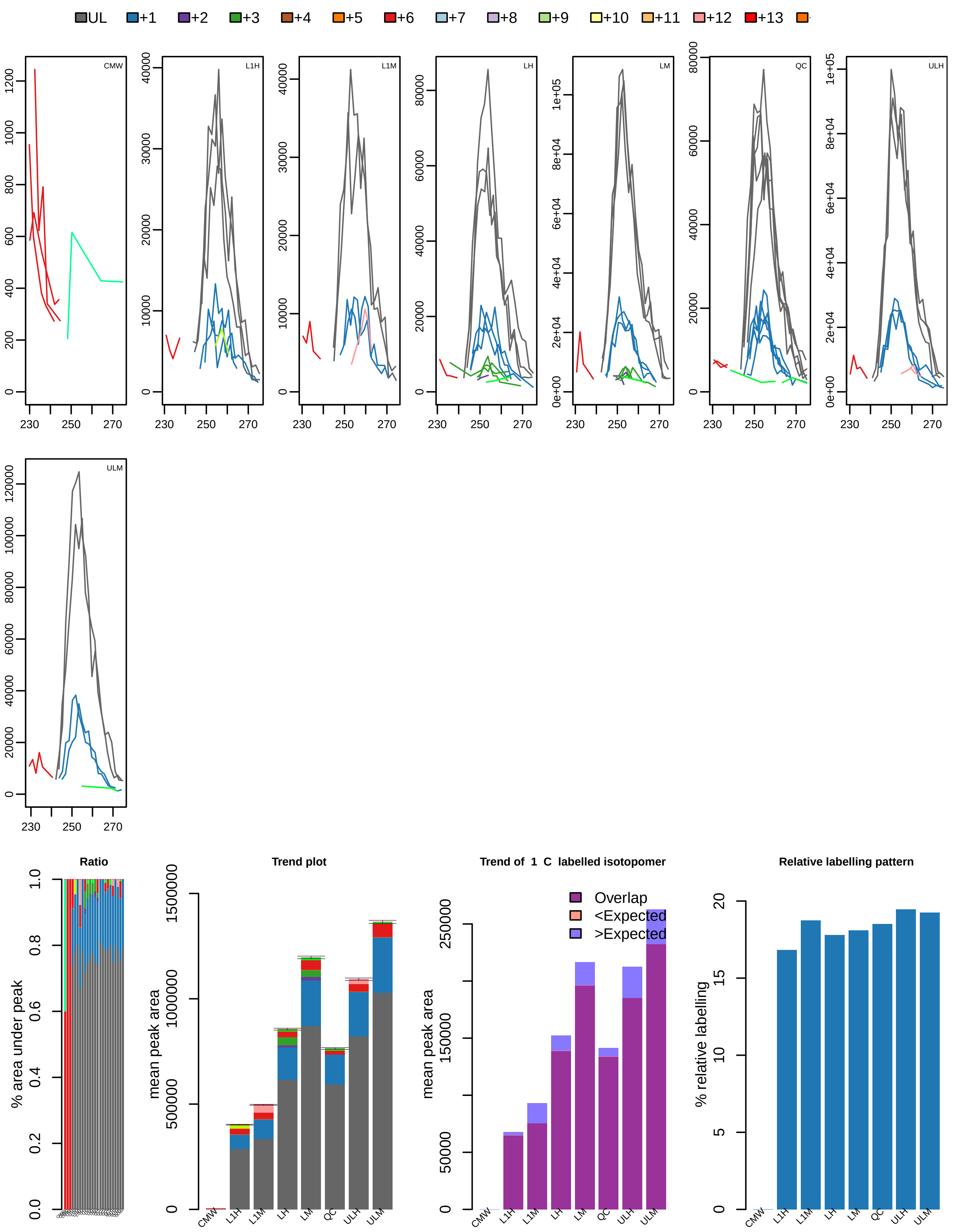

[PC (18:0)] 1−octadecanoyl−sn−glycero−3−phosphoch

Formula: C26H54NO7P Mass: 523.364 Std.RT: 255.66683202 Ion: P

G1

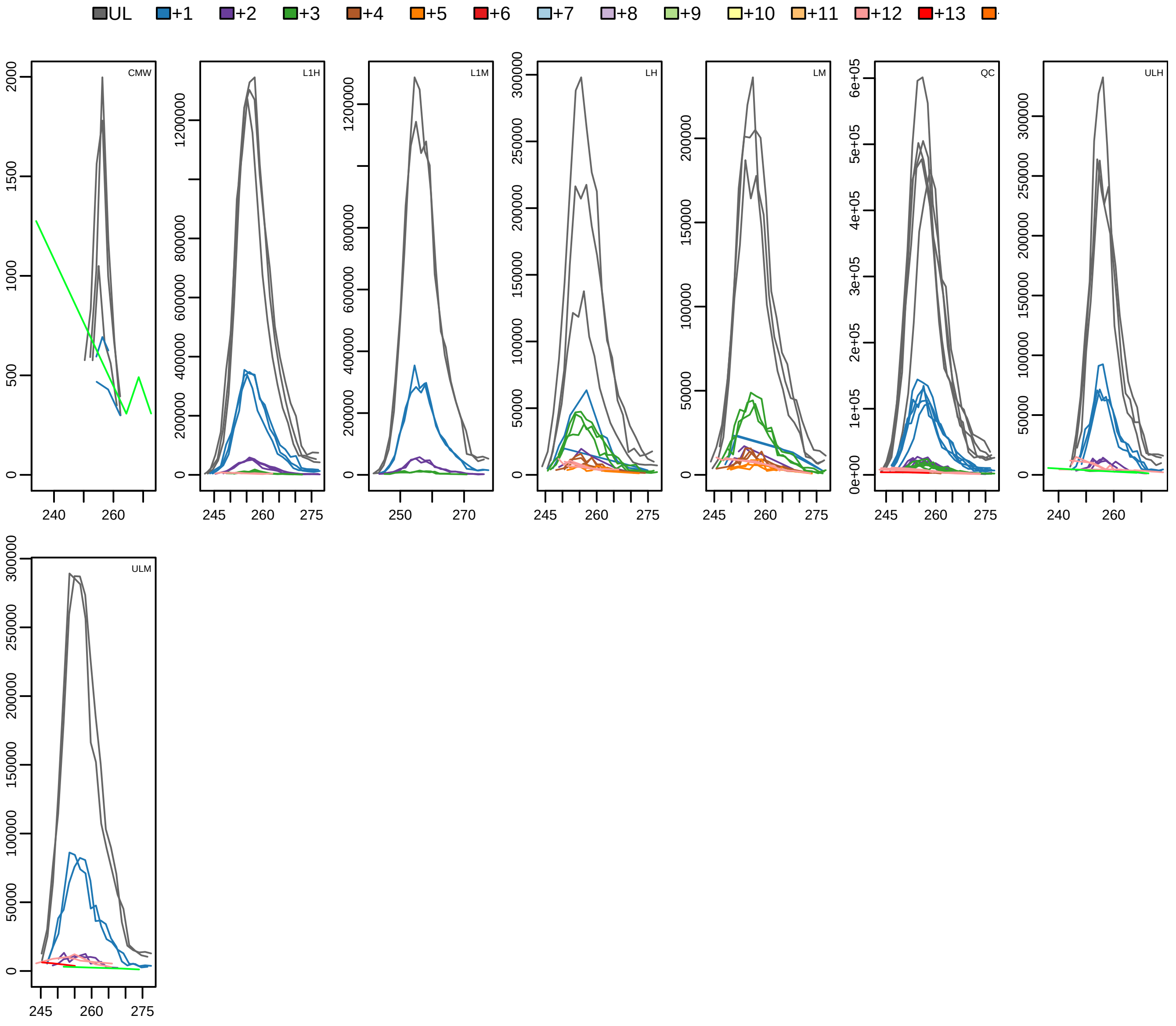

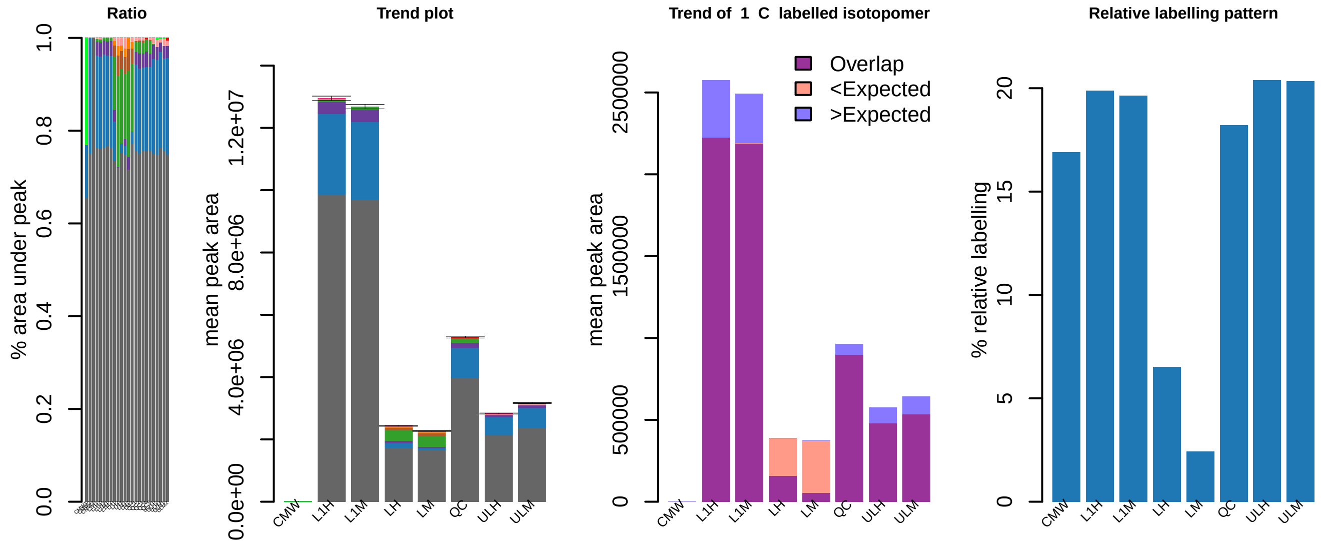

[PC (18:2)] 1−octadecyl−2−(1E−ethenyl)−sn−glycero−3

Formula: C28H58NO6P Mass: 535.4 Std.RT: 249.6475242 Ion: POS

G1

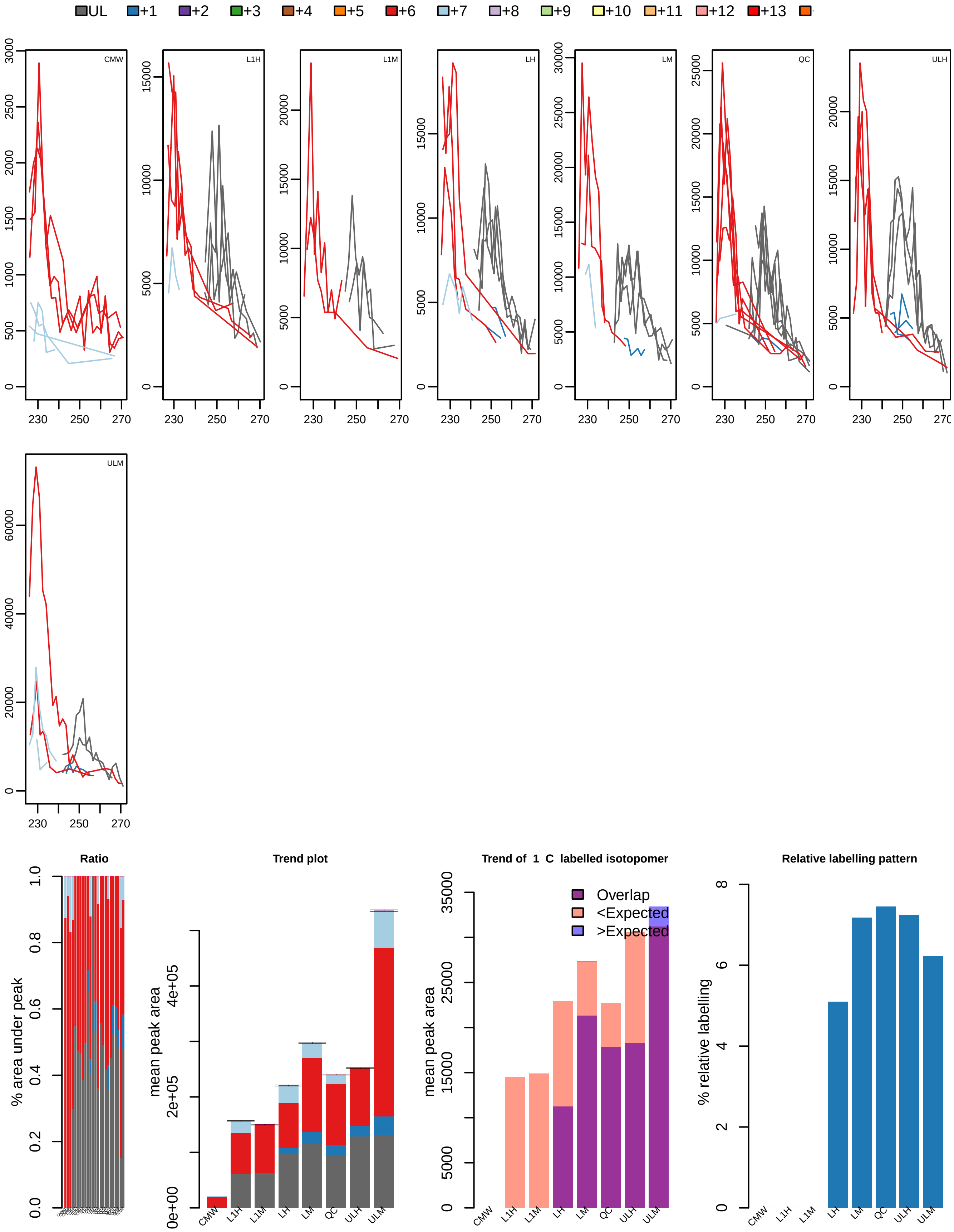

[PC (20:0)] 1−eicosanoyl−sn−glycero−3−phosphocholir

Formula: C28H58NO7P Mass: 551.395 Std.RT: 252.7927002 Ion: PC

G1

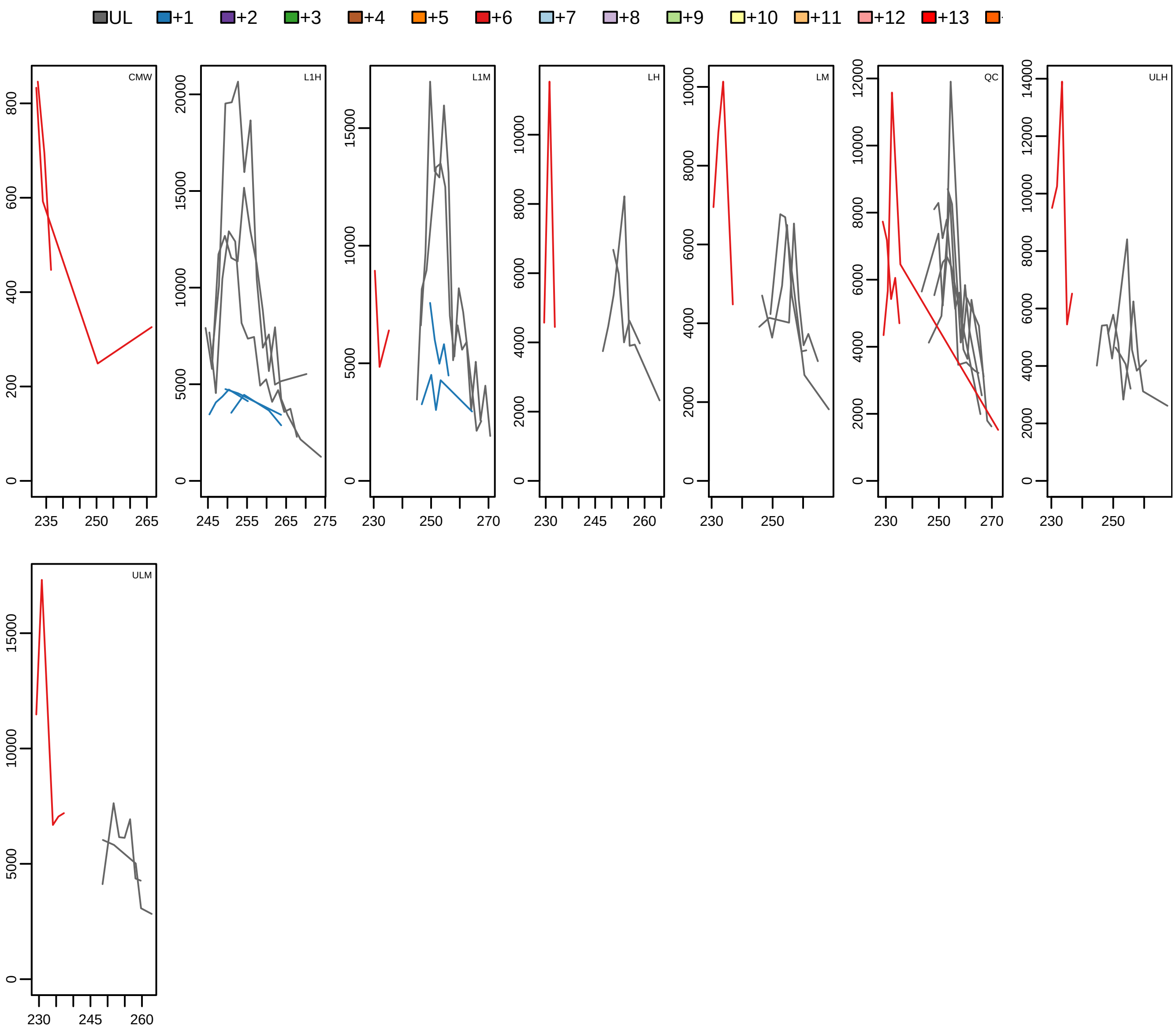

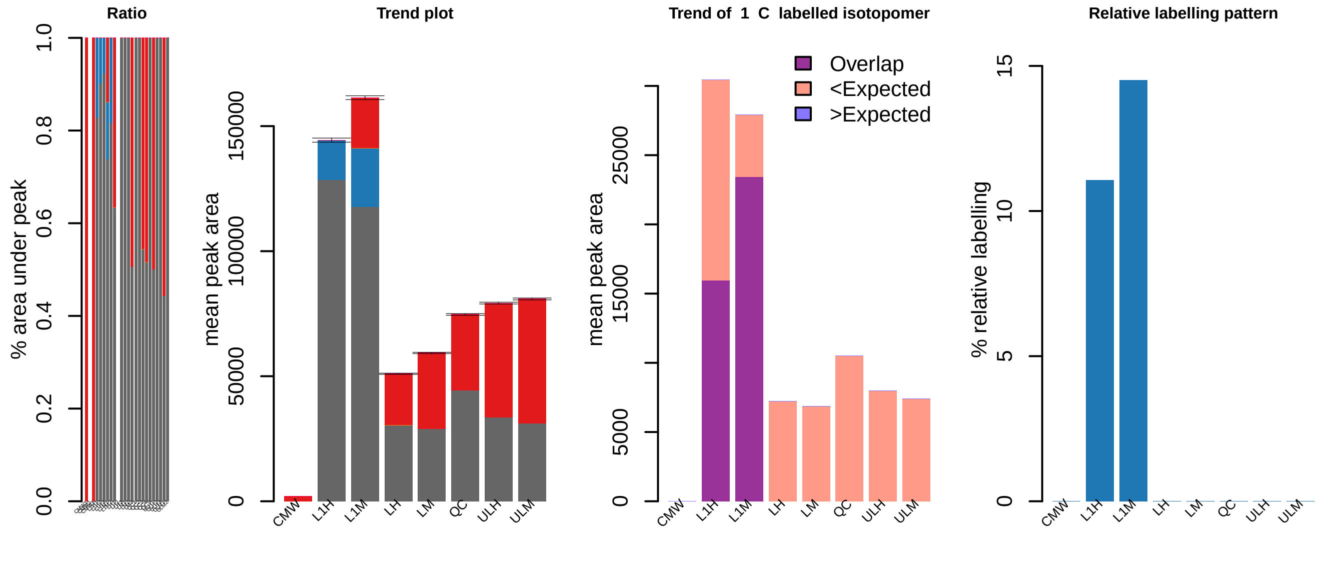

LysoPC(20:3(5Z,8Z,11Z))

Formula: C28H52NO7P Mass: 545.348 Std.RT: 255.02409798 Ion: P

G1

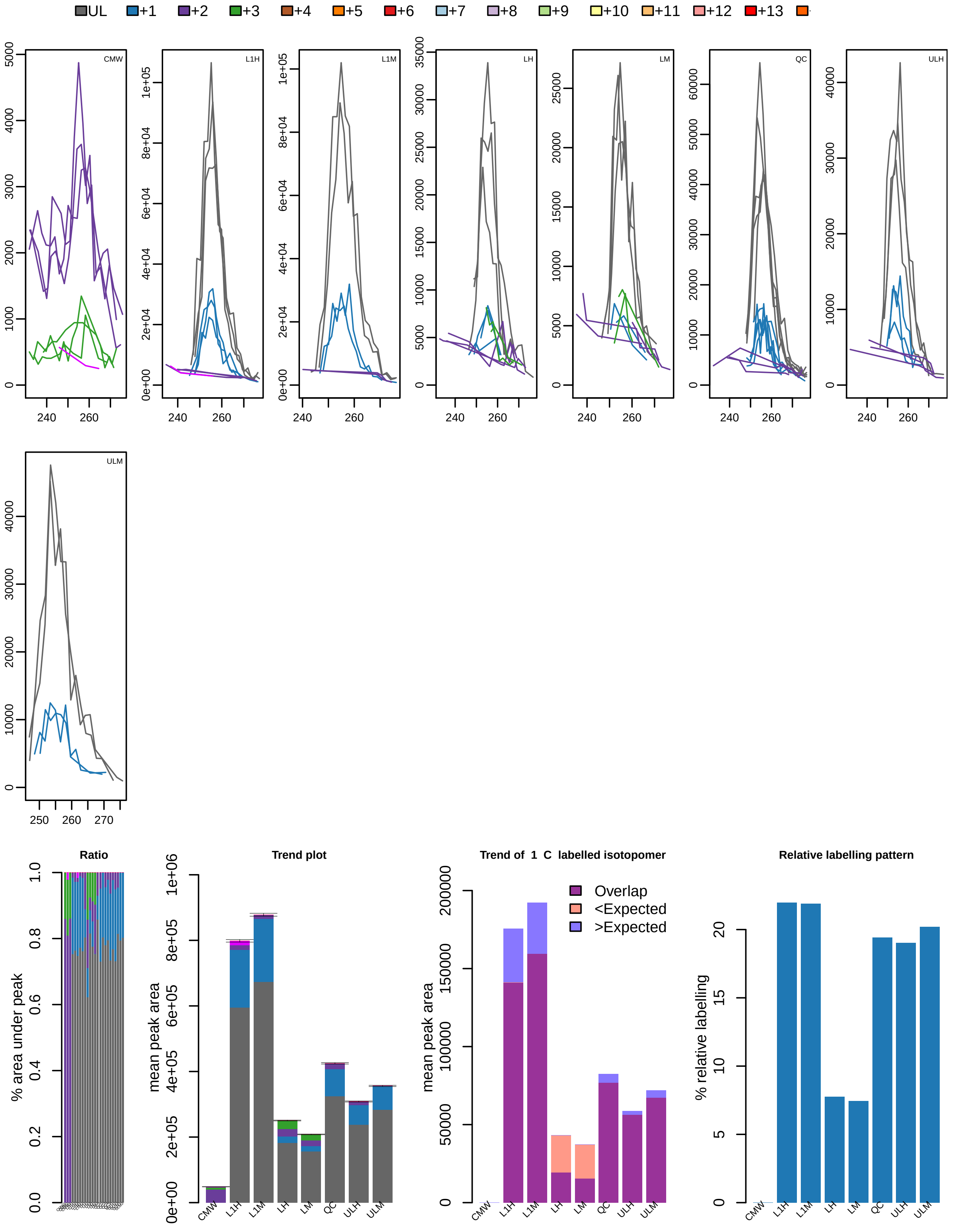

LysoPC(22:5(4Z,7Z,10Z,13Z,16Z))

Formula: C30H52NO7P Mass: 569.348 Std.RT: 252.5845722 Ion: PC

G1

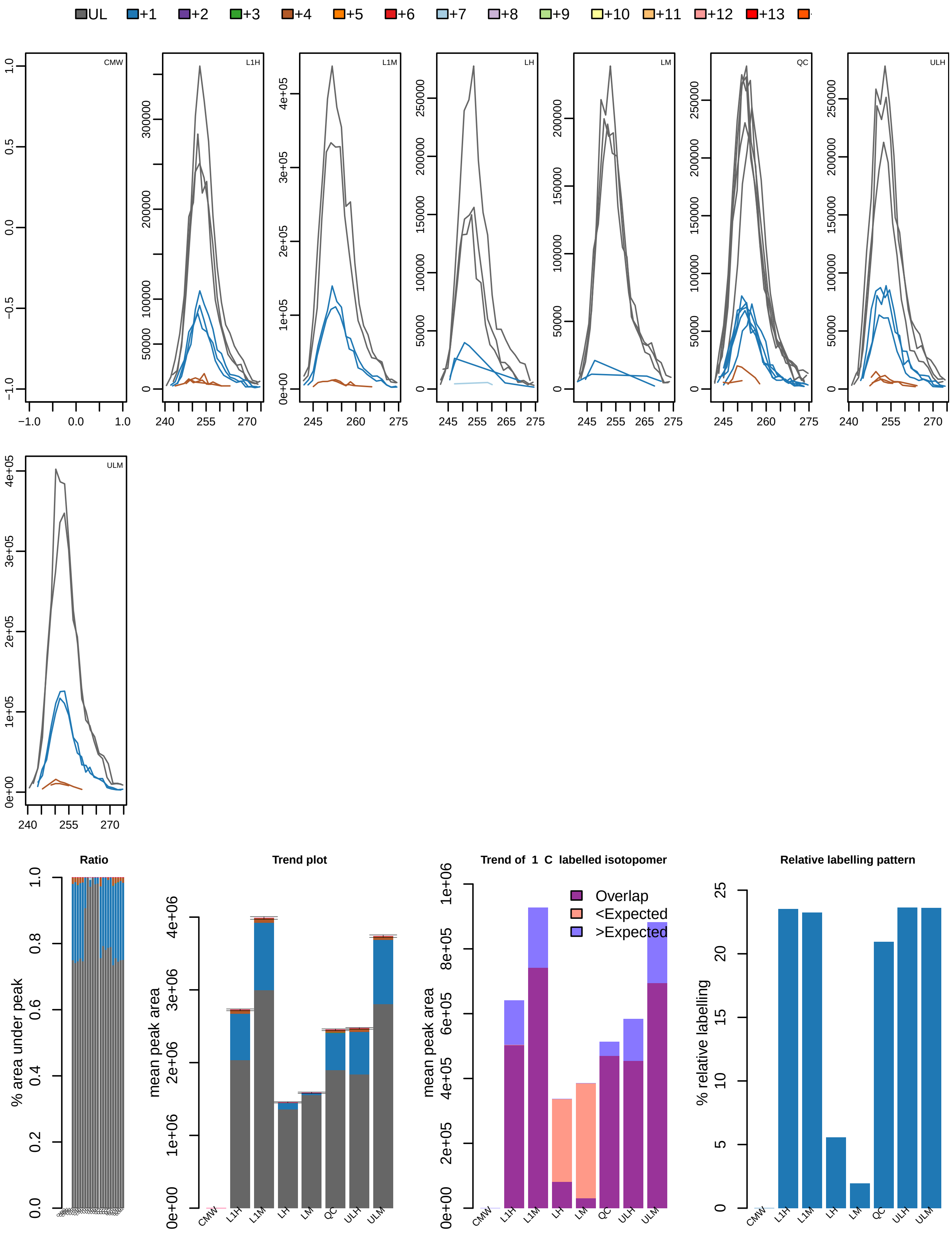

[PC (16:0)] 1−hexadecanoyl−sn−glycero−3−phosphoch

Formula: C24H50NO7P Mass: 495.332 Std.RT: 260.63891052 Ion: P

G1

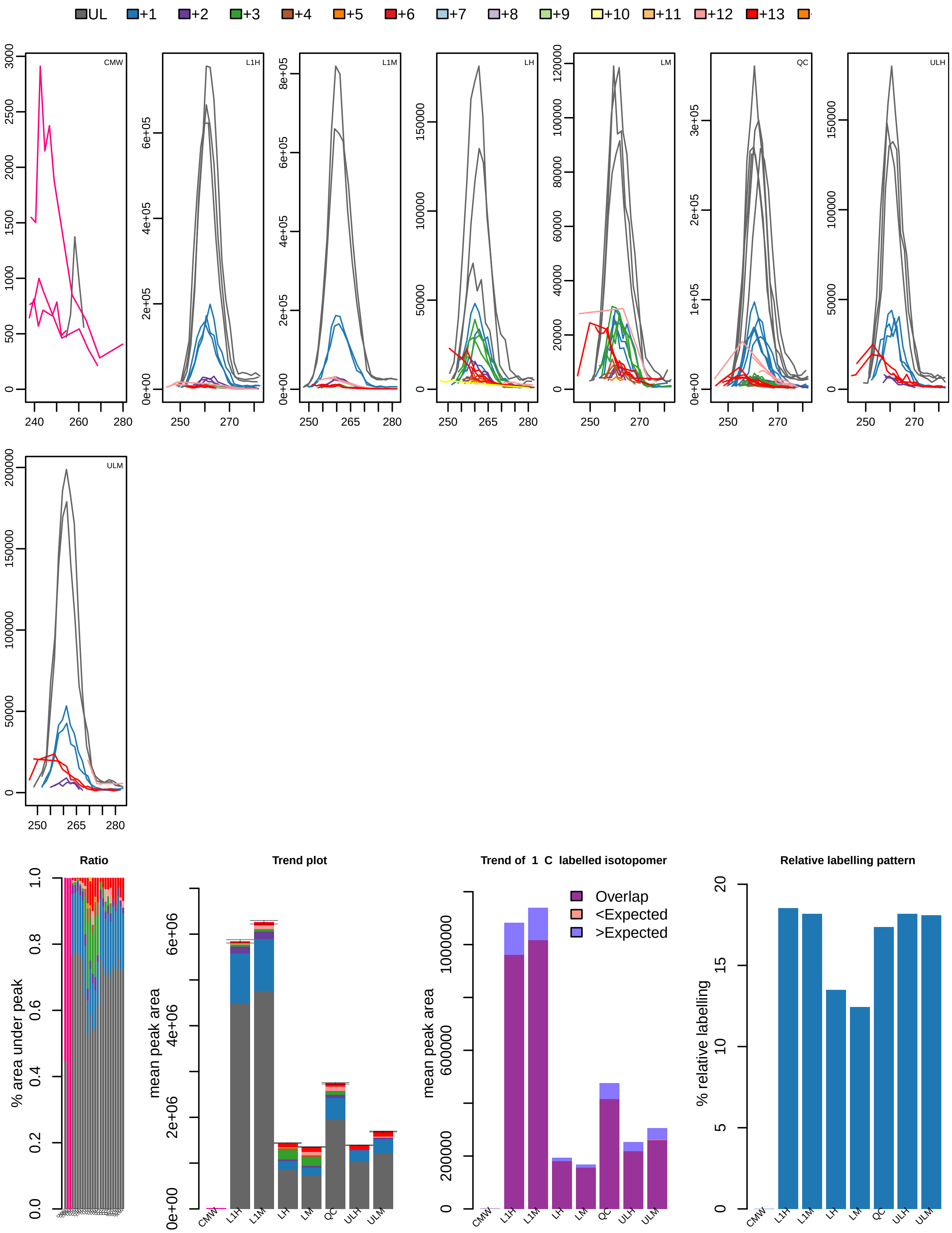

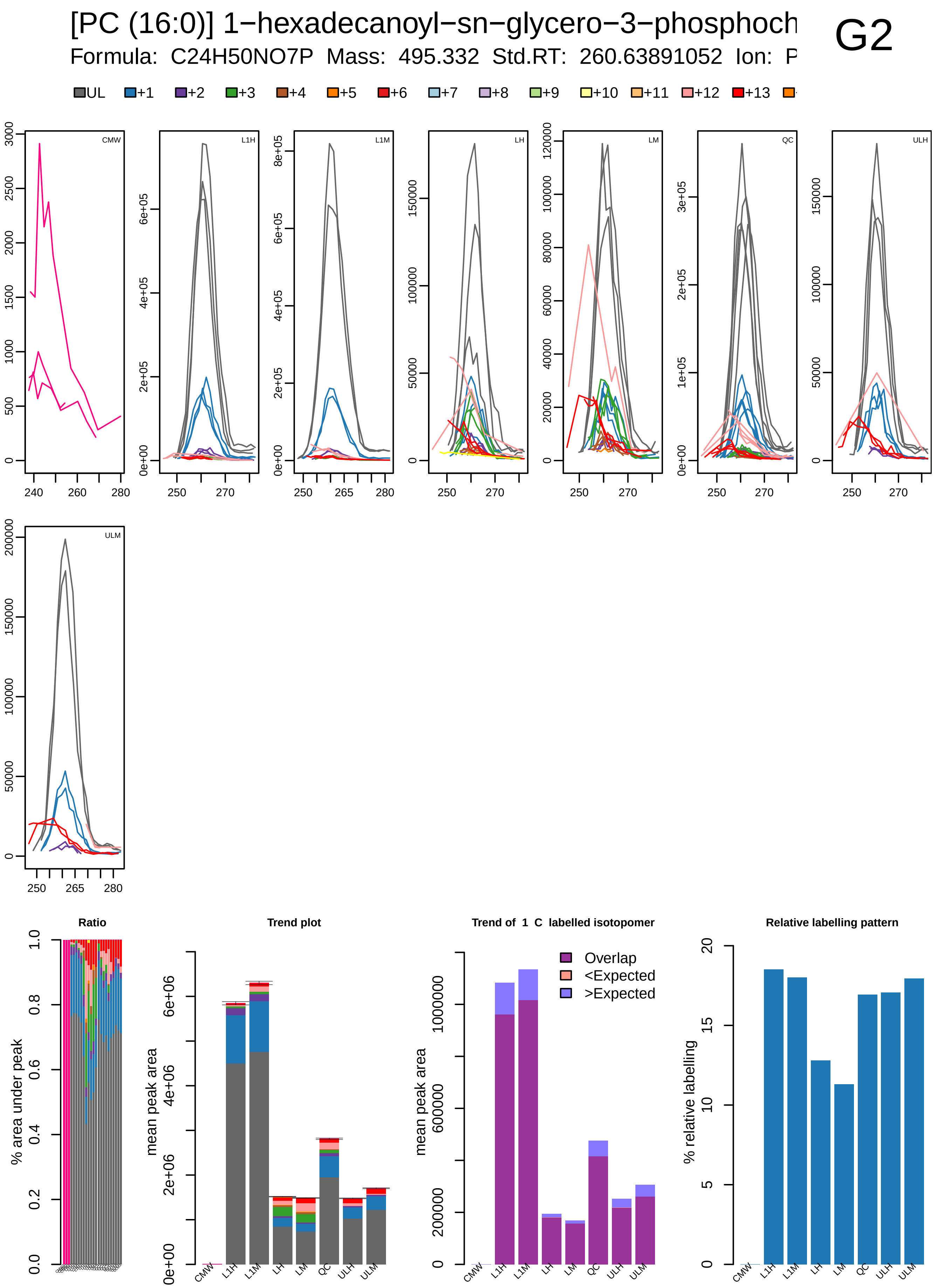
[PC (16:0)] 1−hexadecanoyl−sn−glycero−3−phosphoch
Formula: C24H50NO7P Mass: 495.332 Std.RT: 260.63891052 Ion: P
G2
UL +1 +2 +3 +4 +5 +6 +7 +8 +9 +10 +11 +12 +13
CMW
L1H
L1M
LH
LM
QC
ULH
ULM
Ratio
% area under peak
Trend plot
mean peak area
Trend of 1 C labelled isotopomer
Overlap
<Expected
>Expected
Relative labelling pattern
% relative labelling

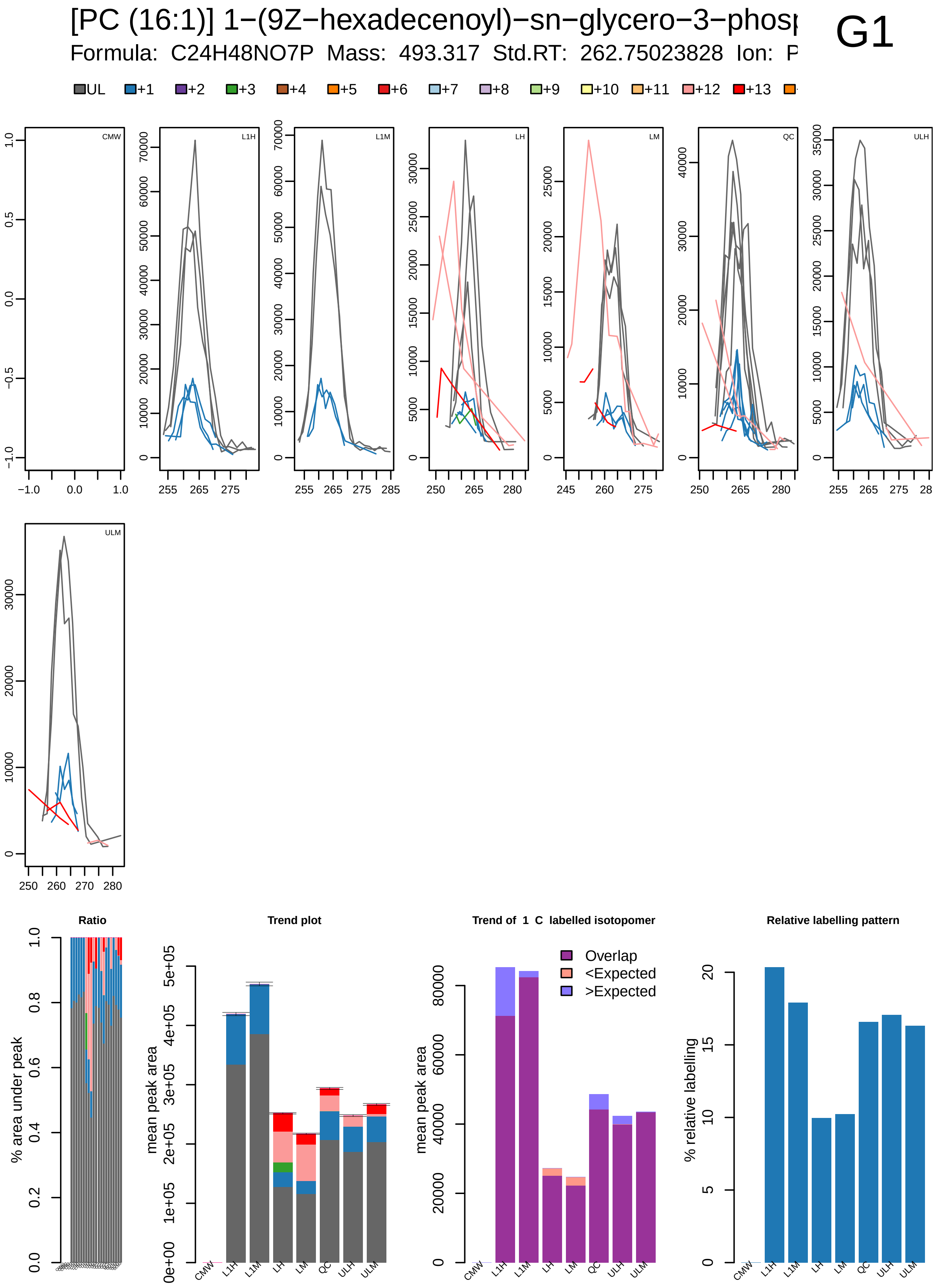
[PC (16:1)] 1−(9Z−hexadecenoyl)−sn−glycero−3−phosp
Formula: C24H48NO7P Mass: 493.317 Std.RT: 262.75023828 Ion: P
G1
UL +1 +2 +3 +4 +5 +6 +7 +8 +9 +10 +11 +12 +13
CMW
L1H
L1M
LH
LM
QC
ULH
ULM
Ratio
% area under peak
Trend plot
mean peak area
Trend of 1 C labelled isotopomer
Overlap
<Expected
>Expected
Relative labelling pattern
% relative labelling

[PC (16:2)] 1−hexadecyl−sn−glycero−3−phosphocholin

Formula: C24H52NO6P Mass: 481.353 Std.RT: 263.568714 Ion: PO[illegible]

G1

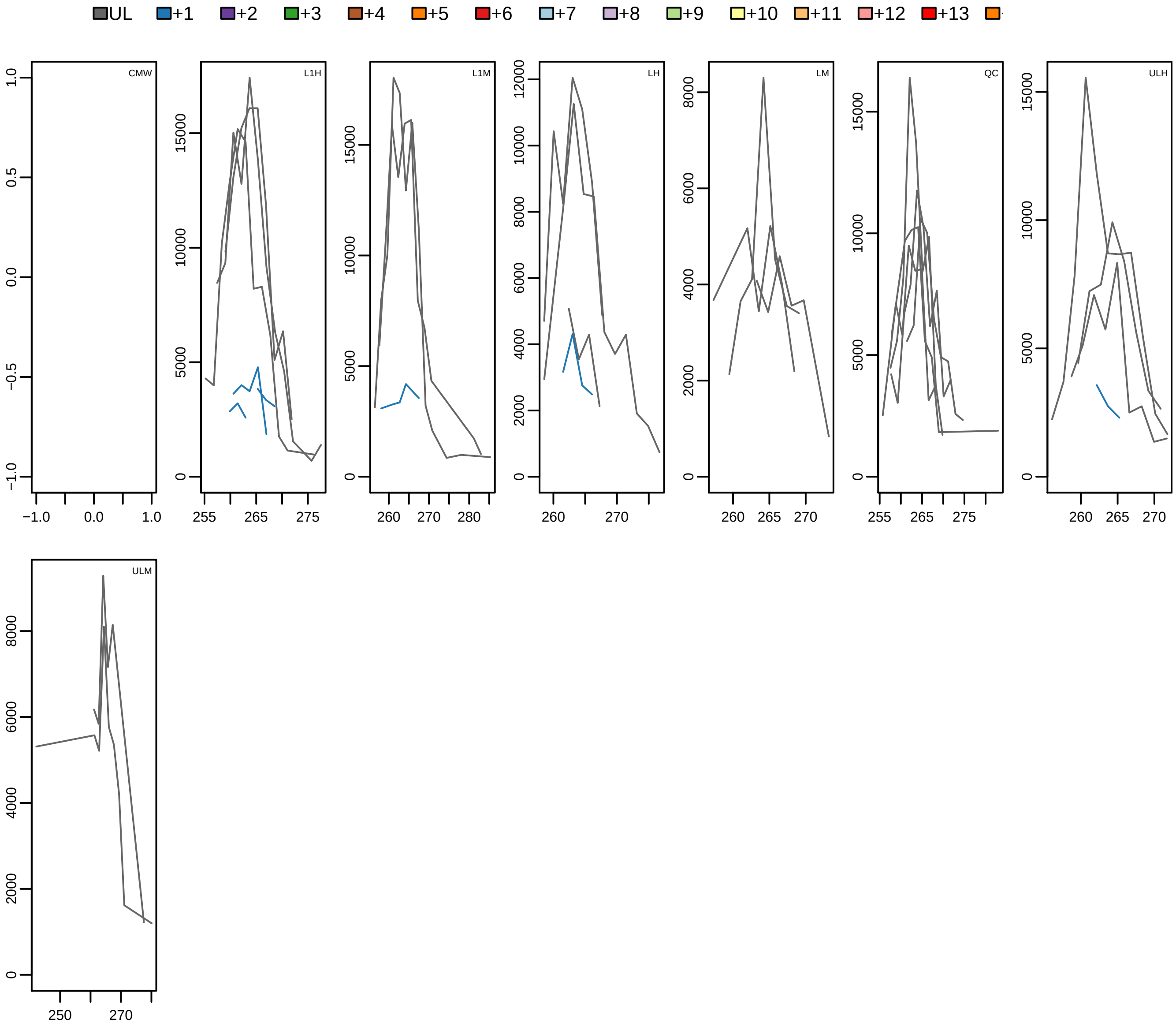

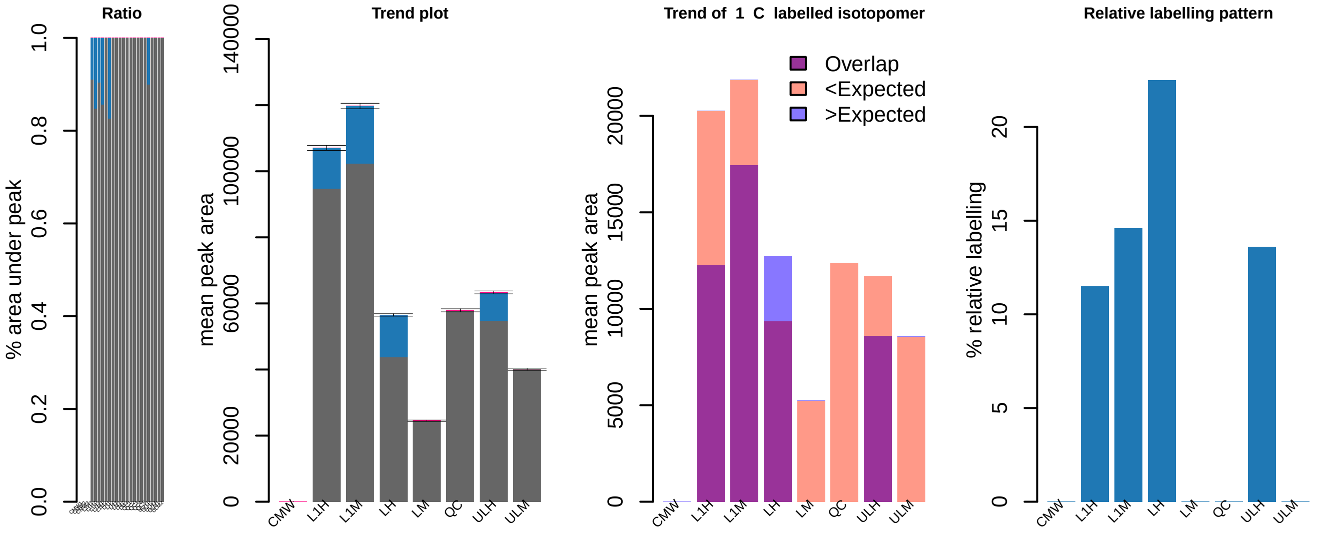

[PC (22:6)] 1−(4Z,7Z,10Z,13Z,16Z,19Z−docosahexaen

Formula: C30H50NO7P Mass: 567.332 Std.RT: 253.3487142 Ion: PC

G1

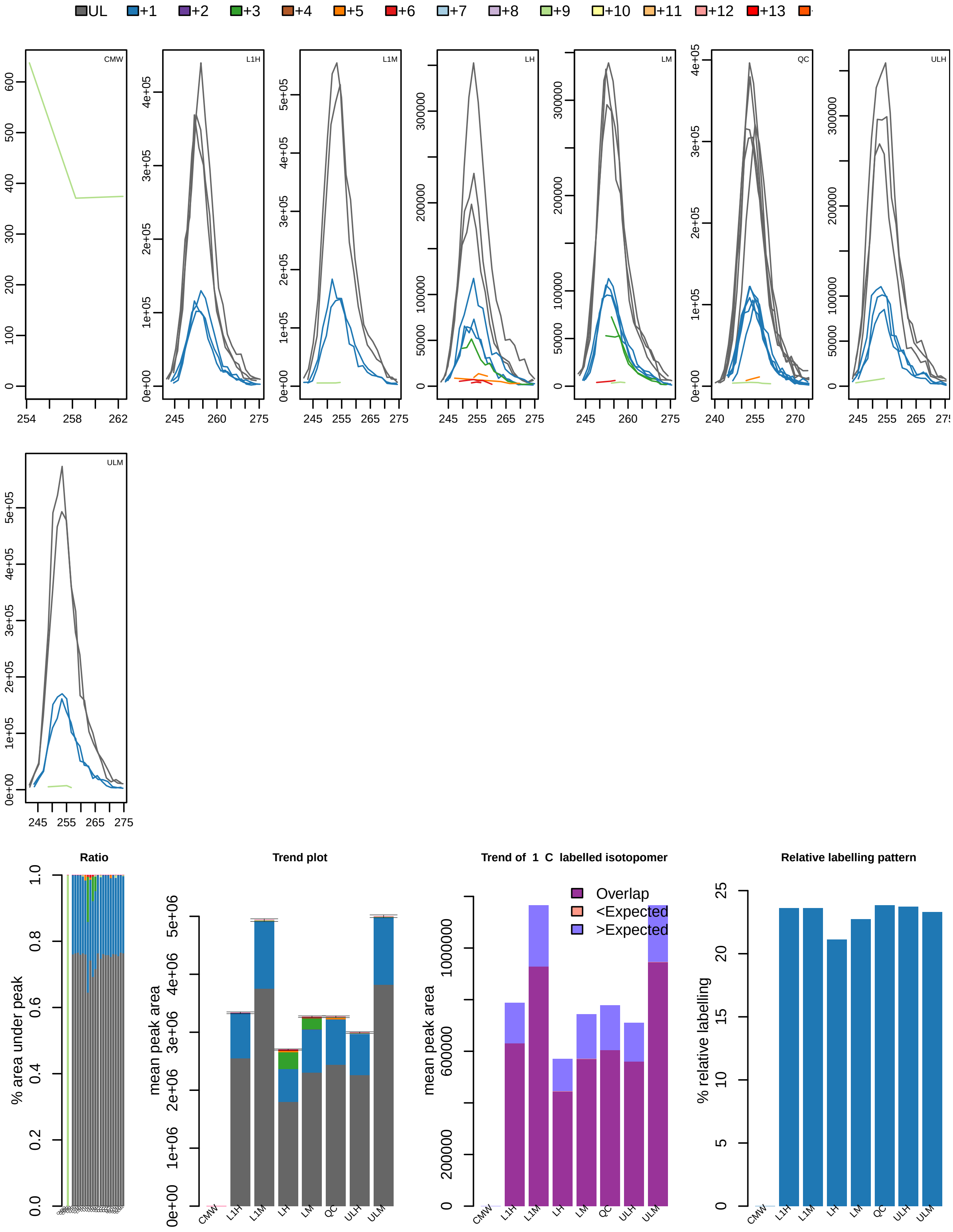

PC(18:2(9Z,12Z)/P−18:1(11Z))

Formula: C44H82NO7P Mass: 767.583 Std.RT: 309.82790412 Ion: P

G1

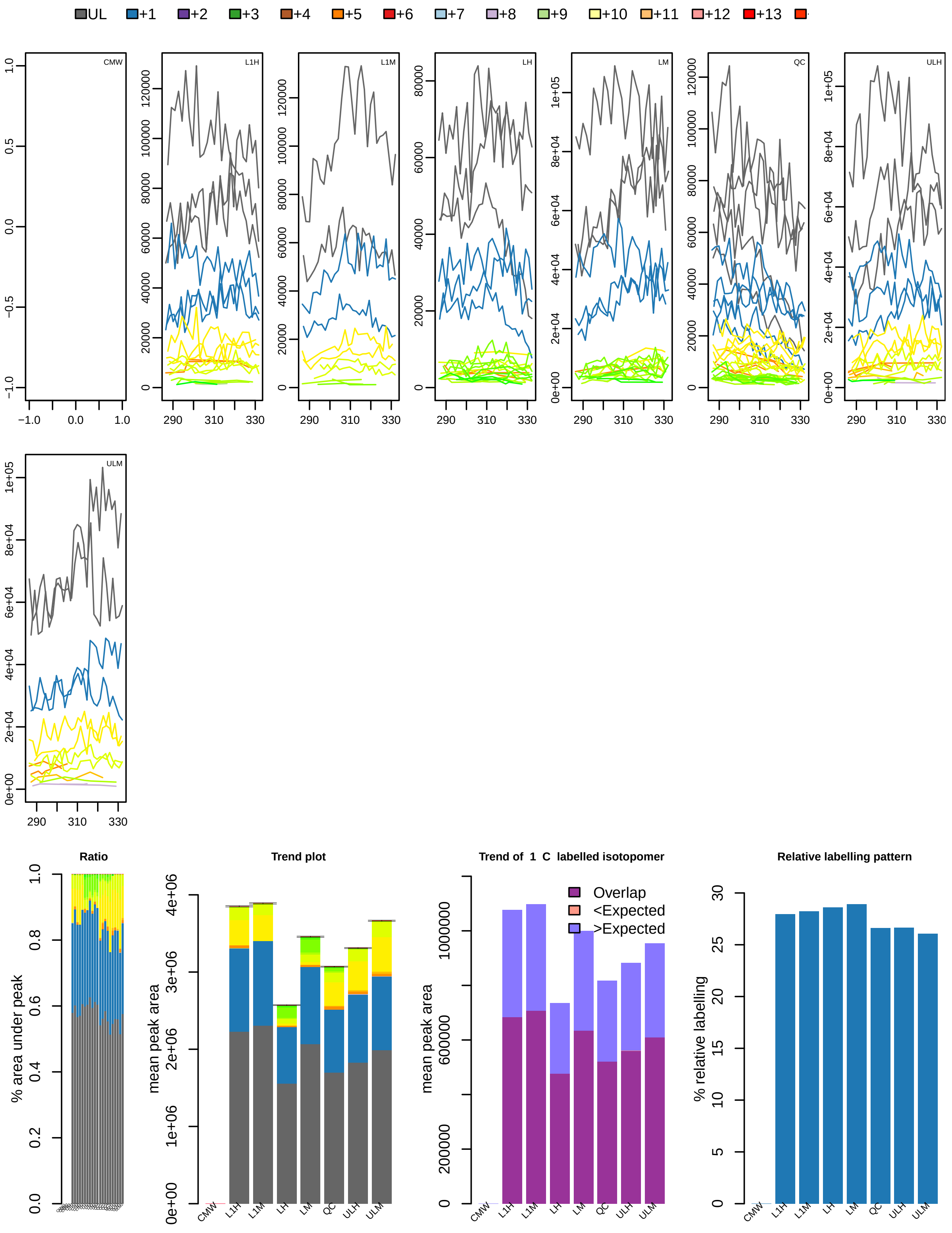

PC(14:0/P−18:1(11Z))

Formula: C40H78NO7P Mass: 715.552 Std.RT: 234.0737145 Ion: PC

G1

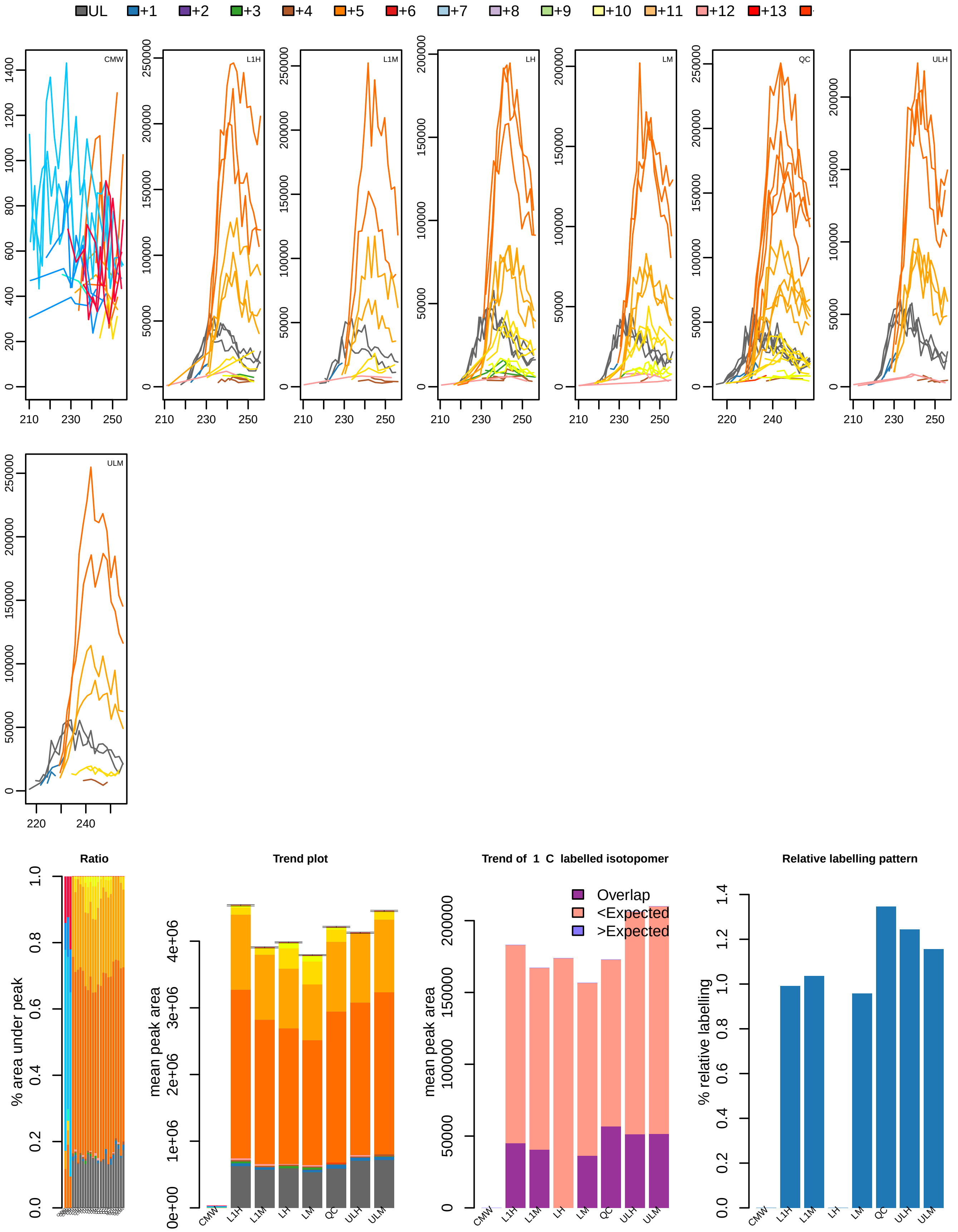

[PC (12:2/8:2)] 1−dodecyl−2−octyl−sn−glycero−3−phos

Formula: C28H60NO6P Mass: 537.416 Std.RT: 253.13700018 Ion: P

G1

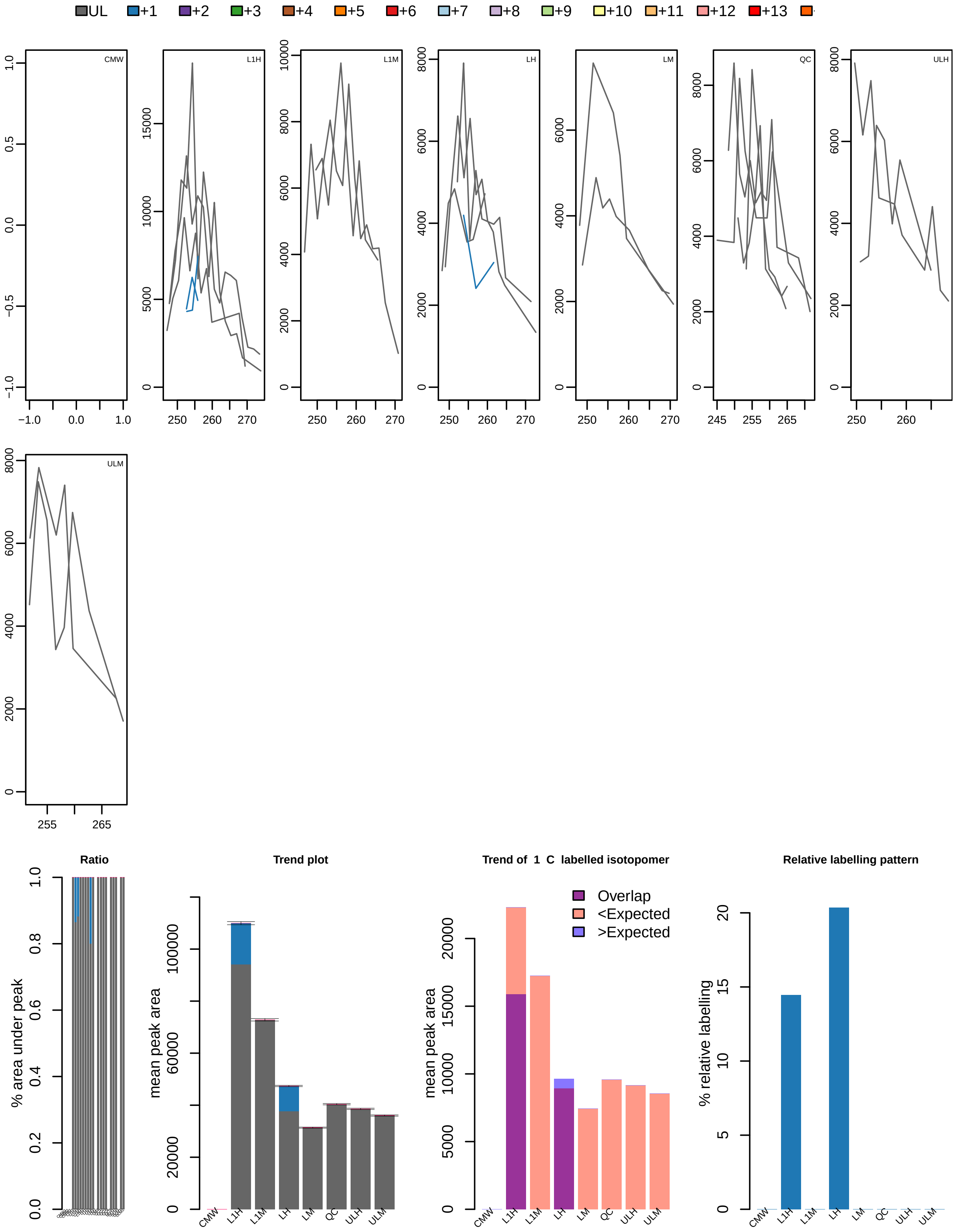

LysoPC(20:4(5Z,8Z,11Z,14Z))

Formula: C28H50NO7P Mass: 543.332 Std.RT: 255.78476322 Ion: P

G1

UL +1 +2 +3 +4 +5 +6 +7 +8 +9 +10 +11 +12 +13

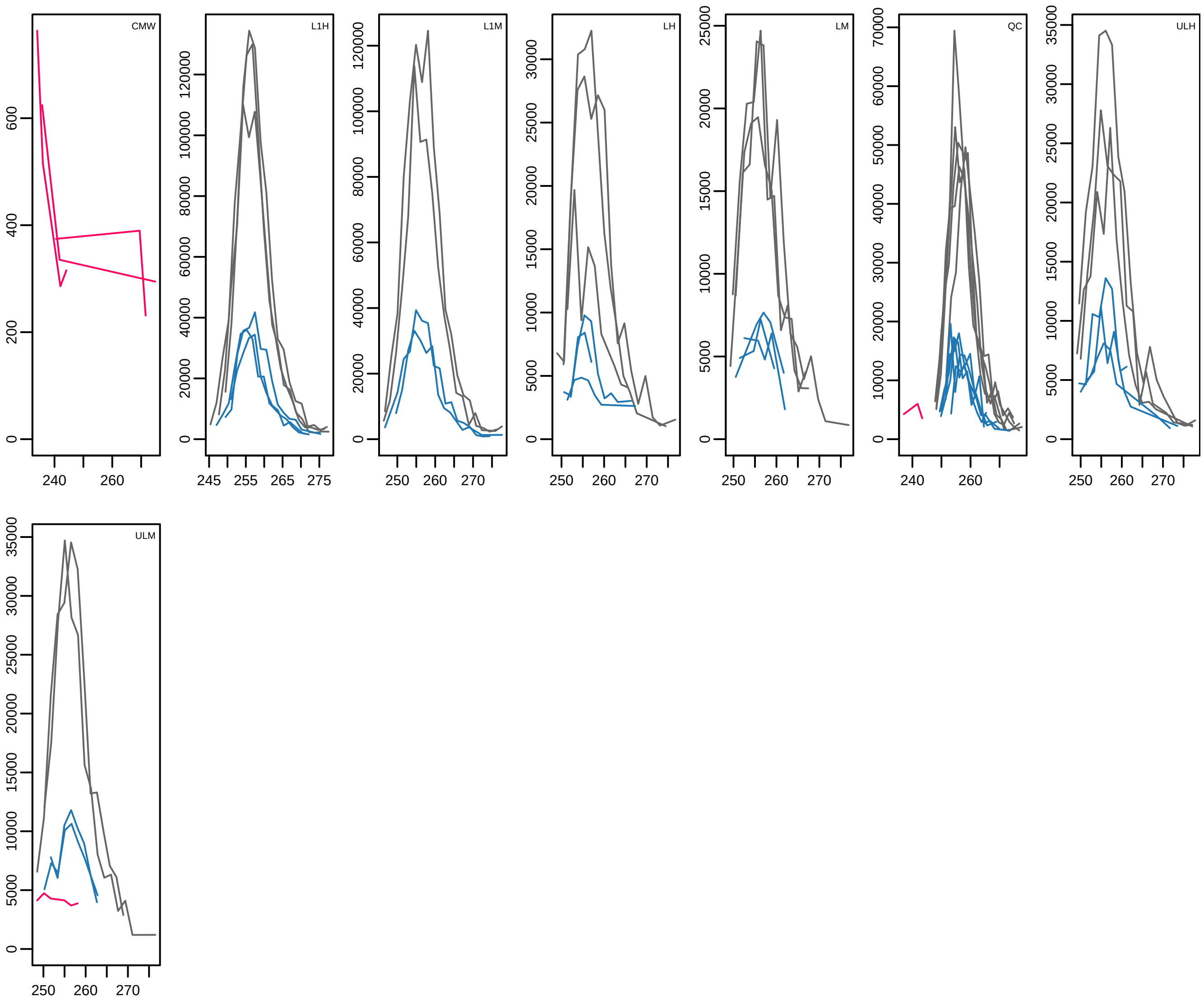

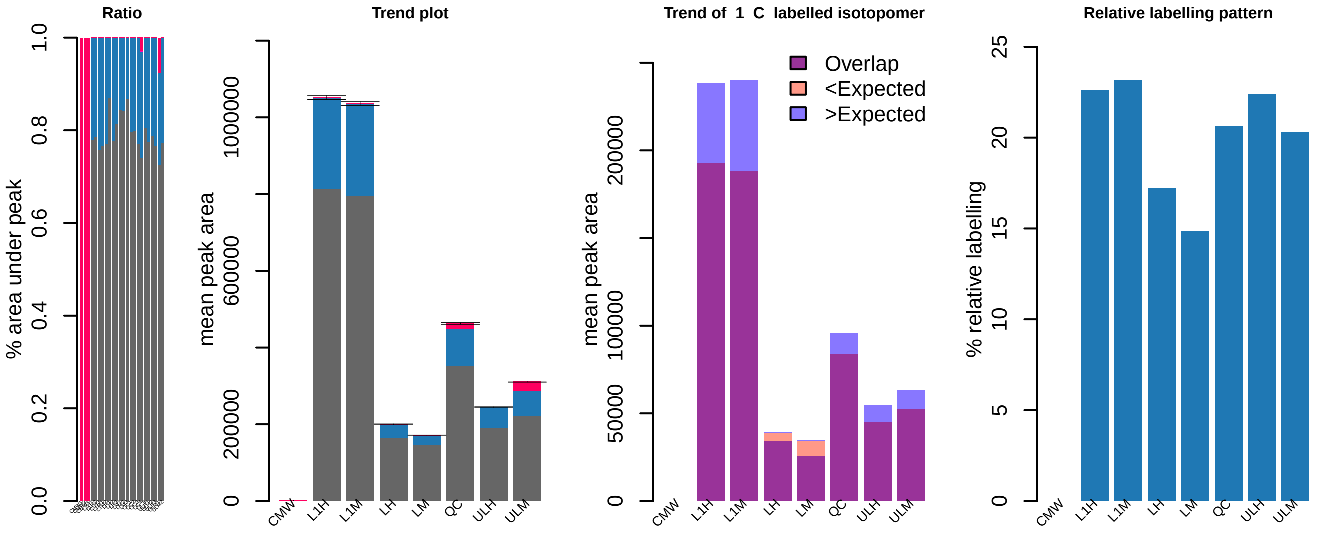

[PC (16:1/18:2)] 1−(1Z−hexadecenyl)−2−(9Z,12Z−octa[illegible]

Formula: C42H80NO7P Mass: 741.567 Std.RT: 232.3786815 Ion: PC[illegible]

G1

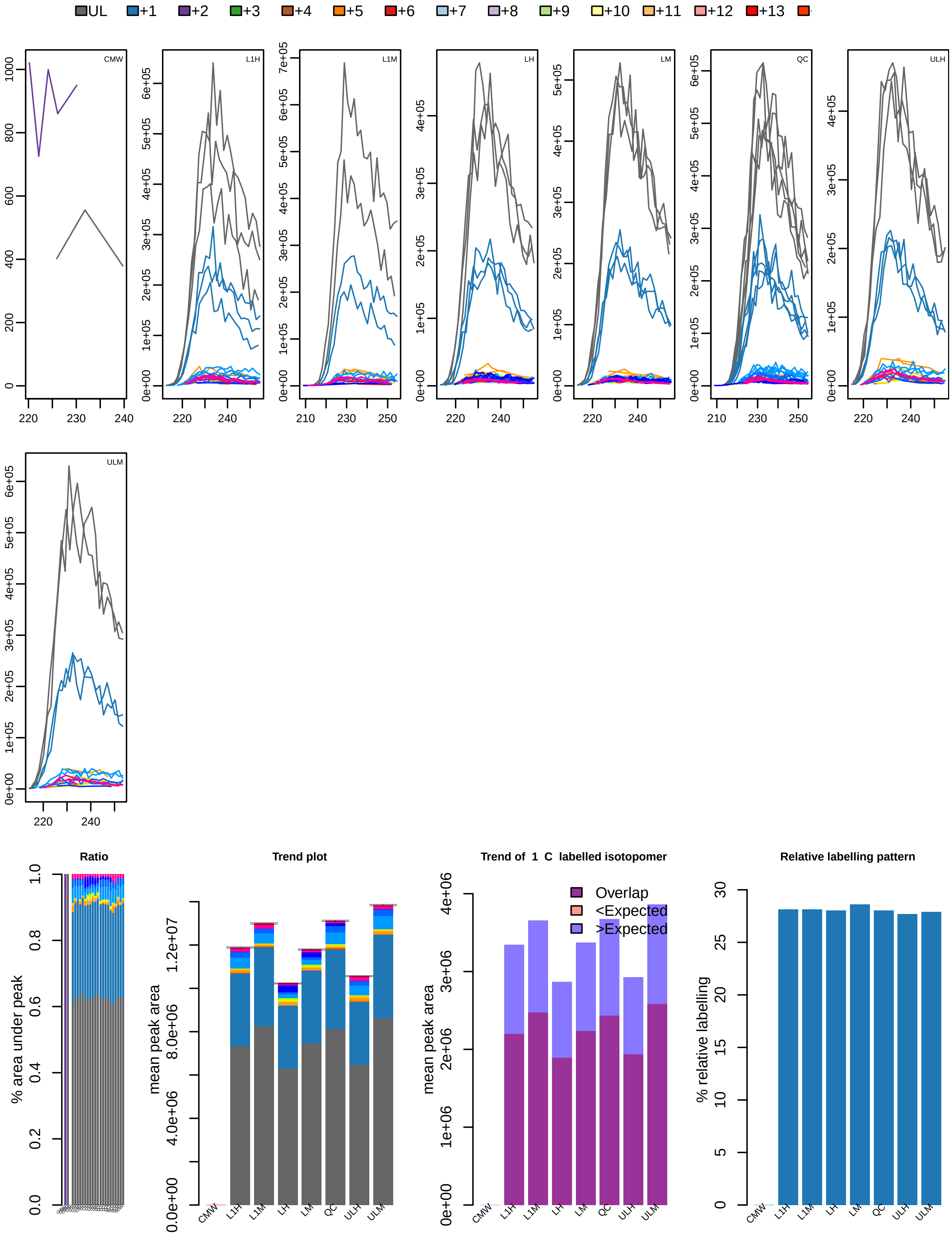

[PC methyl(16:0)] 1−methyl−2−hexadecanoyl−sn−glyce

Formula: C25H52NO7P Mass: 509.348 Std.RT: 431.80200042 Ion: P

G1

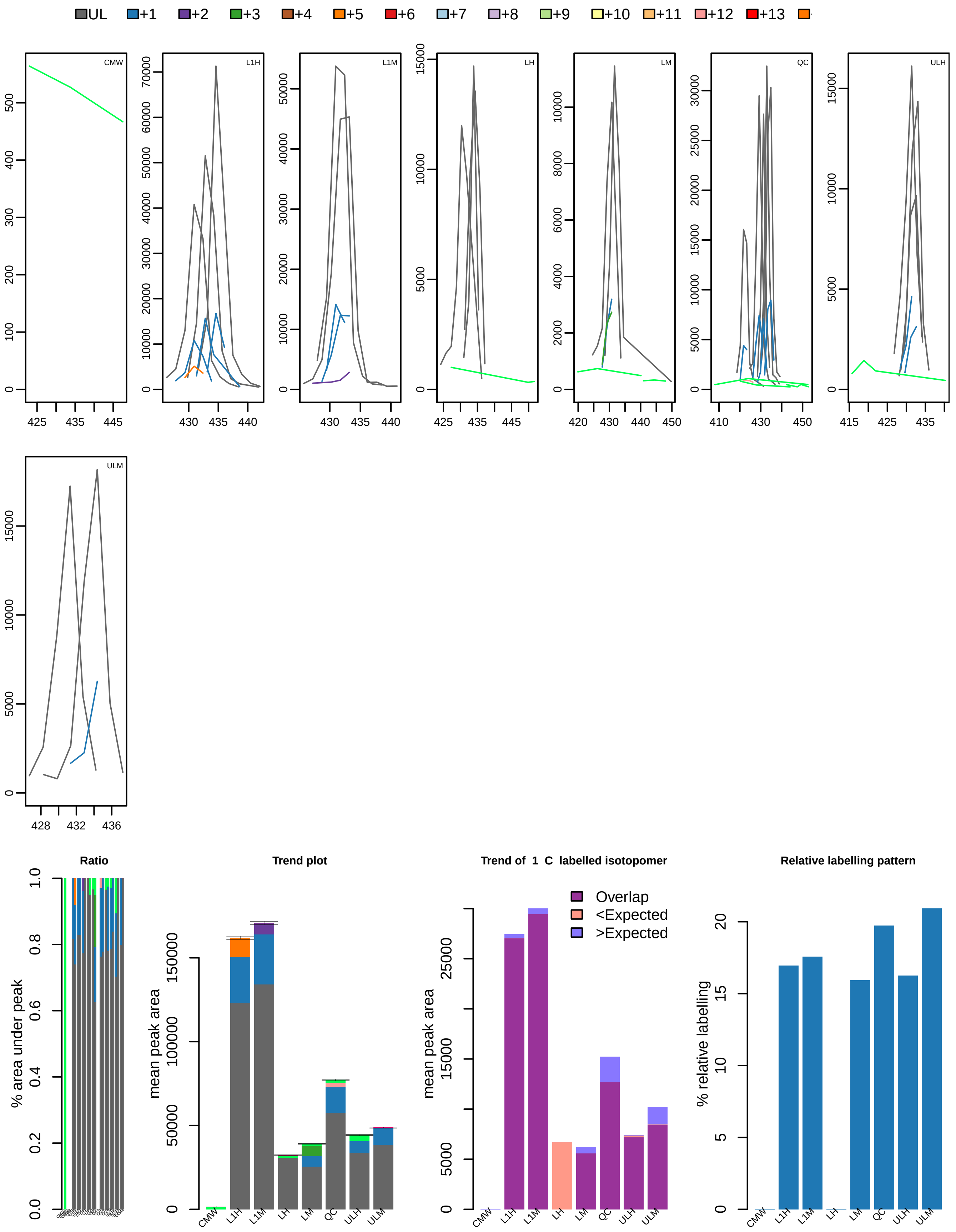

[PE (16:2/18:1)] 1−hexadecyl−2−(9Z−octadecenoyl)−sr

Formula: C39H78NO7P Mass: 703.552 Std.RT: 211.1964459 Ion: PC

G1

UL +1 +2 +3 +4 +5 +6 +7 +8 +9 +10 +11 +12 +13

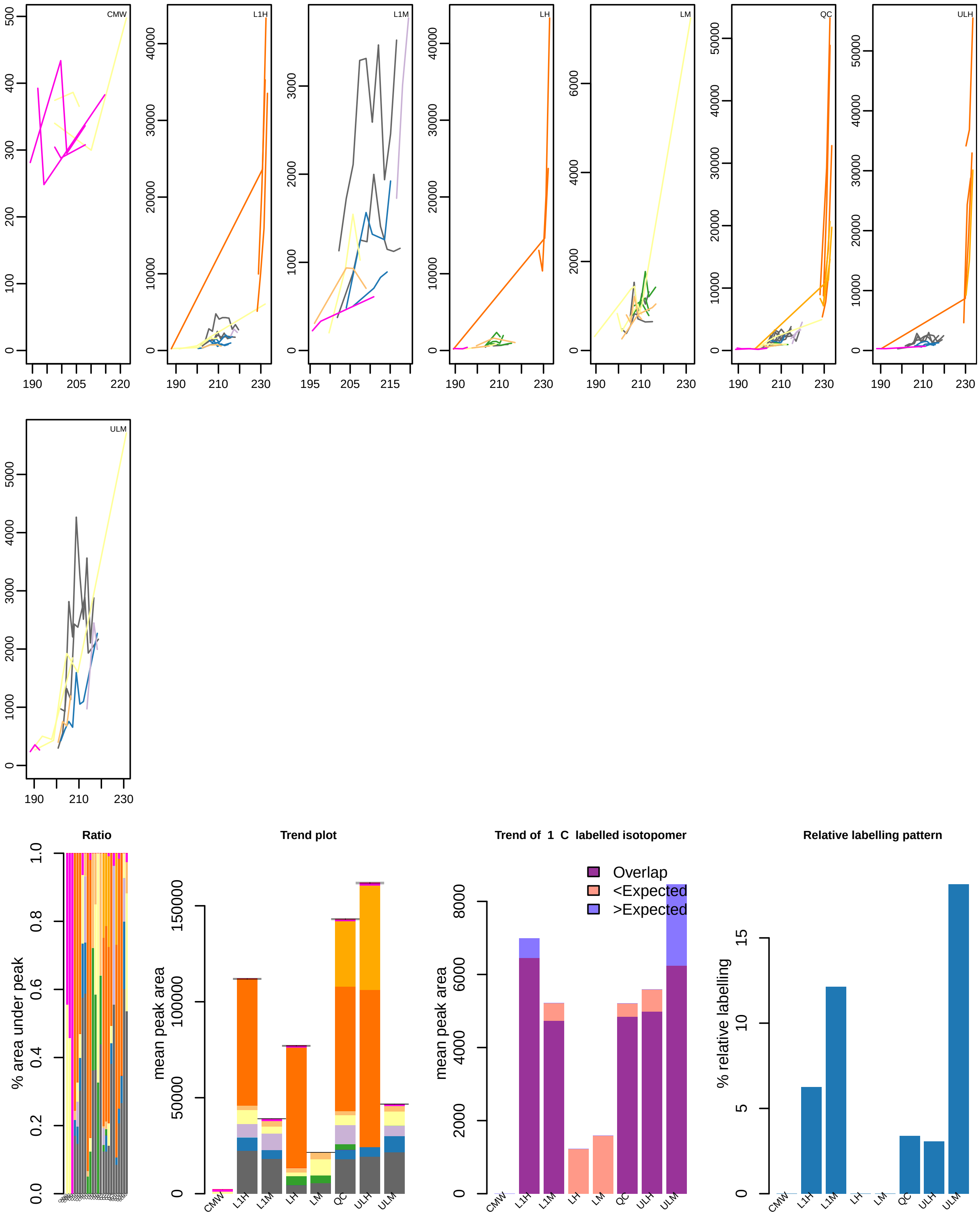

PE(14:0/16:1(9Z))

G1

Formula: C35H68NO8P Mass: 661.468 Std.RT: 208.59037692 Ion: P

UL +1 +2 +3 +4 +5 +6 +7 +8 +9 +10 +11 +12 +13

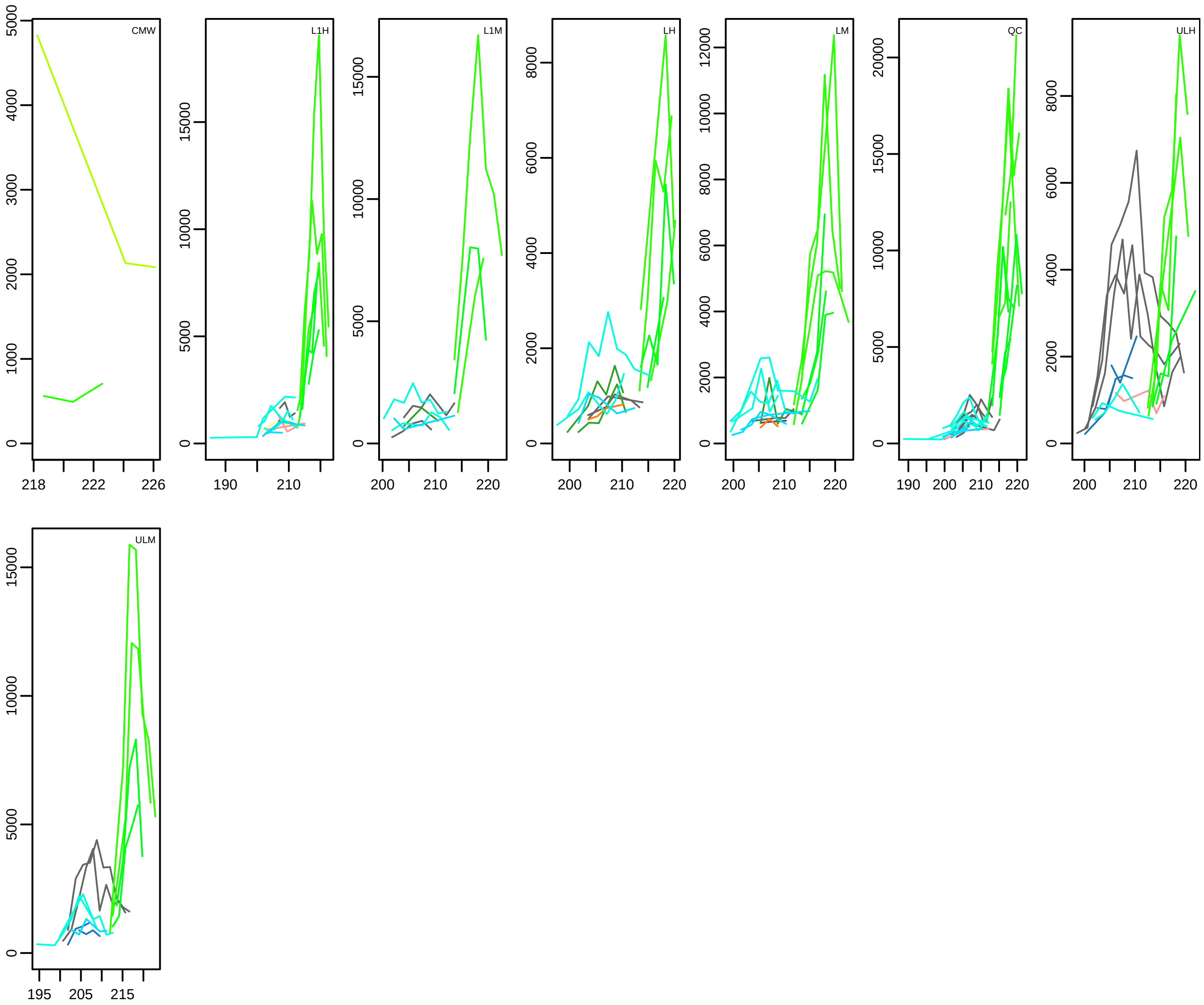

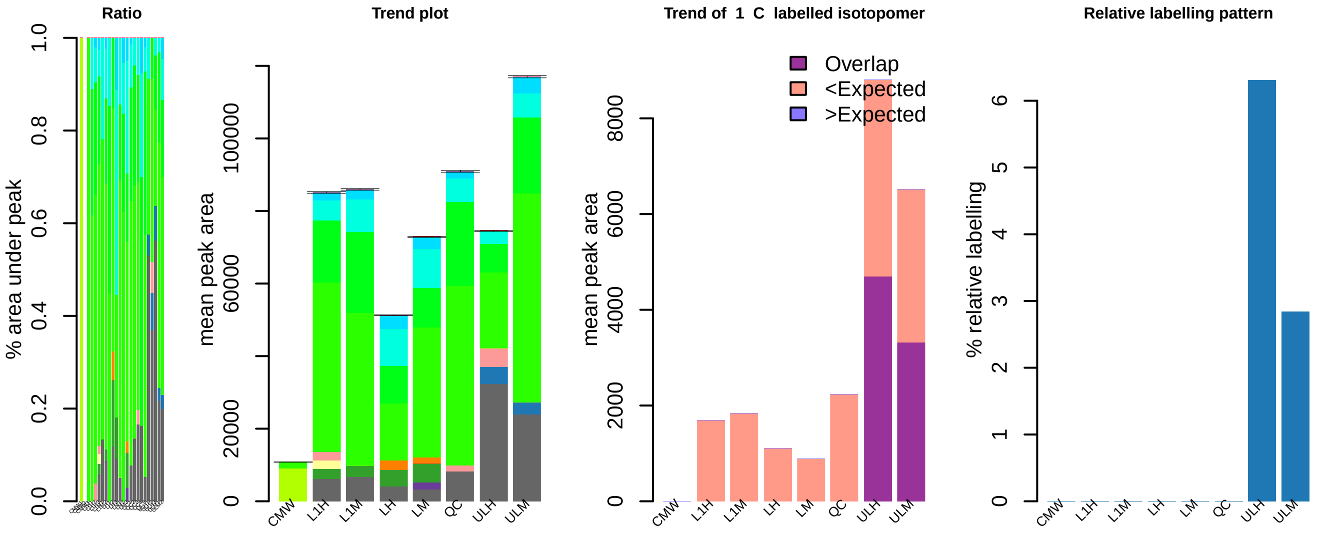

[PE (16:1/16:1)] 1,2−di−(9Z−hexadecenoyl)−sn−glycerc

Formula: C37H70NO8P Mass: 687.484 Std.RT: 225.59861898 Ion: P

G1

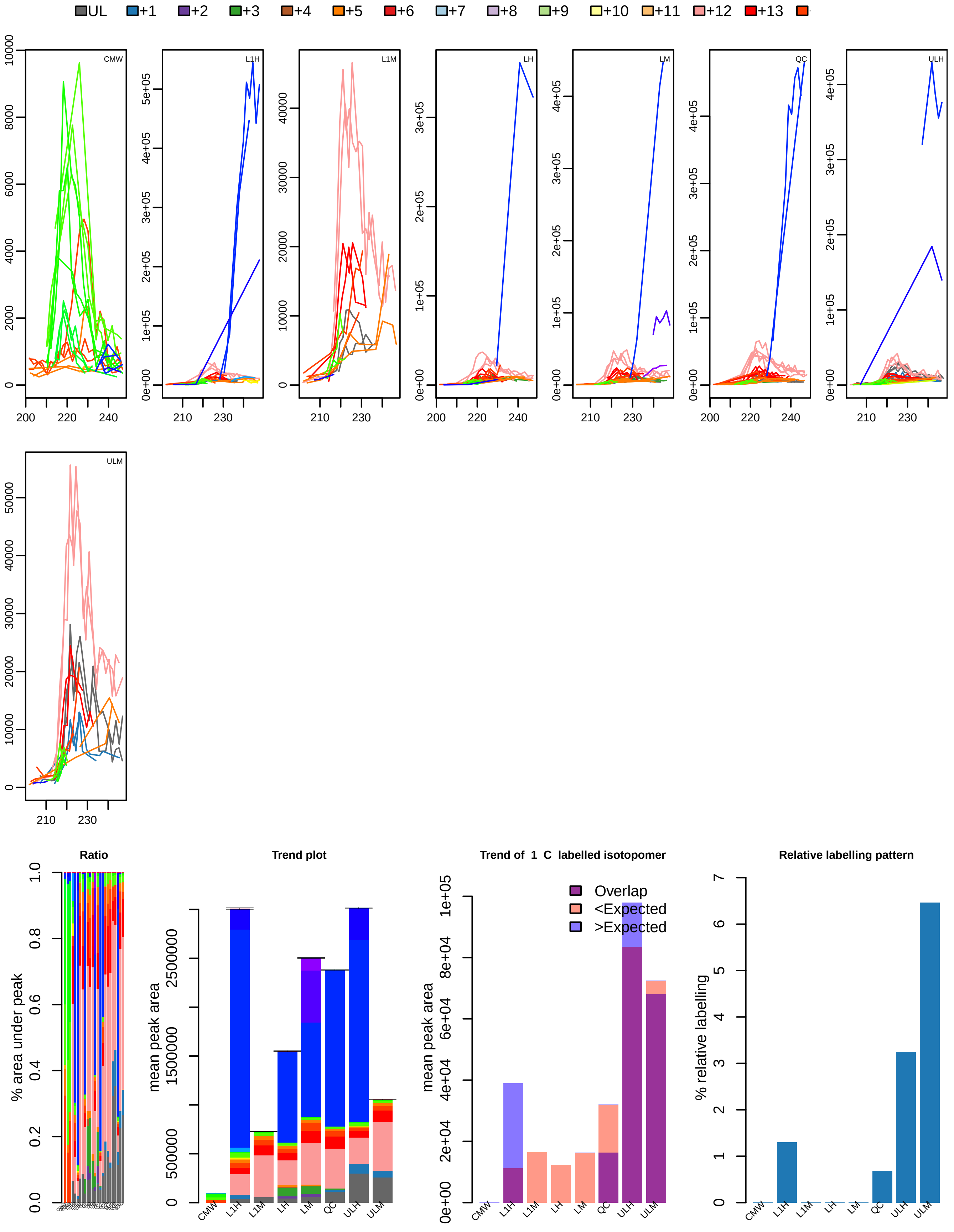

[PE (16:0/16:0)] 1,2−dihexadecanoyl−sn−glycero−3−ph

Formula: C37H74NO8P Mass: 691.515 Std.RT: 209.48019192 Ion: P

G1

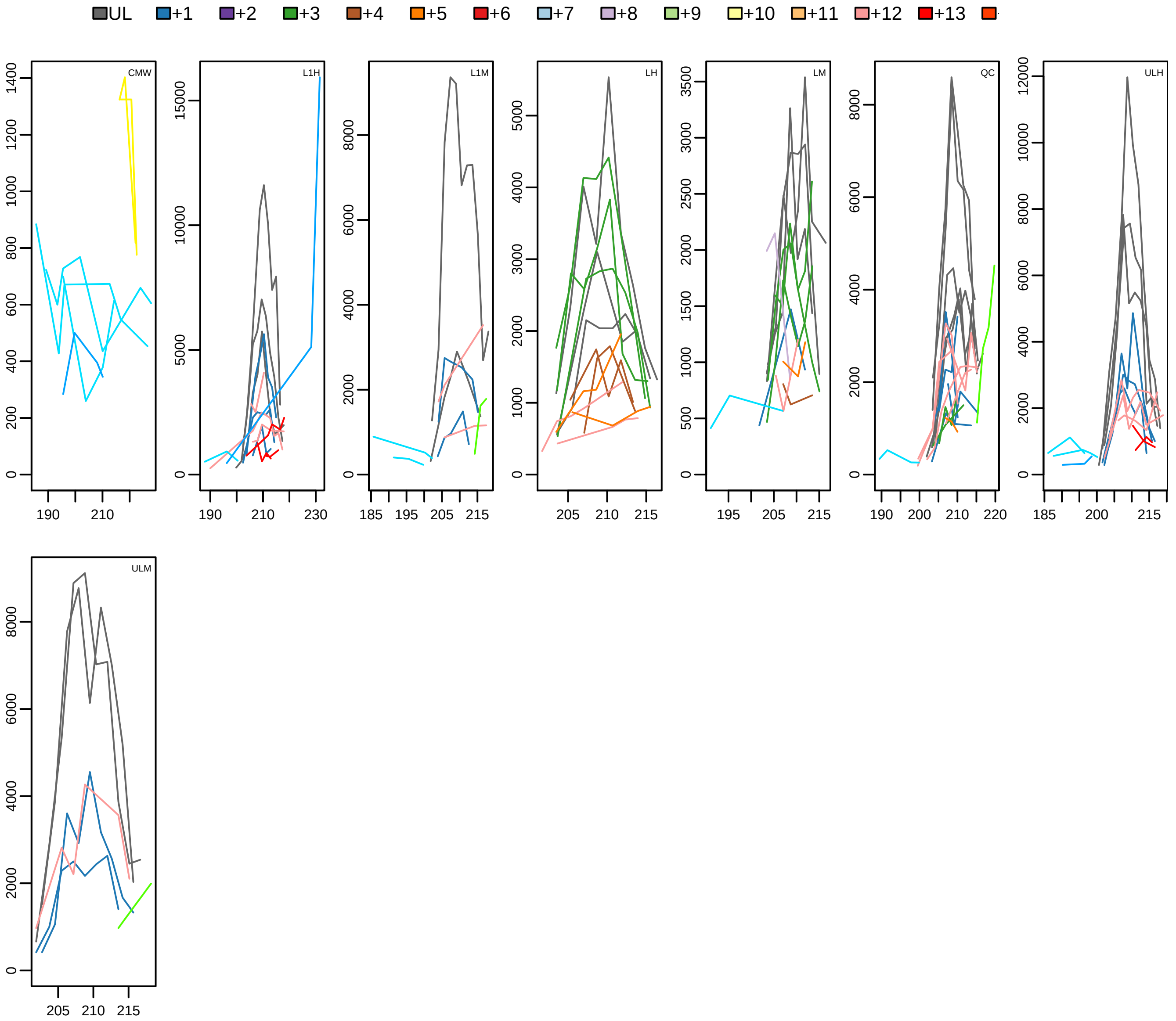

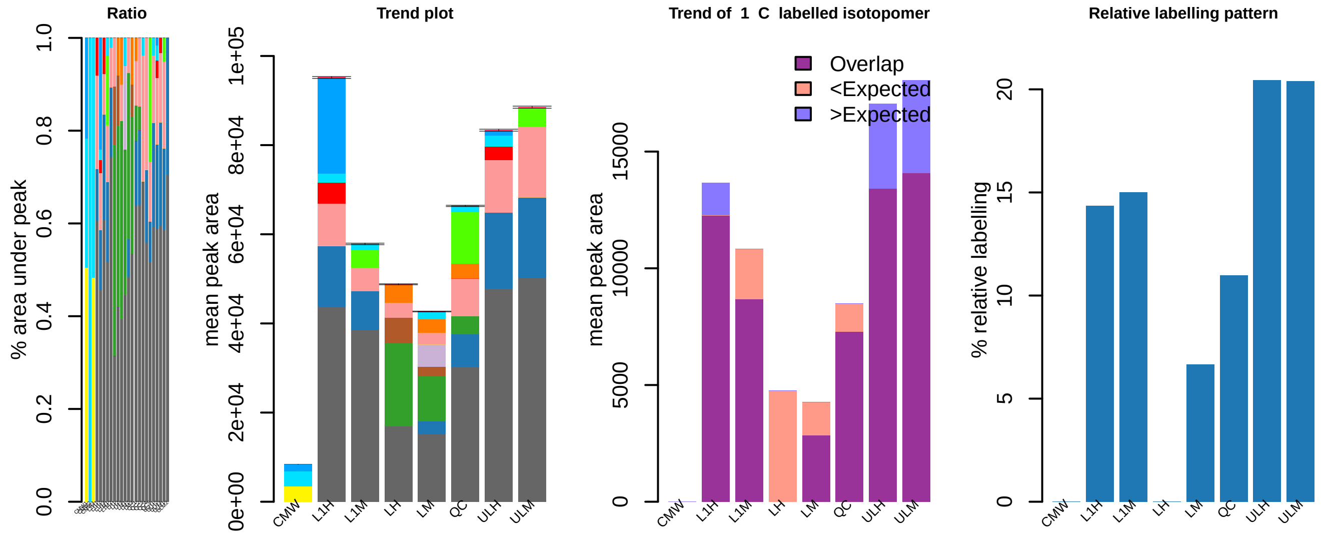

[PE (16:0/18:2)] 1−hexadecanoyl−2−(9Z,12Z−octadeca

Formula: C39H74NO8P Mass: 715.515 Std.RT: 223.3239993 Ion: PC

G1

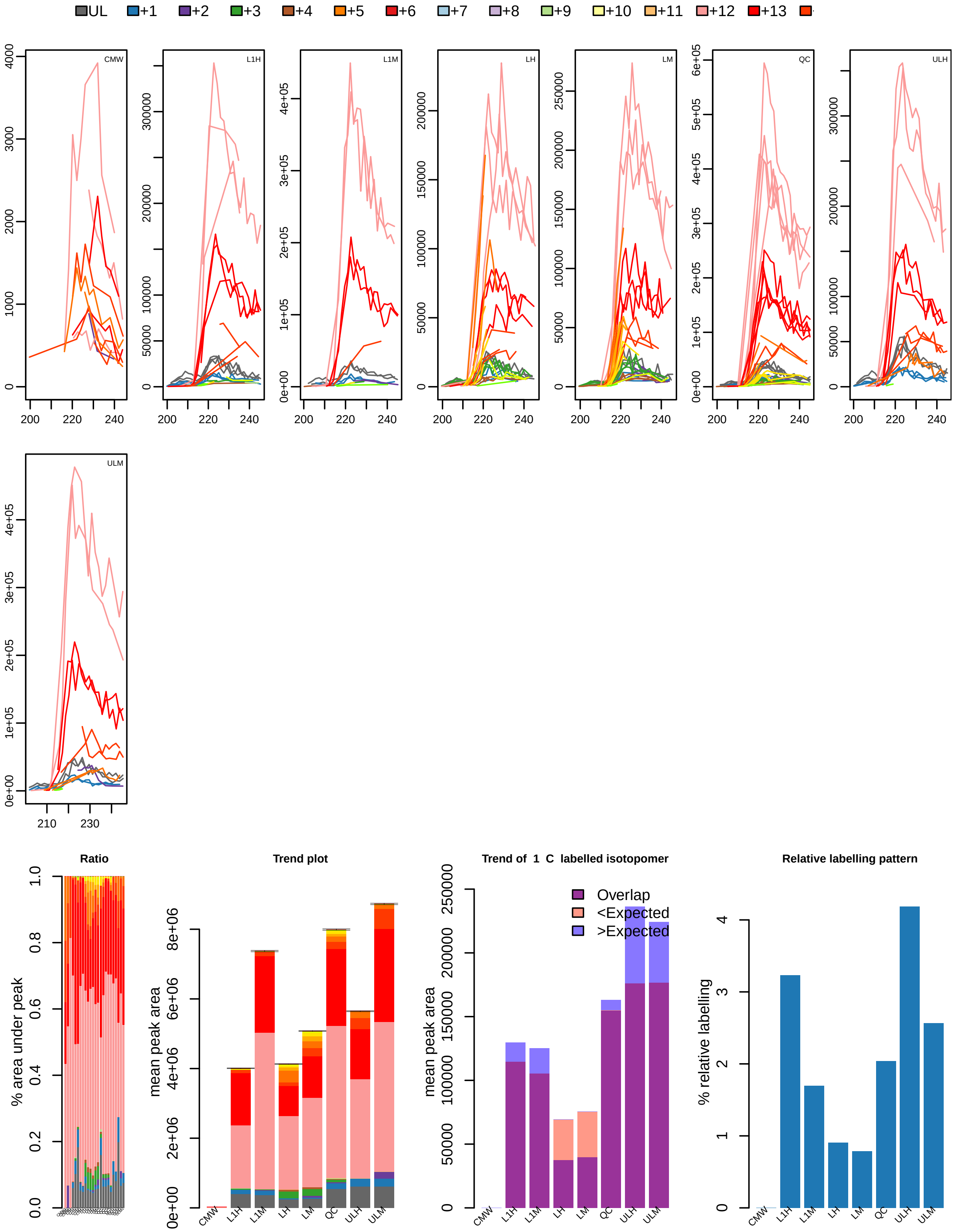

[PE (18:1)] 1−(9Z−octadecenoyl)−sn−glycero−3−phosp G1

Formula: C23H46NO7P Mass: 479.301 Std.RT: 254.1564972 Ion: PC

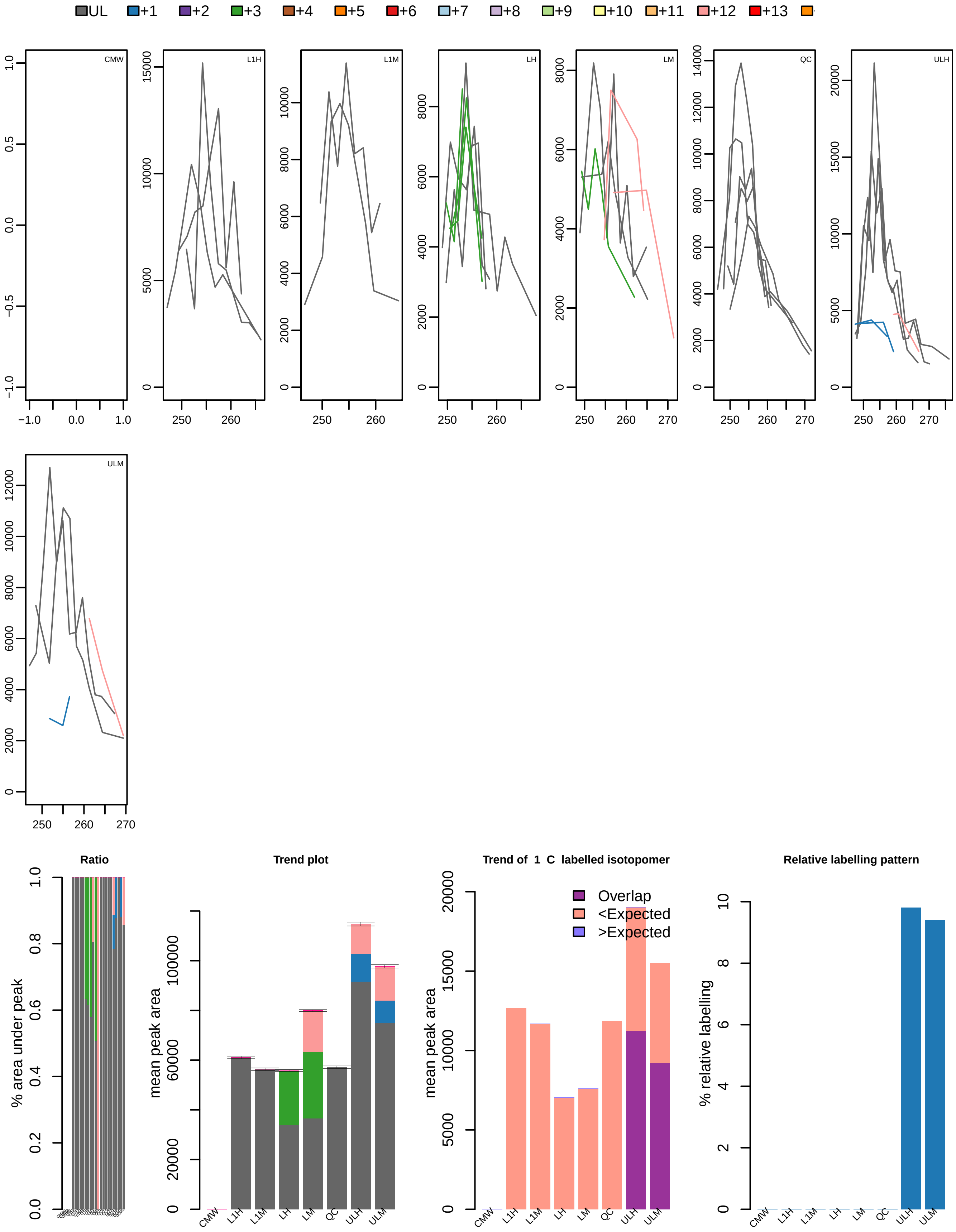

[PE (18:1/18:2)] 1−(9Z−octadecenoyl)−2−(9Z,12Z−octa

Formula: C41H76NO8P Mass: 741.531 Std.RT: 222.44733318 Ion: P

G1

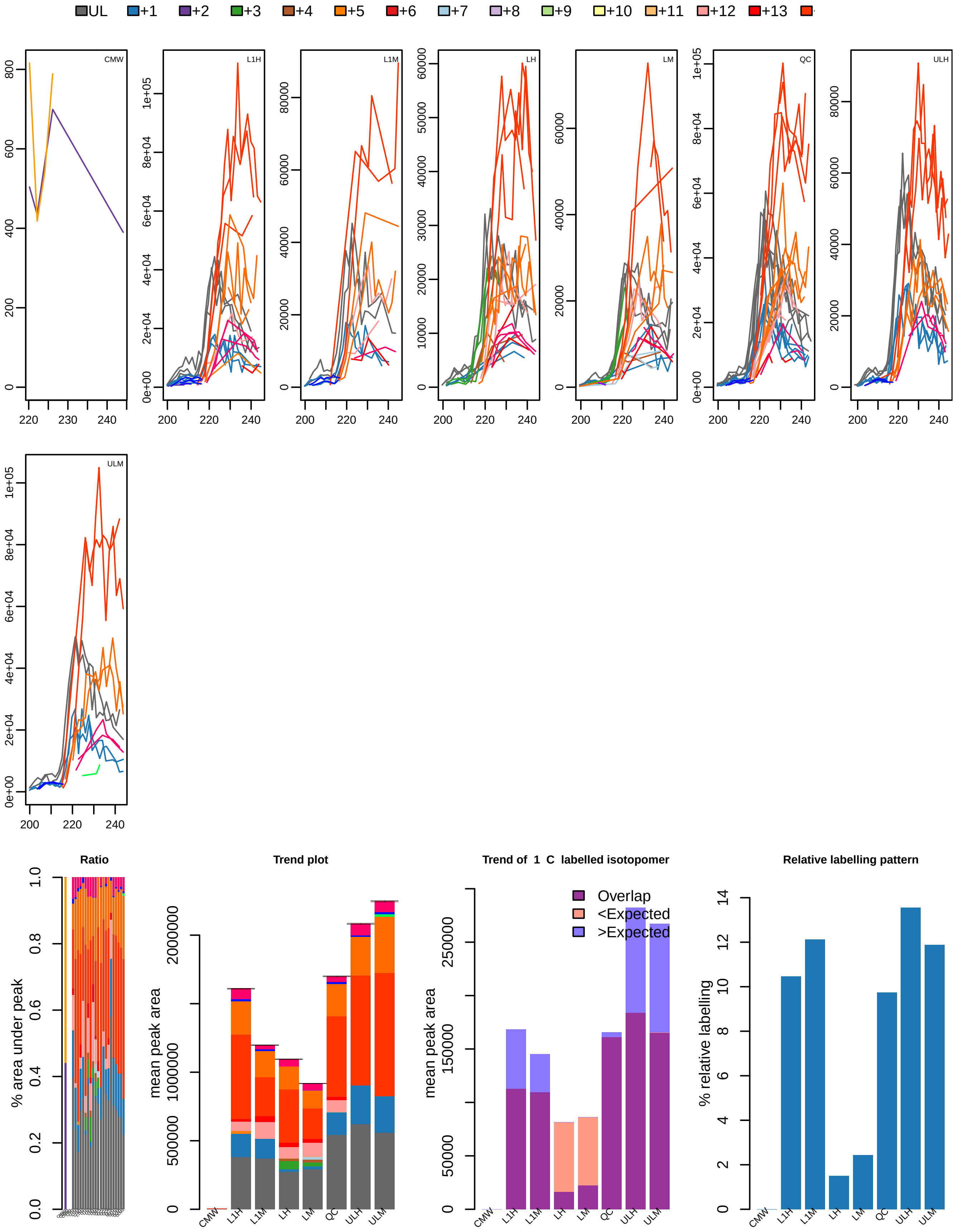

[PE (16:0/20:4)] 1−hexadecanoyl−2−(5Z,8Z,11Z,14Z−e

Formula: C41H74NO8P Mass: 739.515 Std.RT: 222.1413327 Ion: PC

G1

UL +1 +2 +3 +4 +5 +6 +7 +8 +9 +10 +11 +12 +13

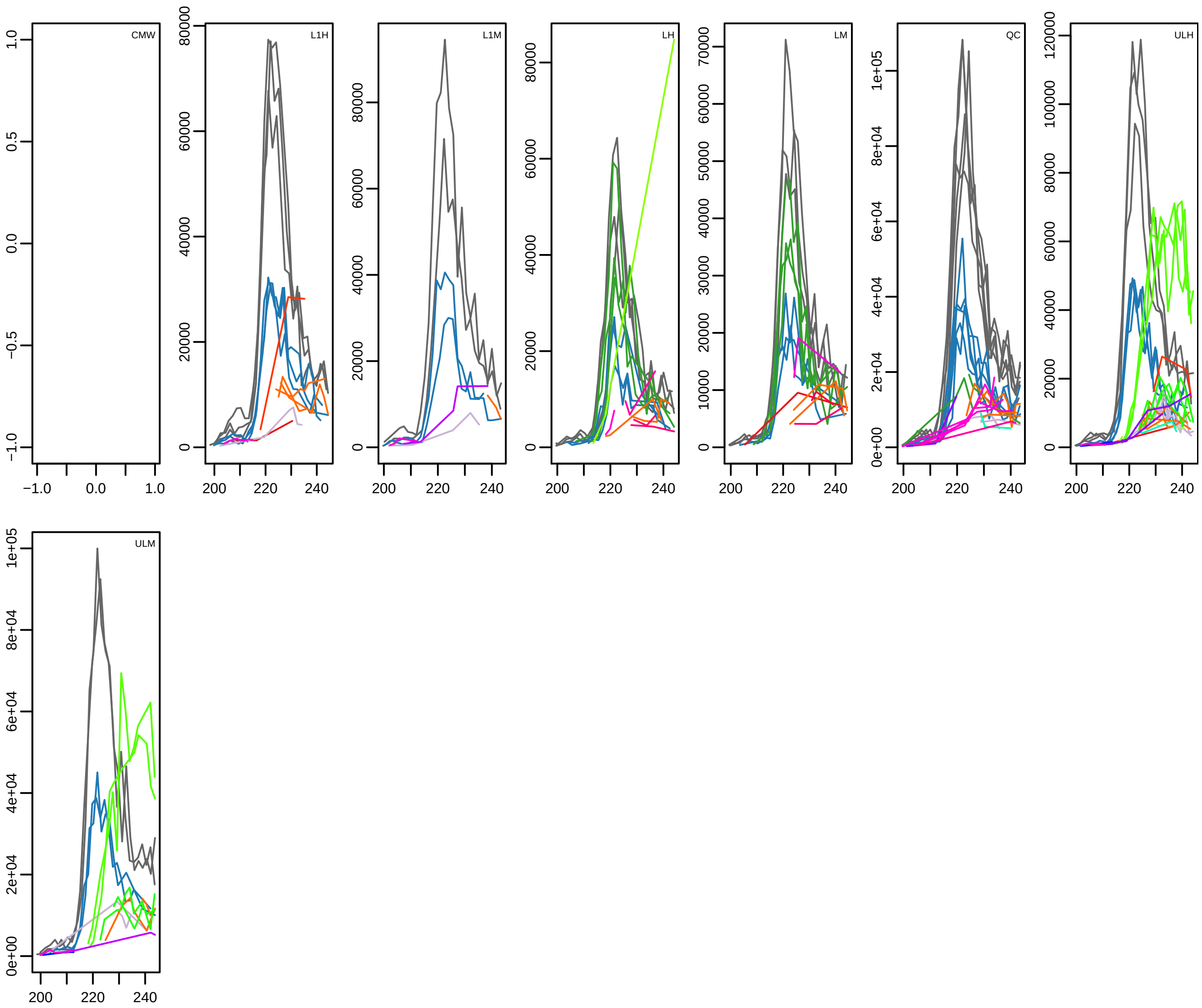

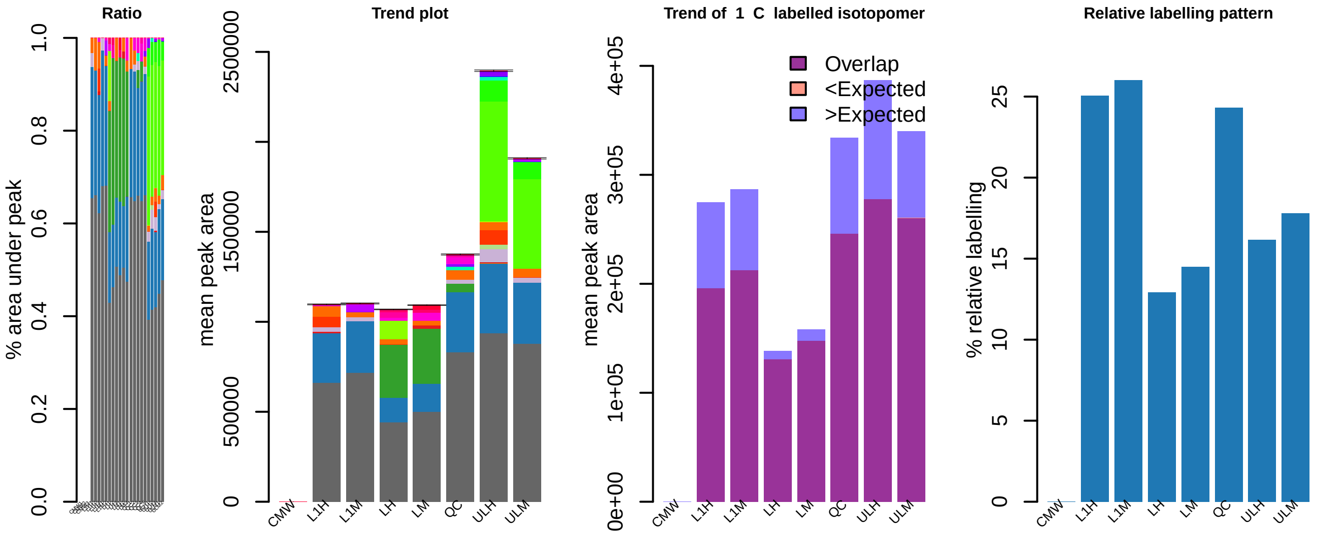

LysoPE(0:0/18:2(9Z,12Z))

Formula: $C_{23}H_{44}NO_7P$ Mass: 477.286 Std.RT: 255.2850405 Ion: PC

G1

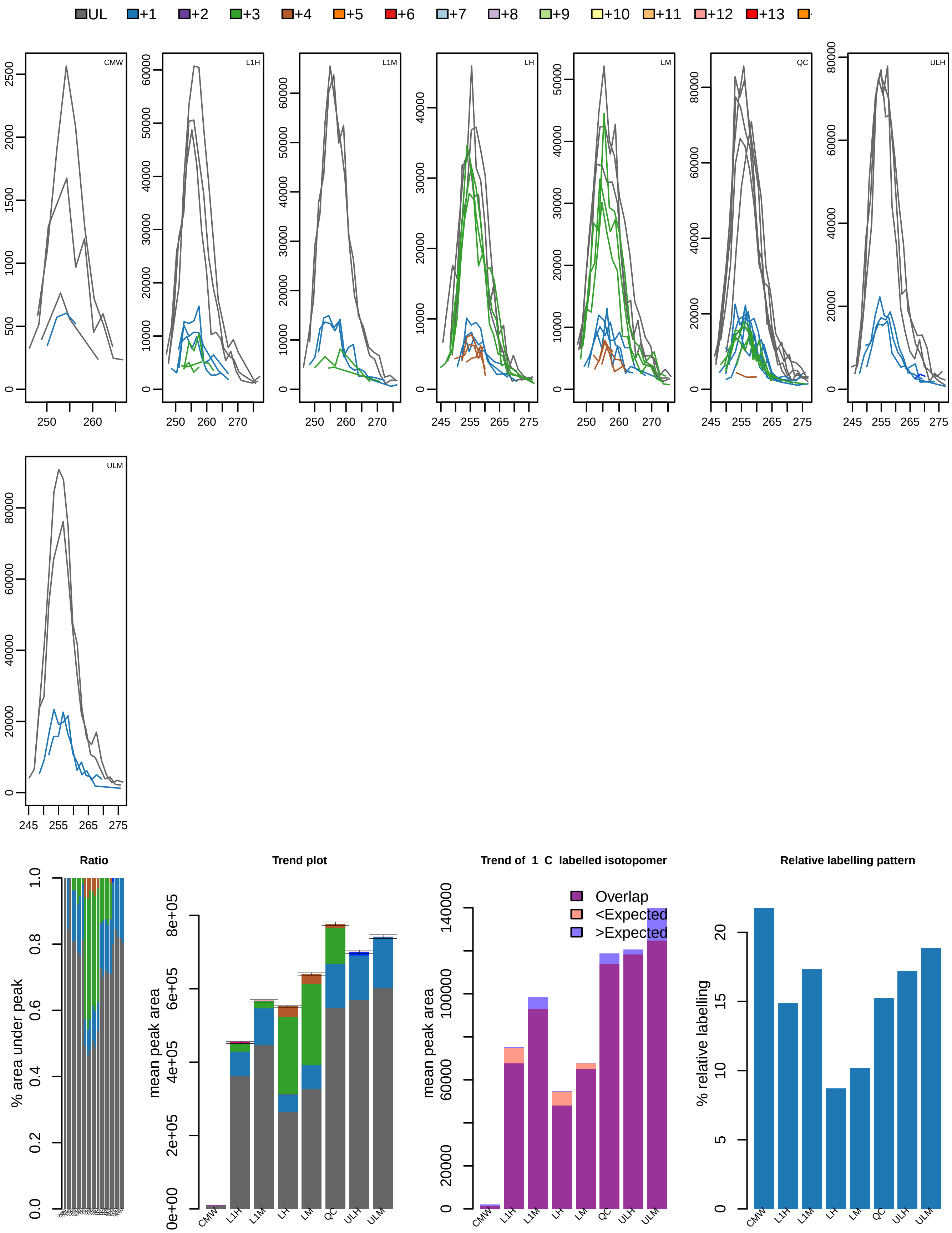

PE(18:1(11Z)/P−18:1(11Z))

Formula: C41H78NO7P Mass: 727.552 Std.RT: 224.69466528 Ion: P

G1

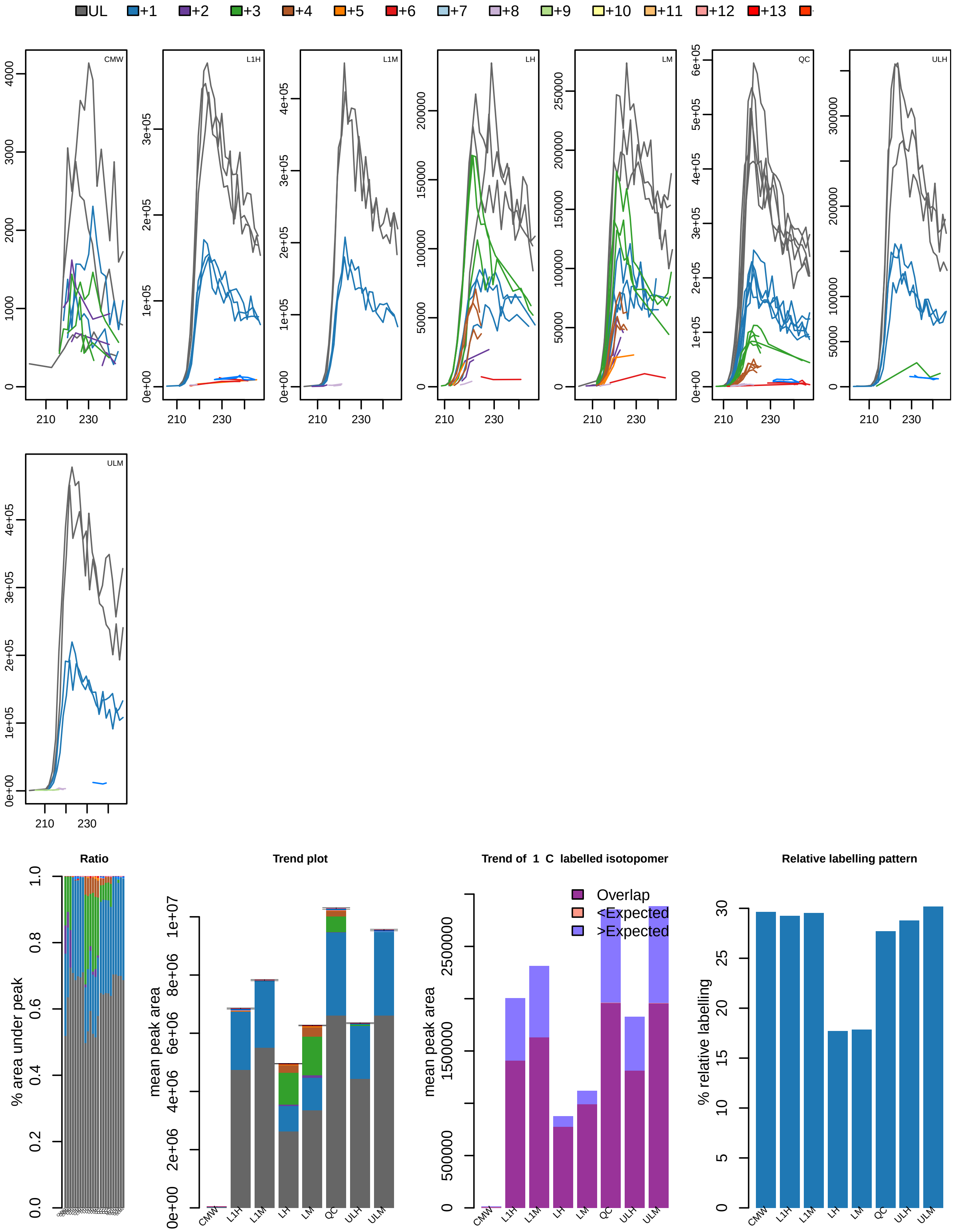

PE(18:1(11Z)/P−18:1(11Z))

Formula: C41H78NO7P Mass: 727.552 Std.RT: 224.69466528 Ion: P

G2

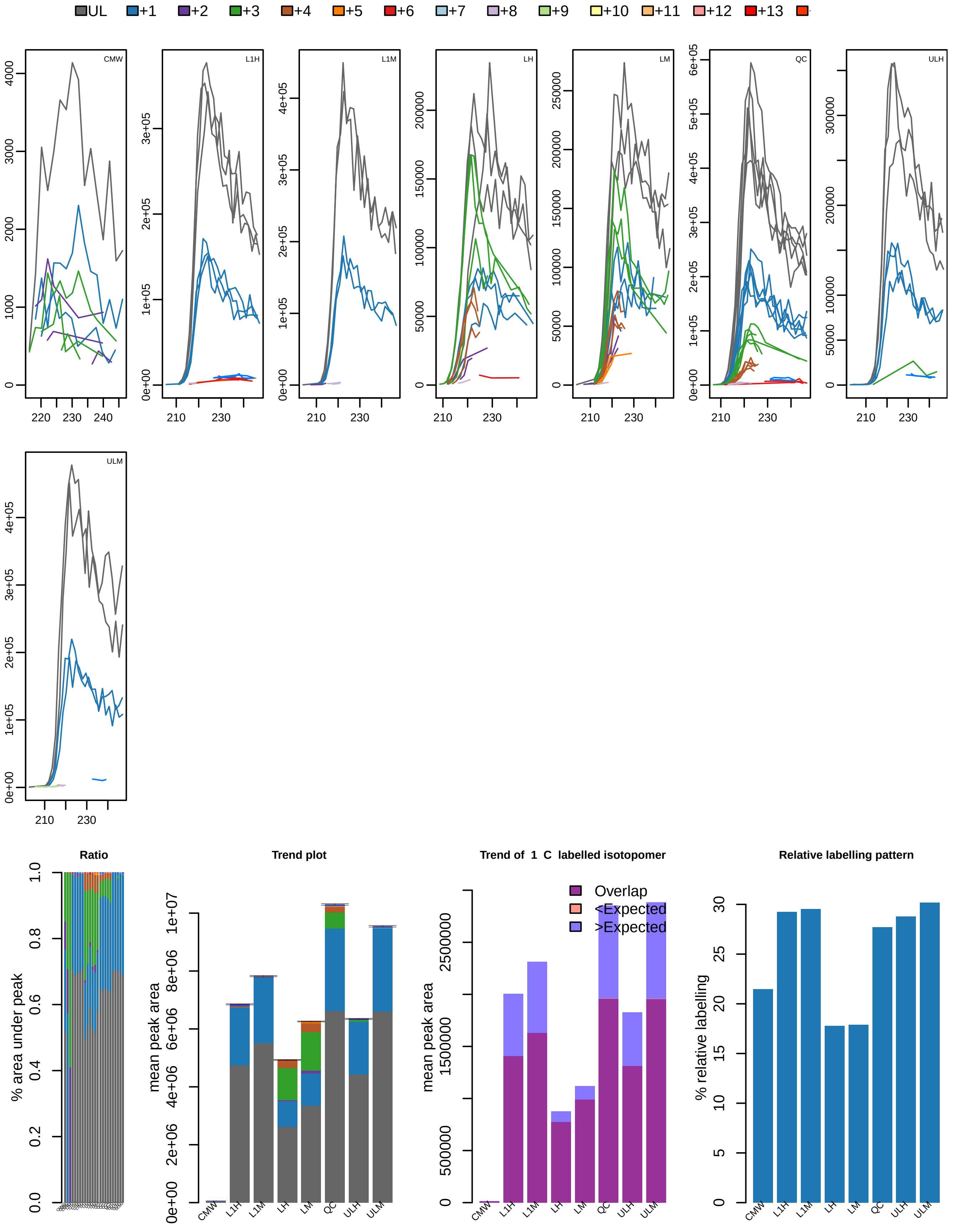

PE(18:2(9Z,12Z)/P−18:1(11Z))

G1

Formula: C41H76NO7P Mass: 725.536 Std.RT: 222.82052388 Ion: P

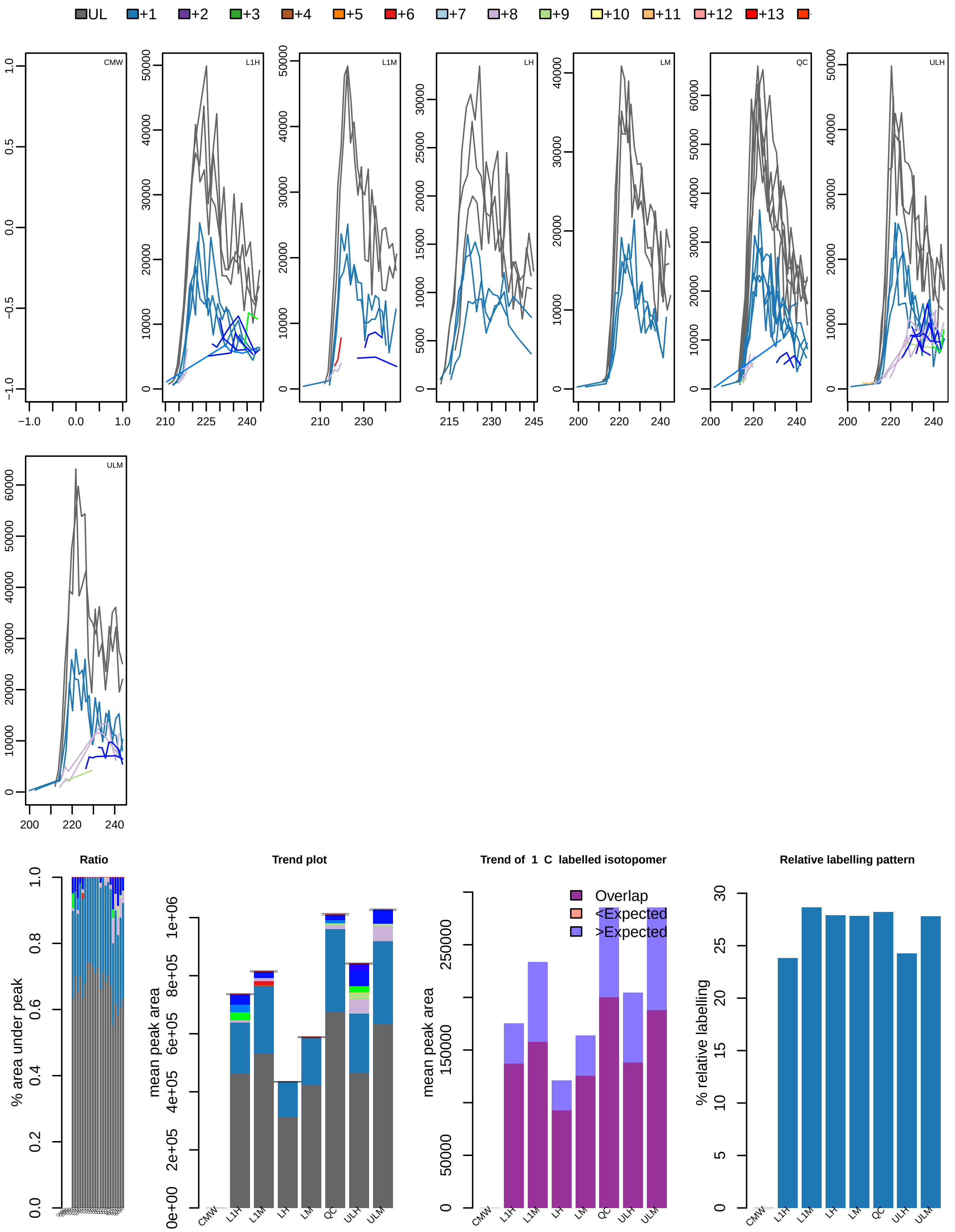

[PE (18:0/20:4)] 1−octadecanoyl−2−(5Z,8Z,11Z,14Z−ei

Formula: C43H78NO8P Mass: 767.547 Std.RT: 230.35666548 Ion: P

G1

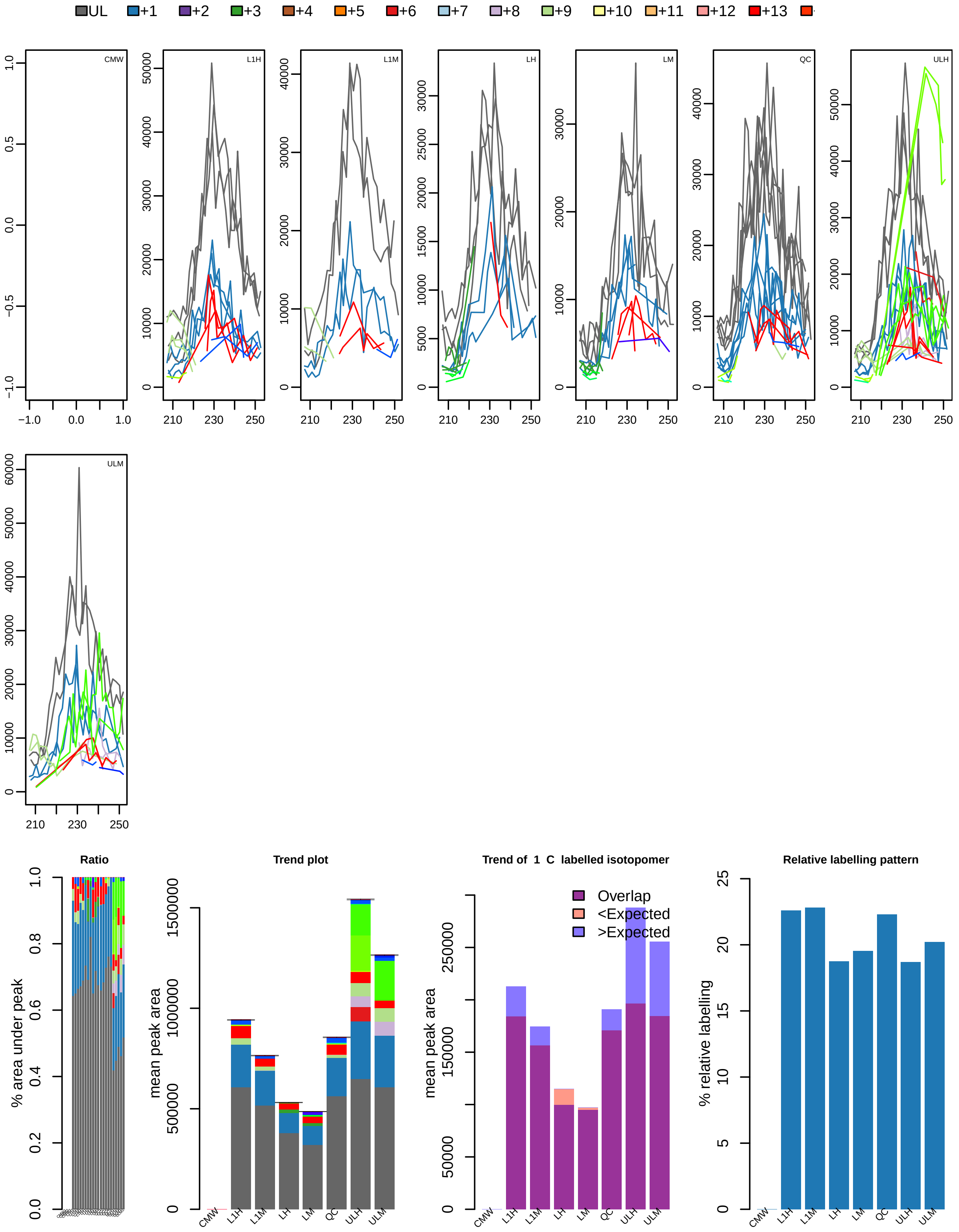

[PE (16:0/18:3)] 1−hexadecanoyl−2−(9Z,12Z,15Z−octa[illegible]

Formula: C39H72NO8P Mass: 713.5 Std.RT: 218.60966562 Ion: PO[illegible]

G1

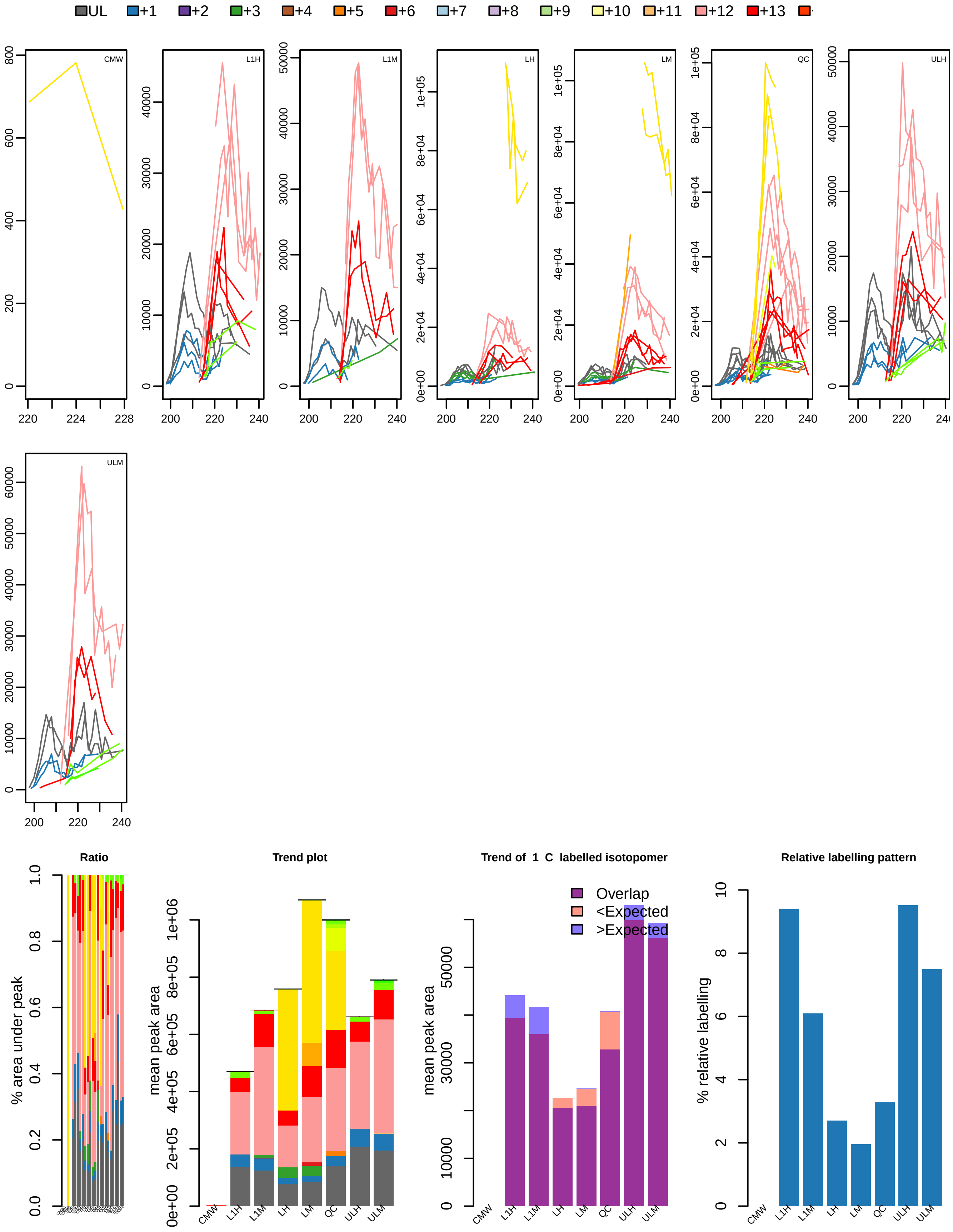

PE(18:1(11Z)/22:2(13Z,16Z))

Formula: C45H84NO8P Mass: 797.593 Std.RT: 231.1237611 Ion: PC

G1

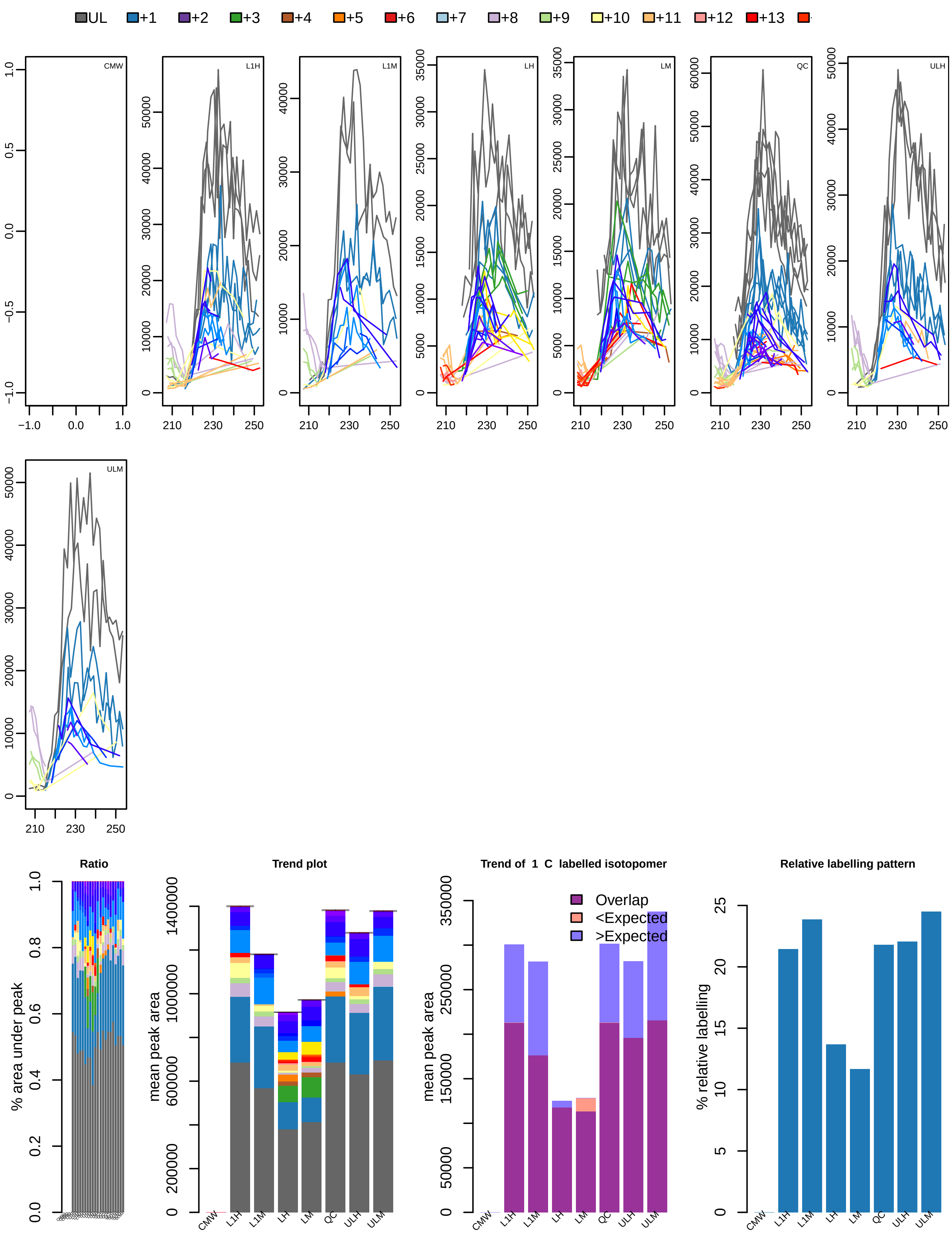

PE(20:2(11Z,14Z)/18:3(6Z,9Z,12Z))

Formula: C43H76NO8P Mass: 765.531 Std.RT: 222.60957048 Ion: P

G1

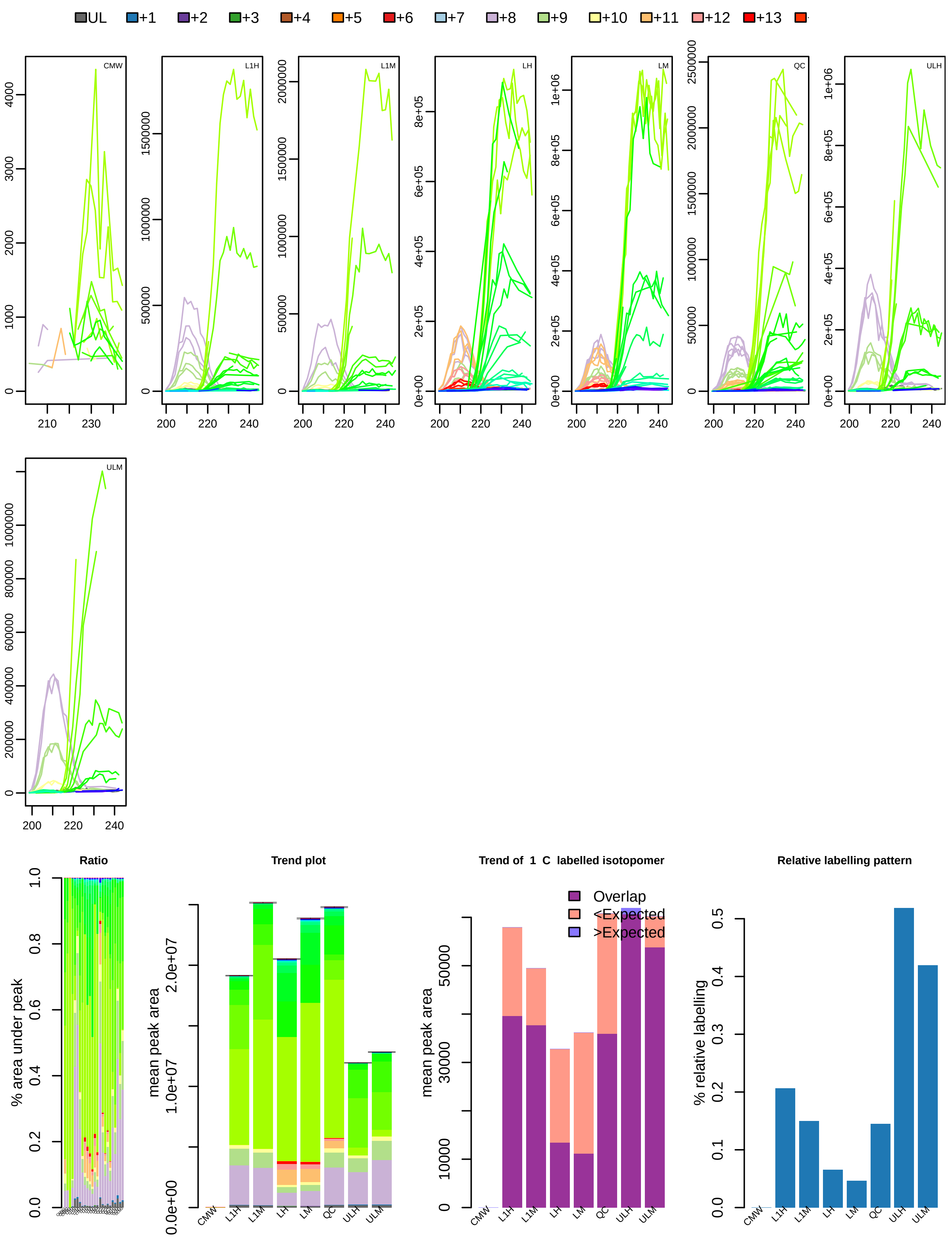

[PE (16:0/18:1)] 1−Hexadecanoyl−2−(9Z−octadecenoyl

Formula: C39H76NO8P Mass: 717.531 Std.RT: 220.32730428 Ion: P

G1

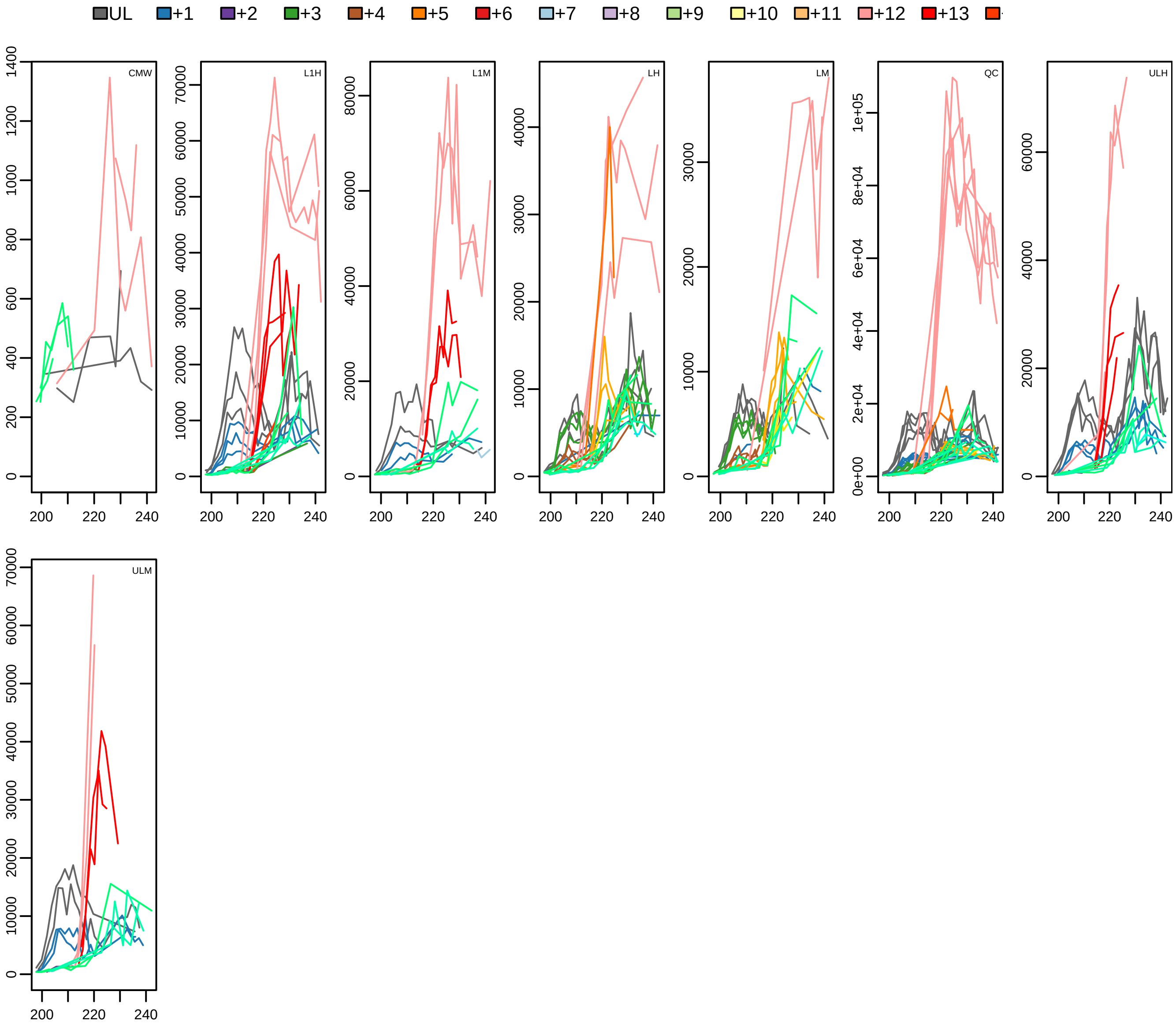

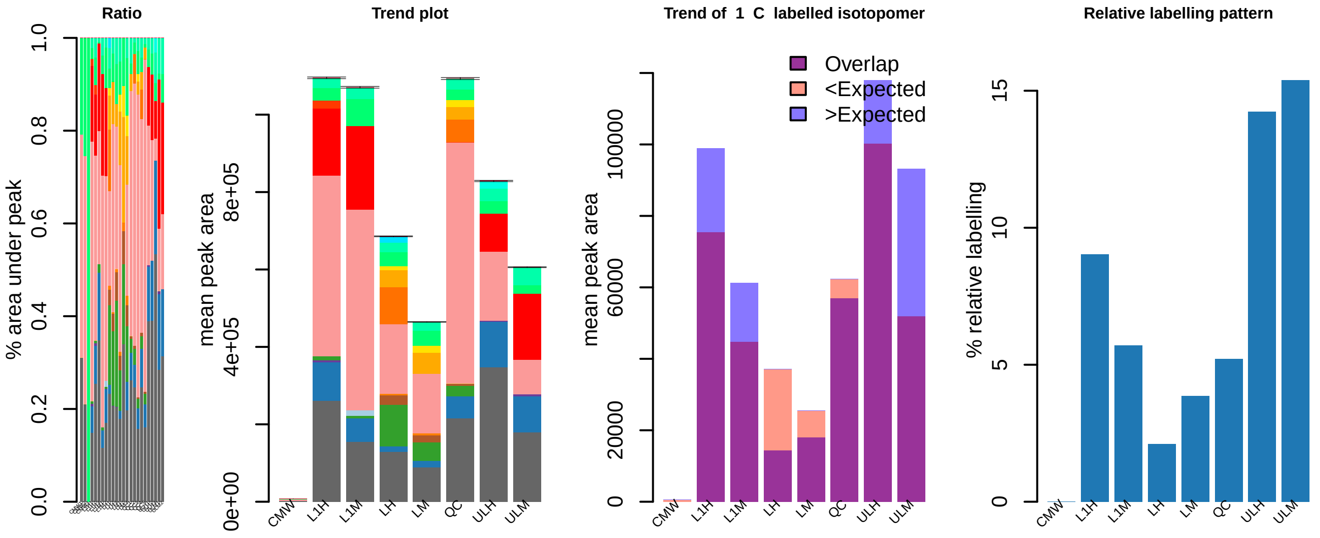

PE(18:4(6Z,9Z,12Z,15Z)/24:1(15Z))

Formula: $C_{47}H_{84}NO_8P$ Mass: 821.593 Std.RT: 228.67471308 Ion: P

G1

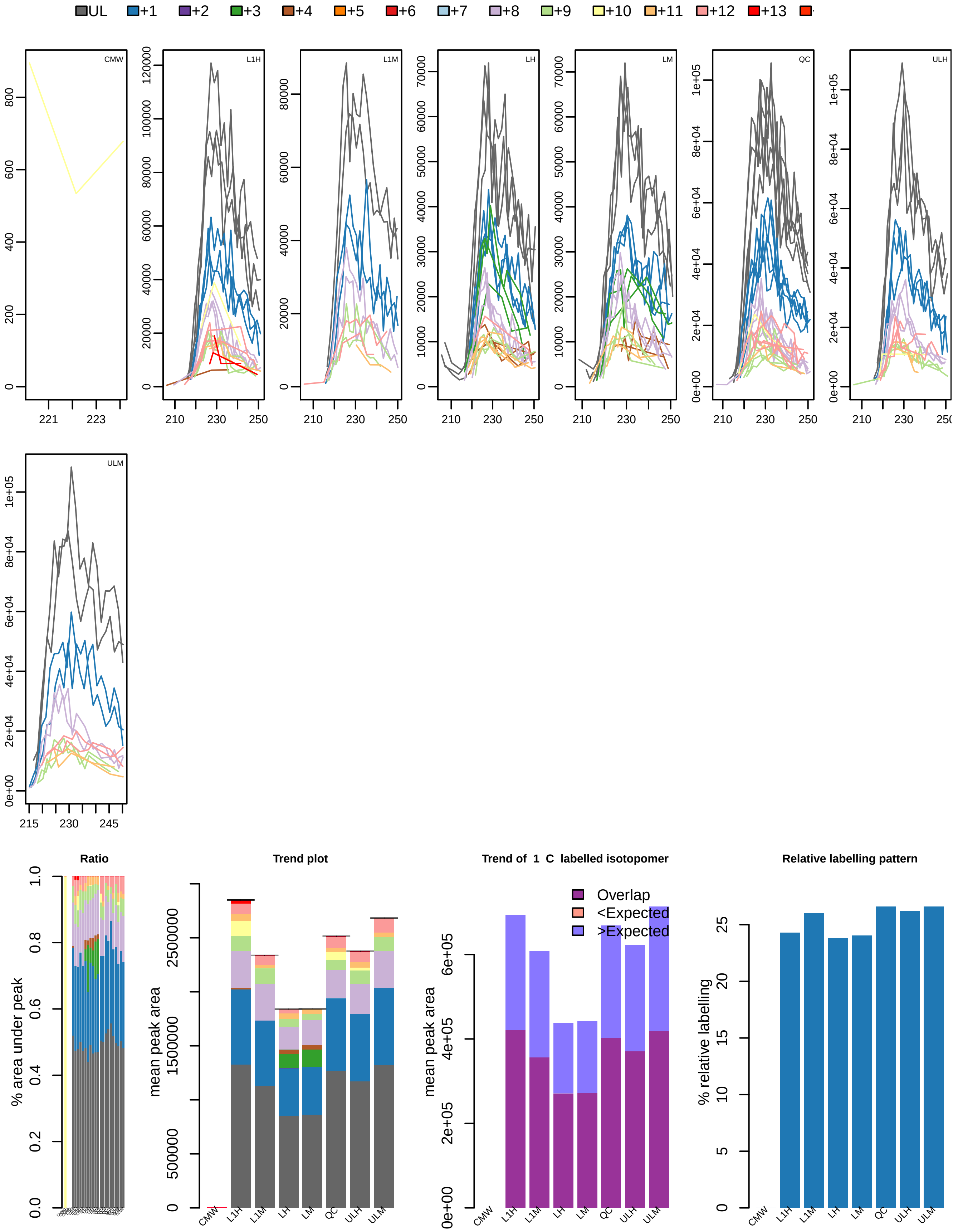

PE(18:3(6Z,9Z,12Z)/24:1(15Z))

Formula: C47H86NO8P Mass: 823.609 Std.RT: 230.50819032 Ion: P

G1

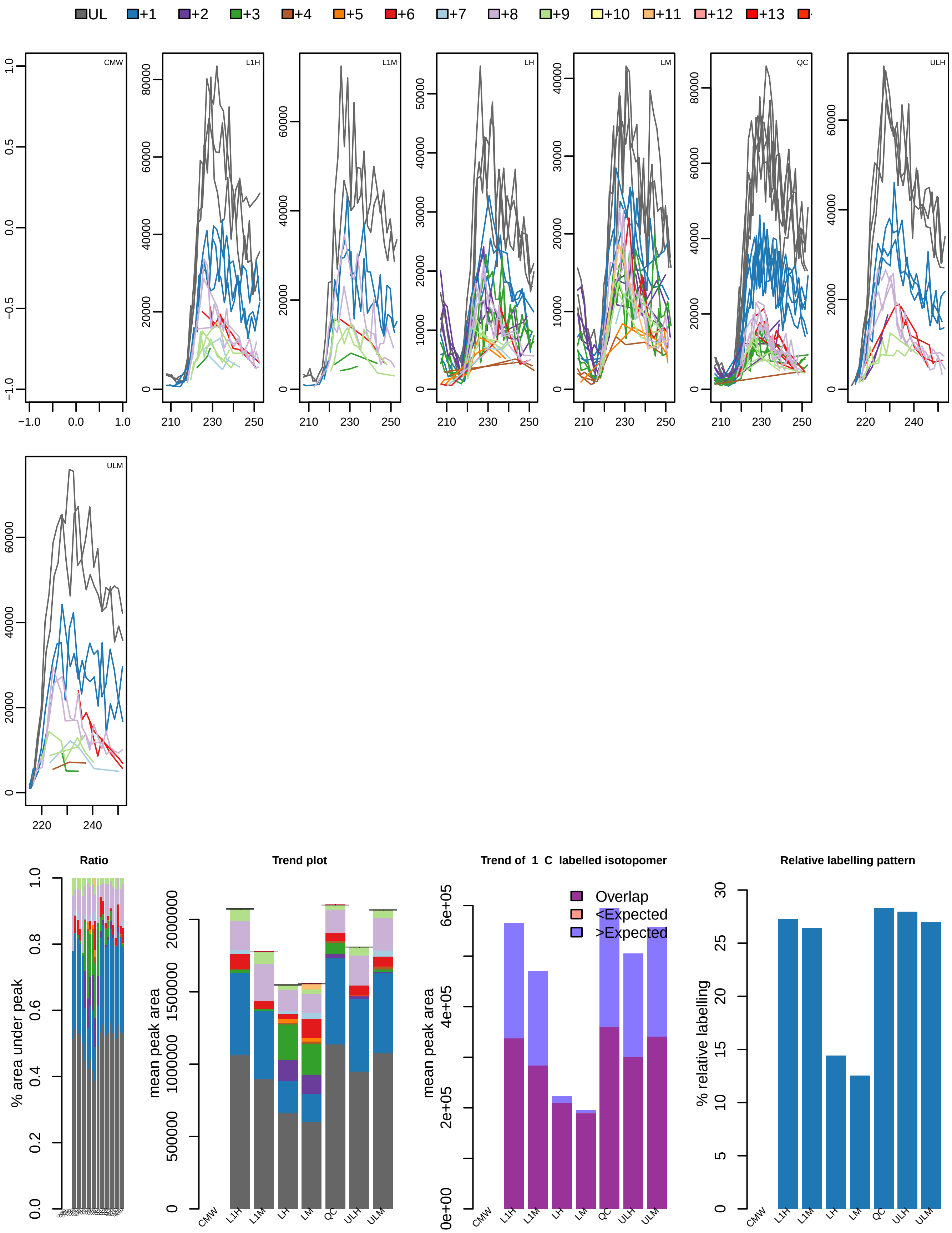

[PE (18:1/18:1)] 1−(1Z−octadecenyl)−2−(9Z−octadecer

G1

Formula: C41H80NO7P Mass: 729.567 Std.RT: 227.0263911 Ion: PC

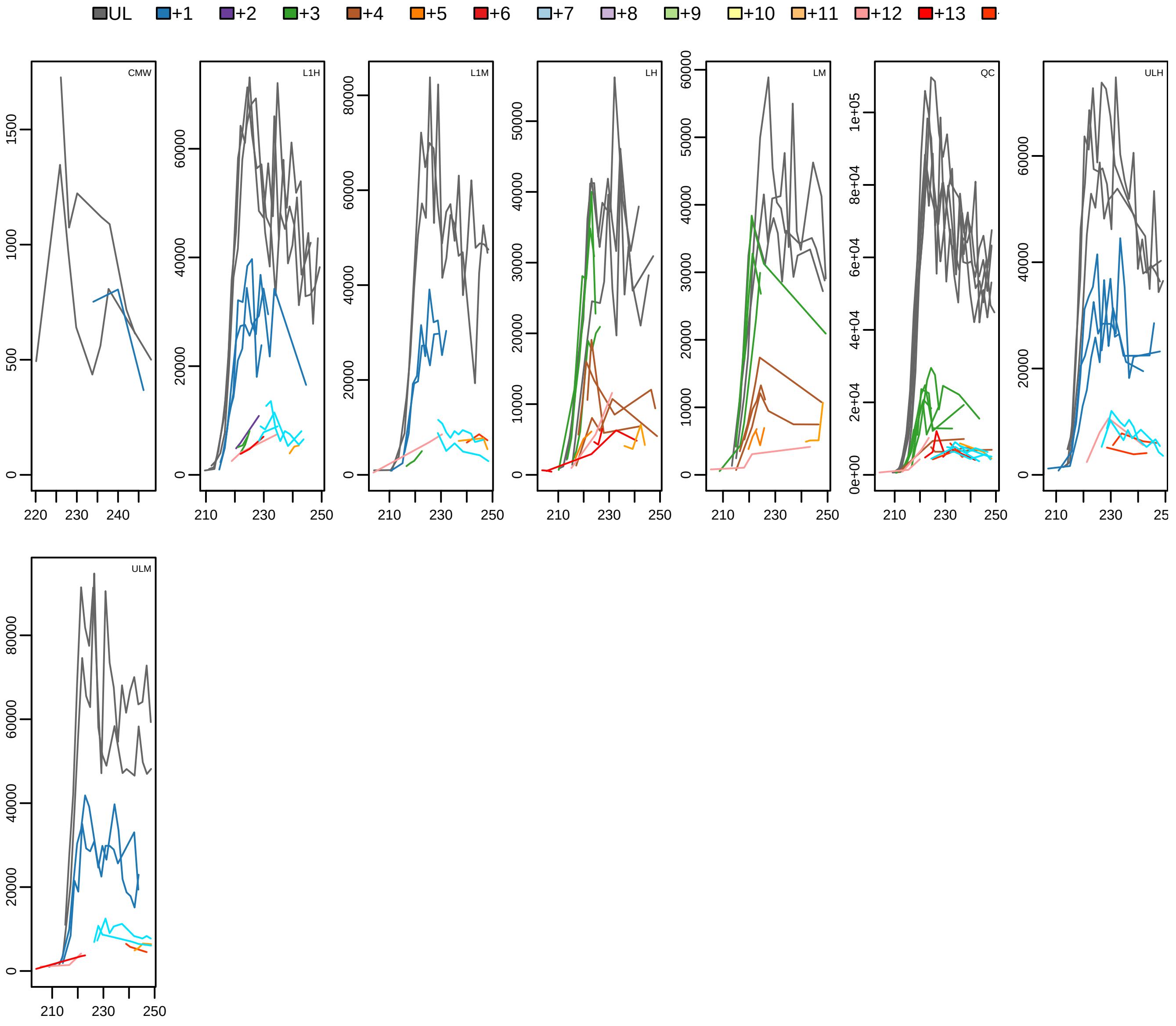

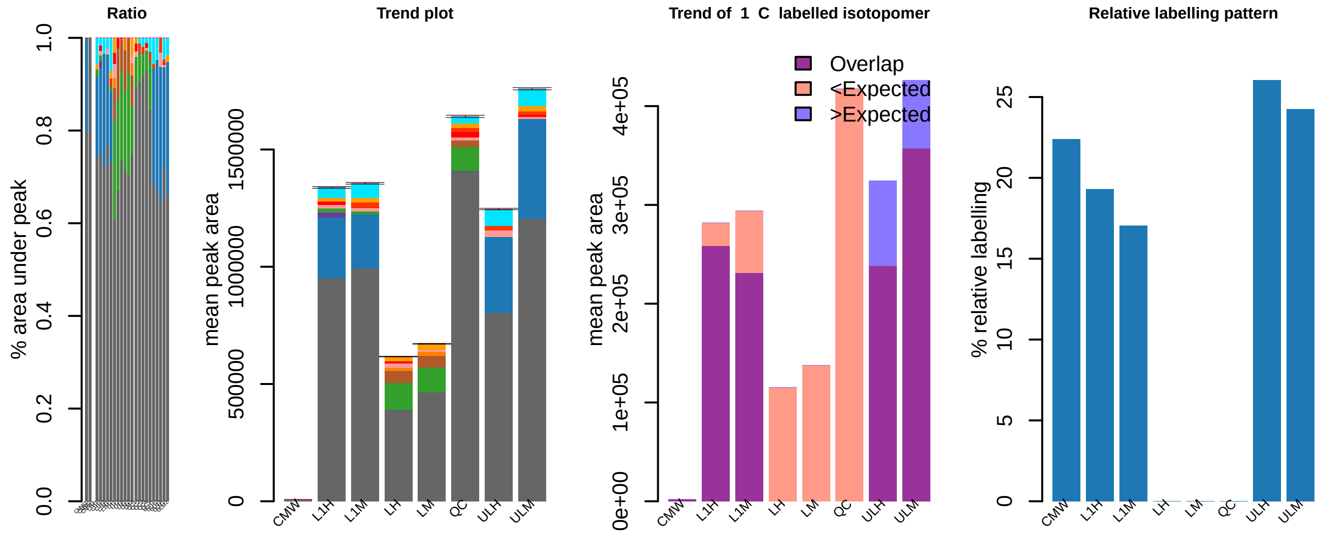

[PE (18:1/18:1)] 1−(1Z−octadecenyl)−2−(9Z−octadecer

Formula: C41H80NO7P Mass: 729.567 Std.RT: 227.0263911 Ion: PC

G2

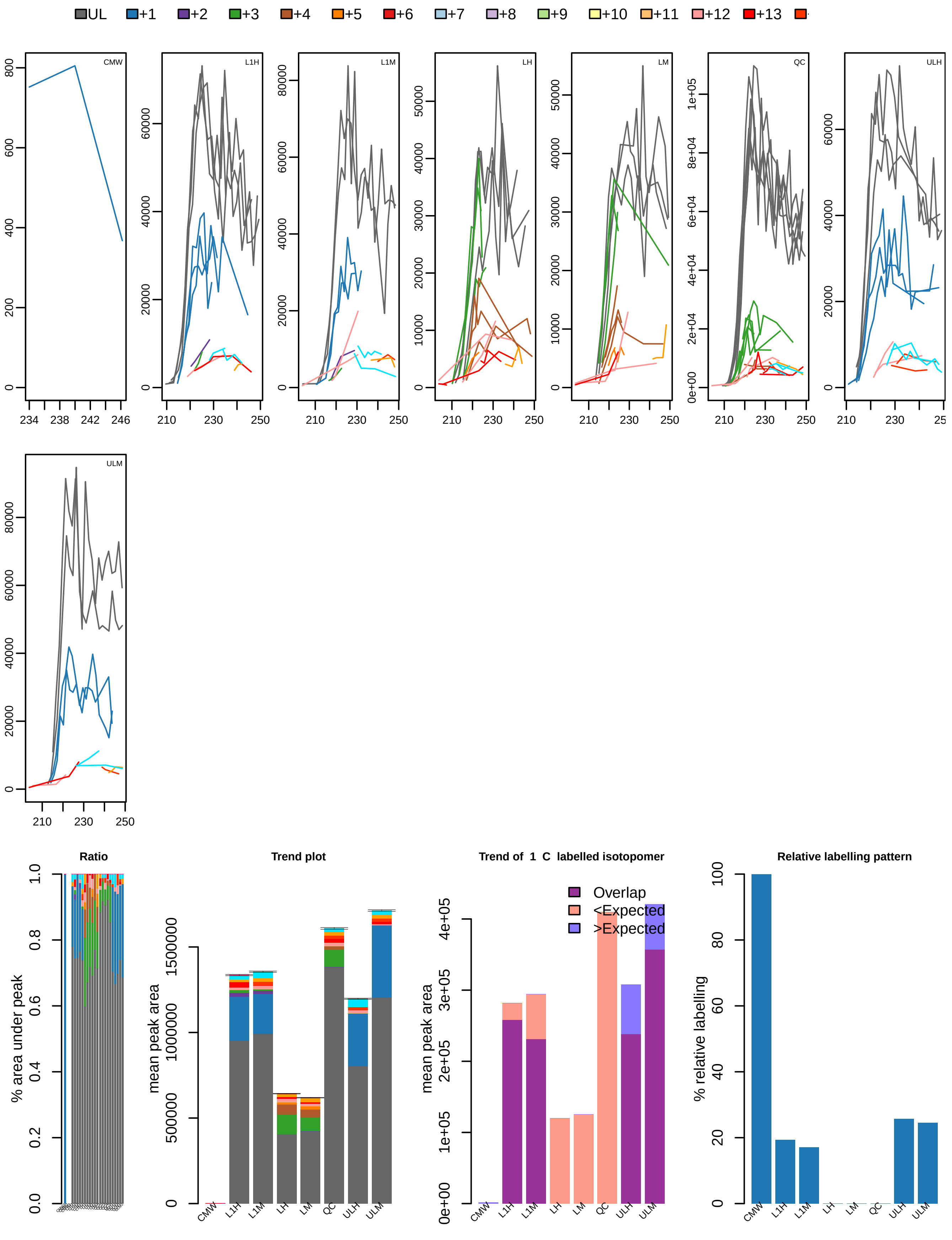

[PE (18:1)] 1−(9Z−octadecenyl)−sn−glycero−3−phosph

Formula: C23H48NO6P Mass: 465.322 Std.RT: 257.75214282 Ion: P

G1

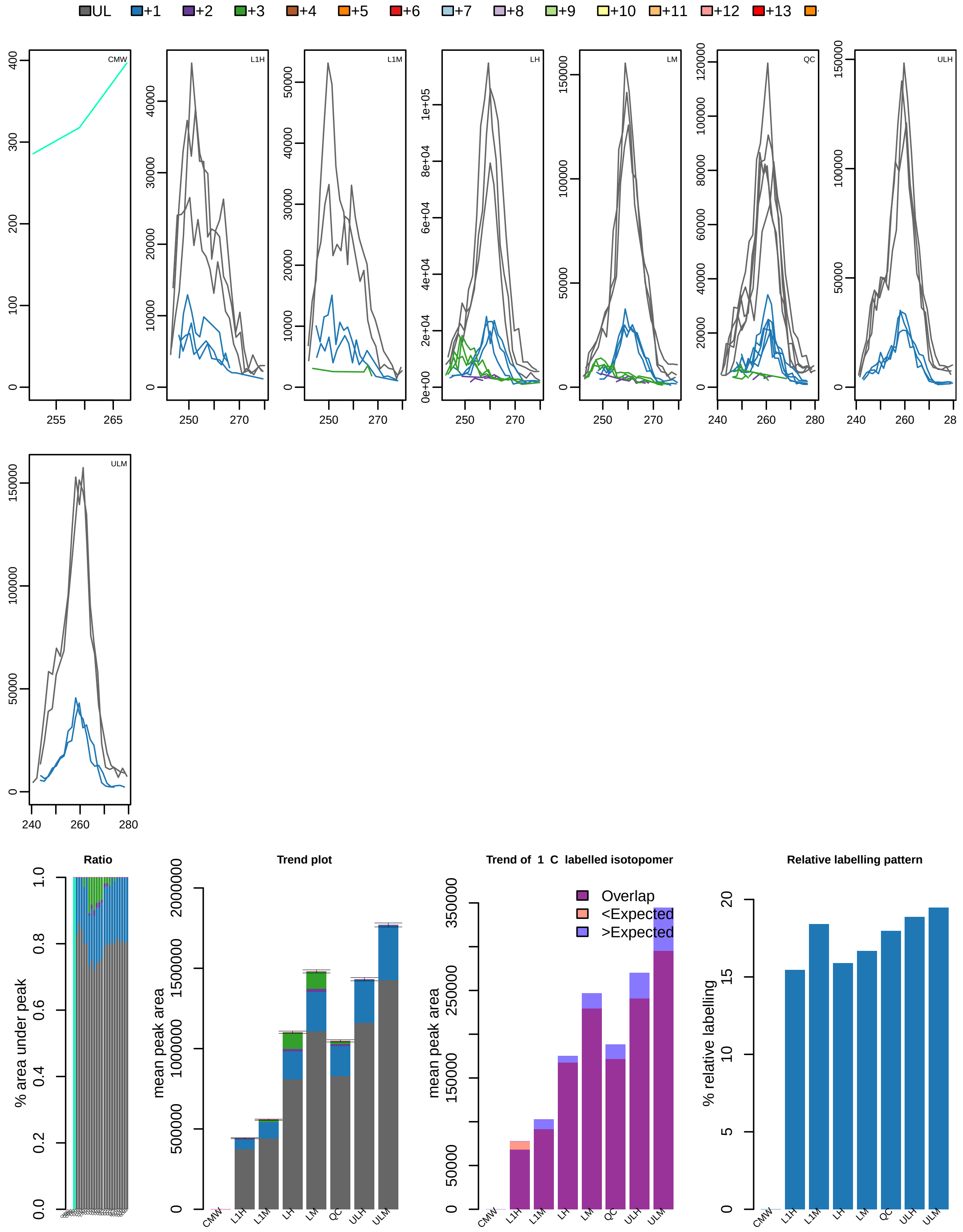

[PE (18:0/22:6)] 1−octadecanoyl−2−(4Z,7Z,10Z,13Z,16

Formula: C45H78NO8P Mass: 791.547 Std.RT: 223.58057148 Ion: P

G1

UL +1 +2 +3 +4 +5 +6 +7 +8 +9 +10 +11 +12 +13

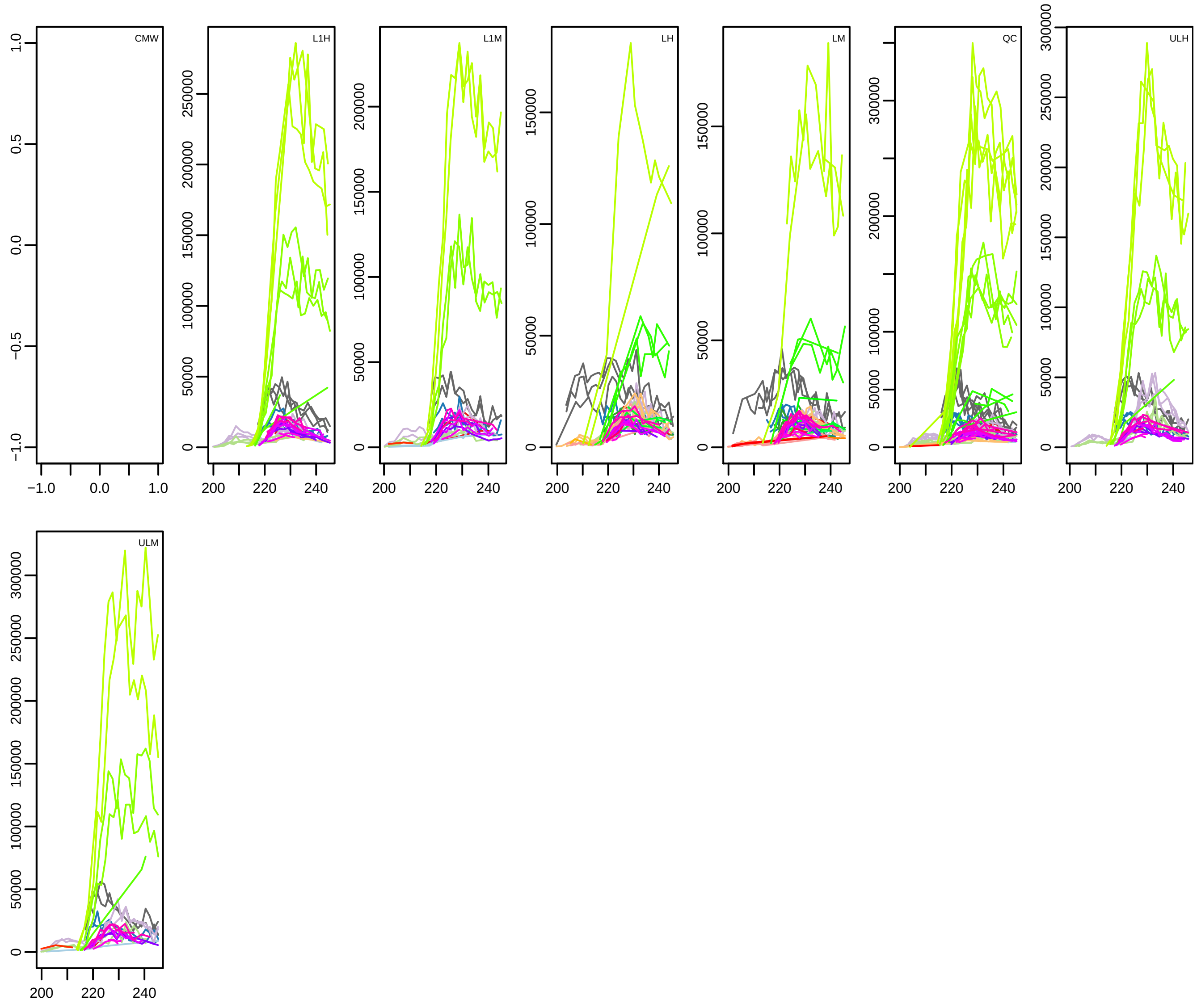

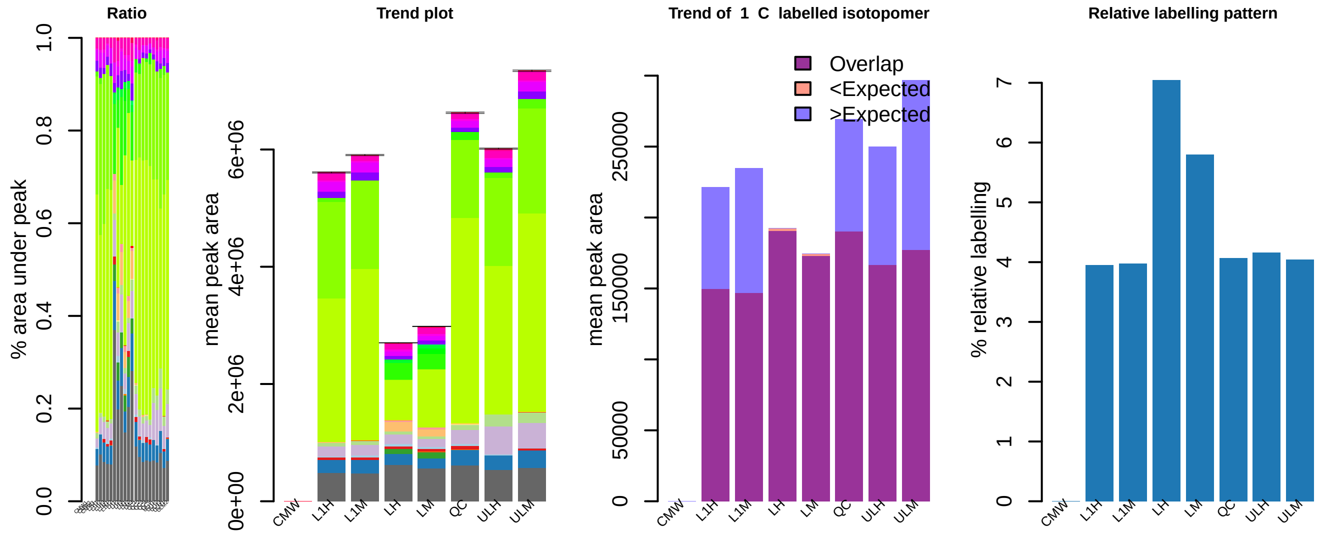

PE(20:0/22:6(4Z,7Z,10Z,13Z,16Z,19Z))

Formula: C47H82NO8P Mass: 819.578 Std.RT: 228.68747532 Ion: P

G1

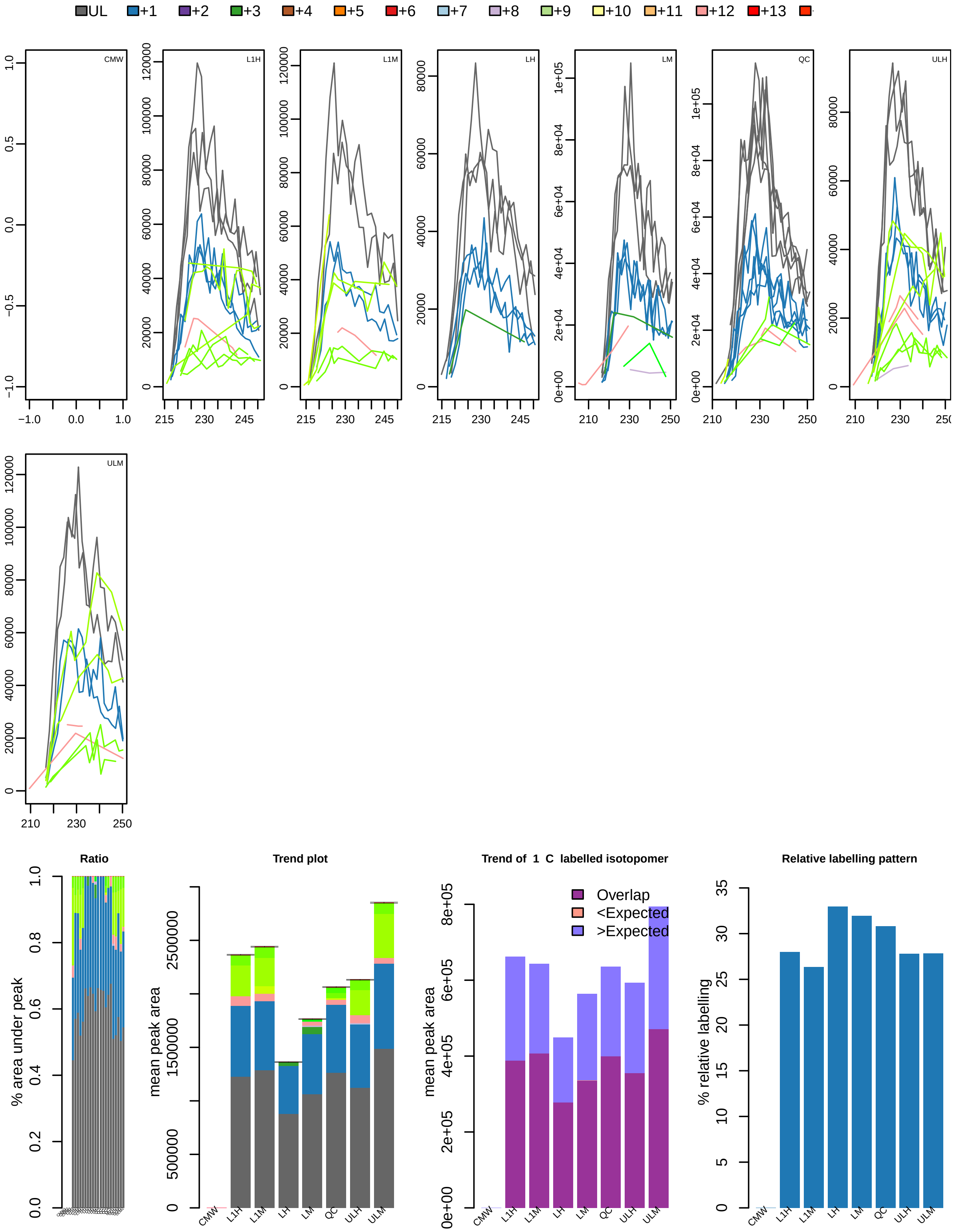

PE(20:1(11Z)/P−18:1(11Z))

Formula: C43H82NO7P Mass: 755.583 Std.RT: 231.87614298 Ion: P

G1

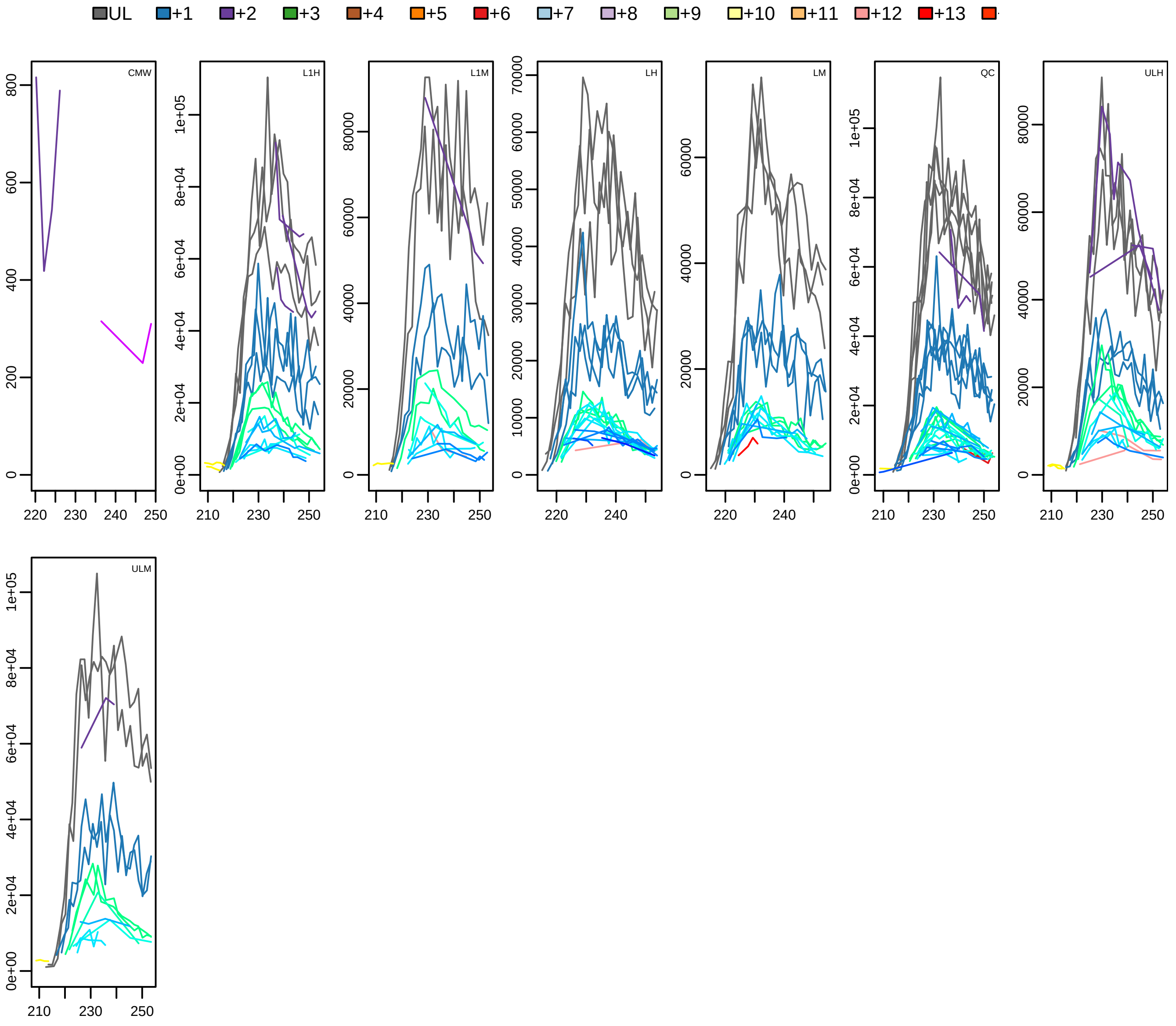

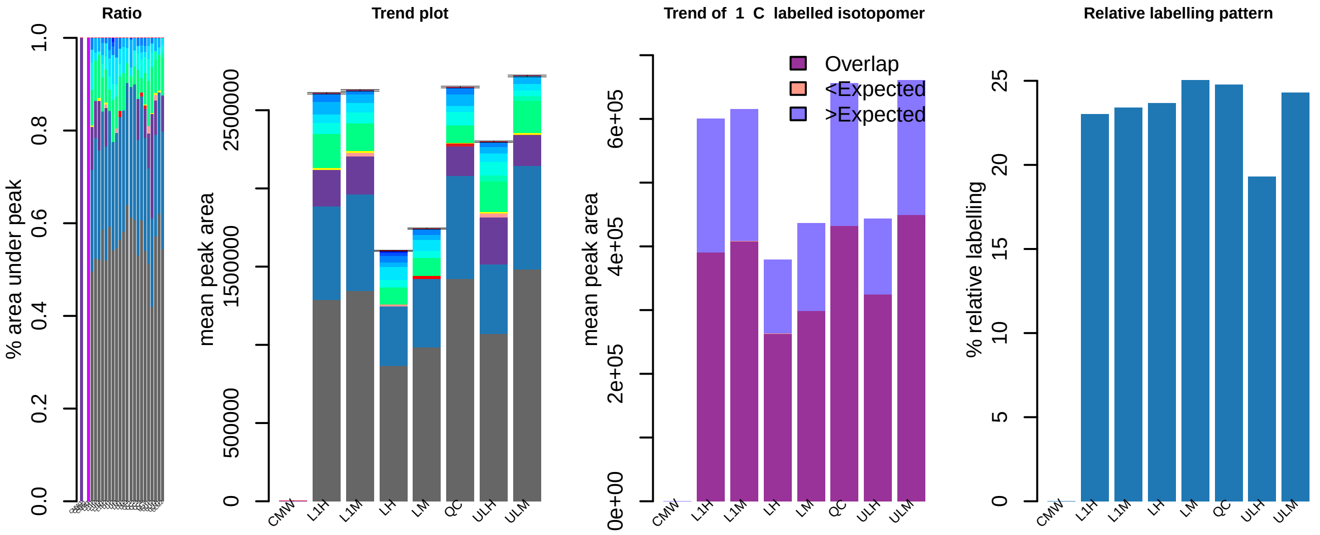

[PE (16:1)] 1−(1Z−hexadecenyl)−sn−glycero−3−phospl

Formula: C21H44NO6P Mass: 437.291 Std.RT: 255.41019042 Ion: P

G1

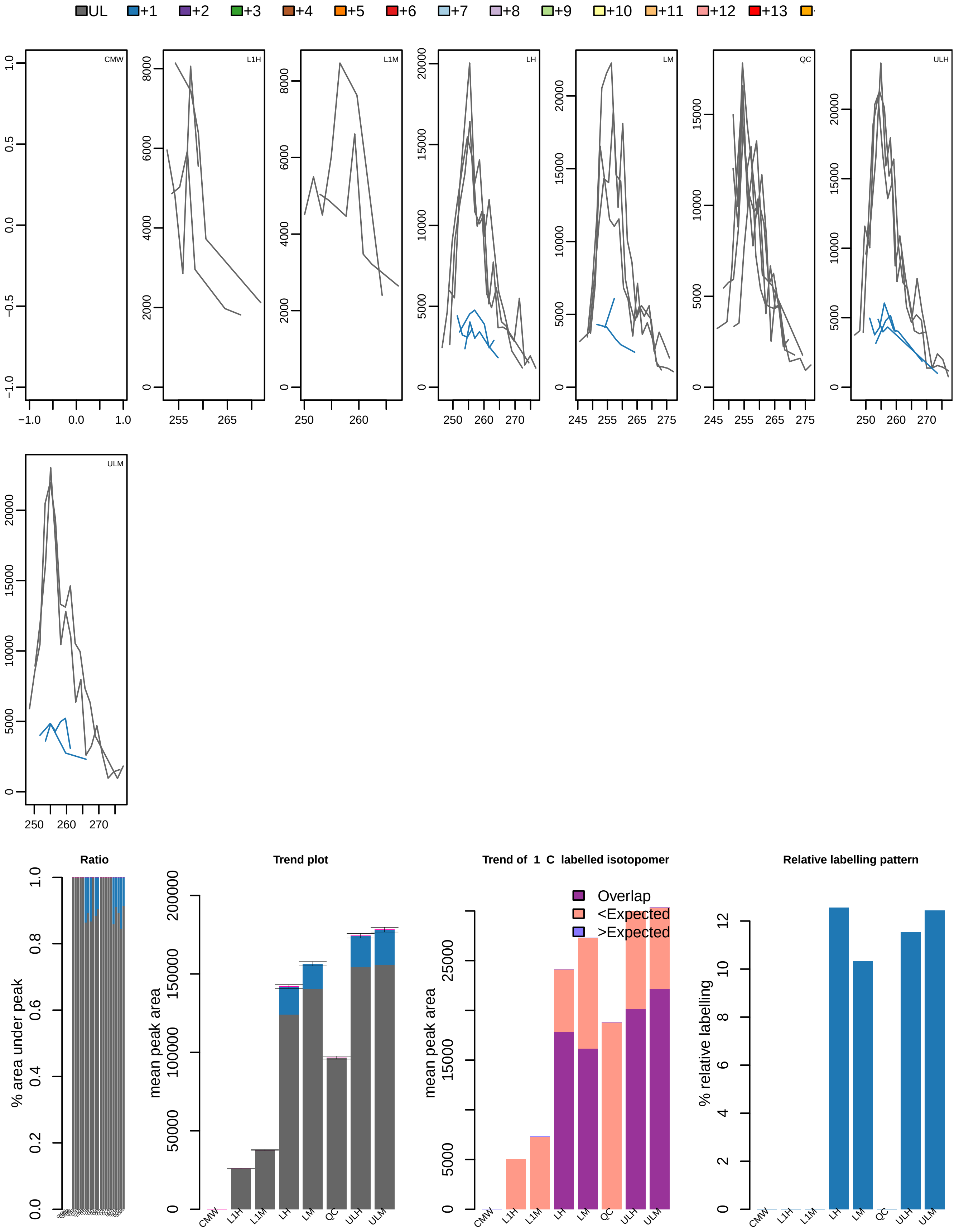

PE(16:1(9Z)/P−18:1(11Z))

Formula: C39H74NO7P Mass: 699.52 Std.RT: 223.49804832 Ion: PC

G1

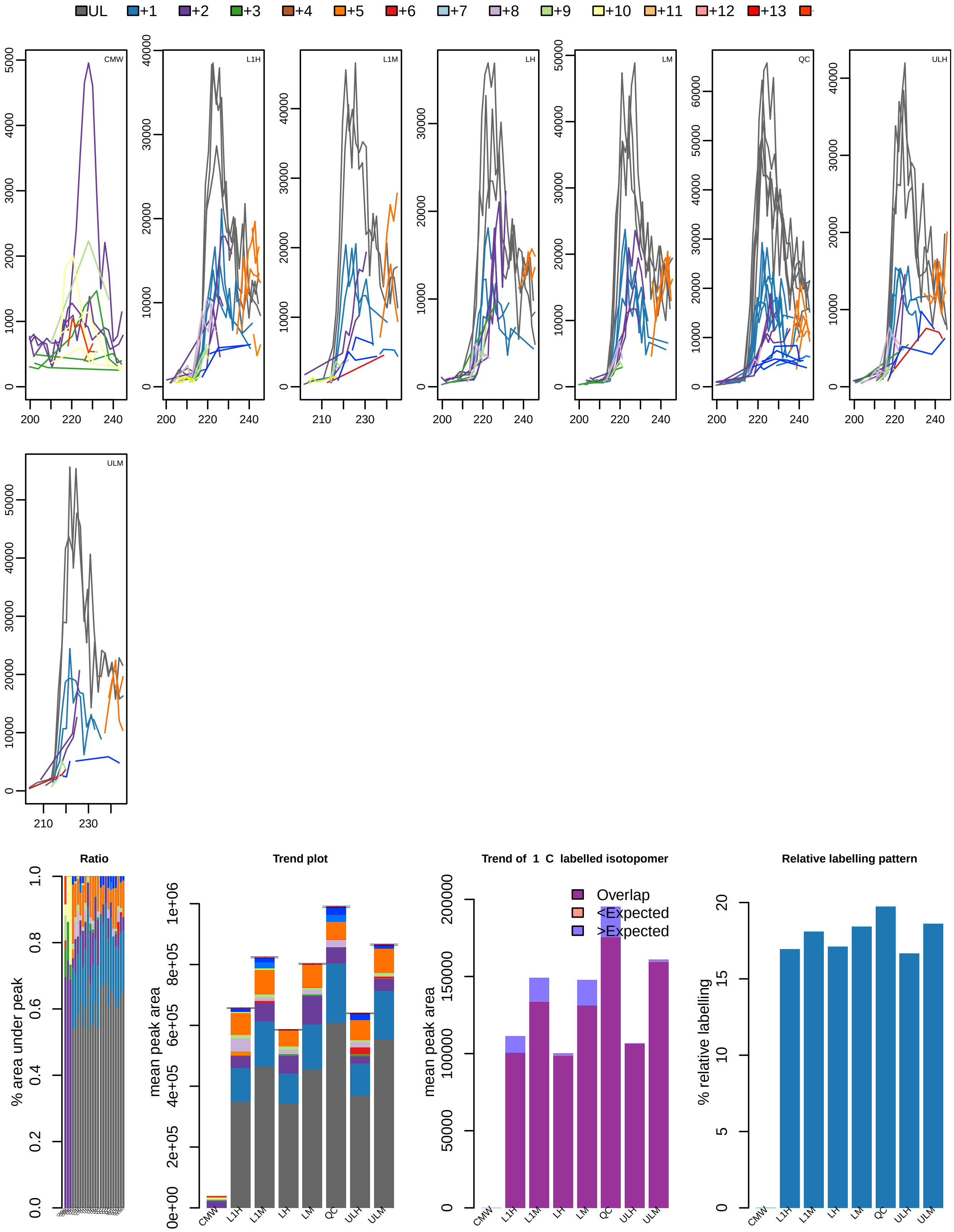

PE(18:2(9Z,12Z)/24:1(15Z))

Formula: $C_{47}H_{88}NO_8P$ Mass: 825.625 Std.RT: 231.6409992 Ion: PC

G1

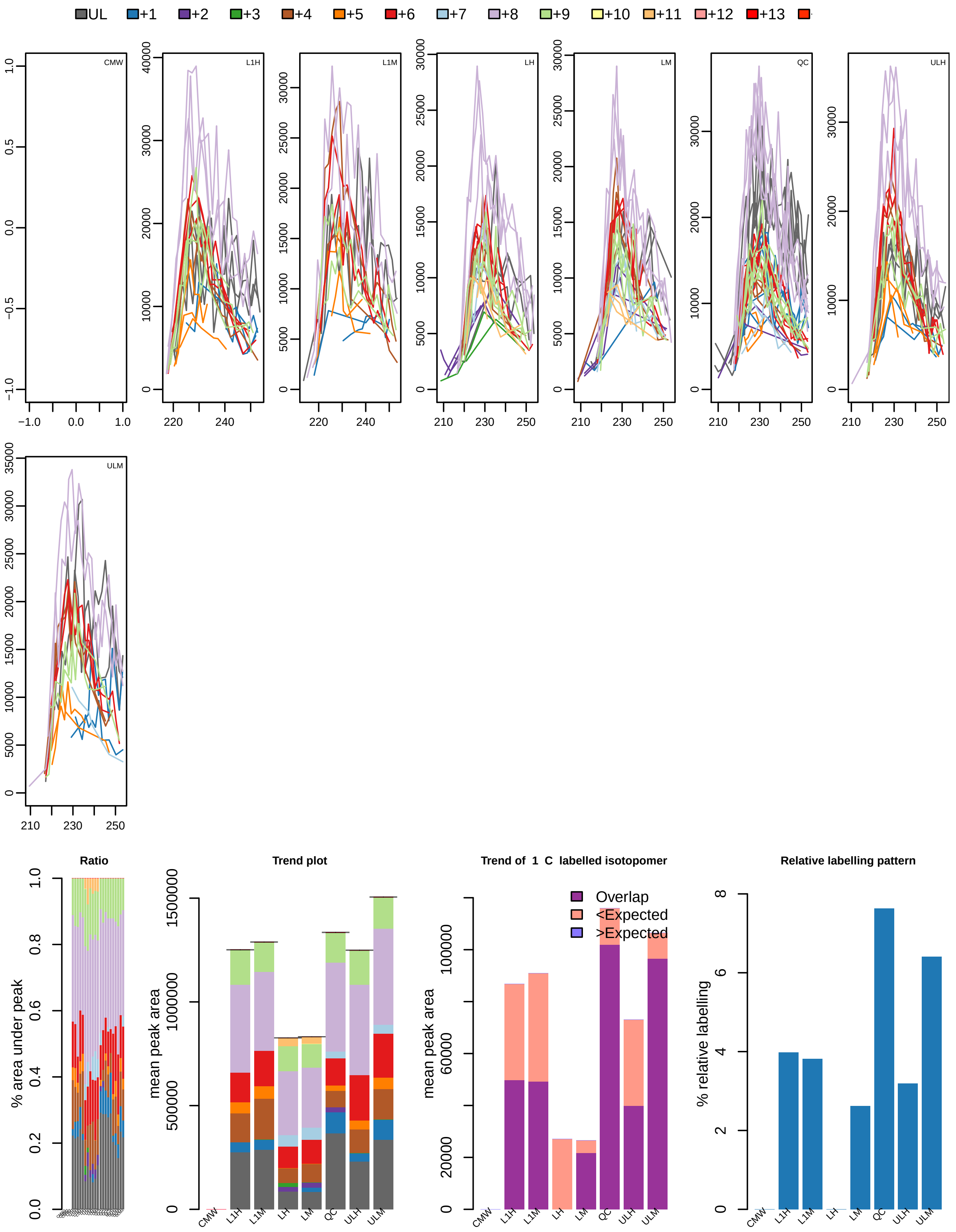

PE(18:0/22:4(7Z,10Z,13Z,16Z))

Formula: C45H82NO8P Mass: 795.578 Std.RT: 225.079143 Ion: PO[illegible]

G1

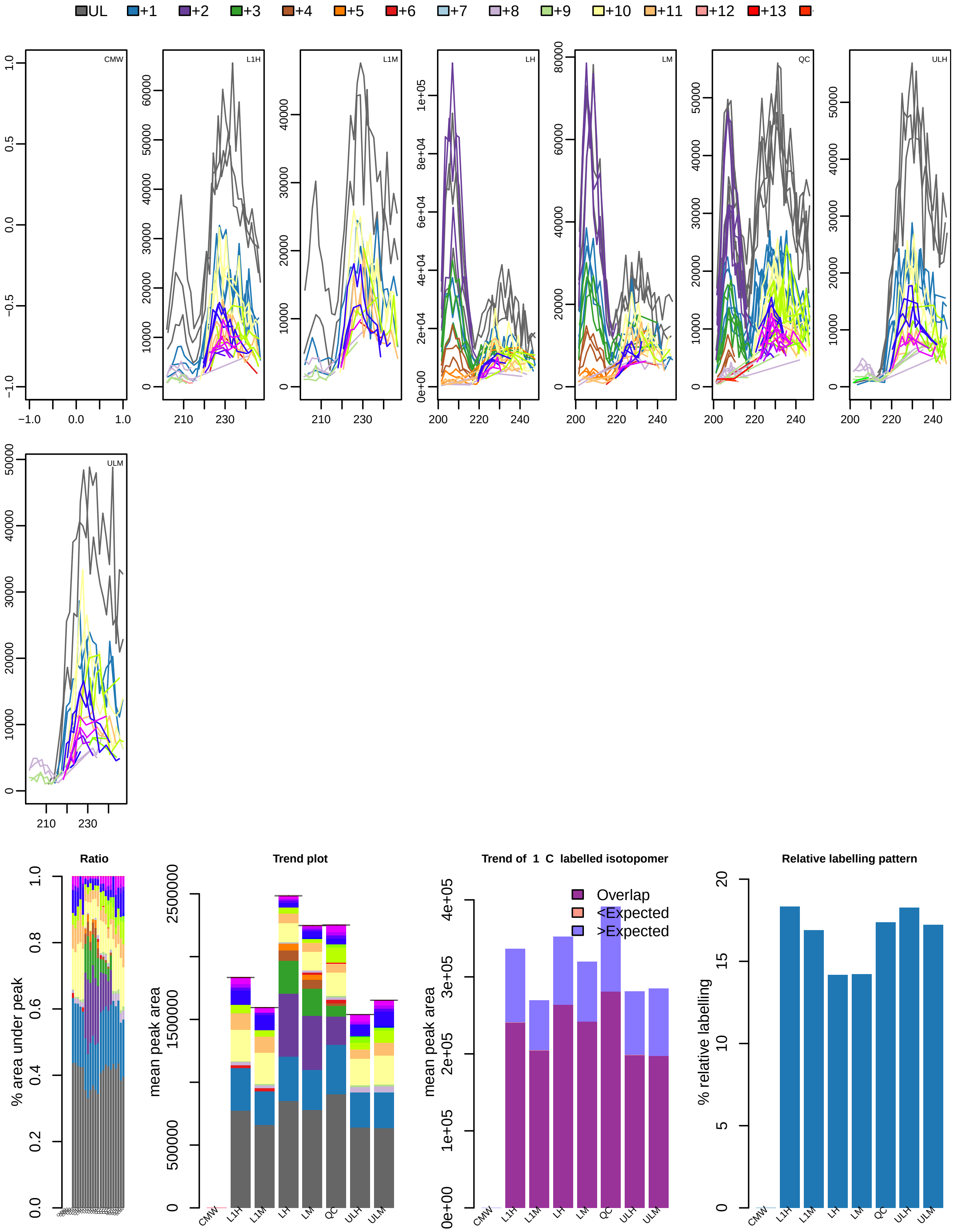

1−Hexadecanoyl−2−(9Z−octadecenoyl)−sn−glycero−3-

Formula: C42H82NO7P Mass: 743.583 Std.RT: 233.30866638 Ion: P

G1

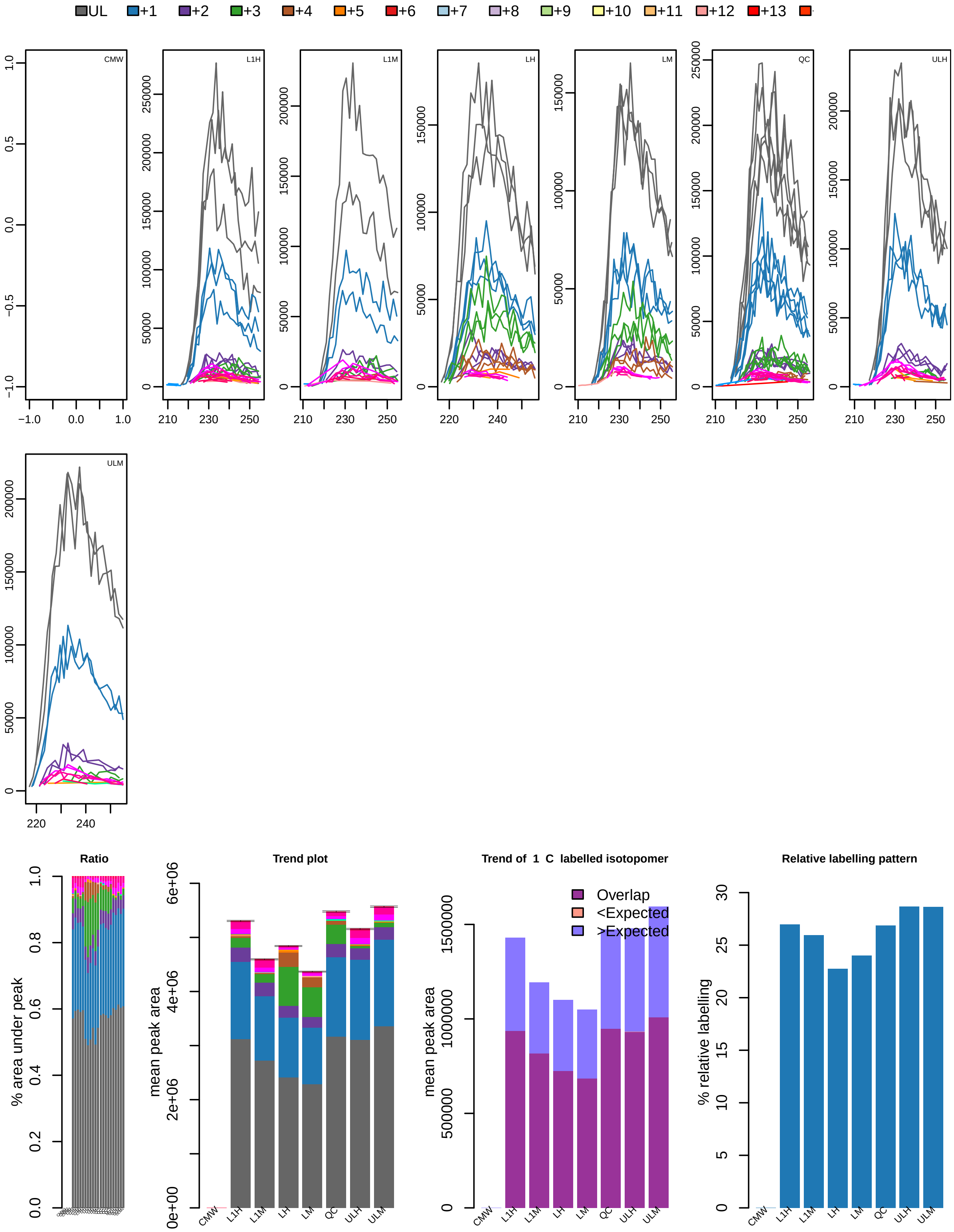

[PS (18:0)] 1−octadecanoyl−sn−glycero−3−phosphoser

Formula: C24H48NO9P Mass: 525.307 Std.RT: 214.87859802 Ion: P

G1

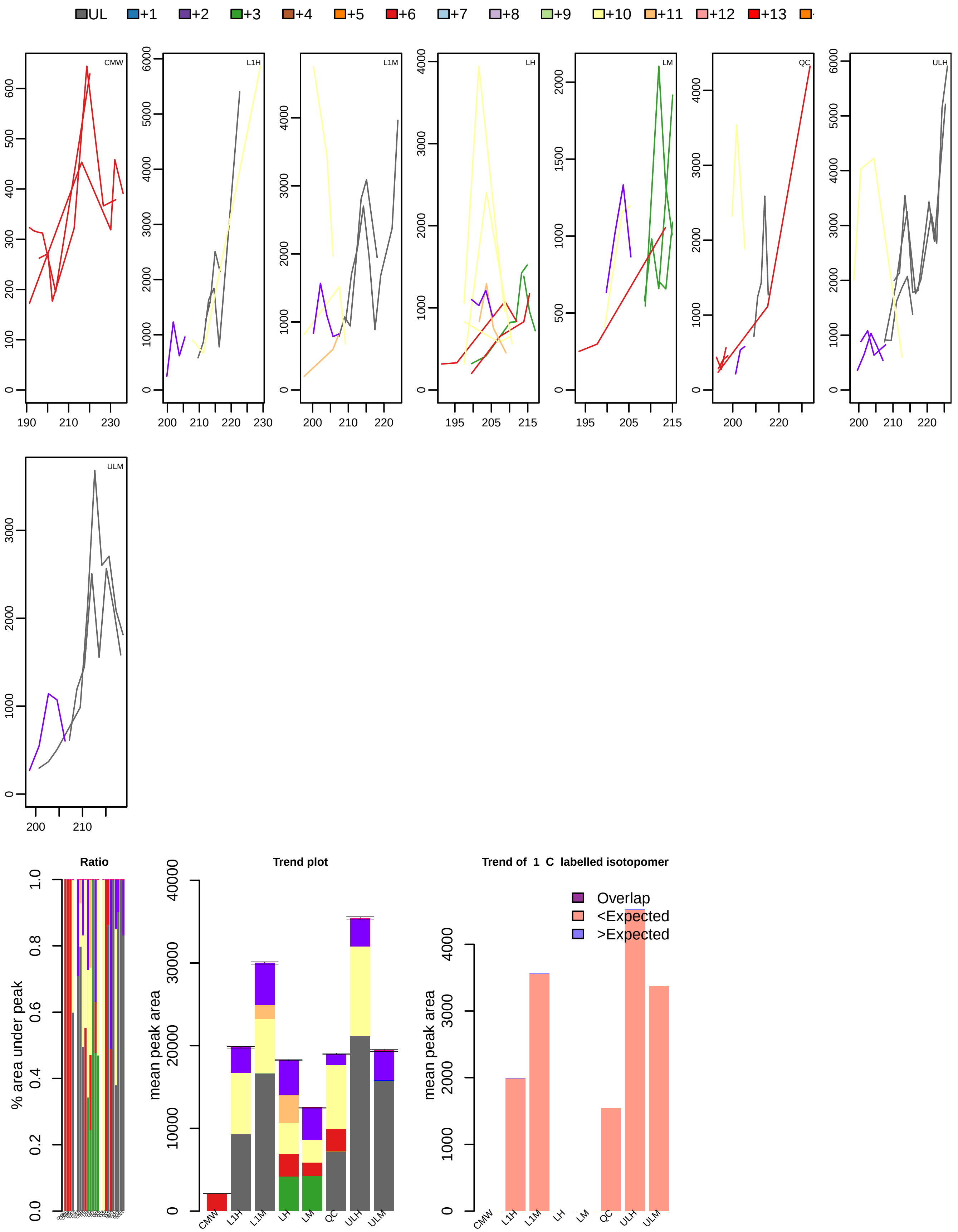

PS(18:0/20:0)

Formula: C44H86NO10P Mass: 819.599 Std.RT: 205.49023872 Ion:

G1

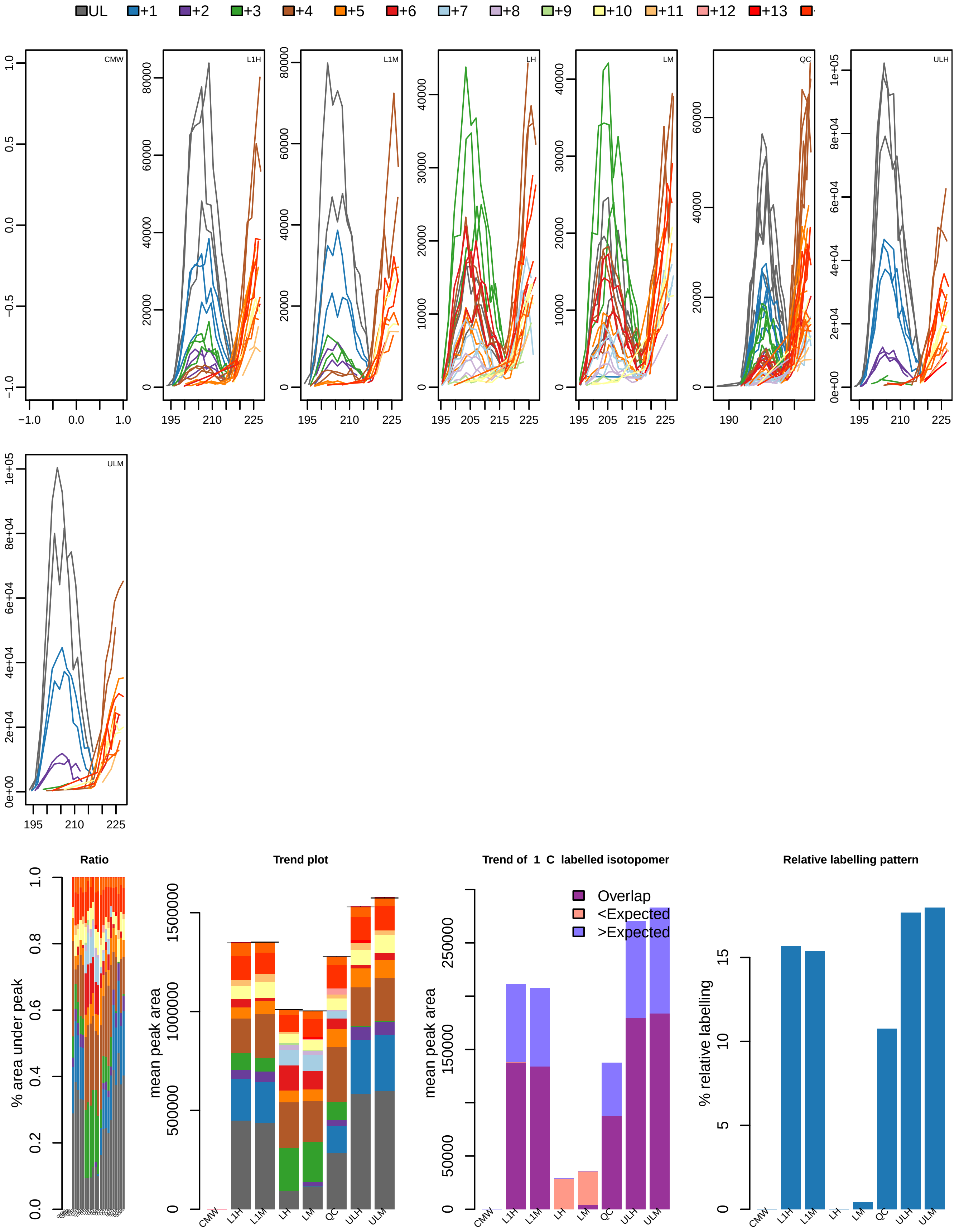

[PS (16:0/20:0)] 1−hexadecanoyl−2−eicosanoyl−sn−gly

Formula: C42H82NO10P Mass: 791.568 Std.RT: 206.97457092 Ion:

G1

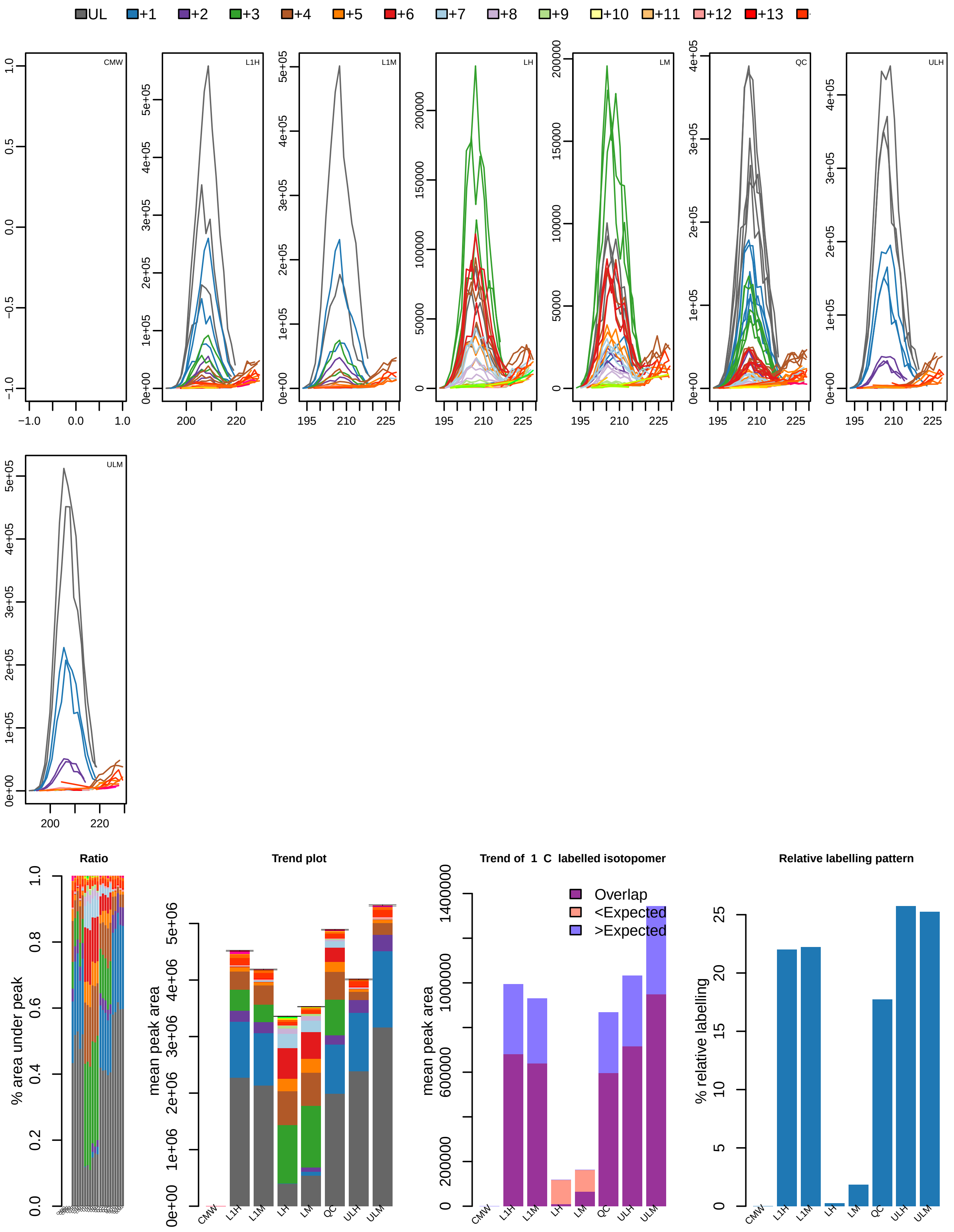

PS(18:0/18:1(9Z))

Formula: C42H80NO10P Mass: 789.552 Std.RT: 207.3781419 Ion: P

G1

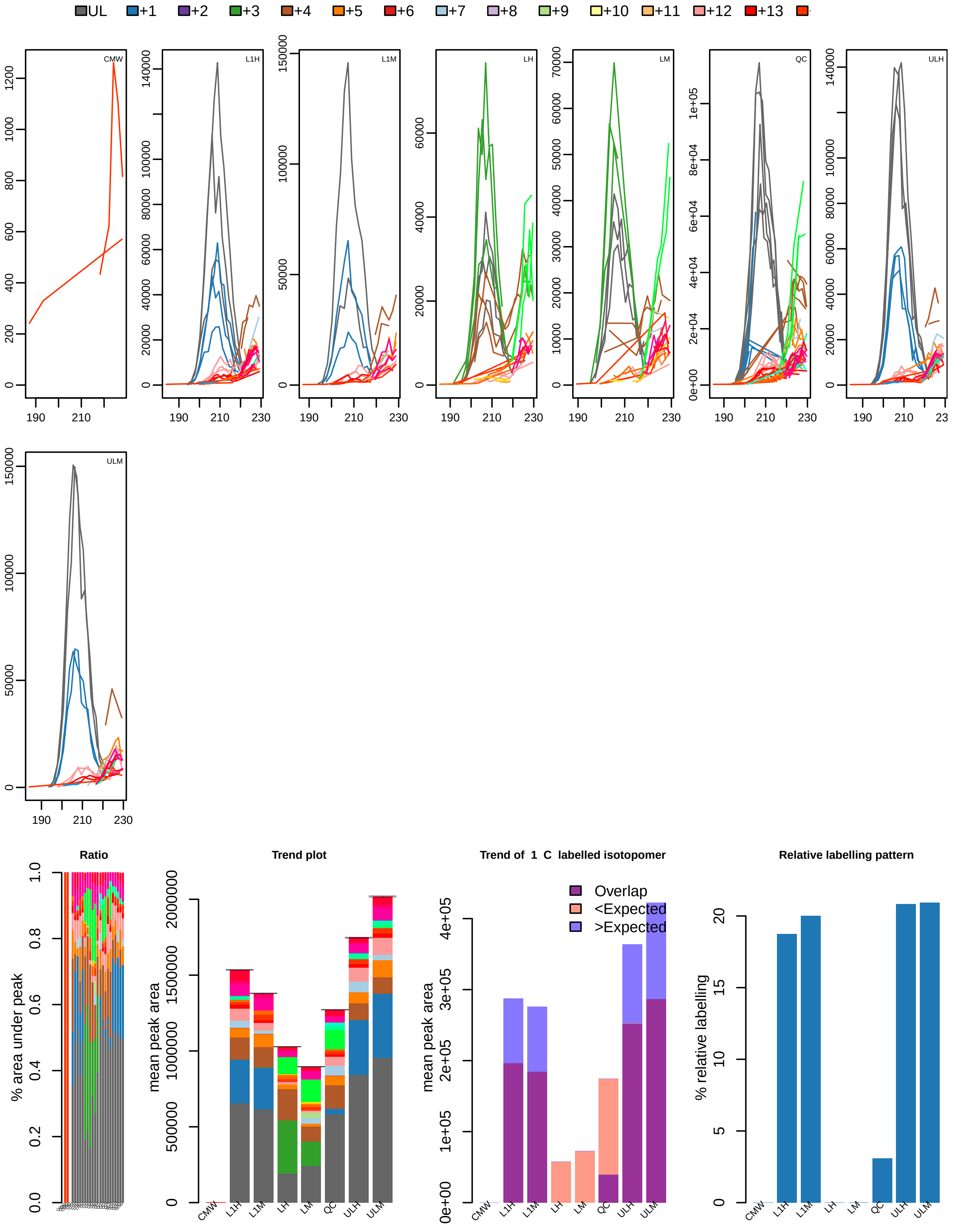

PS(16:0/18:0)

G1

Formula: C40H78NO10P Mass: 763.536 Std.RT: 207.2240997 Ion: P

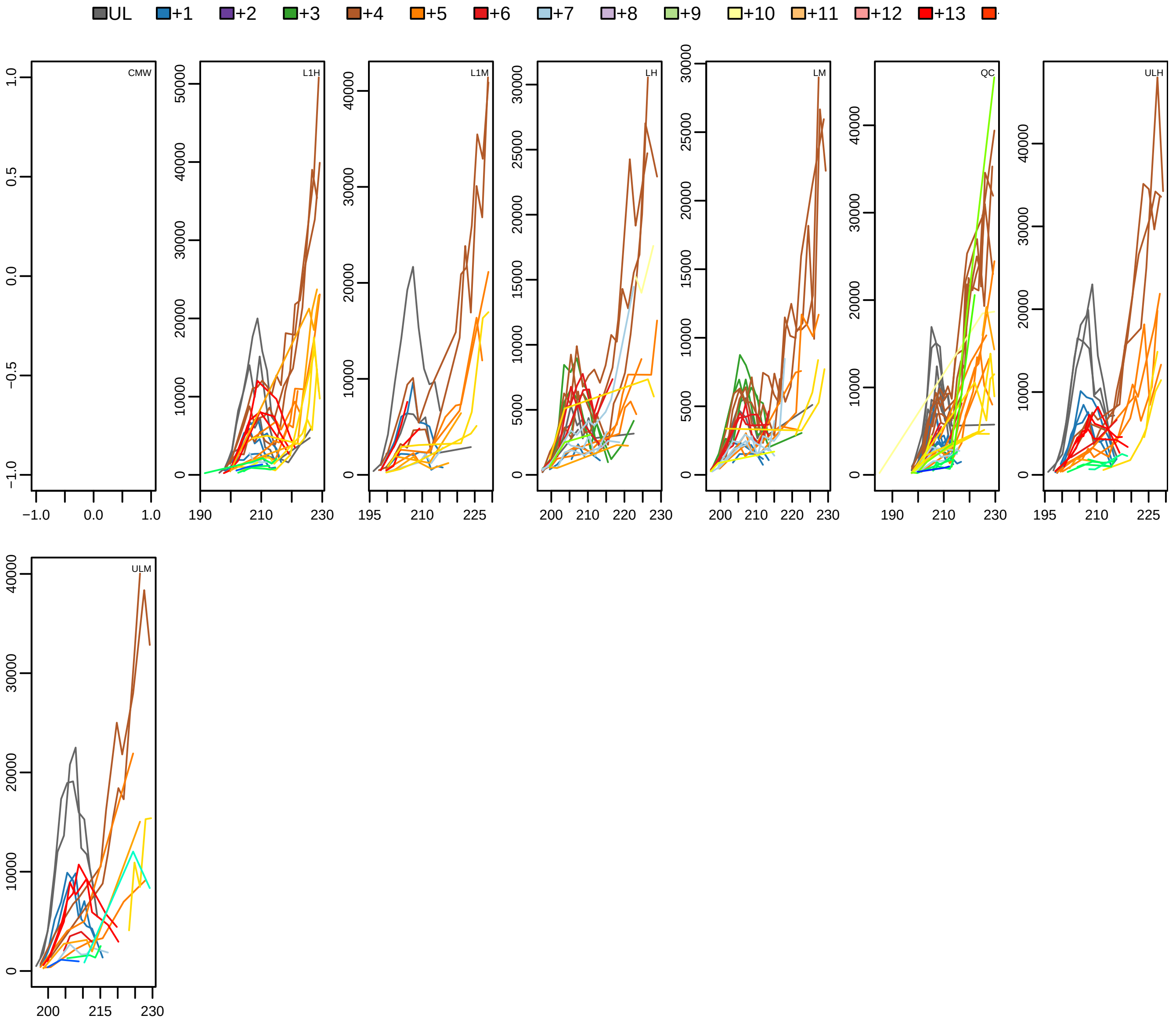

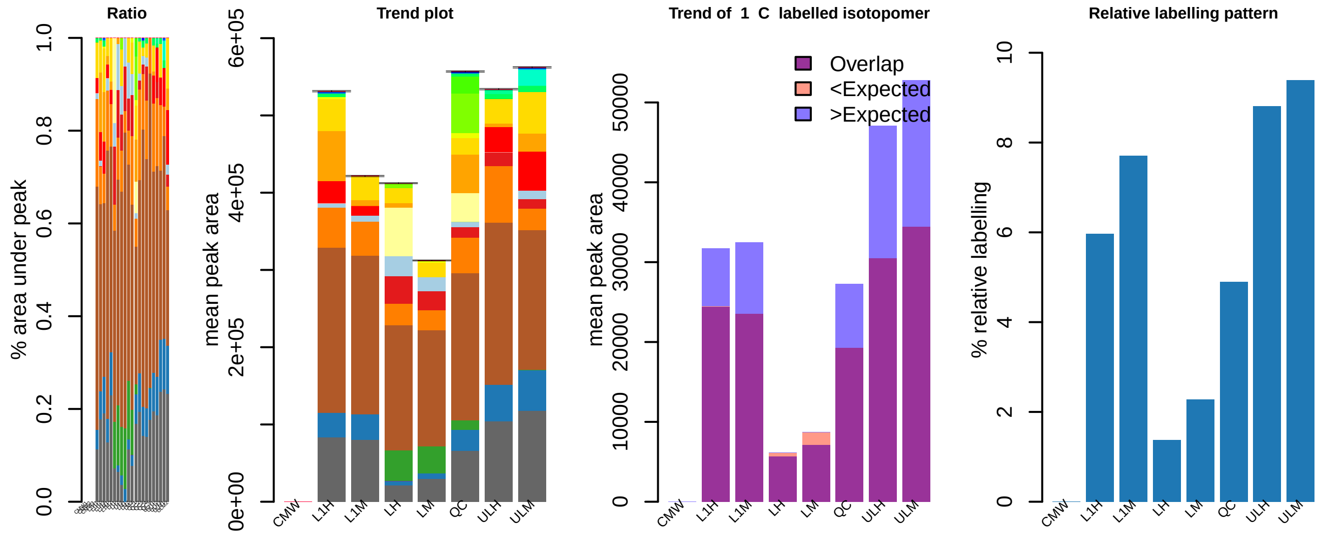

[PS (18:1/18:1)] 1,2−di−(9E−octadecenoyl)−sn−glycero

Formula: C42H78NO10P Mass: 787.536 Std.RT: 209.04431922 Ion:

G1

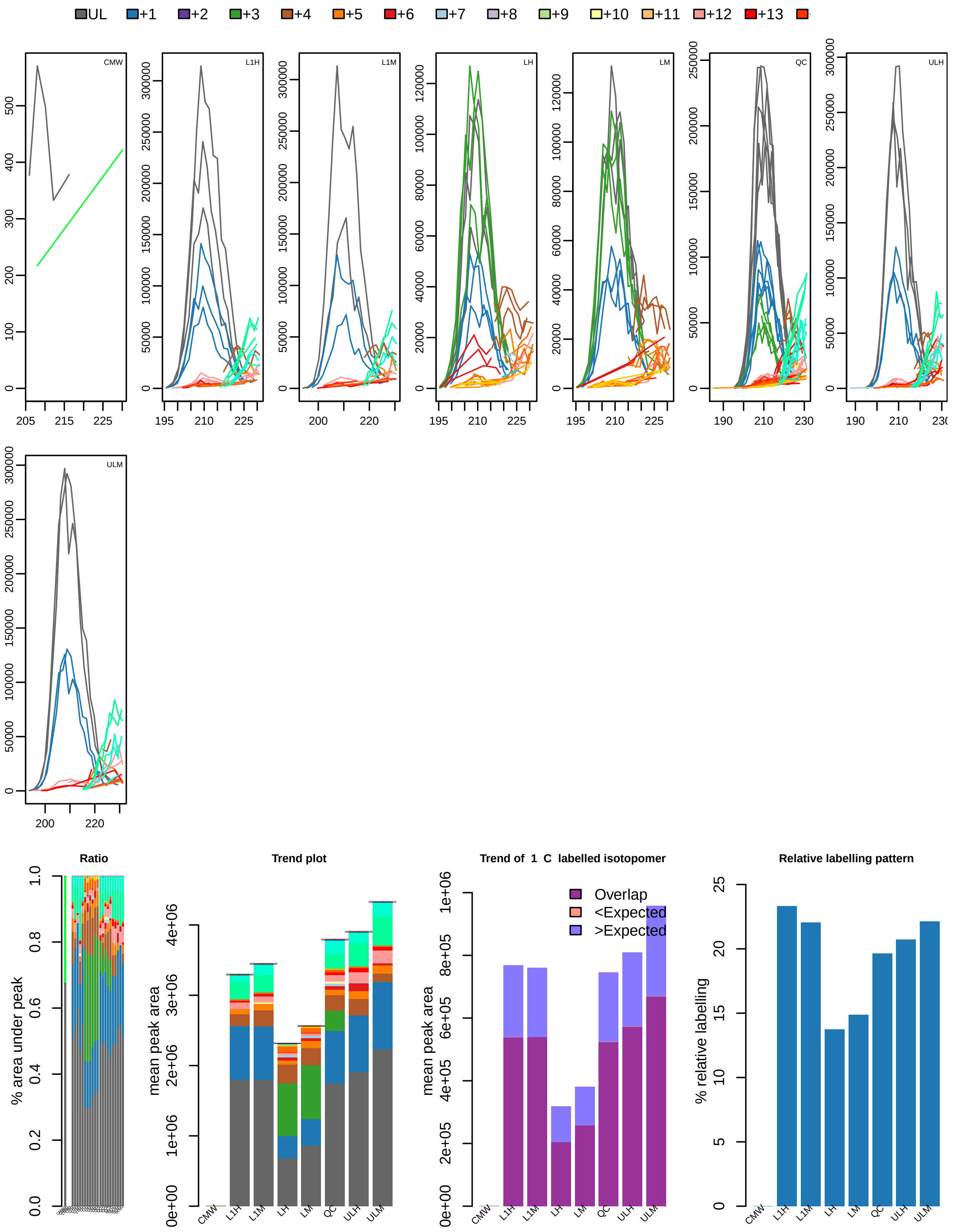

[PS (18:2/18:2)] 1,2−di−(9Z,12Z−octadecadienoyl)−sn−

G1

Formula: $C_{42}H_{74}NO_{10}P$ Mass: 783.505 Std.RT: 208.47799968 Ion:

UL +1 +2 +3 +4 +5 +6 +7 +8 +9 +10 +11 +12 +13

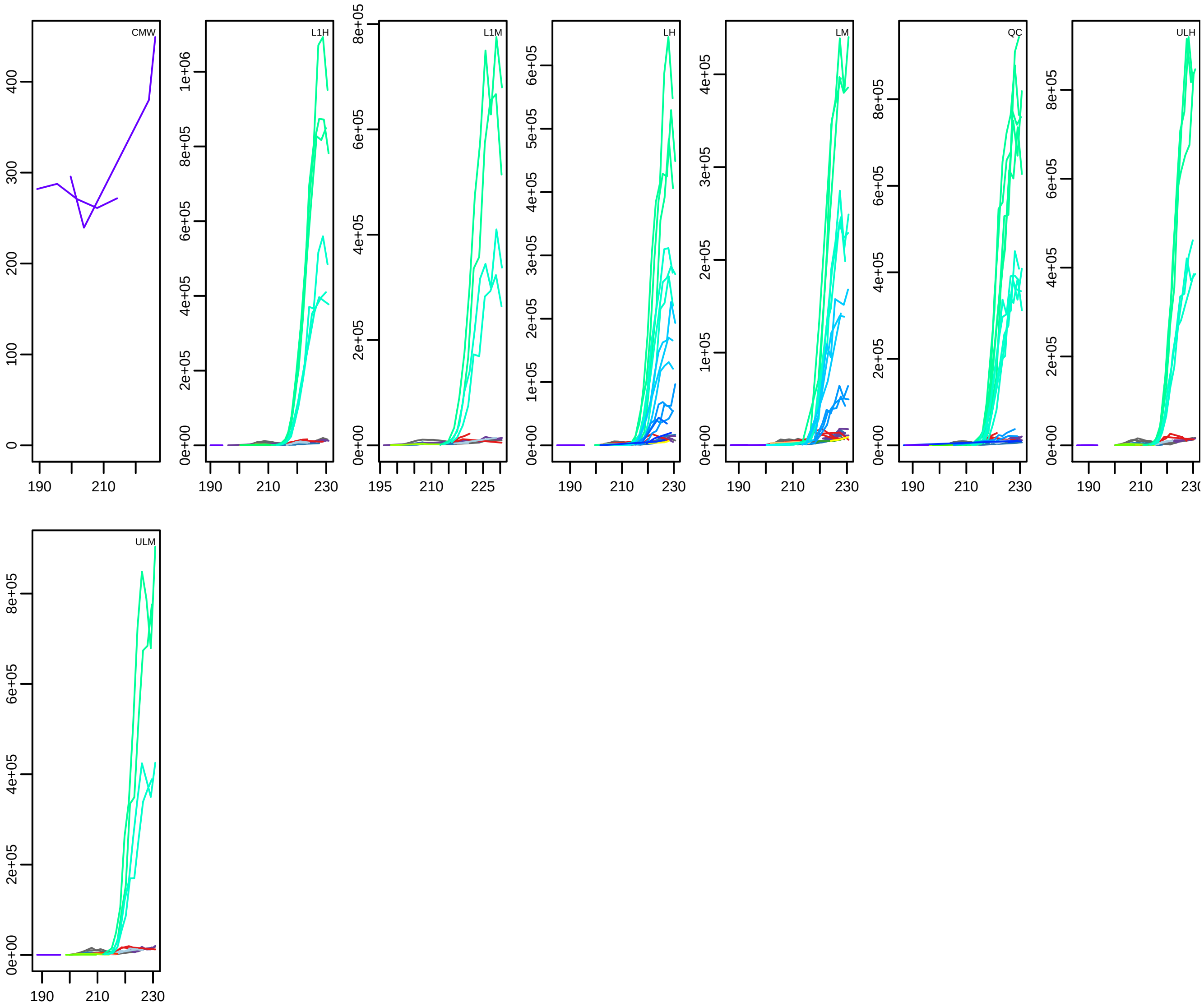

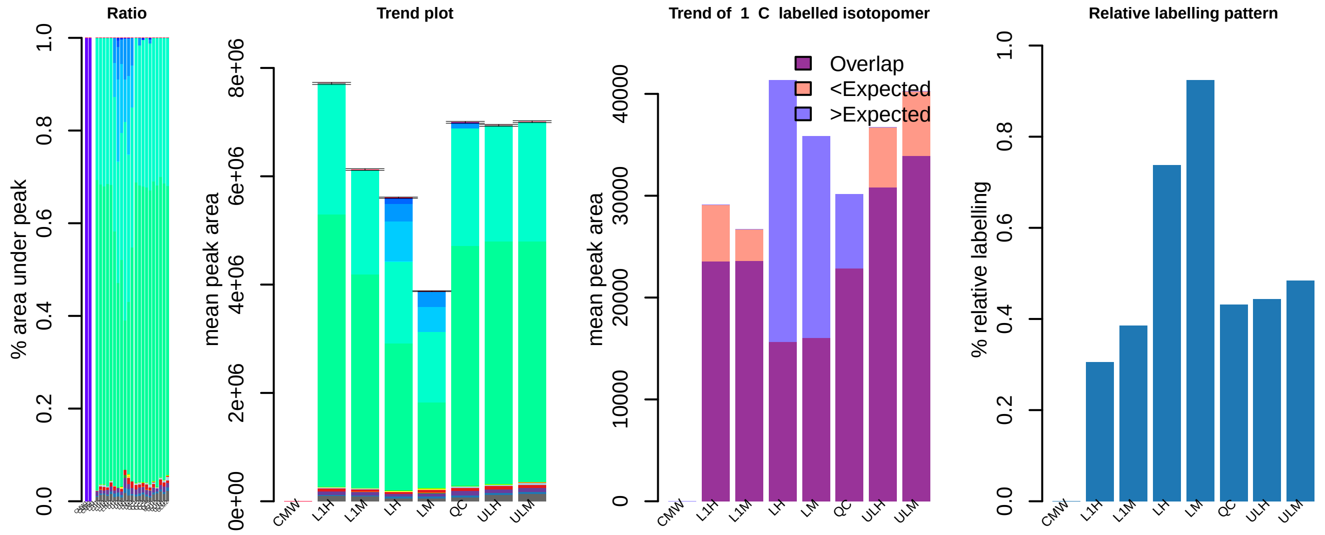

G1

[PS (18:1/18:2)] 1−(9Z−octadecenoyl)−2−(9Z,12Z−octa

Formula: C42H76NO10P Mass: 785.521 Std.RT: 208.7826204 Ion: P

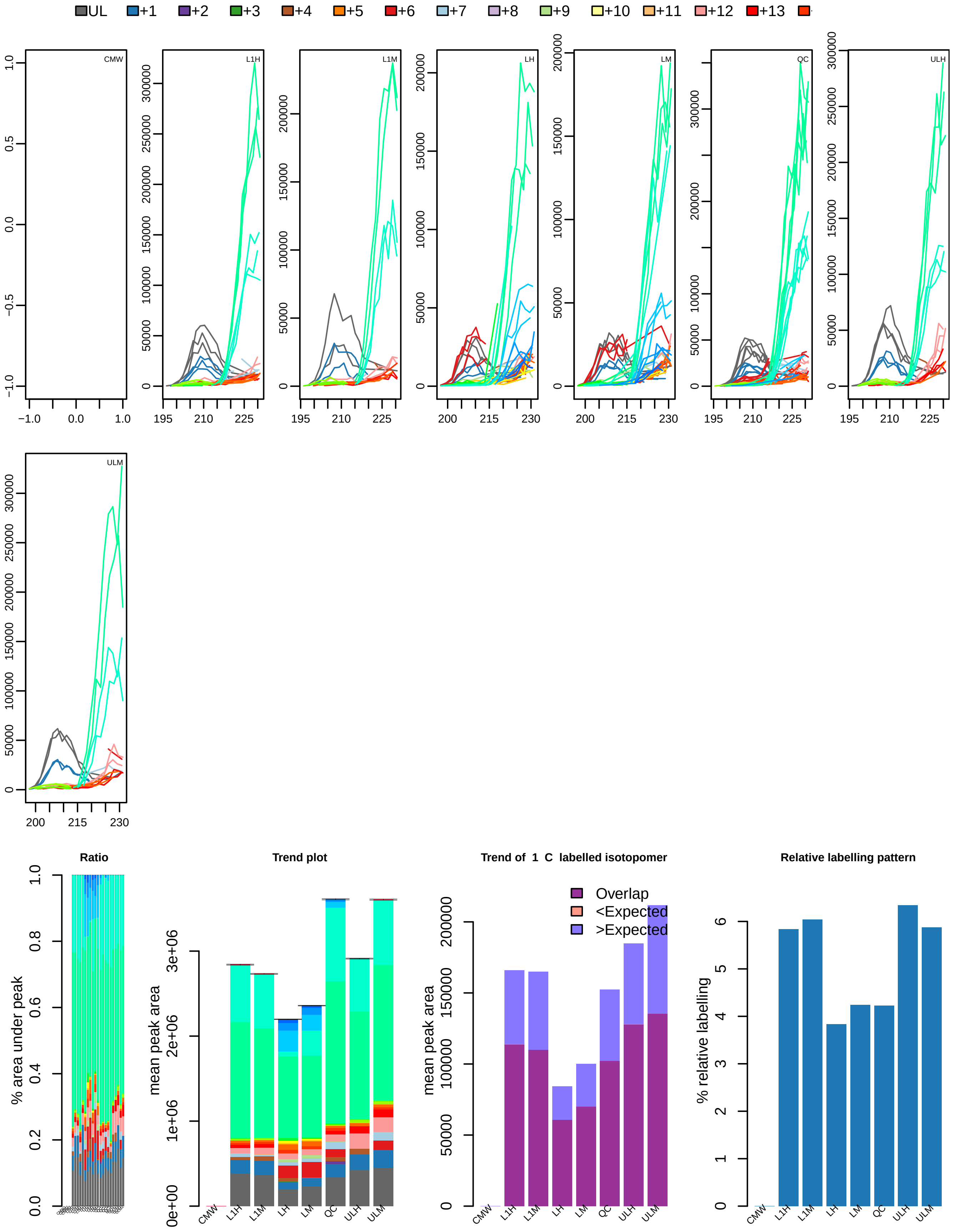

PS(18:0/22:5(7Z,10Z,13Z,16Z,19Z))

Formula: C46H80NO10P Mass: 837.552 Std.RT: 207.05271402 Ion:

G1

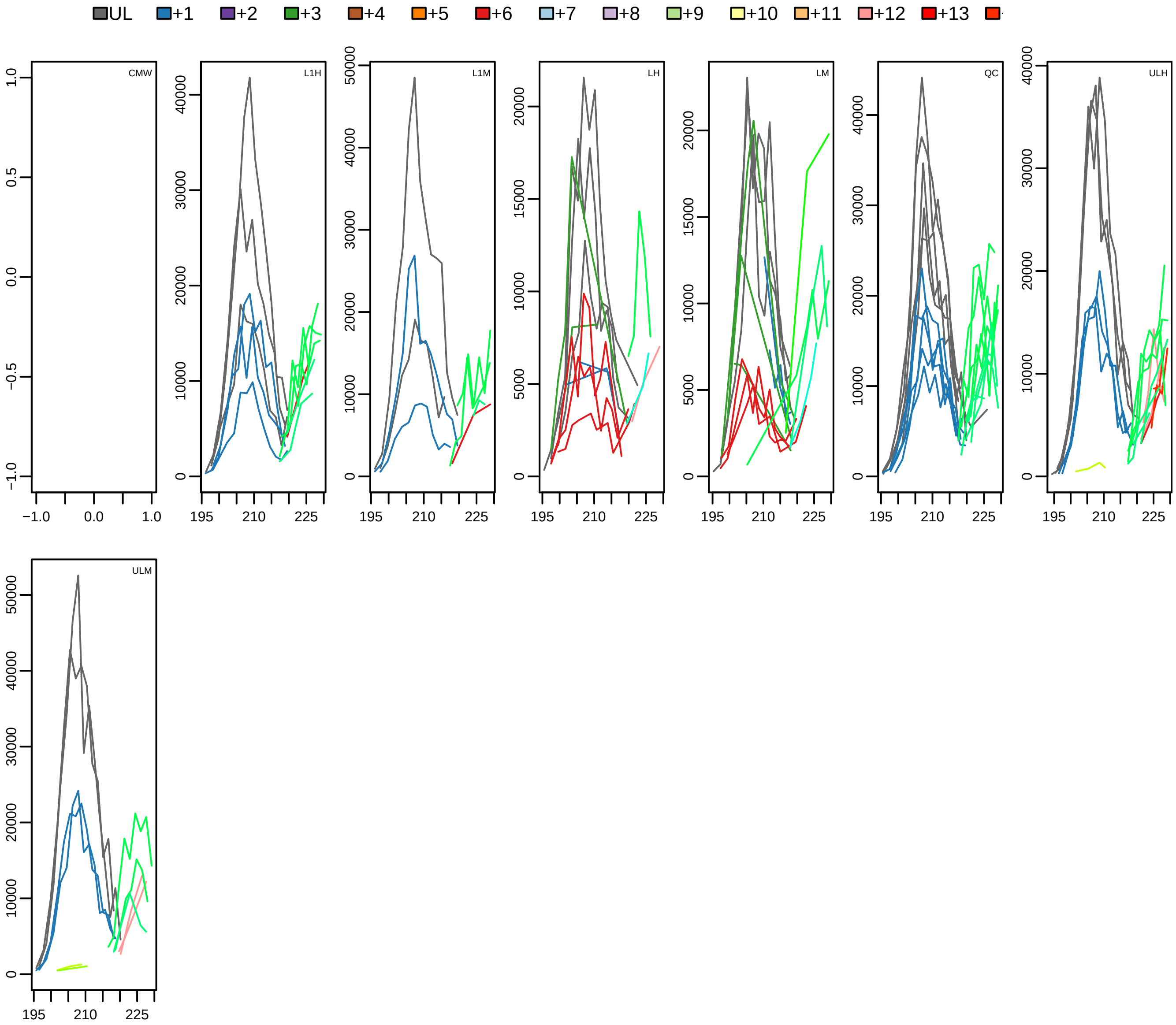

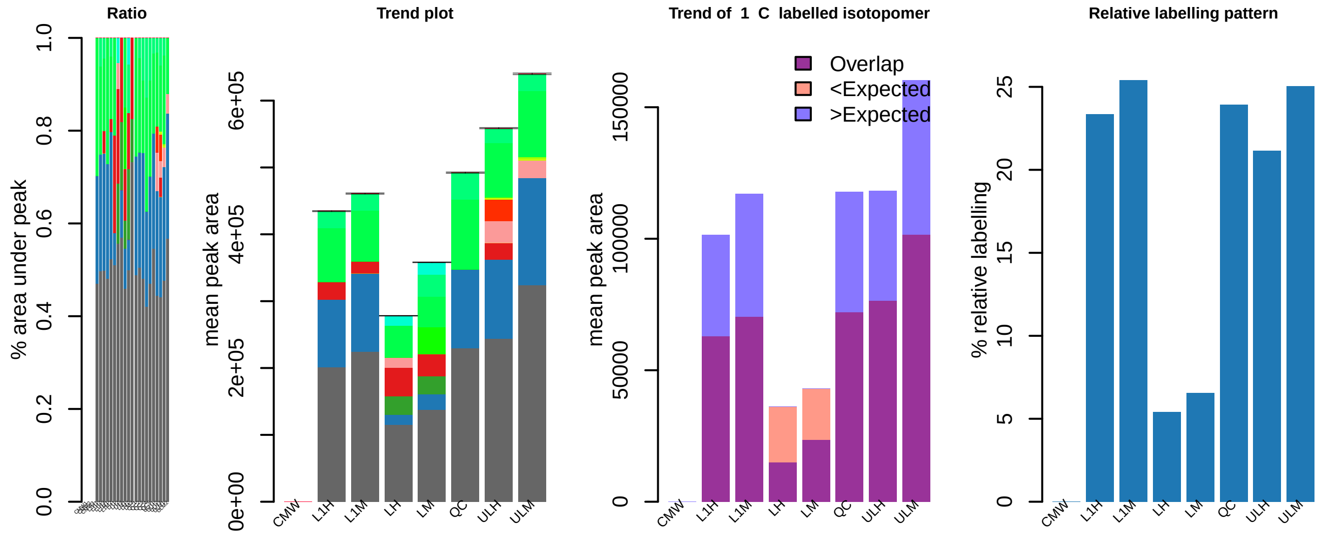

[PS (18:0/20:4)] 1−octadecanoyl−2−(5Z,8Z,11Z,14Z−ei

Formula: C44H78NO10P Mass: 811.536 Std.RT: 207.45318972 Ion:

G1

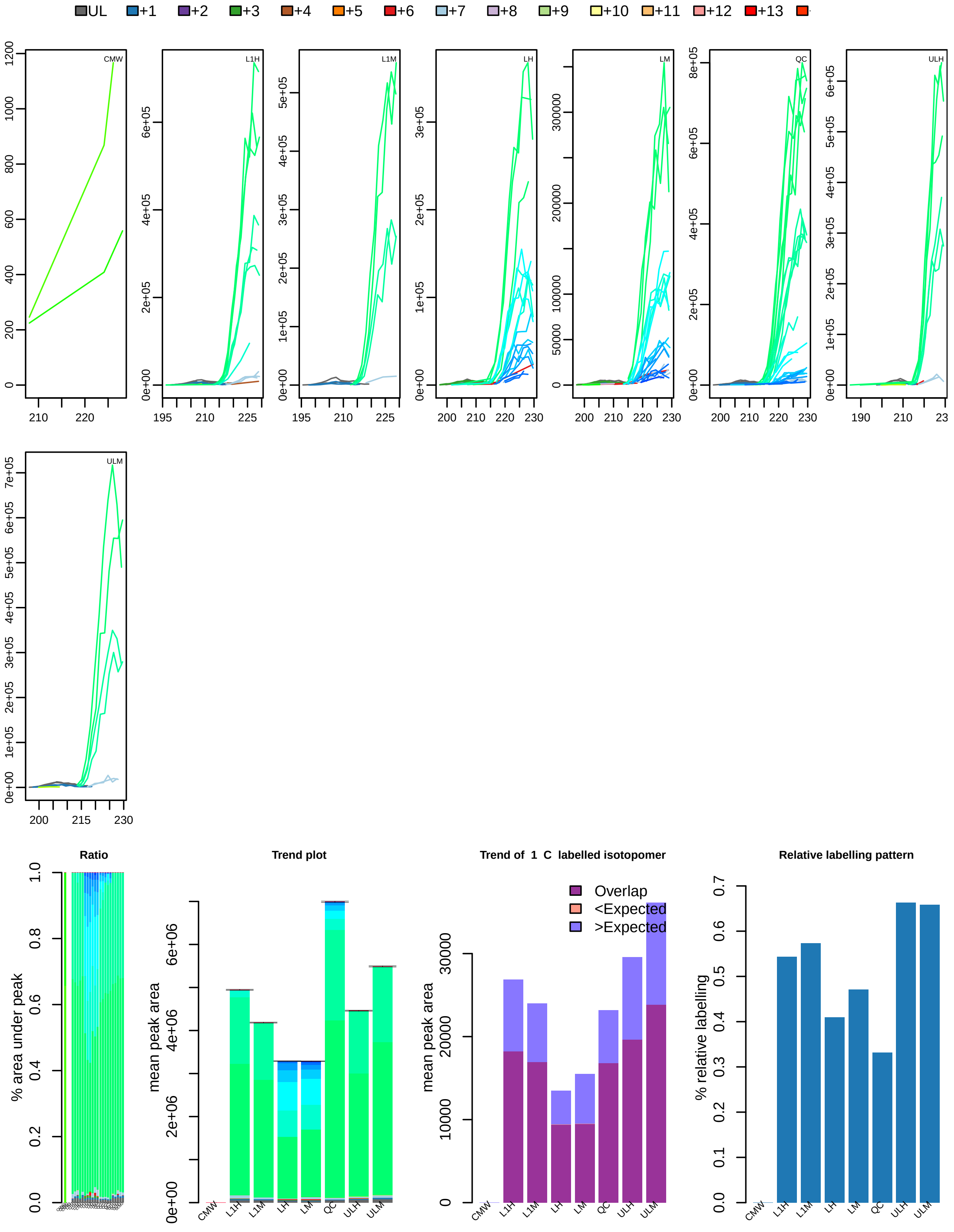

PS(16:0/18:2(9Z,12Z))

Formula: $C_{40}H_{74}NO_{10}P$ Mass: 759.505 Std.RT: 209.85457362 Ion:

G1

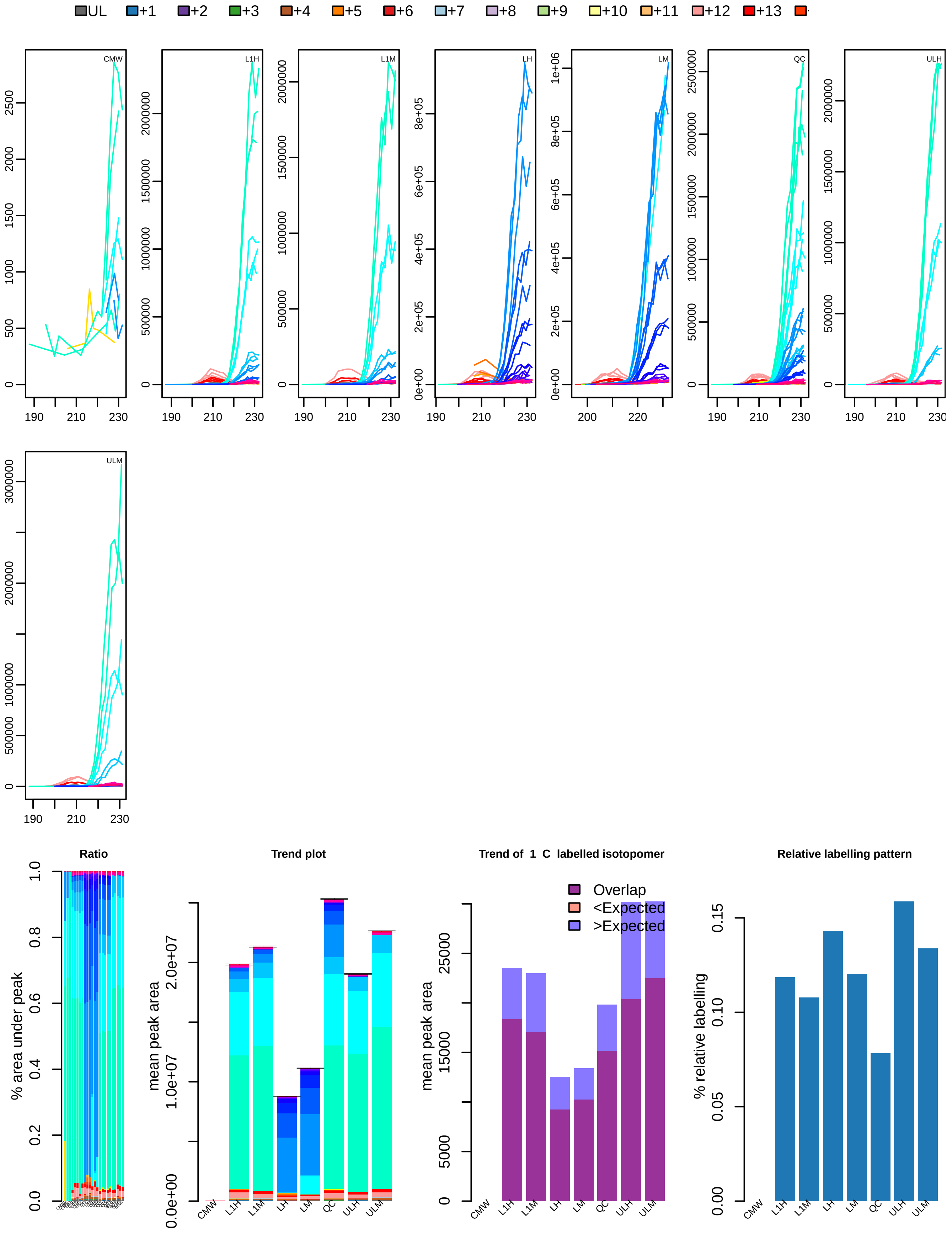

[PK] Chrysophanol

Formula: C15H10O4 Mass: 254.058 Std.RT: 427.44328818 Ion: POS

G1

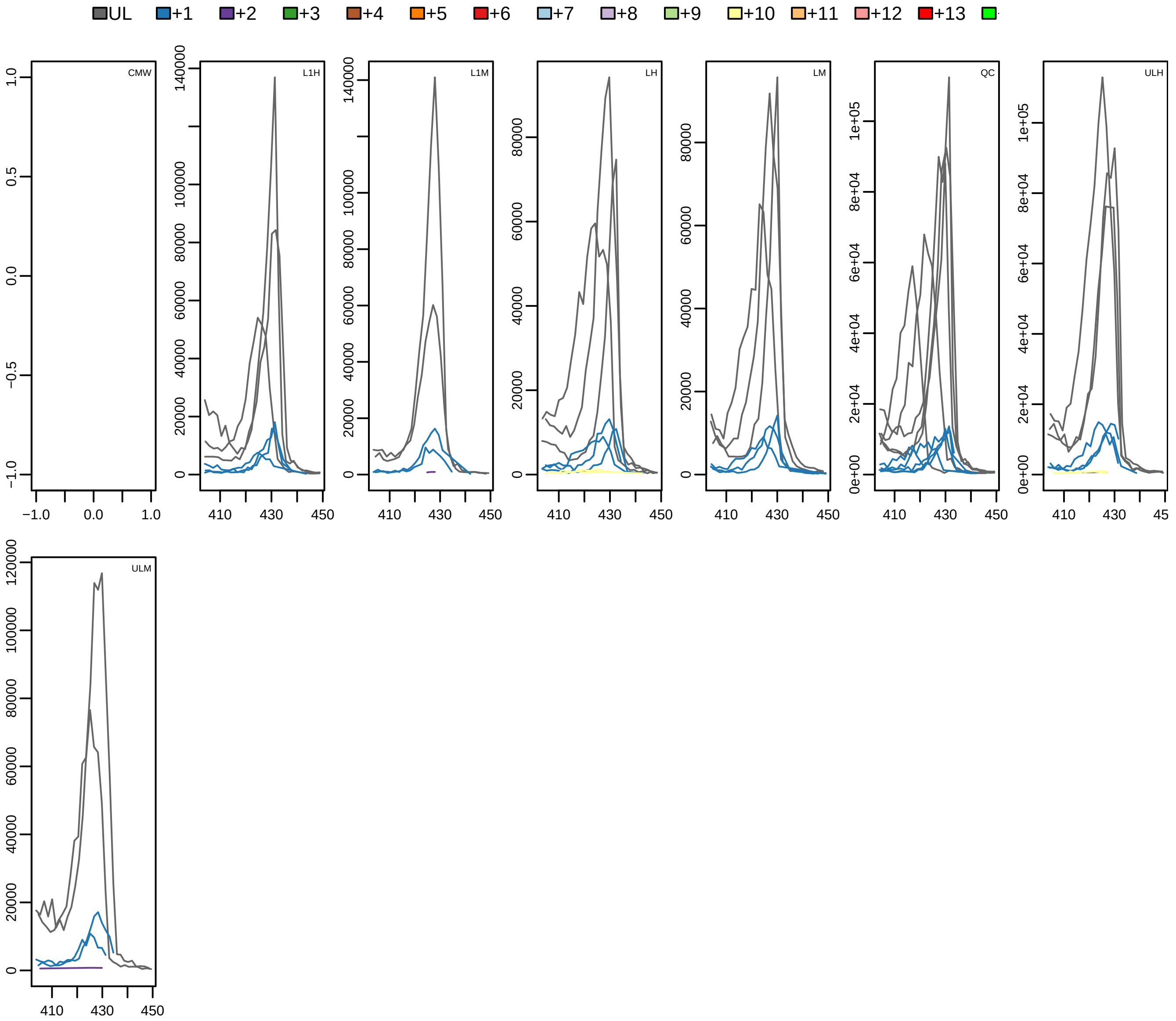

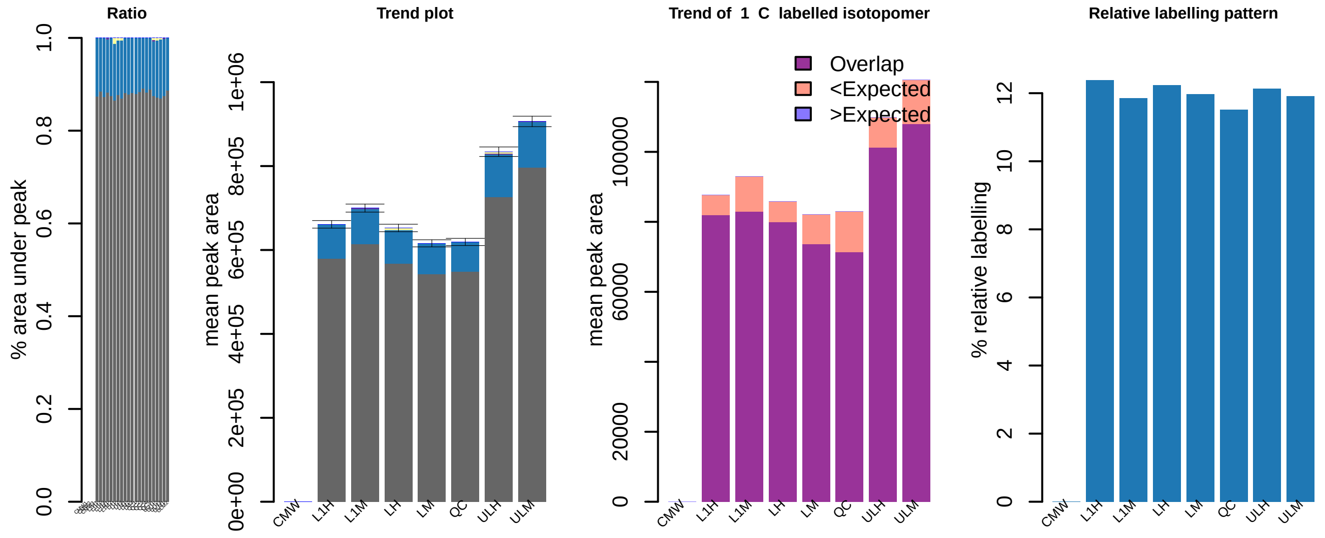

[PR (3:0)] (+)−22−acetoxy−3alpha−hydorxy−lanosta−7,

Formula: C32H48O5 Mass: 512.35 Std.RT: 203.21916708 Ion: POS

G1

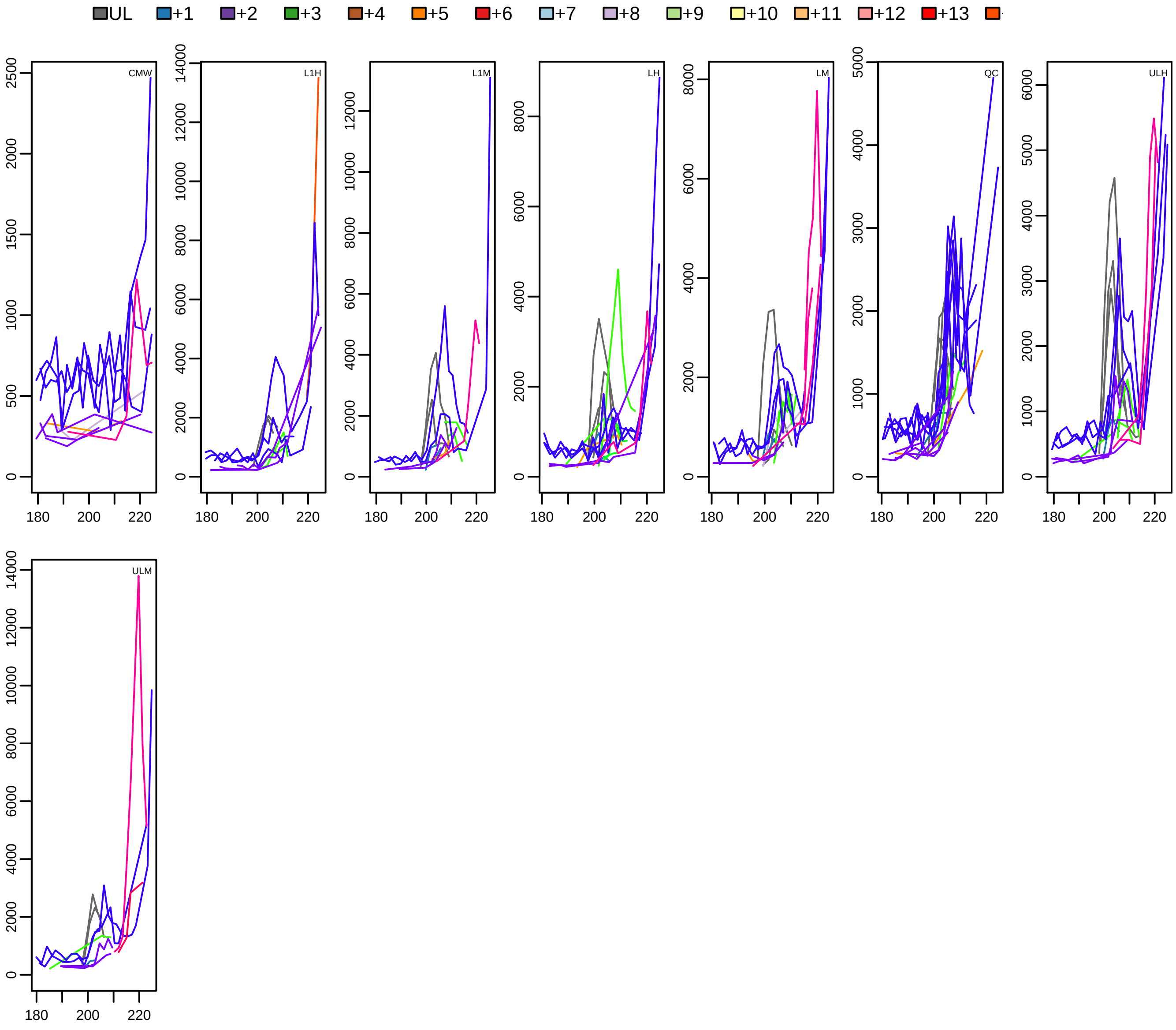

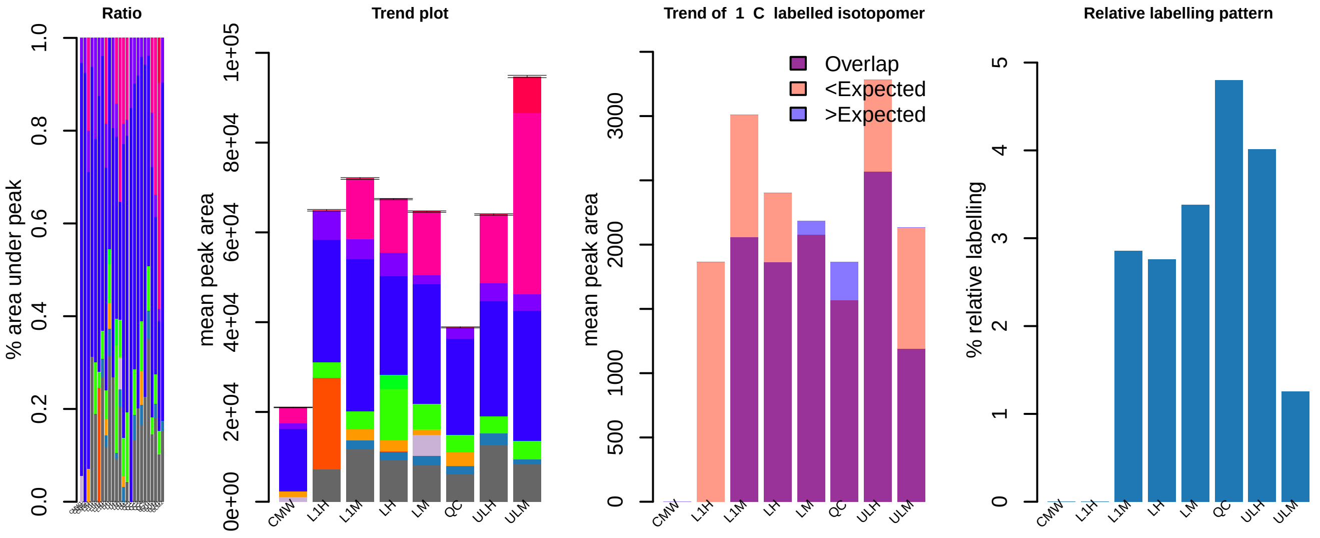

SM(d18:0/18:0)

Formula: C41H85N2O6P Mass: 732.615 Std.RT: 245.6177859 Ion: P

G1

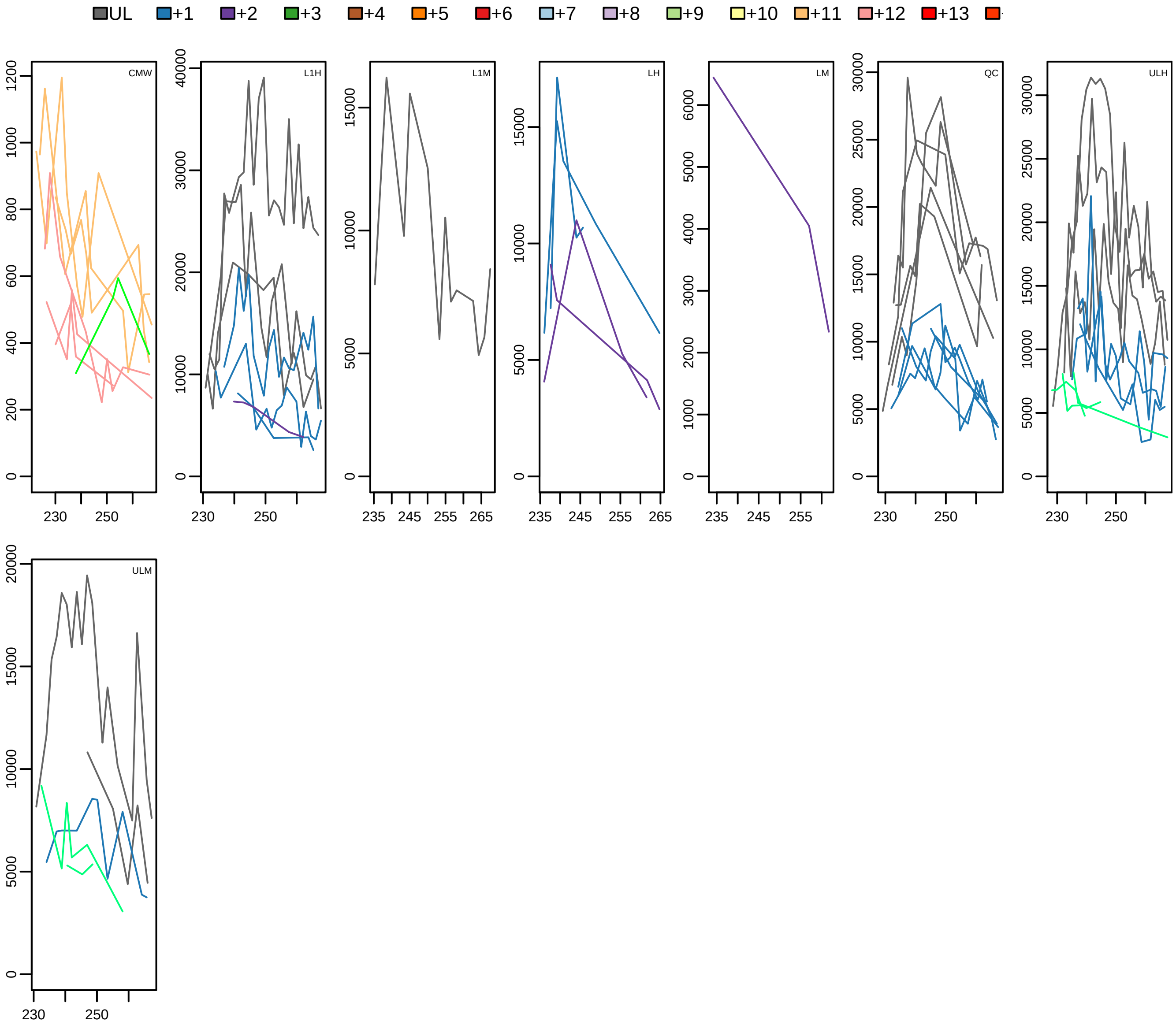

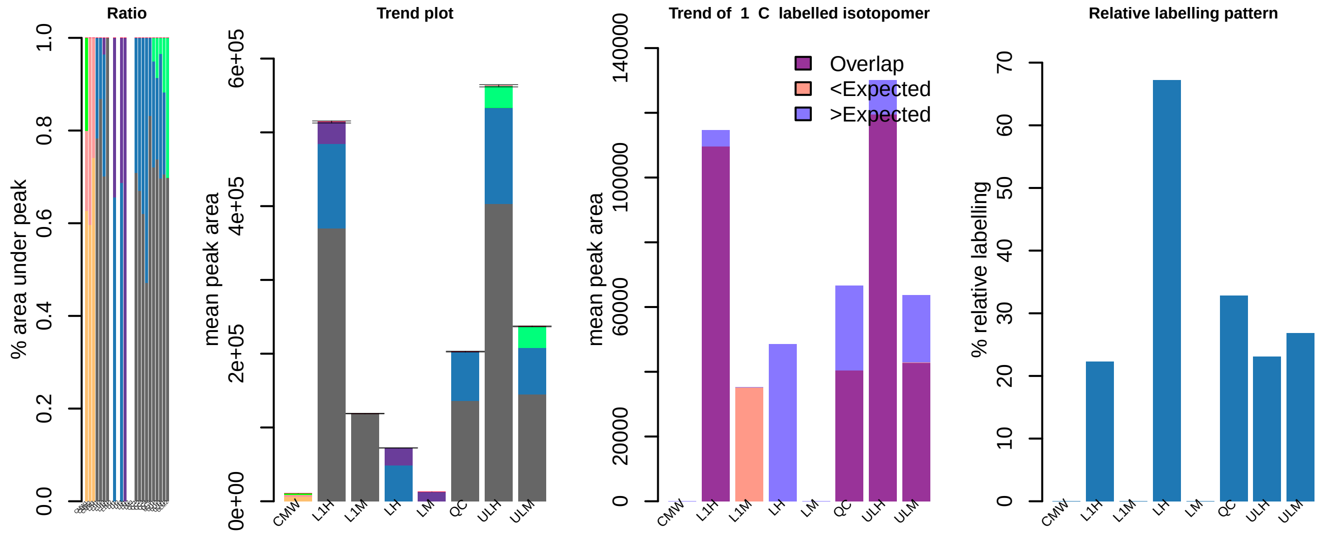

[SP (16:0)] N−(hexadecanoyl)−sphinganine−1−phosph

Formula: C39H81N2O6P Mass: 704.583 Std.RT: 240.50131548 Ion:

G1

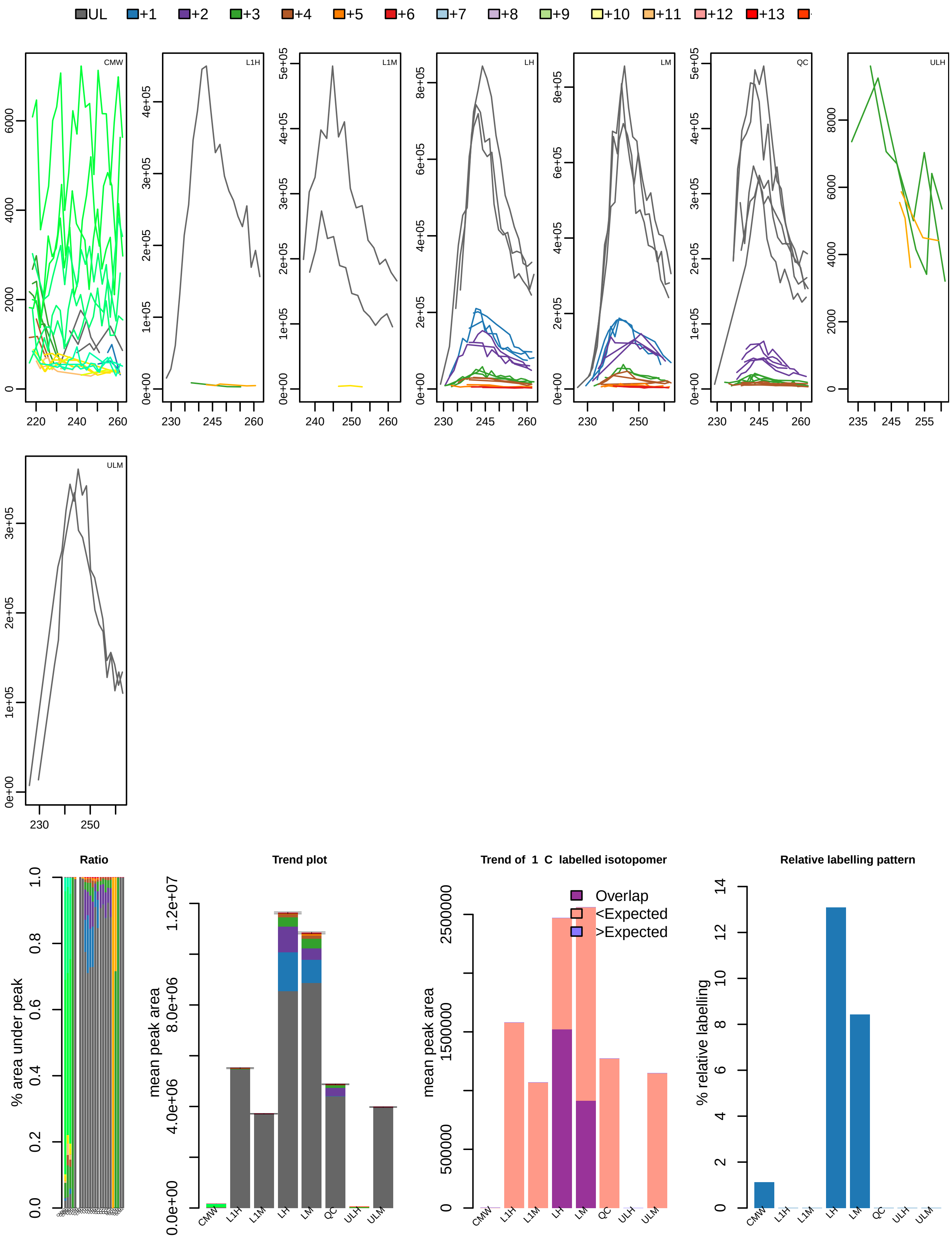

[SP (16:0)] N−(hexadecanoyl)−sphinganine−1−phosph

Formula: C39H81N2O6P Mass: 704.583 Std.RT: 240.50131548 Ion:

G2

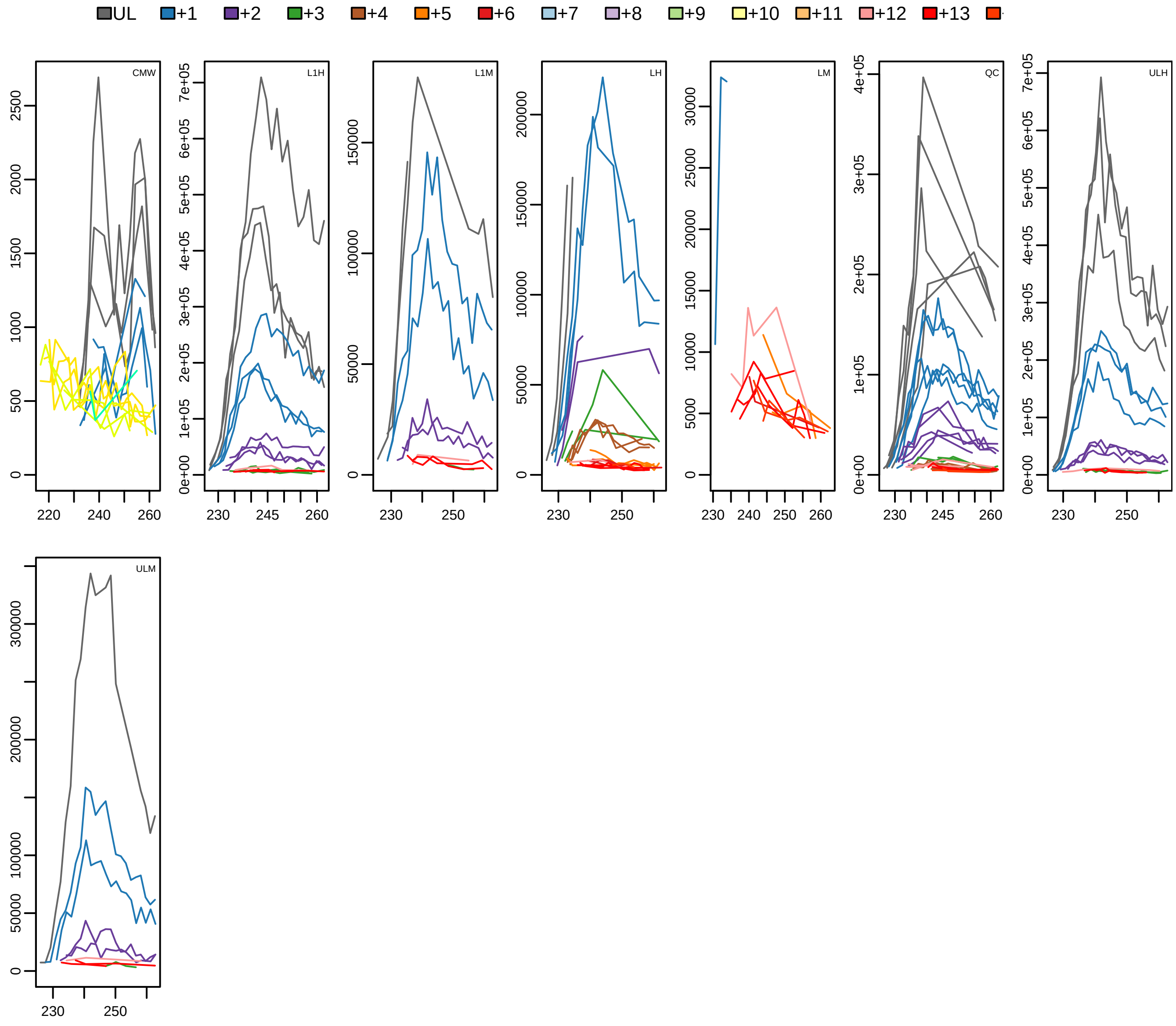

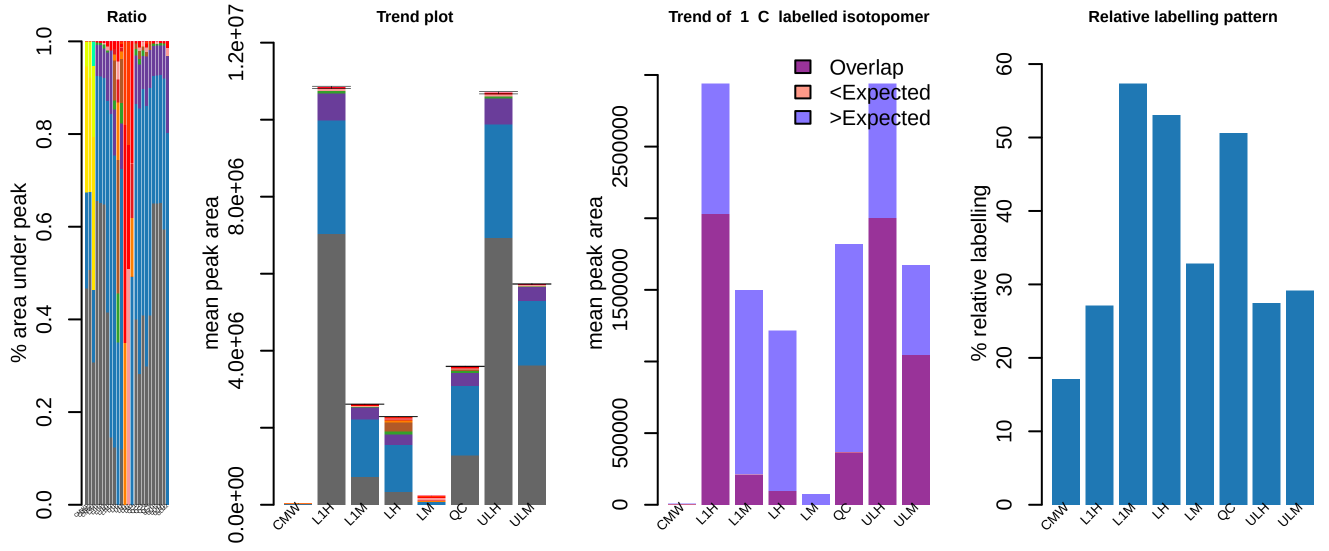

SM(d18:0/14:0)

Formula: C37H77N2O6P Mass: 676.552 Std.RT: 244.53978852 Ion:

G1

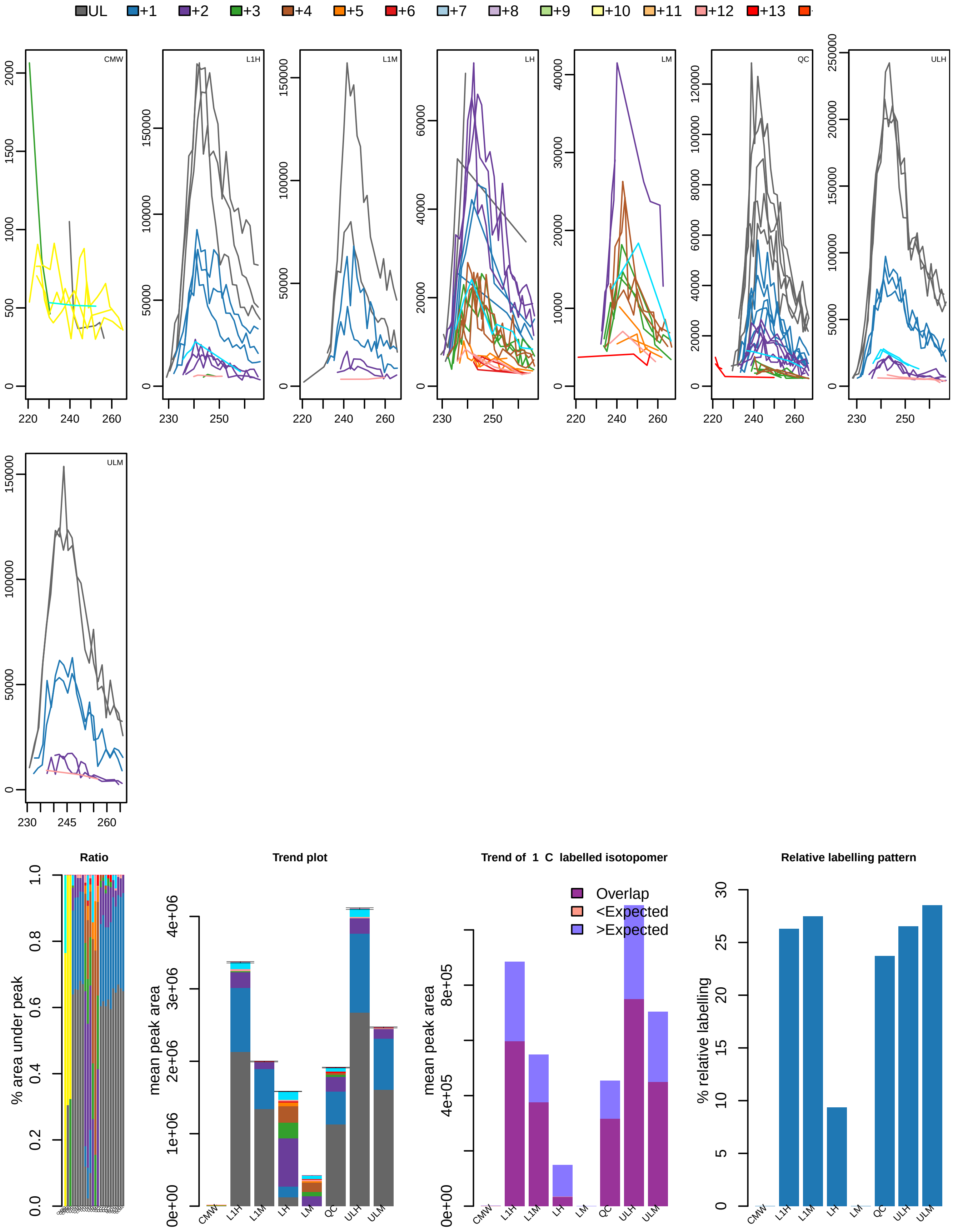

SM(d18:1/12:0)

Formula: C35H71N2O6P Mass: 646.505 Std.RT: 245.52814158 Ion:

G1

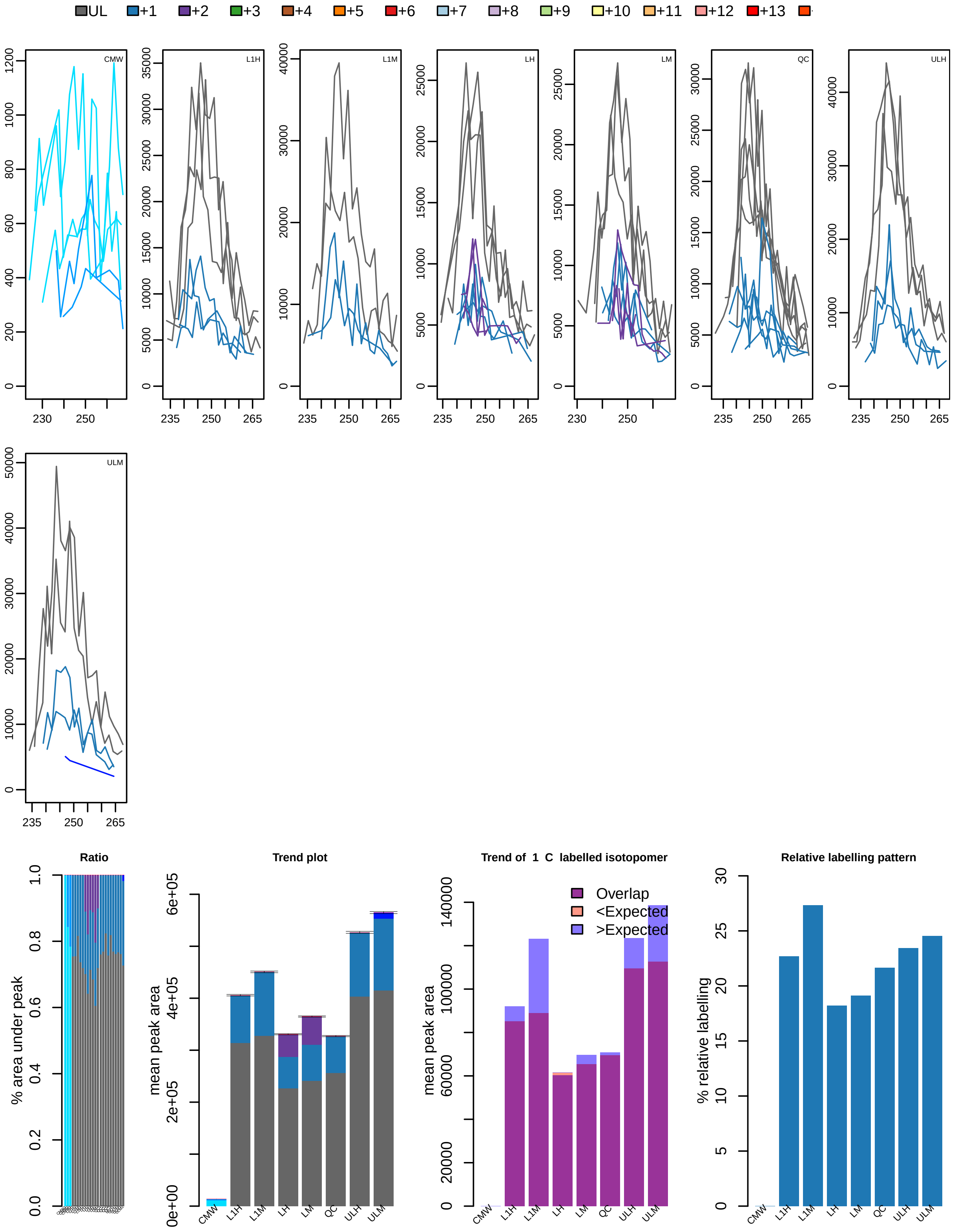

SM(d18:1/14:0)

Formula: C37H75N2O6P Mass: 674.536 Std.RT: 243.9342612 Ion: P

G1

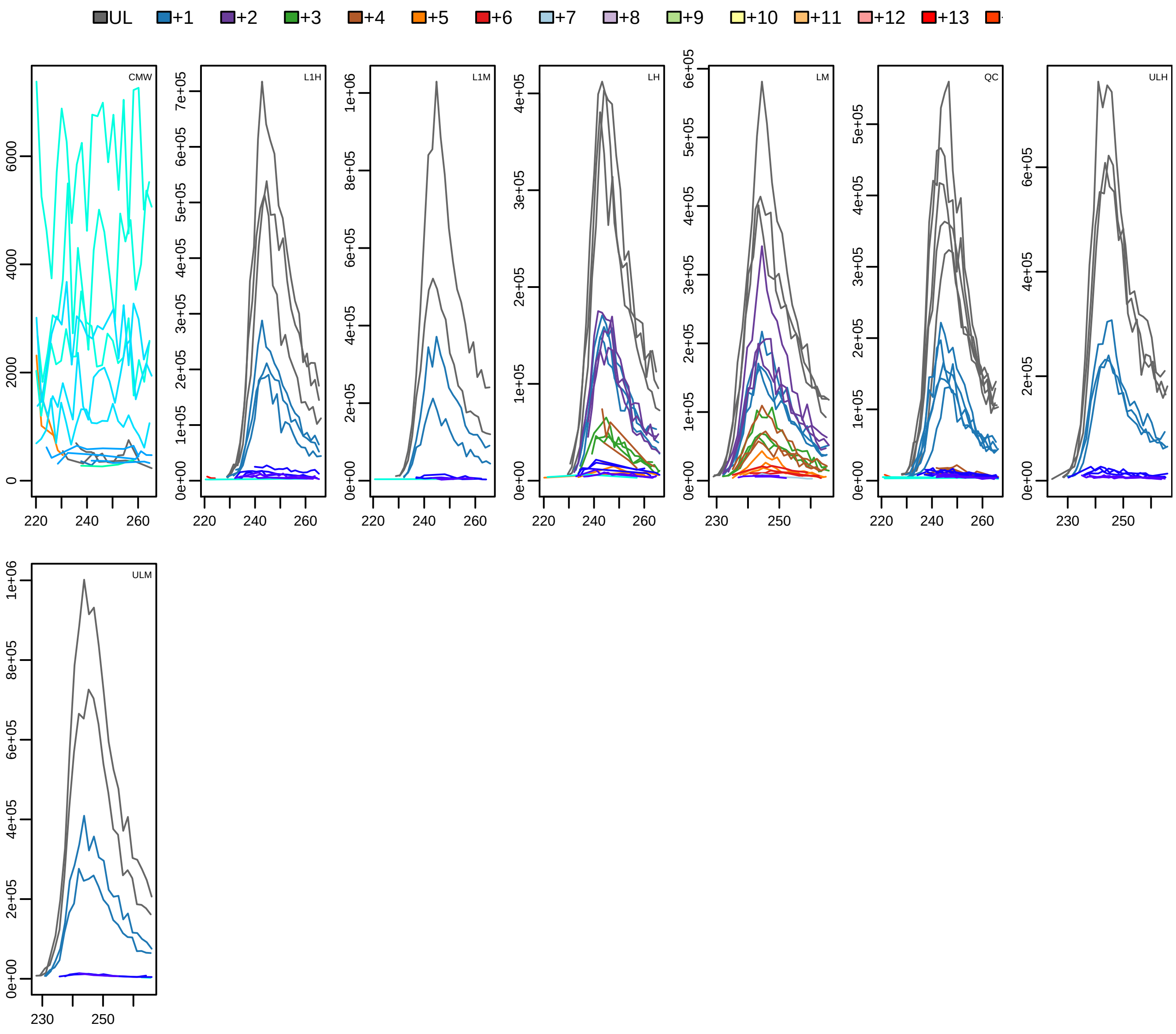

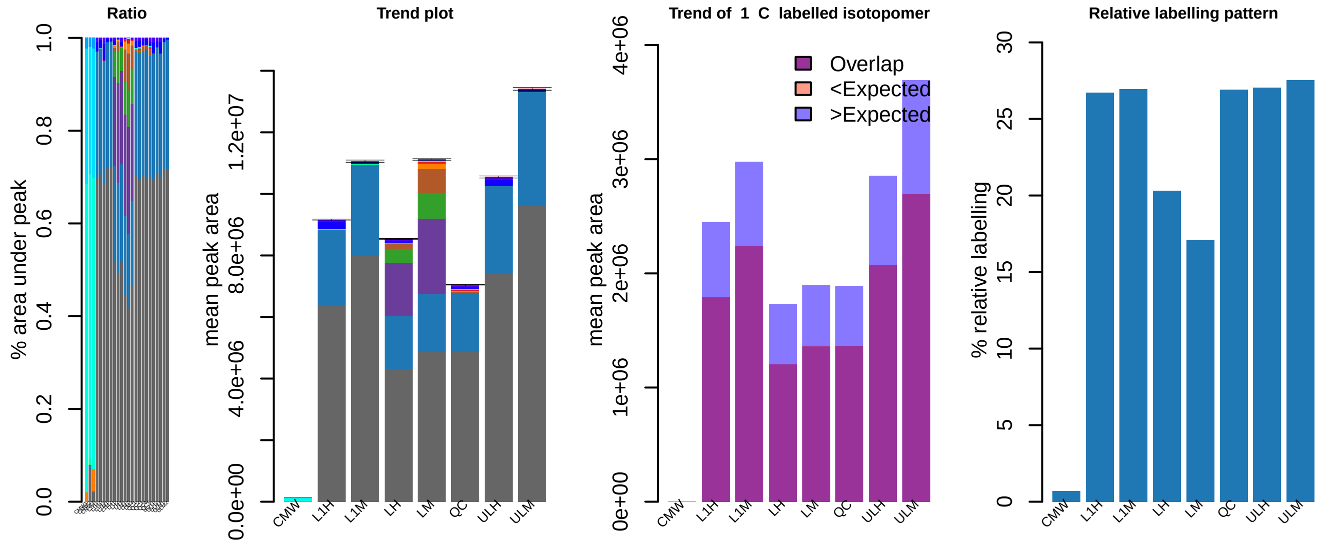

SM(d18:0/18:1(9Z))

Formula: C41H83N2O6P Mass: 730.599 Std.RT: 244.1156667 Ion: P

G1

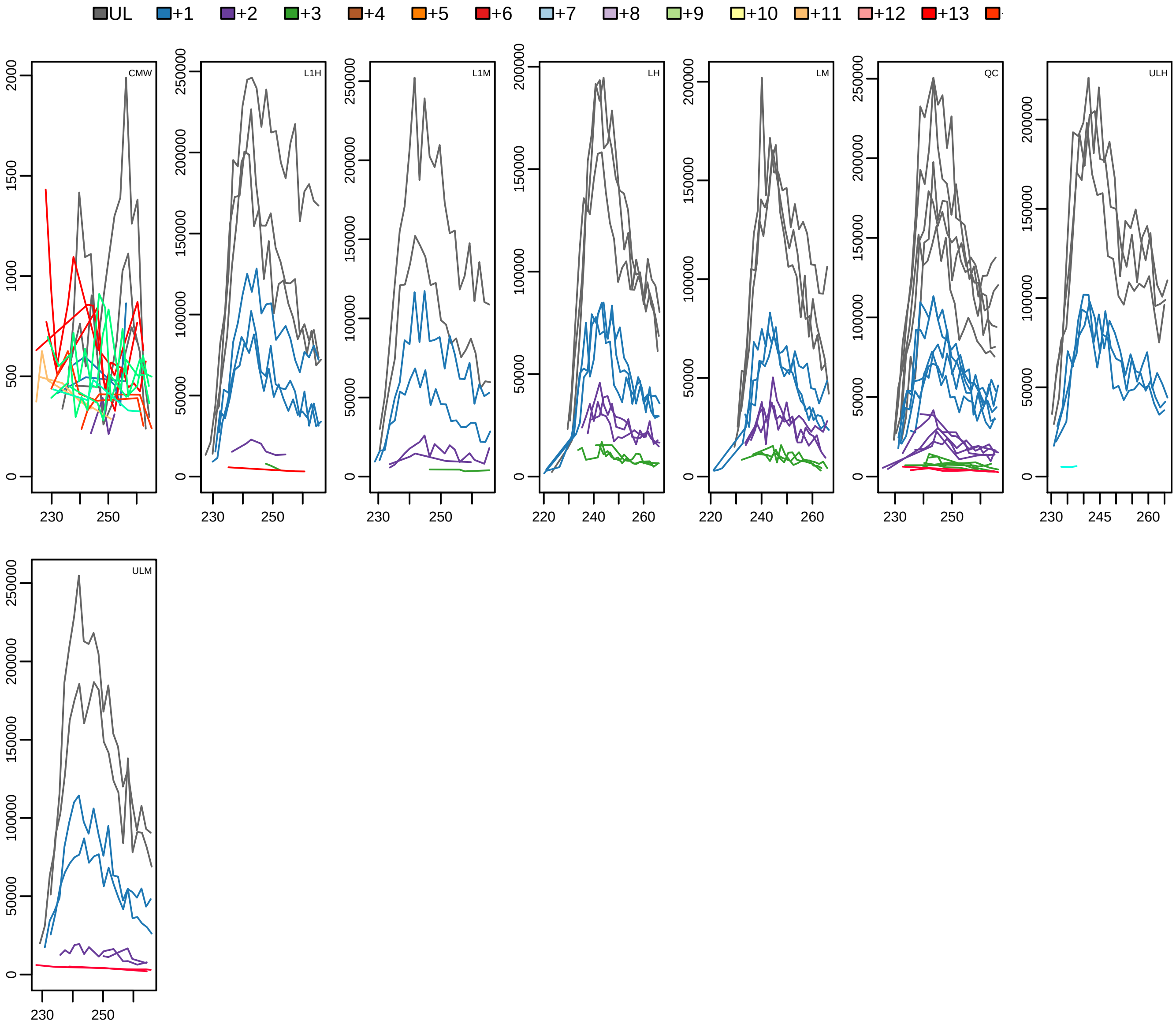

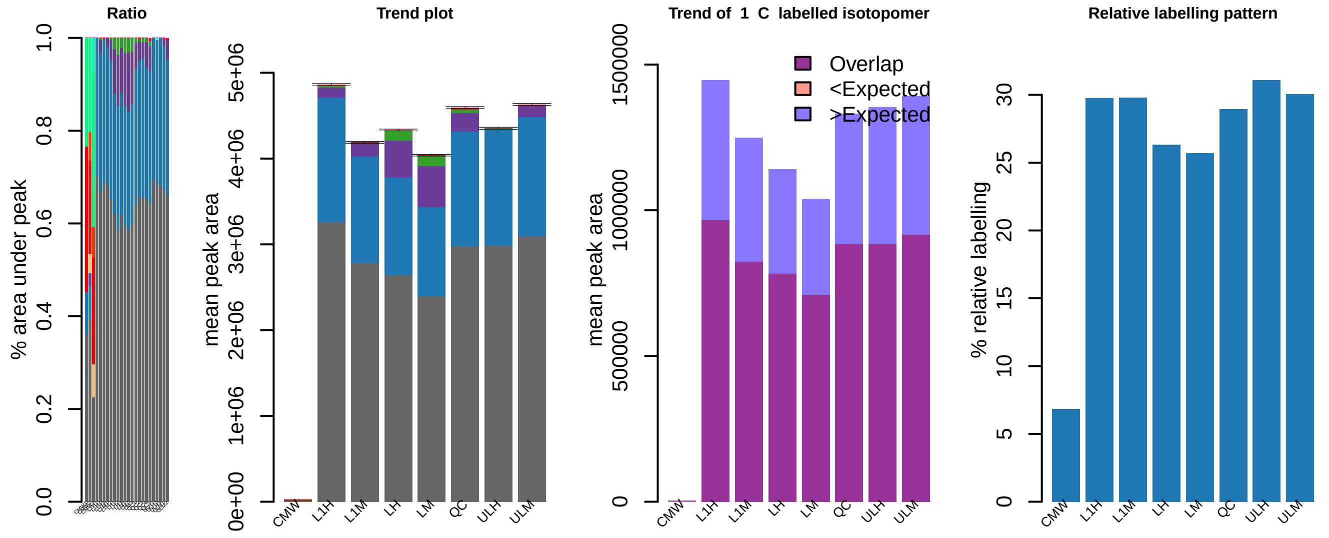

[SP (16:0)] N−(hexadecanoyl)−sphing−4−enine−1−pho[illegible]

G1

Formula: C39H79N2O6P Mass: 702.568 Std.RT: 244.60829292 Ion: [illegible]

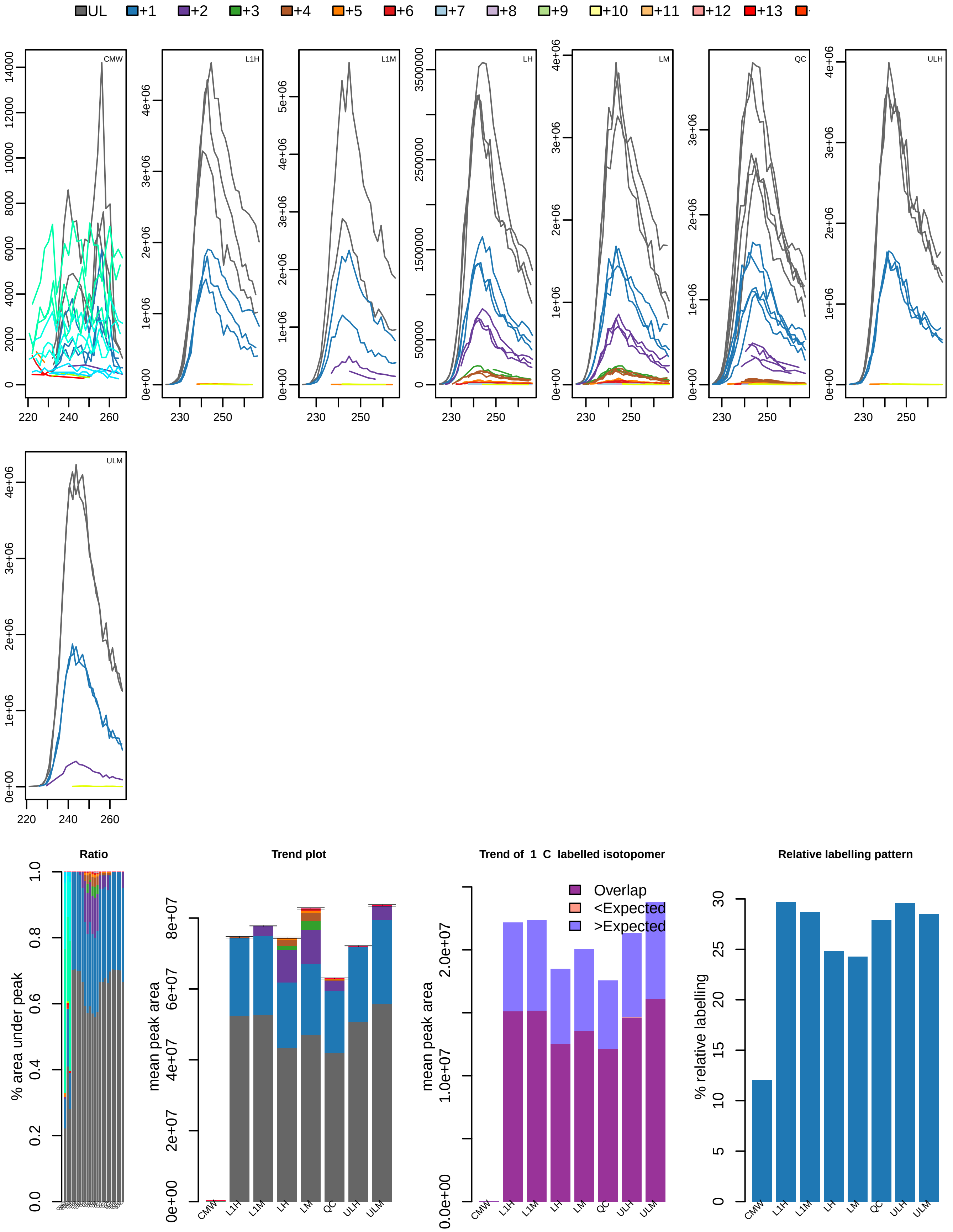

SM(d18:1/18:1(9Z))

Formula: C41H81N2O6P Mass: 728.583 Std.RT: 242.69133288 Ion:

G1

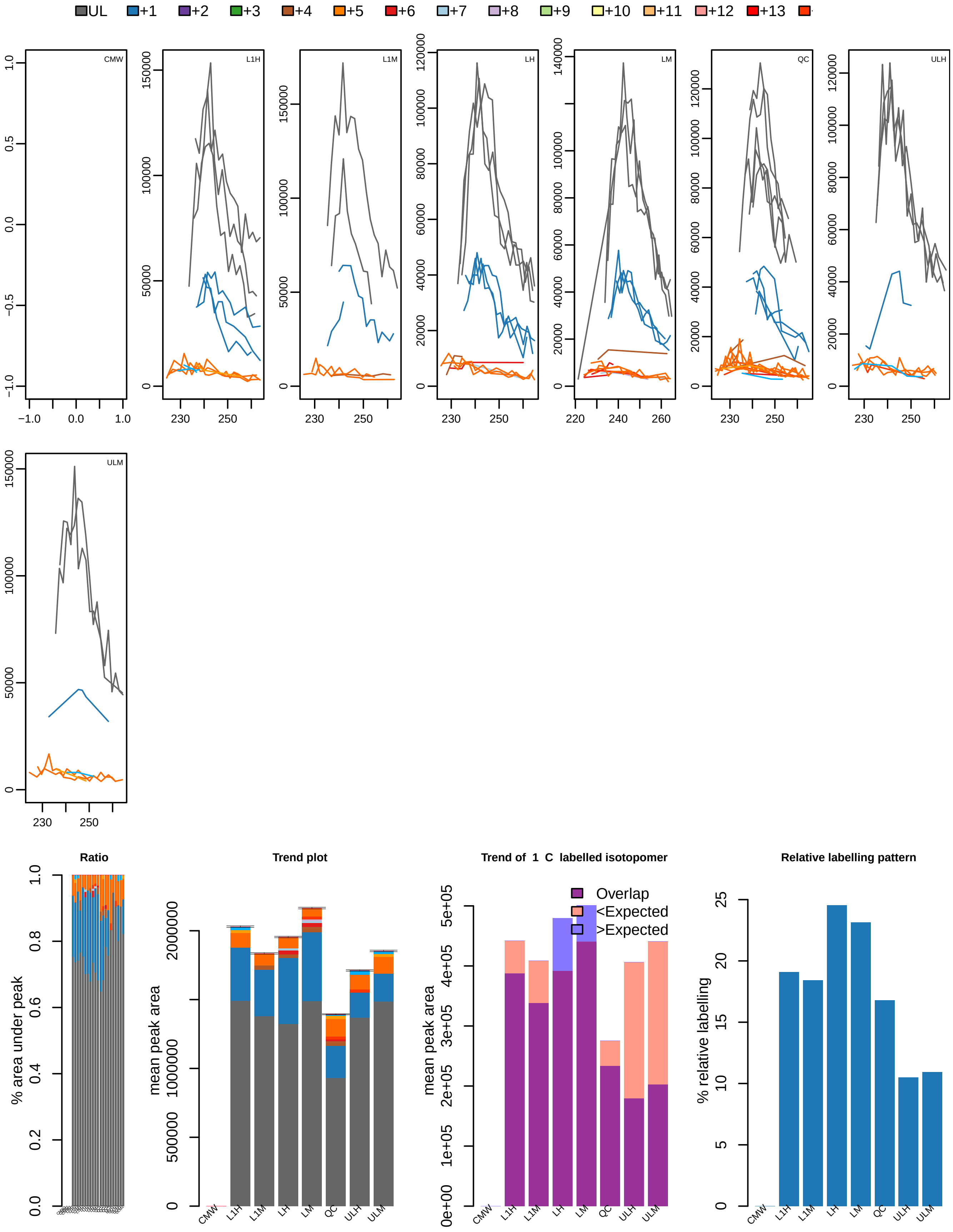

[SP (18:0/14:0)] N−(octadecanoyl)−tetradecasphing−4-

Formula: C38H77N2O6P Mass: 688.552 Std.RT: 244.13512608 Ion:

G1

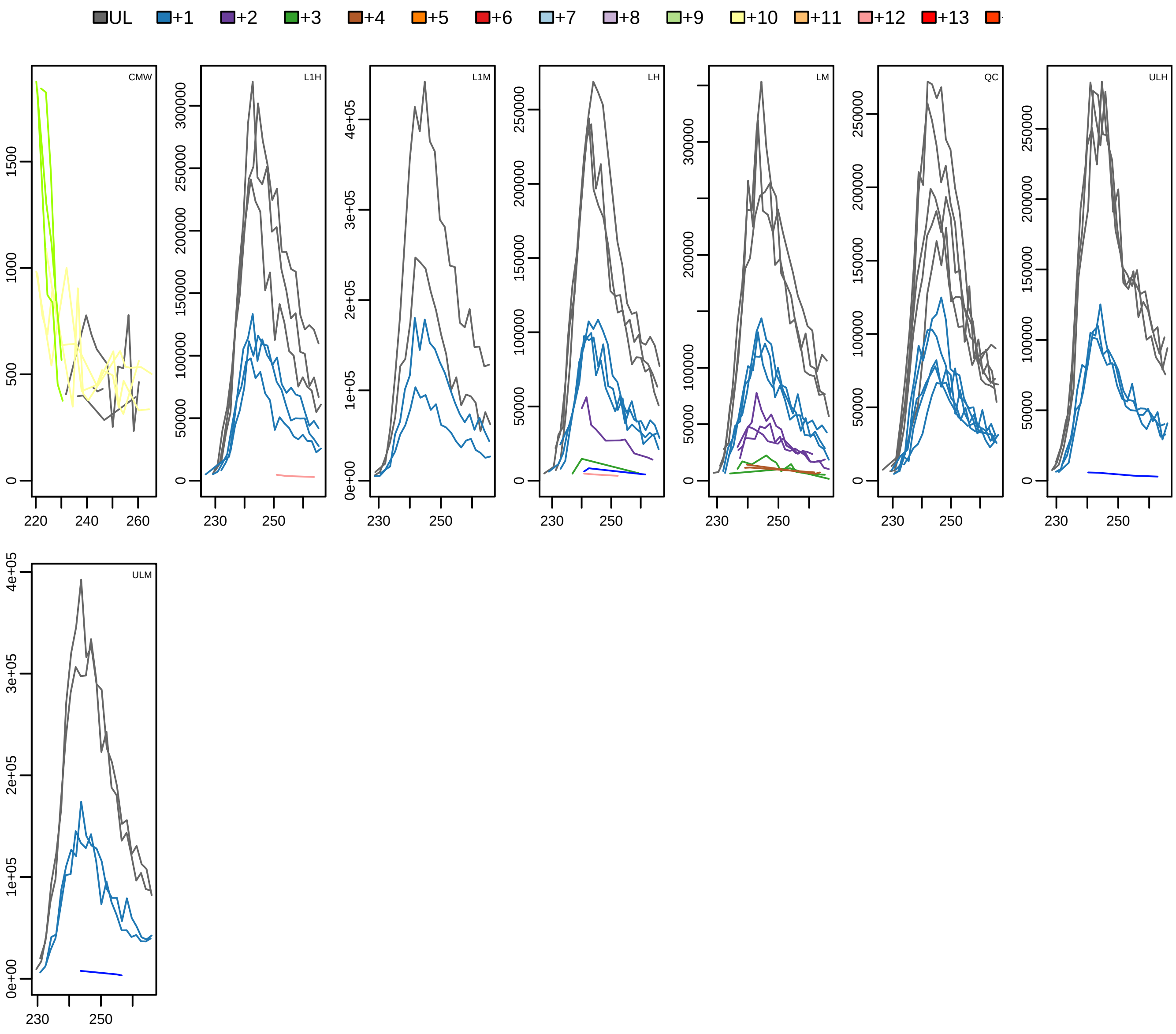

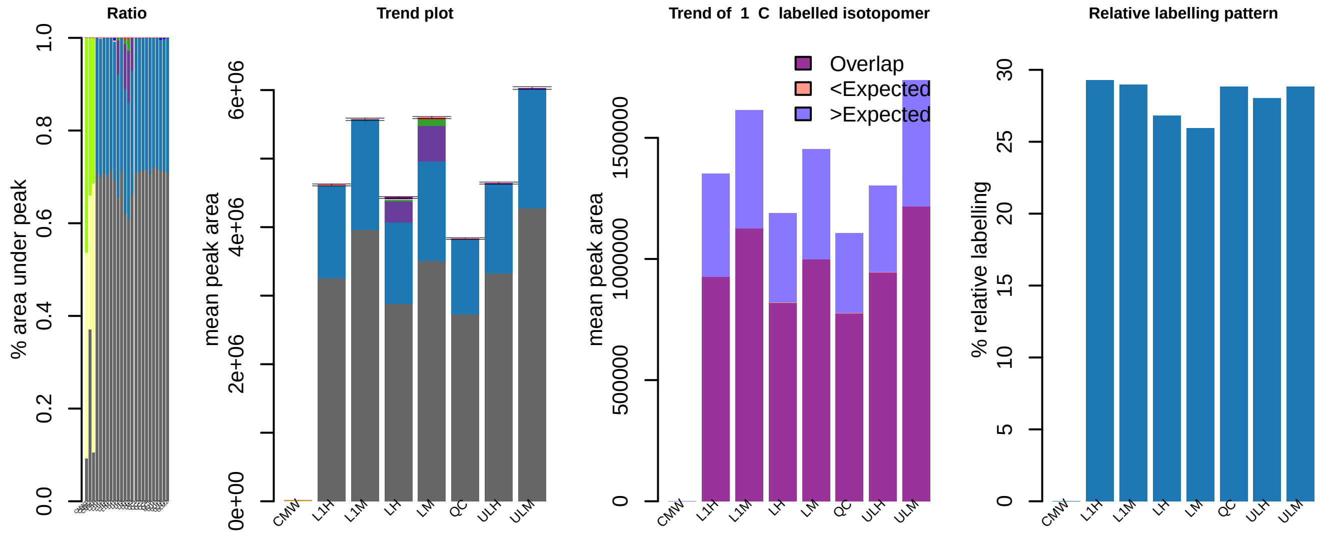

N−(Tetradecanoyl)−sphing−4−enine

Formula: C32H63NO3 Mass: 509.481 Std.RT: 222.61866822 Ion: PC

G1

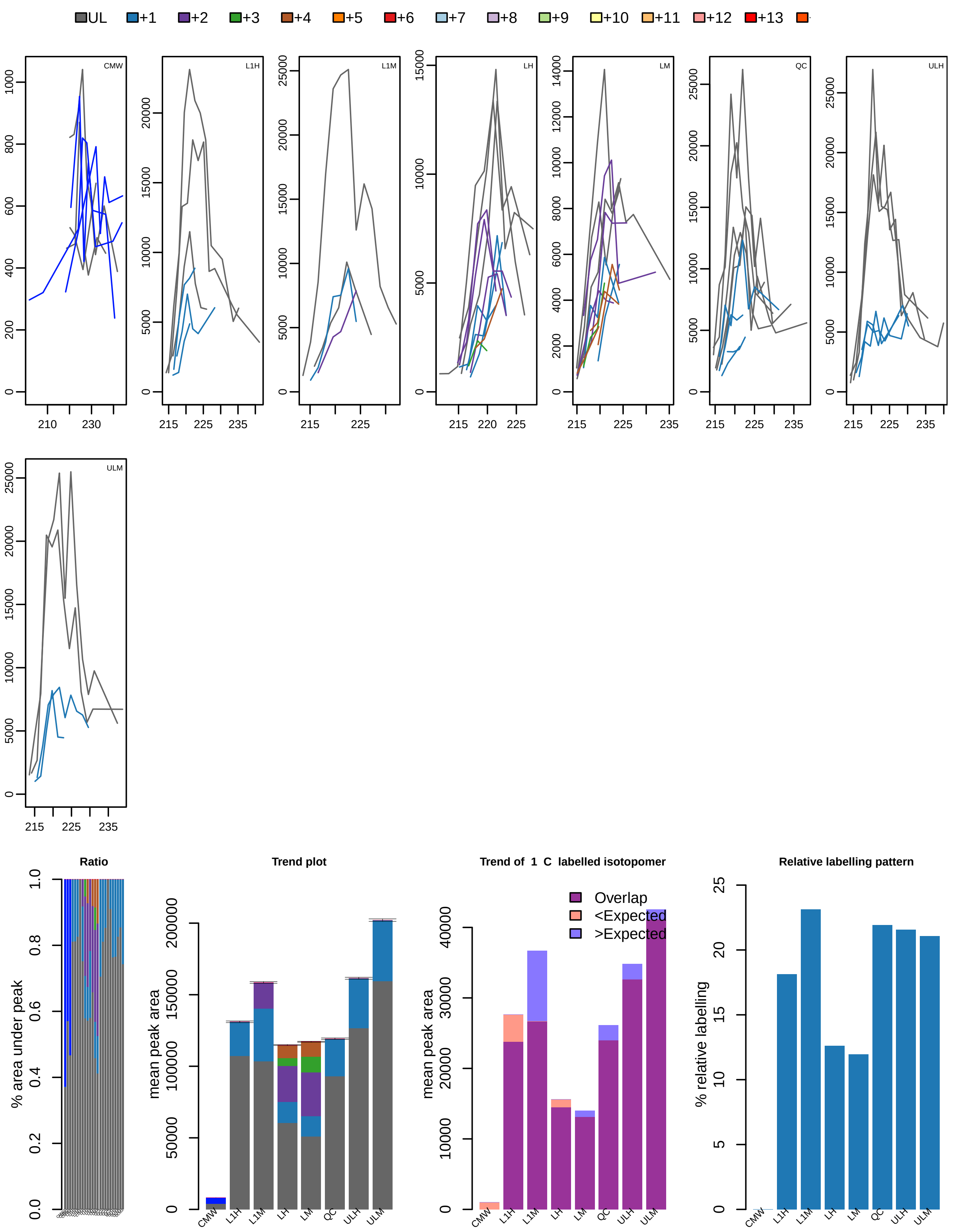

[SP (18:0)] N−(octadecanoyl)−sphing−4−enine

Formula: C36H71NO3 Mass: 565.543 Std.RT: 221.559858 Ion: POS

G1

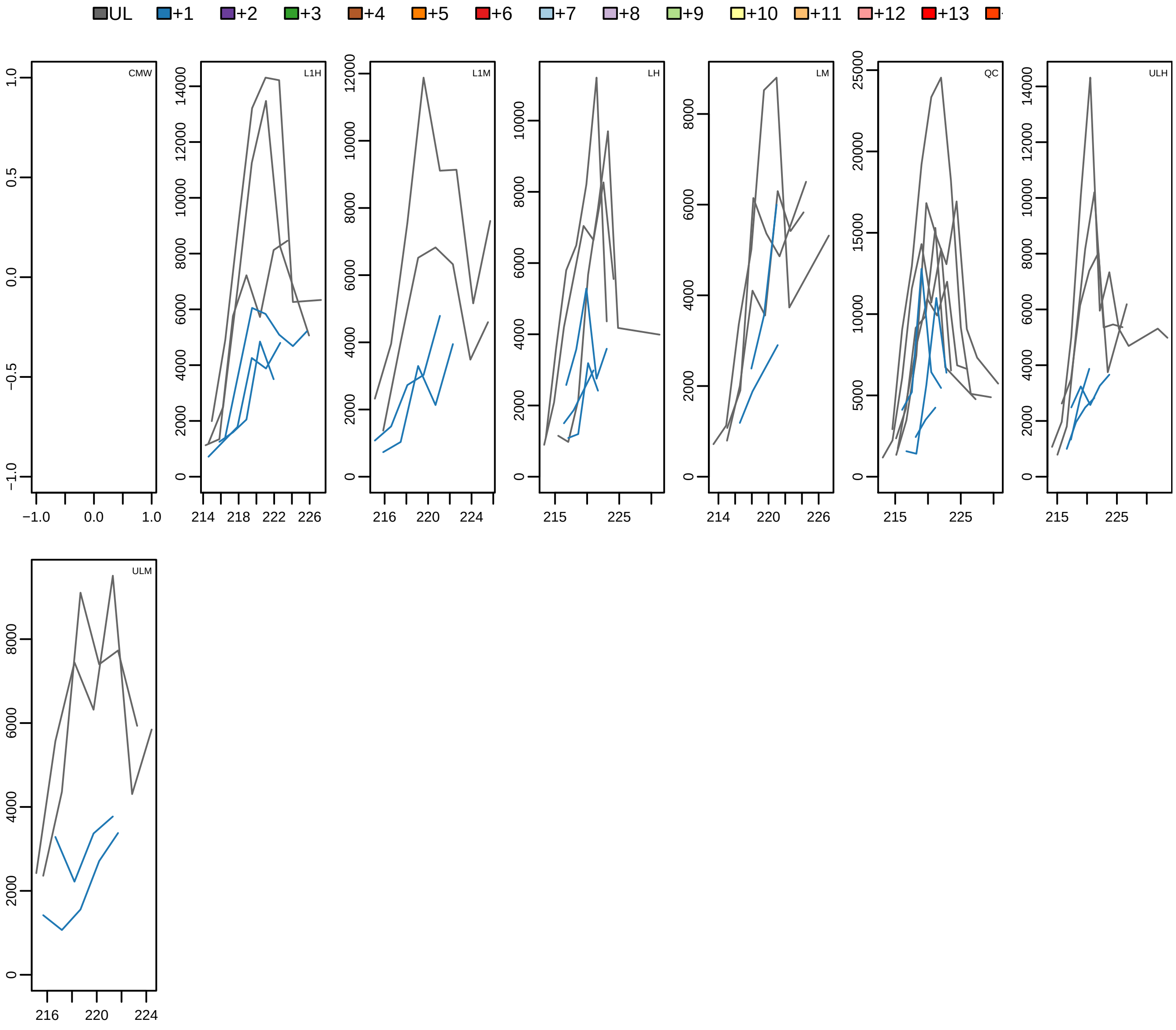

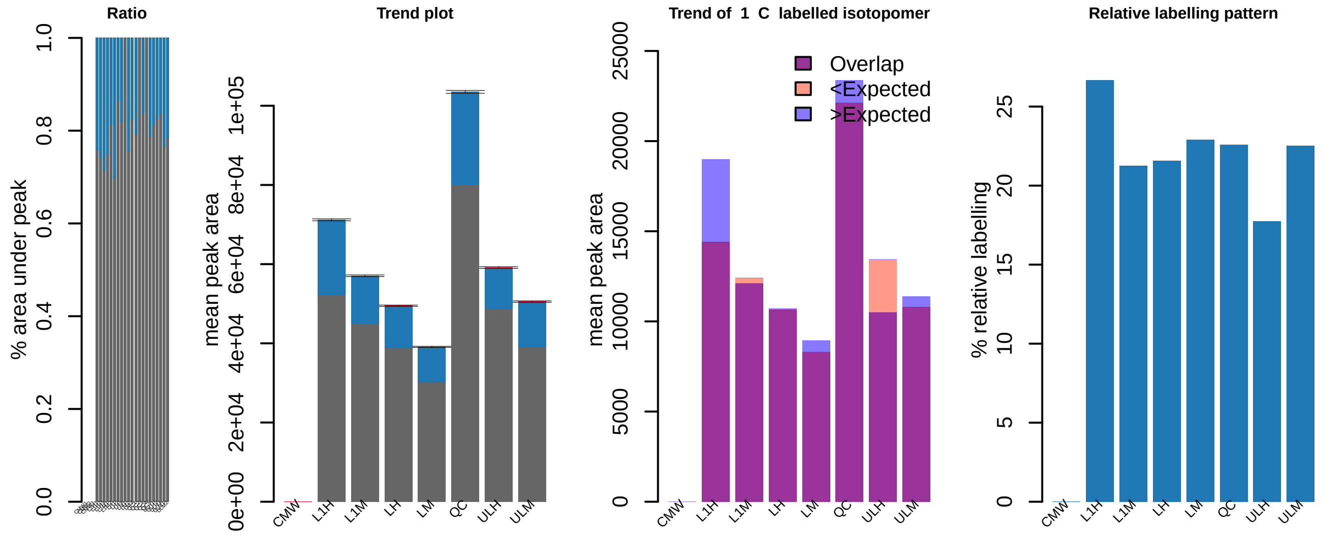

[SP (16:0)] N−(hexadecanoyl)−sphing−4−enine

Formula: C34H67NO3 Mass: 537.512 Std.RT: 221.62275 Ion: POS

G1

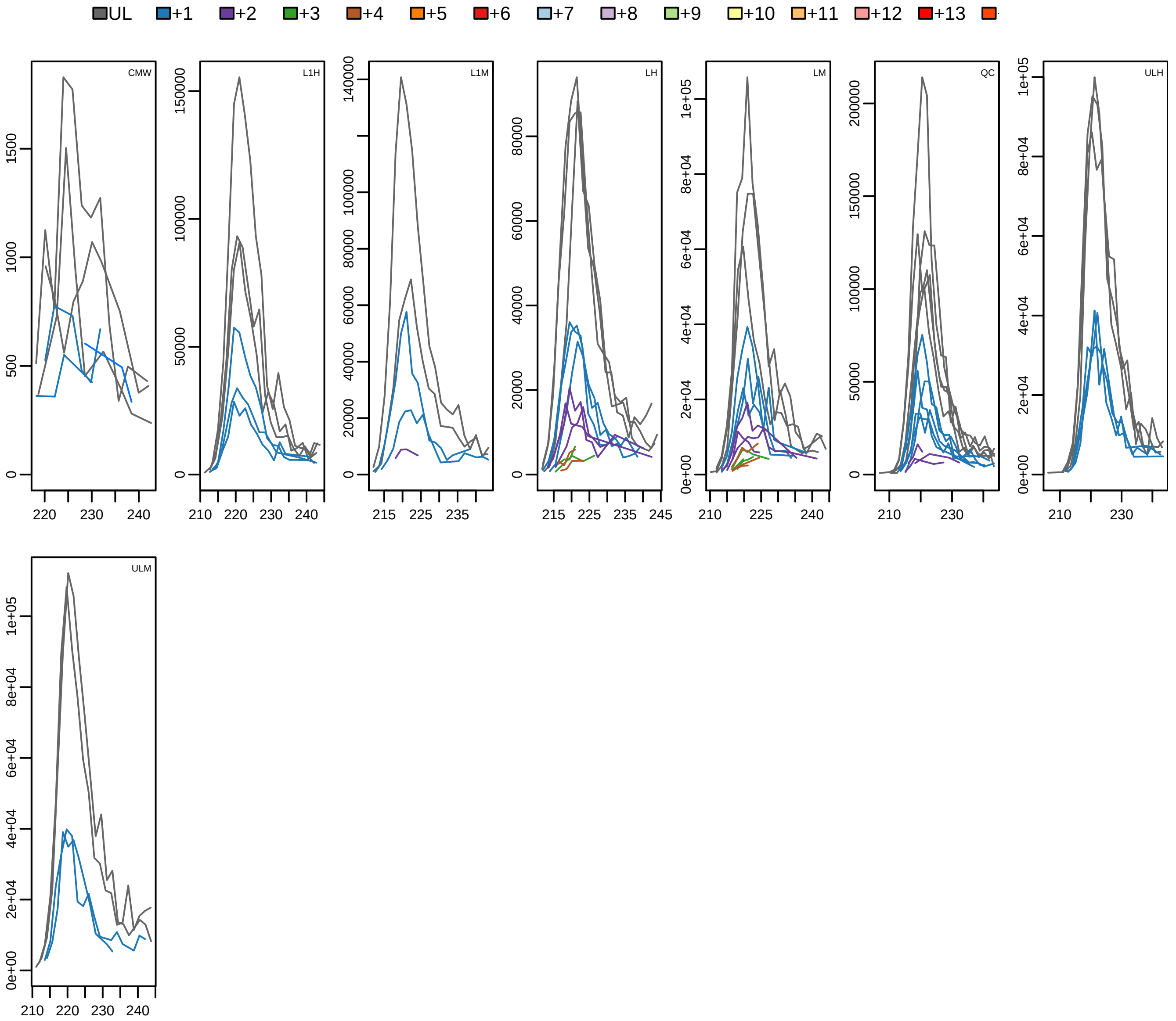

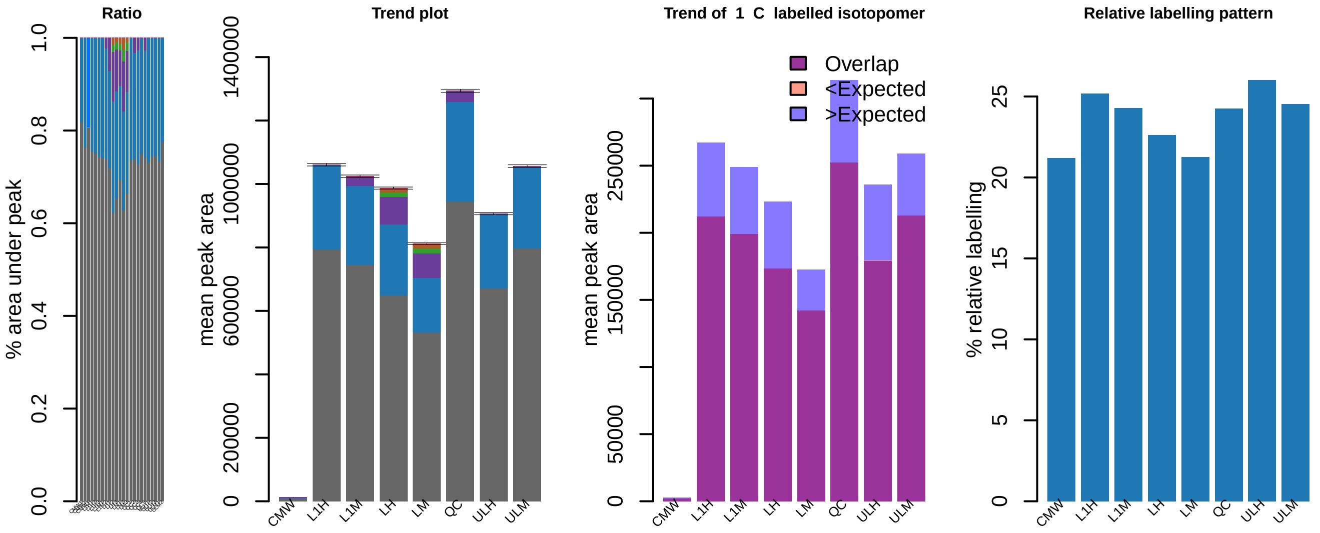

[SP (18:0)] N−(9Z−octadecenoyl)−sphing−4−enine

Formula: C36H69NO3 Mass: 563.528 Std.RT: 220.75285848 Ion: PC

G1

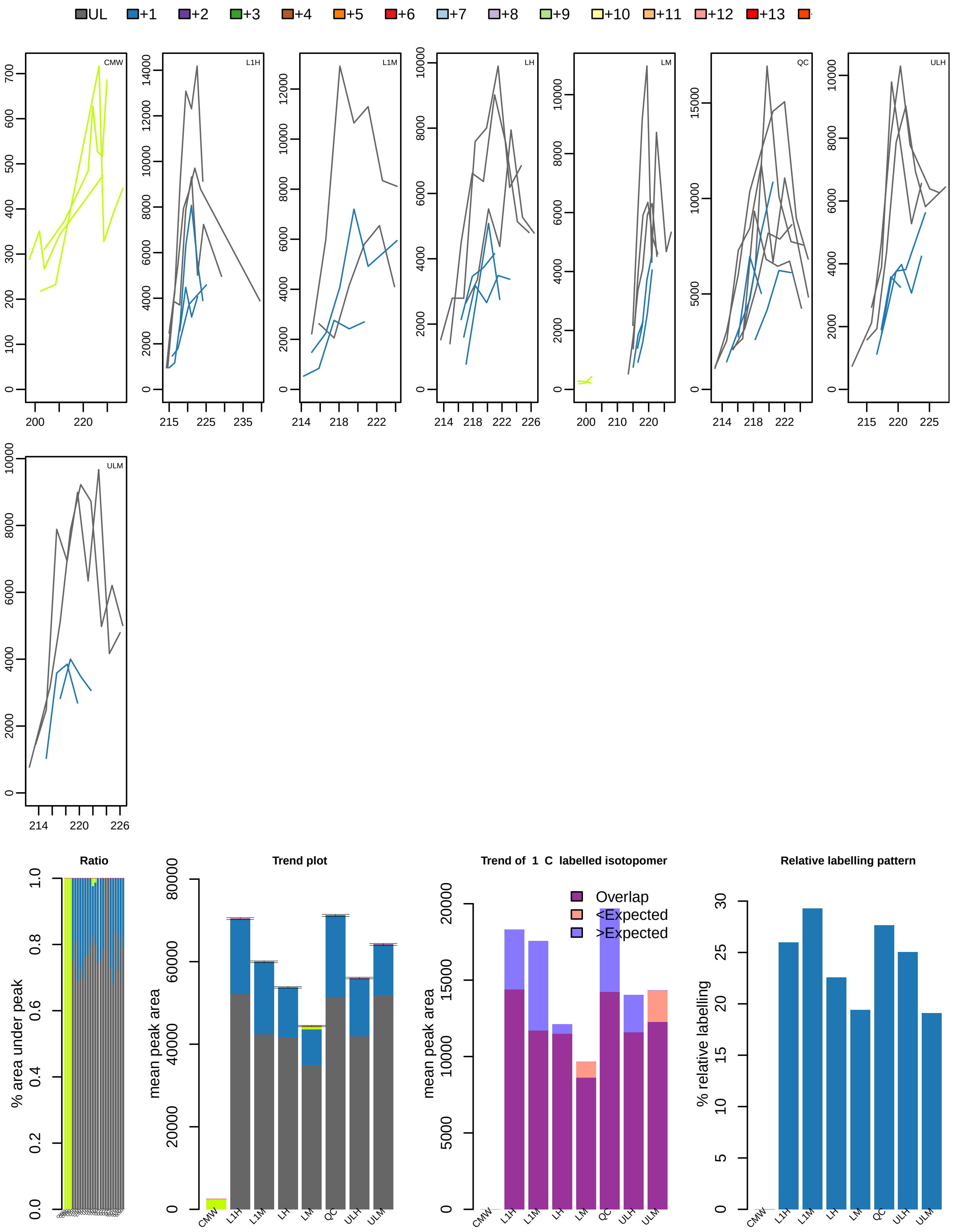

Hexadecasphinganine

G1

Formula: C16H35NO2 Mass: 273.267 Std.RT: 434.75708388 Ion: PC

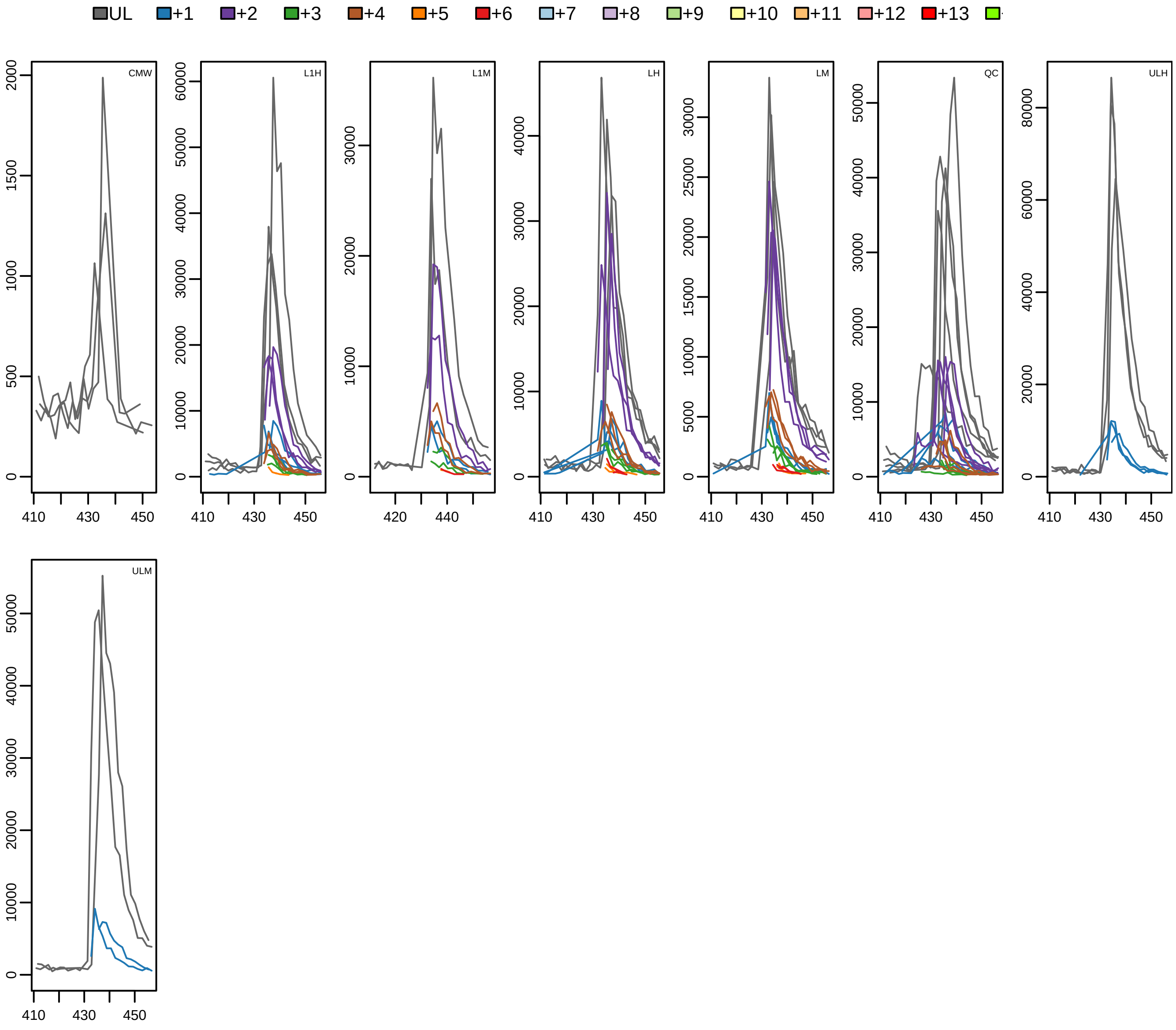

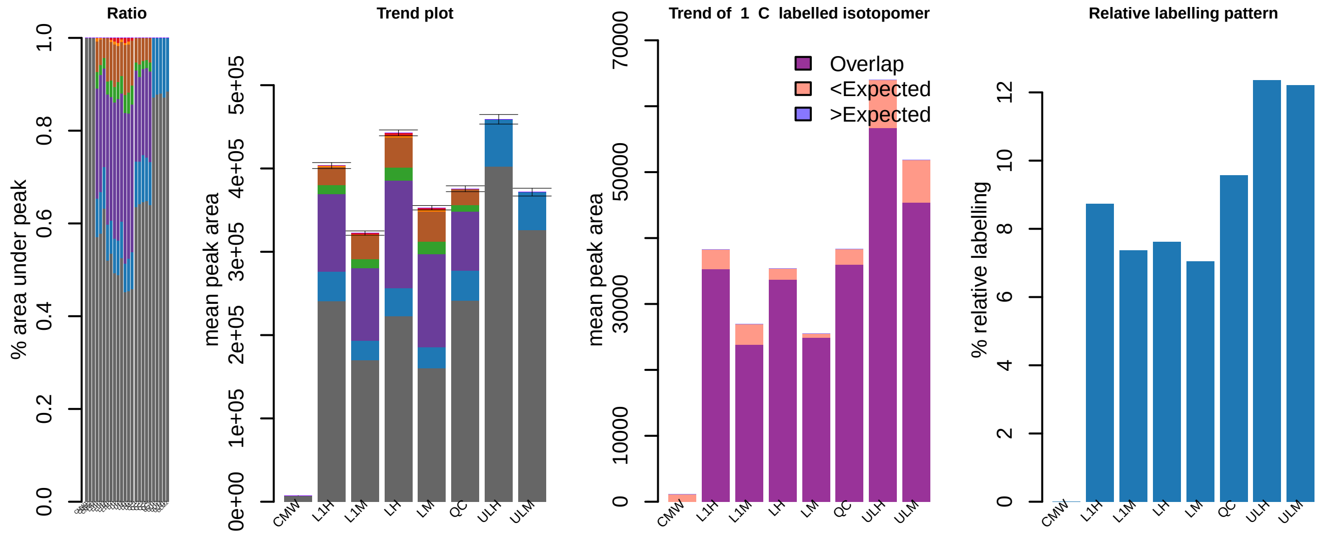

[SP (14:0)] 1−deoxy−tetradecasphinganine

G1

Formula: C14H31NO Mass: 229.241 Std.RT: 267.01229352 Ion: POS

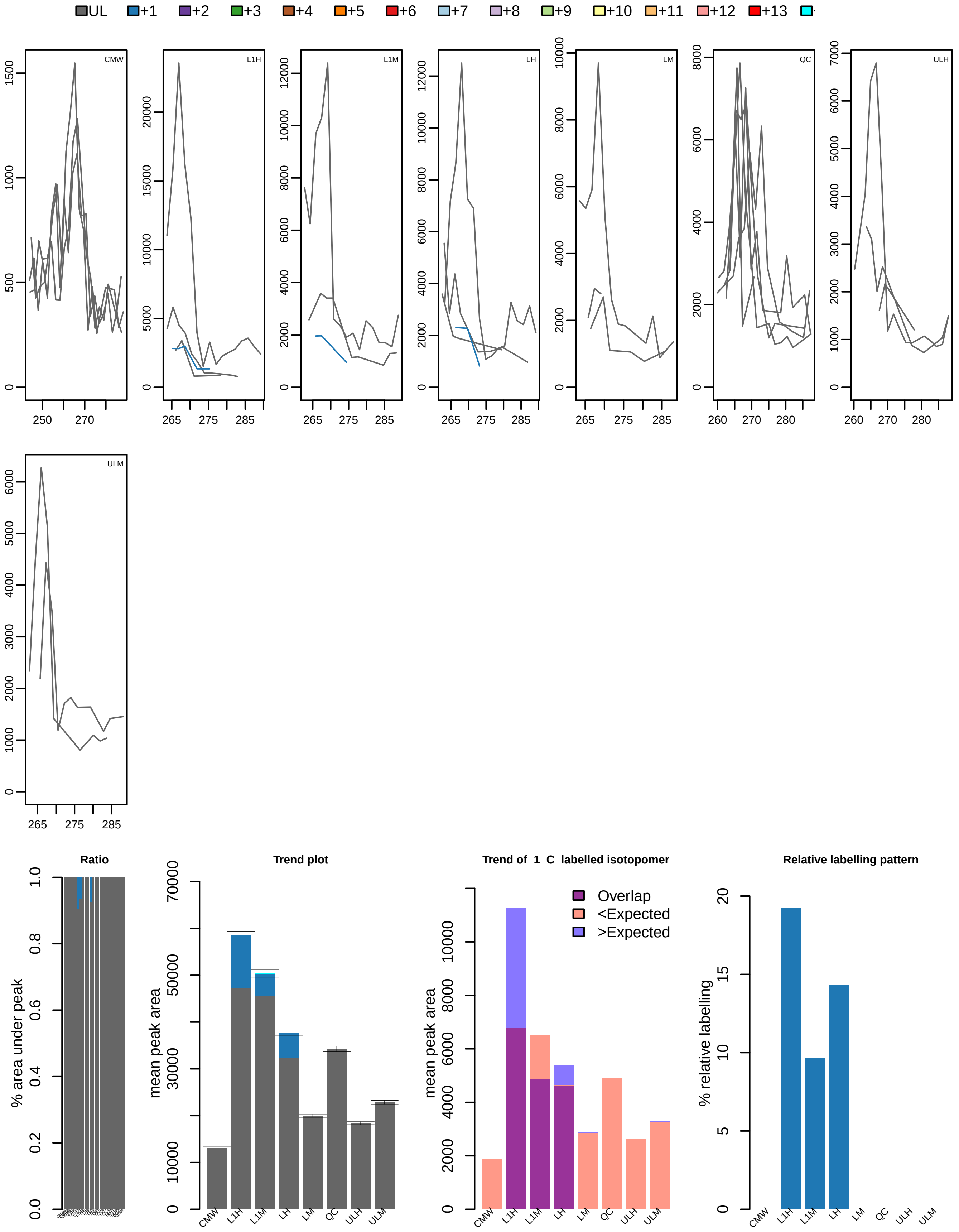

Sphinganine

Formula: C18H39NO2 Mass: 301.298 Std.RT: 433.0766352 Ion: POS

G1

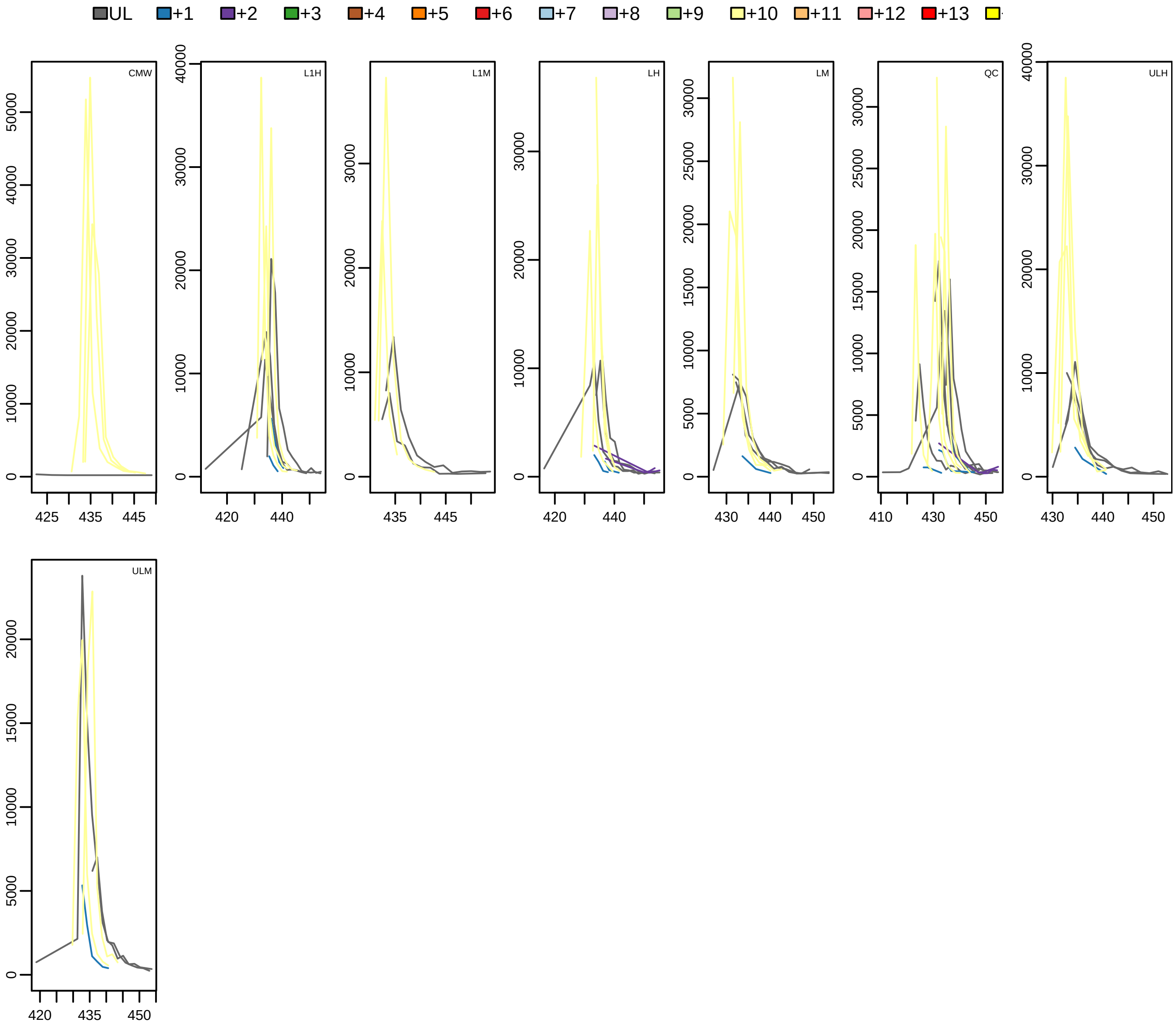

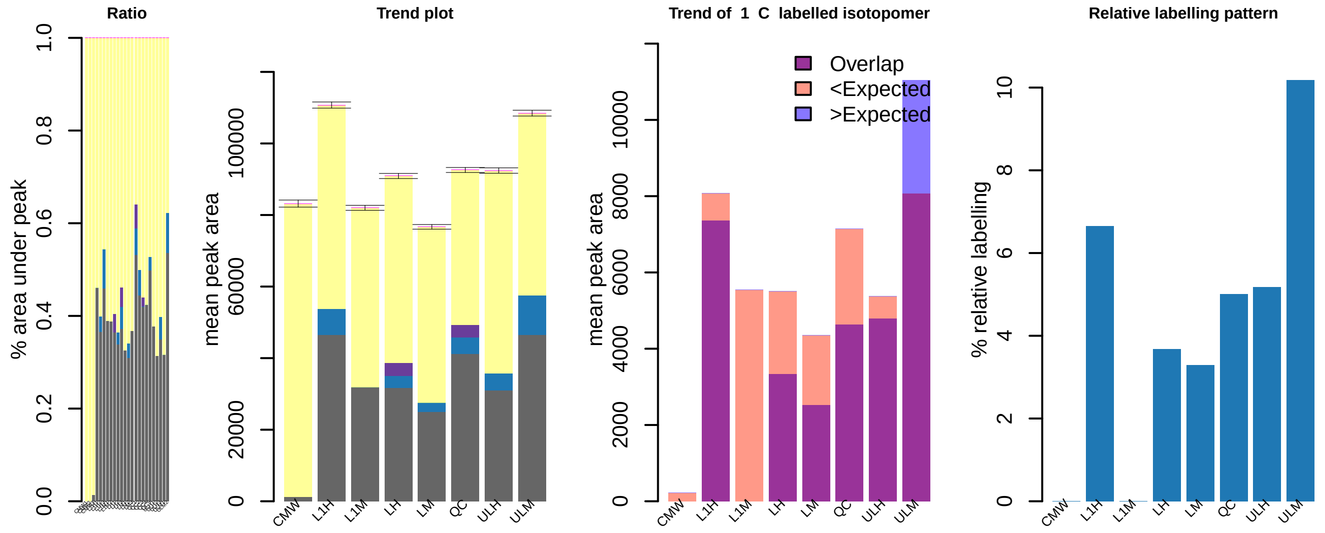

[ST] (5Z,7E)−9,10−seco−5,7,10(19)−cholestatriene

G1

Formula: C27H44 Mass: 368.344 Std.RT: 296.3246349 Ion: POS

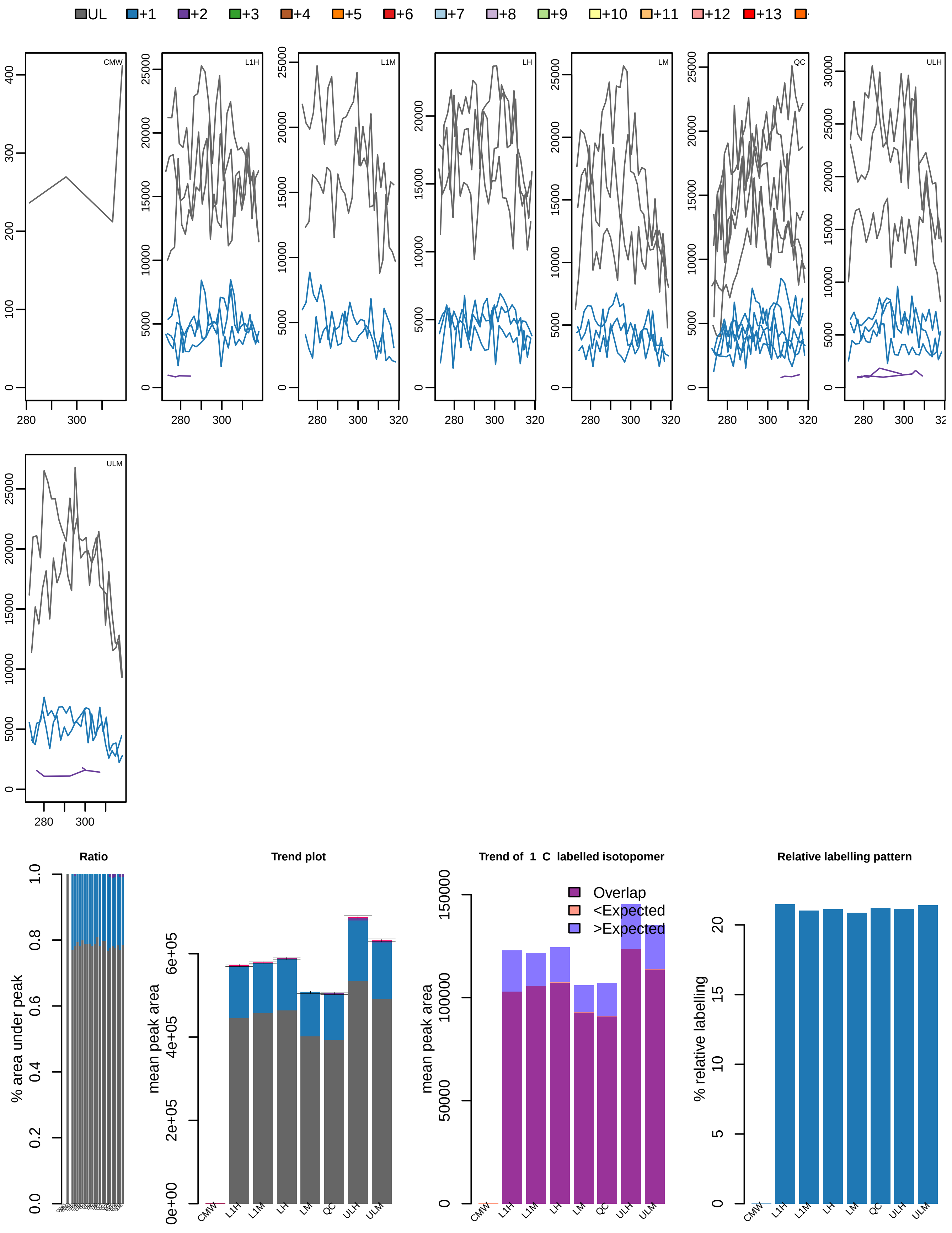

[ST] (5Z,7E)−9,10−seco−5,7,10(19)−cholestatriene

Formula: C27H44 Mass: 368.344 Std.RT: 296.3246349 Ion: POS

G2

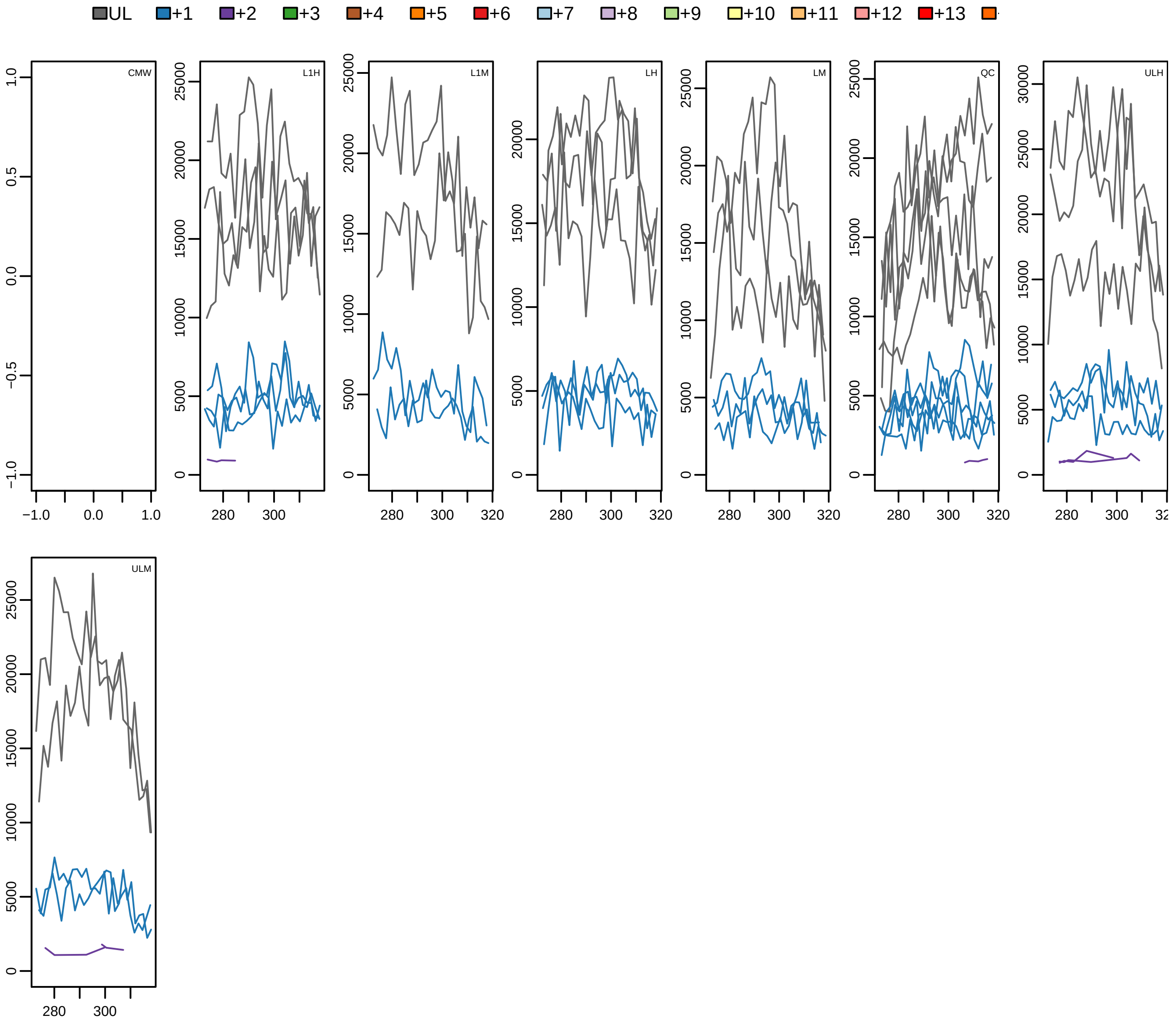

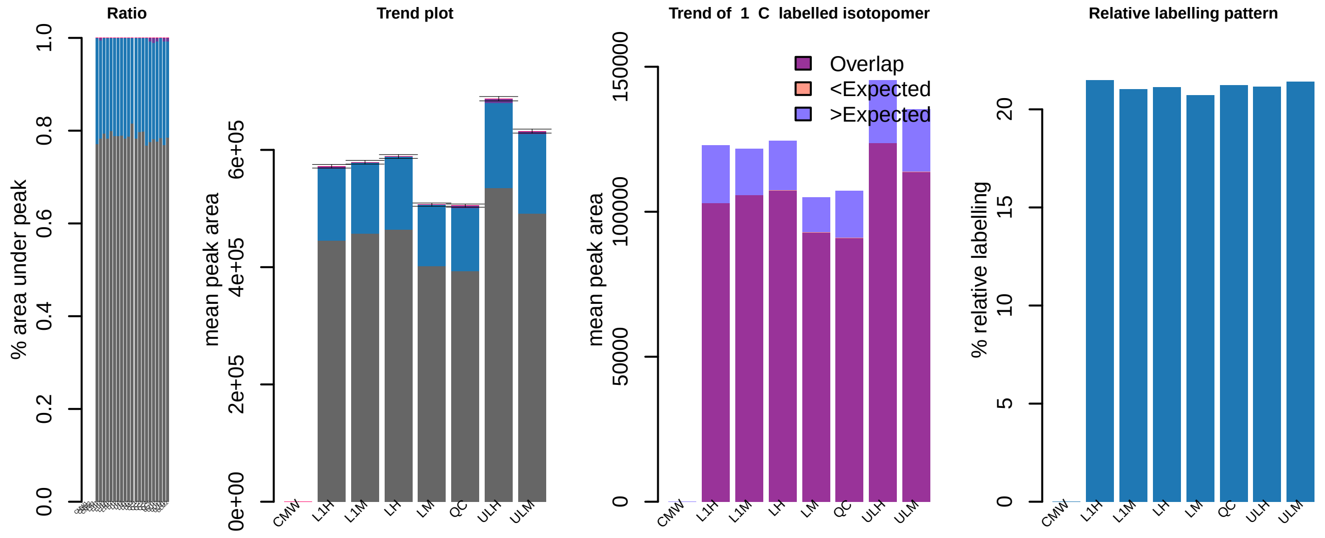

HEPES

Formula: C8H18N2O4S Mass: 238.099 Std.RT: 649.3595046 Ion: PC

G1

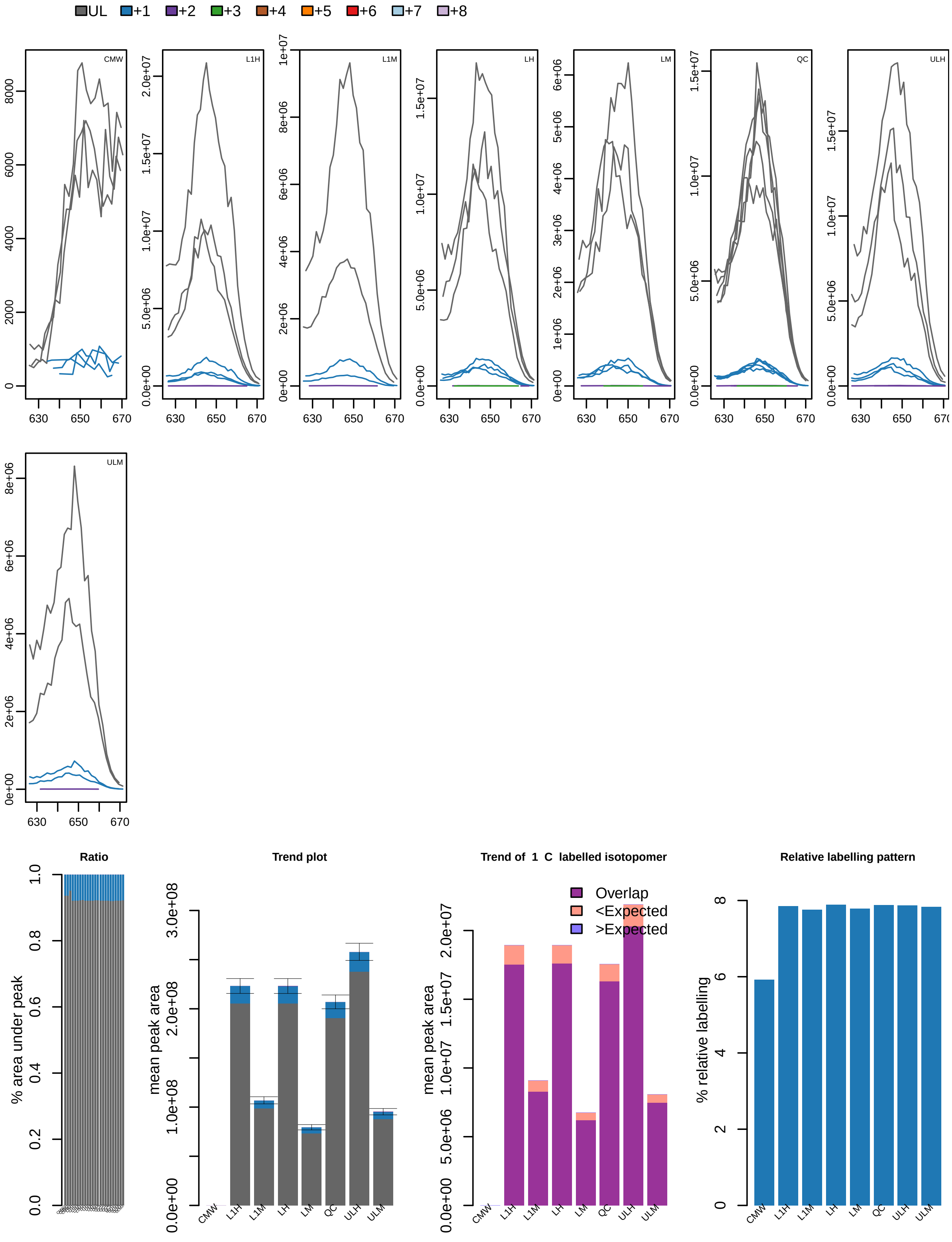

Bathocuproine disulfonic acid

Formula: C26H20N2O6S2 Mass: 520.076 Std.RT: 261.76046118 Ion

G1

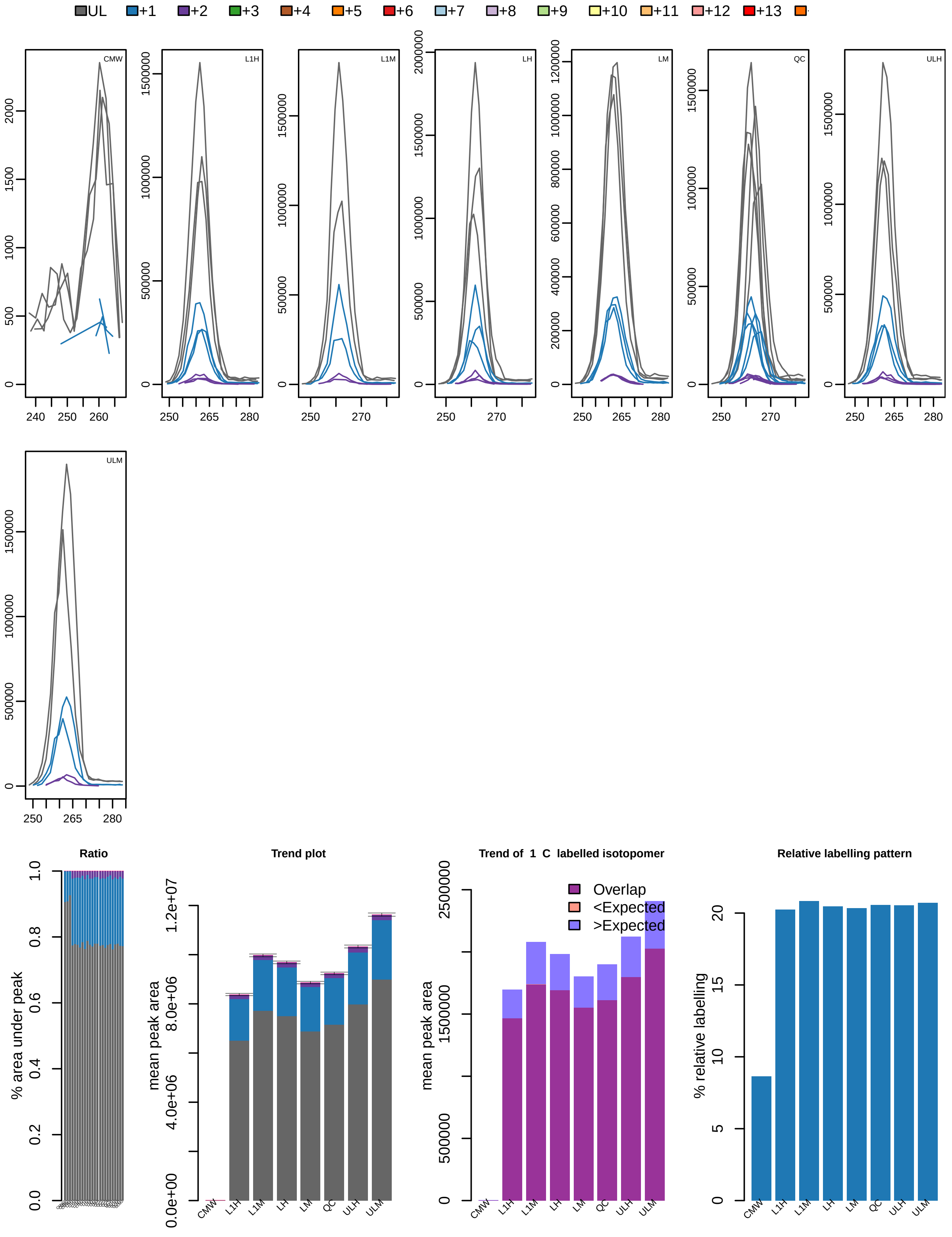

Biotin

Formula: C10H16N2O3S Mass: 244.088 Std.RT: 433.50923808 Ion:

G1

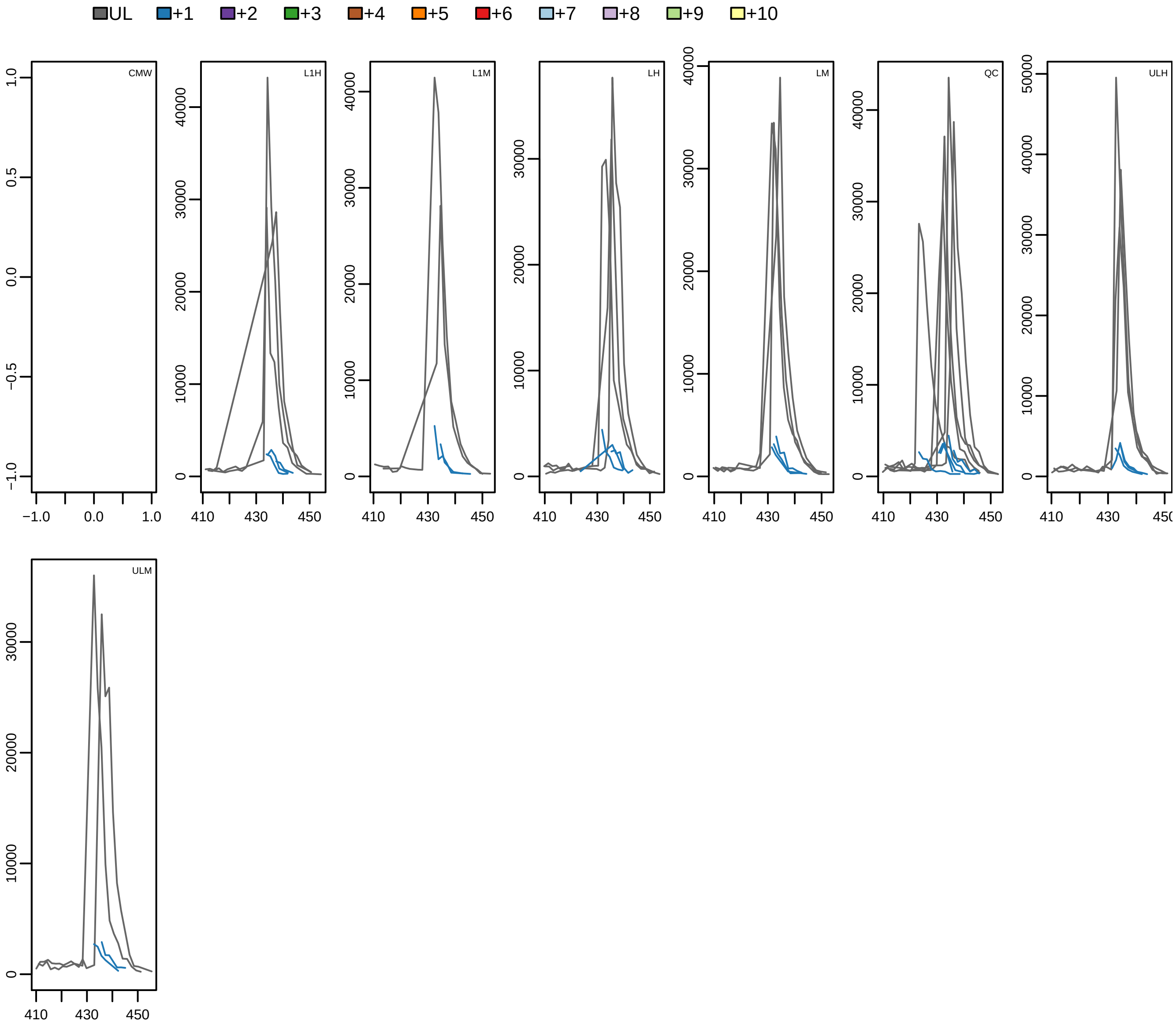

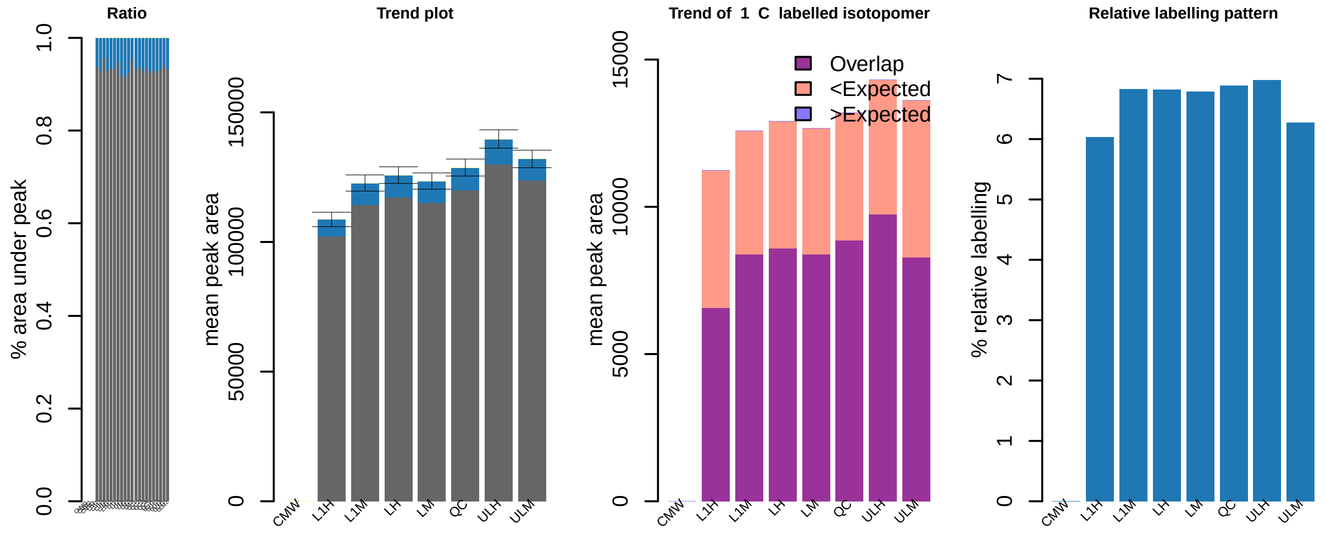

Nicotinate D−ribonucleoside

Formula: $C_{11}H_{13}NO_6$ Mass: 255.074 Std.RT: 852.6408636 Ion: POS

G1

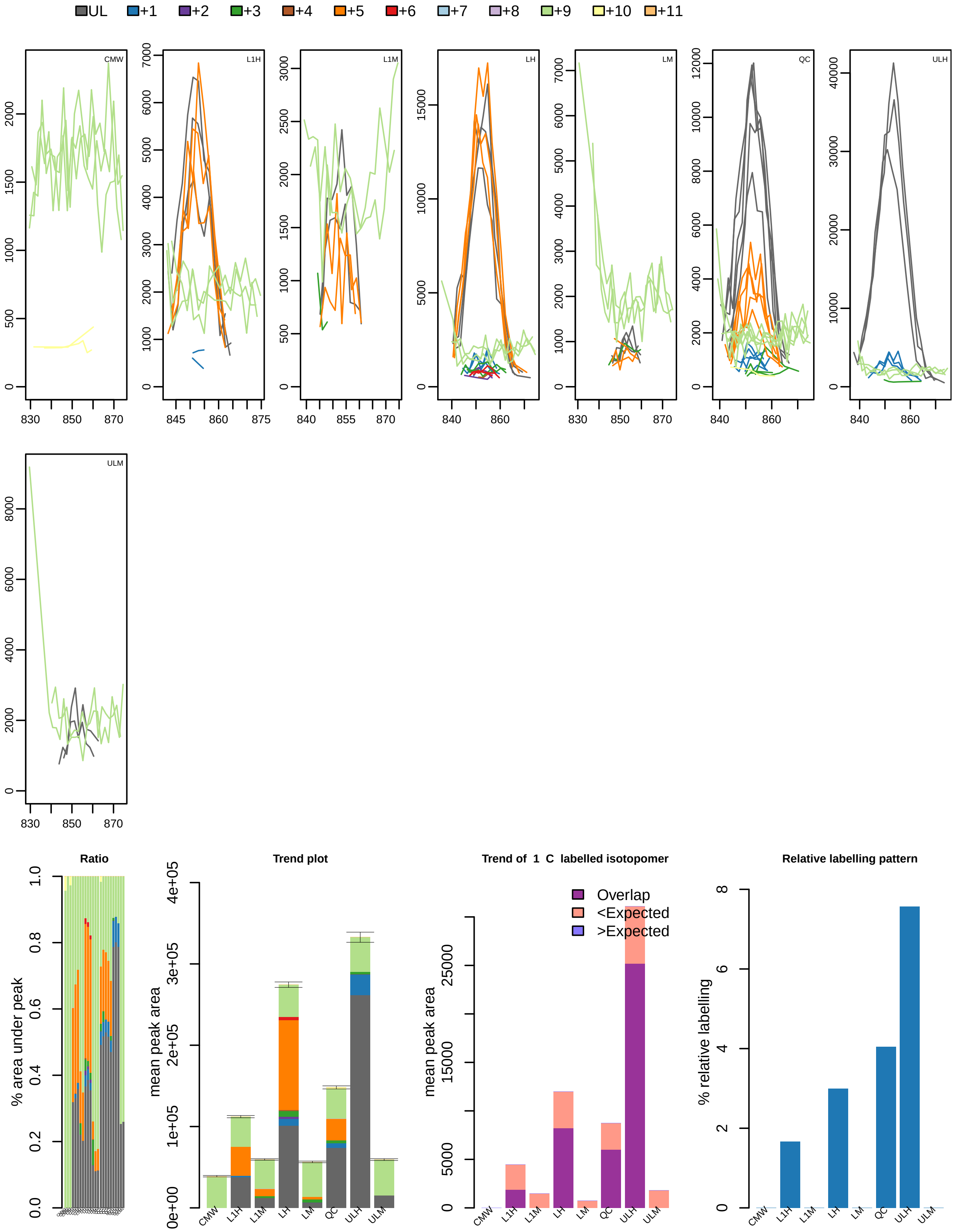

Nicotinamide

Formula: C6H6N2O Mass: 122.048 Std.RT: 434.03299842 Ion: POS

G1

UL +1 +2 +3 +4 +5 +6

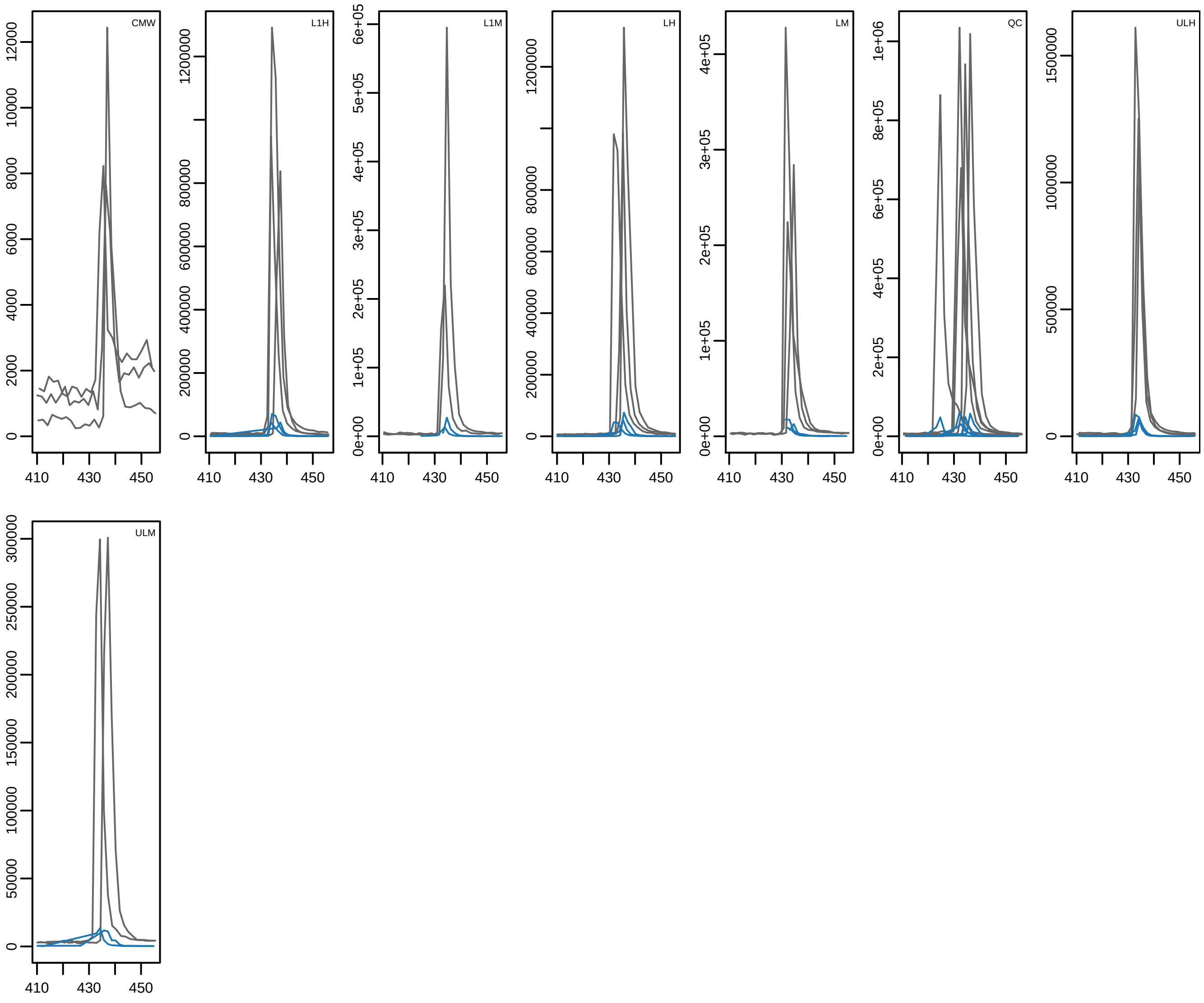

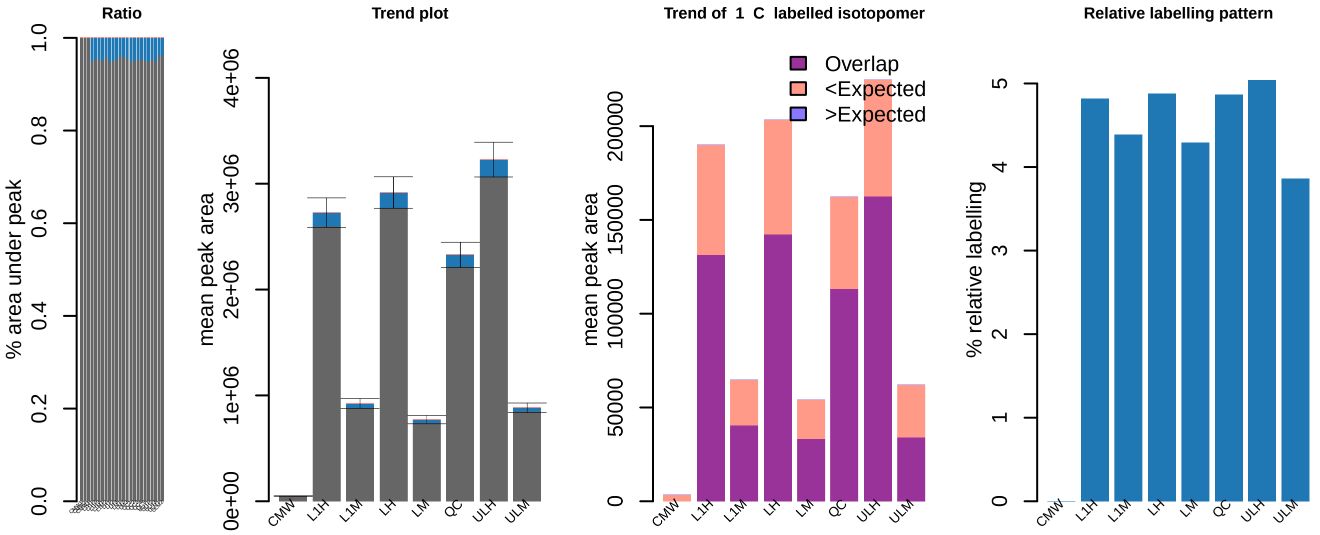

Pantothenol

Formula: $C9H19NO4$ Mass: 205.131 Std.RT: 433.2471657 Ion: POS

G1

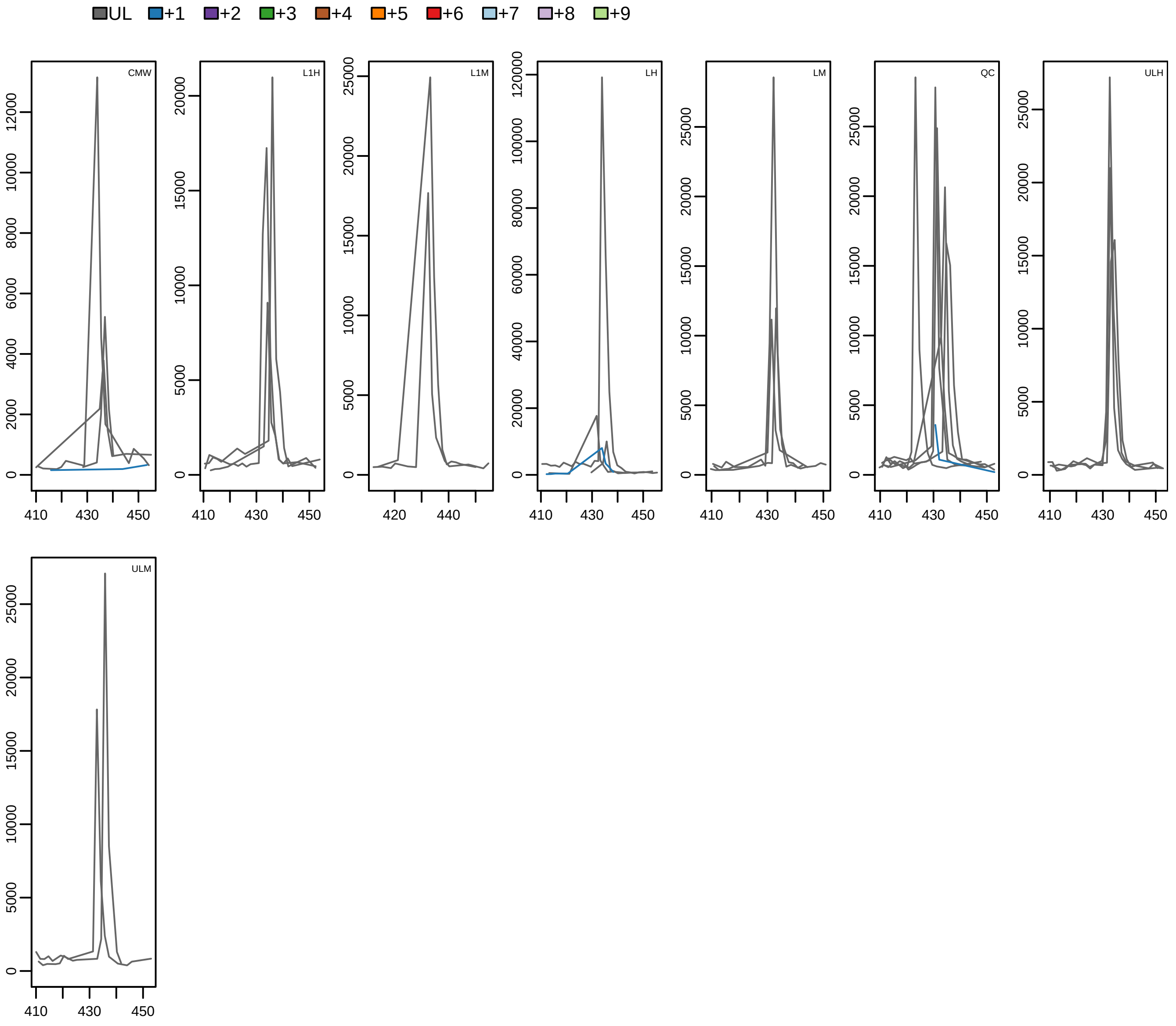

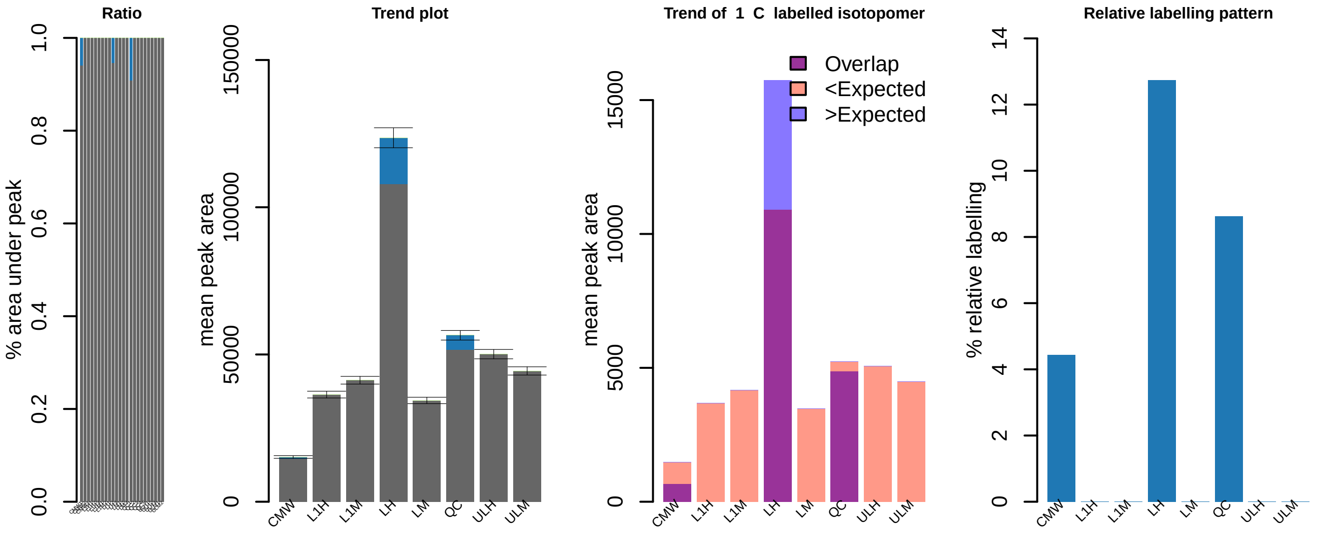

Bilirubin

Formula: C33H36N4O6 Mass: 584.263 Std.RT: 209.48685852 Ion: P

G1

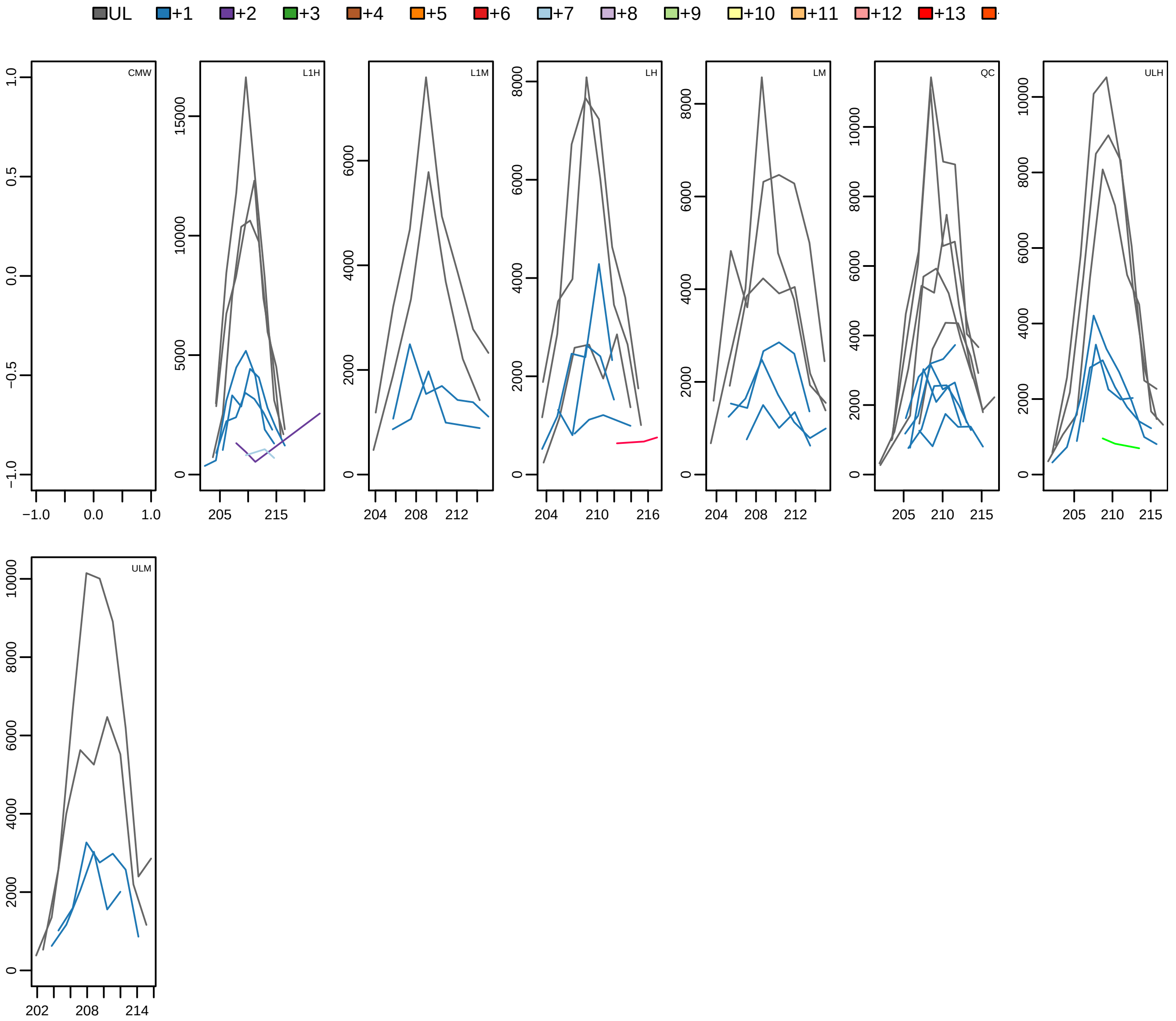

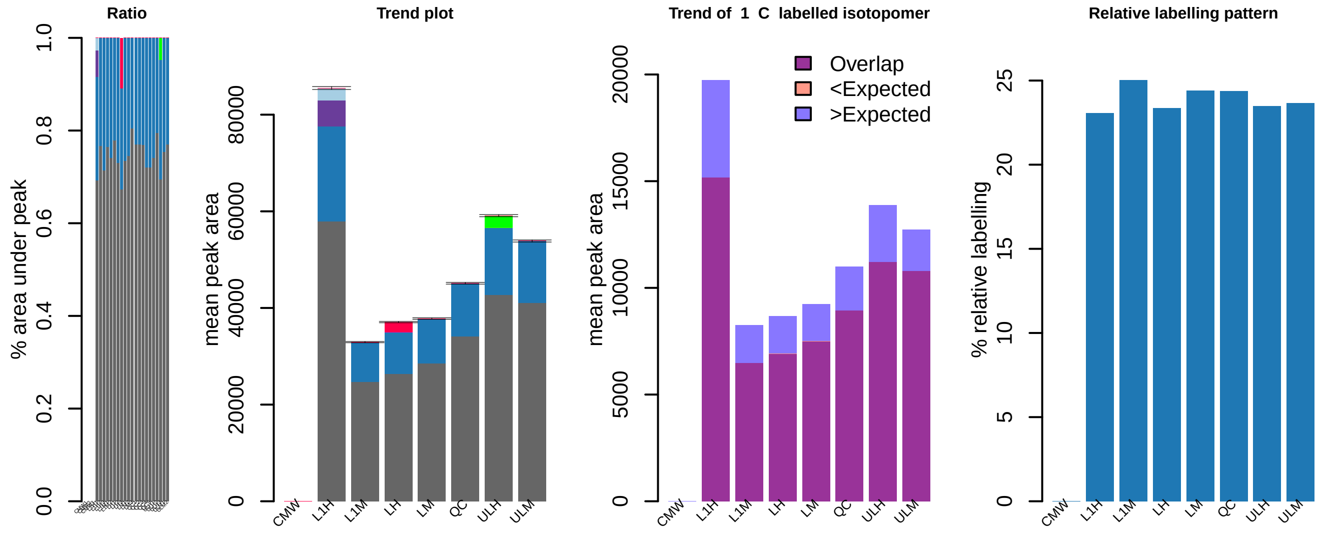

FAD

Formula: C27H33N9O15P2 Mass: 785.157 Std.RT: 729.6317196 Ion:

G1

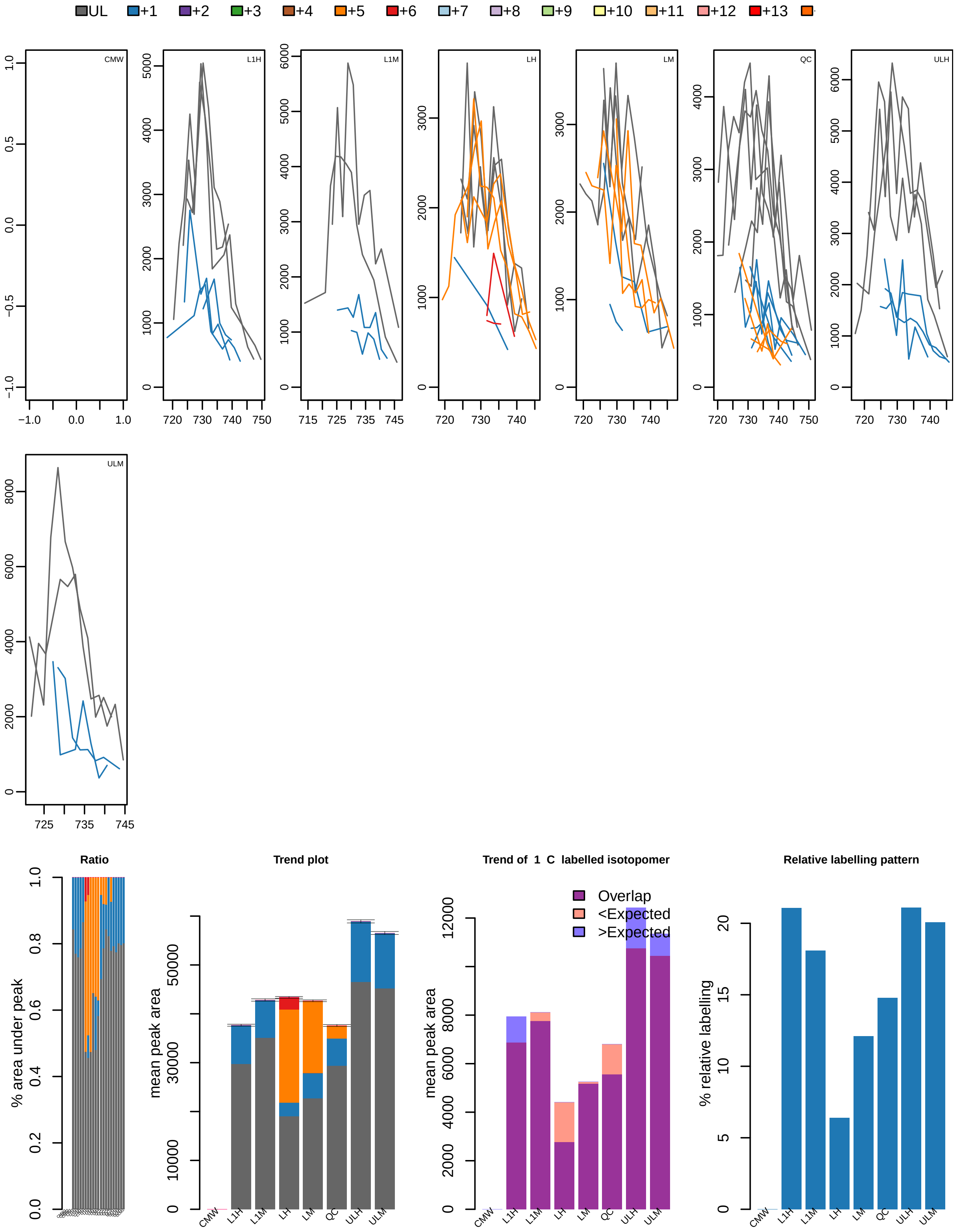

Riboflavin

Formula: C17H20N4O6 Mass: 376.138 Std.RT: 510.59994132 Ion: P

G1

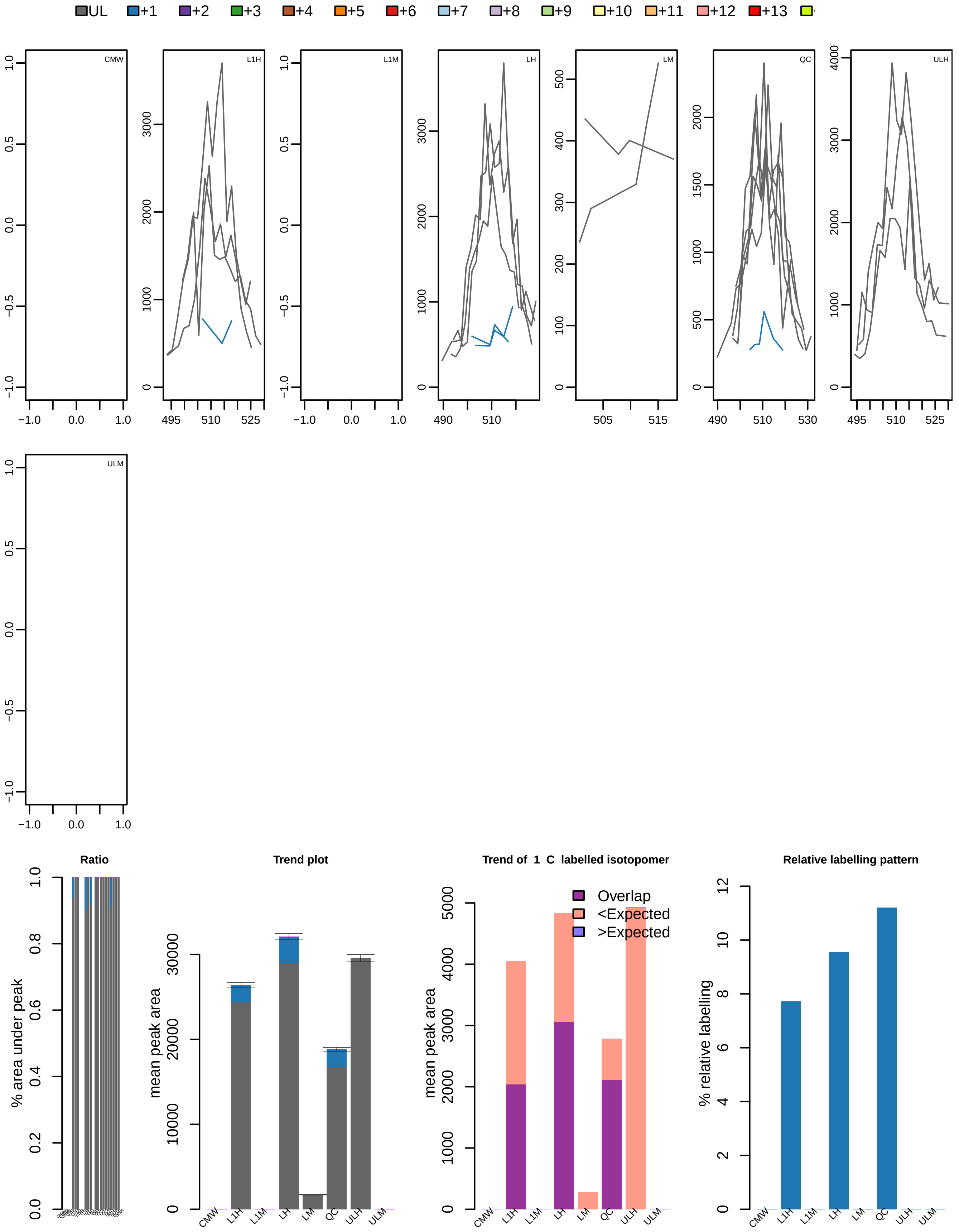

Thiamin

G1

Formula: C12H16N4OS Mass: 264.104 Std.RT: 1373.8408362 Ion: P

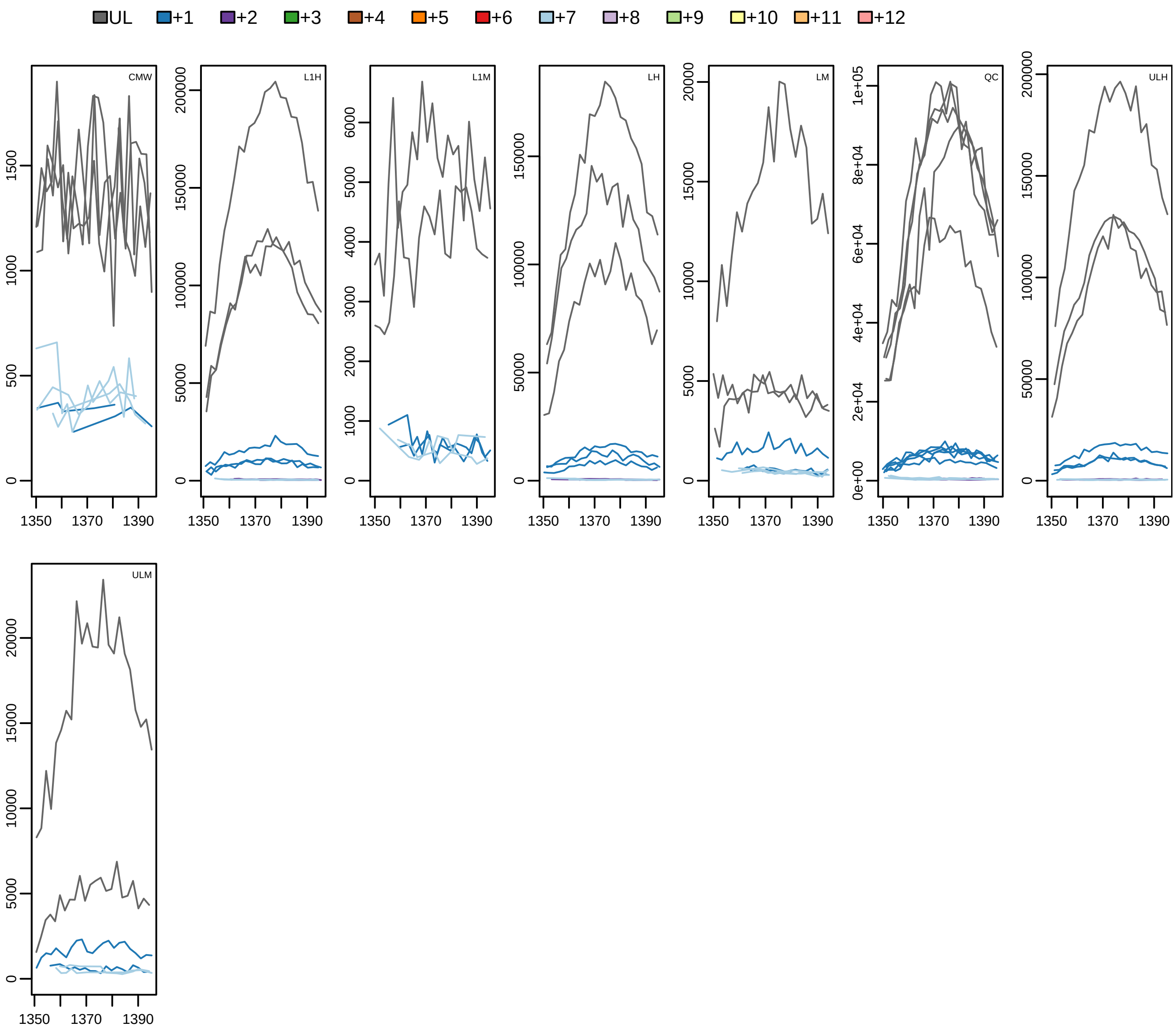

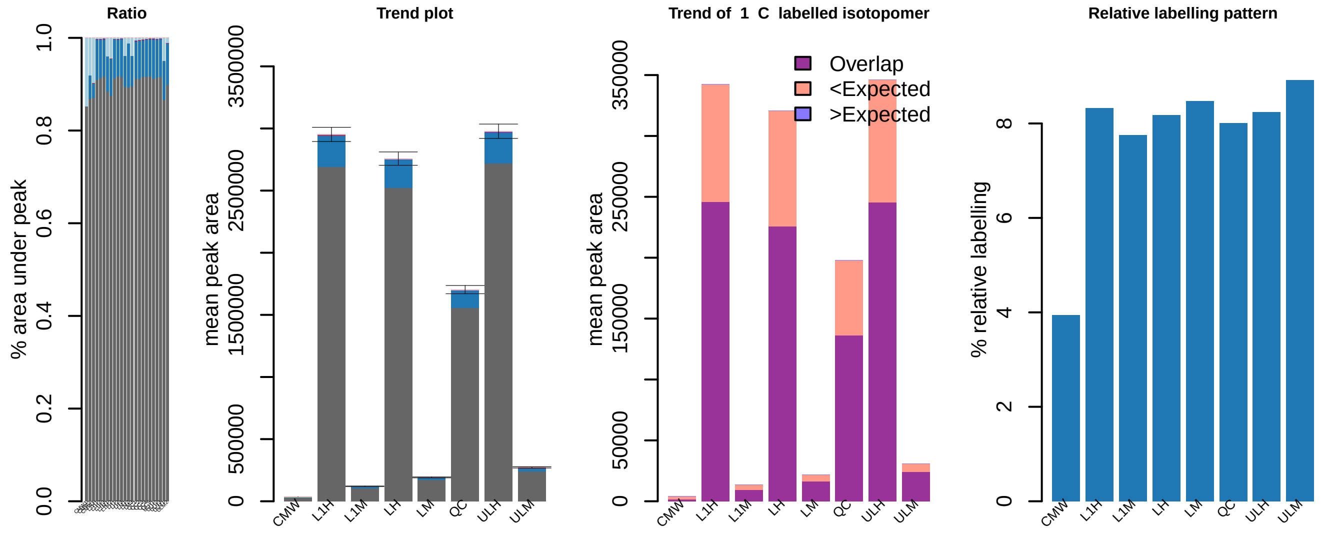

Pyridoxal

Formula: C8H9NO3 Mass: 167.058 Std.RT: 451.2128334 Ion: POS

G1

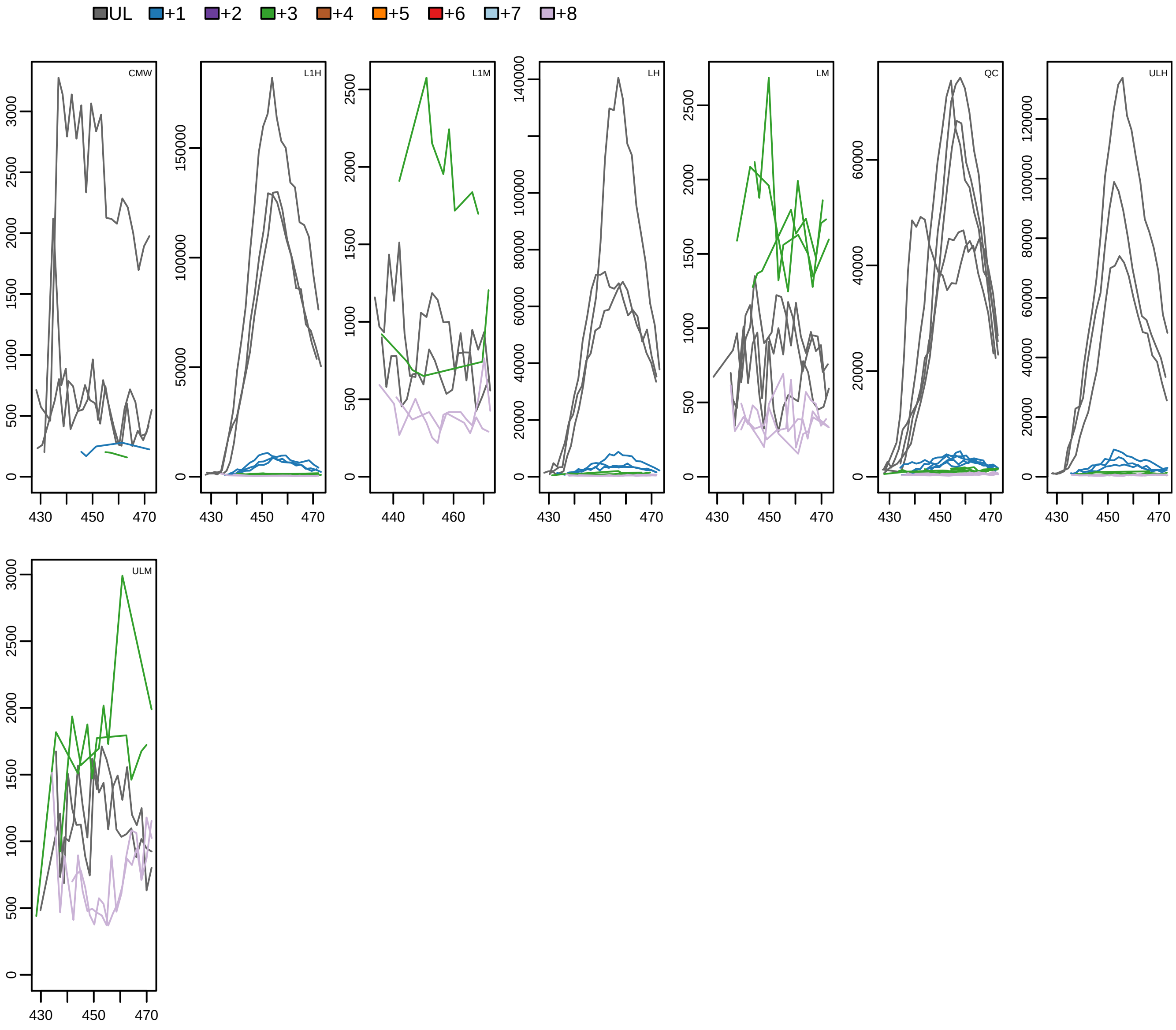

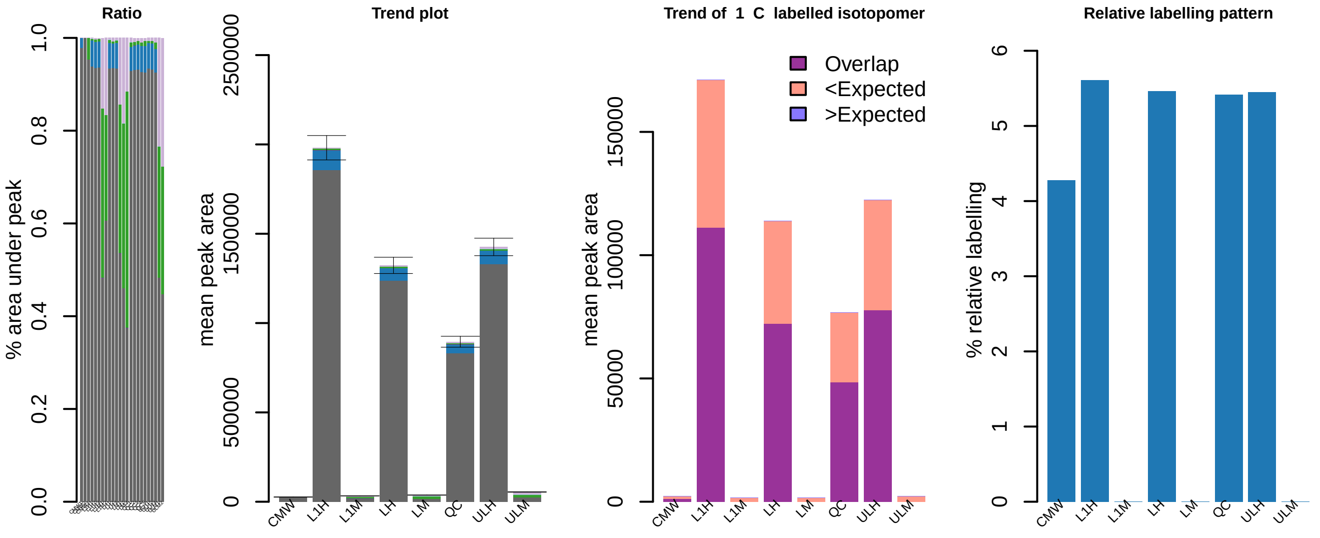

Pyridoxamine

G1

Formula: C8H12N2O2 Mass: 168.09 Std.RT: 704.6400018 Ion: POS

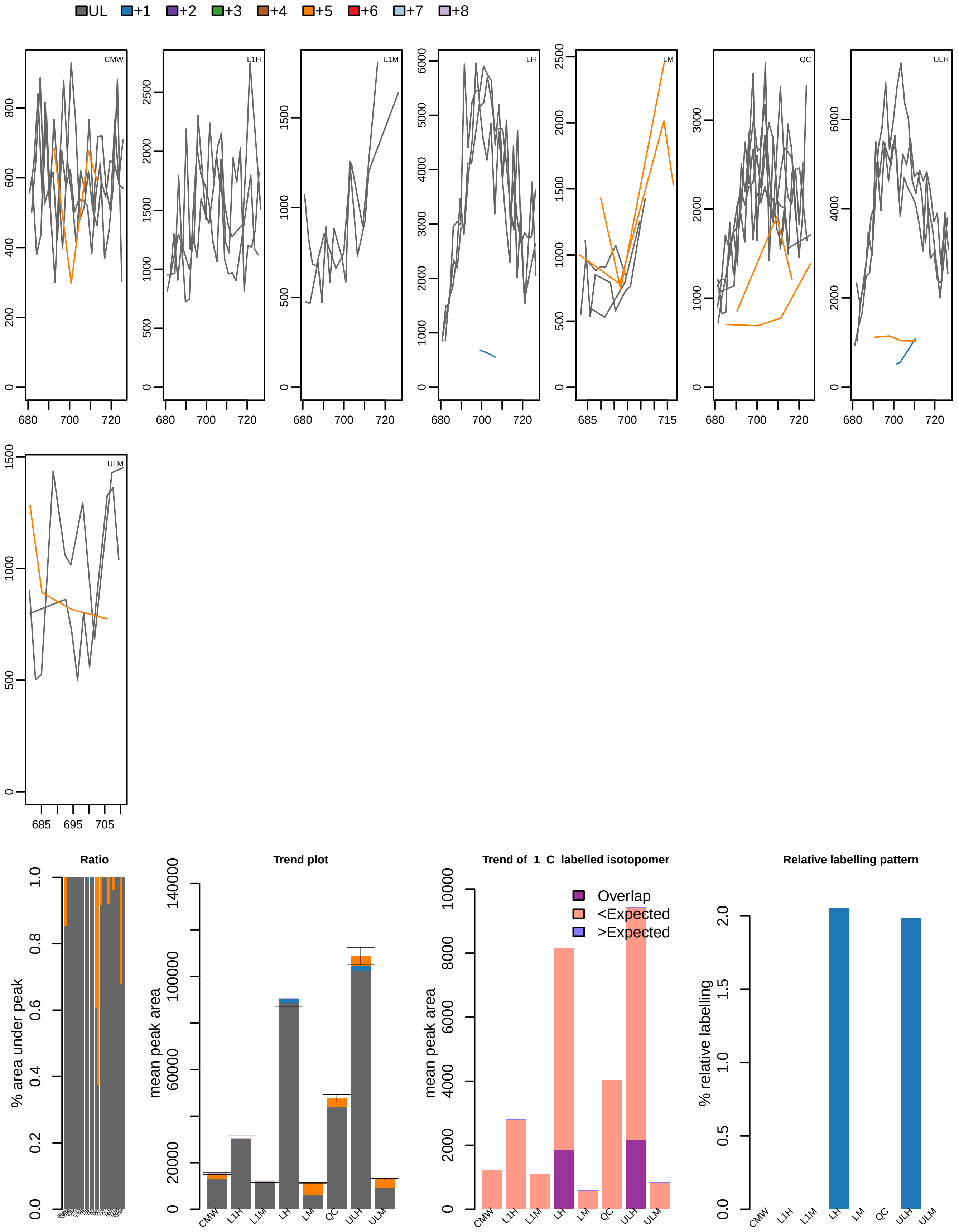

Adenosine

Formula: C10H13N5O4 Mass: 267.097 Std.RT: 547.47705588 Ion: P

G1

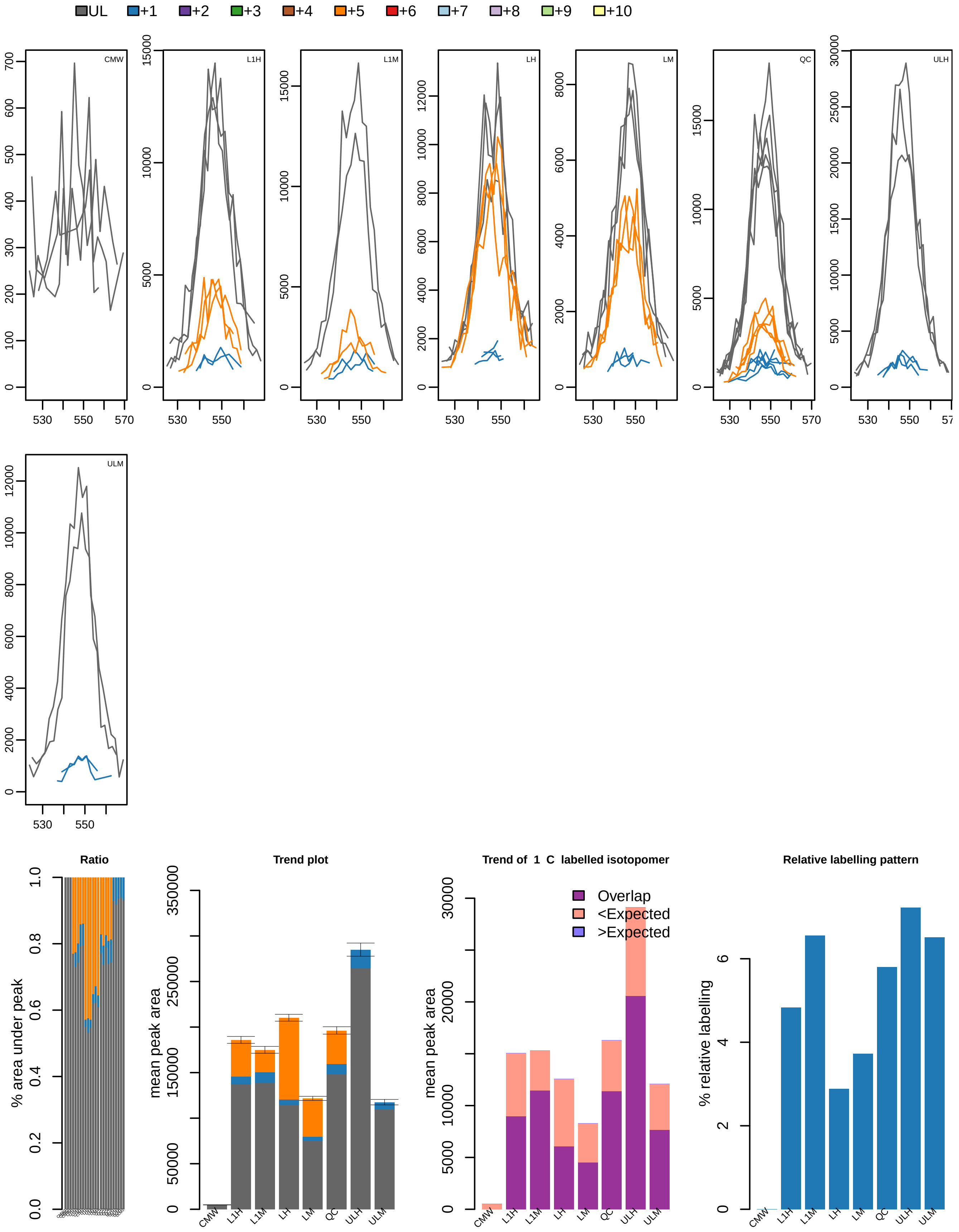

Deoxyadenosine

Formula: C10H13N5O3 Mass: 251.102 Std.RT: 449.5476837 Ion: PC

G1

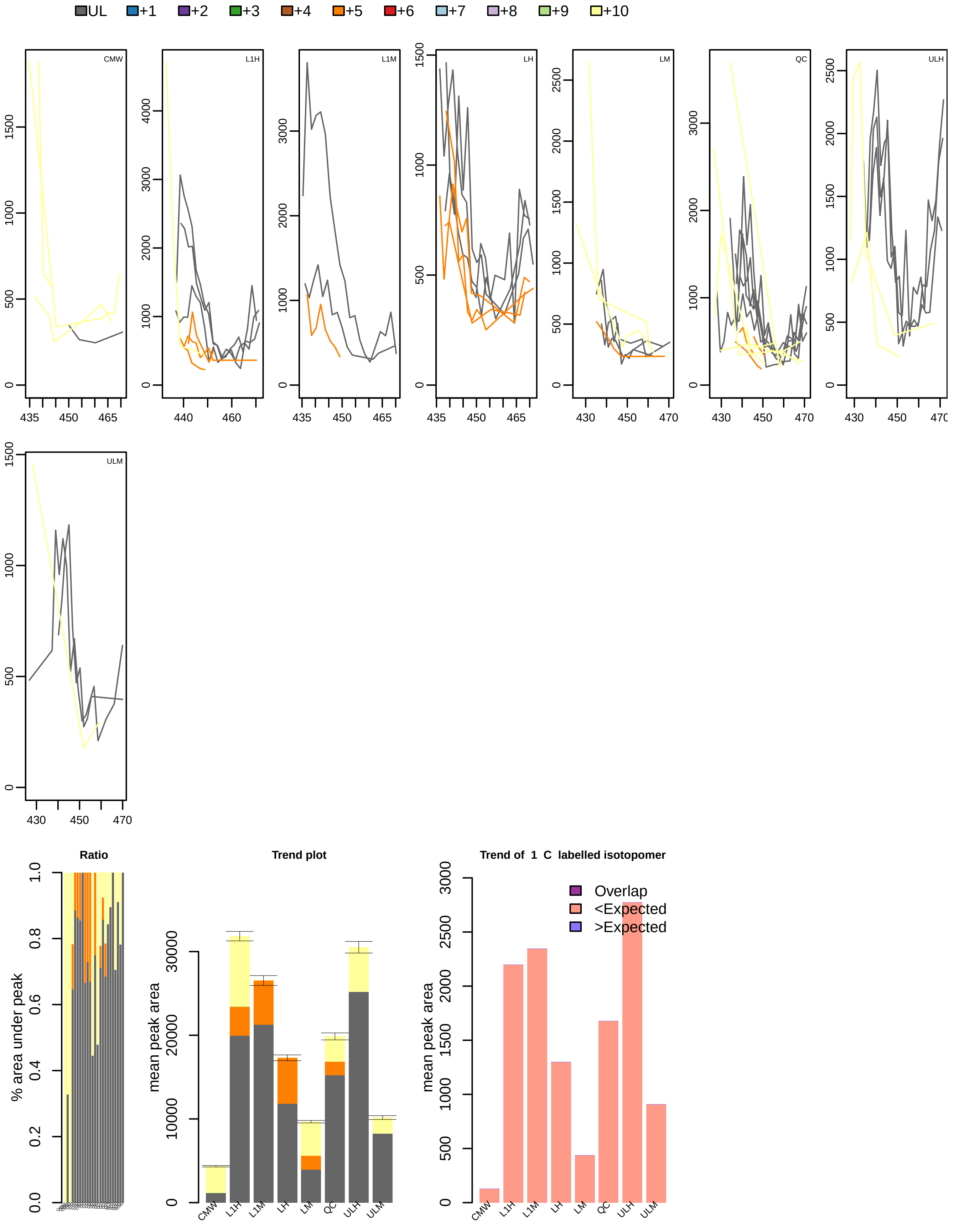

Deoxyadenosine

Formula: C10H13N5O3 Mass: 251.102 Std.RT: 449.5476837 Ion: PC

G2

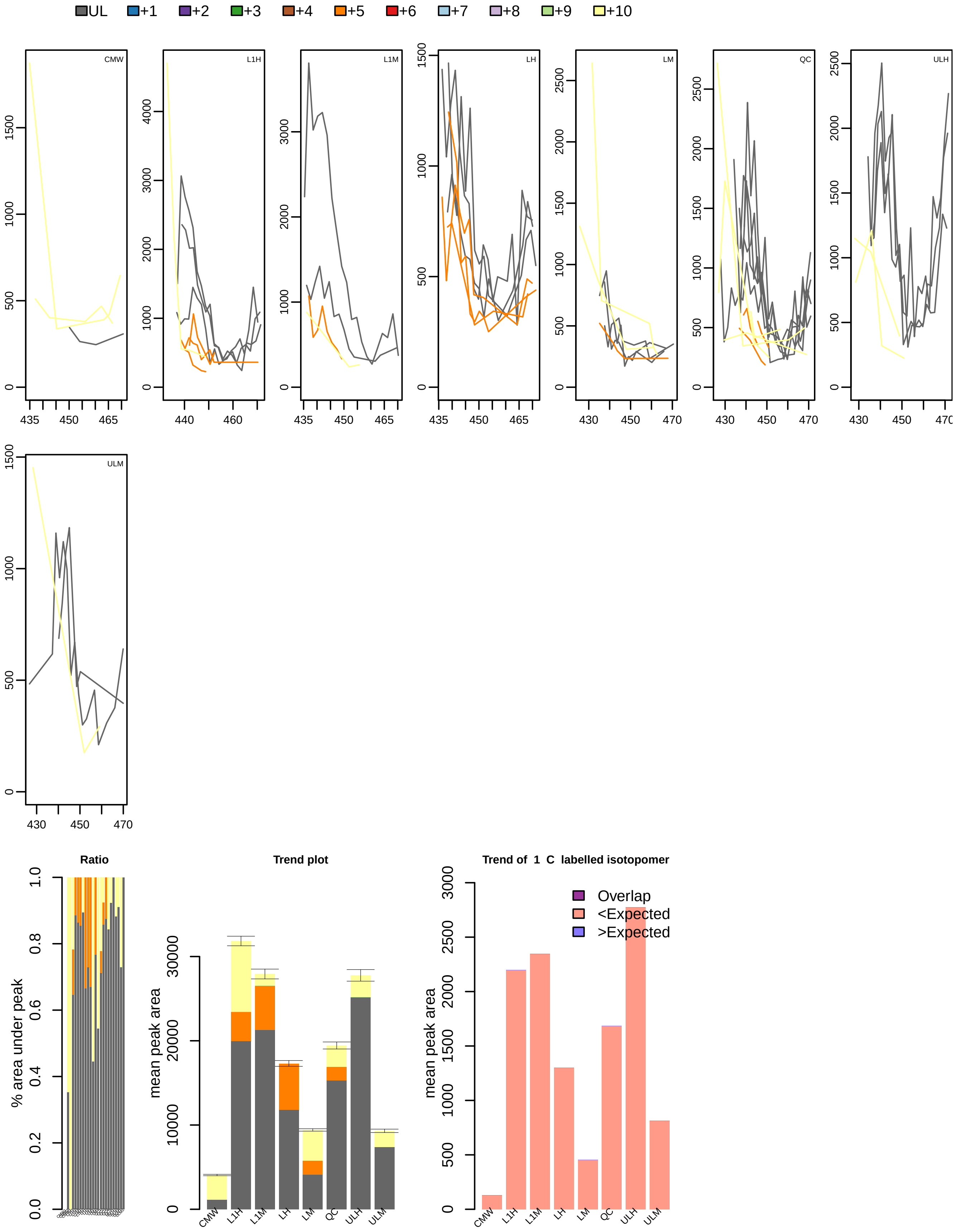

Inosine

Formula: C10H12N4O5 Mass: 268.081 Std.RT: 689.5994322 Ion: PC

G1

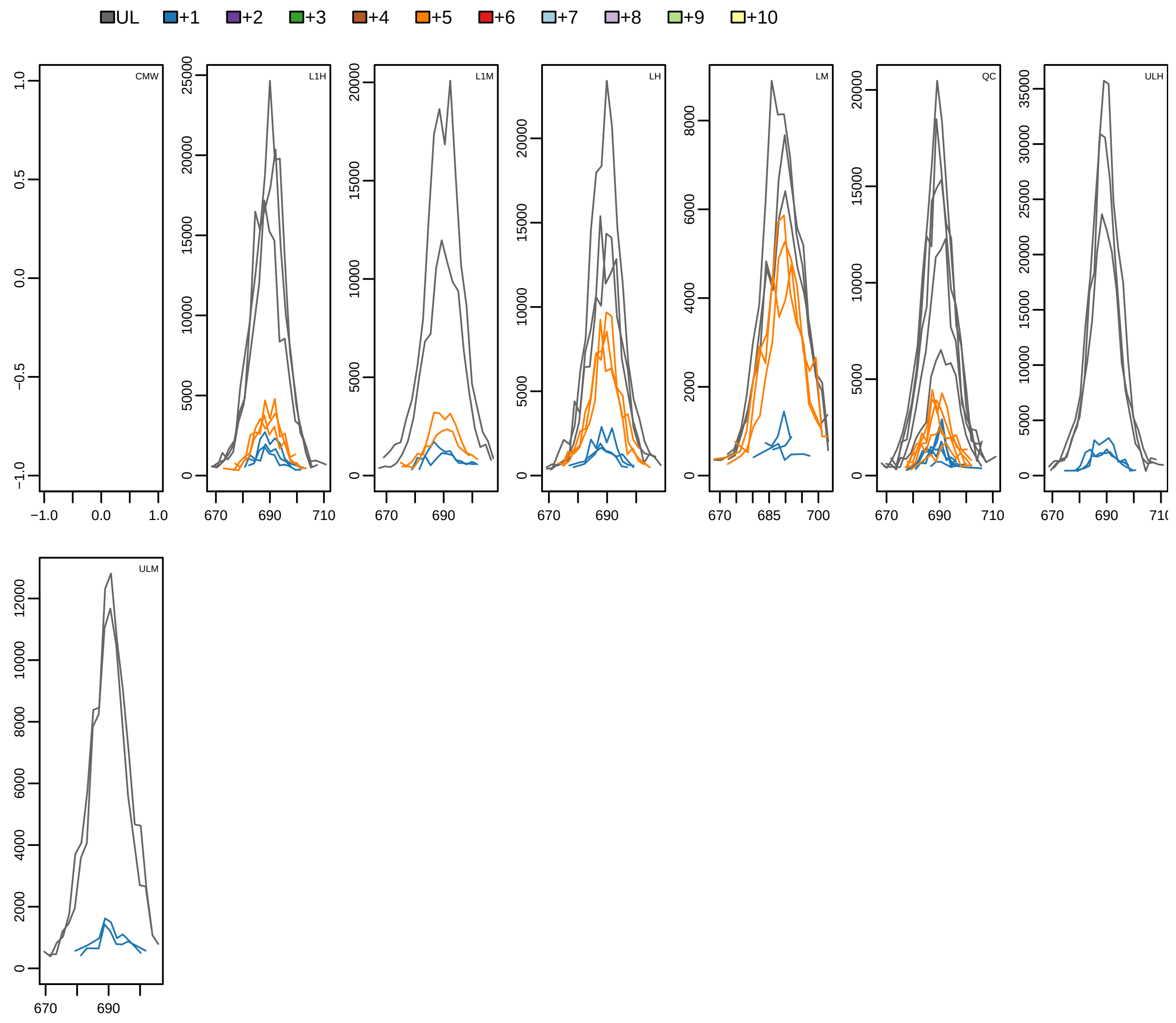

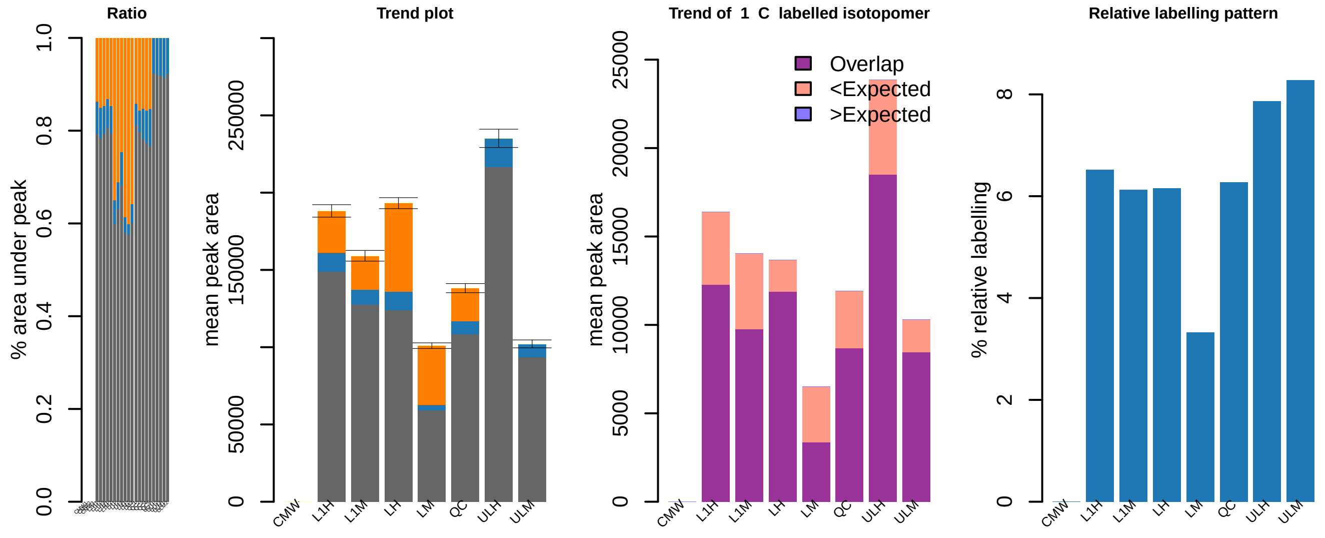

Guanosine

Formula: $C_{10}H_{13}N_5O_5$ Mass: 283.092 Std.RT: 809.696004 Ion: POS

G1

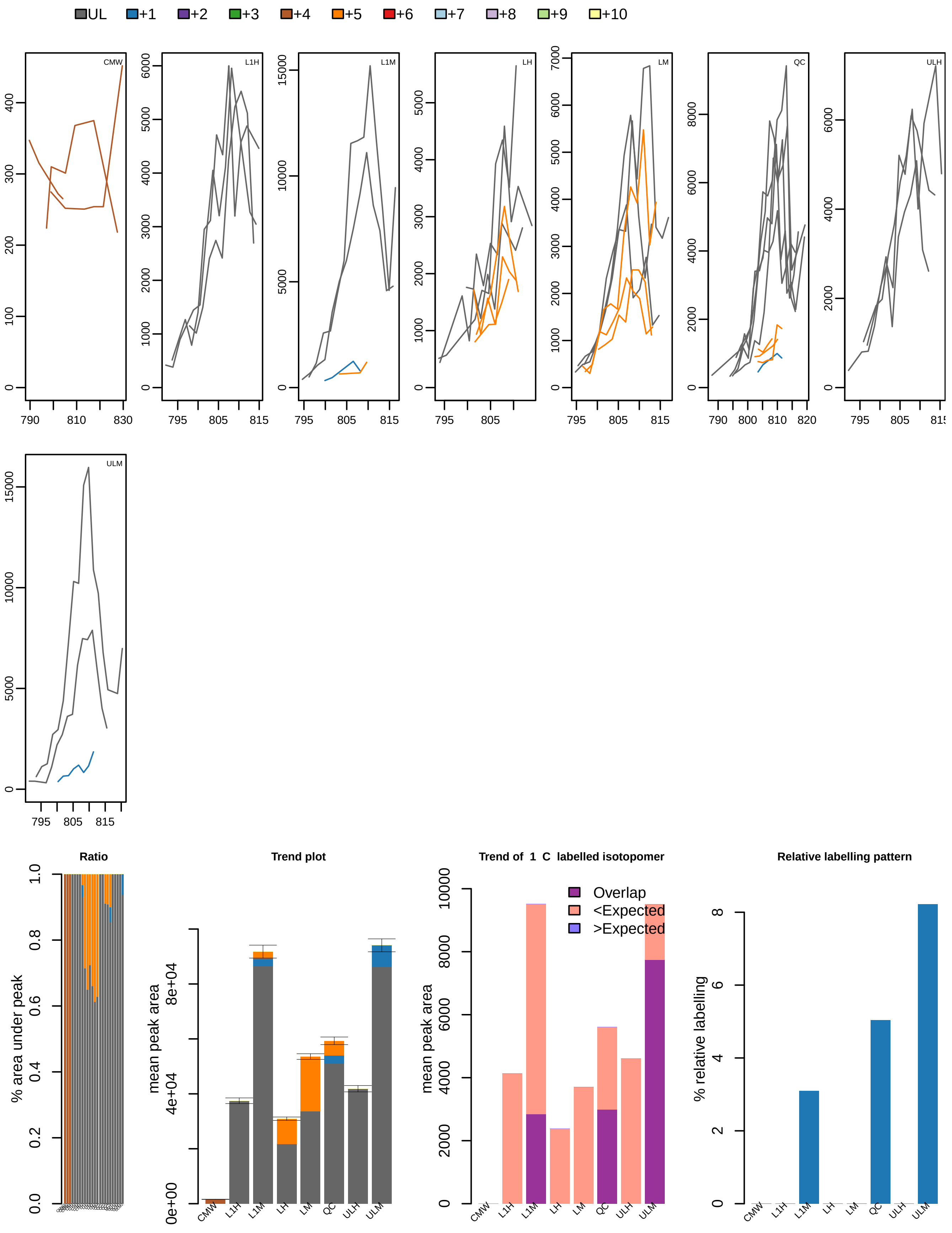

Hypoxanthine

Formula: C5H4N4O Mass: 136.039 Std.RT: 631.2075018 Ion: POS

G1

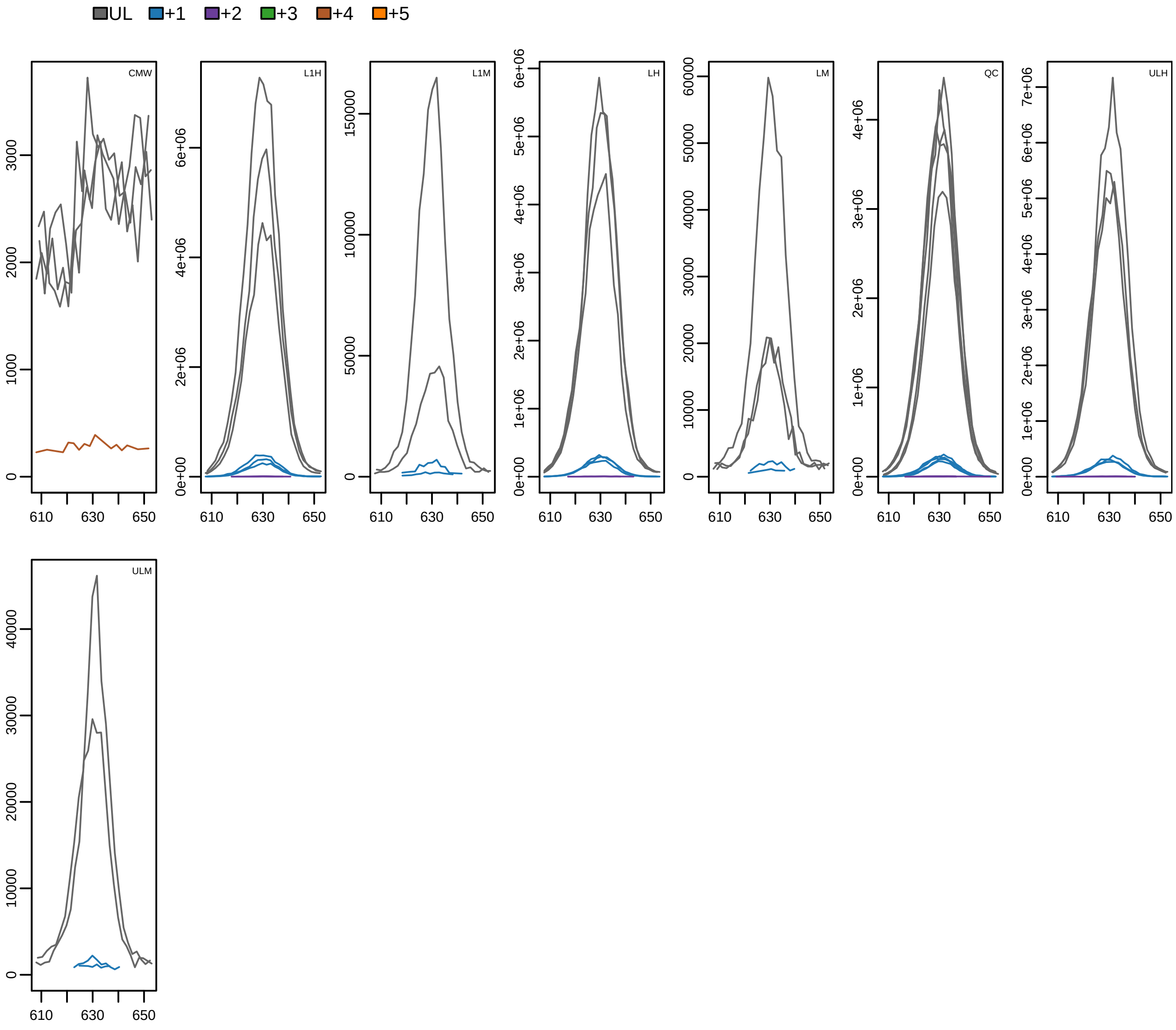

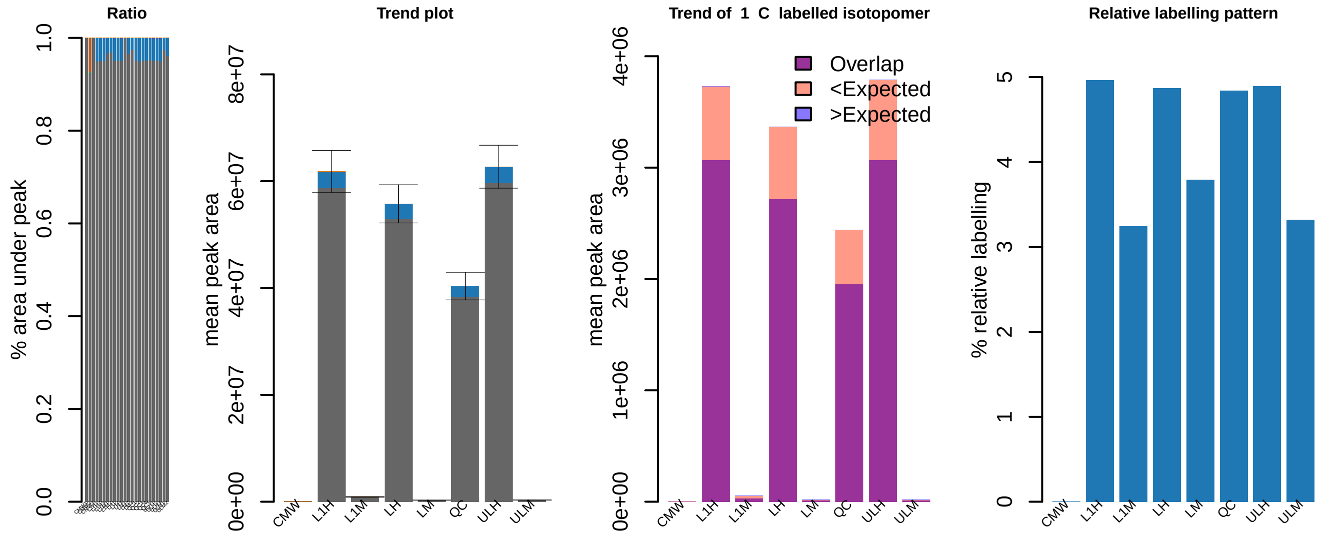

Guanine

Formula: C5H5N5O Mass: 151.049 Std.RT: 788.2633614 Ion: POS

G1

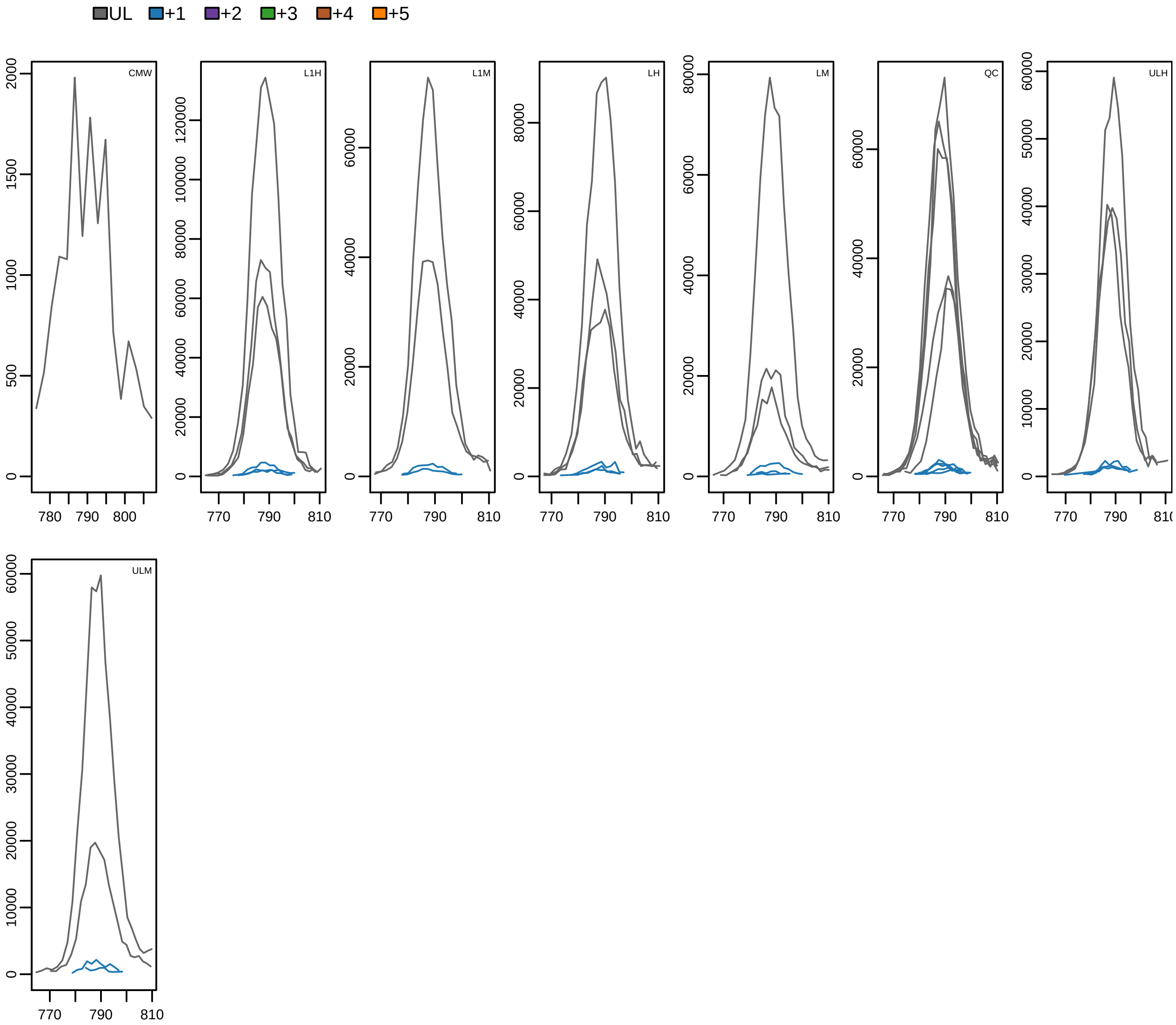

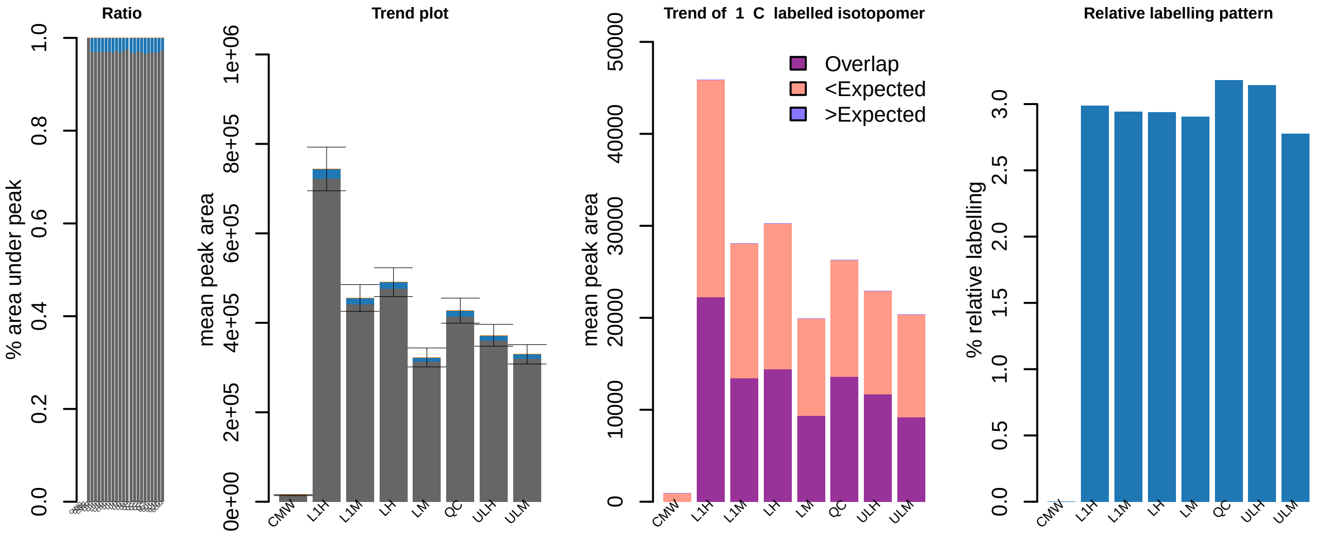

AMP

Formula: $C_{10}H_{14}N_5O_7P$ Mass: 347.063 Std.RT: 895.3342926 Ion: P

G1

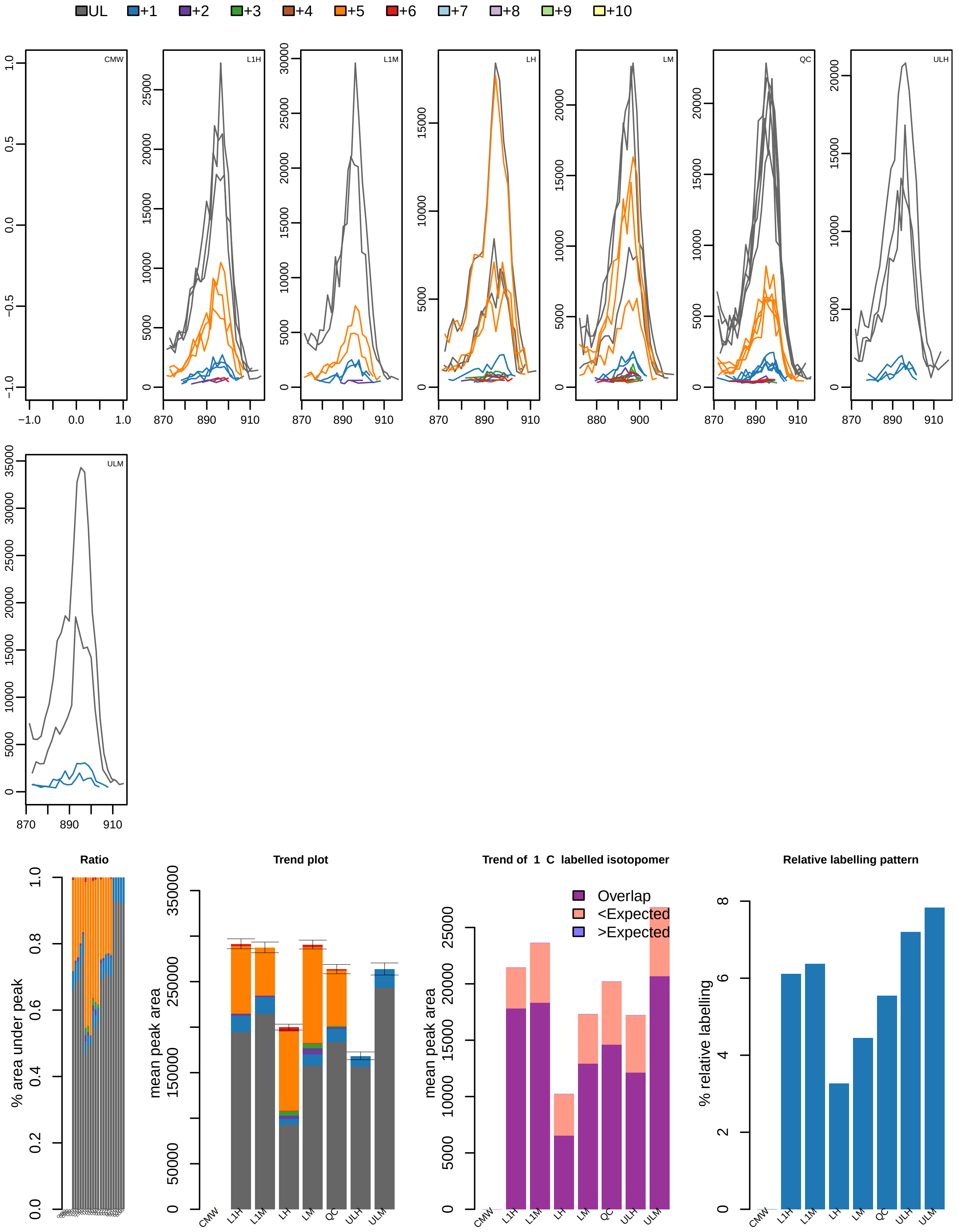

AMP

Formula: C10H14N5O7P Mass: 347.063 Std.RT: 895.3342926 Ion: P

G2

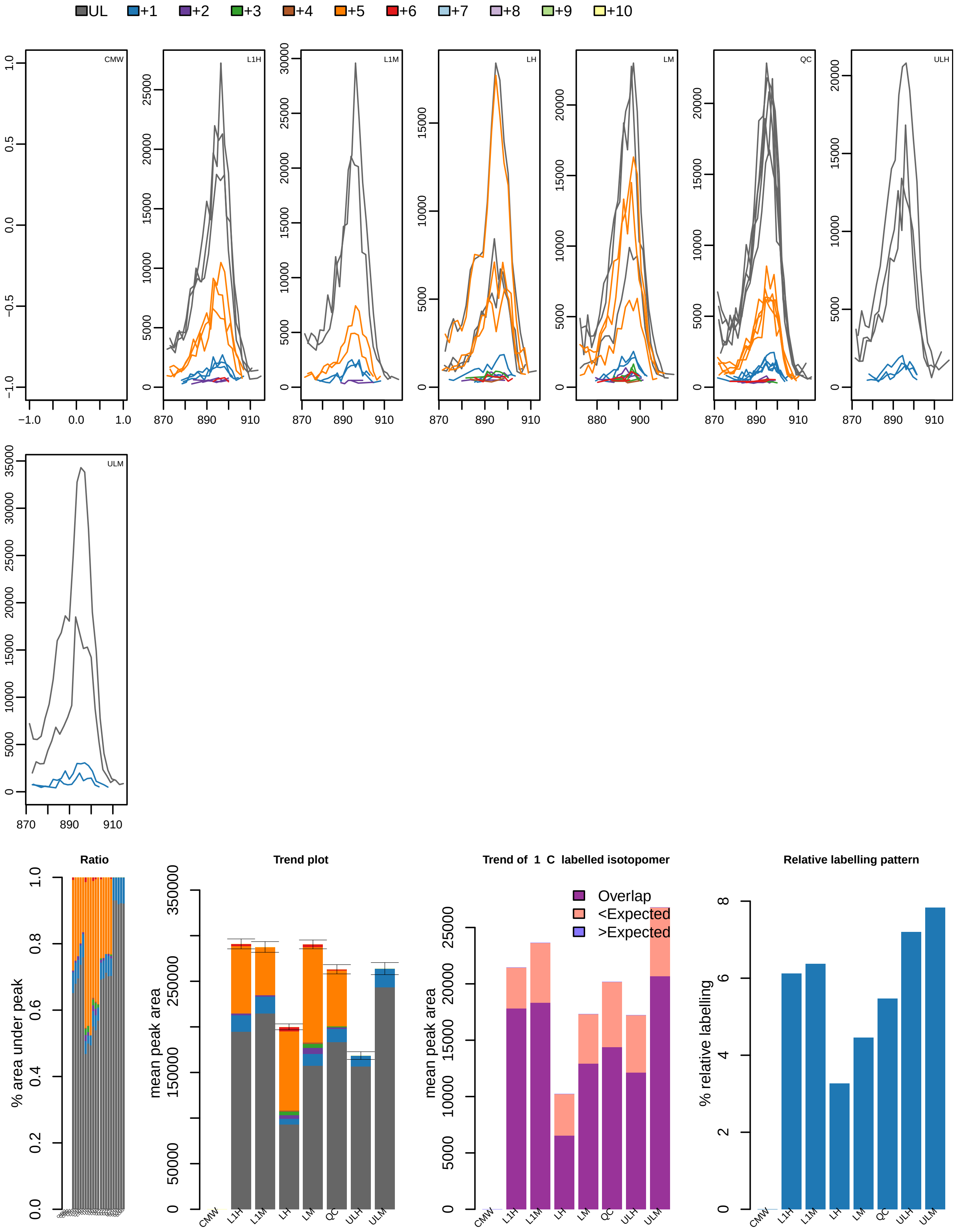

Adenine

Formula: C5H5N5 Mass: 135.054 Std.RT: 585.39645888 Ion: POS

G1

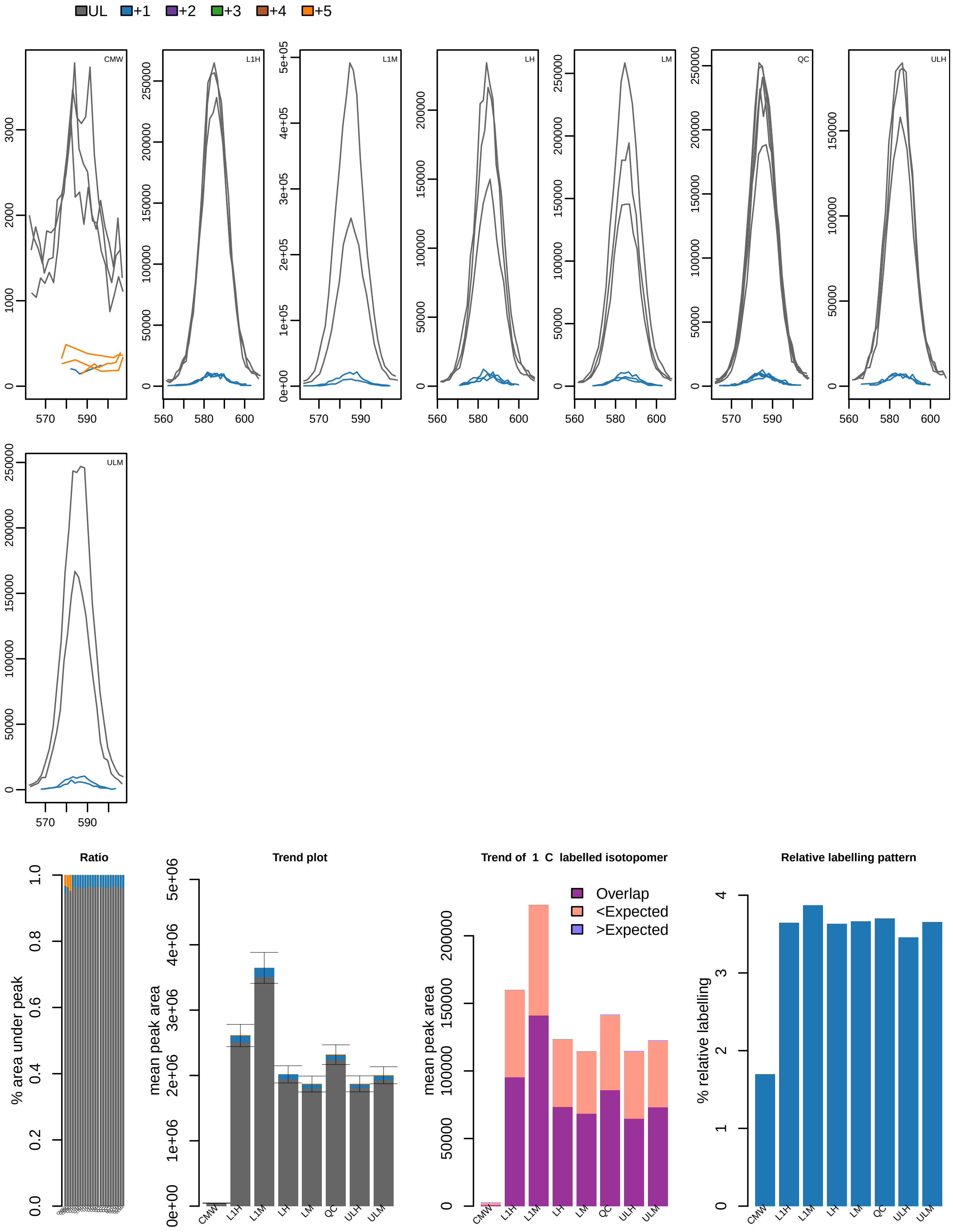

CMP

Formula: C9H14N3O8P Mass: 323.052 Std.RT: 1063.7499942 Ion: P

G1

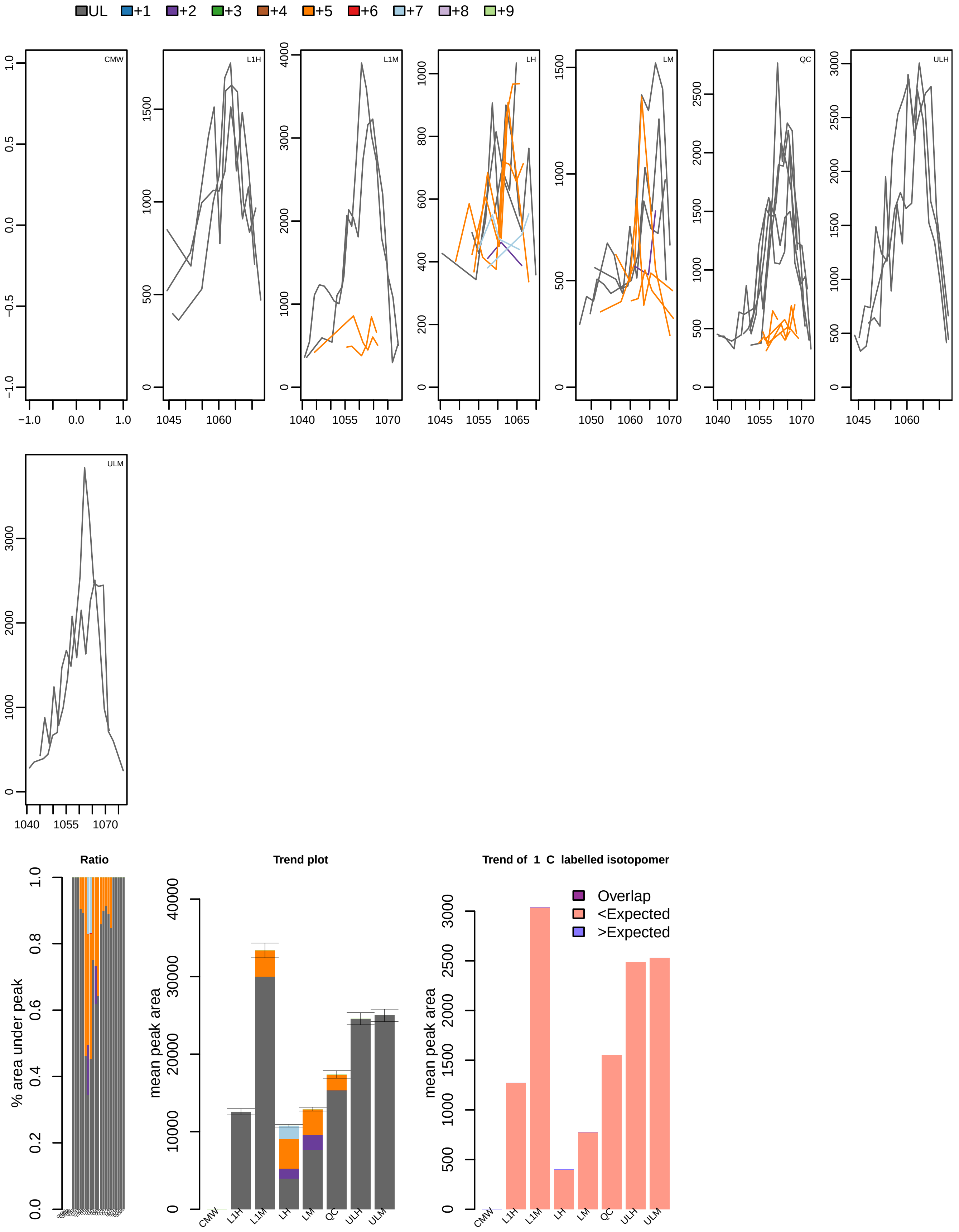

5−Methylcytosine

Formula: C5H7N3O Mass: 125.059 Std.RT: 630.2100036 Ion: POS

G1

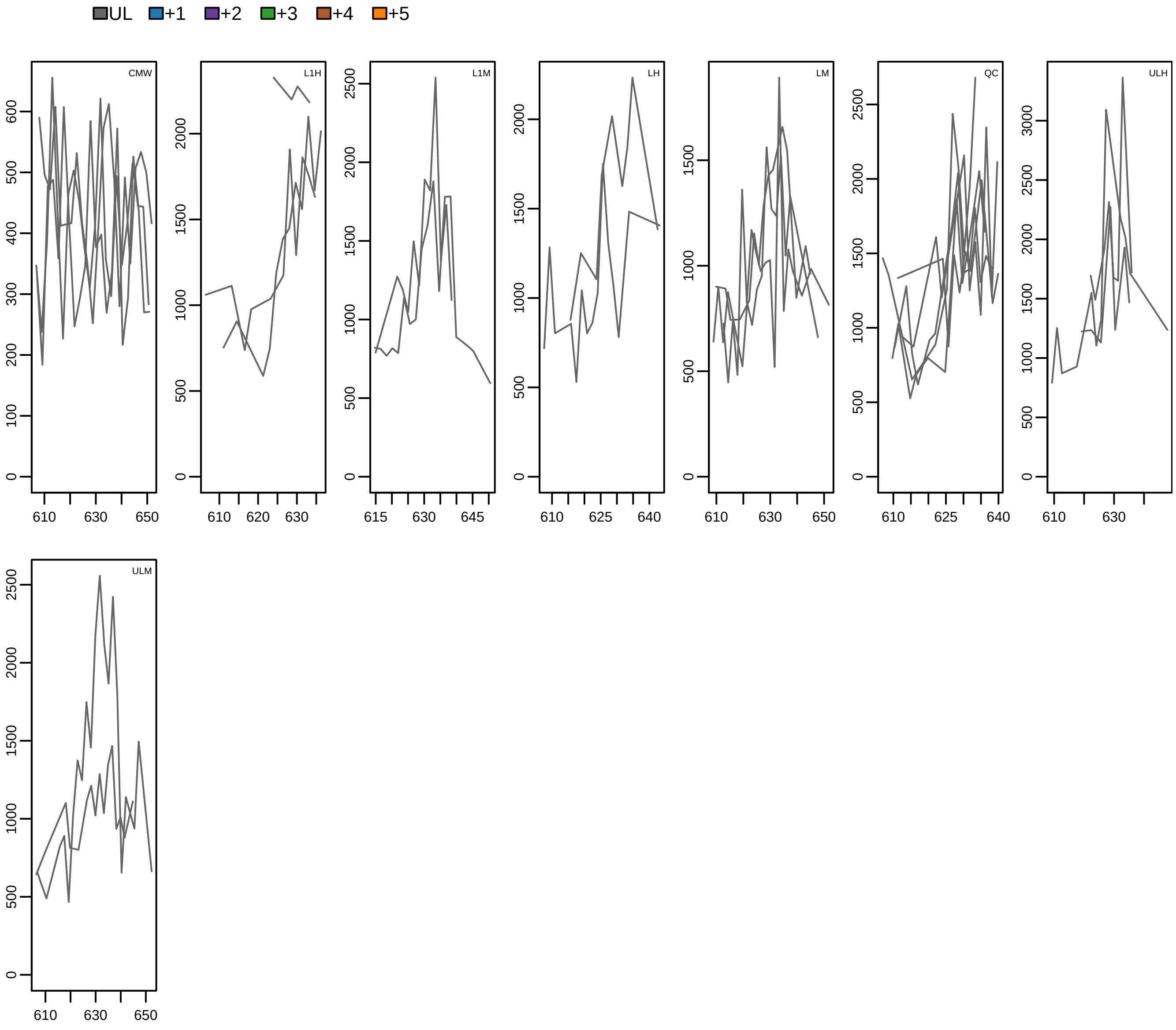

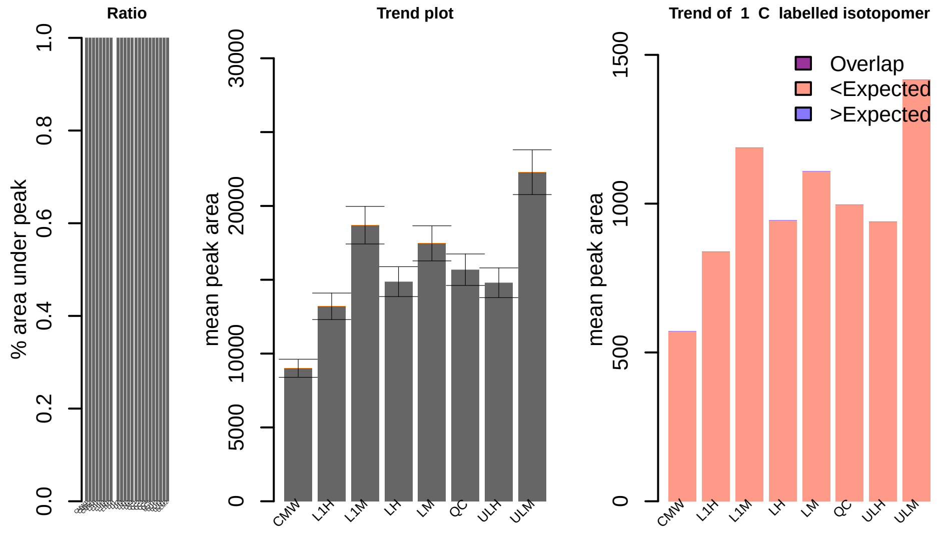

Cytidine

Formula: C9H13N3O5 Mass: 243.086 Std.RT: 761.822001 Ion: POS

G1

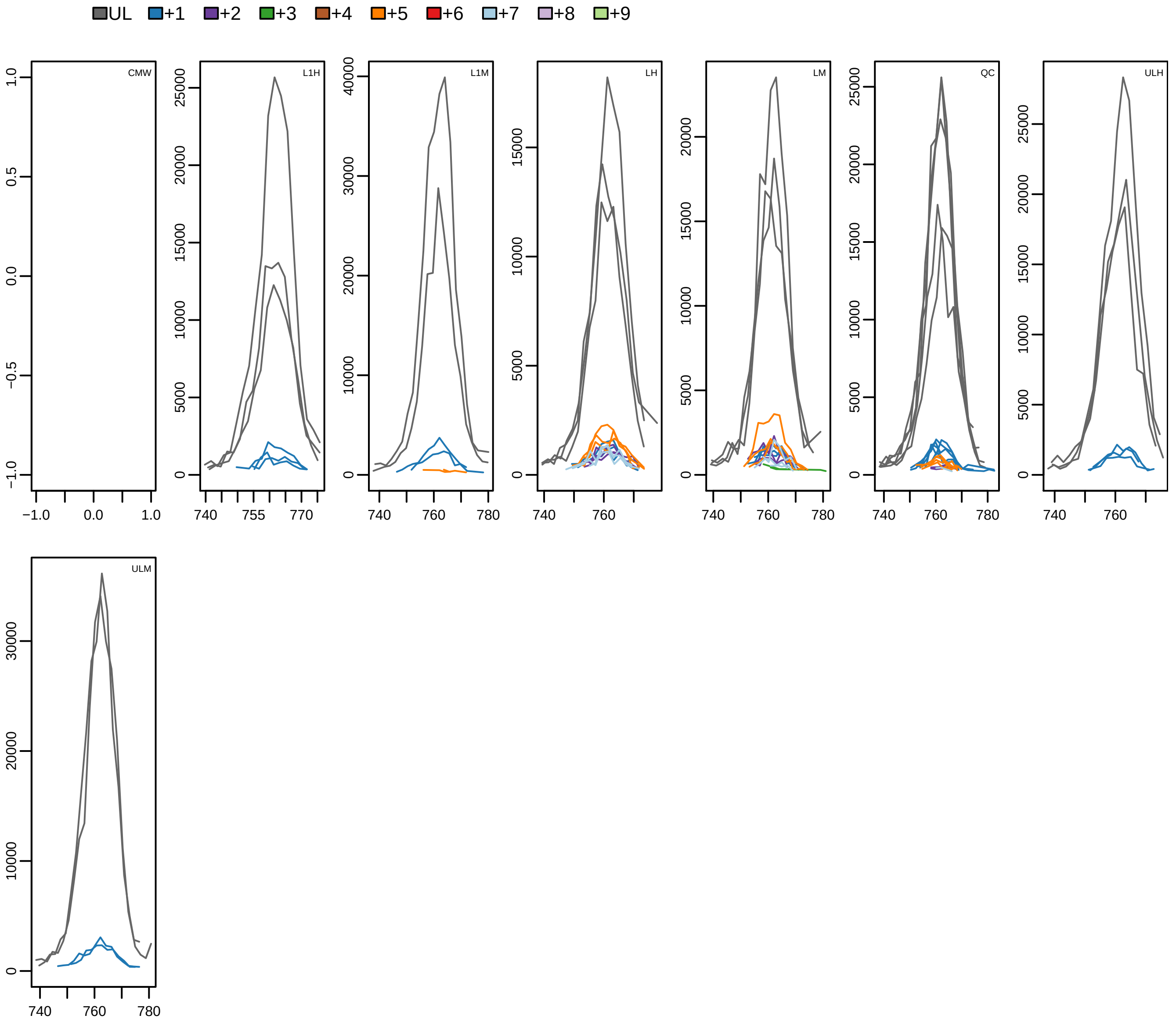

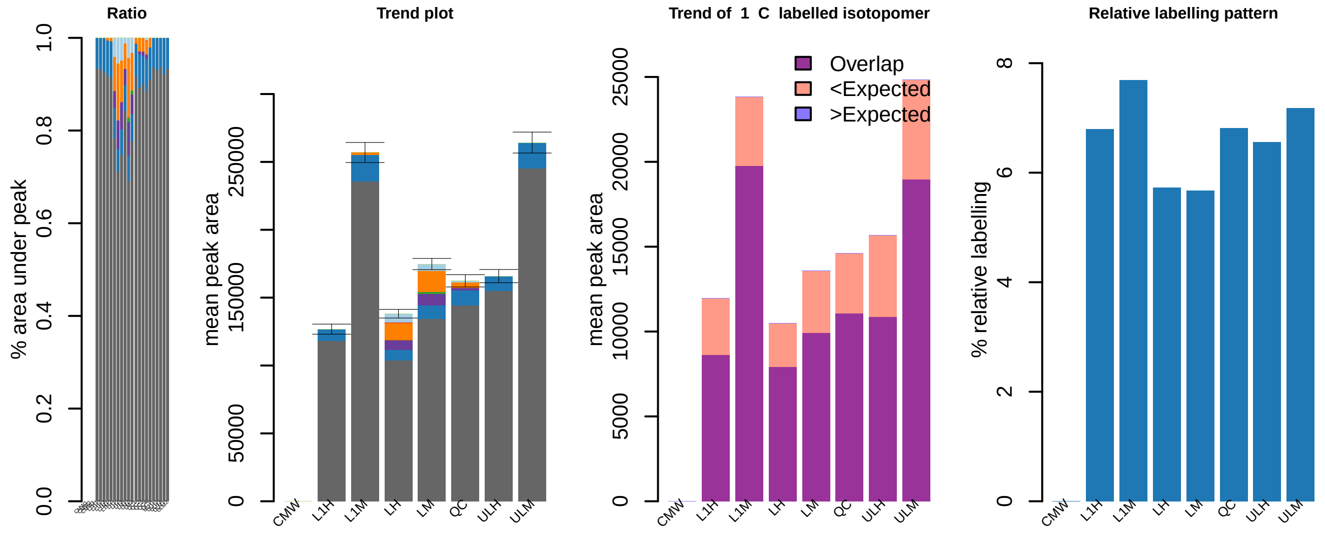

Cytosine

Formula: C4H5N3O Mass: 111.043 Std.RT: 712.0172526 Ion: POS

G1

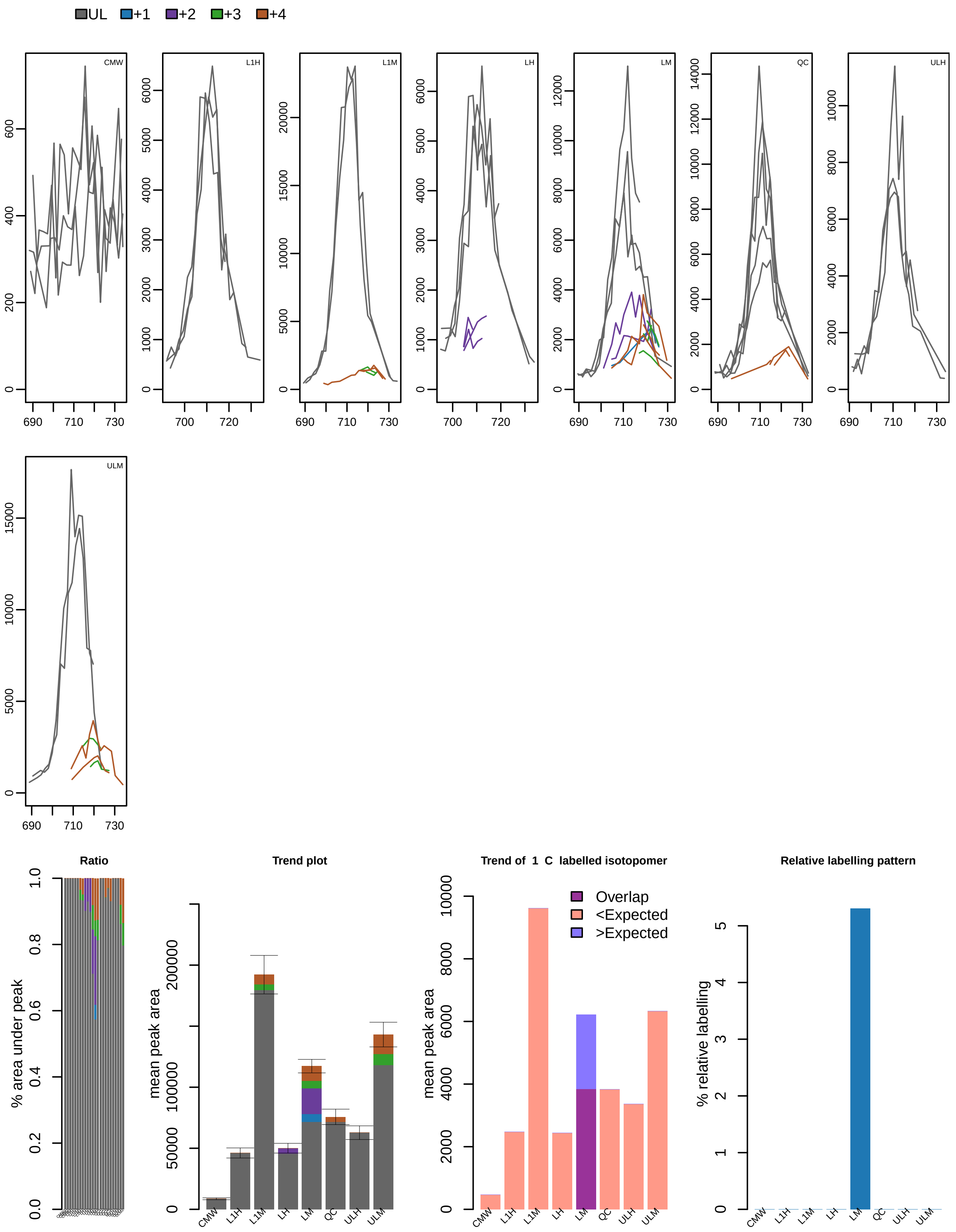

Deoxycytidine

Formula: C9H13N3O4 Mass: 227.091 Std.RT: 688.877139 Ion: POS

G1

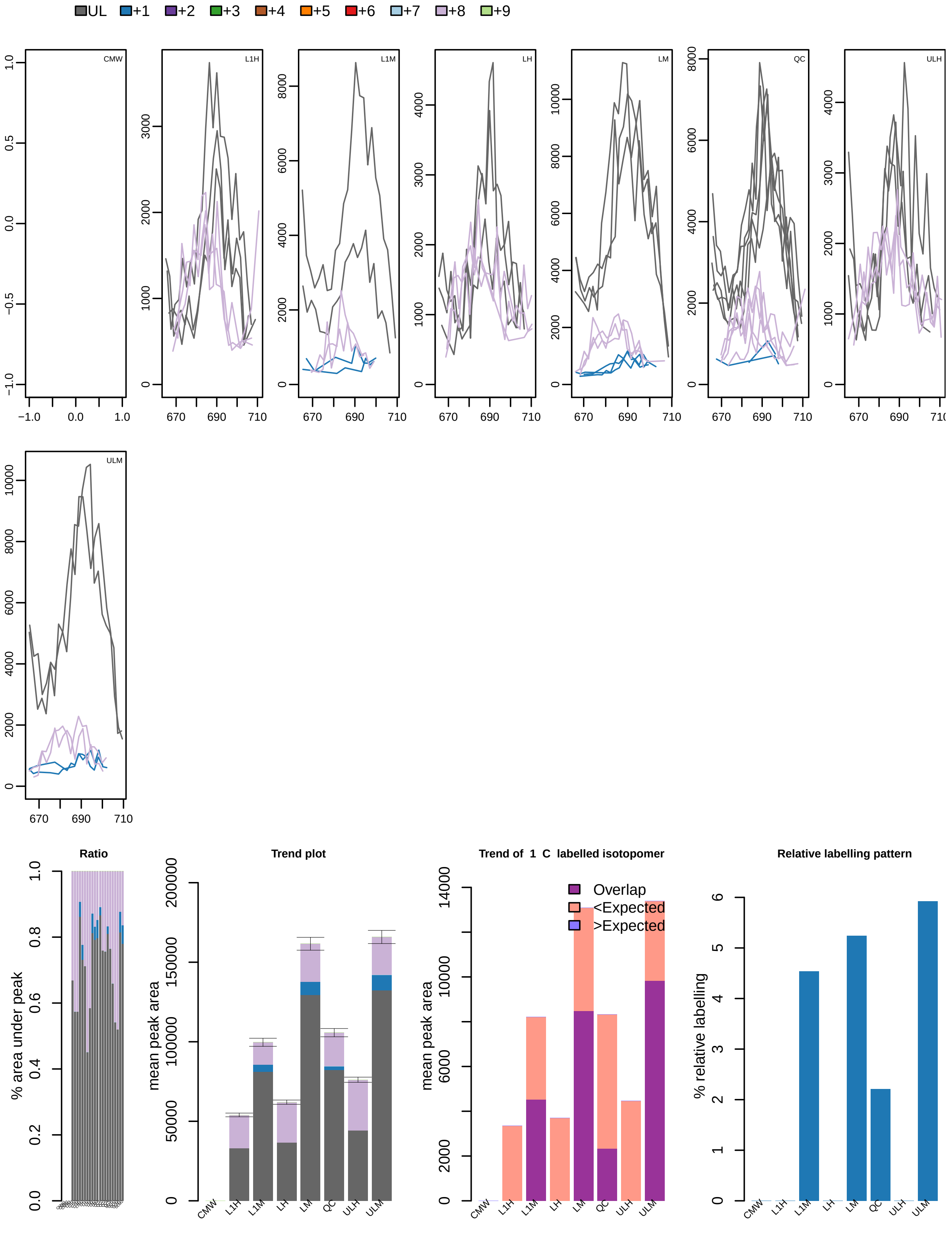

Gamma−Glutamylglutamine

Formula: $C_{10}H_{17}N_3O_6$ Mass: 275.112 Std.RT: 991.192572 Ion: POS

G1

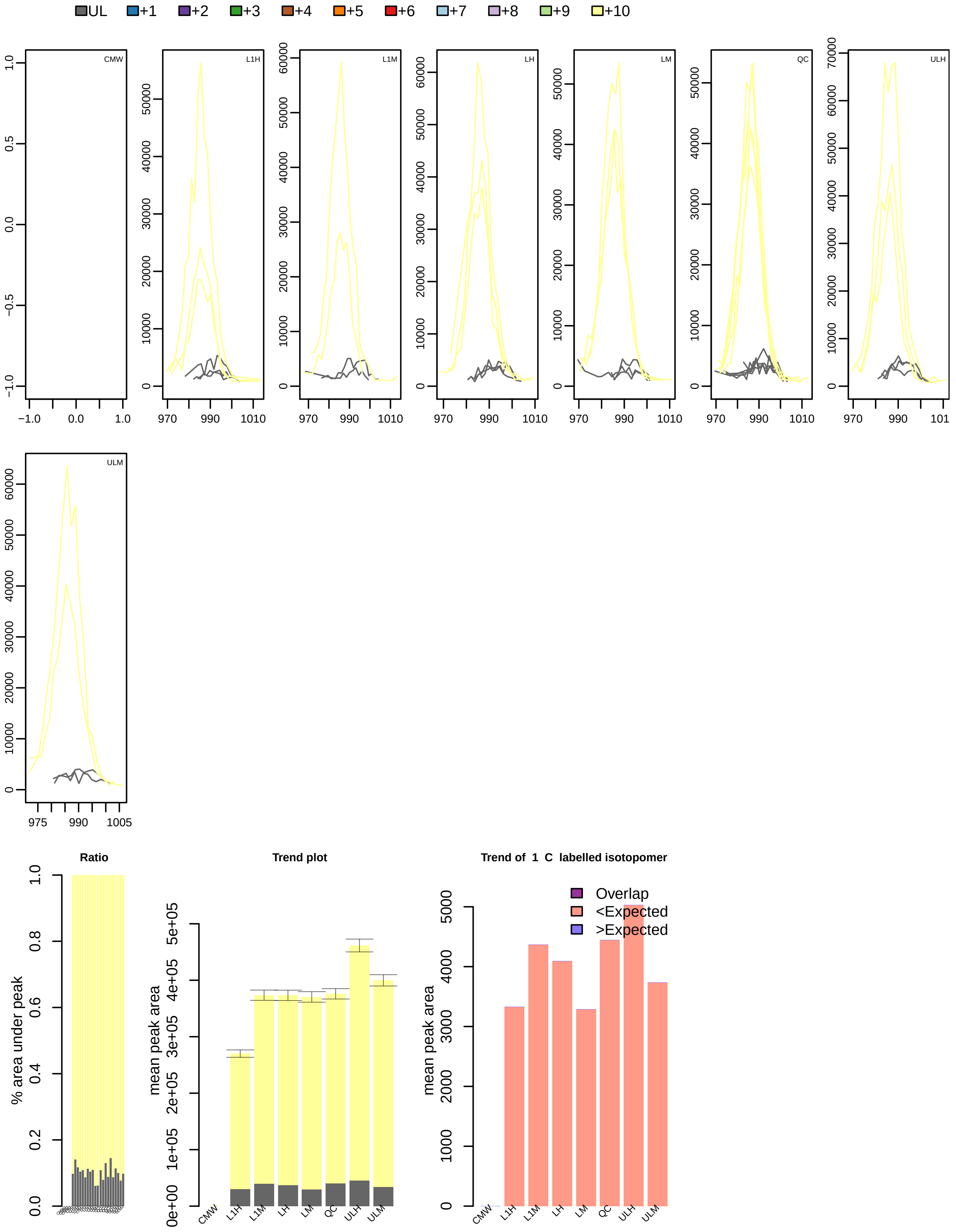

GammaGlutamylglutamicacid

Formula: C10H16N2O7 Mass: 276.096 Std.RT: 1084.2300042 Ion: P

G1

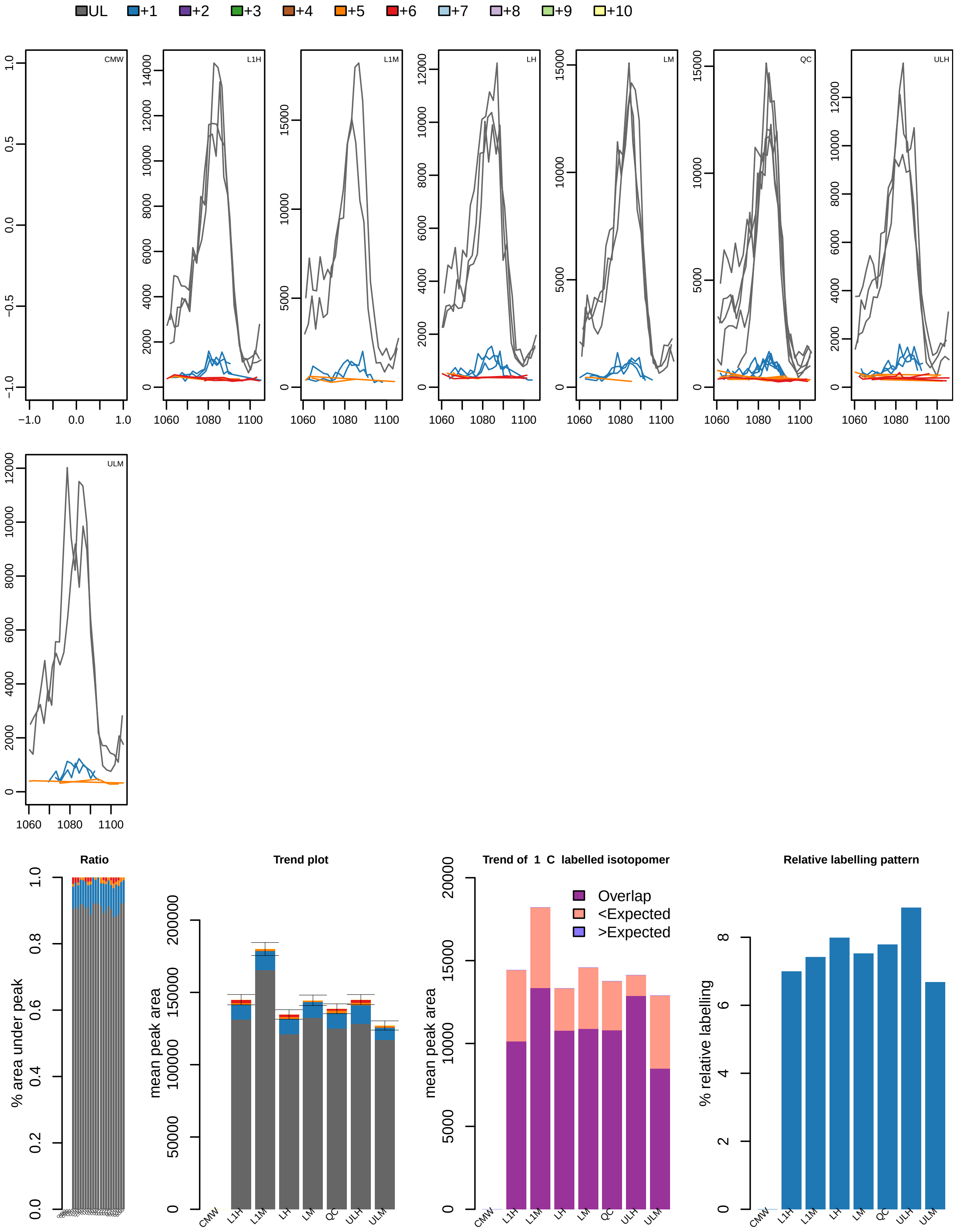

Thr−His

Formula: C10H16N4O4 Mass: 256.117 Std.RT: 722.6022864 Ion: PC

G1

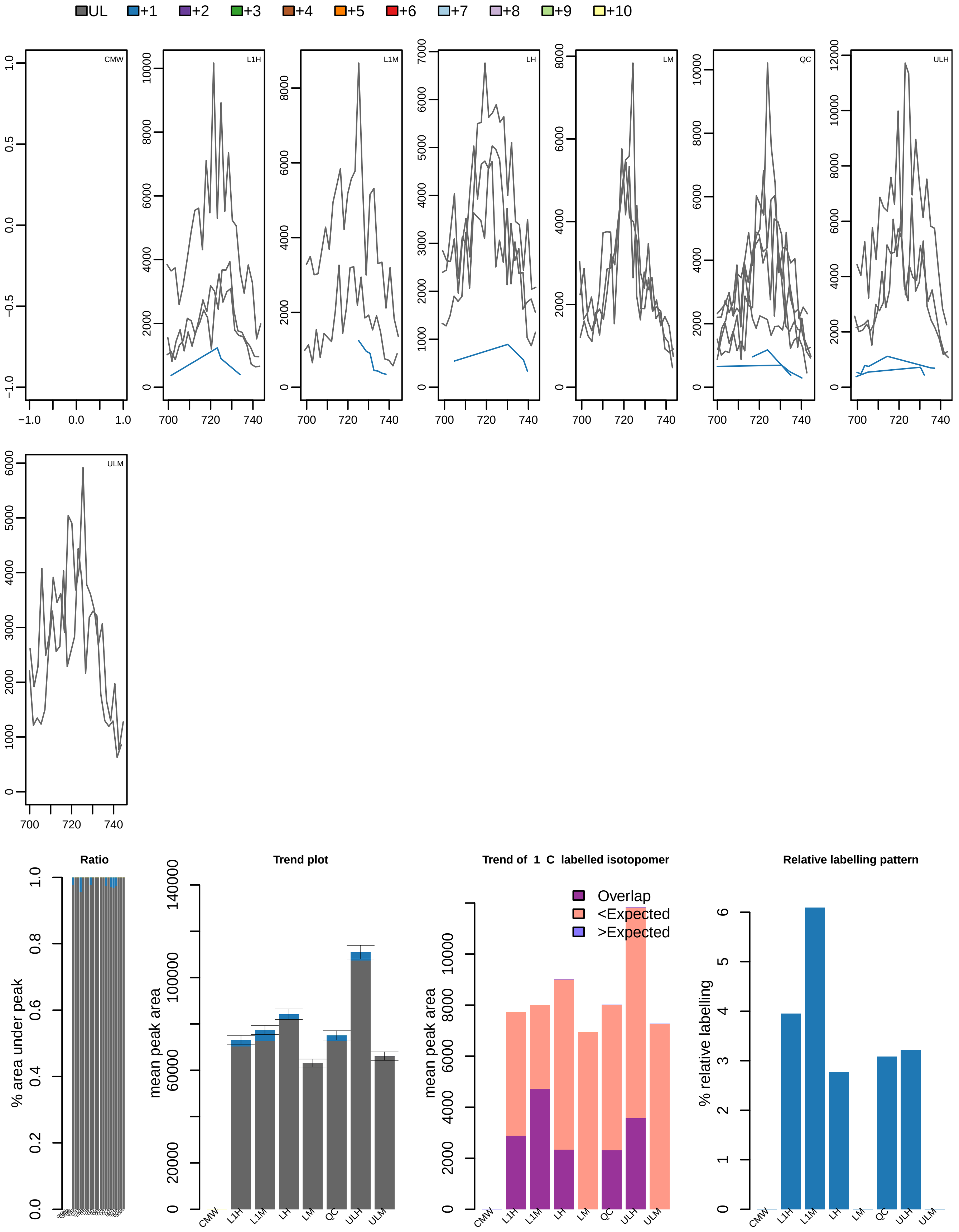

Phe−Arg

Formula: $C_{15}H_{23}N_5O_3$ Mass: 321.18 Std.RT: 764.278005 Ion: POS

G1

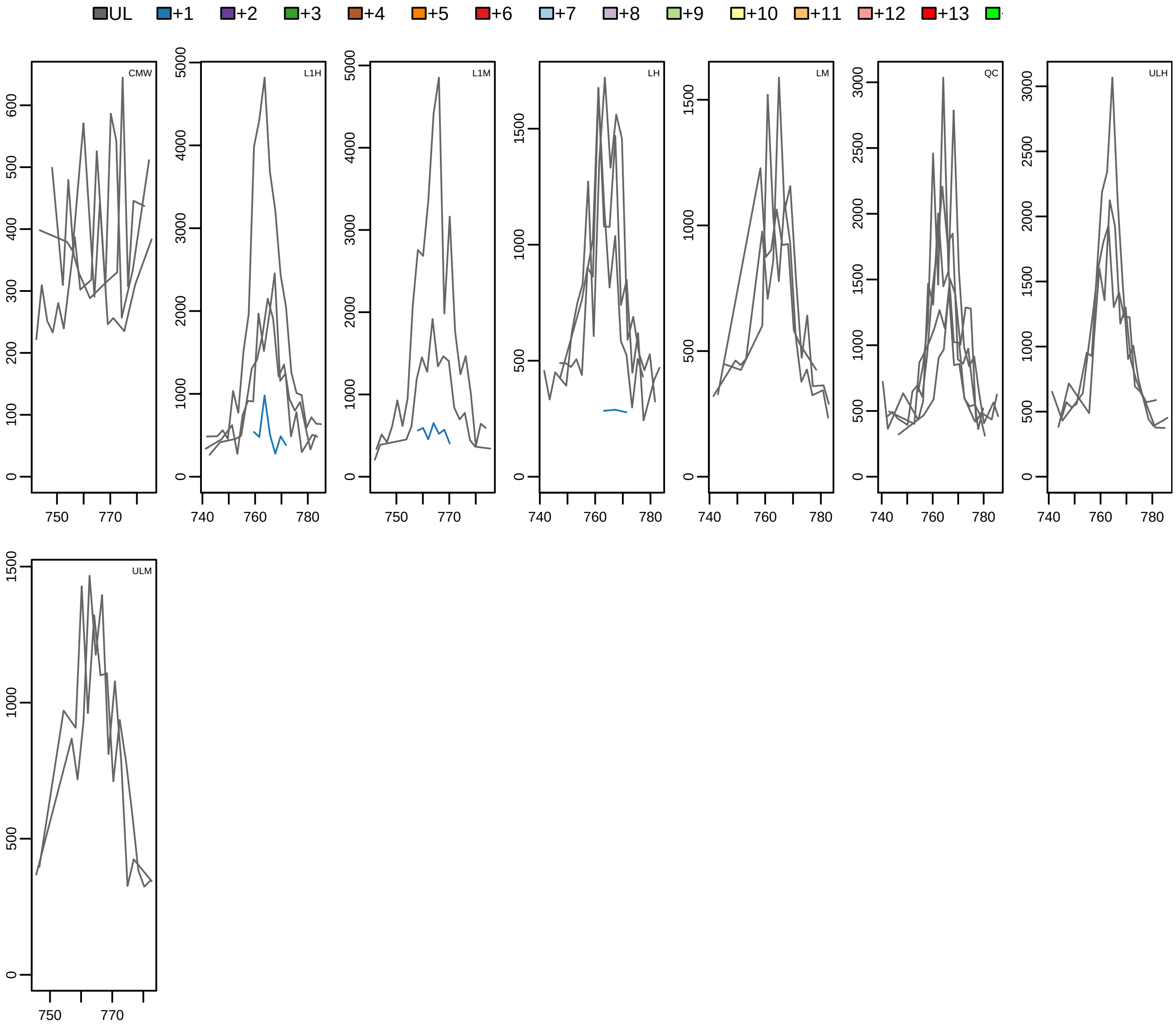

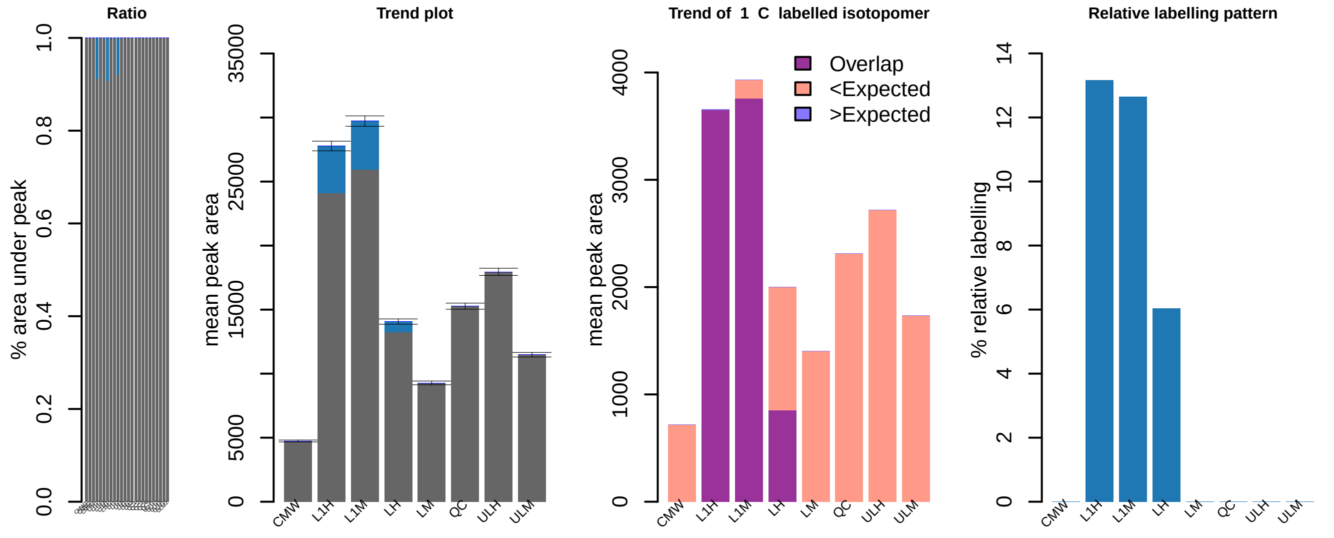

Leu−Lys

Formula: C12H25N3O3 Mass: 259.19 Std.RT: 821.0471424 Ion: POS

G1

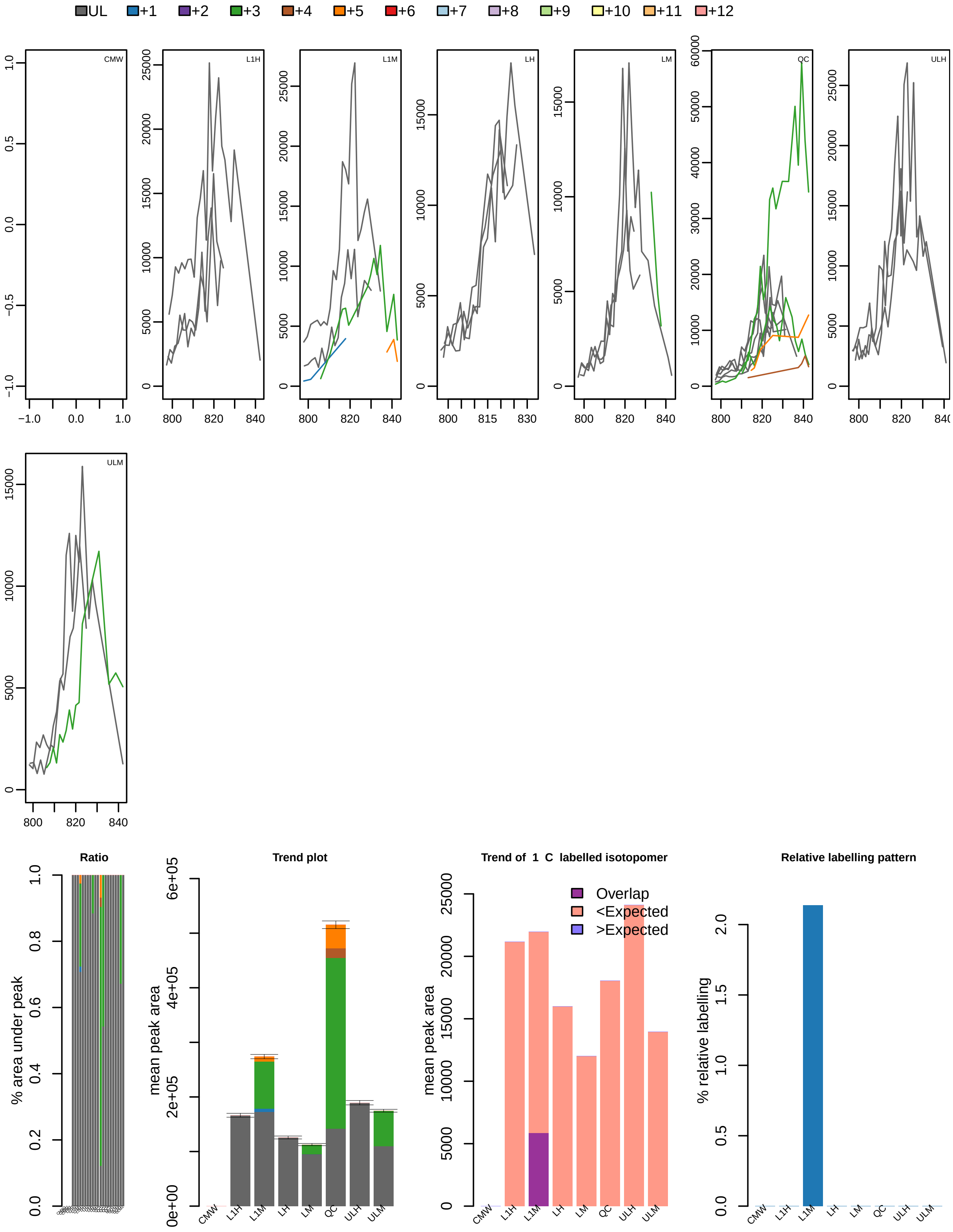

Glu−Arg

Formula: C11H21N5O5 Mass: 303.154 Std.RT: 988.2062784 Ion: PC

G1

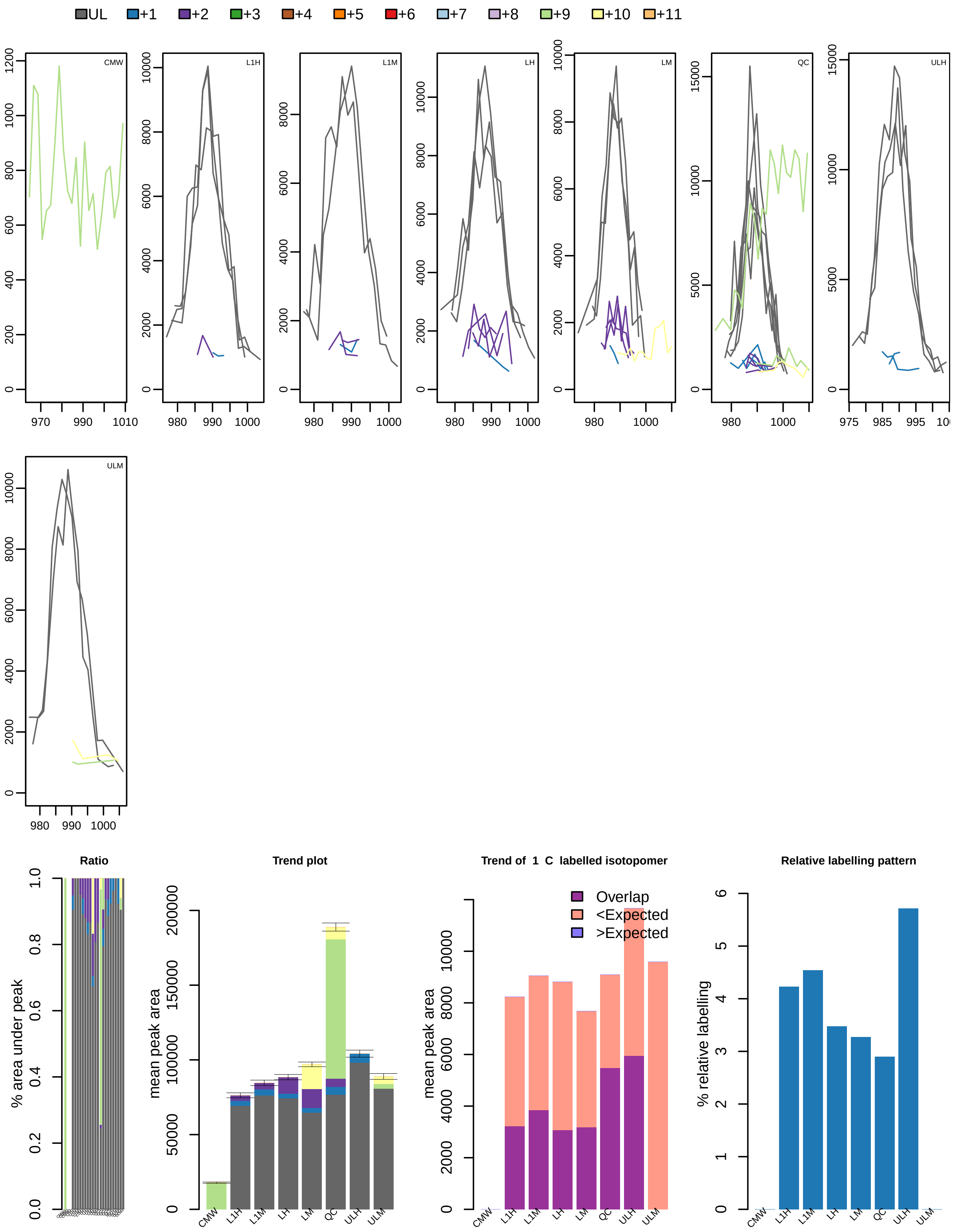

Thr−Arg

G1

Formula: C10H21N5O4 Mass: 275.159 Std.RT: 1020.5909598 Ion: P

UL +1 +2 +3 +4 +5 +6 +7 +8 +9 +10

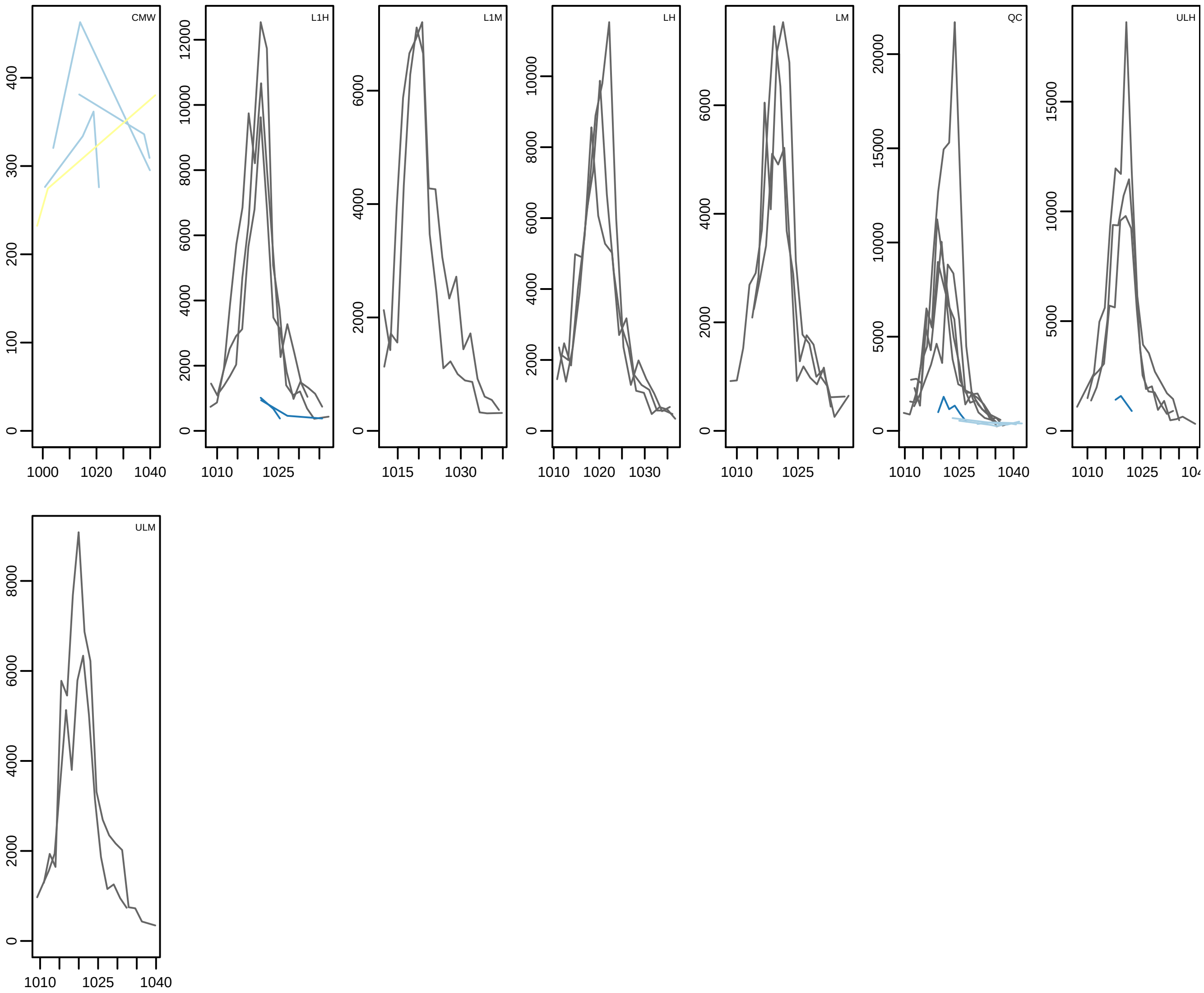

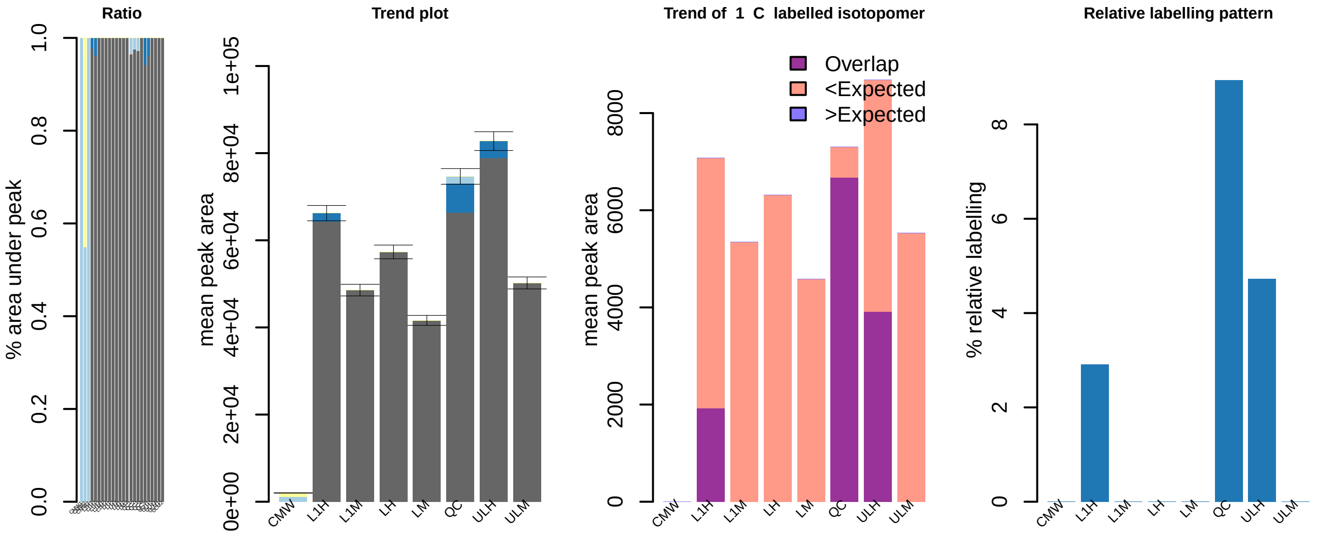

Lys−Val

Formula: C11H23N3O3 Mass: 245.174 Std.RT: 896.284863 Ion: POS

G1

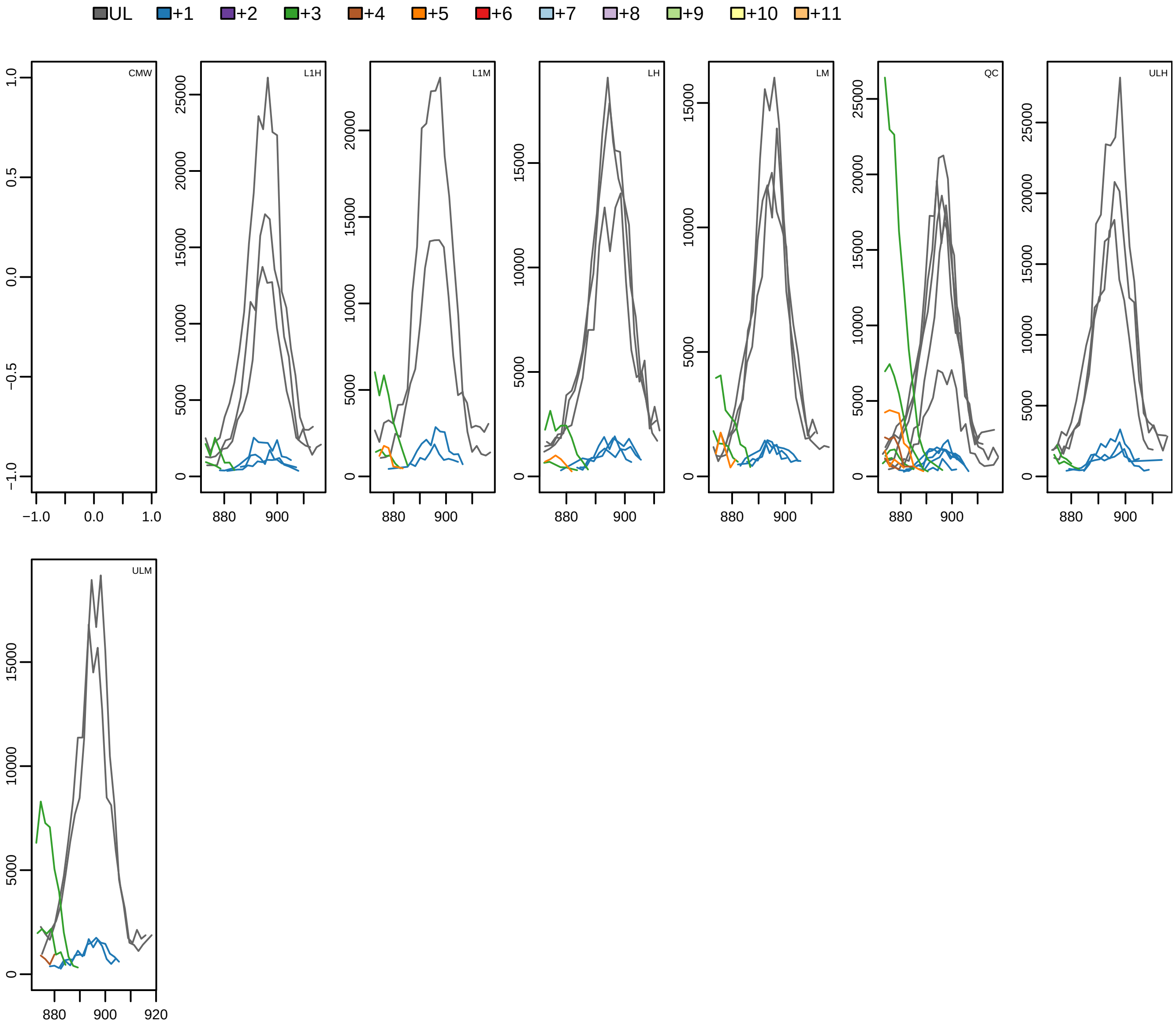

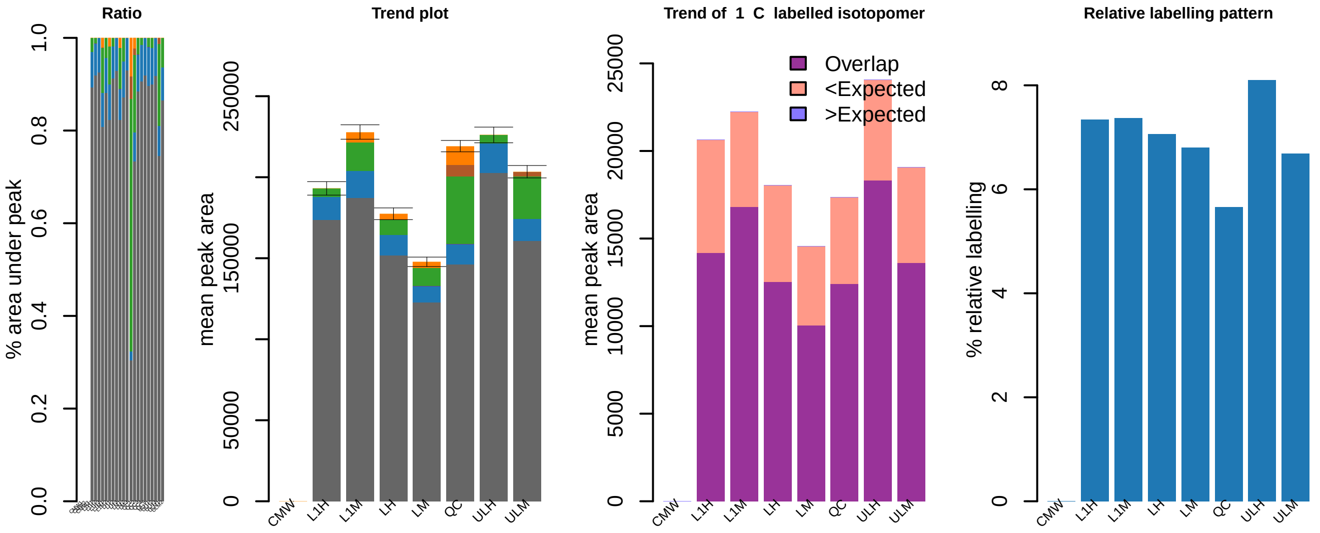

L−a−glutamyl−L−Lysine

Formula: C11H21N3O5 Mass: 275.148 Std.RT: 1003.2421002 Ion: P

G1

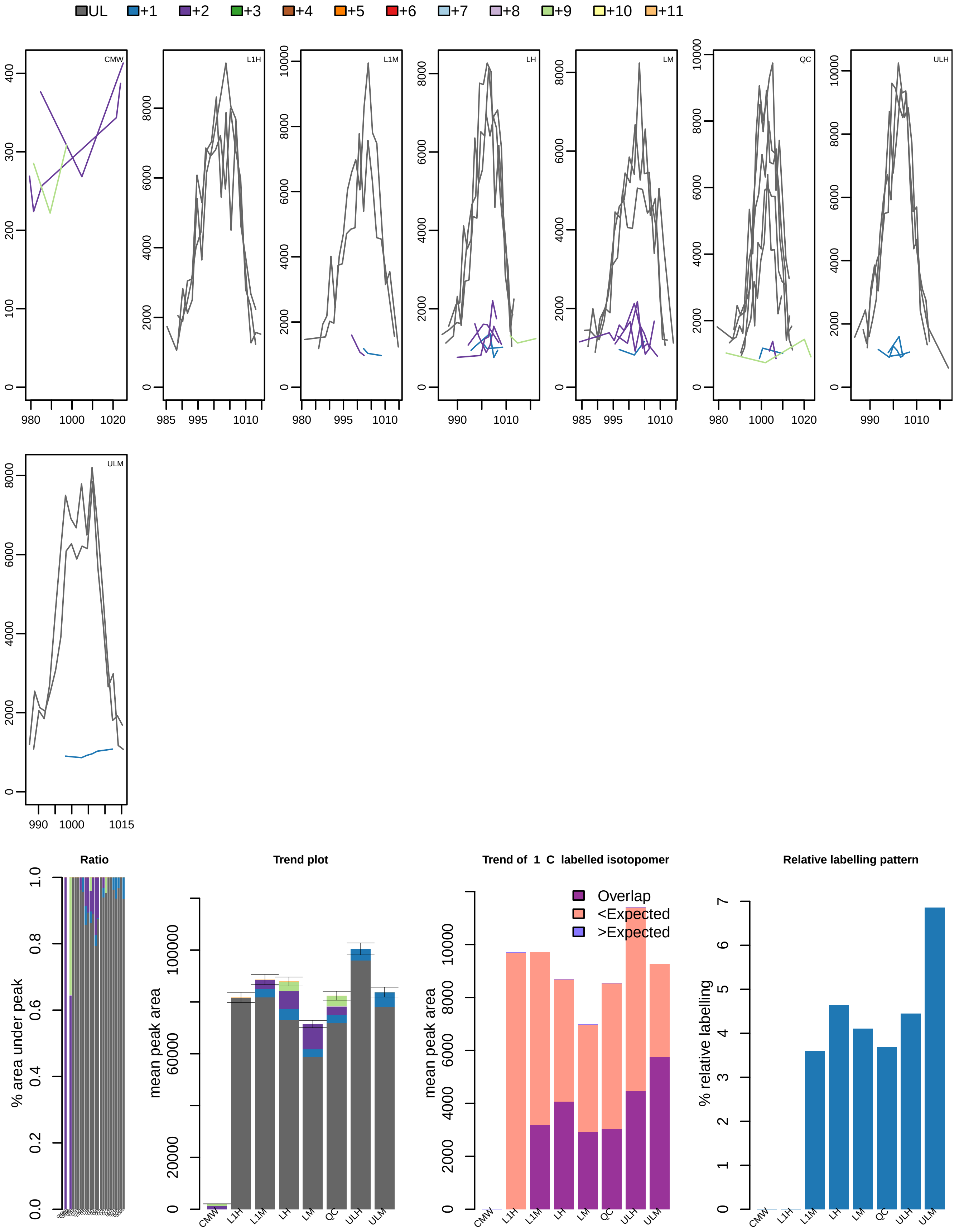

Ala−His

Formula: C9H14N4O3 Mass: 226.107 Std.RT: 1015.950474 Ion: POS

G1

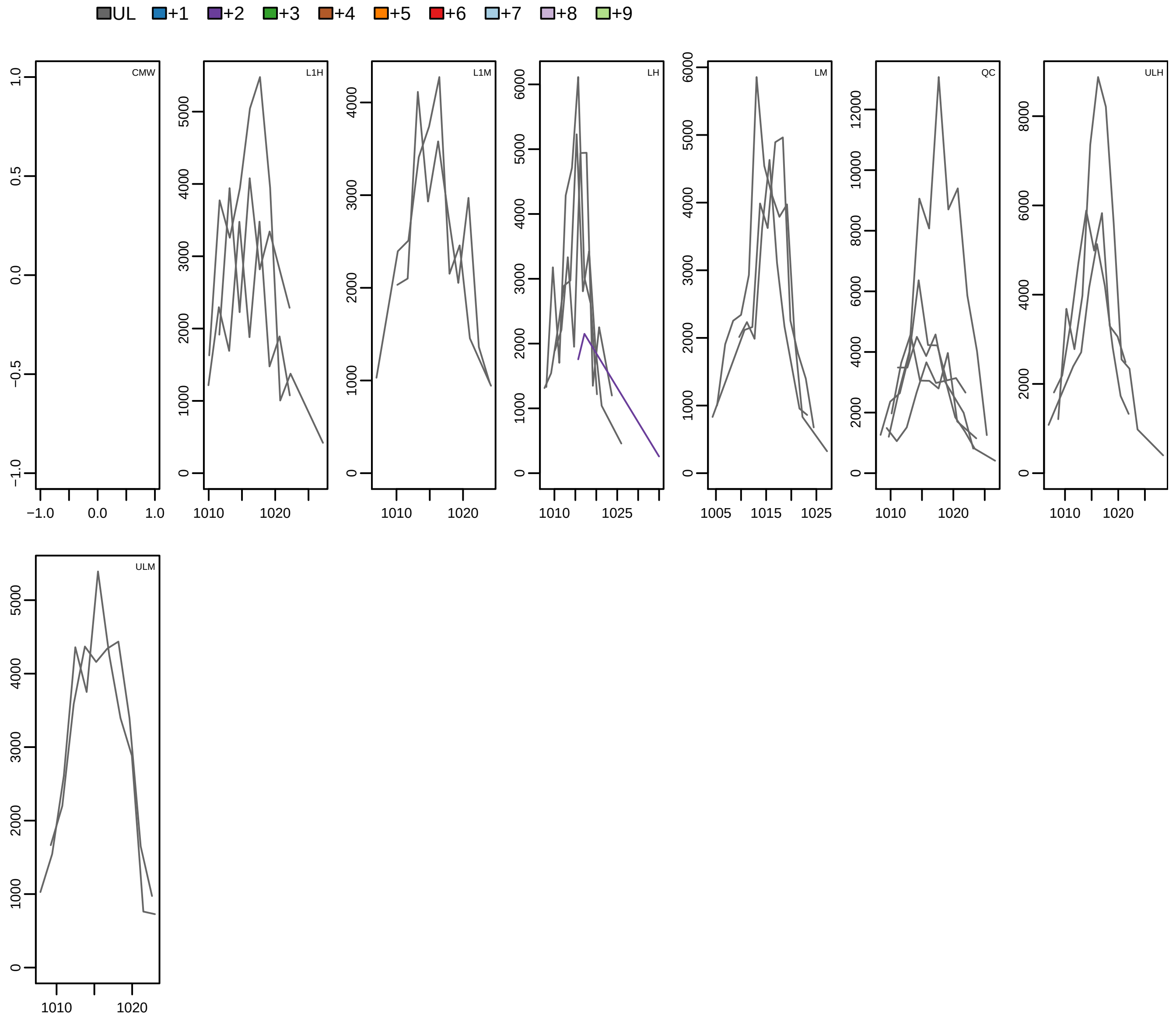

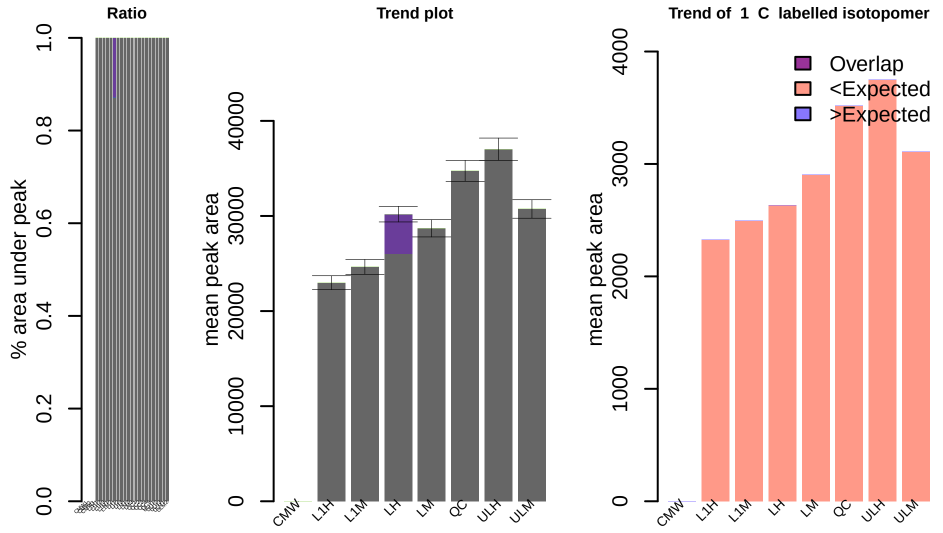

Lys−Gly

Formula: C8H17N3O3 Mass: 203.127 Std.RT: 783.0025752 Ion: POS

G1

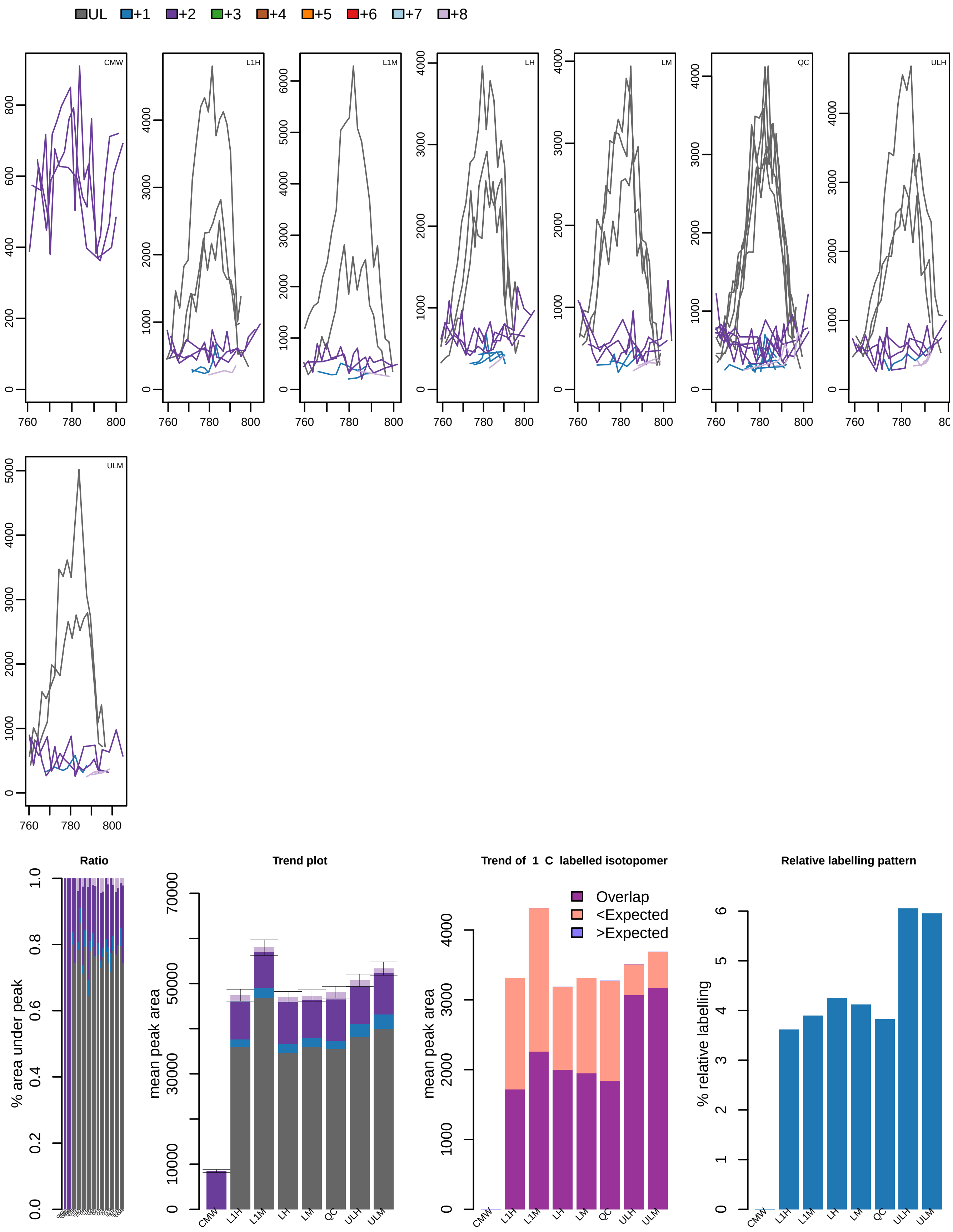

Ser−His

Formula: C9H14N4O4 Mass: 242.102 Std.RT: 796.751427 Ion: POS

G1

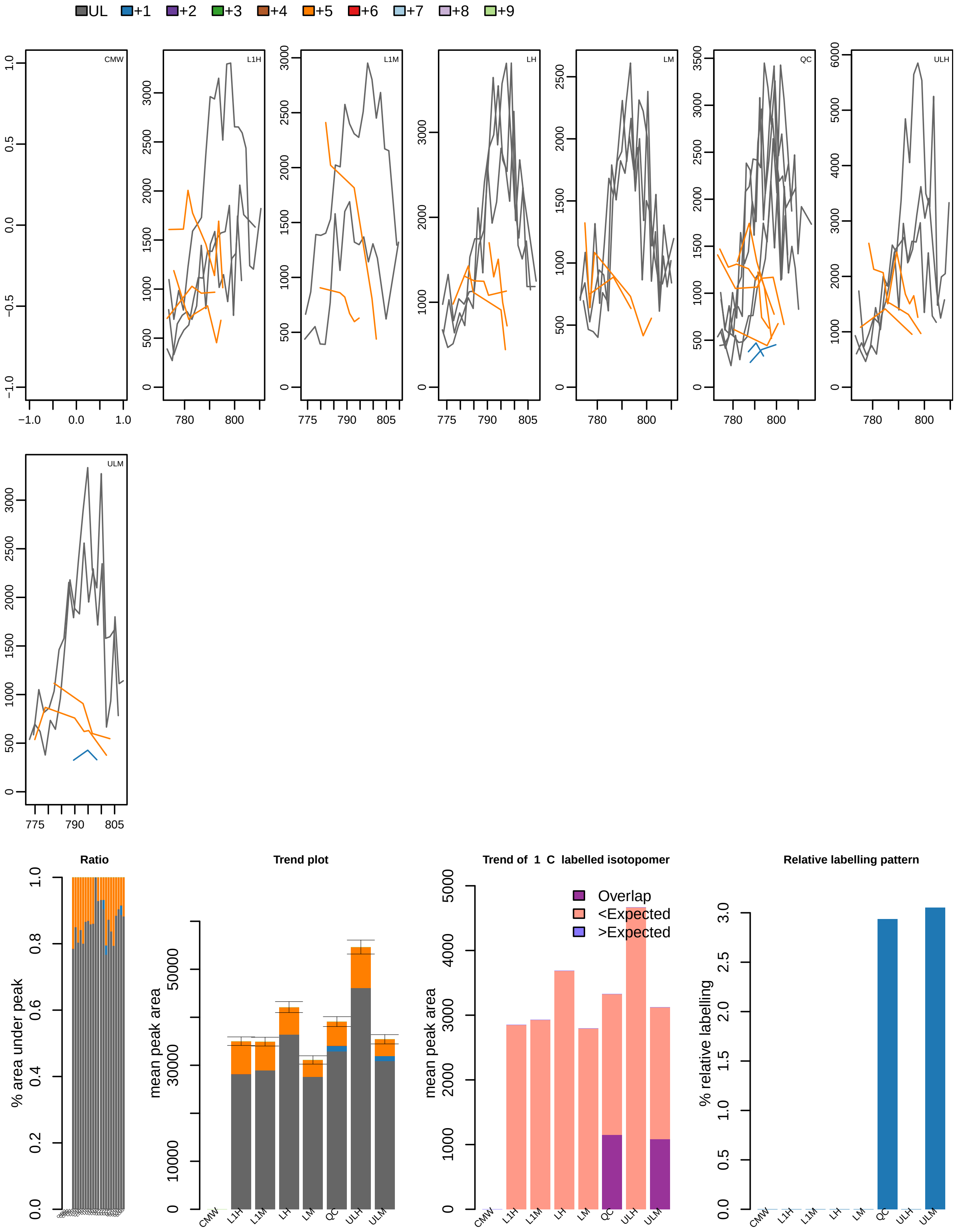

Val−Arg

Formula: C11H23N5O3 Mass: 273.18 Std.RT: 885.6794262 Ion: POS

G1

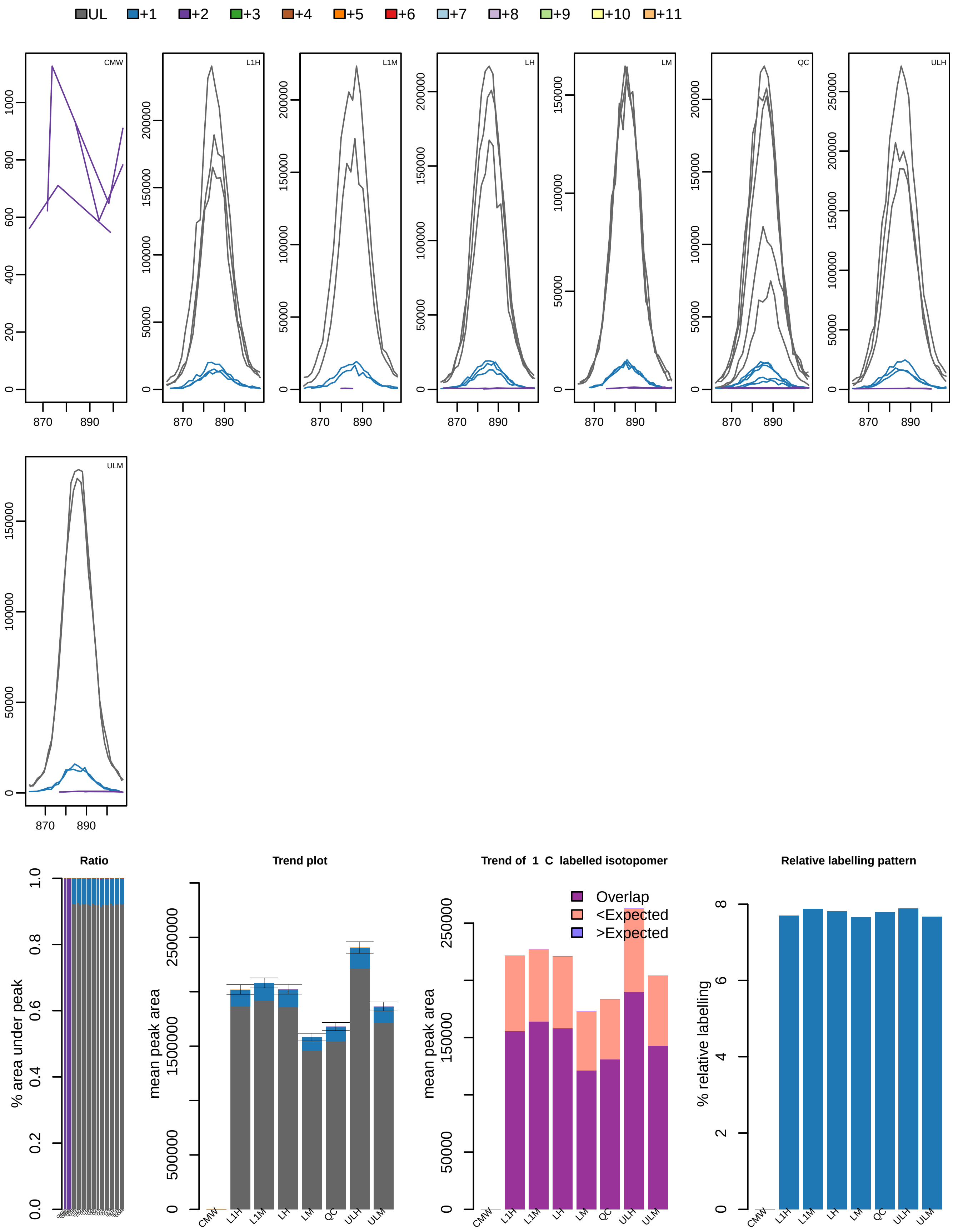

Asp−Arg

Formula: C10H19N5O5 Mass: 289.139 Std.RT: 1178.9242818 Ion: P

G1

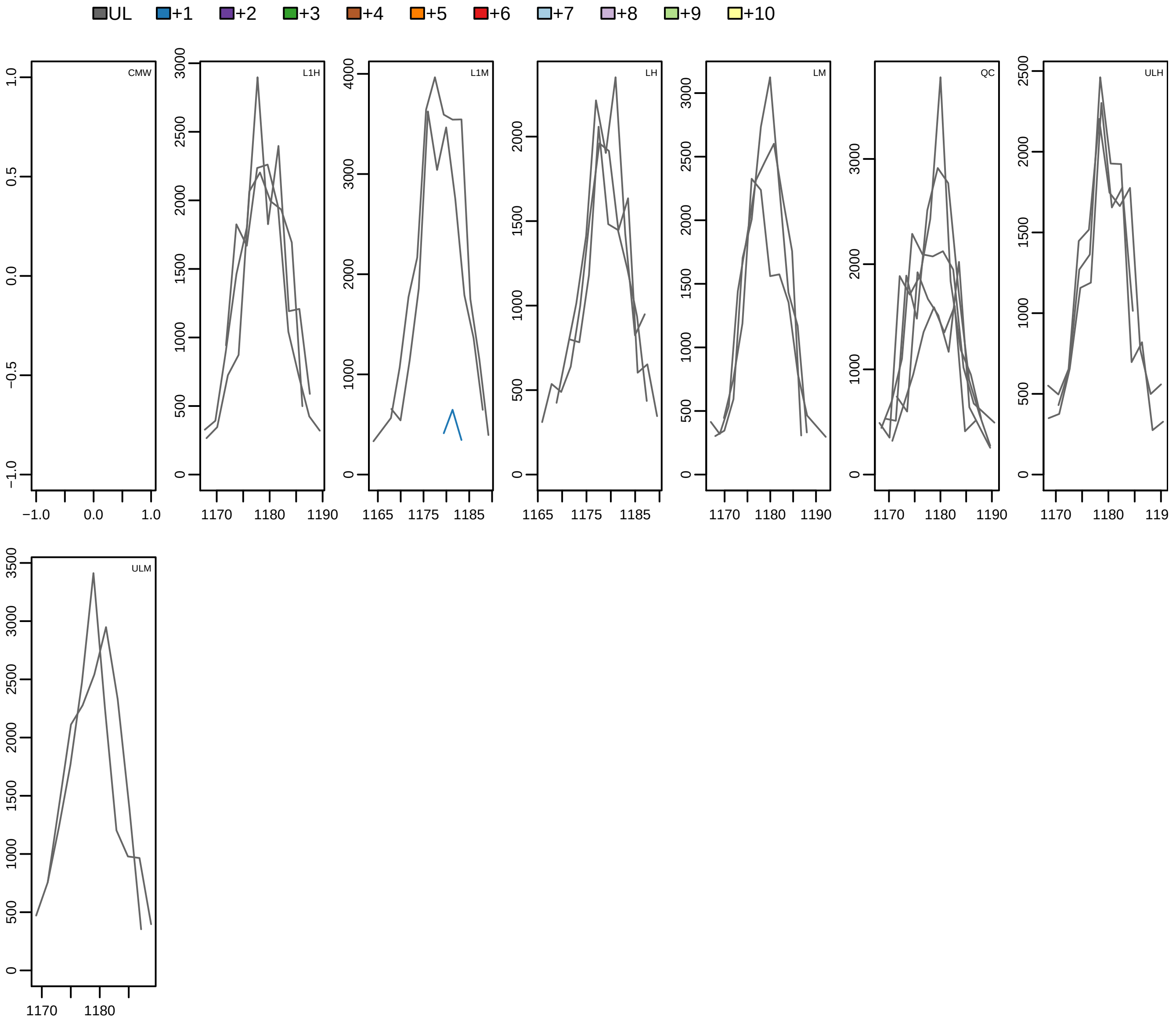

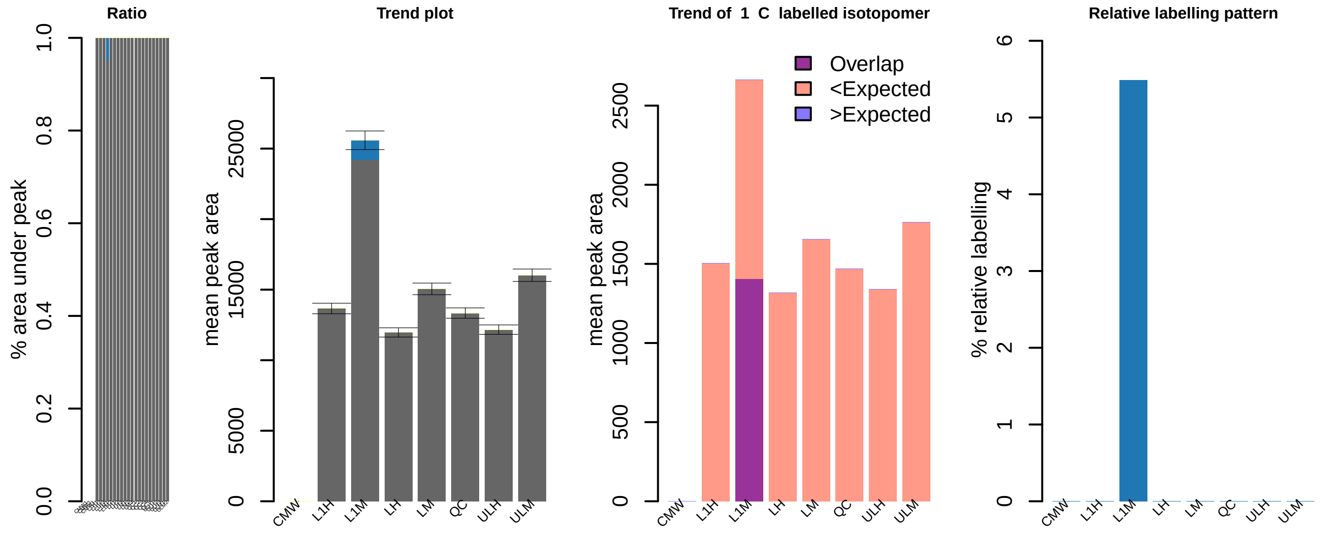

Leu−Arg

Formula: C12H25N5O3 Mass: 287.196 Std.RT: 814.2762828 Ion: PC

G1

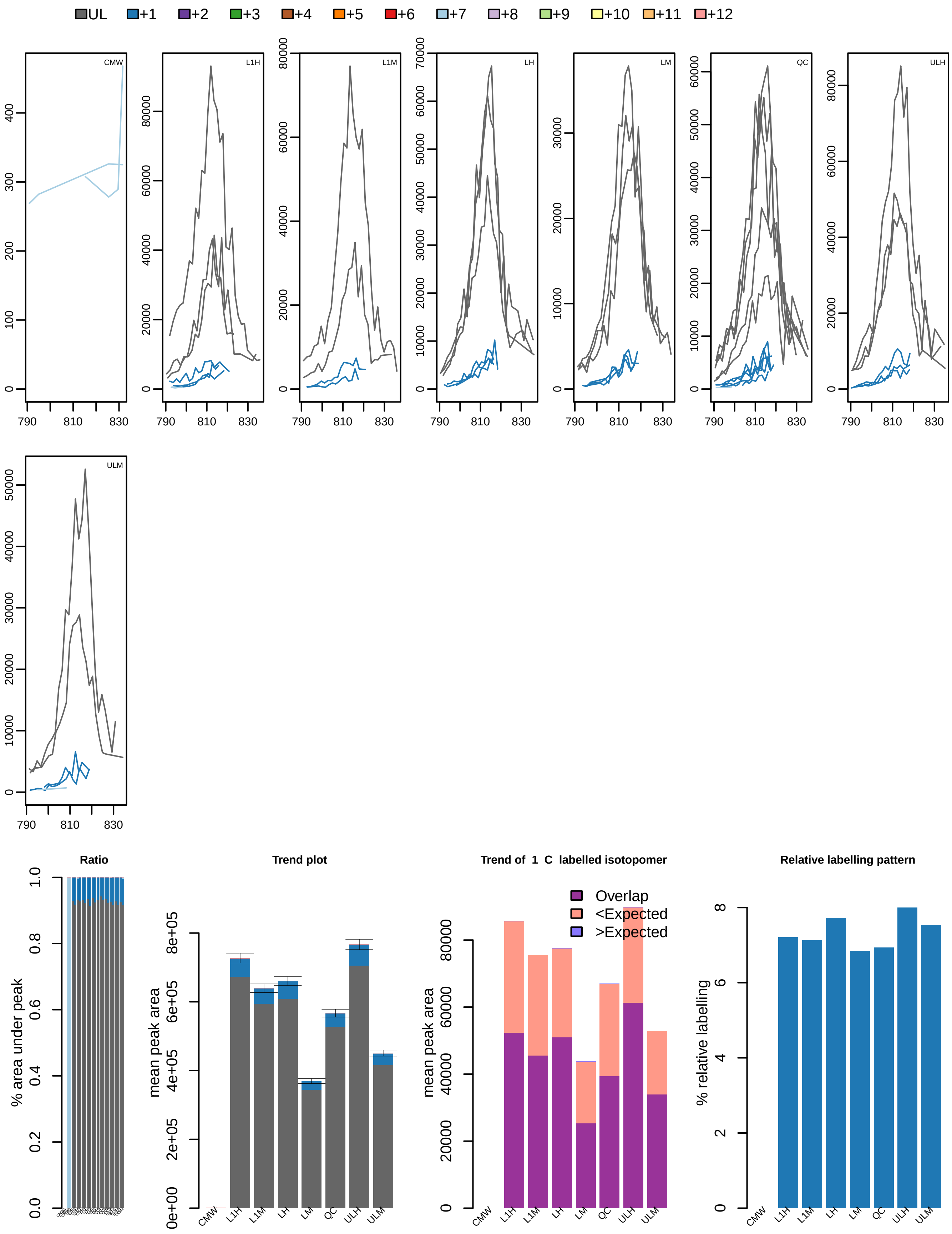

Ile−Ala

Formula: C9H18N2O3 Mass: 202.132 Std.RT: 836.995563 Ion: POS

G1

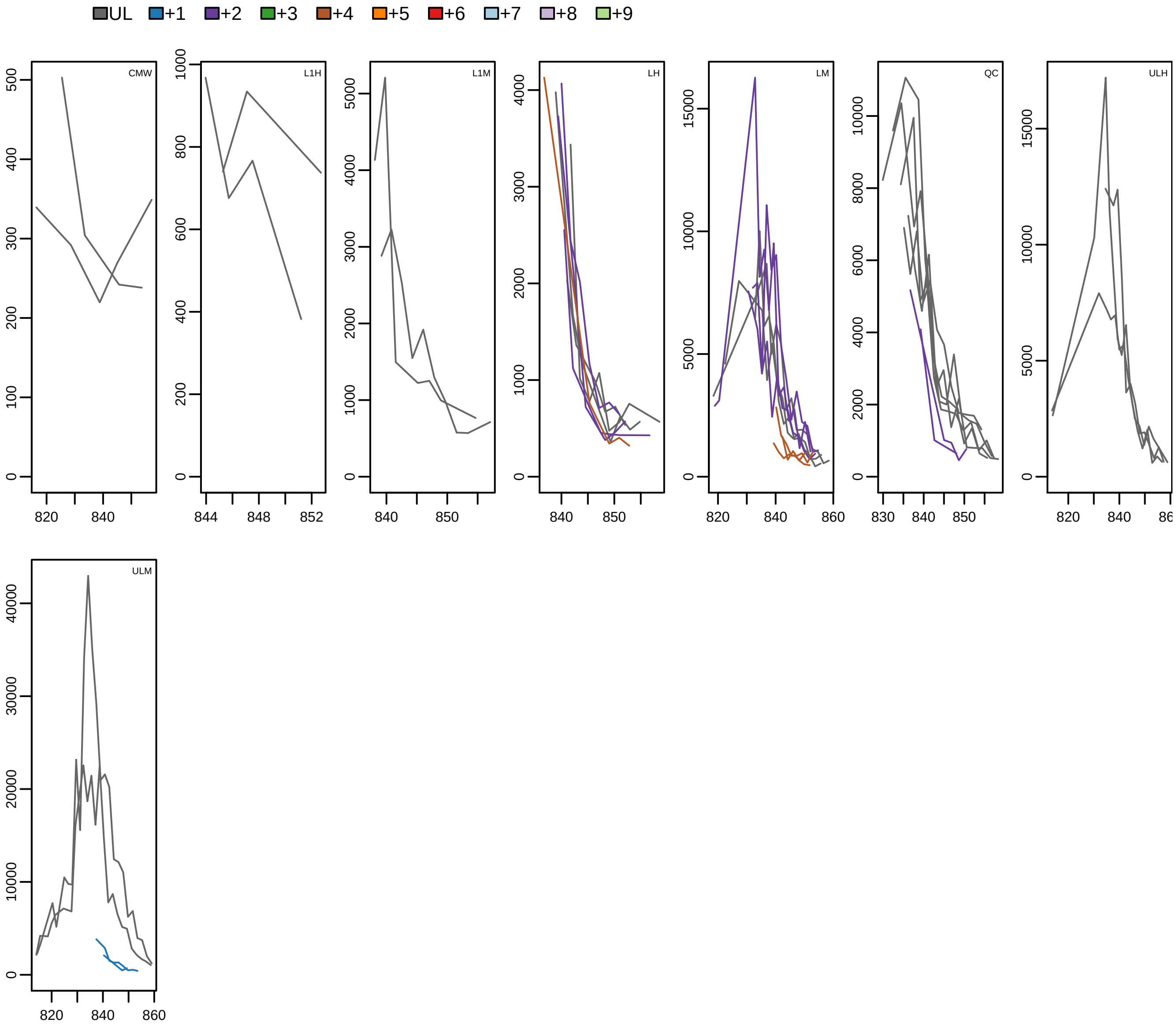

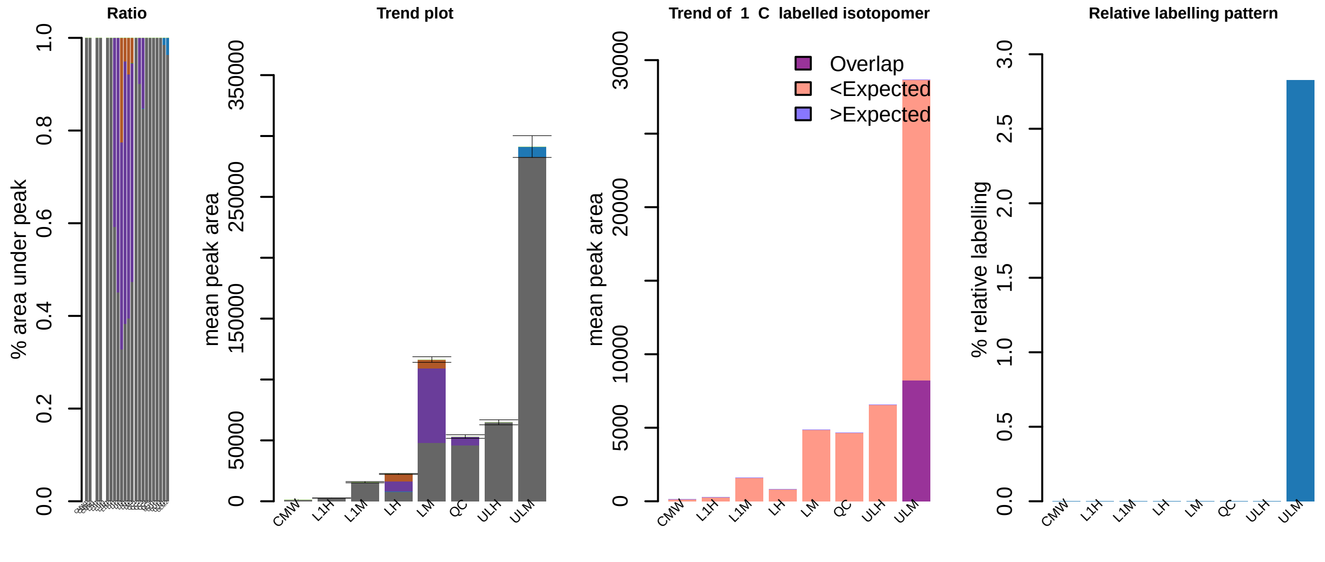

Val−Val

Formula: C10H20N2O3 Mass: 216.147 Std.RT: 898.2317994 Ion: PC

G1

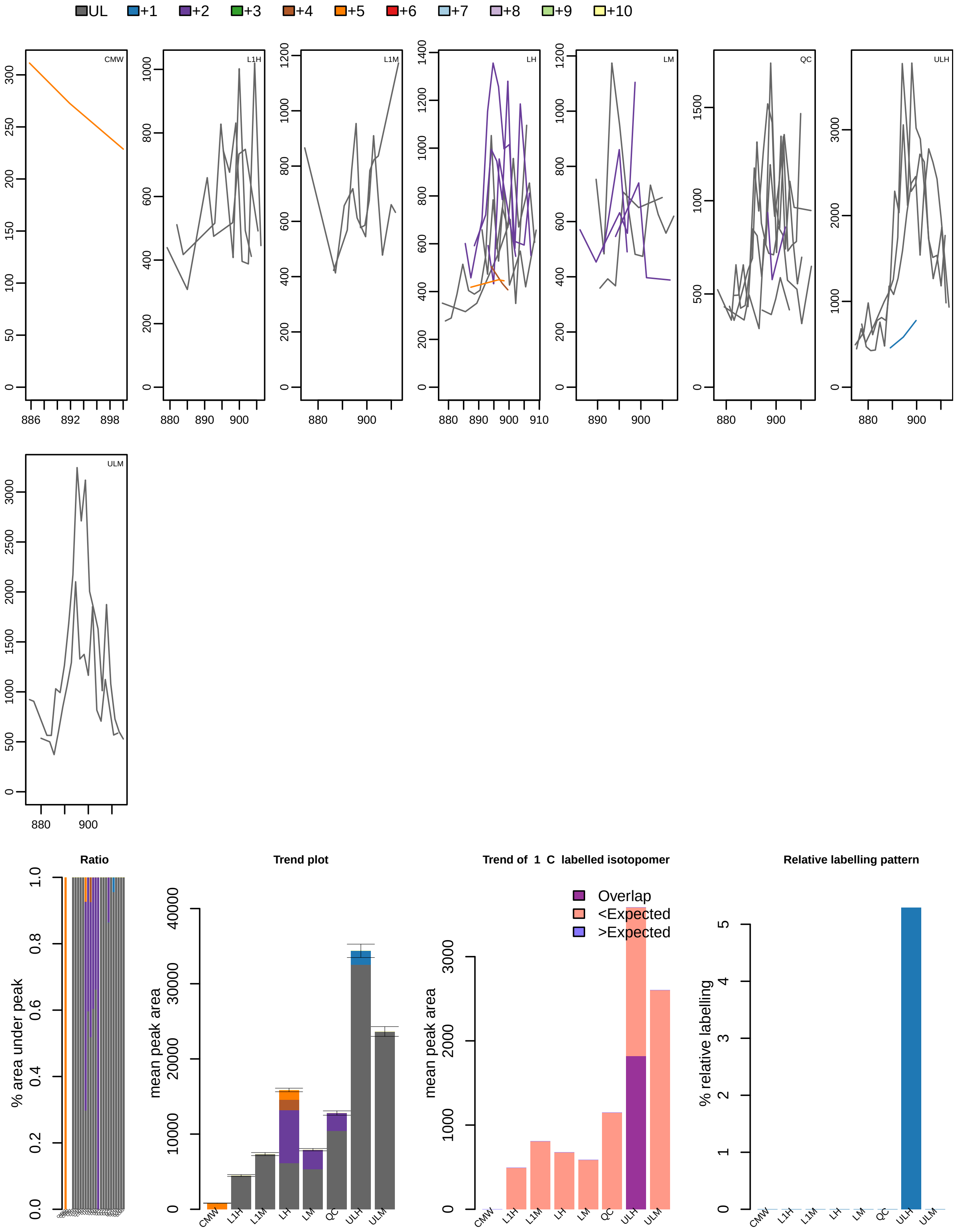

Val−Pro

G1

Formula: C10H18N2O3 Mass: 214.132 Std.RT: 826.8010026 Ion: PC

UL +1 +2 +3 +4 +5 +6 +7 +8 +9 +10

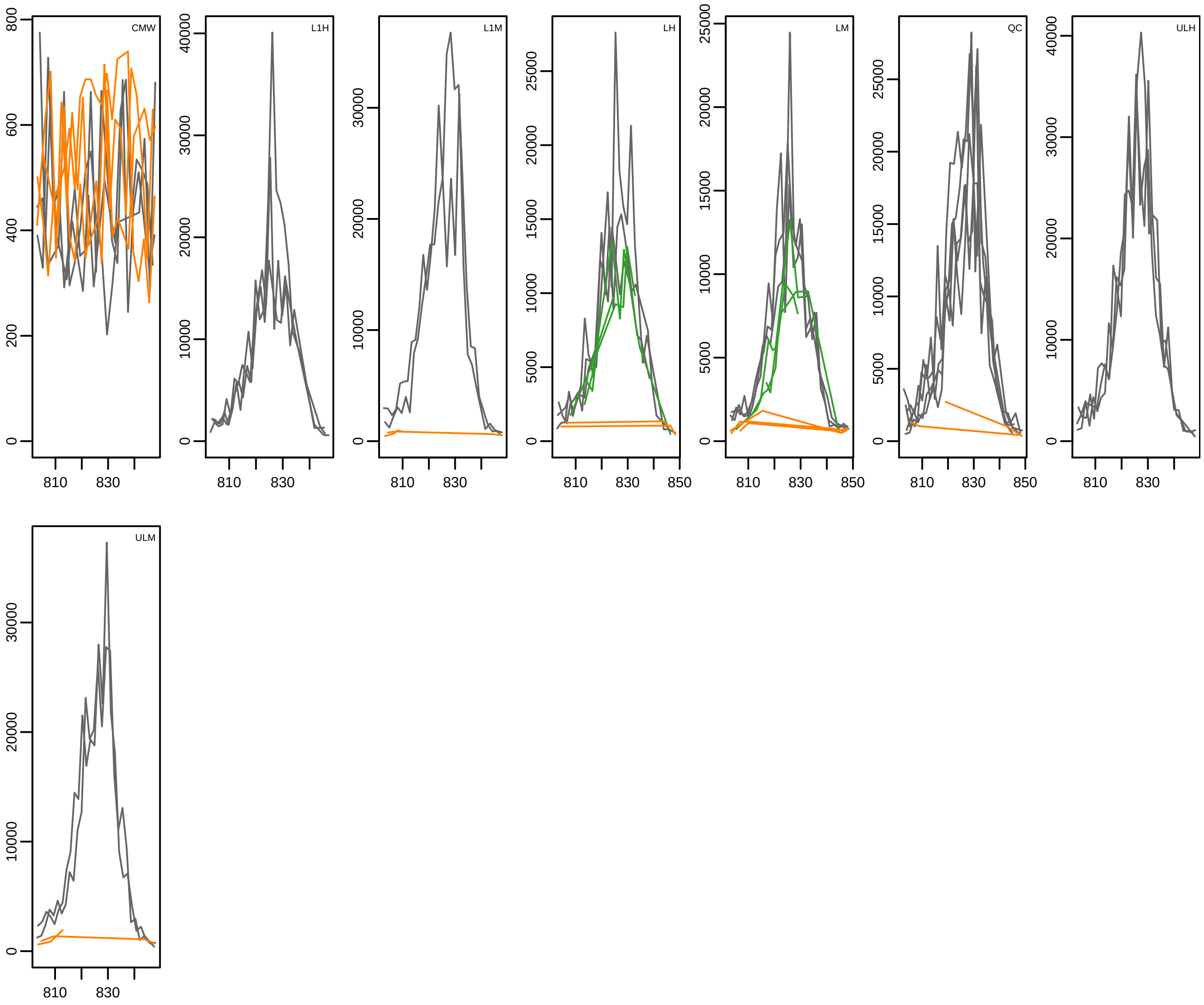

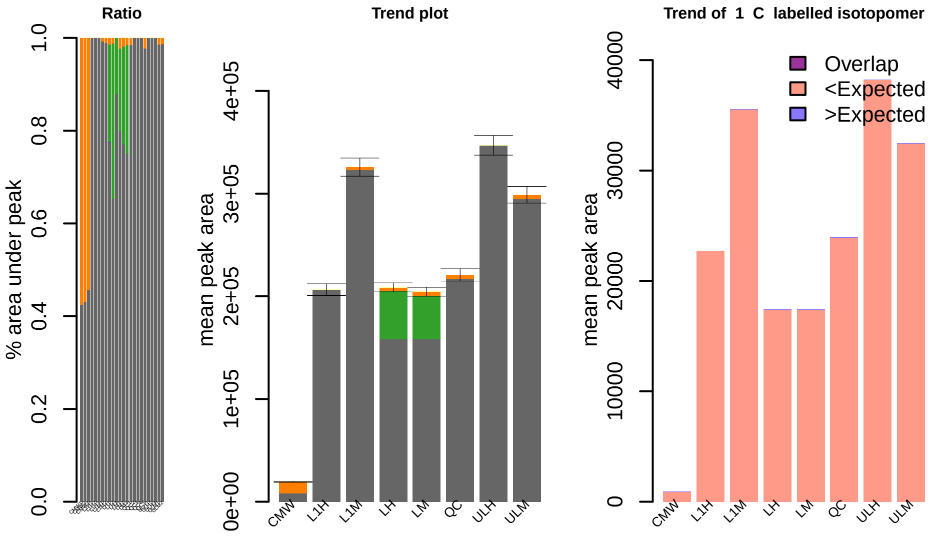

Val−Ser

G1

Formula: C8H16N2O4 Mass: 204.111 Std.RT: 919.7348604 Ion: POS

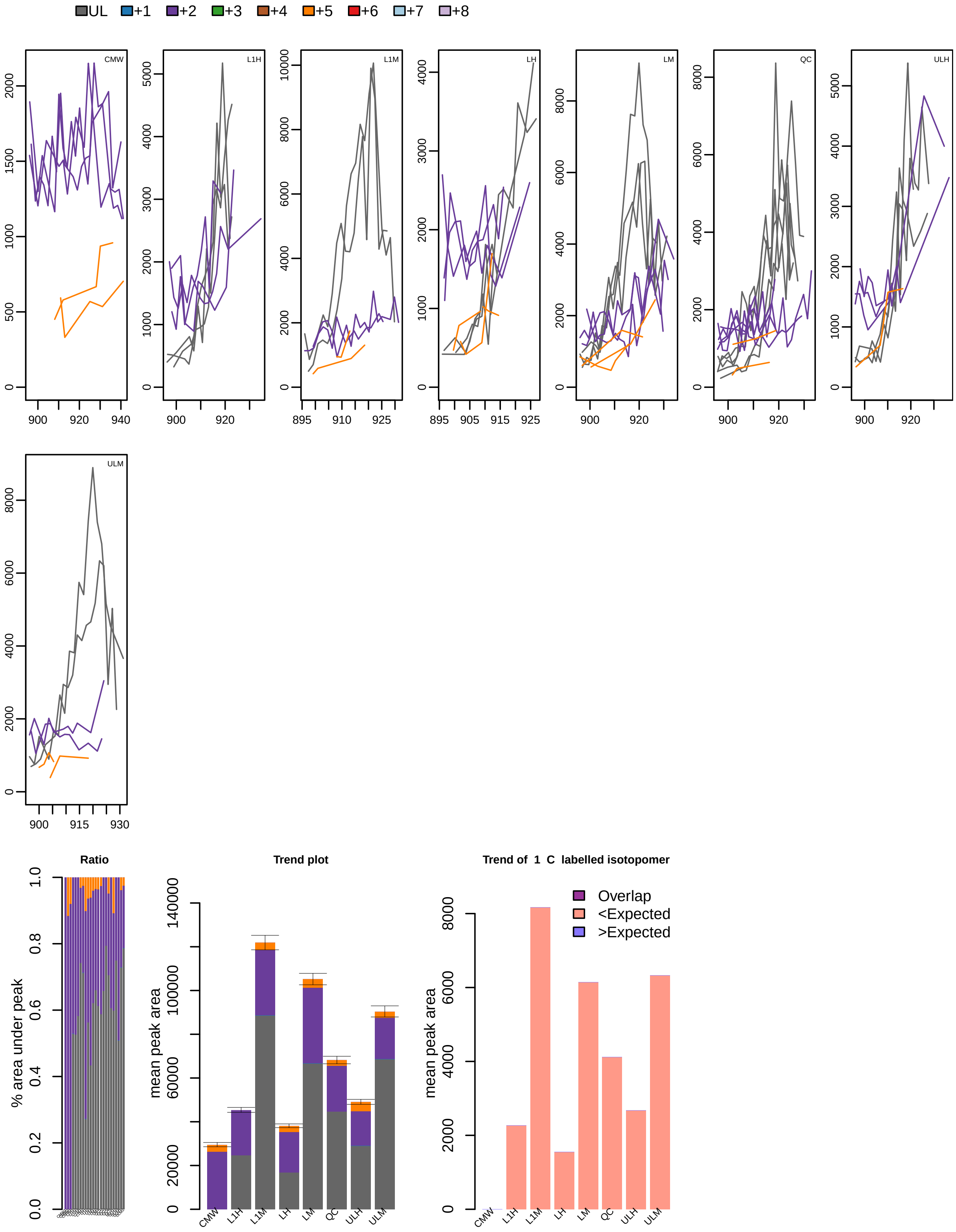

Glycyl−leucine

Formula: $C_8H_{16}N_2O_3$ Mass: 188.116 Std.RT: 432.4188321 Ion: POS

G1

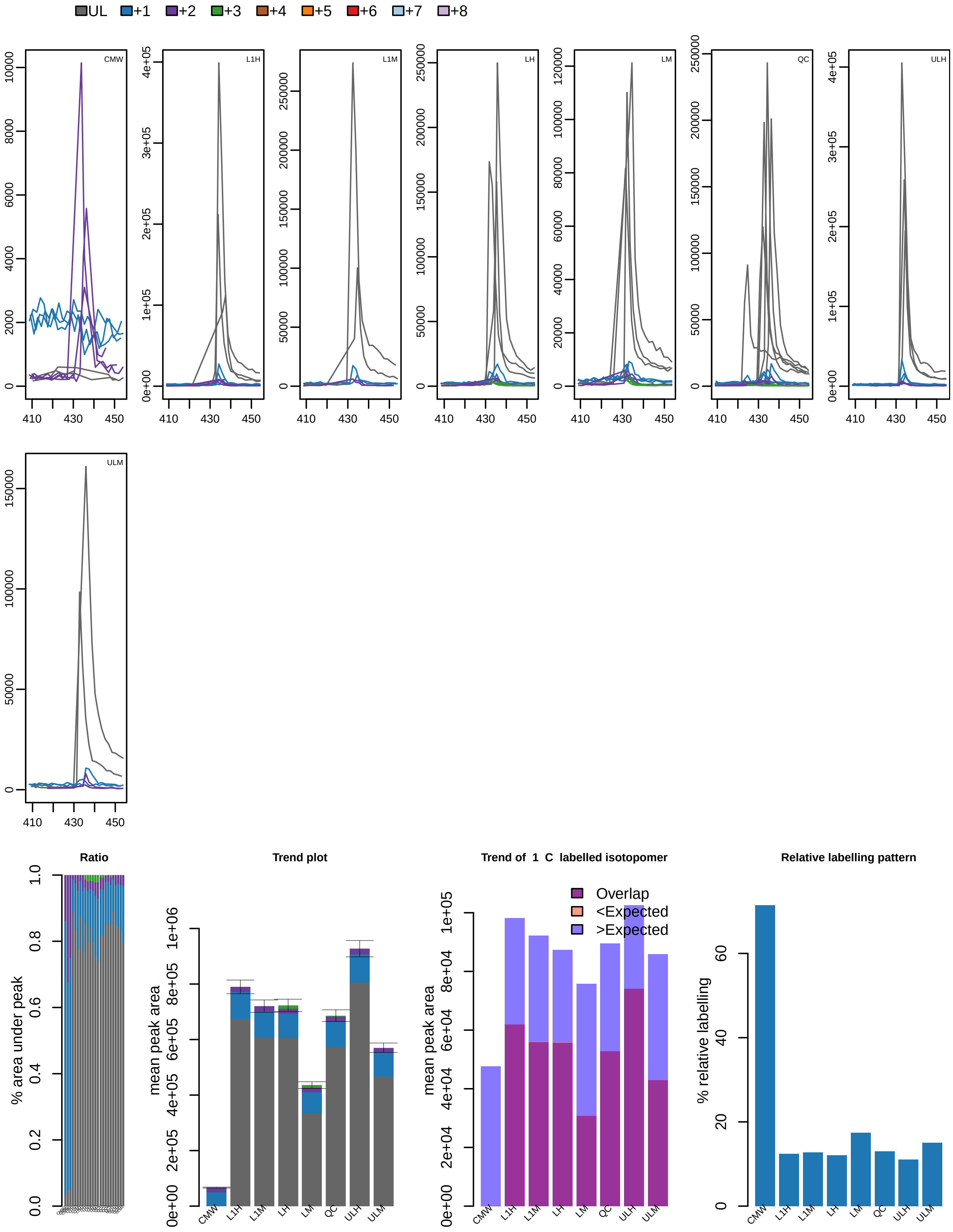

Leu−Ala

Formula: C9H18N2O3 Mass: 202.132 Std.RT: 433.4375814 Ion: POS

G1

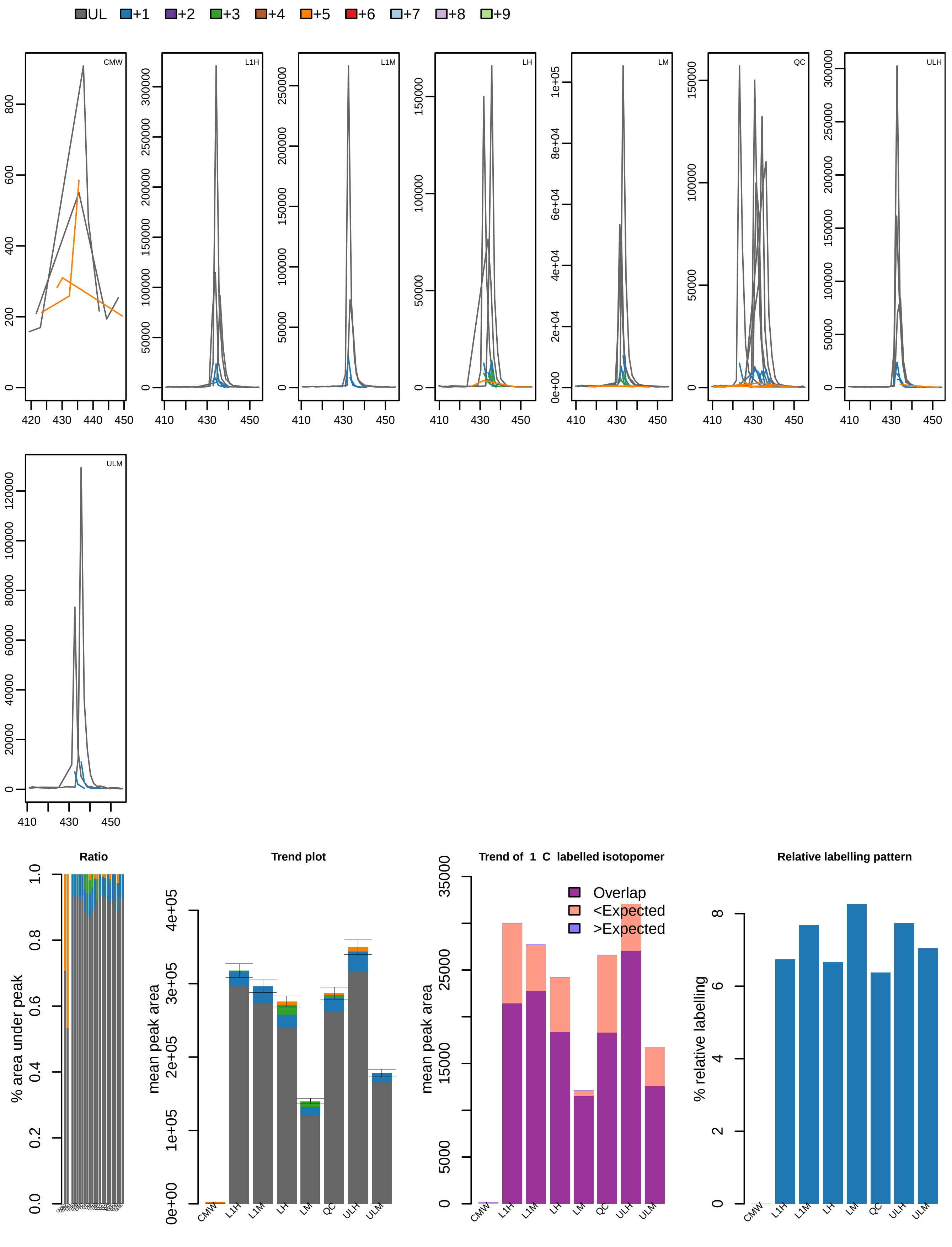

Glu−Leu

Formula: $C11H20N2O5$ Mass: 260.137 Std.RT: 727.1669976 Ion: PC

G1

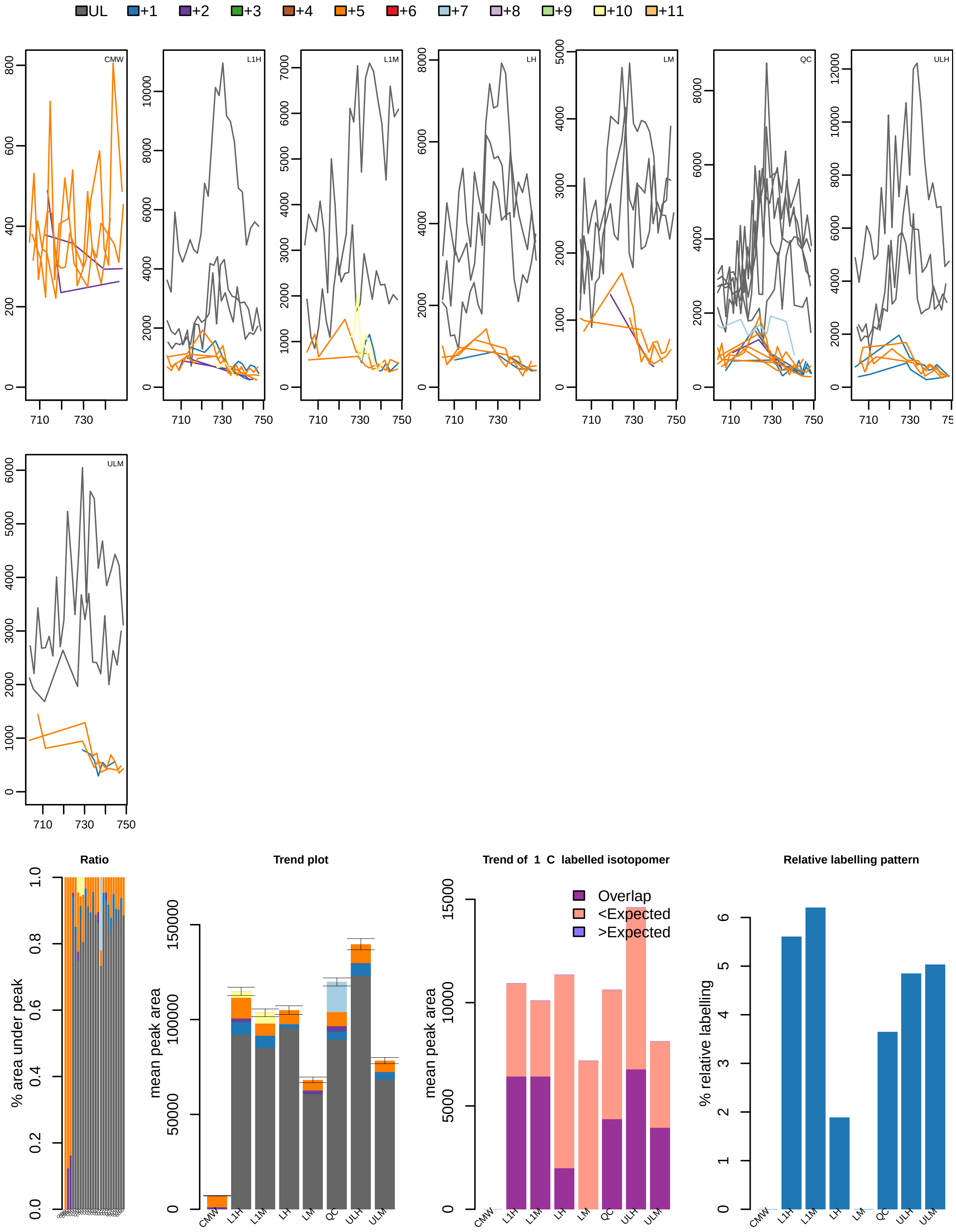

Leu−Thr

Formula: C10H20N2O4 Mass: 232.142 Std.RT: 432.96095058 Ion: P

G1

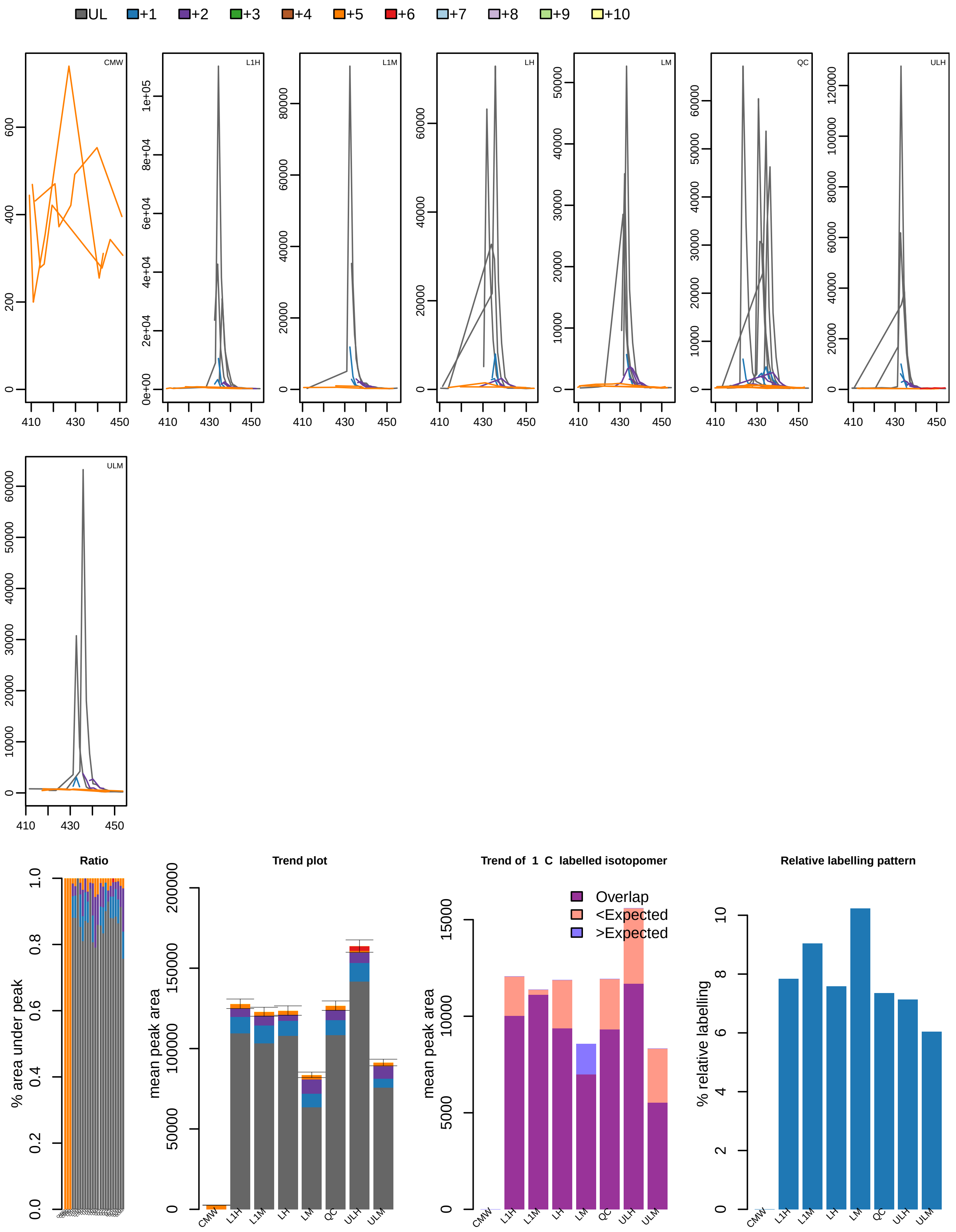

Leu−Pro

Formula: C11H20N2O3 Mass: 228.147 Std.RT: 433.37370678 Ion: P

G1

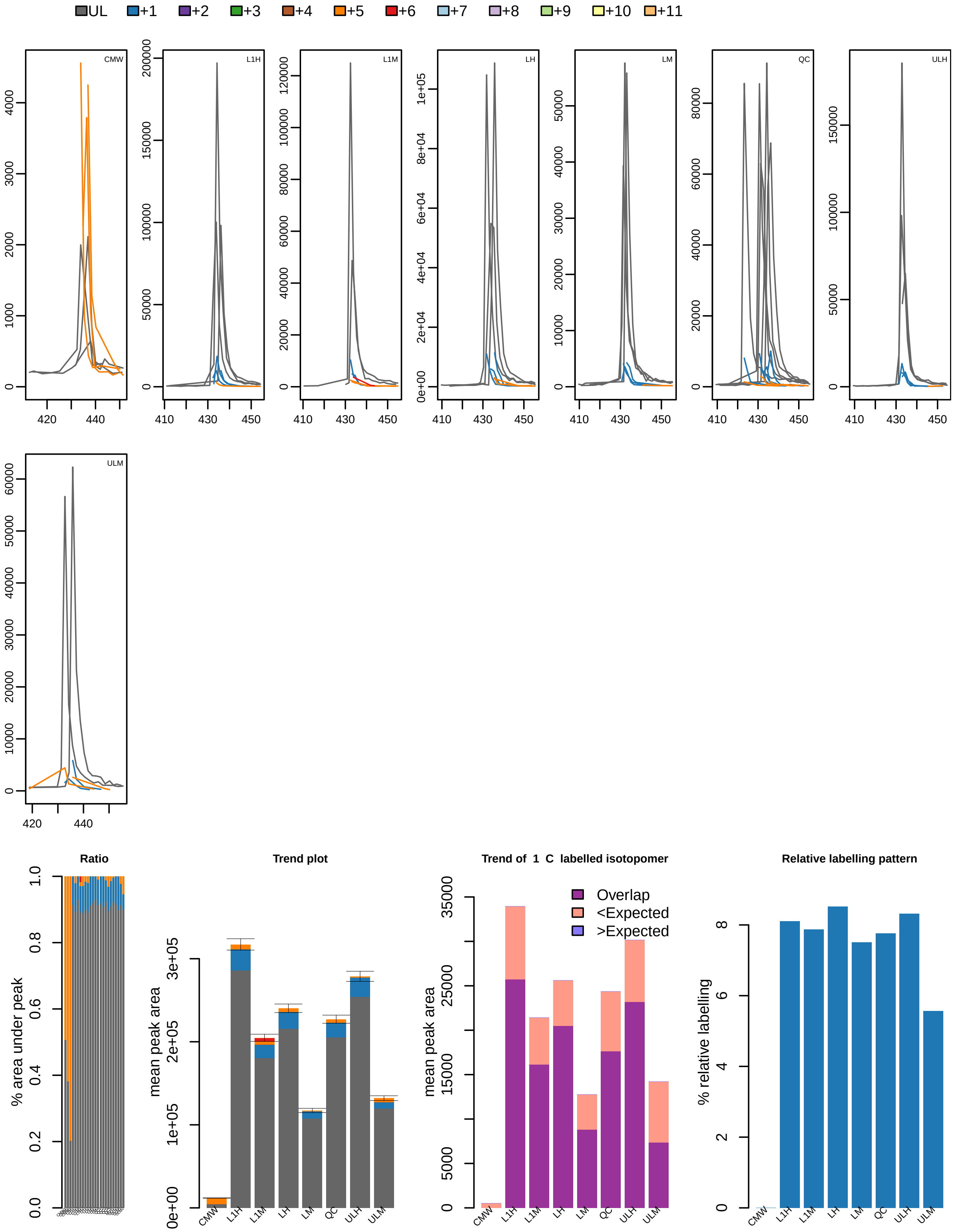

Phe−Gly

Formula: C11H14N2O3 Mass: 222.1 Std.RT: 433.184082 Ion: POS

G1

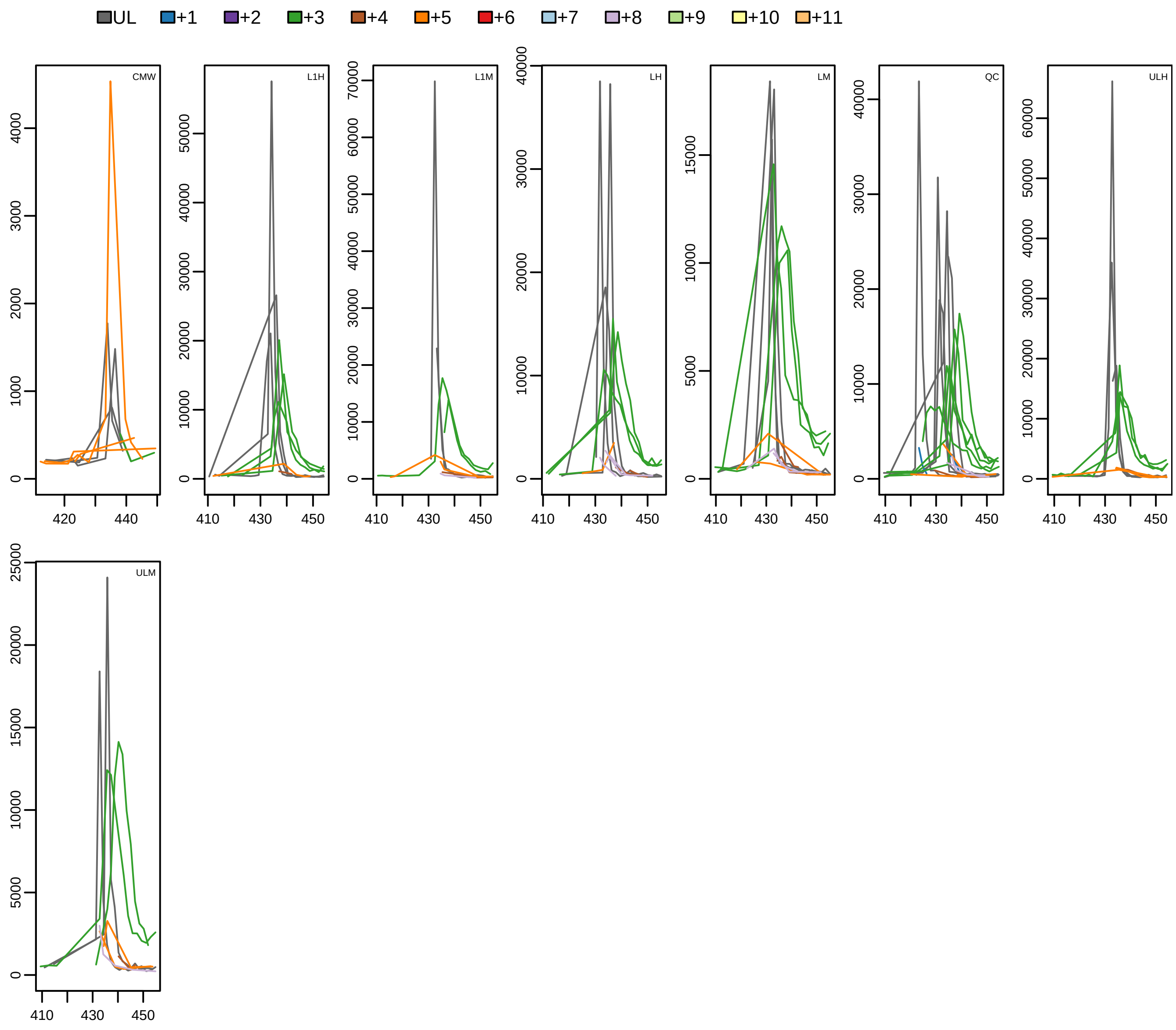

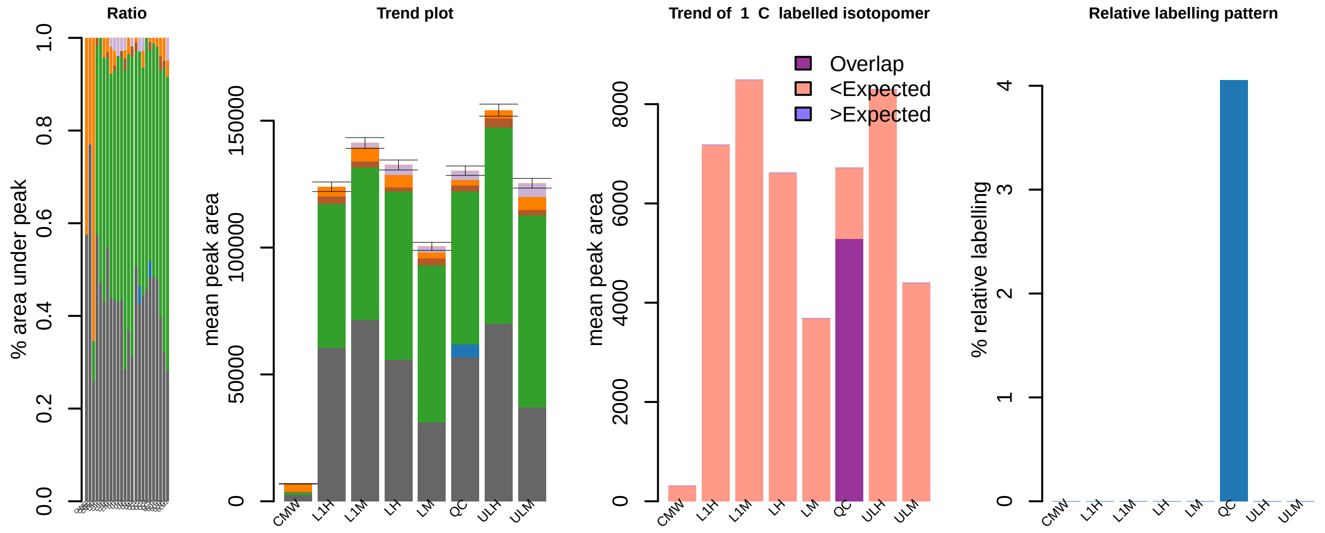

Leu−Ser

Formula: C9H18N2O4 Mass: 218.127 Std.RT: 435.6976374 Ion: POS

G1

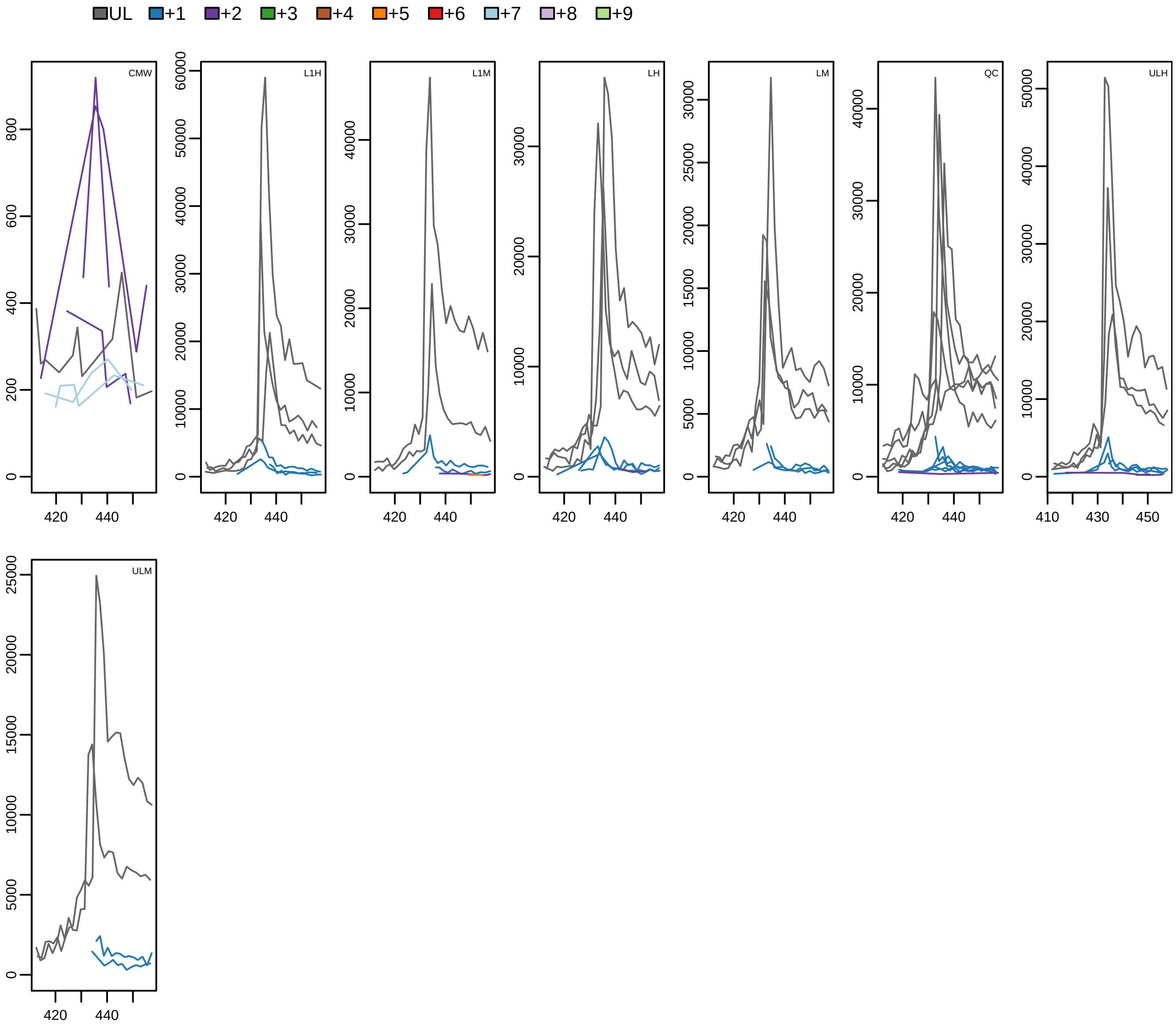

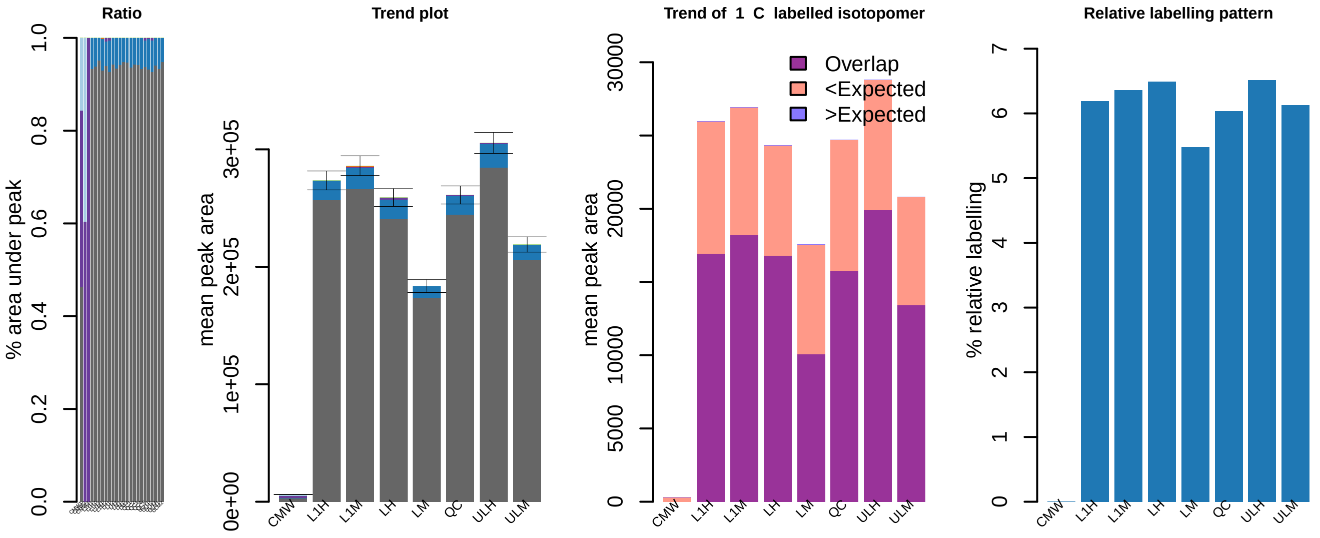

Ala−Tyr

G1

Formula: C12H16N2O4 Mass: 252.111 Std.RT: 433.18399938 Ion: P

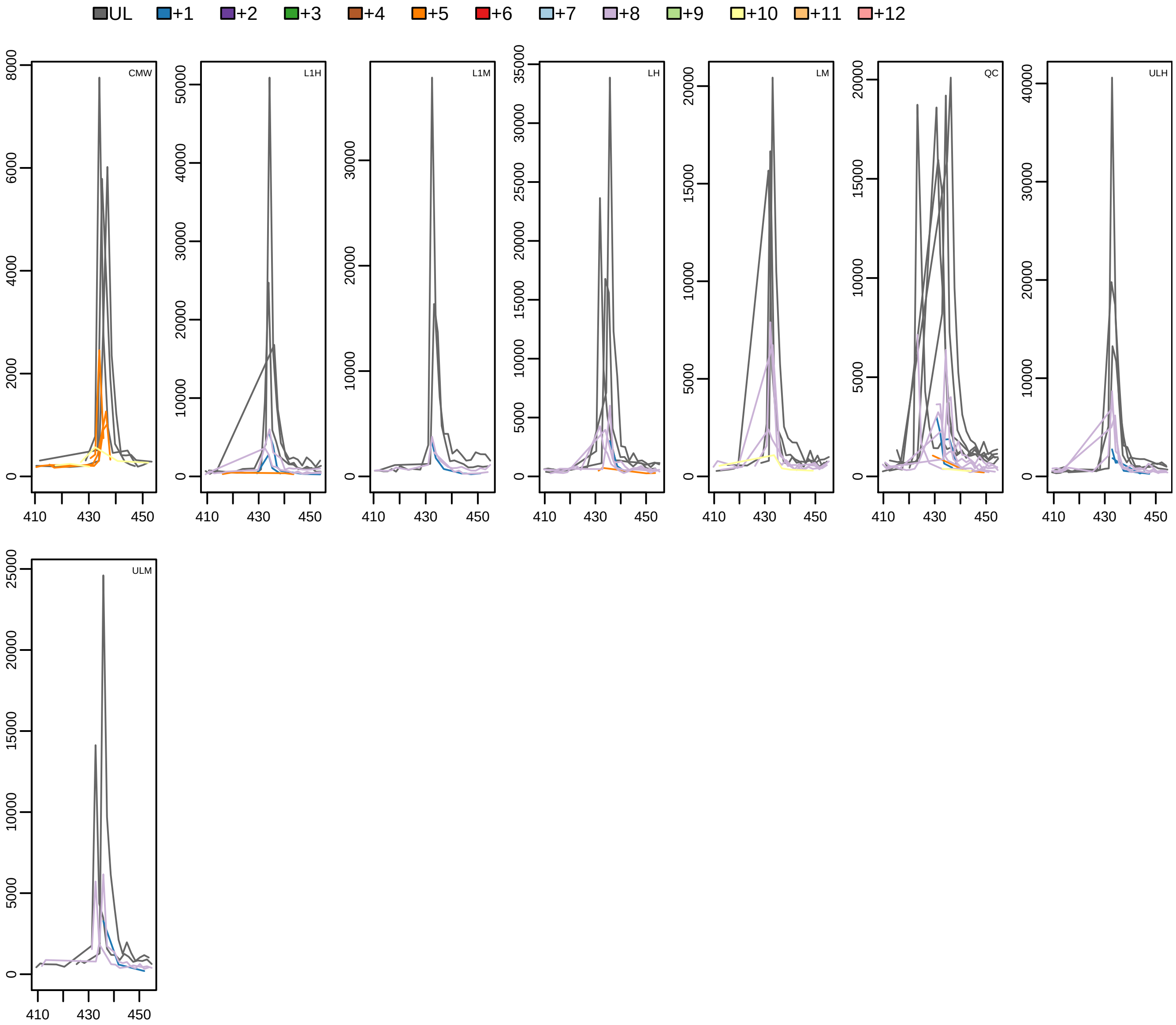

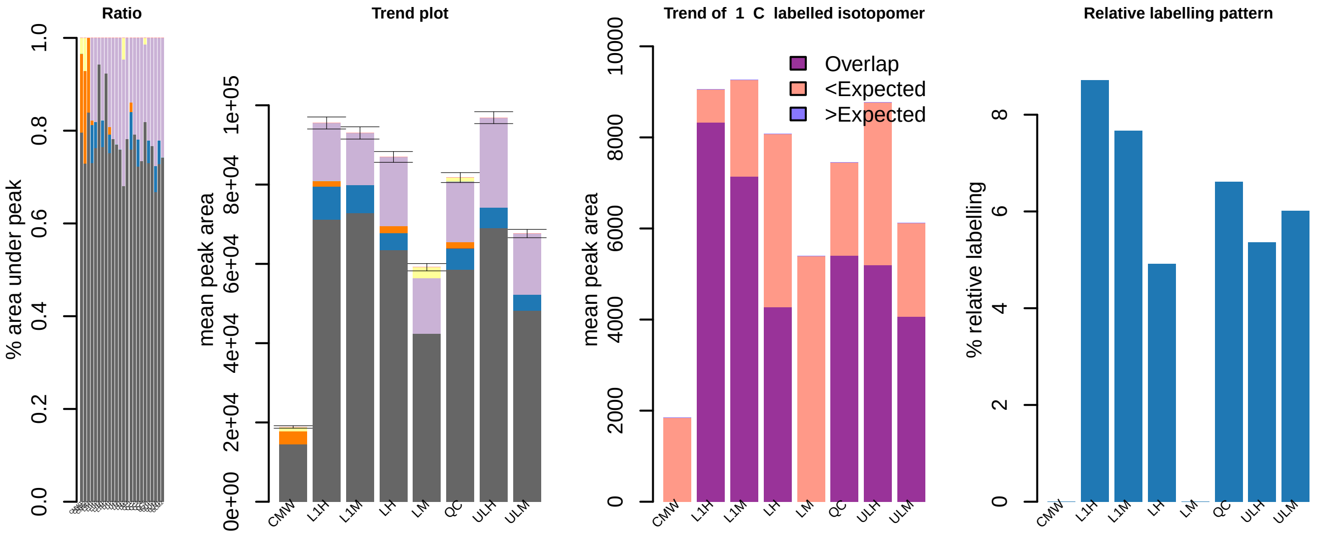

Trp−Ala

Formula: C14H17N3O3 Mass: 275.127 Std.RT: 434.12966628 Ion: P

G1

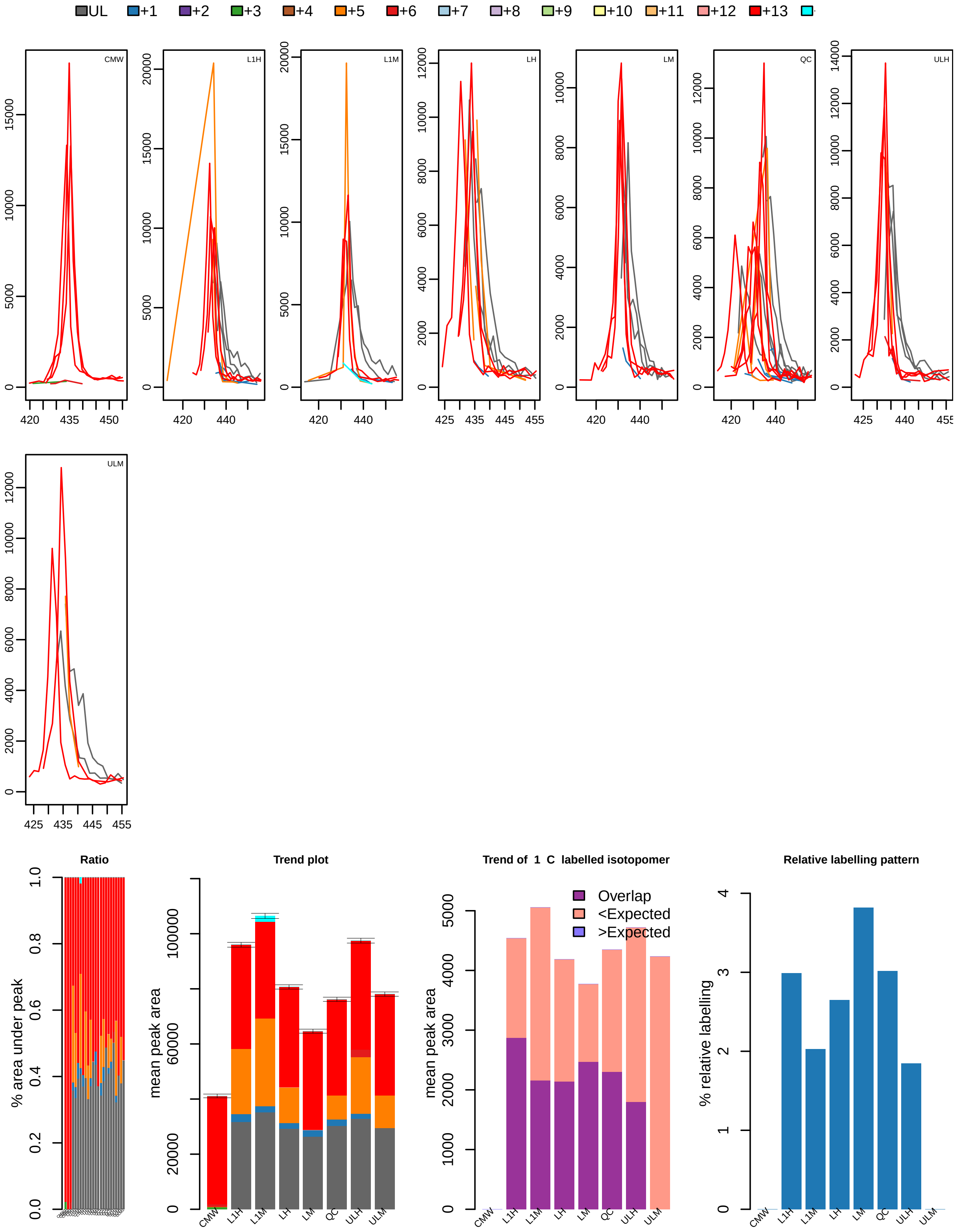

Ala−Pro

G1

Formula: C8H14N2O3 Mass: 186.1 Std.RT: 716.935008 Ion: POS

UL +1 +2 +3 +4 +5 +6 +7 +8

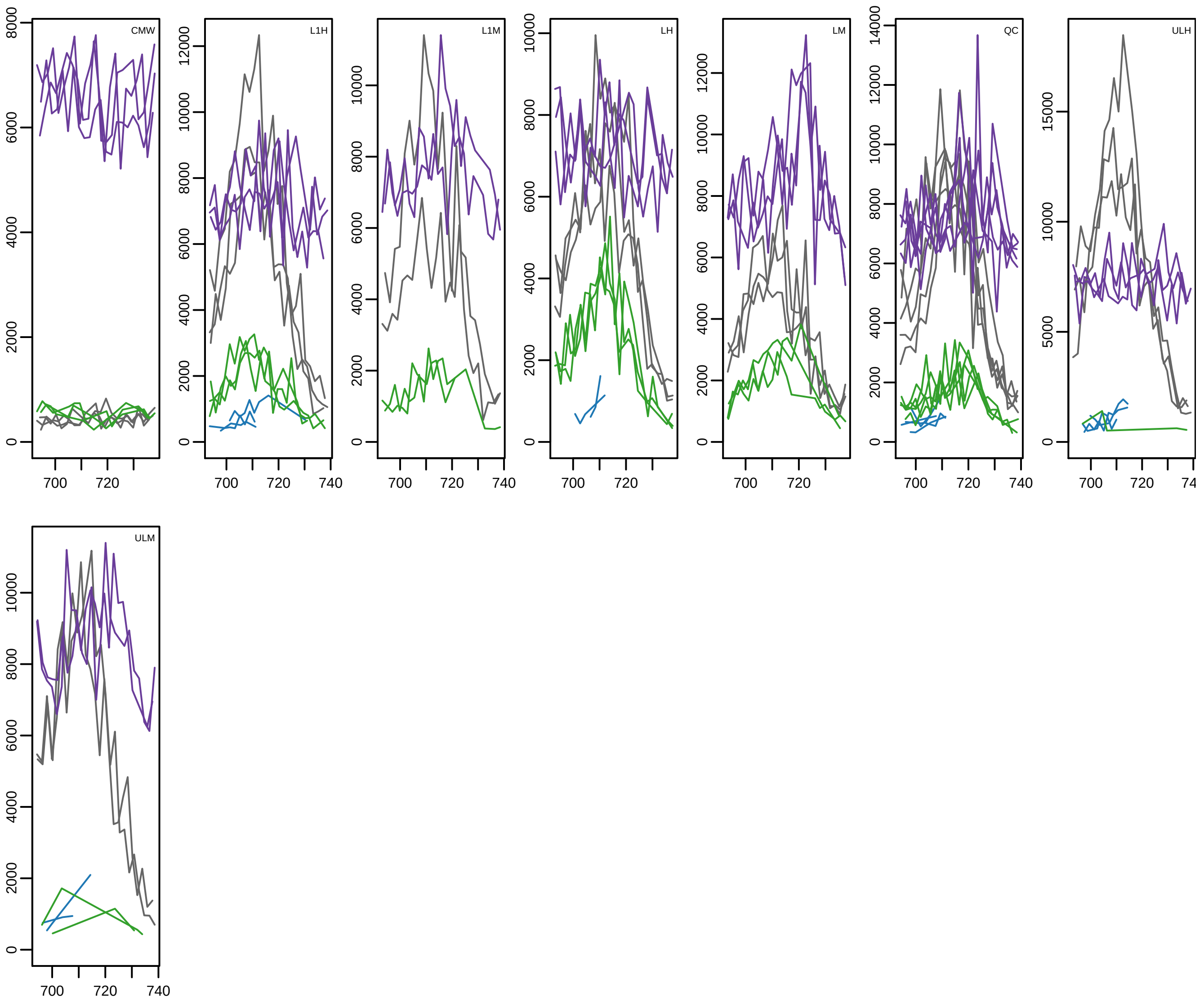

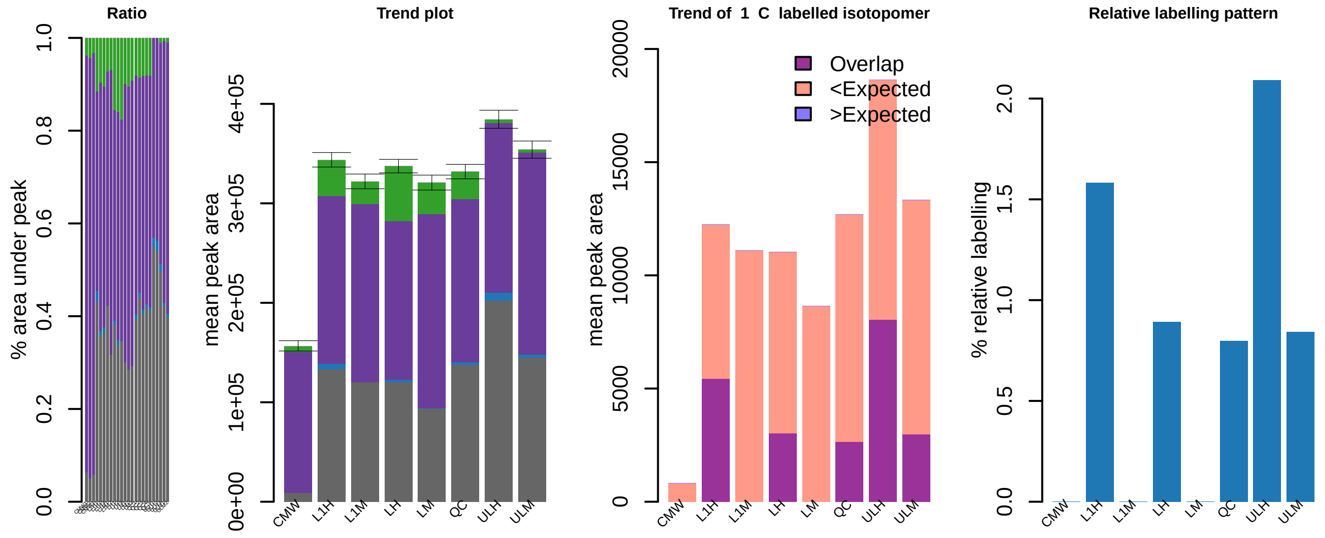

Thr−Pro

Formula: C9H16N2O4 Mass: 216.111 Std.RT: 497.40385512 Ion: PC

G1

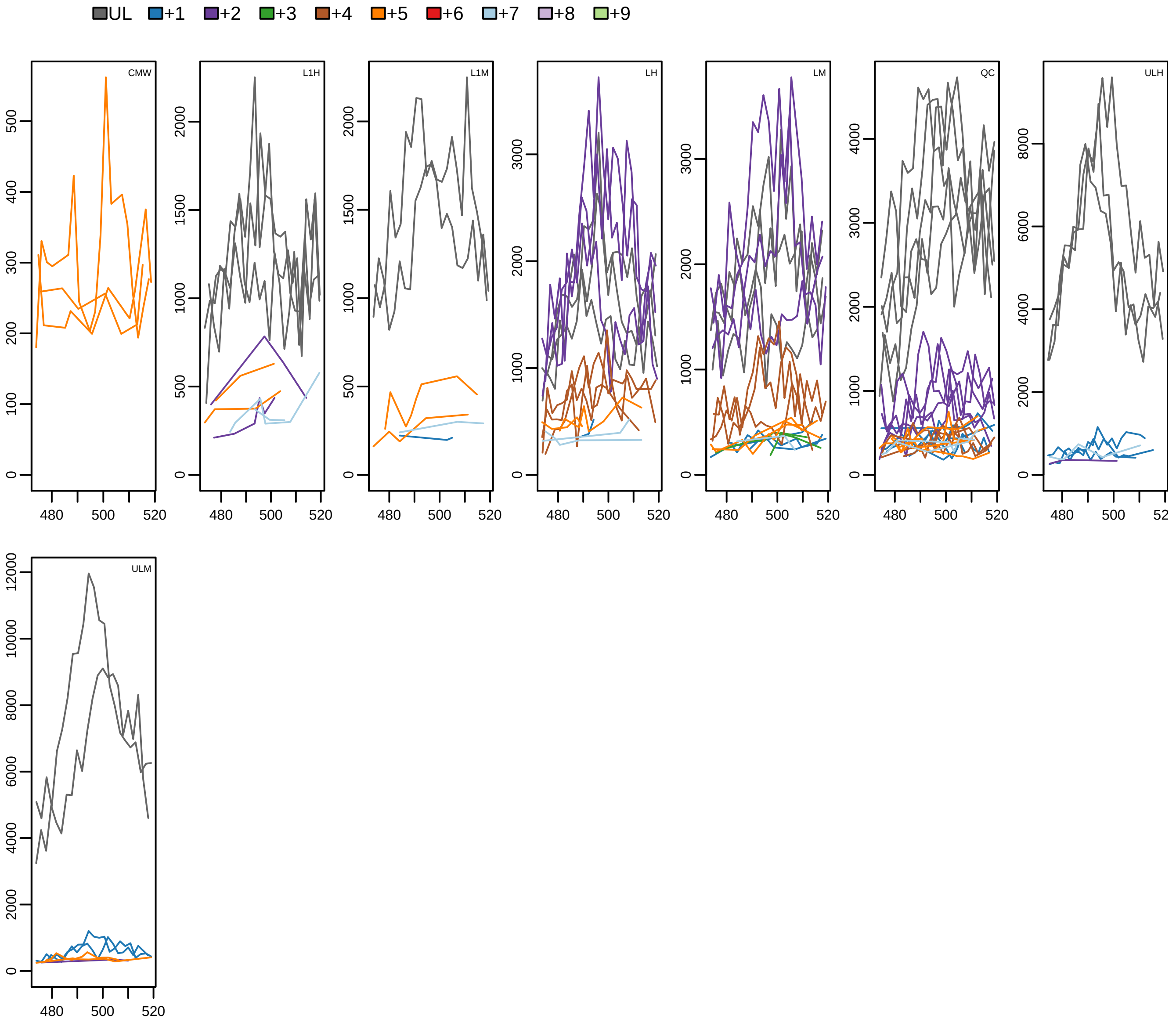

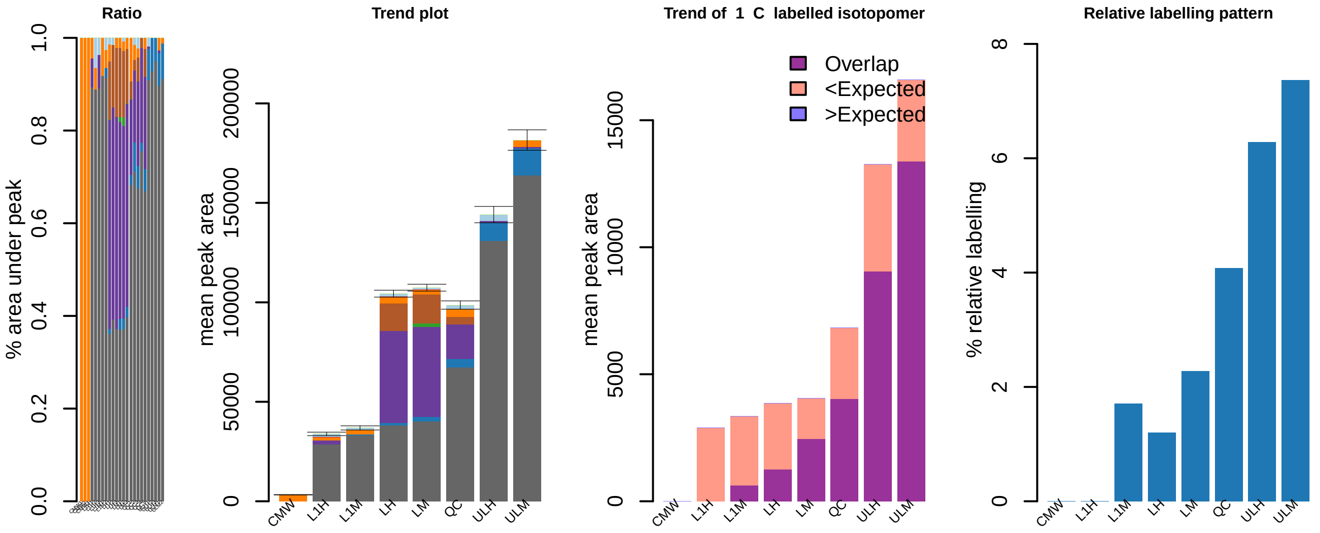

Thr−Ala

Formula: C7H14N2O4 Mass: 190.095 Std.RT: 691.356573 Ion: POS

G1

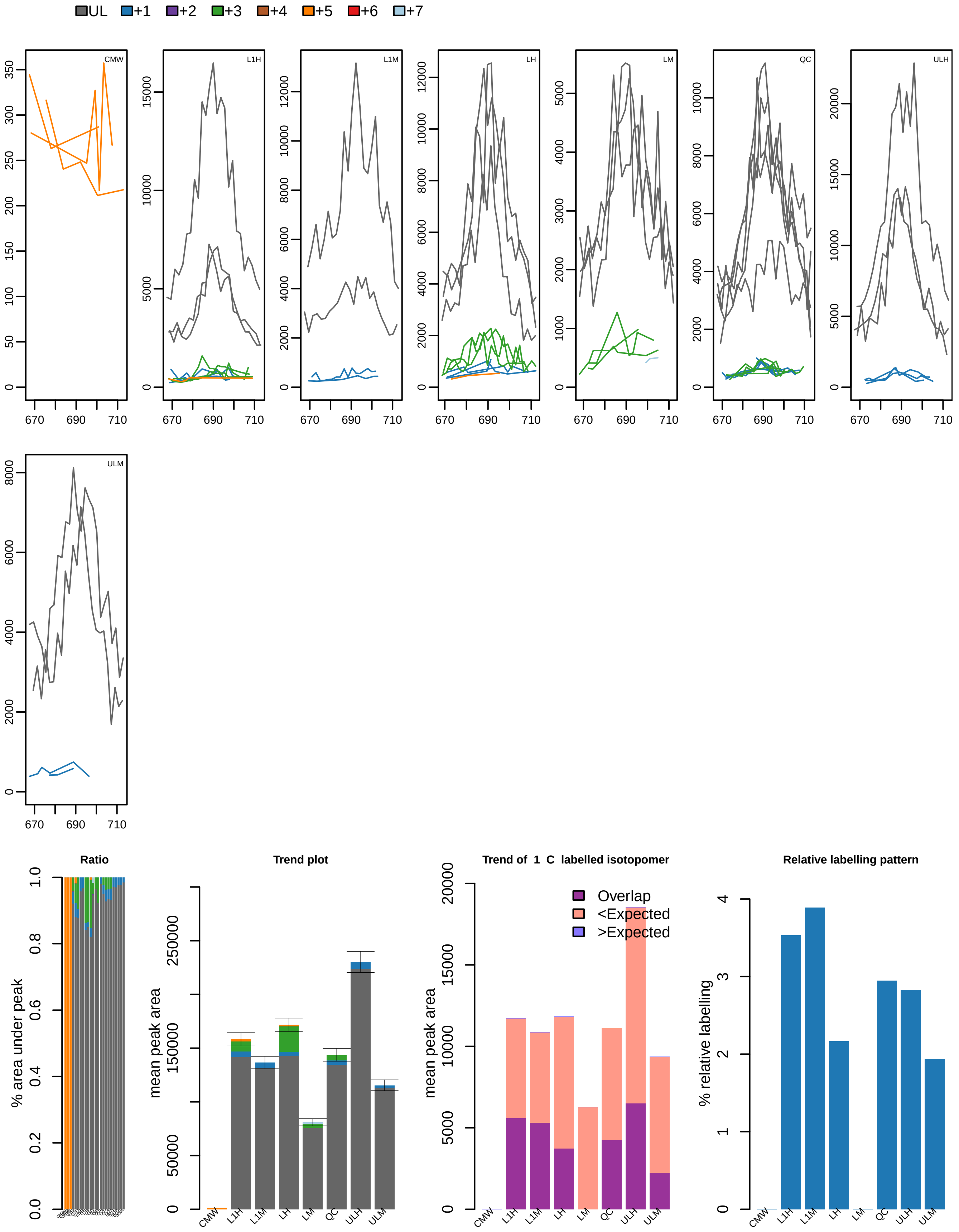

Asn−Ser

Formula: C7H13N3O5 Mass: 219.086 Std.RT: 840.9663024 Ion: POS

G1

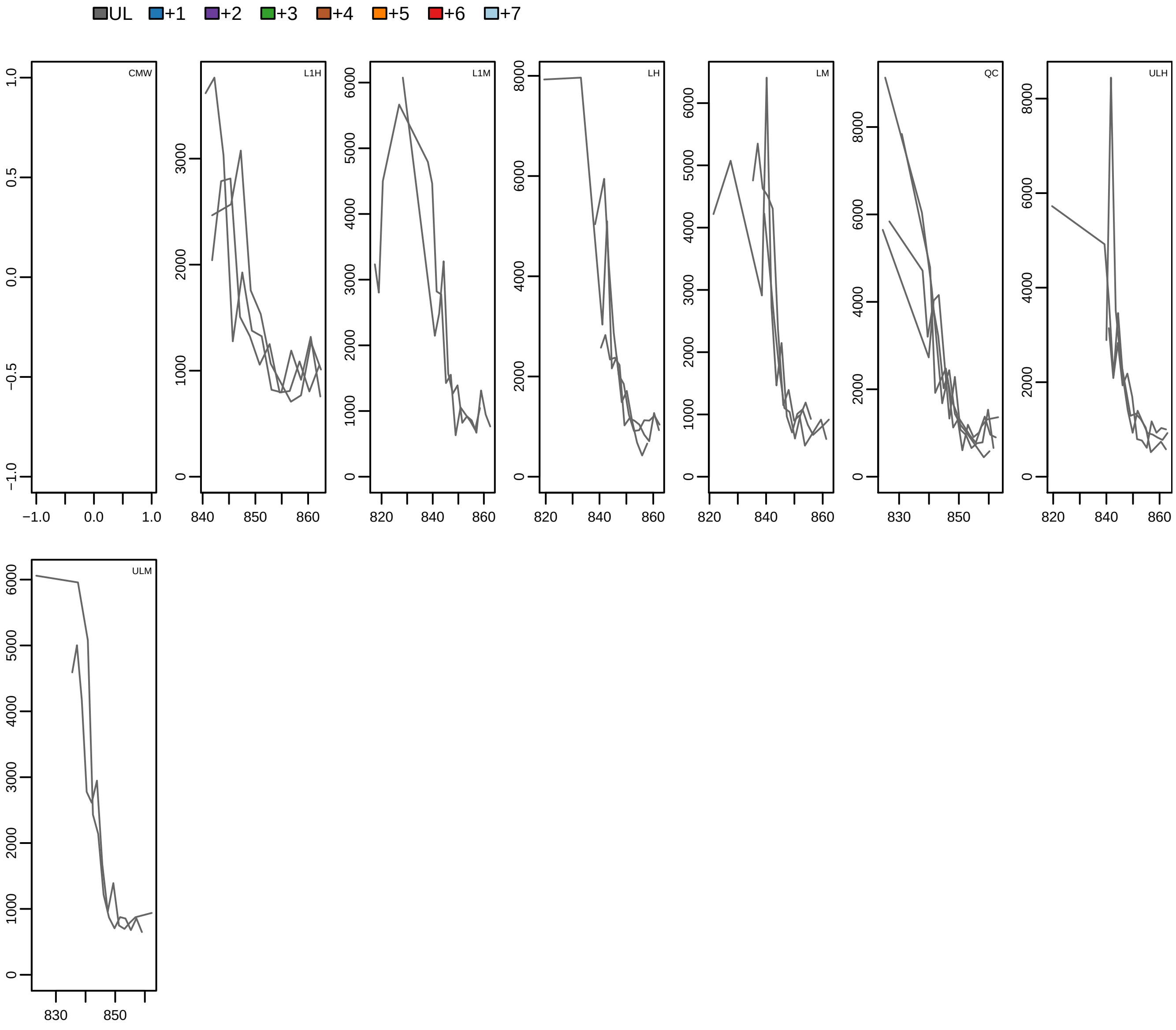

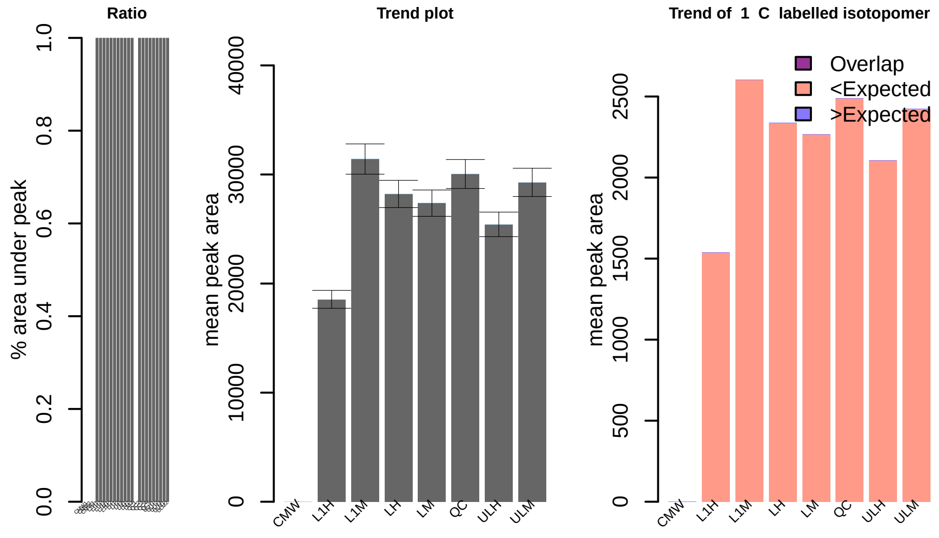

Glycylproline

Formula: C7H12N2O3 Mass: 172.085 Std.RT: 798.3779982 Ion: POS

G1

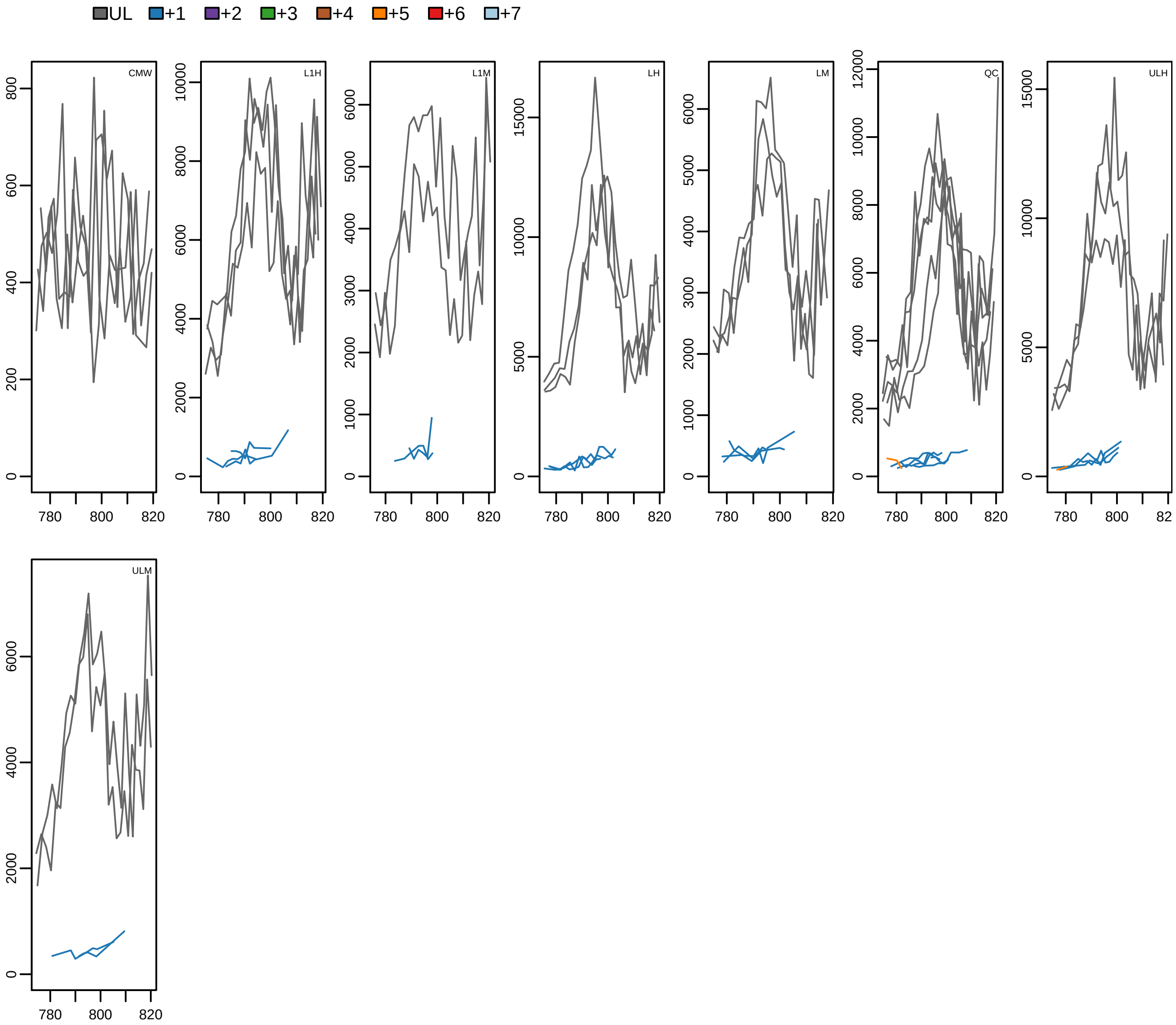

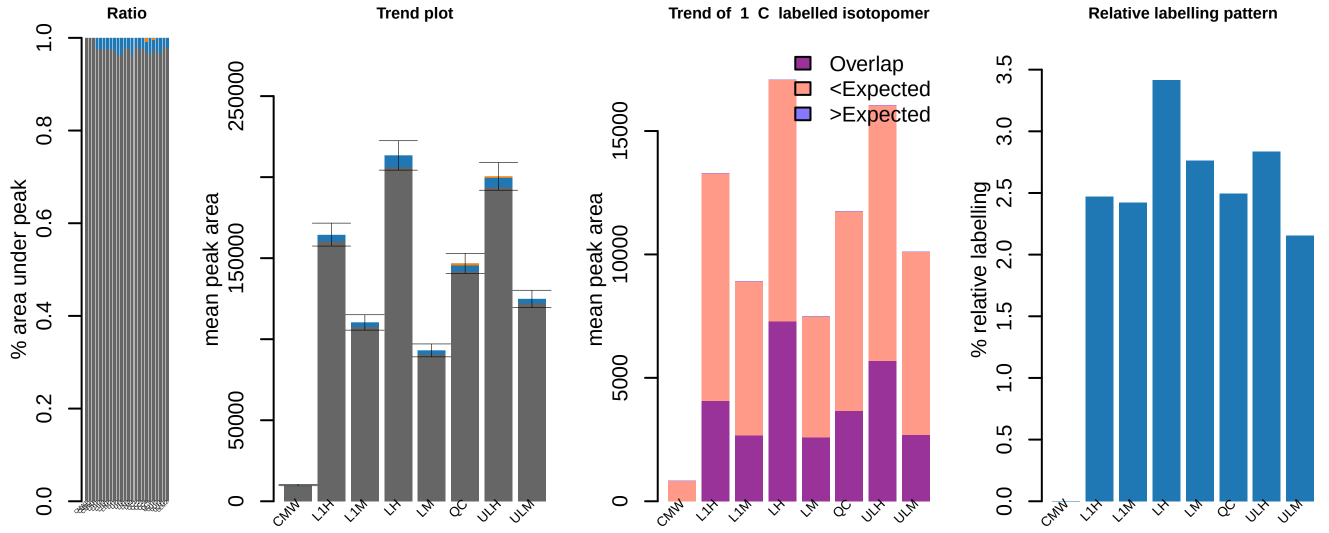

Gln−Lys−Trp−Tyr

Formula: C31H41N7O7 Mass: 623.307 Std.RT: 1274.5078338 Ion: P

G1

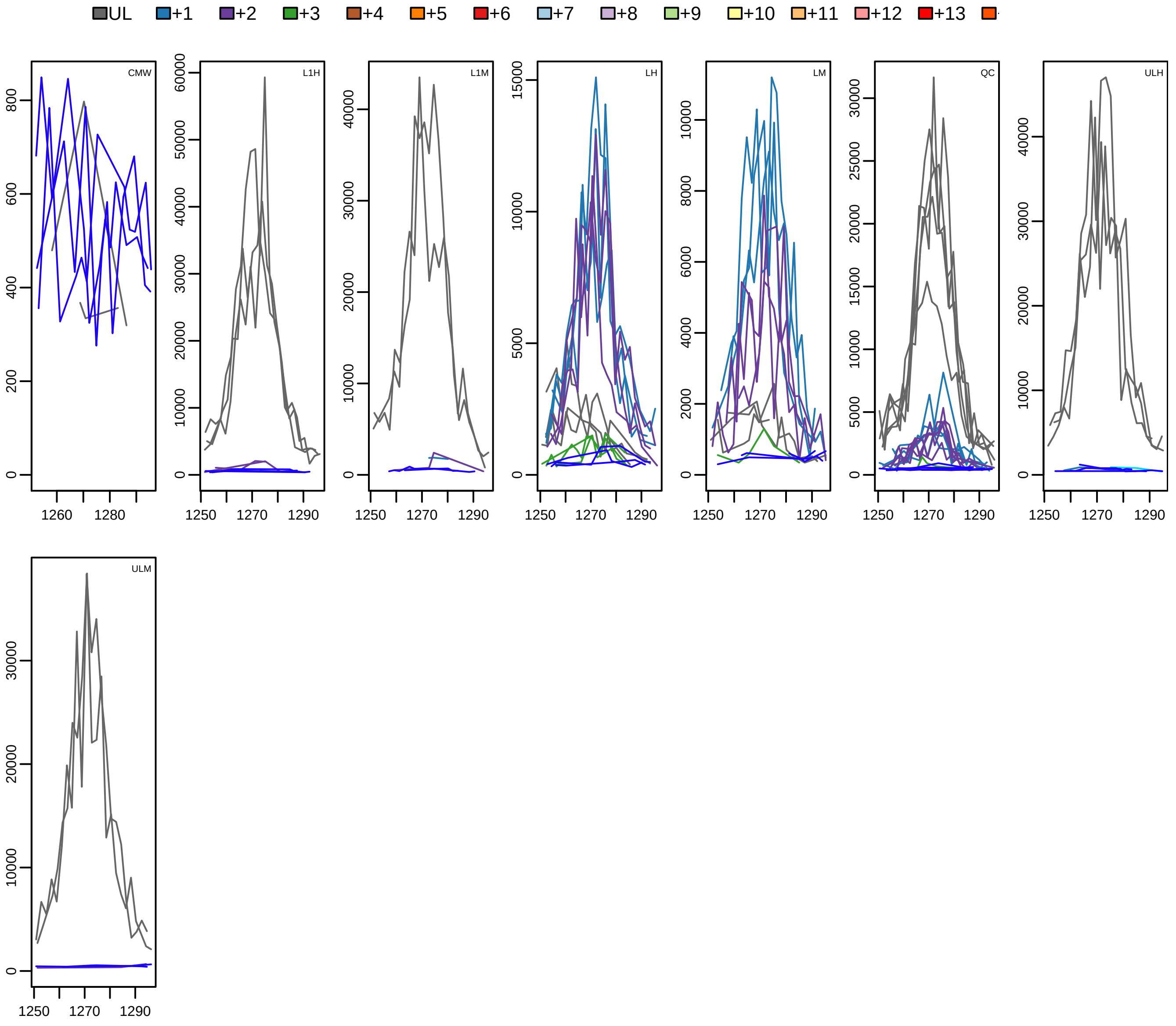

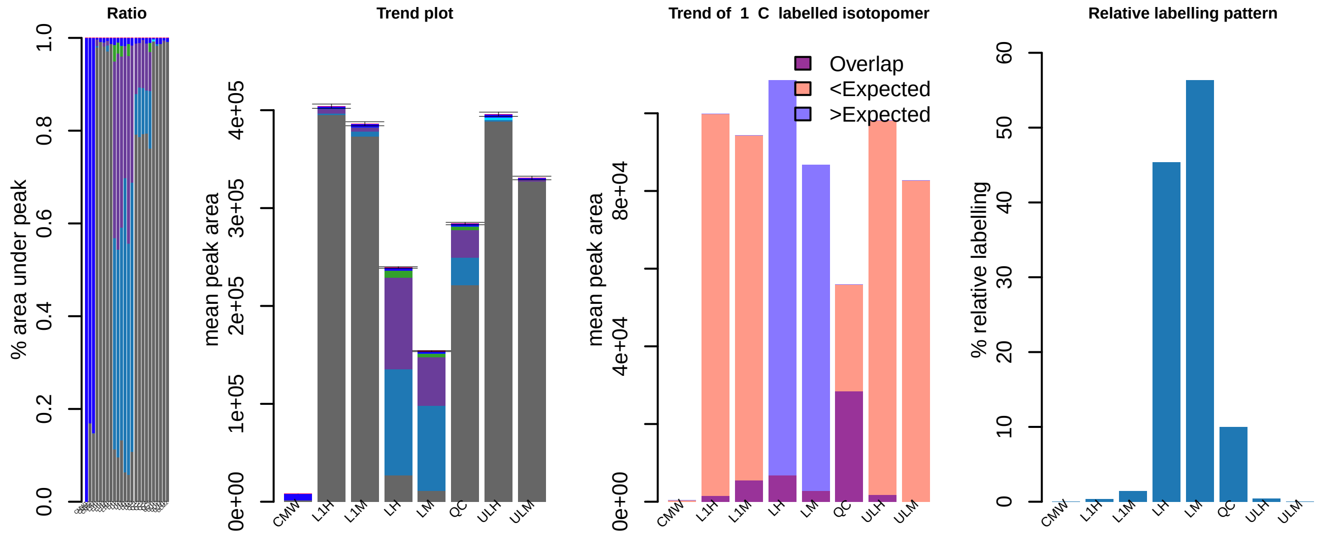

Ala−Leu−Lys−Val

Formula: C20H39N5O5 Mass: 429.295 Std.RT: 706.9491432 Ion: PC

G1

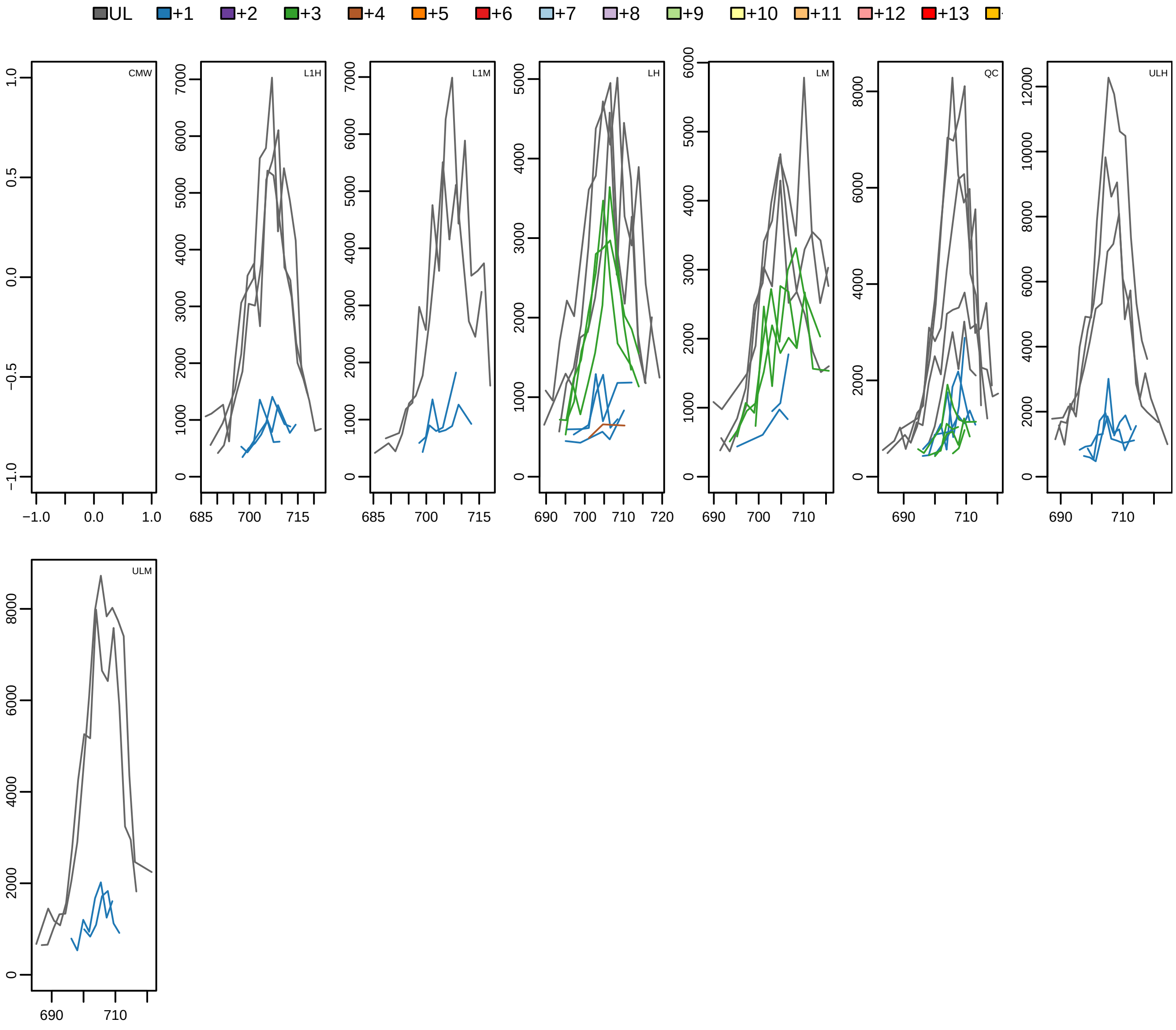

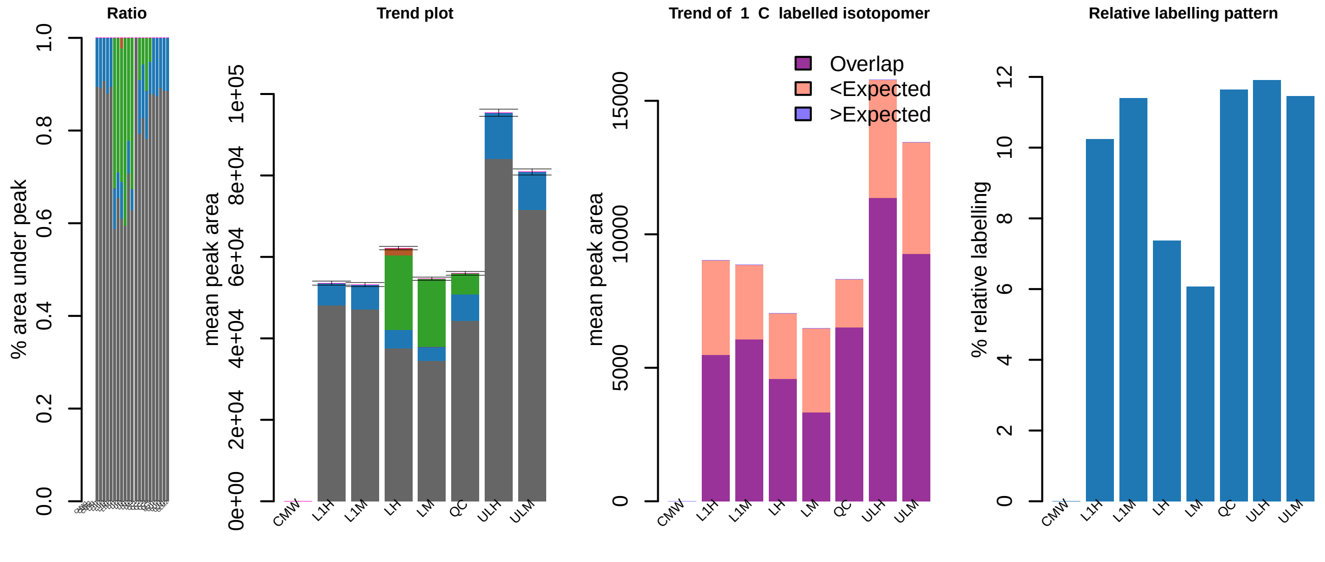

Arg−Phe−Val−Gly

Formula: C22H35N7O5 Mass: 477.27 Std.RT: 686.2133664 Ion: POS

G1

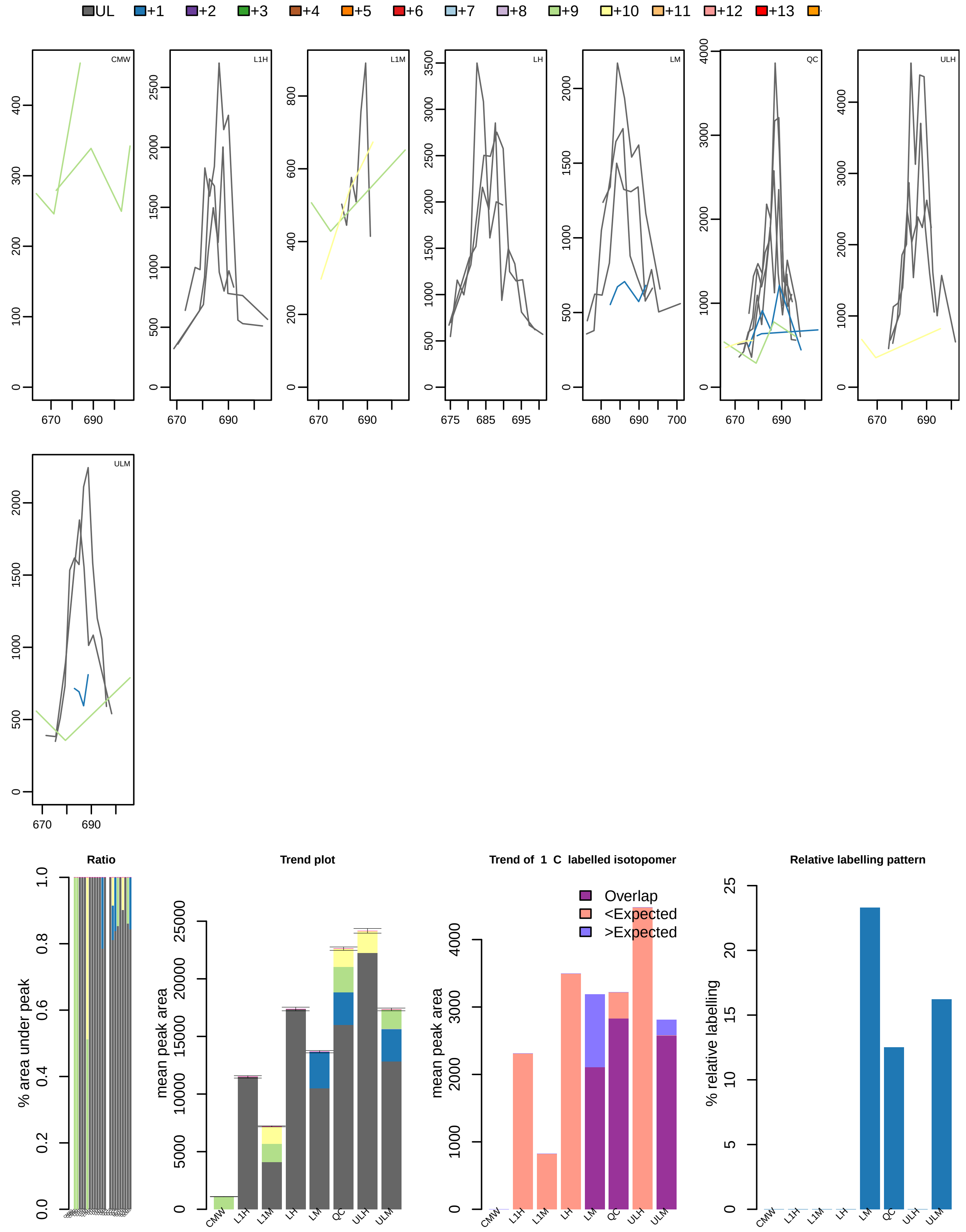

Cys−Lys−Lys−Tyr

Formula: C24H40N6O6S Mass: 540.273 Std.RT: 1676.0691582 Ion:

G1

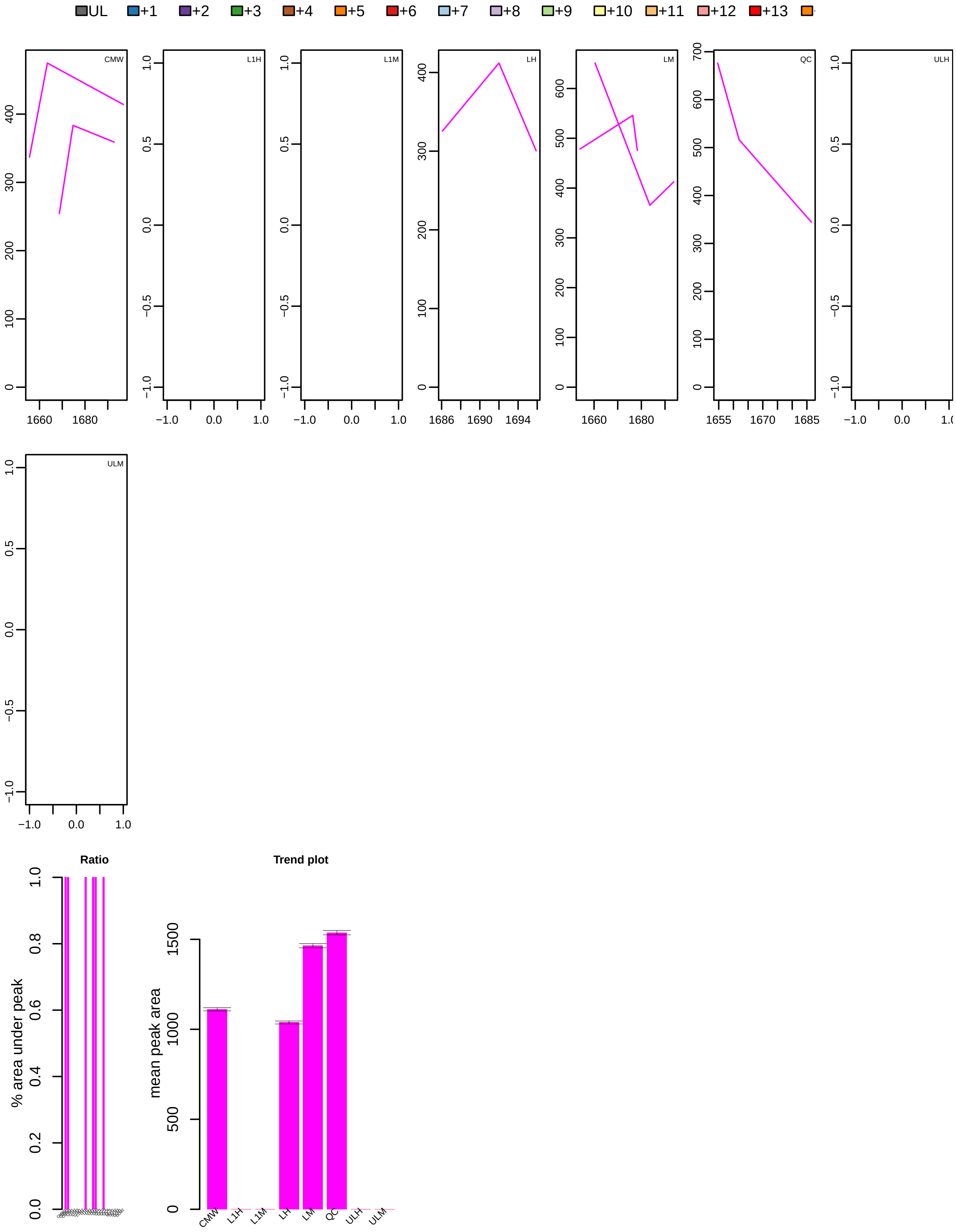

Ala−Leu−Ala−Pro

Formula: C17H30N4O5 Mass: 370.222 Std.RT: 432.3455709 Ion: PC

G1

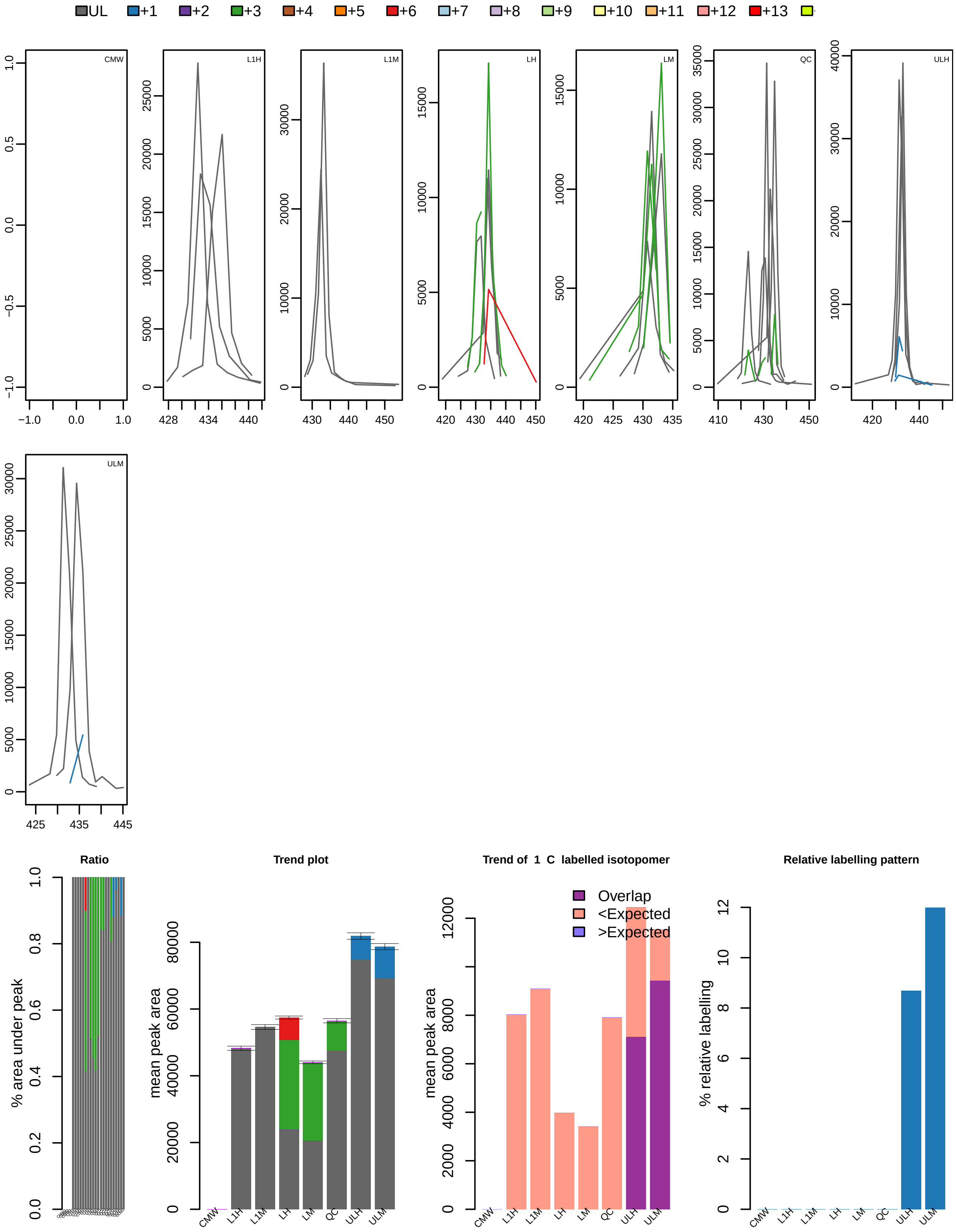

Asn−Met−Asn−Gln

Formula: C18H31N7O8S Mass: 505.195 Std.RT: 547.49890722 Ion:

G1

UL +1 +2 +3 +4 +5 +6 +7 +8 +9 +10 +11 +12 +13

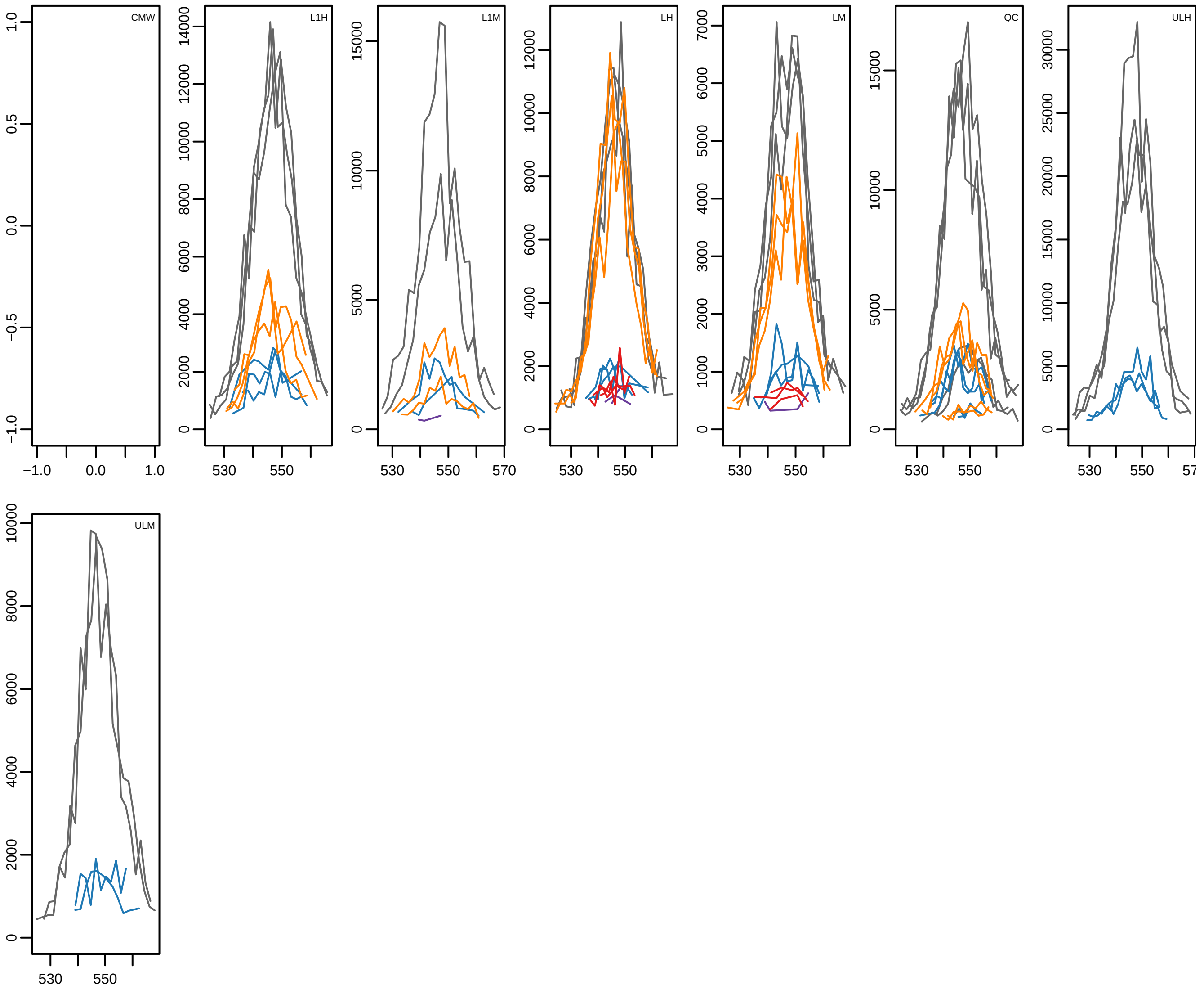

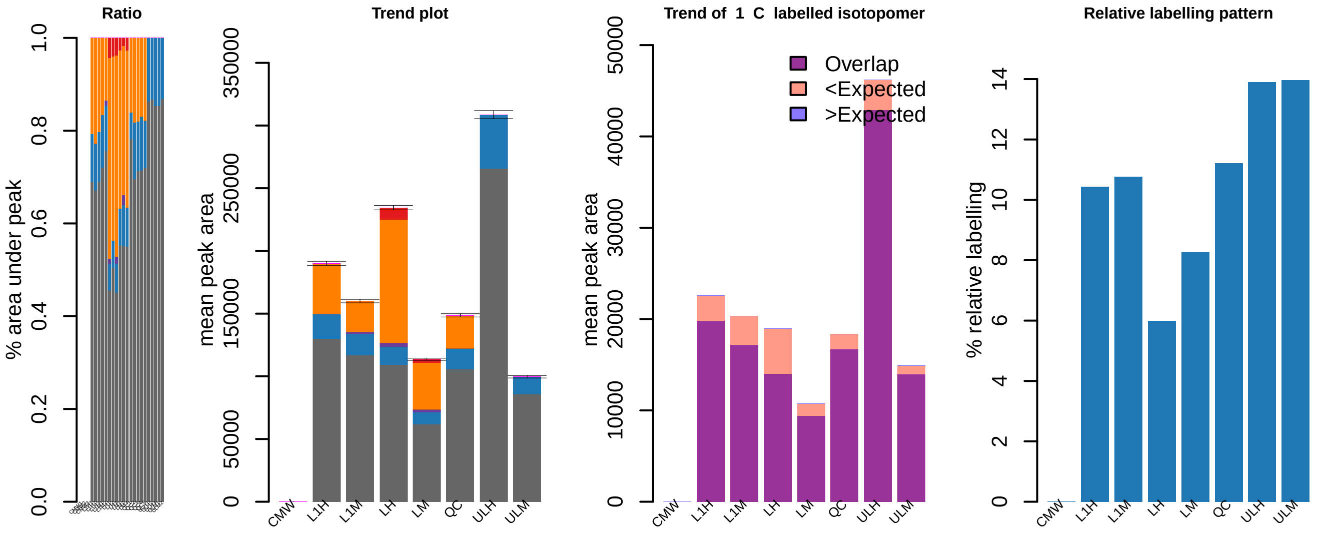

Ala−Leu−Leu−Ser

Formula: C18H34N4O6 Mass: 402.248 Std.RT: 863.7114228 Ion: PC

G1

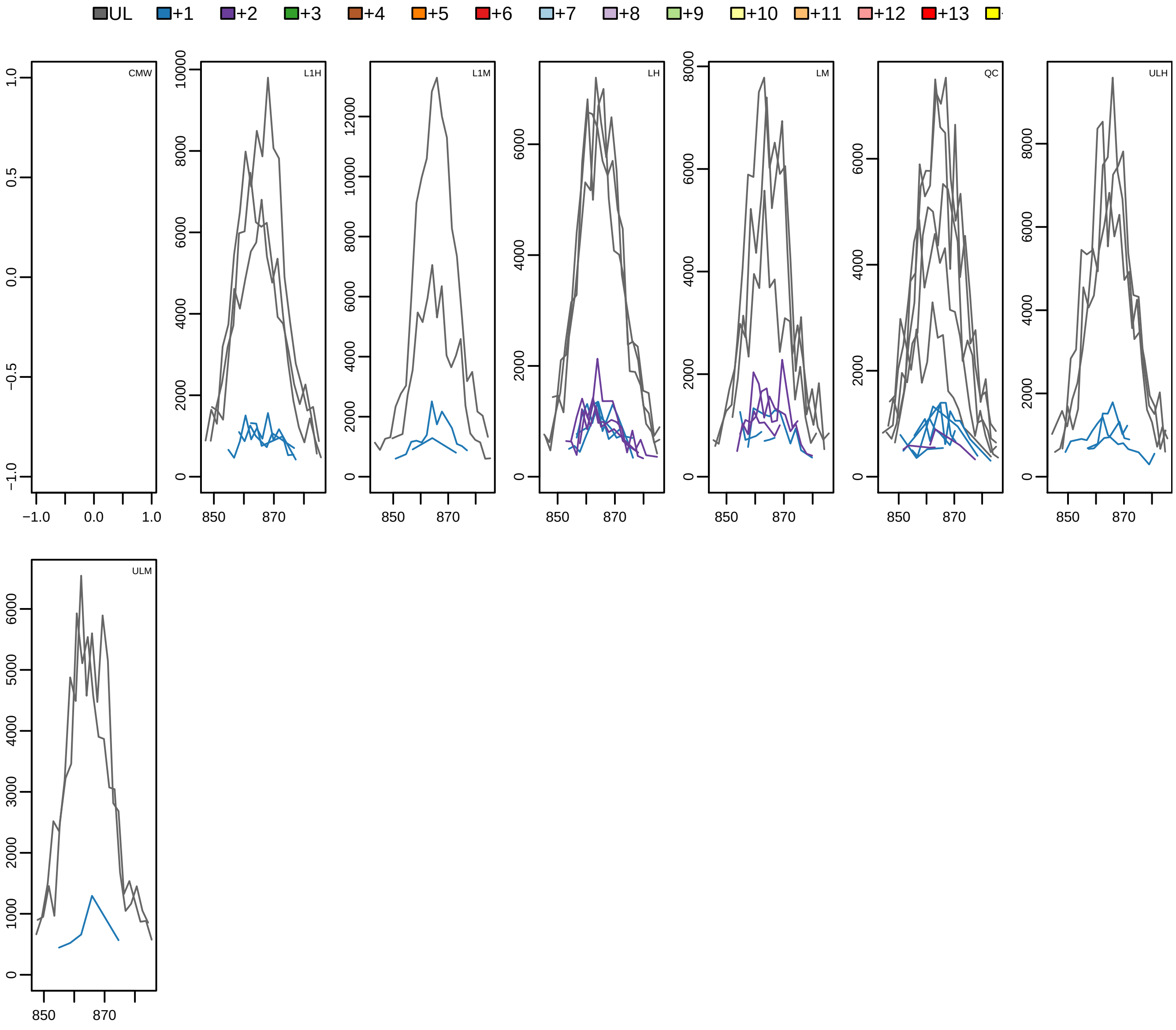

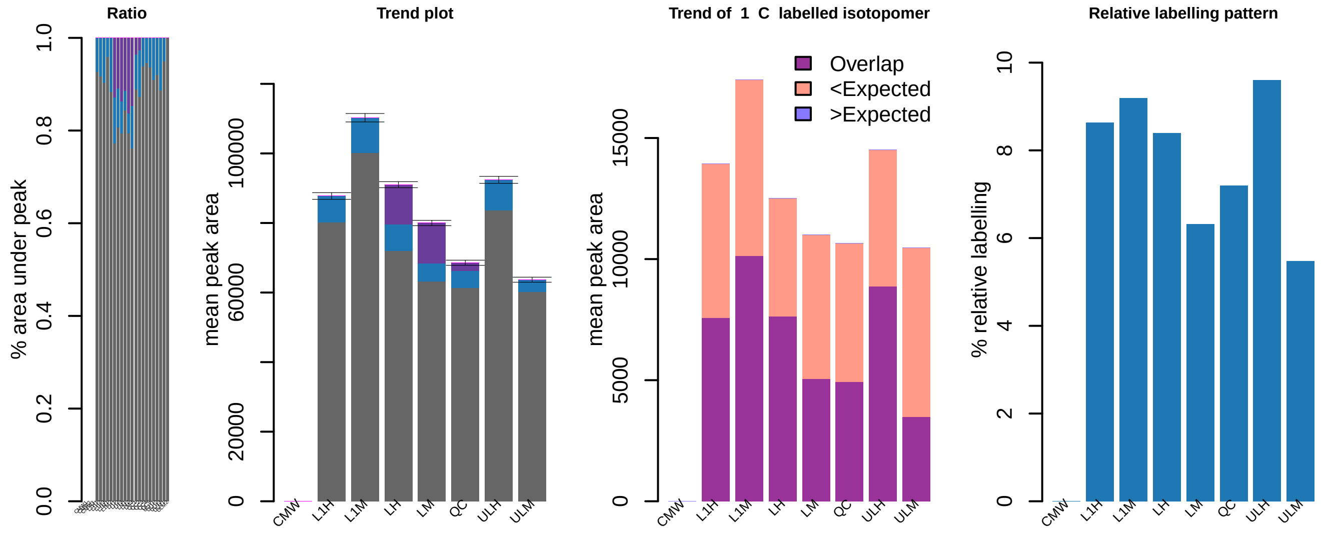

Ala−Leu−Met−Val

Formula: C19H36N4O5S Mass: 432.241 Std.RT: 581.7497559 Ion: P

G1

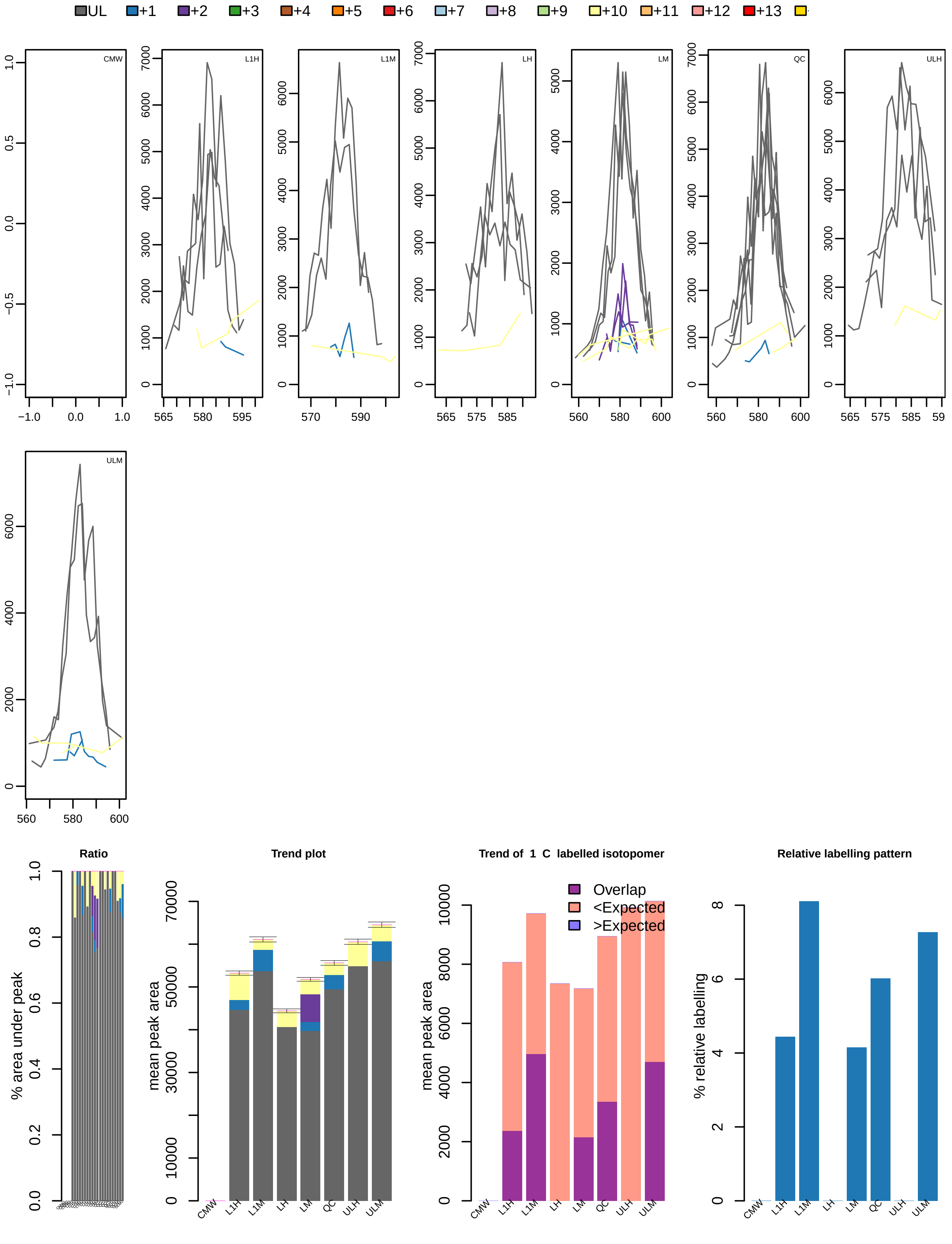

Glu−Glu−Glu

Formula: C15H23N3O10 Mass: 405.138 Std.RT: 1155.6357192 Ion:

G1

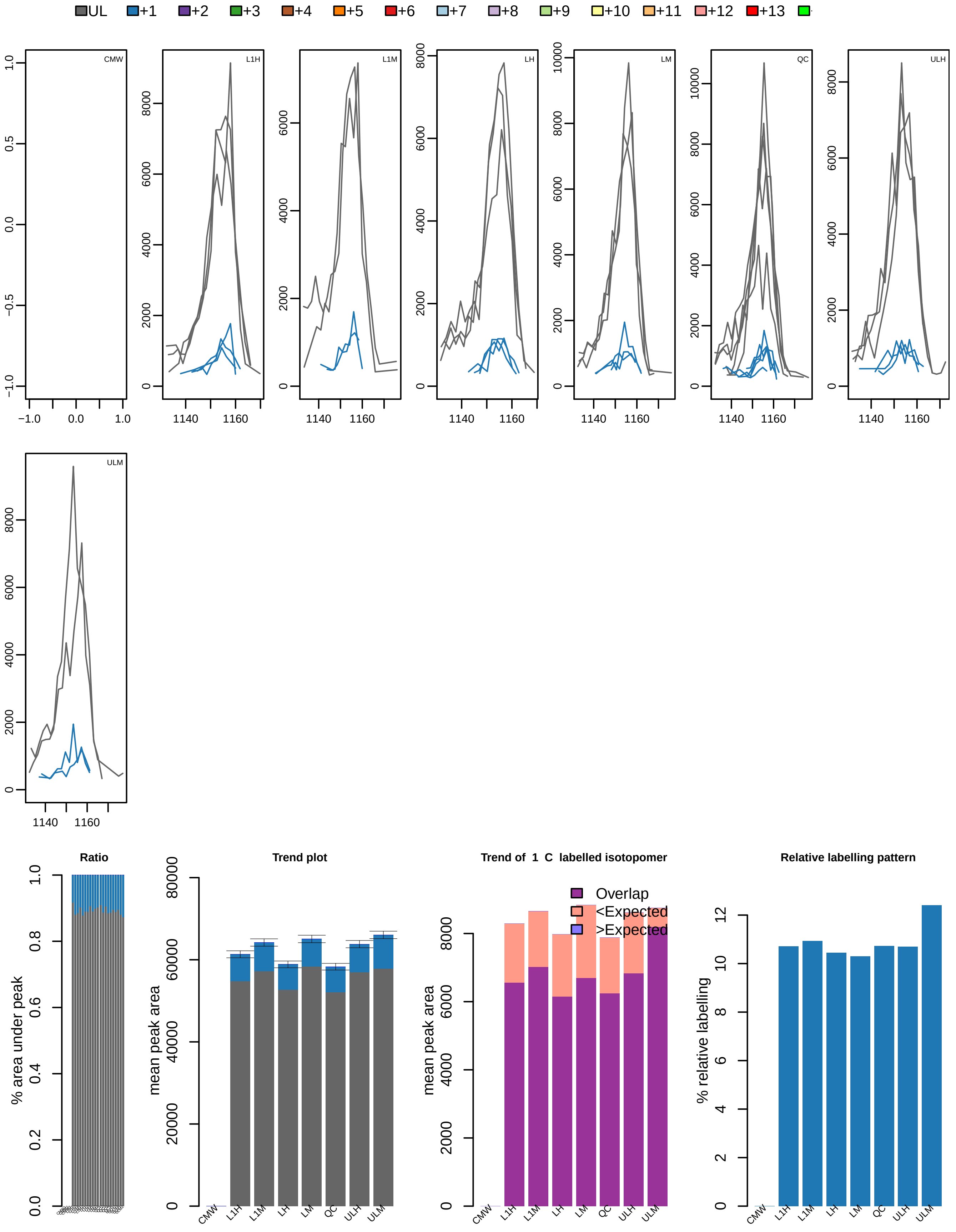

Val−Ser−His

Formula: $C_{14}H_{23}N_5O_5$ Mass: 341.17 Std.RT: 435.44204712 Ion: PC

G1

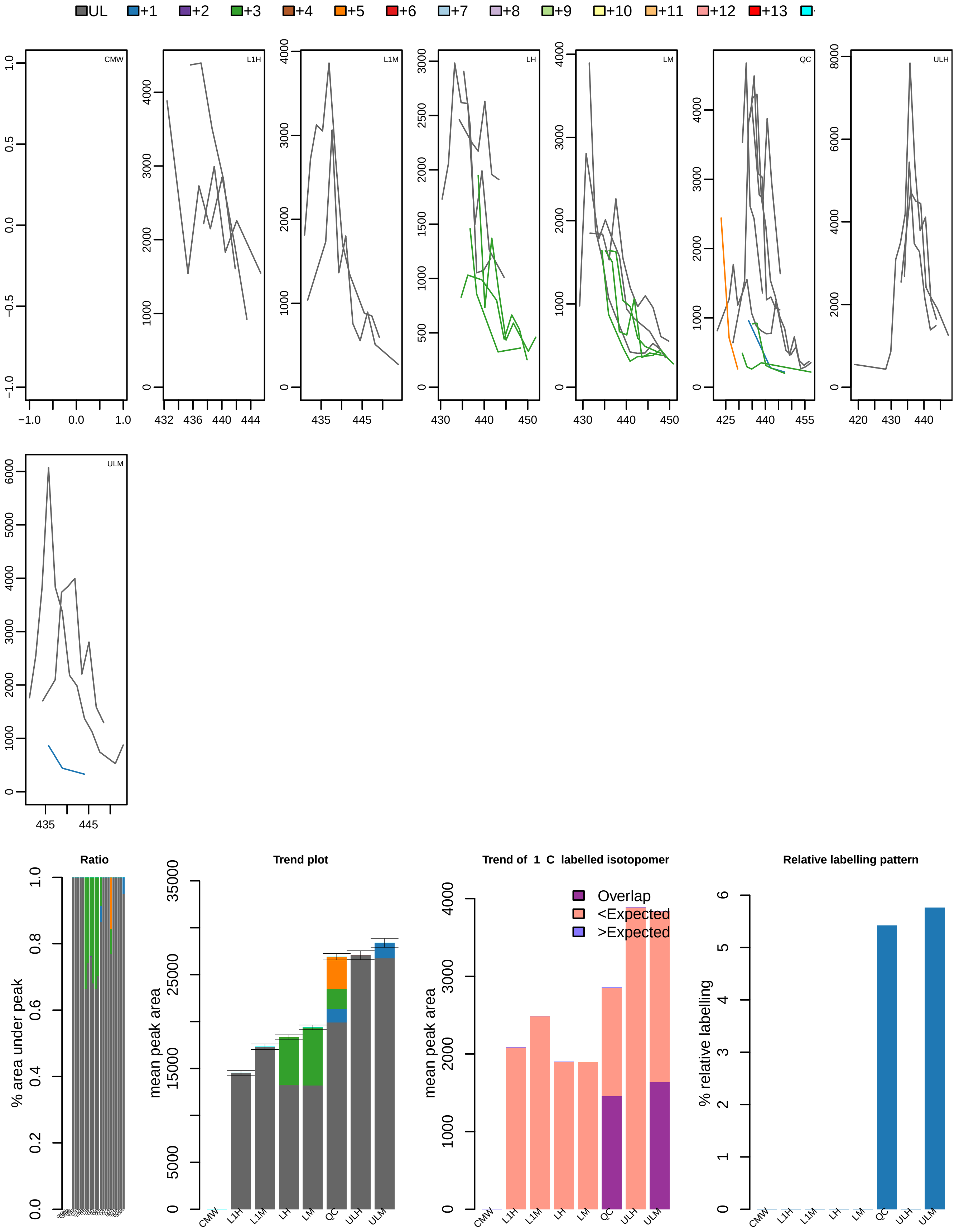

Lys−Val−Pro

Formula: C16H30N4O4 Mass: 342.227 Std.RT: 906.0108612 Ion: PC

G1

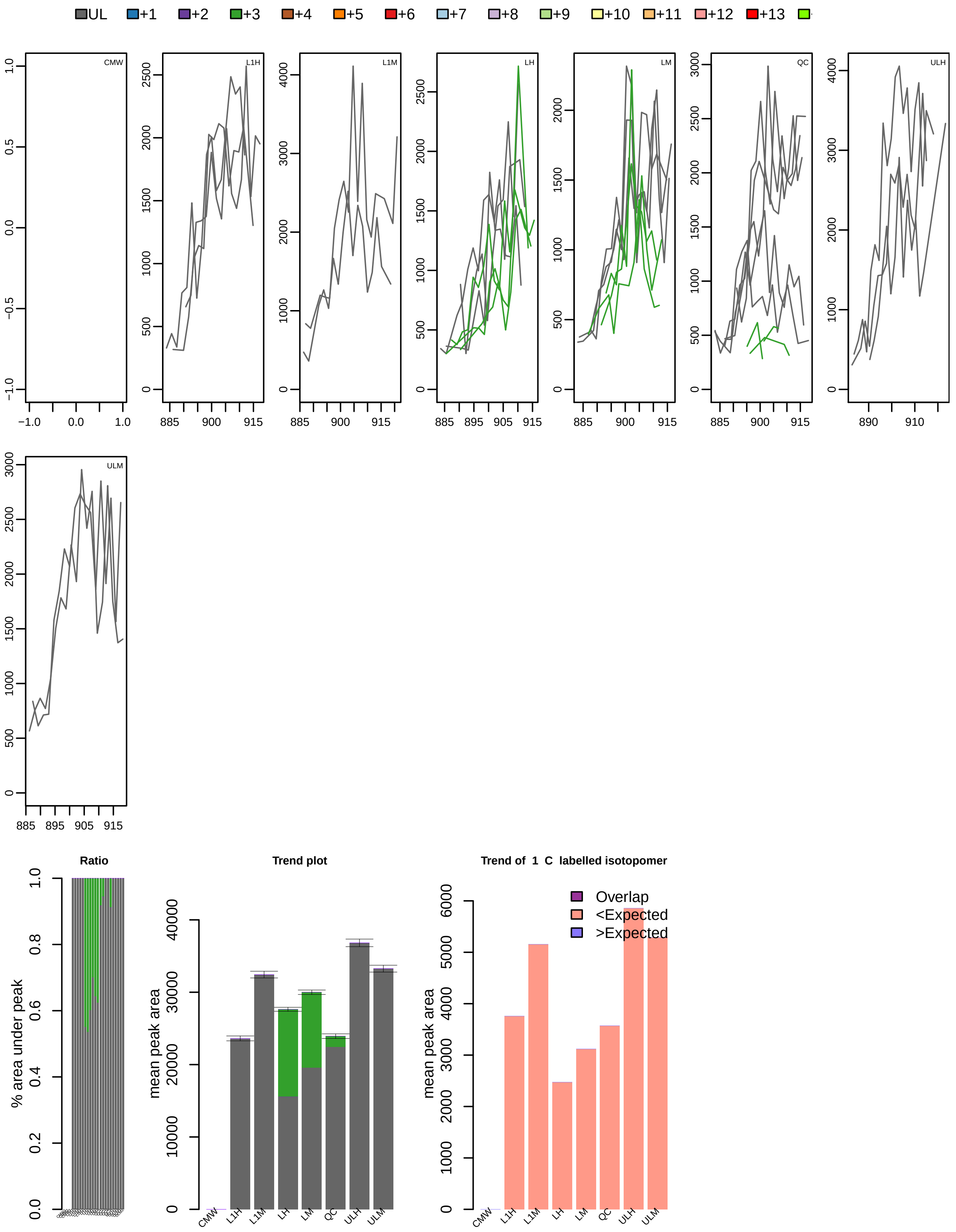

Leu−Asn−His

Formula: C16H26N6O5 Mass: 382.196 Std.RT: 920.4741174 Ion: PC

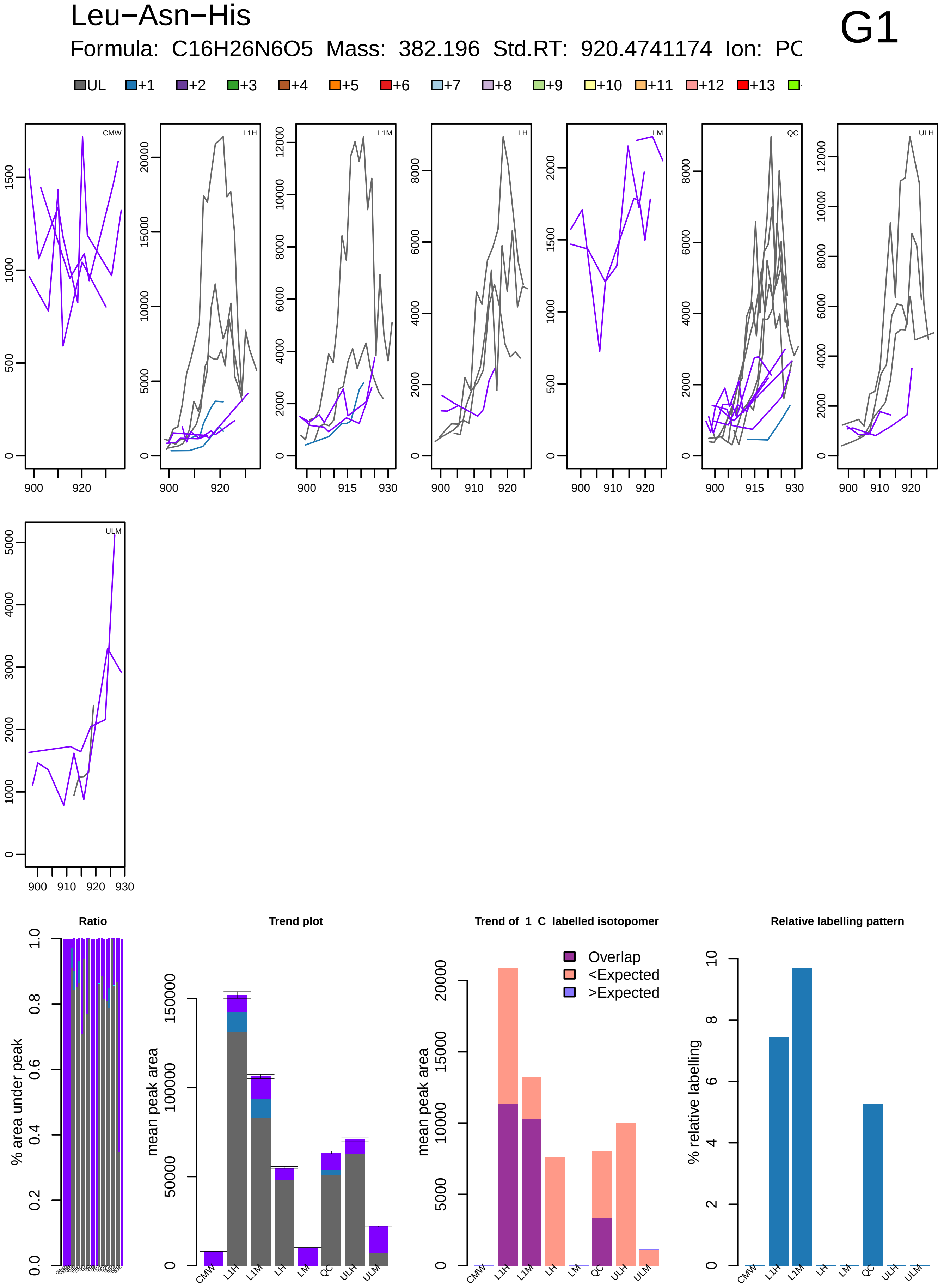

Ala−Gly−His

Formula: $C_{11}H_{17}N_5O_4$ Mass: 283.128 Std.RT: 764.165217 Ion: POS

G1

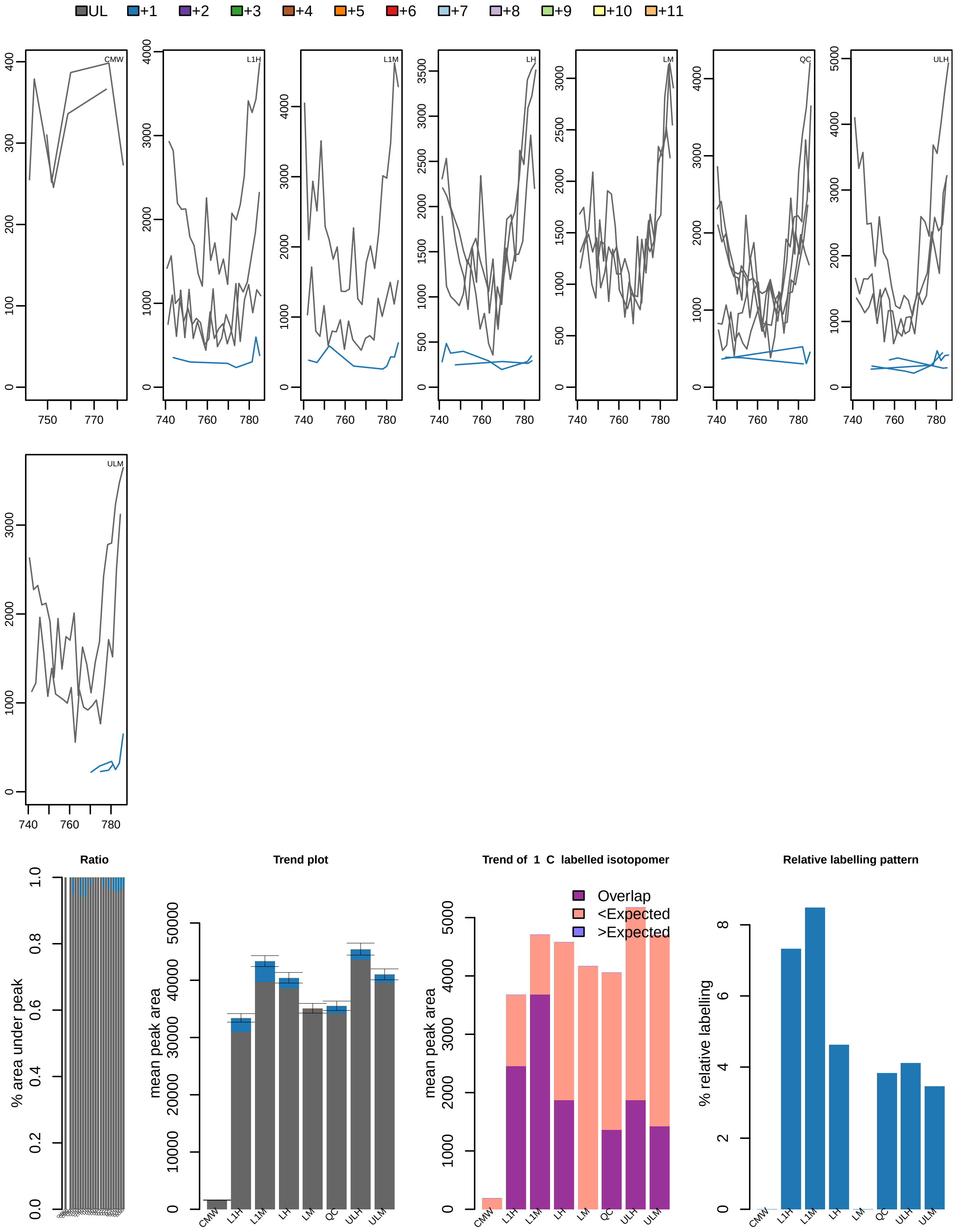

Val−Pro−Ser

Formula: C13H23N3O5 Mass: 301.164 Std.RT: 530.34462918 Ion: P

G1

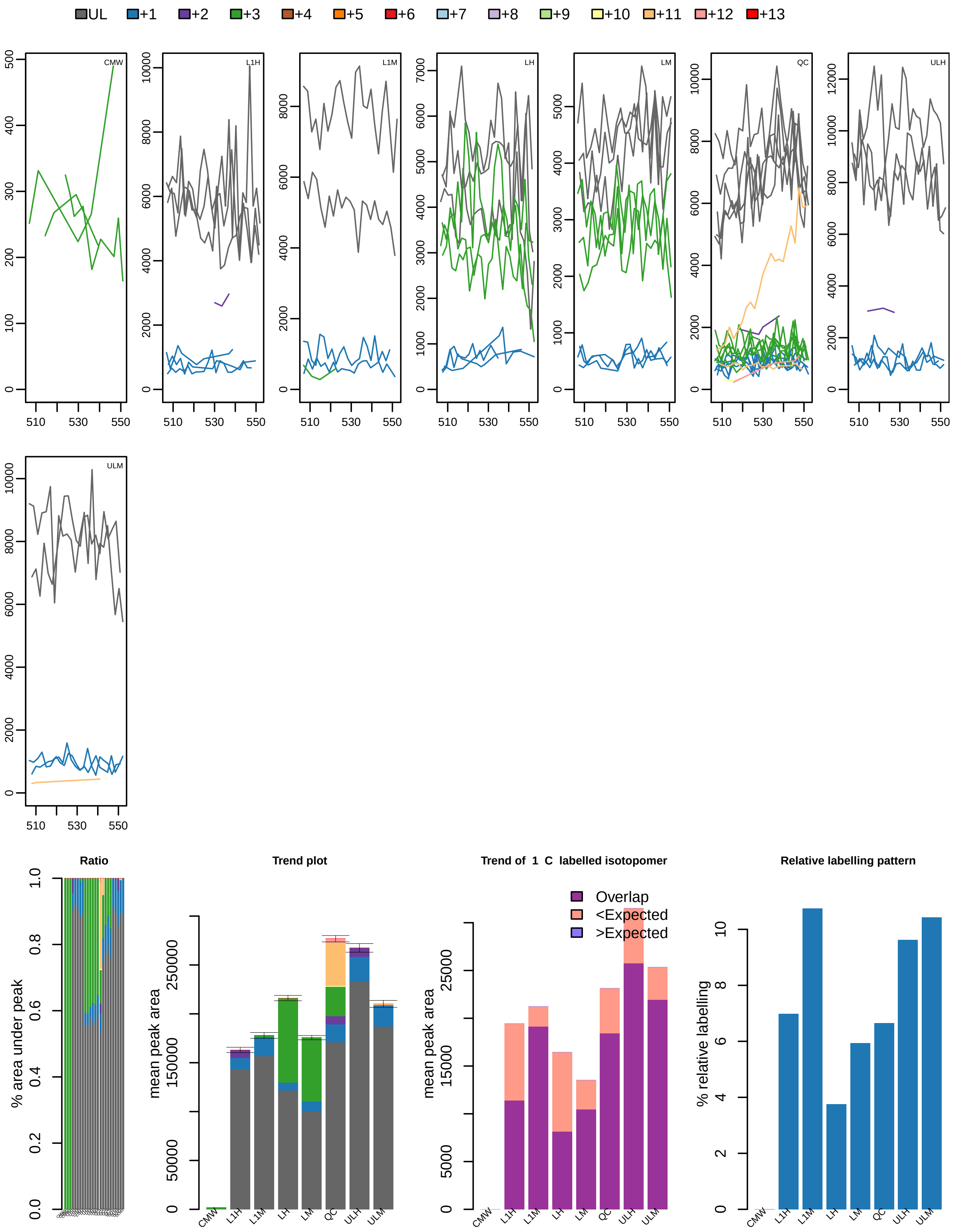

Leu−Asn−Ser

Formula: C13H24N4O6 Mass: 332.17 Std.RT: 1016.7339972 Ion: PC

G1

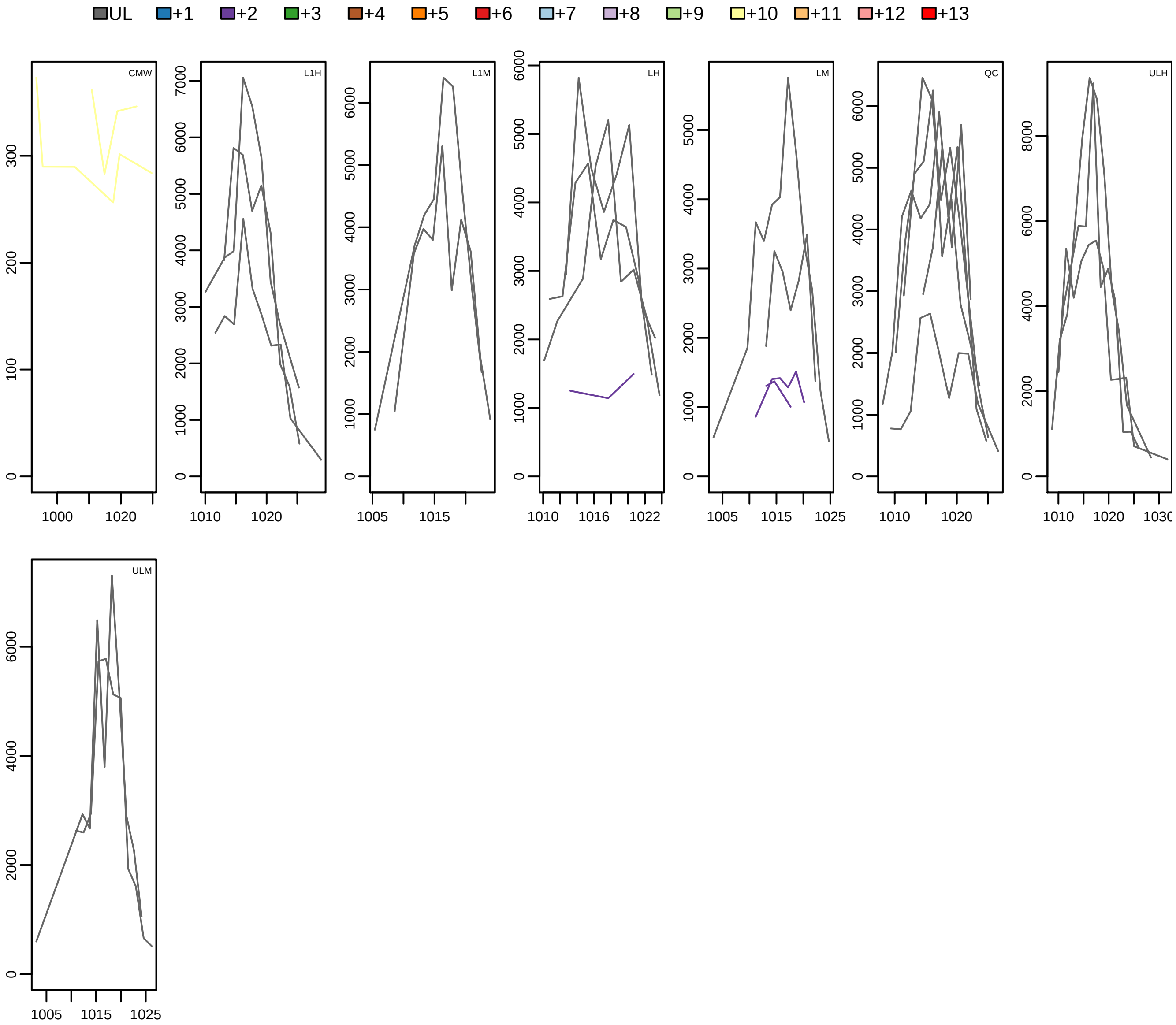

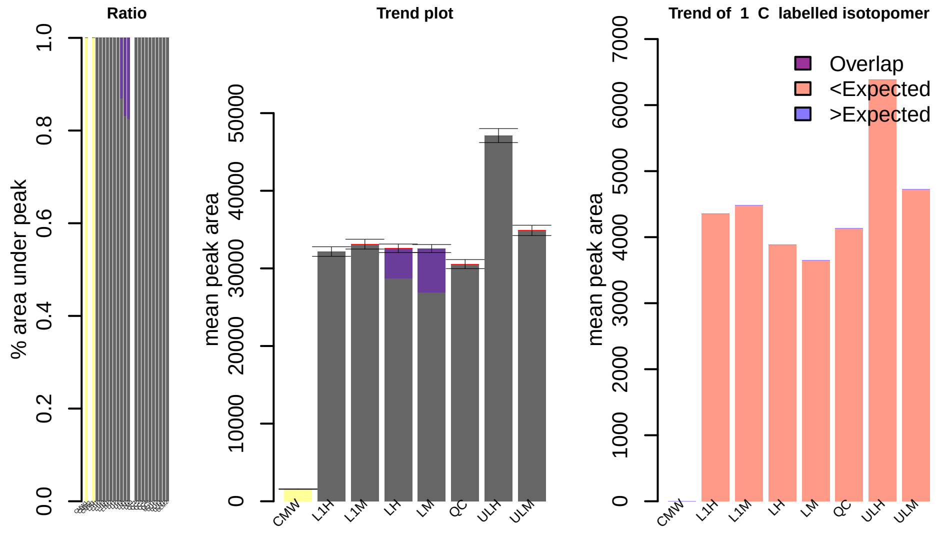

Leu−Met−Gly

Formula: C13H25N3O4S Mass: 319.157 Std.RT: 776.9662914 Ion: P

G1

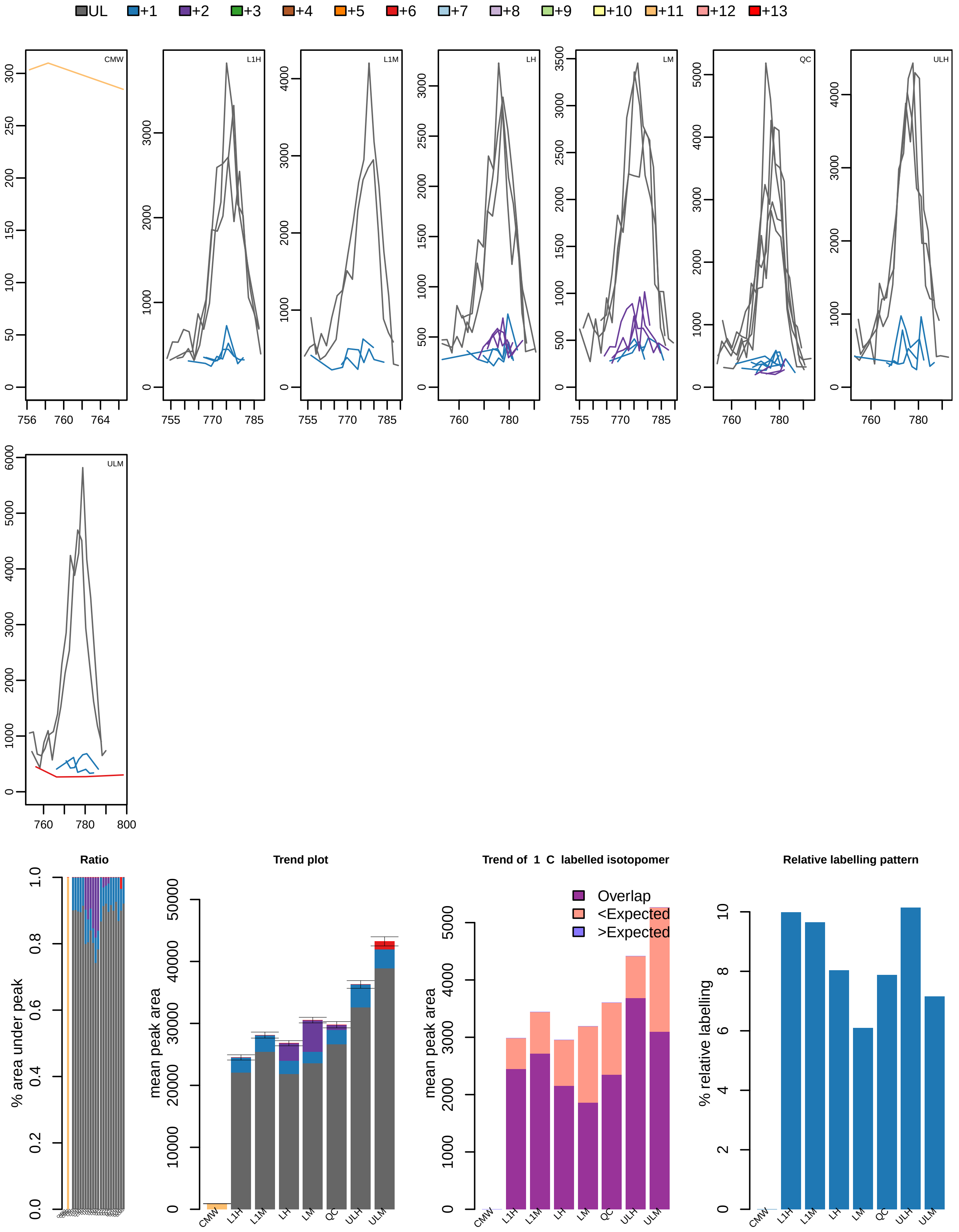

Leu−Ala−Gly

Formula: C11H21N3O4 Mass: 259.153 Std.RT: 432.8911932 Ion: PC

G1

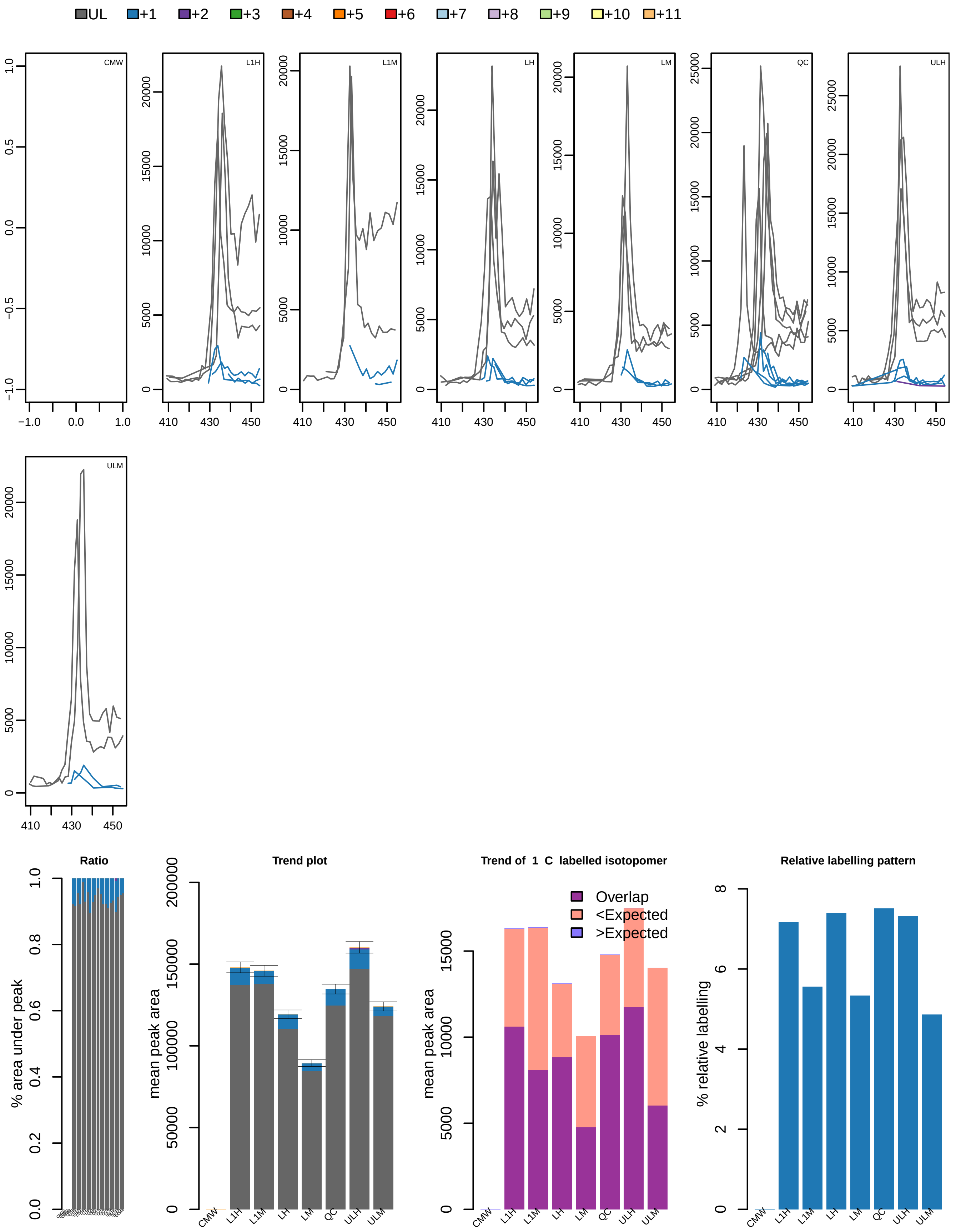

Leu−Ala−Gly

Formula: C11H21N3O4 Mass: 259.153 Std.RT: 432.8911932 Ion: PC

G2

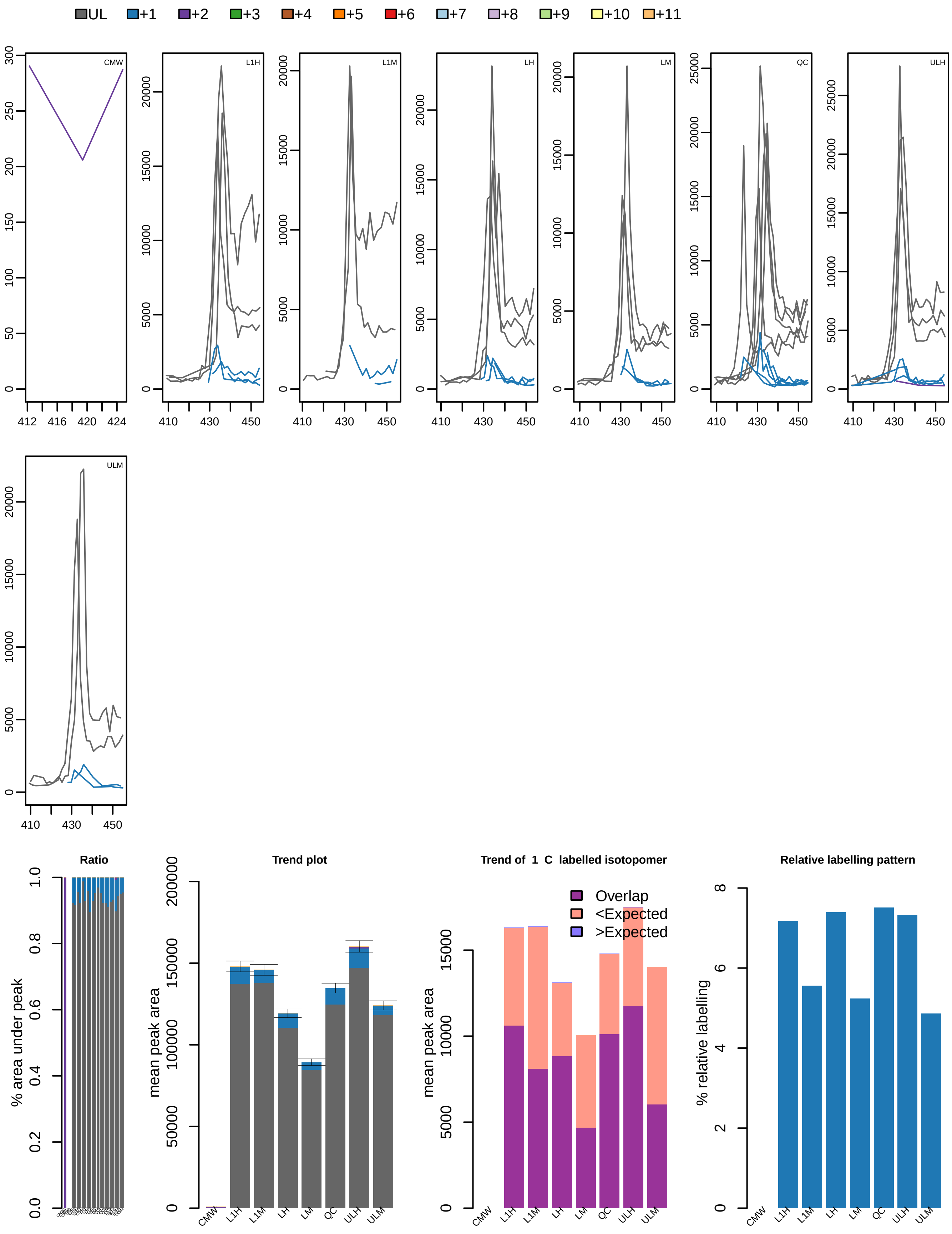

Leu−Asn−Gly

Formula: C12H22N4O5 Mass: 302.159 Std.RT: 680.1705786 Ion: PC

G1

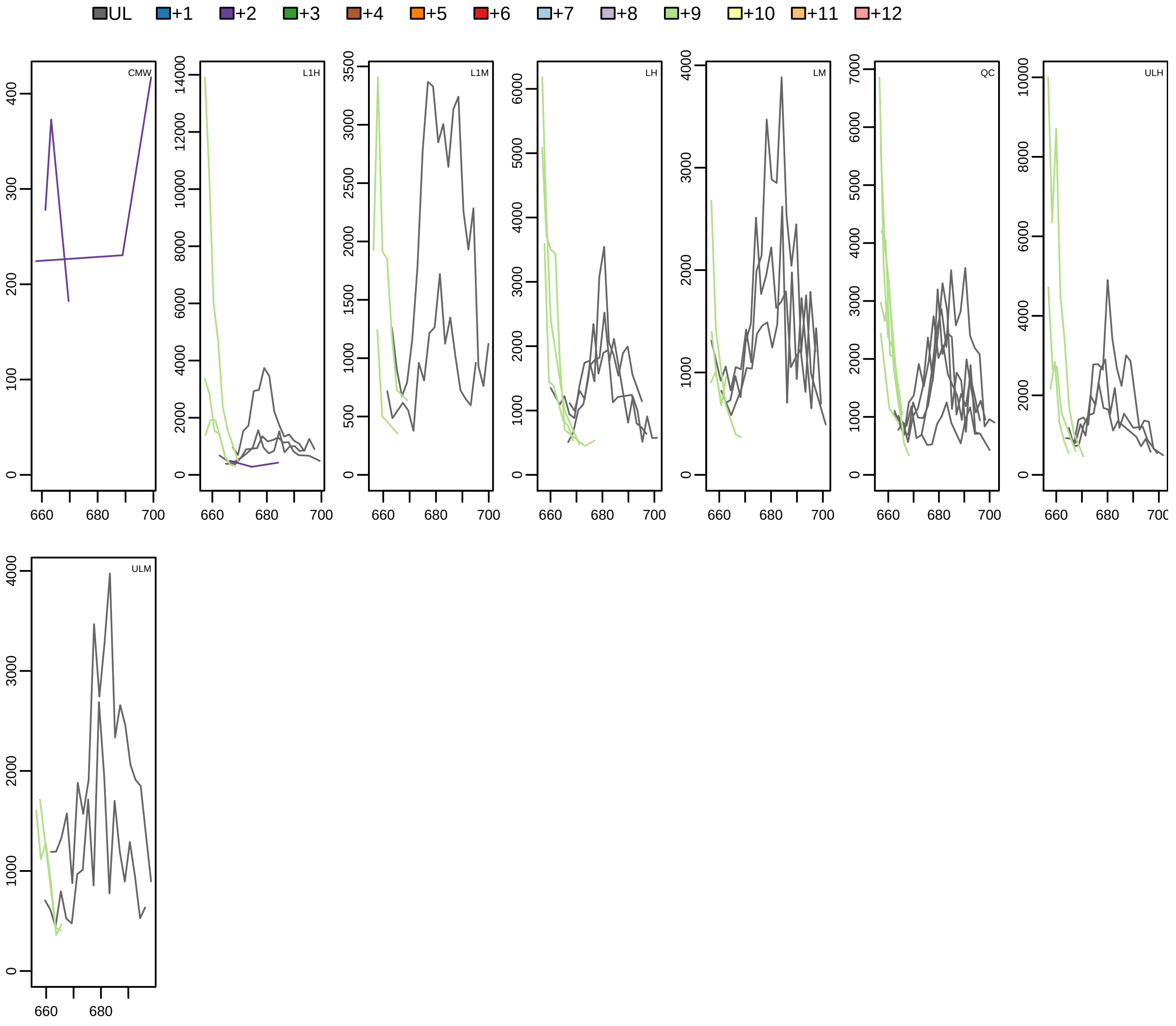

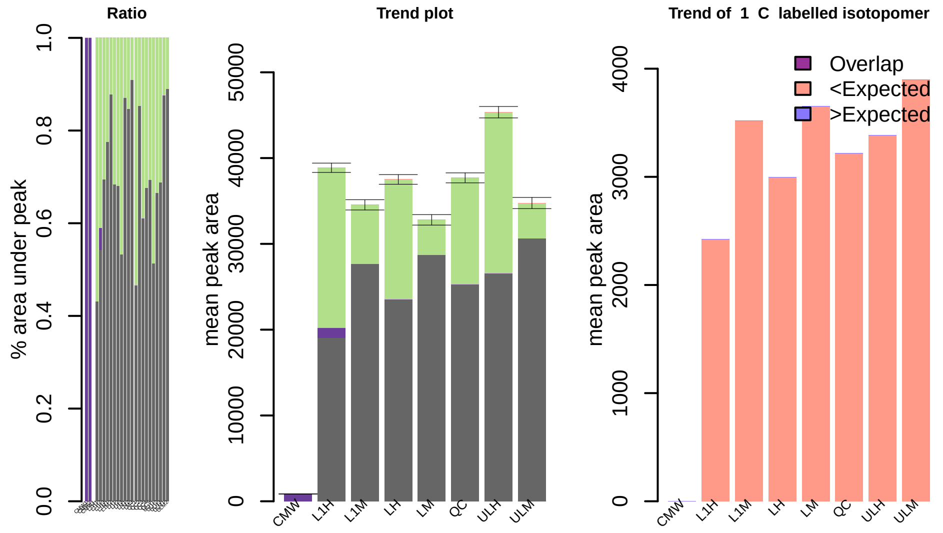

Met−Asp−Gln

Formula: $C_{14}H_{24}N_4O_7S$ Mass: 392.137 Std.RT: 625.8057138 Ion: P

G1

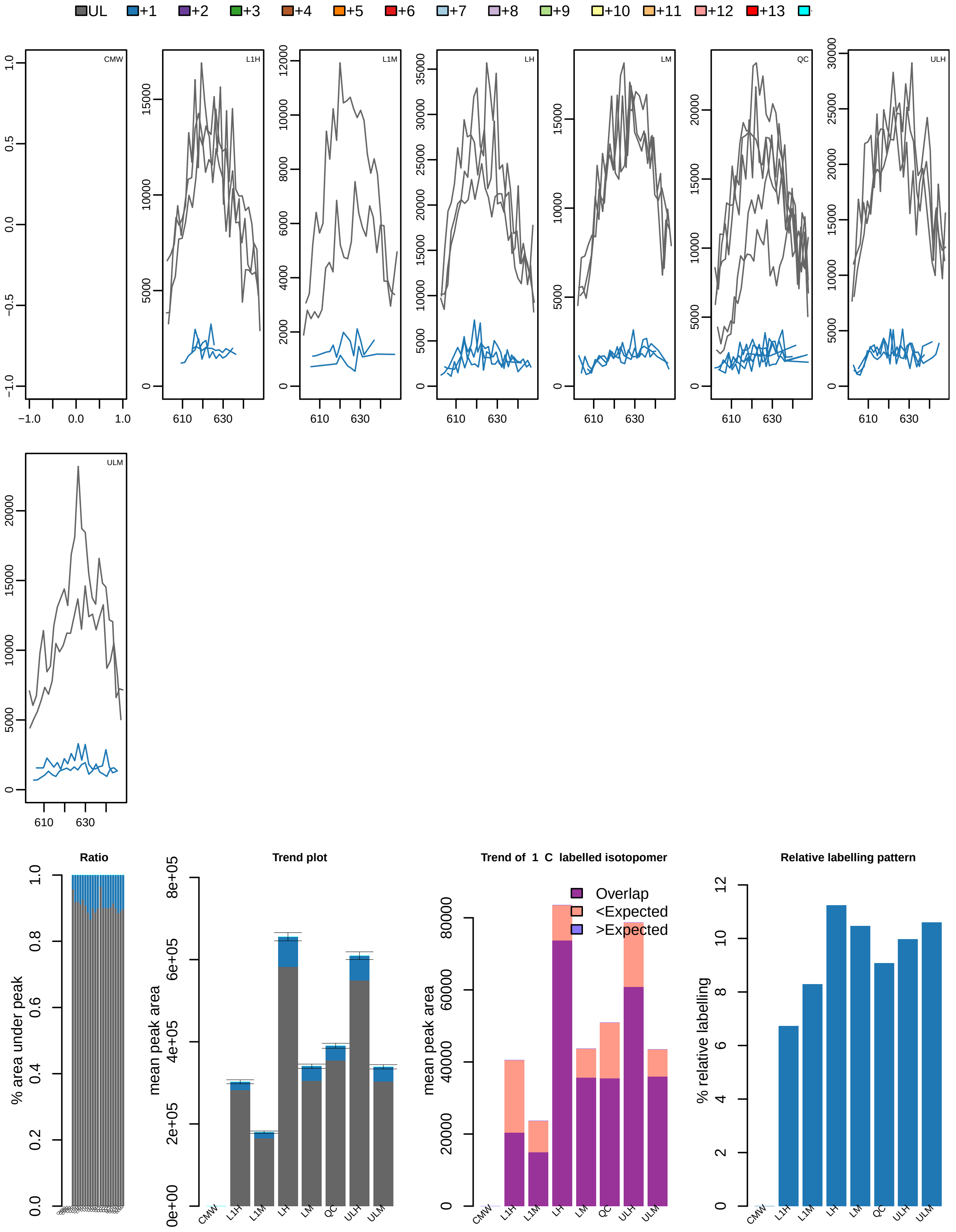

Ala−Gly−Pro

Formula: C10H17N3O4 Mass: 243.122 Std.RT: 801.7054242 Ion: PC

G1

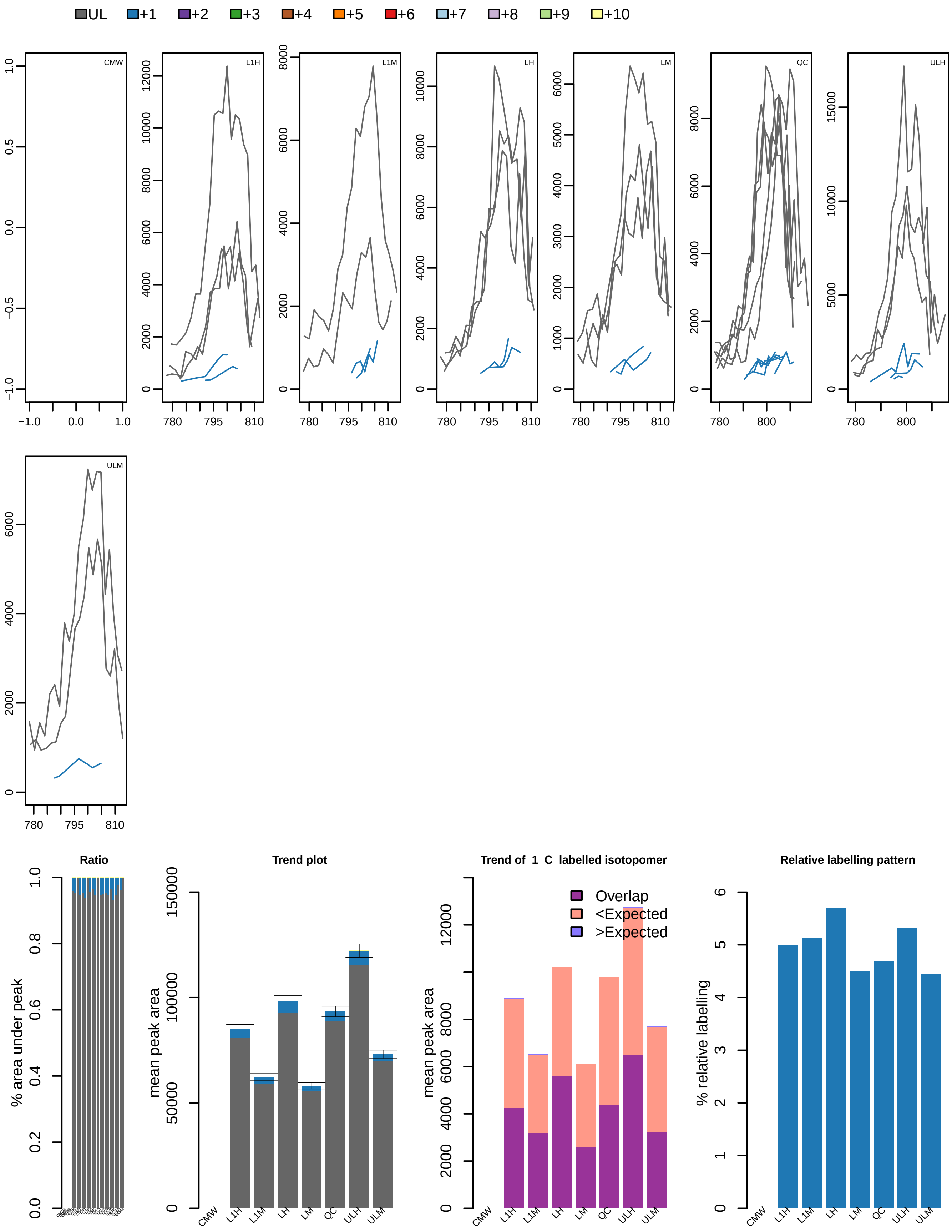

4−Aminobenzoate

Formula: C7H7NO2 Mass: 137.048 Std.RT: 754.8477414 Ion: POS

G1

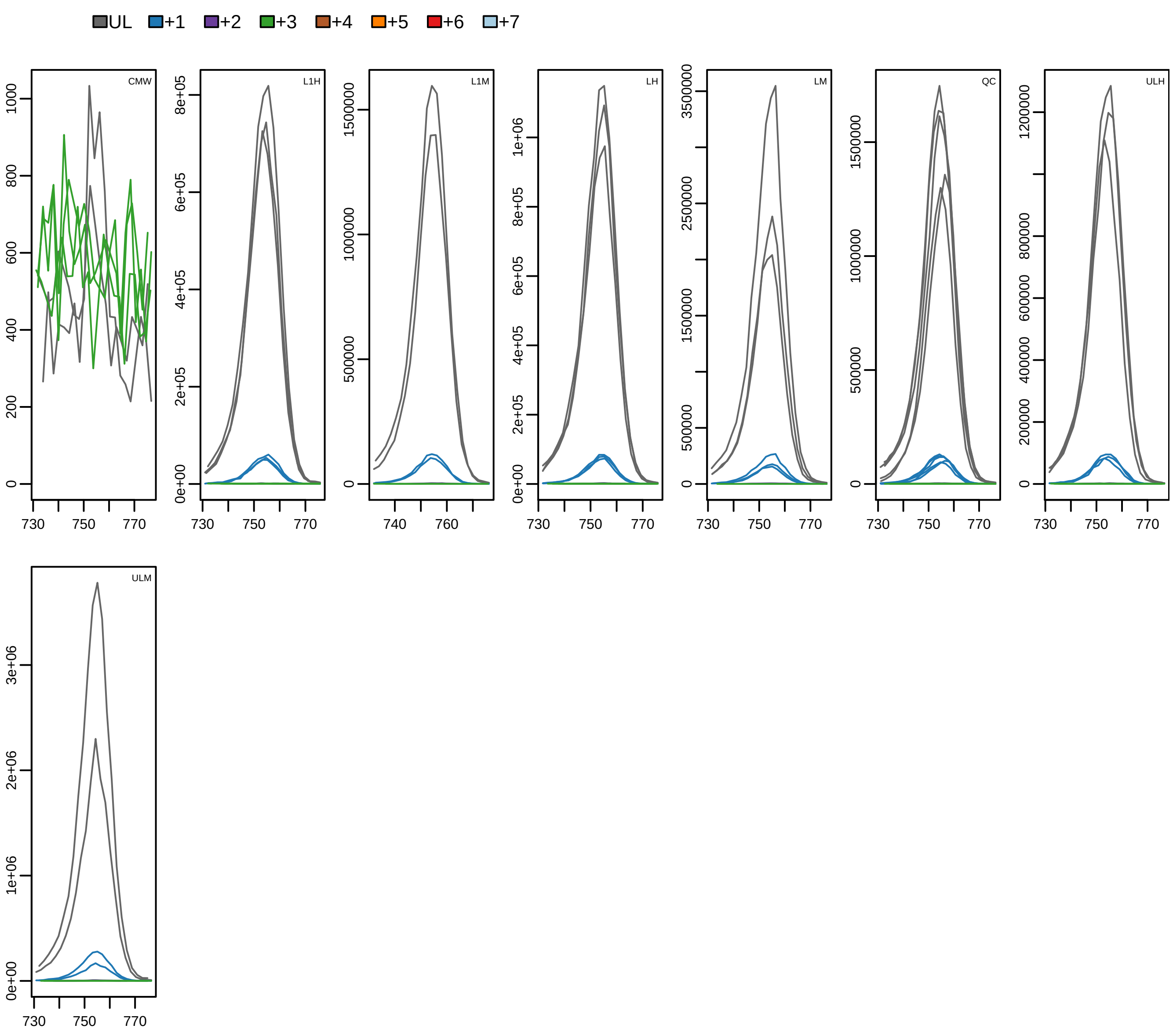

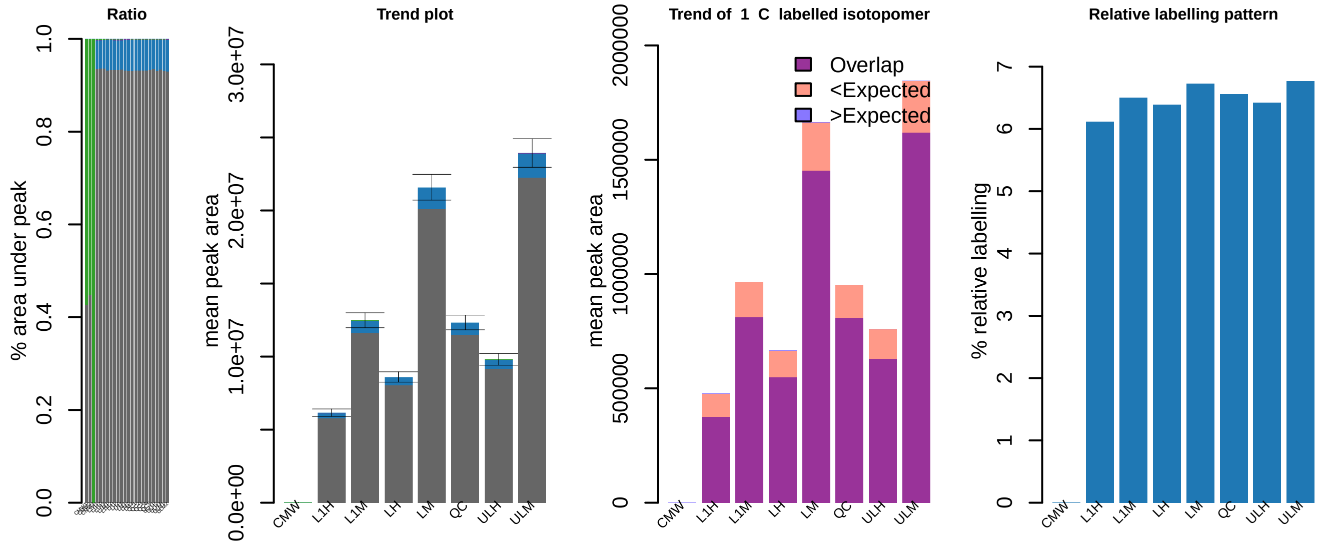

1−24:1−2−18:3−phosphatidylserine

Formula: C48H86NO10P Mass: 867.599 Std.RT: 205.20385632 Ion:

G1

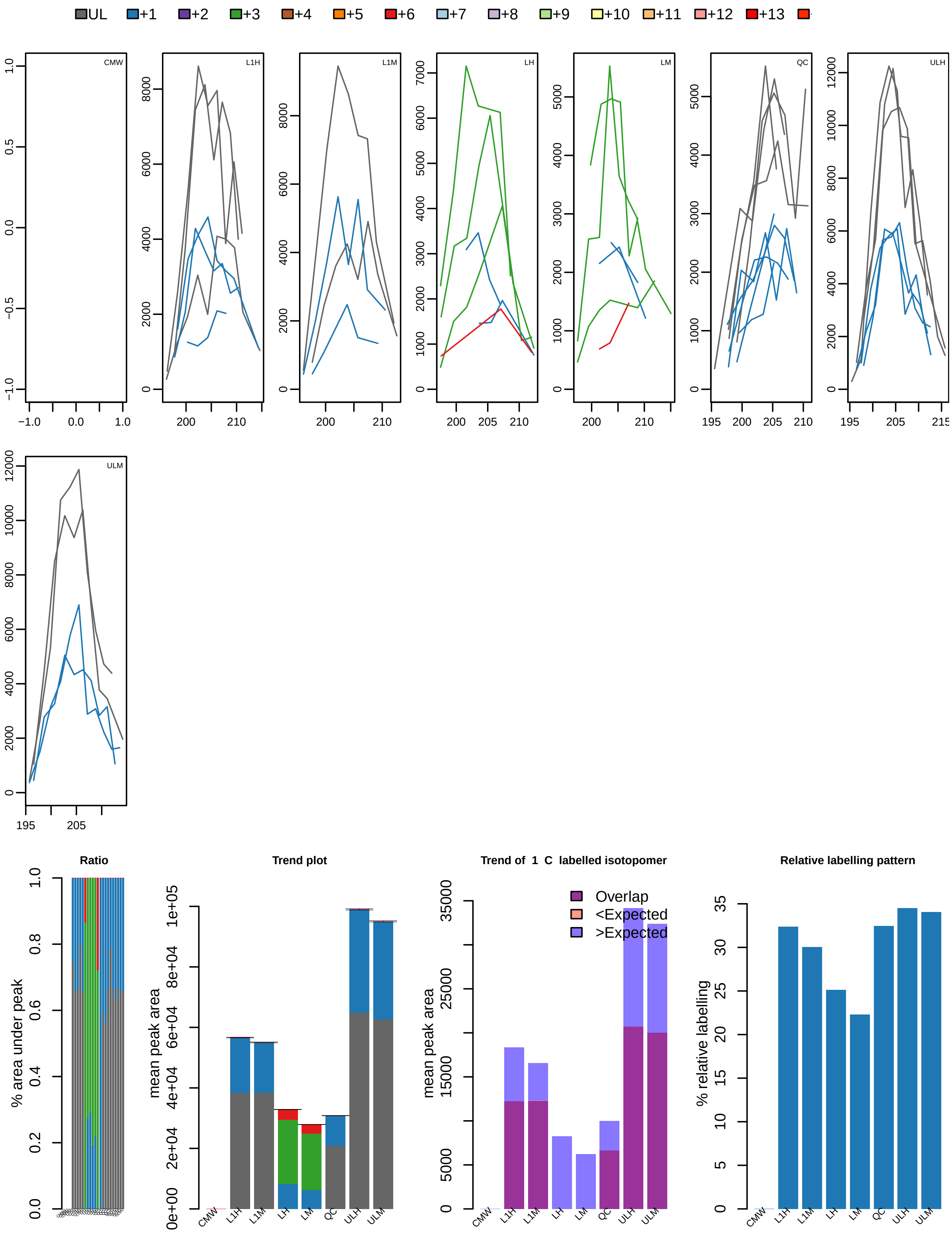

Calystegin B2

Formula: C7H13NO4 Mass: 175.084 Std.RT: 648.7073718 Ion: POS

G1

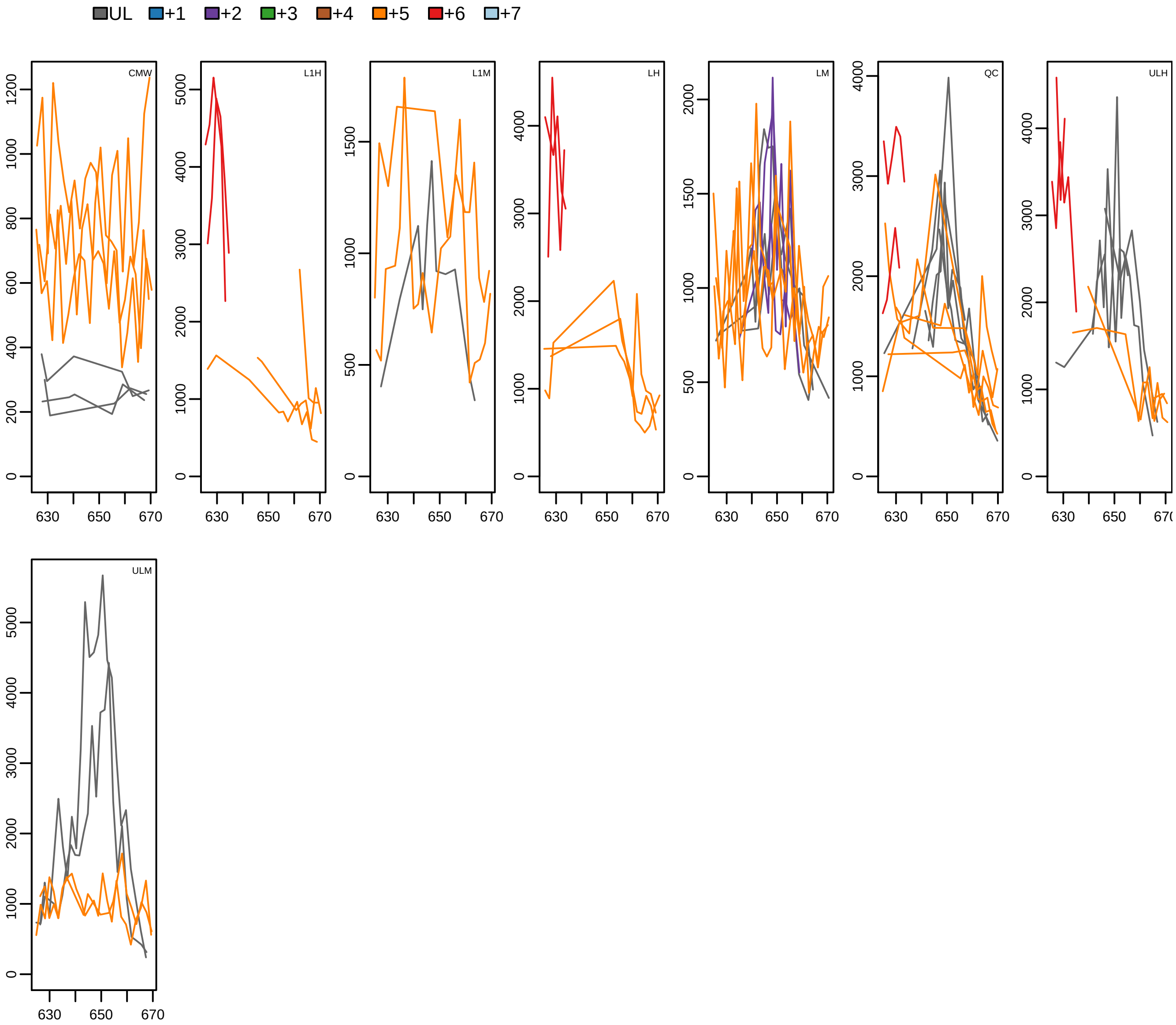

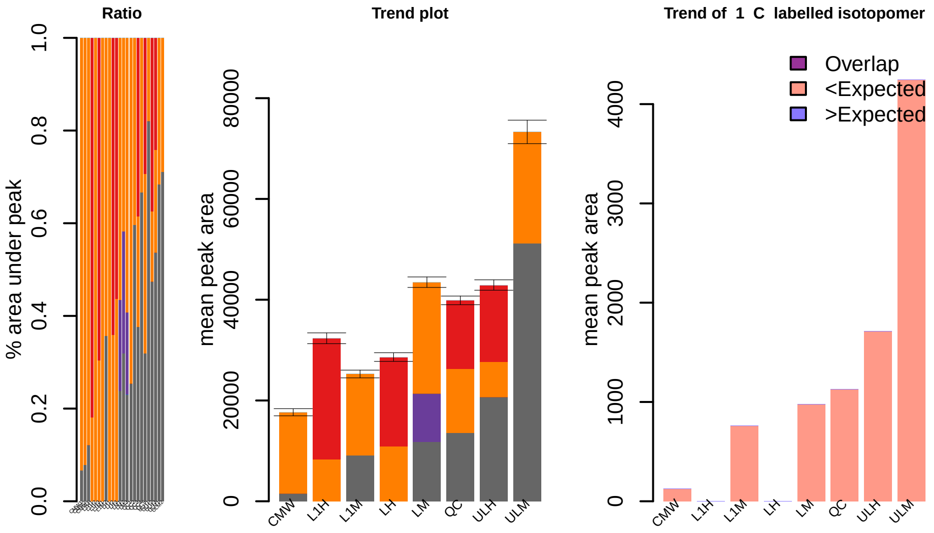

gamma−L−Glutamyl−L−cysteinyl−beta−alanine

Formula: C11H19N3O6S Mass: 321.099 Std.RT: 648.2151402 Ion: P

G1

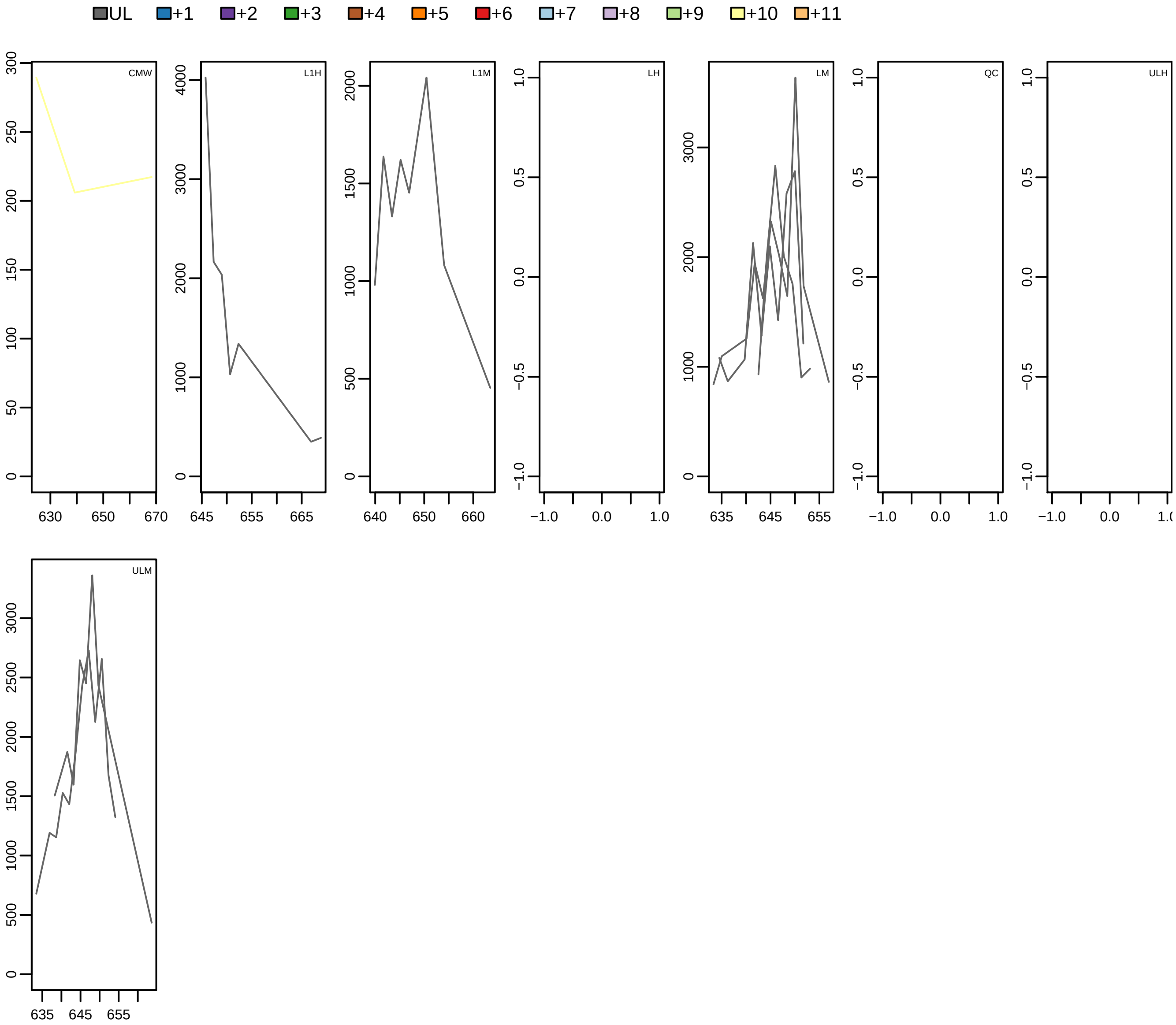

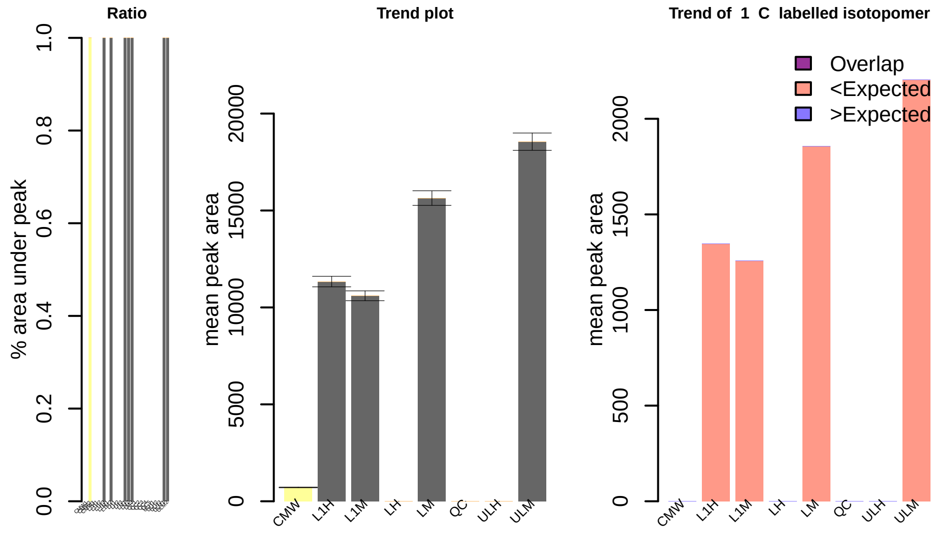

1−20:0−2−18:1−phosphatidylserine

Formula: C44H84NO10P Mass: 817.583 Std.RT: 206.1432858 Ion: P

G1

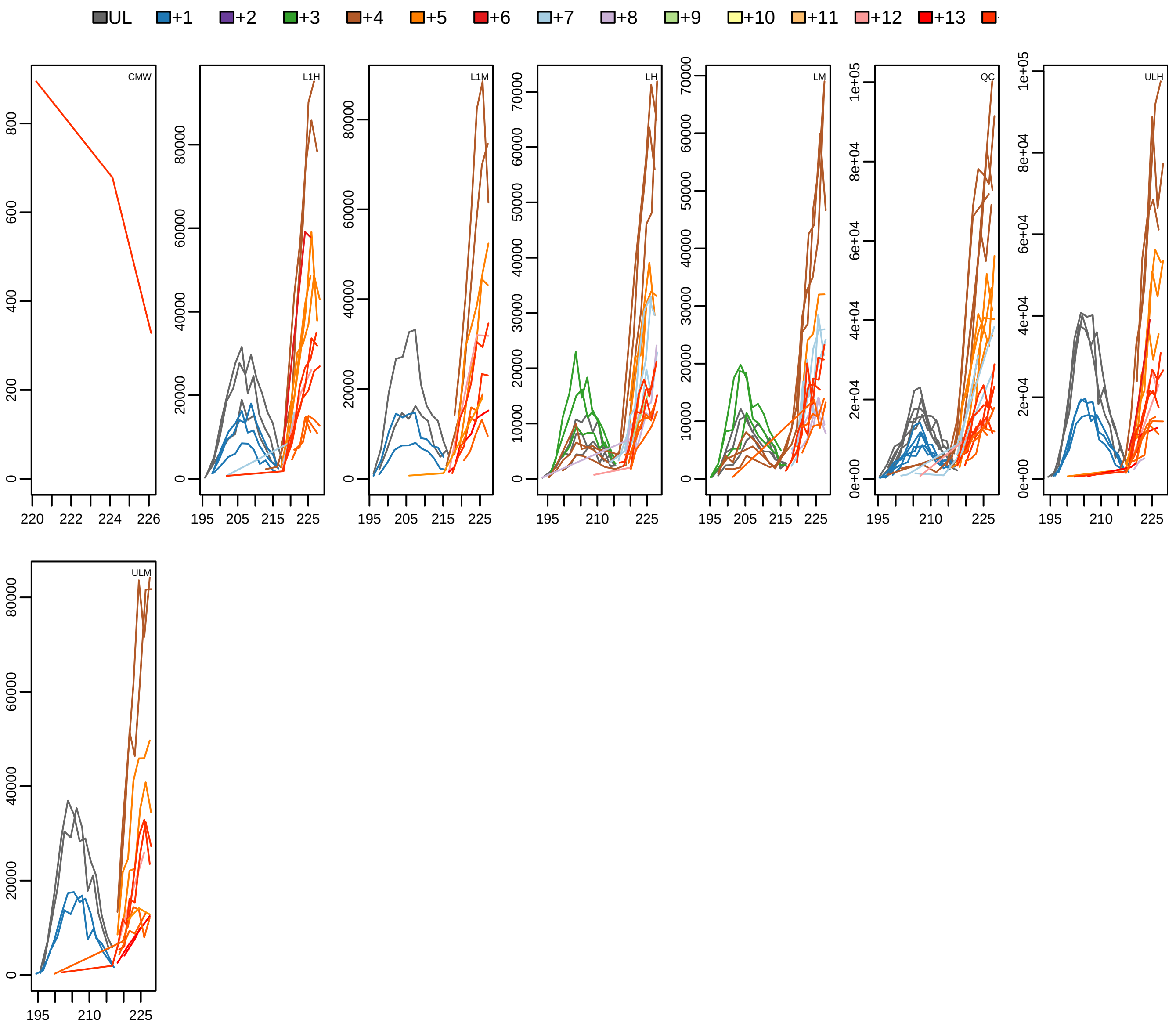

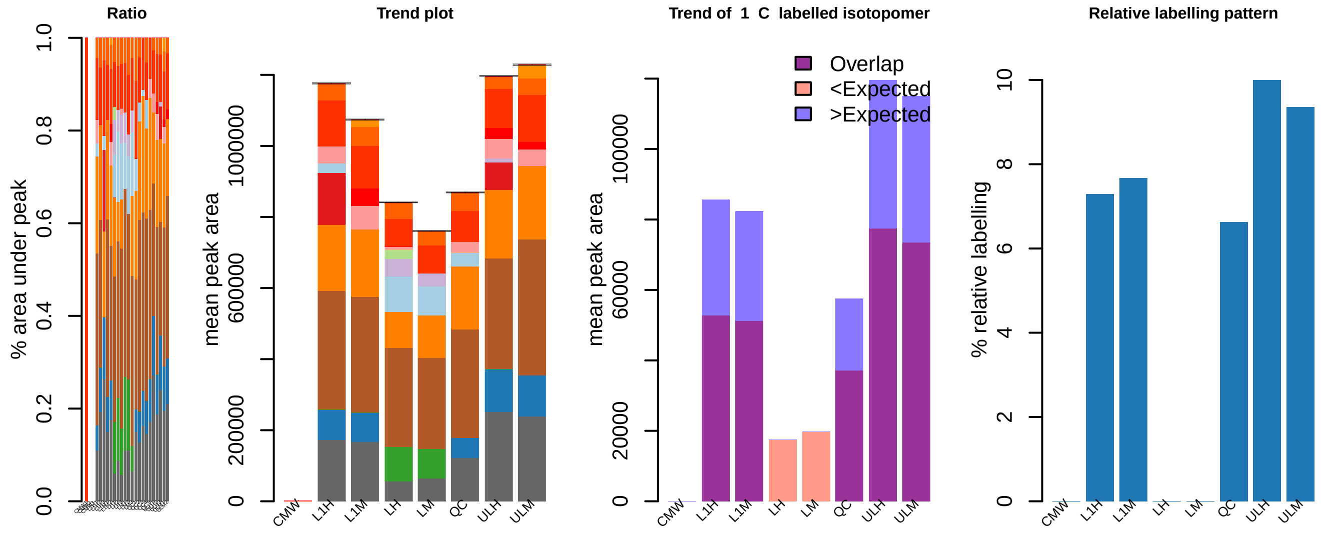

dCMP−choline

Formula: C14H26N4O10P2 Mass: 472.112 Std.RT: 942.8694312 Ion:

G1

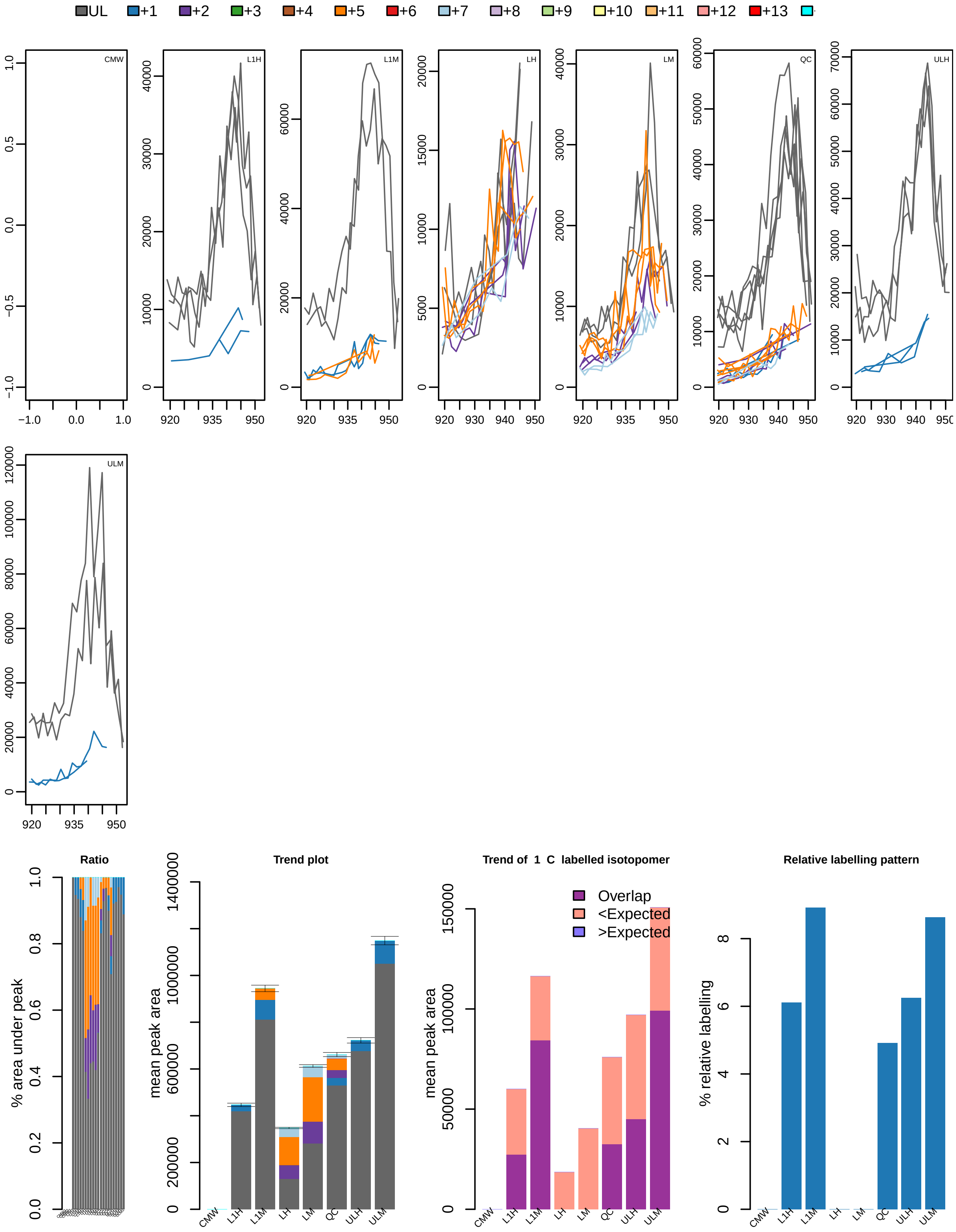

1−24:0−2−18:2−phosphatidylserine

Formula: C48H90NO10P Mass: 871.63 Std.RT: 205.38295272 Ion: P

G1

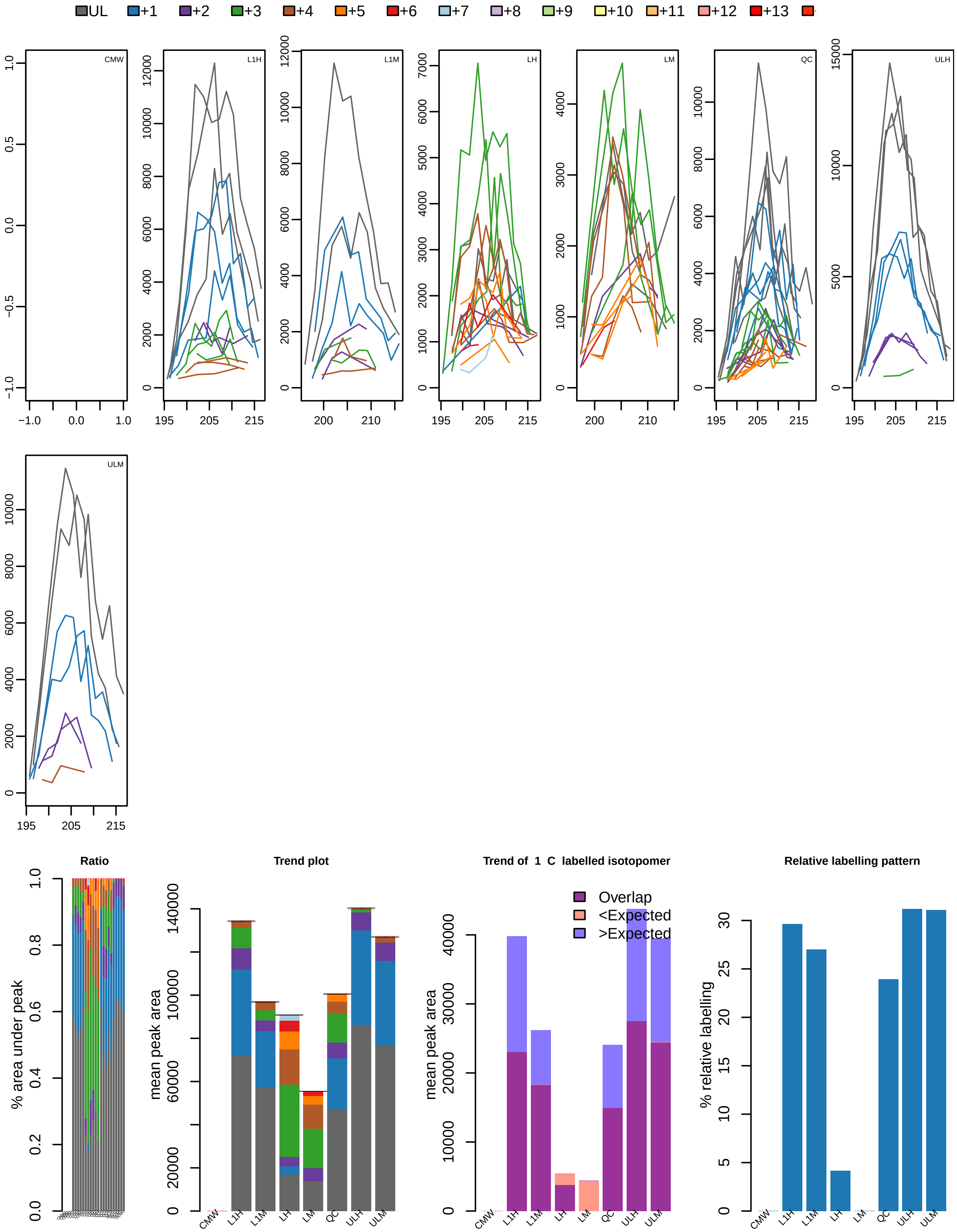

1−22:0−2−18:2−phosphatidylserine

Formula: C46H86NO10P Mass: 843.599 Std.RT: 207.37057062 Ion:

G1

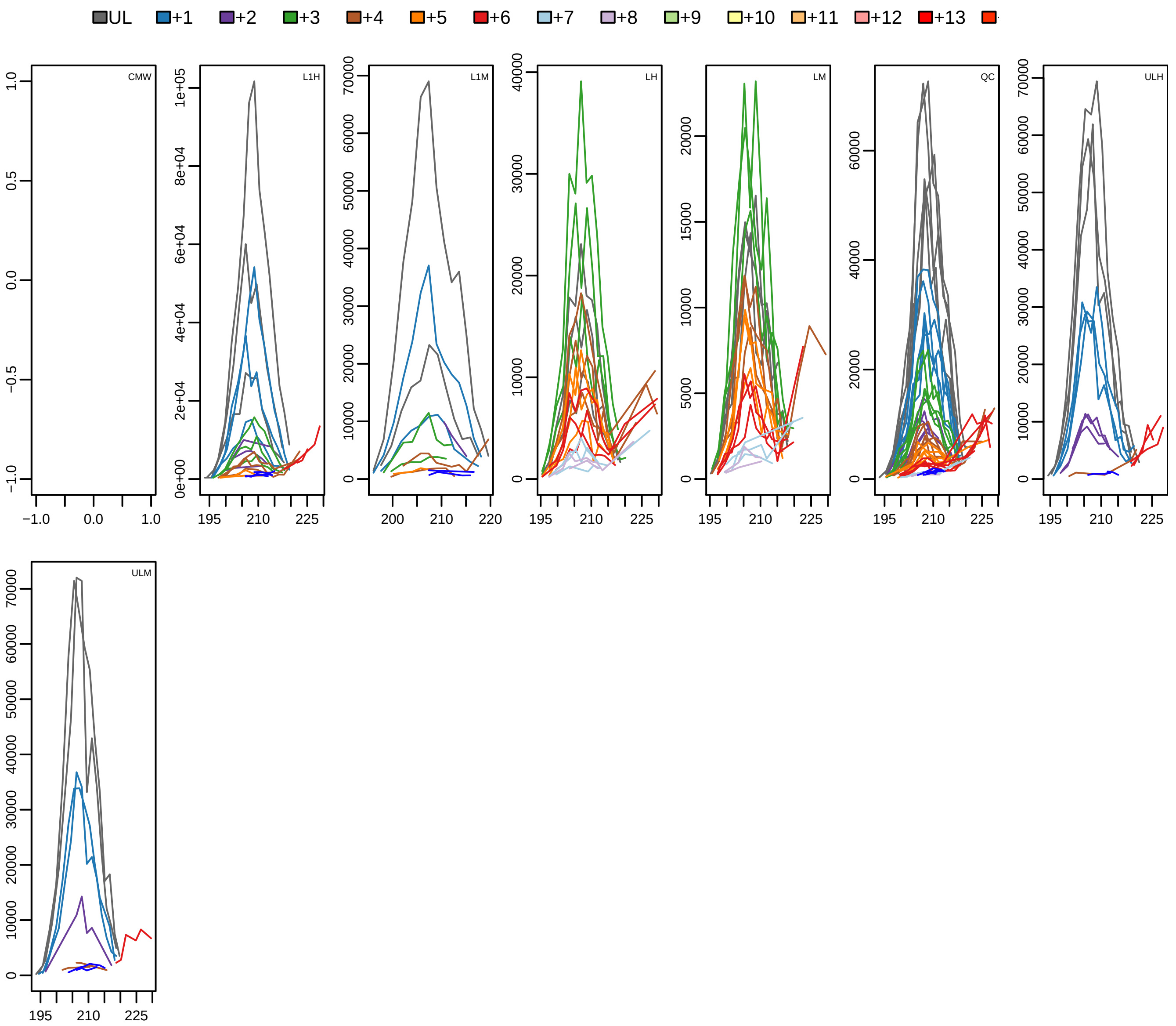

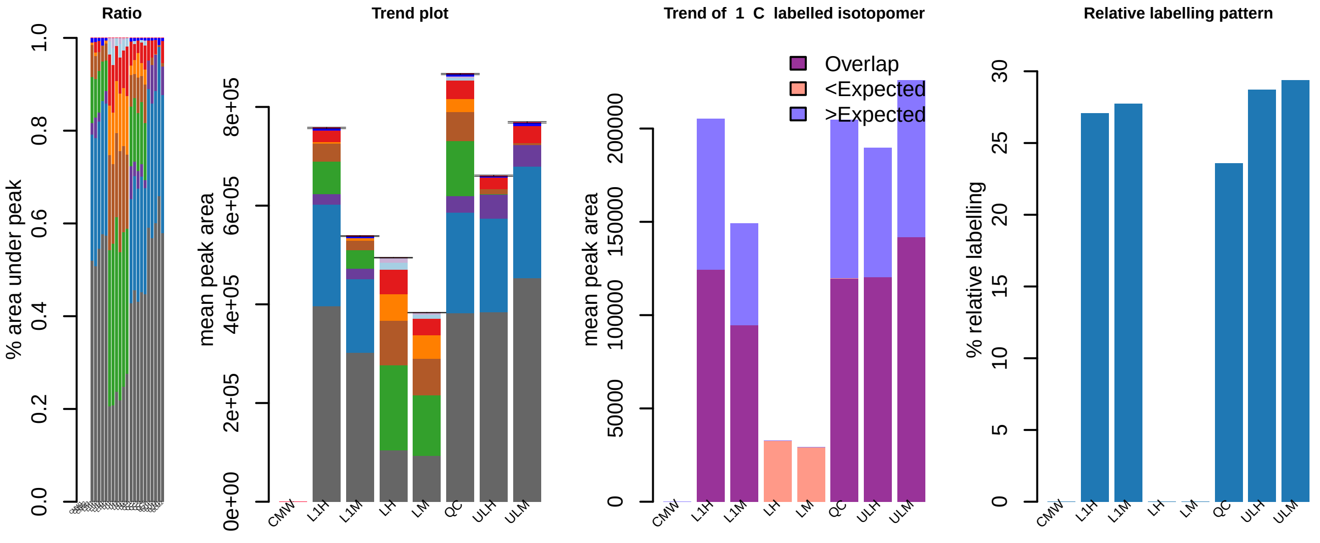

5−chloro−2−methyl−maleylacetate

Formula: C7H7ClO5 Mass: 205.998 Std.RT: 1036.054077 Ion: POS

G1

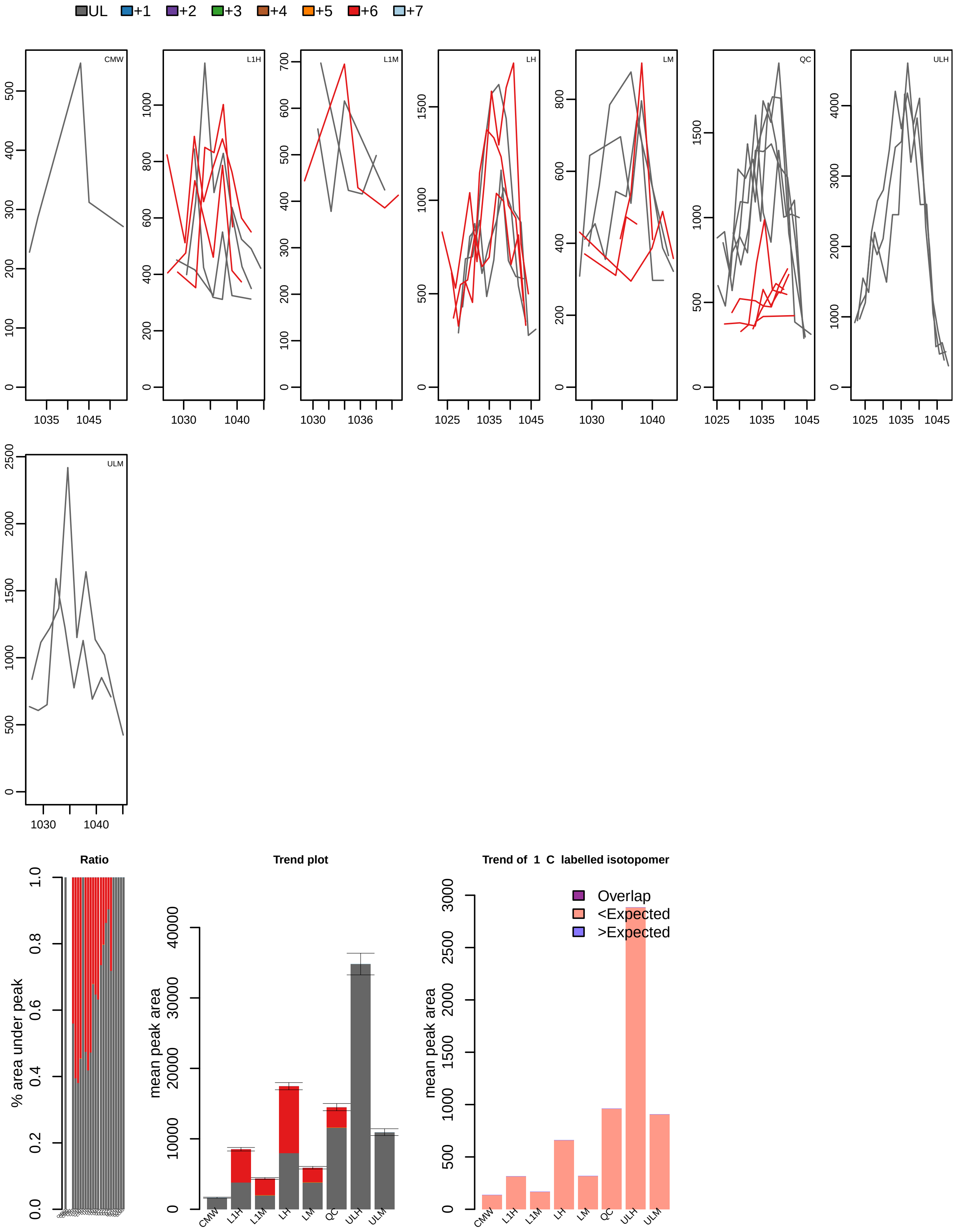

N4−(Acetyl−beta−D−glucosaminyl)asparagine

Formula: C12H21N3O8 Mass: 335.133 Std.RT: 1027.711908 Ion: PC

G1

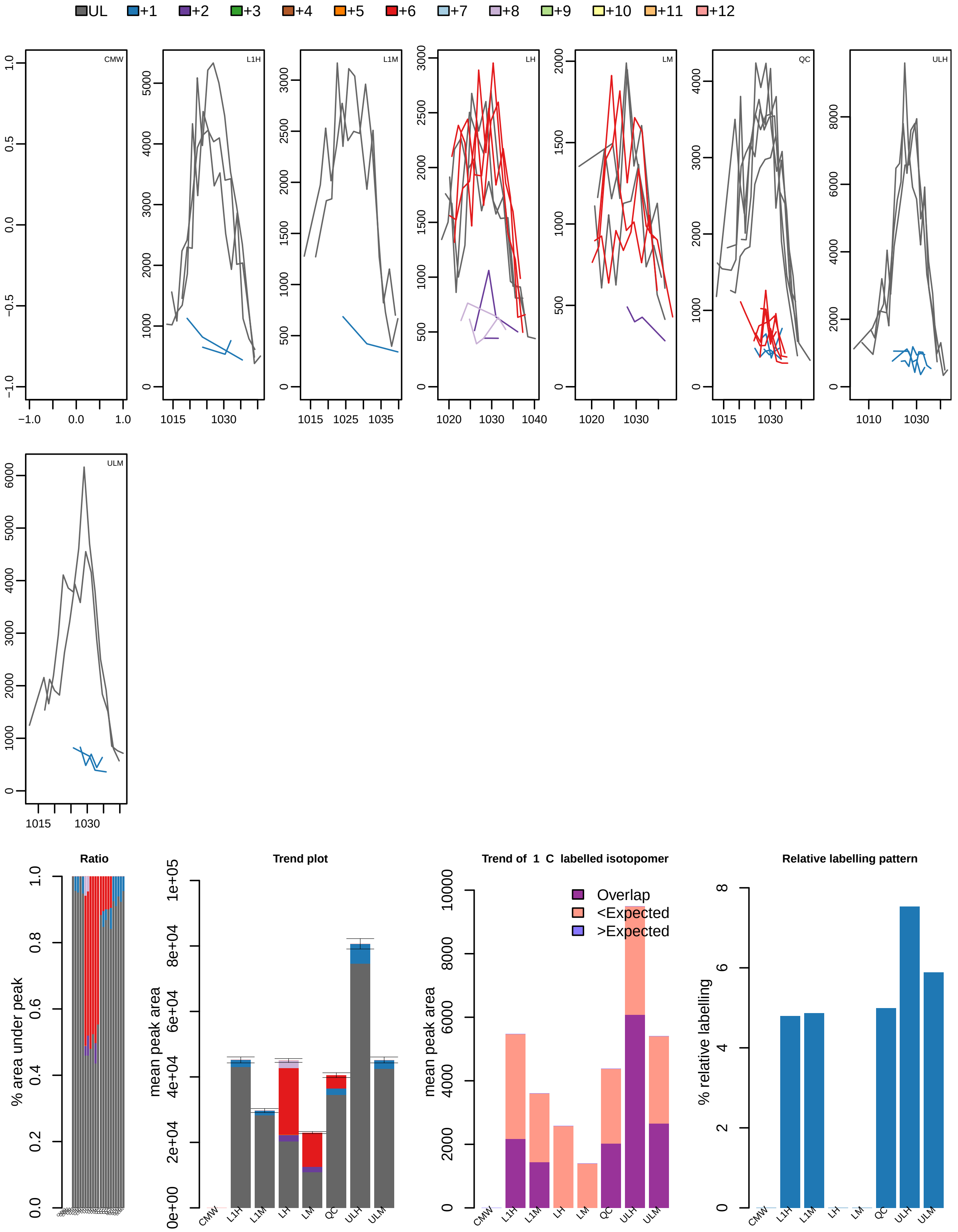

1−22:1−2−18:3−phosphatidylserine

Formula: C46H82NO10P Mass: 839.568 Std.RT: 207.1348572 Ion: P

G1

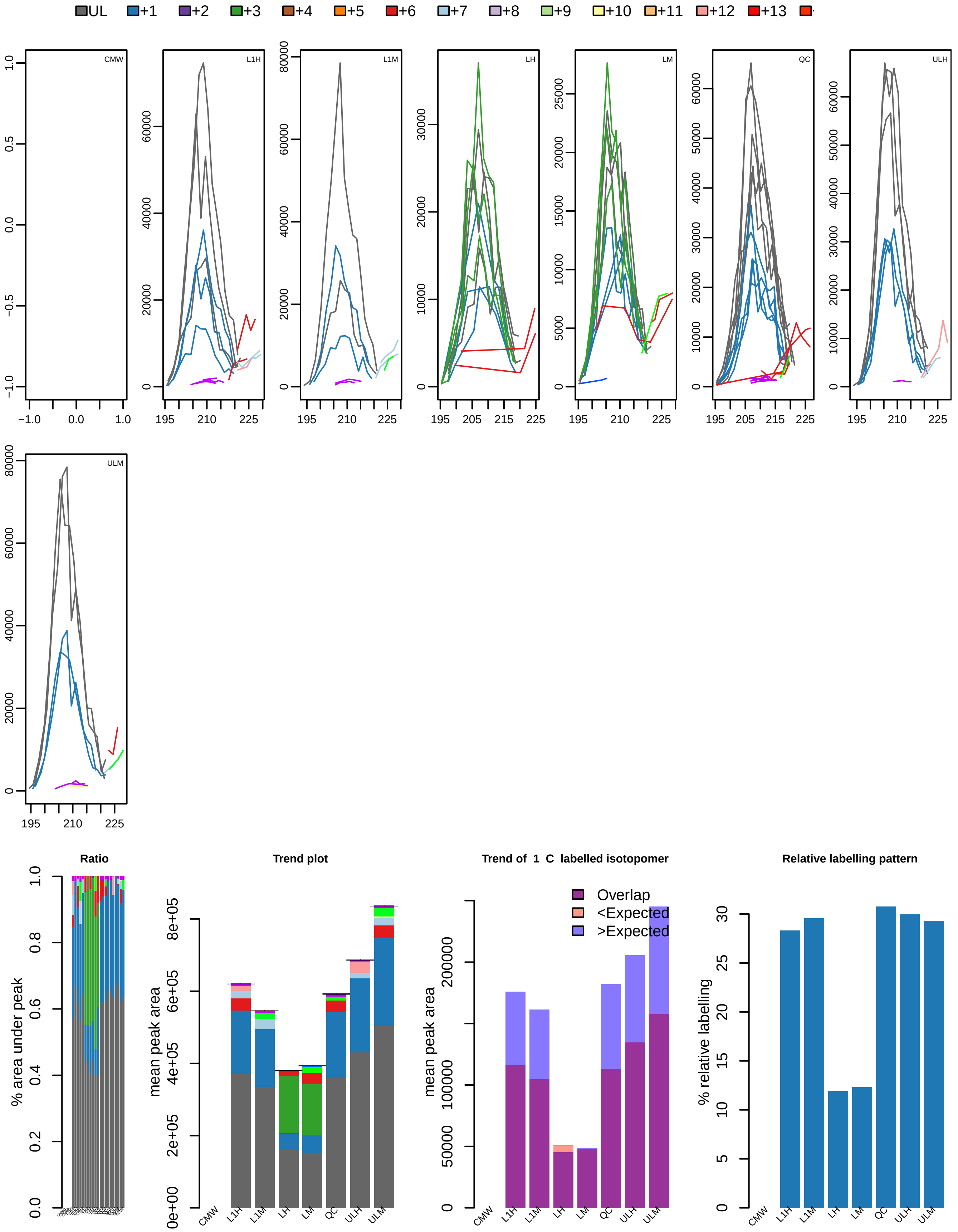

13,14−Dihydro− lipoxin A4

Formula: C20H33O5 Mass: 353.233 Std.RT: 270.3040482 Ion: POS

G1

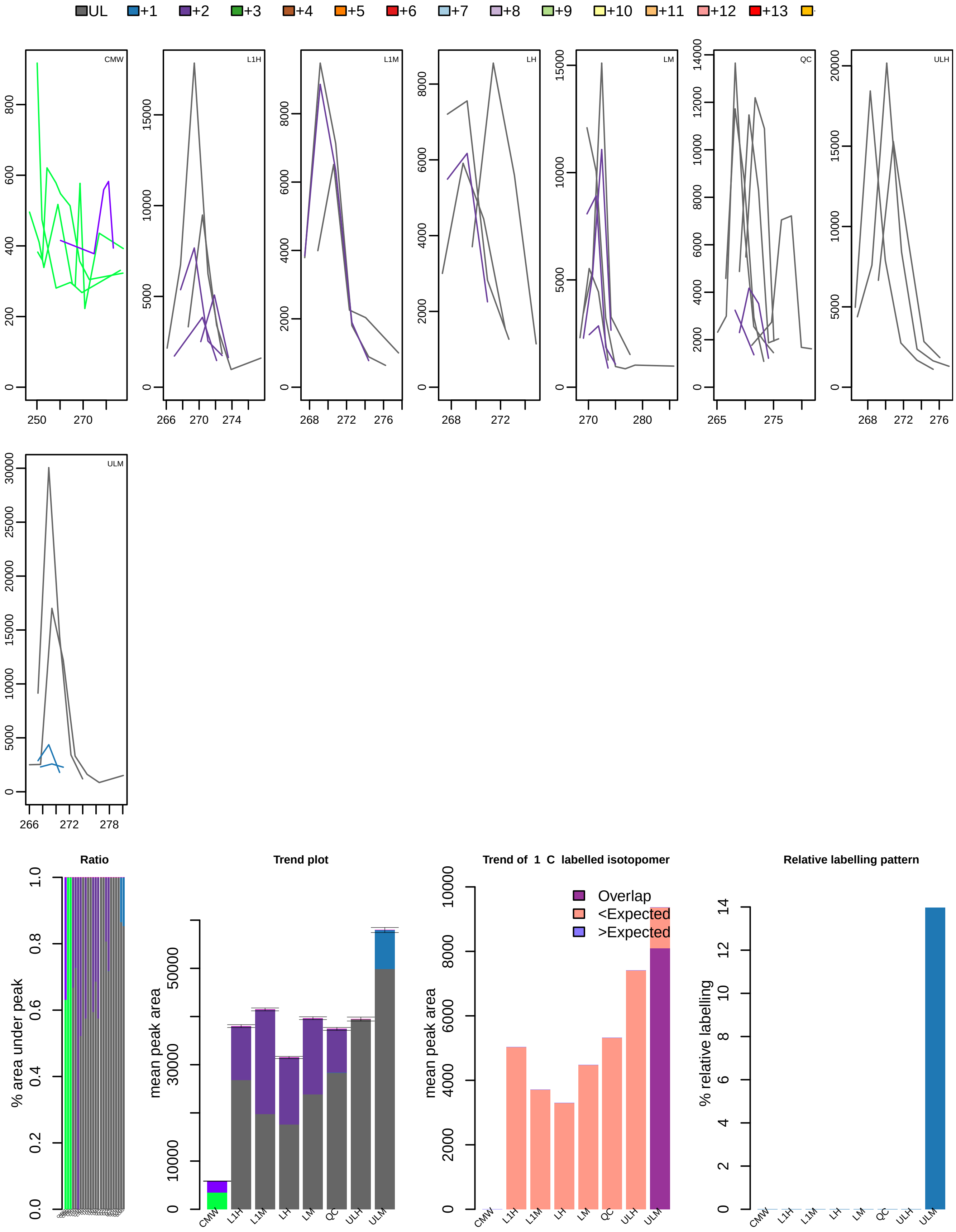

4−amino−4−deoxy−L−arabinose

Formula: C5H11NO4 Mass: 149.069 Std.RT: 693.2222814 Ion: POS

G1

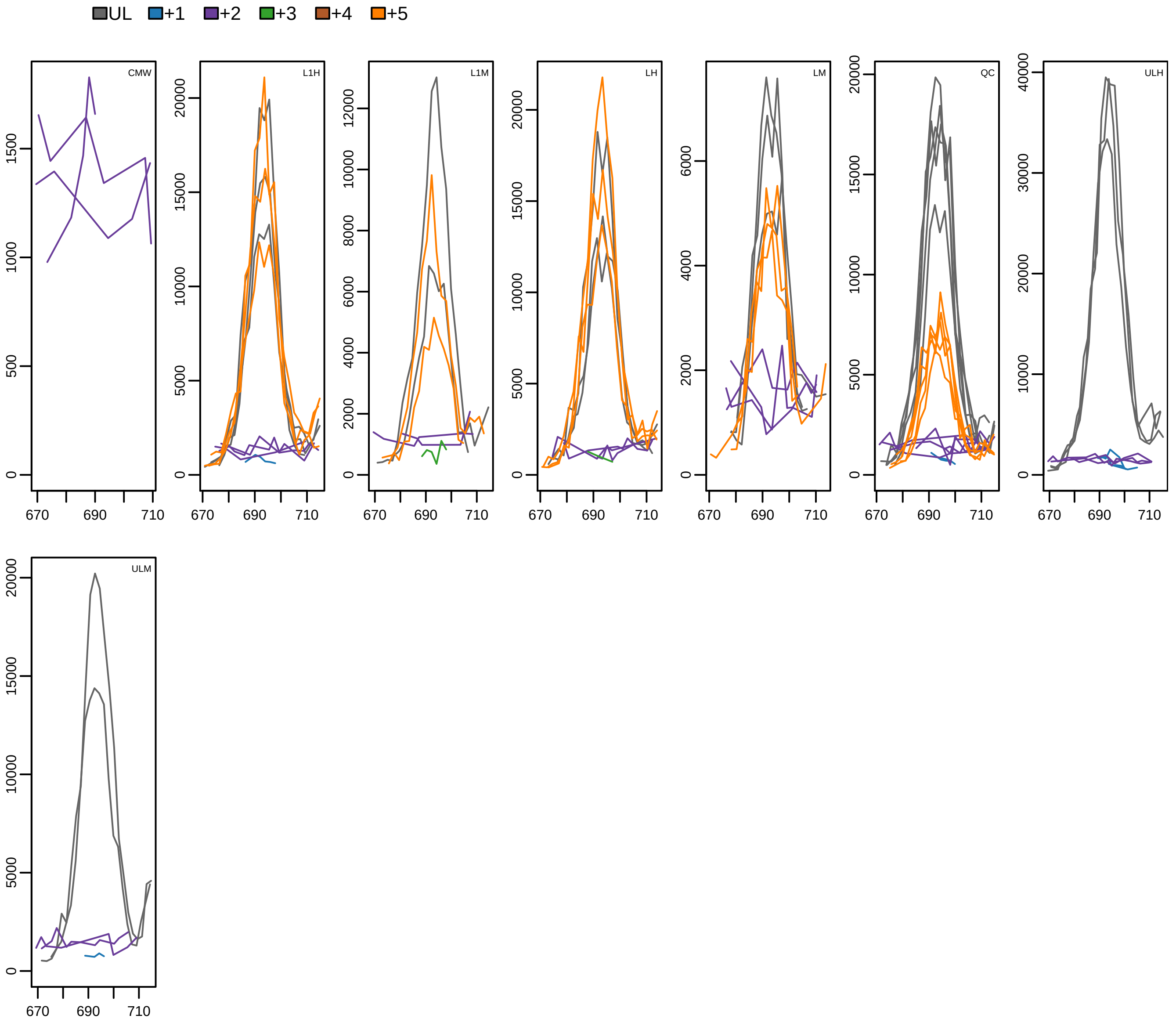

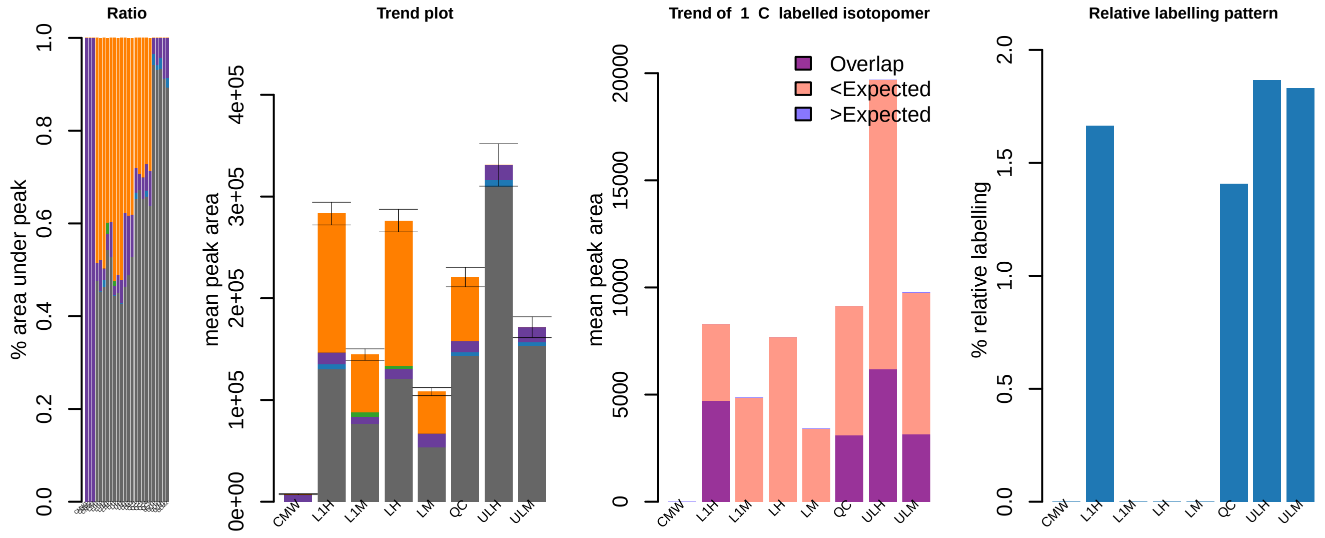

1−Oleoylglycerophosphocholine

Formula: C26H52NO7P Mass: 521.348 Std.RT: 257.14423698 Ion: P

G1

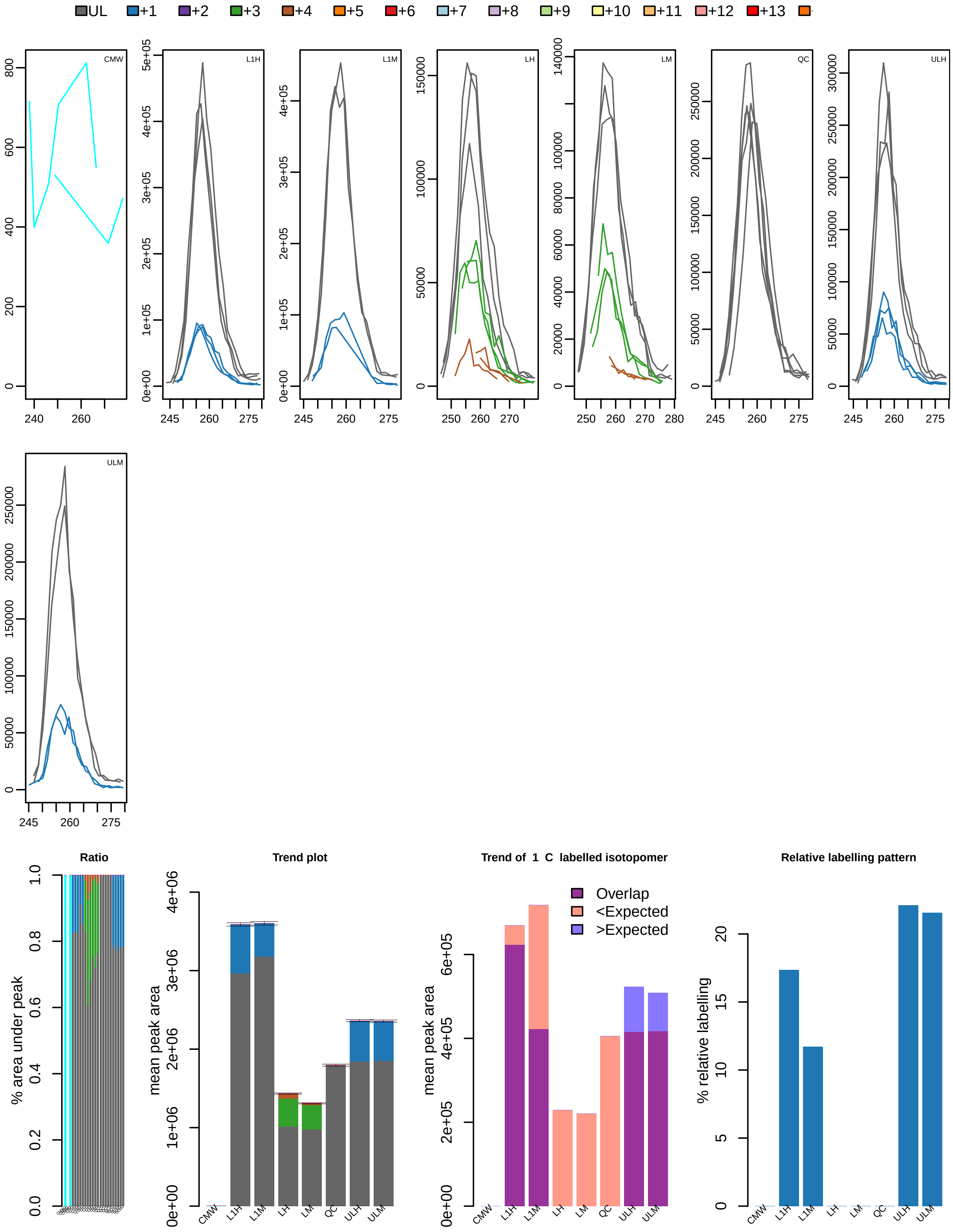

(Ac)2−L−Lys−D−Ala

G1

Formula: C13H23N3O5 Mass: 301.164 Std.RT: 314.92318872 Ion: P

UL +1 +2 +3 +4 +5 +6 +7 +8 +9 +10 +11 +12 +13

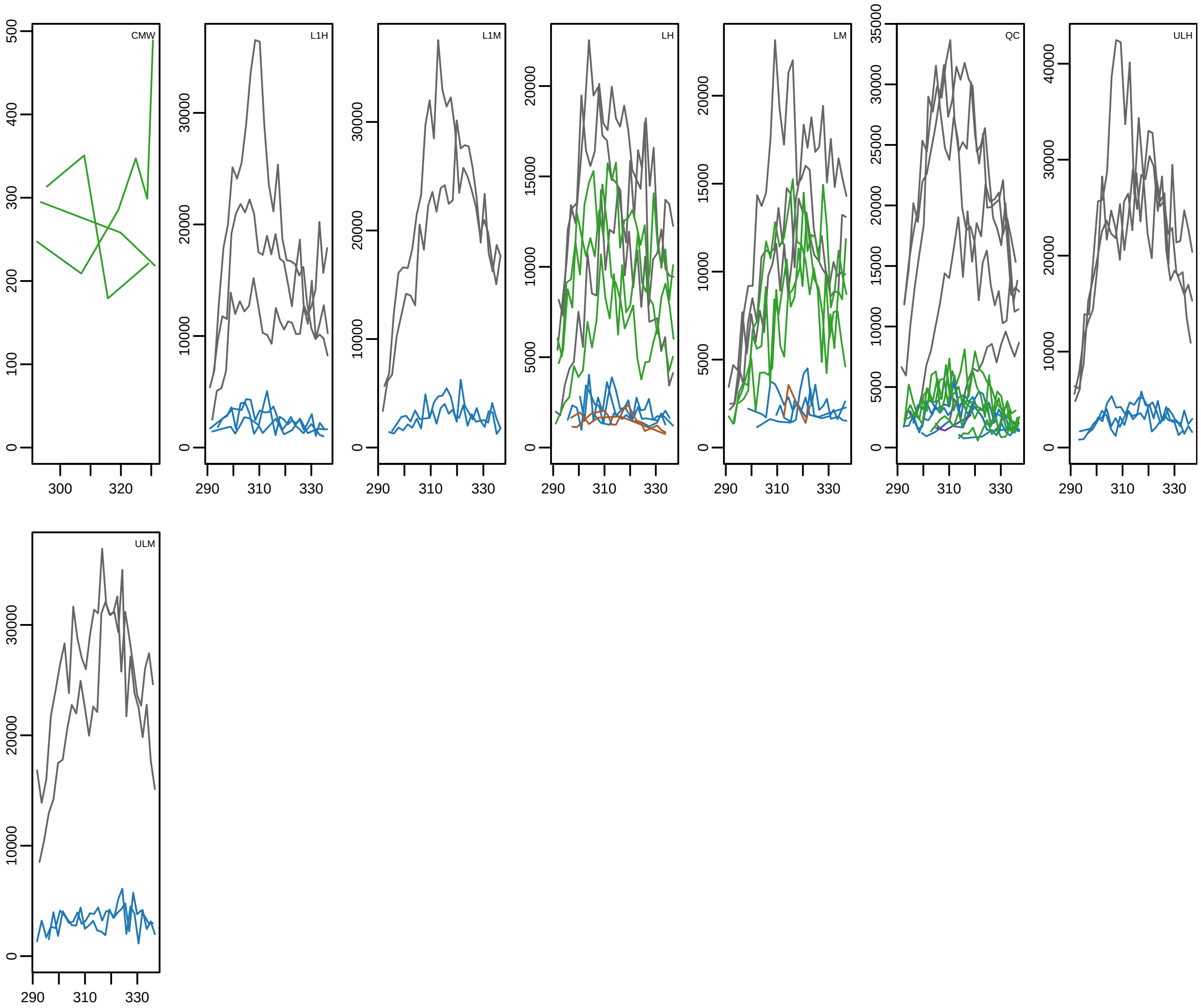

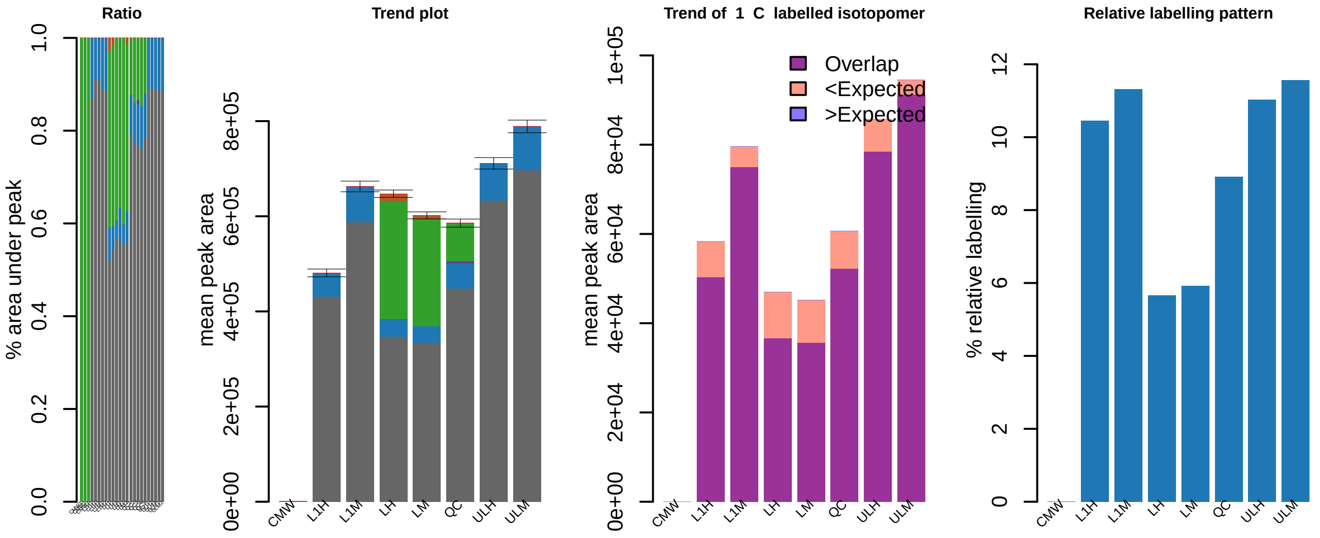

Formylmethionyl−leucyl−phenylalanine methyl ester

Formula: C22H33N3O5S Mass: 451.214 Std.RT: 223.77085662 Ion:

G1

UL +1 +2 +3 +4 +5 +6 +7 +8 +9 +10 +11 +12 +13

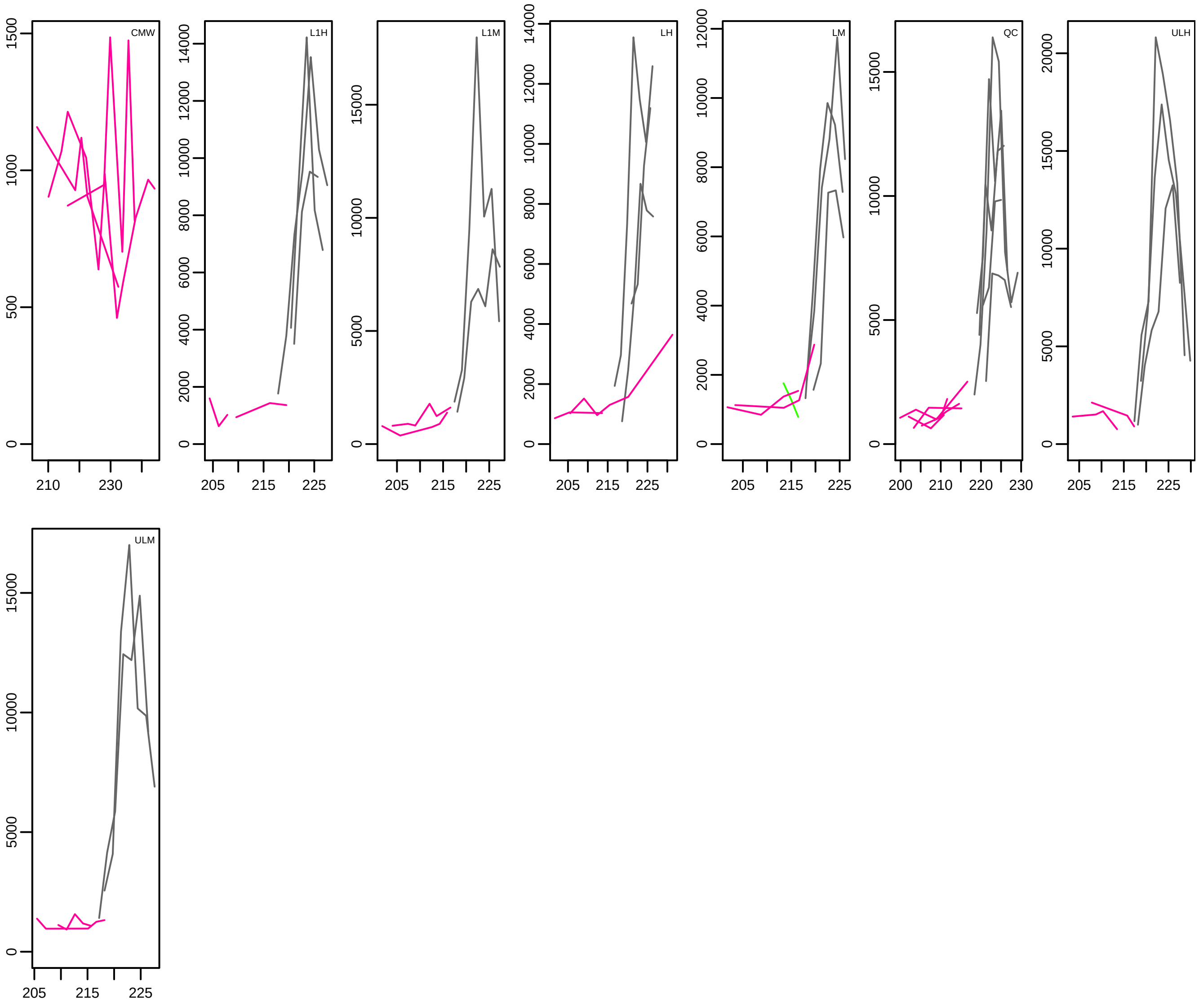

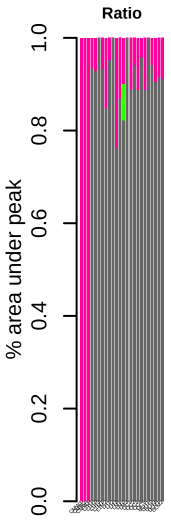

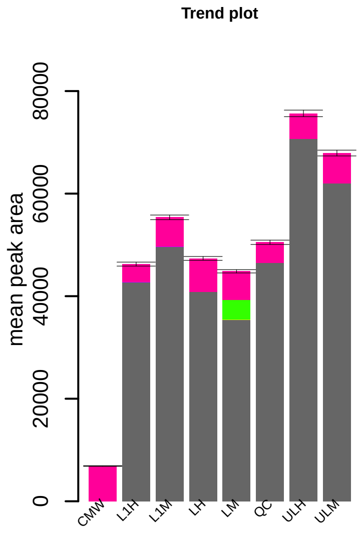

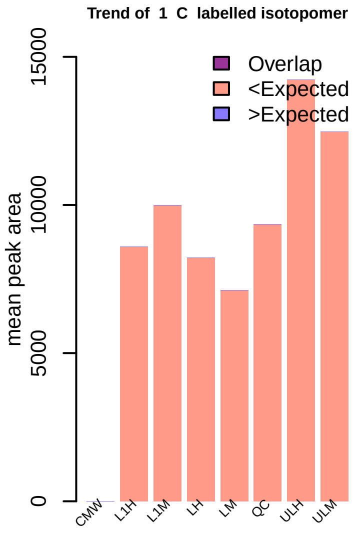

ε−aminocaproamide

G1

Formula: C6H14N2O Mass: 130.111 Std.RT: 1363.049169 Ion: POS

UL +1 +2 +3 +4 +5 +6

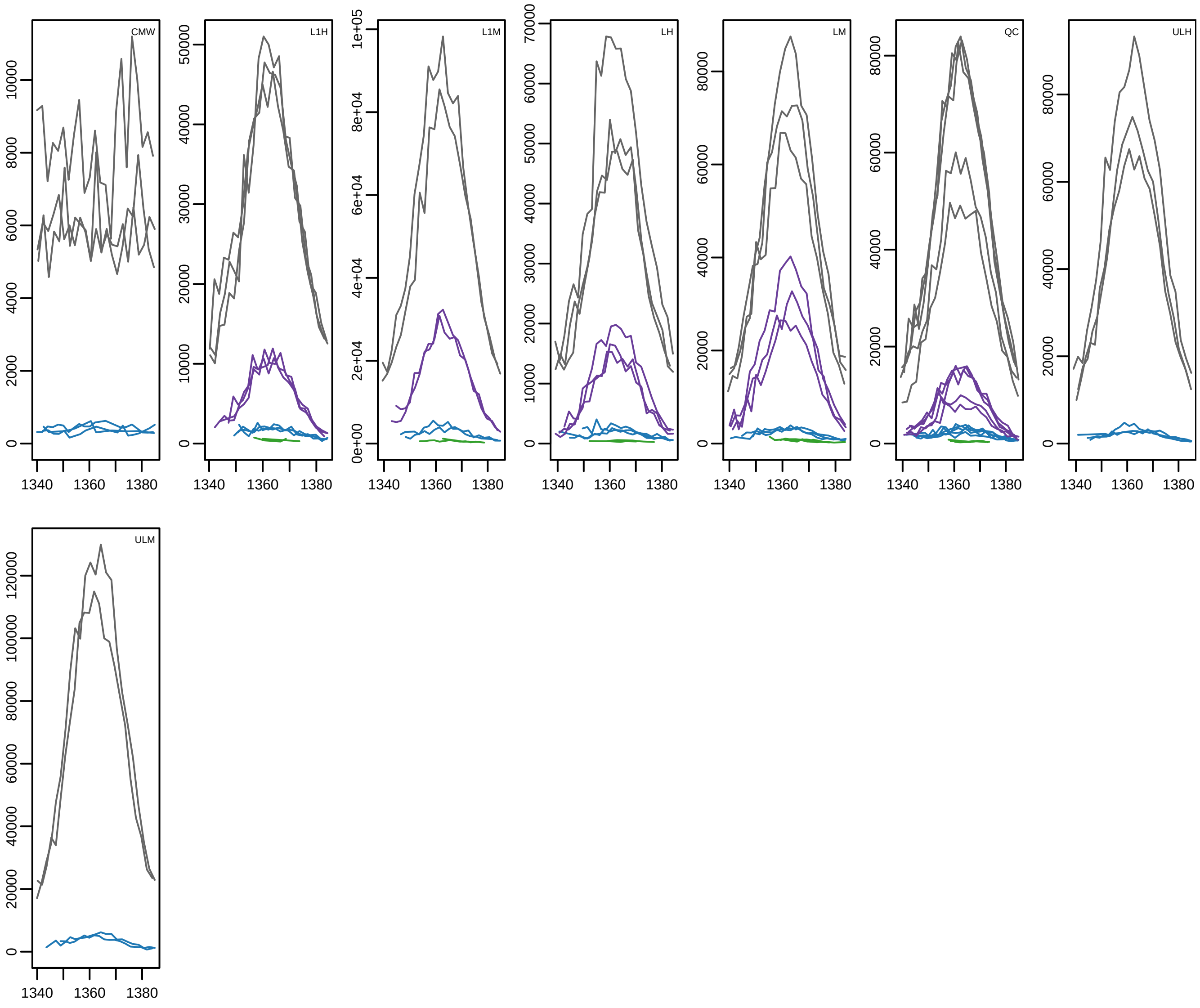

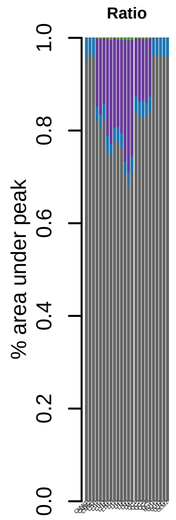

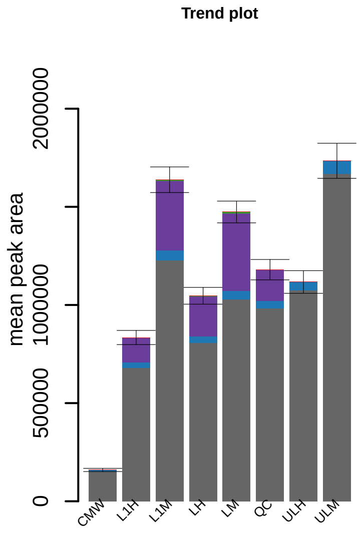

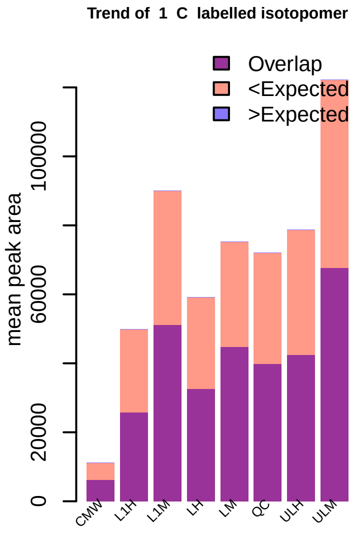

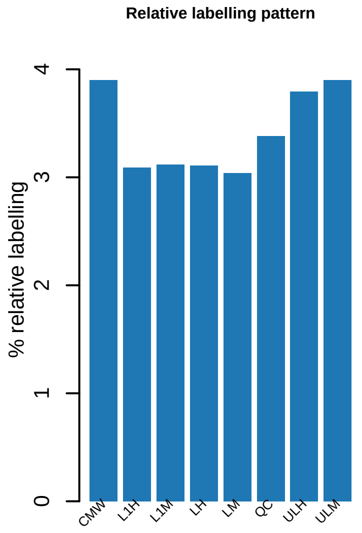

Spiradine A

Formula: C20H25NO2 Mass: 311.189 Std.RT: 644.1827472 Ion: POS

G1

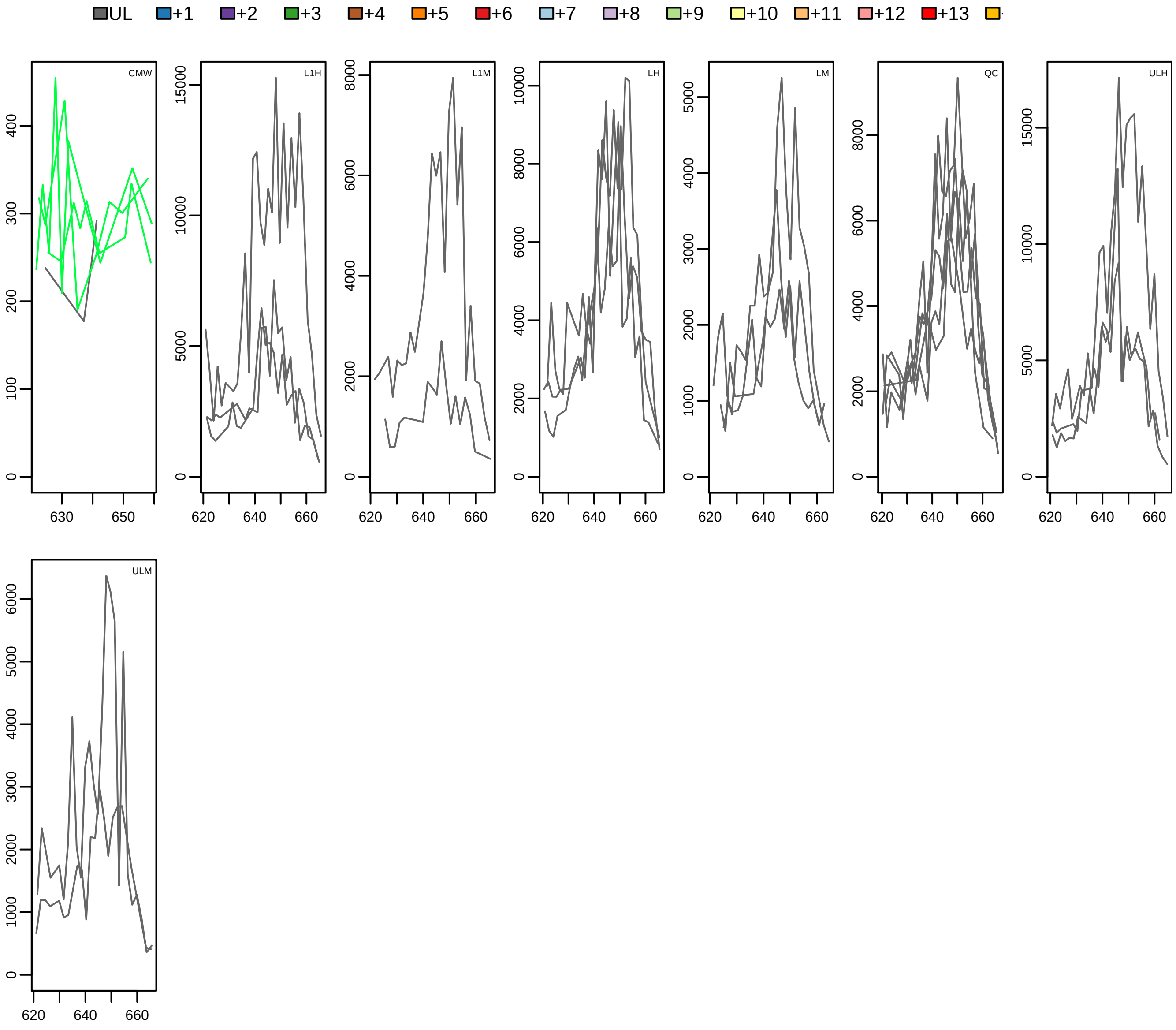

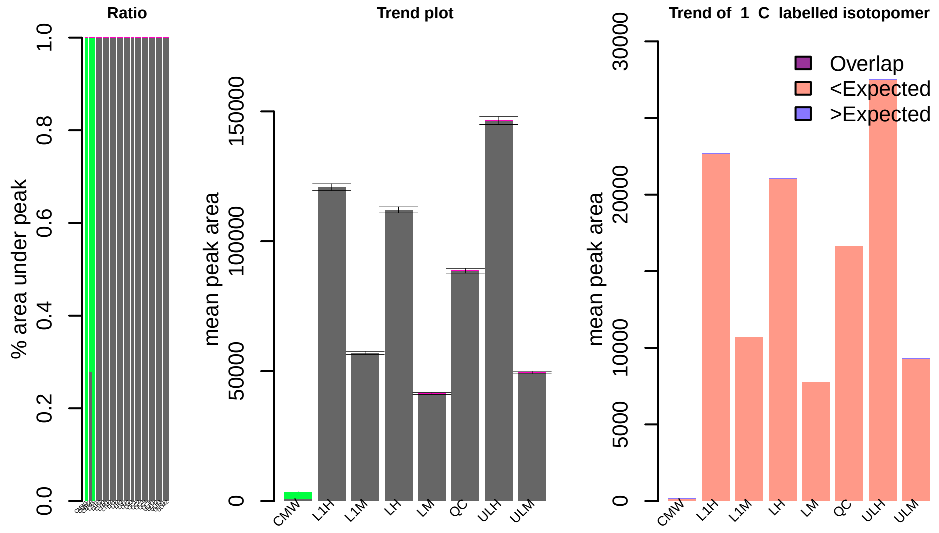

5−Methylcytidine

Formula: C10H15N3O5 Mass: 257.101 Std.RT: 682.2231444 Ion: PC

G1

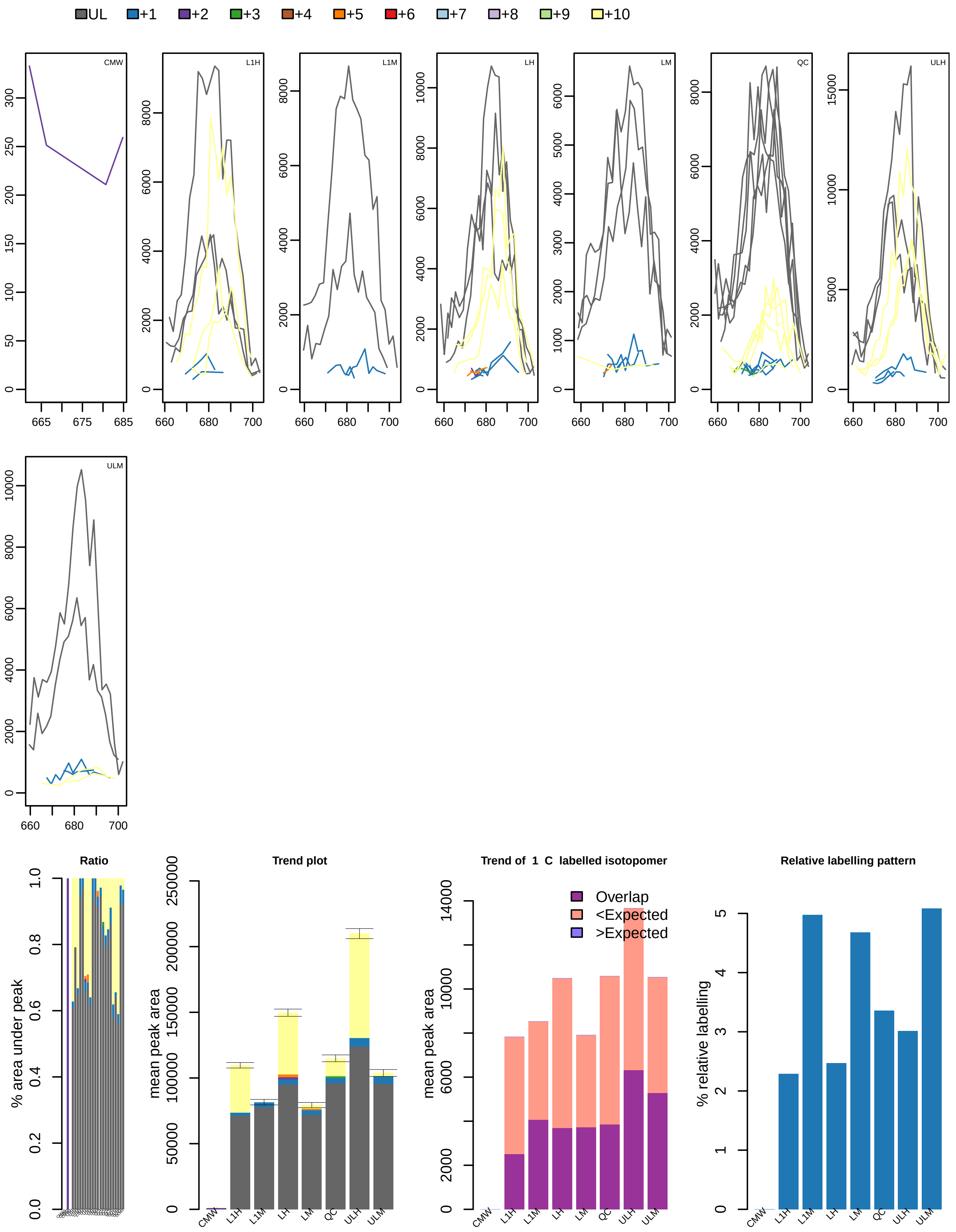

Daminozide

Formula: C6H12N2O3 Mass: 160.085 Std.RT: 435.9063327 Ion: POS

G1

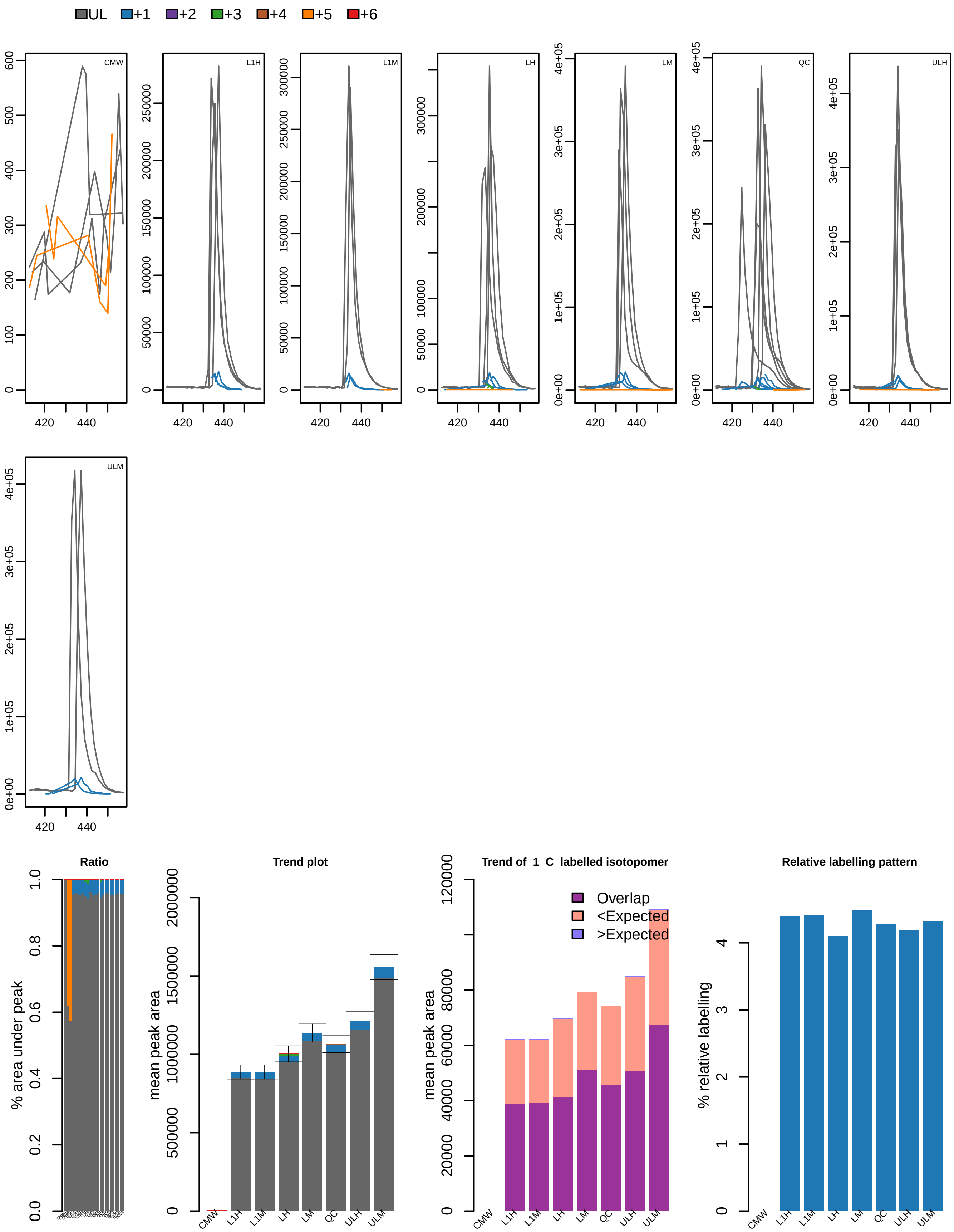

Carbetamide

Formula: C12H16N2O3 Mass: 236.116 Std.RT: 270.53629302 Ion: P

G1

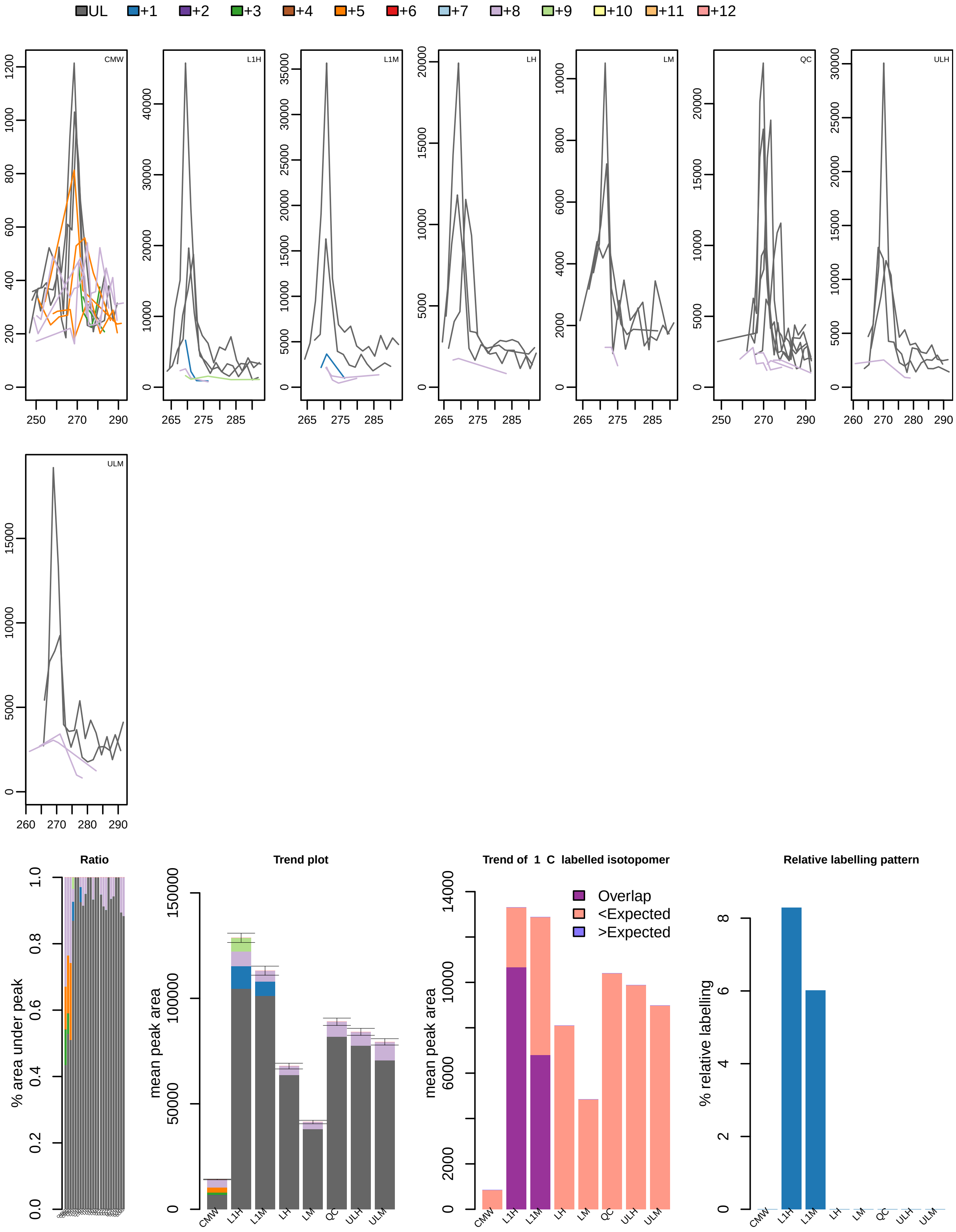

L−Homocitrulline

Formula: C7H15N3O3 Mass: 189.111 Std.RT: 988.149672 Ion: POS

G1

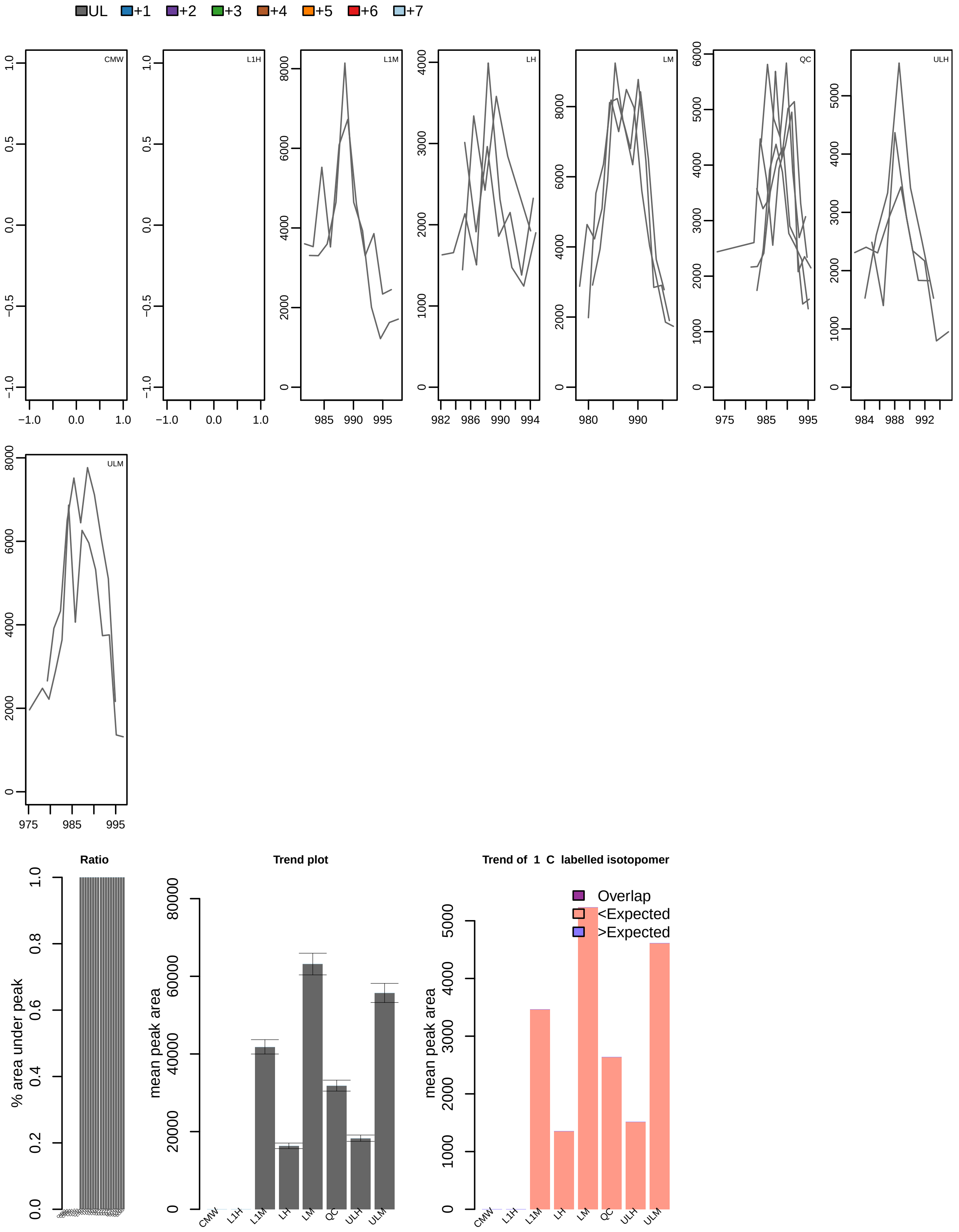

N−Acetyl−L−histidine

Formula: C8H11N3O3 Mass: 197.08 Std.RT: 510.17557632 Ion: POS

G1

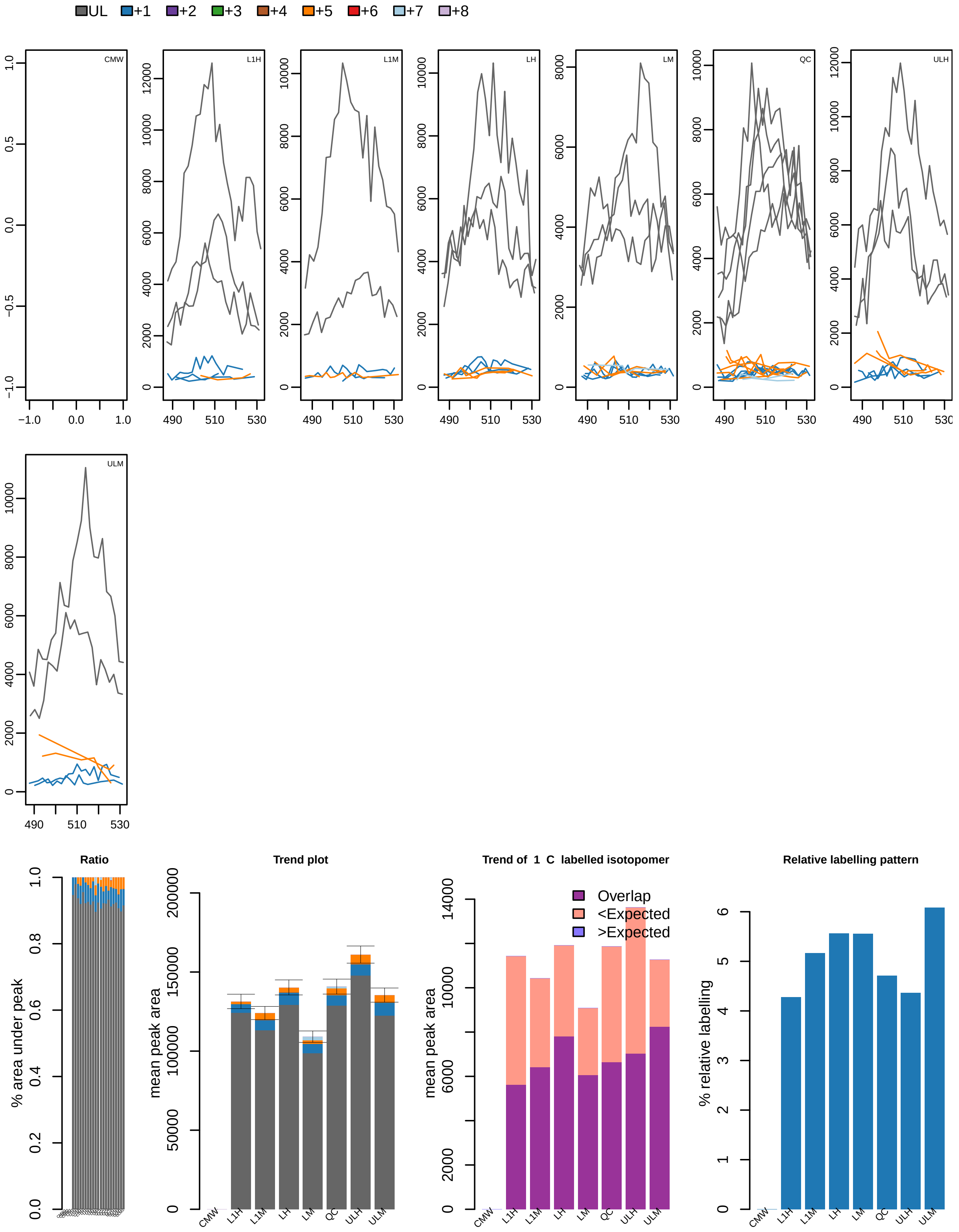

(−)−erythro−(2R,3R)−dihydroxybutylamide

Formula: C4H9NO3 Mass: 119.058 Std.RT: 436.49699778 Ion: POS

G1

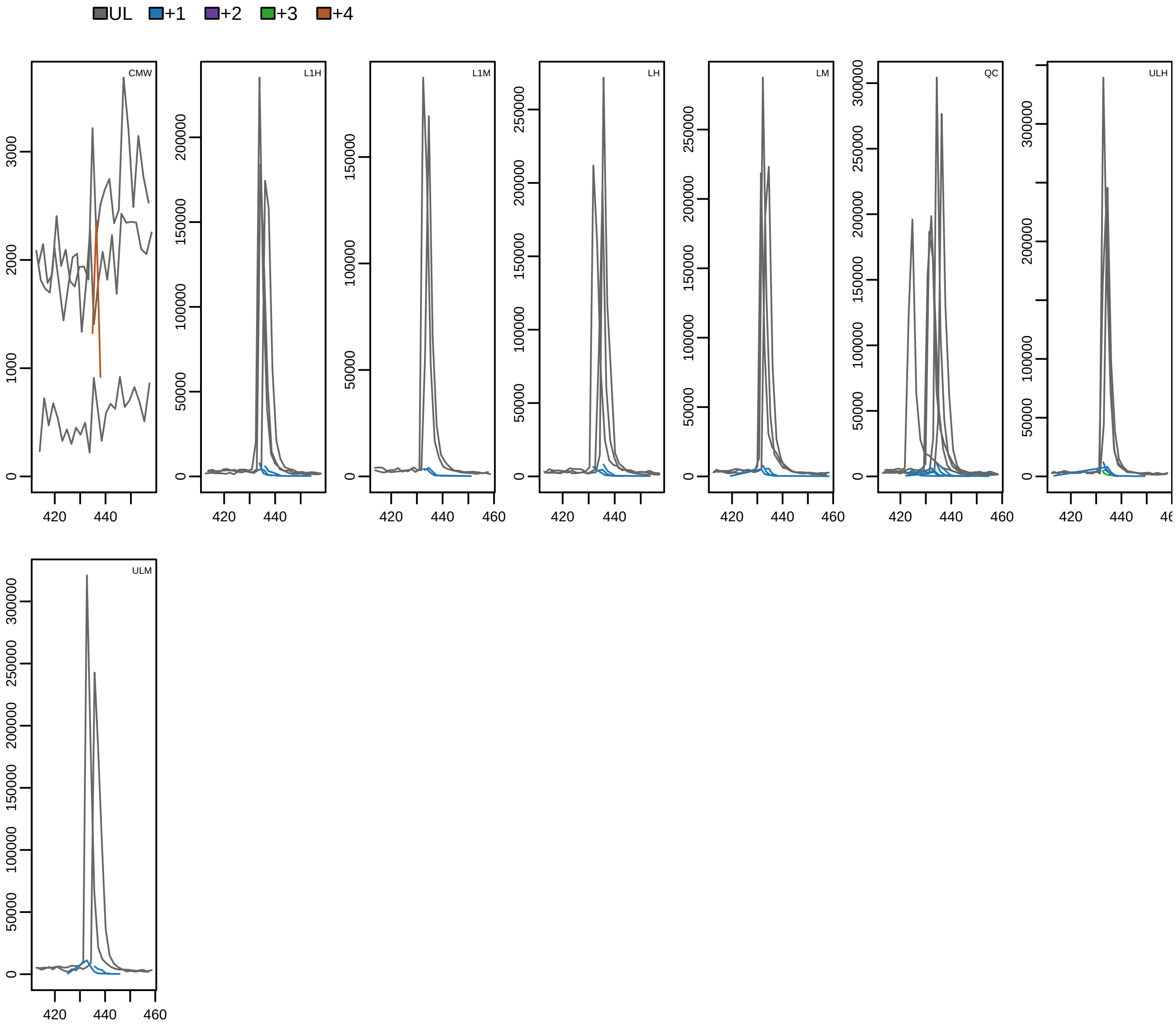

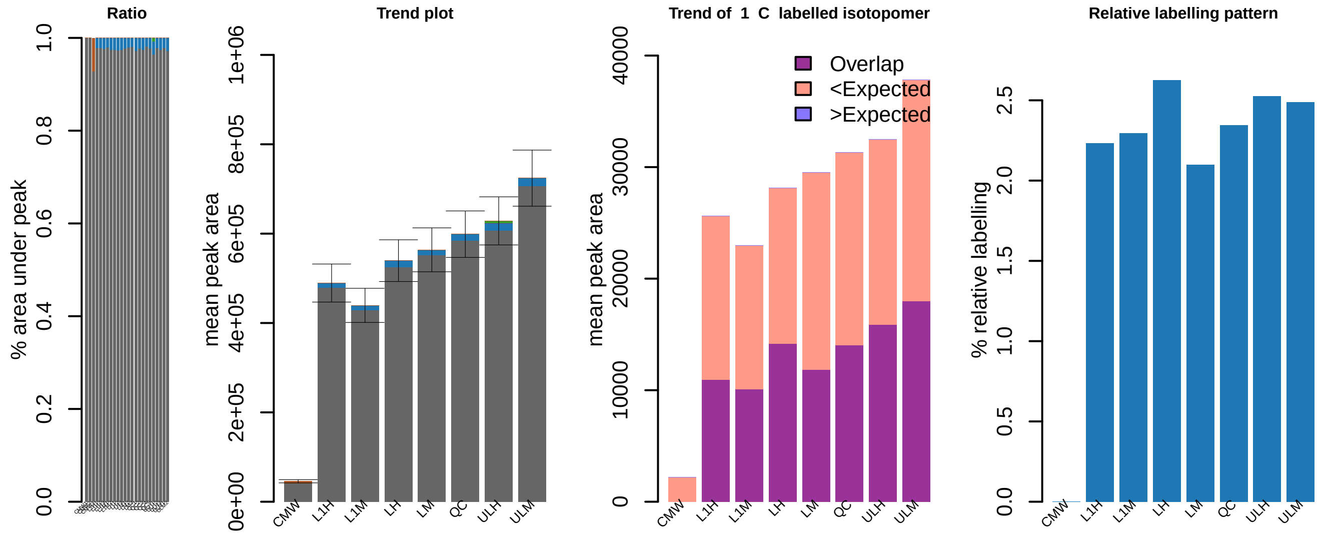

p−aminobenzoyl glutamate

Formula: C12H14N2O5 Mass: 266.09 Std.RT: 909.2144964 Ion: POS

G1

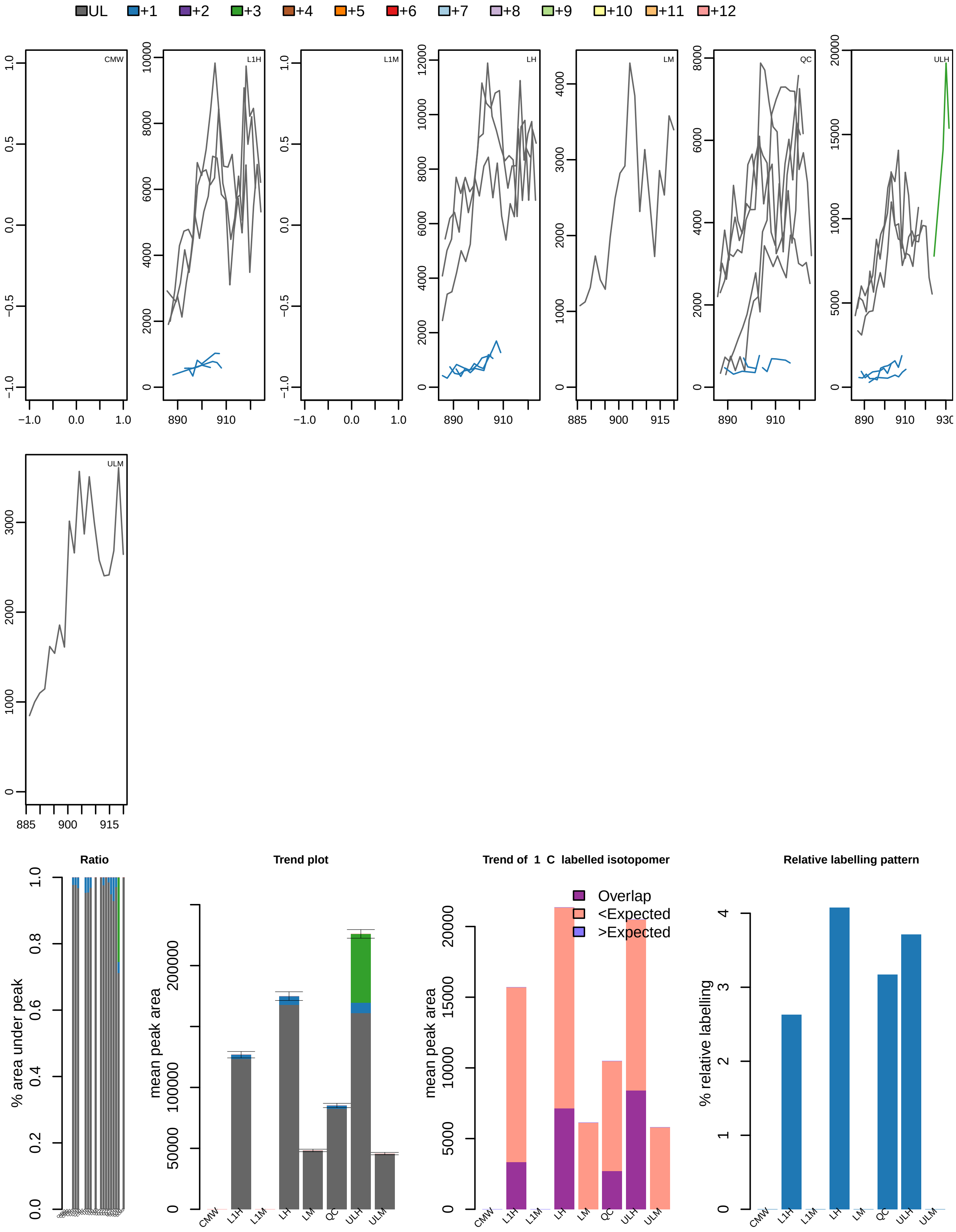

Temurin

Formula: C9H12N4O3 Mass: 224.091 Std.RT: 713.445426 Ion: POS

G1

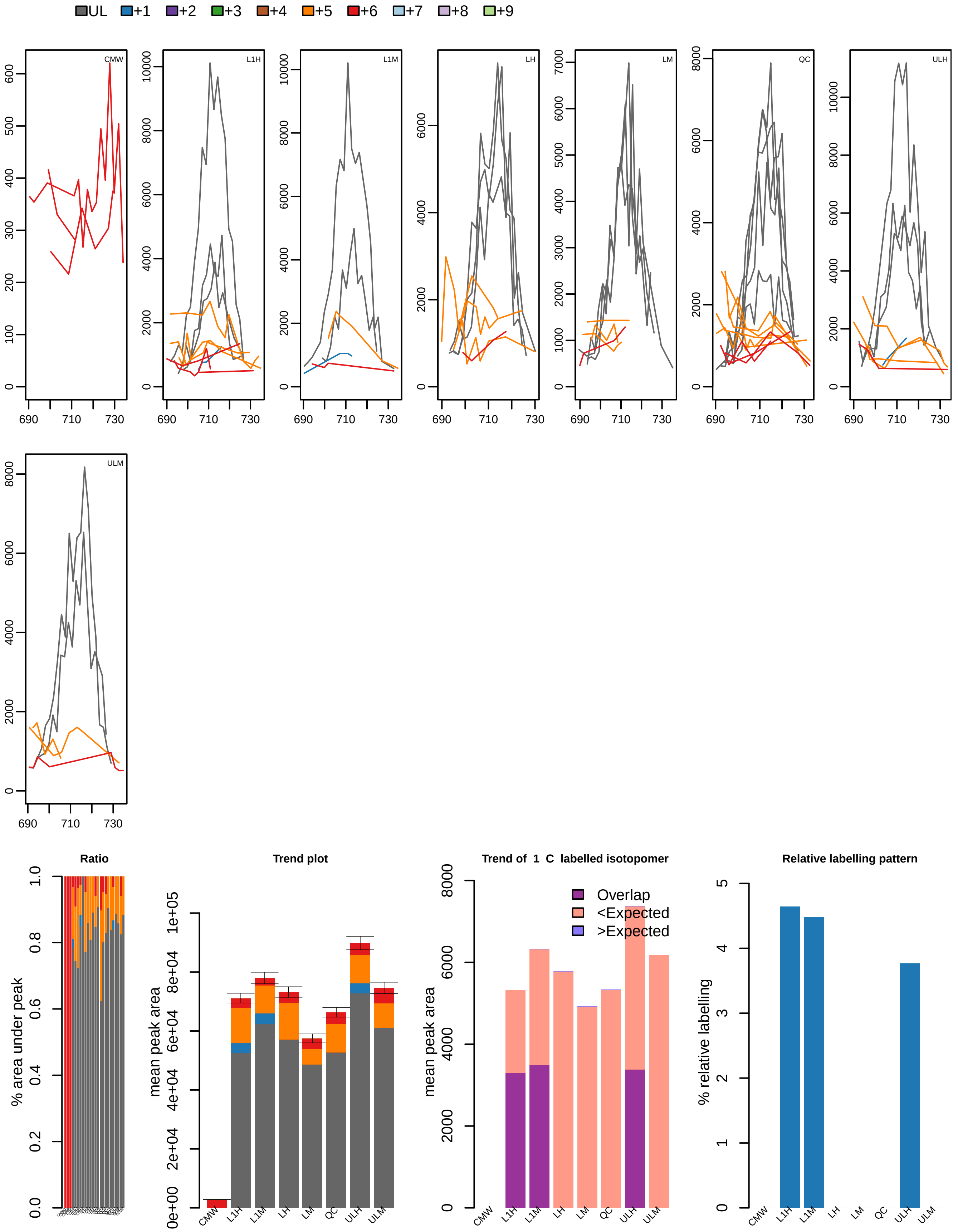

Bis(2−ethylhexyl)phthalate

Formula: $C_{24}H_{38}O_4$ Mass: 390.277 Std.RT: 223.16087472 Ion: POS

G1

UL +1 +2 +3 +4 +5 +6 +7 +8 +9 +10 +11 +12 +13

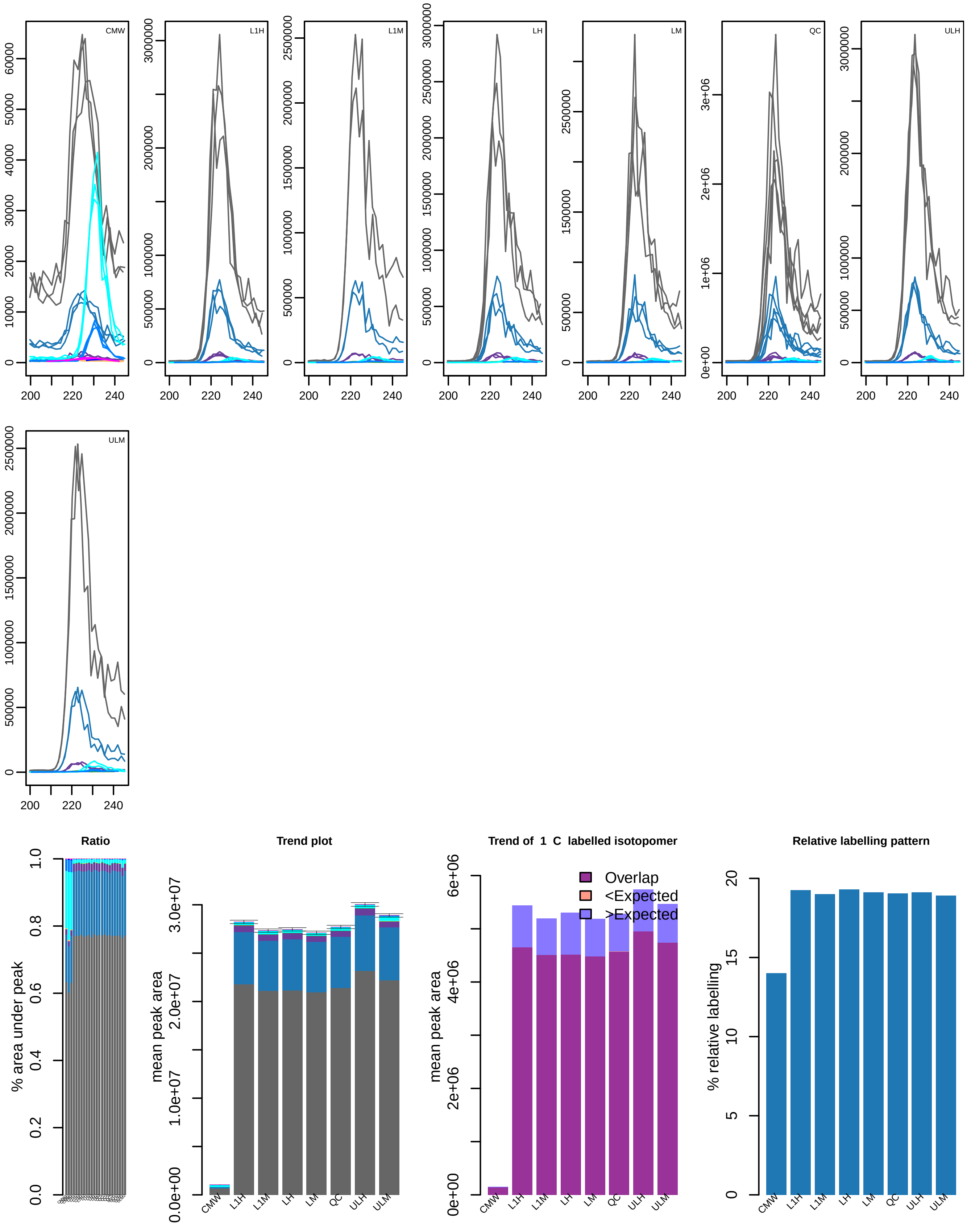

Bis(2−ethylhexyl)phthalate

G2

Formula: C24H38O4 Mass: 390.277 Std.RT: 223.16087472 Ion: POS

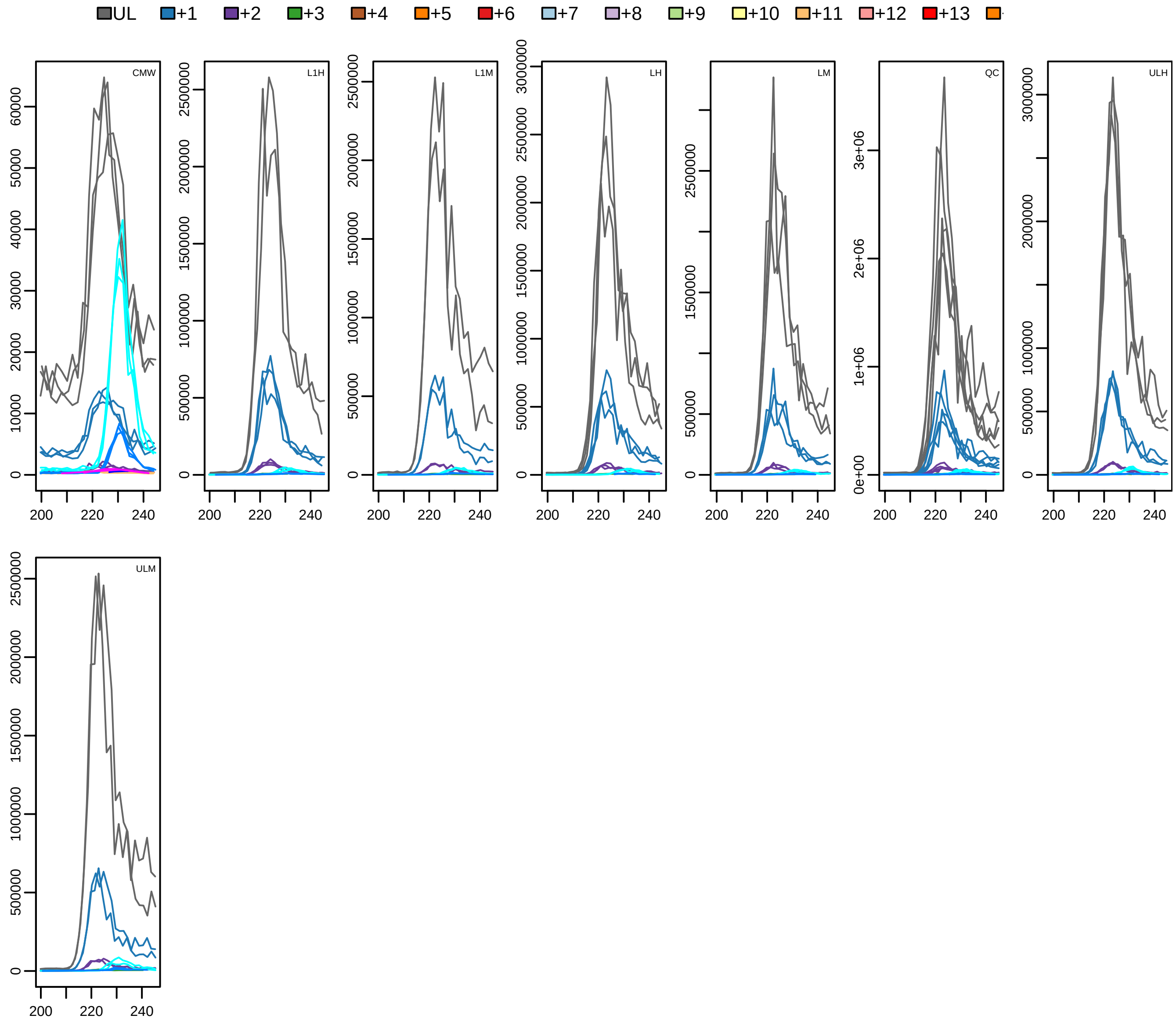

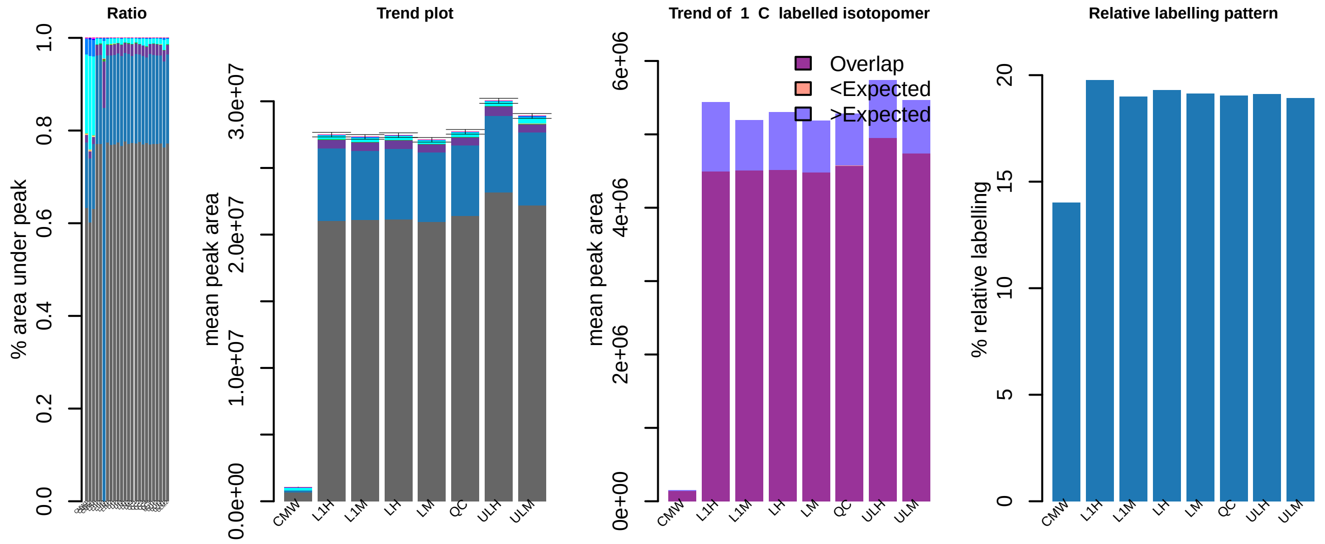

Aurin

G1

Formula: C19H14O3 Mass: 290.094 Std.RT: 431.36418948 Ion: POS

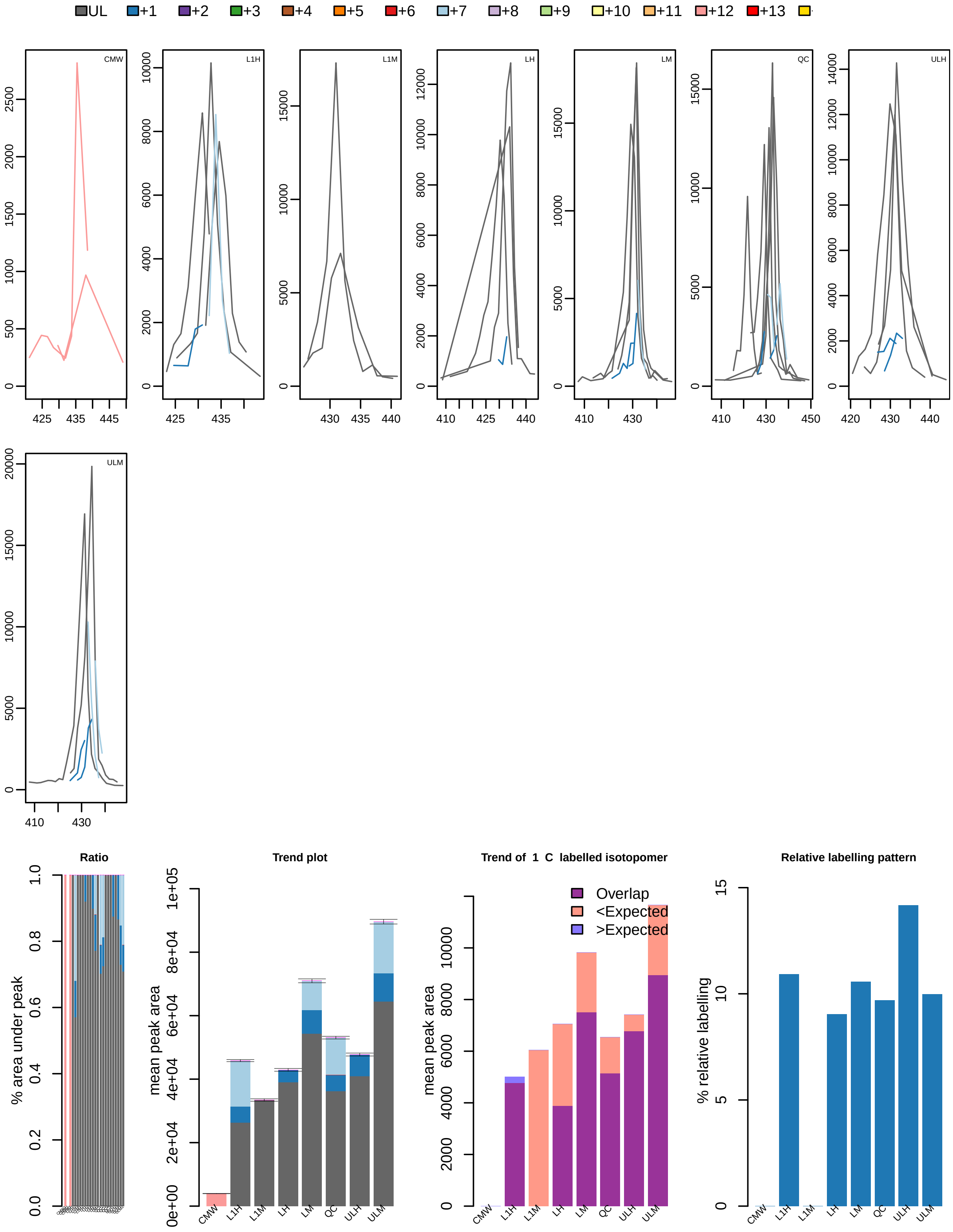

N−Lauroylglycine

Formula: C14H27NO3 Mass: 257.199 Std.RT: 241.50735378 Ion: PC

G1

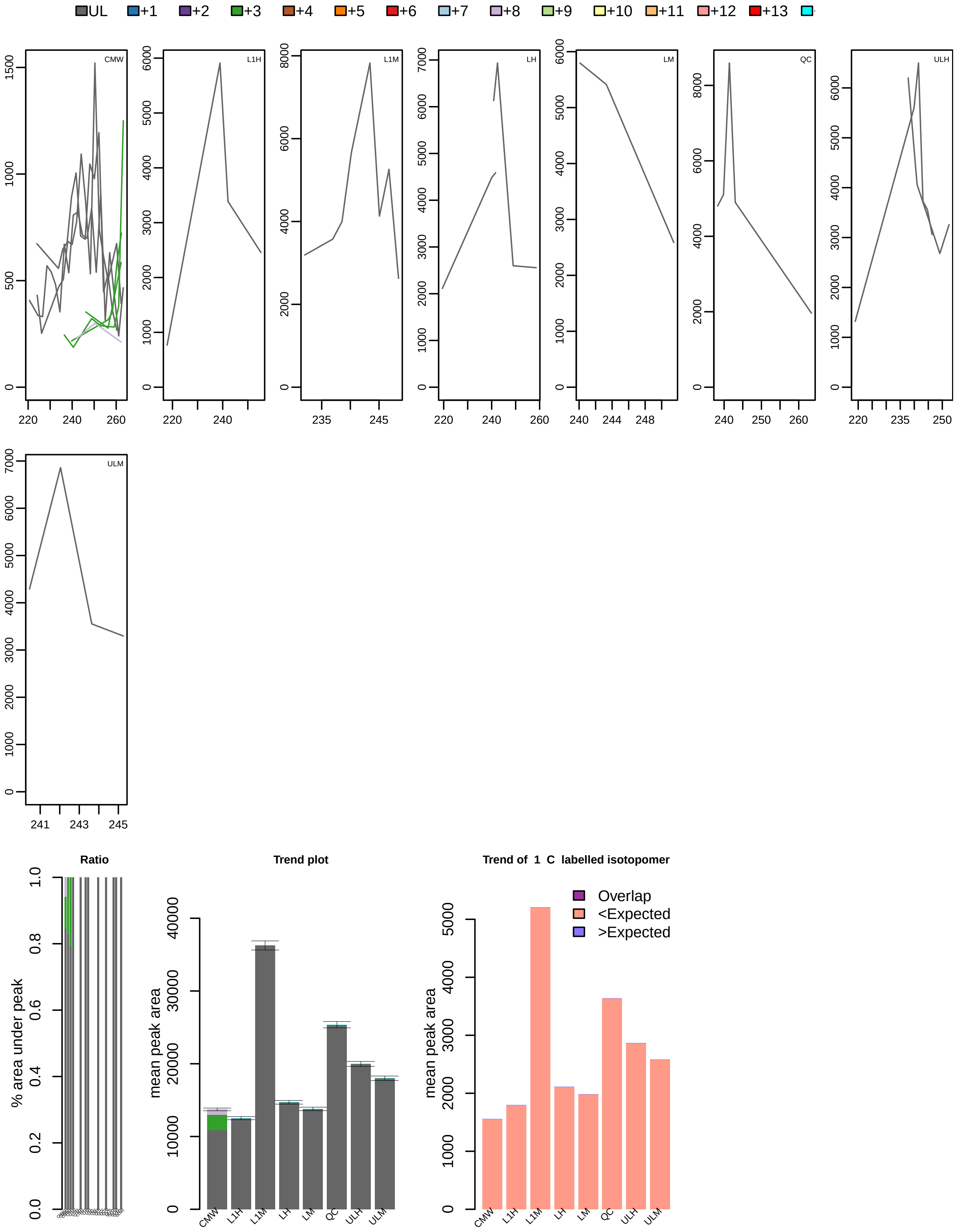

(S)−ATPA

Formula: C10H16N2O4 Mass: 228.111 Std.RT: 913.609749 Ion: POS

G1

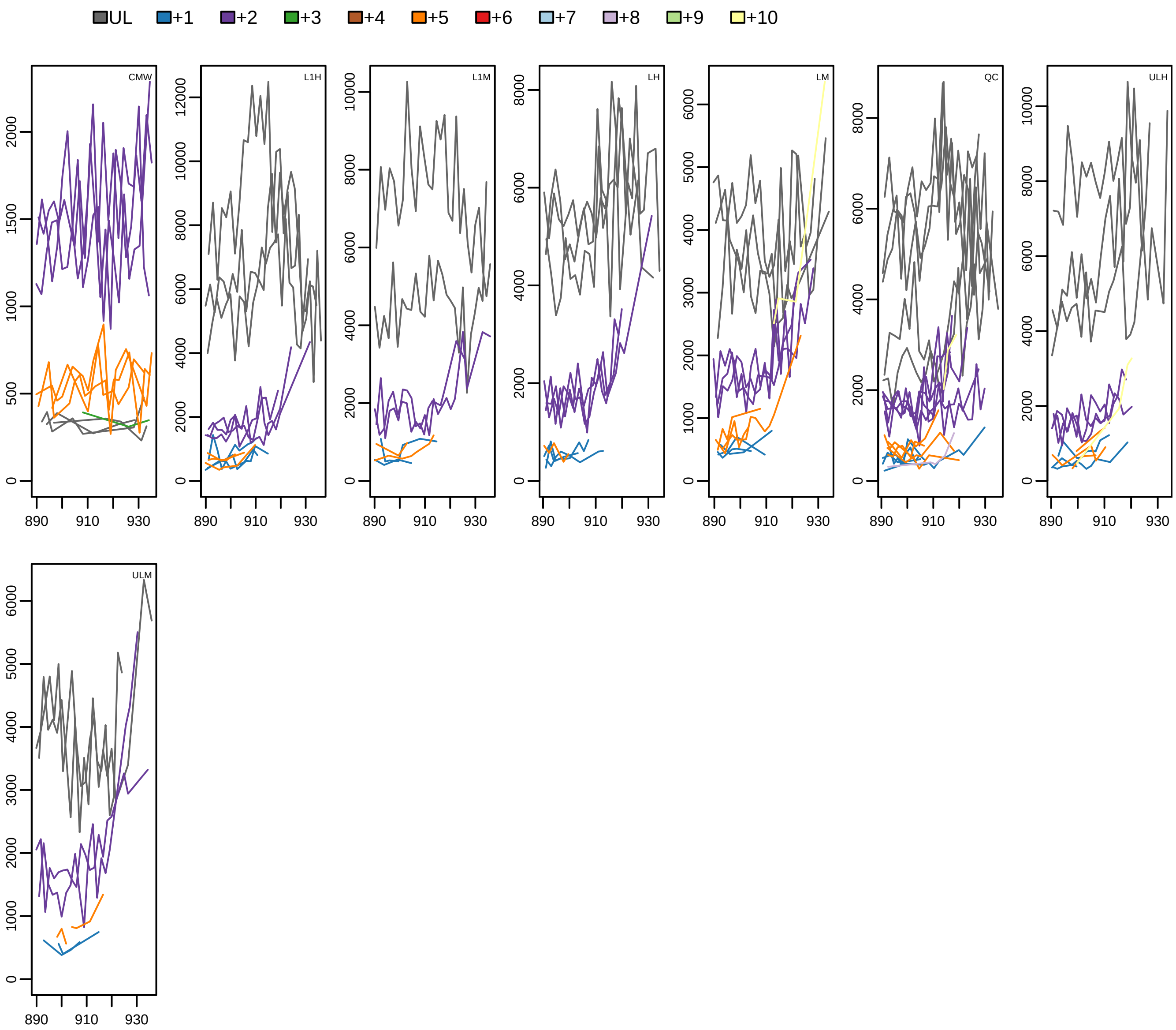

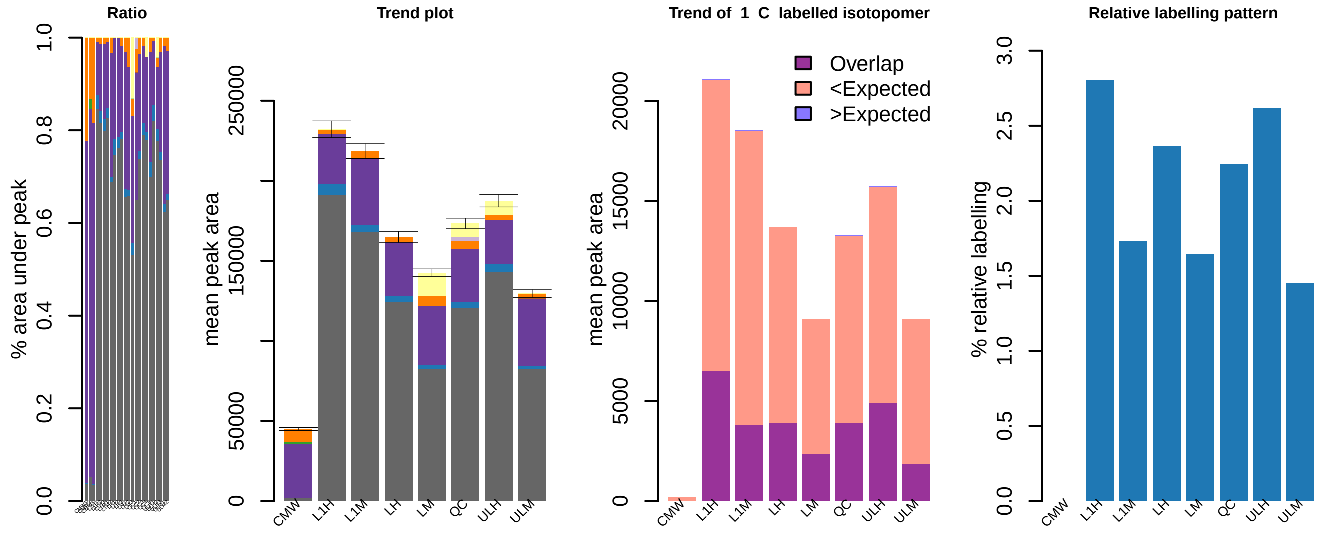

Nalpha,Nalpha−Dimethyl−L−histidine

Formula: C8H13N3O2 Mass: 183.101 Std.RT: 1003.0769088 Ion: PC

G1

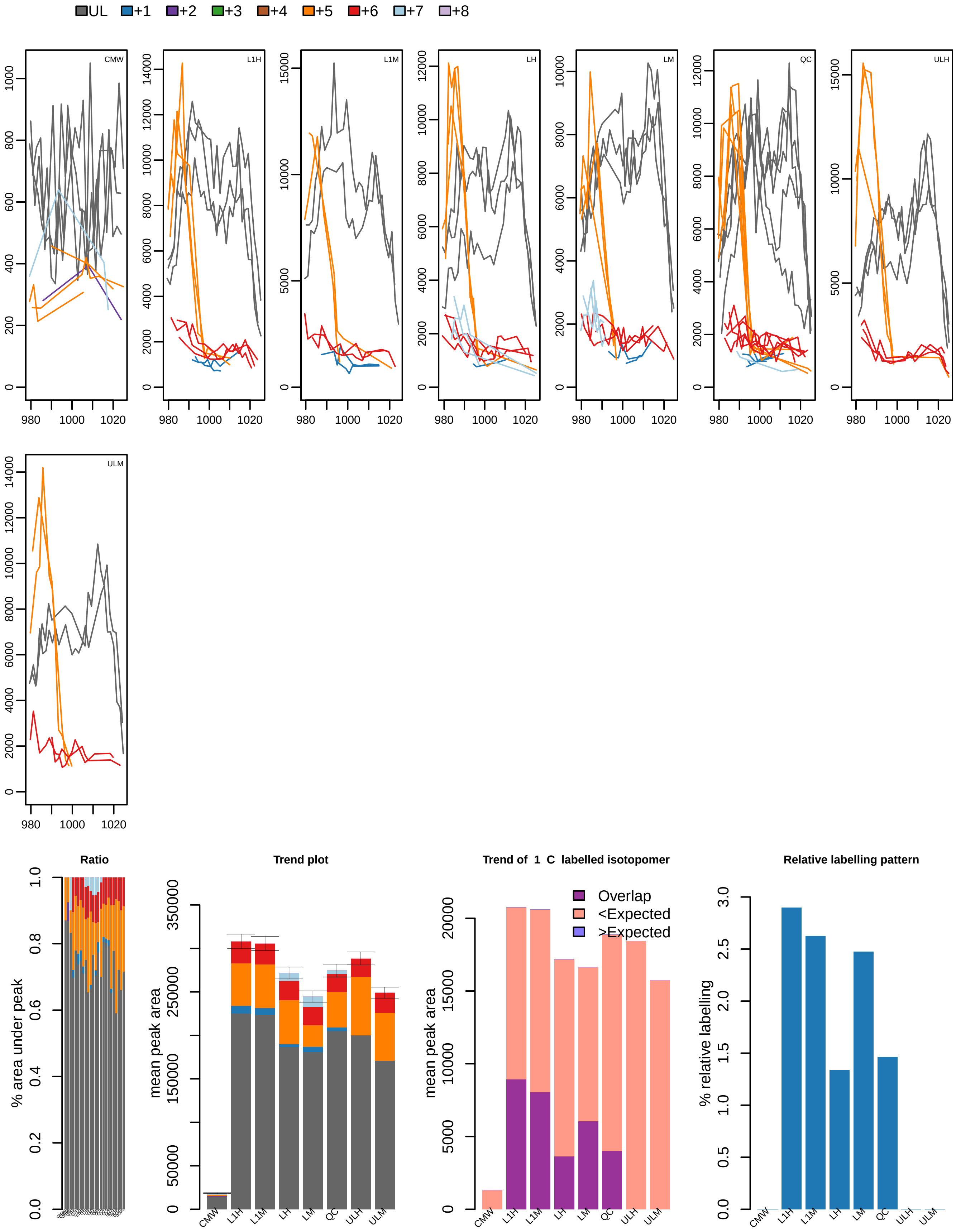

1−deoxyxylonojirimycin

Formula: C5H11NO3 Mass: 133.074 Std.RT: 904.7867484 Ion: POS

G1

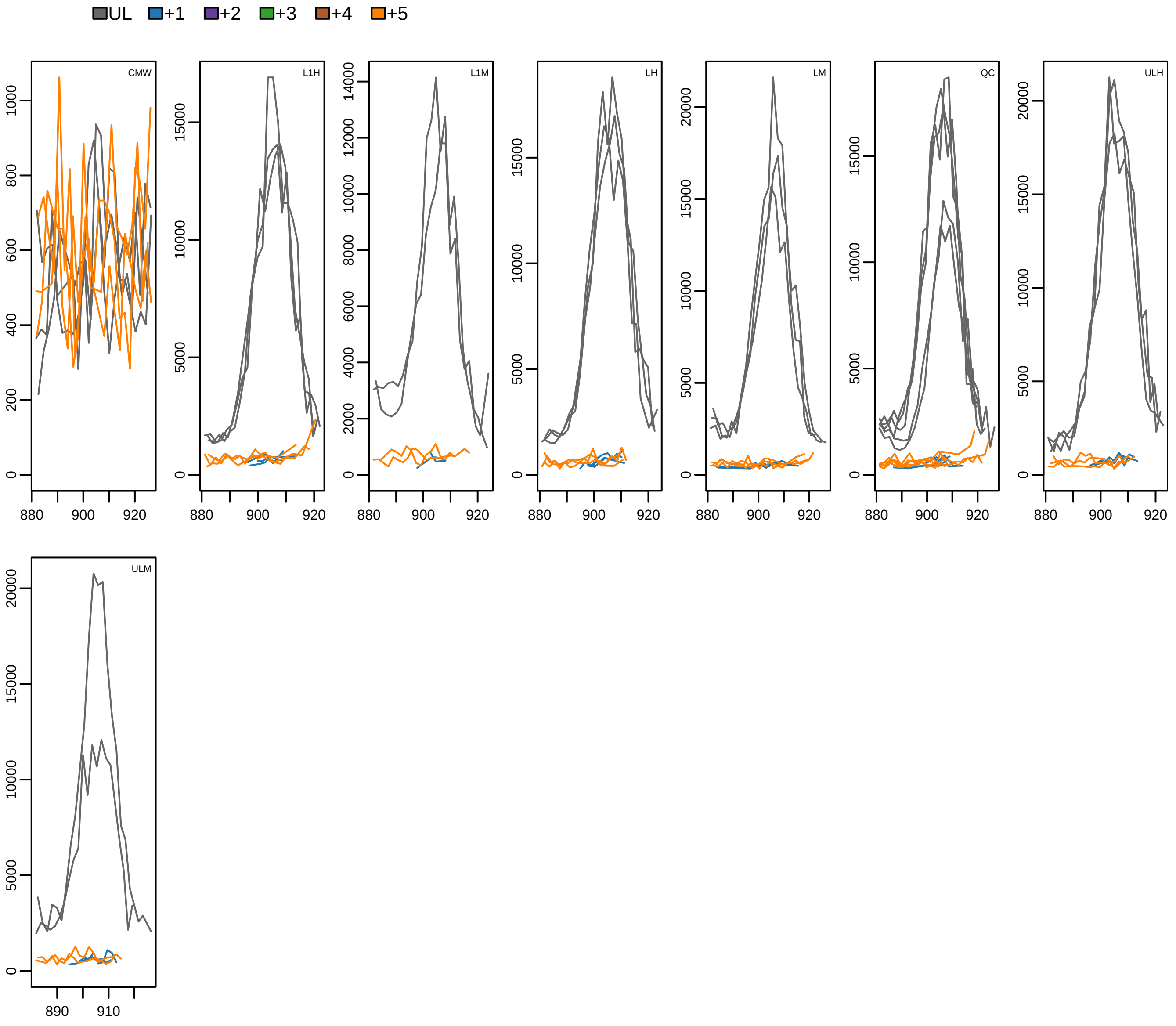

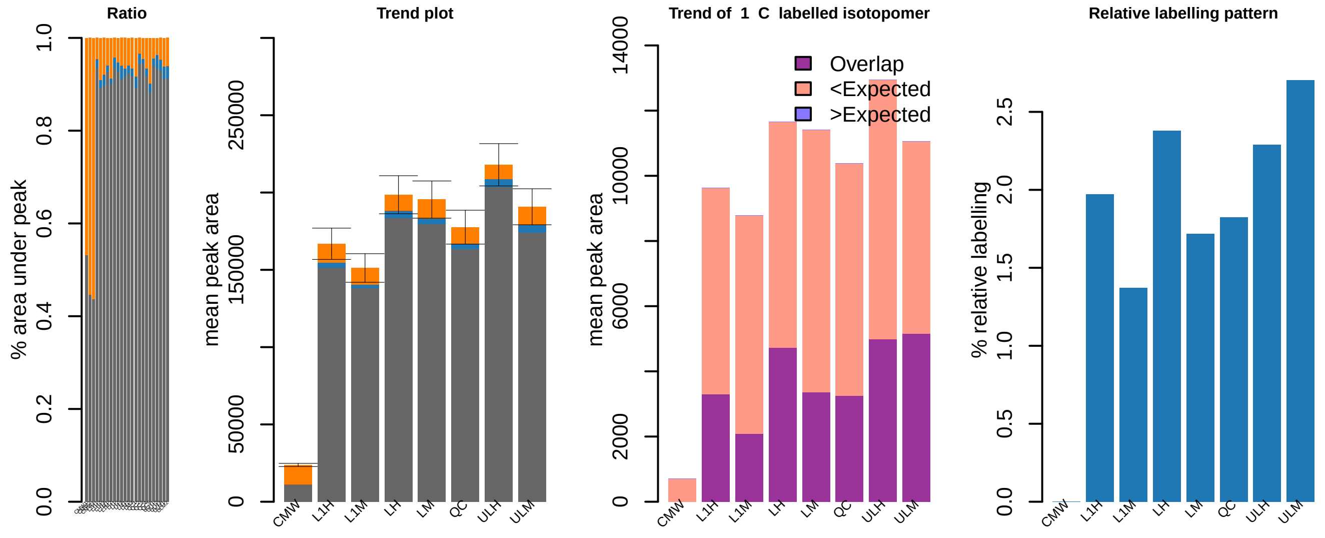

1−deoxyxylonojirimycin

Formula: C5H11NO3 Mass: 133.074 Std.RT: 904.7867484 Ion: POS

G2

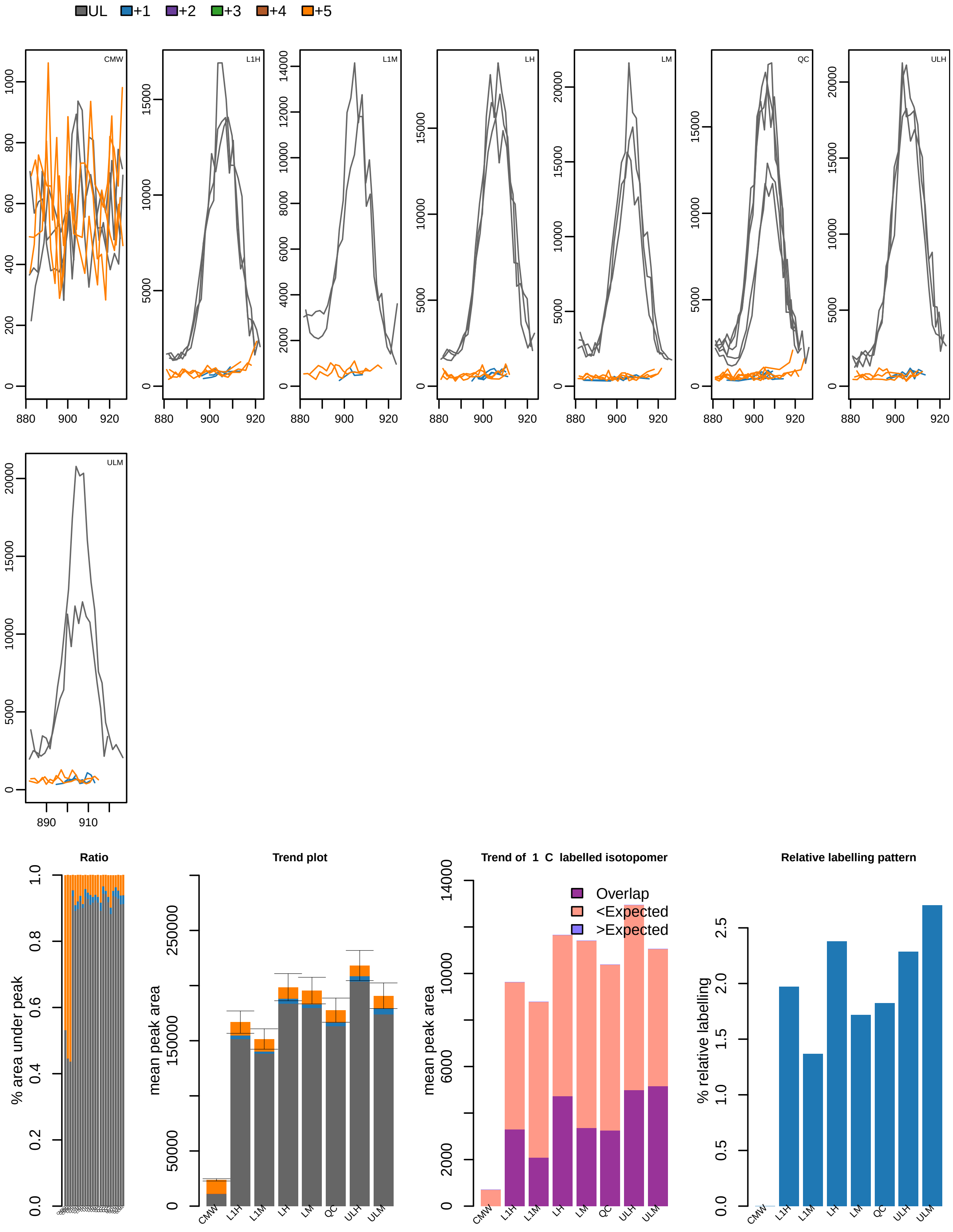

1−deoxyxylonojirimycin

Formula: C5H11NO3 Mass: 133.074 Std.RT: 904.7867484 Ion: POS

G3

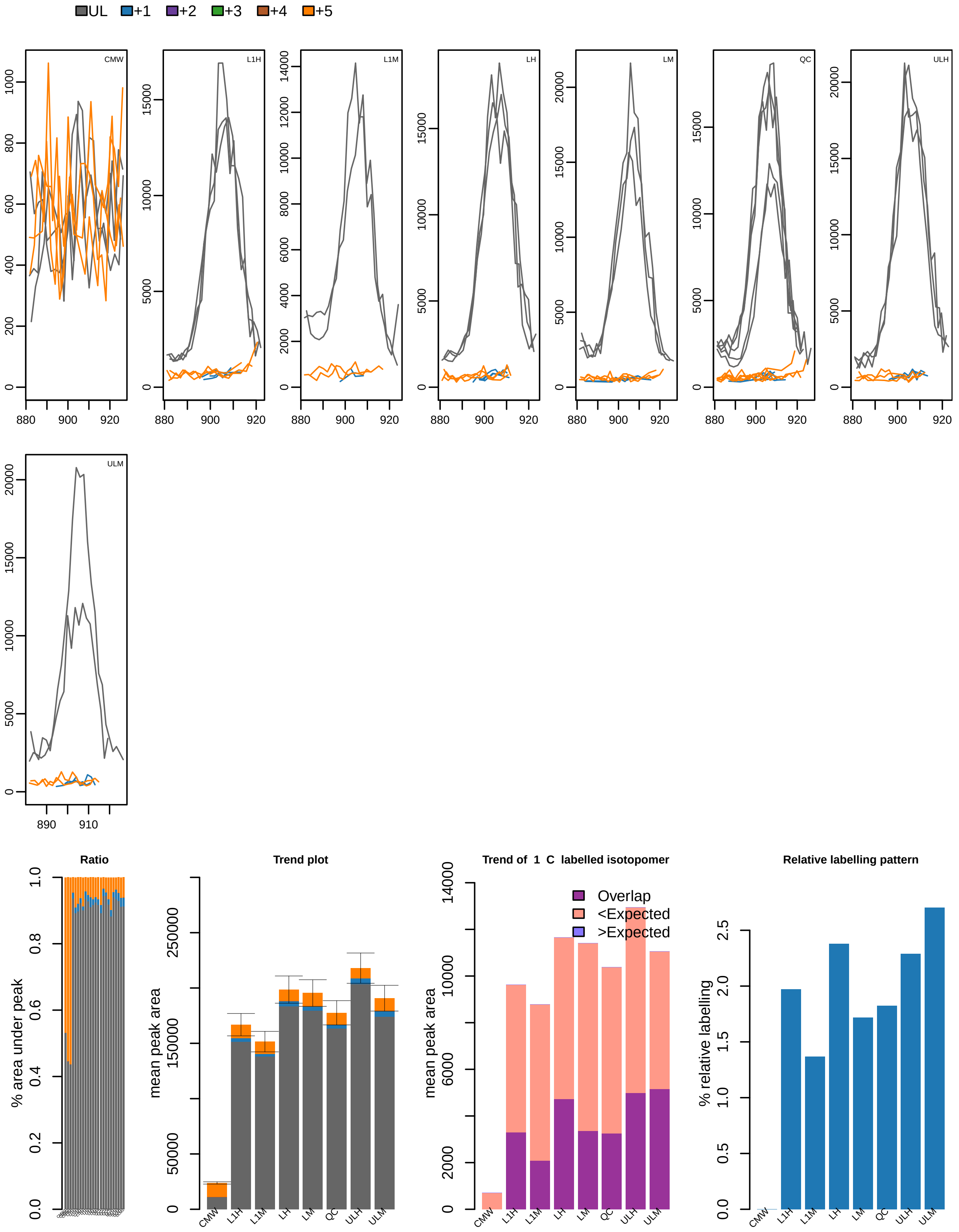

butylene

Formula: C5H10 Mass: 70.078 Std.RT: 223.7263749 Ion: POS

G1

UL +1 +2 +3 +4 +5

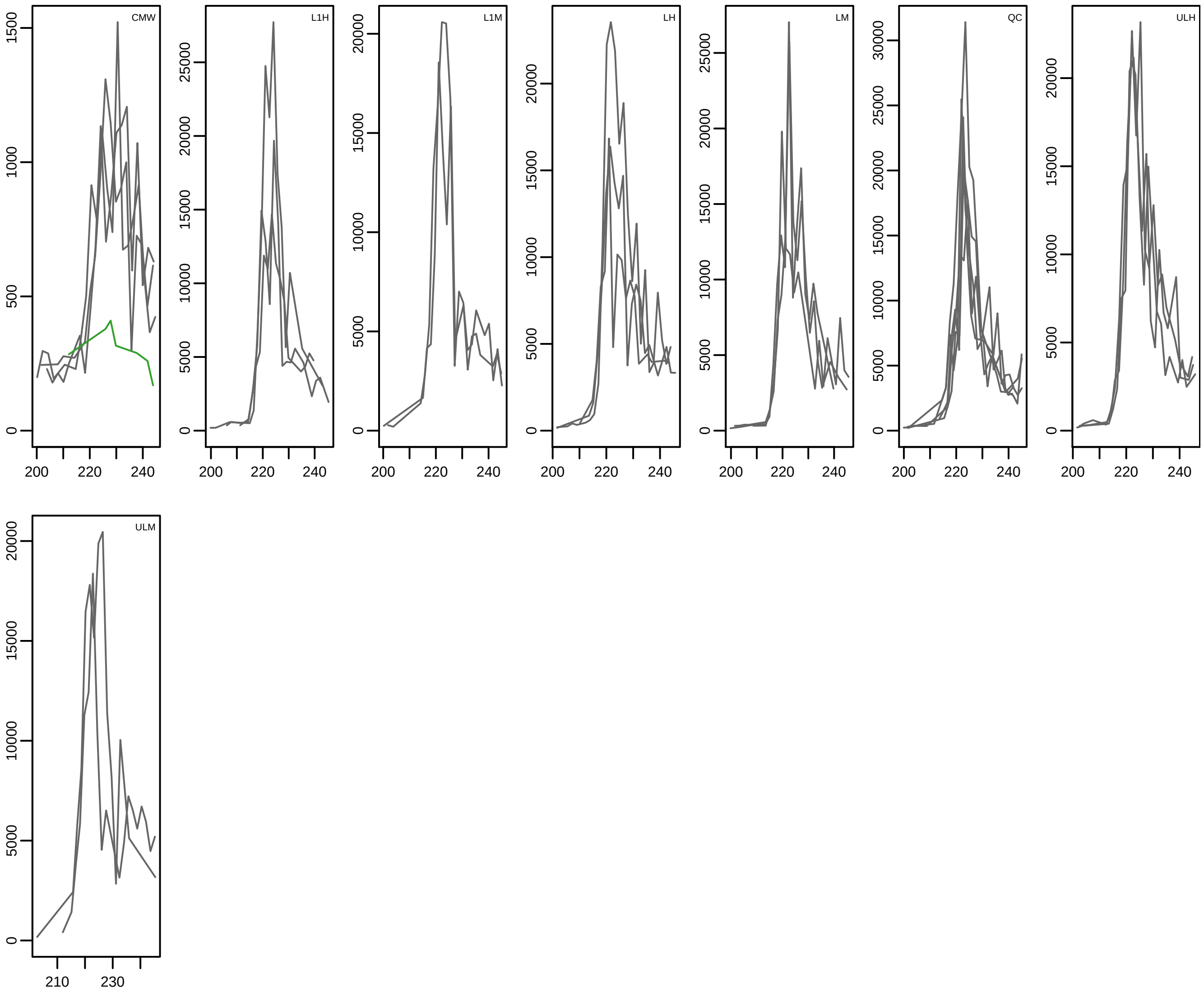

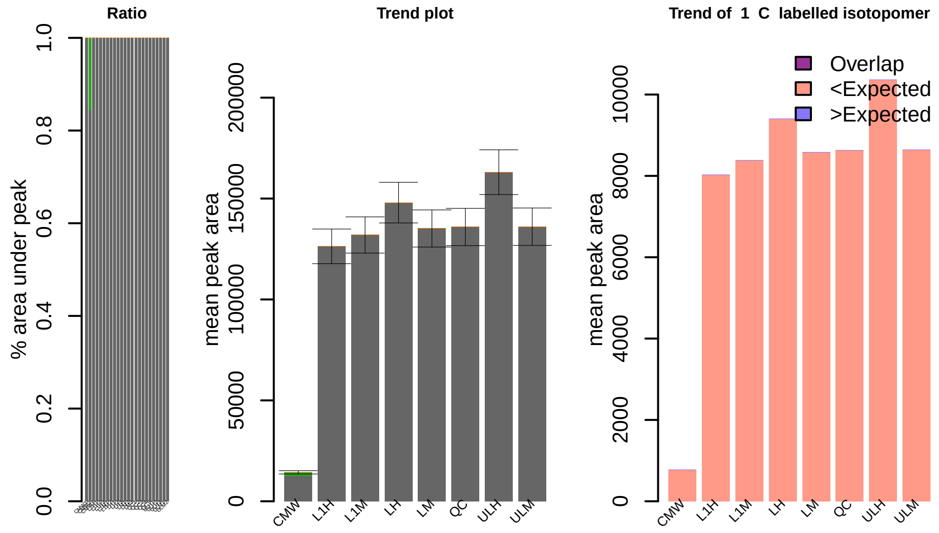

Ovothiol A

Formula: C7H11N3O2S Mass: 201.057 Std.RT: 1005.649995 Ion: PC

G1

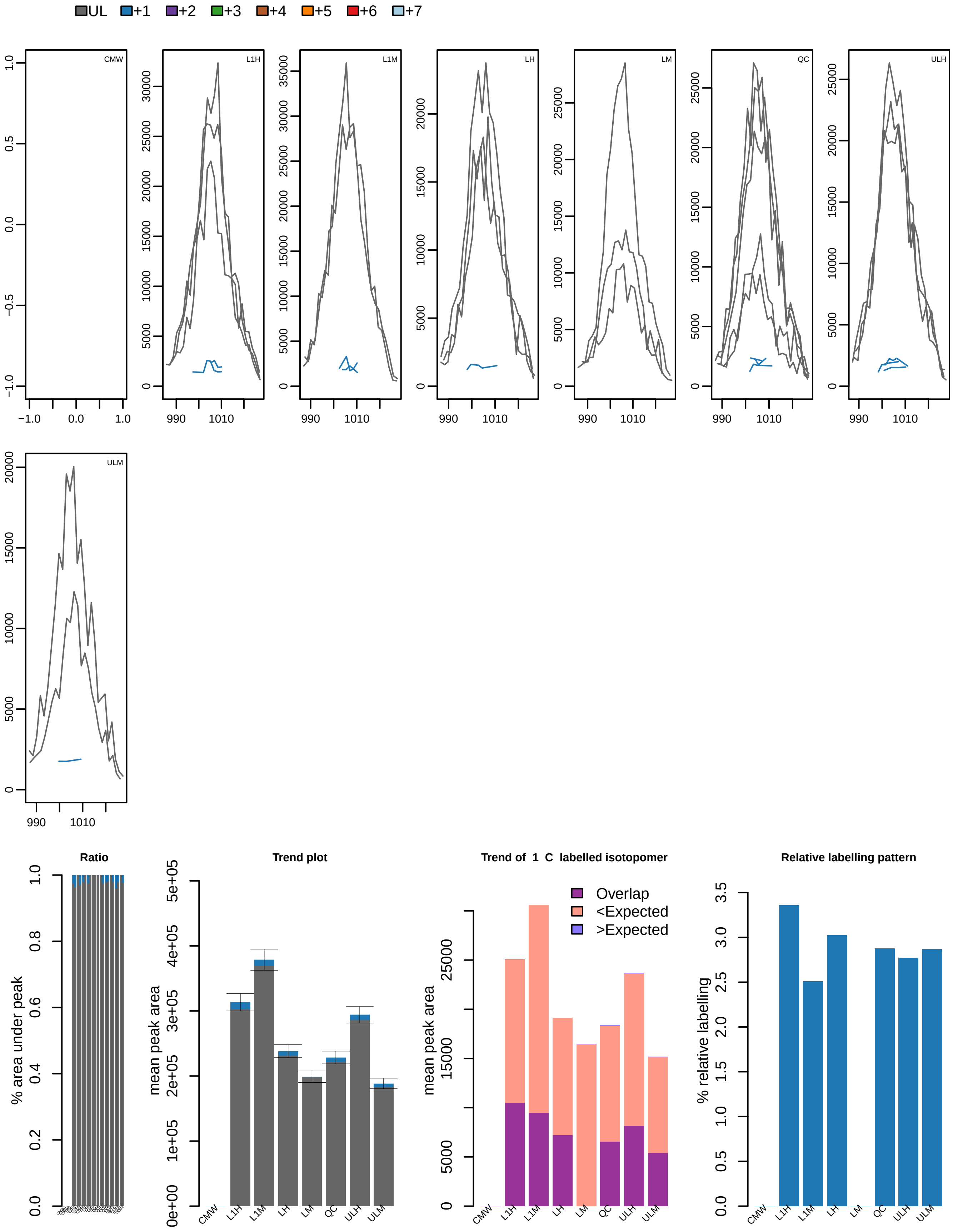

1−Methylhypoxanthine

Formula: C6H6N4O Mass: 150.054 Std.RT: 555.4119219 Ion: POS

G1

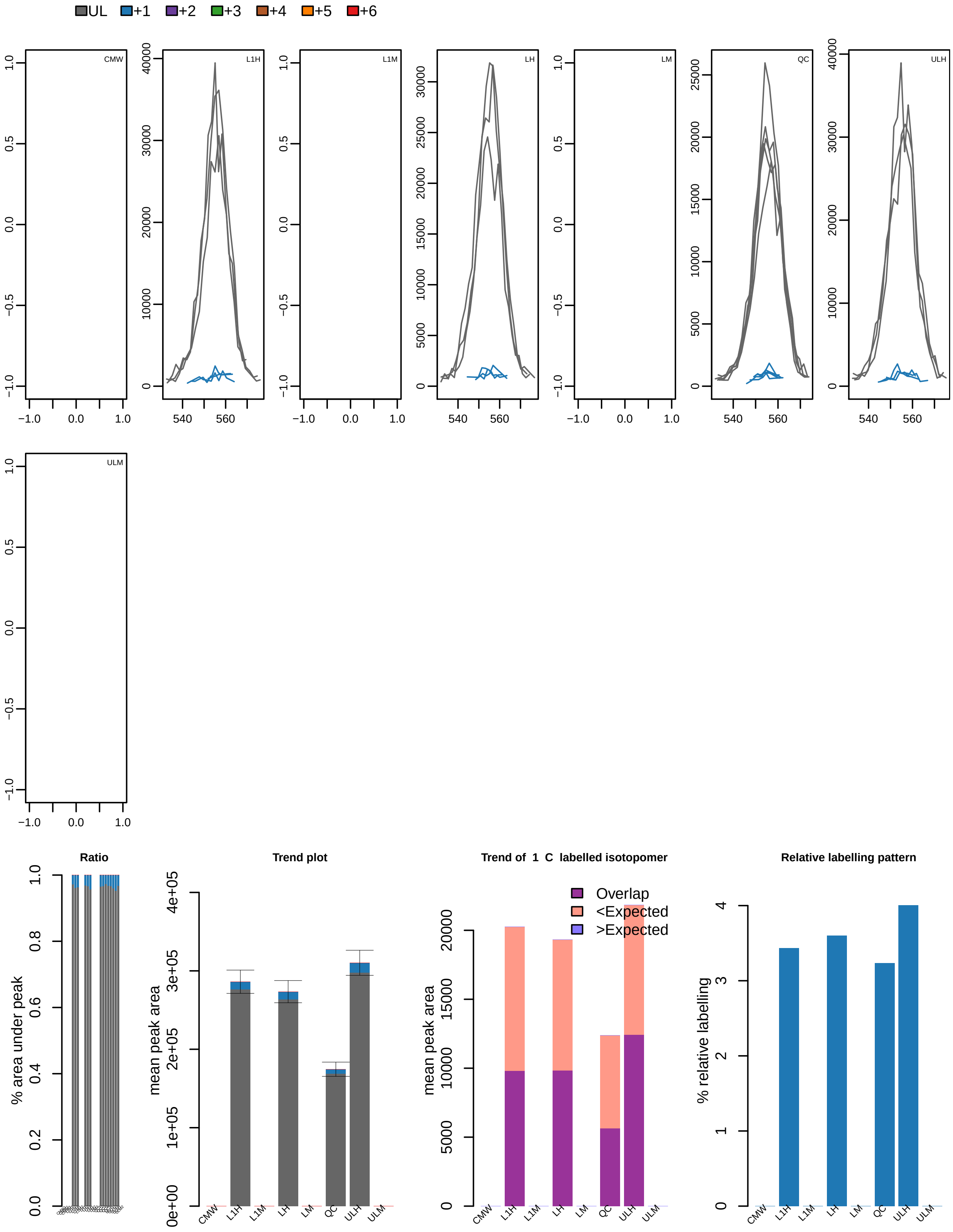

N−Acetyl−beta−D−glucosaminylamine

Formula: C8H16N2O5 Mass: 220.106 Std.RT: 704.1779928 Ion: POS

G1

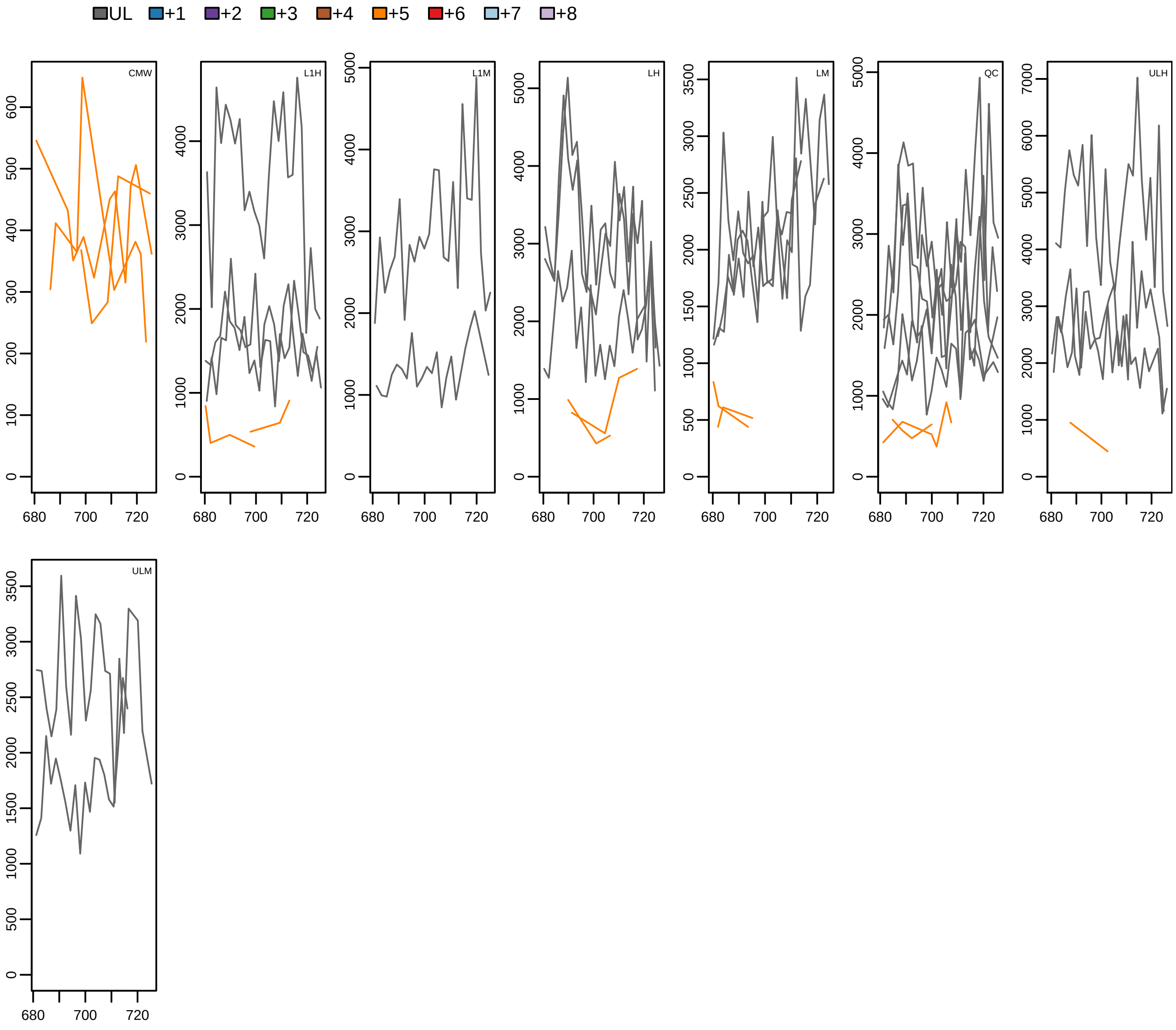

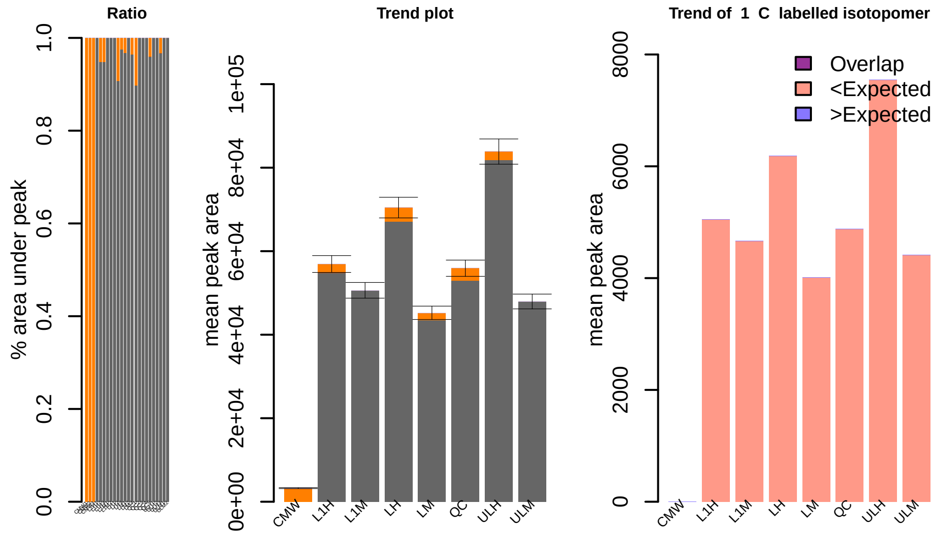

NG,NG−Dimethyl−L−arginine

Formula: C8H18N4O2 Mass: 202.143 Std.RT: 1382.1171468 Ion: PC

G1

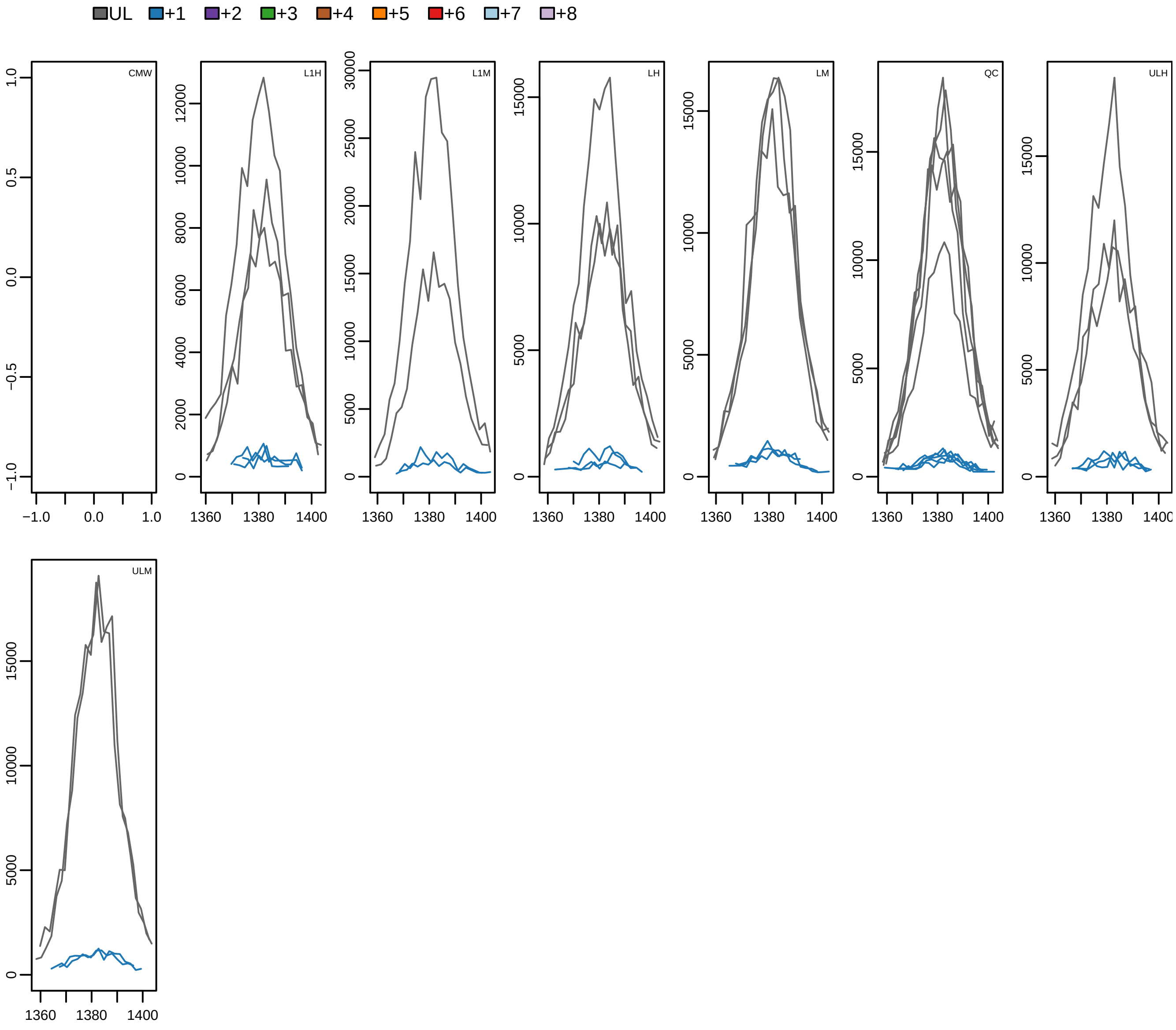

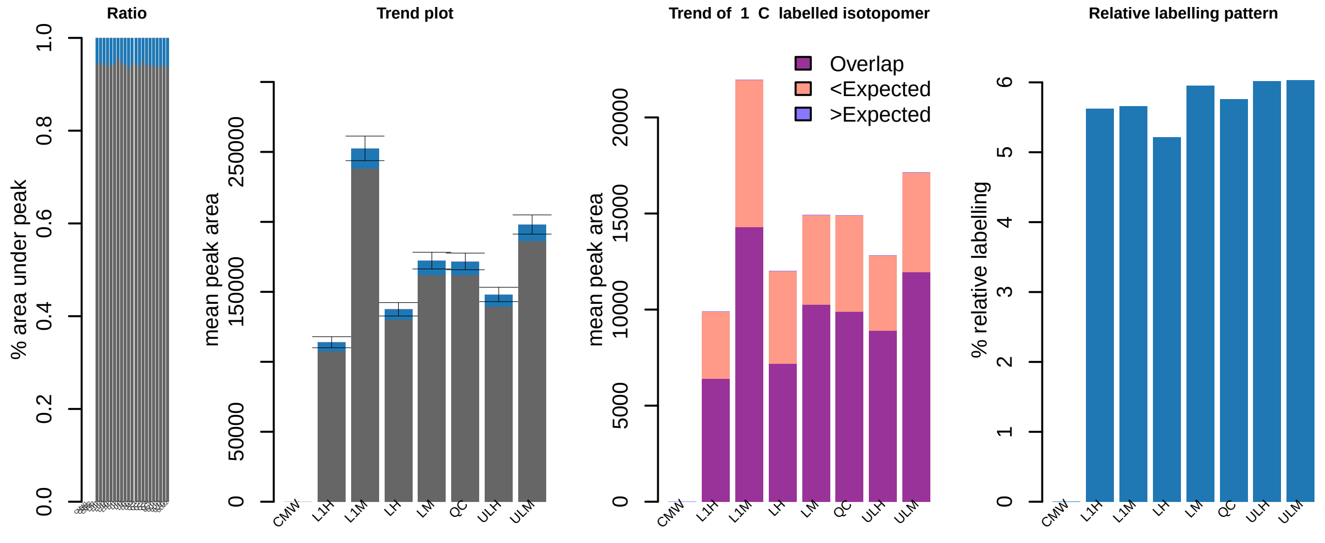

7,8−benzoflavone

G1

Formula: C19H12O2 Mass: 272.084 Std.RT: 303.71722242 Ion: POS

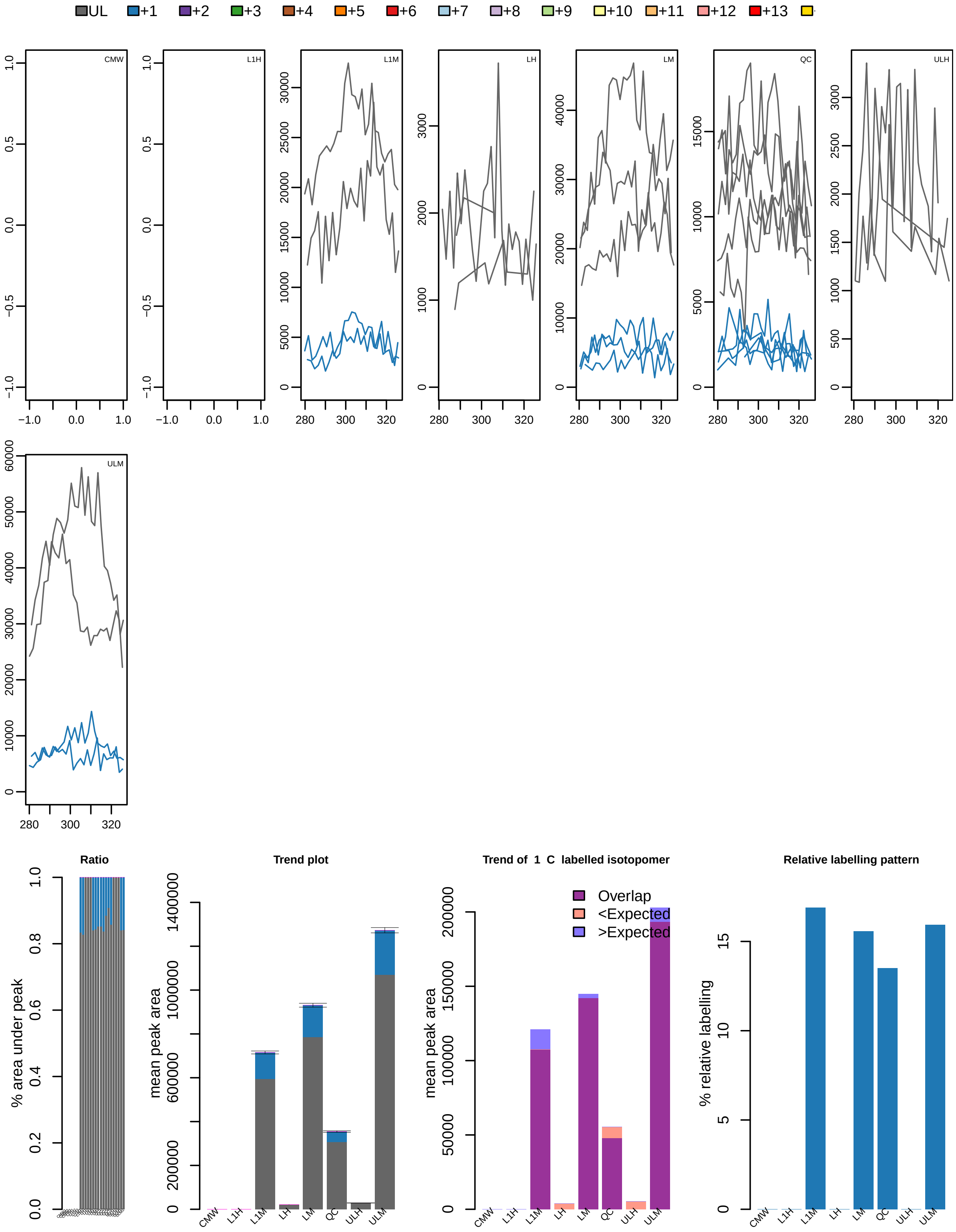

7,8−benzoflavone

Formula: C19H12O2 Mass: 272.084 Std.RT: 303.71722242 Ion: POS

G2

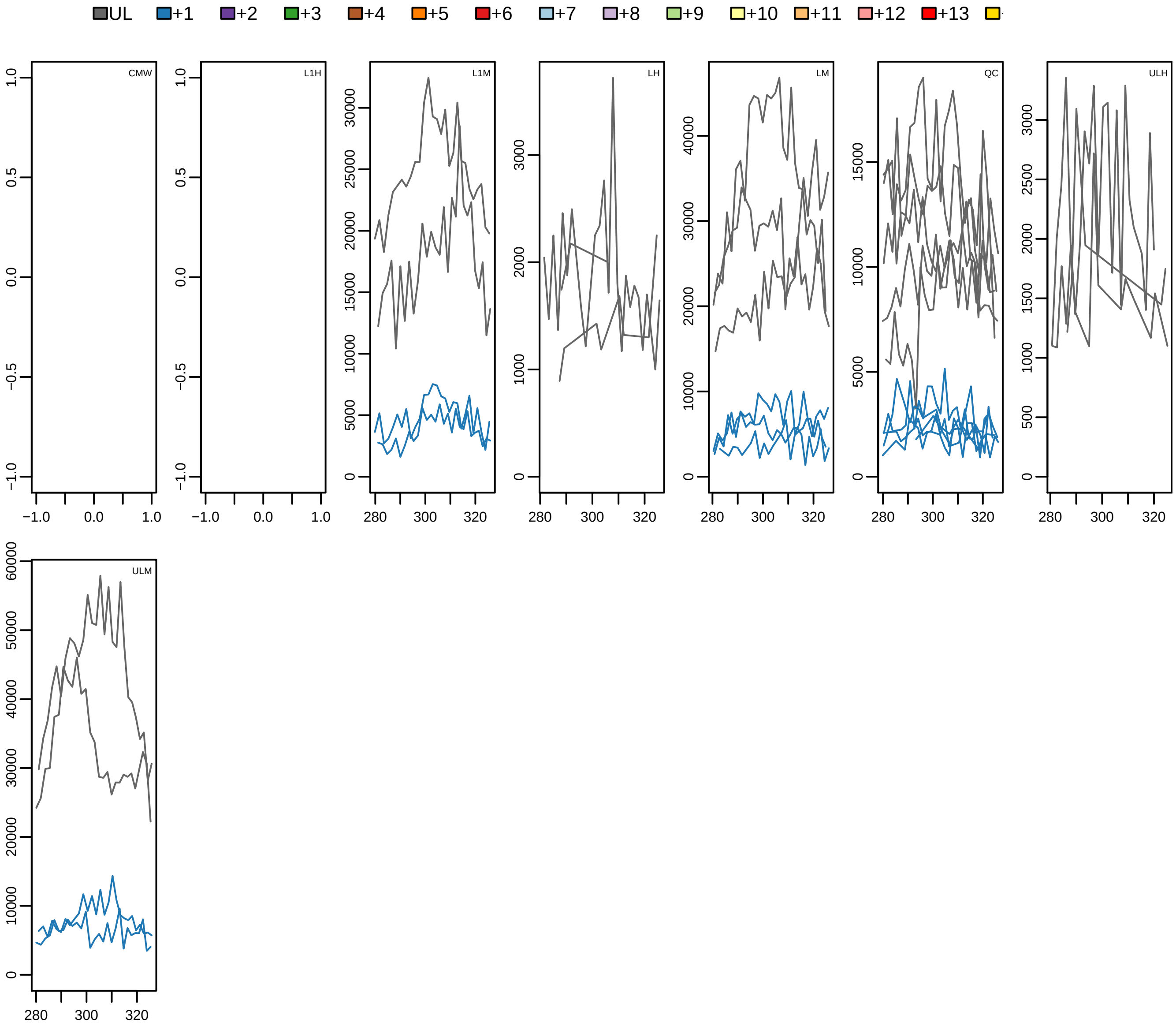

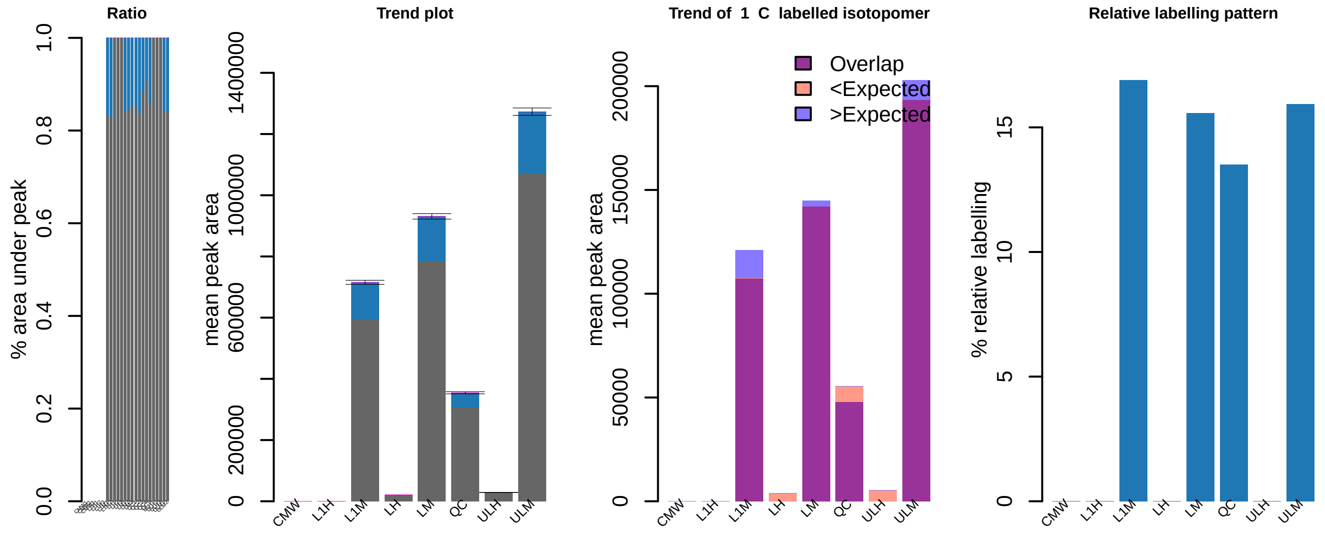

Ne,Ne dimethyllysine

Formula: C8H18N2O2 Mass: 174.137 Std.RT: 1362.8071578 Ion: PC

G1

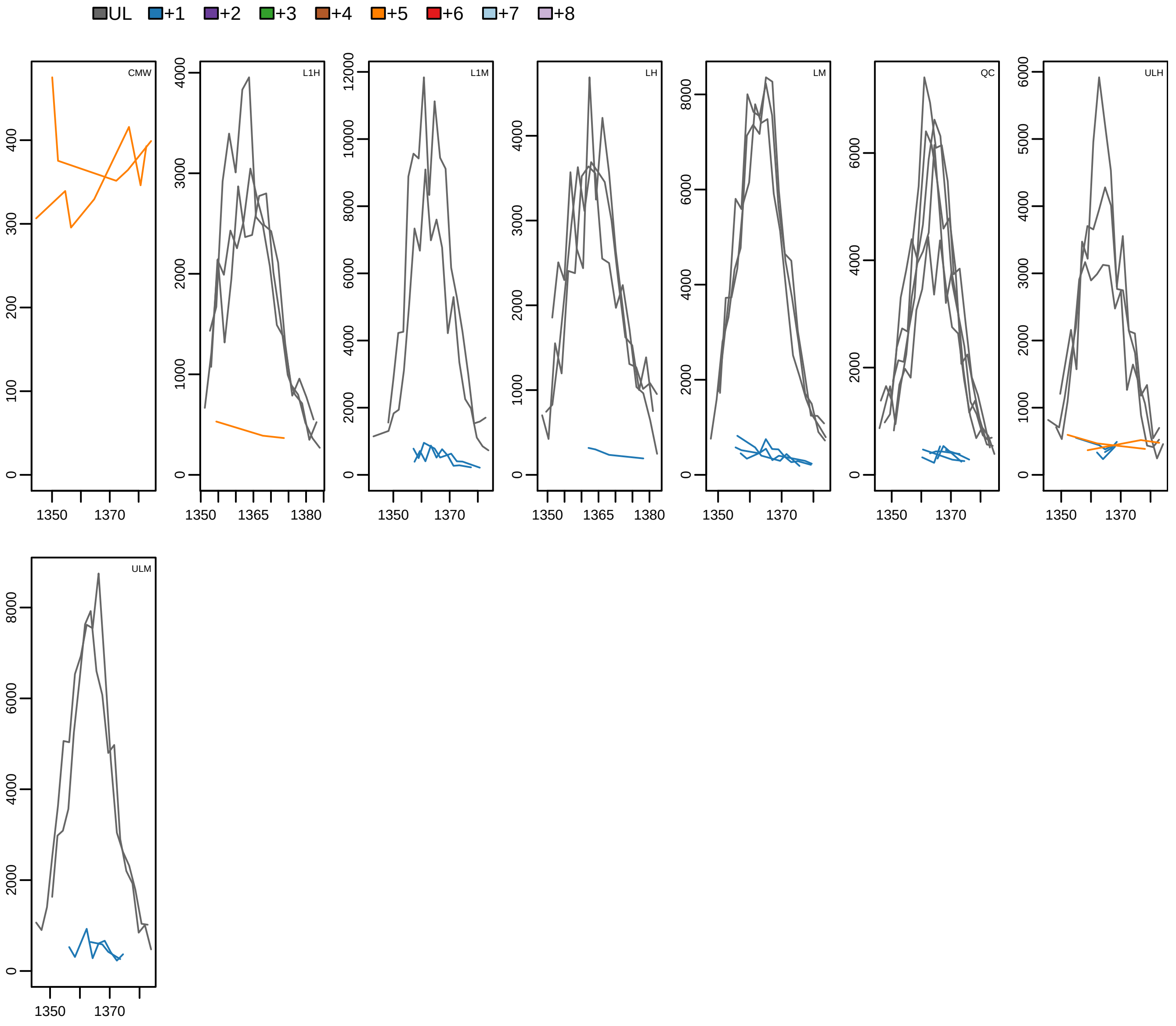

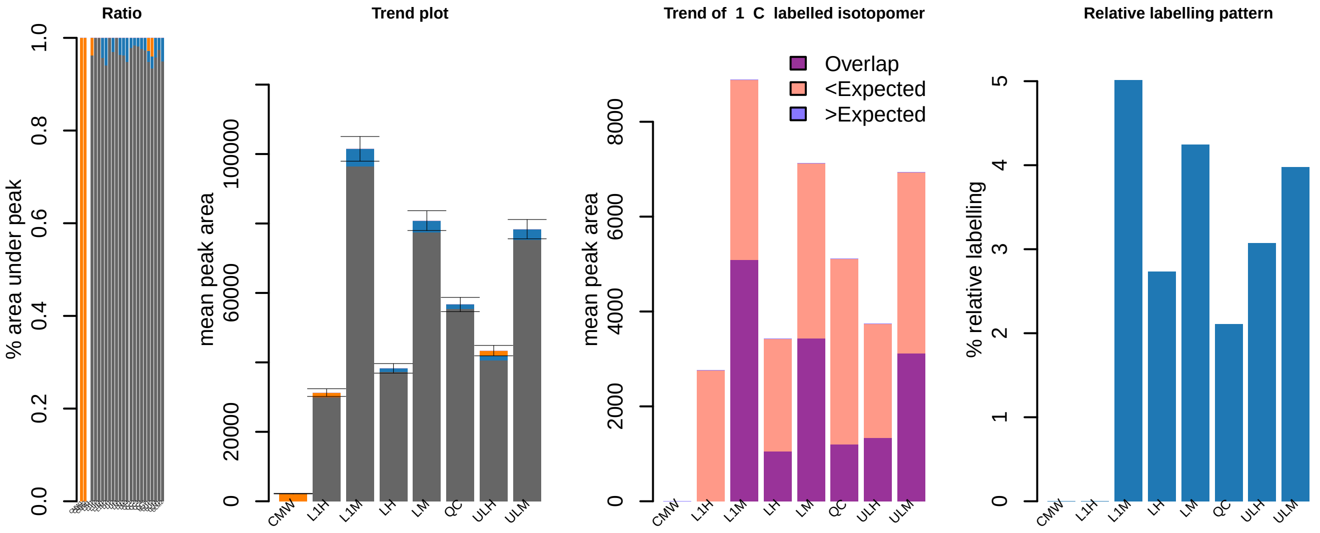

3,4−Dehydrothiomorpholine−3−carboxylate

Formula: C5H7NO2S Mass: 145.02 Std.RT: 433.70433348 Ion: POS

G1

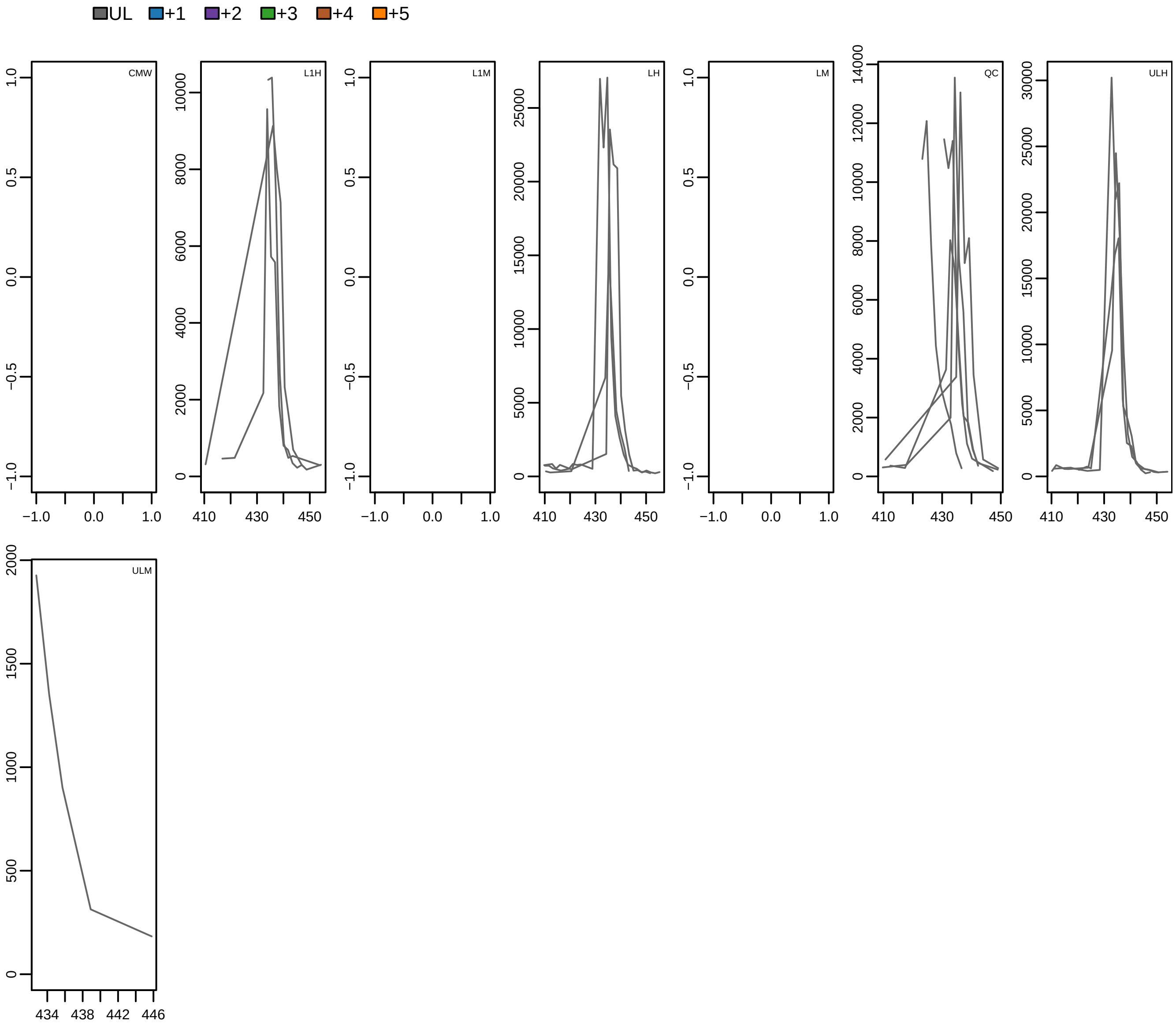

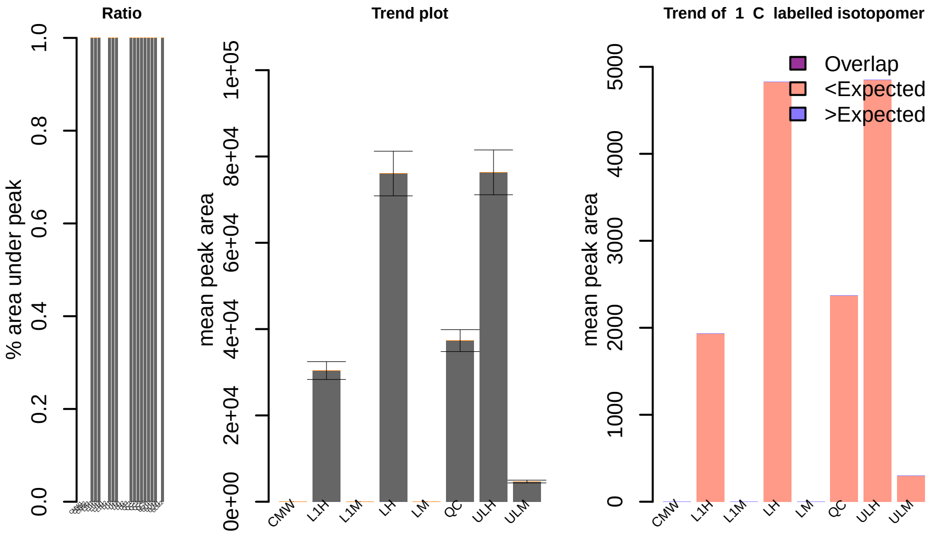

N−Acetylglutamine

Formula: C7H12N2O4 Mass: 188.08 Std.RT: 660.3057222 Ion: POS

G1

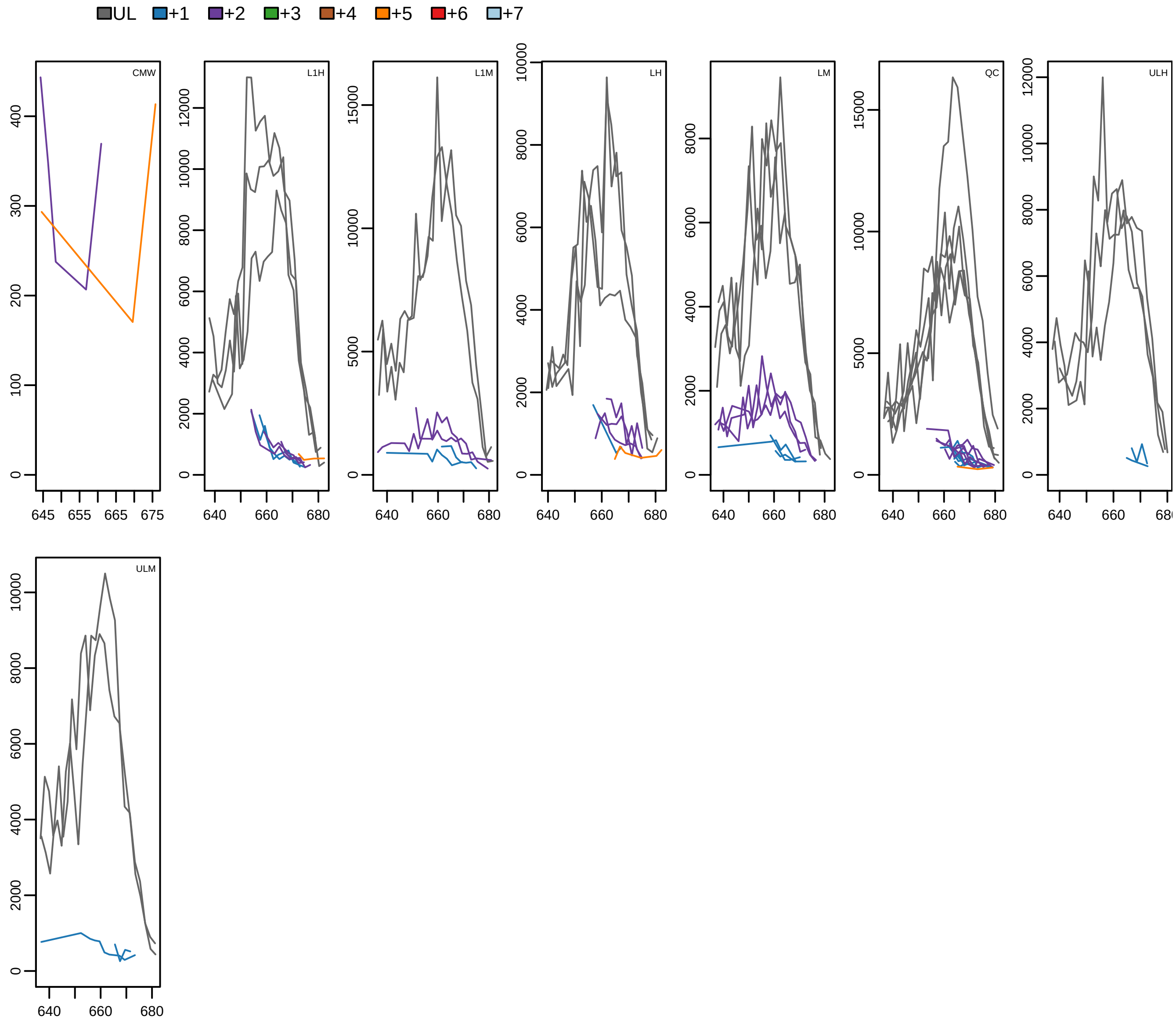

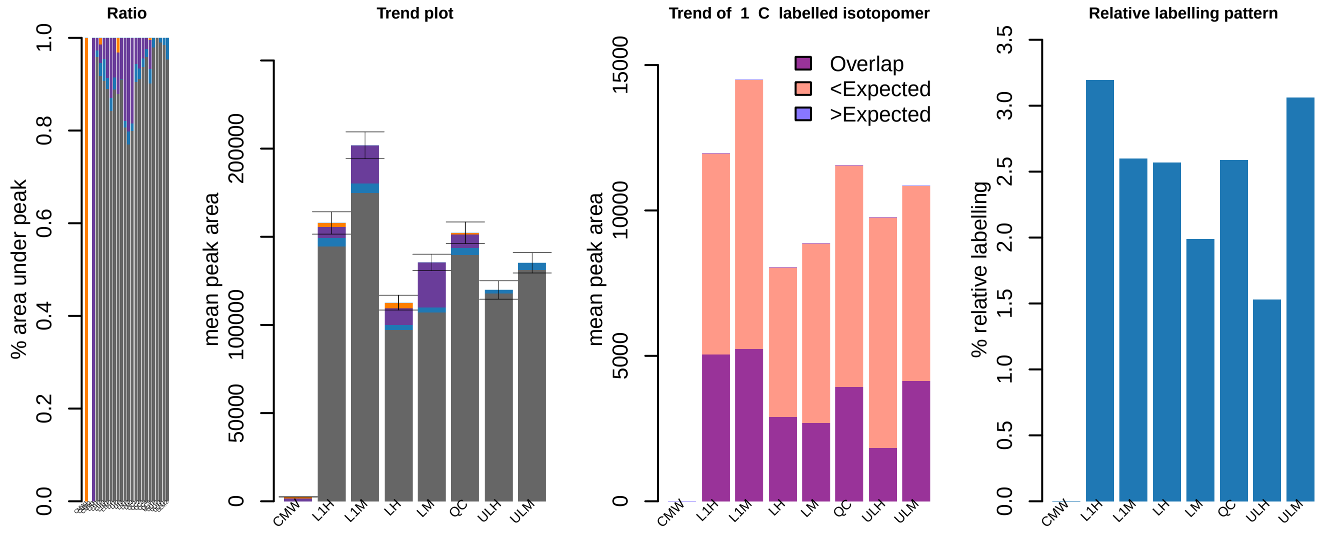

N−Acetylglutamine

Formula: C7H12N2O4 Mass: 188.08 Std.RT: 660.3057222 Ion: POS

G2

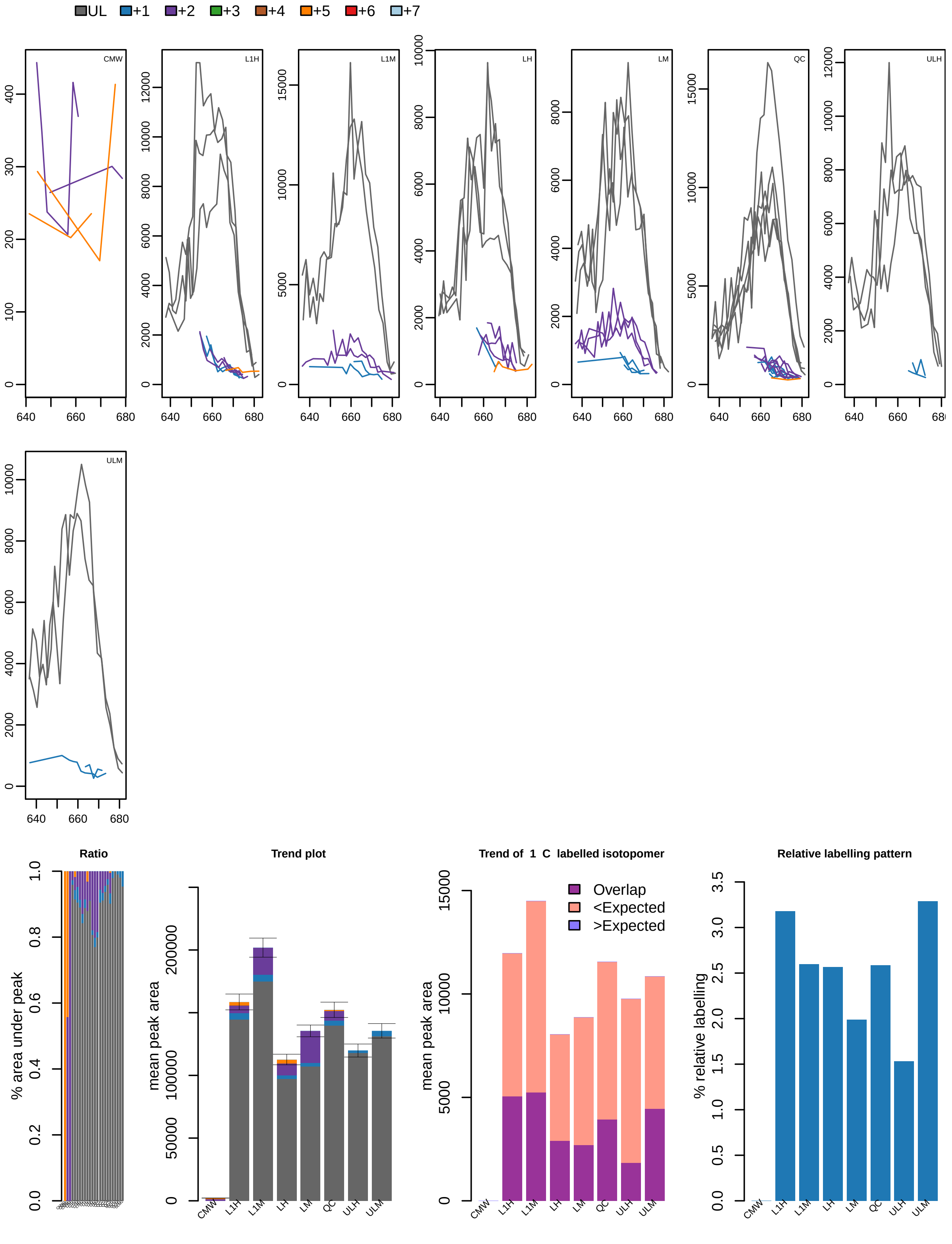

N6−Methyl−2−deoxyadenosine

Formula: C11H15N5O3 Mass: 265.117 Std.RT: 728.3825712 Ion: PC

G1

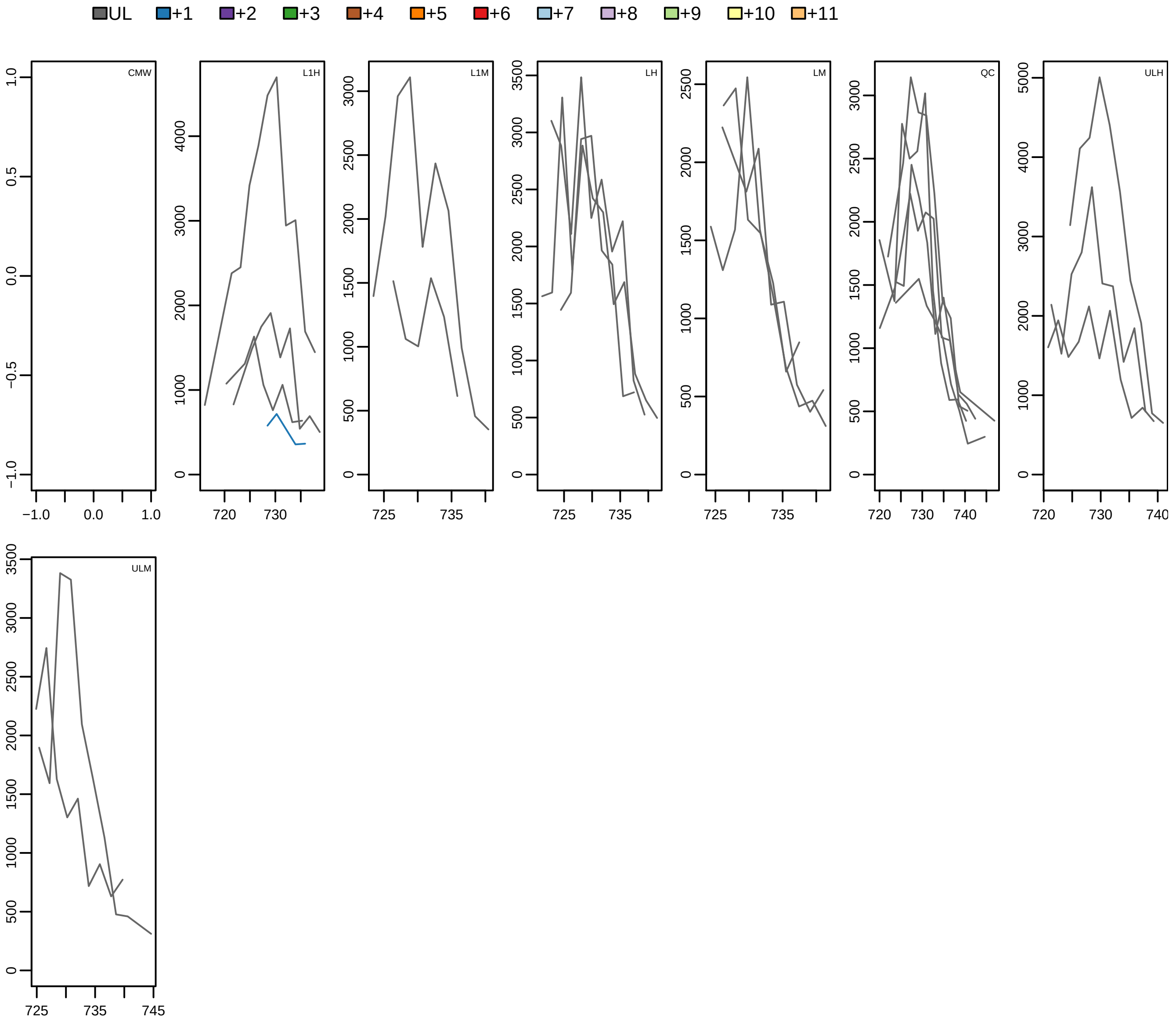

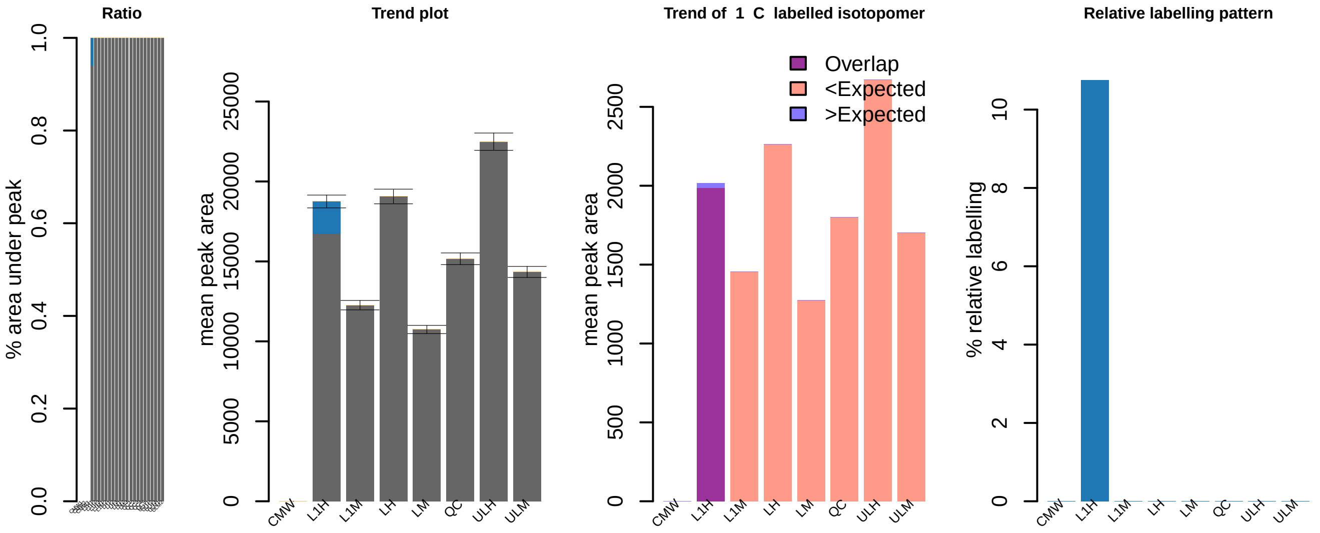

Methylcysteine

Formula: $C4H9NO2S$ Mass: 135.035 Std.RT: 745.8365652 Ion: POS

G1

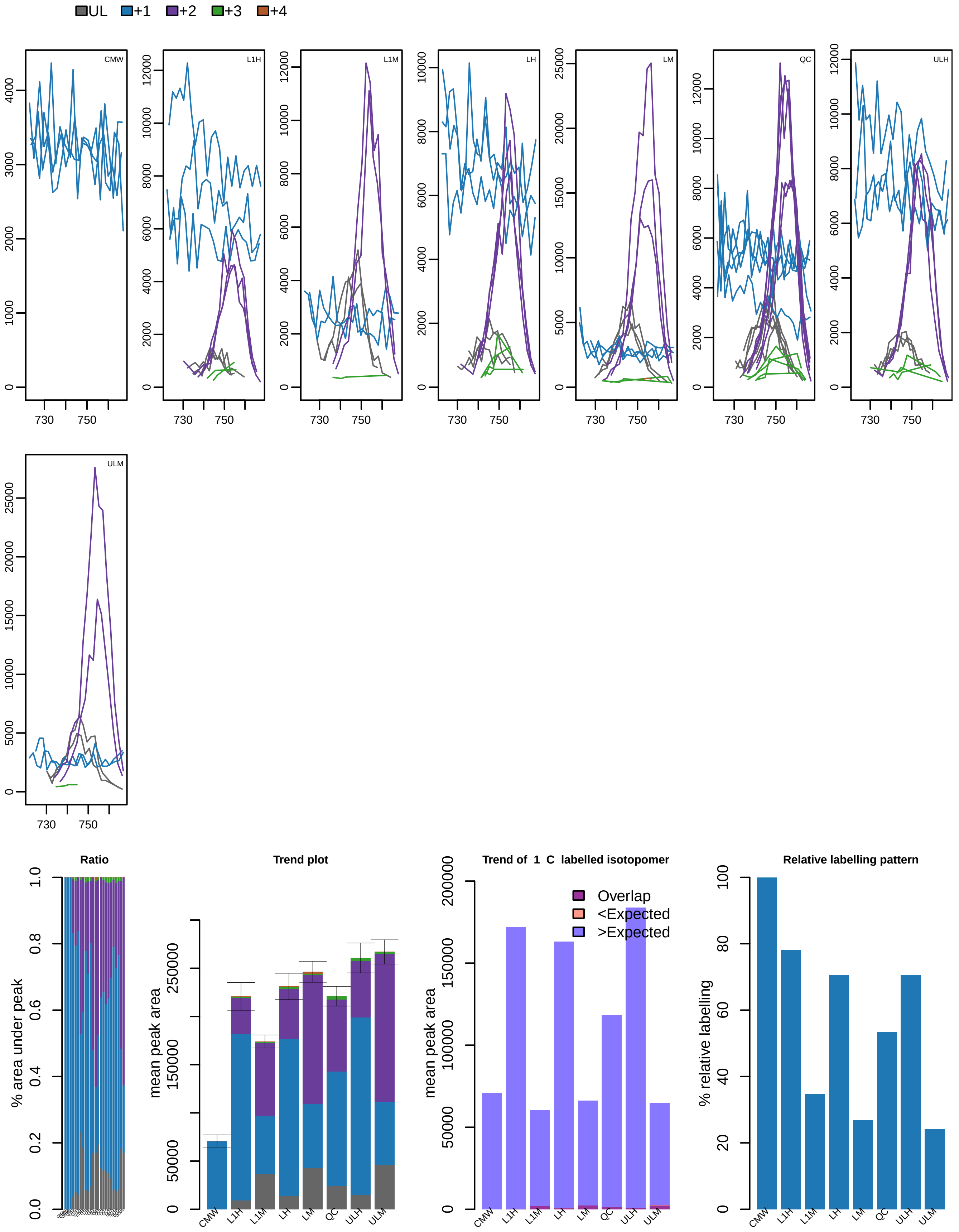

histidine methyl ester

Formula: C7H11N3O2 Mass: 169.085 Std.RT: 450.57383088 Ion: PC

G1

UL +1 +2 +3 +4 +5 +6 +7

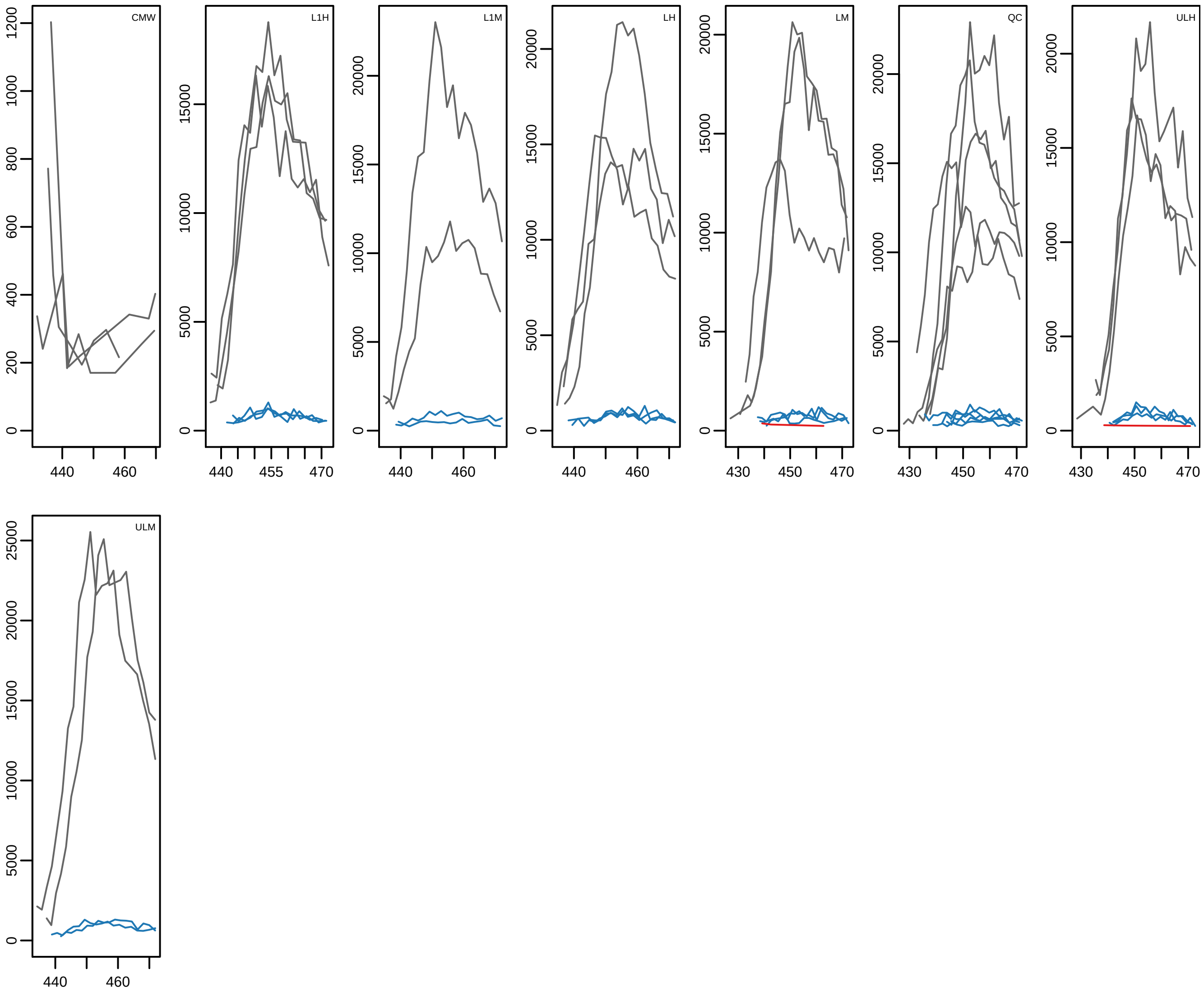

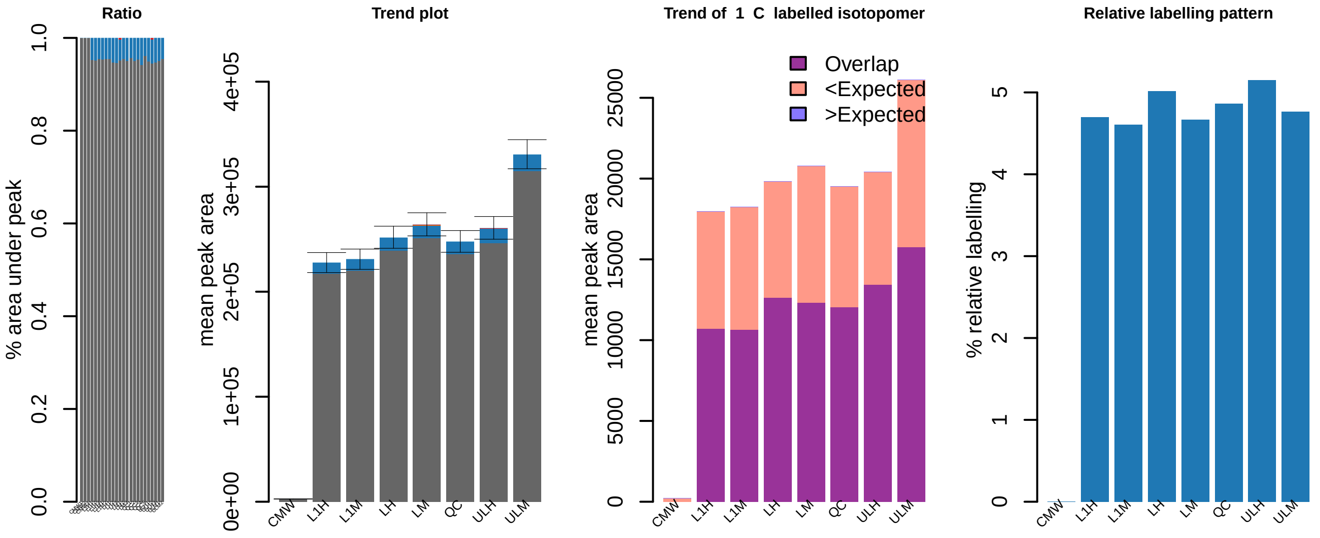

Sinalbin

Formula: C14H18NO10S2 Mass: 424.037 Std.RT: 974.8717158 Ion:

G1

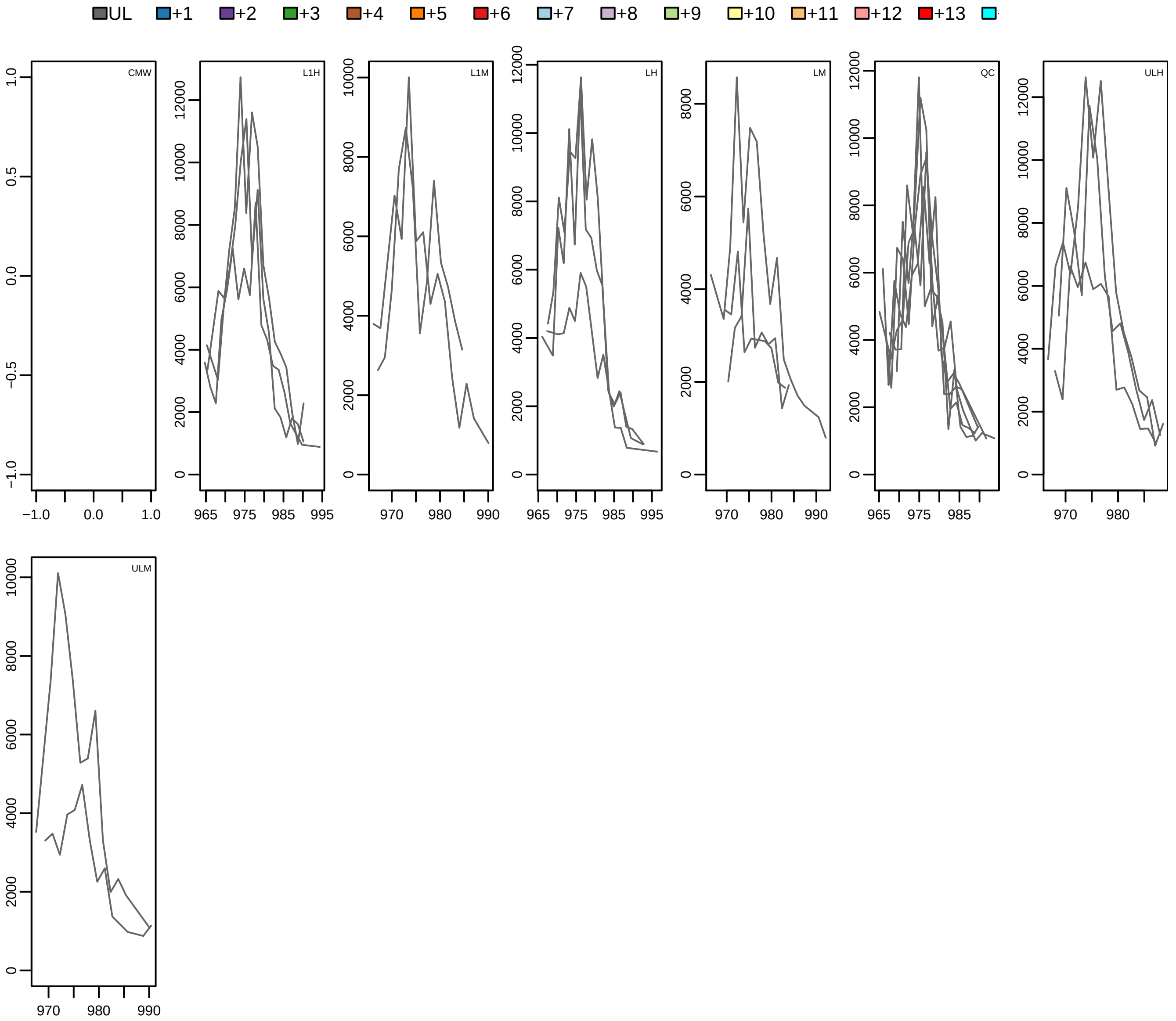

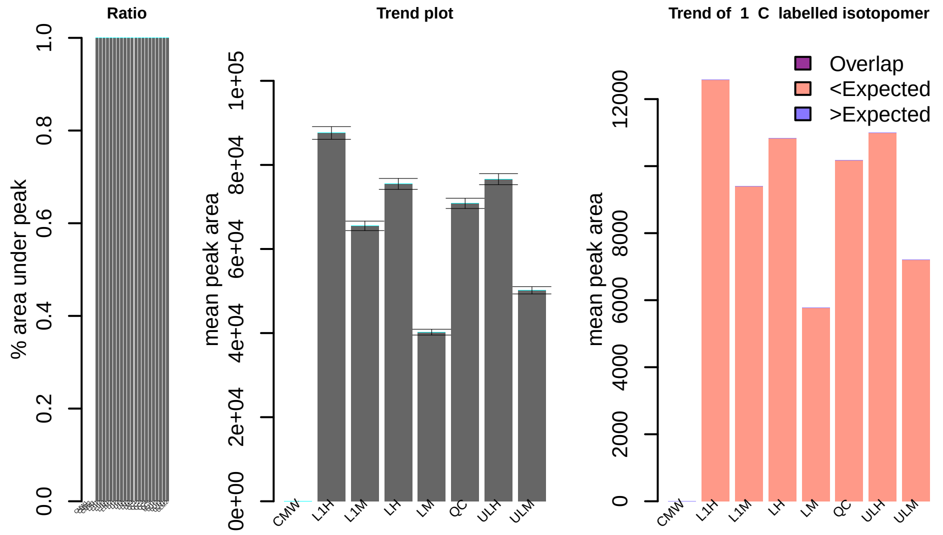

6alpha−Hydroxycortisol

Formula: C21H30O6 Mass: 378.204 Std.RT: 646.9322832 Ion: POS

G1

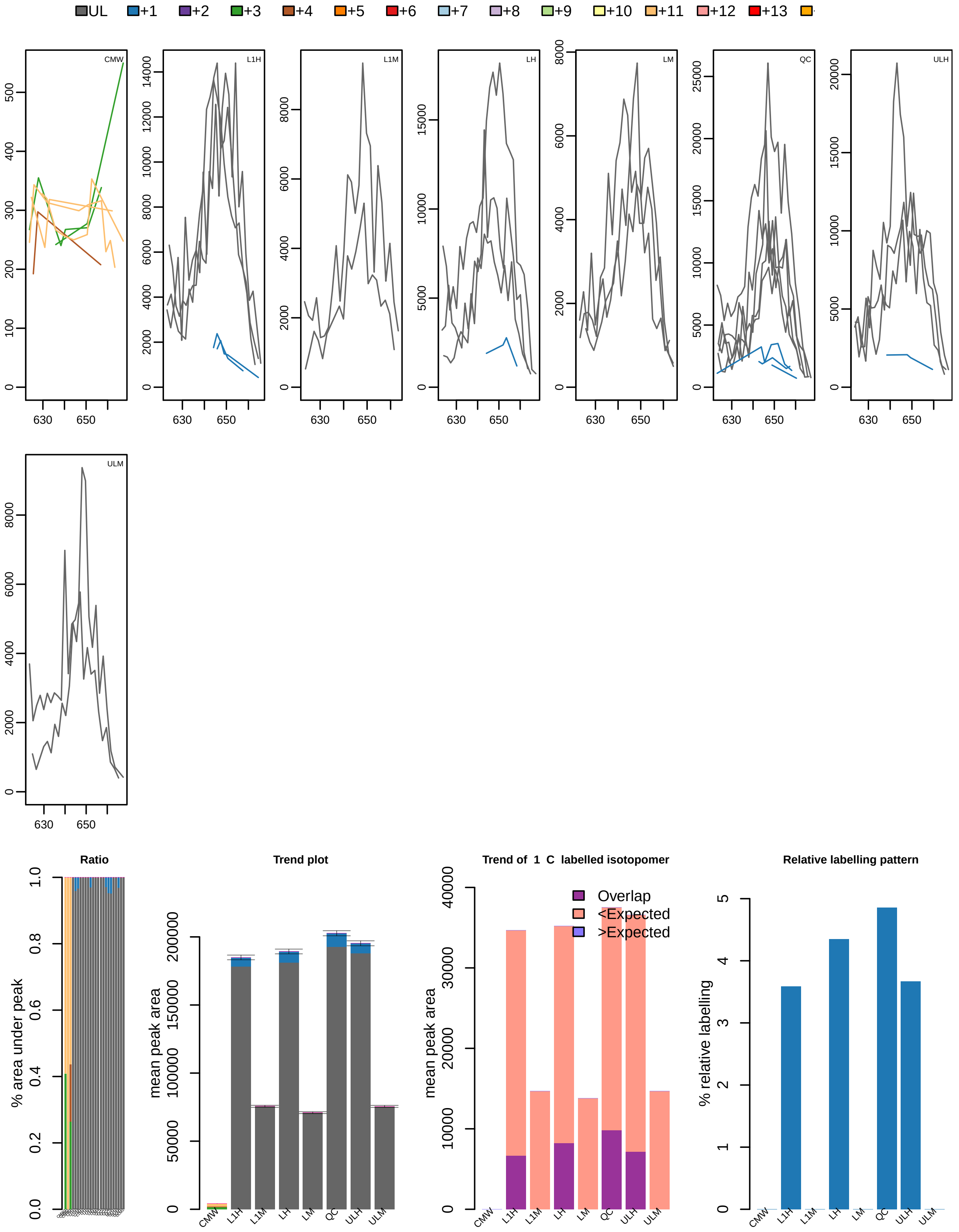

Mefenacet

Formula: C16H14N2O2S Mass: 298.078 Std.RT: 439.9704498 Ion: P

G1

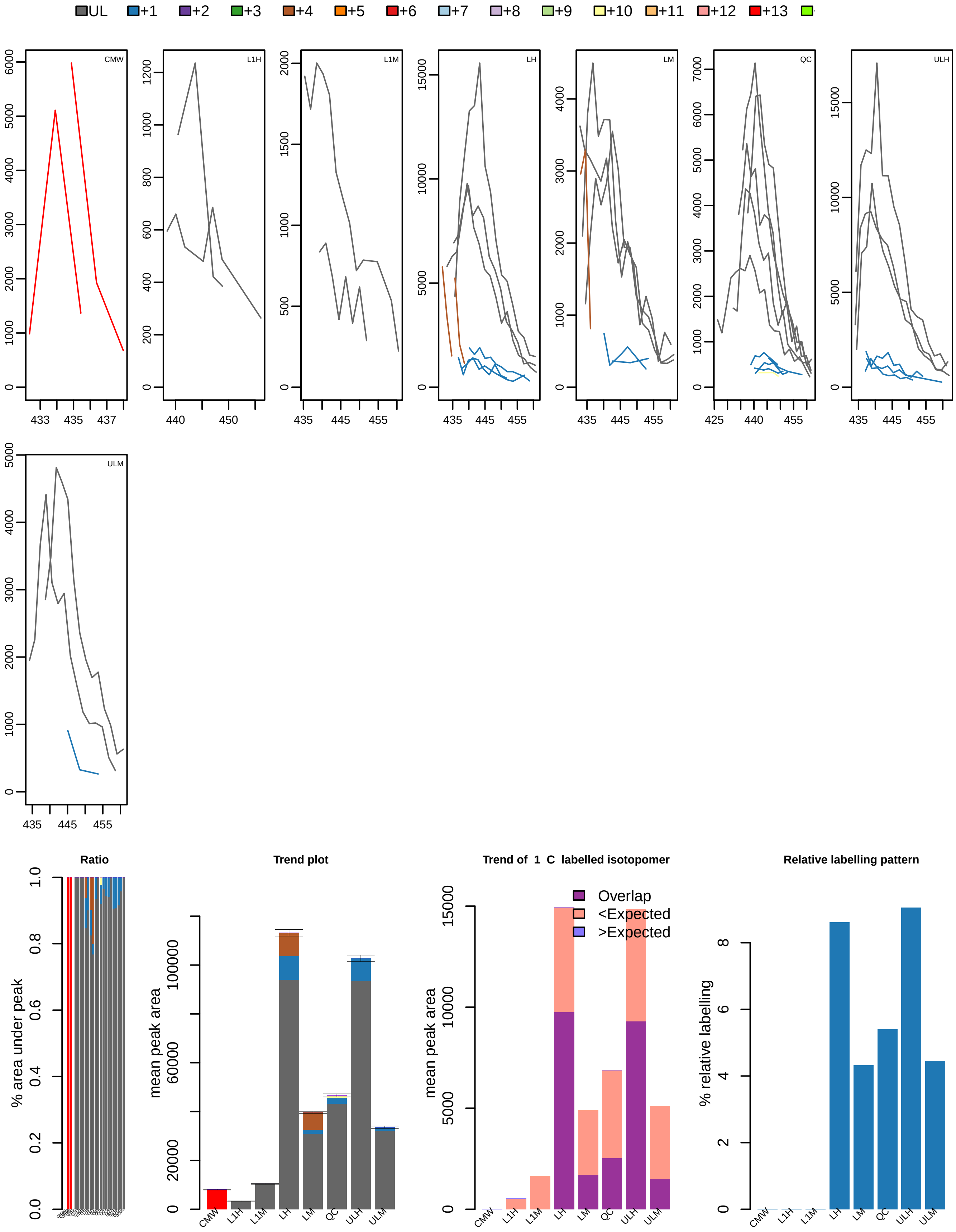

Fagomine

Formula: C6H13NO3 Mass: 147.09 Std.RT: 788.5602468 Ion: POS

G1

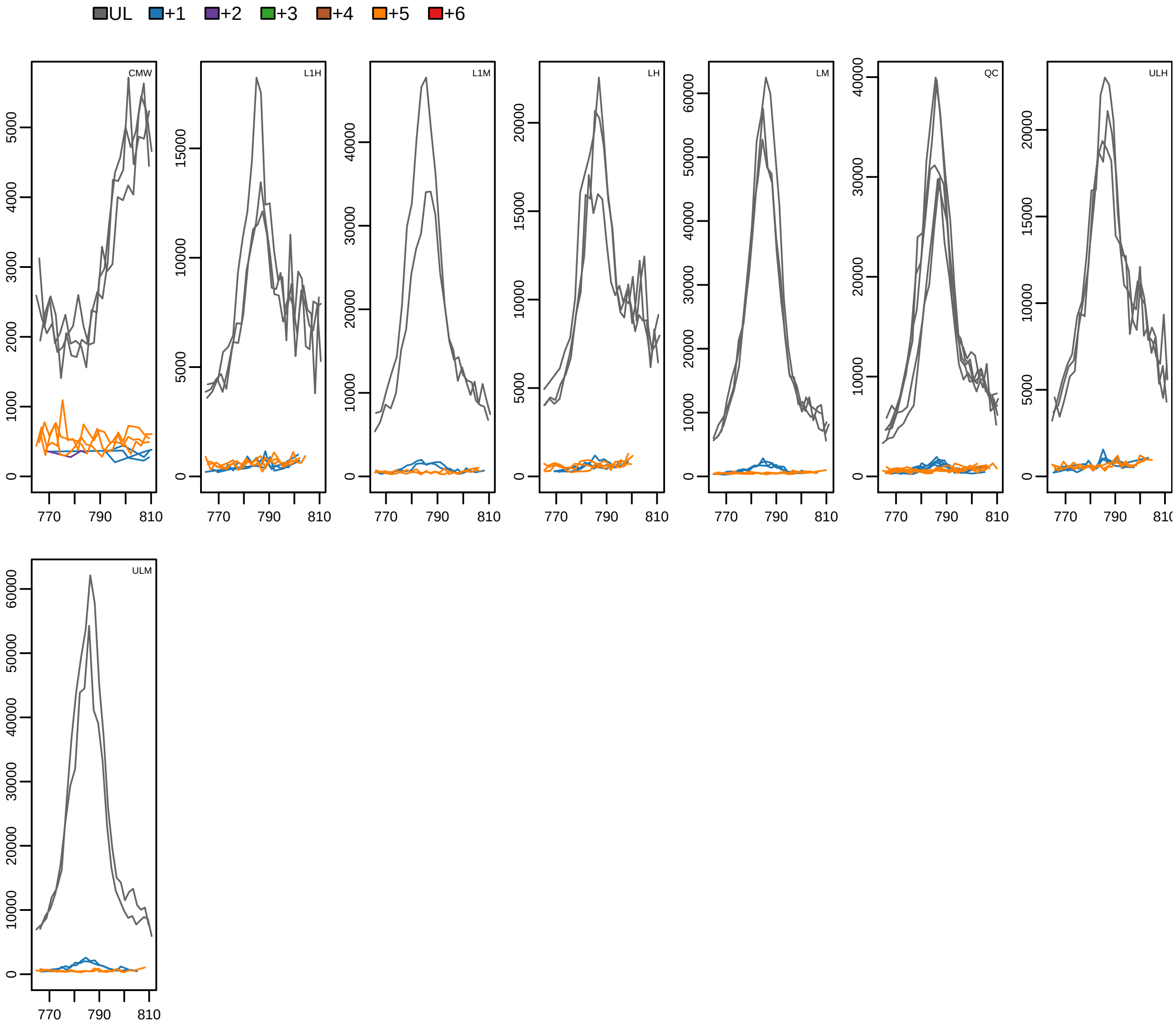

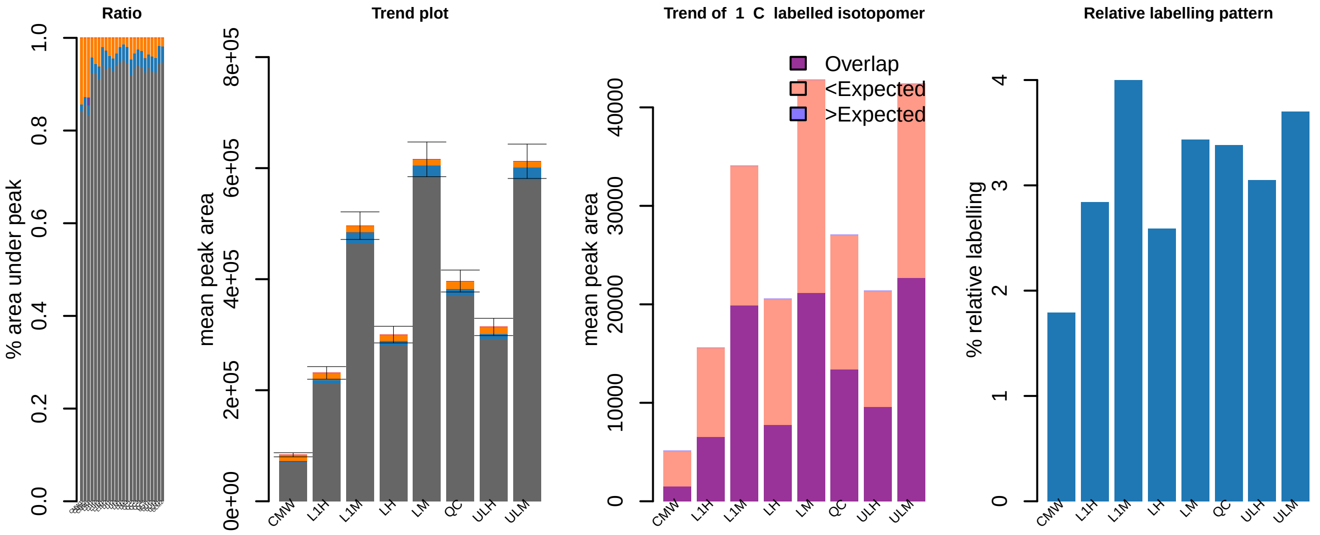

N−Undecanoylglycine

Formula: C13H25NO3 Mass: 243.183 Std.RT: 249.07933302 Ion: PC

G1

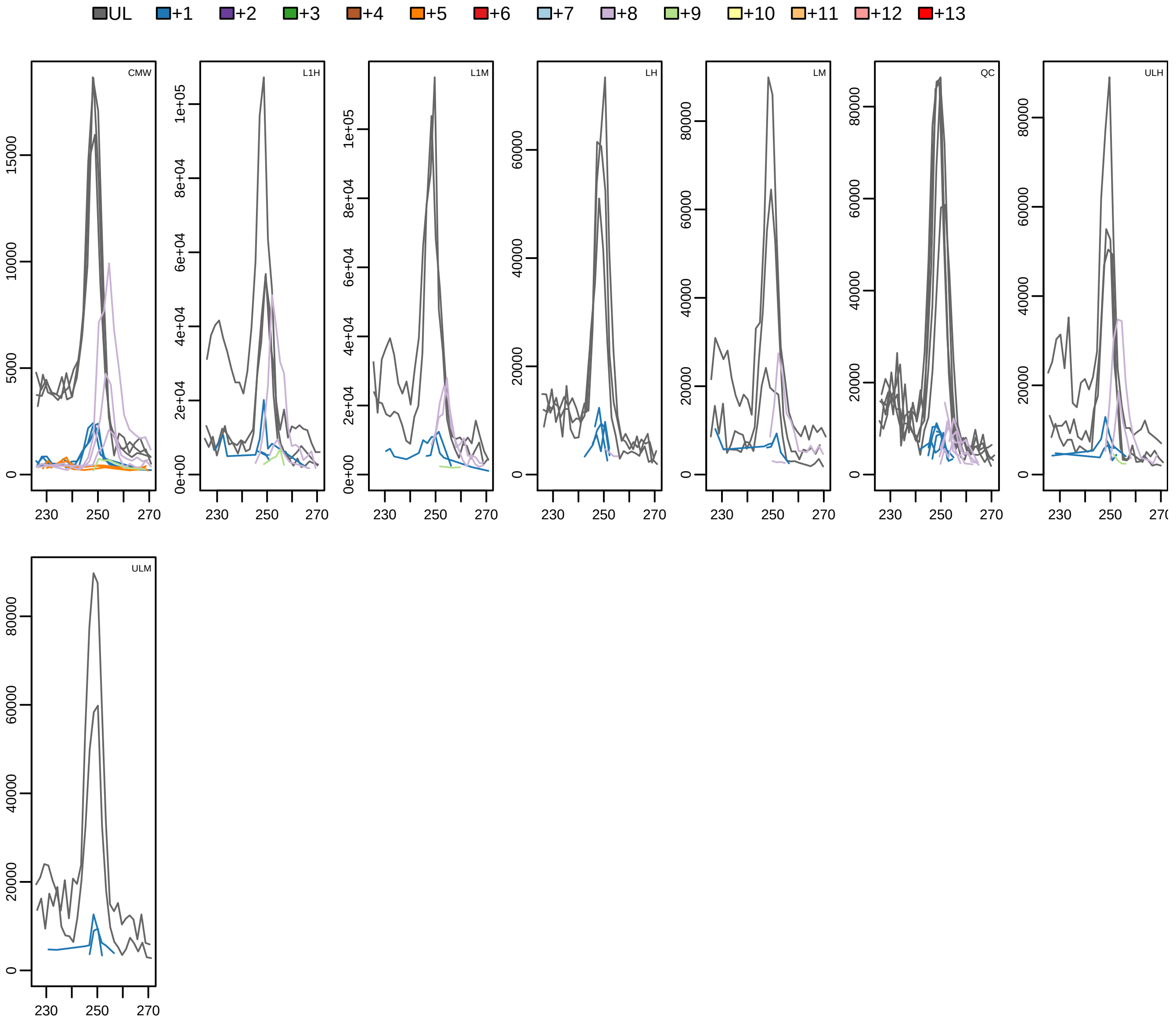

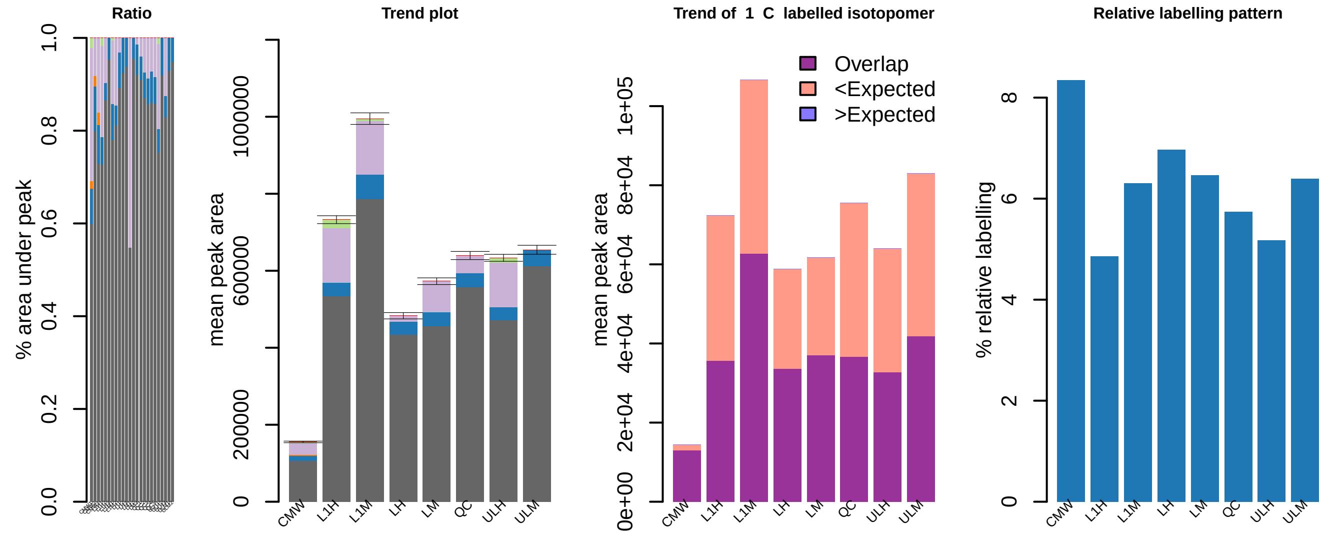

N−Undecanoylglycine

Formula: C13H25NO3 Mass: 243.183 Std.RT: 249.07933302 Ion: PC

G2

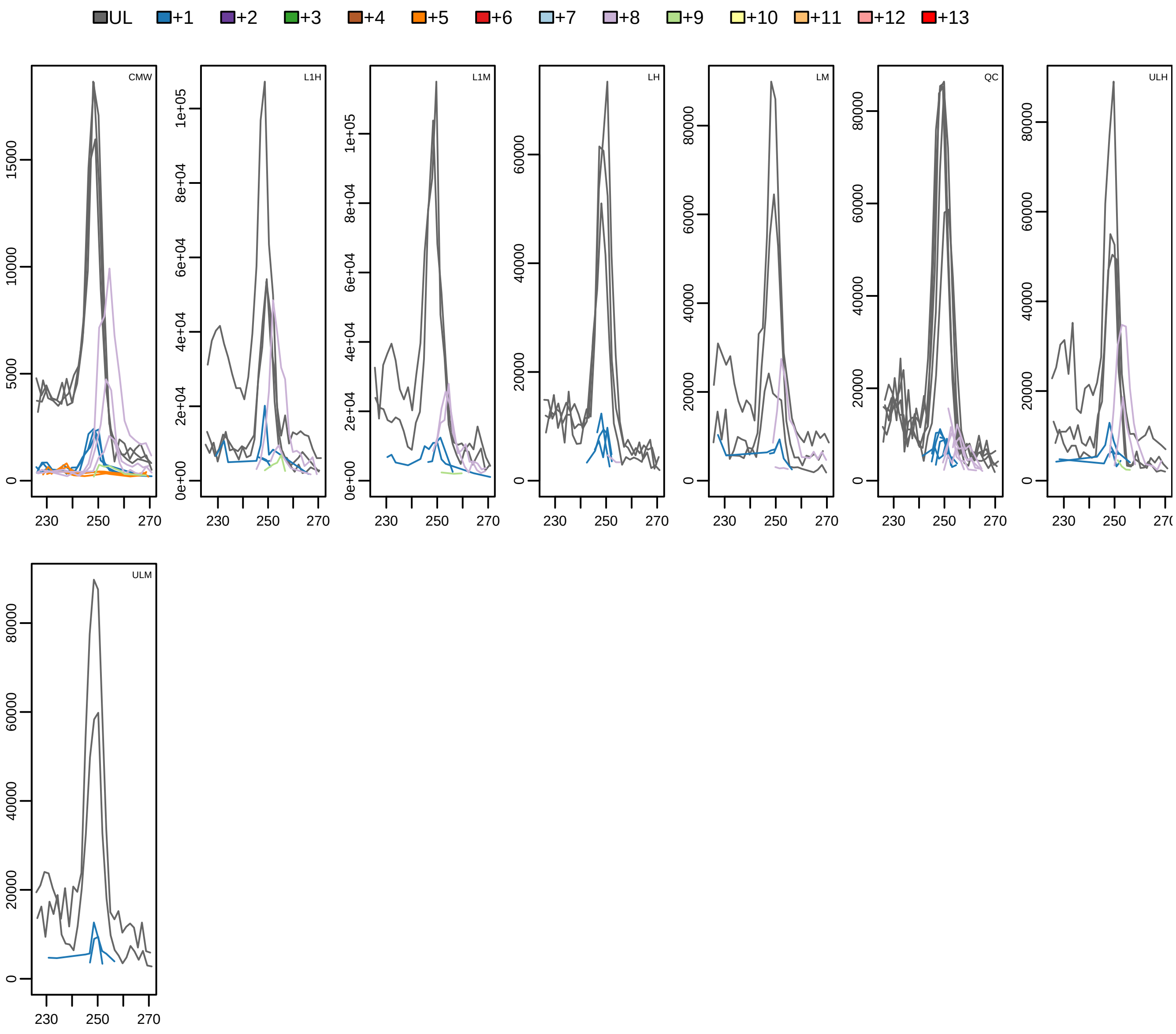

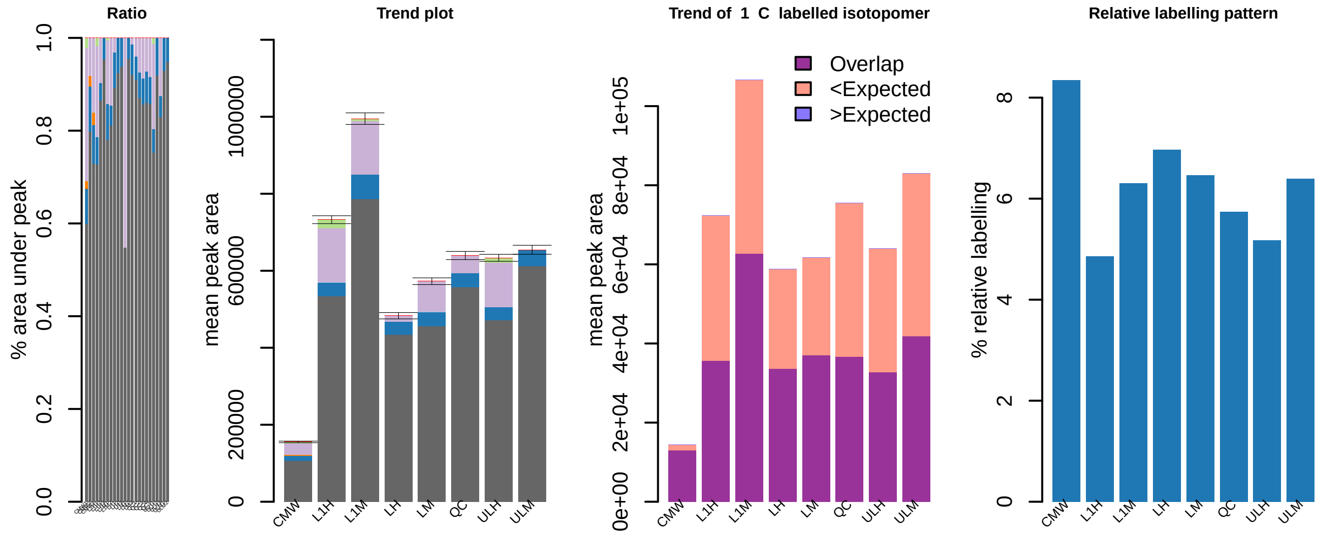

3−Methylguanine

Formula: C6H7N5O Mass: 165.065 Std.RT: 812.0600004 Ion: POS

G1

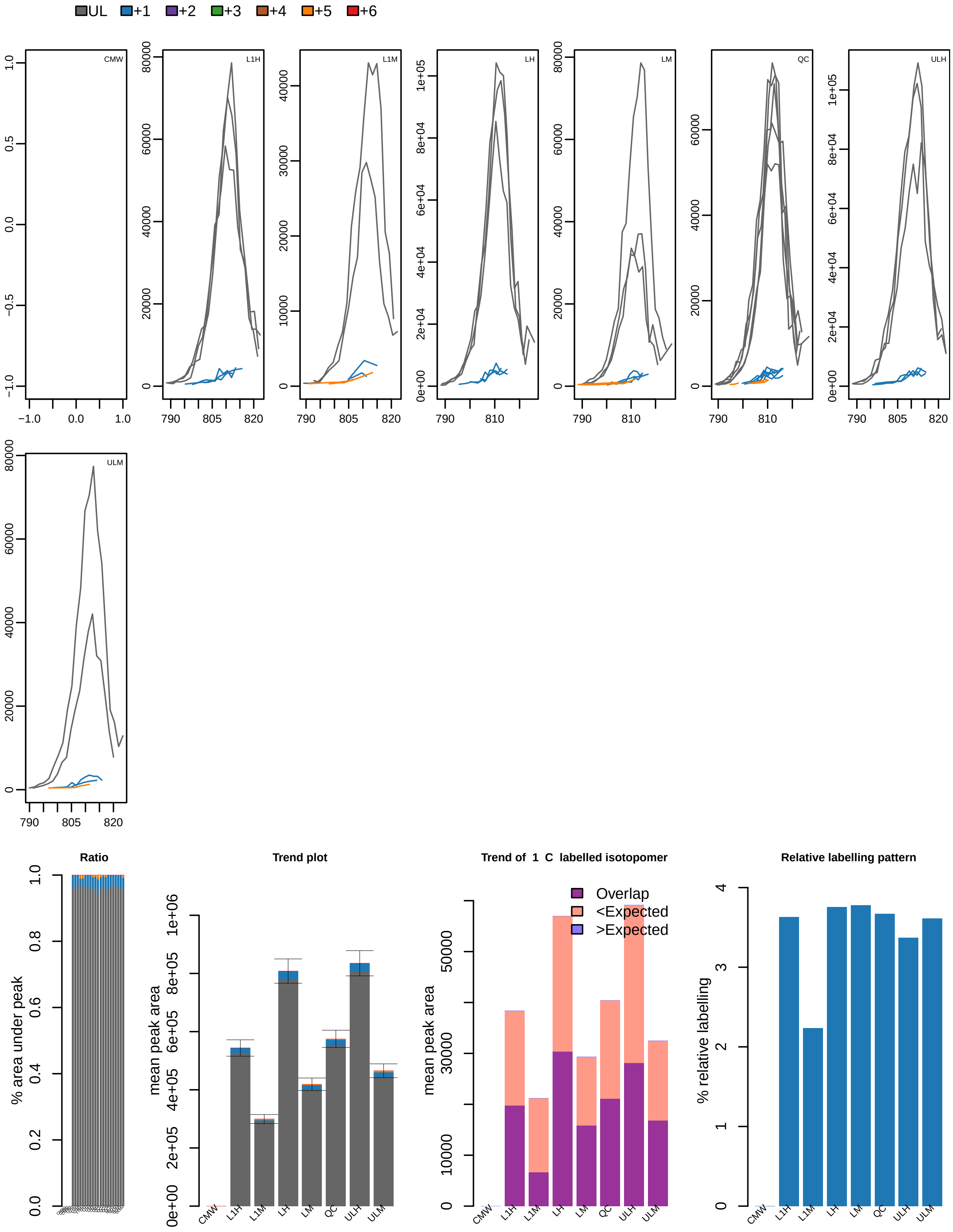

Miroestrol

Formula: C20H22O6 Mass: 358.142 Std.RT: 919.6930578 Ion: POS

G1

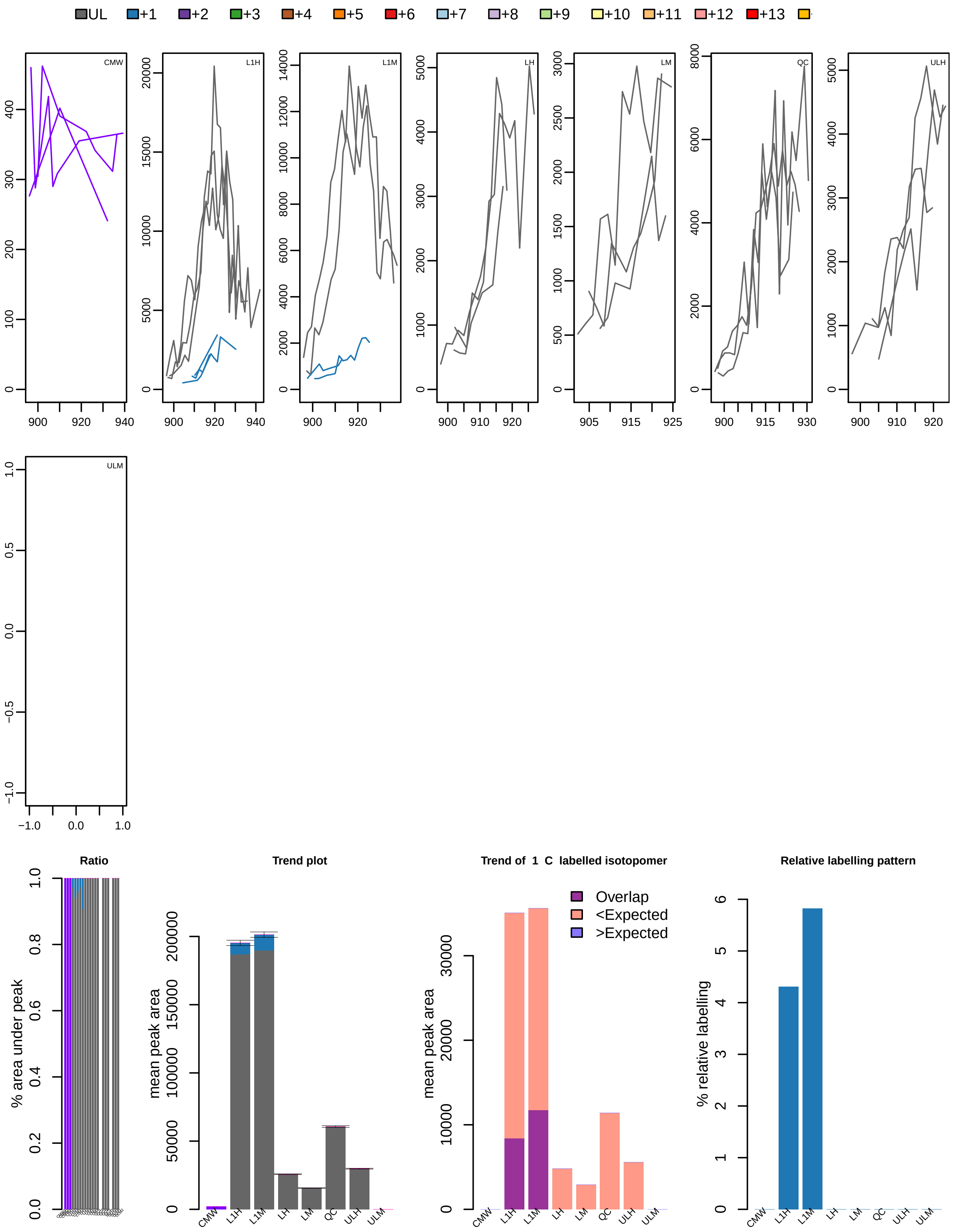

N,N−Dimethyl−1,4−phenylenediamine

Formula: C8H12N2 Mass: 136.1 Std.RT: 436.33770882 Ion: POS

G1

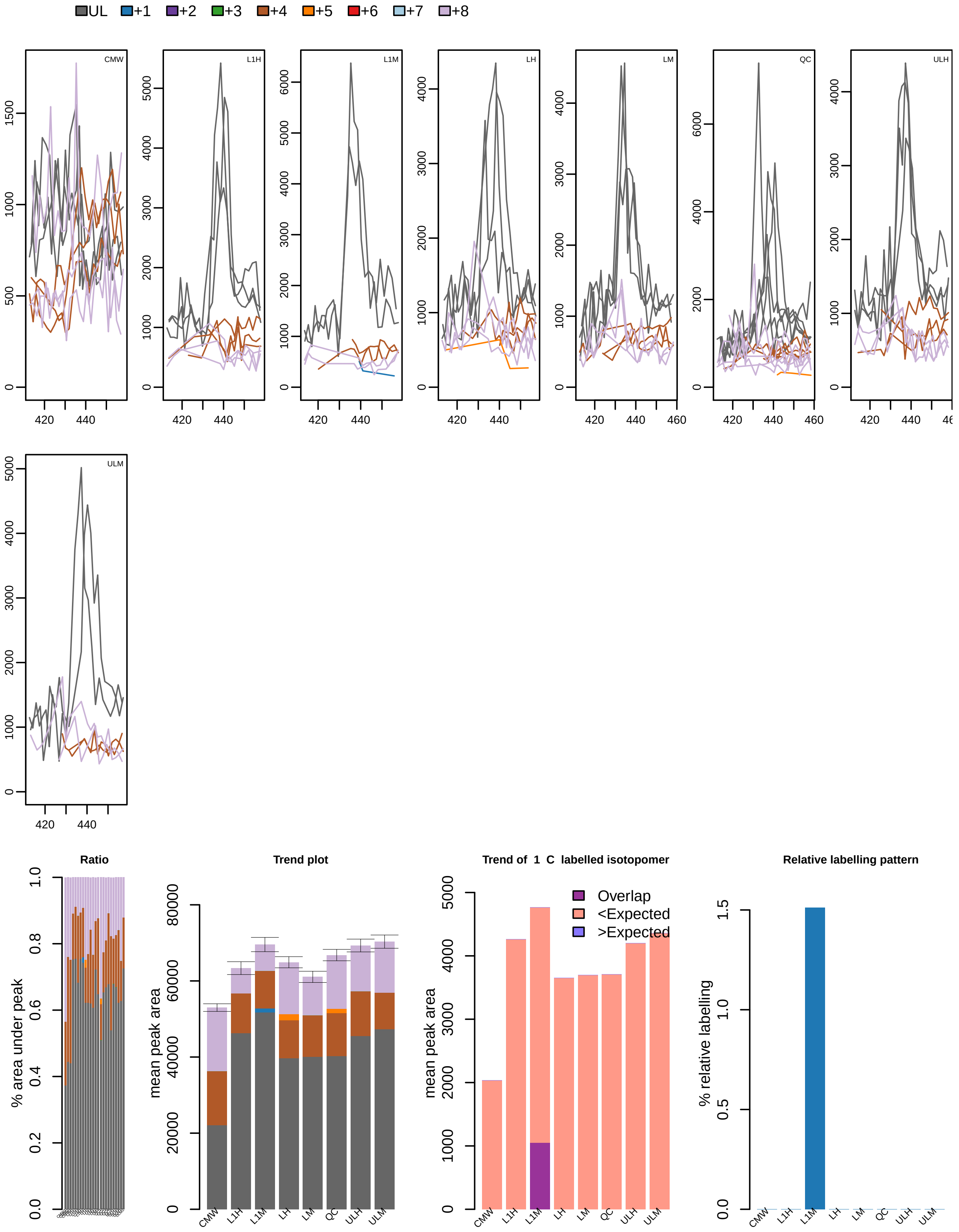

1,2−O−Diacetylzephyranthine

G1

Formula: C20H23NO6 Mass: 373.153 Std.RT: 585.1030041 Ion: POS

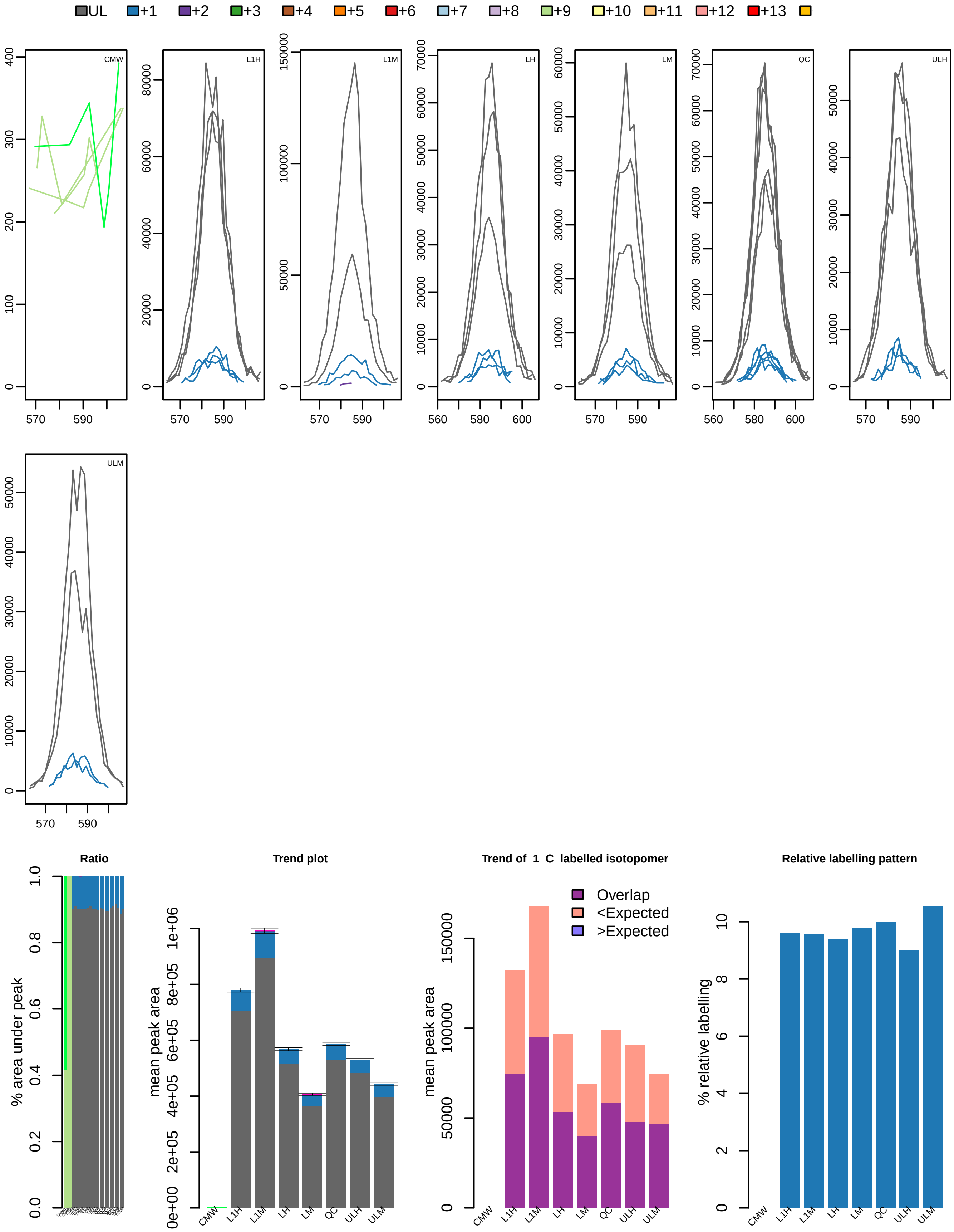

meso−α,δ−diaminopimelate

Formula: C6H12N2O4 Mass: 176.08 Std.RT: 1441.3376232 Ion: POS

G1

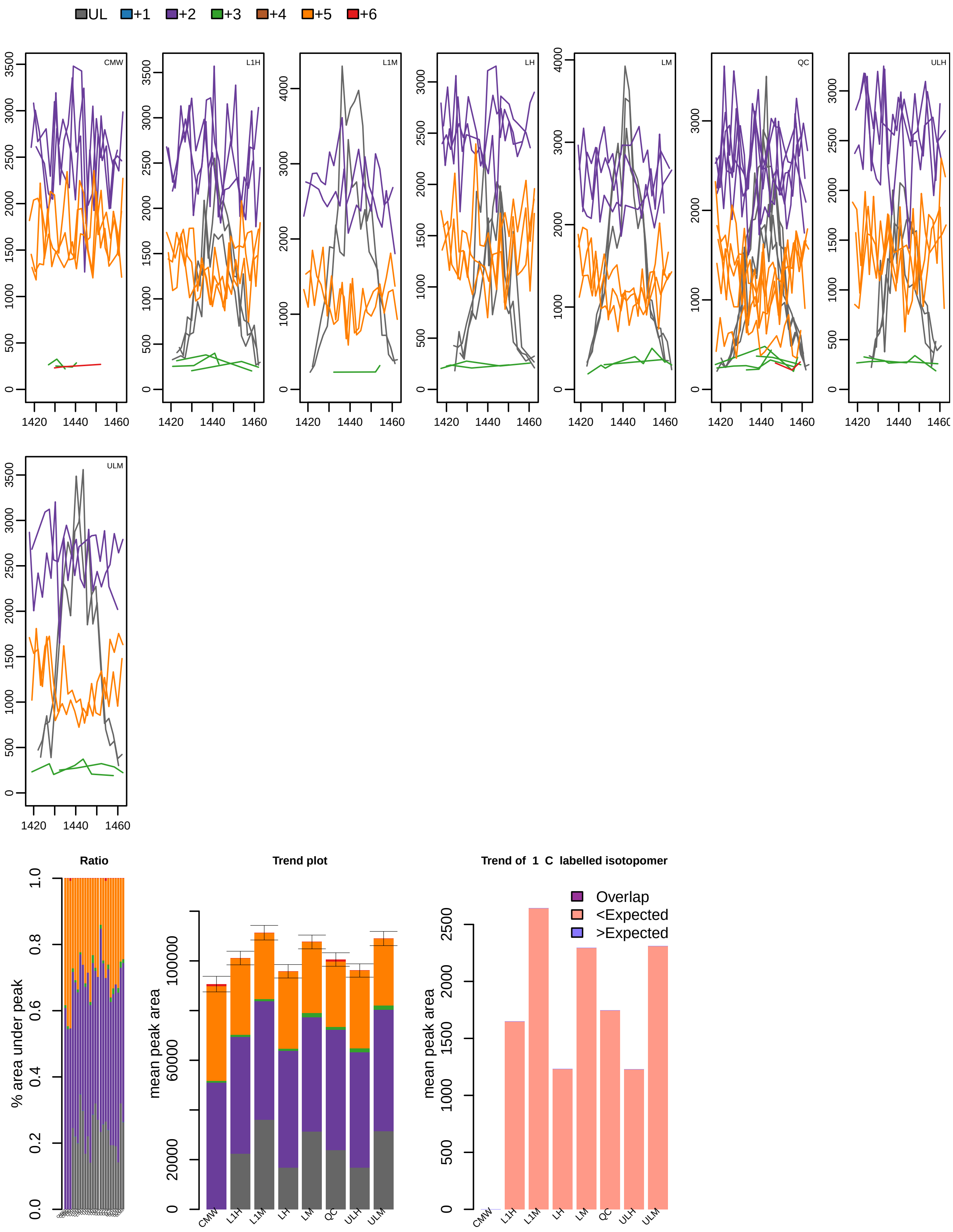

7−Methylguanine

Formula: C6H7N5O Mass: 165.065 Std.RT: 601.6156644 Ion: POS

G1

UL +1 +2 +3 +4 +5 +6

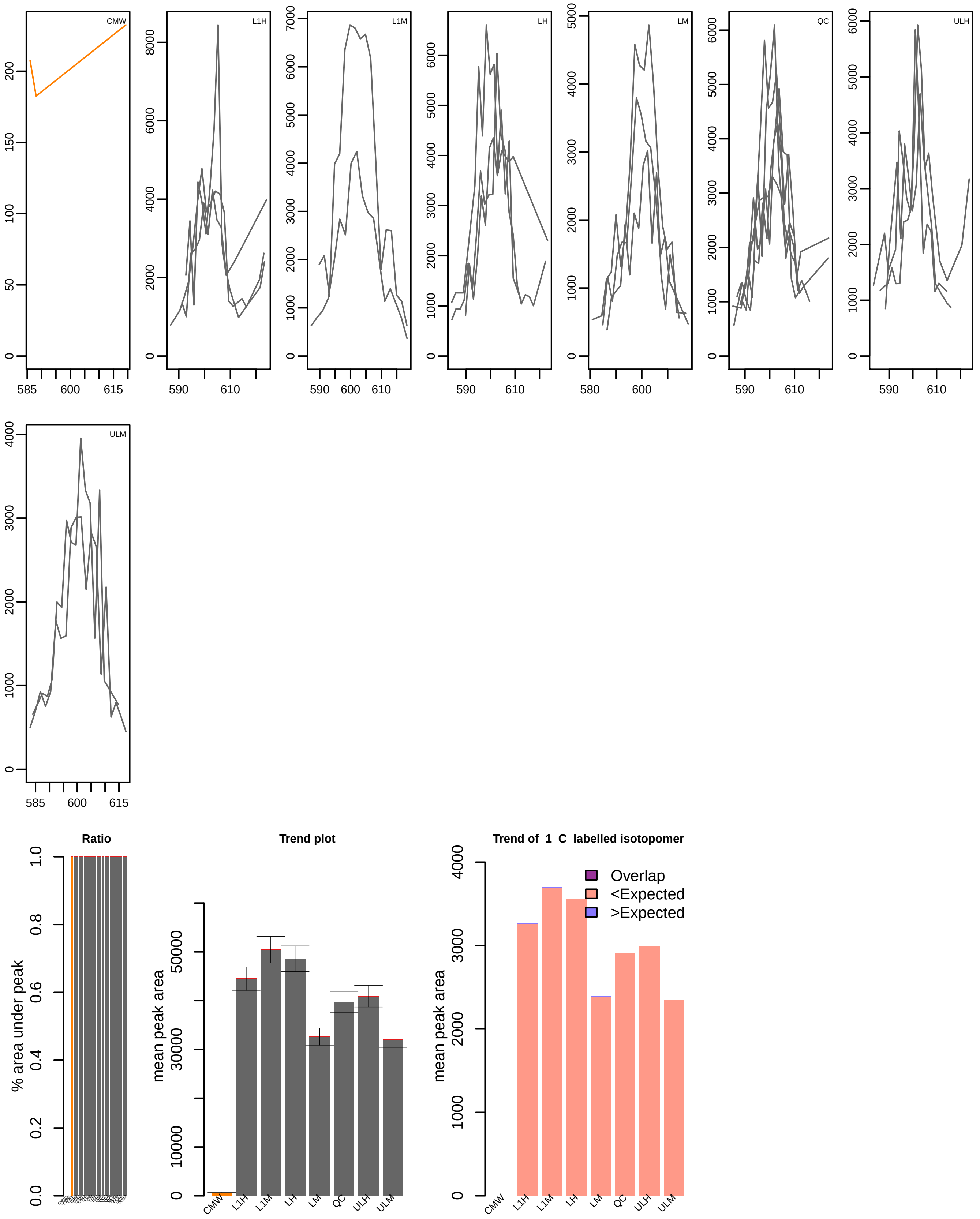

N−Decanoylglycine

Formula: C12H23NO3 Mass: 229.168 Std.RT: 256.91795352 Ion: PC

G1

UL +1 +2 +3 +4 +5 +6 +7 +8 +9 +10 +11 +12

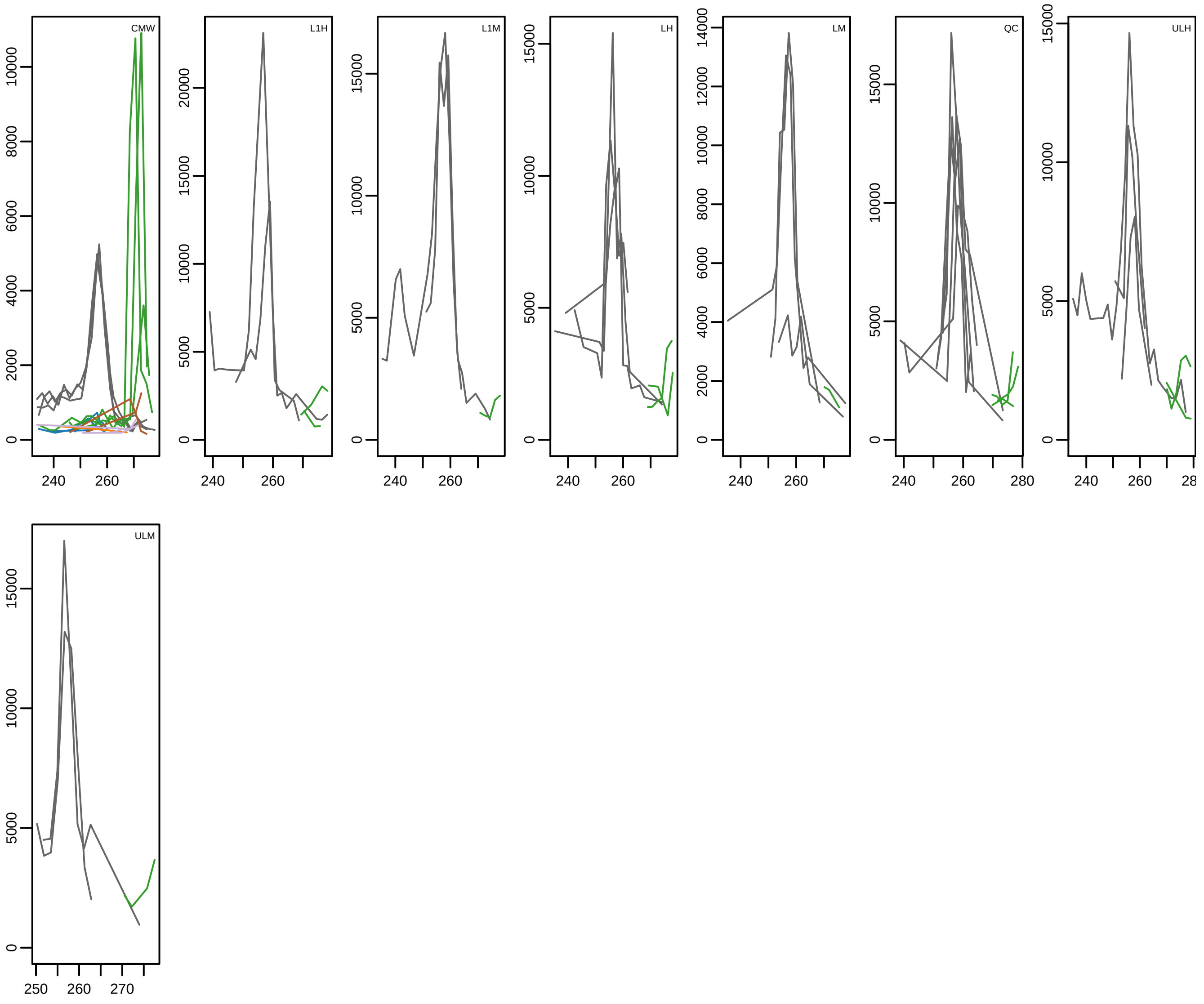

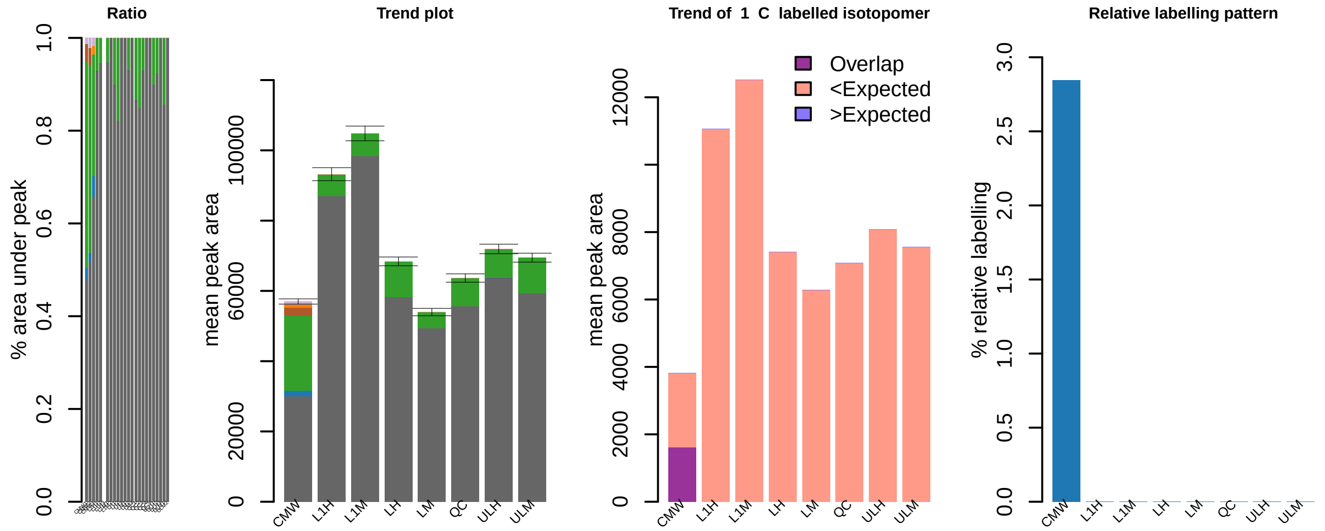

N−acetyl−(L)−arginine

Formula: C8H16N4O3 Mass: 216.122 Std.RT: 972.6468594 Ion: POS

G1

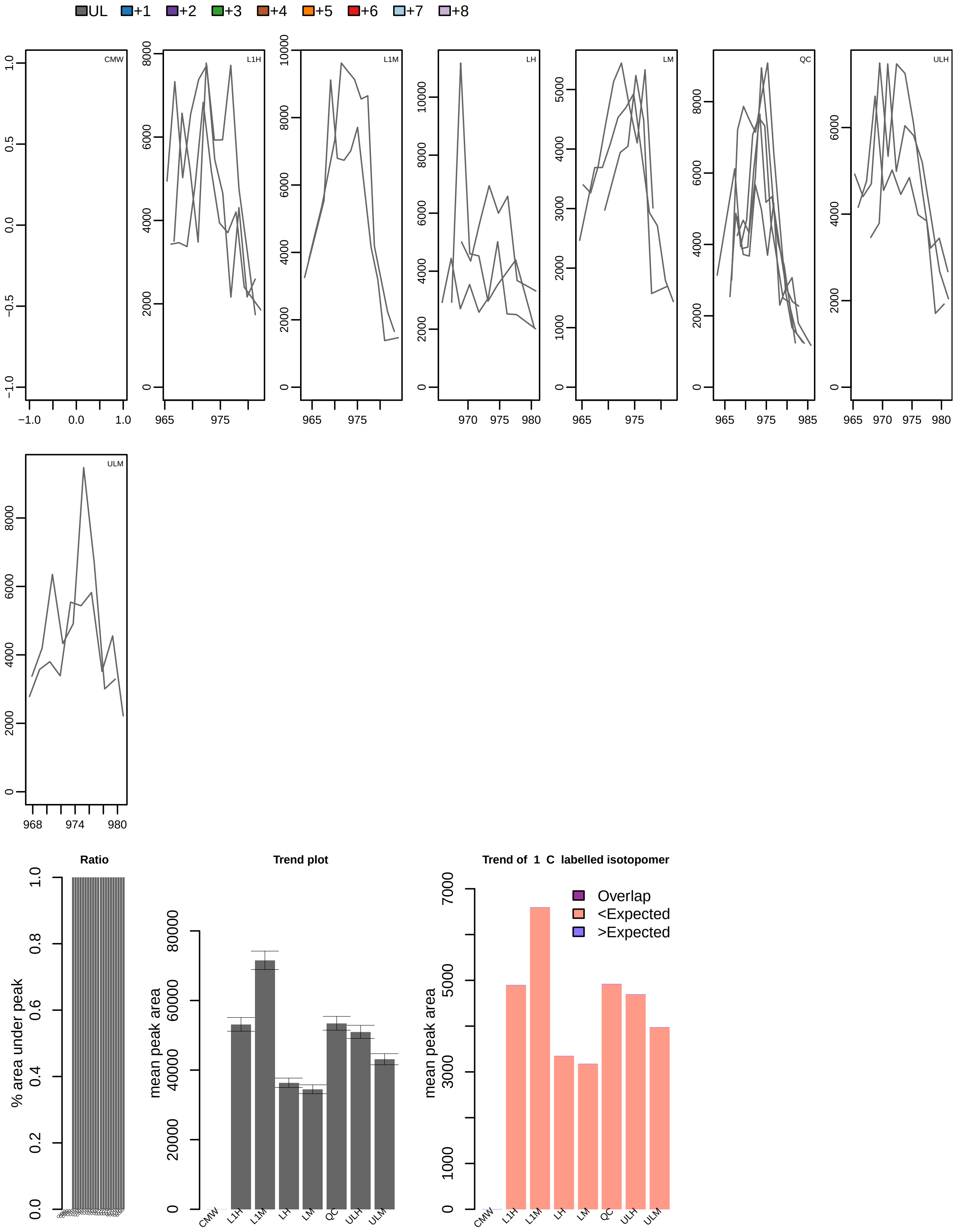

N−Nonanoylglycine

Formula: C11H21NO3 Mass: 215.152 Std.RT: 266.51283648 Ion: PC

G1

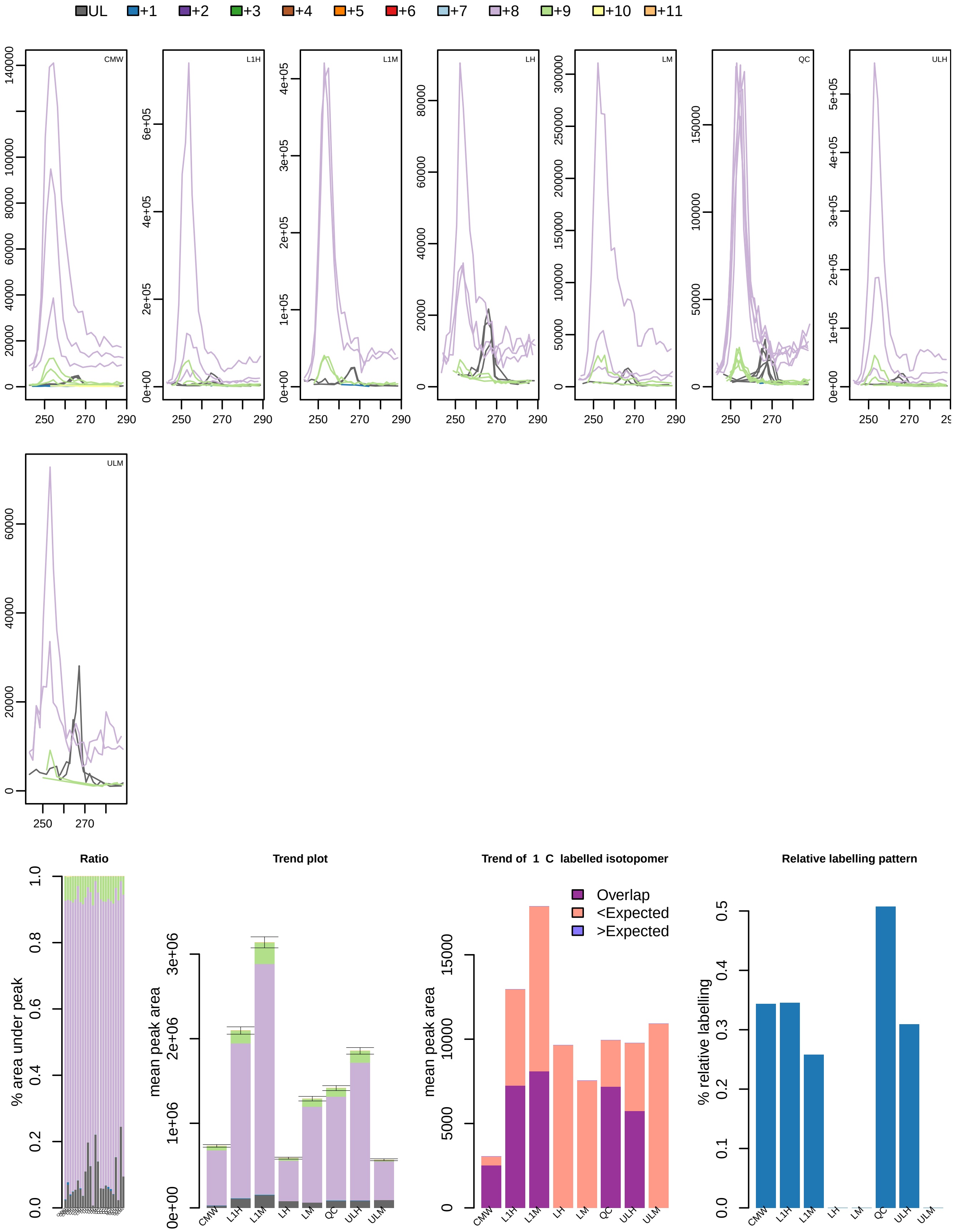

N6,N6−Dimethyladenosine

Formula: C12H17N5O4 Mass: 295.128 Std.RT: 524.54218692 Ion: P

G1

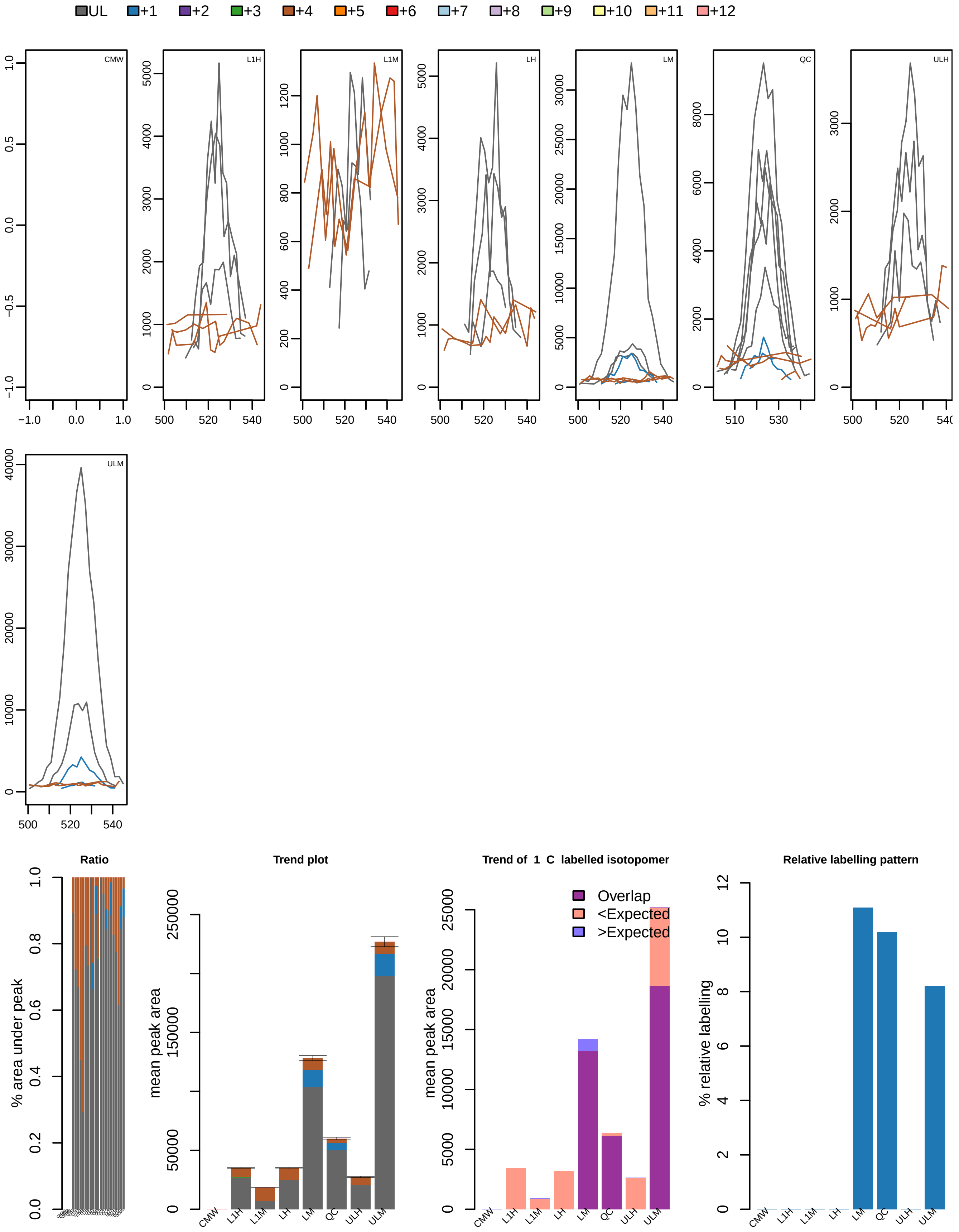

di−n−Undecylamine

Formula: C22H47N Mass: 325.371 Std.RT: 433.36716462 Ion: POS

G1

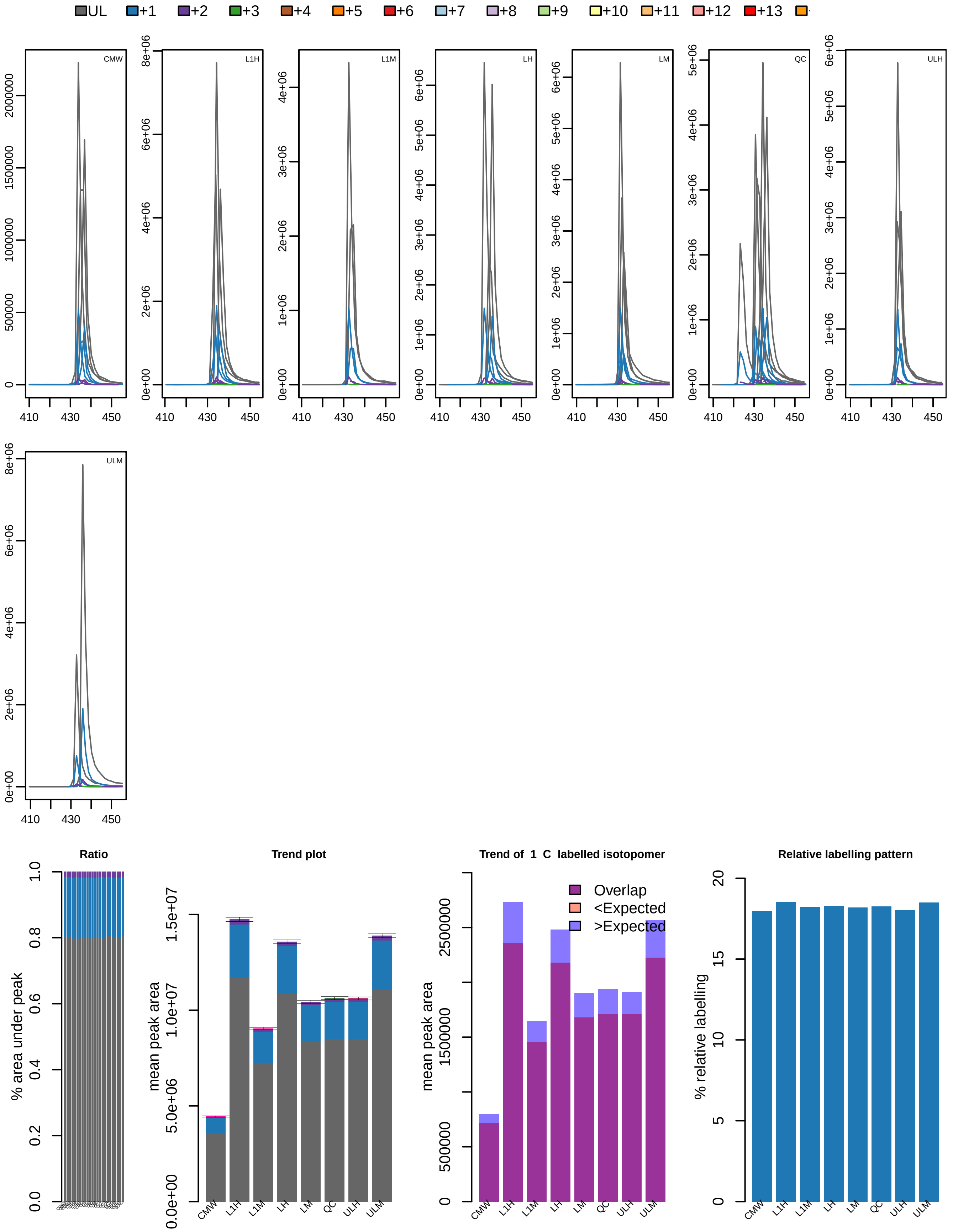

4−(Trimethylammonio)but−2−enoate

Formula: C7H13NO2 Mass: 143.095 Std.RT: 687.9115014 Ion: POS

G1

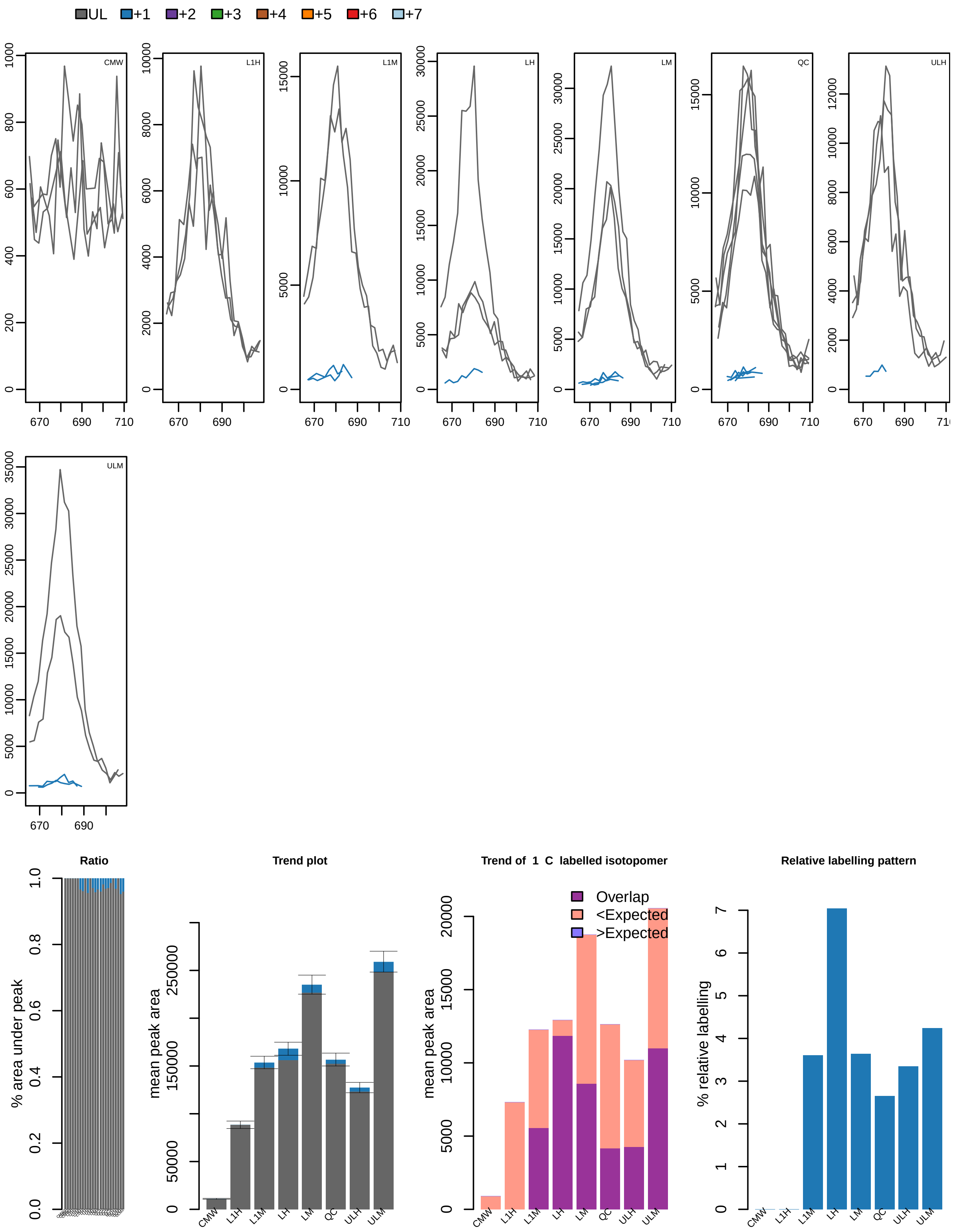

4−(Trimethylammonio)but−2−enoate

Formula: C7H13NO2 Mass: 143.095 Std.RT: 687.9115014 Ion: POS

G2

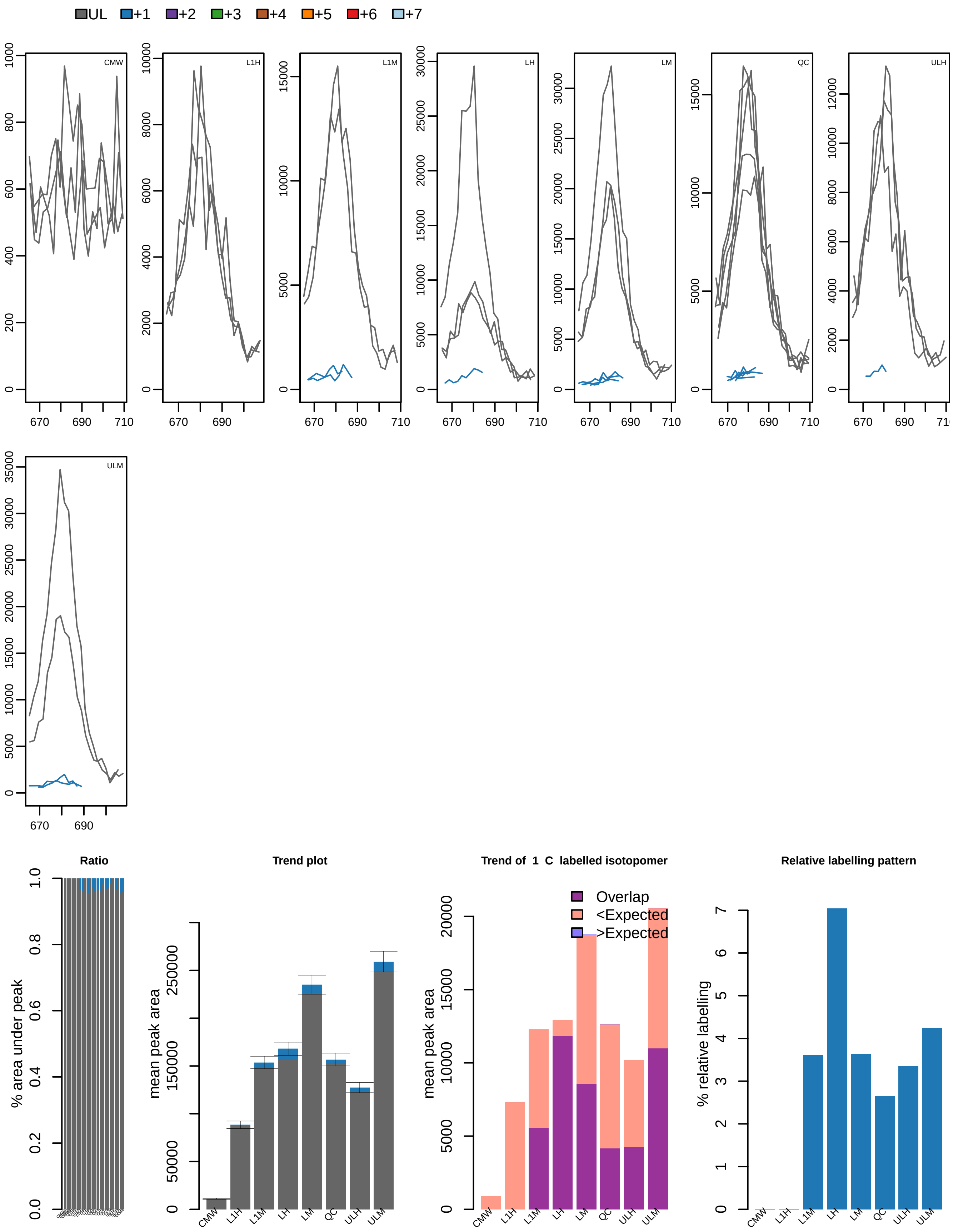

L−proline amide

Formula: C5H10N2O Mass: 114.079 Std.RT: 653.6602476 Ion: POS

G1

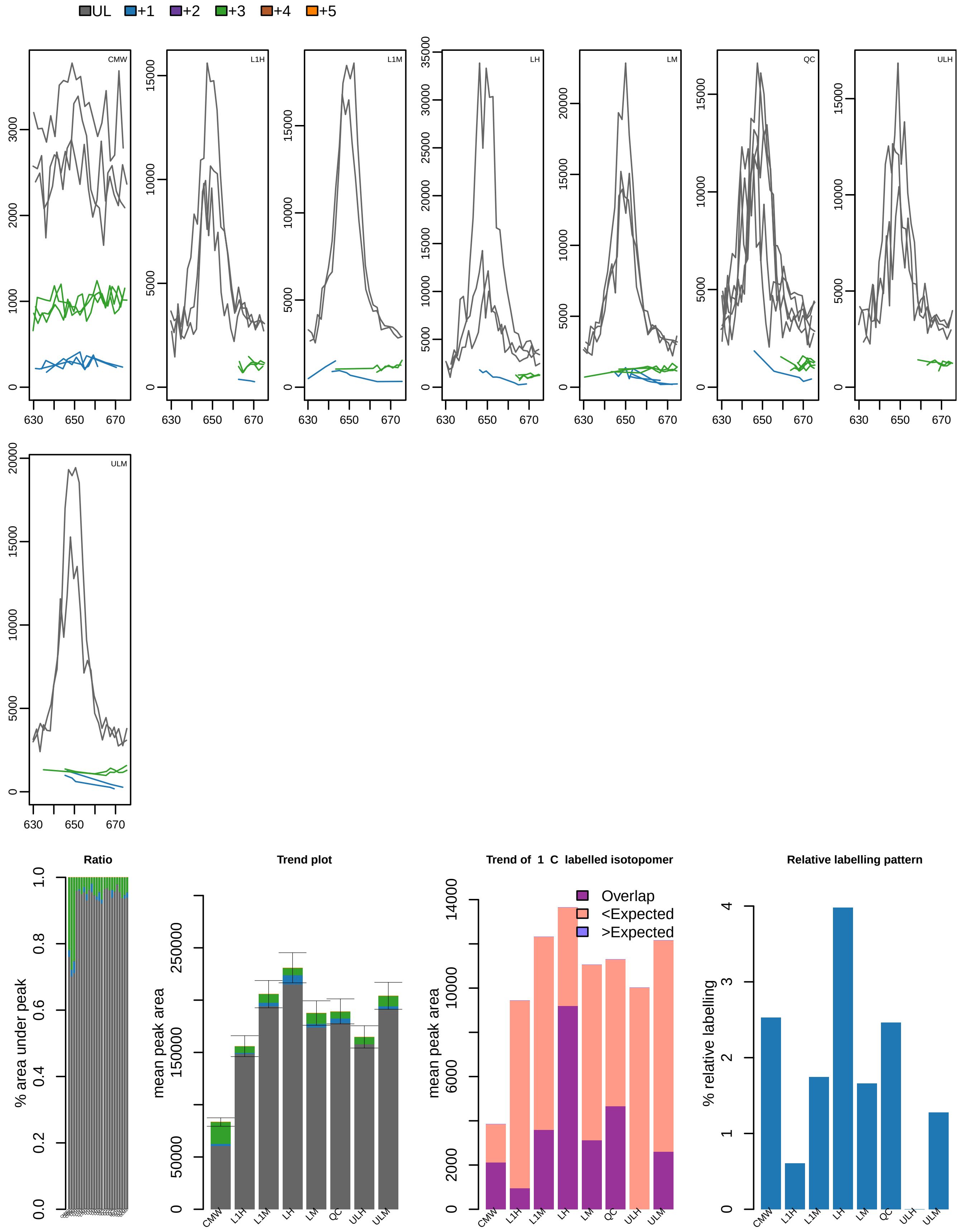

NicotinamideN−oxide

Formula: C6H6N2O2 Mass: 138.043 Std.RT: 666.160749 Ion: POS

G1

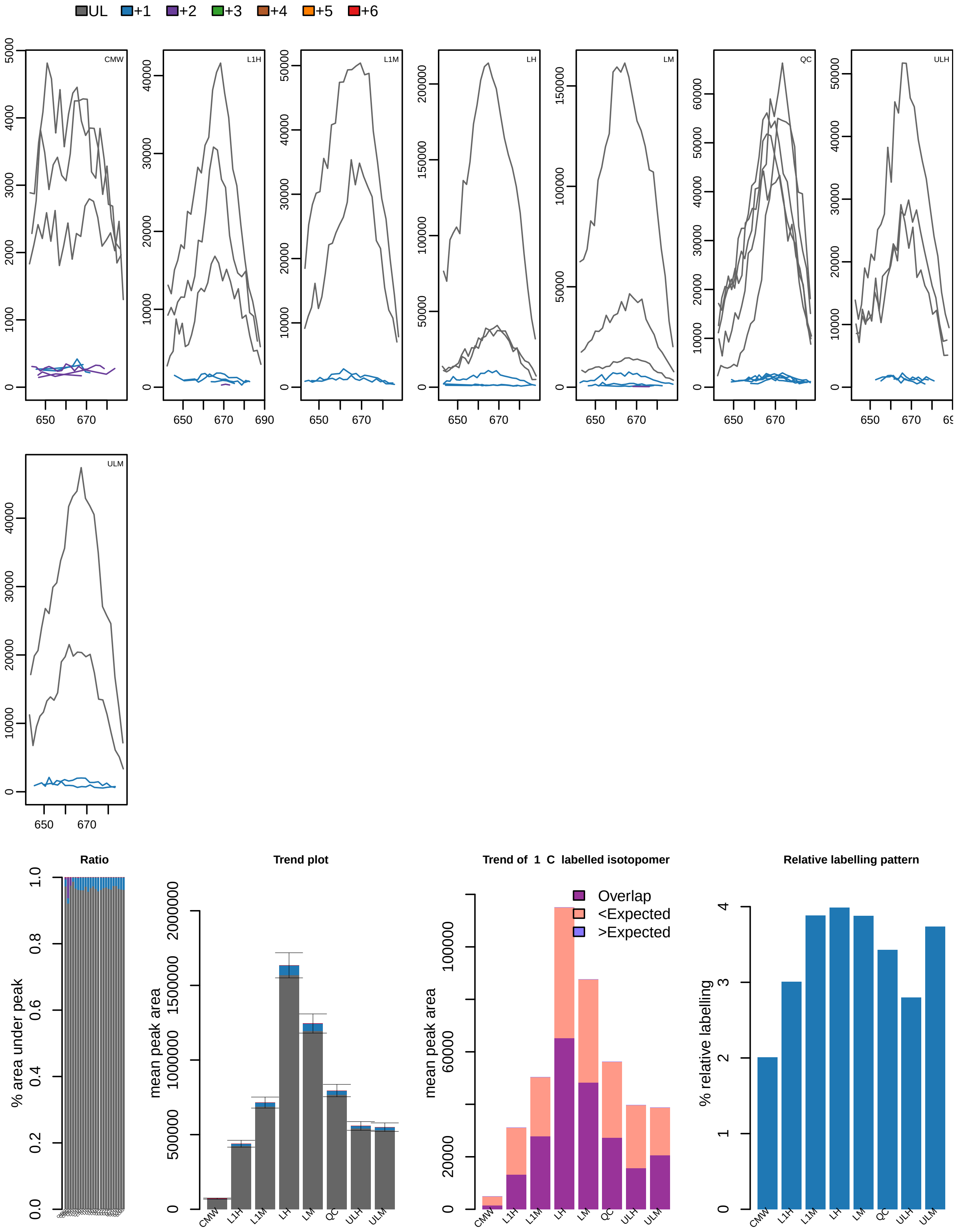

S−Acetyldihydrolipoamide

Formula: C10H19NO2S2 Mass: 249.086 Std.RT: 444.17557338 Ion:

G1

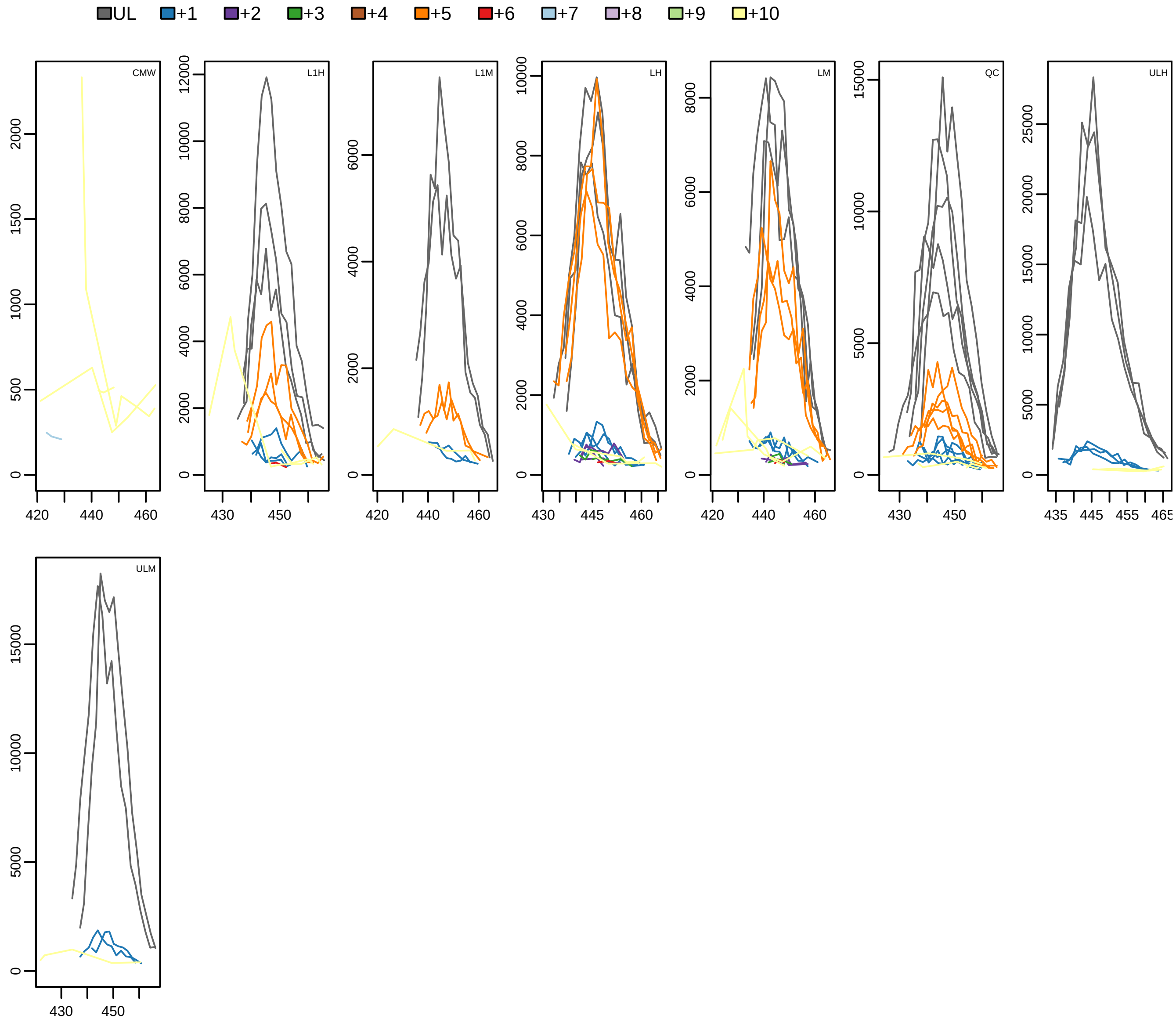

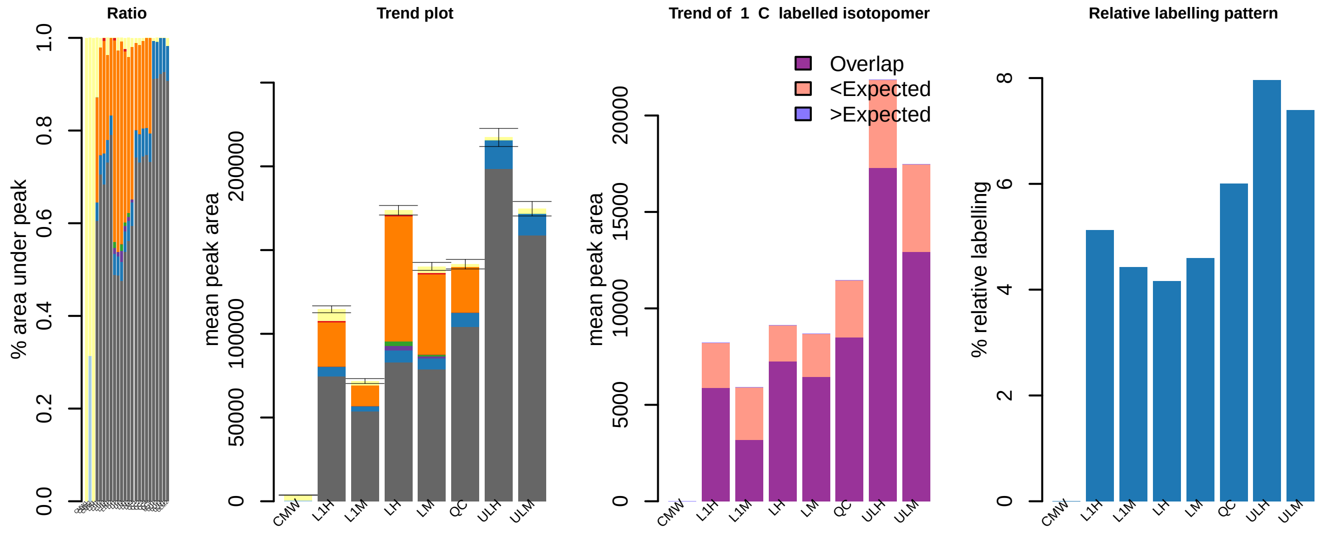

allylcysteine

Formula: C6H11NO2S Mass: 161.051 Std.RT: 722.125809 Ion: POS

G1

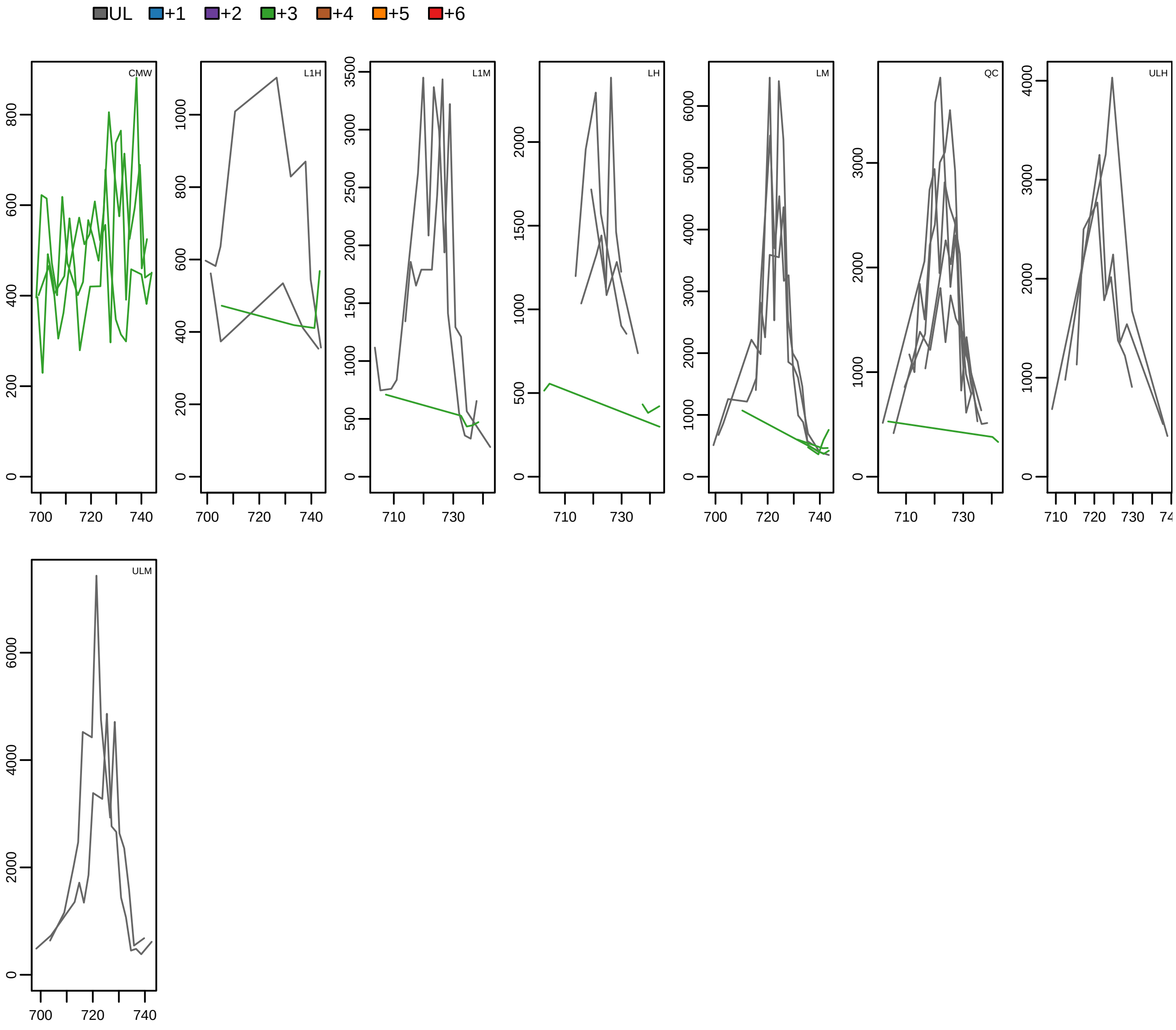

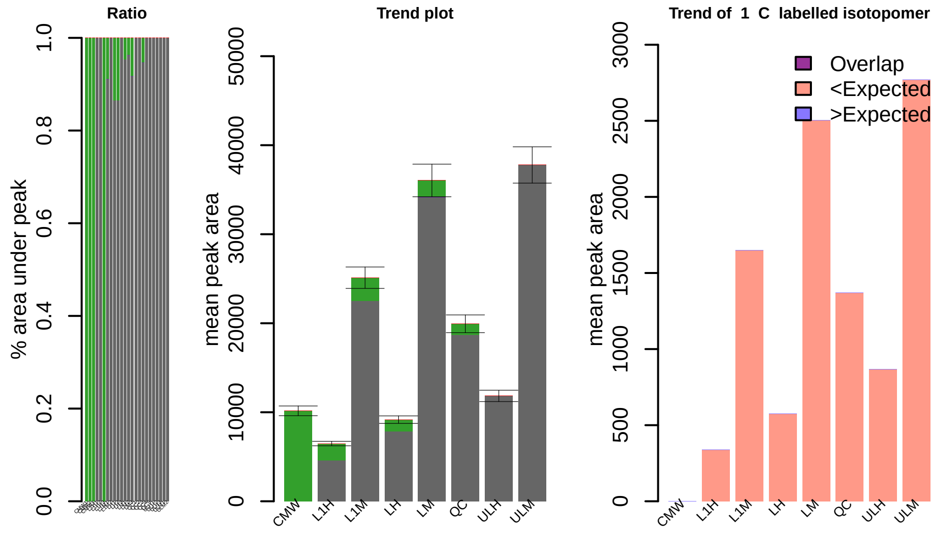

Magnoflorine

G1

Formula: C20H23NO4 Mass: 341.163 Std.RT: 652.3591368 Ion: POS

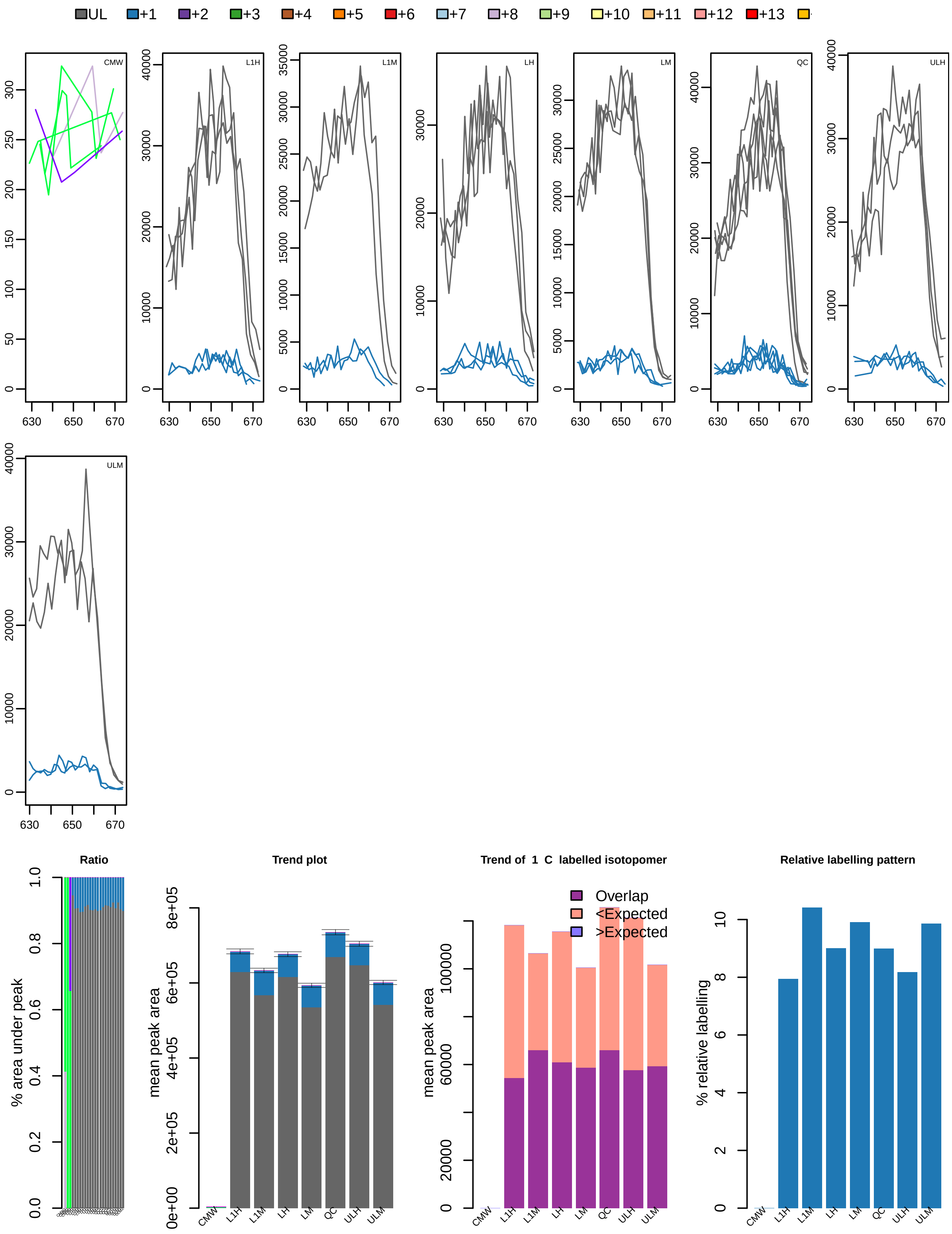

Magnoflorine

Formula: $C_{20}H_{23}NO_4$ Mass: 341.163 Std.RT: 652.3591368 Ion: POS

G2

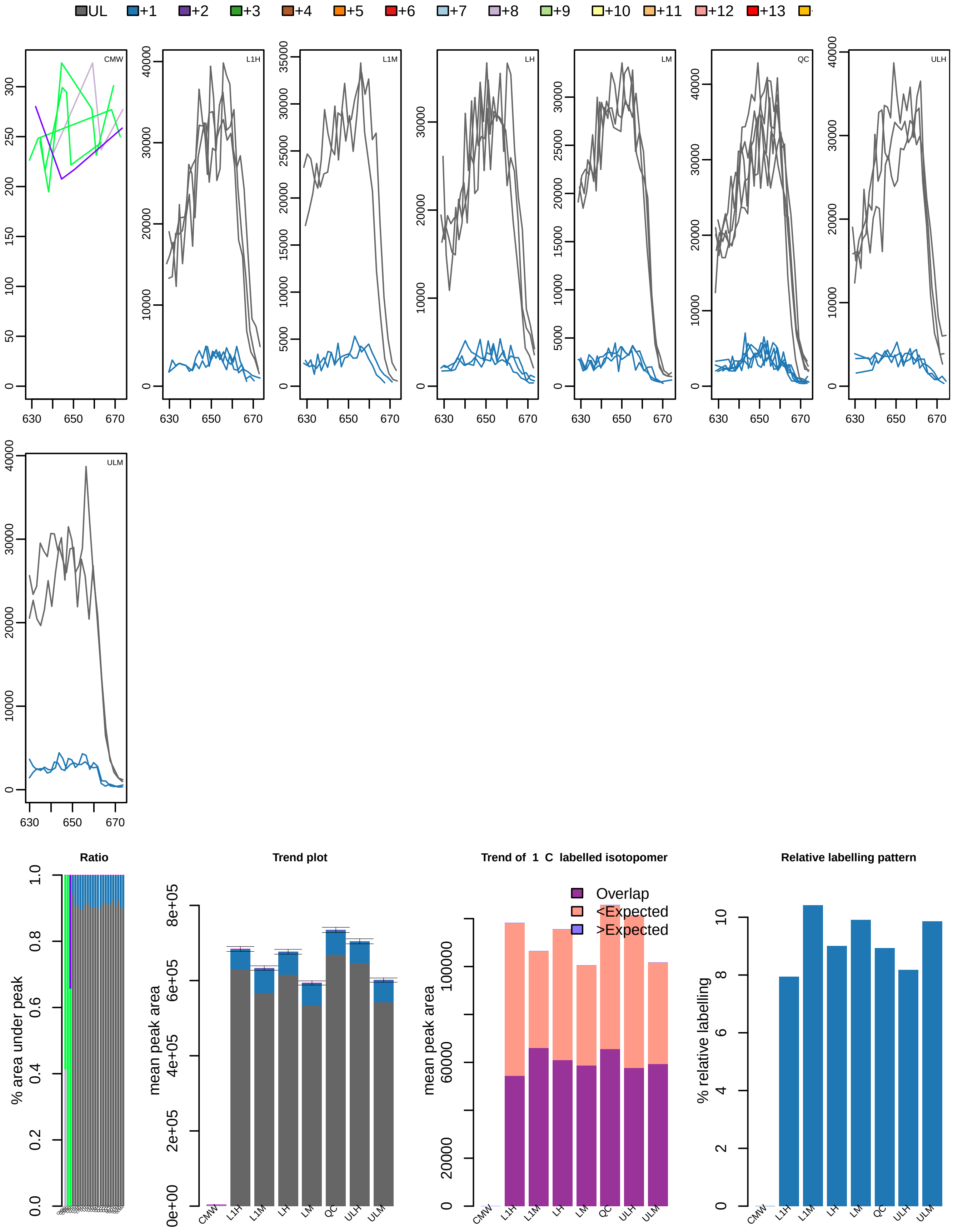

Stachydrine

Formula: C7H13NO2 Mass: 143.095 Std.RT: 874.046496 Ion: POS

G1

UL +1 +2 +3 +4 +5 +6 +7

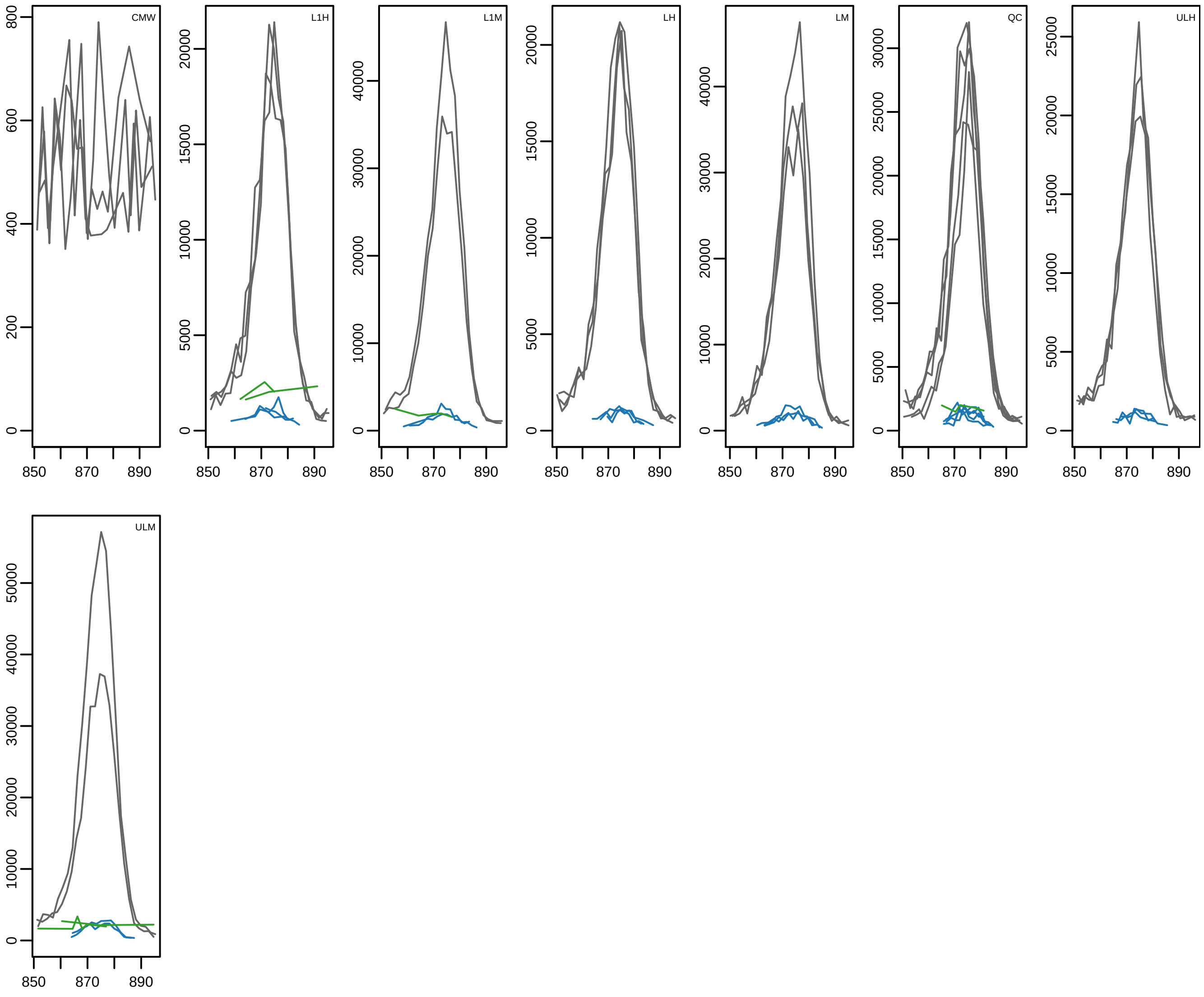

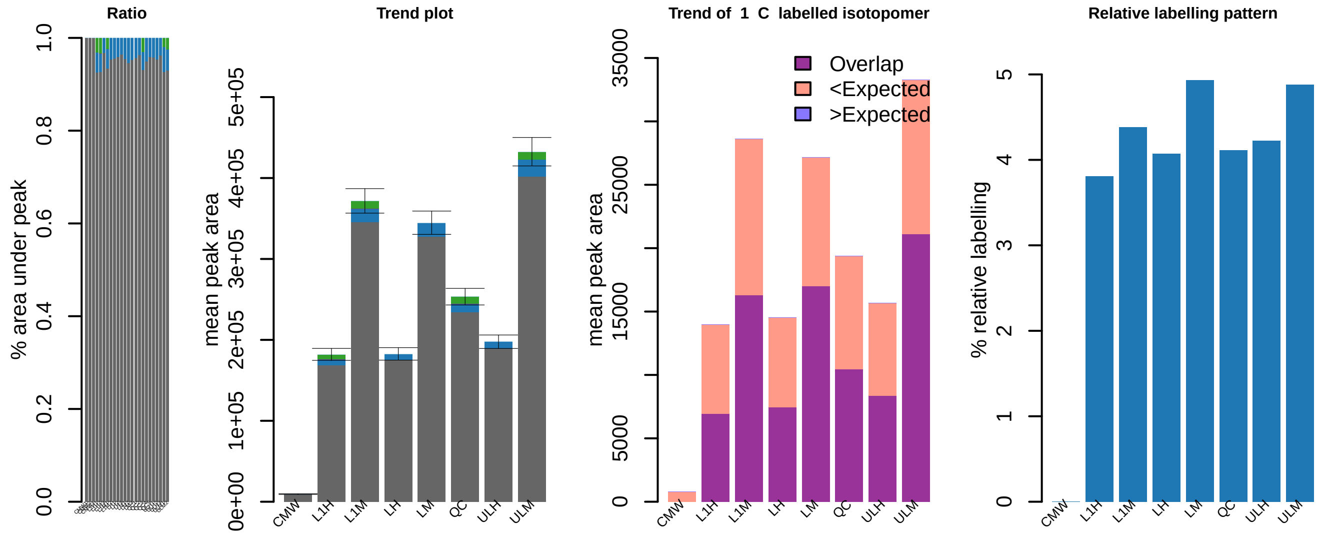

N,N−dihydroxyvaline

Formula: C5H11NO4 Mass: 149.069 Std.RT: 1017.9055578 Ion: POS

G1

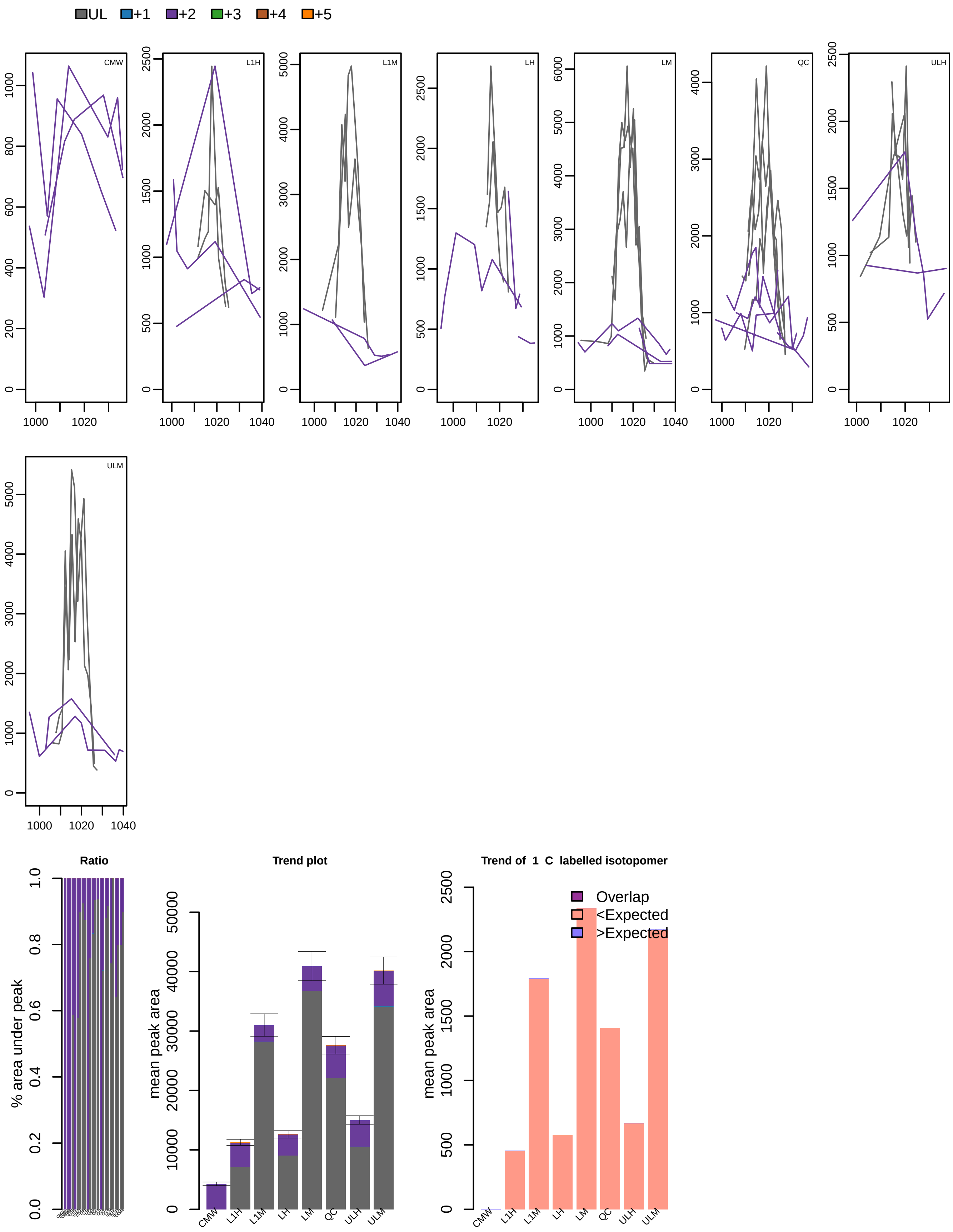

N−hydroxyvaline

Formula: C5H11NO3 Mass: 133.074 Std.RT: 434.24699778 Ion: POS

G1

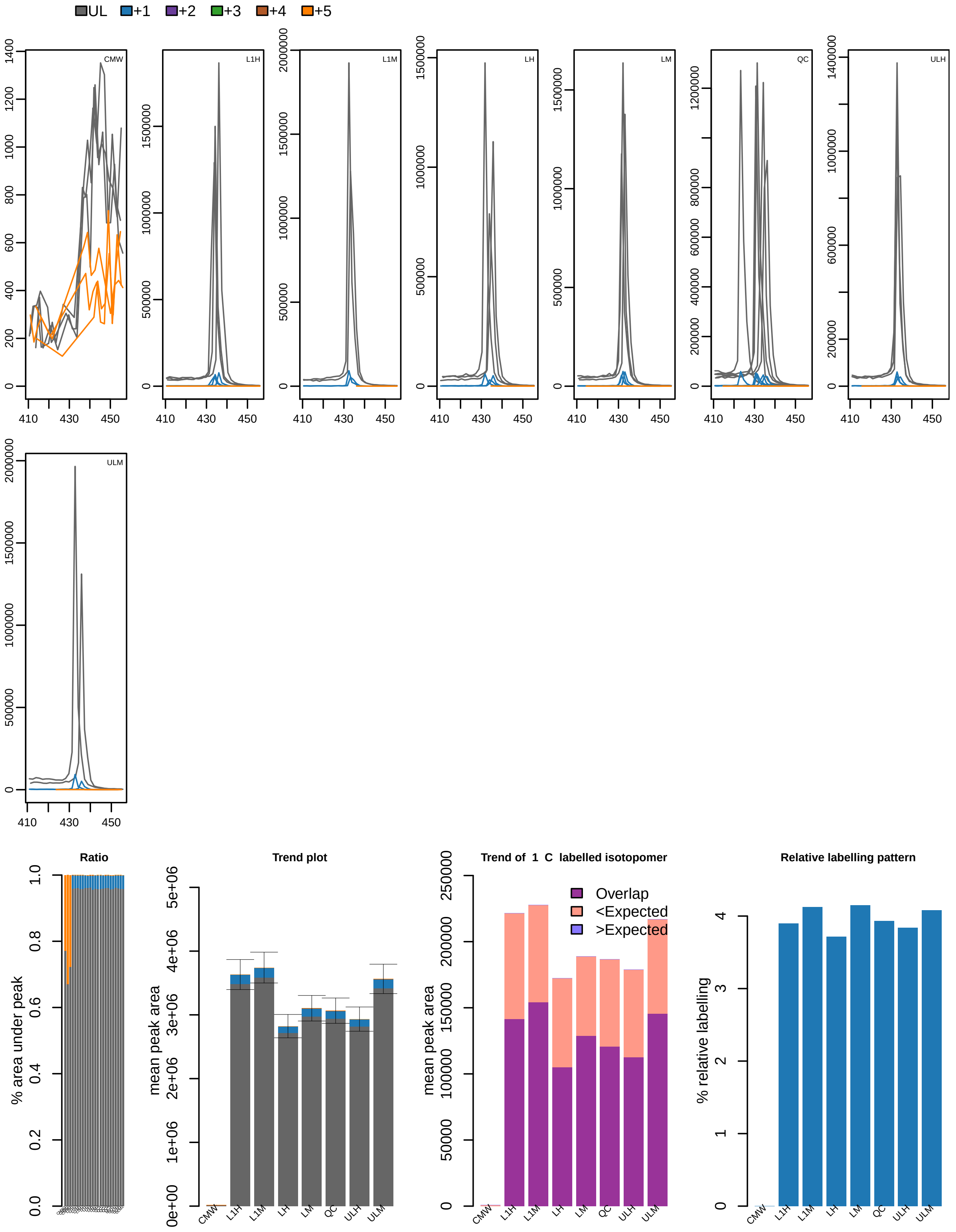

N−hydroxyvaline

Formula: C5H11NO3 Mass: 133.074 Std.RT: 434.24699778 Ion: POS

G2

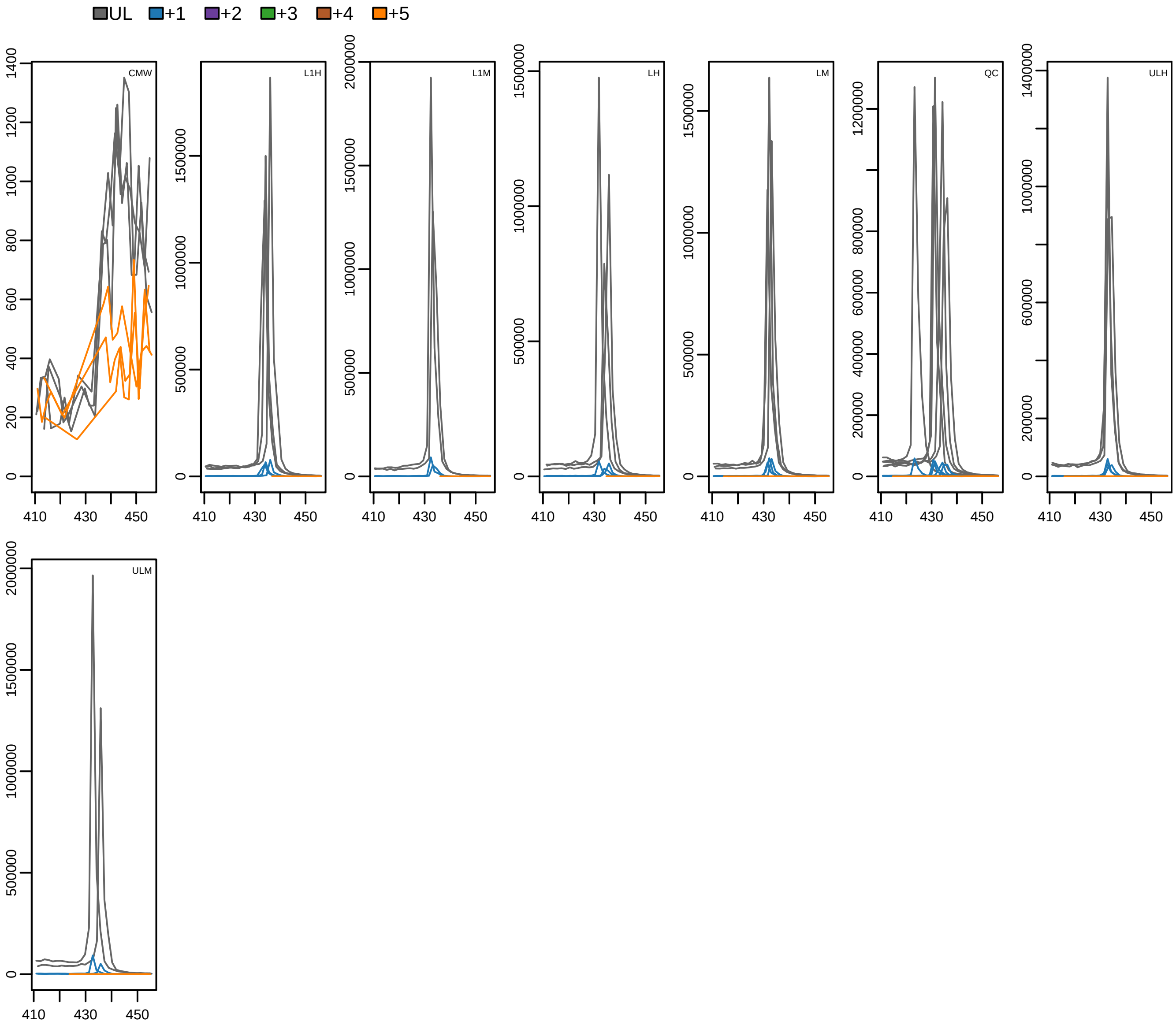

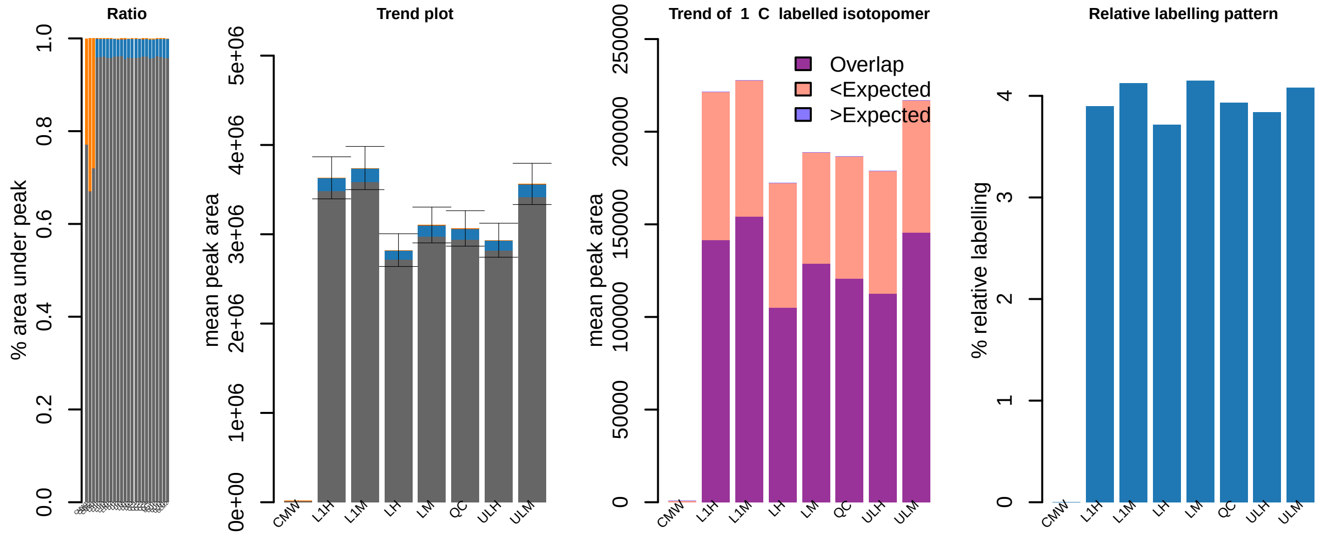

Supplement: S1 Fig — (PDF) [file ppat.1004689.s007.pdf]
